# A Sensitivity Study for Interpreting Nucleic Acid Sequence Screening Regulatory and Guidance Documentation: Toward a Foundational Synthetic Nucleic Acid Sequence Screening Framework

**DOI:** 10.1089/apb.2023.0026

**Published:** 2024-09-18

**Authors:** Bryan T. Gemler, Chiranjit Mukherjee, Patrick A. Fullerton, James Diggans, Craig Bartling

**Affiliations:** ^1^ Battelle Memorial Institute, Columbus, Ohio, USA.; ^2^ Twist Bioscience Corporation, South San Francisco, California, USA.

**Keywords:** biosecurity, sequence screening, DNA synthesis, export control, tier 1 agents

## Abstract

**Objectives:**

The primary objectives of this study were to develop an objective nucleic acid sequence screening framework and to leverage the framework for an empirical sensitivity study that measures the impact of ambiguities in regulatory and guidance documentation regarding the control of synthetic nucleic acids and screening of nucleic acid orders.

**Methods:**

Foundational risk levels were constructed using the bioinformatic sequencing screening tool UltraSEQ. The risk levels range from high (corresponding to regulated sequences) to low (corresponding to nonregulated sequences of concern) to no-risk. A representative sequence data set (141,651 sequences) was constructed from publicly available synthetically derived sequences, and the percentage sequences in each risk level was determined, followed by the impact of changing key UltraSEQ parameters.

**Results:**

The results of this study show that no-risk sequences represent 90–92% of sequences, and nonregulated sequences of concern represented 7–9% of the sequences regardless of the parameters. The parameter with the biggest impact on the number of sequences flagged was the minimum hit homology level, followed by minimum sequence region length, and finally uniqueness of the hit to a select agent sequence.

**Conclusion:**

The results of this empirical study provide a greater understanding for gene synthesis providers, biosafety and biosecurity practitioners, and the scientific community regarding the impact of various interpretations of regulatory and guidance documentation. The risk level framework provides a foundation to build upon for nucleic acid sequence screening as the threat landscape evolves. However, additional development is needed to build tools that connect predictions across sequences and orders to provide contextual risk-based predictions.

## Introduction

Traditional paradigms for biosafety and biosecurity focus on the controlled possession, transfer, and use of viable biological agents and whole genomes. However, synthetic biology is revolutionizing science and engineering through the design and assembly of synthetically made nucleic acids, thus presenting the need for screening synthetic nucleic acid orders to ensure compliance with country-specific Federal regulations and to guard against the synthesis of potentially dangerous biological agents and toxins.

However, categorizing which sequences are regulated and/or potentially dangerous is subject to interpretation of screening guidance documents and regulatory language. Although cautious interpretation would likely ensure full regulatory compliance and biosafety, such overreach may slow the progress of the bioeconomy by raising the cost for synthetic nucleic acid due to labor required to review and interpret screening results. In contrast, strict interpretation may fail to secure against bioterror. Thus, a reasonable balance must be achieved.

Unfortunately, there is a lack of empirical studies that describe the impact of interpretation of regulatory and (potentially of lesser consequence) guidance language for biological sequences. In this study, we describe ambiguity in the regulatory and guidance language and the empirical impact of various interpretations of the language to provide an objective sequence screening framework and quantify the measurable impact of key sequencing screening choices on the number of flagged sequences.

The Possession, Use and Transfer of Select Agents and Toxins^1^ are defined by the following Code of Federal Regulations (CFRs): 42 CFR Part 73 (U.S. Department of Health and Human Services [HHS]),^2^ 7 CFR part 331 (U.S. Department of Agriculture, Animal and Plant Health Inspection Service, USDA APHIS),^3^ and 9 CFR part 121 (USDA APHIS),^4^ which together define the Federal Select Agent Program (FSAP). Individuals with access to Tier 1 agents (a subset of the FSAP agents), must be enrolled in an Occupational Health Program.^5^

Only certain genetic elements are regulated under FSAP, including elements that are “inherently infectious and are immediate precursors to virus production” and “synthetic nucleic acids that encode for the toxic form(s) of the regulated toxin.”^6^ A similar strict interpretation of controlled biological materials is assumed for those organisms on the United States Munitions List (USML) (i.e., control is mainly at the organism and/or whole genome level), which are regulated by the International Traffic in Arms Regulations 22 CFR parts 120–130, with control of genetic elements on the USML in 22 Part 121.^7^

In addition to control within the United States, the U.S. Export Administration Regulations (EARs) are used to determine if a nucleotide product requires an export license under various Export Control Classification Numbers (ECCNs).^8^ Items not available to embargoed destinations and sanctioned end-users but otherwise not controlled are given a classification of EAR99.

Specific to genetic sequences, ECCN 1C353 references pathogens controlled under 1C351 and 1C354, collectively known as the pathogens and toxins on the Commerce Control List. 1C353 further references the Australia Group List of Human and Animal Pathogens and Toxins for Export Control.^9^ Similar to the United States, the European Union Export Control Regulation No 428/2009 controls the export of genetic elements with similar language.^10^

In addition to regulatory control, screening guidance is issued by the U.S. Department of HHS. In 2010, HHS published the “Screening Framework Guidance for Providers of Synthetic Double-Stranded DNA,” which provides guidance on screening customers and sequences.^11^ Although the guidance is voluntary, the FSAP references these guidelines: “even when the double-stranded DNA sequence being processed does not fall under the current select agent regulations, providers are advised to refer to (the HHS) policy for additional guidance on screening such orders”.^6^

The guidance prompted the International Gene Synthesis Consortium (IGSC) to provide a Harmonized Screening Protocol to help both U.S. and non-U.S. providers in their screening practices.^12^ Recently, the HHS guidance has been updated^13^ to provide more explicit definitions of customer, users, providers, vendors, and benchtop device manufacturers.

The guidance further expands sequence screening relative to the 2010 guidance to: (1) screening all types of nucleic acid sequences (e.g., single-stranded DNA in oligo pools and RNA sequences, not just double-stranded DNA), (2) screening sequences of 50 nucleotides (nts) or even shorter within 3 years, (3) screening oligo pools, (4) implementing screening in benchtop devices, and (5) expanding screening to “sequences of concern” (SoCs), not just select agents.

Although the combined guidance and regulations described earlier provide a foundation for potential sequences that may require customer follow-up and/or regulatory control, some ambiguity exists around the specific sequences that should be flagged and/or controlled. More specifically, how designers of sequence screening tools interpret the language within these documents could lead to wide variation in the numbers and types of sequences that get flagged.

Current state-of-the-art bioinformatic tools to match a query sequence against a reference database include homology-based aligners and k-mer matching software. In the case of these tools, this variation centers around three main conditions: (1) length of the matching sequence region (hit), (2) definition of a “SoC,” and (3) homology level/specificity (Table 1).

**Table 1.** Main variables to consider during interpretation of sequence screening guidelines and regulation

***Sequence variable***	***Screening guidelines^13^***	***U.S. and EU export regulations^8,10^***	***Federal Select Agent regulations^6^***
Length	200 nts (current), 50 nts (within 3 years)^a^	Any “genes, translated product or translated products specific to” a controlled virus or a subset of genes for bacteria and fungi (see SoC definition below)^b^	Refers to HHS screening guidelines; only virus genomes that are “inherently infectious and are immediate precursors to virus production” and “synthetic nucleic acids that encode for the toxic form(s) of the regulated toxin” are controlled^c^
Definition of SoC	Current definition: “nucleotide sequence that is a Best Match…to a sequence of a federally regulated agent, except when the sequence is also found in an unregulated organism or toxin” Future definition: sequences “known to contribute to pathogenicity or toxicity…for which a direct and harmful impact on a host has been verified based on published experimental data … [or] based on homology to a sequence encoding a verified function”^d^	Any controlled virus gene (see above) or any controlled bacterial or fungal gene “which in itself or through its transcribed or translated products represents a significant hazard to human, animal or plant health or…[can] endow or enhance pathogenicity… or any [controlled] toxins, or their subunits”^e^	See above, only some viral genomes and toxins are controlled
Homology to verified threat/specificity to controlled agent sequence	Specific homology level not defined; “Best Match” to sequence in select agent unless sequence is “found in” non-select agent (see Current Definition of SoC above)	Homology level not defined; “Specific to” (See length definition above)	Not defined

^a^ The Guidance notes that “some synthetic nucleic acid orders may be appropriate for screening even if all components of the order are nucleic acids shorter than the screening window length” due to potential for constructing longer sequences.

^b^ Follow-up guidance from BIS in a recent letter to the IGSC^17^ suggests that genes can be any length and can be truncated; BIS suggests that if specific evidence exists that the gene product is “nonfunctional” (e.g., due to mutation or truncation) then it is not controlled; furthermore, for viruses, “post translationally cleaved elements from a polyprotein that are shown to be functional proteins should be considered genes.”

^c^ The EU Commission^10^ further defines a toxin subunit as “structurally and functionally discrete component of a whole toxin.”

^d^ The Guidance further defines “pathogenicity or toxicity that threatens public health, agriculture, plants, animals…or the environment,” but specific hosts are not described.

^e^ Follow-up guidance from BIS in a recent letter to the IGSC^17^ potentially broadens this definition for bacteria/fungi as genes “associated with pathogenicity of a controlled bacterium or fungus.”^17^

BIS, Bureau of Industry and Security; EU, European Union; HHS, Health and Human Services; IGSC, International Gene Synthesis Consortium; SoC, Sequence of Concern; U.S., United States.

For example, the HHS guidance suggests screening sequences of >50 nts, but regulatory documentation simply refers to “genes” without a clear definition. HHS initially defined SoCs as sequences that are specific to select agents,^14^ although a more functional-based paradigm is emerging that does not rely on select agent lists. Although we^15^ and others^16^ have provided function-based threat categories that should be considered, these categories are not referenced in official guidelines or regulatory documents.

More generally, regulatory documentation suggests that bacterial or fungal genes that “endow or enhance pathogenicity” should be controlled,^8^ but translation of this phrase for screening implementation can be subjective. Finally, even if a potential SoC is identified, unless the SoC is the exact sequence known to be threatening, homology-based thresholds or predictive-based thresholds must be applied to make a hit/no hit call, but such thresholds are not standardized and the degree to which sequence changes may have an impact on function is difficult to predict accurately without web lab validation.

Without objective definition of phrases and terms, nucleic acid synthesis vendors are left with difficult decisions regarding how conservative screening tools should be without disrupting their ability to compete in a highly international market for synthetic nucleic acids. The IGSC is committed to reducing biosecurity risks,^12^ and leading members have recently called for “gene synthesis companies, science and technology funders, policymakers, and the scientific community as a whole to continue to minimize risk and maximize the safety and security of DNA synthesis” and to develop “effective regulatory and technological frameworks” to manage risk.^17,18^

In this study, we empirically document the potential variation in screening results due to subjective interpretation of regulatory and guidance language using Battelle's UltraSEQ tool^19^ and publicly available synthetic sequences. Based on the results of this study, we define foundational risk levels that can be used by nucleic acid synthesis vendors for categorizing biological sequences to triage sequences ordered by customers.

## Methods

### Test Sequence Selection

Nucleotide sequences annotated as synthetic constructs (TaxID 32630), originating from genomic DNA/RNA, and of length 50–5,000 nts were downloaded from NCBI (*n* = 430,498) on October 12, 2023. The MMSeqs2^20^ *easy-cluster* function was used (*settings—min-seq-id 0.9 -c 0.9—cov-mode 1—dbtype 2*) to cluster highly similar sequences, reducing redundancy bias and computational burden, resulting in 141,651 sequences for testing. A text file of all NCBI accessions is provided in the Supplementary Data S1.

### UltraSEQ Threat Flagging

The sequences were run through the core UltraSEQ engine,^19^ skipping sample genomics preprocessing and downstream metagenomics services. The standard UltraSEQ databases were utilized, including the comprehensive UniRef100 database, the SoC database, a pathogen-specific nucleotide database, and internal ribosome entry site sequences. As described in Gemler et al.,^19^ UltraSEQ automatically identifies regions within a sequence through the Query Mapper Service and applies a threat score for each region (i.e., subsequence) identified using a logical rules engine.

Specifically for gene screening, UltraSEQ's Gene Screening Rules Engine service was created to assign a risk level associated with each sequence *region* (Table 2), and the overall risk level associated with the entire sequence was set to be the “worst” (i.e., lowest numerical value) risk level across all of that sequence's regions. Risk levels can be further divided into those that are controlled and not controlled as defined in the Introduction section.

**Table 2.** UltraSEQ gene screening rules engine description and conditions

***Region risk level***	***Region description***	***Region conditions (all conditions must be met)***
1a	Export controlled and FSAP controlled: Toxin	Region length >L_min_ Threat prediction confidence >C_min_: [FSAP Toxin^a^]
1b	Export controlled: Tier 1 Virus or SoC that transfers virulence	Region length >L_min_ Unique to Select Agent^b^ Threat prediction confidences >C_min_: [Tier 1 Agent] AND ([Virus] OR [SoC that Transfers Virulence])
2	Export controlled: Virus or SoC that transfers virulence	Region length >L_min_ Unique to Select Agent^b^ Threat prediction confidences >C_min_: ([Virus] OR [SoC that Transfers Virulence])
3	Possibly export controlled: SoC from non-virus Select Agent	Region length >L_min_ Unique to (non-virus) Select Agent^b^ Threat prediction confidences >C_min_: [SoC]
4	Not export controlled: non-SoC from Select Agent	Region length >L_min_ Unique to (non-virus) Select Agent^b^
5	Not export controlled: SoC from non-Select Agent	Region length >L_min_ Threat prediction confidences >C_min_: [SoC]
6	None of the above	None of the above conditions

^a^ A protein toxin is composed of one or more subunits, each of which may have one or more chains. UltraSEQ is capable of identifying whether all chains and subunits are present. This threat condition triggers if any toxin component is detected.

^b^ UltraSEQ considers a region to be unique to a select agent if the best match is unique to a select agent OR if at least half the number of TaxID predictions for the region originate from select agents.

FSAP, Federal Select Agent Program.

Each risk level has a set of conditions that are related to *length, uniqueness,* and *homology*. *Length* is the simple threshold L_min,_ defined as the minimum number of bases required to meet the condition. *Uniqueness* is defined by whether the region is unique to a controlled agent; specifically, if the best match is unique to a select agent or if at least half the predicted TaxIDs in the region are select agents. *Uniqueness* captures the principles of “best match” discussed in regulatory guidance by comparing the alignment strength of a sequence with various databases defined earlier.

Finally, *homology* is defined by the threshold C_min_, the minimum UltraSEQ confidence score mapping to a known threat contained in the subject alignment database, which is equal to *percent identity × percent region coverage* as described in Gemler et al.^19^

Known threats are defined either at the taxonomy level (for viruses, risk levels 1 and 2), specific SoC level, or SoC functional level. Risk levels associated with specific SoCs include controlled toxins (risk level 1) and SoCs that are known to transfer virulence (risk levels 1 and 2). For the latter, SoCs that “transfer virulence” are defined as having documented evidence in literature of the SoC being expressed in another organism to enhance or endow pathogenicity.

For the remaining risk levels associated with SoCs, the confidence scores are associated with SoC metadata, which can come from more than one sequence contained in the subject database. Furthermore, functional metadata are limited to those associated with the following threat function categories: Inhibits host cell death, motility, passive host subversion, active host subversion, damage, host cell apoptosis, prion, and transmission, as described previously in Gemler et al.^15^

## Results

### Sensitivity Study Design

As described in the Introduction, screening guidelines and regulatory language are evolving and interpretation can be somewhat subjective. With the understanding in mind that not all sequences are equally threatening, we rationalized the risk levels described in Table 2. UltraSEQ's default parameters for these risk levels include L_min_ ≥ 50 nts for all risk levels (based on the updated HHS Guidance) and that the sequence must be unique to a select agent for risk level 1b-4 (based on regulatory language).

For C_min_, we rationalized that 0.2 is a good foundational value based on the report described by Suzek et al.^21^ in which the authors demonstrated that only 0.62% of UniRef50 cluster members have inconsistent gene ontology terms. UniRef50 clusters bring together sequences of at least 45% protein matching identity and 64% coverage, corresponding to a confidence of 28.8% (multiplying the two values together). Given the functional equivalency of UniRef50 cluster sequences, we conservatively round down 28.8 to 20% (or 0.2) as C_min_. To assess the impact of varying UltraSEQ parameters from these default parameters, the following sensitivity analyses were performed:

- *Impact of minimum region length*. Vary L_min_ from 10 to 200 with step size 10.
- *Impact of specific to select agent uniqueness*. Vary L_min_ from 10 to 200 with step size 10 without unique to select agent condition.
- *Impact of UltraSEQ confidence*. Vary C_min_ from 0.1 to 1 with step size 0.1 at L_min_ = 200 and L_min_ = 50.

To test these variations, nucleotide sequences annotated as synthetic constructs (TaxID 32630), originating from genomic DNA/RNA, and of length 50–5,000 nts were downloaded from NCBI, clustered to remove redundancy, and run through UltraSEQ as described in the Methods section. We rationalized this to be a representative data set of gene orders based on the fact that they are annotated as synthetic constructs from a variety of laboratories across the world. We found that the risk level breakdown of the data set was similar to real orders screened through Battelle's ThreatSEQ™^22^ order stream (data not shown).

### Data Set Baseline Analysis and Impact of Minimum Region Length on Risk Levels

**Figure 1.**
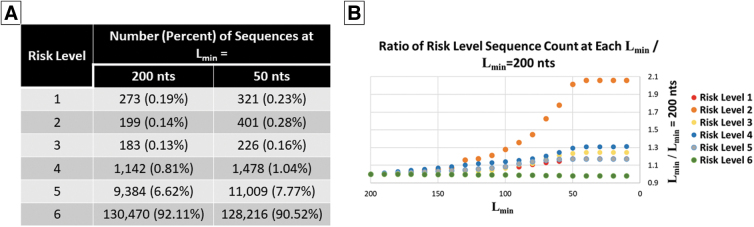
Impact of L_min_ on risk level flagging. (A) Number of sequences flagged at each risk level for L_min_ = 200 and 50 nts. (B) Ratio of sequences flagged at each risk level for every L_min_ compared with L_min_ = 200 nts.

The stratification of results by risk level is shown in Figure 1 for UltraSEQ's default parameters (50 nts, C_min_ = 0.2, and uniqueness included). Overall, 90.52% of sequences fell into risk level 6 (“no risk,” i.e., no human review required). HHS's recent update to screening guidance suggests expansion of SoCs from select agents only (risk levels 1–4) to any SoC that can contribute to pathogenicity. Based on our results for this data set, this expansion could result in an additional 7.77% of total sequences or a 4.5-fold increase (compared with risk levels 1–4 only) that would require follow-up with customer.

HHS's recent screening guidance update suggests updating the window in which tools screen regions of sequences from 200 to 50 nts or lower.^13^ To understand the impact of lowering this threshold, L_min_ was varied from 10 to 200 nts with a step size of 10 nts using the C_min_ default value of 0.2. The ratio of the number of sequences at each risk level for each step was compared with the number of sequences when L_min_ = 200 to understand the magnitude of impact (Figure 1).

As shown in Figure 1a, >90% of sequences fell into risk level 6, and <1% of sequences were flagged as being definitely or possibly export controlled (risk level 1, 2, or 3) at both L_min_ = 50 and 200 nts. For all risk levels, the largest relative shifts were apparent when the L_min_ dropped below 100 nts and levels off at around 40 nts (Figure 1b). The risk level with the largest relative change at L_min_ = 50 compared with L_min_ = 200 is risk level 2, with >100% increase.

However, 156/202 such sequences are <200 nts in length, thus the effective increase in risk level 2 sequences is only 23% when removing these sequences from the calculation. Similarly, but to a lesser extent, risk levels 1, 3, 4, and 5 increase by ∼17–30%. In contrast, risk level 6 decreased by only 2%, but this represents ∼2,600 sequences that were re-characterized from the “no risk” level 6 to at least some risk (i.e., risk levels 1 through 5).

**Figure 2.**
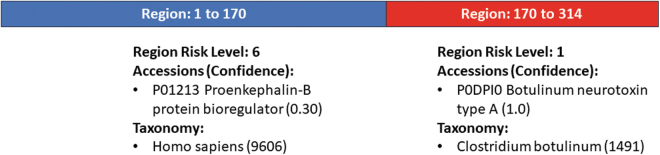
Visualization of UltraSEQ analysis of HW266136.1. Shown are the top level metadata for each region. Additional metadata, including legitimate use case information, are provided in UltraSEQ reports as shown in the Supplementary Data S2.

For risk level 1, 273 sequences were flagged when L_min_ = 200, and an additional 48 sequences (321 total) were flagged when L_min_ = 50. Notably, these sequences went from risk level 5 or 6 at L_min_ = 200 to risk level 1 at L_min_ = 50. Analysis of these sequences confirmed that the shift was due to the sequences being <200 nts or multiple regions being identified by UltraSEQ, and that the risk level 1 flagged region was <200 nts. A demonstration of one such sequence, HW266136.1, a 314 nts sequence, is shown in Figure 2.

For this sequence, UltraSEQ detected one relevant coding frame containing two regions. The first region (170 nts long) aligned to a sequence annotated as a human dynorphin peptide (further discussed in the discussion section), which is consistent with the annotation contained in the GenBank record (“Dynorphin binding domain,” https://www.ncbi.nlm.nih.gov/nuccore/HW266136.1/). The second region aligned to botulinum neurotoxin, which suggests the sequence may be controlled (risk level 1). Because the region is only 144 nts long, the sequence was not flagged when L_min_ = 200, but was flagged at L_min_ = 50.

Although the determination of control status would require additional investigation to determine if its translated product is functional, this sensitivity analysis suggests that reducing the L_min_ from 200 to 50 does not cause a large increase in the number of sequences with high risk levels and may flag potentially threatening sequences that would otherwise not get reviewed if L_min_ = 200.

### Impact of Specific to Select Agent Uniqueness on Risk Levels

To understand the impact of “uniqueness,” we further explored the impact of including uniqueness of the sequence to a select agent as a risk level criteria. Specifically, using a C_min_ value of 0.2 and L_min_ values of 50 and 200 nts, risk levels were computed without considering the uniqueness to select agent condition in the Gene Screening Rules Engine (Table 3). As shown in the table, the number of sequences flagged at each L_min_ level do not change significantly whether considering uniqueness to a select agent or not. For example, risk level 6 sequences change by <0.2%, confirming the UltraSEQ baseline screening conditions for uniqueness as a rational condition that would not introduce significant, if any, false negatives.

**Table 3.** Evaluation of the impact of not considering uniqueness to a Select Agent on risk level sequence counts (percentage)

	***Region description***	***Risk level 1***	***Risk level 2***	***Risk level 3***	***Risk level 4***	***Risk level 5***	***Risk level 6***
L_min_ = 50 nts	Uniqueness considered	321 (0.23%)	401 (0.28%)	226 (0.16%)	1,478 (1.04%)	11,009 (7.77%)	128,216 (90.52%)
	Uniqueness not considered	321 (0.23%)	402 (0.28%)	239 (0.17%)	1,752 (1.24%)	10,965 (7.74%)	127,972 (90.34%)
L_min_ = 200 nts	Uniqueness considered	273 (0.19%)	199 (0.14%)	183 (0.13%)	1,142 (0.81%)	9,384 (6.62%)	130,470 (92.11%)
	Uniqueness not considered	273 (0.19%)	200 (0.14%)	189 (0.13%)	1,266 (0.89%)	9,363 (6.61%)	130,360 (92.03%)

### Impact of UltraSEQ Confidence on Risk Levels

Region length and uniqueness to select agents are variables that are both easy to understand and interpret, especially when the query sequence is a 100% match to a known threat. However, most flagged sequences have homology to a known threat that is <100%. To evaluate the impact of homology on risk levels, we leveraged the UltraSEQ confidence value, which is a measure of percent identity x percent region coverage length as described in the Methods section.

**Figure 3.**
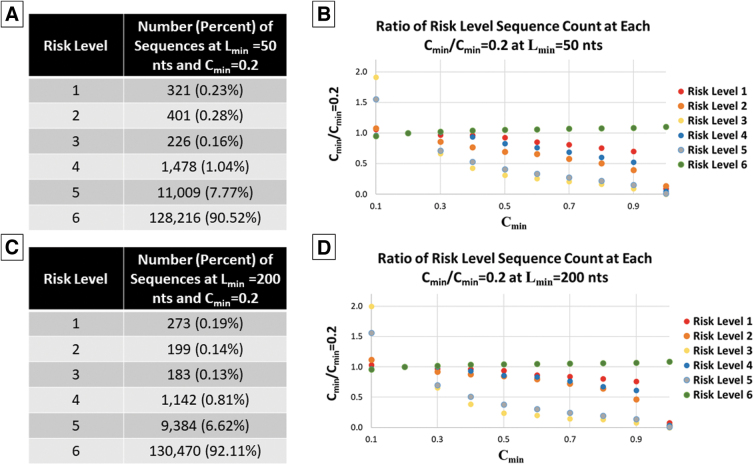
Impact of C_min_ at L_min_ = 50 and 200 nts. (A) Number of sequences flagged at each risk level for C_min_ = 0.2 and L_min_ = 50 nts. (B) Ratio of sequences flagged at each risk level at L_min_ = 50 nts compared with C_min_ = 0.2. (C) Number of sequences flagged at each risk level for C_min_ = 0.2 and L_min_ = 200 nts. (D) Ratio of sequences flagged at each risk level at L_min_ = 200 nts compared with C_min_ = 0.2.

Specifically, using L_min_ values of 50 and 200 nts, C_min_ was varied from 0.1 to 1.0 with a step size of 0.1. The total number of sequences flagged at each risk level when C_min_ = 0.2 is shown in Figure 3 (panel A when L_min_ = 50 nts and panel C when L_min_ = 200 nts). The ratio of flagged sequences at each C_min_ to C_min_ = 0.2 was then computed for each C_min_ (Figure 3, panels B and D) to understand relative impacts.

As expected, the number of sequences flagged at risk levels 1–5 decreases as C_min_ increases, whereas the number of sequences in risk level 6 increases. The largest impact is apparent for risk level 3, in which >50% of sequences were no longer flagged at risk level 3 when C_min_ changes from 0.2 to 0.5. A similar trend is apparent for risk level 5. Similarly, but to a lesser extent, for risk levels 1, 2, and 4, this decrease was <50% when C_min_ increases to 0.9, then sharply decreased to near 100% when C_min_ shifts from 0.9 to 1.0.

In contrast to the minimum length and uniqueness sensitivity analysis, risk level 6 changed by 5.5% when C_min_ changes from 0.2 to 0.5, representing 7,050 additional sequences that would not be flagged for secondary review. If we instead considered changes in flagging from C_min_ = 0.1–0.5, the number of risk level 6 sequences increases by 13,332 (10.9%), largely driven by differences in risk levels 3 and 5 from C_min_ = 0.2–0.1.

## Discussion

Striking a balance between biosafety and biosecurity (avoiding synthesis of a harmful product or a product that could be subject to misuse) and economic margin (minimizing the costs associated with screening) is imperative for the future of the bioeconomy. However, standardized biosecurity screening mechanisms for synthetic biology are lacking in part due to subjective language in both guidance documents and regulation. The results of this study provide empirical evidence of the impact of the interpretation and updates to regulatory and guidance language for screening synthetic nucleic acids that providers can leverage when trying to strike this balance.

Results using our UltraSEQ software show that homology (C_min_) had the greatest impact on number of sequences flagged, followed by region length (L_min_) and, in a distant third place, uniqueness to a controlled agent. Furthermore, our results demonstrate that flagging SoCs that do not align to select agent sequences (as recommended by HHS's updated guidance) results in a 4.5-fold increase in the number of sequences being flagged for the data set used in this study. We note that, in this study, we leverage our SoC database,^15^ which objectively defines SoCs based on threat functions and is specific to sequencing screening goals.

As the number of SoCs within our or databases used by other tools increases, so too will the number of flagged sequences (risk level 5 for UltraSEQ). This reality creates a paradox in screening incentives—the more expansive a SoC database, the more sequences will be flagged for review under the updated HHS guidance. Further refinement in regulatory guidance around which SoCs should be flagged and how to evaluate the degree of biological risk associated with specific SoCs may reduce this relative increase and is critical for future screening guidance.

Although these parameters are unique to UltraSEQ and the results are based on one large publicly available data set whose results mimicked real order stream, we presume that the results are generally applicable to other sequence screening tool providers and data sets. However, similar studies should be performed with multiple tools and relevant data sets, including data sets containing novel sequences, and the results compared and contrasted to achieve the most actionable results possible.

Although screening sequences is critical to ensure proper biosecurity, the results should be taken into consideration with what is known of the customer. In this study, we provide foundational risk levels that can be adopted by nucleotide synthesis vendors when screening orders and customers. The risk levels described in this article represent an objective framework to measure and rank how “risky” a sequence is, where risk encompasses both regulatory status and threat function, which when considered alongside customer identity can be used to make informed decisions about whether to trigger an investigation.

For example, risk level 1 sequences may be less of a concern when ordered from a reoccurring domestic customer licensed under FSAP compared with a new foreign customer with no publications covering similar work.

This risk level framework provided in this study could be adapted by stakeholders such as the IGSC when updating the Harmonized Screening Protocol,^12^ academics when coordinating research activities,^23^ biosafety and biosecurity practitioners, and the U.S. Government agencies when establishing a frameworks for “comprehensive, scalable, and verifiable…screening mechanisms” and regulation of biotechnology in response to the recent Executive Orders on the Safe, Secure, and Trustworthy Development and Use of Artificial Intelligence (AI)^24^ and Advancing Biotechnology and Biomanufacturing Innovation for a Sustainable, Safe, and Secure American Bioeconomy,^25^ respectively.

The results provided by UltraSEQ in this empirical study provide a foundation to build upon as the threat landscape evolves. Although gene screening is becoming more straightforward, many synthetic nucleotide orders are becoming longer and more complex, thus requiring screening tools to evolve as well. Perhaps complicating matters further, AI algorithms that can design entirely novel protein sequences are emerging.^24^ Hence, the updated HHS Guidance suggests the possibility of using “predictive bioinformatic algorithms to screen sequences that are not a match to any known sequences” to identify SoCs.^13^

Further yet, new noninfectious disease biological threats are emerging, such as gene editing tools, gene circuits to recombinantly produce illicit drugs,^15^ and bioregulators. In this study, we identify one such bioregulator (Figure 2) in a sequence that UltraSEQ automatically identified as a chimeric sequence with two regions, a region containing a dynorphin peptide bioregulator component and a botulinum toxin gene component. Dynorphin peptides are known neuroactive peptides that interact with opioid receptors^,26^ and thus may change (enhance perhaps) the toxicity of the toxin component.

This example underscores the need for sequence screening tools to both be able to understand complex multi-region (and multi-coding-frame) sequences and predict a use case context across regions in a human-actionable manner. Additional development is needed to build tools that connect predictions across sequences and across orders to build robust contextual use case predictions.

## Authors' Contributions

B.T.G.: conceptualization, methodology, investigation, software, results generation, and writing; C.B.: conceptualization, methodology, investigation, writing, review, and overall supervision/project administration; J.D. and C.M.: conceptualization, methodology, and critical review/editing; and P.A.F.: software and results generation/validation.

## Authors' Disclosure Statement

The authors declare no competing financial interests.

## Funding Information

This study was supported by Battelle internal funding.

## Supplementary Material

Supplementary Data S1

Supplementary Data S2
