# Supplementary material for: A Sensitivity Study for Interpreting Nucleic Acid Sequence Screening Regulatory and Guidance Documentation: Toward a Foundational Synthetic Nucleic Acid Sequence Screening Framework
Source: Appl Biosaf. 2024 Sep 18;29(3):150–8. doi: 10.1089/apb.2023.0026 (PMC11447129; doi:10.1089/apb.2023.0026)
Supplement: Supplementary Data S1 [file apb.2023.0026_suppl_datas1.pdf]

**Supplementary Material: NCBI nucleotide accessions used as the test dataset:**

| | | | | | | | | |
|---|---|---|---|---|---|---|---|---|
| HI378652.1 | HV512152.1 | HV512194.1 | DL109062.1 | HC490879.1 | HC294696.1 | DD224665.1 | DL113794.1 | CQ982974.1 |
| HI378460.1 | HV512120.1 | HV512162.1 | DL109030.1 | HC490839.1 | HC291174.1 | DD224213.1 | DL113730.1 | CQ982835.1 |
| HI378364.1 | HV491628.1 | HV512130.1 | DL108998.1 | HC490807.1 | HC290994.1 | DD223907.1 | DL113754.1 | CQ982684.1 |
| HI378332.1 | HI378110.1 | HV504793.1 | DL104296.1 | HC490775.1 | HC293577.1 | DD234124.1 | DL113698.1 | CQ982606.1 |
| HI378140.1 | HI377257.1 | HV504761.1 | DL104264.1 | HC490743.1 | HC290617.1 | DD231493.1 | DL108865.1 | CQ981126.1 |
| HI378108.1 | HI376151.1 | HV504697.1 | DL104232.1 | HC490711.1 | HC290169.1 | DD231445.1 | DL108833.1 | CQ975464.1 |
| HI377255.1 | HI375901.1 | HV504665.1 | DL099562.1 | HC490679.1 | HC290137.1 | DD231413.1 | DL108801.1 | CQ974381.1 |
| HI376818.1 | HI371022.1 | HV504633.1 | DL099530.1 | HC490647.1 | HC289878.1 | CS276982.1 | DL108769.1 | CQ973137.1 |
| HI376297.1 | HI369088.1 | HV504601.1 | DL093456.1 | HC490615.1 | HC289844.1 | DD219623.1 | DL108737.1 | CQ972530.1 |
| HI373905.1 | HI424148.1 | HV504984.1 | DL093424.1 | HC490583.1 | HC207907.1 | DD219540.1 | DL104059.1 | CQ977468.1 |
| HI373102.1 | HI424116.1 | HV503838.1 | DL093392.1 | HC490550.1 | HC204111.1 | DD221334.1 | DL104027.1 | CQ976006.1 |
| HI371016.1 | HI424084.1 | HV503806.1 | DL093360.1 | HC490515.1 | HC199297.1 | DD216646.1 | DL099493.1 | CQ975972.1 |
| HI370487.1 | HI424052.1 | HV491261.1 | DL126137.1 | HC490483.1 | DM464963.1 | DD213881.1 | DL099461.1 | CQ975667.1 |
| HI369421.1 | HI423826.1 | HV038646.1 | DL126105.1 | HC490412.1 | DM463865.1 | DD213849.1 | DL099429.1 | CQ972398.1 |
| HI369242.1 | HI416452.1 | HV182346.1 | DL122753.1 | HC490284.1 | HC196125.1 | DD213817.1 | DL099397.1 | CQ972366.1 |
| HI369129.1 | HI414051.1 | HV182314.1 | DL122665.1 | HC472305.1 | HC195232.1 | DD213768.1 | DL099365.1 | CQ972334.1 |
| HI369086.1 | HI414019.1 | HV182282.1 | DL122569.1 | HC472272.1 | HC190851.1 | DD213736.1 | DL099333.1 | CQ971132.1 |
| HI067154.1 | HI413610.1 | HV190951.1 | DL118379.1 | HC472240.1 | HC187612.1 | HC007681.1 | DL093323.1 | CQ971067.1 |
| HI424914.1 | HI413574.1 | HV216210.1 | DL118347.1 | HC472208.1 | HC187068.1 | HC007589.1 | DL093291.1 | CQ970879.1 |
| HI424146.1 | HH822080.1 | HV228415.1 | DL113823.1 | HC471944.1 | HC089584.1 | HC007557.1 | DL089548.1 | CQ969055.1 |
| HI424114.1 | HH821886.1 | HV306496.1 | DL113791.1 | HC471912.1 | HC089552.1 | HC007525.1 | DL089516.1 | CQ967572.1 |
| HI424082.1 | FW369984.1 | HV037785.1 | DL113751.1 | HC471880.1 | HC089520.1 | HC010738.1 | DL089484.1 | CQ964035.1 |
| HI423856.1 | FW375268.1 | CS124658.1 | DL041927.1 | HC471857.1 | HC089488.1 | DM376697.1 | DL089452.1 | CQ963551.1 |
| HI423824.1 | U84739.1 | CS124626.1 | DL038008.1 | HC678782.1 | HC089456.1 | DM375482.1 | DL089420.1 | CQ955898.1 |
| HI423792.1 | HD062028.1 | CS124578.1 | DL069453.1 | HC678459.1 | HC089424.1 | DM370763.1 | DL089388.1 | CQ947125.1 |
| HI423371.1 | J02547.1 | CQ972382.1 | DL049712.1 | HC668135.1 | HC089391.1 | DM370665.1 | DL113618.1 | CQ945557.1 |
| HH762738.1 | M15672.1 | CQ972350.1 | DL049680.1 | HC521221.1 | HC089359.1 | DM370633.1 | DL113586.1 | CQ944195.1 |
| HI416368.1 | AY292199.1 | CQ972318.1 | DL045672.1 | HC509933.1 | HC089326.1 | DM370601.1 | DL113554.1 | CQ944163.1 |
| HI415923.1 | FW342479.1 | CQ971100.1 | DL041857.1 | HC504598.1 | HC089294.1 | DM370569.1 | DL113522.1 | CQ944131.1 |
| HI415891.1 | FW341732.1 | CQ970332.1 | DL041825.1 | HC494555.1 | HC089262.1 | DM370537.1 | DL113458.1 | CQ944099.1 |
| HI415859.1 | FW337274.1 | CQ947141.1 | DL041793.1 | HC494423.1 | HC086254.1 | DM370505.1 | DL108561.1 | CQ944067.1 |
| HI415826.1 | HC874691.1 | CQ947024.1 | DL041761.1 | HC504194.1 | HC085662.1 | DM370473.1 | DL108529.1 | CQ944035.1 |
| HI415794.1 | HC882487.1 | CQ944179.1 | DL041729.1 | HC501173.1 | AY775009.1 | DM367392.1 | DL108497.1 | CQ943971.1 |
| HI415762.1 | HC880625.1 | CQ944147.1 | DL041697.1 | HC500314.1 | DM002009.1 | DM371968.1 | DL103763.1 | CQ943939.1 |
| HI415319.1 | HC880043.1 | CQ944115.1 | DL037778.1 | HC499868.1 | DM001829.1 | DM371859.1 | DL103731.1 | CQ943907.1 |
| HI414049.1 | HC491067.1 | CQ944083.1 | DL049639.1 | FW300964.1 | DM001678.1 | DM209382.1 | DL093147.1 | CQ943875.1 |
| GM643362.1 | HC488151.1 | CQ944051.1 | DL049607.1 | FW300625.1 | DM001472.1 | HC001557.1 | DL049433.1 | A04616.1 |
| GM643330.1 | FV533452.1 | CQ944019.1 | DL049511.1 | FW298779.1 | DM006955.1 | HB865367.1 | DL049401.1 | CQ898780.1 |
| GM643298.1 | FV531695.1 | CQ943987.1 | DL049479.1 | HC470492.1 | DM004994.1 | HB865023.1 | DL049369.1 | CQ898649.1 |
| GM643266.1 | FV530902.1 | CQ943955.1 | DL041560.1 | HC477910.1 | DM003499.1 | HB864981.1 | DL049337.1 | DL120539.1 |
| GM643234.1 | FV522793.1 | CQ943923.1 | DL041528.1 | HB463765.1 | DM002794.1 | HB864983.1 | DL049210.1 | DL120475.1 |
| GM636245.1 | FV534005.1 | CQ943891.1 | DL037673.1 | HB463597.1 | GM998416.1 | HB864927.1 | DL049178.1 | DL115912.1 |
| GM636213.1 | HC461723.1 | CQ943859.1 | DL037641.1 | HB469342.1 | GM996588.1 | HB864895.1 | DL049146.1 | DL115880.1 |
| GM636181.1 | AF537267.1 | CQ898813.1 | DL037609.1 | HB469101.1 | GN010005.1 | HB864863.1 | DL049114.1 | DL115848.1 |
| GM636149.1 | HC453692.1 | CQ898633.1 | DL037577.1 | HB468038.1 | GM994762.1 | HB864831.1 | DL049082.1 | DL106490.1 |
| GM636117.1 | HC453660.1 | CQ898601.1 | DL037545.1 | DM152899.1 | GM992942.1 | HB864799.1 | DL049050.1 | DL106458.1 |

| | | | | | | | | |
|---|---|---|---|---|---|---|---|---|
| GM636085.1 | AF004665.1 | CQ898569.1 | DL037513.1 | DM152699.1 | GM992853.1 | HB864767.1 | DL012574.1 | DL106426.1 |
| GM631677.1 | HC449030.1 | CQ888103.1 | DL033310.1 | DM152553.1 | GM992468.1 | HB864251.1 | DL012542.1 | DL128437.1 |
| GM631645.1 | FU761089.1 | CQ887720.1 | DL033278.1 | DM155839.1 | GM992103.1 | HB866967.1 | DL012510.1 | DL124679.1 |
| GM631613.1 | FU759903.1 | CQ877617.1 | DL033246.1 | DM150252.1 | GN000592.1 | HB859184.1 | DL012478.1 | DL124647.1 |
| GM631581.1 | FU758659.1 | CQ875011.1 | DL033214.1 | DM150088.1 | GM969700.1 | HB866514.1 | DL012446.1 | DL124615.1 |
| GM631549.1 | FU764207.1 | CQ874860.1 | DL033182.1 | DM149922.1 | GM969394.1 | HB866100.1 | DL012414.1 | DL124583.1 |
| GM631517.1 | FU763390.1 | CQ871402.1 | DL033150.1 | HB461398.1 | GM969722.1 | HC000747.1 | DL045424.1 | DL096918.1 |
| GM631485.1 | FU762514.1 | CQ868890.1 | DL033118.1 | HB460458.1 | GM980879.1 | GN045764.1 | DL045392.1 | DL096886.1 |
| GM622714.1 | GN360000.1 | CQ867380.1 | DL029110.1 | HB455309.1 | GM984675.1 | GN042718.1 | DL041248.1 | DL096854.1 |
| GM622682.1 | GN359731.1 | CQ859834.1 | DL029078.1 | HA640312.1 | FB986362.1 | GN034283.1 | DL041216.1 | DL096790.1 |
| GM622650.1 | GN359699.1 | CQ857857.1 | DL029046.1 | HB445380.1 | HW104222.1 | GN038570.1 | DL041184.1 | DL096758.1 |
| GM622618.1 | GN359679.1 | AX145555.1 | DL029014.1 | HB444512.1 | HW104150.1 | GN034789.1 | DL041120.1 | DL146281.1 |
| GM622586.1 | GN359647.1 | AX145523.1 | DL028982.1 | DM140220.1 | HW104110.1 | GN030748.1 | DL041088.1 | DL124471.1 |
| GM622554.1 | GN359583.1 | AX145491.1 | DL028950.1 | DM138463.1 | HW103992.1 | GN030716.1 | DL037201.1 | DL124439.1 |
| GM740803.1 | GN359551.1 | AX145459.1 | DL024976.1 | DM138431.1 | HW070619.1 | GN030652.1 | DL037169.1 | DL124407.1 |
| GM704186.1 | DL040849.1 | AX145427.1 | DL021764.1 | DM137786.1 | HW099608.1 | GN030620.1 | DL037137.1 | DL124375.1 |
| GM657732.1 | DL040817.1 | AX145395.1 | DL021732.1 | DM137119.1 | HW069649.1 | GN030588.1 | DL028734.1 | DL120286.1 |
| GM657668.1 | DL040785.1 | AX145362.1 | DL021668.1 | DM131555.1 | HW069223.1 | GN030556.1 | DL028702.1 | DL120254.1 |
| GM657636.1 | DL036866.1 | AX145330.1 | DL021636.1 | DM131146.1 | HW089208.1 | GN030524.1 | DL028670.1 | DL120222.1 |
| GM657604.1 | DL036834.1 | AX145298.1 | DL017398.1 | HB432759.1 | HW102280.1 | GN030460.1 | DL028638.1 | DL115819.1 |
| GM657572.1 | DL036802.1 | AX145266.1 | DL017366.1 | HB427176.1 | HW067348.1 | GN030429.1 | DL028606.1 | DL115787.1 |
| GM650380.1 | DL036770.1 | AX145234.1 | DL017334.1 | HB427001.1 | HW066660.1 | GN030268.1 | DL028574.1 | DL115755.1 |
| GM650348.1 | DL036738.1 | AX145202.1 | DL012977.1 | DL232998.1 | HW083197.1 | GN030236.1 | DL024760.1 | DL115723.1 |
| GM650316.1 | DL036706.1 | AX145170.1 | DL019479.1 | GN075951.1 | HW088266.1 | GN030204.1 | DL024728.1 | DL115691.1 |
| GM650284.1 | DL032503.1 | AX145138.1 | DL019447.1 | L08950.1 | HW097395.1 | GN030172.1 | DL024696.1 | DL115659.1 |
| GM650252.1 | DL032471.1 | AX145074.1 | DL014916.1 | L08878.1 | HW087876.1 | GN030140.1 | DL016894.1 | DL111233.1 |
| GM650220.1 | DL032439.1 | AX145042.1 | DL014884.1 | DM058851.1 | HW081688.1 | GN030108.1 | DL016862.1 | DL111201.1 |
| GM650188.1 | DL032407.1 | AX145010.1 | DL030383.1 | DM058785.1 | HW085175.1 | GN029850.1 | DL016830.1 | DL111169.1 |
| GM622522.1 | DL032375.1 | AX144978.1 | DL026470.1 | DM063066.1 | HW099460.1 | GN029818.1 | DL012372.1 | DL111105.1 |
| GM622490.1 | DL032343.1 | AX144946.1 | DL026438.1 | DM041595.1 | HW103524.1 | GN013379.1 | DL060760.1 | DL111073.1 |
| GM622458.1 | DL024158.1 | AX144913.1 | DL026374.1 | DM045535.1 | HW103413.1 | GN033552.1 | DL048693.1 | DL109029.1 |
| GM622426.1 | DL024126.1 | AX144881.1 | DL022562.1 | DM045254.1 | HW098201.1 | GN033520.1 | DL048661.1 | DL108997.1 |
| GM622394.1 | DL024094.1 | AX144849.1 | DL022530.1 | DM044976.1 | HW102322.1 | GN033488.1 | GM644791.1 | DL104295.1 |
| GM622362.1 | DL024062.1 | AX144817.1 | DL014649.1 | DM039611.1 | HW061885.1 | GN033456.1 | GM644759.1 | DL104263.1 |
| GM631478.1 | DL024030.1 | AX144785.1 | DL010174.1 | DM055895.1 | HW061806.1 | GN033424.1 | GM644727.1 | DL104231.1 |
| GM631446.1 | DL023998.1 | AX144753.1 | DL010142.1 | GN067894.1 | HW065235.1 | GN033360.1 | GM637601.1 | DL099593.1 |
| GM631414.1 | DL020791.1 | AX144721.1 | DL010110.1 | GN067862.1 | HW058492.1 | GN033328.1 | GM637569.1 | DL099561.1 |
| GM631382.1 | DL016388.1 | AX144689.1 | DL030840.1 | GN067798.1 | HW064836.1 | GN033296.1 | GM637537.1 | DL093455.1 |
| GM631350.1 | DL016356.1 | AX144657.1 | DL030808.1 | GN067770.1 | HW061121.1 | GN033232.1 | GM637505.1 | DL093423.1 |
| GM657516.1 | DL016324.1 | AX144625.1 | DL030776.1 | GN065233.1 | HW056578.1 | GN033200.1 | GM637473.1 | DL093391.1 |
| GM657484.1 | DL016292.1 | AX144509.1 | DL030744.1 | GN063422.1 | HV444463.1 | GN033168.1 | GM633065.1 | DL093359.1 |
| GM657452.1 | DL016260.1 | AX144317.1 | DL019756.1 | GN052462.1 | HV444105.1 | GN033136.1 | GM633033.1 | DL126136.1 |
| GM657420.1 | DL016228.1 | AX144187.1 | DL019724.1 | GN052430.1 | HV437899.1 | GN033072.1 | GM632969.1 | DL126104.1 |
| GM657388.1 | DL028321.1 | AX144123.1 | DL015193.1 | GN052387.1 | HV437582.1 | GN033008.1 | GM632937.1 | DL122752.1 |
| GM657356.1 | DL028289.1 | AX143993.1 | DL015161.1 | GN051435.1 | HV445405.1 | GN032976.1 | GM632905.1 | DL122696.1 |
| GM650164.1 | DL028257.1 | AX143929.1 | DL015033.1 | DM025357.1 | HV349428.1 | GN032944.1 | GM625294.1 | DL122664.1 |
| GM650132.1 | DL028225.1 | AX143865.1 | DL010543.1 | DM028073.1 | HV341315.1 | GN032912.1 | GM625262.1 | DL122568.1 |

| | | | | | | | | |
|---|---|---|---|---|---|---|---|---|
| GM650100.1 | DL028193.1 | AX143801.1 | DL010511.1 | DM022273.1 | HV343808.1 | GN032880.1 | GM625230.1 | DL118378.1 |
| GM650068.1 | DL011776.1 | AX143481.1 | DL010479.1 | DM022201.1 | HV343616.1 | GN032848.1 | GM625198.1 | DL118346.1 |
| GM650036.1 | DL011744.1 | AX143417.1 | DL010447.1 | GM952244.1 | HV343472.1 | GN032816.1 | GM625166.1 | DL113822.1 |
| GM650004.1 | DL011712.1 | AX143289.1 | DL010415.1 | GM963579.1 | HV347855.1 | GN032784.1 | GM625134.1 | DL113790.1 |
| GM643196.1 | DL011680.1 | AX143097.1 | CS631293.1 | GM889323.1 | HV347597.1 | GN032753.1 | GM624044.1 | DL113750.1 |
| GM643164.1 | DL011648.1 | AX143033.1 | CS631214.1 | GM008866.1 | HV344980.1 | GN032721.1 | GM624012.1 | DL113694.1 |
| GM643131.1 | DL011616.1 | AX142969.1 | CS642183.1 | GM008180.1 | HV344800.1 | GN032689.1 | GM623980.1 | DL113662.1 |
| A13388.1 | DL048631.1 | AX142841.1 | CS627760.1 | GM006056.1 | HV347091.1 | GN032657.1 | GM623948.1 | DL108861.1 |
| A12755.1 | DL048599.1 | AX142649.1 | CS632378.1 | GM005888.1 | HV344716.1 | GN032624.1 | GM625122.1 | DL108829.1 |
| A12086.1 | DL048567.1 | AX142585.1 | CS623655.1 | GM867490.1 | HV339960.1 | GN032592.1 | GM625090.1 | DL108797.1 |
| A11245.1 | DL048535.1 | AX142521.1 | CS623597.1 | GM685526.1 | HV339896.1 | GN032560.1 | GM625058.1 | DL108765.1 |
| A10850.1 | DL048503.1 | AX142455.1 | CS623533.1 | GM003578.1 | HV339676.1 | GN032497.1 | GM625026.1 | DL108733.1 |
| A03658.1 | DL048471.1 | AX142135.1 | CS626510.1 | FB983214.1 | HV341800.1 | GN032465.1 | GM624994.1 | DL108701.1 |
| A10276.1 | DL044854.1 | AX142005.1 | CS625894.1 | FB983162.1 | HV322262.1 | GN032433.1 | GM624962.1 | DL104055.1 |
| A08280.1 | DL044822.1 | AX141747.1 | DD438037.1 | FB754121.1 | FW556967.1 | GN032401.1 | GM623936.1 | DL099521.1 |
| A06452.1 | DL044790.1 | AX141555.1 | DD437999.1 | FB753540.1 | FW561202.1 | GN032369.1 | GM623904.1 | DL099489.1 |
| A05151.1 | DL044758.1 | AX141491.1 | DD449090.1 | GM865857.1 | FW566581.1 | GN032336.1 | GM623872.1 | DL099457.1 |
| A04931.1 | DL044726.1 | AX141363.1 | CS619877.1 | GM618784.1 | FW508115.1 | GN032304.1 | GM623840.1 | DL099425.1 |
| A07989.1 | DL044694.1 | AX139411.1 | CS617806.1 | FB725959.1 | FW562460.1 | GN032272.1 | GM623776.1 | DL089512.1 |
| A07009.1 | DL040678.1 | AX127332.1 | CS614469.1 | GM731852.1 | FW560917.1 | GN032208.1 | GM623744.1 | DL089480.1 |
| A02269.1 | DL040646.1 | AX113899.1 | CS613793.1 | GM841620.1 | FW555399.1 | GN032176.1 | GM658923.1 | DL089448.1 |
| A05521.1 | DL040614.1 | AX112906.1 | CS612924.1 | GM840759.1 | FW553180.1 | GN032112.1 | GM658859.1 | DL089416.1 |
| A04418.1 | DL040582.1 | HW101853.1 | CS612787.1 | FB711261.1 | FW553093.1 | GN032080.1 | GM658827.1 | DL089384.1 |
| A02007.1 | DL040550.1 | HW101671.1 | CS611845.1 | GM967126.1 | FW556664.1 | GN032048.1 | GM658795.1 | DL113646.1 |
| A00970.1 | DL040518.1 | HW096971.1 | CS604545.1 | GM840421.1 | FW506875.1 | HV592876.1 | GM649088.1 | DL113614.1 |
| M20913.1 | DL040486.1 | HW096673.1 | CS604513.1 | GM041780.1 | FW510486.1 | HV579010.1 | GM649024.1 | DL113582.1 |
| M10410.1 | DL044606.1 | HW072891.1 | CS604481.1 | GM040858.1 | HI936424.1 | HV578970.1 | GM648992.1 | DL113550.1 |
| M10837.1 | DL044574.1 | HW105060.1 | CS604449.1 | GM040346.1 | HI930641.1 | HV588537.1 | GM642189.1 | DL113518.1 |
| M19153.1 | DL044510.1 | HW096199.1 | CS604417.1 | FB709318.1 | HI929439.1 | HV578523.1 | GM642157.1 | DL113454.1 |
| K00130.1 | DL044478.1 | HW071107.1 | CS604385.1 | CS727331.1 | HI935101.1 | HV582995.1 | GM642125.1 | DL028136.1 |
| M29897.1 | DL040462.1 | HW104017.1 | CS604353.1 | CS727229.1 | HI918264.1 | HV582254.1 | GM642093.1 | DL028104.1 |
| M30840.1 | DL040430.1 | HW103942.1 | CS604321.1 | GM894220.1 | HI661313.1 | HV582152.1 | GM642061.1 | DL028072.1 |
| OM981242.1 | DL040398.1 | HW099576.1 | CS604225.1 | GM887767.1 | HI923631.1 | HV581985.1 | GM642029.1 | DL028040.1 |
| MK674484.1 | DL040366.1 | HW099518.1 | CS603937.1 | DL202015.1 | HI180950.1 | HV601361.1 | GM634842.1 | DL028008.1 |
| AF069379.1 | DL040334.1 | HW068957.1 | CS603841.1 | DL194195.1 | HI552132.1 | HV601189.1 | GM634810.1 | DL036390.1 |
| GN041644.1 | DL040302.1 | HW083279.1 | CS603809.1 | DL196344.1 | HI470599.1 | HV601084.1 | GM634778.1 | DL036358.1 |
| AH002294.2 | DL036678.1 | HW083247.1 | CS603777.1 | DL193917.1 | HI560313.1 | HV574883.1 | GM629748.1 | DL036326.1 |
| M64434.1 | DL036646.1 | HW083215.1 | CS603457.1 | DL196199.1 | HI564704.1 | HV570658.1 | GM629716.1 | DL036294.1 |
| M35116.1 | DL036614.1 | HW081526.1 | CS603425.1 | DL196091.1 | HI568919.1 | HV570597.1 | GM887869.1 | DL044209.1 |
| AY609081.1 | DL036582.1 | HW099478.1 | CS603393.1 | DL193799.1 | HI568852.1 | HV569306.1 | GM718457.1 | DL044177.1 |
| AY331975.1 | DL036550.1 | HW103156.1 | CS603361.1 | DL183350.1 | HI564197.1 | HH974282.1 | GM669615.1 | DL044145.1 |
| AF003709.1 | DL036518.1 | HV704227.1 | CS603329.1 | DL183234.1 | HI583198.1 | HH999463.1 | GM656125.1 | DL044113.1 |
| KR632635.1 | DL032315.1 | HV703878.1 | CS603297.1 | FB513412.1 | HI563617.1 | HH999420.1 | GM656093.1 | DL044081.1 |
| LC006973.1 | DL032283.1 | HV744535.1 | CS603265.1 | FB512599.1 | HI563582.1 | HH999370.1 | GM656061.1 | DL048250.1 |
| HW408836.1 | DL032251.1 | HV703154.1 | CS603233.1 | FB571380.1 | HI558046.1 | HH997657.1 | GM656029.1 | DL048186.1 |
| HW408804.1 | DL032219.1 | HV702488.1 | CS603201.1 | CS696200.1 | HI575627.1 | HH997622.1 | GM655997.1 | DL048154.1 |
| HW408772.1 | DL032155.1 | HV701198.1 | CS603169.1 | CS696168.1 | HI551333.1 | HH997565.1 | GM655965.1 | HI000130.1 |

| | | | | | | | | |
|---|---|---|---|---|---|---|---|---|
| HW408740.1 | DL032123.1 | HV701166.1 | CS603137.1 | CS696136.1 | HI544141.1 | HH997477.1 | GM648972.1 | HC490375.1 |
| HW408708.1 | DL048425.1 | HV708129.1 | CS603105.1 | CS696072.1 | HI508238.1 | HH999315.1 | GM648940.1 | HC472300.1 |
| HW408676.1 | DL048393.1 | HV701083.1 | CS356867.1 | CS696040.1 | HI000041.1 | HH999261.1 | GM648908.1 | HC472267.1 |
| HW408649.1 | DL048361.1 | HV701051.1 | CS355971.1 | CS695975.1 | HI003642.1 | HH999208.1 | GM648876.1 | HC472235.1 |
| HW408617.1 | DL009231.1 | HV700918.1 | CS350544.1 | CS695943.1 | HI003591.1 | HH999160.1 | GM648844.1 | HC472171.1 |
| HW408585.1 | DL018785.1 | HV695513.1 | CS349694.1 | CS695911.1 | HI003505.1 | HH998126.1 | GM648812.1 | HC471971.1 |
| HW408553.1 | DL018753.1 | HV694759.1 | CS353185.1 | CS695879.1 | HI001834.1 | HH999083.1 | GM641913.1 | HC471939.1 |
| HW408521.1 | DL018721.1 | HV694727.1 | CS352983.1 | CS695847.1 | HI001773.1 | HH999043.1 | GM641881.1 | HC471907.1 |
| HW408489.1 | DL018689.1 | HV694658.1 | CS284487.1 | CS695815.1 | HI469589.1 | HH998986.1 | GM641849.1 | HC471875.1 |
| HW408426.1 | DL018657.1 | HV693316.1 | CS283935.1 | CS695751.1 | HI465693.1 | HH998932.1 | GM634661.1 | HC490535.1 |
| HW408394.1 | DL018625.1 | HV698249.1 | CS275988.1 | CS695623.1 | HI567694.1 | HH997210.1 | GM634629.1 | HC678837.1 |
| HW408362.1 | DL009205.1 | HV689456.1 | CS272553.1 | DL176514.1 | HI001670.1 | HH997148.1 | GM634597.1 | HC678772.1 |
| HW399706.1 | DL009173.1 | HV695878.1 | CS252554.1 | DL176453.1 | HI001616.1 | HH970054.1 | GM634565.1 | HC509925.1 |
| HW399341.1 | DL009141.1 | HV688889.1 | CS253980.1 | DL176369.1 | HI001574.1 | HH998885.1 | GM634533.1 | HC504919.1 |
| HW399262.1 | DL009077.1 | HV585141.1 | CS249845.1 | DL176290.1 | HI003400.1 | HH998825.1 | GM634501.1 | HC504590.1 |
| HW261193.1 | DL009045.1 | HV585109.1 | CS249188.1 | DL181370.1 | HI003358.1 | HH998763.1 | GM634469.1 | HC494418.1 |
| HW261161.1 | DL014229.1 | HV579285.1 | CS245392.1 | DL174588.1 | AX113750.1 | HH997101.1 | GM629664.1 | HC504268.1 |
| HW261097.1 | DL014197.1 | FW590747.1 | CS244164.1 | DL164125.1 | AX111697.1 | HH997068.1 | GM629632.1 | HC504189.1 |
| HW261065.1 | DL014165.1 | FW576613.1 | CS244212.1 | DL176779.1 | AX108257.1 | HH997009.1 | GM629600.1 | HC499863.1 |
| HW261001.1 | DL014133.1 | FW576089.1 | CS244180.1 | DL176699.1 | AX103763.1 | HH996955.1 | GM629568.1 | FW301497.1 |
| HW260969.1 | DL014101.1 | FW573338.1 | CS243272.1 | FB355548.1 | AX103640.1 | HH998673.1 | GM629536.1 | FW300575.1 |
| HW260937.1 | GM636490.1 | FW574595.1 | CS243249.1 | FB344544.1 | AX100349.1 | HH998603.1 | GM664728.1 | FW298767.1 |
| HW260905.1 | GM636458.1 | FW570998.1 | CS231713.1 | FB359896.1 | AX097516.1 | HH998551.1 | GM655933.1 | HC488033.1 |
| HW260873.1 | GM632050.1 | EU363767.1 | CS200906.1 | DL088092.1 | AX093096.1 | HH996927.1 | GM648770.1 | HC468966.1 |
| HW260809.1 | GM632018.1 | HI930659.1 | CS227253.1 | DL088060.1 | AX088745.1 | HH996829.1 | GM655765.1 | HC486442.1 |
| HW260777.1 | GM631986.1 | HI930279.1 | CS208294.1 | DL088028.1 | AX088689.1 | HH996772.1 | GM648748.1 | HC475381.1 |
| HW260745.1 | GM631922.1 | AY208922.1 | CS207839.1 | DL087996.1 | AX088070.1 | HH994565.1 | GM648684.1 | HC466636.1 |
| HW260713.1 | GM631890.1 | HI656874.1 | CS186192.1 | DL091937.1 | AX085436.1 | HH980501.1 | GM648652.1 | HC466604.1 |
| HW260681.1 | GM623119.1 | FW495949.1 | CS182197.1 | DL091905.1 | AX082943.1 | HH980433.1 | GM648620.1 | HC474320.1 |
| HW260649.1 | GM623087.1 | FW497349.1 | CS183934.1 | DL091873.1 | AX079162.1 | HH957936.1 | GM641817.1 | HC491791.1 |
| HW260617.1 | GM623055.1 | FW499096.1 | CS174656.1 | DL091841.1 | AX058549.1 | HH980360.1 | GM641785.1 | HC491759.1 |
| HW260521.1 | GM623023.1 | FW420456.1 | DD059293.1 | DL091809.1 | AX057295.1 | HH980301.1 | GM641753.1 | HC491727.1 |
| HW260489.1 | GM622991.1 | FW420367.1 | DD058551.1 | DL091777.1 | AX056091.1 | HH980225.1 | GM641721.1 | GN030103.1 |
| HW260457.1 | GM622959.1 | FW420335.1 | DD041985.1 | DL102898.1 | AX055012.1 | HH980161.1 | GM641689.1 | GN029943.1 |
| HW260425.1 | GM650445.1 | FW420055.1 | DD048082.1 | DL146286.1 | AX047746.1 | HH980128.1 | GM641656.1 | GN029845.1 |
| HW260393.1 | GM650413.1 | FW496630.1 | DD042880.1 | DL124442.1 | AX047117.1 | HH979883.1 | GM641624.1 | GN029813.1 |
| HW260361.1 | GM643605.1 | HI653913.1 | DD037309.1 | DL124410.1 | AX046147.1 | HH996712.1 | GM634436.1 | GN013374.1 |
| HW260297.1 | GM643573.1 | HI002126.1 | DD032606.1 | DL124378.1 | AX040779.1 | HH996646.1 | GM634404.1 | GN010217.1 |
| HW260265.1 | GM643541.1 | HI000498.1 | DD032472.1 | DL120289.1 | AX040187.1 | HH996606.1 | BD283714.1 | GN033547.1 |
| HW260233.1 | GM643509.1 | HI000425.1 | DD023890.1 | DL120257.1 | AX039525.1 | HH996550.1 | BD289754.1 | GN033515.1 |
| HW260201.1 | GM643477.1 | HI000389.1 | DD023358.1 | DL120225.1 | AX038894.1 | HH998456.1 | BD287343.1 | GN033483.1 |
| HW260169.1 | GM643445.1 | HI000322.1 | DD023184.1 | DL115822.1 | AX037325.1 | HH998407.1 | BD278973.1 | GN033451.1 |
| HW260137.1 | GM643412.1 | HI203349.1 | DD028787.1 | DL115790.1 | AX036757.1 | HH998368.1 | BD277775.1 | GN033419.1 |
| HW260105.1 | GM636423.1 | HI179236.1 | GN346582.1 | DL115758.1 | AX036025.1 | HH999954.1 | BD274894.1 | GN033387.1 |
| HW260073.1 | GM636391.1 | HI553275.1 | GN346550.1 | DL115726.1 | AX035973.1 | HH979822.1 | BD273676.1 | GN033323.1 |
| HW260041.1 | GM636359.1 | HI207636.1 | GN340239.1 | DL115694.1 | AX035429.1 | HH979672.1 | BD273295.1 | GN033291.1 |
| HW260009.1 | GM631855.1 | HI003857.1 | GN340165.1 | DL115662.1 | AX034333.1 | HH976498.1 | BD273174.1 | GN033227.1 |

| | | | | | | | | |
|---|---|---|---|---|---|---|---|---|
| HW259977.1 | GM631823.1 | HI002046.1 | GN339919.1 | DL115630.1 | AX028716.1 | HH999869.1 | BD272243.1 | GN033195.1 |
| HW259945.1 | GM631791.1 | HI001957.1 | GN131009.1 | DL111236.1 | AX027718.1 | HH999780.1 | BD271851.1 | GN033163.1 |
| HW259913.1 | GM631759.1 | HI000291.1 | GN334430.1 | DL111172.1 | AX024592.1 | HH998283.1 | BD271046.1 | GN033099.1 |
| HW259790.1 | GM631727.1 | HI000248.1 | GN131889.1 | DL111140.1 | AX023762.1 | HH998245.1 | DD230450.1 | GN033067.1 |
| HW259508.1 | GM631695.1 | HI000204.1 | GN116497.1 | DL111108.1 | AX023649.1 | HH998198.1 | DD229946.1 | GN033035.1 |
| HW259285.1 | GM622924.1 | HI000148.1 | GN116464.1 | DL111076.1 | AX023617.1 | HH998156.1 | DD228594.1 | GN033003.1 |
| HW259068.1 | GM622892.1 | HI000116.1 | GN116432.1 | DL106272.1 | AX023583.1 | HH993611.1 | DD228428.1 | GN032971.1 |
| HW258915.1 | GM622860.1 | HI204482.1 | GN116400.1 | DL106240.1 | HW154442.1 | HH979637.1 | DD227388.1 | GN032939.1 |
| HW258848.1 | GM622828.1 | HI203061.1 | GN116368.1 | CQ944005.1 | HW147476.1 | HH932015.1 | DD226694.1 | GN032907.1 |
| HW257396.1 | GM622796.1 | HI552628.1 | GN116336.1 | CQ943973.1 | HW158146.1 | FW394413.1 | DD225602.1 | GN032875.1 |
| HW257364.1 | GM622764.1 | HI520486.1 | GN116304.1 | CQ943941.1 | HW154113.1 | FW394304.1 | DD224664.1 | GN032843.1 |
| HW257332.1 | FB508029.1 | HI551901.1 | GN094511.1 | CQ943909.1 | HW147114.1 | FW394272.1 | DD224183.1 | GN032811.1 |
| HW257268.1 | GM657913.1 | HI470585.1 | GN094479.1 | CQ943877.1 | HW153792.1 | FW394253.1 | DD223906.1 | GN032779.1 |
| GM660606.1 | GM657849.1 | HI538377.1 | DM073968.1 | CQ918564.1 | HW153756.1 | FW394211.1 | CQ809727.1 | GN032748.1 |
| GM660574.1 | GM657817.1 | HI559234.1 | DM063702.1 | A02839.1 | HW160339.1 | FW394084.1 | CQ803001.1 | GN032716.1 |
| GM660542.1 | GM657784.1 | HI564726.1 | DM069073.1 | A18434.1 | HW151827.1 | DM370762.1 | CQ802134.1 | GN032684.1 |
| GM653355.1 | GM657752.1 | HI564233.1 | GN090937.1 | CQ898651.1 | HW151513.1 | DM370664.1 | CQ802030.1 | GN032652.1 |
| GM653323.1 | GM643394.1 | HI564110.1 | GN087195.1 | CQ898619.1 | HW144743.1 | DM370632.1 | CQ798425.1 | GN032619.1 |
| GM653291.1 | GM643361.1 | HI563635.1 | GM994418.1 | CQ898587.1 | HW144652.1 | DM370600.1 | CQ796751.1 | GN032587.1 |
| GM653259.1 | GM643329.1 | HI563603.1 | GM994308.1 | CQ898555.1 | HW126616.1 | DM370568.1 | CQ795457.1 | GN032556.1 |
| GM653227.1 | GM643297.1 | HI563568.1 | GM992101.1 | CQ895579.1 | HW125422.1 | DM370536.1 | CQ792351.1 | GN032524.1 |
| GM646164.1 | GM643265.1 | HI557537.1 | GM975888.1 | CQ893708.1 | HW144451.1 | DM370504.1 | CQ788166.1 | GN032492.1 |
| GM646132.1 | GM643233.1 | HI000025.1 | GN000590.1 | AX454147.1 | HW144373.1 | DM370472.1 | CQ787488.1 | GN032460.1 |
| GM646100.1 | GM636244.1 | HI003664.1 | GM969393.1 | AX453996.1 | HW144341.1 | DM371967.1 | CQ787456.1 | GN032428.1 |
| GM646068.1 | GM636212.1 | HI003489.1 | GM980878.1 | AX441439.1 | HW144309.1 | DM209381.1 | CQ787418.1 | GN032396.1 |
| GM639233.1 | GM636180.1 | HI001795.1 | GN007492.1 | AX443299.1 | HW124801.1 | HC003105.1 | CQ787378.1 | GN032331.1 |
| GM639201.1 | GM636148.1 | HI001756.1 | GM984674.1 | AX429442.1 | HW124495.1 | HB865366.1 | CQ787344.1 | GN032299.1 |
| GM639169.1 | GM636116.1 | HI469531.1 | FB986360.1 | AX428584.1 | HW124063.1 | HB865022.1 | CQ787308.1 | GN032267.1 |
| GM639137.1 | GM636084.1 | HI544121.1 | GM596741.1 | AX428221.1 | HW123523.1 | HB865014.1 | CQ787276.1 | GN032235.1 |
| GM639105.1 | GM631644.1 | HI575294.1 | FB736139.1 | AX427234.1 | HW121673.1 | HB864982.1 | CQ786940.1 | GN032171.1 |
| GM639073.1 | GM631612.1 | HI001638.1 | GM697227.1 | AX418387.1 | HW120963.1 | CS114660.1 | CQ784667.1 | GN032139.1 |
| GM639041.1 | GM631580.1 | HI001598.1 | DL465745.1 | AX404871.1 | HW120094.1 | CS112341.1 | CQ779569.1 | GN032107.1 |
| GM626494.1 | GM631548.1 | HI001554.1 | DL464551.1 | AX398327.1 | HW118242.1 | CS111502.1 | CQ772720.1 | GN032075.1 |
| GM626430.1 | GM631516.1 | HC452128.1 | DL476971.1 | AX395285.1 | HV766071.1 | CS105994.1 | CQ771660.1 | GN032043.1 |
| GM626398.1 | GM622713.1 | FU756121.1 | DL463071.1 | AX384692.1 | HV766039.1 | CS105936.1 | CQ771628.1 | GN032011.1 |
| GM626366.1 | GM622681.1 | DD027033.1 | DL476422.1 | AX382509.1 | HV765955.1 | CS103430.1 | CQ771596.1 | GN031979.1 |
| GM626334.1 | GM622649.1 | DD023575.1 | DL462977.1 | AX376960.1 | HV750239.1 | CS103329.1 | CQ770991.1 | GN031947.1 |
| GM752757.1 | GM622617.1 | DD020456.1 | DL469776.1 | AX376722.1 | HV755393.1 | CS103071.1 | CQ767921.1 | GN031915.1 |
| GM660519.1 | GM622585.1 | DD019752.1 | DL475569.1 | AX370677.1 | HV755058.1 | CS102948.1 | CQ767059.1 | GN031883.1 |
| GM660487.1 | GM622553.1 | DD017636.1 | DL460977.1 | AX364535.1 | HV744741.1 | CS102756.1 | CQ766068.1 | GN031851.1 |
| GM660455.1 | GM740802.1 | BD412586.1 | GM832155.1 | AX364503.1 | HV753555.1 | CS102692.1 | CQ761201.1 | GN031819.1 |
| GM660391.1 | GM704185.1 | BD434179.1 | GM712166.1 | AX364471.1 | HV753440.1 | CS102628.1 | CQ755001.1 | GN031786.1 |
| GM660359.1 | GM657731.1 | BD412359.1 | GM831162.1 | AX364439.1 | HV502719.1 | CS102596.1 | CQ754042.1 | GN031721.1 |
| GM653172.1 | GM657667.1 | BD453850.1 | GM711232.1 | AX364407.1 | HV502687.1 | CS102564.1 | CQ753252.1 | GN031689.1 |
| GM653140.1 | GM657635.1 | BD453818.1 | GM643500.1 | AX364375.1 | HV502623.1 | CS102532.1 | CQ654378.1 | GN031657.1 |
| GM653108.1 | GM657603.1 | BD453786.1 | GM643468.1 | AX364343.1 | HV502591.1 | CS102500.1 | CQ654288.1 | GN031625.1 |
| GM653076.1 | GM657571.1 | BD433650.1 | GM643435.1 | AX364216.1 | HV502559.1 | CS101211.1 | CQ654195.1 | GN031561.1 |

| | | | | | | | | |
|---|---|---|---|---|---|---|---|---|
| GM653044.1 | GM650379.1 | BD453762.1 | GM636446.1 | AX364183.1 | HV502527.1 | AX011382.1 | CQ654102.1 | GN031529.1 |
| GM653012.1 | GM650347.1 | BD453730.1 | GM636318.1 | AX363237.1 | HV502463.1 | AX011350.1 | CQ654012.1 | GN031497.1 |
| GM652980.1 | GM650315.1 | BD453698.1 | GM631846.1 | BD006786.1 | HV492749.1 | AX011220.1 | AX962038.1 | GN031465.1 |
| GM645981.1 | GM650283.1 | BD453666.1 | GM631814.1 | E50938.1 | HV505303.1 | AX010953.1 | AX960755.1 | GN031432.1 |
| GM645949.1 | GM650251.1 | BD453634.1 | GM631782.1 | BD002002.1 | HV494720.1 | AX006968.1 | AX960372.1 | GN031400.1 |
| GM645917.1 | GM650219.1 | BD453602.1 | GM622851.1 | E54569.1 | HV452151.1 | AX004609.1 | AX960340.1 | FU773364.1 |
| GM645885.1 | GM622521.1 | BD493379.1 | GM657904.1 | E55381.1 | HV453653.1 | AX003045.1 | AX958643.1 | FU759944.1 |
| GM645853.1 | GM622489.1 | BD453552.1 | GM657872.1 | BD000189.1 | HV452976.1 | AX002960.1 | AX052959.1 | FU757634.1 |
| GM645821.1 | GM622457.1 | BD453520.1 | GM657840.1 | AX356508.1 | HV452876.1 | A64188.1 | AX052920.1 | FU757602.1 |
| GM639018.1 | GM622425.1 | BD453488.1 | GM657775.1 | AX352744.1 | HV448936.1 | A48490.1 | AX050243.1 | HC438782.1 |
| GM638986.1 | GM622393.1 | BD453456.1 | GM657743.1 | AX352510.1 | HV450923.1 | A40269.1 | AX046153.1 | HC438416.1 |
| GM638954.1 | GM622361.1 | BD453424.1 | GM643256.1 | AX351100.1 | HV444623.1 | A35710.1 | AX044421.1 | HC441569.1 |
| GM638922.1 | GM631477.1 | BD453392.1 | GM636235.1 | AX349343.1 | HV444462.1 | A31668.1 | AX040794.1 | HC358220.1 |
| GM638890.1 | GM639340.1 | BD453247.1 | GM631635.1 | AX349120.1 | HV348319.1 | A29441.1 | AX040425.1 | HC358090.1 |
| GM638858.1 | GM639308.1 | BD453215.1 | GM631603.1 | AX348961.1 | HV437896.1 | A34231.1 | AX039583.1 | HC358057.1 |
| GM626307.1 | GM639276.1 | BD408090.1 | GM631571.1 | AX348474.1 | HV437581.1 | A32832.1 | AX039324.1 | HC357534.1 |
| GM626275.1 | GM639244.1 | BD450239.1 | GM704170.1 | AX347435.1 | HV437540.1 | A31843.1 | AX039148.1 | HC356044.1 |
| GM626243.1 | GM626697.1 | BD408019.1 | GM657658.1 | AX347357.1 | HV445404.1 | U57437.1 | AX037507.1 | HC324878.1 |
| GM626211.1 | GM626665.1 | BD429583.1 | GM622448.1 | HW381351.1 | HV349427.1 | A27464.1 | AX037015.1 | HC324498.1 |
| GM626179.1 | GM626633.1 | CQ972412.1 | GM622416.1 | HW381046.1 | HV348885.1 | A17655.1 | AX036030.1 | HC314771.1 |
| GM626147.1 | GM626601.1 | CQ972380.1 | GM622384.1 | HW353910.1 | HV347711.1 | A20496.1 | AX035978.1 | HC313691.1 |
| GM660314.1 | GM626569.1 | CQ972348.1 | GM622352.1 | HW353467.1 | HI933875.1 | A19490.1 | AX033249.1 | HC318718.1 |
| GM660282.1 | GM626537.1 | CQ971096.1 | GM657538.1 | HW341642.1 | HI931619.1 | A15939.1 | AX028762.1 | HC308493.1 |
| GM660250.1 | GM754527.1 | CQ970241.1 | GM657506.1 | HW350981.1 | HI936423.1 | A14319.1 | AX027723.1 | HC307849.1 |
| GM660218.1 | GM660702.1 | CQ967260.1 | GM657474.1 | HW350934.1 | HI930640.1 | A10688.1 | AX027303.1 | HC307794.1 |
| GM660186.1 | GM660670.1 | CQ956049.1 | GM657442.1 | HW350868.1 | HI935099.1 | A10383.1 | AX024783.1 | DM110293.1 |
| GM660154.1 | GM660638.1 | CQ947240.1 | GM657410.1 | HW350684.1 | HI934224.1 | A07329.1 | AX023654.1 | DM109927.1 |
| GM652948.1 | DD147669.1 | CQ947139.1 | GM657378.1 | HW350650.1 | HI918263.1 | A01658.1 | AX023622.1 | DM094876.1 |
| GM652916.1 | DD140154.1 | CQ947013.1 | GM650122.1 | HW350548.1 | HI661311.1 | A32073.1 | AX023330.1 | DM103433.1 |
| GM652884.1 | DD081446.1 | CQ944177.1 | GM650090.1 | HW340078.1 | HI923622.1 | A32040.1 | AX019179.1 | GN368660.1 |
| GM652852.1 | DD081167.1 | CQ944145.1 | GM643089.1 | HW349962.1 | HI657186.1 | A30536.1 | AX017436.1 | GN368412.1 |
| GM652820.1 | DD080950.1 | CQ944113.1 | GM627125.1 | HW339555.1 | HI656049.1 | A29948.1 | AX014751.1 | GN373300.1 |
| GM652787.1 | DD080799.1 | CQ944049.1 | GM661109.1 | HW349543.1 | FW420955.1 | A29181.1 | AX011480.1 | GN360155.1 |
| GM645789.1 | DD138770.1 | CQ944017.1 | GM661077.1 | HW347998.1 | FW496029.1 | A20263.1 | AX011448.1 | GN360123.1 |
| GM645757.1 | DD122269.1 | CQ943985.1 | GM661045.1 | HW104194.1 | FW420738.1 | A06570.1 | AX011416.1 | GN360091.1 |
| GM645725.1 | DD137695.1 | CQ943953.1 | GM661013.1 | HW099948.1 | FW504726.1 | A26110.1 | AX011383.1 | GN356160.1 |
| GM645693.1 | CS118962.1 | CQ943921.1 | GM660949.1 | HW104000.1 | FW420469.1 | A25741.1 | AX011351.1 | GN360050.1 |
| GM645661.1 | CS118929.1 | CQ943889.1 | GM653955.1 | HW070675.1 | FW420437.1 | A24905.1 | AX011222.1 | GN360018.1 |
| GM645629.1 | CS118896.1 | CQ943857.1 | GM653923.1 | HW099612.1 | FW420405.1 | A22363.1 | AX010955.1 | GN359890.1 |
| GM638798.1 | CS118863.1 | CQ902718.1 | GM653891.1 | HW099548.1 | FW504525.1 | A22093.1 | AX010625.1 | GN366316.1 |
| GM638734.1 | CS118830.1 | CQ898808.1 | GM653859.1 | HW069669.1 | FW420380.1 | A21421.1 | AX010379.1 | GN366284.1 |
| GM638702.1 | CS118765.1 | CQ898663.1 | GM646786.1 | HW069237.1 | FW420348.1 | A19440.1 | AX009451.1 | GN359845.1 |
| GM638670.1 | CS118700.1 | CQ898631.1 | GM646754.1 | HW089221.1 | FW420316.1 | A19399.1 | AX006969.1 | GN359685.1 |
| DL128734.1 | CS118668.1 | CQ898599.1 | GM646722.1 | HW089007.1 | HH977637.1 | A18338.1 | AX006465.1 | GN359405.1 |
| DL116398.1 | CS118636.1 | CQ898567.1 | GM646690.1 | HW083501.1 | HH980637.1 | A13362.1 | DL047997.1 | GN359373.1 |
| DL116366.1 | CS118604.1 | CQ888101.1 | GM646658.1 | HW088824.1 | HH980496.1 | A12350.1 | DL047965.1 | GN359341.1 |
| DL111645.1 | CS118570.1 | CQ877374.1 | GM646626.1 | HW083233.1 | HH980428.1 | A11180.1 | DL047933.1 | GN365268.1 |

| | | | | | | | | |
|---|---|---|---|---|---|---|---|---|
| DL111549.1 | CS118536.1 | CQ875501.1 | GM639823.1 | HW097607.1 | HH980355.1 | A10760.1 | DL047901.1 | GN359204.1 |
| DL111517.1 | CS118503.1 | CQ873321.1 | GM639791.1 | HW088101.1 | HH980295.1 | A09359.1 | DL047869.1 | GN359172.1 |
| DL111485.1 | CS118470.1 | CQ871400.1 | CS118132.1 | HW081695.1 | HH980220.1 | A16718.1 | DL040070.1 | GN359140.1 |
| DL101703.1 | CS118438.1 | CQ869381.1 | CS118100.1 | HW081584.1 | HH980156.1 | A09021.1 | DL040038.1 | GN359108.1 |
| DL101671.1 | CS118406.1 | CQ868888.1 | CS118067.1 | HW103535.1 | HH980123.1 | HW097030.1 | DL040006.1 | GN363822.1 |
| DL101639.1 | CS118373.1 | CQ867376.1 | CS118033.1 | HW102561.1 | HH979878.1 | HW072599.1 | DL039974.1 | GN363027.1 |
| DL101607.1 | CS118338.1 | CQ859628.1 | CS117997.1 | HW067938.1 | HH996704.1 | HW096225.1 | DL039942.1 | GN346507.1 |
| DL101575.1 | CS118305.1 | CQ859596.1 | CS114657.1 | HW059885.1 | HH996641.1 | HW072142.1 | DL039910.1 | GM625845.1 |
| DL101543.1 | CS118173.1 | AX112903.1 | CS105987.1 | HW061889.1 | HH996593.1 | HW071965.1 | DL047825.1 | GM625813.1 |
| DL097129.1 | CS118141.1 | AX108993.1 | CS102977.1 | HW061856.1 | HH996545.1 | HW104421.1 | DL047793.1 | GM625781.1 |
| DL097097.1 | CS118076.1 | AX107110.1 | CS102913.1 | HW062171.1 | HH998449.1 | HW104341.1 | DL047761.1 | GM625749.1 |
| DL097065.1 | CS118043.1 | AX105820.1 | CS102817.1 | HW061711.1 | HH998402.1 | HW104307.1 | DL047729.1 | GM624725.1 |
| DL097033.1 | CS118008.1 | AX100868.1 | CS102785.1 | HW065304.1 | HH998363.1 | HW071079.1 | DL047697.1 | GM624693.1 |
| DL097001.1 | CS117974.1 | AX097500.1 | CS102753.1 | HW065271.1 | HH999994.1 | HW104265.1 | DL047665.1 | GM624629.1 |
| DL096969.1 | CS115729.1 | AX093080.1 | CS102721.1 | HC867551.1 | HH999941.1 | HW104233.1 | DL043683.1 | GM624326.1 |
| DL095141.1 | CS114666.1 | AX088434.1 | CS102689.1 | HC882287.1 | HH979817.1 | HW103999.1 | DL039770.1 | GM659897.1 |
| DL095109.1 | CS114362.1 | AX085929.1 | CS102625.1 | HC880548.1 | HH979711.1 | HW070662.1 | DL039738.1 | GM659865.1 |
| DL095077.1 | CS113511.1 | AX085149.1 | CS102593.1 | HC879852.1 | HH979667.1 | HW099611.1 | DL039706.1 | GM659833.1 |
| DL095045.1 | CS113447.1 | AX083964.1 | CS102561.1 | DM460281.1 | HH999905.1 | HW069666.1 | DL035922.1 | GM659801.1 |
| AX417481.1 | CS113413.1 | AX080822.1 | CS102529.1 | DM459791.1 | HH999770.1 | HW069233.1 | DL031918.1 | GM659769.1 |
| AX411557.1 | CS113381.1 | AX077694.1 | CS102497.1 | DM459239.1 | HH999711.1 | HW067529.1 | DL031886.1 | GM659737.1 |
| AX398703.1 | CS113349.1 | AX076988.1 | CS101491.1 | DM385648.1 | HH998277.1 | HW089005.1 | DL031854.1 | GM692574.1 |
| AX397811.1 | CS113317.1 | AX074314.1 | CS101205.1 | DM383630.1 | HH998240.1 | HW083500.1 | DL031822.1 | GM659701.1 |
| AX395615.1 | CS112902.1 | AX060358.1 | CS095642.1 | DM382851.1 | HH998192.1 | HW088823.1 | DL031790.1 | GM659669.1 |
| AX392854.1 | CS110955.1 | AX058587.1 | CS089270.1 | DM381806.1 | HH998151.1 | HW083264.1 | DL031758.1 | GM659637.1 |
| AX392075.1 | CS109338.1 | AX057312.1 | CS085797.1 | HC054873.1 | HH993740.1 | HW083232.1 | DL027853.1 | GM659605.1 |
| AX391499.1 | CS103353.1 | AX040663.1 | CS083076.1 | HC053884.1 | HH991319.1 | HW083200.1 | DL027821.1 | GM652316.1 |
| AX384697.1 | CS106378.1 | AX039943.1 | AX773484.1 | HC053723.1 | HH979526.1 | HW082693.1 | DL027789.1 | GM652284.1 |
| AX384390.1 | CS105944.1 | AX036000.1 | AX772850.1 | HC051939.1 | HH979630.1 | HW088013.1 | DL027757.1 | GM645389.1 |
| AX380337.1 | CS103456.1 | AX028786.1 | AX771023.1 | HC049772.1 | HH979589.1 | HW081691.1 | DL023945.1 | GM645357.1 |
| AX376991.1 | CS103335.1 | AX027786.1 | AX769767.1 | HC045503.1 | HH932005.1 | HW081549.1 | DL023913.1 | GM645325.1 |
| AX375325.1 | CS103303.1 | AX026205.1 | AX766270.1 | HC045471.1 | FW394408.1 | HW103534.1 | DL023881.1 | GM645293.1 |
| AX370786.1 | CS103085.1 | AX025075.1 | AX765946.1 | HC045439.1 | FW394267.1 | HW103418.1 | DL023849.1 | GM645261.1 |
| AX366990.1 | CS102954.1 | AX023634.1 | AX755021.1 | HC045407.1 | FW398165.1 | HV304137.1 | DL023817.1 | GM645229.1 |
| AX364540.1 | CS102922.1 | AX023600.1 | AX752642.1 | HC045343.1 | FW398133.1 | HV311091.1 | DL023785.1 | GM652151.1 |
| AX364508.1 | CS102890.1 | AX023568.1 | AX752283.1 | HC045311.1 | FW394248.1 | HV312307.1 | DL020674.1 | GM645196.1 |
| AX364476.1 | CS102858.1 | AX021124.1 | AX751682.1 | HC045279.1 | FW394206.1 | DL024448.1 | DL020642.1 | GM645164.1 |
| AX364444.1 | CS102666.1 | HW315152.1 | BD185628.1 | HC042018.1 | FW397863.1 | DL024416.1 | DL020610.1 | GM645132.1 |
| AX364412.1 | CS102602.1 | HW315056.1 | BD181420.1 | HC047459.1 | FW397482.1 | DL024384.1 | DL016207.1 | GM645100.1 |
| AX364380.1 | CS102570.1 | HW314696.1 | BD180863.1 | HC047427.1 | FW393447.1 | DL021172.1 | DL016143.1 | GM645068.1 |
| AX364253.1 | CS102538.1 | HW160822.1 | AX743998.1 | HC047395.1 | FW392973.1 | DL021140.1 | DJ357782.1 | GM645036.1 |
| AX364221.1 | CS102506.1 | HC502382.1 | AX743501.1 | HC047363.1 | FW396672.1 | DL021108.1 | DJ357750.1 | GM638141.1 |
| AX364188.1 | CS101261.1 | HC502254.1 | AX711945.1 | HC047331.1 | HH936314.1 | DL021076.1 | DJ357718.1 | GM638109.1 |
| HW386087.1 | CS091389.1 | HC500865.1 | AX739859.1 | HC047299.1 | HH833908.1 | DL021012.1 | DJ357684.1 | GM638077.1 |
| HW386055.1 | CS088091.1 | HC499846.1 | AX722058.1 | HC047267.1 | HH833670.1 | DL012188.1 | DJ357652.1 | GM629475.1 |
| HW386023.1 | CS087528.1 | FW301733.1 | AX721693.1 | HC047043.1 | HH833638.1 | DL012156.1 | DJ357620.1 | GM629433.1 |
| HW385927.1 | BD057281.1 | FW300640.1 | BD178178.1 | HC047011.1 | HH833606.1 | DL029779.1 | DJ357588.1 | GM629401.1 |

| | | | | | | | | |
|---|---|---|---|---|---|---|---|---|
| HW380978.1 | BD017761.1 | HB864842.1 | AX720249.1 | HC046979.1 | HH833574.1 | DL029747.1 | DJ357259.1 | GM629369.1 |
| HW380915.1 | BD016706.1 | HB864810.1 | AX717578.1 | HC046947.1 | HH833542.1 | DL026356.1 | DJ363054.1 | GM629337.1 |
| HW380824.1 | BD015229.1 | HB864778.1 | AX709450.1 | HC046915.1 | HB645742.1 | DL018380.1 | DJ349692.1 | GM629305.1 |
| HW368544.1 | BD014238.1 | HB864746.1 | AX699461.1 | HC046887.1 | HB645710.1 | DL018348.1 | DJ355093.1 | GM648568.1 |
| HW367136.1 | BD014205.1 | HB866530.1 | AX699429.1 | HC046855.1 | HB645676.1 | DL018316.1 | DJ354933.1 | GM648536.1 |
| HW366483.1 | BD014165.1 | HB999684.1 | AX685831.1 | HC046823.1 | HB855098.1 | DL018284.1 | DJ361210.1 | GM648504.1 |
| HW375255.1 | BD013749.1 | HB976832.1 | AX684665.1 | HC046791.1 | HB850751.1 | DL018252.1 | DJ354221.1 | GM641605.1 |
| HW375072.1 | AX482628.1 | DM192914.1 | AX683937.1 | HC046759.1 | HB855897.1 | DL018220.1 | DJ339857.1 | GM629164.1 |
| HW374649.1 | AX481972.1 | DM190893.1 | AX675271.1 | HC046727.1 | HB855416.1 | DL013786.1 | DJ339761.1 | GM629132.1 |
| HW364596.1 | E61342.1 | DM189905.1 | AX670751.1 | HC046678.1 | DM179535.1 | DL013754.1 | DJ339729.1 | GM629100.1 |
| HW373306.1 | E64484.1 | DM203065.1 | AX664343.1 | HC046614.1 | DM179147.1 | DL013722.1 | DJ339697.1 | GM634062.1 |
| HW381356.1 | AX468897.1 | HB839497.1 | AX662222.1 | HC046582.1 | DM170819.1 | DL013690.1 | DJ339665.1 | DJ381000.1 |
| HW381051.1 | AX468468.1 | HB847581.1 | AX659105.1 | HC046550.1 | DM185747.1 | DL000813.1 | DJ339600.1 | DJ380968.1 |
| HW344352.1 | AX468334.1 | HB839226.1 | AX658713.1 | HC046518.1 | DM178270.1 | DL000627.1 | DJ339568.1 | DJ380936.1 |
| HW344320.1 | AX466978.1 | HB838859.1 | AX657143.1 | HC046486.1 | DM188216.1 | DJ493971.1 | DJ336587.1 | DJ380904.1 |
| HW344288.1 | A15069.1 | HB805437.1 | AX657111.1 | HC046454.1 | DM188009.1 | DJ493666.1 | DJ334216.1 | DJ380872.1 |
| HW344256.1 | AX458584.1 | HB847061.1 | AX657067.1 | HC046390.1 | DM187713.1 | DJ491559.1 | DJ329162.1 | DJ380820.1 |
| HW353820.1 | AX458207.1 | HB846610.1 | BD174853.1 | HC046358.1 | DM169096.1 | DL008173.1 | DJ328818.1 | DJ389471.1 |
| HW353724.1 | AX456810.1 | HB846185.1 | BD174660.1 | HC046075.1 | HB491787.1 | DJ446858.1 | DJ328676.1 | CS476352.1 |
| HW353635.1 | AX454156.1 | HB837841.1 | AX648076.1 | HC046043.1 | HB489509.1 | DJ446825.1 | DJ327051.1 | CS468418.1 |
| HW353603.1 | AX454001.1 | HB845788.1 | AX058550.1 | HC046251.1 | HB488754.1 | DJ442606.1 | DJ327019.1 | CS467757.1 |
| HW353539.1 | AX453542.1 | HB845506.1 | AX057296.1 | HC046219.1 | DM165601.1 | DJ442461.1 | DJ326600.1 | CS467560.1 |
| HW344026.1 | AX452040.1 | HB837367.1 | AX056630.1 | GN032174.1 | DM164022.1 | DJ433189.1 | DJ128271.1 | CS466934.1 |
| HW353472.1 | AX449465.1 | HB845279.1 | AX055062.1 | GN032142.1 | DM163972.1 | DJ437354.1 | DJ122498.1 | CS466051.1 |
| HW352851.1 | A34770.1 | HB845059.1 | AX052916.1 | GN032110.1 | DM163303.1 | DJ437234.1 | DJ082572.1 | CS464618.1 |
| HW352275.1 | AX443304.1 | HB844705.1 | AX046148.1 | GN032046.1 | DM163262.1 | DJ437175.1 | DJ081980.1 | DD382830.1 |
| HW341291.1 | AX441328.1 | HB844346.1 | AX039526.1 | GN032014.1 | DM162320.1 | DJ437145.1 | DJ086673.1 | DD400154.1 |
| HW351624.1 | AX429495.1 | HB836742.1 | AX039320.1 | GN031982.1 | HB486506.1 | DJ436912.1 | DJ081552.1 | CS460115.1 |
| HW341243.1 | AX429244.1 | HB843984.1 | AX038895.1 | GN031950.1 | GM654005.1 | DJ436835.1 | DJ080252.1 | CS459457.1 |
| HW061674.1 | AX428235.1 | HB843531.1 | AX036758.1 | GN031918.1 | GM653973.1 | DJ436753.1 | DJ081427.1 | DD361285.1 |
| HW058574.1 | AX419094.1 | HB836045.1 | AX036607.1 | GN031886.1 | GM646980.1 | DJ436506.1 | DJ066780.1 | DD367613.1 |
| HW065244.1 | AX418394.1 | HB843236.1 | AX036026.1 | GN031854.1 | GM646948.1 | DJ431115.1 | DJ071358.1 | DD373517.1 |
| HW065101.1 | AX418197.1 | HB842933.1 | AX035974.1 | GN031822.1 | GM646916.1 | DJ445028.1 | DJ066384.1 | DD368340.1 |
| HW061274.1 | HW237020.1 | HB835287.1 | AX035431.1 | GN031789.1 | GM646884.1 | DJ444746.1 | DJ066352.1 | DD368151.1 |
| HW058468.1 | HW243448.1 | HB842808.1 | AX034334.1 | GN031692.1 | GM646852.1 | DJ444566.1 | DJ066285.1 | DD361319.1 |
| HW064845.1 | HW241740.1 | HB842215.1 | AX033067.1 | GN031660.1 | GM646820.1 | DJ434808.1 | DJ066251.1 | CS457446.1 |
| HW055404.1 | HW241544.1 | HB841881.1 | AX029031.1 | GN031628.1 | GM639985.1 | DJ438361.1 | DJ068020.1 | CS457125.1 |
| HW056505.1 | HW243151.1 | HB841649.1 | AX028717.1 | GN031596.1 | GM639921.1 | DJ438355.1 | DJ067012.1 | CS453985.1 |
| HW064668.1 | HW243059.1 | HB841191.1 | AX027719.1 | GN031564.1 | GM639889.1 | DJ438288.1 | DJ061643.1 | DD359158.1 |
| HW064609.1 | HW163872.1 | HB840491.1 | AX024593.1 | GN031532.1 | DL120020.1 | DJ438206.1 | DJ061595.1 | DD357839.1 |
| HW062567.1 | HW163795.1 | HB650068.1 | AX023765.1 | GN031500.1 | DL094242.1 | DJ427971.1 | DJ061547.1 | DD357240.1 |
| HW062412.1 | HW163492.1 | HB645727.1 | AX023618.1 | GN031468.1 | DL094210.1 | DJ402643.1 | DJ061499.1 | CS447935.1 |
| HW060546.1 | HW163435.1 | HB645651.1 | AX021810.1 | GN031435.1 | DL094178.1 | DJ402545.1 | DJ055583.1 | A21910.1 |
| HW062355.1 | HW160774.1 | GM631829.1 | AX020384.1 | GN031403.1 | DL090431.1 | DJ402299.1 | DJ055509.1 | A12551.1 |
| HW062291.1 | HW160633.1 | GM631797.1 | AX019175.1 | GN031371.1 | DL090399.1 | DJ401347.1 | DJ061214.1 | A01386.1 |
| HW042078.1 | HW160601.1 | GM636154.1 | AX018534.1 | GN031307.1 | DL086298.1 | DJ400836.1 | BD269145.1 | CS443944.1 |
| HW042046.1 | HW155691.1 | GM656316.1 | AX011476.1 | GN031274.1 | DL086266.1 | DJ400804.1 | BD268168.1 | CS443399.1 |

| | | | | | | | | |
|---|---|---|---|---|---|---|---|---|
| HW046533.1 | HW155654.1 | GM656284.1 | AX011444.1 | GN031242.1 | DL086234.1 | BD233445.1 | BD268123.1 | CS438963.1 |
| HW043456.1 | HW150076.1 | GM656252.1 | AX011412.1 | GN031210.1 | DL086202.1 | BD232022.1 | BD265617.1 | CS437395.1 |
| HW042002.1 | HW150044.1 | GM634544.1 | AX011379.1 | GN031178.1 | DL110285.1 | BD231935.1 | BD263455.1 | CS436308.1 |
| HW041970.1 | HW150012.1 | GM634512.1 | AX011347.1 | GN031145.1 | DL110168.1 | BD231167.1 | DL041959.1 | BD307640.1 |
| HW041938.1 | HW144827.1 | GM634480.1 | AX010941.1 | GN031113.1 | DL110104.1 | BD231134.1 | DL044534.1 | BD300355.1 |
| HW049392.1 | HW155536.1 | GM664828.1 | AX008237.1 | GN031081.1 | DL105171.1 | BD231056.1 | DL044470.1 | BD294201.1 |
| HW049361.1 | HW155205.1 | GM655912.1 | AX006952.1 | GN031049.1 | DL105139.1 | BD229105.1 | DL040454.1 | BD292742.1 |
| HW049329.1 | HW159074.1 | GM648727.1 | AX006405.1 | GN031017.1 | DL105107.1 | BD227218.1 | DL040422.1 | BD292425.1 |
| HW049297.1 | HV693209.1 | GM648695.1 | AX004604.1 | GN030985.1 | DL100661.1 | BD227170.1 | DL040390.1 | BD291311.1 |
| HW049265.1 | HV699508.1 | GM648663.1 | AX003042.1 | GN030922.1 | DL100597.1 | BD226911.1 | DL040358.1 | BD298671.1 |
| HW049194.1 | HV699088.1 | GM648631.1 | AX002909.1 | GN030858.1 | DL100565.1 | BD225785.1 | DL040326.1 | BD297642.1 |
| HW041804.1 | HV690929.1 | GM648599.1 | A26538.1 | GN030826.1 | DL100533.1 | BD225753.1 | DL036606.1 | BD296224.1 |
| HW041772.1 | HV698178.1 | GM641700.1 | A48484.1 | GN030794.1 | DL119987.1 | BD225721.1 | DL036574.1 | BD295197.1 |
| HW054944.1 | HV689947.1 | GM634447.1 | A38677.1 | GN030764.1 | DL119955.1 | BD225689.1 | DL036542.1 | BD283989.1 |
| HW041551.1 | HV689028.1 | GM629460.1 | A35741.1 | DM001830.1 | DL119923.1 | BD225657.1 | DL032243.1 | BD276261.1 |
| HW050463.1 | HV695809.1 | DL095424.1 | A35705.1 | DM001473.1 | DL119891.1 | BD225625.1 | DL032211.1 | BD274311.1 |
| HW054016.1 | HV688830.1 | DL095392.1 | A29520.1 | DM006956.1 | DL119859.1 | BD224835.1 | DL032179.1 | BD274190.1 |
| HW048304.1 | HV600542.1 | DL095360.1 | A33983.1 | DM004993.1 | DL119827.1 | BD224130.1 | DL048449.1 | BD274102.1 |
| HW053855.1 | HV585539.1 | DL106948.1 | A30329.1 | DM003500.1 | DL115232.1 | BD223257.1 | DL048417.1 | BD273668.1 |
| HV975313.1 | HV579918.1 | DL106916.1 | A28920.1 | DM002795.1 | DL115200.1 | BD222950.1 | DL048385.1 | BD273142.1 |
| HV985976.1 | HV585131.1 | DL101211.1 | A27458.1 | DL488493.1 | DL115136.1 | BD222500.1 | DL048353.1 | BD271227.1 |
| HV985912.1 | HV584913.1 | DL094553.1 | A15658.1 | GM996589.1 | DL115104.1 | DD282959.1 | DL048321.1 | BD271071.1 |
| HV985867.1 | HV579196.1 | DL127365.1 | A15392.1 | GM996493.1 | DL115072.1 | DD287666.1 | DL044435.1 | DD231212.1 |
| HV974800.1 | HV579085.1 | DL114813.1 | A14306.1 | GM994763.1 | DL110072.1 | DD279451.1 | DL044403.1 | DD229867.1 |
| HV982676.1 | HV579014.1 | DL114781.1 | HW267797.1 | GM994313.1 | DL110040.1 | CS355989.1 | DL044371.1 | DD227767.1 |
| DJ437151.1 | HV578980.1 | DL114749.1 | HW267744.1 | GM656239.1 | DL110008.1 | CS350578.1 | DL044339.1 | DD227413.1 |
| DJ437114.1 | HV592531.1 | DL114685.1 | HW267280.1 | GM656207.1 | DL109976.1 | CS349695.1 | DL044307.1 | DD224653.1 |
| DJ437082.1 | HV592428.1 | DL114653.1 | HW265989.1 | GM656175.1 | DL109762.1 | CS353186.1 | DL044275.1 | DD224164.1 |
| DJ436996.1 | HV592363.1 | DL127184.1 | HW263028.1 | GM649182.1 | DL109730.1 | CS352984.1 | DL040099.1 | DD223869.1 |
| DJ436856.1 | HV577376.1 | DL141445.1 | HW262964.1 | GM649150.1 | DL109698.1 | CS283936.1 | DL032042.1 | DD231565.1 |
| DJ436766.1 | HV582281.1 | DL119105.1 | HW262916.1 | GM649118.1 | DL104964.1 | CS275989.1 | DL032010.1 | DD231469.1 |
| DJ431643.1 | HV582160.1 | DL119073.1 | HW262873.1 | GM649086.1 | DL104932.1 | CS272487.1 | DL031978.1 | DD231437.1 |
| DJ440916.1 | HV601422.1 | DL119041.1 | HW261375.1 | GM649022.1 | DL100390.1 | CS272636.1 | DL028137.1 | DD231405.1 |
| DJ434396.1 | HV586271.1 | DL119009.1 | HW261343.1 | GM648990.1 | DL100358.1 | CS272554.1 | DL028105.1 | CS273078.1 |
| DJ443502.1 | HV601196.1 | DL020892.1 | HW261311.1 | GM642155.1 | DL100326.1 | CS273586.1 | DL028073.1 | A12563.1 |
| DJ438367.1 | HV601127.1 | DL040176.1 | HW261279.1 | GM642123.1 | DL046101.1 | CS256600.1 | DL028009.1 | A00931.1 |
| DJ438294.1 | HV574486.1 | DL028022.1 | HW261247.1 | GM642091.1 | DL041955.1 | CS253981.1 | DL027977.1 | DD217644.1 |
| DJ427990.1 | HV585700.1 | DL027990.1 | HW261215.1 | GM642059.1 | DL041923.1 | CS250535.1 | DL036391.1 | DD216975.1 |
| DJ402687.1 | HV580374.1 | DL036404.1 | HW261183.1 | GM642027.1 | DL041891.1 | CS248664.1 | DL036359.1 | DD214015.1 |
| DJ402651.1 | HV574112.1 | DJ399594.1 | HW261151.1 | GM634840.1 | DL069434.1 | DD174385.1 | DL036327.1 | DD216619.1 |
| DJ402293.1 | HV570662.1 | DJ418079.1 | HW261119.1 | GM634808.1 | DL049708.1 | DD173908.1 | DL036295.1 | DD213905.1 |
| DJ400842.1 | HV570602.1 | DJ417450.1 | HW261055.1 | GM634776.1 | DL049676.1 | DD167023.1 | DL044242.1 | DD213873.1 |
| DJ400810.1 | HV567902.1 | DJ380838.1 | HW261023.1 | GM629778.1 | DL045668.1 | DJ339791.1 | DL044210.1 | DD213841.1 |
| DJ400778.1 | HV566993.1 | DJ389576.1 | HW260991.1 | GM629746.1 | DL041757.1 | DJ339759.1 | DL044178.1 | DD213809.1 |
| DJ393151.1 | HV566071.1 | CS723758.1 | HW260959.1 | GM629714.1 | DL041725.1 | DJ339727.1 | DL044146.1 | DD213792.1 |
| DJ393095.1 | HV565809.1 | DJ347806.1 | HW260927.1 | GM736682.1 | DL041693.1 | DJ339695.1 | DL044114.1 | DD213760.1 |
| DJ415904.1 | HV573506.1 | DJ341028.1 | HW260895.1 | GM886940.1 | DL049635.1 | DJ339566.1 | DL044082.1 | DD213728.1 |

| | | | | | | | | |
|---|---|---|---|---|---|---|---|---|
| DJ398310.1 | HV572432.1 | GM643099.1 | HW260863.1 | GM655995.1 | DL049603.1 | DJ328848.1 | DL048251.1 | DD213696.1 |
| DJ418071.1 | HV572127.1 | GM643067.1 | HW260831.1 | GM655963.1 | DL041556.1 | DJ327049.1 | DL048219.1 | DD213664.1 |
| DJ418807.1 | HV571424.1 | GM643035.1 | HW260799.1 | GM648970.1 | DL041524.1 | DJ327017.1 | DL048187.1 | DD213632.1 |
| DJ418648.1 | FW572091.1 | GM636046.1 | HW260767.1 | GM648938.1 | DL037669.1 | DJ326597.1 | DL048155.1 | DD220790.1 |
| DJ417443.1 | FW571781.1 | GM636014.1 | HW260735.1 | GM648906.1 | DL037637.1 | DJ128227.1 | DL048123.1 | AX719174.1 |
| CS604803.1 | HI967437.1 | GM635982.1 | HW260703.1 | GM648874.1 | DL037605.1 | DJ122481.1 | DL048091.1 | AX712201.1 |
| CS604771.1 | HI968461.1 | GM635950.1 | HW260671.1 | GM648842.1 | DL037573.1 | DJ088777.1 | DL048059.1 | AX709089.1 |
| CS604739.1 | HI979301.1 | GM635918.1 | HW260639.1 | GM648810.1 | DL037541.1 | DJ088494.1 | DL048028.1 | AX704558.1 |
| CS604707.1 | HI988944.1 | GM635886.1 | HW260607.1 | GM641911.1 | DL033306.1 | DJ083879.1 | DL047964.1 | AX701545.1 |
| CS604675.1 | HI988402.1 | GM631283.1 | HW260543.1 | GM634659.1 | DL033274.1 | DJ082568.1 | DL047932.1 | AX699458.1 |
| CS604641.1 | HI987437.1 | GM631251.1 | HW260511.1 | GM634627.1 | DL033210.1 | DJ081976.1 | DL047900.1 | AX699426.1 |
| CS604609.1 | HC194690.1 | GM631219.1 | HW260479.1 | GM634595.1 | DL033178.1 | DJ086346.1 | DL047868.1 | AX685570.1 |
| CS604577.1 | FW508689.1 | GM631187.1 | HW260447.1 | GM634531.1 | DL029138.1 | DJ080761.1 | DL040069.1 | AX683916.1 |
| CS597524.1 | FW512091.1 | GM631123.1 | HW260383.1 | GM634499.1 | DL029106.1 | DJ080217.1 | DL040037.1 | AX675247.1 |
| CS589282.1 | FW508358.1 | GM631069.1 | HW260351.1 | GM629694.1 | DL029074.1 | DJ071487.1 | DL040005.1 | AX670716.1 |
| CS585117.1 | FW508316.1 | GM631037.1 | HW260319.1 | GM664883.1 | DL009446.1 | DJ066778.1 | DL039973.1 | AX664340.1 |
| CS584691.1 | FW508219.1 | GM631097.1 | HW260255.1 | GM664726.1 | DL009414.1 | DJ071356.1 | DL039941.1 | AX662218.1 |
| CS575470.1 | FW559264.1 | GM622322.1 | HW260223.1 | GM648772.1 | DL018948.1 | DJ066382.1 | DL039909.1 | GM629162.1 |
| CS574924.1 | FW552307.1 | GM622290.1 | HW260191.1 | GM648746.1 | DL034327.1 | DJ066350.1 | DL048644.1 | GM629130.1 |
| CS574798.1 | FW552273.1 | GM622258.1 | HW260159.1 | GM648714.1 | DL009379.1 | DJ066283.1 | DL020789.1 | GM884096.1 |
| CS573048.1 | FW552236.1 | GM622226.1 | HW260127.1 | GM648682.1 | DL009347.1 | DJ065935.1 | DL047824.1 | GM648356.1 |
| CS565620.1 | FW552177.1 | GM622194.1 | HW260095.1 | GM648650.1 | DL009315.1 | DJ068014.1 | DJ050215.1 | GM648324.1 |
| CS570750.1 | FW552140.1 | GM622162.1 | HW260063.1 | GM648618.1 | DL009283.1 | EU363226.1 | CS793958.1 | GM648292.1 |
| CS570674.1 | FW552101.1 | GM631021.1 | HW259999.1 | GM641783.1 | DL009251.1 | DJ061592.1 | CS800477.1 | GM648260.1 |
| CS545656.1 | FW558939.1 | GM630989.1 | HW259967.1 | GM641751.1 | DL009219.1 | DD345729.1 | CS800099.1 | GM648228.1 |
| CS561194.1 | FW555604.1 | GM630957.1 | HW259739.1 | GM641719.1 | DL018677.1 | CS433054.1 | CS720119.1 | GM648196.1 |
| CS561153.1 | FW554521.1 | GM630925.1 | HW258896.1 | GM634466.1 | DL018645.1 | CS414531.1 | CS719307.1 | GM641393.1 |
| CS560466.1 | FW553223.1 | GM622118.1 | HW258838.1 | GM634434.1 | DL018613.1 | CS412507.1 | CS716850.1 | GM641329.1 |
| DD421977.1 | FW560383.1 | GM622086.1 | HW257418.1 | GM634402.1 | DL014217.1 | CS416214.1 | CS716779.1 | GM641297.1 |
| DD420778.1 | FW563124.1 | GM622054.1 | HW257386.1 | GM634370.1 | DL014153.1 | CS415767.1 | CS806049.1 | GM641265.1 |
| CS543090.1 | FW557314.1 | GM622022.1 | HW257322.1 | GM634338.1 | DL014121.1 | CS415548.1 | CS714324.1 | GM629042.1 |
| CS542565.1 | FW561674.1 | GM646596.1 | HW257226.1 | GM634306.1 | DL014089.1 | CS414841.1 | CS805333.1 | GM629010.1 |
| CS510543.1 | FW557016.1 | GM646532.1 | HW257002.1 | GM634274.1 | DL014002.1 | DD320654.1 | CS810202.1 | GM628946.1 |
| CS502924.1 | FW556982.1 | GM646500.1 | HW256874.1 | GM629479.1 | DL013970.1 | DD327198.1 | CS791258.1 | GM648163.1 |
| CS502782.1 | FW560091.1 | GM646468.1 | HW256810.1 | GM629437.1 | HW337434.1 | DD327028.1 | CS790632.1 | FB343460.1 |
| CS502716.1 | FW565449.1 | GM646436.1 | HV748522.1 | GM629405.1 | HW337178.1 | DD326894.1 | CS792480.1 | DL088084.1 |
| CS502680.1 | FW562681.1 | GM639633.1 | HV748385.1 | GM629373.1 | HW321855.1 | DD329901.1 | DJ028949.1 | DL088052.1 |
| CS502648.1 | FW559901.1 | GM639601.1 | HV751086.1 | GM629309.1 | HW336638.1 | DD326838.1 | DJ044954.1 | DL091929.1 |
| CS502616.1 | HH999168.1 | GM639569.1 | HV753780.1 | DL091903.1 | HW336558.1 | DD328891.1 | DJ044932.1 | DL091865.1 |
| CS502410.1 | M32786.1 | GM639537.1 | HV753558.1 | DL091871.1 | HW321092.1 | DD321801.1 | DJ044891.1 | DL102890.1 |
| CS502422.1 | M17533.1 | GM639505.1 | HV753441.1 | DL091839.1 | HW329328.1 | DD321755.1 | DJ030496.1 | DL102858.1 |
| CS501257.1 | M11517.1 | GM639497.1 | HV743342.1 | DL091807.1 | HW056576.1 | CS389280.1 | DJ033843.1 | DL102826.1 |
| BD225759.1 | M20266.1 | GM639441.1 | HV743302.1 | DL091775.1 | HW056526.1 | CS389229.1 | DJ029999.1 | DL107574.1 |
| BD225727.1 | HV549728.1 | GM626894.1 | HV743265.1 | DL102896.1 | HW064656.1 | CS389190.1 | DJ029929.1 | DL102581.1 |
| BD225695.1 | HV549658.1 | GM626862.1 | HV704983.1 | DL102864.1 | HW060701.1 | CS398651.1 | DJ026764.1 | DL102549.1 |
| BD225663.1 | HV549626.1 | GM626830.1 | HV704940.1 | DL102832.1 | HW062401.1 | CS401605.1 | DJ026281.1 | DL102517.1 |
| BD225631.1 | HV549594.1 | GM626798.1 | HV704661.1 | DL102768.1 | HW060519.1 | CS409983.1 | CS721640.1 | DL098007.1 |

| | | | | | | | | |
|---|---|---|---|---|---|---|---|---|
| BD224847.1 | HV549418.1 | GM626734.1 | HV704445.1 | DL102736.1 | HW060450.1 | CS402981.1 | CS691409.1 | DL097975.1 |
| BD224252.1 | HV549283.1 | GM653741.1 | HV704253.1 | DL107580.1 | HW056307.1 | CS402174.1 | CS693901.1 | DL097943.1 |
| BD222523.1 | HI987580.1 | GM653709.1 | HV704184.1 | DL107488.1 | HW064443.1 | CS402095.1 | CS693245.1 | DL112633.1 |
| BD222015.1 | HI987434.1 | GM653677.1 | HV744582.1 | DL107456.1 | HW062376.1 | CS382130.1 | CS674198.1 | DL112601.1 |
| BD218295.1 | FW508355.1 | GM653645.1 | HV703447.1 | DL107392.1 | HW062344.1 | CS382615.1 | DJ008404.1 | DL112569.1 |
| BD217996.1 | FW508213.1 | GM653613.1 | HV702870.1 | DL102587.1 | HW062312.1 | DD308737.1 | DJ008372.1 | DL095729.1 |
| BD211428.1 | FW552304.1 | GM653581.1 | HV701838.1 | DL102555.1 | HW042067.1 | DD313246.1 | DJ011705.1 | DL095697.1 |
| BD195121.1 | FW552270.1 | GM660906.1 | HV701244.1 | DL102523.1 | HW042035.1 | DD291683.1 | DJ004235.1 | DL095665.1 |
| DD283675.1 | FW552233.1 | GM660874.1 | HV701212.1 | DL098013.1 | HW043565.1 | DD291017.1 | CS389231.1 | DL095633.1 |
| DD283601.1 | FW562043.1 | GM660842.1 | HV701180.1 | DL097981.1 | HW041991.1 | DD319823.1 | HC867638.1 | DL095601.1 |
| DD283504.1 | FW552172.1 | GM660778.1 | HV701148.1 | DL097949.1 | HW041959.1 | CS365239.1 | HC867549.1 | DL095569.1 |
| DD282969.1 | FW552098.1 | GM660746.1 | HV701116.1 | DL112607.1 | HW041927.1 | CS376129.1 | HC882286.1 | DL117406.1 |
| DD281824.1 | FW555601.1 | GM653559.1 | HV708221.1 | DL112575.1 | HW041829.1 | CS375999.1 | HC872295.1 | DL117374.1 |
| DD280860.1 | FW558728.1 | GM653527.1 | HV701065.1 | DL112543.1 | HW049413.1 | CS375796.1 | HC880547.1 | DL091957.1 |
| DD279457.1 | FW557013.1 | GM653495.1 | FB580120.1 | DL112511.1 | HW049381.1 | CS362717.1 | HC879851.1 | DL091601.1 |
| DD278742.1 | FW562768.1 | GM653463.1 | FB573720.1 | DL112479.1 | HW049350.1 | CS366523.1 | HC889203.1 | DL087958.1 |
| CS356011.1 | FW562705.1 | GM653431.1 | DL200050.1 | DL095735.1 | HW049318.1 | CS287624.1 | HC889038.1 | DL087926.1 |
| CS354044.1 | FW562517.1 | GM653399.1 | DL200018.1 | DL095703.1 | HW049286.1 | CS287555.1 | HC869701.1 | DL121757.1 |
| CS348990.1 | FW566747.1 | GM646400.1 | DL199986.1 | DL095671.1 | HW049254.1 | DD240753.1 | HC869541.1 | DL121725.1 |
| CS353192.1 | FW505129.1 | GM646368.1 | DL195048.1 | DL095607.1 | HW046267.1 | DD240704.1 | HC869481.1 | DL121693.1 |
| CS353041.1 | FW508120.1 | GM646336.1 | DL189426.1 | DL095575.1 | HW041793.1 | DD240656.1 | HC876939.1 | DL121661.1 |
| CS352746.1 | FW562465.1 | GM646304.1 | DL199785.1 | DL095543.1 | HW041761.1 | DD240488.1 | FW304502.1 | DL121629.1 |
| CS276794.1 | FW555408.1 | GM646272.1 | DL194842.1 | DL117380.1 | HW043080.1 | DD234702.1 | FW310248.1 | DL121597.1 |
| CS272564.1 | FW553185.1 | GM646240.1 | DL202097.1 | DL117348.1 | HW050607.1 | E48831.1 | HC007554.1 | DL093612.1 |
| CS265876.1 | FW553122.1 | GM639405.1 | DL201918.1 | DL117316.1 | HW041394.1 | E41522.1 | HC010687.1 | DL123161.1 |
| CS260200.1 | FW566287.1 | GM639373.1 | DL191617.1 | DL117284.1 | HW044238.1 | E49207.1 | DM370760.1 | DL123129.1 |
| CS257322.1 | FW566100.1 | GM639341.1 | DL196194.1 | HV503269.1 | HW054037.1 | E35600.1 | DM370662.1 | DL118683.1 |
| CS256706.1 | FW559432.1 | GM639309.1 | DL193762.1 | HV503205.1 | HW053992.1 | E33646.1 | DM370630.1 | DL118651.1 |
| CS255220.1 | FW559395.1 | GM639277.1 | DL184040.1 | HV503182.1 | HW053876.1 | E36142.1 | DM370598.1 | DL118619.1 |
| CS253592.1 | FW559347.1 | GM639245.1 | DL183397.1 | HV503150.1 | HV985933.1 | E35559.1 | DM370566.1 | DL114223.1 |
| CS252618.1 | FW506882.1 | GM626698.1 | FB513371.1 | HV503118.1 | HW028876.1 | E06874.1 | DM370534.1 | DL114191.1 |
| DD326995.1 | FW506231.1 | GM626666.1 | FB512594.1 | HV503086.1 | HV984387.1 | E05793.1 | DM370502.1 | DL114159.1 |
| DD326898.1 | HD066659.1 | GM626602.1 | FB570940.1 | HV503054.1 | HV984263.1 | E05301.1 | DM370470.1 | DL114127.1 |
| DD329905.1 | HD064472.1 | GM626570.1 | CS696195.1 | HV503022.1 | FZ413146.1 | E04906.1 | DM372763.1 | DL114095.1 |
| DD326842.1 | HD063561.1 | GM626538.1 | CS696163.1 | HV502990.1 | FZ418923.1 | E04467.1 | DM205461.1 | DL114063.1 |
| DD329405.1 | HD053021.1 | GM754530.1 | CS696099.1 | HV502958.1 | FZ411746.1 | E03977.1 | DM371965.1 | DL126203.1 |
| DD321805.1 | HD052884.1 | GM660703.1 | CS696067.1 | HV502926.1 | FZ420768.1 | E03512.1 | DM209379.1 | DL126171.1 |
| DD321761.1 | HD052118.1 | GM660671.1 | CS696035.1 | HV502894.1 | FZ419853.1 | E03211.1 | HC003097.1 | DL122955.1 |
| CS390607.1 | HD051871.1 | GM660639.1 | DL106096.1 | HV502830.1 | FZ419819.1 | E02778.1 | HC002976.1 | DL122923.1 |
| CS390496.1 | HD048845.1 | GM660607.1 | DL106064.1 | HV492858.1 | FZ419787.1 | E01771.1 | HB865020.1 | DL113953.1 |
| CS398093.1 | HC920323.1 | GM660575.1 | DL106032.1 | HV502786.1 | FW580407.1 | E01555.1 | HB865012.1 | DL113921.1 |
| CS389317.1 | HD033445.1 | GM660543.1 | DL105968.1 | HV502722.1 | FW580719.1 | E01365.1 | HB864956.1 | DL113889.1 |
| CS389284.1 | HD057692.1 | GM653356.1 | DL105936.1 | HV502690.1 | FW591892.1 | E01231.1 | HB864924.1 | DL113857.1 |
| CS389242.1 | HD057660.1 | GM653292.1 | DL105904.1 | HV502658.1 | FW590842.1 | E01032.1 | HB864892.1 | DL109056.1 |
| CS389194.1 | HD062508.1 | GM653260.1 | DL101195.1 | HV502626.1 | FW590727.1 | E00871.1 | HB864860.1 | DL109024.1 |
| CS389157.1 | HD061466.1 | GM653228.1 | DL101163.1 | HV502594.1 | FW590299.1 | E00130.1 | HB864828.1 | DL108992.1 |
| CS401288.1 | S64559.1 | GM653196.1 | DL096717.1 | HV502562.1 | FW589157.1 | DD100278.1 | HB864796.1 | DL104290.1 |

| | | | | | | | | |
|---|---|---|---|---|---|---|---|---|
| CS406677.1 | M62373.1 | GM646197.1 | DL096685.1 | HV502530.1 | FW593442.1 | DD084819.1 | HB864764.1 | DL104258.1 |
| CS406431.1 | M27327.1 | GM646165.1 | DL096653.1 | HV502498.1 | FW586437.1 | AY967223.1 | HB866946.1 | DL104226.1 |
| CS404659.1 | HC918578.1 | GM646133.1 | DL096621.1 | HV502466.1 | FW592630.1 | AY967191.1 | HB866506.1 | DL099588.1 |
| CS403045.1 | FW334379.1 | GM646101.1 | DL096589.1 | HV505315.1 | FW585099.1 | AY967159.1 | HB981728.1 | DL099556.1 |
| CS408093.1 | FW334603.1 | GM646069.1 | DL096557.1 | HV505210.1 | FW577820.1 | AY967127.1 | HB999867.1 | DL093450.1 |
| CS402195.1 | FW342473.1 | GM646037.1 | DL094729.1 | HV494725.1 | FW576915.1 | AY967095.1 | HB981260.1 | DL093418.1 |
| CS401837.1 | FW337539.1 | DD404997.1 | DL094697.1 | HV507619.1 | FW576641.1 | AY967063.1 | HB977637.1 | DL093386.1 |
| CS382622.1 | FW341705.1 | DD404965.1 | DL094665.1 | HV453659.1 | FW575880.1 | AY967031.1 | HB977440.1 | DL093354.1 |
| CS376595.1 | FW341580.1 | DD404933.1 | DL090954.1 | HV450040.1 | FW575356.1 | AY966999.1 | HB976753.1 | DL126099.1 |
| DD309773.1 | HC868218.1 | DD404901.1 | DL090922.1 | HV451952.1 | HI180947.1 | AY966967.1 | HB976601.1 | DL040063.1 |
| DD308999.1 | HC868075.1 | DD404869.1 | DL086986.1 | HV449892.1 | HI538849.1 | AY966935.1 | HB976177.1 | DL040031.1 |
| DD298470.1 | HC868015.1 | DD401446.1 | DL086954.1 | HV452997.1 | HI470720.1 | CS057780.1 | DM193382.1 | DL039999.1 |
| DD308743.1 | HC887350.1 | DD401414.1 | DL086922.1 | HV452883.1 | HI470597.1 | CS055381.1 | DM195298.1 | DL039967.1 |
| DD291735.1 | HC867553.1 | DD405197.1 | DL086890.1 | HV445486.1 | HI544516.1 | CS054754.1 | HB840279.1 | DL039935.1 |
| DD291021.1 | HC881014.1 | DD405165.1 | DL086858.1 | HV448962.1 | HI560309.1 | CS052434.1 | HB848140.1 | DL039903.1 |
| CS365271.1 | HC880550.1 | DD405133.1 | DL086826.1 | HV448479.1 | HI564701.1 | CS052384.1 | HB847889.1 | DL047658.1 |
| CS365145.1 | HC879860.1 | DD405101.1 | DL086794.1 | HV450927.1 | HI568915.1 | CS052350.1 | HB839321.1 | DL043676.1 |
| CS376004.1 | HC889122.1 | DD405069.1 | DL086762.1 | HV444465.1 | HI568850.1 | CS052316.1 | HB847370.1 | DL027846.1 |
| CS362749.1 | HC449532.1 | DD405037.1 | DL086730.1 | HV443996.1 | HI564257.1 | CS050981.1 | HB832564.1 | DL027814.1 |
| CS378189.1 | FU772386.1 | DD408896.1 | DL086698.1 | HI183835.1 | HI564155.1 | CS048776.1 | HB838544.1 | DL027782.1 |
| CS359746.1 | FU761019.1 | DD404687.1 | DL086666.1 | HI183556.1 | HI583194.1 | CS038683.1 | HB846433.1 | DL027750.1 |
| CS359642.1 | FU764049.1 | DD404521.1 | DL105875.1 | HI180165.1 | HI563647.1 | CS023836.1 | HB815310.1 | DL023874.1 |
| BD269147.1 | FU757608.1 | DD403981.1 | DL105843.1 | HI202837.1 | HI563615.1 | CS022572.1 | GN069322.1 | DL023842.1 |
| BD268935.1 | FU756850.1 | DD403169.1 | DL105811.1 | HI214565.1 | HI563580.1 | AX523915.1 | GN075485.1 | HW260378.1 |
| BD268257.1 | FU762423.1 | DD405840.1 | DL096528.1 | HI214533.1 | HI558032.1 | AX523633.1 | GN067891.1 | HW260346.1 |
| BD268125.1 | HC438959.1 | DD405808.1 | DL096496.1 | HI214435.1 | HI551331.1 | A33387.1 | GN067795.1 | HW260314.1 |
| DD038308.1 | HC438422.1 | DD402412.1 | DL096464.1 | HI185963.1 | HI551127.1 | AX179484.1 | GN067767.1 | HW260282.1 |
| DD037400.1 | HC360366.1 | DD402380.1 | DL110741.1 | HI213005.1 | HI544139.1 | AX179417.1 | GN067735.1 | HW260250.1 |
| DD037078.1 | HC358226.1 | DD402348.1 | DL110709.1 | HI212609.1 | HI508399.1 | AX026628.1 | GN065230.1 | HW260218.1 |
| DD032528.1 | HC358194.1 | DD402316.1 | DL090675.1 | HI546320.1 | HI508233.1 | A09536.1 | GN052459.1 | HW260186.1 |
| DD030686.1 | HC358063.1 | DD402284.1 | DL086574.1 | HI546220.1 | HI000039.1 | AX521538.1 | GN052427.1 | HW260154.1 |
| DD027330.1 | HC357627.1 | DD402252.1 | DL108153.1 | HI586984.1 | HI003676.1 | AX521506.1 | GN052384.1 | HW260122.1 |
| DD025948.1 | HC434752.1 | DD402220.1 | DL098781.1 | HI210998.1 | HI003631.1 | AF430192.1 | GN051388.1 | HW260090.1 |
| DD024028.1 | HC357035.1 | DD402188.1 | DL098749.1 | HI210966.1 | HI003589.1 | AF430160.1 | GN046272.1 | HW260058.1 |
| DD023382.1 | HC325464.1 | DD402156.1 | DL092707.1 | HI210934.1 | HI003502.1 | AX511421.1 | GN045825.1 | HW260026.1 |
| DD023238.1 | A07546.1 | DD402124.1 | DL088964.1 | HI565902.1 | HI001832.1 | AX505209.1 | DM019509.1 | HW259994.1 |
| DD029706.1 | HC324889.1 | DD402092.1 | DL088932.1 | HI473125.1 | HI001771.1 | AX505145.1 | DM012829.1 | HW259962.1 |
| DD028792.1 | HC324504.1 | DD402060.1 | DL103275.1 | HI473011.1 | HI544134.1 | AX498249.1 | DM017760.1 | HW259853.1 |
| DD027672.1 | HC315542.1 | DD405780.1 | DL107963.1 | HI472907.1 | HI465678.1 | BD140160.1 | DM038362.1 | HW259693.1 |
| DD010251.1 | HC315510.1 | DD405748.1 | DL107931.1 | HI577481.1 | HI544133.1 | BD138572.1 | DM021887.1 | HW259381.1 |
| DD017752.1 | HC310244.1 | DD405716.1 | DL107899.1 | HI004309.1 | HW390747.1 | BD138403.1 | DM015977.1 | HW258940.1 |
| DD009542.1 | HC307861.1 | DD405684.1 | DL092441.1 | HI002276.1 | HW405850.1 | BD136883.1 | GN045759.1 | HW258881.1 |
| BD495449.1 | HC307371.1 | DD405652.1 | DL092409.1 | HI002239.1 | HW399409.1 | BD132852.1 | GN042697.1 | HW258828.1 |
| BD495246.1 | FU258272.1 | DD405620.1 | DL088602.1 | HI002189.1 | HW399320.1 | BD131908.1 | GN041643.1 | HW257381.1 |
| BD454040.1 | FU258208.1 | DD405600.1 | DL112948.1 | HI002146.1 | HW408983.1 | BD131203.1 | GN038519.1 | HW257253.1 |
| BD453904.1 | GM623628.1 | DD405568.1 | DL092327.1 | HI002108.1 | HW391391.1 | BD130801.1 | GN037294.1 | HW257061.1 |
| BD453871.1 | GM623564.1 | DD405536.1 | DL092295.1 | HI000453.1 | HW390583.1 | BD130769.1 | GN034786.1 | HW256933.1 |

| | | | | | | | | |
|---|---|---|---|---|---|---|---|---|
| BD453839.1 | GM827562.1 | DD405504.1 | DL092263.1 | HI000405.1 | HW390517.1 | BD130735.1 | GN030745.1 | HW256805.1 |
| BD453807.1 | GM658713.1 | DD405472.1 | DL092231.1 | HI000360.1 | HW389562.1 | BD130703.1 | GN030713.1 | HW256709.1 |
| BD453751.1 | GM658681.1 | DD405440.1 | DL092199.1 | HI553291.1 | HW389139.1 | BD130613.1 | GN030681.1 | HW249738.1 |
| BD453719.1 | GM658649.1 | DD402034.1 | DL088488.1 | HI553259.1 | HW388970.1 | BD105785.1 | GN030649.1 | HW241250.1 |
| BD453687.1 | GM658617.1 | DD402002.1 | DL088456.1 | HI553219.1 | HW388628.1 | BD090566.1 | GN030617.1 | HW247882.1 |
| BD453655.1 | GM658553.1 | DD401970.1 | DL088424.1 | HI553187.1 | HW387849.1 | BD087270.1 | GN030585.1 | HW247830.1 |
| BD453623.1 | GM651361.1 | DD401909.1 | DL088392.1 | HI539599.1 | HW386522.1 | BD085726.1 | GN030553.1 | HW240957.1 |
| BD453586.1 | GM651329.1 | DD401877.1 | DL048687.1 | HI539178.1 | HW380843.1 | BD082908.1 | GN030521.1 | HW240893.1 |
| BD453573.1 | GM651297.1 | DD401845.1 | DL045237.1 | HI583971.1 | HW144740.1 | BD081899.1 | GN030489.1 | HW239307.1 |
| BD453541.1 | GM644402.1 | DD401813.1 | DL045205.1 | HI207668.1 | HW125666.1 | BD081336.1 | GN030457.1 | HW239164.1 |
| BD453509.1 | GM644370.1 | DD401749.1 | DL045173.1 | HI003699.1 | HW125418.1 | BD080679.1 | GN030361.1 | HW238634.1 |
| BD453477.1 | GM644338.1 | DD401717.1 | DL045141.1 | HI002073.1 | HW144370.1 | BD080431.1 | GN030265.1 | HV455310.1 |
| BD453445.1 | GM644274.1 | DD401685.1 | DL045109.1 | HI002023.1 | HW144338.1 | BD077096.1 | GN030233.1 | HV503175.1 |
| BD453413.1 | GM644242.1 | DD401653.1 | DL037078.1 | HI001940.1 | HW124888.1 | BD075541.1 | GN030201.1 | HV503143.1 |
| BD443134.1 | GM637244.1 | DD405399.1 | DL037046.1 | HI000272.1 | HW124792.1 | BD074983.1 | GM658111.1 | HV503111.1 |
| BD453236.1 | GM637212.1 | DD405367.1 | DL037014.1 | HI000232.1 | HW124422.1 | BD074947.1 | HI465511.1 | HV503079.1 |
| BD453204.1 | GM627486.1 | DD405303.1 | DL028355.1 | HI000179.1 | HW123421.1 | BD074776.1 | HC470486.1 | HV502779.1 |
| BD442911.1 | GM627454.1 | DD405271.1 | DL021169.1 | HI000132.1 | HW122428.1 | BD073262.1 | HC488035.1 | HV502747.1 |
| BD442737.1 | GM627390.1 | DD405239.1 | DL021137.1 | AY774952.1 | HW120091.1 | BD063670.1 | HC477799.1 | HV502715.1 |
| BD442213.1 | GM627358.1 | BD311489.1 | DL021105.1 | AY774910.1 | HW118011.1 | BD017722.1 | HC486444.1 | HV502683.1 |
| BD399074.1 | GM627326.1 | BD319671.1 | HC045682.1 | AY774854.1 | HW106668.1 | BD016618.1 | HC475382.1 | HV502651.1 |
| BD441330.1 | GM661260.1 | BD319300.1 | DM378907.1 | AY774739.1 | HW113814.1 | BD014228.1 | HC466637.1 | HV502619.1 |
| BD495914.1 | GM661228.1 | BD318882.1 | HC036995.1 | AY774684.1 | HW087318.1 | BD014196.1 | HC466605.1 | HV502587.1 |
| BD408111.1 | GM661196.1 | BD308198.1 | HC026858.1 | AY774625.1 | HW097006.1 | BD014127.1 | HC474322.1 | HV502555.1 |
| BD408079.1 | GM654138.1 | BD307653.1 | HC025572.1 | AY774560.1 | HW096370.1 | E63778.1 | HC474154.1 | HV502523.1 |
| BD450222.1 | GM654106.1 | BD300466.1 | HC025500.1 | AY774509.1 | HW072716.1 | AX482567.1 | HC491792.1 | HV502491.1 |
| BD429797.1 | GM654074.1 | BD294537.1 | HC025468.1 | AY774457.1 | HW072662.1 | AX481250.1 | HC491760.1 | HV492637.1 |
| BD419021.1 | GM654042.1 | BD293458.1 | HC025436.1 | AY774396.1 | HV586239.1 | AX472008.1 | HC491728.1 | HV505298.1 |
| BD429576.1 | GM654010.1 | BD293418.1 | HC025404.1 | AY774340.1 | HV601072.1 | E60071.1 | HC481592.1 | HV502806.1 |
| BD407507.1 | GM653978.1 | BD293151.1 | HC010422.1 | AY774181.1 | HV574861.1 | AX468946.1 | HC471546.1 | HV453595.1 |
| BD418443.1 | GM646985.1 | BD292451.1 | GM659896.1 | AY774120.1 | HV585641.1 | AX468882.1 | HC491496.1 | HV450065.1 |
| BD429038.1 | GM646953.1 | BD291398.1 | GM659864.1 | AY774048.1 | HV565943.1 | AX468448.1 | FV531715.1 | HV455247.1 |
| BD396348.1 | GM646921.1 | BD291258.1 | GM659800.1 | GM649083.1 | HV573256.1 | HW340172.1 | FV531683.1 | HV451943.1 |
| BD438203.1 | GM646889.1 | BD299788.1 | GM659768.1 | GM649051.1 | HV559940.1 | HW349954.1 | FV522821.1 | HV453160.1 |
| BD396030.1 | GM646857.1 | BD298756.1 | GM659736.1 | GM649019.1 | HV555944.1 | HW339399.1 | FV534450.1 | HV452828.1 |
| BD428093.1 | GM646825.1 | BD298632.1 | GM659700.1 | GM642152.1 | HV561869.1 | HW348519.1 | AY340667.1 | HV449162.1 |
| CQ970056.1 | GM640022.1 | BD297455.1 | GM659668.1 | GM642120.1 | HV560728.1 | HW347994.1 | AF275952.1 | HV450874.1 |
| DL011674.1 | GM639990.1 | BD296891.1 | GM659636.1 | GM642088.1 | HV549966.1 | HW363645.1 | HC456286.1 | HV444168.1 |
| DL011642.1 | GM639958.1 | BD296849.1 | GM659604.1 | GM642056.1 | HV553195.1 | HW363500.1 | HC453680.1 | HV437577.1 |
| DL011610.1 | GM639926.1 | BD295265.1 | GM652315.1 | GM642024.1 | HV552790.1 | HW355440.1 | HC453648.1 | HV437536.1 |
| DL048625.1 | GM639894.1 | BD285363.1 | GM652283.1 | GM629743.1 | HV551455.1 | HW355114.1 | HC459937.1 | HV348805.1 |
| DL048593.1 | GM639862.1 | BD289766.1 | GM645388.1 | GM629711.1 | HV550397.1 | HW344513.1 | HB435366.1 | HV351534.1 |
| DL048561.1 | GM627315.1 | BD281339.1 | GM645356.1 | GM719724.1 | AY659399.1 | HW326502.1 | HB432493.1 | HV341350.1 |
| DL048529.1 | GM627288.1 | BD287360.1 | GM645292.1 | GM886351.1 | AY659367.1 | HW339050.1 | HB423077.1 | HV341231.1 |
| DL048497.1 | GM627256.1 | BD277280.1 | GM645260.1 | GM669151.1 | AY659335.1 | HW338594.1 | HB416453.1 | FW501717.1 |
| DL048465.1 | GM627224.1 | BD276954.1 | GM645228.1 | GM656024.1 | AY659303.1 | HW338466.1 | HB396993.1 | FW496912.1 |
| DL044848.1 | GM627192.1 | BD279397.1 | GM652150.1 | GM655992.1 | AY659271.1 | HW338210.1 | HB394677.1 | FW420344.1 |

| | | | | | | | | |
|---|---|---|---|---|---|---|---|---|
| DL044816.1 | GM627160.1 | BD276129.1 | GM645195.1 | GM655960.1 | AY659239.1 | HW337826.1 | HB394243.1 | FW503232.1 |
| DL044784.1 | GM627128.1 | BD274286.1 | GM645163.1 | GM648967.1 | AY659207.1 | HW337698.1 | HB397726.1 | HI653924.1 |
| DL044720.1 | GM661112.1 | BD274158.1 | GM645131.1 | GM648935.1 | AY659175.1 | HW337570.1 | HB397657.1 | HI653865.1 |
| DL044688.1 | GM661080.1 | BD273447.1 | GM645099.1 | DL112255.1 | AY659143.1 | HW337442.1 | DM118016.1 | HI653797.1 |
| DL044632.1 | GM661048.1 | BD272328.1 | GM645067.1 | DL112223.1 | AY659111.1 | HW337058.1 | DM115615.1 | HI653566.1 |
| DL044536.1 | GM661016.1 | BD271896.1 | GM645035.1 | DL112191.1 | AY659079.1 | HW321878.1 | DM114890.1 | HI653514.1 |
| DL044504.1 | GM660984.1 | BD271121.1 | GM638108.1 | DL112159.1 | AY659047.1 | HW336802.1 | GM668922.1 | HI653402.1 |
| DL044472.1 | GM660952.1 | BD271052.1 | GM638076.1 | DL112127.1 | AY659015.1 | HW336566.1 | GM038728.1 | HI653344.1 |
| DL040456.1 | GM653958.1 | BD270869.1 | GM633338.1 | DL112095.1 | AY658983.1 | HW329580.1 | GM061293.1 | HI651896.1 |
| DL040424.1 | GM653926.1 | BD270209.1 | GM633306.1 | DL107092.1 | AY658951.1 | HW321118.1 | HB388688.1 | HI651033.1 |
| CS604388.1 | GM653894.1 | DD231070.1 | GM633274.1 | DL107060.1 | AY658919.1 | HW329448.1 | HB387949.1 | HI650447.1 |
| CS604356.1 | GM653862.1 | DD230079.1 | GM633251.1 | DL107028.1 | AY658887.1 | HW329330.1 | HB386982.1 | HI646810.1 |
| CS604292.1 | GM653784.1 | HV450592.1 | GM633219.1 | DL106996.1 | AY658855.1 | HW329217.1 | HB385914.1 | AY732495.1 |
| CS604260.1 | GM646757.1 | HV436967.1 | GM633155.1 | DL106964.1 | AY658823.1 | HW336164.1 | HB385770.1 | HH804914.1 |
| CS604164.1 | GM646725.1 | HV436932.1 | GM633123.1 | DL106932.1 | HI660936.1 | HW336064.1 | HB384570.1 | HH802661.1 |
| CS603844.1 | GM646693.1 | HV436864.1 | GM633091.1 | DL102159.1 | HI657180.1 | HW335976.1 | HB338910.1 | HH794944.1 |
| CS603780.1 | GM646661.1 | HV444634.1 | GM745572.1 | DL102127.1 | FW420950.1 | HW328744.1 | HA643287.1 | HH794712.1 |
| CS603716.1 | GM646629.1 | HV444558.1 | GM649460.1 | DL116807.1 | FW503007.1 | HW328680.1 | HA642062.1 | HH792828.1 |
| CS603460.1 | GM639826.1 | HV444350.1 | GM649428.1 | DL116775.1 | FW498005.1 | HW155743.1 | HA641544.1 | HH787616.1 |
| CS603428.1 | GM639794.1 | HV444012.1 | GM670036.1 | DL116743.1 | FW496020.1 | HW155623.1 | HA641143.1 | HH779670.1 |
| CS603396.1 | GM639762.1 | HV350059.1 | GM656531.1 | DL111926.1 | FW504723.1 | HW144782.1 | HA635316.1 | FW345051.1 |
| CS603364.1 | GM639730.1 | HV438125.1 | GM656499.1 | DL106859.1 | FW498887.1 | HW155229.1 | HA639788.1 | FW345005.1 |
| CS603332.1 | GM639698.1 | HV443182.1 | GM656467.1 | DL106827.1 | FW420466.1 | HW158874.1 | HA639025.1 | FW344973.1 |
| CS603300.1 | GM627087.1 | HV443093.1 | GM656435.1 | DL106795.1 | FW420402.1 | HW158483.1 | DM110295.1 | FW344682.1 |
| CS603268.1 | GM646557.1 | HV437488.1 | GM649338.1 | DL106763.1 | FW504520.1 | HW154642.1 | FB509356.1 | FW343730.1 |
| CS603236.1 | GM646525.1 | HV437315.1 | GM649250.1 | DL106731.1 | FW501718.1 | HW154507.1 | FB509322.1 | FW343662.1 |
| CS603204.1 | GM646493.1 | HV349091.1 | GM649218.1 | DL106699.1 | FW496914.1 | HW154443.1 | FB509258.1 | FW343534.1 |
| CS603172.1 | GM646461.1 | HV344048.1 | GM642448.1 | DL091269.1 | FW420377.1 | HW147477.1 | FB509211.1 | HC924162.1 |
| CS603140.1 | GM646429.1 | HV343820.1 | GM635234.1 | DL091237.1 | FW420345.1 | HW154305.1 | FB509179.1 | HC923000.1 |
| CS603108.1 | GM639530.1 | HI593858.1 | GM635170.1 | DL091205.1 | FW420288.1 | HW158147.1 | FB509145.1 | FW343728.1 |
| CS603076.1 | GM639448.1 | HI588402.1 | GM635138.1 | DL091173.1 | FW498500.1 | HW154117.1 | GM772972.1 | FW343596.1 |
| CS603044.1 | GM639490.1 | HI564112.1 | GM635106.1 | DL087205.1 | FW496664.1 | HW153793.1 | GM709012.1 | FW343532.1 |
| CS603012.1 | GM626887.1 | HI636980.1 | GM635074.1 | DL102098.1 | FW503284.1 | HW153757.1 | GM708980.1 | FW343213.1 |
| CS602978.1 | GM626855.1 | HI001457.1 | GM630268.1 | DL102066.1 | HI653925.1 | HW153259.1 | GM655009.1 | HC922999.1 |
| CS602946.1 | GM626823.1 | HI001405.1 | GM630236.1 | DL102034.1 | HI653798.1 | HW151828.1 | GM654977.1 | HC921524.1 |
| CS602914.1 | GM653638.1 | HI001361.1 | GM630204.1 | DL097524.1 | HI653515.1 | HW144744.1 | GM044463.1 | HC920683.1 |
| CS602882.1 | GM653606.1 | HI001327.1 | GM635039.1 | DL097492.1 | HI653403.1 | HV932132.1 | GM837723.1 | HD063944.1 |
| CS602850.1 | GM653574.1 | HI003210.1 | GM635007.1 | DL097460.1 | HI653354.1 | HV929185.1 | GM043745.1 | DM381314.1 |
| CS602786.1 | DL112163.1 | HI003160.1 | GM634975.1 | DL101885.1 | HI651897.1 | HV937092.1 | GM752804.1 | HC054856.1 |
| CS602754.1 | DL112131.1 | HI003092.1 | GM634943.1 | DL101821.1 | HI650448.1 | HV931858.1 | GM752446.1 | HC053864.1 |
| CS602722.1 | DL112099.1 | HI003052.1 | GM634911.1 | DL101789.1 | HI646811.1 | HV936914.1 | DL467581.1 | HC053464.1 |
| CS602690.1 | DL112067.1 | HI003011.1 | GM634879.1 | DL101757.1 | HI646140.1 | HV931353.1 | GM712950.1 | HC051927.1 |
| CS611200.1 | DL107064.1 | HI002912.1 | GM629977.1 | DL101725.1 | HH804915.1 | HV820121.1 | GM652752.1 | HC045494.1 |
| CS611068.1 | DL107032.1 | HI001278.1 | GM629945.1 | DL097215.1 | HH802662.1 | HV936858.1 | GM652720.1 | HC045462.1 |
| CS601988.1 | DL107000.1 | HI001177.1 | GM629913.1 | DL097183.1 | HD058860.1 | HV936389.1 | GM652688.1 | HC045430.1 |
| CS608380.1 | DL106968.1 | HI001103.1 | GM656298.1 | DL097151.1 | HD057683.1 | HV819654.1 | GM652656.1 | HC045398.1 |
| CS607921.1 | DL102219.1 | HI001085.1 | GM656266.1 | DL095227.1 | HW408969.1 | HV819467.1 | GM652624.1 | HC045366.1 |

| | | | | | | | | |
|---|---|---|---|---|---|---|---|---|
| CS607534.1 | DL102163.1 | HI001029.1 | GM656202.1 | DL113988.1 | HW382468.1 | HV814571.1 | GM645498.1 | HC045334.1 |
| CS600632.1 | DL102131.1 | HI000995.1 | GM656170.1 | DL113956.1 | HW381851.1 | HV814358.1 | GM645466.1 | HC045270.1 |
| CS607089.1 | DL116871.1 | HI002851.1 | GM649177.1 | DL113924.1 | HW390564.1 | HV932690.1 | GM645434.1 | HC047034.1 |
| CS607006.1 | DL116839.1 | HI002819.1 | GM649145.1 | DL113892.1 | HW389658.1 | HV932619.1 | GM638541.1 | HC047002.1 |
| CS606800.1 | DL096467.1 | HI002768.1 | GM649113.1 | DL113860.1 | HW389137.1 | HV930027.1 | GM638509.1 | HC046970.1 |
| CS403526.1 | DL094543.1 | HI002719.1 | GM649081.1 | DL109059.1 | HW257348.1 | HV932328.1 | GM638477.1 | HC046938.1 |
| CS403042.1 | DL094511.1 | HI002674.1 | GM642150.1 | DL109027.1 | HW257284.1 | HV781588.1 | GM638445.1 | HC046906.1 |
| CS408088.1 | DL094479.1 | HI002636.1 | GM642118.1 | DL108995.1 | HW257124.1 | HV802942.1 | GM633746.1 | HC046878.1 |
| CS402506.1 | DL110712.1 | HI002601.1 | GM642086.1 | DL104293.1 | HW257060.1 | HV802910.1 | GM633714.1 | HC046846.1 |
| CS402182.1 | DL110680.1 | HI002551.1 | GM642054.1 | DL104261.1 | HW256708.1 | HV813713.1 | GM633682.1 | HC046814.1 |
| CS402101.1 | DL090742.1 | HI004563.1 | GM642022.1 | DL104229.1 | HW251052.1 | HV818593.1 | GM625815.1 | HC046782.1 |
| CS401795.1 | DL090710.1 | HI004435.1 | GM719210.1 | DL099591.1 | HW249737.1 | HV817910.1 | GM625751.1 | HC046750.1 |
| CS376594.1 | DL090678.1 | HI002466.1 | GM717978.1 | DL099559.1 | HW249700.1 | HV775762.1 | GM642240.1 | HC046718.1 |
| DD309772.1 | DL086577.1 | HI002406.1 | GM669149.1 | DL093453.1 | HW241249.1 | HV532751.1 | GM656333.1 | HC039692.1 |
| DD298469.1 | DL086545.1 | HI002329.1 | GM706382.1 | DL093421.1 | HW240924.1 | HV536508.1 | GM656301.1 | HC046066.1 |
| DD308742.1 | DL086513.1 | HI000536.1 | GM603494.1 | DL093389.1 | HW240612.1 | HV535860.1 | GM656269.1 | HC046034.1 |
| DD292517.1 | DL086449.1 | HI180556.1 | FB715284.1 | DL093357.1 | HW239304.1 | HV532106.1 | GM656237.1 | HC045996.1 |
| DD291489.1 | DL086417.1 | HI180173.1 | FB677405.1 | DL126134.1 | HW239163.1 | HV531983.1 | GM656205.1 | HC045964.1 |
| DD291020.1 | DL115519.1 | HI178231.1 | GM061239.1 | DL126102.1 | HW238264.1 | HV516105.1 | GM656173.1 | HC045932.1 |
| CS365144.1 | DL115487.1 | HI202813.1 | GM061134.1 | DL122750.1 | HW242992.1 | HV515581.1 | GM649180.1 | HC045868.1 |
| CS376164.1 | DL115455.1 | HI177879.1 | DL459976.1 | DL122694.1 | HW244247.1 | HV515549.1 | GM649148.1 | HC045804.1 |
| CS375816.1 | DL105639.1 | HI214573.1 | DL258263.1 | DL122662.1 | HW237887.1 | HV515517.1 | CS118527.1 | HC045772.1 |
| CS375761.1 | DL105607.1 | HI214541.1 | DL241176.1 | DL118408.1 | HW237846.1 | HV515485.1 | CS118461.1 | HC045740.1 |
| CS362748.1 | DL100898.1 | HI177809.1 | DL241034.1 | DL118376.1 | HW248676.1 | HV515453.1 | CS118396.1 | HC046094.1 |
| CS378187.1 | DL100866.1 | HI214443.1 | FB748876.1 | DL118344.1 | HW242310.1 | HV515421.1 | CS118364.1 | HC045678.1 |
| CS359745.1 | DL100834.1 | HI200558.1 | FB674303.1 | DL113820.1 | HW243423.1 | HV515357.1 | CS118329.1 | DM378889.1 |
| CS359641.1 | DL100802.1 | HI213785.1 | GN029940.1 | DL113788.1 | HW241636.1 | HV515325.1 | CS118295.1 | HC035659.1 |
| BD269790.1 | DL100770.1 | HI202271.1 | GN029908.1 | DL113748.1 | HW243086.1 | HV515293.1 | CS118263.1 | GN114925.1 |
| BD269146.1 | DL100738.1 | HI213016.1 | GN029842.1 | DL108859.1 | HW243013.1 | HV515261.1 | A23987.1 | GN100131.1 |
| BD268254.1 | DL096324.1 | HI212976.1 | GN029810.1 | DL108827.1 | HW163842.1 | HV515229.1 | AX458513.1 | DM075130.1 |
| BD268124.1 | DL096292.1 | HI547165.1 | GN013594.1 | DL108795.1 | HV984259.1 | HV511228.1 | AX456488.1 | DM078716.1 |
| BD265618.1 | DL096260.1 | HI212477.1 | GN013370.1 | DL108763.1 | HV961484.1 | HV514045.1 | AX455875.1 | DM077871.1 |
| BD263456.1 | DL096228.1 | HI588172.1 | GN010293.1 | DL108731.1 | HV960458.1 | HV510352.1 | AX454139.1 | GN090930.1 |
| BD263342.1 | DL096196.1 | HI546328.1 | GN033576.1 | DL108699.1 | HV959620.1 | HV513326.1 | AX453992.1 | GN087185.1 |
| BD262202.1 | DL096164.1 | HC867568.1 | GN033544.1 | DL104053.1 | HV964130.1 | HV509436.1 | AX452029.1 | GN089990.1 |
| BD251967.1 | DL120184.1 | HC867480.1 | GN033512.1 | DL099519.1 | HV959218.1 | HV508704.1 | A34753.1 | GN089844.1 |
| BD251466.1 | DL120152.1 | HC867392.1 | GN033448.1 | DL099487.1 | HV966032.1 | HV508621.1 | AX443295.1 | GN093651.1 |
| BD251236.1 | DL120120.1 | HC882467.1 | GN033416.1 | DL099455.1 | HV963587.1 | HV508557.1 | AX428025.1 | GN082853.1 |
| BD250291.1 | DL120088.1 | HC872701.1 | GN033384.1 | HC471697.1 | HV969951.1 | HV508497.1 | AX427113.1 | GN089187.1 |
| BD247113.1 | DL120024.1 | HC856035.1 | GN033352.1 | HC471641.1 | HV962320.1 | HV508465.1 | AX421217.1 | GN088031.1 |
| BD246841.1 | DL094342.1 | HC880622.1 | GN033320.1 | HC471609.1 | HV962084.1 | HV512267.1 | AX418553.1 | GN078159.1 |
| BD244790.1 | DL094310.1 | HC878539.1 | GN033288.1 | HC728984.1 | HV965333.1 | HV512235.1 | AX411433.1 | L08953.1 |
| BD243446.1 | DL094246.1 | HC869709.1 | GN033256.1 | HC688539.1 | HV951617.1 | HV512203.1 | AX404752.1 | L08938.1 |
| CQ831499.1 | DL094214.1 | HC869645.1 | GN033160.1 | HC688435.1 | HV951585.1 | HV512171.1 | M35969.1 | L08858.1 |
| CQ830072.1 | DL094182.1 | HC876977.1 | GN033064.1 | HC490833.1 | HV951553.1 | HV512139.1 | AX395278.1 | DM059612.1 |
| CQ828081.1 | DL094150.1 | HC869489.1 | GN032904.1 | HC490801.1 | HV951238.1 | JA660270.1 | AX384851.1 | DM063453.1 |
| CQ827366.1 | DL090467.1 | HC876937.1 | GN032872.1 | HC490769.1 | HV950901.1 | HV492264.1 | AX384579.1 | DM058826.1 |

| | | | | | | | | |
|---|---|---|---|---|---|---|---|---|
| CQ824425.1 | DL090435.1 | HC876898.1 | GN032808.1 | HC490737.1 | HV947216.1 | HV504947.1 | AX384037.1 | DM057750.1 |
| CQ821487.1 | DL113695.1 | HC868272.1 | GN032776.1 | HC490705.1 | HV940412.1 | HV504915.1 | AX382258.1 | GM637315.1 |
| CQ819452.1 | DL113663.1 | FW310412.1 | GM634910.1 | HC490544.1 | HV538570.1 | HV504883.1 | AX378882.1 | GM637283.1 |
| CQ818764.1 | DL108862.1 | FW331857.1 | GM634878.1 | HC490509.1 | HV543335.1 | HV504851.1 | AX378008.1 | GM637251.1 |
| CQ818566.1 | DL108830.1 | FW309714.1 | GM629976.1 | HC490477.1 | HV543208.1 | HV504819.1 | AX364680.1 | GM623650.1 |
| CQ817040.1 | DL108798.1 | FW308800.1 | GM629912.1 | HC490406.1 | HV542740.1 | HV504738.1 | AX364648.1 | GM623618.1 |
| CQ816978.1 | DL108766.1 | FW333478.1 | GM669850.1 | HC490374.1 | HV542708.1 | HV504706.1 | AX364563.1 | GM623586.1 |
| CQ816937.1 | DL108734.1 | FW307696.1 | GM656329.1 | HC472299.1 | HV541664.1 | HV504674.1 | AX364531.1 | GM623554.1 |
| CQ815748.1 | DL108702.1 | FW306954.1 | GM656297.1 | HC472266.1 | HV515097.1 | HV504642.1 | AX364499.1 | GM644232.1 |
| CQ814064.1 | DL104056.1 | FW332603.1 | GM656265.1 | HC472234.1 | HV515729.1 | HV504610.1 | AX364467.1 | GM637106.1 |
| CQ814031.1 | DL099522.1 | FW332319.1 | GM656233.1 | HC472202.1 | HV515575.1 | HI657187.1 | AX364435.1 | GM637074.1 |
| CQ813999.1 | DL099490.1 | HC855997.1 | GM656201.1 | HC472170.1 | HV515511.1 | FW420982.1 | AX364371.1 | GM632672.1 |
| CQ813967.1 | DL099458.1 | HC769809.1 | GM656169.1 | HC471970.1 | HV515415.1 | FW503015.1 | AX364339.1 | GM632640.1 |
| CQ813935.1 | DL099426.1 | HC768625.1 | GM649176.1 | HC471938.1 | HV515351.1 | HW293285.1 | AX364212.1 | GM658450.1 |
| CQ813903.1 | DL099394.1 | HC767576.1 | GM649144.1 | HC471906.1 | HV515319.1 | HW292750.1 | AX364179.1 | GM651042.1 |
| CQ813871.1 | DL099362.1 | HC310381.1 | GM649112.1 | HC471874.1 | HV515255.1 | HW291166.1 | AX359858.1 | GM651010.1 |
| CQ813839.1 | DL099330.1 | HC310303.1 | GM642181.1 | HC490534.1 | HV507920.1 | HW291037.1 | AX358640.1 | GM644211.1 |
| CQ813807.1 | DL089513.1 | HC310072.1 | GM642149.1 | HC678835.1 | HV509347.1 | HW298873.1 | BD006757.1 | GM644147.1 |
| CQ813774.1 | DL089481.1 | GM659417.1 | GM642117.1 | HC504636.1 | HV508682.1 | HW290910.1 | E50934.1 | GM644115.1 |
| CQ813734.1 | DL089449.1 | GM659385.1 | GM642085.1 | HC494417.1 | HV508650.1 | HW290878.1 | E63238.1 | GM636925.1 |
| CQ813113.1 | DL089417.1 | GM659353.1 | GM642053.1 | HC504267.1 | HV508615.1 | HW290846.1 | E55070.1 | GM636893.1 |
| CQ806534.1 | DL089385.1 | GM652161.1 | GM642021.1 | HC500242.1 | HV508551.1 | HW290110.1 | E58877.1 | GM636861.1 |
| CQ802133.1 | DL113647.1 | GM652129.1 | GM719209.1 | HC499862.1 | HV508519.1 | HW298193.1 | AX354672.1 | GM623522.1 |
| CQ798412.1 | DL113615.1 | GM652097.1 | GM717971.1 | HB426103.1 | HV508459.1 | HW298046.1 | HW376091.1 | GM623490.1 |
| CQ796854.1 | DL113583.1 | GM652065.1 | GM669148.1 | HB423114.1 | HV512293.1 | HW289757.1 | HW365420.1 | GM623458.1 |
| CQ796373.1 | DL113551.1 | GM652033.1 | GM648964.1 | HB403848.1 | HV512261.1 | HW289022.1 | HW364740.1 | GM623426.1 |
| CQ795489.1 | DL113519.1 | GM645206.1 | GM648932.1 | HB416451.1 | HV512229.1 | HW285404.1 | HW381042.1 | GM623394.1 |
| CQ793261.1 | DL113455.1 | GM645174.1 | GM648900.1 | HB412672.1 | HV512197.1 | HW279582.1 | HW353906.1 | GM623362.1 |
| CQ789542.1 | DL103760.1 | GM645142.1 | GM648868.1 | HB396951.1 | HV512165.1 | HW267839.1 | HW353779.1 | GM654787.1 |
| CQ788163.1 | DL103728.1 | GM645078.1 | GM648836.1 | HB394775.1 | HV512133.1 | HW267796.1 | HW353594.1 | GM647762.1 |
| CQ787487.1 | DL093144.1 | GM645046.1 | GM648804.1 | HB394501.1 | HV492193.1 | HW267469.1 | HW352330.1 | GM647698.1 |
| CQ787455.1 | DL093112.1 | GM645014.1 | GM634557.1 | HB394273.1 | FW552198.1 | HW266206.1 | HW340703.1 | GM627960.1 |
| CQ787341.1 | DL093080.1 | GM638215.1 | GM634525.1 | HB394241.1 | FW552161.1 | HW265988.1 | HW350977.1 | GM627928.1 |
| CQ787307.1 | DL089273.1 | GM638183.1 | GM634493.1 | HB397724.1 | FW552123.1 | HW263027.1 | HW340485.1 | GM654744.1 |
| CQ787275.1 | DL089241.1 | GM638151.1 | GM629688.1 | DM118012.1 | FW555590.1 | HW262915.1 | HW350852.1 | GM640627.1 |
| CQ786939.1 | DL125932.1 | GM638119.1 | GM664860.1 | DM115608.1 | FW563145.1 | HW262872.1 | HW350680.1 | HV693347.1 |
| CQ784665.1 | DL125900.1 | GM638087.1 | GM648740.1 | DM114864.1 | FW561692.1 | HW262610.1 | HW350646.1 | HV245468.1 |
| CQ784473.1 | DL125868.1 | GM651899.1 | GM648708.1 | GM668920.1 | FW561660.1 | HW262572.1 | HW256714.1 | HV245319.1 |
| CQ779568.1 | DL125836.1 | GM651867.1 | GM648676.1 | GM061288.1 | FW562821.1 | HW261406.1 | HW250937.1 | HV236072.1 |
| AX282869.1 | DL125804.1 | GM651835.1 | GM648644.1 | HB388686.1 | FW562742.1 | HW261342.1 | HW250262.1 | HV247222.1 |
| AX282188.1 | DL125772.1 | GM651803.1 | GM648612.1 | HB387469.1 | FW565210.1 | HW261310.1 | HW249639.1 | HV306173.1 |
| AX280213.1 | DL122562.1 | GM645004.1 | CS728675.1 | HB386561.1 | FW562593.1 | HW261278.1 | HW247839.1 | HV302967.1 |
| AX279950.1 | DL122530.1 | GM644972.1 | CS728643.1 | HB385945.1 | FW566793.1 | HW261246.1 | HW240738.1 | HV302935.1 |
| AX279679.1 | DL118116.1 | GM644940.1 | CS727216.1 | HB385768.1 | FW508100.1 | HW261214.1 | HW240682.1 | HV308614.1 |
| AX278751.1 | DL118084.1 | GM644908.1 | GM036649.1 | HB341668.1 | FW562486.1 | HW261182.1 | HW241315.1 | HV226842.1 |
| AX278087.1 | DL118020.1 | GM644876.1 | GM836388.1 | HB384561.1 | FW553151.1 | HW261150.1 | HW239393.1 | HV235784.1 |
| AX254403.1 | DL113331.1 | GM644844.1 | GM868793.1 | HB340096.1 | FW556658.1 | HW261118.1 | HW239173.1 | HV313063.1 |

| | | | | | | | | |
|---|---|---|---|---|---|---|---|---|
| AX252993.1 | DL113299.1 | GM644812.1 | GM680775.1 | HB338906.1 | FW559378.1 | HW261086.1 | HW238754.1 | HV200501.1 |
| AX242330.1 | DL108466.1 | GM638013.1 | GM618698.1 | HA643036.1 | FW506209.1 | HW261054.1 | HW238561.1 | HV200233.1 |
| AX242298.1 | DL012124.1 | GM637981.1 | GM721270.1 | HA642264.1 | HI936414.1 | HW260990.1 | HW238441.1 | HV182450.1 |
| AX242202.1 | DL012092.1 | GM637949.1 | GM059991.1 | HA641604.1 | HI930635.1 | HW260958.1 | HW238403.1 | HV182418.1 |
| AX242170.1 | DL012060.1 | GM637917.1 | GM841716.1 | HA641540.1 | HI935281.1 | HW260926.1 | HW242998.1 | HV182386.1 |
| AX242074.1 | DL011938.1 | GM637885.1 | GM604025.1 | HA639756.1 | GM630585.1 | HW260894.1 | HW242955.1 | HV203194.1 |
| AX242010.1 | DL011906.1 | GM637853.1 | GM603688.1 | HA637720.1 | GM621650.1 | HW260862.1 | HW237893.1 | HV232479.1 |
| AX241946.1 | DL011874.1 | GM633445.1 | GM706608.1 | DM102630.1 | GM621618.1 | HW260830.1 | HW237852.1 | HV039468.1 |
| AX241914.1 | DL024285.1 | GM633413.1 | GM706381.1 | GM625084.1 | GM621586.1 | HW260798.1 | HW248689.1 | HV038676.1 |
| AX241882.1 | DL024253.1 | GM633381.1 | GM603493.1 | GM625052.1 | GM621554.1 | HW260766.1 | HW242316.1 | HV038588.1 |
| AX241850.1 | AX814296.1 | GM633349.1 | GM839146.1 | GM625020.1 | GM657134.1 | HW260734.1 | HW243429.1 | HV182348.1 |
| AX241818.1 | AX809452.1 | GM633317.1 | GM601143.1 | GM624988.1 | GM656943.1 | HW260702.1 | HW241644.1 | HV182316.1 |
| AX241786.1 | AX798909.1 | GM633285.1 | FB715280.1 | GM624956.1 | GM656911.1 | HW044239.1 | HW241470.1 | HI593985.1 |
| AX241754.1 | AX798341.1 | GM637830.1 | FB706731.1 | GM623834.1 | GM656879.1 | HW053993.1 | HW163853.1 | HI588457.1 |
| AX241722.1 | AX797704.1 | GM637798.1 | FB677402.1 | GM623802.1 | GM656847.1 | HV975521.1 | HW155851.1 | HI637002.1 |
| AX241690.1 | AX796862.1 | GM637766.1 | FB676841.1 | GM623770.1 | GM870221.1 | HV985966.1 | HV774925.1 | HI636921.1 |
| AX241658.1 | AX796747.1 | GM637734.1 | GM061232.1 | GM658917.1 | GM627576.1 | HV985934.1 | HV764769.1 | HI001376.1 |
| AX241626.1 | AX795622.1 | GM637702.1 | GM061133.1 | GM658885.1 | GM627544.1 | HV985902.1 | HV764737.1 | HI001344.1 |
| AX241530.1 | AX795436.1 | GM637670.1 | DL262702.1 | GM658853.1 | GM869935.1 | HW028877.1 | HV774547.1 | HI003188.1 |
| AX241498.1 | BD189023.1 | GM627715.1 | DL260579.1 | GM658821.1 | GM661485.1 | HV984388.1 | HV774476.1 | HI003131.1 |
| AX241466.1 | BD188045.1 | GM627683.1 | DL259494.1 | GM644606.1 | GM661453.1 | HV984264.1 | HV764242.1 | HI003069.1 |
| AX241434.1 | AX786416.1 | GM627651.1 | DL258261.1 | GM637416.1 | GM661421.1 | HV986783.1 | HV763361.1 | HI003034.1 |
| AX241127.1 | AX777463.1 | GM627635.1 | DL241175.1 | GM637352.1 | GM661389.1 | HV961489.1 | HV766040.1 | HI002994.1 |
| AX241095.1 | AX773929.1 | GM627591.1 | DL241033.1 | GM637320.1 | GM661357.1 | HV964441.1 | HV765957.1 | HI002930.1 |
| AX241063.1 | AX772854.1 | GM627559.1 | DL240796.1 | GM637288.1 | GM661325.1 | HV960512.1 | HV755979.1 | HI001295.1 |
| AX241031.1 | AX769823.1 | GM627527.1 | DL124642.1 | GM637256.1 | GM654331.1 | HV960142.1 | HV758532.1 | HI001258.1 |
| AX240999.1 | AX768078.1 | GM714492.1 | DL124610.1 | GM623655.1 | GM654299.1 | HV963641.1 | HV504203.1 | HI001198.1 |
| AX240967.1 | HW408641.1 | GM661500.1 | DL124578.1 | GM623623.1 | GM654267.1 | HV965979.1 | BD176076.1 | HI001147.1 |
| AX240935.1 | HW408577.1 | GM661468.1 | DL096913.1 | GM623591.1 | GM647178.1 | HV969956.1 | BD175967.1 | HI001044.1 |
| AX237003.1 | HW408418.1 | GM661436.1 | DL096849.1 | GM623559.1 | GM647146.1 | HV969803.1 | A27454.1 | HI001010.1 |
| AX236447.1 | HW390753.1 | GM661404.1 | DL120313.1 | GM658708.1 | GM647114.1 | HV961659.1 | A16273.1 | HI000964.1 |
| AX235234.1 | HW399540.1 | GM661372.1 | DL120281.1 | GM658676.1 | GM647082.1 | HV955898.1 | A20270.1 | HI002869.1 |
| AX225202.1 | HW399331.1 | GM661340.1 | DJ339561.1 | GM658644.1 | GM647021.1 | HV950639.1 | A20514.1 | HI002834.1 |
| AX208065.1 | HW399228.1 | GM654346.1 | DJ334568.1 | GM658612.1 | GM661298.1 | HV950378.1 | A13978.1 | HI002796.1 |
| AX207291.1 | HW390664.1 | GM654282.1 | DJ334048.1 | GM658548.1 | GM646879.1 | HV553639.1 | A11624.1 | HI002737.1 |
| AX203114.1 | HW390525.1 | GM654250.1 | DJ326979.1 | GM651324.1 | GM646847.1 | HV551416.1 | A10452.1 | HI002651.1 |
| AX202551.1 | HW390479.1 | GM654218.1 | DJ128113.1 | GM644397.1 | GM646815.1 | HV550401.1 | A08533.1 | HI002617.1 |
| AX202427.1 | HW389027.1 | GM654186.1 | DJ122419.1 | GM644365.1 | GM640012.1 | HV550352.1 | A02810.1 | HI002578.1 |
| AY043012.1 | HW388884.1 | GM647161.1 | DJ086519.1 | GM644333.1 | GM639980.1 | AY659402.1 | A32067.1 | HI004657.1 |
| AX193684.1 | HW388636.1 | GM647129.1 | DJ082684.1 | GM644237.1 | GM639948.1 | AY659370.1 | A31090.1 | HI004529.1 |
| AX188685.1 | HW387855.1 | GM647097.1 | DJ082559.1 | GM637207.1 | GM639916.1 | AY659338.1 | A30427.1 | HI004401.1 |
| AX180279.1 | HW387452.1 | GM647034.1 | DJ086079.1 | GM637175.1 | GM627246.1 | AY659306.1 | A29939.1 | HI002426.1 |
| AX174631.1 | HW387042.1 | GM647057.1 | DJ066773.1 | GM637143.1 | GM627182.1 | AY659274.1 | A29093.1 | HI002309.1 |
| AX172941.1 | HW386304.1 | GM647004.1 | DJ066377.1 | GM637111.1 | GM627150.1 | AY659242.1 | A28875.1 | HI000511.1 |
| AX167412.1 | HW386144.1 | GM640134.1 | DJ066345.1 | HV944622.1 | GM660974.1 | AY659210.1 | A27945.1 | HI006496.1 |
| AX166328.1 | HW380894.1 | GM640102.1 | DJ066312.1 | HV943100.1 | GM660942.1 | AY659178.1 | A27190.1 | HI180156.1 |
| AX155103.1 | HW380817.1 | GM640070.1 | DJ066278.1 | HV779877.1 | GM646747.1 | AY659146.1 | A24678.1 | HI202828.1 |

| | | | | | | | | |
|---|---|---|---|---|---|---|---|---|
| AX149429.1 | HW366919.1 | GM640038.1 | DJ066244.1 | HV773780.1 | GM646715.1 | AY659114.1 | A22539.1 | HI214588.1 |
| AX146695.1 | HW376093.1 | GM627491.1 | DJ068739.1 | HV743750.1 | GM646683.1 | AY659082.1 | A22077.1 | HI214556.1 |
| DL024221.1 | HW365426.1 | GM627427.1 | DJ058770.1 | HV568924.1 | GM646651.1 | AY659050.1 | A16248.1 | HI214524.1 |
| DL024189.1 | HW365428.1 | GM627395.1 | DJ055280.1 | HW286512.1 | GM646619.1 | AY659018.1 | A12818.1 | HI214458.1 |
| DL040777.1 | HW150070.1 | GM627331.1 | DJ055197.1 | HW295866.1 | DL123665.1 | AY658986.1 | A21627.1 | HI214426.1 |
| DL024022.1 | HW150006.1 | GM661315.1 | DJ057592.1 | HW302534.1 | DL123601.1 | AY658954.1 | A21032.1 | HI185523.1 |
| DL016412.1 | HW144803.1 | GM661265.1 | DJ060402.1 | HW295575.1 | DL123569.1 | AY658922.1 | A20267.1 | HI212995.1 |
| DL016348.1 | HW155529.1 | GM661233.1 | DJ056343.1 | HW295055.1 | DL119379.1 | AY658890.1 | A20770.1 | HC292936.1 |
| DL016316.1 | HW158780.1 | GM661201.1 | DJ053107.1 | HW285865.1 | DL119347.1 | AY658858.1 | A16019.1 | HC295705.1 |
| DL016284.1 | HW154737.1 | GM654143.1 | DJ053075.1 | HW294138.1 | DL119315.1 | AY658826.1 | A12612.1 | HC288860.1 |
| DL028153.1 | HW154644.1 | GM654111.1 | CS810631.1 | HW293282.1 | DL114823.1 | AY658794.1 | A12293.1 | HC292506.1 |
| DL011736.1 | HW154477.1 | GM654079.1 | CS810278.1 | HW292747.1 | DL114791.1 | AY658762.1 | A11906.1 | HC292266.1 |
| DL011704.1 | HW154445.1 | GM654047.1 | CS813002.1 | HW291163.1 | DL114759.1 | AY658730.1 | A10359.1 | HC295008.1 |
| DL011672.1 | HW154307.1 | GM654015.1 | DJ021070.1 | HW291066.1 | DL114727.1 | AY658698.1 | A09217.1 | HC294828.1 |
| DL011640.1 | HW147121.1 | GM653983.1 | DJ016468.1 | HW291034.1 | DL114695.1 | AY658666.1 | A08834.1 | HC299123.1 |
| DL011608.1 | HW153719.1 | GM646990.1 | DD402366.1 | HW290970.1 | DL114663.1 | AY658634.1 | A06409.1 | HC294588.1 |
| DL044814.1 | HW145129.1 | GM646958.1 | DD402334.1 | HW290907.1 | DL127330.1 | AY658602.1 | A08995.1 | HC298736.1 |
| DL044750.1 | HW151993.1 | GM646894.1 | DD402270.1 | HW290875.1 | DL141463.1 | AY658570.1 | A01657.1 | HC291066.1 |
| DL044718.1 | HW151656.1 | GM646862.1 | DD402238.1 | HW290333.1 | DL086196.1 | AY658538.1 | A05171.1 | HC290881.1 |
| DL044686.1 | HW144746.1 | GM646830.1 | DD402206.1 | HW290107.1 | DL123465.1 | HW159804.1 | A01580.1 | HC293469.1 |
| DL026174.1 | HW144376.1 | GM639995.1 | DD406119.1 | HW290013.1 | DL123433.1 | HV961488.1 | A00131.1 | HC293349.1 |
| DL018984.1 | HW144344.1 | GM639963.1 | DD405586.1 | HW289019.1 | DL123401.1 | HV960465.1 | M62714.1 | HC293152.1 |
| DL018952.1 | HW144312.1 | GM639931.1 | DD405554.1 | HV695523.1 | DL123369.1 | HV960141.1 | K00516.1 | HC289867.1 |
| DL018920.1 | HV937043.1 | GM639899.1 | DD405522.1 | HV695218.1 | DL119115.1 | HV959244.1 | K01485.1 | HC289692.1 |
| DL018888.1 | HV815180.1 | HH999118.1 | DD405490.1 | HV694836.1 | DL119083.1 | HV963600.1 | M60085.1 | DM464645.1 |
| DL018856.1 | HV931270.1 | HH999088.1 | DD405458.1 | HV694673.1 | DL119051.1 | HV965944.1 | M19079.1 | HC195331.1 |
| DL018824.1 | HV931140.1 | HH999051.1 | DD405426.1 | HV693368.1 | DL119019.1 | HV969955.1 | LC732341.1 | HC195217.1 |
| DL034331.1 | HV936297.1 | HH998990.1 | DD402020.1 | HV699789.1 | DL114623.1 | HV962089.1 | MH238440.1 | HC193589.1 |
| DL014235.1 | HV930399.1 | HH998936.1 | DD401988.1 | HV699148.1 | DL114591.1 | HV965361.1 | M19060.1 | HC191138.1 |
| DL009383.1 | HV819469.1 | HH997214.1 | DD401927.1 | HV698607.1 | DL114559.1 | HV969802.1 | KX377895.1 | HC190459.1 |
| DL009351.1 | HV814573.1 | HH970110.1 | DD401863.1 | HV690769.1 | DL114527.1 | HV961656.1 | M19104.1 | HC089575.1 |
| DL009319.1 | HV814361.1 | HH998889.1 | DD401831.1 | HV689785.1 | DL114495.1 | HV951621.1 | HW144742.1 | HC089543.1 |
| DL009287.1 | HV803286.1 | HH998829.1 | DD401799.1 | HV579904.1 | DL114463.1 | HV951557.1 | HW144486.1 | HC089511.1 |
| DL009255.1 | HV782088.1 | HH998786.1 | DD401623.1 | HV600337.1 | DL104896.1 | HV955896.1 | HW126611.1 | HC089479.1 |
| DL009223.1 | HV932628.1 | HH997135.1 | DD401591.1 | HV585119.1 | DL104864.1 | HV950638.1 | HW125421.1 | HC089447.1 |
| DL018681.1 | HV929922.1 | HH997072.1 | DD401559.1 | HV585087.1 | DL104832.1 | HV957934.1 | HW144450.1 | HC089414.1 |
| DL018649.1 | HV932330.1 | HH997013.1 | DD401527.1 | HV584759.1 | DL104800.1 | HV949620.1 | HW144372.1 | HC089382.1 |
| DL018617.1 | HV803146.1 | HH996963.1 | DD401495.1 | HV579121.1 | DL095941.1 | HV947220.1 | HW144340.1 | HC089350.1 |
| DL009037.1 | HV803114.1 | HH998717.1 | DD401463.1 | HV579002.1 | DL095909.1 | HV945915.1 | HW144308.1 | HC089317.1 |
| DL014221.1 | HV802944.1 | HH998682.1 | CS479879.1 | HV592643.1 | DL095877.1 | HV939907.1 | HW124798.1 | HC089285.1 |
| DL014189.1 | HV802912.1 | HH998607.1 | CS479219.1 | HV578515.1 | DL114428.1 | HV943481.1 | HW124425.1 | HC089253.1 |
| DL014157.1 | HV802880.1 | HH998567.1 | CS476351.1 | HV592285.1 | DL114396.1 | HV943270.1 | HW124059.1 | AX144951.1 |
| DL014125.1 | HV818962.1 | HH996931.1 | CS467756.1 | HV582881.1 | DL114364.1 | HV755057.1 | HW122137.1 | AX144918.1 |
| DL014093.1 | HV818569.1 | HH996869.1 | DD382829.1 | HV601495.1 | DL044266.1 | HV753778.1 | HW121672.1 | AX144886.1 |
| DL014061.1 | HV817759.1 | HH996777.1 | DD400153.1 | HV575506.1 | DL040090.1 | HV753554.1 | HW120093.1 | AX144854.1 |
| DL014032.1 | HV784495.1 | HH994570.1 | CS458931.1 | FW575878.1 | DL032033.1 | HV743340.1 | HW106698.1 | AX144822.1 |
| DL014006.1 | HV778616.1 | HH981030.1 | DD361284.1 | FW571815.1 | DL032001.1 | HV743300.1 | HW113908.1 | AX144790.1 |

| | | | | | | | | |
|---|---|---|---|---|---|---|---|---|
| DL013974.1 | HV778228.1 | HH980684.1 | DD367612.1 | HI980647.1 | DL031969.1 | HV743263.1 | HW087322.1 | AX144758.1 |
| DL013942.1 | HV777261.1 | HH980505.1 | DD368339.1 | HI979270.1 | DL028128.1 | HV743224.1 | HW097012.1 | AX144726.1 |
| DL013910.1 | HV775776.1 | HH980437.1 | DD368148.1 | HI989133.1 | DL028096.1 | HV704411.1 | HW096799.1 | AX144694.1 |
| DL013878.1 | HV775195.1 | HH957940.1 | DD361318.1 | HI988906.1 | DL028064.1 | HV744580.1 | HW105139.1 | AX144662.1 |
| DL030042.1 | HV775031.1 | HH980364.1 | BD450496.1 | HI987467.1 | DL028032.1 | HV703328.1 | HW096453.1 | AX144630.1 |
| DL030010.1 | HV774990.1 | HH980314.1 | BD391410.1 | HI987425.1 | DL028000.1 | HV701836.1 | HW072718.1 | AX144583.1 |
| DL029978.1 | FZ415918.1 | HH980166.1 | BD388861.1 | HH794945.1 | DL027968.1 | HV701242.1 | HW072665.1 | AX144197.1 |
| DL046635.1 | FZ421435.1 | HH980132.1 | BD378634.1 | HH792829.1 | DL044233.1 | HV701210.1 | HW096216.1 | AX144133.1 |
| DL046603.1 | FZ411824.1 | HH980061.1 | BD375869.1 | HH779671.1 | DL044201.1 | HV701178.1 | HW072447.1 | AX144069.1 |
| DL046571.1 | FZ417833.1 | HH979887.1 | BD374741.1 | HH774429.1 | DL044169.1 | HV701146.1 | HW072220.1 | AX144003.1 |
| DL046539.1 | FZ420847.1 | HH996724.1 | BD350065.1 | HH820950.1 | DL048242.1 | HV701114.1 | HV932130.1 | AX143939.1 |
| DL042653.1 | FZ417037.1 | HH996650.1 | BD356416.1 | HH820909.1 | CS244154.1 | HV708219.1 | HV936912.1 | AX143747.1 |
| DL042621.1 | FZ413888.1 | HH996611.1 | BD353556.1 | HH759205.1 | CS244202.1 | HV701095.1 | HV931351.1 | AX143491.1 |
| DL042589.1 | FZ419859.1 | HH996554.1 | BD359032.1 | HH759173.1 | CS243764.1 | HV701063.1 | HV936825.1 | AX143363.1 |
| DL038665.1 | FZ419823.1 | HH998467.1 | BD342462.1 | HH759141.1 | CS243155.1 | HV701031.1 | HV931266.1 | AX143299.1 |
| DL038633.1 | FZ419791.1 | HH998417.1 | BD349498.1 | HH759109.1 | CS239682.1 | HV695525.1 | HV930759.1 | AX143107.1 |
| DL029907.1 | DL017982.1 | HH998373.1 | BD325526.1 | HH759077.1 | CS230953.1 | HV321827.1 | HV819647.1 | AX142787.1 |
| DL029875.1 | DL017950.1 | HH998340.1 | BD314365.1 | HH759045.1 | DD214009.1 | HV334353.1 | HV819465.1 | AX142723.1 |
| DL029843.1 | DL017918.1 | HH999958.1 | BD319288.1 | HH759013.1 | DD213992.1 | HV325843.1 | HV803282.1 | AX142659.1 |
| DD495507.1 | DL017886.1 | HH999909.1 | BD319462.1 | HH758981.1 | DD216436.1 | HV324500.1 | HV932688.1 | AX142531.1 |
| CS671264.1 | DL017854.1 | HH979826.1 | BD318825.1 | HH758949.1 | DD213576.1 | HV187242.1 | HV802940.1 | AX142465.1 |
| DD465260.1 | DL017822.1 | HH979676.1 | BD318128.1 | HH757461.1 | DD213544.1 | HV192774.1 | HV802908.1 | HV969948.1 |
| CS647650.1 | DL017791.1 | HH976527.1 | BD308668.1 | HH757429.1 | DD218527.1 | HV221662.1 | HV813711.1 | HV965330.1 |
| CS646651.1 | DL017727.1 | HH999873.1 | BD307584.1 | HI401436.1 | DD215946.1 | HV247805.1 | HV818591.1 | HV961705.1 |
| CS646198.1 | DL017695.1 | HH999784.1 | BD292601.1 | HI401121.1 | DD222029.1 | HV301792.1 | HV818565.1 | HV950628.1 |
| CS644250.1 | DL017663.1 | HH999730.1 | BD292424.1 | HD117885.1 | DD215567.1 | HV301607.1 | HV817908.1 | HV947313.1 |
| DD462051.1 | DL017631.1 | HH998298.1 | BD291310.1 | HI642895.1 | DD212247.1 | HV302972.1 | HV780270.1 | HV940217.1 |
| DD460660.1 | DL022269.1 | HH998253.1 | BD298668.1 | HH998236.1 | DD211833.1 | HV302940.1 | HV779496.1 | HV939880.1 |
| DD455854.1 | DL022237.1 | HH998216.1 | BD297641.1 | HH998189.1 | DD182426.1 | HV308718.1 | HV778691.1 | HV943370.1 |
| DD455157.1 | DL022205.1 | HH998164.1 | BD295099.1 | HH998148.1 | DD206911.1 | HV308619.1 | HV778612.1 | HV932181.1 |
| DD458366.1 | DL025560.1 | HH993621.1 | BD280283.1 | HH993696.1 | DD206815.1 | HV304167.1 | HV444461.1 | HV936882.1 |
| DD453824.1 | DL029638.1 | HH979642.1 | BD274307.1 | HH986630.1 | DD206785.1 | HV194938.1 | HV444180.1 | HV925519.1 |
| DD457791.1 | DL039099.1 | HH924305.1 | BD273664.1 | HH999706.1 | DD196875.1 | HV313068.1 | HV350009.1 | HV939037.1 |
| DD456889.1 | DL035475.1 | HH932042.1 | CS018792.1 | HH979627.1 | AJ965435.1 | HV214381.1 | HC688428.1 | HV936380.1 |
| CS631723.1 | DL035443.1 | HH931964.1 | CS016577.1 | HH979585.1 | AX924084.1 | HV040126.1 | HC490830.1 | HV936343.1 |
| CS631295.1 | DL035411.1 | FW417373.1 | CS016539.1 | HH932378.1 | AX923396.1 | HV188521.1 | HC490798.1 | HV560562.1 |
| CS631216.1 | DL035379.1 | FW416860.1 | CS008907.1 | HH931988.1 | AX840275.1 | FW573831.1 | HC490766.1 | HV554547.1 |
| CS627762.1 | DL035347.1 | FW394276.1 | CQ987001.1 | AX100675.1 | AX838455.1 | FW570253.1 | HC490734.1 | HV553149.1 |
| CS632844.1 | DL031183.1 | FW416390.1 | HW328559.1 | FW394239.1 | AX828671.1 | FW573669.1 | HB817101.1 | HV551399.1 |
| CS632379.1 | DL031151.1 | FW394257.1 | HW328485.1 | FW394203.1 | AX824420.1 | FW573461.1 | HB840195.1 | HV550392.1 |
| CS626511.1 | DL027342.1 | FW394215.1 | HW328441.1 | FW418238.1 | AX823794.1 | FW571749.1 | HB848114.1 | AY659394.1 |
| CS625895.1 | DL027310.1 | FW394183.1 | HW328482.1 | FW396438.1 | AX823760.1 | HI967414.1 | HB839676.1 | AY659362.1 |
| DD450357.1 | DL027278.1 | FW397520.1 | HW328161.1 | HH936068.1 | AX817767.1 | HI988910.1 | HB847297.1 | AY659330.1 |
| DD438000.1 | DL027246.1 | FW418783.1 | HW318660.1 | HH935094.1 | AX816157.1 | HI987470.1 | HB838883.1 | AY659298.1 |
| DD449091.1 | DL027214.1 | FW418602.1 | HW318474.1 | HH834134.1 | AX816032.1 | HI987427.1 | HB846799.1 | AY659266.1 |
| DD435090.1 | DL027182.1 | FW418140.1 | HW335179.1 | FW383147.1 | AX814430.1 | FW561970.1 | HB838452.1 | AY659234.1 |
| CS619879.1 | DL023370.1 | FW396760.1 | HW314150.1 | HH930552.1 | AX813417.1 | FW552165.1 | HB846371.1 | AY659202.1 |

| | | | | | | | | |
|---|---|---|---|---|---|---|---|---|
| CS617807.1 | DL023338.1 | HC310070.1 | HW311996.1 | HH932402.1 | AX805921.1 | FW552128.1 | HB846072.1 | AY659170.1 |
| CS614472.1 | DL023306.1 | HC310038.1 | HW311032.1 | HC441563.1 | AX800144.1 | FW554619.1 | HB809885.1 | AY659138.1 |
| CS613794.1 | DL023274.1 | HC310006.1 | HW308594.1 | HC358405.1 | AX799985.1 | HI637698.1 | HB826599.1 | AY659106.1 |
| CS612788.1 | DL023242.1 | HC309974.1 | HW307937.1 | HV182451.1 | AX799564.1 | HI380393.1 | HB837574.1 | AY659074.1 |
| CS246937.1 | DL023210.1 | HC309747.1 | HW307863.1 | HV182419.1 | AX798988.1 | HI380323.1 | HB845572.1 | AY659042.1 |
| CS244165.1 | DL023178.1 | HC308856.1 | HW307831.1 | HV182387.1 | AX798330.1 | HI378159.1 | HB845146.1 | AY659010.1 |
| CS244245.1 | DL020099.1 | HC307868.1 | HW307799.1 | HV235489.1 | AX798173.1 | HI378127.1 | HB844414.1 | AY658978.1 |
| CS244213.1 | DL020067.1 | HC307803.1 | HW307761.1 | HV038589.1 | AX797693.1 | HI378095.1 | HB836907.1 | AY658946.1 |
| GN032172.1 | DL010950.1 | HC307374.1 | HW307729.1 | HV182349.1 | AX794736.1 | HI376931.1 | HB836456.1 | AY658914.1 |
| GN032140.1 | DL010918.1 | HC312264.1 | HW307697.1 | HV182317.1 | AX472475.1 | HI376617.1 | HB843555.1 | AY658882.1 |
| GN032108.1 | DL010886.1 | FU258211.1 | HW315557.1 | HV182285.1 | BD194481.1 | HI375920.1 | HB836251.1 | AY658850.1 |
| GN032076.1 | DL010854.1 | FU258179.1 | HW315449.1 | HV182049.1 | BD188798.1 | HI375885.1 | HB830440.1 | AY658818.1 |
| GN032044.1 | DL010822.1 | FU260929.1 | HW315068.1 | HV190954.1 | AX781462.1 | HI372212.1 | HB843099.1 | AY658786.1 |
| GN032012.1 | DL023143.1 | FU257935.1 | HW069916.1 | HV190827.1 | AX773270.1 | HI369625.1 | HB835517.1 | AY658754.1 |
| GN031980.1 | DL023047.1 | FU257839.1 | HW096214.1 | HV216286.1 | AX772705.1 | HI369160.1 | HB807560.1 | AY658722.1 |
| GN031948.1 | DL023015.1 | FU257775.1 | HW104300.1 | HV306507.1 | AX770675.1 | HI369105.1 | HB824019.1 | AY658690.1 |
| GN031916.1 | DL022983.1 | FU260838.1 | HW071175.1 | HV302067.1 | AX766567.1 | HI425011.1 | HB842467.1 | AY658658.1 |
| GN031884.1 | DL010723.1 | FU271866.1 | HW070966.1 | HV037788.1 | AX766177.1 | HI424510.1 | HB850378.1 | AY658594.1 |
| GN031852.1 | DL010691.1 | FU263078.1 | HW070554.1 | FZ435960.1 | AX754964.1 | HI424133.1 | HB840734.1 | AY658562.1 |
| GN031787.1 | DL010659.1 | FU262700.1 | HW099596.1 | FZ427593.1 | AX752755.1 | HI424101.1 | HB828661.1 | AY658530.1 |
| GN031722.1 | DL010627.1 | FU262653.1 | HW066586.1 | FZ430140.1 | AX752627.1 | HI424069.1 | HB648673.1 | AY658498.1 |
| GN031690.1 | DL042909.1 | FU250478.1 | HW083257.1 | FZ429669.1 | AX750676.1 | HI423843.1 | HB645707.1 | AY658466.1 |
| GN031658.1 | DL042877.1 | FU262465.1 | HW083181.1 | FZ429393.1 | AX038741.1 | HI423000.1 | HB855095.1 | AY658434.1 |
| GN031626.1 | DL038964.1 | FU267898.1 | HW088259.1 | HH999902.1 | AX037317.1 | HH762706.1 | HB858478.1 | AY658402.1 |
| GN031562.1 | DL038932.1 | FU253497.1 | HW081764.1 | HH999859.1 | AX036013.1 | HI415749.1 | HB855983.1 | AY658370.1 |
| GN031530.1 | CS671257.1 | FU261571.1 | HW081612.1 | HH998325.1 | AX028805.1 | GN359504.1 | HB855890.1 | AY658338.1 |
| GN031498.1 | CS669947.1 | FU269786.1 | HW085151.1 | HH998271.1 | AX027710.1 | GN359436.1 | DM170849.1 | AY658306.1 |
| GN031466.1 | CS646196.1 | FU258431.1 | HW085070.1 | HH998234.1 | AX024040.1 | GN359404.1 | DM170816.1 | AY658274.1 |
| GN031433.1 | CS644244.1 | FU258399.1 | HW103377.1 | HH998187.1 | AX023691.1 | GN359372.1 | DM188068.1 | AY658242.1 |
| GN031401.1 | DD462049.1 | HC311305.1 | HW103133.1 | HH998136.1 | AX023641.1 | GN365267.1 | GN031235.1 | AY658210.1 |
| GN031369.1 | DD455456.1 | DL105359.1 | HW102893.1 | HH993688.1 | AX023608.1 | GN359203.1 | GN031203.1 | AY658178.1 |
| GN031337.1 | DD455213.1 | DL105327.1 | HW098197.1 | HH986628.1 | AX023575.1 | GN359171.1 | GN031171.1 | AY658146.1 |
| GN031305.1 | DD455187.1 | DL124346.1 | HW061848.1 | HH979555.1 | AX019927.1 | GN363821.1 | GN031106.1 | AY658114.1 |
| GN031272.1 | DD458205.1 | DL124314.1 | HW058814.1 | HH979625.1 | AX018742.1 | GN363026.1 | GN031074.1 | AY658082.1 |
| GN031240.1 | DD453822.1 | DL110175.1 | HW065328.1 | HH979583.1 | AX002861.1 | GN346506.1 | GN031042.1 | AY658050.1 |
| GN031176.1 | DD457761.1 | DL110143.1 | HW065230.1 | HH931986.1 | AX010930.1 | GN346472.1 | GN031010.1 | AY658018.1 |
| GN031143.1 | DD456727.1 | DL110111.1 | HV755087.1 | FW394401.1 | AX008936.1 | GN343927.1 | GN030978.1 | AY657986.1 |
| GN031079.1 | DD456380.1 | DL110079.1 | HV755023.1 | FW394237.1 | AX008143.1 | GN342149.1 | GN030947.1 | AY657954.1 |
| GN031047.1 | CS643141.1 | DL105178.1 | HV749413.1 | FW394201.1 | AX006186.1 | GN348295.1 | GN030915.1 | AY657922.1 |
| GN031015.1 | CS631721.1 | DL105146.1 | HV748626.1 | FW398045.1 | AF133206.1 | GN346715.1 | GN030883.1 | AY657890.1 |
| GN030983.1 | CS631291.1 | DL105114.1 | HV748516.1 | FW397554.1 | A57341.1 | GN346579.1 | GN030819.1 | AY657858.1 |
| GN030952.1 | CS631212.1 | DL100700.1 | HV748372.1 | FW393383.1 | A25438.1 | GN346547.1 | GN030787.1 | AY657826.1 |
| GN030920.1 | CS642182.1 | DL100668.1 | HV747499.1 | FW396436.1 | A35730.1 | GN131886.1 | DM008345.1 | AY657794.1 |
| GN030888.1 | CS627758.1 | DL100636.1 | HV753776.1 | HH935025.1 | A34826.1 | GN116494.1 | DM001983.1 | AY657762.1 |
| GN030856.1 | CS632290.1 | DL100604.1 | HV753633.1 | HH835106.1 | A34018.1 | GN116429.1 | DM001672.1 | AY657730.1 |
| GN030824.1 | CS623654.1 | DL100572.1 | HV753543.1 | HH834132.1 | A33973.1 | GN116397.1 | DM001618.1 | AY657698.1 |
| GN030792.1 | CS623596.1 | DL100540.1 | HV743338.1 | HH833903.1 | A32815.1 | GN116365.1 | DM001466.1 | HI401112.1 |

| | | | | | | | | |
|---|---|---|---|---|---|---|---|---|
| DM001471.1 | CS623532.1 | DL119994.1 | HV743298.1 | FW390825.1 | A32507.1 | GN116333.1 | DM006942.1 | GM652773.1 |
| DM006952.1 | CS626434.1 | DL119962.1 | HV743222.1 | FW392870.1 | A30366.1 | GN115065.1 | DM000513.1 | GM652741.1 |
| DM004995.1 | DD449089.1 | DL119930.1 | HV704249.1 | HH932397.1 | A27336.1 | GN094508.1 | DM005000.1 | GM652709.1 |
| DM003498.1 | CS619875.1 | DL119898.1 | HV703164.1 | HH821125.1 | A22251.1 | GN094476.1 | DM003493.1 | GM652677.1 |
| DL488434.1 | CS616542.1 | DL119866.1 | HV702432.1 | HC466417.1 | A20035.1 | GM658643.1 | GM997885.1 | GM645487.1 |
| GM998415.1 | CS614468.1 | DL119834.1 | HV701594.1 | HC482495.1 | A25932.1 | GM658611.1 | GM983069.1 | GM645455.1 |
| GM996587.1 | CS250565.1 | DL115239.1 | HV701240.1 | HC491785.1 | A16249.1 | GM658547.1 | GM995286.1 | GM645423.1 |
| GM996204.1 | CS249167.1 | DL115207.1 | HV701208.1 | HC491645.1 | A20093.1 | GM644396.1 | GN009997.1 | GM638530.1 |
| GM636900.1 | CS248776.1 | DL115175.1 | HV701176.1 | HC481645.1 | A18690.1 | GM644364.1 | GM992314.1 | GM638498.1 |
| GM636868.1 | CS247247.1 | DL115111.1 | HV701144.1 | HC481580.1 | A16192.1 | GM644236.1 | GM976393.1 | GM638466.1 |
| GM623529.1 | CS245391.1 | DL115079.1 | HV701112.1 | FV531708.1 | A13393.1 | GM637142.1 | GM975787.1 | GM633735.1 |
| GM623497.1 | CS244163.1 | DL115047.1 | HV708145.1 | FV531676.1 | A08278.1 | GM637110.1 | GM990841.1 | GM633703.1 |
| GM623465.1 | CS244243.1 | DL110047.1 | HV701093.1 | FV523747.1 | A04848.1 | GM637078.1 | GM989460.1 | GM633666.1 |
| GM623433.1 | CS244211.1 | DL018013.1 | HV701029.1 | FV522806.1 | A01480.1 | GM624925.1 | GN000560.1 | GM648349.1 |
| GM623401.1 | CS244179.1 | DL013326.1 | HV341796.1 | FV530139.1 | A30282.1 | GM624893.1 | GN000319.1 | GM648317.1 |
| GM623369.1 | CS243271.1 | DL013262.1 | HV335491.1 | FV528444.1 | A29013.1 | GM624861.1 | GM969694.1 | GM648285.1 |
| GM643888.1 | CS243248.1 | DL013230.1 | HV322858.1 | FV534614.1 | A28860.1 | GM632644.1 | GM969573.1 | GM648253.1 |
| GM643856.1 | CS195886.1 | DL013198.1 | HV322204.1 | FV534513.1 | GM037909.1 | GM658454.1 | GM981444.1 | GM648221.1 |
| GM643824.1 | CS227252.1 | DL017987.1 | HV321824.1 | FV534409.1 | FB764683.1 | GM651174.1 | GM631662.1 | GM641386.1 |
| GM636827.1 | CS208291.1 | DL017955.1 | HV333571.1 | FV534239.1 | FB743949.1 | GM651142.1 | GM631630.1 | GM641354.1 |
| GM636795.1 | CS189625.1 | DL017923.1 | HV313387.1 | FV534087.1 | FB743916.1 | GM651110.1 | GM631598.1 | GM641322.1 |
| GM636763.1 | CS186191.1 | DL017891.1 | HV187462.1 | HC465656.1 | FB743884.1 | GM651046.1 | GM622667.1 | GM641290.1 |
| GM632259.1 | CS254955.1 | DL017859.1 | HV187235.1 | HC461203.1 | FB743852.1 | GM651014.1 | GM704216.1 | GM641258.1 |
| GM632227.1 | CS252552.1 | DL017827.1 | HV192772.1 | HC456312.1 | FB743798.1 | GM650982.1 | GM704163.1 | GM641226.1 |
| GM623264.1 | CS249841.1 | DL017796.1 | HV227380.1 | HC453673.1 | FB742930.1 | GM644151.1 | GM622443.1 | GM629067.1 |
| GM623232.1 | CS250642.1 | DL017764.1 | HV247799.1 | HC458606.1 | FB730317.1 | GM644119.1 | GM657405.1 | GM629035.1 |
| GM741838.1 | CS249163.1 | DL017732.1 | HV247161.1 | HC442314.1 | DL095215.1 | GM636929.1 | GM657373.1 | GM629003.1 |
| GM741806.1 | CS244162.1 | DL017700.1 | HV302970.1 | HC452136.1 | DL128876.1 | GM636897.1 | GM650117.1 | GM880088.1 |
| GM658320.1 | CS244242.1 | DL017668.1 | HV302938.1 | HC451942.1 | DL128841.1 | GM636865.1 | GM650085.1 | GM648188.1 |
| GM658288.1 | CS244210.1 | DL017636.1 | HV312298.1 | HC449863.1 | DL128744.1 | GM623526.1 | GM643116.1 | GM662136.1 |
| GM658256.1 | CS244178.1 | DL022274.1 | HV309533.1 | FU756129.1 | DL111655.1 | GM623494.1 | GM643084.1 | FB713784.1 |
| GM658224.1 | CS243177.1 | DL022210.1 | FZ422518.1 | FU774061.1 | DL111591.1 | GM623430.1 | GM643020.1 | FB712618.1 |
| GM658192.1 | CS230015.1 | DL025757.1 | FZ416169.1 | FU761004.1 | DL097043.1 | GM623398.1 | GM622211.1 | FB674474.1 |
| GM658160.1 | CS231711.1 | DL025725.1 | FZ416910.1 | FU759994.1 | DL119476.1 | GM623366.1 | GM622179.1 | FB670510.1 |
| GM650968.1 | CS195883.1 | DL025693.1 | FZ419850.1 | GM969571.1 | DL119444.1 | GM643885.1 | GM622147.1 | FB667476.1 |
| GM741473.1 | CS227438.1 | DL025597.1 | FZ419817.1 | GN007485.1 | DL119412.1 | GM643853.1 | FB506781.1 | FB704837.1 |
| GM658078.1 | CS207917.1 | DL025565.1 | FZ419785.1 | GM984661.1 | DL114817.1 | GM643821.1 | GM646485.1 | FB676590.1 |
| GM658046.1 | CS189624.1 | DL029707.1 | FW580717.1 | FB740455.1 | DL114785.1 | GM636824.1 | GM646453.1 | FB676526.1 |
| GM658014.1 | CS188228.1 | DL029675.1 | FW590725.1 | GM596710.1 | DL114689.1 | GM636792.1 | GM646421.1 | FB705632.1 |
| GM657982.1 | DD161529.1 | DL025538.1 | FW590297.1 | GM969776.1 | DL114657.1 | GM636760.1 | GM647274.1 | FB705923.1 |
| GM657950.1 | DD161459.1 | DL025506.1 | FW589154.1 | DL477729.1 | DL141576.1 | GM632224.1 | GM647242.1 | FB701812.1 |
| GM650758.1 | DD167020.1 | DL025357.1 | FW593440.1 | DL476959.1 | DL141449.1 | GM741867.1 | GM647210.1 | FB660221.1 |
| GM650694.1 | DD173108.1 | DL025325.1 | HQ161063.1 | DL463064.1 | DL123491.1 | GM639524.1 | GM059988.1 | FB659873.1 |
| GM643702.1 | DD172258.1 | DL025293.1 | FW576785.1 | DL470107.1 | DL123459.1 | GM626881.1 | GM841713.1 | FB654401.1 |
| GM643670.1 | DD165145.1 | DL025229.1 | DL094174.1 | DL462902.1 | DL119109.1 | GM626849.1 | GM603971.1 | DL199775.1 |
| GM643630.1 | DD171109.1 | DL021953.1 | DL090495.1 | DL482393.1 | DL119077.1 | GM626817.1 | GM841209.1 | DL128717.1 |
| GM636641.1 | DD164867.1 | DL021921.1 | DL086294.1 | DL475870.1 | DL119045.1 | GM660765.1 | GM603489.1 | DL128683.1 |

| | | | | | | | | |
|---|---|---|---|---|---|---|---|---|
| GM636609.1 | DD163607.1 | DL021857.1 | DL086262.1 | DL475557.1 | DL119013.1 | GM660733.1 | GM602800.1 | DL128616.1 |
| GM636577.1 | CS203959.1 | DL021825.1 | DL086230.1 | DL481426.1 | DL114489.1 | GM653514.1 | FB676835.1 | DL124943.1 |
| GM636545.1 | AF405703.1 | DL021793.1 | DL086198.1 | FB504565.1 | DL114457.1 | GM653482.1 | GM061227.1 | DL128526.1 |
| GM636513.1 | CS157795.1 | DL017427.1 | DL110164.1 | GM832059.1 | DL095935.1 | GM653450.1 | DL262699.1 | DL128494.1 |
| GM636481.1 | CS157956.1 | DL038463.1 | DL110132.1 | GM712710.1 | DL095903.1 | GM653418.1 | DL260533.1 | DL104569.1 |
| GM632073.1 | CS157924.1 | DL038431.1 | DL110100.1 | GM712159.1 | DL095871.1 | GM653386.1 | DL257872.1 | DL104537.1 |
| GM623110.1 | CS157860.1 | DL038367.1 | DL105167.1 | GM692460.1 | CS793881.1 | GM646387.1 | DL241712.1 | DL099931.1 |
| GM623078.1 | CS157828.1 | DL038335.1 | DL105135.1 | GM745712.1 | CS800169.1 | GM646355.1 | DL241172.1 | DL123305.1 |
| FB743929.1 | CS143642.1 | DL013172.1 | DL100657.1 | GM828150.1 | CS720191.1 | GM646323.1 | DL241027.1 | DL109179.1 |
| FB743897.1 | CS048775.1 | DL013140.1 | DL119983.1 | DL183795.1 | CS720060.1 | GM646291.1 | DL240793.1 | DL109147.1 |
| FB743865.1 | CS047653.1 | DL013108.1 | DL119951.1 | FB571235.1 | CS716897.1 | GM646259.1 | FB748911.1 | DL109115.1 |
| FB743823.1 | CS022571.1 | DL013076.1 | DL119919.1 | CS696187.1 | CS716746.1 | GM646227.1 | FB674299.1 | DL109083.1 |
| FB743779.1 | CS018477.1 | DL013044.1 | DL119887.1 | CS696091.1 | CS716071.1 | GM639392.1 | DL233403.1 | DL104381.1 |
| FB761728.1 | CS018334.1 | DL013012.1 | DL119855.1 | CS696059.1 | CS675431.1 | GM639360.1 | FB714433.1 | DL104349.1 |
| GM660767.1 | CS018156.1 | DL033907.1 | DL119823.1 | CS695994.1 | CS680680.1 | GM639328.1 | DL101911.1 | DL104317.1 |
| GM660735.1 | CS016580.1 | DL033875.1 | DL115132.1 | CS695930.1 | CS792377.1 | GM639296.1 | DL101815.1 | DL123154.1 |
| GM653516.1 | CS016542.1 | DL033843.1 | DL110068.1 | CS695866.1 | DJ031170.1 | GM639264.1 | DL101783.1 | DL123122.1 |
| GM653484.1 | CS015787.1 | DL033779.1 | DL110036.1 | CS695738.1 | DJ044908.1 | GM626717.1 | DL101751.1 | DL049699.1 |
| GM653452.1 | CQ990433.1 | DL033747.1 | DL110004.1 | CS695514.1 | DJ028123.1 | GM646088.1 | DL101719.1 | DL049667.1 |
| GM653388.1 | CQ986630.1 | DL033715.1 | DL109972.1 | CS695450.1 | DJ027949.1 | GM646056.1 | DL095221.1 | DL045659.1 |
| GM646389.1 | CQ986596.1 | DL027379.1 | DL109726.1 | CS695418.1 | DJ043829.1 | GM646024.1 | DL121115.1 | DL033297.1 |
| GM646357.1 | CQ986564.1 | DL023567.1 | DL109694.1 | CS695352.1 | DJ026298.1 | GM626386.1 | DL111863.1 | DL033265.1 |
| GM646325.1 | CQ983150.1 | DL023535.1 | DL104960.1 | CS791577.1 | CS721668.1 | GM626354.1 | DL111831.1 | DL029129.1 |
| GM646293.1 | CQ983003.1 | DL023503.1 | DL104928.1 | DL176639.1 | CS675273.1 | GM660379.1 | DL128882.1 | DL029097.1 |
| GM646261.1 | CQ982971.1 | DL023471.1 | DL021100.1 | DL176477.1 | DJ008421.1 | GM660347.1 | DL128847.1 | DL029065.1 |
| GM646229.1 | CQ982824.1 | DL023439.1 | DL021068.1 | DL176434.1 | DJ008389.1 | GM653128.1 | DL128783.1 | DL021751.1 |
| GM639426.1 | CQ982743.1 | DL023407.1 | DL021036.1 | DL174613.1 | DJ008357.1 | GM653096.1 | DL116414.1 | DL021719.1 |
| GM639394.1 | CQ981123.1 | DL023375.1 | DL012116.1 | DL174457.1 | DJ008013.1 | GM653064.1 | DL116382.1 | DL010531.1 |
| GM639362.1 | CQ975461.1 | DL020360.1 | DL012084.1 | DL182869.1 | DJ003330.1 | GM653032.1 | DL116350.1 | DL010499.1 |
| GM639330.1 | CQ974066.1 | DL020328.1 | DL011930.1 | DL176757.1 | CS401400.1 | GM653000.1 | DL111661.1 | DL010467.1 |
| GM639298.1 | CQ973484.1 | DL020296.1 | DL011898.1 | FB343377.1 | CS406376.1 | DL095406.1 | DL111629.1 | DL010435.1 |
| GM639266.1 | AX814469.1 | DL020264.1 | DL020807.1 | DL091956.1 | CS408805.1 | FB676501.1 | DL111597.1 | DL010403.1 |
| GM626719.1 | AX814406.1 | DL020232.1 | DL016404.1 | DL091924.1 | CS401810.1 | FB675466.1 | DL111565.1 | DL046800.1 |
| GM660564.1 | AX809443.1 | DL020200.1 | DL016340.1 | DL091892.1 | CS382593.1 | FB701550.1 | DL111501.1 | DL038777.1 |
| GM660532.1 | AX805933.1 | DL015797.1 | DL016308.1 | DL091860.1 | DD298326.1 | CS809027.1 | DL101687.1 | DL035057.1 |
| GM653345.1 | AX805215.1 | DL015765.1 | DL016276.1 | DL102885.1 | DD295621.1 | FB660483.1 | DL101623.1 | DL030710.1 |
| GM646090.1 | AX799572.1 | AY659343.1 | DL016244.1 | DL102853.1 | DD288827.1 | FB665213.1 | DL106619.1 | DL030678.1 |
| GM646058.1 | AX799144.1 | AY659311.1 | DL011728.1 | DL102821.1 | CS376111.1 | FB654415.1 | DL106555.1 | DL026741.1 |
| GM646026.1 | AX798189.1 | AY659279.1 | DL011696.1 | DL107569.1 | BD269161.1 | FB583259.1 | DL106523.1 | DL026709.1 |
| GM626516.1 | AX797701.1 | AY659247.1 | DL042967.1 | DL107505.1 | BD269129.1 | DL111955.1 | DL136438.1 | DL019498.1 |
| GM626484.1 | AX796859.1 | AY659215.1 | DL042935.1 | DL069374.1 | BD268885.1 | DL111923.1 | DL128687.1 | DL019466.1 |
| GM626452.1 | AX796743.1 | AY659183.1 | DL042903.1 | DL049701.1 | BD265649.1 | DL106856.1 | DL128620.1 | DL019434.1 |
| GM626420.1 | AX795619.1 | AY659151.1 | DL038990.1 | DL049669.1 | BD265599.1 | DL106824.1 | DL128588.1 | DL019402.1 |
| GM626388.1 | AX795586.1 | AY659119.1 | DL038958.1 | DL049628.1 | BD263843.1 | DL106792.1 | DL048681.1 | CS448872.1 |
| GM626356.1 | AX795554.1 | AY659087.1 | DL038926.1 | DL033299.1 | BD263439.1 | DL091266.1 | DL012115.1 | A18867.1 |
| GM660381.1 | AX795430.1 | AY659055.1 | DL030904.1 | DL033267.1 | BD263057.1 | DL091234.1 | DL012051.1 | CS444594.1 |
| GM660349.1 | BD188842.1 | AY659023.1 | DL030737.1 | DL029131.1 | BD261171.1 | DL091202.1 | DL012019.1 | CS446159.1 |

| | | | | | | | | |
|---|---|---|---|---|---|---|---|---|
| GM653130.1 | AX787428.1 | AY658991.1 | DL026928.1 | DL029099.1 | BD250960.1 | DL091170.1 | DL011929.1 | CS438928.1 |
| GM653098.1 | AX787371.1 | AY658959.1 | DL019749.1 | DL029067.1 | BD249687.1 | DL087234.1 | DL011897.1 | CS436018.1 |
| GM653066.1 | AX785163.1 | AY658927.1 | DL019717.1 | DL021753.1 | BD248999.1 | DL087202.1 | DL027843.1 | DD335675.1 |
| GM653034.1 | AX774557.1 | AY658895.1 | DL015154.1 | DL021721.1 | BD248734.1 | DL102095.1 | DL027811.1 | DD345316.1 |
| GM653002.1 | AX773479.1 | AY658863.1 | DL010536.1 | DL017387.1 | BD248119.1 | DL102063.1 | DL027779.1 | CS426818.1 |
| GM639008.1 | AX772848.1 | AY658831.1 | DL010504.1 | DL017355.1 | BD247031.1 | DL102031.1 | DL023871.1 | CS425078.1 |
| GM638976.1 | AX766193.1 | AY658799.1 | DL010472.1 | DL017323.1 | BD245085.1 | DL101914.1 | DL023839.1 | HV512254.1 |
| GM638944.1 | AX765944.1 | AY658767.1 | DL010440.1 | DL041374.1 | BD243790.1 | DL101882.1 | DL023807.1 | HV512222.1 |
| GM638912.1 | AX755020.1 | AY658735.1 | DL010408.1 | DL041310.1 | BD243426.1 | DL101786.1 | DL031537.1 | HV512190.1 |
| GM638880.1 | AX753230.1 | AY658703.1 | DL038782.1 | CS716078.1 | BD242444.1 | DL101754.1 | DL023660.1 | HV504789.1 |
| GM638848.1 | AX752641.1 | AY658671.1 | DL038750.1 | CS806166.1 | BD238537.1 | DL101722.1 | DL023628.1 | HV504757.1 |
| DL095195.1 | BD181403.1 | AY658639.1 | DL038718.1 | CS796316.1 | BD238170.1 | DL097212.1 | DL023596.1 | HV504725.1 |
| DL095163.1 | AX743997.1 | AY658607.1 | DL035094.1 | CS675435.1 | BD235727.1 | DL097180.1 | DL015922.1 | HV504693.1 |
| DL111837.1 | AX741995.1 | AY658575.1 | DL034808.1 | CS790266.1 | BD235442.1 | DL097148.1 | DL015890.1 | HV504661.1 |
| DL111805.1 | AX739854.1 | AY658543.1 | DJ339816.1 | CS792469.1 | AX752751.1 | DL095224.1 | DL015858.1 | HV504629.1 |
| DL111773.1 | AX722055.1 | AY658511.1 | DJ339784.1 | CS791939.1 | AX746420.1 | DL111834.1 | DL047600.1 | HV504597.1 |
| DL128888.1 | AX721692.1 | AY658479.1 | DJ339752.1 | DJ031565.1 | BD184756.1 | DL111802.1 | DL047568.1 | HV504980.1 |
| DL128853.1 | BD177894.1 | AY658447.1 | DJ339720.1 | DJ031174.1 | BD182887.1 | DL111770.1 | DL047536.1 | HI574357.1 |
| DL116420.1 | AX301005.1 | AY658415.1 | DJ339656.1 | DJ044921.1 | BD181490.1 | DL128850.1 | DL043618.1 | HI566248.1 |
| DL116388.1 | AX299846.1 | AY658383.1 | DJ339623.1 | DJ044795.1 | BD180850.1 | DL128786.1 | DL039673.1 | HI636993.1 |
| DL116356.1 | AX297513.1 | AY658351.1 | DJ339591.1 | DJ028161.1 | BD180762.1 | DL128753.1 | DL039641.1 | HI001470.1 |
| DL111603.1 | AX286674.1 | AY658319.1 | DJ339559.1 | DJ027955.1 | AX741745.1 | DL116417.1 | DL039609.1 | HI001370.1 |
| DL111571.1 | AX279944.1 | AY658287.1 | DJ327895.1 | DJ033724.1 | AY181088.1 | DL116385.1 | CS644234.1 | HI001337.1 |
| DL111539.1 | AX279653.1 | AY658255.1 | DJ122229.1 | CS691079.1 | DL095213.1 | DL116353.1 | DD460647.1 | HI003173.1 |
| DL111507.1 | AX278668.1 | AY658223.1 | DJ086035.1 | CS674184.1 | DL128742.1 | DL111664.1 | DD460137.1 | HI003113.1 |
| DL111475.1 | AX278273.1 | AY658191.1 | DJ082554.1 | DJ008393.1 | DL111621.1 | DL111632.1 | DD455442.1 | HI003061.1 |
| DL101693.1 | AX256400.1 | AY658159.1 | DJ086077.1 | DJ008361.1 | DL111589.1 | DL111600.1 | DD456663.1 | HI003025.1 |
| DL101661.1 | AX253573.1 | AY658127.1 | DJ081036.1 | DJ003338.1 | DL101551.1 | DL141430.1 | CS632068.1 | HI002924.1 |
| DL101629.1 | GM627122.1 | AY658095.1 | DJ069485.1 | DD495495.1 | DL096275.1 | DL114431.1 | CS642291.1 | HI001288.1 |
| DL097119.1 | GM660978.1 | AY658063.1 | DJ071395.1 | CS673624.1 | DL094229.1 | DL114399.1 | CS631277.1 | HI001192.1 |
| DL097087.1 | GM660946.1 | FZ421439.1 | DJ066771.1 | DD261104.1 | DL094197.1 | DL114367.1 | CS631198.1 | HI001128.1 |
| DL097055.1 | GM646783.1 | FZ418103.1 | DJ066375.1 | CS297037.1 | DL137217.1 | DL104576.1 | CS631134.1 | HI001094.1 |
| DL095131.1 | GM646751.1 | FZ411866.1 | DJ066343.1 | DD251647.1 | DL113534.1 | DL104544.1 | CS627833.1 | HI001038.1 |
| DL095099.1 | GM646719.1 | FZ420997.1 | DJ066310.1 | DD248661.1 | DL136968.1 | DL104512.1 | CS627181.1 | HI001004.1 |
| DL095035.1 | GM646687.1 | FZ417835.1 | DJ066276.1 | CS288156.1 | DL049695.1 | DL093928.1 | CS632200.1 | HI000957.1 |
| DL095003.1 | GM646655.1 | FZ420849.1 | DJ066242.1 | CS287614.1 | DL049663.1 | DL093896.1 | CS623691.1 | HI002828.1 |
| DL094971.1 | GM646623.1 | FZ417039.1 | DJ066041.1 | DD240738.1 | DL041712.1 | DL123344.1 | CS625511.1 | HI002790.1 |
| DL106657.1 | GM639820.1 | FZ416708.1 | DJ061432.1 | DD240579.1 | DL029125.1 | DL123312.1 | DD438025.1 | HI002730.1 |
| DL106561.1 | GM639788.1 | FZ419861.1 | DJ055278.1 | DD236737.1 | DL029093.1 | DL104506.1 | DD449081.1 | HI002645.1 |
| DL106529.1 | GM639756.1 | FZ419825.1 | DJ055195.1 | DD247136.1 | DL021779.1 | DL109154.1 | DD435966.1 | HI002611.1 |
| DL106497.1 | GM646583.1 | FZ419793.1 | DJ057589.1 | E41544.1 | DL021747.1 | DL109122.1 | CS620291.1 | HI002572.1 |
| DL128694.1 | GM646551.1 | FZ416527.1 | DJ060398.1 | E37143.1 | DL021715.1 | DL109090.1 | CS618910.1 | HI002507.1 |
| DL128594.1 | GM646519.1 | FW580294.1 | DJ060371.1 | E35590.1 | DJ391732.1 | DL104356.1 | CS616489.1 | HI004645.1 |
| DL128541.1 | GM646487.1 | FW582558.1 | DJ060189.1 | E33310.1 | DJ417452.1 | DL104324.1 | CS614401.1 | HI004517.1 |
| DL125064.1 | GM646455.1 | FW591960.1 | DJ053396.1 | E41202.1 | DJ380840.1 | DL098313.1 | CS613368.1 | HI002415.1 |
| DL125032.1 | GM646423.1 | FW591723.1 | DJ056340.1 | E06030.1 | DJ388714.1 | DL098249.1 | DJ050551.1 | HI002302.1 |
| DL125000.1 | GM639556.1 | FW590733.1 | DJ053105.1 | E05659.1 | DJ388671.1 | DL098217.1 | DJ049437.1 | HI000505.1 |

| | | | | | | | | |
|---|---|---|---|---|---|---|---|---|
| DL124968.1 | DL090673.1 | FW590104.1 | DJ053073.1 | E05201.1 | DJ340885.1 | DL098185.1 | DJ048826.1 | HI006490.1 |
| DL139747.1 | DL115546.1 | FW588675.1 | CS810629.1 | E04627.1 | DJ341030.1 | DL091960.1 | CS793885.1 | FV528427.1 |
| DL124921.1 | DL115514.1 | FW593452.1 | BD248299.1 | E04375.1 | DJ354951.1 | CS368384.1 | CS800047.1 | FV534506.1 |
| DL124889.1 | DL115482.1 | FW592795.1 | HC472261.1 | E03617.1 | DJ361566.1 | CS368000.1 | CS799089.1 | FV519159.1 |
| DL124793.1 | DL115450.1 | FW592636.1 | HC472197.1 | E03378.1 | DJ354251.1 | CS367616.1 | CS716824.1 | HC465616.1 |
| DL120768.1 | DL105698.1 | FW576796.1 | HC472165.1 | E02732.1 | DJ361224.1 | DL080671.1 | CS716757.1 | HC462341.1 |
| DL120736.1 | DL105666.1 | FW576687.1 | HC471965.1 | E02165.1 | DJ361191.1 | DL075893.1 | CS716077.1 | HC461783.1 |
| DL121888.1 | DL105634.1 | FW576589.1 | HC471933.1 | E01417.1 | DJ339839.1 | DL075253.1 | CS796315.1 | AY238516.1 |
| DL121856.1 | DL105602.1 | FW575886.1 | HC471901.1 | E01207.1 | DJ339807.1 | DL079075.1 | CS675434.1 | AF377315.1 |
| DL121824.1 | DL100797.1 | FW575362.1 | HC678760.1 | E00814.1 | DJ339743.1 | DL079043.1 | CS791362.1 | HC453698.1 |
| DL121792.1 | DL100765.1 | FW572722.1 | HC511398.1 | E00697.1 | DJ339647.1 | DL078927.1 | CS792426.1 | HC459799.1 |
| DL117529.1 | DL100733.1 | FW573696.1 | HC509304.1 | E00353.1 | DJ339582.1 | DL073073.1 | DJ031564.1 | U88619.1 |
| DL117465.1 | DL096287.1 | FW568969.1 | HC508166.1 | E00303.1 | DD360000.1 | DL013505.1 | DJ031173.1 | HC452129.1 |
| DL117433.1 | DL096255.1 | HI574374.1 | HC504183.1 | CQ986620.1 | DD357902.1 | DL013473.1 | DJ044920.1 | HC449496.1 |
| DL112754.1 | DL096223.1 | HI573725.1 | HC471038.1 | CQ986556.1 | DD357796.1 | DL013441.1 | DJ044793.1 | FU756122.1 |
| DL098316.1 | DL096191.1 | HI566218.1 | HC502095.1 | HW294131.1 | DD357585.1 | DL017978.1 | DJ028160.1 | FU758388.1 |
| DL098284.1 | DL096159.1 | HI553820.1 | HC500970.1 | HW294022.1 | DD355834.1 | DL017946.1 | DJ028126.1 | FU757779.1 |
| DL098252.1 | DL120179.1 | HI210929.1 | HC500236.1 | HW293049.1 | A18612.1 | DL017914.1 | DJ033723.1 | FU757622.1 |
| DL098220.1 | DL120115.1 | HI636971.1 | HC499856.1 | HW291194.1 | A06496.1 | DL017691.1 | CS279164.1 | FU757268.1 |
| DL098188.1 | DL120083.1 | HI001451.1 | FW300773.1 | HW291156.1 | A04097.1 | DL017659.1 | CS275480.1 | FU756567.1 |
| GM650088.1 | DL120051.1 | HI001395.1 | HC486495.1 | HW291091.1 | CS446151.1 | DL017627.1 | CS274779.1 | HC359090.1 |
| GM627283.1 | DL120019.1 | HI001355.1 | HC485144.1 | HW291059.1 | CS438911.1 | DL022297.1 | CS265609.1 | HC358396.1 |
| GM627251.1 | DL094241.1 | HI001321.1 | DM203135.1 | HW291027.1 | DD347404.1 | DL022265.1 | CS263125.1 | HC358208.1 |
| GM627219.1 | DL094209.1 | HI003204.1 | DM203075.1 | HW290900.1 | CS426770.1 | DL048457.1 | CS254303.1 | HC358077.1 |
| GM627187.1 | DL094177.1 | HI003142.1 | HB822351.1 | HW290868.1 | CS425049.1 | DL044744.1 | CS252528.1 | HC326687.1 |
| GM627123.1 | DL090462.1 | HI003046.1 | HB848663.1 | HW290326.1 | CS423009.1 | DL044680.1 | CS247429.1 | HC325112.1 |
| GM661107.1 | DL090430.1 | HI003005.1 | HB848044.1 | HW290100.1 | CS422525.1 | DL036568.1 | CS244153.1 | HC325064.1 |
| GM661075.1 | DL086297.1 | HI002943.1 | HB839644.1 | HW289986.1 | CS417190.1 | DL036536.1 | CS244233.1 | HB475834.1 |
| GM661043.1 | DL086265.1 | HI002906.1 | HB847601.1 | HW289003.1 | CS410928.1 | DL048443.1 | CS244201.1 | HB474747.1 |
| GM661011.1 | DL086201.1 | HI001269.1 | HB847293.1 | HW288927.1 | CS414818.1 | DL048411.1 | CS243763.1 | HB469127.1 |
| GM660979.1 | DL110316.1 | HI001216.1 | HB838879.1 | DI244379.1 | CS410294.1 | DL048379.1 | CS243154.1 | HB468759.1 |
| GM660947.1 | DL110284.1 | HI001166.1 | HB805066.1 | HW279630.1 | DD323520.1 | DL048347.1 | CS239680.1 | DM152630.1 |
| GM646784.1 | DL105319.1 | HI001079.1 | HB846755.1 | HW249438.1 | DD327010.1 | DL044269.1 | CS237710.1 | GN359705.1 |
| GM646752.1 | DL110167.1 | HI001021.1 | HB838375.1 | HW267896.1 | CS389296.1 | DL040093.1 | CS230952.1 | GN359557.1 |
| GM646720.1 | DL110103.1 | HI000989.1 | HB846318.1 | HW267680.1 | BD361422.1 | DL032036.1 | CS228036.1 | GN359493.1 |
| GM646688.1 | DL117865.1 | HI002883.1 | HB837981.1 | HW266181.1 | BD356502.1 | DL032004.1 | CS227319.1 | GN359425.1 |
| GM646656.1 | DL117833.1 | HI002845.1 | HB845944.1 | HW265961.1 | BD341799.1 | DL031972.1 | CS210668.1 | GN365387.1 |
| GM646624.1 | DL108151.1 | HI002809.1 | HB845526.1 | HW263017.1 | BD325687.1 | HI658802.1 | CS204202.1 | GN359256.1 |
| GM639821.1 | DL098779.1 | HI002753.1 | HB836984.1 | HV756238.1 | BD325592.1 | HI657179.1 | CS208082.1 | GN359224.1 |
| GM639789.1 | DL098747.1 | HI002713.1 | HB845964.1 | HV747491.1 | BD325481.1 | FW420949.1 | CS207908.1 | GN359128.1 |
| GM639757.1 | DL092737.1 | HI002664.1 | HB845142.1 | HV753625.1 | BD314342.1 | FW503005.1 | CS189615.1 | GN359096.1 |
| GM646584.1 | DL092705.1 | HI002629.1 | HB844849.1 | HV753517.1 | BD312815.1 | FW498003.1 | CS193202.1 | GN359066.1 |
| GM646520.1 | DL088962.1 | DL113584.1 | HB844410.1 | HV753405.1 | BD312575.1 | FW496006.1 | CS174644.1 | GN359034.1 |
| GM646488.1 | DL088930.1 | DL113552.1 | HB836436.1 | HV743215.1 | BD319274.1 | FW504058.1 | CS172462.1 | GN363078.1 |
| GM646456.1 | DL107961.1 | DL113520.1 | HB830436.1 | HV742537.1 | BD300558.1 | FW503568.1 | CS172316.1 | GN348284.1 |
| GM646424.1 | DL107929.1 | DL113456.1 | HB843095.1 | HV704344.1 | BD300251.1 | FW500535.1 | CS159790.1 | GN346601.1 |
| GM639525.1 | DL107897.1 | DL108559.1 | HB835513.1 | HV703913.1 | BD290038.1 | FW420465.1 | CS159346.1 | GN346568.1 |

| | | | | | | | | |
|---|---|---|---|---|---|---|---|---|
| GM626882.1 | DL088600.1 | DL103761.1 | HB824015.1 | HV744565.1 | BD293465.1 | FW420401.1 | CS155780.1 | GN346536.1 |
| GM626818.1 | DL098495.1 | DL103729.1 | HB842365.1 | HV702747.1 | BD291405.1 | FW503507.1 | CS150507.1 | GN116515.1 |
| GM653633.1 | DL092325.1 | DL016413.1 | HB850373.1 | HV701233.1 | BD299923.1 | HH998752.1 | CS144610.1 | GN116483.1 |
| GM653601.1 | DL092293.1 | DL016381.1 | HB850040.1 | HV701169.1 | BD299626.1 | HH997092.1 | CS144289.1 | GN116418.1 |
| GM660766.1 | DL092261.1 | DL016349.1 | HB841963.1 | HV708205.1 | BD299074.1 | HH996993.1 | CS119204.1 | GN116386.1 |
| GM660734.1 | DL092197.1 | DL016317.1 | HB841295.1 | HV708134.1 | BD295272.1 | HH996945.1 | CS119140.1 | DM075106.1 |
| GM653515.1 | DL092165.1 | DL016285.1 | HB840695.1 | HV701054.1 | BD294883.1 | HH998701.1 | CS119108.1 | DM073112.1 |
| GM653483.1 | DL088486.1 | DL016253.1 | HB828657.1 | HV700921.1 | BD289998.1 | HH998661.1 | CS119073.1 | DM064156.1 |
| GM653451.1 | DL088454.1 | DL028154.1 | HB648626.1 | HV695516.1 | BD274294.1 | HH998536.1 | CS119008.1 | DM077414.1 |
| GM653419.1 | DL088422.1 | DL011769.1 | HB645737.1 | HV695179.1 | BD272754.1 | HH996918.1 | CS118975.1 | GM715054.1 |
| GM653387.1 | DL088390.1 | DL011737.1 | HB645705.1 | HV695003.1 | BD271945.1 | HH996810.1 | CS118942.1 | GM654452.1 |
| GM646388.1 | DL027751.1 | DL011705.1 | HB645661.1 | HV694829.1 | BD271155.1 | HH996762.1 | CS118876.1 | GM647267.1 |
| GM646356.1 | DL023875.1 | DL011673.1 | HB855981.1 | HV694665.1 | DD231079.1 | HH980756.1 | CS118810.1 | HI002642.1 |
| GM646324.1 | DL023843.1 | DL011641.1 | DM178919.1 | HV038672.1 | DD227552.1 | HH980630.1 | CS118778.1 | HI002608.1 |
| GM646292.1 | DL023811.1 | DL011609.1 | DM170846.1 | HV038643.1 | DD227401.1 | HH980538.1 | CS118745.1 | HI002569.1 |
| GM646260.1 | DL047655.1 | DL048624.1 | DM170814.1 | HV038584.1 | DD227174.1 | HH980492.1 | CS118713.1 | HI002495.1 |
| GM646228.1 | DL031637.1 | DL048592.1 | DM178632.1 | HV182044.1 | DD226386.1 | HH987329.1 | CS118681.1 | HI004447.1 |
| GM639393.1 | DL031605.1 | DL048560.1 | GM044198.1 | HV228324.1 | AX766527.1 | HH980351.1 | CS118649.1 | HI002295.1 |
| GM639361.1 | DL031573.1 | DL048528.1 | GM043998.1 | HV029703.1 | AX766136.1 | HH980291.1 | CS118617.1 | HI000502.1 |
| GM639329.1 | DL031541.1 | DL048496.1 | GM755059.1 | HV029963.1 | AX763955.1 | HH980151.1 | CS118584.1 | HI179978.1 |
| GM639297.1 | DL023664.1 | DL048464.1 | GM712943.1 | HV035622.1 | AX746417.1 | HH980099.1 | CS118517.1 | HI202819.1 |
| GM639265.1 | DL023632.1 | DL044847.1 | GM652713.1 | HV035353.1 | BD184740.1 | HH979870.1 | CS118451.1 | HI177961.1 |
| GM626718.1 | DL023600.1 | DL044815.1 | GM652681.1 | FZ435955.1 | BD182874.1 | HH996698.1 | CS118419.1 | HI214579.1 |
| DL096883.1 | DL015958.1 | DL044783.1 | GM652649.1 | FZ427522.1 | BD181488.1 | HH996637.1 | CS118386.1 | HI214547.1 |
| DL096851.1 | DL015926.1 | DL044751.1 | GM652617.1 | FZ430302.1 | BD180848.1 | HH996582.1 | CS118352.1 | HI214512.1 |
| DL128076.1 | DL015894.1 | DL044719.1 | GM652585.1 | FZ430132.1 | BD180737.1 | HH998444.1 | CS118319.1 | HI179810.1 |
| DL124468.1 | DL015862.1 | DL044687.1 | GM645491.1 | FZ437138.1 | AY181086.1 | HH998393.1 | CS118285.1 | HI214449.1 |
| DL124436.1 | DL011244.1 | DL044535.1 | GM645459.1 | FZ422933.1 | AX717713.1 | HH998359.1 | CS118221.1 | HI214417.1 |
| DL124404.1 | DL011212.1 | DL044503.1 | GM645427.1 | FZ415473.1 | AX716776.1 | HH999988.1 | CS118187.1 | HI213810.1 |
| DL124372.1 | DL047636.1 | DL044471.1 | GM638534.1 | HI574359.1 | DD196863.1 | HH999934.1 | CS118154.1 | HI185487.1 |
| DL120283.1 | DL047604.1 | DL040455.1 | GM638502.1 | HI566252.1 | CQ944012.1 | HH979800.1 | CS118122.1 | HI212982.1 |
| DL120251.1 | DL047540.1 | DL040423.1 | GM638470.1 | HI588453.1 | CQ943980.1 | HH999903.1 | CS118090.1 | HI546334.1 |
| DL120219.1 | DL039677.1 | DL040391.1 | GM633739.1 | HI636995.1 | CQ943948.1 | HH999860.1 | CS118057.1 | HI546302.1 |
| DL115816.1 | DL039645.1 | DL040359.1 | GM633707.1 | HI636954.1 | CQ943916.1 | HH998326.1 | CS118023.1 | HI210980.1 |
| DL115784.1 | HV503771.1 | DL040327.1 | GM633675.1 | HI001472.1 | CQ943884.1 | HH998272.1 | CS117987.1 | HI210948.1 |
| DL115752.1 | HV503739.1 | DL036607.1 | GM625840.1 | HI001372.1 | CQ943852.1 | HH998235.1 | CS117955.1 | HI473083.1 |
| DL115720.1 | HV503707.1 | DL036575.1 | GM625808.1 | HI001339.1 | CQ924196.1 | HH998188.1 | CS111755.1 | HI472921.1 |
| DL115688.1 | HV455321.1 | DL036543.1 | GM624656.1 | HI003262.1 | CQ898658.1 | HH993692.1 | CS108627.1 | HI210924.1 |
| DL115656.1 | HV503176.1 | DL032308.1 | GM657768.1 | HI003122.1 | CQ898594.1 | HH986629.1 | CS106791.1 | HI580533.1 |
| DL111198.1 | HV503144.1 | DL032276.1 | GM622743.1 | HI003063.1 | CQ898562.1 | HH998129.1 | CS106391.1 | HI209398.1 |
| DL111166.1 | HV503112.1 | DL032244.1 | GM631660.1 | HI003027.1 | CQ898260.1 | HH979626.1 | CS104884.1 | HI072134.1 |
| DL106095.1 | HV503080.1 | DL032212.1 | GM631596.1 | HI002990.1 | CQ888090.1 | HH979584.1 | CS102967.1 | HC870090.1 |
| DL106063.1 | HV502780.1 | DL032180.1 | GM704214.1 | HI002926.1 | CQ871427.1 | HH931987.1 | CS102935.1 | FW310590.1 |
| DL106031.1 | HV502748.1 | DL032148.1 | GM704160.1 | HI001245.1 | CQ871391.1 | AX100671.1 | CS102871.1 | FW308819.1 |
| DL105967.1 | HV502716.1 | DL048450.1 | GM650147.1 | HI001194.1 | CQ869273.1 | FW394404.1 | CS102743.1 | FW334290.1 |
| DL105935.1 | HV502684.1 | DL048418.1 | GM650115.1 | HI001143.1 | CQ868861.1 | FW394295.1 | CS102679.1 | FW307702.1 |
| DL105903.1 | HV502652.1 | DL048386.1 | GM650083.1 | HI001096.1 | CQ859623.1 | FW394238.1 | CS102647.1 | FW332938.1 |

| | | | | | | | | |
|---|---|---|---|---|---|---|---|---|
| DL101194.1 | HV502620.1 | DL048354.1 | GM643114.1 | HI001040.1 | CQ859326.1 | FW394202.1 | CS102583.1 | FW332328.1 |
| DL101162.1 | HV502524.1 | DL048322.1 | GM643082.1 | HI001006.1 | CQ858724.1 | FW398046.1 | CS102551.1 | FW305531.1 |
| DL101130.1 | HV502492.1 | DL044436.1 | GM643050.1 | HI000960.1 | CQ857847.1 | FW396634.1 | CS102519.1 | FW310632.1 |
| DL096716.1 | HV502460.1 | DL044404.1 | GM643018.1 | HI002862.1 | CQ855759.1 | HC003086.1 | CS102487.1 | HC757701.1 |
| DL096684.1 | HV492707.1 | DL044372.1 | GM719202.1 | HI002830.1 | CQ854062.1 | HB865563.1 | HI553278.1 | HC754639.1 |
| DL096652.1 | HV505299.1 | DL044340.1 | GM656082.1 | HI002792.1 | CQ832288.1 | HB865045.1 | HI553206.1 | HC733494.1 |
| DL096620.1 | HV492452.1 | DL044308.1 | GM648961.1 | HI002732.1 | CQ828285.1 | HB864959.1 | HI553174.1 | HC289968.1 |
| DL096588.1 | HV453596.1 | DL044276.1 | GM648929.1 | HI002647.1 | CQ824768.1 | HB865005.1 | HI583958.1 | HC293044.1 |
| DL096556.1 | HV450066.1 | DL040100.1 | GM648897.1 | HI002613.1 | CQ821303.1 | HB864949.1 | HI565510.1 | HC289858.1 |
| DL094728.1 | HV455248.1 | DL032043.1 | GM646317.1 | HI002574.1 | BD003680.1 | HB864917.1 | HI003872.1 | HC207394.1 |
| DL094696.1 | HV451944.1 | DL032011.1 | GM639386.1 | HI004521.1 | BD002011.1 | HB864885.1 | HI002051.1 | HC202718.1 |
| DL094664.1 | HV453161.1 | DL031979.1 | GM639354.1 | HI002418.1 | AX348530.1 | HB864853.1 | HI001963.1 | HC201539.1 |
| DL090953.1 | HV452933.1 | DL031947.1 | GM626711.1 | HI002304.1 | AX347296.1 | HB864821.1 | HI000295.1 | HC199479.1 |
| DL090921.1 | HV452830.1 | DL028138.1 | GM660620.1 | HI000507.1 | AX347262.1 | HB864789.1 | HI000251.1 | HC197936.1 |
| DL118407.1 | HH714042.1 | DL028106.1 | GM646082.1 | HI006492.1 | AX347226.1 | HB864757.1 | HI000207.1 | DM462693.1 |
| DL118375.1 | HH961378.1 | DL028074.1 | GM646050.1 | HI180144.1 | AX347188.1 | HB859738.1 | HI000151.1 | HC196682.1 |
| DL118343.1 | HH961346.1 | DL028042.1 | DL124945.1 | HI202824.1 | AX347154.1 | HB866561.1 | HI000119.1 | HC195246.1 |
| DL113819.1 | HH961314.1 | DL028010.1 | DL128528.1 | HI214584.1 | AX344884.1 | HB866488.1 | HI137529.1 | HC193430.1 |
| DL113787.1 | HH961282.1 | DJ437109.1 | DL096456.1 | HI214552.1 | AX342437.1 | HC000345.1 | HI204485.1 | HC187671.1 |
| DL113747.1 | HH961250.1 | DJ436988.1 | DL115508.1 | HC318837.1 | AX339389.1 | HB999700.1 | HI470588.1 | HC089566.1 |
| DL108858.1 | HH975176.1 | DJ436914.1 | DL115476.1 | HC318688.1 | AX339192.1 | HB977104.1 | HI544472.1 | HC089534.1 |
| DL108794.1 | HH964410.1 | DJ436754.1 | DL115444.1 | BD453732.1 | AX328134.1 | HB976735.1 | HI559243.1 | HC089502.1 |
| DL108762.1 | HH964279.1 | DJ436507.1 | DL096313.1 | BD453700.1 | AX322859.1 | HB976538.1 | HI564737.1 | HC089470.1 |
| DL108730.1 | HH982230.1 | DJ431117.1 | DL096281.1 | BD453668.1 | AX268992.1 | DM197980.1 | HI564680.1 | HC089438.1 |
| DL108698.1 | HH974541.1 | DJ444747.1 | DL120173.1 | BD453636.1 | AF395807.1 | DM193325.1 | HC305040.1 | HC089405.1 |
| DL104052.1 | HH981858.1 | DJ438362.1 | DL094235.1 | BD453604.1 | AX279787.1 | DM203136.1 | HC305959.1 | HC089373.1 |
| DL039613.1 | HH998067.1 | DJ438356.1 | DL094203.1 | BD453554.1 | AX254828.1 | HB817099.1 | HC294206.1 | HC089341.1 |
| CS726887.1 | HH998006.1 | DJ438289.1 | DL094171.1 | BD453522.1 | AX242274.1 | HB840193.1 | HC291354.1 | HC089308.1 |
| CS724933.1 | HH997950.1 | DJ438208.1 | DL090488.1 | BD453490.1 | AX242210.1 | HB848046.1 | HC289379.1 | HC089276.1 |
| CS724546.1 | HH999653.1 | DJ427977.1 | DL086291.1 | BD453458.1 | AX242146.1 | HB847856.1 | HC289335.1 | HC088971.1 |
| CS724409.1 | HH997904.1 | DJ402580.1 | DL086259.1 | BD453426.1 | AX242050.1 | HB839674.1 | HC292888.1 | HC087497.1 |
| DJ353084.1 | HH997833.1 | DJ402546.1 | DL086227.1 | BD453394.1 | HW257269.1 | HB847603.1 | HC289005.1 | AY774929.1 |
| DJ357776.1 | HH997767.1 | DJ402298.1 | DL104571.1 | BD453249.1 | HW257173.1 | HB847295.1 | HC292756.1 | AY774882.1 |
| DJ357678.1 | HH999507.1 | DJ400837.1 | DL104539.1 | BD453217.1 | HW257045.1 | HB838881.1 | HC288890.1 | AY774821.1 |
| DJ357614.1 | HH999449.1 | DJ400805.1 | DL104507.1 | BD453133.1 | HW257013.1 | HB846776.1 | HC292458.1 | AY774763.1 |
| DJ357506.1 | HH999335.1 | DJ399818.1 | DL123339.1 | BD496302.1 | HW256949.1 | HB838400.1 | HC292218.1 | AY774705.1 |
| DJ362237.1 | HH997646.1 | DJ393086.1 | DL123307.1 | BD497446.1 | HW256917.1 | HB846369.1 | HC294960.1 | AY774651.1 |
| DJ344150.1 | HH997614.1 | DJ419649.1 | DL109149.1 | BD398647.1 | HW256853.1 | HB846070.1 | HC294780.1 | AY774527.1 |
| DJ361204.1 | HH995705.1 | DJ419610.1 | DL109117.1 | BD408092.1 | HW256821.1 | HB826597.1 | HC291774.1 | AY774487.1 |
| DJ354199.1 | HH997437.1 | DJ419536.1 | DL109085.1 | BD408021.1 | HW256789.1 | HB837983.1 | HC294552.1 | AY774261.1 |
| DJ360418.1 | HH997384.1 | DJ418062.1 | DL104383.1 | BD429585.1 | HW256757.1 | HB837572.1 | HC291018.1 | AY774205.1 |
| DJ340545.1 | HH999245.1 | DJ417437.1 | DL104351.1 | BD407578.1 | HW256725.1 | HB845946.1 | HC290833.1 | AY774074.1 |
| DJ339851.1 | HH999200.1 | DJ381038.1 | DL104319.1 | BD397229.1 | HW254456.1 | HB836986.1 | HC293421.1 | AY774010.1 |
| DJ339755.1 | HH999151.1 | DJ381006.1 | DL123156.1 | BD439077.1 | HW251350.1 | DL240794.1 | HC293301.1 | AF216703.1 |
| DJ339723.1 | HH998117.1 | DJ380974.1 | DL123124.1 | BD081731.1 | HW251028.1 | DL106854.1 | HC289863.1 | HC083535.1 |
| DJ339659.1 | HH999075.1 | DJ380942.1 | DL080584.1 | BD081548.1 | HW250642.1 | DL106822.1 | HC289680.1 | CS727259.1 |
| DJ339594.1 | HH999030.1 | DJ380910.1 | DL075888.1 | BD080724.1 | HW250353.1 | DL106790.1 | HC199622.1 | FB709016.1 |

| | | | | | | | | |
|---|---|---|---|---|---|---|---|---|
| DJ339562.1 | HH998964.1 | DJ380878.1 | DL078922.1 | BD080655.1 | HW249722.1 | DL091200.1 | DM464639.1 | FB715324.1 |
| DJ334569.1 | HH998921.1 | HC438419.1 | DL074239.1 | BD080158.1 | HW241210.1 | DL087232.1 | DM462705.1 | GM949525.1 |
| DJ334050.1 | HH997140.1 | HC358223.1 | DL073046.1 | BD080126.1 | HW247863.1 | DL087200.1 | HC195327.1 | GM009440.1 |
| DJ327444.1 | HH998916.1 | HC358093.1 | DL022292.1 | BD074967.1 | HW247812.1 | DL102093.1 | HC193919.1 | GM887850.1 |
| DJ327045.1 | HH998864.1 | HC358060.1 | DL022260.1 | BD070009.1 | HW240973.1 | DL102061.1 | HC193435.1 | GM869484.1 |
| DJ327013.1 | HH998806.1 | HC357538.1 | DL048310.1 | BD057309.1 | HW240909.1 | DL102029.1 | HC188550.1 | GM869164.1 |
| DJ122432.1 | HH998753.1 | HC356976.1 | DL040088.1 | BD016715.1 | HW240877.1 | DL101912.1 | HC089571.1 | GM773638.1 |
| DJ090085.1 | HH997038.1 | HB837698.1 | DL032031.1 | BD015987.1 | HW240813.1 | DL101880.1 | HC089539.1 | GM879304.1 |
| DJ086520.1 | HH996995.1 | HB845380.1 | DL031999.1 | BD015609.1 | HW240781.1 | DL101816.1 | HC089507.1 | GM842701.1 |
| DJ082765.1 | HH996946.1 | HB837119.1 | DL031967.1 | BD014214.1 | HW240749.1 | DL101784.1 | HC089475.1 | FB722585.1 |
| DJ082560.1 | HH998702.1 | HB813977.1 | DL028126.1 | BD014174.1 | HW240725.1 | DL101752.1 | HC089443.1 | GM706732.1 |
| DJ081968.1 | HH998662.1 | HB845188.1 | DL028094.1 | AX490791.1 | HW240693.1 | DL101720.1 | HC089410.1 | GM600750.1 |
| DJ086080.1 | HH998595.1 | HB844426.1 | DL028062.1 | E66929.1 | HW240661.1 | DL095222.1 | HC089378.1 | FB715262.1 |
| DJ081526.1 | HH998538.1 | HB844269.1 | DL028030.1 | AX468914.1 | HW240597.1 | DL111832.1 | HC089346.1 | FB706351.1 |
| DJ081373.1 | HH996919.1 | HB836529.1 | DL027998.1 | AX467748.1 | HW239412.1 | DL111800.1 | HC089281.1 | GM061214.1 |
| DJ069488.1 | HH996763.1 | HB843789.1 | DL044263.1 | A22048.1 | HW239190.1 | DL128784.1 | HC089249.1 | DL258286.1 |
| DJ066774.1 | HH977582.1 | HB836300.1 | DL044231.1 | AX458611.1 | HW238938.1 | DL116415.1 | HC087515.1 | DL233426.1 |
| DJ066414.1 | HH980633.1 | HB843111.1 | DL044199.1 | AX454047.1 | HW238600.1 | DL116383.1 | AY774984.1 | DL019492.1 |
| DJ066378.1 | HH980539.1 | HB835586.1 | DL044167.1 | A35243.1 | HW238540.1 | DL116351.1 | AY774895.1 | HW096579.1 |
| DJ066346.1 | HH980493.1 | HB819127.1 | DL048240.1 | A34181.1 | HW238200.1 | DL100386.1 | AY774833.1 | HW105487.1 |
| DJ066279.1 | HH987330.1 | HB835172.1 | DL048208.1 | AX441501.1 | HW242971.1 | DL100354.1 | AY774536.1 | HW072852.1 |
| DD349663.1 | HH980352.1 | HB842570.1 | DL048176.1 | AX430185.1 | HW242932.1 | DL124135.1 | DM000226.1 | HV778346.1 |
| DD348426.1 | HH980292.1 | HB806721.1 | DL047857.1 | AX428410.1 | HW242868.1 | DL124103.1 | DM005067.1 | HV777431.1 |
| DD331155.1 | HH980217.1 | HB842028.1 | DL040026.1 | AX427068.1 | HW242804.1 | DL119483.1 | DM004985.1 | HV763963.1 |
| CS425086.1 | HH980152.1 | HB841727.1 | DL039994.1 | AX418267.1 | HW238013.1 | DL119451.1 | DM003486.1 | HV766820.1 |
| CS422900.1 | HH980100.1 | HB806434.1 | DL043671.1 | AX406731.1 | HW237434.1 | DL119419.1 | GM979905.1 | HV750384.1 |
| CS417763.1 | HH979875.1 | HB840860.1 | DJ491580.1 | AX399394.1 | HW242327.1 | DL123666.1 | GM992961.1 | HV752894.1 |
| CS421335.1 | HH996699.1 | HB833626.1 | DJ446847.1 | AX397671.1 | HW242295.1 | DL123634.1 | GN007447.1 | HV758630.1 |
| CS415536.1 | HH996638.1 | HB650363.1 | DJ432658.1 | AX393437.1 | HW242263.1 | DL123602.1 | GM992438.1 | HV694857.1 |
| CS410557.1 | HH996583.1 | HB648691.1 | DJ445563.1 | AX384563.1 | HW241791.1 | DL123570.1 | GM991939.1 | HV694789.1 |
| CS414836.1 | HH996542.1 | HB645745.1 | DJ445137.1 | AX383805.1 | HW241743.1 | DL119380.1 | GM976372.1 | HV694754.1 |
| DD320524.1 | HH998396.1 | HB645713.1 | DJ444552.1 | AX380800.1 | HW241547.1 | DL119348.1 | GM989762.1 | HV693311.1 |
| DD327190.1 | HH998360.1 | HB645681.1 | DJ433811.1 | HW350995.1 | HW243062.1 | DL114824.1 | GM969685.1 | HV698243.1 |
| DD327023.1 | HH999989.1 | HB855124.1 | DJ438344.1 | HW350963.1 | HW243030.1 | DL114792.1 | GM983620.1 | HV697824.1 |
| DD326890.1 | HH999935.1 | HB850754.1 | DJ438309.1 | HW340513.1 | HW241370.1 | DL114760.1 | GM984656.1 | HV689280.1 |
| DD329896.1 | HH993943.1 | HB850609.1 | DJ438277.1 | HW350887.1 | HW163495.1 | DL114728.1 | FB740442.1 | HV688877.1 |
| DD329692.1 | HH979811.1 | DM179540.1 | DJ402568.1 | HW340463.1 | HW163444.1 | DL114696.1 | GM596699.1 | HV600956.1 |
| DD321797.1 | HH999904.1 | DM179150.1 | DJ402532.1 | HW350664.1 | HW163317.1 | DL114664.1 | GM970099.1 | HV570613.1 |
| CS398016.1 | HH999861.1 | DM170822.1 | DJ400825.1 | HW340080.1 | HW161437.1 | DL141464.1 | GM009567.1 | HV565830.1 |
| CS389274.1 | HH999818.1 | DM188012.1 | DJ400793.1 | HW350296.1 | HW160997.1 | DL123466.1 | DL464792.1 | HV572453.1 |
| CS389223.1 | DD405585.1 | DM187724.1 | DJ398709.1 | HW350166.1 | HW160636.1 | DL123434.1 | DL464539.1 | HV571056.1 |
| CS389186.1 | DD405553.1 | DM187603.1 | DJ391329.1 | HW339644.1 | HW065213.1 | DL123402.1 | DL476668.1 | HV552994.1 |
| CS401601.1 | DD405489.1 | HB491790.1 | CS498826.1 | HW363663.1 | HW065180.1 | DL123370.1 | GM635129.1 | HV552736.1 |
| CS406187.1 | DD405457.1 | HB489513.1 | CS496119.1 | HW363632.1 | HW064887.1 | DL119116.1 | GM635097.1 | HV555165.1 |
| CS403492.1 | DD405425.1 | HB488764.1 | DD418603.1 | HW153777.1 | HW061091.1 | DL119084.1 | GM634966.1 | HV550159.1 |
| CS402168.1 | DD402019.1 | HB488677.1 | DD410119.1 | HW153378.1 | HW055435.1 | DL119052.1 | GM669842.1 | AY659384.1 |
| CS382094.1 | DD401987.1 | DM164024.1 | CS489135.1 | HW152024.1 | HW060947.1 | DL119020.1 | GM656289.1 | AY659352.1 |

| | | | | | | | | |
|---|---|---|---|---|---|---|---|---|
| CS380058.1 | DD401926.1 | GM752447.1 | CS486438.1 | HW126448.1 | HW056507.1 | DL114624.1 | GM656257.1 | AY659320.1 |
| CS382609.1 | DD401894.1 | DL480743.1 | CS482992.1 | HW125561.1 | HW056407.1 | DL114592.1 | GM656193.1 | AY659288.1 |
| CS376543.1 | DD401862.1 | DL467582.1 | CS482957.1 | HW125384.1 | HW064743.1 | DL114560.1 | GM656161.1 | AY659256.1 |
| DD308732.1 | DD401830.1 | GM712952.1 | CS482919.1 | HW144360.1 | HW064670.1 | DL114528.1 | GM717406.1 | AY659224.1 |
| DD291445.1 | DD401622.1 | GM652753.1 | CS482875.1 | HW124858.1 | HW062572.1 | DL114496.1 | GM648988.1 | AY659192.1 |
| DD290407.1 | DD401590.1 | GM652721.1 | CS482405.1 | HW122418.1 | HW062414.1 | DL114464.1 | GM648956.1 | AY659160.1 |
| BD450793.1 | DD401558.1 | GM652689.1 | DD401427.1 | HW117990.1 | HW060583.1 | DL104897.1 | GM634549.1 | AY659128.1 |
| BD408103.1 | DD057935.1 | GM652657.1 | DD401395.1 | HV951958.1 | HW056240.1 | DL104833.1 | GM634517.1 | AY659096.1 |
| BD449818.1 | DD057903.1 | GM652625.1 | DD405210.1 | HV939875.1 | HW062357.1 | DL104801.1 | GM634485.1 | AY659064.1 |
| BD397413.1 | DD057871.1 | GM652593.1 | DD405178.1 | HV945599.1 | HW062325.1 | DL011664.1 | GM629680.1 | AY659032.1 |
| BD429568.1 | DD057843.1 | GM645499.1 | DD405146.1 | HV925512.1 | HW056065.1 | DL011632.1 | GM647235.1 | AY659000.1 |
| BD407497.1 | DD053299.1 | GM645467.1 | DD405114.1 | HV819453.1 | HW060296.1 | DL011600.1 | GM647203.1 | AY658968.1 |
| BD429343.1 | DD052283.1 | GM645435.1 | DD408524.1 | HV932709.1 | HW055646.1 | DL048455.1 | GM714617.1 | AY658936.1 |
| BD397103.1 | DD052225.1 | GM638606.1 | DD406761.1 | HV932676.1 | HW042080.1 | DL044742.1 | GM661510.1 | AY658904.1 |
| BD396343.1 | DD051342.1 | GM638574.1 | DD402425.1 | HV932644.1 | HW042048.1 | DL044710.1 | GM647171.1 | AY658872.1 |
| BD406401.1 | DD058541.1 | GM638510.1 | HW041949.1 | HV932394.1 | HW046535.1 | DL044494.1 | GM647014.1 | AY658840.1 |
| BD437692.1 | DD041904.1 | GM638478.1 | HW041917.1 | HV344584.1 | HW043500.1 | DL044267.1 | GM640144.1 | AY658808.1 |
| BD395083.1 | DD042874.1 | GM638446.1 | HW041819.1 | HV339946.1 | HW042004.1 | DL040091.1 | FB676587.1 | AY658776.1 |
| BD426880.1 | DD039013.1 | GM633747.1 | HW049403.1 | HV344470.1 | HW041972.1 | DL032034.1 | FB676555.1 | AY658744.1 |
| BD445720.1 | DD037265.1 | GM633715.1 | HW049371.1 | HV342121.1 | HW041940.1 | DL032002.1 | FB676491.1 | AY658712.1 |
| BD453345.1 | DD032571.1 | GM633683.1 | HW049340.1 | HV321733.1 | HW041908.1 | DL031970.1 | FB705619.1 | AY658680.1 |
| BD453316.1 | DD032257.1 | GM625816.1 | HW049308.1 | HV323290.1 | HW041846.1 | DL028129.1 | FB705918.1 | AY658648.1 |
| BD453284.1 | DD030667.1 | GM625784.1 | HW049276.1 | HV119958.1 | HW049488.1 | DL028097.1 | FB701803.1 | HH961299.1 |
| BD391411.1 | DD025687.1 | GM625752.1 | HW049244.1 | HV187363.1 | HW049394.1 | DL028065.1 | FB700929.1 | HH961267.1 |
| BD375870.1 | DD023884.1 | GM624728.1 | HW046206.1 | HV195747.1 | HW041817.1 | DL028033.1 | FB654395.1 | HH961235.1 |
| BD374742.1 | DD029391.1 | GM624664.1 | HW041815.1 | HV208795.1 | HW049331.1 | DL028001.1 | FB657096.1 | HH975402.1 |
| BD350078.1 | DD028674.1 | GM624632.1 | HV592440.1 | HV208587.1 | HW049299.1 | DL027969.1 | FB573255.1 | HH975098.1 |
| BD356417.1 | BD412641.1 | GM633646.1 | HV592408.1 | DL028087.1 | HW049267.1 | DL044234.1 | DL199772.1 | HH974918.1 |
| BD353557.1 | BD454383.1 | GM633614.1 | HV585558.1 | DL028055.1 | HW049198.1 | DL044202.1 | DL199708.1 | HH998095.1 |
| BD359033.1 | BD443868.1 | GM633582.1 | HV570626.1 | DL028023.1 | HW043301.1 | DL044170.1 | DL184308.1 | HH998034.1 |
| BD342469.1 | BD453893.1 | GM633550.1 | HV566134.1 | DL027991.1 | HW041806.1 | DL048243.1 | DL193813.1 | HH997988.1 |
| BD341826.1 | BD453861.1 | GM633518.1 | HV572325.1 | DL027866.1 | HW041774.1 | DL048211.1 | DL193781.1 | HH999673.1 |
| DL114793.1 | BD453829.1 | GM633486.1 | HV560184.1 | DL027834.1 | HW054947.1 | DL048179.1 | DL193716.1 | HH999551.1 |
| DL114761.1 | BD453797.1 | GM625580.1 | FW570862.1 | DL027802.1 | HW041553.1 | DL048147.1 | FB571346.1 | HH997863.1 |
| DL114729.1 | BD443408.1 | GM638395.1 | HV554544.1 | DL027770.1 | HW047144.1 | CS811268.1 | FB571290.1 | HH997797.1 |
| DL114697.1 | BD453741.1 | GM638363.1 | HV342079.1 | CS486427.1 | HW054059.1 | DJ021068.1 | FB573117.1 | HH997752.1 |
| DL114665.1 | BD453709.1 | GM624329.1 | HV341786.1 | DD401420.1 | HW054018.1 | DJ016323.1 | CS696182.1 | HH999477.1 |
| DL127334.1 | BD453677.1 | GM624297.1 | HV322671.1 | DD401380.1 | HW053889.1 | CS803360.1 | CS696150.1 | HH999431.1 |
| DL141535.1 | BD453645.1 | GM624265.1 | HV321735.1 | DD405203.1 | HW053776.1 | DJ052633.1 | CS696054.1 | HH999391.1 |
| DL141465.1 | BD453613.1 | GM659900.1 | HV187366.1 | DD405171.1 | HW046739.1 | DJ046980.1 | CS695989.1 | HH997674.1 |
| DL123467.1 | BD453576.1 | GM659868.1 | HV195754.1 | DD405139.1 | HW044192.1 | DJ050560.1 | CS695957.1 | HH997631.1 |
| DL123435.1 | BD493232.1 | GM659836.1 | HV208797.1 | DD405107.1 | HW029710.1 | DJ050207.1 | CS695829.1 | HH997574.1 |
| DL123403.1 | BD453563.1 | GM659772.1 | HV208589.1 | DD406597.1 | HV985978.1 | DJ049441.1 | CS695797.1 | HH997499.1 |
| DL123371.1 | BD453531.1 | GM659740.1 | HV217973.1 | DD401915.1 | HV985946.1 | DJ048830.1 | CS695765.1 | HH997360.1 |
| DL119117.1 | BD453499.1 | GM712212.1 | HV302962.1 | DD401883.1 | HV985914.1 | DJ048798.1 | DL099578.1 | HH999225.1 |
| DL119085.1 | BD453467.1 | GM659704.1 | HV308609.1 | DD401851.1 | HV985869.1 | CS721579.1 | DL099546.1 | HH999181.1 |
| DL119053.1 | BD453435.1 | GM649308.1 | FZ435986.1 | DD401819.1 | HV974802.1 | CS800051.1 | DL137229.1 | HH999136.1 |

| | | | | | | | | |
|---|---|---|---|---|---|---|---|---|
| DL119021.1 | BD453403.1 | GM649310.1 | FZ427521.1 | DD401547.1 | HV985429.1 | CS717360.1 | DL113535.1 | HH999098.1 |
| DL114625.1 | BD453258.1 | GM649254.1 | FZ430299.1 | DD401515.1 | HV445661.1 | CS716762.1 | DL041544.1 | HH999056.1 |
| DL114593.1 | BD453226.1 | GM649222.1 | FZ437137.1 | DD401483.1 | HV449247.1 | GM755058.1 | DL029126.1 | HH998996.1 |
| DL114561.1 | BD453194.1 | GM649190.1 | FZ422906.1 | CS476135.1 | HV448646.1 | GM834968.1 | DL021780.1 | HH998948.1 |
| DL114529.1 | BD411234.1 | GM642581.1 | FZ421510.1 | CS468139.1 | HV448376.1 | GM712942.1 | DL021748.1 | HH997222.1 |
| DL114497.1 | BD442622.1 | GM642549.1 | FZ421036.1 | CS287607.1 | HV437000.1 | GM652744.1 | DL021716.1 | HH997157.1 |
| DL114465.1 | BD399403.1 | GM642467.1 | FZ417511.1 | DD236708.1 | HV436933.1 | GM652712.1 | DL035086.1 | HH978149.1 |
| DL104898.1 | BD398784.1 | CS182195.1 | FZ419843.1 | DD246575.1 | HV436897.1 | GM652680.1 | DL035054.1 | HH998895.1 |
| DL104866.1 | AX028815.1 | CS174655.1 | FZ419809.1 | E41537.1 | HV436865.1 | GM652648.1 | DL042672.1 | HH998842.1 |
| DL104834.1 | AX028515.1 | CS172833.1 | FW590749.1 | E41743.1 | HV444635.1 | GM652616.1 | DL030675.1 | HH998791.1 |
| DL104802.1 | AX027714.1 | CS161306.1 | FW590717.1 | E49740.1 | HV444351.1 | GM652584.1 | DL026642.1 | HH998732.1 |
| DL095879.1 | AX023612.1 | CS159800.1 | FW589141.1 | E40779.1 | HV444117.1 | GM625186.1 | DL019463.1 | HH997077.1 |
| DL114430.1 | AX023579.1 | CS159570.1 | HI653035.1 | E36563.1 | HV444014.1 | GM625154.1 | DL019431.1 | HH996969.1 |
| DL114398.1 | AX021721.1 | CS155614.1 | HI651013.1 | E33812.1 | HV443862.1 | GM624032.1 | DL019399.1 | HH981094.1 |
| DL114366.1 | AX015788.1 | CS148537.1 | HI646959.1 | E32412.1 | HV350092.1 | GM624000.1 | CS546680.1 | HH998635.1 |
| DL104575.1 | AX002865.1 | CS144916.1 | AY145514.1 | E05895.1 | HV443183.1 | GM623968.1 | CS561351.1 | HH998572.1 |
| DL104543.1 | AX011503.1 | CS144347.1 | HH804678.1 | E04548.1 | HV443094.1 | GM658911.1 | CS559350.1 | HH996936.1 |
| DL104511.1 | AX011471.1 | CS142068.1 | HH797415.1 | E03592.1 | HV437558.1 | GM658879.1 | DD420233.1 | HH996904.1 |
| DL038453.1 | AX011439.1 | CS141574.1 | HH794937.1 | E03101.1 | HV437503.1 | GM658847.1 | DD431306.1 | HH996783.1 |
| DL038421.1 | AX011407.1 | CS141526.1 | HH794705.1 | E02964.1 | HV437316.1 | GM658815.1 | DD421989.1 | HH996748.1 |
| DL038389.1 | AX010936.1 | CS141494.1 | HH774421.1 | E02693.1 | HV349092.1 | GM637346.1 | DD431733.1 | HH980614.1 |
| DL038357.1 | AX010503.1 | CS141121.1 | HH758941.1 | E02431.1 | HV344165.1 | GM637314.1 | CS540033.1 | HH980510.1 |
| DL038325.1 | AX010326.1 | CS140533.1 | HI203406.1 | E01823.1 | HV344126.1 | GM637282.1 | CS537816.1 | HH980442.1 |
| DL013162.1 | AX009253.1 | CS133449.1 | HI179267.1 | E01657.1 | HV344088.1 | GM637250.1 | CS501452.1 | HH980369.1 |
| DL013130.1 | AX008207.1 | CS132438.1 | HI553277.1 | E01485.1 | HV344050.1 | GM623649.1 | CS498445.1 | HV503342.1 |
| DL013098.1 | AX006899.1 | CS123548.1 | HI583957.1 | E00953.1 | HV344011.1 | GM623617.1 | CS495890.1 | HV503310.1 |
| DL013066.1 | AX006190.1 | CS122492.1 | HI003859.1 | E00781.1 | HV343975.1 | GM623585.1 | DD418598.1 | HV503278.1 |
| DL013034.1 | AX003727.1 | CS106079.1 | HI002050.1 | DD088121.1 | HV343937.1 | GM623553.1 | DD412403.1 | HV503246.1 |
| DL013002.1 | AX003037.1 | CS181448.1 | HI001959.1 | DD142885.1 | HV343898.1 | GM637137.1 | DD418146.1 | HV503214.1 |
| DL033737.1 | AF191640.1 | CS179736.1 | HI000293.1 | DD142708.1 | HV343860.1 | GM637105.1 | DD411492.1 | HV503191.1 |
| DL042171.1 | A57349.1 | CS179612.1 | HI000250.1 | DD154002.1 | HV343822.1 | GM637073.1 | DD417791.1 | HV503159.1 |
| DL042139.1 | A38672.1 | CS180020.1 | HI000206.1 | DD158385.1 | HV343783.1 | GM632671.1 | CS492168.1 | HV503127.1 |
| DL042107.1 | A35736.1 | DD228420.1 | HI000150.1 | DD158376.1 | HV343745.1 | GM632639.1 | CS492951.1 | HV503095.1 |
| DL029528.1 | A35533.1 | DD227770.1 | HI000118.1 | AX711901.1 | HV343706.1 | GM654743.1 | CS490483.1 | HV503063.1 |
| DL029496.1 | A29491.1 | DD227384.1 | HI137490.1 | AX708723.1 | HV343668.1 | GM640626.1 | CS486429.1 | HV503031.1 |
| DL029464.1 | A29433.1 | DD226684.1 | HI204484.1 | AX703499.1 | HV343630.1 | GM640562.1 | CS352390.1 | HV502999.1 |
| DL046130.1 | A35195.1 | DD224656.1 | HI552630.1 | AX657127.1 | HV343591.1 | GM647241.1 | CS283952.1 | HV502935.1 |
| DL049705.1 | A33977.1 | DD231572.1 | HI470587.1 | AX657095.1 | HV343562.1 | GM647209.1 | CS283074.1 | HV502871.1 |
| DL023170.1 | A30383.1 | DD231476.1 | HI538383.1 | AX642243.1 | HV343524.1 | GM870220.1 | CS276850.1 | HV502839.1 |
| DL023138.1 | A29917.1 | DD231408.1 | HI564736.1 | AX642075.1 | HV343486.1 | GM627575.1 | CS266078.1 | HV502795.1 |
| DL023106.1 | A23353.1 | CS273081.1 | HI564237.1 | BD171818.1 | HV340908.1 | GM627543.1 | CS265601.1 | HV502763.1 |
| DL023074.1 | A31928.1 | DD214713.1 | HI564113.1 | AX608836.1 | HV338414.1 | GM661484.1 | CS260263.1 | HV502731.1 |
| DL023042.1 | A26318.1 | DD212845.1 | HI583100.1 | AX601774.1 | HV347949.1 | GM661452.1 | CS244229.1 | HV502699.1 |
| DL023010.1 | A26690.1 | DD214018.1 | HI563637.1 | AX601371.1 | HV347830.1 | GM661420.1 | CS244197.1 | HV502667.1 |
| DL022978.1 | A16265.1 | DD216630.1 | HI563605.1 | AX601339.1 | HV347792.1 | GM661388.1 | CS244709.1 | HV502635.1 |
| DL010750.1 | A20101.1 | DD213908.1 | HI563570.1 | AX600170.1 | HV347726.1 | GM654330.1 | CS243148.1 | HV502603.1 |
| DL010718.1 | DM459958.1 | DD213876.1 | HI508523.1 | AX587890.1 | HV347687.1 | GM654298.1 | CS240015.1 | HV502571.1 |

| | | | | | | | | |
|---|---|---|---|---|---|---|---|---|
| DL010686.1 | DM381918.1 | DD213844.1 | HI544255.1 | AX587796.1 | HV347649.1 | GM654266.1 | CS227309.1 | HV502539.1 |
| DL042968.1 | DM381317.1 | DD213812.1 | HI508213.1 | BD160935.1 | HV347611.1 | GM647177.1 | CS223503.1 | HV502507.1 |
| DL042936.1 | HC054857.1 | DD216445.1 | HI000027.1 | AX145713.1 | HV340215.1 | GM647145.1 | CS208078.1 | HV502475.1 |
| DL042904.1 | HC051931.1 | DD213795.1 | HI003666.1 | AX145649.1 | HV344994.1 | GM647113.1 | CS207904.1 | HV502443.1 |
| DL042872.1 | HC050686.1 | DD213763.1 | HI003541.1 | AX145617.1 | HV347394.1 | GM647081.1 | CS207865.1 | HV492764.1 |
| DL038991.1 | HC050264.1 | DD213731.1 | HI003491.1 | AX145585.1 | HV344929.1 | GM647020.1 | CS189611.1 | HV492725.1 |
| DL038959.1 | HC045495.1 | DD213667.1 | HI001758.1 | AX145553.1 | HV344890.1 | GM640054.1 | CS174640.1 | HV492687.1 |
| DL038927.1 | HC045463.1 | DD213635.1 | HI001724.1 | AX145521.1 | HV344852.1 | GM661299.1 | CS172457.1 | HV505235.1 |
| DL027138.1 | HC045431.1 | DD213603.1 | HI544123.1 | AX145489.1 | HV344814.1 | FB654407.1 | BD295273.1 | HV507746.1 |
| DL027106.1 | HC045399.1 | DD213585.1 | HI465293.1 | AX145457.1 | HV344775.1 | FB654863.1 | BD294884.1 | HV505011.1 |
| DL027074.1 | HC045367.1 | DD213553.1 | HI001641.1 | AX145425.1 | HV340189.1 | FB583240.1 | BD283816.1 | HV452329.1 |
| DL027010.1 | HC045335.1 | DD220650.1 | HI001600.1 | AX145392.1 | HV340157.1 | FB573850.1 | BD289999.1 | HV453669.1 |
| DL026978.1 | HC045271.1 | DD216033.1 | HI001557.1 | AX145360.1 | HV340125.1 | FB573758.1 | BD277392.1 | HV450049.1 |
| DL030905.1 | HC047451.1 | DD216001.1 | HI001504.1 | AX145328.1 | HV340093.1 | DL193905.1 | BD274295.1 | HV449925.1 |
| DL030738.1 | HC047419.1 | DD215969.1 | HI003379.1 | AX145296.1 | HV340061.1 | DL196187.1 | BD272755.1 | HV449757.1 |
| DD414128.1 | HC047387.1 | DD215923.1 | HI003341.1 | AX145264.1 | HV340029.1 | DL193819.1 | BD271947.1 | HV453041.1 |
| CS490612.1 | HC047355.1 | DD215891.1 | HI003302.1 | AX145232.1 | HV339997.1 | DL193787.1 | AX028784.1 | FW576697.1 |
| CS489139.1 | HC047323.1 | DD215859.1 | HC201574.1 | AX145200.1 | HV346981.1 | DL193755.1 | AX027734.1 | FW575893.1 |
| CS482997.1 | HC047291.1 | DD215795.1 | HC199616.1 | AX145168.1 | HV344730.1 | DL183796.1 | AX023633.1 | FW575460.1 |
| CS482961.1 | HC047259.1 | DD215763.1 | HC197948.1 | AX145136.1 | HV344691.1 | DL183386.1 | AX023599.1 | FW569376.1 |
| CS482925.1 | HC047035.1 | DD217982.1 | DM464638.1 | AX145104.1 | HV344653.1 | FB571236.1 | AX021123.1 | FW572729.1 |
| CS482879.1 | HC047003.1 | DD212312.1 | DM462704.1 | AX145072.1 | HV344614.1 | CS696188.1 | AX019527.1 | FW574942.1 |
| DD401399.1 | HC046971.1 | DD192508.1 | HC195265.1 | AX145040.1 | HV344576.1 | CS696156.1 | AX014764.1 | FW568977.1 |
| DD405214.1 | HC046939.1 | DD210004.1 | HC193434.1 | AX145008.1 | HV339939.1 | CS696124.1 | AX010922.1 | FW574733.1 |
| DD405182.1 | HC046907.1 | DD208690.1 | HC187557.1 | AX144976.1 | HV339875.1 | CS696092.1 | AX008549.1 | FW572656.1 |
| DD405150.1 | HC046879.1 | DD187262.1 | HC089570.1 | AX144944.1 | HV342434.1 | CS696060.1 | AX003260.1 | FW572607.1 |
| DD405118.1 | HC046815.1 | DD206888.1 | HC089538.1 | AX144911.1 | HV344539.1 | CS696028.1 | AX002981.1 | FW573490.1 |
| DD408548.1 | HA638556.1 | DD206856.1 | HC089506.1 | AX144879.1 | HV344500.1 | CS695995.1 | U49849.1 | FW572263.1 |
| DD406784.1 | HA637706.1 | CS249478.1 | HC089474.1 | AX144847.1 | HV344462.1 | CS695963.1 | A41838.1 | FW568769.1 |
| DD402429.1 | DM102626.1 | AX642218.1 | HC089442.1 | AX144815.1 | HV344424.1 | CS695867.1 | A25420.1 | FW573277.1 |
| DD402397.1 | DM109921.1 | AX601387.1 | HC089409.1 | AX144783.1 | HV344383.1 | CS695835.1 | A35722.1 | FW570982.1 |
| DD402365.1 | DM094841.1 | AX601355.1 | HC089377.1 | AX144751.1 | HV335693.1 | CS695803.1 | A35695.1 | FW572988.1 |
| DD402333.1 | DM107423.1 | AX598767.1 | HC089345.1 | AX144719.1 | HV342151.1 | CS695739.1 | A34511.1 | FW571784.1 |
| DD402301.1 | GN368311.1 | AX598185.1 | HC089312.1 | HV555596.1 | HV342113.1 | CS695707.1 | A32419.1 | FW570777.1 |
| DD402269.1 | GN360151.1 | AX593520.1 | HC089280.1 | HV553097.1 | HV342067.1 | CS695547.1 | A30352.1 | HI967953.1 |
| DD402237.1 | GN360087.1 | AX593326.1 | HC089248.1 | HV552738.1 | HV342029.1 | CS695483.1 | A04442.1 | HI967440.1 |
| DD402205.1 | GN360046.1 | AX587843.1 | HC087513.1 | HV550382.1 | HH797007.1 | CS695419.1 | A22357.1 | HI964509.1 |
| DD405617.1 | GN360014.1 | AX592802.1 | AY774983.1 | AY659386.1 | HH796604.1 | DL176478.1 | A21952.1 | HI979681.1 |
| HC294840.1 | GN359982.1 | AX589666.1 | AY774933.1 | AY659354.1 | HH796424.1 | DL176438.1 | A19971.1 | HI979306.1 |
| HC294600.1 | GN359950.1 | BD161534.1 | AY774894.1 | AY659322.1 | HH794687.1 | DL176339.1 | A15644.1 | HI990093.1 |
| HC298739.1 | GN359809.1 | AX587904.1 | AY774826.1 | AY659290.1 | HH793041.1 | DL122687.1 | HW155698.1 | HI989492.1 |
| HC291078.1 | GN359777.1 | AX587809.1 | AY774768.1 | AY659258.1 | HH792785.1 | DL122655.1 | HW144845.1 | HI989108.1 |
| HC290893.1 | GN359745.1 | AX577655.1 | AY774712.1 | AY659226.1 | HH791755.1 | DL122591.1 | HW155430.1 | HI988950.1 |
| HC293481.1 | GN359713.1 | AX574411.1 | AY774602.1 | AY659194.1 | HH779720.1 | DL113813.1 | HW155296.1 | HI996566.1 |
| HC293361.1 | GN359661.1 | AX573157.1 | AY774534.1 | AY659162.1 | HH777929.1 | DL099512.1 | HW155212.1 | HI988405.1 |
| HC290193.1 | GN359565.1 | AX556822.1 | AY774491.1 | AY659130.1 | HH774444.1 | DL099480.1 | HW154657.1 | HI987440.1 |
| HC289868.1 | GN359369.1 | AX555191.1 | AY774432.1 | AY659098.1 | HH774199.1 | DL099448.1 | HW154490.1 | HC194693.1 |

| | | | | | | | | |
|---|---|---|---|---|---|---|---|---|
| HC207533.1 | GN365403.1 | AX538885.1 | AY774368.1 | AY659066.1 | HH820926.1 | DL113541.1 | HW154059.1 | FW512094.1 |
| HC199246.1 | GN365260.1 | AX538641.1 | AY774316.1 | AY659034.1 | HH819336.1 | FB316732.1 | HW145235.1 | FW511922.1 |
| HC198938.1 | GN359200.1 | AX538321.1 | AY774269.1 | AY659002.1 | HH759220.1 | FJ154952.1 | HW153372.1 | FW508361.1 |
| AF237862.1 | GN359136.1 | AX537562.1 | AY774214.1 | AY658970.1 | HH759188.1 | CS368376.1 | HW153284.1 | FW508319.1 |
| HC195218.1 | GM638535.1 | AX536406.1 | AY774152.1 | AY658938.1 | HH759156.1 | CS368248.1 | HW126582.1 | FW508222.1 |
| HC193592.1 | GM638503.1 | AX529070.1 | AY366934.1 | AY658906.1 | HH759124.1 | CS367992.1 | HW071649.1 | FW552414.1 |
| DM045514.1 | GM638471.1 | AX528104.1 | AF394894.1 | AY658874.1 | HH759092.1 | CS367544.1 | HW071104.1 | FW559177.1 |
| DM045371.1 | GM633740.1 | AX526703.1 | HC084824.1 | AY658842.1 | HH759028.1 | DL080585.1 | HW103938.1 | HI002552.1 |
| DM045215.1 | GM633708.1 | AX524853.1 | HC083539.1 | AY658810.1 | HH758996.1 | HW049247.1 | HW083276.1 | HI002490.1 |
| DM044915.1 | GM633676.1 | AX351704.1 | DM460993.1 | AY658778.1 | HH758964.1 | HW043247.1 | HW084179.1 | HI004565.1 |
| DM039600.1 | GM625841.1 | AX351206.1 | M35652.1 | AY658746.1 | HH758932.1 | HW041786.1 | HW061868.1 | HI004501.1 |
| DM060879.1 | GM625809.1 | AX349190.1 | GN368281.1 | AY658714.1 | HH758900.1 | HW050556.1 | HW062182.1 | HI004437.1 |
| DM044805.1 | GM625777.1 | AX348917.1 | GN360007.1 | AY658682.1 | HH758868.1 | HW047223.1 | HW065283.1 | HI002467.1 |
| DM039276.1 | GM625745.1 | HV764630.1 | GN359975.1 | AY658650.1 | HH757764.1 | HW053869.1 | HW061317.1 | HI002407.1 |
| DM060700.1 | GM624689.1 | HV764568.1 | GN359943.1 | AY658618.1 | HH757732.1 | HW049579.1 | HW064851.1 | HI002330.1 |
| DM060668.1 | GM624657.1 | HV764241.1 | GN359911.1 | AY658586.1 | HH757700.1 | HV985990.1 | HV750932.1 | HI000538.1 |
| GN067915.1 | GM624322.1 | HV766925.1 | GN359866.1 | AY658554.1 | HH757668.1 | HV985926.1 | HV747484.1 | HI069829.1 |
| GN067599.1 | GM659925.1 | HV344914.1 | GN359590.1 | AY658522.1 | HH757636.1 | HV985894.1 | HV753619.1 | HI180578.1 |
| GN052376.1 | GM659893.1 | HV347358.1 | GN359526.1 | AY658490.1 | HH757604.1 | HV961481.1 | HV753495.1 | HI201461.1 |
| GN051259.1 | GM659861.1 | HV340017.1 | GN359458.1 | AY658458.1 | HH757572.1 | HV964432.1 | HV743101.1 | HI180174.1 |
| GN046256.1 | GM659829.1 | HV347090.1 | GN359426.1 | HI180650.1 | HH757540.1 | HV964308.1 | HV704843.1 | HI202814.1 |
| DM010500.1 | GM659797.1 | HV344638.1 | GN359362.1 | HI179975.1 | HH757508.1 | HV959611.1 | HV704296.1 | HI177880.1 |
| DM010519.1 | GM659765.1 | HV339927.1 | GN359298.1 | HI202818.1 | HH757476.1 | HV963339.1 | HV704220.1 | HI214574.1 |
| DM021917.1 | GM659733.1 | HV342454.1 | GN365388.1 | HI177960.1 | HH757444.1 | HV505394.1 | HV704145.1 | HI214542.1 |
| DM016241.1 | GM692566.1 | HV339675.1 | GN364278.1 | HI214578.1 | HH756257.1 | HV502775.1 | HV703780.1 | HI179725.1 |
| DM021622.1 | GM652344.1 | HV344524.1 | GN359225.1 | HI214546.1 | HI401247.1 | HV502743.1 | HV702485.1 | HI214444.1 |
| DM026467.1 | GM652312.1 | HV342175.1 | GN359193.1 | HI214510.1 | HI401100.1 | HV502711.1 | HV701289.1 | HI200562.1 |
| DM026248.1 | GM652280.1 | HV342093.1 | GN359129.1 | HI179807.1 | HD118643.1 | HV502679.1 | HV701227.1 | HI213786.1 |
| GN043986.1 | GM645385.1 | HV341945.1 | GN359097.1 | HI214448.1 | HD118573.1 | HV502615.1 | HV701195.1 | HI202279.1 |
| GN043308.1 | GM645353.1 | HV341799.1 | GN362987.1 | HI213798.1 | HD118535.1 | HV502583.1 | HV701163.1 | HI181603.1 |
| GN042517.1 | GM645321.1 | HV325844.1 | GN348285.1 | HI185485.1 | HI645154.1 | HV502551.1 | HV701131.1 | HC047275.1 |
| GN034657.1 | GM645289.1 | HV332088.1 | GN346602.1 | HI212981.1 | HI643064.1 | HV502519.1 | HV708199.1 | HC047243.1 |
| GM659124.1 | GM645257.1 | HV331745.1 | GN346569.1 | HI546333.1 | HI642946.1 | HV502487.1 | HV708125.1 | HC047211.1 |
| GM658964.1 | GM645225.1 | HV324037.1 | GN346537.1 | HI546301.1 | HI642842.1 | HV492701.1 | HV701080.1 | HC047179.1 |
| GM651772.1 | GM647591.1 | HV189866.1 | GN340258.1 | HI211522.1 | HI642738.1 | HV453572.1 | HV701048.1 | HC047147.1 |
| GM644780.1 | GM640628.1 | HV192775.1 | GM625181.1 | HI210979.1 | HI642622.1 | HV450061.1 | HV695542.1 | HC047115.1 |
| GM644748.1 | GM647275.1 | HV218131.1 | GM625149.1 | HI210947.1 | HI642518.1 | HV453156.1 | HV444130.1 | HC047083.1 |
| GM644716.1 | GM647243.1 | HV221667.1 | GM637341.1 | HI570423.1 | HI642402.1 | HV449111.1 | HV444022.1 | HC047051.1 |
| GM625187.1 | GM647211.1 | HV247806.1 | GM637309.1 | HI570371.1 | HI642298.1 | HV448772.1 | HV341781.1 | HC047019.1 |
| GM625155.1 | GM870222.1 | HV247164.1 | GM637277.1 | HI565803.1 | HI642194.1 | HV450668.1 | HV322584.1 | HC046987.1 |
| GM624033.1 | GM627617.1 | HV301793.1 | GM644205.1 | HI473082.1 | HI642078.1 | HV444705.1 | FZ413933.1 | HC046955.1 |
| GM624001.1 | GM627577.1 | HV301608.1 | GM642720.1 | HI472920.1 | HI641974.1 | HV444094.1 | FW590744.1 | HC046923.1 |
| GM623969.1 | GM627545.1 | HI001772.1 | GM630380.1 | HI210923.1 | HI641858.1 | HV437532.1 | FW590130.1 | HC040796.1 |
| GM625047.1 | GM661486.1 | HI001735.1 | GM656521.1 | HI580532.1 | HH999787.1 | HV348780.1 | FW589131.1 | HC046863.1 |
| GM625015.1 | GM661454.1 | HI465688.1 | GM656489.1 | HI209397.1 | HH998305.1 | HV341345.1 | FW577606.1 | HC046831.1 |
| GM658912.1 | GM661422.1 | HI465493.1 | GM656457.1 | HI072133.1 | HH998256.1 | HV341224.1 | FW577148.1 | HC046799.1 |
| GM658880.1 | GM661390.1 | HI465366.1 | GM656425.1 | HI004271.1 | HH998219.1 | FZ417514.1 | FW575914.1 | HC046767.1 |

| | | | | | | | | |
|---|---|---|---|---|---|---|---|---|
| GM658848.1 | GM654332.1 | HI001667.1 | GM649272.1 | HI002289.1 | DL476814.1 | FZ413943.1 | HI657444.1 | HC046735.1 |
| GM658816.1 | GM654300.1 | HI001615.1 | GM649240.1 | HI002257.1 | DL462987.1 | FZ419846.1 | FW420807.1 | HC046703.1 |
| GM637411.1 | GM654268.1 | HI001573.1 | GM649208.1 | HI002160.1 | DL476036.1 | FZ419812.1 | FW420555.1 | HC039927.1 |
| GM637379.1 | GM647179.1 | HI003399.1 | GM635128.1 | HI002123.1 | DL481990.1 | FW584442.1 | FW420453.1 | HC046686.1 |
| GM646483.1 | GM647147.1 | HI003356.1 | GM635096.1 | HI000473.1 | DL481722.1 | FW582236.1 | FW420421.1 | HC046654.1 |
| GM646451.1 | A34024.1 | HI003320.1 | GM634965.1 | HI000418.1 | DL460989.1 | FW590754.1 | FW420396.1 | HC046622.1 |
| GM646419.1 | A33975.1 | HI550425.1 | GM669841.1 | HI000376.1 | GM833924.1 | FW590292.1 | FW420364.1 | HC046590.1 |
| GM626877.1 | A30379.1 | HI464726.1 | GM656320.1 | HI000319.1 | FB504549.1 | FW589144.1 | FW420332.1 | HC046558.1 |
| GM626845.1 | A29910.1 | HI464694.1 | GM656288.1 | HI553272.1 | GM831909.1 | HQ161058.1 | HI651874.1 | HC046526.1 |
| GM626813.1 | A31642.1 | HI464662.1 | GM656256.1 | HI583853.1 | GM692564.1 | FW575549.1 | AY145510.1 | HC046494.1 |
| GM653510.1 | A16255.1 | HI549985.1 | GM656224.1 | HI577094.1 | GM712144.1 | FW574611.1 | HH804286.1 | HC046462.1 |
| GM653478.1 | A19187.1 | HI549560.1 | GM656192.1 | HI002088.1 | GM651998.1 | FW574306.1 | HH797407.1 | HC046430.1 |
| GM653446.1 | A13605.1 | HI542543.1 | GM656160.1 | HI002037.1 | GM651966.1 | HH999195.1 | HI551898.1 | HC046398.1 |
| GM653414.1 | A09461.1 | HI462686.1 | DL193894.1 | HI001953.1 | GM651934.1 | HH999146.1 | HI470582.1 | HC046366.1 |
| GM653382.1 | A08463.1 | HI503812.1 | DL193814.1 | HI000285.1 | GM831284.1 | HH999115.1 | HI551640.1 | HC046334.1 |
| GM646383.1 | A04978.1 | HH716016.1 | FB571347.1 | HI000245.1 | GM831182.1 | HH999070.1 | HI538366.1 | HC046083.1 |
| GM646351.1 | A01950.1 | HH714148.1 | CS696151.1 | HI000201.1 | GM676157.1 | HH999025.1 | HI564722.1 | HC046019.1 |
| GM646319.1 | A31932.1 | HH961381.1 | CS696119.1 | HI000145.1 | GM830192.1 | HH998958.1 | HI568867.1 | HC046291.1 |
| GM646287.1 | A30290.1 | HH961349.1 | CS696087.1 | HI000113.1 | GM829997.1 | HH997291.1 | HI564227.1 | HC046259.1 |
| GM646255.1 | A28862.1 | HH961317.1 | CS696055.1 | HI471367.1 | FB509368.1 | HH997173.1 | HI564107.1 | HC046227.1 |
| GM646223.1 | A27718.1 | HH961285.1 | CS696023.1 | HB455126.1 | FB509332.1 | HH998910.1 | HI583095.1 | HC046195.1 |
| GM639388.1 | A20025.1 | HH961253.1 | CS695990.1 | HB463482.1 | FB509300.1 | HH998856.1 | HI586486.1 | HC046163.1 |
| GM639356.1 | A28462.1 | HH975186.1 | CS695958.1 | HB444719.1 | FB509268.1 | HH998801.1 | HH980337.1 | HC046131.1 |
| GM639324.1 | A26060.1 | HH982629.1 | CS695894.1 | DM140520.1 | FB509236.1 | HH998745.1 | HH980172.1 | HC046099.1 |
| GM639292.1 | A25587.1 | HH982233.1 | CS695798.1 | DM139715.1 | FB509221.1 | HH997087.1 | HH980137.1 | HC045981.1 |
| GM639260.1 | A24404.1 | HH974544.1 | CS695734.1 | DM138065.1 | FB509157.1 | HH996983.1 | HH979892.1 | HC045949.1 |
| GM646084.1 | A24544.1 | HH963800.1 | CS695702.1 | DM130824.1 | FB509119.1 | HH996939.1 | HH996736.1 | HC045917.1 |
| GM646052.1 | A21893.1 | HH998070.1 | FB747856.1 | HB436881.1 | FB509085.1 | HH998697.1 | HH996661.1 | HC045885.1 |
| GM646020.1 | A14586.1 | HH998018.1 | FB743944.1 | HB427199.1 | GM827572.1 | HH998656.1 | HH996621.1 | HC045853.1 |
| GM660375.1 | HV962083.1 | HH997958.1 | FB743911.1 | HB403650.1 | GM674201.1 | HH998584.1 | HH996559.1 | HC045821.1 |
| GM660343.1 | HV965332.1 | HH999656.1 | FB743879.1 | HB397685.1 | FB508992.1 | HH998528.1 | HH998474.1 | HC045789.1 |
| GM653156.1 | HV950900.1 | HH997907.1 | FB743847.1 | DM115188.1 | FB508960.1 | HH996914.1 | HH998378.1 | HC045757.1 |
| DL097589.1 | HV947315.1 | HH997836.1 | FB743793.1 | DM044732.1 | FB508928.1 | HH996797.1 | HH998345.1 | HC045725.1 |
| DL097557.1 | HV951880.1 | HH997771.1 | FB761767.1 | GN067947.1 | GM773016.1 | HH996758.1 | HH999969.1 | HC045695.1 |
| DL095402.1 | HV940409.1 | HH999510.1 | FB742920.1 | GN067936.1 | GM709022.1 | HH994542.1 | HH999916.1 | HC045663.1 |
| DL095370.1 | HV940230.1 | HH999453.1 | FB708874.1 | GN067904.1 | GM708990.1 | HH980744.1 | HH979834.1 | HC045631.1 |
| DL107086.1 | HV939882.1 | HH999415.1 | GM036175.1 | GN067808.1 | GM708970.1 | HH977456.1 | HH979766.1 | HC045599.1 |
| DL107054.1 | HV810459.1 | HH999358.1 | GM894290.1 | GN067652.1 | GM655019.1 | HH977356.1 | HH979684.1 | HC045567.1 |
| DL106990.1 | HV936907.1 | HH997651.1 | GM890139.1 | DM025165.1 | GM654987.1 | HH980624.1 | HH999880.1 | HC045535.1 |
| DL106958.1 | HV925521.1 | HH997617.1 | GM963591.1 | HW259980.1 | GM694858.1 | HH980524.1 | HH999736.1 | DM380121.1 |
| DL106926.1 | HV939302.1 | HH995712.1 | GM889529.1 | HW259948.1 | GM838176.1 | HH980455.1 | HH998307.1 | HC036174.1 |
| DL102191.1 | HV936382.1 | HH999698.1 | GM685818.1 | HW259513.1 | GM629754.1 | HH980381.1 | HH998258.1 | HC035741.1 |
| DL106882.1 | HV819632.1 | HH999248.1 | DL127183.1 | HW258918.1 | GM629722.1 | HH980347.1 | HH998221.1 | HC035632.1 |
| DL106853.1 | HV698604.1 | HH999203.1 | DL137141.1 | HW258851.1 | GM642020.1 | HH980287.1 | HH998169.1 | HC037065.1 |
| GM042865.1 | HV698304.1 | HH999154.1 | DL073710.1 | HW257303.1 | GM697110.1 | HH980186.1 | HH996498.1 | HC036951.1 |
| GM841815.1 | HV698137.1 | HH998121.1 | DL028053.1 | HW257271.1 | GM656131.1 | HH980147.1 | HH993635.1 | HB402983.1 |
| GM706920.1 | HV689463.1 | HH999078.1 | DL028021.1 | HW257015.1 | GM656099.1 | HH980095.1 | HH986615.1 | HB398942.1 |

| | | | | | | | | |
|---|---|---|---|---|---|---|---|---|
| GM602752.1 | HV695885.1 | HH999034.1 | DL027989.1 | HV321541.1 | GM656067.1 | HH979860.1 | GM715926.1 | HB397677.1 |
| FB718179.1 | HV688649.1 | HH998972.1 | DL047740.1 | HV119951.1 | GM656035.1 | HH996694.1 | FB661318.1 | DM118346.1 |
| FB715270.1 | HV585084.1 | HH998926.1 | DJ042540.1 | HV187354.1 | GM656003.1 | HH996633.1 | FB984993.1 | DM113495.1 |
| GM061224.1 | HV579312.1 | HH997143.1 | DJ045306.1 | HV195742.1 | GM655971.1 | HH996578.1 | FB983170.1 | DM119396.1 |
| GM060729.1 | HV579109.1 | HH969989.1 | DJ031163.1 | HV208790.1 | GM648946.1 | HH998388.1 | FB753840.1 | DM116213.1 |
| DL259483.1 | HV592479.1 | HH998872.1 | DJ044966.1 | HV214873.1 | GM648914.1 | HH998355.1 | GM618724.1 | DM115172.1 |
| DL241170.1 | HV592413.1 | HH998820.1 | DJ044951.1 | HV302955.1 | GM648882.1 | HH999979.1 | GM842506.1 | HA138173.1 |
| DL241025.1 | HV592327.1 | HH998756.1 | DJ044903.1 | HV309035.1 | GM648850.1 | HH999930.1 | GM615967.1 | GM614113.1 |
| DL240791.1 | HV578511.1 | HH997096.1 | DJ040560.1 | HV312357.1 | GM648818.1 | HH979784.1 | GM840820.1 | GM691484.1 |
| FB748858.1 | HV577239.1 | HH997045.1 | DJ026293.1 | HH794730.1 | GM642015.1 | HH979694.1 | GM840667.1 | GM000707.1 |
| FB674297.1 | HV582878.1 | HH997004.1 | CS721658.1 | HH777931.1 | GM641983.1 | HH999899.1 | CS728563.1 | GM000679.1 |
| DL231405.1 | HV601484.1 | HH996950.1 | CS688535.1 | HH819338.1 | GM641951.1 | HH999856.1 | CS728531.1 | GM043446.1 |
| DL206276.1 | HV585638.1 | HH998666.1 | DJ008384.1 | HI401249.1 | GM641919.1 | HH999806.1 | CS727238.1 | HB388651.1 |
| DL101909.1 | HV571037.1 | HH998598.1 | DJ008320.1 | HI401102.1 | GM641887.1 | HH998320.1 | GM887799.1 | HB386669.1 |
| DL101877.1 | HV566142.1 | HH998543.1 | DJ003318.1 | HI002409.1 | GM641855.1 | GM642954.1 | GM869161.1 | HB385957.1 |
| DL101813.1 | HV573109.1 | HH996922.1 | DD491127.1 | HI002291.1 | GM641823.1 | GM640560.1 | GM879362.1 | HB385925.1 |
| DL101781.1 | HV559809.1 | HH996766.1 | CS671005.1 | HI069840.1 | GM634635.1 | GM647271.1 | GM865467.1 | HB385780.1 |
| DL101749.1 | FW553150.1 | HB865017.1 | CS646210.1 | HI180644.1 | GM634603.1 | GM647239.1 | FB709344.1 | HB342014.1 |
| DL095219.1 | FW556657.1 | HB865009.1 | CS645038.1 | HI178354.1 | GM634539.1 | GM647207.1 | GM863436.1 | HB341966.1 |
| DL111861.1 | FW560804.1 | AY658793.1 | DD461200.1 | HI202816.1 | GM634507.1 | GM627573.1 | GM603725.1 | HB341596.1 |
| DL111829.1 | FW559377.1 | AY658761.1 | DD460028.1 | HI214576.1 | GM634475.1 | GM627541.1 | DL026280.1 | HB340463.1 |
| DL111797.1 | FW510511.1 | AY658729.1 | DD458761.1 | HI214544.1 | GM629670.1 | GM661482.1 | DJ045304.1 | HB339964.1 |
| DL111733.1 | FW510479.1 | AY658697.1 | DD453836.1 | HI179801.1 | GM629606.1 | GM661450.1 | DJ044964.1 | HA642424.1 |
| DL128880.1 | FW506208.1 | AY658665.1 | DD453491.1 | HI214446.1 | GM629574.1 | GM661418.1 | DJ044949.1 | HA641908.1 |
| DL111659.1 | HI931504.1 | AY658633.1 | CS354133.1 | HI212979.1 | GM629542.1 | GM661386.1 | DJ044901.1 | HA641564.1 |
| DL111627.1 | HI936413.1 | FW555594.1 | CS283948.1 | HI546331.1 | GM629510.1 | GM661322.1 | DJ028150.1 | HA641163.1 |
| DL111595.1 | HI930634.1 | FW556532.1 | CS283067.1 | HI546299.1 | GM884933.1 | GM654232.1 | DJ026291.1 | HA641105.1 |
| DL111563.1 | HI935091.1 | FW562827.1 | CS277042.1 | HI211515.1 | GM655939.1 | GM654200.1 | CS686973.1 | HA635425.1 |
| DL111499.1 | HI934971.1 | FW565214.1 | CS276845.1 | HI210977.1 | GM655907.1 | GM647175.1 | CS688516.1 | HA639890.1 |
| DL101653.1 | HI918289.1 | FW562621.1 | CS272574.1 | HI210945.1 | GM655875.1 | GM647143.1 | DJ011752.1 | HA639280.1 |
| DL101621.1 | HI918257.1 | FW562544.1 | CS265239.1 | HI570421.1 | GM655843.1 | GM647111.1 | DD491117.1 | DM106885.1 |
| DL106553.1 | HI916845.1 | FW561200.1 | CS254295.1 | HI209395.1 | GM655811.1 | GM647047.1 | CS671003.1 | DM106853.1 |
| DL106521.1 | HI659100.1 | FW505278.1 | CS244257.1 | HI072081.1 | GM648764.1 | GM647018.1 | CS646208.1 | DM102740.1 |
| DL128719.1 | HI657168.1 | FW508156.1 | CS244225.1 | HI004395.1 | GM655771.1 | DL176638.1 | CS485306.1 | DM106310.1 |
| DL128618.1 | HI655089.1 | FW508113.1 | CS244193.1 | HI004331.1 | GM648754.1 | DL176476.1 | CS485188.1 | DM105484.1 |
| DL117891.1 | HI660317.1 | FW562490.1 | CS243251.1 | HI002287.1 | GM648722.1 | DL176423.1 | DD408904.1 | DM100712.1 |
| DL117859.1 | FW498000.1 | FW555397.1 | CS227301.1 | HI002254.1 | GM648690.1 | DL176335.1 | DD406593.1 | DM095106.1 |
| DL117827.1 | FW500533.1 | FW553091.1 | CS207861.1 | HI002200.1 | GM648658.1 | DL174456.1 | CS479726.1 | DM094914.1 |
| DL092731.1 | HC509298.1 | FW556662.1 | CS189606.1 | HI002157.1 | GM648626.1 | DL176756.1 | DD283508.1 | DM103612.1 |
| DL092699.1 | HC504630.1 | FW559384.1 | CS194008.1 | HI002121.1 | GM648594.1 | DL091955.1 | CS283946.1 | DM093417.1 |
| DL088956.1 | HC504261.1 | FW510516.1 | BD408017.1 | HI000469.1 | GM641791.1 | DL091923.1 | CS276840.1 | DM093349.1 |
| DL088924.1 | HC471037.1 | FW506213.1 | BD429581.1 | HI000416.1 | GM641759.1 | DL091891.1 | CS273109.1 | DM107360.1 |
| DL103299.1 | HC502094.1 | HI936421.1 | BD397191.1 | HI000374.1 | GM641727.1 | DL102884.1 | CS272568.1 | DM093167.1 |
| DL088626.1 | HC500969.1 | HI930639.1 | BD396358.1 | HI203331.1 | GM641695.1 | DL102852.1 | CS265237.1 | GN367595.1 |
| DL088594.1 | HC500235.1 | HI661309.1 | BD396048.1 | HI553270.1 | GM641662.1 | DL102820.1 | CS255264.1 | GN367232.1 |
| DL044878.1 | HC499855.1 | HH976250.1 | BD394873.1 | HI577092.1 | GM641630.1 | DL098001.1 | CS253657.1 | GN370769.1 |
| DL016401.1 | FW300532.1 | HH979587.1 | BD453364.1 | HI520876.1 | GM634442.1 | DL097937.1 | CS244175.1 | GM624306.1 |

| | | | | | | | | |
|---|---|---|---|---|---|---|---|---|
| DL016369.1 | FW298760.1 | HH932002.1 | BD453348.1 | HI520494.1 | GM634410.1 | DL095723.1 | CS244255.1 | GM624274.1 |
| DL016337.1 | HC486493.1 | AX100683.1 | BD453301.1 | HI003852.1 | GM634378.1 | DL095691.1 | CS244223.1 | GM624242.1 |
| DL016305.1 | HC486427.1 | FW394266.1 | BD453269.1 | HI002086.1 | GM634346.1 | DL095659.1 | CS244191.1 | GM624210.1 |
| DL016273.1 | HC485143.1 | FW394247.1 | BD445338.1 | HI002034.1 | GM634282.1 | DL117400.1 | CS243296.1 | GM624178.1 |
| DL016241.1 | HC474872.1 | FW394205.1 | BD376113.1 | HI001951.1 | GM629442.1 | DL101218.1 | CS243076.1 | GM624146.1 |
| DL011725.1 | HC466416.1 | FW393980.1 | BD375398.1 | HI000283.1 | GM629381.1 | DL094720.1 | CS227295.1 | GM652549.1 |
| DL011661.1 | HC482398.1 | FW397858.1 | BD356488.1 | HI000243.1 | CQ778922.1 | DL094688.1 | CS203617.1 | GM652517.1 |
| DL011629.1 | HC491784.1 | FW393439.1 | BD350538.1 | HI000199.1 | CQ778512.1 | DL090945.1 | CS207989.1 | GM652485.1 |
| DL011597.1 | HC491752.1 | FW392972.1 | BD359231.1 | HI000143.1 | CQ774655.1 | DL090913.1 | CS207859.1 | GM652453.1 |
| DL048452.1 | HC491644.1 | FW396671.1 | BD341688.1 | HI000111.1 | CQ771666.1 | DL086977.1 | CS189604.1 | GM652421.1 |
| DL044739.1 | HC481579.1 | HH936313.1 | BD325681.1 | HC010405.1 | CQ771634.1 | DL086945.1 | DD165454.1 | GM652389.1 |
| DL044707.1 | CS359732.1 | HH833669.1 | BD319675.1 | HC010125.1 | CQ771602.1 | DL105834.1 | DD159204.1 | GM659909.1 |
| DL014505.1 | CS359591.1 | HH833637.1 | BD319536.1 | HC008574.1 | CQ770997.1 | DL105802.1 | DD182192.1 | GM659877.1 |
| DL014473.1 | BD269133.1 | HH833605.1 | BD300804.1 | HC011021.1 | CQ768943.1 | DL096519.1 | CS157808.1 | GM659845.1 |
| DL014441.1 | BD268154.1 | HH833573.1 | BD300480.1 | DM370673.1 | CQ768483.1 | DL096487.1 | CS102797.1 | GM659813.1 |
| DL030164.1 | BD268063.1 | HH833541.1 | BD294755.1 | DM370641.1 | CQ768120.1 | DL096455.1 | CS102733.1 | GM659781.1 |
| CS646219.1 | BD263538.1 | HH833509.1 | BD290035.1 | DM370609.1 | CQ766074.1 | DL086405.1 | CS102637.1 | GM659749.1 |
| CS646187.1 | BD263443.1 | HH833477.1 | BD161168.1 | DM370577.1 | CQ766042.1 | DL108372.1 | CS102605.1 | GM712112.1 |
| DD460501.1 | BD263411.1 | HH833445.1 | AX587624.1 | DM370545.1 | CQ761211.1 | DL117890.1 | CS102573.1 | GM659713.1 |
| DD460135.1 | BD251206.1 | HH833413.1 | AX556838.1 | DM370513.1 | CQ761155.1 | DL117858.1 | CS102541.1 | GM659681.1 |
| DD459657.1 | BD249869.1 | HH833381.1 | AX555843.1 | DM370481.1 | CQ760735.1 | DL117826.1 | CS102509.1 | GM659649.1 |
| DD458772.1 | BD247390.1 | HH932679.1 | AX555096.1 | DM371294.1 | CQ758835.1 | DL029523.1 | CS102477.1 | GM659617.1 |
| DD453845.1 | BD247041.1 | HH931884.1 | AX543904.1 | DM209281.1 | CQ757808.1 | DL029491.1 | CS091847.1 | GM659585.1 |
| DD453749.1 | BD246965.1 | HH925735.1 | AX539583.1 | HC003059.1 | CQ757776.1 | DL037996.1 | CS086832.1 | GM659552.1 |
| DD453380.1 | BD245163.1 | FV533143.1 | AX537269.1 | HB865031.1 | CQ757744.1 | DL049700.1 | CS084188.1 | GM652360.1 |
| DD456661.1 | BD243813.1 | FV531712.1 | AX525185.1 | HB864973.1 | CQ757712.1 | DL049668.1 | CS082037.1 | GM652328.1 |
| CS632063.1 | BD243430.1 | FV531680.1 | AX179514.1 | HB864991.1 | CQ756683.1 | DL049627.1 | CS079579.1 | GM652296.1 |
| CS642289.1 | BD243258.1 | FV531016.1 | AX179451.1 | HB864935.1 | CQ754048.1 | DL016634.1 | CS075368.1 | GM652264.1 |
| CS631258.1 | BD242633.1 | FV522810.1 | AX164061.1 | HB864903.1 | CQ654958.1 | DL011862.1 | HV338402.1 | GM652232.1 |
| CS642141.1 | BD242448.1 | FV530226.1 | AX028005.1 | HB864871.1 | CQ654708.1 | DL016400.1 | HV347645.1 | GM652200.1 |
| CS627829.1 | BD238553.1 | FV528455.1 | A28442.1 | HB864839.1 | CQ654453.1 | DL016368.1 | HV347375.1 | GM645401.1 |
| CS632991.1 | BD237707.1 | FV534545.1 | AF430206.1 | HB864807.1 | CQ654395.1 | DL016336.1 | HV344848.1 | GM645369.1 |
| CS632198.1 | BD236967.1 | FV534415.1 | AF430174.1 | HB864775.1 | CQ654297.1 | DL016304.1 | HV347321.1 | GM645337.1 |
| CS632143.1 | BD235481.1 | FV534243.1 | CS118111.1 | HB866525.1 | CQ654215.1 | DL030899.1 | HV340058.1 | GM645305.1 |
| CS626249.1 | BD234405.1 | HC461380.1 | CS118078.1 | DM193658.1 | AX963159.1 | DL030900.1 | HV340026.1 | GM645273.1 |
| CS625506.1 | BD232451.1 | HC465810.1 | CS118045.1 | DM192907.1 | AX962552.1 | DL019745.1 | HV346972.1 | GM645241.1 |
| CS616589.1 | BD231830.1 | AF533146.1 | CS117976.1 | DM195145.1 | AX961578.1 | DL019713.1 | HV344572.1 | GM659532.1 |
| CS614384.1 | BD231156.1 | HC456316.1 | CS115732.1 | DM189899.1 | AX960378.1 | DL010532.1 | HV339968.1 | GM659515.1 |
| CS613352.1 | BD226840.1 | HC453645.1 | CS110174.1 | DM203094.1 | AX958732.1 | DL010500.1 | HV339872.1 | GM659483.1 |
| CS604535.1 | CS228305.1 | HC452100.1 | CS106380.1 | HB828502.1 | AX957836.1 | DL010468.1 | HV344458.1 | GM659451.1 |
| CS402154.1 | AF397137.1 | HC450056.1 | CS102892.1 | HB839920.1 | AX957802.1 | DL010436.1 | HV342109.1 | GM659419.1 |
| CS376533.1 | CS157787.1 | FU774065.1 | CS102860.1 | HB848598.1 | BD010845.1 | DL010404.1 | HV342018.1 | GM659387.1 |
| CS374665.1 | CS157948.1 | FU761008.1 | CS102636.1 | GN031059.1 | AX958023.1 | DL038778.1 | HV341808.1 | GM659355.1 |
| DD308882.1 | CS157916.1 | FU759998.1 | CS102604.1 | GN031027.1 | AX957210.1 | DL038746.1 | HV341782.1 | GM652163.1 |
| DD298330.1 | CS157884.1 | FU759943.1 | CS102572.1 | GN030995.1 | AX952275.1 | CS601954.1 | HV322366.1 | GM652131.1 |
| DD292504.1 | CS124705.1 | FU757633.1 | CS102540.1 | HI508271.1 | AX938908.1 | CS608410.1 | HV333154.1 | GM652099.1 |
| DD291067.1 | CS124665.1 | FU757601.1 | CS102508.1 | HI585908.1 | AX934514.1 | CS608339.1 | HV332150.1 | GM652067.1 |

| | | | | | | | | |
|---|---|---|---|---|---|---|---|---|
| CS365222.1 | CS124631.1 | HC438415.1 | CS091845.1 | HI000015.1 | AX930404.1 | CS600609.1 | HV325400.1 | GM652035.1 |
| CS376408.1 | CS124583.1 | HC441567.1 | CS084185.1 | HI003654.1 | AX926385.1 | CS607048.1 | HV331824.1 | GM645208.1 |
| CS363422.1 | CS119871.1 | DM152675.1 | AX472661.1 | HI003604.1 | AX925257.1 | CS606960.1 | HV324884.1 | GM645176.1 |
| HW350889.1 | CS119533.1 | DM152529.1 | E64486.1 | HI003570.1 | AX923416.1 | CS606874.1 | HV323700.1 | GM645144.1 |
| HW350666.1 | CS119500.1 | DM156191.1 | AX469477.1 | HI003519.1 | AX923383.1 | CS604977.1 | HV313399.1 | GM645112.1 |
| HW340082.1 | CS119433.1 | HB455502.1 | AX468899.1 | HI001845.1 | AX840804.1 | CS604913.1 | HV111862.1 | GM645080.1 |
| HW350168.1 | CS119400.1 | HB455302.1 | AX466980.1 | HI001785.1 | AX840152.1 | CS592363.1 | HV119946.1 | GM645048.1 |
| HW339668.1 | CS119367.1 | HB455022.1 | A15348.1 | HI001749.1 | AX832721.1 | CS592091.1 | HV189880.1 | GM645016.1 |
| HW363788.1 | CS119335.1 | HB453845.1 | AX460811.1 | HI469631.1 | AX827177.1 | CS589304.1 | HI378105.1 | GM638185.1 |
| HW326532.1 | CS119301.1 | HB451921.1 | AX458660.1 | HI469511.1 | AX317562.1 | CS597714.1 | HI377879.1 | GM638153.1 |
| HW339002.1 | CS119269.1 | DM134731.1 | AX458588.1 | HI508181.1 | AX306883.1 | CS584091.1 | HI377252.1 | GM638121.1 |
| HW338930.1 | CS119237.1 | DM143064.1 | AX458209.1 | HI465632.1 | AX306324.1 | CS583654.1 | HI376815.1 | GM638089.1 |
| HW338674.1 | CS119173.1 | DM137106.1 | AX456875.1 | HI575287.1 | AX268766.1 | CS546686.1 | HI375524.1 | GM638057.1 |
| HW070166.1 | CS119141.1 | HB441225.1 | AX454160.1 | HI465378.1 | AX260250.1 | CS141516.1 | HI372275.1 | GM651901.1 |
| HW069808.1 | CS119109.1 | HB432483.1 | AX453558.1 | HI465281.1 | AX304322.1 | CS141484.1 | HI370392.1 | GM651869.1 |
| HW084930.1 | CS119041.1 | HB426102.1 | AX451643.1 | HI001627.1 | AX303588.1 | CS138718.1 | HI369905.1 | GM651837.1 |
| HW068048.1 | CS119009.1 | HB416449.1 | AX443309.1 | HI001585.1 | AX302479.1 | CS143201.1 | HI369126.1 | GM651805.1 |
| HW097667.1 | CS118976.1 | HB403480.1 | AX429275.1 | HI001540.1 | AX300065.1 | CS123414.1 | HI369083.1 | GM645006.1 |
| HW057892.1 | CS118943.1 | HB412640.1 | AX418397.1 | HI003678.1 | AX297774.1 | CS122929.1 | HI369027.1 | GM644974.1 |
| HW049138.1 | CS118911.1 | HB394240.1 | AX402398.1 | HI003370.1 | AX287565.1 | CS122374.1 | HI424143.1 | GM644942.1 |
| HV959857.1 | CS118877.1 | HB397722.1 | AX398977.1 | HI003332.1 | AX287273.1 | HC922352.1 | HI424111.1 | GM644910.1 |
| HV956197.1 | CS118845.1 | HB397654.1 | AX397847.1 | HI550394.1 | AX286562.1 | HC920506.1 | HI424079.1 | GM644878.1 |
| HV951097.1 | CS118811.1 | DM118009.1 | AX394862.1 | HI550324.1 | AX283695.1 | HD066672.1 | HI423789.1 | GM644846.1 |
| HV957544.1 | CS118779.1 | GN030229.1 | AX377467.1 | HI464738.1 | AX283229.1 | HD064838.1 | HI423565.1 | GM644814.1 |
| HV957475.1 | CS118746.1 | GN030197.1 | AX367111.1 | HI464706.1 | AF387345.1 | HD053216.1 | HI422830.1 | GM638015.1 |
| HV953051.1 | CS118714.1 | A19299.1 | AX364659.1 | HI464674.1 | AX282200.1 | HD033942.1 | HI416365.1 | GM637983.1 |
| HV943453.1 | CS118618.1 | A10257.1 | AX364627.1 | HI464642.1 | AX281227.1 | HD033691.1 | HI416160.1 | GM637951.1 |
| HV777031.1 | CS118550.1 | A02702.1 | AX364542.1 | HI528993.1 | AX279966.1 | HD057704.1 | HI415888.1 | GM637919.1 |
| HV775604.1 | CS118484.1 | A31086.1 | AX364510.1 | HI549900.1 | AX279780.1 | HD057672.1 | HI415856.1 | GM637887.1 |
| HW287348.1 | CS118452.1 | A30407.1 | AX364478.1 | HI549593.1 | AX278767.1 | HD062032.1 | HI415823.1 | GM637855.1 |
| HW302738.1 | CS118420.1 | A27896.1 | AX364446.1 | HI548323.1 | AX278290.1 | U14012.1 | HI415791.1 | GM633447.1 |
| HW302518.1 | CS118387.1 | A25894.1 | AX364414.1 | HC490813.1 | AX278108.1 | M57695.1 | HI415759.1 | GM633415.1 |
| HW295691.1 | CS118353.1 | A28480.1 | AX364382.1 | HC490781.1 | AX254827.1 | J02905.1 | HI415184.1 | GM633383.1 |
| HW302336.1 | CS118254.1 | A26796.1 | AX364350.1 | HC490749.1 | AX253169.1 | HC325502.1 | HI414014.1 | GM633351.1 |
| HW302011.1 | CS118155.1 | A26063.1 | AX364190.1 | HC490717.1 | AX249916.1 | HC325096.1 | HI413766.1 | GM633319.1 |
| HW294999.1 | CS118123.1 | A25699.1 | BD011668.1 | HC490685.1 | AX244003.1 | HC316591.1 | HI413452.1 | GM633287.1 |
| HW285857.1 | CS118091.1 | A24417.1 | BD006204.1 | HC490653.1 | AX242241.1 | HC309217.1 | HI516089.1 | GM637832.1 |
| HW294130.1 | CS118024.1 | A23619.1 | DL106508.1 | HC490621.1 | AX242209.1 | HC307887.1 | HI541038.1 | GM637800.1 |
| HW294066.1 | CS117956.1 | A22074.1 | DL128705.1 | HC490589.1 | AX242145.1 | HC307812.1 | HI547696.1 | GM637736.1 |
| HW294021.1 | A23265.1 | A16122.1 | DL128671.1 | HC490557.1 | AX242081.1 | HC307391.1 | HI284308.1 | GM637704.1 |
| HW293000.1 | A22511.1 | A12408.1 | DL125139.1 | HC490521.1 | AX242049.1 | HC306805.1 | HI284276.1 | GM637672.1 |
| HW291252.1 | A21573.1 | A21602.1 | DL125107.1 | HC490489.1 | AX241985.1 | FU262765.1 | HI571712.1 | GM633264.1 |
| HV822663.1 | A14552.1 | A20702.1 | DL125075.1 | HC490450.1 | AX241953.1 | FU265453.1 | HI574497.1 | GM633232.1 |
| HV786533.1 | A21832.1 | A20098.1 | DL125043.1 | HC490418.1 | AX241889.1 | FU265389.1 | HI574465.1 | GM633200.1 |
| HV784690.1 | A21545.1 | HW082953.1 | DL125011.1 | HC490386.1 | AX241857.1 | FU262084.1 | HI574433.1 | GM633168.1 |
| HV778941.1 | A20500.1 | HW088258.1 | DL120986.1 | HC490354.1 | AX241825.1 | HC306795.1 | HI571461.1 | GM633136.1 |
| HV778382.1 | A18736.1 | HW087843.1 | DL120954.1 | HC490322.1 | AX241761.1 | HC306069.1 | HI561765.1 | GM633104.1 |

| | | | | | | | | |
|---|---|---|---|---|---|---|---|---|
| HV777983.1 | A18114.1 | HW081761.1 | DL120922.1 | HC490290.1 | AX241697.1 | HC306029.1 | HI578558.1 | GM625525.1 |
| HV777180.1 | A14397.1 | HW085108.1 | DL088997.1 | HC490257.1 | AX241665.1 | DM000219.1 | HC868030.1 | GM625493.1 |
| HV776126.1 | A12564.1 | HW103519.1 | DL122356.1 | HC490225.1 | AX241601.1 | DM005063.1 | HC887353.1 | GM629176.1 |
| HV774734.1 | A11724.1 | HW098169.1 | DL122324.1 | HC472311.1 | AX241569.1 | DM004981.1 | HC867653.1 | GM629112.1 |
| HV764469.1 | A11117.1 | HW102317.1 | DL122292.1 | HC472278.1 | AX241505.1 | DM003482.1 | HC867475.1 | GM634042.1 |
| HV763976.1 | A10161.1 | HW065295.1 | DL122228.1 | HC472246.1 | AX241473.1 | GM983948.1 | HC867389.1 | GM634010.1 |
| HV767478.1 | A15473.1 | HW065262.1 | DL122196.1 | HC472214.1 | AX241441.1 | GM996603.1 | HC882463.1 | GM633978.1 |
| HV761253.1 | A08104.1 | HW065229.1 | DL118006.1 | HC472182.1 | AX241409.1 | GM996507.1 | HC872743.1 | GM633946.1 |
| HV766835.1 | A07728.1 | HW064870.1 | DL117974.1 | HC471918.1 | AX241102.1 | GM993555.1 | HC889128.1 | GM633914.1 |
| HV760594.1 | A06290.1 | HW064831.1 | DL117942.1 | HC471886.1 | AX241070.1 | GM992506.1 | HC888944.1 | GM633882.1 |
| HV766336.1 | A08599.1 | HW057853.1 | DL117910.1 | HC471972.1 | AX241038.1 | GM976368.1 | HC869707.1 | GM883255.1 |
| HV450365.1 | A05107.1 | HW056523.1 | DL117878.1 | HC471831.1 | AX241006.1 | GM989957.1 | HC869579.1 | GM662862.1 |
| HV453153.1 | A02711.1 | HW060697.1 | DL117846.1 | HC471799.1 | GM635086.1 | GN000749.1 | HC876972.1 | GM655523.1 |
| HV449101.1 | A01535.1 | HW062526.1 | DL117814.1 | HC679503.1 | GM630280.1 | GM969681.1 | HC869519.1 | GM655491.1 |
| HV448767.1 | K00469.1 | HW062398.1 | DL113221.1 | HC679098.1 | GM630248.1 | GM999458.1 | HC876935.1 | GM655459.1 |
| HV450665.1 | M22053.1 | HW060512.1 | DL113189.1 | HC678795.1 | GM630216.1 | FB986406.1 | HC876896.1 | GM655427.1 |
| HV444451.1 | M18398.1 | HW062373.1 | DL113157.1 | HC678635.1 | GM630184.1 | GM970626.1 | HC876690.1 | GM655395.1 |
| HV444088.1 | DD453844.1 | HW062277.1 | DL113093.1 | HC668142.1 | GM630152.1 | GM649269.1 | FW304572.1 | GM655363.1 |
| HV437529.1 | DD453748.1 | HW062245.1 | DL113061.1 | HC668109.1 | GM630120.1 | GM649237.1 | FW310254.1 | GM648370.1 |
| HV344824.1 | CS631256.1 | HW056168.1 | DL108260.1 | HC510424.1 | GM654080.1 | GM649205.1 | FW331844.1 | GM648338.1 |
| HV344740.1 | CS631192.1 | HW042064.1 | DL108228.1 | HC508648.1 | GM654048.1 | GM656317.1 | FW309707.1 | GM648306.1 |
| HI401109.1 | CS627827.1 | HW042032.1 | DL108196.1 | HC504661.1 | GM654016.1 | GM656285.1 | FW334260.1 | GM648274.1 |
| HI637688.1 | CS632984.1 | HW043562.1 | DL108164.1 | HC504604.1 | GM653984.1 | GM656253.1 | FW333476.1 | GM648242.1 |
| HI380632.1 | BD263442.1 | HW041956.1 | DL108132.1 | HC493205.1 | GM646991.1 | GM656221.1 | FW332862.1 | GM648210.1 |
| HI378757.1 | BD261346.1 | HW041924.1 | DL108100.1 | HC503284.1 | GM646959.1 | GM656189.1 | FW306907.1 | GM641407.1 |
| HI378149.1 | BD251205.1 | HW041826.1 | DL103494.1 | HC502241.1 | GM646927.1 | GM656157.1 | FW332800.1 | GM641343.1 |
| HI375908.1 | BD249012.1 | HW049410.1 | DL103462.1 | HC501826.1 | GM646895.1 | GM719321.1 | FW332695.1 | GM641311.1 |
| HI369148.1 | BD248131.1 | HI401433.1 | DL103430.1 | HC500850.1 | GM646863.1 | GM697142.1 | FW306535.1 | GM641279.1 |
| HI369095.1 | BD247034.1 | HI401120.1 | DL103398.1 | HC499874.1 | GM646831.1 | GM037902.1 | FW305397.1 | GM641247.1 |
| HI424340.1 | BD246964.1 | HD117884.1 | DL103366.1 | FW304054.1 | GM639996.1 | GM036864.1 | AY017184.1 | GM629088.1 |
| HI464753.1 | BD243812.1 | HI642674.1 | DL103334.1 | FW302610.1 | GM639964.1 | FB766251.1 | HC084631.1 | GM629024.1 |
| HI464715.1 | BD243429.1 | HI637695.1 | DL098920.1 | FW302076.1 | GM639932.1 | FB766150.1 | HC070335.1 | GM628992.1 |
| HI464683.1 | BD242632.1 | HI379172.1 | DL098888.1 | FW301648.1 | GM639900.1 | FB746058.1 | HC083880.1 | GM628960.1 |
| HI464651.1 | BD242505.1 | HI378124.1 | DL098856.1 | FW301209.1 | GM639868.1 | FB743946.1 | HC083736.1 | GM628928.1 |
| HI464592.1 | BD242447.1 | HI376916.1 | DL098824.1 | FW300632.1 | GM639836.1 | FB743913.1 | AY774957.1 | GM716204.1 |
| HI505027.1 | BD238299.1 | HI376613.1 | DL098792.1 | FW300423.1 | GM627294.1 | FB743849.1 | AY774915.1 | GM655330.1 |
| HI463139.1 | BD237699.1 | HI573168.1 | DL098760.1 | FW299343.1 | GM627262.1 | FB743795.1 | AY774860.1 | GM655298.1 |
| HH725184.1 | BD236966.1 | HI566036.1 | DL092750.1 | FW299311.1 | GM627230.1 | FB761769.1 | AY774799.1 | GM655266.1 |
| HH714034.1 | BD235951.1 | HI472900.1 | DL092718.1 | FW299279.1 | GM627198.1 | FB761507.1 | AY774630.1 | GM655234.1 |
| HH713498.1 | BD235469.1 | HI580546.1 | DL092686.1 | FW299247.1 | GM627166.1 | FB708876.1 | AY774569.1 | GM655202.1 |
| HH961370.1 | BD234404.1 | HI072384.1 | DL092654.1 | FW298801.1 | GM627134.1 | FB708632.1 | AY774513.1 | GM648177.1 |
| HH961338.1 | BD232443.1 | HI004359.1 | DL092622.1 | FW298595.1 | GM661118.1 | GM036213.1 | AY774402.1 | GM648145.1 |
| HH961306.1 | BD231828.1 | HI004231.1 | DL088975.1 | HC470576.1 | GM661086.1 | GM894298.1 | AY774241.1 | GM648113.1 |
| HH961274.1 | BD231155.1 | HI002269.1 | DL088943.1 | HC487982.1 | GM661054.1 | GM890142.1 | AY774185.1 | GM648081.1 |
| HH961242.1 | BD227112.1 | HI002232.1 | DL088911.1 | HC469469.1 | GM661022.1 | GM963595.1 | AY774053.1 | GM648049.1 |
| HH974533.1 | BD226839.1 | HI002182.1 | DL088815.1 | HC469009.1 | GM660990.1 | GM747065.1 | HC084453.1 | GM648017.1 |
| HH998102.1 | BD224584.1 | HI002135.1 | DL088783.1 | HC476819.1 | GM660958.1 | FB983629.1 | HC070332.1 | GM641214.1 |

| | | | | | | | | |
|---|---|---|---|---|---|---|---|---|
| HH997995.1 | BD223824.1 | HI002100.1 | DL103286.1 | HC486632.1 | GM660926.1 | FB983194.1 | HC083874.1 | GM641182.1 |
| HH997936.1 | BD222643.1 | HI000446.1 | DL103254.1 | HC486464.1 | GM653932.1 | DL137757.1 | HC083524.1 | GM641150.1 |
| HH999645.1 | BD218730.1 | HI000398.1 | DL103222.1 | HC486385.1 | GM653900.1 | DL123332.1 | HC081826.1 | GM641118.1 |
| HH997896.1 | BD218042.1 | HI000343.1 | DL098712.1 | HC467987.1 | GM653868.1 | DL089921.1 | DM460343.1 | GM641086.1 |
| HH997815.1 | BD204957.1 | HI553284.1 | DL098680.1 | HC475797.1 | GM653836.1 | DL114115.1 | HC062145.1 | GM641054.1 |
| HH997759.1 | DD283244.1 | HI553180.1 | DL098648.1 | HC492622.1 | GM653772.1 | DL114083.1 | HC061918.1 | GM628894.1 |
| HH999493.1 | DD282376.1 | HI539333.1 | DL098616.1 | HC492565.1 | GM646763.1 | DL114051.1 | HC057887.1 | GM628862.1 |
| HH999440.1 | DD281291.1 | HI583964.1 | DL098584.1 | HC475392.1 | GM646731.1 | DL125593.1 | HC057732.1 | GM628830.1 |
| HH999398.1 | DD279234.1 | HI568957.1 | DL098552.1 | HC483194.1 | GM646699.1 | DL121905.1 | DM460305.1 | GM628798.1 |
| HD033945.1 | CS353466.1 | HI002058.1 | DL108038.1 | HC466615.1 | GM646667.1 | DL112675.1 | HB856018.1 | GM628766.1 |
| HD033693.1 | CS353174.1 | HI002016.1 | DL108006.1 | HC466486.1 | GM646635.1 | FB316571.1 | HB855434.1 | GM628734.1 |
| HD051915.1 | CS352515.1 | HI000265.1 | DL107974.1 | HC474343.1 | GM639832.1 | CS368104.1 | DM179153.1 | GM628702.1 |
| HD057706.1 | CS352394.1 | HI000219.1 | DL107942.1 | HC474175.1 | GM639800.1 | DL080570.1 | DM179058.1 | GM628701.1 |
| HD057674.1 | CS283957.1 | HI000163.1 | DL107910.1 | HC466388.1 | GM639768.1 | DL074109.1 | DM178818.1 | GM628686.1 |
| HD062034.1 | CS283851.1 | HI000125.1 | DL092452.1 | HC492008.1 | GM639736.1 | DL011782.1 | DM170825.1 | GM628654.1 |
| J03566.1 | CQ957073.1 | HI203123.1 | DL092420.1 | HC292624.1 | GM639704.1 | DL010060.1 | DM185692.1 | GM628590.1 |
| AY456384.1 | CQ944183.1 | HI180944.1 | DL092388.1 | HC288845.1 | GM639672.1 | DL014434.1 | DM188242.1 | GM628558.1 |
| HC917648.1 | CQ944151.1 | HI538845.1 | DL088773.1 | HC295598.1 | GM639640.1 | DL014203.1 | DM188043.1 | GM716111.1 |
| HC294182.1 | CQ944119.1 | HI551674.1 | DL088741.1 | HC295520.1 | GM627061.1 | DL014171.1 | HB491795.1 | GM715923.1 |
| HC291330.1 | CQ944087.1 | HI544510.1 | DL088709.1 | HC292074.1 | GM627053.1 | DL014139.1 | HB491463.1 | GM662189.1 |
| HC289367.1 | CQ944055.1 | HI559267.1 | DL088645.1 | HC291906.1 | GM626997.1 | DJ042542.1 | HB488825.1 | GM662157.1 |
| HC289333.1 | CQ944023.1 | HI568847.1 | DL088613.1 | HC291642.1 | GM626965.1 | DJ045314.1 | HB488774.1 | GM662125.1 |
| HC292864.1 | CQ943991.1 | HI564251.1 | DL088581.1 | HC294410.1 | GM626933.1 | DJ031165.1 | HB488691.1 | GM655131.1 |
| HC289003.1 | CQ943959.1 | HI564120.1 | DL113023.1 | HC293846.1 | GM646597.1 | DJ044905.1 | DM164027.1 | GM655099.1 |
| HC292732.1 | CQ943927.1 | HI563644.1 | DL112991.1 | HC293666.1 | GM646563.1 | DJ028154.1 | DM164012.1 | GM655035.1 |
| HC288888.1 | CQ943895.1 | HI563612.1 | DL112959.1 | HC290713.1 | GM646531.1 | CS684775.1 | DM163978.1 | GM647983.1 |
| HC295618.1 | CQ943863.1 | HI563577.1 | DL112927.1 | HC290263.1 | GM646499.1 | FB360086.1 | DM163321.1 | GM647919.1 |
| HC295474.1 | CQ924520.1 | HI508547.1 | DL112895.1 | HC208419.1 | GM646467.1 | FB342841.1 | HB486514.1 | GM647887.1 |
| HC292434.1 | CQ918247.1 | HI551324.1 | DL112863.1 | HC289886.1 | GM646435.1 | DL088354.1 | HB485580.1 | GM647855.1 |
| HC295337.1 | CQ899270.1 | U26410.1 | DL098508.1 | HC289852.1 | GM639632.1 | DL088322.1 | GN335362.1 | GM647823.1 |
| HC292182.1 | CQ898669.1 | HC057023.1 | DL098476.1 | HC207918.1 | GM639600.1 | DL088290.1 | GN116533.1 | GM641019.1 |
| HC294756.1 | CQ898637.1 | HC478536.1 | DL098444.1 | HC207675.1 | GM639568.1 | DL088258.1 | GN116501.1 | GM640987.1 |
| HC299172.1 | CQ898605.1 | FV531707.1 | DL098412.1 | HC207370.1 | GM639536.1 | DL088226.1 | GN116468.1 | GM640923.1 |
| HC294528.1 | CQ898573.1 | FV531675.1 | DL098380.1 | HC202648.1 | GM639440.1 | DL088194.1 | GN116436.1 | GM640891.1 |
| HC293954.1 | CQ898271.1 | FV523746.1 | DL098348.1 | HC201464.1 | GM626893.1 | DL088162.1 | GN116372.1 | GM640859.1 |
| HC293774.1 | CQ895917.1 | FV522845.1 | DL092338.1 | HC197710.1 | GM626861.1 | DL088130.1 | GN116340.1 | GM628505.1 |
| HC293277.1 | CQ895547.1 | FV522805.1 | DL092306.1 | DM465190.1 | GM626829.1 | DL088098.1 | GN116308.1 | GM628473.1 |
| HC293080.1 | CQ890949.1 | FV528441.1 | DL092274.1 | DM464474.1 | GM626797.1 | DL088066.1 | GN114936.1 | GM628441.1 |
| HC289861.1 | CQ887982.1 | FV534441.1 | DL092210.1 | HC196175.1 | GM626765.1 | DL088002.1 | GN095164.1 | GM628409.1 |
| HC296937.1 | CQ875526.1 | FV534238.1 | DL092178.1 | HC195240.1 | GM626733.1 | DL091943.1 | GN114207.1 | GM628377.1 |
| HC207397.1 | CQ868894.1 | FV534085.1 | DL044961.1 | HC194251.1 | GM653708.1 | DL091911.1 | GN094515.1 | GM628345.1 |
| HC201560.1 | CQ861366.1 | HC465653.1 | DL044929.1 | HC193419.1 | GM653676.1 | DL091847.1 | GN094483.1 | GM628313.1 |
| HC199615.1 | CQ859634.1 | HB485497.1 | DL044865.1 | HC190398.1 | GM653644.1 | DL091815.1 | GN094451.1 | GM654955.1 |
| DM464634.1 | CQ859602.1 | HB475498.1 | DL016291.1 | HC187663.1 | GM653580.1 | DL091783.1 | GN113449.1 | DL102627.1 |
| DM462696.1 | CQ854739.1 | HB475031.1 | DL016259.1 | HC187077.1 | GM660905.1 | DL102904.1 | DM074534.1 | DL102595.1 |
| HC195262.1 | CQ849451.1 | HB469139.1 | DL016227.1 | HC090269.1 | GM660873.1 | DL102872.1 | DM078970.1 | DL102563.1 |
| HC193433.1 | CQ846942.1 | HB468765.1 | DL028320.1 | HC089560.1 | GM660841.1 | DL102840.1 | DM063718.1 | DL102531.1 |

| | | | | | | | | |
|---|---|---|---|---|---|---|---|---|
| HC190453.1 | CQ846364.1 | HB461512.1 | DL028288.1 | HC089528.1 | GM660809.1 | DL102808.1 | DM070730.1 | DL098021.1 |
| HC089569.1 | BD074936.1 | HB455480.1 | DL028256.1 | HC089496.1 | GM660777.1 | DL102776.1 | DM069077.1 | DD181287.1 |
| HC089537.1 | BD016719.1 | HB455136.1 | DL028224.1 | HC089464.1 | GM660745.1 | DL102744.1 | DM091323.1 | DD163532.1 |
| HC089505.1 | BD014218.1 | HB454951.1 | DL028192.1 | HC089432.1 | GM653558.1 | DL107684.1 | DM068816.1 | HW307877.1 |
| HC089473.1 | BD014183.1 | HB451911.1 | DL009969.1 | HC089399.1 | GM653526.1 | DL107652.1 | GN091069.1 | HW307845.1 |
| HC089441.1 | BD014031.1 | GN359230.1 | DL009937.1 | HC089367.1 | GM653494.1 | DL107620.1 | GN087204.1 | HW307813.1 |
| HC089408.1 | AX490802.1 | GN359198.1 | DL009905.1 | HC089335.1 | GM653430.1 | DL107588.1 | GN093771.1 | HW307781.1 |
| HC089376.1 | AX474386.1 | GN359166.1 | DL009873.1 | HC089302.1 | GM653398.1 | DL107556.1 | GN089866.1 | HW316593.1 |
| HC089344.1 | E58953.1 | GN359102.1 | DL009841.1 | HC089270.1 | GM646399.1 | DL107524.1 | GN089802.1 | HW307743.1 |
| HC089311.1 | AX468157.1 | GN359072.1 | DL009809.1 | HC087853.1 | GM646367.1 | DL107492.1 | GN082896.1 | HW307711.1 |
| HC089279.1 | AX463612.1 | GN359040.1 | DL009777.1 | HC086701.1 | GM646335.1 | DL107464.1 | GN082825.1 | HW307588.1 |
| HC089201.1 | AX458619.1 | GN363009.1 | DL009745.1 | HC086291.1 | GM646303.1 | DL107416.1 | GN088988.1 | HW315571.1 |
| AY774932.1 | AX457968.1 | GN346574.1 | DL009713.1 | HC085682.1 | GM646271.1 | DL107424.1 | GN092820.1 | HW315538.1 |
| AY774891.1 | AX454119.1 | GN346542.1 | DL009681.1 | DQ459359.1 | GM646239.1 | DL107368.1 | GM827552.1 | HW315146.1 |
| AY774825.1 | AX451949.1 | GN340272.1 | DL009649.1 | AY774920.1 | GM639436.1 | DL107336.1 | GM709016.1 | HW314961.1 |
| AY774710.1 | A35277.1 | GN337781.1 | DL009617.1 | AY774809.1 | GM639404.1 | DL107304.1 | GM708984.1 | HW314795.1 |
| GN067844.1 | A34185.1 | GN131881.1 | DL009585.1 | AY774752.1 | GM639372.1 | DL102691.1 | GM708803.1 | HW314750.1 |
| GN067624.1 | AX443268.1 | GN116521.1 | DL009553.1 | AY774696.1 | DL087811.1 | DL102659.1 | GM655013.1 | HW314658.1 |
| GN067592.1 | A25445.1 | GN116489.1 | DL009521.1 | AY774637.1 | CS606926.1 | DL094391.1 | GM654981.1 | HW160798.1 |
| GN052369.1 | AX427569.1 | GN116456.1 | DL009457.1 | AY774520.1 | CS605119.1 | DL110848.1 | GM838469.1 | HW106141.1 |
| GN059768.1 | AX427161.1 | GN116424.1 | DL026341.1 | AY774410.1 | CS605087.1 | DL110816.1 | GM044476.1 | HW114530.1 |
| GN051249.1 | AX419817.1 | GN116392.1 | DL026309.1 | AY774351.1 | CS605055.1 | DL110784.1 | GM754519.1 | HW113128.1 |
| DM014283.1 | AX418273.1 | GN115060.1 | DL026277.1 | AY774303.1 | CS599816.1 | DL110752.1 | GM754234.1 | HW112987.1 |
| DM021909.1 | CQ979343.1 | GN094659.1 | DL026245.1 | AY774249.1 | CS605028.1 | DL110720.1 | GM752450.1 | HW112857.1 |
| DM016035.1 | CQ977702.1 | DM078715.1 | DL026181.1 | AY774194.1 | CS604996.1 | DL090750.1 | DL467585.1 | HW112707.1 |
| DM015780.1 | CQ972385.1 | DM077798.1 | DL018991.1 | AY774132.1 | CS604932.1 | DL090718.1 | GM652756.1 | HW112627.1 |
| DM026234.1 | CQ972353.1 | GM645190.1 | DL018959.1 | AF455787.1 | CS604900.1 | DL090686.1 | GM652724.1 | HW112560.1 |
| GN041494.1 | CQ971106.1 | GM638135.1 | DL018927.1 | HC070220.1 | CS604836.1 | DL090654.1 | GM652692.1 | HW112468.1 |
| GN039770.1 | CQ970338.1 | GM638103.1 | DL018895.1 | HC070172.1 | CS604804.1 | DL090622.1 | GM652660.1 | HW112377.1 |
| GN034804.1 | CQ957072.1 | GM638071.1 | DL018863.1 | HC070337.1 | CS604772.1 | DL090590.1 | GM652628.1 | HW112286.1 |
| GN030762.1 | CQ944182.1 | GM644828.1 | DL018831.1 | GM645475.1 | CS604740.1 | DL086585.1 | GM652596.1 | HW072757.1 |
| GN030634.1 | CQ944150.1 | GM633365.1 | DL034498.1 | GM645443.1 | CS604708.1 | DL086553.1 | GM645502.1 | HW084954.1 |
| GN030602.1 | CQ944118.1 | GM633333.1 | DL034466.1 | GM645411.1 | CS604676.1 | DL086521.1 | GM645470.1 | HW084915.1 |
| GN030538.1 | CQ944086.1 | GM633301.1 | DL034434.1 | GM638614.1 | CS604642.1 | DL086489.1 | GM645438.1 | HW097748.1 |
| GN030506.1 | CQ944054.1 | GM633214.1 | DL034402.1 | GM638582.1 | CS604610.1 | DL086457.1 | GM746436.1 | HW088959.1 |
| GN030474.1 | CQ944022.1 | GM633182.1 | DL034370.1 | GM638550.1 | CS604578.1 | DL086425.1 | GM638609.1 | HW065063.1 |
| GN030346.1 | CQ943958.1 | GM651771.1 | DL034338.1 | GM638518.1 | CS597525.1 | DL115623.1 | GM638577.1 | HW062592.1 |
| GN030250.1 | CQ943926.1 | GM643113.1 | DL030130.1 | GM638486.1 | CS593093.1 | DL115591.1 | GM638545.1 | HW043638.1 |
| GN030218.1 | CQ943894.1 | GM643049.1 | DL014402.1 | GM638454.1 | CS592820.1 | DL115559.1 | GM638513.1 | HW050533.1 |
| GN029898.1 | CQ943862.1 | GM643017.1 | DL014338.1 | GM633851.1 | CS589283.1 | DL115527.1 | GM638481.1 | HV969761.1 |
| GN029866.1 | CQ918246.1 | GM657341.1 | DL014306.1 | GM633819.1 | CS593372.1 | DL115495.1 | HC289349.1 | HV956160.1 |
| GN029832.1 | CQ898819.1 | GM642956.1 | DL014274.1 | GM633787.1 | CS585118.1 | DL115463.1 | HC289317.1 | HV951087.1 |
| GN014337.1 | CQ898668.1 | GM738841.1 | DL014242.1 | GM633755.1 | CS584695.1 | DL115438.1 | HC289116.1 | HV957533.1 |
| GM634547.1 | CQ898636.1 | GM635065.1 | DL009358.1 | GM633723.1 | CS583917.1 | DL115406.1 | HC289002.1 | HV957463.1 |
| GM634515.1 | CQ898604.1 | GM634970.1 | DL009326.1 | GM633691.1 | CS581992.1 | DL115374.1 | HC296025.1 | HV948607.1 |
| GM634483.1 | CQ898270.1 | GM656325.1 | DL009294.1 | GM833885.1 | CS575751.1 | DL115342.1 | HC292984.1 | HV946165.1 |
| GM884941.1 | CQ895915.1 | GM656293.1 | DL009262.1 | GM625920.1 | CS574926.1 | DL115310.1 | HC288871.1 | HV944852.1 |

| | | | | | | | | |
|---|---|---|---|---|---|---|---|---|
| GM664833.1 | CQ895546.1 | GM656261.1 | DL009230.1 | GM625856.1 | CS574799.1 | DL115278.1 | HC295570.1 | HV943418.1 |
| GM648730.1 | CQ890946.1 | GM656229.1 | DL018656.1 | GM625824.1 | CS573049.1 | DL115246.1 | HC292386.1 | HV780042.1 |
| GM648698.1 | AX142655.1 | GM656197.1 | DL009204.1 | GM625792.1 | CS579866.1 | DL110644.1 | HC295133.1 | HV778438.1 |
| GM648666.1 | AX142591.1 | GM656165.1 | DL009172.1 | GM625760.1 | CS565621.1 | DL110612.1 | HC294948.1 | HV773872.1 |
| GM648634.1 | AX142527.1 | GM649172.1 | DL009140.1 | GM624730.1 | CS570751.1 | DL110580.1 | HC291822.1 | HV757472.1 |
| GM648602.1 | AX142461.1 | GM642177.1 | DL009108.1 | GM624704.1 | CS570681.1 | DL110548.1 | HC299191.1 | HV757249.1 |
| GM634450.1 | AX142333.1 | GM642145.1 | DL009076.1 | GM624672.1 | CS570675.1 | DL110516.1 | HC299133.1 | HV743390.1 |
| GM629463.1 | AX142077.1 | GM642113.1 | DL009044.1 | GM624640.1 | CS545657.1 | DL110484.1 | HC294708.1 | HV819500.1 |
| GM619898.1 | AX142011.1 | GM642081.1 | DL014228.1 | GM624608.1 | CS566234.1 | DL105679.1 | HC291186.1 | HV803294.1 |
| GM648314.1 | AX141753.1 | GM642049.1 | DL014196.1 | GM624576.1 | CS561154.1 | DL105647.1 | HC291006.1 | HV927368.1 |
| GM641383.1 | AX141561.1 | GM719201.1 | DL014164.1 | GM624544.1 | CS560467.1 | DL105615.1 | HC291210.1 | HV932700.1 |
| GM641351.1 | AX141497.1 | GM648960.1 | DL014132.1 | GM624511.1 | BD251242.1 | DL105583.1 | HC290629.1 | HV929741.1 |
| DL106951.1 | AX136888.1 | GM648928.1 | DL014100.1 | GM624479.1 | BD250817.1 | DL105551.1 | HC290170.1 | HV929582.1 |
| DL106919.1 | AX127335.1 | GM648896.1 | DL014068.1 | GM624447.1 | BD247118.1 | DL105519.1 | HC290138.1 | HV803250.1 |
| DL106889.1 | AX118834.1 | GM648864.1 | DL014036.1 | GM624415.1 | BD247021.1 | DL100906.1 | HC289879.1 | HV803218.1 |
| AY658843.1 | AX113383.1 | GM648832.1 | DL026126.1 | GM624383.1 | BD246887.1 | DL100874.1 | HC289845.1 | HV803186.1 |
| AY658811.1 | AX108998.1 | GM648800.1 | DL026094.1 | GM624351.1 | BD244809.1 | DL100842.1 | HC207908.1 | HV803154.1 |
| AY658779.1 | AX107115.1 | GM634553.1 | DL026062.1 | GM633654.1 | BD243980.1 | DL100810.1 | GN130954.1 | HV803122.1 |
| AY658747.1 | AX106711.1 | GM634521.1 | DL026030.1 | GM633622.1 | BD242466.1 | DL100778.1 | GN336958.1 | HV803090.1 |
| AY658715.1 | AX100877.1 | GM634489.1 | DL025998.1 | GM633590.1 | BD238493.1 | DL100746.1 | GN116531.1 | HV819022.1 |
| AY658683.1 | AX097928.1 | GM664841.1 | DL025966.1 | GM633558.1 | BD237564.1 | DL096332.1 | GN116499.1 | HV802952.1 |
| AY658651.1 | AX093444.1 | GM664582.1 | DL018575.1 | GM633526.1 | BD236914.1 | DL096300.1 | GN116434.1 | HV802920.1 |
| AY658619.1 | AX093085.1 | DL176266.1 | DL018543.1 | GM633494.1 | BD235854.1 | DL096268.1 | GN116402.1 | HV802888.1 |
| AY658587.1 | AX085326.1 | DL174458.1 | DL018511.1 | GM625716.1 | BD235377.1 | DL096236.1 | GN116370.1 | HV802859.1 |
| AY658555.1 | AX076557.1 | DL182870.1 | DL018479.1 | GM625684.1 | BD234883.1 | DL096204.1 | GN116338.1 | HV780970.1 |
| AY658523.1 | AX074062.1 | DL176758.1 | DL018447.1 | GM625652.1 | BD232425.1 | DL096172.1 | GN116306.1 | HV813457.1 |
| AY658491.1 | AX068065.1 | DL176653.1 | DL018415.1 | GM625588.1 | BD232108.1 | DL120192.1 | GN095156.1 | HV822603.1 |
| AY658459.1 | AX058593.1 | DL091925.1 | DL014013.1 | GM625556.1 | BD231949.1 | DL120160.1 | GN094513.1 | HV817941.1 |
| AY658427.1 | AX057148.1 | DL091893.1 | DL013981.1 | GM638435.1 | BD231446.1 | DL120128.1 | GN094481.1 | HV822530.1 |
| AY658395.1 | AX052940.1 | DL091861.1 | DL013949.1 | GM638403.1 | BD231141.1 | DL120096.1 | DM074532.1 | HV822397.1 |
| AY658363.1 | AX052905.1 | DL102886.1 | DL013917.1 | GM638371.1 | BD231103.1 | DL120064.1 | DM077892.1 | HV822308.1 |
| AY658331.1 | HW247745.1 | DL102854.1 | DL013885.1 | GM638339.1 | BD231065.1 | DL120032.1 | DM069075.1 | HV817657.1 |
| AY658299.1 | HW239294.1 | DL102822.1 | DL013849.1 | GM638307.1 | BD229939.1 | HW335988.1 | DM091321.1 | HV817130.1 |
| AY658267.1 | HW238610.1 | DL107570.1 | DL030113.1 | GM638275.1 | BD227586.1 | HW335937.1 | GN087803.1 | HV816796.1 |
| AY658235.1 | HW238390.1 | DL102609.1 | DL030081.1 | GM633471.1 | BD227177.1 | HW328608.1 | GN089862.1 | HV784518.1 |
| AY658203.1 | HW238254.1 | DL098003.1 | DL030049.1 | GM624305.1 | BD226659.1 | HW328393.1 | GN089798.1 | HV821235.1 |
| AY658171.1 | HW237926.1 | DL097971.1 | DL030017.1 | GM624273.1 | BD225792.1 | HW328301.1 | GN089196.1 | HV780282.1 |
| AY658139.1 | HW248665.1 | GM658845.1 | DL029985.1 | GM624241.1 | BD225760.1 | HW335666.1 | GM633647.1 | HV780160.1 |
| AY658107.1 | HW237525.1 | GM658813.1 | DL029953.1 | GM624209.1 | BD225728.1 | HW328242.1 | GM633615.1 | HV779811.1 |
| AY658075.1 | HW237348.1 | GM637408.1 | DL046642.1 | GM624177.1 | BD225696.1 | HW328098.1 | GM633583.1 | HV779594.1 |
| AY658043.1 | HW242305.1 | GM637344.1 | DD431473.1 | GM624145.1 | BD225664.1 | HW318510.1 | GM633551.1 | HV778905.1 |
| AY658011.1 | HW243339.1 | GM637312.1 | DD420083.1 | GM652548.1 | BD225632.1 | HW314325.1 | GM633519.1 | HV778343.1 |
| AY657979.1 | HW241620.1 | GM637280.1 | DD432979.1 | GM652516.1 | BD224253.1 | HW313624.1 | GM633487.1 | HV777608.1 |
| AY657947.1 | HW243081.1 | GM637135.1 | DD434696.1 | GM652484.1 | BD222525.1 | HW312139.1 | GM638396.1 | HV777426.1 |
| AY657915.1 | HW161105.1 | GM637103.1 | DD421980.1 | GM652452.1 | BD218296.1 | HW312081.1 | GM624330.1 | HV777076.1 |
| AY657883.1 | HW160495.1 | GM637071.1 | DD420779.1 | GM652420.1 | BD218005.1 | HW311848.1 | GM624298.1 | HV775948.1 |
| AY657851.1 | HW155791.1 | GM646876.1 | CS541732.1 | GM652388.1 | BD211429.1 | HW311340.1 | GM624266.1 | HV775656.1 |

| | | | | | | | | |
|---|---|---|---|---|---|---|---|---|
| AY657819.1 | HW155770.1 | GM646844.1 | CS543092.1 | GM659908.1 | BD209871.1 | HW309718.1 | GM624170.1 | HV774957.1 |
| AY657787.1 | HW155704.1 | GM646812.1 | CS542566.1 | GM659876.1 | BD195122.1 | HW309503.1 | GM659901.1 | HV764779.1 |
| AY657755.1 | HW155502.1 | GM640009.1 | DL087342.1 | GM659844.1 | DD283676.1 | HW309437.1 | GM659869.1 | HV764747.1 |
| AY657723.1 | HW155327.1 | GM639977.1 | DL087310.1 | GM659812.1 | DD283608.1 | HW120105.1 | GM659773.1 | HV769883.1 |
| AY657691.1 | HW056102.1 | GM639913.1 | DL087278.1 | GM659780.1 | DD283505.1 | HW119943.1 | GM659741.1 | HV774644.1 |
| AY657659.1 | HW042090.1 | GM639849.1 | DL087246.1 | GM659748.1 | DD282970.1 | HW119911.1 | GM712518.1 | HV774493.1 |
| AY657627.1 | HW042058.1 | GM627275.1 | DL087214.1 | GM712111.1 | DD282293.1 | HW119879.1 | GM659705.1 | HV763600.1 |
| AY657595.1 | HW042026.1 | GM627243.1 | DL102107.1 | GM659712.1 | DD281825.1 | HW119847.1 | GM659673.1 | HV214870.1 |
| AY657563.1 | HW043556.1 | GM627211.1 | DL102075.1 | GM659680.1 | DD281083.1 | HW119815.1 | GM659641.1 | HV227052.1 |
| AY657531.1 | HW042014.1 | GM627179.1 | DL102043.1 | GM659648.1 | DD287745.1 | HW119740.1 | GM659609.1 | HV247239.1 |
| AY657499.1 | HW041982.1 | GM627147.1 | DL102011.1 | GM659616.1 | DD287637.1 | HW119644.1 | GM659576.1 | HV247207.1 |
| AY657467.1 | HW041950.1 | GM660971.1 | DL101979.1 | GM659584.1 | DD279030.1 | HW118928.1 | GM659544.1 | HV301753.1 |
| AY657435.1 | HW041918.1 | GM660939.1 | DL097533.1 | GM659551.1 | DD278874.1 | HW118896.1 | GM652352.1 | HV306190.1 |
| AY657403.1 | HW041820.1 | GM646776.1 | DL097501.1 | GM652359.1 | CS354064.1 | HW118444.1 | GM652320.1 | HV302952.1 |
| AY657371.1 | HW049404.1 | GM646744.1 | DL097469.1 | GM652327.1 | CS349759.1 | HW115561.1 | GM652288.1 | HV312749.1 |
| AY657339.1 | HW049372.1 | GM646712.1 | DL097437.1 | GM652295.1 | CS353274.1 | HW114440.1 | GM652256.1 | HV312676.1 |
| AY657307.1 | HW049341.1 | GM646648.1 | DL097405.1 | GM652263.1 | CS353045.1 | HW114392.1 | GM645393.1 | HV308919.1 |
| AY657275.1 | HW049277.1 | GM646616.1 | DL097373.1 | GM652231.1 | CS283943.1 | HW101699.1 | GM645361.1 | HV302695.1 |
| AY657243.1 | HW049245.1 | GM044573.1 | DL114235.1 | GM652199.1 | CS282619.1 | HW101661.1 | GM645329.1 | HV311111.1 |
| AY657211.1 | HW041816.1 | GM646480.1 | DL114203.1 | GM645400.1 | CS276836.1 | HW087123.1 | GM645297.1 | HV318756.1 |
| AY657179.1 | HW047221.1 | GM646448.1 | DL114171.1 | GM645336.1 | BD274285.1 | HW087091.1 | GM645265.1 | HV312253.1 |
| AY657147.1 | HW053867.1 | GM646416.1 | DL114139.1 | GM645304.1 | BD273684.1 | HW087079.1 | GM645233.1 | HV310998.1 |
| AY657115.1 | HV985988.1 | GM626874.1 | DL114107.1 | GM645272.1 | BD273391.1 | HW087047.1 | GM659379.1 | HV310925.1 |
| AY657083.1 | HV961479.1 | GM626842.1 | DL114075.1 | GM645240.1 | BD272327.1 | HW087015.1 | GM659347.1 | HV317251.1 |
| AY657051.1 | HV964303.1 | GM626778.1 | DL126215.1 | GM652174.1 | DL033718.1 | HW086976.1 | GM652155.1 | HV221455.1 |
| U39202.1 | HV959607.1 | GM653539.1 | DL126183.1 | GM659514.1 | DL042248.1 | HW086942.1 | GM652123.1 | HV217830.1 |
| M16805.1 | HV515506.1 | GM653507.1 | DL126151.1 | GM659482.1 | DL042216.1 | HW086907.1 | GM652091.1 | HV235905.1 |
| M35113.1 | HV515474.1 | GM653475.1 | DL122935.1 | GM659450.1 | DL042184.1 | HW105285.1 | GM652059.1 | HV316812.1 |
| M34329.1 | HV515442.1 | GM653443.1 | DL122903.1 | GM659386.1 | DL042152.1 | HW105052.1 | GM652027.1 | HV310802.1 |
| DM459947.1 | HH997638.1 | GM653411.1 | DL122839.1 | GM659354.1 | DL042120.1 | HW072606.1 | GM645200.1 | HV309495.1 |
| DM381886.1 | HH997581.1 | GM653379.1 | DL122775.1 | GM652162.1 | DL042088.1 | HW104686.1 | GM645168.1 | HV221079.1 |
| HC053844.1 | HH997515.1 | GM646380.1 | DL118585.1 | GM648112.1 | DL046435.1 | HW096236.1 | GM645136.1 | HV040142.1 |
| HC051845.1 | HH997420.1 | DL109180.1 | DL118553.1 | GM648080.1 | DL046403.1 | HW086515.1 | GM645104.1 | HV114416.1 |
| DM191208.1 | HH997367.1 | DL109148.1 | DL118521.1 | GM648048.1 | DL046371.1 | HW104621.1 | GM645072.1 | HV203317.1 |
| HC047472.1 | HH999687.1 | DL109116.1 | DL118489.1 | GM648016.1 | DL046339.1 | HW071997.1 | GM645040.1 | HV182467.1 |
| HC046868.1 | HH999232.1 | DL109084.1 | DL118457.1 | GM641213.1 | DL046307.1 | HW104428.1 | GM638241.1 | HV182435.1 |
| HC046836.1 | HH999189.1 | DL104382.1 | DL118425.1 | GM641181.1 | DL046275.1 | HW104382.1 | GM638209.1 | HV182403.1 |
| HC046804.1 | HH999143.1 | DL104350.1 | DL113997.1 | GM641149.1 | DL033709.1 | HW104282.1 | GM638177.1 | HV182371.1 |
| HC046772.1 | HH999112.1 | DL104318.1 | DL113965.1 | GM641117.1 | DL033677.1 | HW104240.1 | GM638145.1 | HV246860.1 |
| HC046740.1 | HH999063.1 | DL089959.1 | DL113933.1 | GM641085.1 | DL033645.1 | HW104160.1 | GM638113.1 | HV216318.1 |
| HC046708.1 | HH999022.1 | DL123155.1 | DL113901.1 | GM641053.1 | DL033613.1 | HW104120.1 | GM638081.1 | HV039651.1 |
| HC039682.1 | HH998955.1 | DL123123.1 | DL113869.1 | GM628893.1 | DL033581.1 | HW103887.1 | GM644838.1 | HV117907.1 |
| HB436916.1 | HH997267.1 | DL114121.1 | DL109068.1 | GM628861.1 | DL033549.1 | HW099559.1 | GM638039.1 | HV117765.1 |
| HB435571.1 | HH978166.1 | DL114089.1 | DL109036.1 | GM628829.1 | DL029509.1 | HW085489.1 | GM648572.1 | HV117671.1 |
| HB433138.1 | HH998907.1 | DL114057.1 | DL109004.1 | GM628797.1 | DL029477.1 | HW085348.1 | GM648540.1 | HV116994.1 |
| HB423171.1 | HH998851.1 | DL126229.1 | DL108972.1 | GM628765.1 | DL029445.1 | HW068949.1 | GM648508.1 | HV038605.1 |
| HB394258.1 | HH998798.1 | DL126197.1 | DL108940.1 | GM628733.1 | DL029413.1 | HV690932.1 | HV701182.1 | HV038549.1 |

| | | | | | | | | |
|---|---|---|---|---|---|---|---|---|
| HB397687.1 | HH998739.1 | DL126165.1 | DL108908.1 | GM618952.1 | DL029381.1 | HV698117.1 | HV701150.1 | HV182333.1 |
| DM114644.1 | HH997030.1 | DL122949.1 | DL104302.1 | GM628685.1 | DL046239.1 | HV697913.1 | HV701118.1 | HV182301.1 |
| GM000753.1 | HH996980.1 | DL122917.1 | DL104270.1 | GM628653.1 | DL046207.1 | HV689965.1 | HV708223.1 | HD086809.1 |
| HB386791.1 | HH998694.1 | DL114043.1 | DL104238.1 | GM628621.1 | DL046175.1 | HV689071.1 | HV701067.1 | HD078959.1 |
| HB340003.1 | HH998653.1 | DL113915.1 | DL104206.1 | GM628589.1 | DL046143.1 | HV695819.1 | HV701035.1 | HD077681.1 |
| HA641574.1 | HH998580.1 | DL113883.1 | DL104174.1 | GM628557.1 | DL046111.1 | HV688837.1 | HV695529.1 | HD080464.1 |
| HA641510.1 | HH998525.1 | DL113851.1 | DL104142.1 | GM628525.1 | DL046079.1 | HV600544.1 | HV349482.1 | HD070775.1 |
| HA641443.1 | HH996911.1 | DL109050.1 | DL104110.1 | GM715922.1 | DL041965.1 | HV579862.1 | HV351582.1 | HD070418.1 |
| HA641379.1 | HH996792.1 | DL109018.1 | DL099664.1 | GM662188.1 | DL041933.1 | HV585133.1 | HV341780.1 | HD069971.1 |
| HA641315.1 | HH996755.1 | DL108986.1 | DL099632.1 | GM662156.1 | DL041901.1 | HV585069.1 | HV344000.1 | FW345539.1 |
| HA641173.1 | HH994533.1 | DL099582.1 | DL099600.1 | GM662124.1 | DL037918.1 | HV584996.1 | HV343964.1 | FW345140.1 |
| HA640320.1 | HH977453.1 | DL099550.1 | DL099568.1 | GM655098.1 | DL037886.1 | HV584869.1 | HV343772.1 | FW345020.1 |
| HA638528.1 | HH980621.1 | DL035090.1 | DL099536.1 | GM655066.1 | DL029316.1 | HV579206.1 | HV341055.1 | FW344910.1 |
| HA643886.1 | HH980518.1 | DL035058.1 | DL093526.1 | GM655034.1 | DL029284.1 | HV579087.1 | HV347753.1 | FW344214.1 |
| DM092267.1 | HH980449.1 | DL030711.1 | DL093494.1 | GM647982.1 | DL036055.1 | HV588570.1 | HV344956.1 | FW344035.1 |
| GM638345.1 | HH987322.1 | DL030679.1 | DL093462.1 | GM647950.1 | DL035991.1 | HV578982.1 | HV340052.1 | FW343694.1 |
| GM625338.1 | HH980377.1 | DL026742.1 | DL093430.1 | GM647918.1 | DL035959.1 | HV592462.1 | HV347104.1 | FW343628.1 |
| GM741821.1 | HH980284.1 | DL026710.1 | DL093398.1 | GM647886.1 | DL035927.1 | HV578529.1 | HV344680.1 | FW343564.1 |
| FB506767.1 | HH980182.1 | DL026646.1 | DL093366.1 | GM647854.1 | DL035895.1 | HV578496.1 | HV339962.1 | FW343500.1 |
| GM642980.1 | GN116516.1 | DL026614.1 | DL089751.1 | GM647822.1 | DL031891.1 | HV592299.1 | HV339930.1 | FW342679.1 |
| GM621672.1 | GN116484.1 | DL019499.1 | DL089719.1 | GM641018.1 | DL031859.1 | HV577538.1 | HV342416.1 | HC923237.1 |
| A23375.1 | GN116451.1 | DL019467.1 | DL089687.1 | GM640986.1 | DL031827.1 | HV582307.1 | HV339679.1 | HC921646.1 |
| A23142.1 | GN116419.1 | DL019435.1 | DL089655.1 | GM640922.1 | DL031795.1 | HV582012.1 | HV342352.1 | HC921415.1 |
| A22412.1 | DM075107.1 | DL019403.1 | DL089623.1 | GM640890.1 | DL031763.1 | HV575487.1 | HV342179.1 | HC920491.1 |
| A19907.1 | DM073113.1 | DJ026311.1 | DL089591.1 | GM640858.1 | DL027858.1 | HV586594.1 | HV323121.1 | HD068785.1 |
| A16630.1 | DM064157.1 | DJ026269.1 | DL126143.1 | GM628504.1 | DL027826.1 | HV594618.1 | HV322264.1 | HD067656.1 |
| A01971.1 | DM077415.1 | CS721604.1 | DL126111.1 | GM628472.1 | DL027794.1 | HV586273.1 | HV334422.1 | HD066665.1 |
| A21811.1 | GN089936.1 | CS693340.1 | DL126079.1 | GM628440.1 | DL027762.1 | HV601132.1 | HV325847.1 | HD064773.1 |
| A19566.1 | GN089832.1 | DJ008424.1 | DL126047.1 | GM628408.1 | DL023950.1 | HV585955.1 | HV325712.1 | HD063863.1 |
| A16245.1 | GN082838.1 | DJ008392.1 | DL126015.1 | GM628344.1 | DL023918.1 | HV590023.1 | FW570257.1 | HD053204.1 |
| A18870.1 | GN089173.1 | DJ008360.1 | DL125983.1 | GM628312.1 | DL023886.1 | HV585763.1 | FW568967.1 | HD052977.1 |
| A18667.1 | GN082808.1 | DJ004223.1 | DL125951.1 | GM647796.1 | DL023854.1 | HV585750.1 | FW573469.1 | HD051890.1 |
| A17971.1 | GM709130.1 | DJ003337.1 | DL122759.1 | GM662085.1 | DL023822.1 | HV585702.1 | FW572209.1 | HD048979.1 |
| A15416.1 | GM708999.1 | CS671013.1 | DL122703.1 | GM662053.1 | DL023790.1 | HV585659.1 | FW574379.1 | HD052441.1 |
| A16035.1 | GM755053.1 | CS673618.1 | DL122671.1 | GM662021.1 | DL020775.1 | HV580376.1 | FW568742.1 | HD033862.1 |
| A14307.1 | DL480709.1 | DD464833.1 | DL122639.1 | GM661989.1 | DL020743.1 | HV570608.1 | FW571753.1 | HD033655.1 |
| A13391.1 | GM660126.1 | DD463571.1 | DL122607.1 | GM661957.1 | DL020711.1 | HV568116.1 | HI967431.1 | HD057697.1 |
| A13038.1 | GM645485.1 | CS647390.1 | DL122575.1 | GM661925.1 | DL020679.1 | HV568056.1 | HI964494.1 | HD057665.1 |
| A12446.1 | GM645453.1 | CS646218.1 | DL118385.1 | GM654931.1 | DL020647.1 | FW374988.1 | HI988924.1 | HD062764.1 |
| A11323.1 | GM645421.1 | CS646186.1 | DL118353.1 | GM654899.1 | DL020615.1 | FW369657.1 | HI987431.1 | HD057651.1 |
| A10869.1 | GM638528.1 | DD461456.1 | DL118321.1 | GM654867.1 | DL016212.1 | FW374490.1 | FW552301.1 | HD062025.1 |
| A03751.1 | GM638496.1 | DD459656.1 | DL118289.1 | GM654835.1 | DL016180.1 | FW374390.1 | FW552267.1 | HD061775.1 |
| A08448.1 | GM638464.1 | DD458771.1 | DL118257.1 | GM654803.1 | DL016148.1 | FW379851.1 | FW552230.1 | HD061739.1 |
| A08082.1 | GM633733.1 | HB340022.1 | DL118225.1 | GM654771.1 | DL016116.1 | FW369333.1 | FW562040.1 | M27784.1 |
| A07588.1 | GM633701.1 | HB338849.1 | DL113829.1 | GM647778.1 | DL016084.1 | U25669.1 | FW552169.1 | M33949.1 |
| A06620.1 | FB506771.1 | HA641584.1 | DL113797.1 | GM647746.1 | DL016052.1 | HH736051.1 | FW552134.1 | U46664.1 |
| A05159.1 | GM642984.1 | HA641520.1 | DL113727.1 | GM647714.1 | DL011594.1 | HH735629.1 | FW554627.1 | FW334451.1 |

| | | | | | | | | |
|---|---|---|---|---|---|---|---|---|
| A08316.1 | GM630643.1 | HA641183.1 | DL113757.1 | GM647682.1 | DL011562.1 | HH734152.1 | FW555598.1 | FW340294.1 |
| K02391.1 | GM630611.1 | HA640384.1 | DL113701.1 | GM647650.1 | DL011530.1 | HH733711.1 | FW561700.1 | FW342476.1 |
| M34726.1 | GM630579.1 | HA639658.1 | DL108868.1 | GM640815.1 | DL011498.1 | HD119624.1 | FW561668.1 | FW341660.1 |
| M35058.1 | GM621676.1 | HA638533.1 | DL108836.1 | GM640782.1 | DL011466.1 | FW360401.1 | HI379297.1 | FW336757.1 |
| M12441.1 | GM621644.1 | HA643907.1 | DL108804.1 | GM640750.1 | DL011434.1 | FW360003.1 | HI379107.1 | FW335890.1 |
| M55325.1 | GM621612.1 | DM102620.1 | DL108772.1 | GM640718.1 | DL011402.1 | FW359912.1 | HI378579.1 | HC868228.1 |
| M35458.1 | GM621580.1 | GN368282.1 | DL108740.1 | GM640686.1 | DL031712.1 | FW359766.1 | HI378131.1 | HC868088.1 |
| LC415908.1 | GM657128.1 | GN360072.1 | DL108708.1 | GM640654.1 | DL031680.1 | FW359684.1 | HI376942.1 | HC868035.1 |
| AF069382.1 | GM646773.1 | GN360008.1 | GM712979.1 | GM628300.1 | DL031648.1 | FW359600.1 | HI375924.1 | HC874654.1 |
| AH002297.2 | GM646741.1 | GN359976.1 | GM652759.1 | GM628268.1 | DL031616.1 | FW359527.1 | HI375889.1 | HC867835.1 |
| K01761.1 | GM646709.1 | GN359944.1 | GM652727.1 | GM628236.1 | DL031584.1 | FW368789.1 | HI375008.1 | HC867753.1 |
| HV455600.1 | GM646477.1 | GN359803.1 | GM652695.1 | GM628172.1 | DL031552.1 | FW368691.1 | HI370998.1 | HC867656.1 |
| HV502797.1 | GM646445.1 | GN359707.1 | GM622519.1 | GM628140.1 | DL027743.1 | FW368398.1 | HI370359.1 | DM370478.1 |
| HV502765.1 | GM646413.1 | DL463059.1 | GM622487.1 | GM628104.1 | DL027711.1 | FW368153.1 | HI369109.1 | DM367500.1 |
| HV502733.1 | GM626871.1 | DL462997.1 | GM622455.1 | GM628008.1 | DL027679.1 | FW363206.1 | HI424137.1 | DM205854.1 |
| HV502701.1 | GM626839.1 | DL462897.1 | GM622423.1 | GM627976.1 | DL027647.1 | FW367258.1 | HI424105.1 | DM371255.1 |
| HV502541.1 | GM626807.1 | DL481565.1 | GM622391.1 | GM627944.1 | DL027615.1 | FW362879.1 | HI424073.1 | DM365914.1 |
| HV502509.1 | GM639414.1 | DL467554.1 | GM622359.1 | GM715483.1 | DL027583.1 | FW362716.1 | HI423847.1 | HC003052.1 |
| HV502445.1 | GM639382.1 | GM678827.1 | GM631347.1 | GM661914.1 | DL027551.1 | FW362635.1 | HI423478.1 | HC001921.1 |
| HV452349.1 | GM639350.1 | FB509199.1 | GM631315.1 | GM661882.1 | DL023739.1 | FW349843.1 | HH762722.1 | HB865028.1 |
| HV453671.1 | DL094231.1 | FB509167.1 | GM703825.1 | GM661850.1 | DL023707.1 | FW351519.1 | HI416359.1 | HB864976.1 |
| HV451137.1 | DL094167.1 | GM709131.1 | GM657513.1 | GM661818.1 | DL023675.1 | FW366221.1 | HI415850.1 | HB864988.1 |
| HV450950.1 | DL114118.1 | GM709000.1 | GM657481.1 | GM661786.1 | DL023643.1 | FW351421.1 | HI415785.1 | HB864932.1 |
| FW571120.1 | DL114086.1 | GM694889.1 | GM657449.1 | GM661754.1 | DL023579.1 | FW351311.1 | HI415753.1 | HB864900.1 |
| FW568782.1 | DL114054.1 | GM043993.1 | GM657385.1 | GM661722.1 | DL020532.1 | FW366106.1 | HI414040.1 | HB864868.1 |
| FW570987.1 | DL126226.1 | GM755054.1 | GM657353.1 | GM654728.1 | DL020468.1 | FW361518.1 | HI414003.1 | HB864836.1 |
| HI967448.1 | DL126194.1 | DL480710.1 | GM650161.1 | DL181437.1 | DL020436.1 | FW360590.1 | HI413444.1 | HB864804.1 |
| HI979309.1 | DL126162.1 | FB506772.1 | GM650129.1 | DL181383.1 | DL016001.1 | FW360424.1 | HI516524.1 | HB864772.1 |
| HI987446.1 | DL122946.1 | GM629002.1 | GM650097.1 | DL174593.1 | DL015969.1 | HD122556.1 | HI516083.1 | HB859429.1 |
| FW512096.1 | DL122914.1 | GM047092.1 | GM643193.1 | DL174399.1 | DL015937.1 | HD122220.1 | HI547685.1 | HB866519.1 |
| FW511948.1 | DL104217.1 | GM654814.1 | GM643128.1 | DL176790.1 | DL013792.1 | HD121609.1 | HI284302.1 | HC001062.1 |
| FW554689.1 | DL099547.1 | GM654782.1 | GM643096.1 | FB357049.1 | DL013760.1 | HD115946.1 | HI284270.1 | HC000284.1 |
| HI503521.1 | DL122682.1 | GM647725.1 | GM643064.1 | FB344637.1 | A02591.1 | HD113253.1 | HI574491.1 | HB999515.1 |
| HI516551.1 | DL122650.1 | GM647693.1 | GM643032.1 | FB344289.1 | A06038.1 | HD082378.1 | FW309560.1 | HB999240.1 |
| HI516094.1 | DL122586.1 | GM638809.1 | GM636011.1 | FB360085.1 | A04070.1 | HD081498.1 | FW309164.1 | HB977452.1 |
| HI000213.1 | HI993733.1 | GM638777.1 | GM635979.1 | DL088353.1 | CS444192.1 | HC460535.1 | FW334254.1 | HB976764.1 |
| HI284281.1 | HI989540.1 | GM638745.1 | GM635947.1 | DL088321.1 | CS439051.1 | AY028212.1 | FW333961.1 | HB976205.1 |
| HI574534.1 | HI987669.1 | GM037971.1 | GM635915.1 | DL088289.1 | CS435196.1 | HC456295.1 | FW332558.1 | DM197715.1 |
| HI574470.1 | FW552205.1 | FB766161.1 | GM635883.1 | DL088257.1 | DD334543.1 | HC453721.1 | FW306430.1 | DM196034.1 |
| HI574438.1 | FW552189.1 | FB764684.1 | GM631094.1 | DL088225.1 | DD347369.1 | HC453689.1 | FW332184.1 | DM195107.1 |
| HI574406.1 | FW552150.1 | FB746162.1 | GM622319.1 | DL088193.1 | DD332706.1 | HC453657.1 | HC769917.1 | DM203059.1 |
| HI584650.1 | FW552114.1 | FB743950.1 | GM622287.1 | DL088161.1 | DD345870.1 | HC460199.1 | HC769793.1 | DM194333.1 |
| HI636986.1 | FW561802.1 | FB743917.1 | GM622255.1 | DL088129.1 | DD353621.1 | U78761.1 | HC767341.1 | HB828254.1 |
| HI001416.1 | FW555613.1 | FB743885.1 | GM622223.1 | DL088097.1 | CS430726.1 | HC452252.1 | HC754728.1 | HB805797.1 |
| HI001364.1 | FW555581.1 | FB743853.1 | GM622191.1 | DL088065.1 | CS426731.1 | HC452170.1 | HC733691.1 | HB839914.1 |
| HI001330.1 | FW561683.1 | FB743799.1 | GM622159.1 | DL088033.1 | CS423574.1 | HC452062.1 | HC732145.1 | HB847934.1 |
| HI003213.1 | FW561651.1 | FB742934.1 | GM631018.1 | DL088001.1 | CS418631.1 | HC451432.1 | HC471772.1 | HB847717.1 |

| | | | | | | | | |
|---|---|---|---|---|---|---|---|---|
| HI003166.1 | HI520485.1 | FB708880.1 | GM630986.1 | DL091942.1 | CS419549.1 | HC450955.1 | HC471700.1 | HB839485.1 |
| HI003098.1 | HI568948.1 | GM720577.1 | GM630954.1 | DL091910.1 | CS419062.1 | HC450923.1 | HC471644.1 | HB847466.1 |
| HI003055.1 | HI551900.1 | GM963766.1 | GM630897.1 | DL091878.1 | CS417159.1 | HC450601.1 | HC471612.1 | HB839214.1 |
| HI003015.1 | HI470584.1 | GM890146.1 | GM622115.1 | DL091846.1 | CS416727.1 | HC449542.1 | HC729061.1 | HB838780.1 |
| HI002953.1 | HI551646.1 | GM011263.1 | GM622083.1 | DL091814.1 | CS416227.1 | FU761023.1 | HC728990.1 | HB805289.1 |
| HI002915.1 | HI538375.1 | GM009358.1 | GM622051.1 | DL091782.1 | CS410858.1 | FU760889.1 | HW292751.1 | HB847005.1 |
| HI001281.1 | HI564725.1 | GM008834.1 | GM622019.1 | DL102903.1 | CS410694.1 | FU760038.1 | HW291290.1 | HB838736.1 |
| HI001232.1 | HI564231.1 | GM888367.1 | GM621987.1 | DL102871.1 | CS410579.1 | FU773343.1 | HW291125.1 | HB832059.1 |
| HI001182.1 | HI564109.1 | FB985016.1 | GM621955.1 | DL102839.1 | CS414853.1 | FU759555.1 | HW291070.1 | HB846173.1 |
| HI001110.1 | HI583097.1 | FB983198.1 | GM635854.1 | DL102807.1 | CS414783.1 | FU758617.1 | HW291038.1 | HB815695.1 |
| HI001088.1 | HI563634.1 | FB983178.1 | GM635822.1 | DL102775.1 | DD321003.1 | FU763328.1 | HW290974.1 | HB815017.1 |
| HI001032.1 | HI563602.1 | FB754309.1 | GM635790.1 | DL102743.1 | DD327514.1 | FU762436.1 | HW290942.1 | HB837818.1 |
| HI000998.1 | HI563566.1 | FB727491.1 | GM635758.1 | DL107683.1 | DD323297.1 | FU756335.1 | HW298874.1 | HB808937.1 |
| HI000951.1 | HI508518.1 | FB726887.1 | GM635726.1 | DL107651.1 | DD329909.1 | FU756301.1 | HW290911.1 | HB845706.1 |
| HI002854.1 | HI544243.1 | GM863544.1 | GM635694.1 | DL107619.1 | DD329746.1 | HC438980.1 | HW290879.1 | HB845494.1 |
| HI002822.1 | HI508464.1 | DL022289.1 | GM635662.1 | DL107587.1 | DD326845.1 | HC438526.1 | HW290847.1 | HB837309.1 |
| HI002778.1 | HI000023.1 | DL022257.1 | GM630858.1 | DL107523.1 | DD321766.1 | HC435963.1 | HW290112.1 | HB813993.1 |
| HI002722.1 | HI003663.1 | DL022225.1 | GM630794.1 | DL102713.1 | DD325238.1 | HC358231.1 | HW298194.1 | HB845204.1 |
| HI002677.1 | HI001793.1 | DL025308.1 | GM630762.1 | DL107463.1 | CS389320.1 | HC358199.1 | HW298047.1 | HB844990.1 |
| HI002639.1 | HI001755.1 | DL025212.1 | GM630698.1 | DL107417.1 | CS389287.1 | HC358068.1 | HW289023.1 | HB844565.1 |
| HI002605.1 | HI001718.1 | DL011722.1 | GM621923.1 | DL107423.1 | CS389198.1 | HC357222.1 | HW274665.1 | HB844285.1 |
| HI002554.1 | HI469529.1 | DL011690.1 | GM621891.1 | DL107303.1 | CS389160.1 | HC321181.1 | HW279583.1 | HB836628.1 |
| HI002492.1 | HI544120.1 | DL011658.1 | GM621859.1 | DL123357.1 | CS401291.1 | HC356202.1 | HV961490.1 | HB836380.1 |
| HI004505.1 | HI575293.1 | DL011626.1 | GM621827.1 | DL123325.1 | CS406444.1 | HC325481.1 | HV969518.1 | HB835906.1 |
| HC358390.1 | HI001636.1 | DL044736.1 | GM621795.1 | DL123261.1 | CS406002.1 | A06498.1 | HV960513.1 | HB843175.1 |
| HC358202.1 | HI001594.1 | DL044704.1 | GM621763.1 | DL123229.1 | CS405404.1 | HC324980.1 | HV960202.1 | HB835677.1 |
| HC358071.1 | HI001553.1 | DL032028.1 | GM657324.1 | DL123197.1 | CS408100.1 | HC324509.1 | HV960143.1 | HB819370.1 |
| DL035613.1 | HI003376.1 | DL031996.1 | GM657292.1 | DL123165.1 | CS402204.1 | HC324121.1 | HV964139.1 | HB818777.1 |
| DL035581.1 | HI003338.1 | DL031964.1 | GM657228.1 | DL118911.1 | DD146386.1 | HC323214.1 | HV966038.1 | HB842715.1 |
| DL035549.1 | HI003299.1 | DL028123.1 | GM657196.1 | DL118879.1 | DD115367.1 | HC323146.1 | HV963602.1 | HB806857.1 |
| DL035517.1 | HI464744.1 | DL028091.1 | GM657164.1 | DL118847.1 | DD146289.1 | HC315515.1 | HV958116.1 | HB842138.1 |
| DL031513.1 | HI464712.1 | DL028059.1 | GM649972.1 | DL118815.1 | DD146257.1 | HC320349.1 | HV966012.1 | DM010300.1 |
| DL031481.1 | HI464680.1 | DL028027.1 | GM649940.1 | DL109263.1 | DD146202.1 | HC319653.1 | HV965948.1 | DM008740.1 |
| DL031449.1 | HI464648.1 | DL027995.1 | GM649876.1 | DL109231.1 | DD101527.1 | HC318081.1 | HV969957.1 | GM998435.1 |
| DL031417.1 | HI550134.1 | DL027963.1 | GM649844.1 | DL109199.1 | DD086342.1 | HC316428.1 | HV961714.1 | GM996596.1 |
| DL031385.1 | HI504974.1 | CS601856.1 | GM649812.1 | DL038112.1 | DD069885.1 | HC040795.1 | HV961660.1 | GM980123.1 |
| DL031353.1 | HI462583.1 | CS600963.1 | GM643004.1 | DL033678.1 | DD143276.1 | HC046862.1 | HV951623.1 | GM994900.1 |
| DL027544.1 | HH714031.1 | CS607556.1 | GM642972.1 | DL033646.1 | DD098836.1 | HC046830.1 | HV951591.1 | GM994348.1 |
| DL027512.1 | HH713475.1 | CS607043.1 | GM642939.1 | DL033582.1 | DD084339.1 | HC046798.1 | HV951559.1 | GM978376.1 |
| DL027480.1 | HH961367.1 | CS606951.1 | GM642907.1 | DL033550.1 | DD098191.1 | HC046766.1 | HV955899.1 | GM977876.1 |
| DL027448.1 | HH961335.1 | CS606816.1 | GM642875.1 | DL029510.1 | DD142048.1 | HC046734.1 | HV947322.1 | GM992486.1 |
| DL027416.1 | HH961303.1 | CS604687.1 | GM642843.1 | DL029478.1 | DD132458.1 | HC046702.1 | HV947285.1 | GM992414.1 |
| DL027384.1 | HH961271.1 | CS592361.1 | GM653458.1 | DL029446.1 | DD141292.1 | HC039673.1 | HV953236.1 | GM992206.1 |
| DL027352.1 | HH961239.1 | CS592089.1 | GM653426.1 | DL029382.1 | DD153587.1 | HC046685.1 | HV940440.1 | GM992174.1 |
| DL023540.1 | HH964262.1 | CS589294.1 | GM653394.1 | DL046240.1 | DD158451.1 | GN030561.1 | HV940306.1 | GM991917.1 |
| DL023508.1 | HH974587.1 | CS598042.1 | GM646395.1 | DL046208.1 | DD157783.1 | GN030529.1 | HV939942.1 | GM991753.1 |
| DL023476.1 | HH974530.1 | CS597711.1 | GM646363.1 | DL046176.1 | DD153671.1 | GN030497.1 | HV943483.1 | GM976290.1 |

| | | | | | | | | |
|---|---|---|---|---|---|---|---|---|
| DL023444.1 | HH998099.1 | CS593344.1 | GM646331.1 | DL046144.1 | DD152126.1 | GN030407.1 | HV325951.1 | GM975134.1 |
| DL023412.1 | DM163991.1 | CS585219.1 | GM646299.1 | DL046112.1 | DD082381.1 | GN030273.1 | HV325845.1 | GM990790.1 |
| DL023380.1 | DM163958.1 | HV535610.1 | GM646267.1 | DL046080.1 | DD081763.1 | GN030241.1 | HV331746.1 | GM989738.1 |
| DL020365.1 | DM163575.1 | HV515599.1 | GM646235.1 | DL041966.1 | DD123293.1 | GN030209.1 | HV324502.1 | GM989066.1 |
| DL020333.1 | DM163288.1 | HV515567.1 | GM639432.1 | DL041934.1 | DD148503.1 | GN030081.1 | HV041720.1 | GM986844.1 |
| DL020301.1 | DM161510.1 | HV515439.1 | GM639400.1 | DL041902.1 | DD147796.1 | GN030049.1 | HV120057.1 | GM999533.1 |
| DL020269.1 | HB475832.1 | HV515407.1 | GM639368.1 | DL038079.1 | DD147764.1 | GN030017.1 | HV187246.1 | GM969674.1 |
| DL020237.1 | HB475481.1 | HV515375.1 | GM639336.1 | DL038047.1 | DD147732.1 | GN029953.1 | HV189867.1 | GM969402.1 |
| DL020205.1 | HB475310.1 | HV515343.1 | GM639304.1 | DL038015.1 | DD147700.1 | GN029889.1 | HV192776.1 | GM986690.1 |
| DL015802.1 | HB474895.1 | HV515311.1 | GM639272.1 | DL037983.1 | DD147294.1 | GN029857.1 | HV301794.1 | FB986399.1 |
| DL015770.1 | HB474743.1 | HV515279.1 | GM639240.1 | DL037951.1 | DD081166.1 | GN029823.1 | HV301609.1 | FB986061.1 |
| DL015738.1 | HB469629.1 | HV515247.1 | GM754503.1 | DL037919.1 | DD122268.1 | GN029791.1 | HV302974.1 | GM969702.1 |
| DL015706.1 | HB468757.1 | HV511444.1 | GM660698.1 | DL037887.1 | DD137694.1 | GN014103.1 | HV302942.1 | GM970614.1 |
| DL015674.1 | DM148853.1 | HV514259.1 | GM660666.1 | DL029317.1 | DD137310.1 | GN013385.1 | HV308790.1 | GM970191.1 |
| DL015642.1 | DM152591.1 | HV507844.1 | HI002595.1 | DL029285.1 | DD136445.1 | GN034223.1 | HV309570.1 | FB736415.1 |
| DL011184.1 | DM147634.1 | HV513563.1 | HI002533.1 | DL029253.1 | DD135767.1 | GN010227.1 | HV040128.1 | GM969861.1 |
| DL011152.1 | DL479498.1 | HV510209.1 | HI001486.1 | DL029221.1 | DD135720.1 | GN033557.1 | HV188523.1 | GM969742.1 |
| DL011120.1 | DL464587.1 | HV509917.1 | HI004551.1 | DL029157.1 | DD134023.1 | GN033525.1 | HV200536.1 | FB778024.1 |
| DL011088.1 | DL463055.1 | HV508639.1 | HI004423.1 | DL046045.1 | DD133028.1 | GN033493.1 | HV182489.1 | GM009521.1 |
| DL011056.1 | DL462993.1 | HI401107.1 | HI002451.1 | DL046013.1 | DD132293.1 | GN033461.1 | HV182457.1 | CQ818018.1 |
| DL011024.1 | FB504555.1 | HI637686.1 | HI002400.1 | DL045981.1 | DD080377.1 | GN033397.1 | HV182425.1 | CQ816984.1 |
| DL023373.1 | GM831915.1 | HI380626.1 | HI002320.1 | DL045949.1 | DD107424.1 | GN033365.1 | FZ419475.1 | CQ816944.1 |
| DL047228.1 | GM678545.1 | HI378147.1 | HI000526.1 | DL045917.1 | DD107147.1 | GN033333.1 | FZ413190.1 | CQ815394.1 |
| DL047164.1 | GM709028.1 | HI375906.1 | HI183837.1 | DL045885.1 | DD093918.1 | GN033301.1 | FZ415415.1 | CQ814070.1 |
| DL047132.1 | GM708996.1 | HI369146.1 | HI178213.1 | DL069483.1 | DD093886.1 | GN033269.1 | FZ421422.1 | CQ814037.1 |
| DL047100.1 | GM043989.1 | HI369093.1 | HI202839.1 | DL049719.1 | DD093854.1 | GN033237.1 | FZ415204.1 | CQ814005.1 |
| DL043246.1 | GM637338.1 | HH931936.1 | FW306897.1 | DL049687.1 | DD093639.1 | GN033205.1 | FZ411666.1 | CQ813973.1 |
| DL043214.1 | GM637306.1 | HH827046.1 | FW306469.1 | DL049655.1 | DD092961.1 | GN033173.1 | FZ420738.1 | CQ813941.1 |
| DL043182.1 | GM637274.1 | HH826943.1 | FW306109.1 | DL045711.1 | DD092326.1 | GN033141.1 | FZ416968.1 | CQ813909.1 |
| DL043150.1 | GM658662.1 | HH822193.1 | FW332223.1 | DL045679.1 | DD119265.1 | GN033109.1 | FZ419856.1 | CQ813877.1 |
| DL043118.1 | FB715264.1 | HH821923.1 | HC769919.1 | DL041864.1 | DD061429.1 | GM658506.1 | FZ419821.1 | CQ813845.1 |
| DL043086.1 | FB677559.1 | HH735637.1 | HC769797.1 | DL041832.1 | DD118654.1 | GM658424.1 | FZ419789.1 | CQ813813.1 |
| DL039269.1 | GM061217.1 | HH733726.1 | HC767343.1 | DL041800.1 | DD091312.1 | GM658466.1 | FW580409.1 | CQ813692.1 |
| DL039237.1 | DL256290.1 | FW368415.1 | HC757152.1 | DL041768.1 | DD061339.1 | GM658410.1 | FW581357.1 | CQ813610.1 |
| DL039205.1 | DL241163.1 | FW367495.1 | HC742909.1 | DL041736.1 | DD118414.1 | GM658378.1 | FW590845.1 | CQ807216.1 |
| DL039173.1 | DL240248.1 | FW366230.1 | HC755780.1 | DL041704.1 | DD091164.1 | GM658346.1 | FW590729.1 | CQ807164.1 |
| DL039141.1 | DL236209.1 | FW351373.1 | HC754731.1 | DL037881.1 | DD118111.1 | GM651154.1 | FW589162.1 | CQ807088.1 |
| DL039109.1 | DL233427.1 | FW351211.1 | HC733770.1 | DL037849.1 | AY967071.1 | GM651122.1 | FW588670.1 | CQ806942.1 |
| DL035485.1 | FB713971.1 | HD122564.1 | HC733693.1 | DL037817.1 | AY967039.1 | GM651090.1 | FW593446.1 | CQ802141.1 |
| DL035453.1 | FB712613.1 | HD122320.1 | HC742009.1 | DL037785.1 | AY967007.1 | GM651058.1 | FW586439.1 | CQ801233.1 |
| DL035421.1 | FB708248.1 | HD115787.1 | HC732147.1 | DL037753.1 | AY966975.1 | GM651026.1 | FW592560.1 | CQ799104.1 |
| DL035389.1 | HV208792.1 | HD079724.1 | HC731705.1 | DL037721.1 | AY966943.1 | GM650994.1 | FW577822.1 | CQ796795.1 |
| DL035357.1 | HV245447.1 | HD071236.1 | HC471774.1 | DL037689.1 | CS056310.1 | GM644195.1 | FW577425.1 | CQ795498.1 |
| DL035325.1 | HV214875.1 | FW345565.1 | HC471742.1 | DL049614.1 | CS055983.1 | GM644163.1 | FW576683.1 | CQ795462.1 |
| DL031321.1 | HV227064.1 | FW345226.1 | HC471702.1 | DL049486.1 | CS053082.1 | GM644131.1 | FW576647.1 | CQ793269.1 |
| DL031289.1 | HV302957.1 | FW345028.1 | HC471646.1 | DL049454.1 | CS052393.1 | GM644099.1 | FW575882.1 | CQ792420.1 |
| DL031257.1 | HV312365.1 | FW344996.1 | HC471614.1 | DL041663.1 | CS052358.1 | GM644067.1 | FW379642.1 | CQ790362.1 |

| | | | | | | | | |
|---|---|---|---|---|---|---|---|---|
| DL031225.1 | HV200445.1 | FW343710.1 | HC471535.1 | DL041631.1 | CS052324.1 | GM644035.1 | FW376411.1 | CQ789696.1 |
| DL031161.1 | HH974920.1 | FW343516.1 | HC729191.1 | DL041599.1 | CS052263.1 | GM637005.1 | FW381327.1 | CQ788362.1 |
| DL031129.1 | HH974560.1 | FW343192.1 | HC728705.1 | DL041567.1 | CS050954.1 | GM636973.1 | FW375469.1 | CQ787676.1 |
| DL027320.1 | HH974525.1 | HC923351.1 | HC726062.1 | DL041535.1 | CS048861.1 | GM636941.1 | FW375241.1 | CQ787493.1 |
| DL027288.1 | HH998097.1 | HC922978.1 | HC688444.1 | DL041503.1 | CS047692.1 | GM636909.1 | FW369635.1 | CQ787461.1 |
| DL027256.1 | HH998043.1 | HC486542.1 | HC490974.1 | DL037680.1 | CS047260.1 | GM636877.1 | FW380258.1 | CQ787423.1 |
| DL027224.1 | HH997990.1 | HC483596.1 | HC490942.1 | DL037648.1 | CS039315.1 | GM632469.1 | HH736006.1 | CQ787385.1 |
| DL027192.1 | HH999677.1 | HC474607.1 | HC490910.1 | DL037616.1 | CS039283.1 | GM632437.1 | HH735914.1 | CQ787349.1 |
| DL027160.1 | HH997874.1 | HC491777.1 | HC490878.1 | DL037584.1 | CS039251.1 | GM632405.1 | HH733943.1 | CQ787313.1 |
| DL023348.1 | HH997799.1 | HC491745.1 | HC490838.1 | DL037552.1 | CS039219.1 | GM632373.1 | HH733486.1 | CQ787281.1 |
| DL023316.1 | HH997754.1 | HC491629.1 | HC490806.1 | DL037520.1 | CS038874.1 | GM632341.1 | FW368811.1 | CQ787249.1 |
| DL023284.1 | HH999480.1 | HC488307.1 | HC490774.1 | DL033285.1 | CS037901.1 | GM632309.1 | DL026980.1 | CQ786955.1 |
| DD462120.1 | HH999435.1 | FV533460.1 | HC490742.1 | DL033253.1 | CS037243.1 | GM623538.1 | DL026953.1 | CQ785943.1 |
| DD460836.1 | HH999393.1 | FV531700.1 | HC490710.1 | DL033221.1 | CS025552.1 | GM623506.1 | DL030907.1 | CQ784677.1 |
| DD460546.1 | HH997633.1 | FV522838.1 | HC490678.1 | DL020204.1 | CS023856.1 | GM623474.1 | DL030868.1 | CQ778920.1 |
| DD459907.1 | HH997576.1 | FV522798.1 | HC490646.1 | DL015801.1 | CS023749.1 | GM623442.1 | DL030836.1 | CQ778511.1 |
| DD455197.1 | HH997501.1 | HC465614.1 | HC490582.1 | DL015769.1 | CS022580.1 | GM623410.1 | DL030804.1 | CQ774635.1 |
| DD455165.1 | HH997362.1 | HC461779.1 | HC490549.1 | DL015737.1 | CS017173.1 | GM623378.1 | DL030772.1 | CQ771665.1 |
| DD458757.1 | HH999183.1 | AY205157.1 | HC490514.1 | DL015705.1 | CS016845.1 | GM623346.1 | DL030740.1 | CQ771633.1 |
| DD457902.1 | HH999138.1 | HC453697.1 | HC490482.1 | DL015673.1 | CS016589.1 | GM643994.1 | DL026771.1 | CQ770996.1 |
| DD452808.1 | HH999107.1 | HC453665.1 | HC490411.1 | DL011183.1 | CS016557.1 | GM643962.1 | DL019752.1 | CQ768901.1 |
| DD456328.1 | HH999058.1 | AF050524.1 | HC490283.1 | DL011151.1 | CS016480.1 | GM643930.1 | DL019720.1 | CQ768054.1 |
| DD456095.1 | HH998950.1 | HB856264.1 | HC490218.1 | DL011119.1 | CS016121.1 | GM643897.1 | CS792475.1 | CQ766073.1 |
| CS631735.1 | HH997230.1 | HB855965.1 | HC472304.1 | DL011087.1 | CS007947.1 | GM643865.1 | CS791970.1 | CQ765975.1 |
| CS642224.1 | HH997159.1 | HB855726.1 | HC472239.1 | DL011055.1 | CQ986639.1 | GM643833.1 | DJ044927.1 | CQ760564.1 |
| CS631232.1 | HH978153.1 | DM170838.1 | HC472207.1 | DL011023.1 | AX598879.1 | GM636836.1 | DJ044886.1 | CQ757807.1 |
| CS631168.1 | HH998902.1 | DM170806.1 | HB837183.1 | DL047227.1 | AX593879.1 | GM636804.1 | DJ028181.1 | AX088761.1 |
| CS630936.1 | HH998844.1 | DM187952.1 | HB813981.1 | DL047163.1 | AX593401.1 | GM636772.1 | DJ030383.1 | AX088384.1 |
| CS627793.1 | HH998793.1 | DM187854.1 | HB845192.1 | CS603529.1 | AX587871.1 | GM636740.1 | DJ029984.1 | AX085506.1 |
| CS632852.1 | HH998734.1 | HB491548.1 | HB844934.1 | CS603465.1 | AX587774.1 | GM636708.1 | DJ026323.1 | AX084331.1 |
| CS632820.1 | HH997079.1 | HB489348.1 | HB844481.1 | CS603433.1 | AX591044.1 | GM636676.1 | CS691346.1 | AX082494.1 |
| CS632619.1 | HH996971.1 | HB488931.1 | HB844273.1 | CS603401.1 | AX589085.1 | GM632268.1 | CS693538.1 | AX081675.1 |
| CS627083.1 | HH998637.1 | DM163992.1 | HB836569.1 | CS603369.1 | BD161161.1 | GM632236.1 | CS675317.1 | AX081156.1 |
| HV704622.1 | HH998574.1 | DM163959.1 | HB843805.1 | CS603337.1 | AX587995.1 | GM632204.1 | CS674190.1 | AX080801.1 |
| HV704455.1 | HH998520.1 | DM163576.1 | HB835830.1 | CS603305.1 | AX587962.1 | GM632172.1 | CS691852.1 | AX078377.1 |
| HV704423.1 | HH996906.1 | DM163289.1 | HB843123.1 | CS603273.1 | AX587868.1 | GM632140.1 | DJ012073.1 | AX077301.1 |
| HV744625.1 | HH996785.1 | DM161511.1 | HB835665.1 | CS603241.1 | AX587821.1 | GM632108.1 | DJ008399.1 | AX076908.1 |
| HV703546.1 | HH996750.1 | HB486323.1 | HB819131.1 | CS603209.1 | AX587768.1 | GM623337.1 | DJ008367.1 | AX076534.1 |
| HV703144.1 | HH980705.1 | GN112642.1 | HB835176.1 | CS603177.1 | AX587715.1 | GM623305.1 | DJ008047.1 | AX074253.1 |
| HV702480.1 | HH980616.1 | DM065120.1 | HB842574.1 | CS603145.1 | AX587665.1 | GM623273.1 | DJ004230.1 | AX073936.1 |
| HV702376.1 | HH980512.1 | DM064155.1 | HB806798.1 | CS603113.1 | AX587614.1 | GM623241.1 | CS671225.1 | AJ296083.1 |
| HV702266.1 | HH980444.1 | DM077413.1 | HB842032.1 | CS603081.1 | AX573572.1 | GM623209.1 | CS671105.1 | AX068436.1 |
| HV702234.1 | HH980371.1 | GN091495.1 | HB841731.1 | CS603049.1 | AX556830.1 | GM623145.1 | CS669944.1 | AX061398.1 |
| HV701222.1 | HH980339.1 | GN090810.1 | HB806655.1 | CS603017.1 | AX555815.1 | GM741847.1 | DD468055.1 | AX060093.1 |
| HV701190.1 | HH980254.1 | GN089894.1 | HB840909.1 | CS602983.1 | AX555567.1 | GM741815.1 | CS646225.1 | AX058573.1 |
| HV701158.1 | HH980175.1 | GN089828.1 | HB840364.1 | CS602951.1 | AX555089.1 | GM658329.1 | CS646193.1 | AX057305.1 |
| HV701126.1 | HH980139.1 | GN082909.1 | HB649982.1 | CS602919.1 | AX552003.1 | GM658297.1 | CS644241.1 | AX052925.1 |

| | | | | | | | | |
|---|---|---|---|---|---|---|---|---|
| HV708783.1 | HH979898.1 | GM652492.1 | HB645747.1 | CS602887.1 | AX551096.1 | GM658265.1 | DD462046.1 | AX050411.1 |
| HV708530.1 | HH996663.1 | GM712195.1 | HB645715.1 | CS602855.1 | AX543890.1 | GM658233.1 | DD460507.1 | AX047760.1 |
| HV708480.1 | HH996623.1 | GM659688.1 | HB645683.1 | CS602823.1 | AX539577.1 | GM658201.1 | DD460141.1 | AX047569.1 |
| HV708193.1 | HH996561.1 | GM651908.1 | HB856015.1 | CS602791.1 | AX538728.1 | GM658169.1 | DD455447.1 | AX046263.1 |
| HV701107.1 | HH998476.1 | GM633294.1 | HB855905.1 | CS602759.1 | AX538329.1 | GM650848.1 | CS183169.1 | AX043879.1 |
| HV701075.1 | HH998380.1 | GM625340.1 | HB855431.1 | CS602727.1 | AX538271.1 | GM650816.1 | CS174652.1 | AX041928.1 |
| HV701043.1 | HH998347.1 | GM625179.1 | DM179542.1 | CS602695.1 | AX537261.1 | GM650784.1 | CS176622.1 | AX040465.1 |
| HV695394.1 | HH999918.1 | GM625147.1 | DM179152.1 | CS602663.1 | AX528955.1 | GM658119.1 | CS172540.1 | AX039593.1 |
| HV694818.1 | HH979836.1 | DJ327061.1 | DM178817.1 | CS602567.1 | AX528119.1 | GM658055.1 | CS172231.1 | AX039184.1 |
| HV694786.1 | HH979768.1 | DJ327029.1 | DM170824.1 | CS274952.1 | AX526830.1 | GM658023.1 | CS166437.1 | AX037565.1 |
| HV700739.1 | HH979686.1 | DJ326997.1 | DM188042.1 | CS272427.1 | AX524913.1 | GM657991.1 | CS159797.1 | AX037303.1 |
| HV700675.1 | HH999885.1 | DJ127215.1 | DM187977.1 | CS260201.1 | AX523923.1 | GM657959.1 | CS144310.1 | AX036653.1 |
| HV694687.1 | HH999791.1 | EU020108.1 | DM169853.1 | CS257323.1 | AX523714.1 | GM650767.1 | CS141736.1 | AX034873.1 |
| HV694548.1 | HH999739.1 | DJ082653.1 | DM173720.1 | CS256707.1 | AX521948.1 | GM650735.1 | CS141569.1 | AX033490.1 |
| HV694225.1 | HH998309.1 | DJ080509.1 | HB491794.1 | CS255242.1 | AX179443.1 | GM650703.1 | CS141523.1 | AX030193.1 |
| HV700232.1 | HH998260.1 | DJ080043.1 | HB491462.1 | CS253593.1 | AX076855.1 | GM650671.1 | CS141491.1 | AX028772.1 |
| HV693643.1 | HH998223.1 | DJ071360.1 | HB489247.1 | CS253401.1 | AX027999.1 | GM650639.1 | CS140822.1 | AX027728.1 |
| HV699750.1 | HH998176.1 | DJ066362.1 | HB488824.1 | CS253949.1 | A09552.1 | GM650607.1 | CS140530.1 | AX027335.1 |
| HV699510.1 | HH996535.1 | DJ066330.1 | HB488688.1 | CS254104.1 | AX521546.1 | GM643807.1 | CS138832.1 | AX024950.1 |
| HV699163.1 | HH993643.1 | DJ066296.1 | DM165689.1 | CS254072.1 | AX521514.1 | GM643775.1 | CS123427.1 | AX023661.1 |
| HV698558.1 | HH986617.1 | DJ066262.1 | AX145732.1 | CS253988.1 | AX513507.1 | GM643743.1 | CS122439.1 | AX023627.1 |
| HV343628.1 | HH979571.1 | DJ065244.1 | AX145700.1 | CS250654.1 | AF430200.1 | GM643711.1 | CS106075.1 | AX023593.1 |
| HV343484.1 | GN029862.1 | DJ055682.1 | AX145668.1 | CS244970.1 | AF430168.1 | GM643679.1 | CS179724.1 | AX020987.1 |
| HV340907.1 | GN013580.1 | DJ061658.1 | AX145636.1 | CS244172.1 | AX511467.1 | GM643639.1 | CS179476.1 | AX019191.1 |
| HV347867.1 | GN033530.1 | DJ061610.1 | AX145604.1 | CS244252.1 | AX511245.1 | GM636650.1 | CS326344.1 | AX018600.1 |
| HV340622.1 | GN033498.1 | DJ061562.1 | AX145572.1 | CS244220.1 | AX505225.1 | GM636618.1 | CS323672.1 | DL125041.1 |
| HV347609.1 | GN033466.1 | DJ054748.1 | HW260290.1 | CS244188.1 | AX505161.1 | GM636586.1 | CS323590.1 | DL125009.1 |
| HV344992.1 | GN033402.1 | DJ054331.1 | HW260258.1 | CS243280.1 | AX496841.1 | GM636522.1 | CS322839.1 | DL124977.1 |
| HV347393.1 | GN033370.1 | FB291786.1 | HW260226.1 | CS243073.1 | AX142243.1 | GM654696.1 | CS330141.1 | DL120984.1 |
| HV344812.1 | GN033338.1 | CS810551.1 | HW260194.1 | CS239629.1 | AX142115.1 | GM654664.1 | CS329663.1 | DL120952.1 |
| HV347323.1 | GN033242.1 | FB292513.1 | HW260162.1 | CS239083.1 | AX141985.1 | GM654632.1 | CS322797.1 | DL120920.1 |
| HV340060.1 | GN033210.1 | FB292271.1 | HW260130.1 | CS228648.1 | AX141921.1 | GM654600.1 | DD271408.1 | DL120888.1 |
| HV340028.1 | GN033082.1 | CS811178.1 | HW260098.1 | CS228081.1 | AX141727.1 | GM654567.1 | DD272639.1 | DL120856.1 |
| HV346980.1 | GN033050.1 | DJ019848.1 | HW260066.1 | CS227877.1 | AX141599.1 | GM647574.1 | CS302570.1 | DL120824.1 |
| HV339970.1 | GN032986.1 | DD017629.1 | HW260034.1 | CS200887.1 | AX141407.1 | GM640611.1 | CS299497.1 | DL124930.1 |
| HV335690.1 | GN032922.1 | BD434176.1 | HW260002.1 | CS227288.1 | AX135827.1 | GM640579.1 | CS102654.1 | DL124898.1 |
| HV344192.1 | GN032890.1 | BD454142.1 | HW259970.1 | CS226736.1 | AX135074.1 | GM640547.1 | CS102622.1 | DL124866.1 |
| HV341875.1 | GN032858.1 | BD434158.1 | HW259938.1 | CS208972.1 | AX133527.1 | GM640482.1 | CS102590.1 | DL124834.1 |
| HV322456.1 | GN032826.1 | BD453912.1 | HW259742.1 | CS208935.1 | AX127704.1 | GM640450.1 | CS102558.1 | DL124802.1 |
| HV321876.1 | GN032794.1 | BD453879.1 | HW259463.1 | CS208871.1 | AX127186.1 | GM627903.1 | CS102526.1 | DL124770.1 |
| HV321460.1 | GN032699.1 | BD453847.1 | HW259273.1 | CS208839.1 | AX113585.1 | GM627871.1 | CS102494.1 | DL120777.1 |
| HV333166.1 | GN032667.1 | BD453815.1 | HW258973.1 | CS203554.1 | AX111978.1 | GM627839.1 | CS101486.1 | DL120745.1 |
| HV325402.1 | GN032634.1 | BD453783.1 | HW258906.1 | CS207927.1 | GM639231.1 | GM627807.1 | CS089036.1 | DL116214.1 |
| HV331833.1 | GN032570.1 | BD453759.1 | HW258841.1 | CS186212.1 | GM639199.1 | GM627775.1 | CS080790.1 | DL116182.1 |
| HV325226.1 | GN032539.1 | BD453727.1 | HW257389.1 | CS182523.1 | GM639167.1 | GM627743.1 | CS070407.1 | DL116150.1 |
| HV324785.1 | GN032475.1 | BD453695.1 | HW257357.1 | CS177755.1 | GM639135.1 | GM640430.1 | CS063833.1 | DL116118.1 |
| HV324287.1 | GN032443.1 | BD453663.1 | HW257325.1 | CS174631.1 | GM639103.1 | GM661693.1 | A08215.1 | DL116086.1 |

| | | | | | | | | |
|---|---|---|---|---|---|---|---|---|
| HV313401.1 | HI002855.1 | BD453631.1 | HW257133.1 | CS176091.1 | GM639071.1 | GM661661.1 | CS060522.1 | DL116054.1 |
| HV313308.1 | HI002823.1 | BD453599.1 | HW256813.1 | CS172441.1 | GM626524.1 | GM661629.1 | AY967380.1 | DL143518.1 |
| HV111948.1 | HI002785.1 | BD493363.1 | HW256781.1 | CS172290.1 | GM626492.1 | GM661597.1 | AY967348.1 | DL120578.1 |
| HV111875.1 | HI002725.1 | BD453549.1 | HW251065.1 | CS173160.1 | GM626428.1 | GM661565.1 | AY967316.1 | DL120546.1 |
| HV041735.1 | HI002678.1 | BD453517.1 | HW250602.1 | CS165435.1 | GM626396.1 | GM661533.1 | AY967284.1 | DL116015.1 |
| HV120189.1 | HI002640.1 | BD453485.1 | HW250265.1 | CS159809.1 | GM626364.1 | GM654539.1 | AY967252.1 | DL115983.1 |
| HV187351.1 | HI002606.1 | BD453453.1 | HW249649.1 | CS159777.1 | GM626332.1 | GM654507.1 | AY967220.1 | DL115887.1 |
| HV189734.1 | HI002555.1 | BD453421.1 | HW247843.1 | CS159588.1 | GM835306.1 | GM654475.1 | AY967188.1 | DL115855.1 |
| HV218625.1 | HI002493.1 | BD453389.1 | HW247803.1 | CS159214.1 | GM660517.1 | GM654443.1 | AY967156.1 | DL106465.1 |
| HV208786.1 | HI004507.1 | BD453244.1 | HW240965.1 | CS148784.1 | GM660357.1 | GM654411.1 | AY967124.1 | DL106355.1 |
| HV218466.1 | HI002333.1 | BD453212.1 | HW240805.1 | CS145754.1 | GM653170.1 | GM654379.1 | AY967092.1 | DL106393.1 |
| HV245434.1 | HI002292.1 | BD497807.1 | HW240741.1 | CS144366.1 | GM653138.1 | GM647386.1 | AY967060.1 | DL106305.1 |
| HH757731.1 | HI066293.1 | BD450235.1 | HW240653.1 | CS142501.1 | GM653106.1 | GM647354.1 | AY967028.1 | DL136025.1 |
| HH757699.1 | HI180647.1 | BD408016.1 | HW241318.1 | CS141937.1 | GM653074.1 | GM647322.1 | AY966996.1 | DL128513.1 |
| HH757667.1 | HI179972.1 | BD438981.1 | HW239396.1 | CS141592.1 | GM653042.1 | GM647290.1 | AY966964.1 | DL128444.1 |
| HH757635.1 | HI202817.1 | BD437676.1 | HW238757.1 | CS141535.1 | GM652978.1 | GM647258.1 | AY966932.1 | DL124750.1 |
| HH757603.1 | HI214577.1 | BD437644.1 | HW238493.1 | CS141503.1 | GM645979.1 | GM647226.1 | CS057774.1 | DL124718.1 |
| HH757571.1 | HI214545.1 | BD394872.1 | HW242960.1 | CS141221.1 | GM645947.1 | GM640423.1 | CQ898684.1 | DL124686.1 |
| HH757539.1 | HI214508.1 | BD453363.1 | HW237896.1 | CS140737.1 | GM645915.1 | GM640391.1 | CQ898611.1 | DL124654.1 |
| HH757507.1 | HI179804.1 | BD453341.1 | HW237857.1 | DD200041.1 | GM645883.1 | GM640359.1 | CQ898579.1 | DL124622.1 |
| HH757475.1 | HI214447.1 | BD453300.1 | HW248697.1 | DD187298.1 | GM645851.1 | GM627716.1 | CQ898504.1 | DL124590.1 |
| HH757443.1 | HI213797.1 | BD453268.1 | HW242507.1 | DD206897.1 | GM645819.1 | GM627684.1 | CQ895553.1 | DL124558.1 |
| HH756691.1 | HI185483.1 | BD413668.1 | HV508592.1 | DD206865.1 | GM639016.1 | GM627652.1 | CQ894668.1 | DL096925.1 |
| HH756256.1 | HI212980.1 | AX954792.1 | HV508560.1 | DD206833.1 | GM638952.1 | GM870286.1 | CQ893664.1 | DL096893.1 |
| HI401635.1 | HI546332.1 | AX938917.1 | HV508528.1 | DD206803.1 | GM638888.1 | GM627592.1 | CQ890963.1 | DL096861.1 |
| HI401246.1 | HI546300.1 | AX937438.1 | HV508502.1 | DD212663.1 | GM638856.1 | GM627528.1 | CQ888119.1 | DL096829.1 |
| HI401099.1 | HI211516.1 | AX927079.1 | HV508468.1 | CS236400.1 | GM626305.1 | GM661501.1 | CQ879729.1 | DL096797.1 |
| HD118337.1 | HI210978.1 | AX923419.1 | HV512392.1 | DD163499.1 | GM626273.1 | GM661469.1 | CQ877134.1 | DL096765.1 |
| HI645153.1 | HI210946.1 | AX923386.1 | HV512270.1 | DD181032.1 | GM626241.1 | GM661437.1 | CQ875532.1 | DL094905.1 |
| HI642725.1 | HI570369.1 | AX840600.1 | HV512238.1 | DD163309.1 | GM626209.1 | GM661405.1 | CQ874250.1 | DL094873.1 |
| HI642181.1 | HI472919.1 | AX839011.1 | HV512206.1 | DD163196.1 | GM626177.1 | GM661373.1 | CQ871412.1 | DL094841.1 |
| HI641637.1 | HC453661.1 | AX832724.1 | HV512174.1 | DD167388.1 | GM626145.1 | GM661341.1 | CQ871218.1 | DL094777.1 |
| HI641093.1 | AF013602.1 | AX824350.1 | HV512142.1 | DD173111.1 | GM660312.1 | GM654347.1 | CQ869290.1 | DL091162.1 |
| HI640549.1 | HC449470.1 | AX823784.1 | HV511831.1 | DD178496.1 | GM660280.1 | GM654315.1 | CQ868900.1 | DL091130.1 |
| HI639897.1 | FU760984.1 | AX823749.1 | HV504950.1 | DL105851.1 | GM660248.1 | GM654283.1 | CQ859640.1 | DL091098.1 |
| HI637818.1 | FU759904.1 | AX814420.1 | HV504918.1 | DL105819.1 | GM660216.1 | GM654219.1 | CQ859608.1 | DL091066.1 |
| HI637632.1 | FU758383.1 | AX812735.1 | HV504886.1 | DL105723.1 | GM660184.1 | GM654187.1 | CQ858156.1 | DL091034.1 |
| HI379490.1 | FU767871.1 | AX805768.1 | HV504854.1 | DL101014.1 | GM660152.1 | GM647194.1 | CQ858055.1 | DL090970.1 |
| HI378107.1 | HC358391.1 | AX798939.1 | HV504822.1 | DL100982.1 | GM652946.1 | GM647162.1 | CQ855922.1 | DL087098.1 |
| HC089332.1 | HC358203.1 | AX798479.1 | HV507452.1 | DL100950.1 | GM652914.1 | GM647130.1 | CQ855139.1 | DL087066.1 |
| HC089300.1 | HC358072.1 | AX798062.1 | HV504773.1 | DL096536.1 | GM652882.1 | GM647098.1 | CQ849475.1 | DL087034.1 |
| HC089268.1 | HC321209.1 | AX797334.1 | HV504741.1 | DL096504.1 | GM652850.1 | GM647033.1 | CQ840187.1 | DL087002.1 |
| HC086569.1 | HC325499.1 | AX796950.1 | HV504709.1 | DL096472.1 | GM652818.1 | GM647058.1 | CQ828073.1 | DL080912.1 |
| HC086283.1 | HC325087.1 | BD194471.1 | HV504677.1 | DL094516.1 | GM652785.1 | GM647005.1 | AX241907.1 | DL076140.1 |
| HC085673.1 | HC325025.1 | BD187477.1 | HV504645.1 | DL094484.1 | GM645787.1 | GM640135.1 | AX241875.1 | DL080829.1 |
| AY774963.1 | HC314444.1 | AX787394.1 | HV504613.1 | DL110845.1 | GM645755.1 | GM640103.1 | AX241843.1 | DL080513.1 |
| AY774866.1 | HC309082.1 | A28463.1 | HV504594.1 | DL110813.1 | GM645723.1 | GM640071.1 | AX241747.1 | DL075905.1 |

| | | | | | | | | |
|---|---|---|---|---|---|---|---|---|
| AY774746.1 | HC307881.1 | A21549.1 | HV504430.1 | DL110781.1 | GM645691.1 | GM640039.1 | AX241683.1 | DL075873.1 |
| AY774694.1 | HC312270.1 | A19569.1 | HV504206.1 | DL110749.1 | GM645659.1 | GM627492.1 | AX241651.1 | DL075846.1 |
| AY774634.1 | HB468030.1 | A17279.1 | HV455590.1 | DL110717.1 | FB660055.1 | GM627460.1 | AX241619.1 | DL075814.1 |
| AY774517.1 | HB475475.1 | A15506.1 | HV504170.1 | DL090747.1 | FB654437.1 | GM627428.1 | AX241587.1 | DL075769.1 |
| AY774469.1 | DM152588.1 | A12583.1 | HV504138.1 | DL090715.1 | FB654127.1 | GM627364.1 | AX241555.1 | DL079945.1 |
| AY774408.1 | DM156352.1 | A11050.1 | HV504106.1 | DL090683.1 | FB654882.1 | GM627332.1 | AX241523.1 | DL074582.1 |
| AY774349.1 | HB460607.1 | A07742.1 | HV504074.1 | DL090651.1 | FB582056.1 | GM661314.1 | AX241491.1 | DL074550.1 |
| AY774301.1 | HB451953.1 | A28931.1 | HV504042.1 | DL090619.1 | FB573201.1 | GM661266.1 | AX241459.1 | DL079055.1 |
| AY774247.1 | HB463481.1 | A28797.1 | HV504010.1 | DL090587.1 | FB580231.1 | GM661234.1 | AX241427.1 | DL078971.1 |
| AY774129.1 | HB444718.1 | A22631.1 | HV503978.1 | DL086582.1 | FB580192.1 | CS119744.1 | AX241120.1 | DL078939.1 |
| AY774061.1 | DM139908.1 | A27356.1 | HV503946.1 | DL086550.1 | FB580160.1 | CS119486.1 | AX235764.1 | DL078908.1 |
| AY773995.1 | DM138038.1 | A26529.1 | HV503914.1 | DL086518.1 | FB580128.1 | CS119452.1 | AX223908.1 | DL073613.1 |
| HA641494.1 | DM137795.1 | A26047.1 | HV503882.1 | DL086454.1 | FB580087.1 | CS119418.1 | AX207277.1 | DL072409.1 |
| HA641235.1 | GN091351.1 | A23312.1 | HV503850.1 | DL086422.1 | DL199994.1 | CS119385.1 | AX205119.1 | DL072288.1 |
| HA641157.1 | GN090906.1 | DM187582.1 | HV503818.1 | DL115524.1 | DL189556.1 | CS119353.1 | AX202535.1 | DL072235.1 |
| HA639883.1 | GN090774.1 | DM177087.1 | HV503802.1 | DL115492.1 | DL199825.1 | CS119255.1 | AX194356.1 | DL071963.1 |
| DM106882.1 | GN089885.1 | HB559417.1 | HV491221.1 | DL115460.1 | DL194198.1 | CS119223.1 | AX190496.1 | DL013613.1 |
| DM106850.1 | GN089820.1 | DM164034.1 | HV503778.1 | DL115435.1 | DL188829.1 | CS119191.1 | AX188574.1 | DL013581.1 |
| DM102732.1 | GN082905.1 | DM164019.1 | HV503746.1 | DL115403.1 | DL196370.1 | CS119159.1 | AX180270.1 | DL013549.1 |
| DM092259.1 | GN080175.1 | DM163986.1 | HV503714.1 | DL115371.1 | DL193834.1 | CS119127.1 | AX173118.1 | DL013517.1 |
| DM105628.1 | GN082834.1 | DM163952.1 | HV503494.1 | DL115339.1 | DL193802.1 | CS119093.1 | AX172473.1 | DL013485.1 |
| DM091597.1 | GN082358.1 | DM162221.1 | HV503462.1 | DL110641.1 | DL193701.1 | CS119060.1 | AX167244.1 | DL013453.1 |
| DM095103.1 | L09142.1 | HB483691.1 | HV503430.1 | DL110609.1 | DL183244.1 | CS119027.1 | AX167084.1 | DL013421.1 |
| DM094908.1 | L08921.1 | GN045777.1 | HV503398.1 | DL110577.1 | FB513472.1 | CS118994.1 | AX166290.1 | DL013389.1 |
| DM093341.1 | L08914.1 | GN044854.1 | HV503366.1 | DL109580.1 | FB571335.1 | CQ757775.1 | AX155096.1 | DL018080.1 |
| DM107357.1 | DM058864.1 | GN042967.1 | HV503334.1 | DL109548.1 | FB571846.1 | CQ757743.1 | AX154558.1 | DL018016.1 |
| GN374381.1 | DM058804.1 | GN041962.1 | AX144640.1 | DL109516.1 | CS696203.1 | CQ757711.1 | AX150969.1 | DL013233.1 |
| GN367592.1 | DM045544.1 | GN030755.1 | AX144539.1 | DL109484.1 | CS696139.1 | CQ756682.1 | AX146312.1 | DL013201.1 |
| GN367552.1 | DM056790.1 | GN030691.1 | AX144411.1 | DL104878.1 | CS696075.1 | CQ755548.1 | AX145725.1 | DL017990.1 |
| GN370764.1 | DM045296.1 | GN030627.1 | AX144347.1 | DL104846.1 | CS696043.1 | CQ755283.1 | AX145693.1 | DL017958.1 |
| GN369739.1 | DM045024.1 | GN030595.1 | AX144217.1 | DL104814.1 | CS696010.1 | CQ754047.1 | AX145629.1 | DL017926.1 |
| GN360562.1 | DM044729.1 | GN030563.1 | AX144025.1 | DL095923.1 | CS695978.1 | CQ654955.1 | AX145597.1 | GM629312.1 |
| GN360164.1 | GN067935.1 | GN030531.1 | AX143959.1 | DL095891.1 | CS695946.1 | CQ654825.1 | AX145565.1 | GM634268.1 |
| GN360132.1 | GN067871.1 | GN030499.1 | AX143831.1 | DL095859.1 | CS695882.1 | CQ654531.1 | AX145533.1 | GM655600.1 |
| GN356181.1 | DM025162.1 | GN030444.1 | AX143703.1 | DL095827.1 | CS695722.1 | CQ654394.1 | AX145501.1 | GM655568.1 |
| GN366660.1 | GM619889.1 | GN030275.1 | AX143639.1 | DL095795.1 | CS695690.1 | CQ654294.1 | AX145469.1 | GM648575.1 |
| GN366511.1 | GM716207.1 | GN030243.1 | AX143575.1 | DL094140.1 | CS695658.1 | CQ654205.1 | AX145437.1 | GM648543.1 |
| GN366479.1 | GM648180.1 | GN030211.1 | AX143447.1 | DL094108.1 | CS695562.1 | CQ654111.1 | AX145405.1 | GM648511.1 |
| GN366447.1 | CS728564.1 | GN029859.1 | AX143383.1 | DL094076.1 | CS695530.1 | CQ654017.1 | AX145372.1 | GM648479.1 |
| GN366414.1 | CS727257.1 | GN033559.1 | AX143255.1 | DL094044.1 | CS695402.1 | AX963157.1 | AX145340.1 | GM648447.1 |
| GN366382.1 | FB709004.1 | GN033527.1 | AX143063.1 | DL094012.1 | DL091612.1 | AX961577.1 | AX145308.1 | GM648415.1 |
| GN366350.1 | FB715320.1 | GN033495.1 | AX142935.1 | DL093980.1 | DL091580.1 | AX960377.1 | AX145276.1 | GM641612.1 |
| GN360059.1 | GM890241.1 | GN033463.1 | AX142871.1 | DL090269.1 | DL087969.1 | AX958672.1 | AX145244.1 | GM634264.1 |
| GN360027.1 | GM887803.1 | GN033335.1 | AX142807.1 | DL090237.1 | DL087937.1 | AX957835.1 | AX145212.1 | GM629267.1 |
| GN359899.1 | GM869162.1 | GN033239.1 | AX142485.1 | DL090205.1 | DL087905.1 | BD010844.1 | AX145180.1 | GM629235.1 |
| GN366325.1 | GM773636.1 | GN033175.1 | AX142421.1 | DL090173.1 | DL087873.1 | AX958022.1 | AX145148.1 | GM629203.1 |
| GN366293.1 | GM879302.1 | GN033047.1 | AX142357.1 | DL086168.1 | DL087841.1 | AX952274.1 | AX145116.1 | GM629139.1 |

| | | | | | | | | |
|---|---|---|---|---|---|---|---|---|
| GN359822.1 | GM721237.1 | GN032983.1 | AX142293.1 | DL086136.1 | DL087809.1 | AX938907.1 | AX145084.1 | GM629107.1 |
| GN359790.1 | GM603726.1 | GN032951.1 | AX142165.1 | DL086104.1 | DL105393.1 | AX937434.1 | AX145052.1 | GM634069.1 |
| GN359726.1 | GM600702.1 | GN032887.1 | AX142101.1 | DL086072.1 | DL105361.1 | AX923415.1 | AX145020.1 | GM634037.1 |
| GN359694.1 | FB715256.1 | GN032855.1 | AX141971.1 | DL086040.1 | DL105329.1 | AX923382.1 | AX144988.1 | GM634005.1 |
| GN359674.1 | GM061169.1 | GN032823.1 | AX141841.1 | DL086008.1 | DL124348.1 | AX840801.1 | AX144956.1 | GM633973.1 |
| GN359642.1 | FB748846.1 | GN032791.1 | AX141777.1 | DL114442.1 | DL124316.1 | AX840542.1 | AX144923.1 | GM633941.1 |
| GN359546.1 | HV946828.1 | DL026699.1 | AX141713.1 | DL114410.1 | DL101120.1 | AX840016.1 | AX144891.1 | GM633909.1 |
| GN359514.1 | HV940473.1 | DJ402656.1 | AX141585.1 | DL114378.1 | DL110241.1 | AX832720.1 | AX144859.1 | GM633877.1 |
| GN359482.1 | HV946282.1 | DJ401117.1 | AX141521.1 | DL114346.1 | DL110209.1 | AX827000.1 | AX144827.1 | GM883091.1 |
| GN359478.1 | HV940065.1 | DJ418074.1 | AX141457.1 | DL109449.1 | DL110177.1 | AX825992.1 | AX144795.1 | GM655518.1 |
| GN359446.1 | HV945868.1 | DJ417446.1 | AX139815.1 | DL109417.1 | DL110113.1 | AX825287.1 | AX144763.1 | GM655486.1 |
| GN359414.1 | HV945834.1 | DJ386389.1 | AX133310.1 | DL109385.1 | DL110081.1 | AX824456.1 | AX144731.1 | GM655454.1 |
| GN359382.1 | FW552277.1 | DJ389572.1 | AX127565.1 | DL109353.1 | DL105180.1 | AX824346.1 | AX144699.1 | GM648365.1 |
| GM632576.1 | FW552239.1 | CS724699.1 | AX119972.1 | DL109321.1 | DL105148.1 | AX823780.1 | AX144667.1 | GM648333.1 |
| GM632614.1 | FW562061.1 | DJ340965.1 | HW103138.1 | DL109289.1 | DL105116.1 | AX822402.1 | AX144635.1 | GM648301.1 |
| GM632558.1 | FW552180.1 | DJ344506.1 | HW102898.1 | DL104555.1 | DL100702.1 | AX821572.1 | HW268413.1 | GM648269.1 |
| GM632526.1 | FW552144.1 | DJ361218.1 | HW098202.1 | DL104523.1 | DL100670.1 | AX816112.1 | HW267772.1 | GM648237.1 |
| GM632494.1 | FW552104.1 | DJ361185.1 | HW102324.1 | DL093939.1 | DL100638.1 | AX815014.1 | HW267740.1 | GM648205.1 |
| GM658536.1 | FW554687.1 | DJ339737.1 | HW059855.1 | DL093907.1 | DL100606.1 | AX814416.1 | HW267434.1 | GM641402.1 |
| GM658504.1 | FW563217.1 | DD361301.1 | HW061886.1 | DL093875.1 | DL100574.1 | AX802758.1 | HW267190.1 | GM641370.1 |
| GM658426.1 | FW555607.1 | CS456712.1 | HW061853.1 | DL093843.1 | DL100542.1 | AX802170.1 | HW266147.1 | GM641338.1 |
| GM658464.1 | FW555575.1 | CS453708.1 | HW059136.1 | DL093811.1 | DL119996.1 | AX805538.1 | HW262992.1 | GM641274.1 |
| GM658408.1 | FW560403.1 | CS450622.1 | HW062168.1 | DL093779.1 | DL119964.1 | AX800023.1 | HW262856.1 | GM641242.1 |
| GM658376.1 | FW563130.1 | DD357896.1 | HW061807.1 | DL090068.1 | DL119932.1 | AX798416.1 | HC731681.1 | GM629051.1 |
| GM658344.1 | FW558555.1 | DD357478.1 | HW061708.1 | DL090036.1 | DL119900.1 | AX798316.1 | HC471765.1 | GM629019.1 |
| GM651152.1 | FW557321.1 | DD355438.1 | HW065301.1 | DL090004.1 | DL119868.1 | AX797116.1 | HC471733.1 | DL116500.1 |
| GM651120.1 | FW565831.1 | A17518.1 | HW065268.1 | DL089972.1 | DL119836.1 | AX797327.1 | HC471725.1 | DL116468.1 |
| GM651088.1 | FW561677.1 | A02597.1 | HW065236.1 | DL085903.1 | DL119804.1 | AX797104.1 | HC471669.1 | DL116436.1 |
| GM651056.1 | FW557084.1 | A04078.1 | HW065203.1 | DL085871.1 | DL115209.1 | AX796936.1 | HC471637.1 | DL111843.1 |
| GM651024.1 | FW557052.1 | CS434836.1 | HW058493.1 | DL126610.1 | DL115177.1 | AX144937.1 | HC471605.1 | DL111779.1 |
| GM650992.1 | FW557019.1 | DD347374.1 | HW064909.1 | DL123355.1 | DL115145.1 | AX144904.1 | HC729129.1 | DL111747.1 |
| GM644193.1 | FW562774.1 | CS417162.1 | HW064837.1 | DL123323.1 | DL115113.1 | AX144872.1 | HC688427.1 | DL111715.1 |
| GM644161.1 | FW560094.1 | CS416267.1 | HW061122.1 | DL123259.1 | DL115081.1 | AX144840.1 | HC679673.1 | DL111683.1 |
| GM644129.1 | FW560062.1 | CS410922.1 | HW056751.1 | DL123227.1 | DL115049.1 | AX144808.1 | HC490829.1 | DL128860.1 |
| GM644097.1 | FW560030.1 | DD017621.1 | HW064659.1 | DL123195.1 | DL110049.1 | AX144776.1 | HC490797.1 | DL128762.1 |
| GM644065.1 | FW559998.1 | BD495455.1 | HW060704.1 | DL089278.1 | DL110017.1 | AX144744.1 | HC490733.1 | DL116424.1 |
| GM644033.1 | FW559925.1 | BD434169.1 | HW062404.1 | DL089246.1 | DL109985.1 | AX144712.1 | HC490669.1 | DL116394.1 |
| GM636939.1 | FW566910.1 | BD434156.1 | HW060456.1 | DL125937.1 | DL109953.1 | AX144680.1 | HC490540.1 | DL116362.1 |
| GM636907.1 | FW562557.1 | BD453910.1 | HW056310.1 | DL125905.1 | DL109867.1 | AX144648.1 | HC472295.1 | DL111641.1 |
| GM636875.1 | FW559904.1 | BD453845.1 | HW062379.1 | DL125873.1 | DL118909.1 | AX144616.1 | HC472262.1 | DL111609.1 |
| GM632467.1 | FW566812.1 | BD453813.1 | HW062347.1 | DL125841.1 | DL118877.1 | AX144555.1 | HC472230.1 | DL111577.1 |
| GM632435.1 | FW505291.1 | BD453781.1 | HW062283.1 | DL125809.1 | DL118813.1 | AX144427.1 | HC472198.1 | DL111545.1 |
| GM632403.1 | FW505218.1 | BD453757.1 | HW042070.1 | DL125777.1 | DL109261.1 | AX144233.1 | HC472166.1 | DL111513.1 |
| GM632371.1 | FW505147.1 | BD453725.1 | HW042038.1 | DL125947.1 | DL109229.1 | AX143847.1 | HC471966.1 | DL111481.1 |
| GM632307.1 | FW508126.1 | BD453693.1 | HW043568.1 | DL122535.1 | DL109197.1 | AX143783.1 | HC471934.1 | DL101699.1 |
| DL075845.1 | FW508068.1 | BD453661.1 | HW043435.1 | DL122503.1 | DL109165.1 | AX143655.1 | HC471902.1 | DL101667.1 |
| DL075813.1 | FW562471.1 | BD453629.1 | HW041994.1 | DL122471.1 | DL109133.1 | AX143591.1 | HC471870.1 | DL101635.1 |

| | | | | | | | | |
|---|---|---|---|---|---|---|---|---|
| DL075768.1 | FW562407.1 | BD453597.1 | HW041962.1 | DL122439.1 | DL109101.1 | AX143399.1 | HC508167.1 | DL101603.1 |
| DL079905.1 | FW559642.1 | BD453547.1 | HW041930.1 | DL122407.1 | DL104367.1 | AX143335.1 | HB828659.1 | DL097125.1 |
| DL079802.1 | FW559592.1 | BD453515.1 | HW041832.1 | DL122375.1 | DL104335.1 | AX143143.1 | HB648653.1 | DL097093.1 |
| DL074581.1 | FW553191.1 | BD453483.1 | HW049384.1 | DL118121.1 | DL099921.1 | AX142951.1 | HB647047.1 | DL097029.1 |
| DL079054.1 | FW553134.1 | BD453451.1 | HW049353.1 | DL118089.1 | DL099889.1 | AX142823.1 | HB645738.1 | DL095137.1 |
| DL078970.1 | FW563902.1 | BD453419.1 | HW049321.1 | DL118057.1 | DL099857.1 | AX142759.1 | HB645706.1 | DL095105.1 |
| DL078938.1 | FW559424.1 | BD453380.1 | HW049289.1 | DL118025.1 | DL099825.1 | AX142695.1 | HB855082.1 | DL095041.1 |
| DL078907.1 | FW559403.1 | BD453242.1 | HW049257.1 | DL113336.1 | DL099793.1 | AX142567.1 | HB858475.1 | DL094977.1 |
| DL073612.1 | FW559355.1 | BD453210.1 | HW041796.1 | DL113304.1 | DL099761.1 | AX142501.1 | HB855982.1 | DL094945.1 |
| DL072408.1 | FW507404.1 | BD497803.1 | HW041428.1 | DL113272.1 | DL099729.1 | AX142437.1 | HB855889.1 | DL106663.1 |
| DL072287.1 | FW510251.1 | BD495997.1 | HW045678.1 | DL108471.1 | DL093719.1 | AX142373.1 | DM179949.1 | DL106567.1 |
| DL071962.1 | FW506408.1 | BD408124.1 | HW054002.1 | DL108439.1 | DL093687.1 | AX142245.1 | DM179527.1 | DL106535.1 |
| DL013612.1 | FW506095.1 | BD408085.1 | HV340083.1 | DL108355.1 | DL093655.1 | AX142117.1 | DM178922.1 | DL106503.1 |
| DL013580.1 | HI949469.1 | BD450231.1 | HV347093.1 | DL108343.1 | DL093591.1 | AX141987.1 | DM170848.1 | DL136296.1 |
| DL013548.1 | HI933923.1 | BD408014.1 | HV344602.1 | DL108311.1 | DL093559.1 | AX141923.1 | DM170815.1 | DL128700.1 |
| DL013516.1 | HI933807.1 | BD396045.1 | HV339897.1 | DL103641.1 | DL089944.1 | AX141857.1 | DM185743.1 | DL128666.1 |
| DL013484.1 | HI933703.1 | BD394870.1 | HV344488.1 | DL099099.1 | DL089912.1 | AX141793.1 | DM178633.1 | DL128632.1 |
| DL013452.1 | HI933599.1 | BD446182.1 | HV342139.1 | DL099067.1 | DL089848.1 | AX141473.1 | DM188067.1 | DL128547.1 |
| DL013420.1 | HI933483.1 | BD453361.1 | HV342055.1 | DL099035.1 | DL089784.1 | AX141409.1 | DM187871.1 | DL125070.1 |
| DL013388.1 | HI932163.1 | BD453330.1 | HV341801.1 | DL099003.1 | DL085779.1 | AX141281.1 | HB559461.1 | DL125038.1 |
| DL018079.1 | HI936912.1 | DL123682.1 | HV322263.1 | DL098971.1 | DL085747.1 | AX138930.1 | HB491702.1 | DL125006.1 |
| DL018015.1 | HI932082.1 | DL123650.1 | HV332611.1 | DL098939.1 | DL085715.1 | AX135075.1 | HB489482.1 | DL124974.1 |
| DL013264.1 | HI936487.1 | DL021798.1 | HV325952.1 | DL092929.1 | DL085683.1 | AX133528.1 | DM164001.1 | DL124927.1 |
| DL013232.1 | HI930652.1 | DL017592.1 | HV325846.1 | DL092897.1 | DL085651.1 | AX127713.1 | DM163968.1 | DL124895.1 |
| DL036958.1 | HI935248.1 | DL017560.1 | HV325711.1 | DL092865.1 | DL137525.1 | AX127187.1 | DM163733.1 | DL124863.1 |
| DL036926.1 | HI935125.1 | DL017496.1 | HV331762.1 | DL092833.1 | DL123140.1 | AX113821.1 | DM163299.1 | DL124831.1 |
| DL032691.1 | HI935073.1 | DL017464.1 | HV324873.1 | DL092801.1 | DL123108.1 | AX111979.1 | DM162309.1 | DL124799.1 |
| DL032659.1 | HI934109.1 | DL017432.1 | HV324509.1 | DL089154.1 | DL123076.1 | AX109018.1 | GN032934.1 | DL124767.1 |
| DL024549.1 | HI918902.1 | DL038468.1 | HV313392.1 | DL089122.1 | DL123044.1 | AX108707.1 | GN032902.1 | DL120774.1 |
| DL024517.1 | FW362775.1 | DL038436.1 | HV111924.1 | DL089090.1 | DL123012.1 | AX108341.1 | GN032870.1 | DL120742.1 |
| DL024453.1 | FW362717.1 | DL038404.1 | HV111840.1 | DL089058.1 | DL122980.1 | AX107885.1 | GN032806.1 | DL116211.1 |
| DL024421.1 | FW362636.1 | DL038372.1 | HV120058.1 | DL089026.1 | DL118790.1 | AX107385.1 | GN032774.1 | DL116179.1 |
| DL024389.1 | FW349844.1 | DL038340.1 | AX348468.1 | DL088994.1 | DL118758.1 | AX107090.1 | GN032743.1 | DL116147.1 |
| DL021177.1 | FW362085.1 | DL013177.1 | AX347323.1 | DL122353.1 | DL118726.1 | AX103785.1 | GN032711.1 | DL116115.1 |
| DL021145.1 | FW351541.1 | DL013145.1 | AX347285.1 | DL122321.1 | DL118694.1 | AX103650.1 | GN032679.1 | DL116083.1 |
| DL021113.1 | FW366222.1 | DL013113.1 | AX347247.1 | DL118003.1 | DL118662.1 | AX099997.1 | GN032647.1 | DL116051.1 |
| DL021081.1 | FW361637.1 | DL013081.1 | AX347215.1 | DL117907.1 | DL118630.1 | AX098293.1 | GN032614.1 | DL120575.1 |
| DL021049.1 | FW351314.1 | DL013049.1 | AX347177.1 | DL117875.1 | DL114234.1 | AX097529.1 | GN032582.1 | A28188.1 |
| DL021017.1 | FW351254.1 | DL013017.1 | AX347029.1 | DL117843.1 | DL114202.1 | AX093405.1 | GN032551.1 | A20314.1 |
| DL016811.1 | FW351192.1 | DL012985.1 | AX344531.1 | DL113250.1 | DL114170.1 | AX093005.1 | GN032487.1 | A20581.1 |
| DL016779.1 | FW366107.1 | DL033848.1 | AX339646.1 | DL113218.1 | DL114138.1 | HW112370.1 | GN032455.1 | A19502.1 |
| DL016715.1 | FW361523.1 | DL033816.1 | AX339353.1 | DL113186.1 | DL114106.1 | HW112285.1 | GN032423.1 | A16292.1 |
| DL016683.1 | FW350350.1 | DL033784.1 | AX338532.1 | DL113154.1 | DL114074.1 | HW072756.1 | GN032391.1 | A14611.1 |
| DL016651.1 | FW360425.1 | DL033752.1 | AX328281.1 | DL113122.1 | DL126214.1 | HW069961.1 | GN032358.1 | A10465.1 |
| DL016619.1 | HD122557.1 | DL033720.1 | AX327951.1 | DL113090.1 | DL126182.1 | HW069833.1 | GN032326.1 | A09817.1 |
| DL012161.1 | HD122228.1 | DL042437.1 | AX306731.1 | DL113058.1 | DL126150.1 | HW069797.1 | GN032294.1 | A30538.1 |
| DL012129.1 | HD122271.1 | DL042405.1 | AX306485.1 | DL108257.1 | DL122934.1 | HW069765.1 | GN032262.1 | A29193.1 |

| | | | | | | | | |
|---|---|---|---|---|---|---|---|---|
| DL012097.1 | HD115947.1 | DL042373.1 | AX277040.1 | DL108225.1 | DL122902.1 | HW084953.1 | GN032230.1 | A21396.1 |
| DL012065.1 | HD085182.1 | DL042250.1 | AX266975.1 | DL108193.1 | DL122870.1 | HW084914.1 | GN032198.1 | A06572.1 |
| DL012033.1 | HD084276.1 | DL042218.1 | AX258849.1 | DL108161.1 | DL122838.1 | HW065062.1 | GN032166.1 | A27786.1 |
| DL012001.1 | HD112953.1 | DL042186.1 | AX303576.1 | DL108129.1 | DL122806.1 | HW050531.1 | GN032134.1 | A26377.1 |
| DL016593.1 | HD112833.1 | DL042154.1 | AX301006.1 | DL108097.1 | DL122774.1 | HV969756.1 | GN032102.1 | A25746.1 |
| DL016561.1 | HD082456.1 | DL042090.1 | AX299847.1 | DL103491.1 | DL118584.1 | HV956159.1 | GN032070.1 | A24908.1 |
| DL016529.1 | HD082381.1 | DL046437.1 | AX297548.1 | DL103459.1 | DL118552.1 | HV951086.1 | GN032038.1 | A22369.1 |
| DL016497.1 | HD081499.1 | DL046373.1 | AX286675.1 | DL103427.1 | DL017766.1 | HV940367.1 | GN032006.1 | A24685.1 |
| DL016465.1 | HD088559.1 | DL046341.1 | AX281576.1 | DL103395.1 | DL017734.1 | HV944851.1 | GN031974.1 | A23019.1 |
| DL016433.1 | HD087154.1 | DL046309.1 | AX279947.1 | DL098853.1 | DL017702.1 | HV943417.1 | GN031942.1 | A22233.1 |
| DL011911.1 | HD079249.1 | DL046277.1 | AX279655.1 | DL098821.1 | DL017670.1 | HV778434.1 | GN031878.1 | A16272.1 |
| DL011879.1 | HD077686.1 | DL038273.1 | AX278689.1 | DL098789.1 | DL017638.1 | HV777005.1 | GN031846.1 | A21738.1 |
| DL011847.1 | BD188249.1 | DL038241.1 | AX278274.1 | DL098757.1 | DL022372.1 | HV773803.1 | GN031814.1 | A21423.1 |
| DL011815.1 | BD187474.1 | DL038209.1 | AX278072.1 | DL092747.1 | DL022340.1 | HV757243.1 | GN031781.1 | A19448.1 |
| DL024354.1 | AX787391.1 | DL038177.1 | AX256401.1 | DL046434.1 | DL022308.1 | HV743692.1 | GN031749.1 | A13669.1 |
| DL024322.1 | AX786793.1 | DL038145.1 | AX253574.1 | DL046402.1 | DL022276.1 | HV743389.1 | GN031684.1 | A18340.1 |
| DL024290.1 | AX785123.1 | DL033711.1 | AX247643.1 | DL046370.1 | DL022244.1 | HW302705.1 | GN031652.1 | A17477.1 |
| DL024258.1 | AX777599.1 | DL033679.1 | AX242327.1 | DL046306.1 | DL022212.1 | HW302378.1 | GN031556.1 | A16026.1 |
| DL024194.1 | AX776522.1 | DL033647.1 | AX242295.1 | DL033676.1 | DL017610.1 | HW295619.1 | GN031524.1 | A14656.1 |
| DL045054.1 | AX774647.1 | DL033615.1 | AX242263.1 | DL033644.1 | DL025727.1 | HW295588.1 | GN031492.1 | A13839.1 |
| DL045022.1 | AX773257.1 | DL033583.1 | AX242231.1 | DL033580.1 | DL025695.1 | HW294981.1 | GN031427.1 | A12750.1 |
| DL044990.1 | AX771075.1 | DL033551.1 | AX242199.1 | DL033548.1 | DL025567.1 | HW307053.1 | GN031363.1 | A12354.1 |
| DL044958.1 | AX770157.1 | DL033519.1 | AX242103.1 | DL029508.1 | DL029709.1 | HW294087.1 | GN031331.1 | A11186.1 |
| DL044926.1 | AX769460.1 | DL029511.1 | AX242071.1 | DL029476.1 | DL029677.1 | HW293212.1 | GN031299.1 | A10786.1 |
| DL044894.1 | AX767523.1 | DL029479.1 | AX241943.1 | DL029444.1 | DL029645.1 | HW293037.1 | GN031266.1 | A10367.1 |
| DL040846.1 | AX766742.1 | DL029447.1 | AX241879.1 | DL029412.1 | DL029613.1 | HW292760.1 | GN031234.1 | A09361.1 |
| DL040814.1 | AX766408.1 | DL029383.1 | AX241815.1 | DL029380.1 | DL029581.1 | HW291301.1 | GN031202.1 | A16720.1 |
| DL040782.1 | AX765970.1 | DL029351.1 | AX241783.1 | DL046110.1 | DL025540.1 | HW291241.1 | GN031170.1 | A09226.1 |
| DL040750.1 | AX764655.1 | DL046241.1 | AX241719.1 | DL046078.1 | DL025508.1 | HW291176.1 | GN031137.1 | A08019.1 |
| DL036863.1 | AX764618.1 | DL046209.1 | AX241687.1 | DL041964.1 | DL025476.1 | HW291134.1 | HW261208.1 | A07326.1 |
| DL036831.1 | AX764586.1 | DL046177.1 | AX241655.1 | DL041932.1 | DL025444.1 | HW291079.1 | HW261176.1 | A06446.1 |
| DL032436.1 | AX764554.1 | DL046145.1 | AX241623.1 | DL041900.1 | DL025412.1 | HW291047.1 | HW261144.1 | A06009.1 |
| DL032404.1 | AX755104.1 | DL046113.1 | AX241591.1 | DL037917.1 | DL025380.1 | HW291015.1 | HW261112.1 | HW243434.1 |
| DL032372.1 | AX753584.1 | DL046081.1 | AX241559.1 | DL037885.1 | DL033938.1 | HW290983.1 | HW261080.1 | HW248185.1 |
| DL032340.1 | AX752739.1 | DL042063.1 | HW144374.1 | DL029315.1 | DL025359.1 | HW290919.1 | HW261016.1 | HW243148.1 |
| DL016417.1 | AX752423.1 | DL042031.1 | HW144342.1 | DL029283.1 | DL025327.1 | HW290888.1 | HW260984.1 | HW243056.1 |
| DL016385.1 | BD183726.1 | DL041999.1 | HW144310.1 | DL046011.1 | DL025295.1 | HW290856.1 | HW260952.1 | HW163792.1 |
| DL016353.1 | BD183123.1 | DL041967.1 | HW124804.1 | DL045979.1 | DL025263.1 | HW150559.1 | HW260920.1 | HW163489.1 |
| DL016321.1 | BD182777.1 | DL041935.1 | HW124519.1 | DL069480.1 | DL025231.1 | HW159728.1 | HW260888.1 | HW160630.1 |
| DL016289.1 | BD181896.1 | DL041903.1 | HW124064.1 | DL049717.1 | DL025199.1 | HW144670.1 | HW260824.1 | HW160598.1 |
| DL044087.1 | BD181090.1 | DL038080.1 | HW123524.1 | DL049685.1 | DL025167.1 | HW144630.1 | HW260792.1 | HW155910.1 |
| DL048224.1 | BD180841.1 | DL038048.1 | HW123458.1 | DL049653.1 | DL021955.1 | HW144533.1 | HW260760.1 | HW155754.1 |
| DL048192.1 | HW392231.1 | DL038016.1 | HW122139.1 | DL045709.1 | DL021923.1 | HW126646.1 | HW260696.1 | HW150105.1 |
| DL048160.1 | HW391548.1 | DL037984.1 | HW120964.1 | DL045677.1 | DL021891.1 | HW126557.1 | HW260664.1 | HW150041.1 |
| DL048128.1 | HW408900.1 | DL037952.1 | HW120170.1 | DL041862.1 | DL021859.1 | HW144351.1 | HW260632.1 | HW150009.1 |
| DL048064.1 | HW408868.1 | DL037920.1 | HW120095.1 | DL041830.1 | DL021795.1 | HW144287.1 | HW260600.1 | HW149977.1 |
| DL048033.1 | HW382309.1 | DL037888.1 | HW117536.1 | CQ877239.1 | DL017429.1 | HW124831.1 | HW260568.1 | HW144820.1 |

| | | | | | | | | |
|---|---|---|---|---|---|---|---|---|
| DL048001.1 | HW390538.1 | DL029318.1 | HW115650.1 | CQ876213.1 | DL038465.1 | HW124536.1 | HW260536.1 | HW155202.1 |
| DL047969.1 | HW390298.1 | DL029286.1 | HW101689.1 | CQ876146.1 | DL038433.1 | HW123545.1 | HW260504.1 | HW158783.1 |
| DL047937.1 | HW389599.1 | DL029222.1 | HW087394.1 | CQ875543.1 | DL038369.1 | HW123472.1 | HW260472.1 | HW158488.1 |
| DL047905.1 | HW389514.1 | DL029190.1 | HW105141.1 | CQ874996.1 | DL038337.1 | HW122766.1 | HW260440.1 | HW154647.1 |
| AY658962.1 | HW387484.1 | DL046046.1 | HW096495.1 | CQ874728.1 | DL038305.1 | HW121651.1 | HW260408.1 | HW154480.1 |
| AY658930.1 | HW386632.1 | DL046014.1 | HW072723.1 | CQ873296.1 | DL013174.1 | HW121253.1 | HW260376.1 | HW147482.1 |
| AY658898.1 | HW386568.1 | DL045982.1 | HW072671.1 | CQ871368.1 | DL013142.1 | HW118927.1 | HW260344.1 | HW147438.1 |
| AY658866.1 | HW386536.1 | DL045950.1 | HW096218.1 | CQ869301.1 | DL013110.1 | HW118895.1 | HW260312.1 | HW154312.1 |
| AY658834.1 | HW386472.1 | DL045918.1 | HW072445.1 | CQ868935.1 | DL013078.1 | HW118443.1 | HW260280.1 | HW147123.1 |
| AY658802.1 | HW386440.1 | DL047324.1 | HW072232.1 | CQ868376.1 | DL013046.1 | HW115124.1 | HW260248.1 | HW153798.1 |
| AY658770.1 | HW386408.1 | DL047292.1 | HV694909.1 | CQ867013.1 | DL013014.1 | HW087122.1 | HW260216.1 | HW153762.1 |
| AY658738.1 | HW386376.1 | DL047260.1 | HV694843.1 | CQ861216.1 | DL033909.1 | HW087090.1 | HW260184.1 | HW153722.1 |
| AY658706.1 | HW386344.1 | DL043438.1 | HV700729.1 | CQ860281.1 | DL033877.1 | HV822304.1 | HW260152.1 | HW145782.1 |
| AY658674.1 | HW386312.1 | DL043406.1 | HV700697.1 | CQ859651.1 | DL033845.1 | HV816795.1 | HW260120.1 | HW145517.1 |
| AY658642.1 | HW386248.1 | DL043374.1 | HV700665.1 | CQ859619.1 | DL033813.1 | HV821234.1 | HW260056.1 | HV951899.1 |
| AY658610.1 | HW386152.1 | DL043342.1 | HV694677.1 | CQ859235.1 | DL033781.1 | HV780159.1 | HW260024.1 | HV951796.1 |
| AY658578.1 | HW386120.1 | DL043310.1 | HV700247.1 | CQ858536.1 | DL033749.1 | HV779591.1 | HW259992.1 | HV946532.1 |
| AY658546.1 | HW386056.1 | DL043278.1 | HV693380.1 | CQ857843.1 | DL033717.1 | HV778904.1 | HW259960.1 | HV946276.1 |
| AY658514.1 | HW386024.1 | DL039461.1 | HV699825.1 | CQ857500.1 | DL042247.1 | HV778284.1 | HW259834.1 | HV940047.1 |
| AY658482.1 | HW385992.1 | DL039429.1 | HV699152.1 | CQ855755.1 | DL042215.1 | HV777075.1 | HW259689.1 | HV945935.1 |
| AY658450.1 | HW385960.1 | DL039397.1 | HV698338.1 | CQ854057.1 | DL042183.1 | HV775945.1 | HW258936.1 | HV943535.1 |
| AY658418.1 | HW385928.1 | DL039365.1 | HV698107.1 | CQ847390.1 | DL042151.1 | HV775038.1 | HW258826.1 | HV943279.1 |
| AY658386.1 | HW381011.1 | DL039333.1 | HV690005.1 | AX797102.1 | DL042119.1 | HV774956.1 | HW257315.1 | HV942761.1 |
| AY658354.1 | HW380916.1 | DL039301.1 | HV695919.1 | AX796934.1 | DL042087.1 | HV764778.1 | HW257283.1 | HV942058.1 |
| AY658322.1 | HW380825.1 | DL035677.1 | HV688790.1 | AX796755.1 | DL037582.1 | HV764746.1 | HW257251.1 | HV815896.1 |
| AY658290.1 | HW368547.1 | DL035645.1 | HV579908.1 | AX795594.1 | DL037550.1 | HV764714.1 | HW257187.1 | HV932147.1 |
| AY658258.1 | HW367137.1 | HH997154.1 | HV585091.1 | AX795443.1 | DL037518.1 | HV769882.1 | HW257123.1 | HV931873.1 |
| AY658226.1 | HW366940.1 | HH998891.1 | HV588894.1 | BD194404.1 | DL037486.1 | HV774492.1 | HW257091.1 | HV937063.1 |
| AY658194.1 | HW376133.1 | HH998831.1 | HV584896.1 | BD189471.1 | DL033283.1 | HV762871.1 | HW257059.1 | HV931452.1 |
| AY658162.1 | HW366484.1 | HH998788.1 | HV584787.1 | BD187409.1 | DL033251.1 | HV767907.1 | HW256995.1 | HV931381.1 |
| AY658130.1 | HW375076.1 | HH998720.1 | HV579125.1 | AX786790.1 | DL033219.1 | HV766816.1 | HW256931.1 | HV815184.1 |
| AY658098.1 | HW375070.1 | HH997074.1 | HV579006.1 | AX777471.1 | DL033187.1 | HV766730.1 | HW256899.1 | HV925497.1 |
| AY658066.1 | HW375045.1 | HH997016.1 | HV599523.1 | AX776169.1 | DL033155.1 | HV760621.1 | HW256835.1 | HV819678.1 |
| AY658034.1 | HW374879.1 | HH996965.1 | HV592489.1 | AX773254.1 | DL033123.1 | HV766534.1 | HW251051.1 | HV930405.1 |
| AY658002.1 | HW374640.1 | HH998718.1 | HV578519.1 | AX771049.1 | DL029115.1 | HV766097.1 | HW249736.1 | HV819219.1 |
| AY657970.1 | HW364600.1 | HH998684.1 | HV577340.1 | AX768206.1 | DL029083.1 | HV766049.1 | HW241242.1 | HV803289.1 |
| AY657938.1 | FW348581.1 | HH998609.1 | HV598705.1 | AX767440.1 | DL029051.1 | HV766017.1 | HW240731.1 | HV930197.1 |
| AY657906.1 | HW373309.1 | HH998569.1 | HV582974.1 | AX766886.1 | DL029019.1 | HV750374.1 | HV343502.1 | HV932631.1 |
| AY657874.1 | HW381357.1 | HH996933.1 | AX247556.1 | AX766736.1 | DL028987.1 | HV750342.1 | HV345269.1 | HV932591.1 |
| AY657842.1 | HW381052.1 | HH996901.1 | AX242326.1 | AX766398.1 | DL028955.1 | HV758992.1 | HV344830.1 | HV929736.1 |
| AY657810.1 | HW344449.1 | HH996780.1 | AX242294.1 | AX765967.1 | DL024981.1 | HV755503.1 | HV340011.1 | HV932333.1 |
| AY657778.1 | HW344385.1 | HH994573.1 | AX242262.1 | AX764647.1 | DL021769.1 | HV504956.1 | HV347058.1 | HV932294.1 |
| AY657746.1 | HW344353.1 | HH980690.1 | AX242230.1 | AX764615.1 | DL021737.1 | HV504924.1 | HV344746.1 | HV803149.1 |
| AY657714.1 | HW344321.1 | HH954756.1 | AX242198.1 | AX764583.1 | DL021705.1 | HV504892.1 | HV339661.1 | GM629018.1 |
| AY657682.1 | HW344289.1 | HH977157.1 | AX242166.1 | AX756512.1 | DL021673.1 | HV504828.1 | HV342085.1 | GM628986.1 |
| AY657650.1 | HW353885.1 | HH980611.1 | AX242134.1 | AX755069.1 | DL021641.1 | HV504796.1 | HV341791.1 | GM628954.1 |
| AY657618.1 | HW353853.1 | HH980507.1 | AX242038.1 | AX752696.1 | DL021609.1 | HV504779.1 | HV335463.1 | GM881899.1 |

| | | | | | | | | |
|---|---|---|---|---|---|---|---|---|
| AY657586.1 | HW353789.1 | HH980439.1 | AX241974.1 | AX752163.1 | DL017403.1 | HV504747.1 | HV322821.1 | GM655228.1 |
| AY657554.1 | HW353757.1 | HH957942.1 | AX241942.1 | AX746400.1 | DL017371.1 | HV504715.1 | HV322201.1 | GM655196.1 |
| AY657522.1 | HW344244.1 | HH980366.1 | AX241910.1 | BD183120.1 | DL017339.1 | HV504683.1 | HV321821.1 | GM655164.1 |
| AY657490.1 | HW344212.1 | HH980319.1 | AX241878.1 | BD182774.1 | DL012982.1 | HV504651.1 | HV333658.1 | GM648171.1 |
| AY657458.1 | HW344180.1 | HH980169.1 | AX241846.1 | BD180907.1 | DL045630.1 | HV504619.1 | HV333567.1 | GM641208.1 |
| AY657426.1 | HW344148.1 | HH980134.1 | AX241718.1 | BD180838.1 | DL045598.1 | HV491624.1 | HV187417.1 | GM641176.1 |
| AY657394.1 | HV753607.1 | HH980063.1 | AX241686.1 | AX744005.1 | DL045566.1 | HV504970.1 | HV189859.1 | GM641144.1 |
| AY657362.1 | HV755989.1 | HH979889.1 | AX241654.1 | AX741028.1 | DL045534.1 | HV504564.1 | HV192763.1 | GM641112.1 |
| AY657330.1 | HV753490.1 | HH996730.1 | AX241622.1 | AX739986.1 | DL045470.1 | HV504532.1 | HV208654.1 | GM641080.1 |
| AY657298.1 | HV743906.1 | HH996658.1 | AX241590.1 | AX722067.1 | DL041358.1 | HV504500.1 | FW571812.1 | GM628888.1 |
| AY657266.1 | HV743871.1 | HH996618.1 | AX241558.1 | AX721700.1 | DL041326.1 | HV504468.1 | HI968054.1 | GM628856.1 |
| AY657234.1 | HV743839.1 | HH996556.1 | AX241462.1 | BD178185.1 | DL041294.1 | HV504436.1 | HI967873.1 | GM628792.1 |
| AY657202.1 | HV743807.1 | HH998469.1 | AX241430.1 | BD177390.1 | DL037407.1 | HV504404.1 | HI988895.1 | GM618947.1 |
| AY657170.1 | HV743315.1 | HH998423.1 | AX241123.1 | BD177102.1 | CQ807212.1 | HV504372.1 | HI987461.1 | GM628680.1 |
| AY657138.1 | HV743277.1 | HH998375.1 | AX241091.1 | AX720709.1 | CQ803012.1 | HV504340.1 | HI987422.1 | GM628616.1 |
| AY657106.1 | HV743239.1 | HH998342.1 | AX241059.1 | AX718200.1 | CQ802139.1 | HV504308.1 | FW508659.1 | GM628584.1 |
| AY657074.1 | HV742928.1 | HH999966.1 | AX241027.1 | AX708518.1 | CQ802033.1 | HV504276.1 | FW552197.1 | GM628552.1 |
| AY657042.1 | HV742501.1 | HH999911.1 | AX240995.1 | AX705397.1 | CQ801231.1 | HV504244.1 | FW552160.1 | GM716100.1 |
| M20739.1 | CS695692.1 | HH979828.1 | AX240963.1 | AX705188.1 | CQ800799.1 | HV455596.1 | FW552122.1 | GM662557.1 |
| M24739.1 | CS695660.1 | HH979763.1 | AX240931.1 | AX701062.1 | CQ800621.1 | HV504176.1 | FW555589.1 | GM654970.1 |
| M15619.1 | CS695500.1 | HH979722.1 | AX235767.1 | AX699468.1 | CQ798432.1 | HV504144.1 | FW563144.1 | GM005812.1 |
| M32785.1 | CS695468.1 | HH979678.1 | AX224369.1 | AX699436.1 | CQ797564.1 | HV504112.1 | FW556524.1 | GM685505.1 |
| J02523.1 | CS695338.1 | HH976530.1 | AX214191.1 | AX698739.1 | CQ796754.1 | HV504080.1 | FW561691.1 | FB753778.1 |
| M12170.1 | DL176562.1 | HH999875.1 | AX207893.1 | BD074807.1 | CQ795496.1 | HV504048.1 | FW561659.1 | GM680783.1 |
| HV549868.1 | DL176462.1 | HH999786.1 | AX207287.1 | BD070701.1 | CQ795460.1 | HV504016.1 | FW565209.1 | GM879327.1 |
| HV549732.1 | DL176380.1 | HH998304.1 | AX205122.1 | BD069458.1 | CQ794580.1 | HV503984.1 | FW562577.1 | FB727450.1 |
| HV549692.1 | DL176305.1 | HH998255.1 | AX202540.1 | BD063686.1 | CQ793267.1 | HV503952.1 | FW508098.1 | GM618722.1 |
| HV549660.1 | DL175863.1 | HH998218.1 | AX202416.1 | BD062541.1 | CQ792370.1 | HV503920.1 | HI563610.1 | GM040588.1 |
| HV549628.1 | DL100873.1 | HH998166.1 | AF401225.1 | BD017755.1 | CQ788208.1 | HV503888.1 | HI563575.1 | FB709040.1 |
| HV549596.1 | DL100841.1 | HH996478.1 | AX193609.1 | BD016704.1 | CQ787491.1 | HV503856.1 | HI538014.1 | GM049546.1 |
| HV549425.1 | DL100809.1 | HH986566.1 | AX188577.1 | BD015186.1 | CQ787459.1 | HV503824.1 | HI000034.1 | GM841828.1 |
| HV549253.1 | DL100777.1 | HH979499.1 | AX174627.1 | BD014236.1 | CQ787421.1 | HV491138.1 | HI003671.1 | GM603717.1 |
| HV549221.1 | DL100745.1 | HH979644.1 | AX172937.1 | BD014203.1 | CQ787381.1 | HV503784.1 | HI003623.1 | GM706718.1 |
| HV549157.1 | DL096331.1 | HH979566.1 | AX172478.1 | BD014163.1 | CQ787347.1 | HV503752.1 | HI003584.1 | GM706686.1 |
| HV549087.1 | DL096299.1 | HH932046.1 | AX167092.1 | BD013386.1 | CQ787311.1 | HV503688.1 | HI003496.1 | GM706654.1 |
| HV544353.1 | DL096267.1 | HH931966.1 | AX166298.1 | AX482618.1 | CQ787279.1 | HV503656.1 | GM631774.1 | GM706584.1 |
| HV494738.1 | DL096235.1 | FW417389.1 | AX155099.1 | AX481676.1 | CQ787247.1 | HV503624.1 | GM622843.1 | GM706389.1 |
| HV502192.1 | DL096203.1 | FW394278.1 | HW260670.1 | E61337.1 | CQ786943.1 | HV503564.1 | GM741188.1 | GM706355.1 |
| HV502149.1 | DL096171.1 | FW394259.1 | HW260638.1 | E64482.1 | CQ784673.1 | HV503532.1 | GM657800.1 | FB742337.1 |
| HV453665.1 | DL120191.1 | FW394217.1 | HW260574.1 | AX469469.1 | CQ779572.1 | HV503468.1 | GM657767.1 | GM704819.1 |
| HV450086.1 | DL120159.1 | FW394185.1 | HW260542.1 | AX468463.1 | CQ778917.1 | HV503372.1 | GM704212.1 | FB720260.1 |
| HV450045.1 | DL120127.1 | FW418992.1 | HW260510.1 | AX466948.1 | CQ775508.1 | HV503340.1 | GM704159.1 | FB718245.1 |
| HV451960.1 | DL120095.1 | FW393129.1 | HW260478.1 | A15053.1 | CQ774609.1 | HV503308.1 | GM646878.1 | FB717766.1 |
| HV449753.1 | DL120063.1 | FW418614.1 | HW260446.1 | AX460510.1 | CQ772938.1 | HV503276.1 | GM646846.1 | FB677519.1 |
| HV445545.1 | DL120031.1 | FW418142.1 | HW260414.1 | AX458656.1 | CQ771663.1 | HV503244.1 | GM646814.1 | FB677433.1 |
| HV451131.1 | DL100722.1 | FW396786.1 | HW260382.1 | AX458574.1 | CQ771631.1 | HV503212.1 | GM640011.1 | FB676994.1 |
| HV448168.1 | DL094317.1 | FW396649.1 | HW260350.1 | AX456154.1 | CQ771599.1 | HV503189.1 | GM639979.1 | GM061151.1 |

| | | | | | | | | |
|---|---|---|---|---|---|---|---|---|
| HV437027.1 | DL094285.1 | FW395739.1 | HW260318.1 | AX454152.1 | CQ770994.1 | HV503157.1 | GM639947.1 | DL459983.1 |
| HV436861.1 | DL094253.1 | HH944566.1 | HW260286.1 | AX453999.1 | CQ768852.1 | HV503125.1 | GM639915.1 | DL260587.1 |
| HV444631.1 | DL094221.1 | HC504660.1 | HW260254.1 | AX453508.1 | CQ768408.1 | HV503093.1 | GM627277.1 | DL260064.1 |
| HV444006.1 | DL094189.1 | HC504603.1 | HW260222.1 | AX452038.1 | CQ767995.1 | HV503061.1 | GM627245.1 | DL241346.1 |
| HV438481.1 | DL094157.1 | HC494565.1 | HW260190.1 | A34767.1 | CQ766071.1 | HV502997.1 | GM627213.1 | DL241048.1 |
| HV350088.1 | DL090542.1 | HC493127.1 | HW260126.1 | HW264492.1 | AX685159.1 | HV502965.1 | GM627181.1 | DL240964.1 |
| HV438113.1 | DL090510.1 | HC504225.1 | HW260094.1 | HW263003.1 | AX682993.1 | HV502933.1 | GM627149.1 | DL240639.1 |
| HV437639.1 | DL090474.1 | HC502188.1 | HW260062.1 | HW262971.1 | AX674834.1 | HV502901.1 | GM660973.1 | FB674310.1 |
| HV443178.1 | DL090442.1 | HC501825.1 | HW260030.1 | HW262777.1 | AX665508.1 | HV502869.1 | GM660941.1 | DL236687.1 |
| HV443090.1 | DL090410.1 | HC500847.1 | HW259998.1 | HW262731.1 | AX664350.1 | HV502837.1 | GM646778.1 | DL236560.1 |
| GM625092.1 | DL090378.1 | HC499873.1 | HW259934.1 | HW262586.1 | AX662239.1 | HV502793.1 | GM646746.1 | DL233415.1 |
| GM625028.1 | DL086373.1 | FW304050.1 | HW259732.1 | HW261414.1 | AX662168.1 | HV502761.1 | GM646714.1 | DL206822.1 |
| GM624996.1 | DL086341.1 | FW302607.1 | HW259269.1 | HW261382.1 | AX659114.1 | HV502729.1 | GM646682.1 | DL101011.1 |
| GM624964.1 | DL086309.1 | FW302075.1 | HW258895.1 | HW261350.1 | AX657271.1 | HV502697.1 | GM646650.1 | DL100979.1 |
| GM624932.1 | DL086245.1 | FW301647.1 | HW258833.1 | HW261318.1 | AX657118.1 | HV502665.1 | GM646618.1 | DL100947.1 |
| GM623906.1 | DL086213.1 | FW300631.1 | HW257385.1 | HW261286.1 | BD176085.1 | HV502633.1 | GM639815.1 | DL096533.1 |
| GM623874.1 | DL110456.1 | FW299342.1 | HW257353.1 | HW261254.1 | BD175986.1 | HV502601.1 | GM646482.1 | DL096501.1 |
| GM623842.1 | DL110424.1 | FW299310.1 | HW257225.1 | HW261190.1 | BD174683.1 | HV502569.1 | GM646450.1 | DL096469.1 |
| GM623778.1 | DL110392.1 | FW299278.1 | HW257129.1 | HW261158.1 | BD174398.1 | HV502537.1 | GM646418.1 | DL094545.1 |
| GM623746.1 | DL110360.1 | FW298788.1 | HW257033.1 | HW261126.1 | AX645659.1 | HV502505.1 | GM626844.1 | DL094513.1 |
| GM658925.1 | DL110328.1 | FW298594.1 | HW256905.1 | HW261094.1 | AJ005290.1 | HV502473.1 | GM626812.1 | DL094481.1 |
| GM658893.1 | DL110296.1 | HC487980.1 | HW256809.1 | HW261062.1 | AX642228.1 | HV492761.1 | GM653509.1 | DL110714.1 |
| GM658861.1 | DL105491.1 | HC469468.1 | HW256745.1 | HW261030.1 | AX641862.1 | HV505352.1 | GM653477.1 | DL110682.1 |
| GM658829.1 | DL105459.1 | HC469406.1 | HW250936.1 | HW260998.1 | BD173314.1 | HV505231.1 | GM653445.1 | DL090744.1 |
| GM658797.1 | DL105427.1 | HC469008.1 | HW249638.1 | HW260934.1 | AX614973.1 | HV494742.1 | GM653413.1 | DL090712.1 |
| GM651541.1 | DL105395.1 | HC486612.1 | HW247836.1 | HW260902.1 | AX601759.1 | HV507711.1 | GM653381.1 | DL090680.1 |
| GM651509.1 | DL105363.1 | HC486527.1 | HW240929.1 | HW260870.1 | AX601594.1 | HV505009.1 | GM646382.1 | DL086579.1 |
| GM651445.1 | DL105331.1 | HC486462.1 | HW241314.1 | HW260838.1 | AX600112.1 | HV502155.1 | GM646350.1 | DL086547.1 |
| GM622222.1 | DL124350.1 | HC475391.1 | HW239329.1 | HW260806.1 | AX599036.1 | HI935112.1 | GM646318.1 | DL086515.1 |
| GM622190.1 | DL124318.1 | HC466646.1 | HW238753.1 | HW260774.1 | AX598986.1 | HI935063.1 | DL128749.1 | DL086483.1 |
| GM622158.1 | DL124286.1 | HC492345.1 | HW238560.1 | HW260742.1 | AX598934.1 | HI949808.1 | DL116413.1 | DL086451.1 |
| GM631017.1 | DL124254.1 | HC474339.1 | HW238320.1 | HW260710.1 | AX598776.1 | HI929258.1 | DL116381.1 | DL086419.1 |
| GM630985.1 | DL124222.1 | DJ065240.1 | HW242997.1 | HW260678.1 | AX597788.1 | HI918325.1 | DL116349.1 | DL115521.1 |
| GM630953.1 | DL124190.1 | DJ067044.1 | HW242952.1 | HW260646.1 | AX593877.1 | HI918273.1 | DL111660.1 | DL115489.1 |
| GM630921.1 | DL110273.1 | DJ052750.1 | HV802941.1 | HW260614.1 | AX587866.1 | HI917021.1 | DL111628.1 | DL115457.1 |
| GM630896.1 | DL110243.1 | DJ061652.1 | HV802909.1 | HW260582.1 | AX587771.1 | HI661441.1 | DL111596.1 | DL105673.1 |
| GM635853.1 | DL110211.1 | DJ061508.1 | HV818592.1 | HW260550.1 | AX587679.1 | HI661267.1 | DL111564.1 | DL105641.1 |
| GM635821.1 | DL110115.1 | DJ061460.1 | HV818566.1 | HW260518.1 | AX587571.1 | HI661026.1 | DL111500.1 | DL105609.1 |
| GM635789.1 | DL110083.1 | DJ055589.1 | HV817909.1 | HW260486.1 | AX591043.1 | HI659191.1 | DL101686.1 | DL100900.1 |
| GM635757.1 | DL105150.1 | DJ055546.1 | HV778613.1 | HW260454.1 | BD161160.1 | HI657827.1 | DL101654.1 | DL100868.1 |
| GM635725.1 | DL105118.1 | DJ061302.1 | HV775028.1 | HW260422.1 | BD160915.1 | HI656348.1 | DL101622.1 | DL100836.1 |
| GM635693.1 | DL100704.1 | DJ061134.1 | HV764768.1 | HW260390.1 | AX587915.1 | HI654466.1 | DL106554.1 | DL100804.1 |
| GM630888.1 | DL100672.1 | DJ060415.1 | HV764704.1 | HW260358.1 | AX587867.1 | FW421371.1 | DL106522.1 | DL100772.1 |
| GM630857.1 | DL100640.1 | DJ054291.1 | HV769981.1 | HW260326.1 | AX587766.1 | FW498198.1 | DL120594.1 | DL100740.1 |
| GM630825.1 | DL100608.1 | DJ060350.1 | AX146314.1 | HW260294.1 | AX587713.1 | FW503025.1 | DL120562.1 | DL096326.1 |
| GM630793.1 | DL100576.1 | DJ060308.1 | AX145727.1 | HW260262.1 | AX587663.1 | FW502936.1 | DL088957.1 | DL096294.1 |
| GM630761.1 | DL100544.1 | DJ060274.1 | AX145695.1 | HW260230.1 | AX587612.1 | FW497437.1 | DL088925.1 | DL096262.1 |

| | | | | | | | | |
|---|---|---|---|---|---|---|---|---|
| GM630729.1 | DL119998.1 | DJ056900.1 | AX145663.1 | HW260166.1 | AX587562.1 | FW503886.1 | DL088595.1 | DL096230.1 |
| GM630697.1 | DL119966.1 | DJ053668.1 | AX145631.1 | HW260102.1 | AX575318.1 | FW499109.1 | DL092352.1 | DL096166.1 |
| GM621858.1 | DL119934.1 | DJ056360.1 | AX145567.1 | HW260070.1 | AX573176.1 | FW499036.1 | DL092320.1 | DD158409.1 |
| GM657323.1 | DL119902.1 | DJ053220.1 | AX145535.1 | HW260038.1 | AX556829.1 | FW504942.1 | DL092288.1 | DD157380.1 |
| GM657291.1 | DL119870.1 | DJ052902.1 | AX145503.1 | HW260006.1 | AX556195.1 | FW503699.1 | DL092256.1 | DD157353.1 |
| GM657227.1 | DL119838.1 | CS810686.1 | AX145471.1 | HW259974.1 | AX555699.1 | FW497160.1 | DL092224.1 | DD154091.1 |
| GM657195.1 | DL119806.1 | CS810644.1 | AX145439.1 | HW259942.1 | AX364410.1 | FW420479.1 | DL092192.1 | DD153646.1 |
| GM657163.1 | DL115211.1 | CS810543.1 | AX145407.1 | HW105245.1 | AX364378.1 | FW420447.1 | DL092160.1 | DD152594.1 |
| GM649971.1 | DL115179.1 | CS139102.1 | AX145374.1 | HW104683.1 | AX364346.1 | FW420415.1 | DL088417.1 | DD151836.1 |
| GM649939.1 | DL115147.1 | CS138402.1 | AX145342.1 | HW072157.1 | AX364251.1 | FW503327.1 | DL088385.1 | DD096999.1 |
| GM649907.1 | DL115115.1 | CS134724.1 | AX145310.1 | HW104514.1 | AX364186.1 | FW501749.1 | DL028657.1 | CQ772715.1 |
| GM649843.1 | DL115083.1 | CS132068.1 | AX145278.1 | HW104457.1 | AX359936.1 | FW498690.1 | DL028625.1 | CQ771659.1 |
| GM649811.1 | DL115051.1 | CS131927.1 | AX145214.1 | HW104425.1 | AX358651.1 | FW420390.1 | DL028593.1 | CQ771627.1 |
| GM642971.1 | DL110051.1 | CS131490.1 | AX145182.1 | HW104379.1 | AX357303.1 | FW420358.1 | DL028561.1 | CQ771595.1 |
| GM642938.1 | DL110019.1 | CS123620.1 | AX145086.1 | HW104345.1 | BD006813.1 | FW420326.1 | DL048680.1 | CQ768818.1 |
| GM642906.1 | DL109987.1 | CS123357.1 | AX145022.1 | HW104279.1 | BD002173.1 | FW496853.1 | DL023659.1 | CQ768378.1 |
| GM642874.1 | DL109955.1 | CS122759.1 | AX144990.1 | HW071087.1 | E63247.1 | FW496529.1 | DL023627.1 | CQ767044.1 |
| GM642842.1 | DL109923.1 | CS122284.1 | AX144958.1 | HW104269.1 | BD002005.1 | HI653977.1 | DL023595.1 | CQ766067.1 |
| GM635660.1 | DL109891.1 | CS106100.1 | AX144925.1 | HW104157.1 | E54572.1 | HI653811.1 | DL015921.1 | CQ764840.1 |
| GM635628.1 | DL109869.1 | CS181891.1 | AX144893.1 | HW104117.1 | BD000415.1 | HI653639.1 | DL015889.1 | CQ759571.1 |
| GM635596.1 | DL109837.1 | CS179795.1 | AX144861.1 | HW104074.1 | BD000199.1 | HI653605.1 | DL015857.1 | CQ758821.1 |
| GM635564.1 | DL109805.1 | CS341903.1 | AX144829.1 | HW104006.1 | E59171.1 | HI653535.1 | DL047599.1 | CQ756676.1 |
| GM635532.1 | DL109773.1 | CS328408.1 | AX144797.1 | HW103882.1 | AX354700.1 | HI653494.1 | DL047567.1 | CQ754041.1 |
| GM635500.1 | DL109741.1 | CS327971.1 | AX144765.1 | HW099619.1 | AX352800.1 | HI653286.1 | DL047535.1 | CQ753251.1 |
| GM635468.1 | DL041831.1 | CS323762.1 | AX144701.1 | HW099552.1 | AX351103.1 | HI653228.1 | DL039672.1 | AX962037.1 |
| GM630662.1 | DL041799.1 | CS323602.1 | AX144669.1 | HW069681.1 | AX349044.1 | HI652983.1 | DL039640.1 | AX960569.1 |
| GM630630.1 | DL041767.1 | CS323256.1 | AX144637.1 | HW089224.1 | AX348477.1 | HI648799.1 | DL039608.1 | AX960371.1 |
| GM630598.1 | DL041735.1 | CS322994.1 | AX144469.1 | HW067358.1 | AX277250.1 | HI647562.1 | DL039315.1 | AX958072.1 |
| GM621663.1 | DL041703.1 | CS330845.1 | HW336161.1 | HW088827.1 | AX347456.1 | HI646927.1 | DL023554.1 | AX952808.1 |
| GM621631.1 | DL037880.1 | CS329310.1 | HW336062.1 | HW083268.1 | AX347374.1 | HI646309.1 | DL023522.1 | AX938892.1 |
| GM621599.1 | DL037848.1 | CS320117.1 | HW335974.1 | HW083236.1 | AX347292.1 | AY145506.1 | DL023490.1 | AX937040.1 |
| GM621567.1 | DL037816.1 | CS319609.1 | HW328742.1 | HW083204.1 | AX347254.1 | HH807095.1 | DL023458.1 | AX935367.1 |
| GM657115.1 | DL037784.1 | CS318764.1 | HW328448.1 | HW082737.1 | AX347222.1 | HH806446.1 | DL023426.1 | AX934337.1 |
| GM657083.1 | DL037752.1 | CS322155.1 | HW328340.1 | HW088204.1 | AX347184.1 | HH999910.1 | DL023394.1 | AX924435.1 |
| GM657051.1 | DL037720.1 | DD261139.1 | HW328201.1 | HW081793.1 | AX347126.1 | HH979827.1 | DL020379.1 | AX923409.1 |
| GM657019.1 | DL037688.1 | DD259890.1 | HW318665.1 | HW099467.1 | AX345382.1 | HH979762.1 | DL020347.1 | AX923376.1 |
| GM656987.1 | DL049613.1 | DD259354.1 | HW318485.1 | HW103538.1 | AX344854.1 | HH979721.1 | DL020315.1 | AX840530.1 |
| GM656955.1 | DL049485.1 | DD259181.1 | HW335181.1 | HW084528.1 | AX344130.1 | HH979677.1 | DD449169.1 | AX829104.1 |
| GM656924.1 | DL049453.1 | DD265480.1 | HW335060.1 | HW102875.1 | AX342857.1 | HH976529.1 | CS620290.1 | AX825051.1 |
| GM656828.1 | DL041662.1 | DD272167.1 | HW314156.1 | HW102608.1 | AX339188.1 | HH999874.1 | CS616590.1 | AX824450.1 |
| GM656796.1 | DL041630.1 | CS305246.1 | HW314028.1 | HW067943.1 | AX328288.1 | HH999785.1 | CS616488.1 | AX824340.1 |
| GM656764.1 | DL041598.1 | CS302607.1 | HW311827.1 | HW056693.1 | AX327977.1 | HH999731.1 | GM619910.1 | AX823898.1 |
| GM649771.1 | DL041566.1 | CS302525.1 | HW311066.1 | HW061827.1 | AX326767.1 | HH998300.1 | GM648284.1 | AX823772.1 |
| GM649739.1 | DL041534.1 | CS299667.1 | HW309400.1 | HV822302.1 | AX319639.1 | HH998254.1 | GM648252.1 | AX822294.1 |
| GM649707.1 | DL037679.1 | CS299441.1 | HW307939.1 | HV817654.1 | AX306837.1 | HH998217.1 | GM648220.1 | AX816379.1 |
| GM649675.1 | DL037647.1 | CS297023.1 | HW307865.1 | HV817125.1 | AX268698.1 | HH998165.1 | GM641385.1 | AX816103.1 |
| GM649643.1 | DL037615.1 | DD252257.1 | HW307833.1 | HV816789.1 | AX259229.1 | HH996474.1 | GM641353.1 | AX815005.1 |

| | | | | | | | | |
|---|---|---|---|---|---|---|---|---|
| GM649611.1 | DL037583.1 | DD252142.1 | HW307801.1 | HV780279.1 | AX304316.1 | HH993625.1 | GM629066.1 | AX814473.1 |
| GM642707.1 | DL037551.1 | DD252000.1 | HW307769.1 | HV779732.1 | AX299858.1 | HH986544.1 | CS695415.1 | AX814297.1 |
| GM642675.1 | DL037519.1 | DD250297.1 | HW307763.1 | HV778628.1 | AX286556.1 | HH979643.1 | DL176634.1 | AX809453.1 |
| GM642643.1 | DL033252.1 | DD258251.1 | HW307731.1 | HV775036.1 | AX283692.1 | HH979561.1 | DL176416.1 | AX805939.1 |
| GM642127.1 | DL033220.1 | DD258159.1 | HW307699.1 | HV764776.1 | AX283226.1 | HH932045.1 | DL180979.1 | AX108616.1 |
| GM642095.1 | DL033188.1 | DD257581.1 | HW315944.1 | HV764744.1 | AX279939.1 | HH931965.1 | DL176745.1 | AX108279.1 |
| GM642063.1 | DL033124.1 | CS287568.1 | HW315559.1 | HV764712.1 | AX282193.1 | FW416907.1 | DL163232.1 | AX107013.1 |
| GM642031.1 | DL029116.1 | DD240765.1 | HW315070.1 | HV769880.1 | AX280354.1 | FW394277.1 | FB343411.1 | AX103644.1 |
| GM634844.1 | DL029084.1 | DD240716.1 | HW314775.1 | HV764641.1 | AX279958.1 | FW394258.1 | DL088108.1 | AX100367.1 |
| GM634812.1 | DL029052.1 | DD240668.1 | HW314724.1 | HV774554.1 | AX279761.1 | FW394216.1 | DL102882.1 | AX097520.1 |
| GM634780.1 | DL029020.1 | DD240612.1 | HW314612.1 | HV774490.1 | AX278762.1 | FW394184.1 | DL102850.1 | AX097486.1 |
| GM629878.1 | DL028988.1 | DD240496.1 | HW121185.1 | HV774124.1 | AX278094.1 | FW418991.1 | DL102818.1 | AX088801.1 |
| GM629814.1 | DL028956.1 | DD240464.1 | HW106128.1 | HV767899.1 | AX256352.1 | FW418990.1 | DL097999.1 | AX088749.1 |
| GM629782.1 | DL021770.1 | DD238954.1 | HW112765.1 | HV766947.1 | AX256272.1 | FW397438.1 | DL097935.1 | AX088693.1 |
| GM629750.1 | DL021738.1 | DD236646.1 | HW112665.1 | HV766728.1 | A05308.1 | FW418785.1 | DL112529.1 | AX085496.1 |
| GM629718.1 | DL021706.1 | DD235859.1 | HW112607.1 | HV760619.1 | A04274.1 | FW418604.1 | DL120273.1 | AX082979.1 |
| DD057161.1 | DL021674.1 | DD234718.1 | HW069950.1 | HV766529.1 | A01352.1 | FW418141.1 | DL120241.1 | AX080917.1 |
| DD054819.1 | DL021642.1 | DD248264.1 | HW069822.1 | HV766313.1 | A00167.1 | FW396785.1 | DL120209.1 | AX079167.1 |
| DD054159.1 | DL017404.1 | DD247189.1 | HW069786.1 | HV766047.1 | K02388.1 | FW396374.1 | DL114490.1 | AX077285.1 |
| DD053310.1 | DL017372.1 | DD246850.1 | HW084942.1 | HV766015.1 | K02764.1 | FW396264.1 | DL114458.1 | AX073919.1 |
| DD052460.1 | DL017340.1 | DD241490.1 | HV571048.1 | HV750340.1 | M12261.1 | FW396648.1 | DL104731.1 | AJ286125.1 |
| DD052236.1 | DL017308.1 | E41530.1 | HV565945.1 | HV582261.1 | J00606.1 | HH932767.1 | DL095936.1 | AX067623.1 |
| DD051775.1 | DL017276.1 | CS027608.1 | HV573444.1 | HV582156.1 | M16784.1 | HH935168.1 | DL095904.1 | AX063412.1 |
| DD059417.1 | DL017244.1 | CS027205.1 | HV560307.1 | HV581988.1 | M33415.1 | HH934831.1 | DL095872.1 | AX060693.1 |
| DD058667.1 | DL012983.1 | CS025559.1 | HV560057.1 | HV601421.1 | M55327.1 | HH834795.1 | DL100323.1 | AX058560.1 |
| DD048087.1 | DL012951.1 | CS023756.1 | HV555946.1 | HV586270.1 | OP819668.1 | HH833648.1 | DL104568.1 | AX058345.1 |
| DD030078.1 | DL012919.1 | CS022581.1 | HV560895.1 | HV601126.1 | MG561430.1 | HH833616.1 | DL104536.1 | AX057299.1 |
| DD038491.1 | DL012887.1 | CS021423.1 | HV553638.1 | HV574886.1 | U21231.1 | HH833584.1 | DL123336.1 | AX056665.1 |
| DD038312.1 | DL012823.1 | CS018497.1 | HV551457.1 | HV574485.1 | KY286122.1 | HH833552.1 | DL033296.1 | AX052958.1 |
| DD037079.1 | DL012791.1 | CS018355.1 | HV551414.1 | HV585699.1 | AH002291.2 | HH833488.1 | DL033264.1 | AX052919.1 |
| DD032535.1 | DL045631.1 | CS016847.1 | HV550400.1 | HV570600.1 | M19105.1 | HH833456.1 | DL029128.1 | AX046152.1 |
| DD030758.1 | DL045599.1 | CS016558.1 | AY659401.1 | HV569350.1 | M18101.1 | HH833424.1 | DL029096.1 | AX040787.1 |
| DD030687.1 | DL045567.1 | CS016482.1 | AY659369.1 | HV569041.1 | AY521453.1 | HH833392.1 | DL028968.1 | AX039147.1 |
| DD027332.1 | DL045535.1 | CS016122.1 | AY659337.1 | HV568109.1 | AY339062.1 | GM653075.1 | DL021782.1 | AX037329.1 |
| DD026862.1 | DL045503.1 | CS016027.1 | AY659305.1 | HV568053.1 | AF003706.1 | GM653043.1 | DL021750.1 | AX035434.1 |
| DD025979.1 | DL045471.1 | CS007948.1 | AY659273.1 | HV567901.1 | AF003722.1 | GM653011.1 | DL021718.1 | AX029304.1 |
| DD023833.1 | DL041359.1 | CQ986640.1 | AY659241.1 | HV566951.1 | KJ668039.1 | GM652979.1 | DL021590.1 | AX028760.1 |
| DD023245.1 | DL041327.1 | CQ986606.1 | AY659209.1 | HV566064.1 | HW408801.1 | GM645980.1 | DL028825.1 | AX027722.1 |
| DD020773.1 | DL026878.1 | HB427180.1 | AY659177.1 | HV565807.1 | HW408769.1 | GM645948.1 | DL042639.1 | AX024597.1 |
| DD029707.1 | DL026846.1 | HB426470.1 | AY659145.1 | HV573539.1 | HW408737.1 | GM645916.1 | DL042575.1 | AX023653.1 |
| DD010163.1 | DL026814.1 | HB423125.1 | AY659113.1 | HV572429.1 | HW408705.1 | GM645884.1 | DL029765.1 | AX023621.1 |
| DD019576.1 | DL026782.1 | HB423086.1 | AY659081.1 | HV572126.1 | HW408646.1 | GM645852.1 | DL007474.1 | AX023587.1 |
| DD017810.1 | DL022970.1 | HB403631.1 | AY659049.1 | HV571423.1 | HW408582.1 | GM645820.1 | DJ493879.1 | AX023274.1 |
| DD014217.1 | DL022938.1 | HB416465.1 | AY659017.1 | HV558754.1 | HW408486.1 | GM639017.1 | DJ491545.1 | AX019767.1 |
| DD009543.1 | DL022906.1 | HB397001.1 | AY658985.1 | HV555464.1 | HW408455.1 | GM638953.1 | DJ446844.1 | AX016807.1 |
| BD495450.1 | DL022874.1 | HB396571.1 | AY658953.1 | HV554302.1 | HW408391.1 | GM638889.1 | DJ446702.1 | AX012340.1 |
| BD412413.1 | DL022842.1 | HB394727.1 | AY658921.1 | HV550965.1 | HW408359.1 | GM638857.1 | DJ446140.1 | AX011479.1 |

| | | | | | | | | |
|---|---|---|---|---|---|---|---|---|
| BD454041.1 | DL022810.1 | HB394315.1 | AY658889.1 | HV550409.1 | HW390758.1 | GM626306.1 | DD401424.1 | AX011447.1 |
| BD453905.1 | DL019787.1 | GM654985.1 | AY658857.1 | AY659410.1 | HW390726.1 | GM626274.1 | DD401392.1 | AX011415.1 |
| BD453872.1 | DL019731.1 | GM694856.1 | AY658825.1 | AY659378.1 | HW399691.1 | GM626242.1 | DD405207.1 | HW314729.1 |
| BD453840.1 | DL019699.1 | GM694790.1 | CS603420.1 | HV547411.1 | HW399337.1 | GM626210.1 | DD405175.1 | HW106134.1 |
| BD453808.1 | DL019667.1 | GM044487.1 | CS603388.1 | HV543502.1 | HW399253.1 | GM626146.1 | DD405143.1 | HW112793.1 |
| BD453774.1 | DL019635.1 | GM755085.1 | CS603356.1 | HV539698.1 | HW382301.1 | GM660281.1 | DD405111.1 | HW112684.1 |
| BD411891.1 | DL019603.1 | GM693901.1 | CS603324.1 | HV538613.1 | HW390532.1 | GM660249.1 | DD402422.1 | HW112614.1 |
| BD453752.1 | DL015200.1 | GM836204.1 | CS603228.1 | HV538457.1 | HW390484.1 | GM660217.1 | DD402390.1 | HW070115.1 |
| BD453720.1 | DL015168.1 | GM752858.1 | CS603196.1 | HV537609.1 | HW388919.1 | GM660185.1 | DD401980.1 | CS456701.1 |
| BD453688.1 | DL015136.1 | DL467589.1 | CS603164.1 | HV543180.1 | HW388644.1 | GM660153.1 | DD401919.1 | CS453657.1 |
| BD453656.1 | DL015104.1 | GM659987.1 | CS603132.1 | HV542753.1 | HW387861.1 | GM652947.1 | DD401887.1 | CS452802.1 |
| BD453624.1 | DL015040.1 | GM659955.1 | CS603100.1 | HV542721.1 | HW387478.1 | GM652915.1 | DD401855.1 | CS451659.1 |
| BD453587.1 | DL010582.1 | GM652760.1 | CS603068.1 | HV542689.1 | HW387381.1 | GM652883.1 | DD401823.1 | DD105862.1 |
| BD453542.1 | DL010550.1 | GM652728.1 | CS603036.1 | HV542184.1 | HW386629.1 | GM652851.1 | DD401551.1 | DD061277.1 |
| BD453510.1 | DL010518.1 | GM652696.1 | CS603004.1 | HV541497.1 | HW386597.1 | GM652819.1 | DD401519.1 | DD091023.1 |
| A19212.1 | DL010486.1 | GM652664.1 | CS602970.1 | HV540032.1 | HW386565.1 | GM652786.1 | DD401487.1 | DD118105.1 |
| A01561.1 | DL010454.1 | GM652632.1 | CS602938.1 | HV533088.1 | HW386309.1 | GM645788.1 | DD401455.1 | DD090411.1 |
| DD219541.1 | DL010422.1 | GM652600.1 | CS602906.1 | HV536541.1 | HW337726.1 | GM645756.1 | CS469261.1 | DD117207.1 |
| DD221338.1 | DL046819.1 | GM645474.1 | CS602810.1 | HV536000.1 | HW337598.1 | GM645724.1 | CS141616.1 | DD102547.1 |
| DD216679.1 | DL046787.1 | GM645442.1 | CS602714.1 | HV532383.1 | HW337214.1 | GM645692.1 | CS141551.1 | DD052069.1 |
| DD213975.1 | DL046755.1 | GM645609.1 | CS602108.1 | HV537236.1 | HW337086.1 | GM645660.1 | CS141514.1 | DD057942.1 |
| DD213882.1 | DL042869.1 | GM638613.1 | E06814.1 | HV535445.1 | HW336958.1 | GM645628.1 | CS134735.1 | DD057910.1 |
| DD213850.1 | DL042805.1 | GM638581.1 | E05664.1 | HV516271.1 | HW321652.1 | GM638829.1 | CS135968.1 | DD057878.1 |
| DD213818.1 | DL042773.1 | GM638549.1 | E04460.1 | HV528092.1 | HW321202.1 | GM638797.1 | CS123412.1 | DD057846.1 |
| DD216451.1 | DL042741.1 | GM638517.1 | E04179.1 | HV515588.1 | HW336466.1 | GM638765.1 | CS122925.1 | DD056393.1 |
| DD216419.1 | DL042709.1 | GM638485.1 | E03004.1 | HV515556.1 | DM198840.1 | GM626122.1 | CS122353.1 | DD054768.1 |
| DD213769.1 | DL042677.1 | GM638453.1 | E02773.1 | HV515524.1 | HB806117.1 | GM626058.1 | CS106120.1 | DD054039.1 |
| DD213737.1 | DL038860.1 | GM633754.1 | E02032.1 | HV515492.1 | HB822111.1 | GM626026.1 | CS106058.1 | DD053306.1 |
| DD213705.1 | DL038796.1 | GM633722.1 | E01879.1 | HV515460.1 | HB805791.1 | GM625994.1 | CS179688.1 | DD052232.1 |
| DD213673.1 | DL038764.1 | GM633690.1 | E01426.1 | HV515428.1 | HB839908.1 | GM625962.1 | CS327368.1 | DD052177.1 |
| DD213641.1 | HI002817.1 | GM833883.1 | E01344.1 | HV515396.1 | HB848212.1 | GM625930.1 | CS326334.1 | DD059294.1 |
| DD213609.1 | HI002759.1 | GM625823.1 | E01212.1 | HV515364.1 | HB847928.1 | GM037795.1 | CS323580.1 | DD041987.1 |
| DD213591.1 | HI002717.1 | GM625791.1 | E00838.1 | HV515332.1 | HB847699.1 | FB775659.1 | CS329385.1 | DD048083.1 |
| DD213559.1 | HI002668.1 | GM625759.1 | E00592.1 | HV515300.1 | HB839400.1 | FB764713.1 | CS329181.1 | DD043425.1 |
| DD213527.1 | HI002634.1 | GM659928.1 | E00308.1 | HV515268.1 | HB847380.1 | FB764679.1 | DD261076.1 | DD030074.1 |
| DD216039.1 | HI002599.1 | GM624703.1 | E00110.1 | HV515236.1 | HB839178.1 | FB747069.1 | DD260301.1 | DD038487.1 |
| DD216007.1 | HI002542.1 | GM624671.1 | DD115289.1 | HV511669.1 | HB838774.1 | FB744815.1 | DD259333.1 | DD037311.1 |
| DD215975.1 | HI002489.1 | GM624639.1 | DD144078.1 | HV510359.1 | HB840327.1 | FB744004.1 | CS298538.1 | DD032624.1 |
| DD215929.1 | HI004495.1 | GM624607.1 | DD084754.1 | HV510057.1 | HB846167.1 | FB743938.1 | CS297034.1 | DD027324.1 |
| DD215897.1 | HI002464.1 | GM624575.1 | DD112542.1 | HV513365.1 | HB815011.1 | FB743905.1 | DD251644.1 | DD023829.1 |
| DD215865.1 | HI002404.1 | GM624543.1 | DD084673.1 | HV509290.1 | HB838116.1 | FB743873.1 | CS287611.1 | DD023359.1 |
| DD215833.1 | HI002326.1 | GM624446.1 | DD098151.1 | HV508596.1 | HB837768.1 | FB743841.1 | DD240733.1 | DD023223.1 |
| DD215801.1 | HI000532.1 | GM624414.1 | DD152075.1 | HV508532.1 | HB808931.1 | FB743787.1 | DD238992.1 | DD029703.1 |
| DD215769.1 | HI180550.1 | GM624382.1 | DD158402.1 | HV508472.1 | HB845689.1 | FB761602.1 | CS119397.1 | DD028789.1 |
| DD213048.1 | HI202811.1 | GM624350.1 | DD157378.1 | HV512415.1 | HB845413.1 | FB742835.1 | CS119364.1 | DD010450.1 |
| CS254892.1 | HI177877.1 | GM633653.1 | DD155704.1 | HV512274.1 | HB837209.1 | FB761491.1 | CS119332.1 | DD010247.1 |
| DD212342.1 | HI214571.1 | GM633621.1 | DD152994.1 | HV512242.1 | HB813987.1 | FB708868.1 | CS119266.1 | DD017356.1 |

| | | | | | | | | |
|---|---|---|---|---|---|---|---|---|
| DD211939.1 | HI214539.1 | GM633589.1 | DD152585.1 | HV512210.1 | HB845198.1 | GM877101.1 | HI002609.1 | DD009539.1 |
| DD210921.1 | HI177806.1 | GM633557.1 | DD151915.1 | HV512178.1 | HB844534.1 | GM036120.1 | HI002570.1 | BD495446.1 |
| DD209219.1 | HI214441.1 | GM633525.1 | DD097299.1 | HV339903.1 | HB844279.1 | FB713963.1 | HI002497.1 | BD453900.1 |
| DD208697.1 | FW381544.1 | GM633493.1 | DD096865.1 | HV335562.1 | HB836342.1 | FB713739.1 | HI004513.1 | BD453868.1 |
| DD206862.1 | FW376283.1 | GM625715.1 | DD082121.1 | HV342015.1 | HB835836.1 | FB713639.1 | HI002345.1 | BD453836.1 |
| DD206830.1 | FW381347.1 | GM625683.1 | DD081749.1 | HV323143.1 | HB843149.1 | FB708585.1 | HI002297.1 | BD453804.1 |
| DD206800.1 | FW381125.1 | GM625651.1 | DD149120.1 | HV332147.1 | HB835671.1 | FB708162.1 | HI000503.1 | CQ895565.1 |
| DD205875.1 | FW381017.1 | GM625619.1 | DD147997.1 | HV325765.1 | HB819137.1 | FB675156.1 | HC010670.1 | CQ895475.1 |
| CS236389.1 | FW375523.1 | GM625587.1 | DD147784.1 | HV331812.1 | HB818771.1 | FB670979.1 | DM375808.1 | CQ893706.1 |
| DD163496.1 | FW369979.1 | GM630401.1 | DD147752.1 | HV324881.1 | HB842620.1 | FB669039.1 | DM373420.1 | CQ890053.1 |
| DD163295.1 | FW375249.1 | GM630369.1 | DD147720.1 | HV313398.1 | HB842038.1 | FB667426.1 | DM370677.1 | CQ889051.1 |
| AY967261.1 | FW374986.1 | GM630337.1 | DD147688.1 | HV313248.1 | HB835192.1 | FB667115.1 | DM370645.1 | CQ879656.1 |
| AY967229.1 | FW380266.1 | GM630305.1 | DD147096.1 | HV111945.1 | HB841506.1 | FB721080.1 | DM370613.1 | CQ877814.1 |
| AY967197.1 | FW379847.1 | GM621530.1 | DD138532.1 | HV111859.1 | HB840937.1 | FB676864.1 | DM370581.1 | CQ876176.1 |
| AY967165.1 | FW369331.1 | GM621498.1 | DD132346.1 | HV119945.1 | HB840414.1 | FB676545.1 | DM370549.1 | CQ876128.1 |
| AY967133.1 | U06925.1 | GM739528.1 | DD135263.1 | HV227960.1 | HB649985.1 | FB676513.1 | DM370517.1 | CQ875538.1 |
| AY967101.1 | HH736047.1 | GM698718.1 | DD133743.1 | HV214867.1 | HB649253.1 | FB705981.1 | DM370485.1 | CQ874719.1 |
| AY967069.1 | HH735627.1 | GM656720.1 | DD132327.1 | HI563665.1 | HB646993.1 | FB704845.1 | DM205259.1 | CQ873291.1 |
| AY967037.1 | HH734136.1 | GM656688.1 | CQ819076.1 | HI558084.1 | HB645718.1 | FB701760.1 | DM209285.1 | CQ871418.1 |
| AY967005.1 | HH733637.1 | GM656656.1 | CQ818931.1 | HI563624.1 | HB645611.1 | FB701528.1 | HC003068.1 | CQ871224.1 |
| AY966973.1 | FW359903.1 | GM656624.1 | CQ818559.1 | HI563591.1 | HB850801.1 | CS810727.1 | HB865035.1 | CQ869296.1 |
| AY966941.1 | FW359843.1 | GM656592.1 | CQ817009.1 | HI558080.1 | HB858351.1 | FB701784.1 | HB864969.1 | CQ868906.1 |
| GM657479.1 | FW368819.1 | GM656560.1 | CQ816963.1 | HI508559.1 | HB856834.1 | FB702557.1 | HB864995.1 | CQ858162.1 |
| GM657447.1 | FW368689.1 | GM649567.1 | CQ815739.1 | HI583082.1 | HB855439.1 | FB660056.1 | HB864939.1 | CQ858070.1 |
| GM657383.1 | FW363796.1 | GM649535.1 | CQ814522.1 | HI575433.1 | HB475455.1 | FB580599.1 | HB864907.1 | CQ857740.1 |
| GM657351.1 | FW368151.1 | GM649503.1 | CQ814057.1 | HI544148.1 | HB474885.1 | FB654439.1 | HB864875.1 | CQ857495.1 |
| GM650159.1 | FW367653.1 | GM649471.1 | CQ814024.1 | HI508417.1 | HB464817.1 | FB656262.1 | HB864843.1 | CQ856033.1 |
| GM650127.1 | FW363361.1 | GM649439.1 | CQ813992.1 | HI508250.1 | HB469604.1 | FB654883.1 | HB864811.1 | CQ855928.1 |
| GM650095.1 | FW363204.1 | GM649407.1 | CQ813960.1 | HI000054.1 | HB468040.1 | FB583534.1 | HB864779.1 | CQ854363.1 |
| GM643126.1 | FW367459.1 | GM670047.1 | CQ813928.1 | HI000011.1 | DM152701.1 | FB582141.1 | HB864747.1 | CQ849500.1 |
| GM643094.1 | FW362875.1 | GM656542.1 | CQ813896.1 | HI003599.1 | DM152555.1 | FB582122.1 | HB999685.1 | CQ849244.1 |
| GM643062.1 | FW362665.1 | GM656510.1 | CQ813864.1 | HI003515.1 | DM151014.1 | FB580232.1 | HB976834.1 | CQ840690.1 |
| GM643030.1 | FW362076.1 | GM656478.1 | CQ813832.1 | HI001841.1 | DM155727.1 | FB580193.1 | HB976629.1 | CQ840492.1 |
| GM636009.1 | FW366347.1 | GM656414.1 | CQ813800.1 | HI001781.1 | DM150254.1 | FB580161.1 | DM189906.1 | CQ831872.1 |
| GM635977.1 | FW351419.1 | GM656382.1 | CQ813667.1 | HI465749.1 | DM149926.1 | FB580129.1 | HB840027.1 | CQ831498.1 |
| GM635945.1 | FW351305.1 | GM656350.1 | CQ803125.1 | HI465581.1 | HB461400.1 | FB580090.1 | HB848026.1 | CQ830724.1 |
| GM635913.1 | FW351189.1 | GM649357.1 | CQ802853.1 | HI575283.1 | HB460468.1 | DL189558.1 | HB839545.1 | CQ829232.1 |
| GM635881.1 | FW366101.1 | GM649301.1 | CQ801672.1 | HI001623.1 | HB455311.1 | DL199826.1 | HB847583.1 | CQ828080.1 |
| GM631092.1 | HD122554.1 | GM649317.1 | CQ800952.1 | HI001581.1 | HB455089.1 | DL199794.1 | HB847210.1 | CQ826849.1 |
| GM622221.1 | HD121700.1 | GM649261.1 | CQ800788.1 | HI003366.1 | HA640315.1 | AX805536.1 | HB838861.1 | CQ821486.1 |
| GM622189.1 | HD121605.1 | GM649229.1 | CQ798791.1 | HI003328.1 | HB445382.1 | AX800022.1 | HB805439.1 | CQ821294.1 |
| GM622157.1 | HD114847.1 | GM642588.1 | CQ795903.1 | HI071599.1 | HB444514.1 | AX799701.1 | HB847063.1 | CQ818565.1 |
| GM631016.1 | HD084273.1 | GM642556.1 | CQ795481.1 | HI505598.1 | DM121075.1 | AX798916.1 | HB846624.1 | CQ817039.1 |
| GM630984.1 | HC291474.1 | GM642516.1 | CQ795449.1 | HH928812.1 | DM139935.1 | AX798414.1 | HB846211.1 | CQ816976.1 |
| GM630952.1 | HC289354.1 | CS376216.1 | CQ788134.1 | HH931893.1 | DM139814.1 | AX798315.1 | GN029864.1 | CQ816936.1 |
| GM630895.1 | HC289322.1 | CS376010.1 | CQ787512.1 | HH821546.1 | DM134462.1 | AX796906.1 | GN029830.1 | CQ815747.1 |
| GM635852.1 | HC289184.1 | CS362752.1 | CQ787448.1 | HH821174.1 | DM138465.1 | AX797286.1 | GN013582.1 | CQ814095.1 |

| | | | | | | | | |
|---|---|---|---|---|---|---|---|---|
| GM635820.1 | HC296073.1 | CS367236.1 | CQ787368.1 | HH827003.1 | DM138433.1 | AX797136.1 | GN033532.1 | CQ814063.1 |
| GM635788.1 | HC295911.1 | CS359938.1 | CQ787334.1 | HH826836.1 | DM143374.1 | AX797103.1 | GN033500.1 | CQ814030.1 |
| GM635756.1 | HC292600.1 | CS359645.1 | CQ787268.1 | HH826499.1 | DM137121.1 | AX796902.1 | GN033468.1 | CQ813998.1 |
| GM635724.1 | HC288843.1 | CS230196.1 | CQ787236.1 | HH822042.1 | DM131194.1 | AX796868.1 | GN033436.1 | CQ813966.1 |
| GM635692.1 | HC295594.1 | BD269150.1 | CQ786932.1 | HH821980.1 | HB441234.1 | AX796758.1 | GN033308.1 | CQ813934.1 |
| GM621947.1 | HC295516.1 | BD268977.1 | CQ784715.1 | HH821912.1 | HB435287.1 | AX795563.1 | GN033244.1 | CQ813902.1 |
| GM630856.1 | HC295452.1 | BD268128.1 | CQ784651.1 | FW379714.1 | HB427178.1 | AX795445.1 | GN033084.1 | AX143449.1 |
| GM630792.1 | HC295193.1 | BD267608.1 | CQ778530.1 | FW379682.1 | HB426468.1 | BD188245.1 | GN032956.1 | AX143385.1 |
| GM630760.1 | HC292050.1 | BD265622.1 | CQ774429.1 | FW379649.1 | HB423122.1 | BD187410.1 | GN032924.1 | AX143257.1 |
| GM630728.1 | HC291882.1 | BD265527.1 | GM647115.1 | FW379617.1 | HB423084.1 | AX786791.1 | GN032892.1 | AX143129.1 |
| GM630696.1 | HC299097.1 | BD263696.1 | GM647083.1 | FW379575.1 | HB403629.1 | AX777477.1 | GN032860.1 | AX143065.1 |
| GM621857.1 | HC294386.1 | BD263460.1 | GM647022.1 | FW377384.1 | HB416463.1 | AX776177.1 | GN032828.1 | AX143001.1 |
| GM621825.1 | HC293822.1 | BD263428.1 | GM627509.1 | FW376187.1 | HB413922.1 | AX773255.1 | GN032796.1 | AX142809.1 |
| GM621793.1 | HC293642.1 | BD251748.1 | GM661297.1 | FW376282.1 | GN116470.1 | AX771050.1 | GN032764.1 | AX142745.1 |
| GM621761.1 | HC290689.1 | BD251241.1 | GM654129.1 | FW381123.1 | GN116438.1 | AX768207.1 | GN032733.1 | CS608369.1 |
| GM657322.1 | HC208417.1 | BD247117.1 | GM654097.1 | FW375248.1 | GN116406.1 | AX766888.1 | GN032701.1 | CS607084.1 |
| GM657290.1 | HC289884.1 | BD246861.1 | GM654065.1 | FW380265.1 | GN116374.1 | AX766738.1 | GN032669.1 | CS607003.1 |
| GM657258.1 | HC289850.1 | BD243979.1 | GM654033.1 | FW379840.1 | GN116342.1 | AX765968.1 | GN032636.1 | CS606913.1 |
| GM657226.1 | HC207916.1 | BD243451.1 | GM654001.1 | U09476.1 | GN116310.1 | AX764648.1 | GN032604.1 | CS606030.1 |
| GM657194.1 | HC207361.1 | BD243415.1 | GM653969.1 | HH736045.1 | GN116042.1 | AX764616.1 | GN032572.1 | CS599850.1 |
| GM649874.1 | HC203217.1 | BD242725.1 | DL174460.1 | HH735921.1 | GN112654.1 | AX764584.1 | GN032477.1 | CS605020.1 |
| GM627360.1 | HC199343.1 | BD242563.1 | DL182872.1 | HH735626.1 | GN115074.1 | AX756516.1 | GN032445.1 | CS604988.1 |
| GM661262.1 | HC197994.1 | BD242465.1 | DL176658.1 | HH755057.1 | GN094581.1 | AX752722.1 | GN032413.1 | CS604924.1 |
| GM661230.1 | HC196131.1 | BD242385.1 | FB343456.1 | HH734127.1 | GN094549.1 | AX205162.1 | GN032381.1 | CS604892.1 |
| GM661198.1 | HC195238.1 | BD238043.1 | DL088082.1 | HH733636.1 | GN094517.1 | AX203367.1 | GN032348.1 | CS604860.1 |
| GM654140.1 | HC193975.1 | BD237511.1 | DL088050.1 | FW359992.1 | GN094485.1 | AX202432.1 | GN032316.1 | CS604828.1 |
| GM654076.1 | HC190395.1 | BD236912.1 | DL091927.1 | FW359902.1 | GN094453.1 | AX193727.1 | GN032284.1 | CS604796.1 |
| GM654044.1 | HC187075.1 | BD235827.1 | DL091863.1 | FW359841.1 | GN113454.1 | AX189288.1 | GN032252.1 | CS604764.1 |
| GM654012.1 | HC090115.1 | BD235376.1 | DL102856.1 | FW359675.1 | DM074458.1 | AX180854.1 | GN032220.1 | CS604732.1 |
| GM653980.1 | HC089558.1 | BD234882.1 | DL102824.1 | FW368818.1 | DM065330.1 | AX180284.1 | GN032188.1 | CS604700.1 |
| GM646987.1 | HC089526.1 | BD234570.1 | DL102792.1 | HD048866.1 | DM064969.1 | AF304150.1 | GN032156.1 | CS604668.1 |
| GM646955.1 | HC089494.1 | BD232424.1 | DL107572.1 | HD033606.1 | GM633752.1 | AX175358.1 | GN032124.1 | CS604634.1 |
| GM646923.1 | HC089462.1 | BD231947.1 | DL102515.1 | HD057694.1 | GM633720.1 | AX173378.1 | GN032092.1 | CS604602.1 |
| GM646891.1 | HC089430.1 | BD231234.1 | DL098005.1 | HD057662.1 | GM633688.1 | AX172946.1 | GN032028.1 | CS604570.1 |
| GM646859.1 | HC089397.1 | BD231140.1 | DL097973.1 | HD057648.1 | GM625821.1 | AX167503.1 | GN031996.1 | CS593085.1 |
| GM646827.1 | HC089365.1 | BD231063.1 | DL097941.1 | HD061470.1 | GM625789.1 | AX167417.1 | GN031964.1 | CS592924.1 |
| GM640024.1 | DL107585.1 | BD229937.1 | DL112631.1 | J03850.1 | GM625757.1 | AX155318.1 | GN031932.1 | CS596760.1 |
| GM639992.1 | DL107521.1 | BD227584.1 | DL112599.1 | M20974.1 | GM624701.1 | AX145737.1 | GN031900.1 | CS598816.1 |
| GM639960.1 | DL107461.1 | BD226454.1 | DL112567.1 | M20407.1 | GM624669.1 | AX145673.1 | GN031868.1 | CS597730.1 |
| GM639928.1 | DL107421.1 | BD225791.1 | DL095727.1 | HC916998.1 | GM624637.1 | AX145641.1 | GN031836.1 | CS584987.1 |
| GM639896.1 | DL102592.1 | BD325567.1 | DL095695.1 | HC918580.1 | GM624605.1 | AX145609.1 | GN031803.1 | CS588485.1 |
| GM639864.1 | DL102560.1 | BD314206.1 | DL095663.1 | FW334408.1 | GM624573.1 | AX145577.1 | GN031706.1 | CS575318.1 |
| GM627317.1 | DL102528.1 | BD313819.1 | DL095631.1 | FW338091.1 | GM624541.1 | AX145545.1 | GN031674.1 | BD429347.1 |
| GM627290.1 | DL098018.1 | BD313628.1 | DL095599.1 | FW337542.1 | GM624444.1 | AX145513.1 | GN031642.1 | BD429008.1 |
| GM627258.1 | DL097986.1 | BD319666.1 | DL095567.1 | FW341709.1 | GM624412.1 | AX145481.1 | GN031610.1 | BD438158.1 |
| GM627194.1 | DL097954.1 | BD319298.1 | DL117404.1 | FW341212.1 | GM624380.1 | AX145449.1 | GN031578.1 | BD396026.1 |
| GM627162.1 | DL112644.1 | BD318880.1 | DL122689.1 | FW335718.1 | GM624348.1 | AX145417.1 | GN031546.1 | BD428078.1 |

| | | | | | | | | |
|---|---|---|---|---|---|---|---|---|
| GM627130.1 | DL112612.1 | BD317372.1 | DL122657.1 | HC868080.1 | GM633651.1 | AX145384.1 | GN031514.1 | BD437664.1 |
| GM661114.1 | DL112580.1 | BD309080.1 | DL122593.1 | HC868028.1 | GM633619.1 | AX145352.1 | GN031482.1 | BD437167.1 |
| GM661082.1 | DL112548.1 | BD308195.1 | DL118403.1 | HC887352.1 | GM633587.1 | AX145320.1 | GN031450.1 | BD394851.1 |
| GM661050.1 | DL112516.1 | BD307651.1 | DL118371.1 | HC867652.1 | GM633555.1 | AX145288.1 | GN031417.1 | BD393735.1 |
| GM661018.1 | DL112484.1 | BD300457.1 | DL108790.1 | HC867561.1 | GM633523.1 | AX145256.1 | GN031385.1 | BD453350.1 |
| GM660986.1 | DL097831.1 | BD293416.1 | DL108726.1 | HC873723.1 | GM651413.1 | AX145224.1 | GN031353.1 | BD453320.1 |
| GM660954.1 | DL097799.1 | BD293147.1 | DL108694.1 | HC880552.1 | GM651381.1 | AX145192.1 | CS716029.1 | BD453288.1 |
| GM653960.1 | DL097767.1 | BD292449.1 | DL099514.1 | HC889127.1 | GM644582.1 | AX145128.1 | CS675426.1 | BD391453.1 |
| GM653928.1 | DL095740.1 | BD291396.1 | DL099482.1 | HC475388.1 | GM644550.1 | AX145096.1 | CS790644.1 | BD388964.1 |
| GM653896.1 | DL095708.1 | BD299786.1 | DL099450.1 | HC474967.1 | GM644518.1 | AX145064.1 | DD400172.1 | BD375875.1 |
| GM653864.1 | DL095644.1 | BD298149.1 | DL089569.1 | HC466643.1 | GM644454.1 | AX145032.1 | DD400139.1 | BD375677.1 |
| GM646759.1 | DL095612.1 | BD296947.1 | DL089537.1 | HC492340.1 | GM644422.1 | AX145000.1 | CS459121.1 | BD373911.1 |
| GM646727.1 | DL095580.1 | BD296889.1 | DL089505.1 | HC466289.1 | GM637424.1 | AX144968.1 | DD367716.1 | BD361642.1 |
| GM646695.1 | DL095548.1 | BD296847.1 | DL089441.1 | HC491798.1 | GM637392.1 | AX144936.1 | DD361270.1 | BD356562.1 |
| GM646663.1 | DL117385.1 | BD295263.1 | DL113543.1 | HC491766.1 | GM637360.1 | AX144903.1 | DD368132.1 | BD356459.1 |
| GM646631.1 | DL117353.1 | BD285360.1 | DL093136.1 | HC491734.1 | GM637328.1 | AX144871.1 | DD361304.1 | BD353561.1 |
| GM639828.1 | DL117321.1 | BD281335.1 | DL093104.1 | HC481598.1 | GM637296.1 | AX144839.1 | CS457891.1 | BD342481.1 |
| GM639796.1 | DL117289.1 | BD287837.1 | DL093072.1 | HC481560.1 | GM637264.1 | AX144807.1 | CS456715.1 | BD341868.1 |
| GM639764.1 | DL117257.1 | BD276937.1 | DL017625.1 | HC491459.1 | GM623727.1 | AX144775.1 | CS453767.1 | BD349812.1 |
| GM639732.1 | DL117225.1 | BD279382.1 | DL022295.1 | HC491058.1 | GM623695.1 | AX144743.1 | DD357935.1 | BD325637.1 |
| GM639700.1 | DL091644.1 | BD274930.1 | DL022263.1 | HC472607.1 | GM623663.1 | AX144711.1 | DD357899.1 | BD325544.1 |
| GM639668.1 | DL111749.1 | BD274659.1 | DL022231.1 | HC479078.1 | GM623631.1 | AX144679.1 | CS448349.1 | BD313685.1 |
| GM639636.1 | DL111717.1 | BD274226.1 | DL025314.1 | FV533438.1 | GM623599.1 | AX144647.1 | A07635.1 | BD319652.1 |
| GM627089.1 | DL111685.1 | BD273683.1 | DL025218.1 | GM661051.1 | GM623567.1 | AX144615.1 | CS446148.1 | BD319471.1 |
| GM627005.1 | DL128896.1 | BD272292.1 | DL038452.1 | GM661019.1 | GM827568.1 | AX144361.1 | DD053318.1 | BD318832.1 |
| GM626993.1 | DL128862.1 | BD271883.1 | DL038420.1 | GM660987.1 | GM658716.1 | AX144297.1 | DD053286.1 | BD308147.1 |
| GM646601.1 | DL128797.1 | BD271050.1 | DL038388.1 | GM660955.1 | GM658652.1 | AX144103.1 | DD047303.1 | BD293048.1 |
| GM646559.1 | DL128764.1 | BD270858.1 | DL038356.1 | GM653961.1 | GM658620.1 | AX144039.1 | DD038499.1 | BD292429.1 |
| DL095200.1 | DL116396.1 | BD270576.1 | DL038324.1 | GM653929.1 | GM658556.1 | AX143973.1 | DD030810.1 | BD291380.1 |
| DL125335.1 | DL116364.1 | DD228689.1 | DL013161.1 | GM653865.1 | GM651364.1 | AX143909.1 | DD027031.1 | AX809451.1 |
| DL125303.1 | DL111643.1 | DD228656.1 | DL013129.1 | GM653781.1 | GM651332.1 | AX143653.1 | DD017631.1 | AX805936.1 |
| DL125271.1 | DL111611.1 | DD228600.1 | DL013097.1 | GM646760.1 | GM651300.1 | AX143589.1 | BD495458.1 | AX805217.1 |
| DL125239.1 | DL111547.1 | DD228442.1 | DL013065.1 | GM646728.1 | GM651268.1 | AX143397.1 | BD434177.1 | AX798871.1 |
| DL125207.1 | DL111515.1 | DD228391.1 | DL013033.1 | GM646696.1 | GM651236.1 | AX143333.1 | BD443796.1 | AX798340.1 |
| DL125175.1 | DL111483.1 | DD227516.1 | DL013001.1 | GM646664.1 | GM651204.1 | AX143269.1 | BD453880.1 | AX798197.1 |
| DL116595.1 | DL101701.1 | DD227392.1 | DL033736.1 | GM680785.1 | GM644405.1 | AX143205.1 | BD453848.1 | AX797703.1 |
| DL116563.1 | DL101669.1 | DD224687.1 | DL042170.1 | GM879330.1 | GM644373.1 | AX142885.1 | BD453816.1 | AX796928.1 |
| DL111842.1 | DL101637.1 | DD224216.1 | DL042138.1 | FB728328.1 | GM644341.1 | AX142757.1 | BD453784.1 | AX796746.1 |
| DL111810.1 | DL101605.1 | DD223910.1 | DL042074.1 | FB722370.1 | GM644309.1 | AX142629.1 | BD453760.1 | AX795588.1 |
| DL111778.1 | DL101573.1 | DD234127.1 | DL035405.1 | GM863399.1 | GM644277.1 | AX142565.1 | BD453728.1 | AX795556.1 |
| DL128893.1 | DL101541.1 | DD232714.1 | DL035373.1 | GM841830.1 | GM644245.1 | AX142499.1 | BD453696.1 | AX795433.1 |
| DL128794.1 | DL097127.1 | DD231448.1 | DL035341.1 | GM841726.1 | GM637247.1 | AX142371.1 | BD453664.1 | BD188846.1 |
| DL111665.1 | DL097095.1 | DD231416.1 | DL031177.1 | GM603719.1 | GM637215.1 | HW261415.1 | BD453632.1 | BD187845.1 |
| DL116361.1 | DL097063.1 | A19503.1 | DL031145.1 | GM706688.1 | GM637183.1 | HW261383.1 | BD453600.1 | AX787438.1 |
| DL111640.1 | DL097031.1 | DD217682.1 | DL023364.1 | GM706656.1 | GM637151.1 | HW261351.1 | BD453550.1 | AX786406.1 |
| DL111608.1 | DL095139.1 | BD183724.1 | DL023332.1 | GM706586.1 | GM637119.1 | HI464669.1 | BD453518.1 | AX777461.1 |
| DL111576.1 | DL095107.1 | BD183121.1 | DL023300.1 | GM706392.1 | GM637087.1 | HI464637.1 | BD453486.1 | AX774559.1 |

| | | | | | | | | |
|---|---|---|---|---|---|---|---|---|
| DL111544.1 | DL095075.1 | BD182775.1 | DL023268.1 | GM706357.1 | GM637055.1 | HI464427.1 | BD453454.1 | AX772852.1 |
| DL111512.1 | DL095043.1 | BD181829.1 | DL023236.1 | GM603510.1 | GM624902.1 | HI528751.1 | BD453422.1 | AX771024.1 |
| DL111480.1 | DL094979.1 | BD181049.1 | DL023204.1 | GM704823.1 | GM624870.1 | HI549572.1 | BD453390.1 | AX768067.1 |
| DL101698.1 | DL094947.1 | GM623536.1 | DL023169.1 | GM600488.1 | GM624830.1 | HI542550.1 | BD453245.1 | AX766358.1 |
| DL101666.1 | DL106665.1 | GM623504.1 | DL023137.1 | FB720264.1 | GM624774.1 | HI504383.1 | BD453213.1 | AX753410.1 |
| DL101634.1 | DL106633.1 | GM623472.1 | DL023105.1 | FB718512.1 | GM632653.1 | HI462738.1 | BD409952.1 | AX752284.1 |
| DL101602.1 | DL106601.1 | GM623440.1 | DL023073.1 | FB718247.1 | GM632577.1 | HH961356.1 | BD398643.1 | BD185651.1 |
| DL097124.1 | DL106569.1 | GM623408.1 | DL023041.1 | FB717768.1 | GM632613.1 | HH961324.1 | BD408088.1 | BD183011.1 |
| DL097092.1 | DL106537.1 | GM623376.1 | DL023009.1 | FB715234.1 | GM658427.1 | HH961260.1 | BD450236.1 | BD180864.1 |
| DL097060.1 | DL106505.1 | GM623344.1 | DL022977.1 | FB706293.1 | GM658463.1 | HH961228.1 | AX814421.1 | AX743502.1 |
| DL097028.1 | DL128702.1 | GM643895.1 | DL010749.1 | FB677437.1 | GM658407.1 | HH975393.1 | AX814389.1 | AX742847.1 |
| DL095136.1 | DL128668.1 | GM643863.1 | DL010717.1 | FB676996.1 | GM658375.1 | HH975091.1 | AX812737.1 | AX722059.1 |
| DL095104.1 | DL128634.1 | GM643831.1 | DL010685.1 | GM061155.1 | GM658343.1 | HH982296.1 | AX799555.1 | AX721694.1 |
| DL095040.1 | DL128602.1 | GM636834.1 | DJ438313.1 | DL260589.1 | GM651151.1 | HH974551.1 | AX798970.1 | BD178179.1 |
| DL095008.1 | DL125072.1 | GM636802.1 | DJ438280.1 | DL260146.1 | GM651119.1 | HH981894.1 | AX798065.1 | AX720260.1 |
| DL094976.1 | DL125040.1 | GM636770.1 | E41545.1 | DL260066.1 | GM651087.1 | HH998078.1 | AX796951.1 | AX717580.1 |
| DL106662.1 | DL125008.1 | GM636738.1 | BD426875.1 | DL259356.1 | GM651055.1 | HH998025.1 | AX663059.1 | A09225.1 |
| DL086037.1 | DL124976.1 | GM636706.1 | BD394715.1 | DL258277.1 | GM651023.1 | HH997981.1 | AX384799.1 | A09019.1 |
| DL086005.1 | DL120951.1 | GM636674.1 | BD394368.1 | DL241353.1 | GM650991.1 | HH999663.1 | BD187478.1 | A08187.1 |
| DL114439.1 | DL120919.1 | GM632266.1 | BD393889.1 | DL240641.1 | GM644192.1 | HH997928.1 | AX787395.1 | A07248.1 |
| DL114407.1 | DL120887.1 | GM632202.1 | BD453339.1 | FB742058.1 | GM644160.1 | HH997843.1 | AX773261.1 | CS608366.1 |
| DL114375.1 | DL120855.1 | GM632170.1 | BD453311.1 | FB748889.1 | GM644128.1 | HH997781.1 | AX771244.1 | CS607950.1 |
| DL114343.1 | DL120823.1 | GM632138.1 | BD453279.1 | FB674312.1 | GM644096.1 | HH997745.1 | BD184728.1 | CS607529.1 |
| DL109350.1 | DL124929.1 | GM632106.1 | BD445538.1 | DL236443.1 | GM644064.1 | HH999465.1 | BD183210.1 | CS600618.1 |
| DL109318.1 | DL124897.1 | GM623335.1 | BD413548.1 | DL233417.1 | GM644032.1 | HH999422.1 | BD181483.1 | CS607460.1 |
| DL109286.1 | DL124865.1 | GM623303.1 | BD388700.1 | DL213333.1 | GM636874.1 | HH999372.1 | BD180845.1 | CS607079.1 |
| DL104552.1 | DL124833.1 | GM623271.1 | BD353552.1 | DL206824.1 | GM632466.1 | HH997660.1 | BD180734.1 | CS606973.1 |
| DL104520.1 | DL124801.1 | GM623239.1 | BD359731.1 | DL219578.1 | GM632434.1 | HH997624.1 | AY181084.1 | CS606911.1 |
| DL093936.1 | DL124769.1 | GM623207.1 | BD342719.1 | DL107520.1 | GM632338.1 | FW332693.1 | BD177400.1 | CS604858.1 |
| DL093904.1 | DL120776.1 | GM623143.1 | BD341813.1 | DL107460.1 | GM623535.1 | FW306533.1 | AX395630.1 | CS593083.1 |
| DL093872.1 | DL120744.1 | GM741845.1 | BD349670.1 | DL102591.1 | GM623503.1 | FW306124.1 | AX391538.1 | CS592905.1 |
| DL090097.1 | DL116213.1 | GM741813.1 | BD325711.1 | DL102559.1 | GM623471.1 | HC769801.1 | AX384556.1 | CS598814.1 |
| DL090065.1 | DL116181.1 | GM658327.1 | BD325511.1 | DL102527.1 | GM623439.1 | HC767563.1 | AX380781.1 | CS597727.1 |
| DL090033.1 | DL116149.1 | GM658295.1 | BD324882.1 | DL098017.1 | GM623407.1 | HC767345.1 | AX371331.1 | CS593362.1 |
| DL090001.1 | DL116117.1 | GM658263.1 | BD314353.1 | DL097985.1 | GM623375.1 | HC757512.1 | AX364545.1 | CS585635.1 |
| DL123352.1 | DL116085.1 | GM658231.1 | BD319281.1 | DL097953.1 | GM623343.1 | HC757154.1 | AX364513.1 | CS584933.1 |
| DL123320.1 | DL116053.1 | GM658199.1 | BD319401.1 | DL112643.1 | GM622889.1 | HC757122.1 | AX364449.1 | CS177186.1 |
| DL123288.1 | DL143517.1 | GM658167.1 | BD318903.1 | DL112611.1 | DL030916.1 | HC755782.1 | AX364417.1 | CS174654.1 |
| DL123256.1 | DL120577.1 | GM650846.1 | BD318124.1 | DL112579.1 | DL030877.1 | HC733772.1 | AX364385.1 | CS176630.1 |
| DL123224.1 | DL120545.1 | GM650814.1 | BD307821.1 | DL112547.1 | DL034335.1 | HC733695.1 | AX364353.1 | CS166439.1 |
| DL123192.1 | DL116014.1 | GM650782.1 | BD306308.1 | DL112515.1 | DL014303.1 | HC742011.1 | AX364226.1 | CS159799.1 |
| DL118906.1 | DL115982.1 | GM658117.1 | BD300350.1 | DL112483.1 | DL014271.1 | HC732411.1 | AX364193.1 | CS159569.1 |
| DL118874.1 | DJ003109.1 | GM658085.1 | BD294175.1 | DL097830.1 | DL014239.1 | HC731347.1 | AX361110.1 | CS158034.1 |
| DL118810.1 | DD495646.1 | GM658053.1 | BD293544.1 | DL097798.1 | DL009387.1 | HC731315.1 | AX358446.1 | CS150843.1 |
| DL109258.1 | DD494967.1 | GM658021.1 | BD291306.1 | DL097766.1 | DL009355.1 | HC731283.1 | AX358191.1 | CS148530.1 |
| DL109226.1 | CS669144.1 | GM657989.1 | BD298439.1 | DL095739.1 | DL009323.1 | HC471744.1 | BD007224.1 | CS144908.1 |
| DL109194.1 | CS667960.1 | GM657957.1 | BD294900.1 | DL095707.1 | DL009291.1 | HC471686.1 | BD006209.1 | CS144312.1 |

| | | | | | | | | |
|---|---|---|---|---|---|---|---|---|
| DL109162.1 | DD464394.1 | GM650765.1 | BD277131.1 | DL095643.1 | DL009259.1 | HC471704.1 | BD004269.1 | CS142067.1 |
| DL109130.1 | CS647718.1 | GM650733.1 | BD279835.1 | DL095611.1 | DL009227.1 | HC471648.1 | BD002014.1 | CS141572.1 |
| DL109098.1 | CS646853.1 | GM650701.1 | CQ755442.1 | DL095579.1 | DL018685.1 | HC471616.1 | BD000448.1 | CS141525.1 |
| DL104364.1 | CS646704.1 | GM643805.1 | CQ754497.1 | DL095547.1 | DL018653.1 | HC730942.1 | AX351220.1 | CS141493.1 |
| DL099790.1 | CS646203.1 | GM643773.1 | CQ754030.1 | DL117384.1 | DL018621.1 | HC471537.1 | AX350316.1 | CS140532.1 |
| DL099758.1 | CS645287.1 | GM643741.1 | AX962026.1 | DL117352.1 | DL009073.1 | HC471596.1 | AX348533.1 | CS132437.1 |
| DL093748.1 | CS645215.1 | GM643709.1 | AX960392.1 | DL117320.1 | DL009041.1 | HC728998.1 | AX347299.1 | CS122480.1 |
| DL093716.1 | CS644262.1 | GM661880.1 | AX960360.1 | DL117288.1 | DL014225.1 | HC727688.1 | AX347265.1 | CS106078.1 |
| DL093684.1 | DD460517.1 | GM661848.1 | AX958371.1 | DL117256.1 | DL014193.1 | HC727402.1 | AX347229.1 | CS179732.1 |
| DL093652.1 | DD460157.1 | GM661816.1 | AX937798.1 | DL117224.1 | DL014161.1 | HC688448.1 | AX347159.1 | CS323674.1 |
| DL093588.1 | DD459944.1 | GM661784.1 | AX924085.1 | DL091643.1 | DL014129.1 | HC491008.1 | AX346279.1 | CS323592.1 |
| DL093556.1 | DD455162.1 | GM661752.1 | AX923397.1 | DL091611.1 | DL014065.1 | HC490976.1 | AX344903.1 | CS322882.1 |
| DL089941.1 | DD459040.1 | GM661720.1 | AX838501.1 | DL091579.1 | DL014033.1 | HC490944.1 | AY774813.1 | CS330143.1 |
| DL089909.1 | DD453829.1 | GM654726.1 | AX828672.1 | DL087968.1 | DL026123.1 | HC490912.1 | AY774698.1 | CS322140.1 |
| DL089877.1 | DD457863.1 | GM654694.1 | AX824329.1 | DL087936.1 | DL026091.1 | HC490880.1 | AY774642.1 | DD261129.1 |
| DL089845.1 | DD456898.1 | GM654662.1 | AX823795.1 | DL087904.1 | DL026059.1 | HC490840.1 | AY774476.1 | DD261001.1 |
| DL089813.1 | DD451433.1 | GM654630.1 | AX817768.1 | DL087872.1 | DL026027.1 | HC490808.1 | AY774413.1 | DD259344.1 |
| DL089781.1 | CS631732.1 | GM654598.1 | AX816158.1 | DL087840.1 | DL025995.1 | HC490776.1 | AY774354.1 | DD266535.1 |
| DL140939.1 | CS642305.1 | GM654565.1 | AX816034.1 | DL087808.1 | DL025963.1 | HC490744.1 | AY774305.1 | CS302574.1 |
| DL046008.1 | CS642210.1 | GM647572.1 | AX814399.1 | DL121767.1 | DL018540.1 | HC490712.1 | AY774251.1 | DD251987.1 |
| DL045976.1 | CS631226.1 | GM627901.1 | AX799565.1 | DL121735.1 | DL018476.1 | HC490680.1 | AY774197.1 | DD251592.1 |
| DL049714.1 | CS630927.1 | GM627869.1 | AX798504.1 | DL121703.1 | DL018444.1 | HC490616.1 | AY774136.1 | CQ859611.1 |
| DL049682.1 | CS627772.1 | GM627805.1 | AX797694.1 | DL121639.1 | DL018412.1 | HC490584.1 | AY774067.1 | CQ858159.1 |
| DL045674.1 | CS632688.1 | GM627773.1 | AX144984.1 | DL121607.1 | DL014010.1 | HC490516.1 | AY774004.1 | CQ857420.1 |
| DL041859.1 | CS632430.1 | GM627741.1 | AX144952.1 | DL087780.1 | DL013978.1 | HC490445.1 | AY167891.1 | CQ856027.1 |
| DL041827.1 | CS632182.1 | GM871611.1 | AX144919.1 | DL087748.1 | DL013946.1 | HC490349.1 | AY017183.1 | CQ855925.1 |
| DL041795.1 | CS626888.1 | GM870421.1 | AX144887.1 | DL087716.1 | DL013914.1 | HC490317.1 | HC083740.1 | CQ855148.1 |
| DL041763.1 | CS623725.1 | GM661691.1 | AX144855.1 | DL087684.1 | DL013846.1 | HC490285.1 | HC083708.1 | CQ854233.1 |
| DL041731.1 | CS623661.1 | GM661659.1 | AX144823.1 | DL087652.1 | DL030110.1 | HC490252.1 | HC083531.1 | CQ849486.1 |
| DL041699.1 | CS602181.1 | GM661627.1 | AX144791.1 | CQ867011.1 | DL030078.1 | HC472306.1 | DM460864.1 | CQ834129.1 |
| DL037748.1 | CS611206.1 | GM661595.1 | AX144759.1 | CQ861215.1 | DL030046.1 | HC472273.1 | FB775672.1 | CQ831414.1 |
| DL037716.1 | CS610992.1 | GM661563.1 | AX144727.1 | CQ860280.1 | DL030014.1 | HC472241.1 | FB766245.1 | CQ830720.1 |
| DL049609.1 | CS607926.1 | GM661531.1 | AX144695.1 | CQ859650.1 | DL029982.1 | HC472209.1 | FB766135.1 | CQ829827.1 |
| DL049481.1 | CS607838.1 | GM654537.1 | AX144663.1 | CQ859618.1 | DL029950.1 | HC472177.1 | FB765672.1 | CQ828076.1 |
| DD215926.1 | CS607754.1 | GM654505.1 | AX144631.1 | CQ858080.1 | DL046639.1 | HC471945.1 | FB764682.1 | CQ827549.1 |
| DD215894.1 | CS607537.1 | GM654473.1 | AX144521.1 | CQ857499.1 | DL046607.1 | HC047302.1 | FB743941.1 | CQ826846.1 |
| DD215862.1 | CS607093.1 | GM654441.1 | AX144393.1 | CQ855754.1 | DL046575.1 | HC047046.1 | FB743908.1 | CQ824421.1 |
| DD215830.1 | CS607012.1 | GM654409.1 | AX144199.1 | CQ849417.1 | DL046543.1 | HC047014.1 | FB743876.1 | CQ821237.1 |
| DD215798.1 | CS599886.1 | GM654377.1 | AX144135.1 | CQ847389.1 | DL046511.1 | HC046982.1 | FB743844.1 | CQ818937.1 |
| DD215766.1 | CS606924.1 | GM647384.1 | AX143941.1 | CQ847234.1 | DL046479.1 | HC046950.1 | FB743790.1 | CQ818562.1 |
| AX575307.1 | CS606845.1 | GM647352.1 | AX143685.1 | CQ840696.1 | DL042657.1 | HC046918.1 | FB742914.1 | CQ817012.1 |
| AX573164.1 | CS605117.1 | GM647320.1 | AX143621.1 | CQ828165.1 | DL042625.1 | HC040757.1 | FB708871.1 | CQ816971.1 |
| AX556825.1 | CS605085.1 | GM647288.1 | AX143365.1 | CQ827556.1 | DL042593.1 | HC046858.1 | GM952838.1 | CQ816933.1 |
| AX544467.1 | CS605053.1 | GM647256.1 | AX143301.1 | CQ826863.1 | DL038669.1 | HC046826.1 | GM890256.1 | CQ815742.1 |
| AX543885.1 | CS599814.1 | GM647224.1 | AX142853.1 | CQ818569.1 | DL038637.1 | HC046794.1 | GM963588.1 | CQ814527.1 |
| AX536409.1 | CS605026.1 | GM640421.1 | AX142789.1 | CQ818013.1 | DL038605.1 | HC046762.1 | GM889471.1 | CQ814092.1 |
| AX529526.1 | CS604994.1 | GM640389.1 | AX142597.1 | CQ816940.1 | DL034140.1 | HC046730.1 | GM008879.1 | CQ814060.1 |

| | | | | | | | | |
|---|---|---|---|---|---|---|---|---|
| AX528112.1 | CS604962.1 | GM640357.1 | AX142339.1 | CQ815751.1 | DL034502.1 | HC046698.1 | FB661313.1 | CQ814027.1 |
| AX526823.1 | CS604930.1 | GM627714.1 | AX142275.1 | CQ815056.1 | DL029911.1 | HC046681.1 | FB983221.1 | CQ813995.1 |
| AX524908.1 | CS604866.1 | GM627682.1 | AX142211.1 | CQ814099.1 | DL029879.1 | HC046649.1 | FB983169.1 | CQ813963.1 |
| AX523918.1 | CS604834.1 | GM627650.1 | AX142083.1 | CQ814067.1 | DL029847.1 | HC046617.1 | GM681760.1 | CQ813931.1 |
| AX523636.1 | CS604802.1 | GM627558.1 | AX141887.1 | CQ814034.1 | DL029815.1 | HC046585.1 | GM731780.1 | CQ813899.1 |
| A33401.1 | CS604770.1 | GM661499.1 | AX141823.1 | CQ814002.1 | DL029751.1 | HC046553.1 | GM840666.1 | CQ813867.1 |
| AX179490.1 | CS604738.1 | GM661467.1 | AX141759.1 | CQ813970.1 | DL025941.1 | HC046521.1 | CS607541.1 | CQ813835.1 |
| AX179428.1 | CS604706.1 | GM661435.1 | AX141631.1 | CQ813938.1 | DL025909.1 | HC046489.1 | CS600339.1 | CQ813803.1 |
| AX027962.1 | CS604674.1 | GM661403.1 | AX141567.1 | CQ813906.1 | DL025877.1 | HC046457.1 | CS607103.1 | CQ813770.1 |
| A09545.1 | CS604640.1 | DL047873.1 | AX141375.1 | CQ813874.1 | DL025845.1 | HC046425.1 | CS607025.1 | CQ813730.1 |
| AX521541.1 | CS604608.1 | DL044019.1 | AX138282.1 | CQ813842.1 | DL025813.1 | HC046393.1 | CS606928.1 | CQ802869.1 |
| AX521509.1 | CS604576.1 | DL043987.1 | AX137835.1 | CQ813810.1 | DL025781.1 | HC046361.1 | CS604710.1 | CQ802129.1 |
| AF430195.1 | CS597523.1 | DL043955.1 | AX134957.1 | CQ813777.1 | DL069491.1 | HC046329.1 | CS592977.1 | CQ800992.1 |
| AF430163.1 | CS593091.1 | DL043923.1 | AX134400.1 | CQ813741.1 | DL018352.1 | HC046078.1 | CS592822.1 | CQ798795.1 |
| AX505215.1 | CS592948.1 | DL043891.1 | AX119322.1 | CQ813588.1 | DL018320.1 | HC046046.1 | CS592340.1 | CQ798242.1 |
| AX505151.1 | CS592380.1 | DL040074.1 | AX107118.1 | AX752162.1 | DJ088992.1 | HC046014.1 | CS589285.1 | CQ796850.1 |
| AX497098.1 | CS599610.1 | DL040042.1 | AX100342.1 | BD183119.1 | DJ082698.1 | HC046254.1 | CS585681.1 | CQ795484.1 |
| BD139555.1 | CS589277.1 | DL040010.1 | AX097931.1 | BD182773.1 | DJ082577.1 | HC046222.1 | CS588637.1 | CQ790241.1 |
| BD138746.1 | CS597739.1 | DL039978.1 | AX097509.1 | BD180900.1 | DJ081985.1 | HC046158.1 | CS574801.1 | CQ789538.1 |
| BD138653.1 | CS593370.1 | DL039946.1 | HW155830.1 | AX744004.1 | DJ078396.1 | HC046126.1 | CS573051.1 | CQ787515.1 |
| BD138406.1 | CS585655.1 | DL039914.1 | HW155707.1 | AX742856.1 | DJ080275.1 | HC046008.1 | CS580002.1 | CQ787483.1 |
| BD138370.1 | CS585081.1 | DL039883.1 | HI657152.1 | AX739985.1 | DJ081434.1 | HC045976.1 | CS570683.1 | CQ787451.1 |
| BD135859.1 | CS584690.1 | DL047829.1 | HI660315.1 | AX722066.1 | DJ079781.1 | HC045912.1 | CS546667.1 | CQ787411.1 |
| BD133197.1 | CS588493.1 | DL047797.1 | FW503729.1 | AX721699.1 | DJ071525.1 | HC045880.1 | CS559286.1 | CQ787371.1 |
| BD131216.1 | CS574797.1 | DL047765.1 | FW381568.1 | BD178184.1 | DJ066387.1 | HC045816.1 | BD413660.1 | A12644.1 |
| BD130804.1 | CS573047.1 | DL047701.1 | FW381492.1 | BD177377.1 | DJ066323.1 | HC045784.1 | BD360147.1 | A12323.1 |
| BD130772.1 | CS579864.1 | DL047669.1 | FW375613.1 | AX718198.1 | DJ066289.1 | HC045752.1 | BD356482.1 | GM638787.1 |
| BD130739.1 | CS570749.1 | DL039774.1 | FW375398.1 | AX710314.1 | DJ066255.1 | HC045720.1 | BD342579.1 | GM638755.1 |
| BD130624.1 | CS570598.1 | DL039742.1 | FW375285.1 | AX708517.1 | DJ063551.1 | HC045690.1 | BD341995.1 | GM626112.1 |
| BD130489.1 | DD400132.1 | DL036086.1 | HH736079.1 | AX703426.1 | DJ065983.1 | HC045658.1 | BD341627.1 | GM626080.1 |
| BD107710.1 | CS463230.1 | DL036054.1 | HH733736.1 | AX701593.1 | DJ068024.1 | HC045626.1 | BD338965.1 | GM626048.1 |
| BD105788.1 | CS461221.1 | DL035990.1 | HD119572.1 | AX701061.1 | DJ065235.1 | HC045594.1 | BD325670.1 | GM626016.1 |
| BD087474.1 | CS459114.1 | DL035958.1 | FW365540.1 | AX699467.1 | DJ067021.1 | HC045562.1 | BD300467.1 | GM625984.1 |
| BD085729.1 | DD361295.1 | DL035926.1 | FW368769.1 | AX699435.1 | DJ055586.1 | HC045530.1 | BD294538.1 | GM625952.1 |
| BD082986.1 | DD367629.1 | DL031922.1 | FW368171.1 | AX685157.1 | DJ061267.1 | DM379706.1 | BD290006.1 | GM038284.1 |
| A08195.1 | DD367339.1 | DL031890.1 | FW362403.1 | AX682976.1 | DJ055290.1 | HC035727.1 | BD299620.1 | FB775379.1 |
| A07790.1 | DD370036.1 | DL031858.1 | FW351220.1 | AX671521.1 | DJ061125.1 | HC035625.1 | BD295266.1 | FB774881.1 |
| A07251.1 | CS457159.1 | DL031826.1 | FW351157.1 | AX670725.1 | DJ054269.1 | HC037058.1 | BD285365.1 | FB765851.1 |
| A05993.1 | CS456708.1 | DL031794.1 | HD122369.1 | AX665500.1 | DJ060347.1 | HC025873.1 | BD277302.1 | DL101628.1 |
| A10544.1 | CS451672.1 | DL031762.1 | HD115794.1 | AX664349.1 | DJ060305.1 | HC025476.1 | BD271906.1 | DL097118.1 |
| A07982.1 | CS450921.1 | DL027857.1 | HD084540.1 | AX662238.1 | DJ060271.1 | HC025444.1 | BD271122.1 | DL097086.1 |
| A04822.1 | CS450540.1 | DL027793.1 | HD113819.1 | AX658890.1 | DJ060225.1 | HC025412.1 | AX963269.1 | DL097054.1 |
| A01732.1 | DD361044.1 | DL027761.1 | HC504620.1 | AX657150.1 | DJ056893.1 | HC010448.1 | AX958745.1 | DL095130.1 |
| A05198.1 | DD357892.1 | DL023949.1 | HC504259.1 | AX657117.1 | AX144901.1 | HC010387.1 | BD010846.1 | DL095098.1 |
| A04245.1 | DD357214.1 | DL023917.1 | HC504176.1 | AX657080.1 | AX144869.1 | HC010184.1 | AX957212.1 | DL095034.1 |
| A01347.1 | DD355756.1 | DL023885.1 | HC471035.1 | BD175985.1 | AX144837.1 | HC010003.1 | AX938909.1 | DL095002.1 |
| A00196.1 | CS449869.1 | DL023853.1 | HC500967.1 | BD175652.1 | AX144805.1 | HC007834.1 | AX926920.1 | DL094970.1 |

| | | | | | | | | |
|---|---|---|---|---|---|---|---|---|
| M24176.1 | A14569.1 | DL023821.1 | HC499853.1 | BD175612.1 | AX144773.1 | HC007682.1 | AX923417.1 | DL106656.1 |
| M28044.1 | HC007747.1 | DL023789.1 | FW301301.1 | BD174682.1 | AX144741.1 | HC007650.1 | AX923384.1 | DL106560.1 |
| M60893.1 | HC007715.1 | DL020774.1 | FW300530.1 | BD174397.1 | AX144709.1 | HC007622.1 | AX832722.1 | DL106528.1 |
| M13109.1 | HC007683.1 | DL020742.1 | HC486489.1 | AJ005289.1 | AX144677.1 | HC007558.1 | AX827178.1 | DL128726.1 |
| J02540.1 | HC007651.1 | DL020710.1 | HC486425.1 | AX643688.1 | AX144645.1 | HC010739.1 | AX824589.1 | DL128659.1 |
| M14441.1 | HC007623.1 | DL020678.1 | HC485139.1 | AX642226.1 | AX144613.1 | GN067643.1 | AX824348.1 | DL125095.1 |
| M10914.1 | HC007591.1 | DL020646.1 | HC484124.1 | AX641861.1 | AX144549.1 | GN065243.1 | AX823782.1 | DL125063.1 |
| LC732346.1 | HC007559.1 | DL020614.1 | HC491750.1 | BD172275.1 | AX144421.1 | GN061279.1 | AX814386.1 | DL125031.1 |
| MF989992.1 | HC007527.1 | DL016211.1 | HC491642.1 | AX601457.1 | AX144293.1 | GN052463.1 | AX802324.1 | DL124999.1 |
| AF220498.1 | HC010740.1 | DL016179.1 | HC481631.1 | AX344643.1 | AX144227.1 | GN052431.1 | AX800025.1 | DL124967.1 |
| AH002278.2 | DM377061.1 | DL016147.1 | FV533474.1 | AX344129.1 | AX144035.1 | GN052388.1 | AX799796.1 | DL139746.1 |
| M16211.1 | DM375507.1 | DL011593.1 | FV531705.1 | AX342855.1 | AX143521.1 | GN052356.1 | AX798029.1 | DL124952.1 |
| M14485.1 | HC005249.1 | DL011561.1 | FV522843.1 | AX339373.1 | AX143457.1 | GN059741.1 | AX797330.1 | DL124920.1 |
| DQ250244.1 | HC003671.1 | DL011529.1 | FV522803.1 | AX338576.1 | AX143329.1 | GN048060.1 | AX796947.1 | DL124888.1 |
| DQ250180.1 | DM370969.1 | DL011497.1 | FV522620.1 | AX328287.1 | AX143201.1 | DM025360.1 | A32359.1 | DL120767.1 |
| AY569330.1 | DM370937.1 | DL011465.1 | FV528439.1 | AX327962.1 | AX143137.1 | DM012882.1 | A30348.1 | DL120735.1 |
| AF074853.1 | DM370905.1 | DL011433.1 | FV534406.1 | AX326766.1 | AX143009.1 | DM024191.1 | A06242.1 | DL116172.1 |
| S39590.1 | DM370834.1 | DL031647.1 | DM370683.1 | AX319637.1 | AX142753.1 | DM028074.1 | A21012.1 | DL116140.1 |
| KJ598131.1 | DM370800.1 | DL031615.1 | DM370651.1 | AX268696.1 | A26379.1 | DM022389.1 | DL023282.1 | DL116108.1 |
| HW408642.1 | DM370667.1 | DL038699.1 | DM370619.1 | AX260028.1 | A22984.1 | DM022307.1 | DL023250.1 | DL116076.1 |
| HW408610.1 | DM370635.1 | DL035075.1 | DM370587.1 | AX258938.1 | A23022.1 | DM038577.1 | DL023218.1 | DL116044.1 |
| HW408482.1 | DM370603.1 | DL035043.1 | DM370555.1 | AX304315.1 | A23859.1 | DM022276.1 | DL023186.1 | DL120568.1 |
| HW390754.1 | DM370571.1 | DL034895.1 | DM370523.1 | AX303583.1 | A22235.1 | DM022208.1 | DL020171.1 | DL120536.1 |
| HW399541.1 | DM370539.1 | DL034892.1 | DM370491.1 | AX301153.1 | A16276.1 | DM022154.1 | DL020139.1 | DL115909.1 |
| HW399333.1 | DM370507.1 | DL034860.1 | DM370459.1 | AX299856.1 | A21424.1 | DM015981.1 | DL020107.1 | DL115877.1 |
| HW399230.1 | DM370475.1 | DL034836.1 | DM205450.1 | AX297668.1 | A21208.1 | DM015949.1 | DL020075.1 | DL115845.1 |
| HW392025.1 | DM367583.1 | DL030728.1 | DM371921.1 | AX287556.1 | A20283.1 | DM027043.1 | DL020043.1 | DL106455.1 |
| HW390667.1 | DM371970.1 | DL030696.1 | HC003078.1 | AX286554.1 | A19455.1 | GN045770.1 | DL020011.1 | DL128468.1 |
| HW390526.1 | DM204176.1 | DL030536.1 | HB865041.1 | AX283691.1 | A13670.1 | GN042719.1 | DL015448.1 | DL124676.1 |
| HW389028.1 | HC001593.1 | DL026727.1 | HB864963.1 | AX283225.1 | A18342.1 | GN041846.1 | DL010990.1 | DL124644.1 |
| HW388638.1 | HB865974.1 | DL026695.1 | HB865001.1 | AX279744.1 | A17478.1 | GN041667.1 | DL010958.1 | DL124612.1 |
| HW387857.1 | HB865370.1 | DL026663.1 | HB864945.1 | AX282192.1 | A17033.1 | GN037322.1 | DL010926.1 | DL124580.1 |
| HW387470.1 | HB865025.1 | DL026631.1 | HB864913.1 | AX280254.1 | A16027.1 | GN034790.1 | DL010894.1 | DL096915.1 |
| HW098204.1 | HB864979.1 | DL026599.1 | HB864881.1 | AX279957.1 | A14893.1 | GN030749.1 | DL010862.1 | DL102915.1 |
| HW102453.1 | HB864985.1 | DL026567.1 | HB864849.1 | AX279738.1 | A12751.1 | GN030717.1 | DL010830.1 | FB317773.1 |
| HW059859.1 | HB864929.1 | DL022755.1 | HB864817.1 | DD406928.1 | A12356.1 | GN030653.1 | DL047058.1 | CS368324.1 |
| HW061888.1 | HB864897.1 | DL022723.1 | HB864785.1 | DD406129.1 | A12024.1 | GN030621.1 | DL023151.1 | CS368260.1 |
| HW061855.1 | HB864865.1 | DL022691.1 | HB864753.1 | DD402581.1 | A11189.1 | GN030589.1 | DL023119.1 | CS368196.1 |
| HW061823.1 | HB864833.1 | DL022659.1 | HB859734.1 | DD405835.1 | A10368.1 | GN030557.1 | DL023087.1 | CS368068.1 |
| HW062170.1 | HB864801.1 | DL022627.1 | HB866543.1 | DD405803.1 | A16762.1 | GN030525.1 | DL023055.1 | CS367940.1 |
| HV700730.1 | HB864769.1 | DL022595.1 | HC000337.1 | DD402375.1 | A08021.1 | GN030493.1 | DL023023.1 | CS367684.1 |
| HV700698.1 | HB864261.1 | DL019580.1 | HB999694.1 | DD402343.1 | A06447.1 | GN030461.1 | DL022991.1 | CS367556.1 |
| HV694742.1 | HB866976.1 | DL019548.1 | HB976703.1 | DD402311.1 | A06011.1 | GN030428.1 | DL019944.1 | EU099578.1 |
| HV694710.1 | HB859190.1 | DL019516.1 | DM201538.1 | DD402279.1 | A05144.1 | GN030301.1 | DL019912.1 | DL075895.1 |
| HV694641.1 | HB866516.1 | DL019484.1 | DM193209.1 | DD402247.1 | A01180.1 | GN030269.1 | DL019816.1 | DL079085.1 |
| HV700250.1 | HB866104.1 | DL019452.1 | DM199591.1 | DD402215.1 | A01737.1 | GN030237.1 | DL015413.1 | DL079045.1 |
| HV693381.1 | HC000878.1 | DL019420.1 | DM194718.1 | DD402183.1 | A01350.1 | GN030205.1 | DL015381.1 | DL078961.1 |

| | | | | | | | | |
|---|---|---|---|---|---|---|---|---|
| HV699826.1 | HB999505.1 | DL015017.1 | DM189628.1 | DD402151.1 | M67487.1 | GN030109.1 | DL015349.1 | DL078929.1 |
| HV699498.1 | HB976761.1 | DL014985.1 | HB840185.1 | DD402119.1 | M19736.1 | GN029981.1 | DL015317.1 | DL078898.1 |
| HV699154.1 | HB976182.1 | DL014953.1 | HB848038.1 | DD402087.1 | M33414.1 | GN029949.1 | DL015285.1 | DL073096.1 |
| HV699081.1 | HB396999.1 | DL014921.1 | HB839616.1 | DD402055.1 | M14739.1 | GN029917.1 | DL010795.1 | DL013507.1 |
| HV698229.1 | HB396569.1 | DL014889.1 | HB847595.1 | DD405775.1 | L35895.1 | GN029851.1 | DL010763.1 | DL013475.1 |
| HV690017.1 | HB394313.1 | DL014857.1 | HB847222.1 | DD405743.1 | LT799420.1 | GN029819.1 | DL010731.1 | DL013443.1 |
| HV695920.1 | HB394249.1 | DL010335.1 | HB838873.1 | DD405711.1 | AH002290.2 | GN013380.1 | DL010699.1 | DL013411.1 |
| HV688791.1 | HB394169.1 | DL010303.1 | HB846710.1 | DD405679.1 | M17537.1 | GN010223.1 | DL010667.1 | DL013255.1 |
| HV600527.1 | HB393648.1 | DL010271.1 | HB838303.1 | DD405647.1 | DQ250247.1 | GN033553.1 | DL010635.1 | DL013223.1 |
| HV579745.1 | DM119387.1 | DL010207.1 | HB837564.1 | DD405595.1 | DQ250215.1 | GN033521.1 | DL010603.1 | DL013191.1 |
| HV585124.1 | DM118036.1 | DL034672.1 | HB845856.1 | DD405563.1 | AY521452.1 | GM649185.1 | DL047027.1 | DL017980.1 |
| FW590846.1 | GM715170.1 | DL034640.1 | HB826494.1 | DD405499.1 | AY328472.1 | GM649153.1 | DL046995.1 | DL017948.1 |
| FW590730.1 | GM616293.1 | DL034576.1 | HB845342.1 | DD405467.1 | AF003716.1 | GM649121.1 | DL046963.1 | DL017916.1 |
| FW589163.1 | GM604353.1 | DL034544.1 | HB844795.1 | DD405435.1 | S63967.1 | GM649089.1 | DL046931.1 | DL017789.1 |
| FW593447.1 | GM038801.1 | DL034512.1 | HB844360.1 | DD402029.1 | KJ668038.1 | GM649057.1 | DL046899.1 | DL017757.1 |
| FW586440.1 | GM000647.1 | BD279381.1 | HB836756.1 | DD401997.1 | HW408672.1 | GM649025.1 | DL046867.1 | DL017725.1 |
| FW592665.1 | HB386583.1 | BD274929.1 | HB844059.1 | DD401965.1 | HW408613.1 | GM648993.1 | DL043045.1 | DL017693.1 |
| FW592633.1 | HB341592.1 | BD274658.1 | HB836212.1 | DD401904.1 | HW408581.1 | GM642158.1 | DL043013.1 | DL017661.1 |
| FW577823.1 | HB338953.1 | BD274225.1 | HB825527.1 | DD401872.1 | HW408549.1 | GM642126.1 | DL042981.1 | DL017629.1 |
| FW577428.1 | HA641620.1 | BD274152.1 | HB843089.1 | DD401840.1 | HW408517.1 | GM642094.1 | DL042949.1 | DL022267.1 |
| FW576791.1 | HA641155.1 | BD273682.1 | GM625184.1 | DD401808.1 | HW408454.1 | GM642062.1 | DL042917.1 | DL022235.1 |
| FW576756.1 | HA639882.1 | BD271876.1 | GM625152.1 | DD405394.1 | HW353849.1 | GM642030.1 | DL042885.1 | DL022203.1 |
| FW576722.1 | HA639229.1 | BD270857.1 | GM624126.1 | DD401632.1 | HW353817.1 | GM634843.1 | DL039068.1 | DL035473.1 |
| FW576338.1 | DM102730.1 | BD270552.1 | GM623998.1 | DD401600.1 | HW353785.1 | GM634811.1 | DL039036.1 | DL035441.1 |
| FW575883.1 | DM106432.1 | BD270123.1 | GM623966.1 | DD401568.1 | HW353632.1 | GM634779.1 | DL039004.1 | DL035409.1 |
| FW575359.1 | DM092258.1 | DD231060.1 | GM658909.1 | DD401536.1 | HW353536.1 | GM629877.1 | DL038972.1 | DL035377.1 |
| FW571611.1 | DM110452.1 | DD230061.1 | GM658877.1 | DD401504.1 | HW353469.1 | GM629813.1 | DL038940.1 | DL035345.1 |
| FW572719.1 | DM091596.1 | DD228655.1 | HV515410.1 | DD401472.1 | HW352848.1 | GM629781.1 | DL038908.1 | DL031181.1 |
| FW570256.1 | DM094907.1 | DD228599.1 | HV515378.1 | CS480730.1 | HW341644.1 | GM629749.1 | DL035284.1 | DL031149.1 |
| FW573467.1 | L09133.1 | DD227515.1 | HV515346.1 | CS479245.1 | HW341236.1 | GM629717.1 | DL035252.1 | DL027244.1 |
| FW574378.1 | DM063291.1 | DD227391.1 | HV515282.1 | CS476469.1 | HW351156.1 | GM669618.1 | DL035220.1 | DL027212.1 |
| FW571919.1 | DM058789.1 | DD224669.1 | HV515250.1 | CS469178.1 | HW350983.1 | GM669374.1 | DL035188.1 | DL027180.1 |
| FW571875.1 | DM057725.1 | DD224215.1 | HV511462.1 | CS467571.1 | HW350940.1 | GM656126.1 | DL035156.1 | HB843679.1 |
| FW571752.1 | DM041707.1 | DD223909.1 | HV507915.1 | CS464686.1 | HW350686.1 | GM656094.1 | DL035124.1 | HB835799.1 |
| HI968135.1 | DM040541.1 | DD234126.1 | HV508677.1 | CS464626.1 | HW350652.1 | GM656062.1 | DL031120.1 | HB843103.1 |
| HI967430.1 | DM045537.1 | DD233503.1 | HV508642.1 | CS464830.1 | HW350557.1 | GM656030.1 | DL031088.1 | HB835521.1 |
| HI004371.1 | DM056695.1 | DD232695.1 | HV508610.1 | CS464498.1 | HW340187.1 | GM655998.1 | DL031024.1 | HB824120.1 |
| HI004243.1 | DM045265.1 | DD231499.1 | HV508578.1 | DD392049.1 | HW349973.1 | GM655966.1 | DL030992.1 | HB842481.1 |
| HI002275.1 | DM044982.1 | DD231447.1 | HV508546.1 | DD147761.1 | HW339557.1 | GM648973.1 | DL027149.1 | HB850384.1 |
| HI002238.1 | DM039613.1 | DD231415.1 | HV508514.1 | DD147729.1 | HW349884.1 | GM648941.1 | DL027119.1 | HB841719.1 |
| HI002188.1 | DM060681.1 | A19491.1 | HV508486.1 | DD147697.1 | HW349770.1 | GM648909.1 | DL027087.1 | HB841303.1 |
| HI002145.1 | DM044350.1 | DD221340.1 | HV512288.1 | DD147150.1 | HW349545.1 | GM648877.1 | DL027055.1 | HB840793.1 |
| HI002107.1 | GN067896.1 | DD212974.1 | HV512256.1 | DD122265.1 | HW348005.1 | GM648845.1 | DL027023.1 | HB828768.1 |
| HI000452.1 | GN067864.1 | DD213976.1 | HV512224.1 | DD137242.1 | HW347799.1 | GM648813.1 | DL026991.1 | HB648682.1 |
| HI000404.1 | GN067832.1 | DD213883.1 | HV512192.1 | DD132691.1 | HW347767.1 | GM641914.1 | DL026959.1 | HB647050.1 |
| HI000359.1 | GN067800.1 | DD213851.1 | HV512160.1 | DD132290.1 | HW347703.1 | GM641850.1 | DL030918.1 | HB645741.1 |
| HI553290.1 | GN067740.1 | DD213819.1 | HV512128.1 | DD093952.1 | HW347639.1 | GM634662.1 | DL030879.1 | HB645709.1 |

| | | | | | | | | |
|---|---|---|---|---|---|---|---|---|
| HI553258.1 | GN067708.1 | DD216452.1 | HV491731.1 | DD106665.1 | HW347606.1 | GM634630.1 | DL030847.1 | HB645675.1 |
| HI553218.1 | GN067644.1 | DD216420.1 | HV504791.1 | DD092958.1 | HW262776.1 | GM634598.1 | DL030815.1 | HB855097.1 |
| HI553186.1 | GN063763.1 | DD213770.1 | HV504727.1 | DD119262.1 | GM657289.1 | GM634566.1 | DL030751.1 | HB850750.1 |
| HI002091.1 | GN052465.1 | DD213738.1 | HV504695.1 | DD118651.1 | GM657257.1 | GM634534.1 | DL026942.1 | HB850591.1 |
| HI539177.1 | GN052432.1 | DD213706.1 | HV504663.1 | DD091230.1 | GM657225.1 | GM634502.1 | DL026910.1 | HB855985.1 |
| HI583970.1 | GN052357.1 | DD213642.1 | HV504631.1 | DD061280.1 | GM657193.1 | GM634470.1 | CS798099.1 | HB855896.1 |
| HI583871.1 | GN048962.1 | DD213610.1 | HV504599.1 | DD118402.1 | GM657161.1 | GM629665.1 | CS715320.1 | DM179952.1 |
| HI207666.1 | DL105611.1 | DD213592.1 | HV504982.1 | DD118108.1 | GM642969.1 | GM841622.1 | CS805421.1 | DM179145.1 |
| HI003698.1 | DL105579.1 | DD213560.1 | HV455642.1 | DD117210.1 | GM642936.1 | GM840772.1 | CS675246.1 | DM178931.1 |
| HI002072.1 | DL105547.1 | DD213528.1 | HV503804.1 | DD116351.1 | GM642904.1 | FB711262.1 | CS809321.1 | DM170851.1 |
| HI002022.1 | DL105515.1 | DD220702.1 | HI401260.1 | DD090310.1 | GM642872.1 | GM967129.1 | CS790244.1 | DM170818.1 |
| HI001939.1 | DL100902.1 | DD216040.1 | HI401110.1 | DD089595.1 | GM642840.1 | GM840426.1 | CS791229.1 | DM178267.1 |
| HI000271.1 | DL100870.1 | DD216008.1 | HI642980.1 | DD103268.1 | GM635658.1 | GM041781.1 | CS791005.1 | DM188215.1 |
| HI000231.1 | DL100838.1 | DD215930.1 | HI638905.1 | DD103193.1 | GM635626.1 | FB709519.1 | CS790968.1 | DM188008.1 |
| HI000178.1 | DL100806.1 | DD215898.1 | HI637689.1 | DD102577.1 | GM635594.1 | GM040347.1 | CS792486.1 | HB491786.1 |
| HI000131.1 | DL100774.1 | DD215866.1 | HI378566.1 | DD102446.1 | GM635562.1 | CS728684.1 | DJ031157.1 | DL467563.1 |
| HI204940.1 | DL100742.1 | DD215834.1 | HD082405.1 | DD052021.1 | GM635530.1 | CS728652.1 | DJ044960.1 | GM652749.1 |
| HI204459.1 | DL096328.1 | DD215802.1 | HD081717.1 | DD057913.1 | GM635498.1 | CS727586.1 | DJ044938.1 | GM652717.1 |
| HI539071.1 | DL096296.1 | DD215770.1 | HD087590.1 | DD057881.1 | GM635466.1 | CS727332.1 | DJ044897.1 | GM652685.1 |
| HI538853.1 | DL096264.1 | DD215740.1 | HD079749.1 | DD057849.1 | GM630660.1 | CS727230.1 | DJ027927.1 | GM652653.1 |
| HI470726.1 | DL096232.1 | DD215708.1 | FW345229.1 | DD054773.1 | GM630628.1 | GM952640.1 | DJ030183.1 | GM652621.1 |
| HI470600.1 | DL096168.1 | DD215676.1 | FW345031.1 | DD054043.1 | GM630596.1 | GM748297.1 | DJ030061.1 | GM652589.1 |
| HI564705.1 | DL120188.1 | CS254893.1 | FW344999.1 | DD053309.1 | GM621661.1 | FB662046.1 | DJ030015.1 | GM645495.1 |
| HI568921.1 | DL120156.1 | DD212343.1 | FW344944.1 | DD052459.1 | GM621629.1 | GM005813.1 | DJ029938.1 | GM645463.1 |
| HI568853.1 | DL120124.1 | DD211940.1 | FW343650.1 | DD052235.1 | GM621597.1 | GM888350.1 | FW394255.1 | GM645431.1 |
| HI554935.1 | DL120092.1 | CS037216.1 | FW343586.1 | DD059305.1 | GM621565.1 | GM865990.1 | FW394213.1 | GM638538.1 |
| HI564199.1 | DL120060.1 | CS027203.1 | HC922990.1 | DD042400.1 | FB505725.1 | GM680784.1 | FW394088.1 | GM638506.1 |
| HI564093.1 | DL120028.1 | CS023746.1 | HC922470.1 | DD048086.1 | GM657113.1 | FB712564.1 | HC301022.1 | GM638474.1 |
| HI583199.1 | DL094346.1 | CS022579.1 | HC920511.1 | DD044285.1 | GM657081.1 | GM040591.1 | HC291450.1 | GM638442.1 |
| HI563618.1 | DL094314.1 | CS017171.1 | HD068865.1 | DD030077.1 | GM657049.1 | FB709042.1 | HC294134.1 | GM633743.1 |
| HI563583.1 | DL094282.1 | CS016843.1 | HD064847.1 | CS082479.1 | GM657017.1 | GM841829.1 | HC289556.1 | GM633711.1 |
| HI551334.1 | DL094250.1 | CS016588.1 | HD063939.1 | CS082415.1 | GM656985.1 | GM841725.1 | HC289352.1 | GM633679.1 |
| HI551130.1 | DL094218.1 | CS016478.1 | HD033947.1 | CS082351.1 | GM656953.1 | GM603718.1 | HC289320.1 | GM625844.1 |
| HI544142.1 | DL094186.1 | CS016120.1 | HD057708.1 | CS082280.1 | GM656922.1 | GM706719.1 | HC289120.1 | GM625812.1 |
| HI508405.1 | DL094154.1 | CQ986638.1 | HD057676.1 | CS080402.1 | GM656890.1 | GM706687.1 | HC296057.1 | GM625780.1 |
| HI508239.1 | DL090539.1 | CQ986572.1 | HD062036.1 | CS079041.1 | GM656826.1 | GM706655.1 | HC295907.1 | GM625748.1 |
| HI000042.1 | DL090507.1 | CQ986538.1 | U14637.1 | CS075497.1 | GM656794.1 | GM706390.1 | HC288963.1 | GM624724.1 |
| HI000004.1 | DL090471.1 | CQ983158.1 | M31426.1 | CS073844.1 | GM649769.1 | GM706356.1 | HC288874.1 | GM624660.1 |
| HI003643.1 | DL090439.1 | CQ983112.1 | J04707.1 | CS070564.1 | GM649737.1 | GM603509.1 | HC295590.1 | GM624628.1 |
| HI003592.1 | DL090407.1 | CQ983023.1 | M63852.1 | CS070059.1 | GM649705.1 | FB742338.1 | HC295512.1 | GM642994.1 |
| HI003557.1 | DL090375.1 | CQ982979.1 | AY569172.1 | CS065939.1 | GM649673.1 | FB720262.1 | HC295448.1 | GM642961.1 |
| HI001835.1 | DL086370.1 | CQ982910.1 | HC917652.1 | DL010450.1 | GM649641.1 | FB718510.1 | HC288605.1 | GM642929.1 |
| HI001775.1 | DL086338.1 | CQ982842.1 | FW341961.1 | DL010418.1 | GM649609.1 | FB717767.1 | HC292026.1 | GM630653.1 |
| HI001737.1 | DL086306.1 | CQ982691.1 | FV528429.1 | DL042673.1 | GM641013.1 | FB715218.1 | HC291858.1 | GM630621.1 |
| HI465697.1 | DL086274.1 | CQ982541.1 | FV534508.1 | DL038792.1 | GM640981.1 | FB706264.1 | HC294744.1 | GM630589.1 |
| HI506050.1 | DL086242.1 | CQ980553.1 | FV519225.1 | DL038760.1 | GM640949.1 | FB677435.1 | HC293798.1 | GM621654.1 |
| AX145568.1 | DL086210.1 | CQ973142.1 | FV534233.1 | DL038728.1 | GM640917.1 | GM061153.1 | HC293618.1 | GM621622.1 |

| | | | | | | | | |
|---|---|---|---|---|---|---|---|---|
| AX145536.1 | DL110453.1 | CQ972640.1 | FV534077.1 | DL038696.1 | GM640885.1 | DL459984.1 | HC290665.1 | GM621590.1 |
| AX145504.1 | DL110421.1 | CQ977473.1 | HC465620.1 | DL035040.1 | GM640853.1 | DL106970.1 | HC290141.1 | GM621558.1 |
| AX145472.1 | DL110389.1 | CQ977101.1 | HC461791.1 | DL034898.1 | GM628499.1 | DL106938.1 | HC208415.1 | GM657138.1 |
| AX145440.1 | DL110357.1 | CQ976301.1 | HC460861.1 | DL034833.1 | GM047417.1 | DL106906.1 | HC289882.1 | GM657106.1 |
| AX145408.1 | DL109448.1 | CQ972371.1 | AY273892.1 | DL030725.1 | GM662112.1 | DL102293.1 | HC289848.1 | GM657074.1 |
| AX145375.1 | DL109416.1 | CQ972339.1 | AF394661.1 | DL030693.1 | GM662080.1 | DL102261.1 | HC207913.1 | GM657042.1 |
| AX145343.1 | DL109352.1 | CQ970074.1 | HC456306.1 | DL030533.1 | GM662048.1 | DL102179.1 | HC201444.1 | GM657010.1 |
| AX145311.1 | DL109320.1 | CQ969105.1 | HC453700.1 | DL026724.1 | GM662016.1 | DL102221.1 | HC199336.1 | GM656946.1 |
| AX145279.1 | DL109288.1 | CQ967772.1 | HC453668.1 | DL026692.1 | GM661984.1 | DL102165.1 | HC197990.1 | GM656915.1 |
| AX145247.1 | DL104554.1 | CQ765888.1 | HC460363.1 | DL026660.1 | GM661952.1 | DL102133.1 | HC197696.1 | GM656851.1 |
| AX145215.1 | DL104522.1 | CQ760550.1 | HC452131.1 | DL026628.1 | GM661920.1 | DL116873.1 | DM464464.1 | GM656787.1 |
| AX145183.1 | DL093938.1 | CQ758829.1 | FU756124.1 | DL026596.1 | GM654830.1 | DL116841.1 | HC196129.1 | GM656755.1 |
| AX145151.1 | DL093906.1 | CQ756680.1 | FU772122.1 | DL026564.1 | GM654798.1 | DL116813.1 | HC195236.1 | GM649634.1 |
| AX145119.1 | DL093874.1 | CQ754045.1 | FU759985.1 | DL022720.1 | GM654766.1 | DL116781.1 | HC193973.1 | GM630486.1 |
| AX145055.1 | DL093842.1 | CQ753330.1 | FU757781.1 | DL022688.1 | GM647773.1 | DL116749.1 | HC087886.1 | GM621519.1 |
| AX144991.1 | DL093810.1 | CQ654946.1 | FU757624.1 | DL022656.1 | GM647741.1 | DL112060.1 | HC187072.1 | GM621487.1 |
| AX144959.1 | DL093778.1 | CQ654820.1 | FU757297.1 | DL022592.1 | GM647709.1 | DL112028.1 | HC089988.1 | FB505211.1 |
| AX144926.1 | DL090099.1 | CQ654694.1 | FU756571.1 | DL019577.1 | GM640810.1 | DL111996.1 | HC089556.1 | GM739503.1 |
| AX144894.1 | DL090035.1 | CQ654529.1 | HC438657.1 | DL019545.1 | GM640777.1 | DL111964.1 | HC089524.1 | GM867483.1 |
| AX144862.1 | DL090003.1 | CQ654449.1 | HC358398.1 | DL019513.1 | GM640745.1 | DL111932.1 | HC089492.1 | GM685439.1 |
| AX144830.1 | DL089971.1 | CQ654387.1 | HC358210.1 | DL019481.1 | GM640713.1 | DL111900.1 | HC089460.1 | GM003571.1 |
| AX144798.1 | DL123354.1 | HC490517.1 | HC358079.1 | DL019449.1 | GM640681.1 | DL106865.1 | HC089428.1 | FB983207.1 |
| AX144766.1 | DL123258.1 | HC490485.1 | HC324604.1 | DL014982.1 | GM640649.1 | DL106833.1 | HC089395.1 | FB754264.1 |
| AX144734.1 | DL123226.1 | HC490446.1 | HB461477.1 | DL014950.1 | GM628295.1 | DL106801.1 | HC089363.1 | GM865635.1 |
| AX144702.1 | DL123194.1 | HC490414.1 | HB455474.1 | DJ418066.1 | GM627971.1 | DL106769.1 | HC089330.1 | FB727107.1 |
| AX144670.1 | DL118908.1 | HC490382.1 | HB451968.1 | DJ417438.1 | GM627939.1 | DL106737.1 | HC089298.1 | FB726020.1 |
| AX144638.1 | DL118876.1 | HC490350.1 | HB447908.1 | DJ381039.1 | GM715474.1 | DL106705.1 | HC089266.1 | GM841604.1 |
| AX144599.1 | DL118812.1 | HC490318.1 | HB441564.1 | DJ381007.1 | GM661749.1 | DL091211.1 | HC086272.1 | GM879664.1 |
| AX144471.1 | DL109260.1 | HC490286.1 | HB441216.1 | DJ380975.1 | GM654755.1 | DL091179.1 | HC085670.1 | GM723332.1 |
| AX144279.1 | DL109228.1 | HC490253.1 | HB423259.1 | DJ380943.1 | GM654723.1 | DL087371.1 | AY774916.1 | GM840870.1 |
| AX143763.1 | DL109196.1 | HC490221.1 | DM115560.1 | DJ380911.1 | GM654691.1 | DL087339.1 | AY774802.1 | FB709979.1 |
| AX143443.1 | DL109164.1 | HC472307.1 | DM114853.1 | DJ380879.1 | GM654659.1 | DL087307.1 | AY774632.1 | GM041773.1 |
| AX143379.1 | DL109132.1 | HC472274.1 | HB385790.1 | DJ380847.1 | GM654627.1 | DL087275.1 | AY774571.1 | GM040847.1 |
| AX143251.1 | DL109100.1 | HC472242.1 | AX429292.1 | DJ380827.1 | GM654595.1 | DL087243.1 | AY774514.1 | GM840197.1 |
| AX143123.1 | DL104366.1 | HC472210.1 | AX427153.1 | DJ379269.1 | GM654562.1 | DL087211.1 | AY774464.1 | GM040285.1 |
| AX143059.1 | DL104334.1 | HC472178.1 | AX419667.1 | DJ389530.1 | GM647569.1 | DL102104.1 | AY774403.1 | CS728644.1 |
| AX142995.1 | DL099920.1 | HC471946.1 | AX418268.1 | DJ388697.1 | GM640574.1 | DL102072.1 | AY774347.1 | CS728548.1 |
| AX142931.1 | DL099888.1 | HC471914.1 | AX411734.1 | DJ388657.1 | GM627898.1 | DL102040.1 | HB477031.1 | CS727424.1 |
| AX142867.1 | DL099792.1 | HC471882.1 | AX406734.1 | CS724493.1 | GM627866.1 | DL102008.1 | HB485451.1 | AY657159.1 |
| AX142803.1 | DL099728.1 | HC471827.1 | AX399395.1 | CS724460.1 | GM627834.1 | DL101976.1 | HB475454.1 | AY657127.1 |
| AX142739.1 | DL093718.1 | HC471795.1 | AX397747.1 | CS724419.1 | GM627802.1 | DL101944.1 | HB474884.1 | AY657095.1 |
| AX142547.1 | DL093686.1 | HC668138.1 | AX384564.1 | DJ357815.1 | GM871607.1 | DL097530.1 | HB464815.1 | AY657063.1 |
| AX142481.1 | DL093654.1 | HC668002.1 | AX380802.1 | DJ357783.1 | GM870418.1 | DL097498.1 | HB463773.1 | AY657031.1 |
| AX142225.1 | DL093590.1 | HC504657.1 | AX375491.1 | DJ357719.1 | GM661688.1 | DL097466.1 | HB463631.1 | M10291.1 |
| AX142031.1 | DL093558.1 | HC494559.1 | AX367154.1 | DJ357685.1 | GM661656.1 | DL121293.1 | HB471270.1 | K02037.1 |
| AX141773.1 | DL089943.1 | HC494425.1 | AX364550.1 | DJ349693.1 | GM661624.1 | DL121261.1 | HB469602.1 | M74305.1 |
| AX141709.1 | DL089911.1 | HC504196.1 | AX364518.1 | DJ361245.1 | GM661592.1 | DL121229.1 | HB469102.1 | K00902.1 |

| | | | | | | | | |
|---|---|---|---|---|---|---|---|---|
| AX141645.1 | DL089879.1 | HC502880.1 | AX364486.1 | DJ361211.1 | GM661560.1 | DL101891.1 | HB468039.1 | HV549781.1 |
| AX141581.1 | DL089847.1 | HC500770.1 | AX364454.1 | DJ354225.1 | GM661528.1 | DL101827.1 | DM152700.1 | HV549074.1 |
| AX141517.1 | DL089783.1 | HC499870.1 | AX364422.1 | DJ354085.1 | GM654534.1 | DL101795.1 | DM152554.1 | HV544271.1 |
| AX141389.1 | DL085778.1 | FW303972.1 | AX364390.1 | DJ339858.1 | GM654470.1 | DL101763.1 | DM155840.1 | HV543982.1 |
| AX137849.1 | DL085746.1 | FW302604.1 | AX364358.1 | DJ339826.1 | GM035215.1 | DL101731.1 | DM155726.1 | HV543859.1 |
| HW237892.1 | DL085714.1 | FW301169.1 | AX364263.1 | DJ339762.1 | GM017392.1 | DL097317.1 | DM150253.1 | HV543554.1 |
| HW248687.1 | DL085682.1 | HC470495.1 | AX364198.1 | DJ339730.1 | GM833930.1 | DL097285.1 | DM149924.1 | HV538572.1 |
| HW237422.1 | DL085650.1 | HC488043.1 | AX364166.1 | DJ339698.1 | GM952643.1 | DL097253.1 | HB460460.1 | HV543337.1 |
| HW242315.1 | DL123139.1 | HC486597.1 | AX357129.1 | DJ339666.1 | CS803337.1 | DL097221.1 | HB455310.1 | HV543212.1 |
| HW237205.1 | DL123107.1 | HC467977.1 | BD008859.1 | DJ339601.1 | FB295834.1 | DL097189.1 | HB455088.1 | HV542742.1 |
| HW241643.1 | DL123043.1 | HC197992.1 | BD006216.1 | DJ339569.1 | FB294112.1 | DL097157.1 | HB445381.1 | FW379638.1 |
| HW163852.1 | DL123011.1 | HC197697.1 | E55313.1 | DJ339537.1 | DJ047003.1 | DL095297.1 | DM121074.1 | FW379606.1 |
| HW155837.1 | DL122979.1 | DM464465.1 | E54593.1 | DJ339291.1 | DJ052009.1 | DL095233.1 | DM139813.1 | FW378090.1 |
| HW155682.1 | AX358427.1 | HC196130.1 | BD000220.1 | DJ339078.1 | DJ045570.1 | DL095201.1 | DM134331.1 | FW377342.1 |
| HW155622.1 | AX357300.1 | HC195237.1 | E59808.1 | DJ336588.1 | DJ050217.1 | DL095169.1 | DM143742.1 | FW381577.1 |
| HW144781.1 | AX356673.1 | HC193974.1 | AX351252.1 | DJ335793.1 | DJ048840.1 | DL125336.1 | DM138464.1 | FW381497.1 |
| HW155228.1 | BD006812.1 | HC192584.1 | HW381366.1 | DJ333880.1 | DJ048808.1 | DL125304.1 | DM138432.1 | FW376113.1 |
| HW158937.1 | E63246.1 | HC190394.1 | HW381029.1 | DJ329164.1 | CS793960.1 | DL125272.1 | DM143373.1 | FW375416.1 |
| HW154986.1 | BD002004.1 | HC187074.1 | HW352701.1 | DJ328851.1 | CS330148.1 | DL125240.1 | DM137787.1 | FW375235.1 |
| HW154877.1 | E54571.1 | HC090110.1 | HW350996.1 | DJ328682.1 | CS319140.1 | DL125208.1 | DM137120.1 | FW380415.1 |
| HW158482.1 | E55386.1 | HC089557.1 | HW350964.1 | DD357925.1 | CS318711.1 | DL125176.1 | DM136668.1 | FW369464.1 |
| HW154729.1 | BD000394.1 | HC089525.1 | HW340569.1 | DD357627.1 | DD261009.1 | DL116596.1 | HB441233.1 | HH736084.1 |
| HW154641.1 | E58976.1 | HC089493.1 | HW350888.1 | DD357466.1 | CS305312.1 | DL116564.1 | HB427177.1 | HH735910.1 |
| HW081539.1 | AX356510.1 | HC089461.1 | HW350665.1 | CS449754.1 | CS305188.1 | DL116532.1 | HB426467.1 | HH735610.1 |
| HW085169.1 | AX354696.1 | HC089429.1 | HW350517.1 | A29482.1 | CS302517.1 | DL123257.1 | HB423121.1 | HH733939.1 |
| HW099459.1 | AX352747.1 | HC089396.1 | HW350167.1 | A05260.1 | CS298415.1 | DL123225.1 | HB423083.1 | HH733746.1 |
| HW084520.1 | AX351102.1 | HC089364.1 | HW339648.1 | A04060.1 | DD252137.1 | DL123193.1 | HB416462.1 | HH733266.1 |
| HW103412.1 | AX349126.1 | HC089331.1 | HW363786.1 | CS446132.1 | DD250833.1 | DL118907.1 | HB412961.1 | FW368807.1 |
| HW103136.1 | AX349043.1 | HC089299.1 | HW070901.1 | CS443888.1 | DD248871.1 | DL118875.1 | HB396998.1 | FW368176.1 |
| HW098200.1 | AX348476.1 | HC089267.1 | HW083280.1 | CS438988.1 | DD258244.1 | DL118811.1 | HB396568.1 | FW367210.1 |
| HW061884.1 | AX347453.1 | HC086564.1 | HW083248.1 | CS438574.1 | DD257317.1 | DL109259.1 | GN032016.1 | HC089578.1 |
| HW059134.1 | AX347363.1 | HC086279.1 | HW083216.1 | CS436512.1 | DD253716.1 | DL109227.1 | GM642705.1 | HC089546.1 |
| HW065331.1 | AX347291.1 | HC085672.1 | HW097575.1 | CS435192.1 | CS287681.1 | DL109195.1 | GM642673.1 | HC089514.1 |
| HW065234.1 | AX347253.1 | AY774960.1 | HW097491.1 | DD347354.1 | CS287563.1 | DL109163.1 | GM642641.1 | HC089482.1 |
| HW065201.1 | AX347183.1 | AY774917.1 | HW099479.1 | DD346818.1 | DD240758.1 | DL109131.1 | GM635331.1 | HC089450.1 |
| HW061333.1 | AX345347.1 | AY774804.1 | HW102823.1 | DD331840.1 | DD240709.1 | DL109099.1 | GM635299.1 | HC089417.1 |
| HW058491.1 | AX044423.1 | AY774744.1 | HW089264.1 | CS430549.1 | DD240661.1 | DL104365.1 | GM635267.1 | HC089385.1 |
| HW064835.1 | AX040462.1 | AY774693.1 | HW061872.1 | CS426716.1 | DD240599.1 | DL104333.1 | GM630461.1 | HC089353.1 |
| HW061120.1 | AX039585.1 | AY774633.1 | HW061759.1 | CS433057.1 | DD240491.1 | DL099791.1 | GM630429.1 | HC089320.1 |
| HW056692.1 | AX039326.1 | AY774516.1 | HW065319.1 | CS419507.1 | DD240459.1 | DL099759.1 | GM630397.1 | HC089288.1 |
| HW064731.1 | AX039154.1 | AY774466.1 | HW065221.1 | CS418672.1 | DD240098.1 | DL093749.1 | GM630365.1 | HC089256.1 |
| HW060702.1 | AX037559.1 | AY774057.1 | HW065189.1 | CS417155.1 | DD236636.1 | DL093717.1 | GM630333.1 | AY774728.1 |
| HW062402.1 | AX037087.1 | AY773994.1 | HW061321.1 | CS416483.1 | DD235243.1 | DL093685.1 | GM630301.1 | AY774671.1 |
| HW060452.1 | AX036033.1 | HC084629.1 | HW064895.1 | CS416218.1 | DD234708.1 | DL093653.1 | GM621526.1 | AY774617.1 |
| HW062281.1 | AX035456.1 | HC070334.1 | HW055559.1 | CS415770.1 | DD247164.1 | DL093589.1 | GM621494.1 | AY774547.1 |
| HW065986.1 | AX034870.1 | HC083877.1 | HW064687.1 | CS410902.1 | DD246845.1 | DL093557.1 | GM698711.1 | AY774382.1 |
| HW042068.1 | AX024792.1 | HC083735.1 | HW064643.1 | CS410686.1 | E41525.1 | DL089942.1 | GM656588.1 | AY774330.1 |

| | | | | | | | | |
|---|---|---|---|---|---|---|---|---|
| HW042036.1 | AX023656.1 | HC083526.1 | HW060680.1 | DD052070.1 | E44270.1 | DL089910.1 | GM656556.1 | AY774225.1 |
| HW043566.1 | AX023624.1 | HC081833.1 | HW062422.1 | DD057943.1 | E37989.1 | DL089846.1 | GM649467.1 | AY774165.1 |
| HW043433.1 | AX023342.1 | DM460345.1 | HW062390.1 | DD057911.1 | E41220.1 | DL085777.1 | GM649435.1 | AY774039.1 |
| HW041960.1 | AX020390.1 | HC062001.1 | HV119965.1 | DD057879.1 | E39199.1 | DL085745.1 | GM649403.1 | AY645667.1 |
| HW041928.1 | AX020247.1 | HC061922.1 | HV187365.1 | DD057847.1 | E06925.1 | DL085713.1 | GM670043.1 | M16249.1 |
| HV437891.1 | AX019632.1 | HC061373.1 | HV195748.1 | DD057158.1 | E06577.1 | DL085681.1 | GM656538.1 | HC083724.1 |
| HV443228.1 | AX019188.1 | HC057891.1 | HV226914.1 | DD056403.1 | E05672.1 | DL123138.1 | GM656506.1 | HC083515.1 |
| HV445403.1 | AX018589.1 | HC055950.1 | HV305470.1 | DD054040.1 | E05360.1 | DL123106.1 | GM656474.1 | U26411.1 |
| HV349426.1 | AX014753.1 | HC045721.1 | HV188408.1 | DD053307.1 | E04187.1 | DL123074.1 | GM656410.1 | DM383620.1 |
| HV348883.1 | AX002753.1 | HC045691.1 | HV182444.1 | DD052456.1 | E03660.1 | DL123042.1 | GM649305.1 | DM381920.1 |
| HV341638.1 | AX011482.1 | HC045659.1 | HV182412.1 | DD052233.1 | E03021.1 | DL123010.1 | GM649313.1 | DM381492.1 |
| HV341047.1 | AX011450.1 | HC045627.1 | HV182380.1 | DD052178.1 | E02902.1 | DL122978.1 | GM649257.1 | HC051932.1 |
| HV343508.1 | AX011418.1 | HC045595.1 | HV235482.1 | DD059303.1 | E02785.1 | DL118692.1 | GM649225.1 | DM120695.1 |
| HV347633.1 | AX011385.1 | HC045563.1 | FW573339.1 | DD048084.1 | E01774.1 | DL118660.1 | GM649193.1 | DM118005.1 |
| HV344836.1 | AX011353.1 | HC045531.1 | FW574596.1 | CS038823.1 | DD161080.1 | DL118628.1 | GM642584.1 | DM115567.1 |
| HV347089.1 | AX011251.1 | HC035626.1 | FW570999.1 | CS023847.1 | DD178465.1 | DL114232.1 | GM642552.1 | DM114860.1 |
| HV344752.1 | AX010957.1 | HC021024.1 | FW571805.1 | CS023734.1 | DD166303.1 | DL114200.1 | GM642455.1 | HB387463.1 |
| HV339926.1 | AX010811.1 | HC025874.1 | HI979318.1 | CS022575.1 | DD165510.1 | DL114168.1 | GM642423.1 | HB386549.1 |
| HV342453.1 | AX010627.1 | HC025509.1 | HI969612.1 | CS021536.1 | DD170791.1 | DL114136.1 | GM635241.1 | HB384550.1 |
| HV344446.1 | AX009537.1 | HC025477.1 | HI987452.1 | CS021416.1 | DD182184.1 | DL114104.1 | GM635209.1 | HA642847.1 |
| HV342091.1 | AX007101.1 | HC025445.1 | HI987251.1 | CS018549.1 | DD175951.1 | DL114072.1 | GM635177.1 | HA642259.1 |
| HV335541.1 | AX006467.1 | HC025413.1 | FW512270.1 | CS018485.1 | DD175918.1 | DL126212.1 | GM635145.1 | HA641598.1 |
| HI657185.1 | AX004612.1 | HC024926.1 | FW552191.1 | CS018340.1 | CS017165.1 | DL126180.1 | GM635113.1 | HA641133.1 |
| HI656048.1 | AX004142.1 | HC010449.1 | FW552152.1 | CS018266.1 | CS016585.1 | DL126148.1 | GM635081.1 | HA637707.1 |
| FW496026.1 | AX003076.1 | HC010186.1 | FW552116.1 | CS016833.1 | CS016472.1 | DL122932.1 | GM630275.1 | HA644354.1 |
| FW498985.1 | AX002972.1 | HC007811.1 | FW561804.1 | CQ990437.1 | CS016113.1 | DL122900.1 | GM630243.1 | DM102627.1 |
| FW503512.1 | AF116871.1 | HC007779.1 | FW555583.1 | CQ986634.1 | CQ990438.1 | DL122836.1 | GM630211.1 | DM094864.1 |
| FW420468.1 | A48496.1 | GM653395.1 | FW563138.1 | CQ986600.1 | CQ986635.1 | DL122804.1 | GM630179.1 | GN368312.1 |
| FW420404.1 | A35774.1 | GM646396.1 | FW561653.1 | CQ986568.1 | CQ986601.1 | DL122772.1 | GM737381.1 | GN360152.1 |
| FW501724.1 | A35713.1 | GM646364.1 | HD113804.1 | CQ986534.1 | CQ986569.1 | DL114026.1 | GM698657.1 | GM044200.1 |
| FW496918.1 | A31763.1 | GM646332.1 | HD079733.1 | CQ986502.1 | CQ986535.1 | DL113994.1 | GM698561.1 | GM044000.1 |
| FW420379.1 | A33989.1 | GM646300.1 | FW345029.1 | CQ983154.1 | CQ986503.1 | DL113962.1 | GM635046.1 | GM755061.1 |
| FW420347.1 | A32835.1 | GM646236.1 | FW344997.1 | CQ983108.1 | CQ983190.1 | DL113930.1 | GM635014.1 | DL467561.1 |
| FW420315.1 | A30341.1 | GM639433.1 | FW343712.1 | CQ983073.1 | CQ983109.1 | DL113898.1 | GM634982.1 | GM712945.1 |
| FW496672.1 | A23367.1 | GM639401.1 | HC923699.1 | CQ983016.1 | CQ982838.1 | DL113866.1 | GM634950.1 | GM652747.1 |
| FW503286.1 | A06235.1 | GM639369.1 | HC922980.1 | CQ982975.1 | CQ982769.1 | DL109065.1 | GM634918.1 | GM652715.1 |
| HI653930.1 | A30285.1 | GM639337.1 | HC920508.1 | CQ982836.1 | CQ982687.1 | DL109033.1 | GM634886.1 | GM652683.1 |
| HI653868.1 | A28233.1 | GM639305.1 | HD064845.1 | CQ982686.1 | CQ982608.1 | DL109001.1 | GM629952.1 | GM652651.1 |
| HI653800.1 | A20316.1 | GM639273.1 | HD063937.1 | CQ981127.1 | CQ975466.1 | DL108969.1 | GM642406.1 | GM652619.1 |
| HI653519.1 | A20583.1 | GM639241.1 | AX364632.1 | CQ975465.1 | CQ972556.1 | DL108905.1 | GM642236.1 | GM652587.1 |
| HI653405.1 | A19549.1 | GM626694.1 | AX364547.1 | CQ973138.1 | CQ976903.1 | DL104299.1 | GM642270.1 | GM645493.1 |
| HI653356.1 | A17064.1 | GM626662.1 | AX364515.1 | CQ972555.1 | CQ975683.1 | DL104267.1 | GM697221.1 | GM645461.1 |
| HI651902.1 | A14632.1 | GM626630.1 | AX364483.1 | CQ975973.1 | CQ972368.1 | DL104203.1 | GM656337.1 | GM645429.1 |
| HI651036.1 | A12040.1 | GM626598.1 | AX364451.1 | CQ975668.1 | CQ972336.1 | DL104171.1 | GM656305.1 | GM638536.1 |
| HI650450.1 | A31186.1 | GM626566.1 | AX364419.1 | BD081483.1 | CQ971810.1 | DL104139.1 | GM656273.1 | GN359500.1 |
| HI647271.1 | A30557.1 | GM626534.1 | AX364387.1 | BD080682.1 | CQ971136.1 | DL099725.1 | GM656209.1 | GN359464.1 |
| HI646877.1 | A28888.1 | GM754524.1 | AX364355.1 | BD080143.1 | CQ971071.1 | DL042149.1 | GM656177.1 | GN359432.1 |

| | | | | | | | | |
|---|---|---|---|---|---|---|---|---|
| HI646157.1 | A21397.1 | GM660699.1 | AX364260.1 | BD074950.1 | CQ970890.1 | DL042117.1 | GM649184.1 | GN359368.1 |
| HH802667.1 | A27810.1 | GM660667.1 | AX364195.1 | BD074801.1 | CQ970055.1 | DL042085.1 | GM649152.1 | GN365397.1 |
| HD062427.1 | HV778431.1 | GM660635.1 | AX364146.1 | BD063673.1 | CQ969080.1 | DL033706.1 | GM649120.1 | GN364284.1 |
| M31103.1 | HV773800.1 | GM660603.1 | AX361207.1 | BD062188.1 | CQ967766.1 | DL033674.1 | GM646758.1 | GN359231.1 |
| J04193.1 | HV745925.1 | GM660571.1 | AX358449.1 | BD057155.1 | CQ963553.1 | DL033642.1 | GM646726.1 | GN359167.1 |
| FW334596.1 | HV743386.1 | GM660539.1 | AX357080.1 | BD017739.1 | CQ957851.1 | DL033610.1 | GM646694.1 | GN359135.1 |
| FW342466.1 | HV702945.1 | GM653352.1 | BD004707.1 | BD016700.1 | CQ955576.1 | DL033578.1 | GM646662.1 | GN359103.1 |
| FW337200.1 | HW302702.1 | GM653320.1 | E64035.1 | BD015798.1 | CQ947127.1 | DL033546.1 | GM639827.1 | GN364131.1 |
| HC868187.1 | HW286630.1 | GM653288.1 | BD002016.1 | BD014232.1 | CQ944197.1 | DL029538.1 | GM639795.1 | GN359041.1 |
| HC887862.1 | HW286490.1 | GM653256.1 | E54588.1 | BD014159.1 | CQ944165.1 | DL029506.1 | GM639763.1 | GN363010.1 |
| HC887343.1 | HW295715.1 | GM653224.1 | BD001723.1 | AX482608.1 | CQ944133.1 | DL029474.1 | GM639731.1 | GN348291.1 |
| HC883725.1 | HW295585.1 | GM653192.1 | BD000460.1 | AX481599.1 | CQ944101.1 | DL029442.1 | GM639699.1 | GN346575.1 |
| HC880438.1 | HW295124.1 | GM646193.1 | AX354964.1 | AX477682.1 | CQ944069.1 | DL029410.1 | GM639667.1 | GN346543.1 |
| HC877785.1 | HW294976.1 | GM646161.1 | AX347303.1 | AX472342.1 | CQ944037.1 | DL029378.1 | GM627088.1 | GN340290.1 |
| HC869820.1 | HW307046.1 | GM646129.1 | AX347231.1 | AX468322.1 | DL049334.1 | DL029346.1 | GM627006.1 | GN337783.1 |
| HC869697.1 | HW294149.1 | GM646097.1 | AX347193.1 | AX463661.1 | DL049111.1 | DL046108.1 | GM646602.1 | GN335341.1 |
| HC869633.1 | HW294050.1 | GM646065.1 | AX347161.1 | A10497.1 | DL012571.1 | DL046076.1 | GM646558.1 | GN131882.1 |
| HC358219.1 | HW306932.1 | GM646033.1 | AX346745.1 | AX458652.1 | DL012539.1 | DL041962.1 | GM646526.1 | GN116522.1 |
| HC358089.1 | HW293167.1 | GM639230.1 | AX344905.1 | AX458536.1 | DL012507.1 | DL041930.1 | GM646494.1 | GN116490.1 |
| HC358056.1 | DL045243.1 | GM639198.1 | AX328398.1 | AX455878.1 | DL012475.1 | DL041898.1 | GM646462.1 | GN116457.1 |
| A01011.1 | DL045211.1 | GM639166.1 | AX328143.1 | AX454144.1 | DL012411.1 | DL029313.1 | GM646430.1 | GN116393.1 |
| HC324876.1 | DL045179.1 | GM639134.1 | AX306616.1 | AX452033.1 | DL045453.1 | DL029281.1 | GM639531.1 | GN116361.1 |
| HC324497.1 | DL045147.1 | GM639102.1 | AX268997.1 | A34763.1 | DL045421.1 | DL046009.1 | GM639447.1 | GN116329.1 |
| HC318715.1 | DL045115.1 | GM639070.1 | AX259248.1 | AX443298.1 | DL045389.1 | DL045977.1 | GM639491.1 | GN116297.1 |
| HC317601.1 | DL045083.1 | GM626523.1 | AX300701.1 | AX441275.1 | DL045357.1 | DL069477.1 | GM626888.1 | GN115061.1 |
| HC310092.1 | DL040971.1 | GM626491.1 | HW294126.1 | AX429441.1 | DL041085.1 | DL049715.1 | GM626856.1 | GM741831.1 |
| HC309996.1 | DL019813.1 | GM626459.1 | HW293264.1 | AX428583.1 | DL031018.1 | DL049683.1 | GM626824.1 | GM658313.1 |
| HC309964.1 | DL015378.1 | GM626427.1 | HW293044.1 | AX427116.1 | DL030986.1 | DL049651.1 | GM653639.1 | GM658281.1 |
| HC307848.1 | DL015346.1 | GM626395.1 | HW291308.1 | AX418597.1 | DL030957.1 | DL041860.1 | GM653607.1 | GM658217.1 |
| HC307793.1 | DL015314.1 | DL219494.1 | HW291183.1 | AX404869.1 | DL027145.1 | DL041828.1 | GM653575.1 | GM658103.1 |
| FU263021.1 | DL015282.1 | DL220940.1 | HW291151.1 | AX403276.1 | DL027113.1 | DL041796.1 | GM660868.1 | GM658071.1 |
| FU259871.1 | DL010792.1 | DL206307.1 | HW291054.1 | AX398674.1 | DL027081.1 | DL041764.1 | GM660772.1 | GM658039.1 |
| FU265434.1 | DL010760.1 | DL213603.1 | HW291022.1 | AX398296.1 | DL027049.1 | DL041732.1 | GM660740.1 | GM658007.1 |
| FU265370.1 | DL010728.1 | FB714505.1 | HW290958.1 | AX395284.1 | DL027017.1 | DL041700.1 | GM653521.1 | GM657975.1 |
| FU262487.1 | DL010696.1 | FB713801.1 | HW290895.1 | AX394270.1 | DL046859.1 | DL037781.1 | GM653489.1 | GM657943.1 |
| FU264504.1 | DL010664.1 | FB712993.1 | HW289974.1 | AX391844.1 | DL030912.1 | DL037749.1 | GM653457.1 | GM650751.1 |
| FU261552.1 | DL010632.1 | FB672296.1 | HW267813.1 | AX384624.1 | DL030873.1 | DL037685.1 | FB704809.1 | GM650719.1 |
| HC313119.1 | DL047056.1 | FB670948.1 | HW267760.1 | AX382508.1 | DL030841.1 | DL049610.1 | FB701522.1 | GM650687.1 |
| DM475554.1 | DL047024.1 | FB668510.1 | HW267406.1 | AX379338.1 | DL030809.1 | DL049482.1 | CS809045.1 | GM643695.1 |
| HB489179.1 | DL046960.1 | FB667422.1 | HW266135.1 | HW326311.1 | DL030777.1 | DL041659.1 | FB701080.1 | GM636538.1 |
| HB488753.1 | DL046928.1 | FB667111.1 | HW266052.1 | HW332572.1 | DL030745.1 | DL041627.1 | FB660050.1 | GM636506.1 |
| DM164072.1 | DL046896.1 | FB666800.1 | HW263044.1 | HW338862.1 | DL019781.1 | DL041595.1 | FB580555.1 | GM636474.1 |
| DM167766.1 | DL046864.1 | CS725981.1 | HW262837.1 | HW338350.1 | DL019725.1 | DL016415.1 | FB654877.1 | GM643493.1 |
| DM164004.1 | DL043042.1 | FB676575.1 | HW262743.1 | HW338222.1 | DL015194.1 | DL016383.1 | FB653509.1 | GM643461.1 |
| DM163971.1 | DL043010.1 | FB676511.1 | HW261391.1 | HW338094.1 | DL015162.1 | DL016351.1 | FB580222.1 | GM643428.1 |
| DM163903.1 | DL042978.1 | FB705975.1 | HW261359.1 | HW337838.1 | DL015130.1 | DL016319.1 | FB580187.1 | GM636439.1 |
| DM163302.1 | DL042946.1 | FB704842.1 | HW261295.1 | HW337710.1 | DL015066.1 | DL016287.1 | FB580123.1 | GM631839.1 |

| | | | | | | | | |
|---|---|---|---|---|---|---|---|---|
| DM163260.1 | DL042914.1 | FB701526.1 | HW261263.1 | HW337326.1 | DL015034.1 | DL016255.1 | FB580069.1 | GM631807.1 |
| DM162318.1 | DL042882.1 | FB701782.1 | HW261231.1 | HW337198.1 | DL010544.1 | DL016223.1 | DL200021.1 | GM631775.1 |
| DM162151.1 | DL038969.1 | FB701106.1 | HW261199.1 | HW337070.1 | DL010512.1 | DL028220.1 | DL199989.1 | GM622844.1 |
| HB477364.1 | DL038937.1 | FB660700.1 | HW261135.1 | HW336942.1 | DL010480.1 | DL028156.1 | DL199820.1 | GM741189.1 |
| HB486505.1 | DL038905.1 | FB580563.1 | HW261103.1 | HW321646.1 | DL010448.1 | DL011771.1 | DL090403.1 | GM657801.1 |
| HB486000.1 | DL035121.1 | FB580595.1 | HW261071.1 | HW329648.1 | DL010416.1 | DL011739.1 | DL090371.1 | GM009434.1 |
| HB474822.1 | DL031117.1 | FB654435.1 | HW261007.1 | HI949791.1 | DL038790.1 | DL011707.1 | DL086366.1 | GM008848.1 |
| HB469149.1 | DL031085.1 | FB654125.1 | HW260943.1 | HI918267.1 | DL038758.1 | DL011675.1 | DL086334.1 | FB660838.1 |
| HB468031.1 | DL031053.1 | FB582037.1 | HW260911.1 | HI917009.1 | DL038726.1 | DL011643.1 | DL086302.1 | GM959552.1 |
| DM078720.1 | DL031021.1 | FB580228.1 | HW260847.1 | HI657789.1 | DL038694.1 | DL011611.1 | DL086270.1 | FB983203.1 |
| DM077887.1 | DL030989.1 | FB580191.1 | HW260815.1 | HI657201.1 | DL035070.1 | DL048626.1 | DL086238.1 | FB983183.1 |
| BD453283.1 | DL030960.1 | FB580159.1 | HW260783.1 | FW498177.1 | DL034831.1 | DL048594.1 | DL086206.1 | GM866037.1 |
| AX536402.1 | DL027148.1 | FB580127.1 | HW260751.1 | FW503019.1 | DL030723.1 | DL048562.1 | DL110289.1 | FB728230.1 |
| AX534749.1 | DL027116.1 | FB580085.1 | HW260719.1 | FW496474.1 | DL030691.1 | DL048530.1 | DL105484.1 | GM751787.1 |
| AX528975.1 | DL027084.1 | DL200025.1 | HW260687.1 | FW503814.1 | DL030531.1 | DL048498.1 | DL105452.1 | FB726926.1 |
| AX528943.1 | DL027052.1 | DL189541.1 | HW260655.1 | FW499102.1 | DL026722.1 | DL048466.1 | DL105420.1 | GM864063.1 |
| AX528516.1 | DL027020.1 | DL199824.1 | HW260623.1 | FW504762.1 | DL026690.1 | DL044849.1 | DL105388.1 | FB725720.1 |
| AX528098.1 | DL026988.1 | DL199792.1 | HW260559.1 | FW500405.1 | DL026658.1 | DL044817.1 | DL105356.1 | GM968168.1 |
| AX523911.1 | DL026956.1 | DL143739.1 | HW260527.1 | FW498926.1 | DL026626.1 | DL044785.1 | DL105324.1 | GM879660.1 |
| A33288.1 | DL030915.1 | DL107276.1 | HV802926.1 | FW420441.1 | DL026594.1 | DL044721.1 | DL124343.1 | GM723328.1 |
| AX179478.1 | DL030876.1 | DL107244.1 | HV802894.1 | FW420409.1 | DL026562.1 | DL044689.1 | DL124311.1 | GM840858.1 |
| AX179409.1 | DL030844.1 | DL107212.1 | HV778376.1 | FW501790.1 | DL019575.1 | DL044633.1 | DL110172.1 | FB712041.1 |
| AX028019.1 | DL030812.1 | DL107148.1 | HV763967.1 | FW496932.1 | DL019543.1 | DL044601.1 | DL110140.1 | GM723191.1 |
| AX521534.1 | DL030780.1 | DL107116.1 | HV767473.1 | FW420384.1 | DL019511.1 | DL044569.1 | DL110108.1 | GM840556.1 |
| AX521502.1 | DL030748.1 | DL102503.1 | HV766824.1 | FW420352.1 | CS611848.1 | DL044537.1 | DL110076.1 | GM040842.1 |
| AF430188.1 | DL019784.1 | DL102471.1 | HV550136.1 | FW420320.1 | CS604546.1 | DL044505.1 | DL105175.1 | GM040270.1 |
| AF430156.1 | DL015165.1 | DL102439.1 | HV552739.1 | FW496734.1 | CS604514.1 | DL044473.1 | DL105143.1 | FB709062.1 |
| AX505137.1 | DL015133.1 | DL102407.1 | HV550383.1 | HI653875.1 | CS604482.1 | DL040457.1 | DL105111.1 | CS728576.1 |
| BD140614.1 | DL015101.1 | DL102375.1 | AY659387.1 | HI653805.1 | CS604450.1 | DL040425.1 | DL100601.1 | CS727420.1 |
| BD140003.1 | DL015069.1 | DL102343.1 | AY659355.1 | HI653633.1 | CS604418.1 | DL040393.1 | DL100569.1 | GM036646.1 |
| BD138397.1 | DL015037.1 | DL097634.1 | AY659323.1 | HC889117.1 | CS604354.1 | DL040361.1 | DL100537.1 | GM836383.1 |
| BD138022.1 | DL010547.1 | DL097602.1 | AY659291.1 | HC869869.1 | CS604322.1 | DL040329.1 | DL119991.1 | GM949539.1 |
| BD136697.1 | DL010515.1 | DL097570.1 | AY659259.1 | HC876941.1 | CS604226.1 | DL036673.1 | DL119959.1 | GM867661.1 |
| BD136054.1 | DL010483.1 | CQ771662.1 | AY659227.1 | HC868464.1 | CS604194.1 | DL036641.1 | DL119927.1 | FB717485.1 |
| BD135541.1 | DL010451.1 | CQ771630.1 | AY659195.1 | FW310249.1 | CS603906.1 | DL036609.1 | DL119895.1 | GM879375.1 |
| BD133901.1 | DL010419.1 | CQ771598.1 | AY659163.1 | FW309612.1 | CS603810.1 | DL036577.1 | DL119863.1 | GM879314.1 |
| BD131937.1 | DL046848.1 | CQ770993.1 | AY659131.1 | FW308791.1 | CS603458.1 | DL036545.1 | DL119831.1 | GM732417.1 |
| BD131904.1 | DL046816.1 | CQ768840.1 | AY659099.1 | FW334255.1 | CS603426.1 | DL032310.1 | DL115236.1 | GM840925.1 |
| BD130899.1 | DL046784.1 | CQ768384.1 | AY659067.1 | FW333962.1 | CS603394.1 | DL032278.1 | DL115204.1 | DL115510.1 |
| BD130829.1 | DJ056892.1 | CQ767961.1 | AY659035.1 | FW306896.1 | CS603362.1 | DL032246.1 | DL115172.1 | DL115478.1 |
| BD130797.1 | DJ053502.1 | CQ767214.1 | AY659003.1 | FW306435.1 | CS603330.1 | DL032214.1 | DL115140.1 | DL115446.1 |
| BD130765.1 | DJ053216.1 | CQ766070.1 | AY658971.1 | FW332186.1 | CS603298.1 | DL032182.1 | DL115108.1 | DL105694.1 |
| BD130604.1 | DJ053084.1 | CQ761147.1 | AY658939.1 | HC769918.1 | CS603266.1 | DL032150.1 | DL115076.1 | DL105662.1 |
| BD129649.1 | CS810640.1 | CQ759574.1 | AY658907.1 | HC769796.1 | CS603234.1 | DL048420.1 | DL115044.1 | DL105630.1 |
| BD107648.1 | CS810535.1 | CQ758827.1 | AY658875.1 | HC767814.1 | CS603202.1 | DL048388.1 | DL110044.1 | DL105598.1 |
| BD103202.1 | DJ020649.1 | CQ754044.1 | GN032411.1 | HC767342.1 | CS603170.1 | DL048356.1 | DL110012.1 | DL096283.1 |
| BD082686.1 | DJ017821.1 | CQ654945.1 | GN032379.1 | HC757151.1 | CS603138.1 | DL048324.1 | DL109980.1 | DL096251.1 |

| | | | | | | | | |
|---|---|---|---|---|---|---|---|---|
| BD081297.1 | DJ015681.1 | CQ654818.1 | GN032346.1 | HC742904.1 | CS603106.1 | DL044438.1 | DL109766.1 | DL120175.1 |
| BD080326.1 | CS803377.1 | CQ654291.1 | GN032314.1 | HC755779.1 | CS603074.1 | DL044406.1 | DL109734.1 | DL120143.1 |
| BD080136.1 | CS803338.1 | CQ654202.1 | GN032282.1 | HC754729.1 | CS603042.1 | DL044374.1 | DL109702.1 | DL120111.1 |
| BD078529.1 | FB295837.1 | CQ654106.1 | GN032250.1 | HC733792.1 | CS603010.1 | DL044342.1 | DL104968.1 | DL120079.1 |
| BD077087.1 | FB294128.1 | CQ654014.1 | GN032218.1 | HC733769.1 | CS602976.1 | DL044310.1 | DL104936.1 | DL120047.1 |
| BD074978.1 | DJ047308.1 | AX963147.1 | GN032186.1 | HC733692.1 | CS602944.1 | DL044278.1 | DL100394.1 | DL120015.1 |
| BD074942.1 | DJ047005.1 | AX962040.1 | GN032154.1 | HC732146.1 | CS602912.1 | DL040102.1 | DL100362.1 | DL094237.1 |
| BD074155.1 | DJ052320.1 | AX960374.1 | GN032122.1 | HC471773.1 | CS602880.1 | DL032109.1 | DL100330.1 | DL094205.1 |
| BD070060.1 | DJ052011.1 | AX960342.1 | GN032090.1 | HC471741.1 | CS602848.1 | DL032077.1 | DL096083.1 | DL094173.1 |
| BD063665.1 | DJ045575.1 | BD010841.1 | GN032058.1 | HC471701.1 | CS602816.1 | DL032045.1 | DL124143.1 | DL090494.1 |
| BD016725.1 | DJ050218.1 | AX957040.1 | GN032026.1 | HC471645.1 | CS602752.1 | DL032013.1 | DL124111.1 | DL086389.1 |
| BD014521.1 | DJ048841.1 | AX938904.1 | GN031994.1 | HC200250.1 | CS602720.1 | DL031981.1 | DL119793.1 | DL086293.1 |
| BD014224.1 | CS793984.1 | AX601361.1 | GN031962.1 | HC199333.1 | CS602688.1 | DL031949.1 | DL119761.1 | DL086261.1 |
| BD014189.1 | CS800118.1 | AX601329.1 | GN031930.1 | DM463982.1 | CS602560.1 | DL028140.1 | DL119729.1 | DL086229.1 |
| AX491315.1 | CS720124.1 | AX599035.1 | GN031898.1 | HC196126.1 | CS602528.1 | DL028108.1 | DL119697.1 | DL086197.1 |
| HW340696.1 | CS799993.1 | AX598985.1 | GN031834.1 | HC194239.1 | CS610258.1 | DL028076.1 | DL119665.1 | DL080647.1 |
| HW350674.1 | CS806393.1 | AX598933.1 | GN031801.1 | HC193954.1 | CS602114.1 | DL028044.1 | DL037198.1 | DL075890.1 |
| HW340166.1 | CS796354.1 | AX598775.1 | GN031672.1 | HC193615.1 | CS608451.1 | DL028012.1 | DL037166.1 | DL078924.1 |
| HW339915.1 | CS715299.1 | AX593379.1 | GN031640.1 | HC190863.1 | CS608375.1 | DL027980.1 | DL037134.1 | DL073053.1 |
| HW349950.1 | CS806055.1 | AX587863.1 | GN031608.1 | HC188502.1 | CS607919.1 | DL036394.1 | DL028731.1 | DL081569.1 |
| HW348594.1 | CS805340.1 | AX587764.1 | GN031576.1 | HC187069.1 | AY773985.1 | DL036362.1 | DL028635.1 | DL013502.1 |
| HW347957.1 | CS790965.1 | AX587673.1 | GN031544.1 | HC089585.1 | M30841.1 | DL036330.1 | DL028603.1 | DL013470.1 |
| HW363496.1 | CS792483.1 | AX587568.1 | GN031480.1 | HC089553.1 | HC070454.1 | DL013977.1 | DL028571.1 | DL013438.1 |
| HW355220.1 | DJ042531.1 | AX591635.1 | GN031415.1 | HC089521.1 | HC070104.1 | DL013945.1 | DL024757.1 | DL013406.1 |
| HW326540.1 | DJ044957.1 | AX591042.1 | GN031383.1 | HC089489.1 | HC083554.1 | DL013913.1 | DL024725.1 | DL017624.1 |
| HW339034.1 | HI207610.1 | BD162147.1 | GN031286.1 | HC089457.1 | HC083521.1 | DL013845.1 | DL024693.1 | DL022294.1 |
| HW338450.1 | HI003702.1 | BD161159.1 | GN031254.1 | HC089425.1 | HC069868.1 | DL030109.1 | DL041077.1 | DL022262.1 |
| HW338322.1 | HI002077.1 | BD160914.1 | GN031222.1 | HC089392.1 | HC081535.1 | DL030077.1 | DL048690.1 | DL025558.1 |
| HW338194.1 | HI002026.1 | AX587960.1 | GN031190.1 | HC089360.1 | HC057881.1 | DL030045.1 | DL048658.1 | DL034020.1 |
| HW337810.1 | HI001943.1 | AX587765.1 | GN031157.1 | HC089327.1 | DM459965.1 | DL030013.1 | DL045240.1 | DL025217.1 |
| HW337298.1 | HI000275.1 | AX587610.1 | GN031125.1 | HC089295.1 | DM459790.1 | DL029981.1 | DL045208.1 | DL023331.1 |
| HW337042.1 | HI000235.1 | AX575317.1 | GN031093.1 | HC089263.1 | DM381541.1 | DL029949.1 | DL045176.1 | DL023299.1 |
| HW336758.1 | HI000191.1 | AX556828.1 | GN031061.1 | HC086261.1 | HC053881.1 | DL046638.1 | DL045144.1 | DL023267.1 |
| HW336550.1 | HI000135.1 | AX555197.1 | GN031029.1 | HC085663.1 | HC053722.1 | DL046606.1 | DL045112.1 | DL023235.1 |
| HW329549.1 | HI205052.1 | AX551076.1 | GN030997.1 | AY775010.1 | HC051938.1 | DL046574.1 | DL045080.1 | DL023203.1 |
| HW321090.1 | HI204463.1 | AX544473.1 | GN030965.1 | AY774953.1 | HC051643.1 | DL046542.1 | DL037081.1 | DL022976.1 |
| HW058488.1 | HI180956.1 | AX538675.1 | GN030934.1 | AY774913.1 | HC050949.1 | DL046510.1 | DL037049.1 | DL010748.1 |
| HW064904.1 | HI178980.1 | AX537251.1 | GN030902.1 | AY774857.1 | HC050284.1 | DL046478.1 | DL037017.1 | DL010716.1 |
| HW064832.1 | HI539009.1 | AX529532.1 | CS492945.1 | AY774740.1 | HC049745.1 | DL042656.1 | DL028358.1 | DL010684.1 |
| HW061117.1 | HI470670.1 | AX528953.1 | CS485317.1 | AY774510.1 | HC045502.1 | DL042624.1 | DL024544.1 | DL042966.1 |
| HW057854.1 | HI470570.1 | AX528115.1 | DD401450.1 | AY774342.1 | HC045470.1 | DL042592.1 | DL024512.1 | DL042934.1 |
| HW056574.1 | HI544678.1 | AX526827.1 | DD401418.1 | AY774182.1 | HC045438.1 | DL038668.1 | DL024480.1 | DL042902.1 |
| HW056524.1 | HI564710.1 | AX524911.1 | DD401224.1 | AY774121.1 | HC045406.1 | DL038636.1 | FB761230.1 | DL038989.1 |
| HW056475.1 | HI568929.1 | AX523921.1 | DD404555.1 | K00562.1 | HC045374.1 | DL034501.1 | GM720838.1 | DL038925.1 |
| HW064728.1 | HI568857.1 | AX523639.1 | DD406595.1 | AF284215.1 | HC045310.1 | DL029910.1 | GM017328.1 | DL030903.1 |
| HW064654.1 | HI559128.1 | AX521938.1 | DD401545.1 | HC084356.1 | HC045278.1 | DL029878.1 | GM952619.1 | DL030736.1 |
| HW060699.1 | HI564207.1 | A33415.1 | DD401513.1 | HC070330.1 | GN366255.1 | DL029846.1 | GM952050.1 | DJ069484.1 |

| | | | | | | | | |
|---|---|---|---|---|---|---|---|---|
| HW062399.1 | HI564097.1 | AX179438.1 | DD401481.1 | HC083872.1 | GN359848.1 | DL029814.1 | GM949484.1 | DJ066770.1 |
| HW060513.1 | HI563622.1 | AX027997.1 | CS479757.1 | HC083731.1 | GN359816.1 | DL029782.1 | GM890239.1 | DJ066410.1 |
| HW060448.1 | HI563589.1 | AX027965.1 | DD347862.1 | HC083556.1 | GN359668.1 | DL025876.1 | GM963576.1 | DJ066374.1 |
| HW056305.1 | HI558076.1 | A09548.1 | CS419068.1 | HC083522.1 | GN359636.1 | DL018351.1 | GM889439.1 | DJ066342.1 |
| HW064441.1 | HI508557.1 | AX521544.1 | CS410876.1 | HC069869.1 | GN359408.1 | DL018319.1 | GM889320.1 | DJ066309.1 |
| HW062374.1 | HI575655.1 | AX521512.1 | CS414865.1 | HC057883.1 | GN359312.1 | DL018287.1 | GM008861.1 | DJ066275.1 |
| HW062246.1 | HI551354.1 | AX513490.1 | CS414815.1 | GM991853.1 | GN365275.1 | DL018255.1 | GM007136.1 | DJ066241.1 |
| HW065909.1 | HI544146.1 | AF430198.1 | DD327636.1 | GN000596.1 | GN359271.1 | DL018223.1 | GM867487.1 | DJ066040.1 |
| HW042065.1 | HI508413.1 | AX443301.1 | DD327007.1 | GM969395.1 | GN359239.1 | DL013821.1 | GM685455.1 | DJ061628.1 |
| HW042033.1 | HI508247.1 | AX429492.1 | DD326761.1 | GM969723.1 | GN359207.1 | DL013789.1 | GM003575.1 | DJ061580.1 |
| HW043563.1 | HI000051.1 | AX429238.1 | CS389326.1 | GM980880.1 | GN359143.1 | DL013757.1 | FB754405.1 | DJ061484.1 |
| HW041957.1 | HI000009.1 | AX428225.1 | CS389293.1 | GM984676.1 | GN359111.1 | DL013725.1 | DL087479.1 | DJ061428.1 |
| HW041925.1 | HI003597.1 | AX419090.1 | CS389251.1 | DL117635.1 | GN364168.1 | DL013693.1 | DL087447.1 | DJ055277.1 |
| HW041827.1 | HI003562.1 | AX418390.1 | CS389204.1 | DL122094.1 | GN363909.1 | DL013661.1 | DL087415.1 | DJ055193.1 |
| HW049411.1 | HI003512.1 | AX417945.1 | CS389167.1 | DL125739.1 | GN363565.1 | DL000608.1 | DL112257.1 | DJ060188.1 |
| HW049379.1 | HI001839.1 | AX404881.1 | CS406466.1 | DL125707.1 | GN363030.1 | DJ493817.1 | DL112225.1 | DJ053394.1 |
| HW049348.1 | HI001779.1 | AX403347.1 | CS403227.1 | DL125675.1 | GN362479.1 | DL008722.1 | DL112193.1 | DJ056339.1 |
| GN033222.1 | HI001741.1 | AX398677.1 | CS383382.1 | DL125611.1 | GN346510.1 | DL008507.1 | DL112161.1 | DJ053104.1 |
| GN033190.1 | HI465733.1 | AX394736.1 | DD291030.1 | DL125579.1 | GN346476.1 | DJ491889.1 | DL112129.1 | DJ053072.1 |
| GN033158.1 | HI465556.1 | AX391943.1 | DD289507.1 | DL125547.1 | GN344914.1 | DJ491594.1 | DL112097.1 | CS810628.1 |
| GN033126.1 | HI574583.1 | AX391495.1 | BD291402.1 | DL121891.1 | GN343938.1 | DL002527.1 | DL112065.1 | CS812994.1 |
| GN033062.1 | HI001680.1 | AX384694.1 | BD295269.1 | DL121859.1 | GN342226.1 | DJ446861.1 | DL107062.1 | DJ021067.1 |
| GN033030.1 | HI001621.1 | AX384384.1 | BD289851.1 | DL121827.1 | GN346583.1 | DJ446828.1 | DL107030.1 | CS803359.1 |
| GN032998.1 | HI001579.1 | AX382511.1 | BD277318.1 | DL121795.1 | GN346551.1 | DJ437324.1 | DL106998.1 | CS803326.1 |
| GN032966.1 | HI001529.1 | AX379341.1 | BD274172.1 | DL117532.1 | GN340166.1 | DJ437276.1 | DL106966.1 | DJ052628.1 |
| GM643494.1 | HI003363.1 | AX364692.1 | BD273545.1 | DL117500.1 | GN340035.1 | DJ437242.1 | DL102217.1 | DJ046979.1 |
| GM643429.1 | HI003326.1 | AX364654.1 | BD272487.1 | DL117468.1 | GN339050.1 | DJ437178.1 | DL102161.1 | DJ050559.1 |
| GM636440.1 | HC486588.1 | AX364608.1 | BD271919.1 | DL117436.1 | GN116530.1 | DJ437111.1 | DL102129.1 | DJ049440.1 |
| GM636312.1 | HC486518.1 | AX364537.1 | BD270243.1 | DL112757.1 | GN116498.1 | DJ437072.1 | DL116809.1 | DJ048829.1 |
| GM631840.1 | HC486452.1 | AX364505.1 | DD227538.1 | DL112725.1 | GN116465.1 | DJ436849.1 | DL116777.1 | CS793889.1 |
| GM631808.1 | HC486337.1 | AX364473.1 | DD227398.1 | DL112693.1 | GN116433.1 | DJ436760.1 | DL111928.1 | CS793496.1 |
| GM631776.1 | HC475386.1 | AX364441.1 | DD227169.1 | DL112661.1 | GN116401.1 | CS632180.1 | DL106861.1 | CS701576.1 |
| GM622845.1 | HC474965.1 | AX364409.1 | DD226211.1 | DL107760.1 | GN116369.1 | CS626884.1 | DL106829.1 | CS800050.1 |
| GM741191.1 | HC466641.1 | AX364377.1 | DD224936.1 | DL107728.1 | GN116337.1 | CS623717.1 | DL106797.1 | CS798834.1 |
| GM657802.1 | HC466609.1 | AX364345.1 | DD224376.1 | DL107696.1 | GN116305.1 | DD402406.1 | DL106733.1 | CS716761.1 |
| GM657769.1 | HC474331.1 | AX364218.1 | DD223918.1 | DL098319.1 | GN095155.1 | DD402374.1 | DL106701.1 | DD308884.1 |
| GM631661.1 | HC466285.1 | AX364185.1 | DD231422.1 | DL098287.1 | GN114132.1 | DD402342.1 | DL091271.1 | DD307931.1 |
| GM631629.1 | HC491796.1 | AX359931.1 | DD214000.1 | DL098255.1 | GN094512.1 | DD402310.1 | DL091239.1 | DD313220.1 |
| GM631597.1 | HC491764.1 | AX358650.1 | DD213983.1 | DL098223.1 | GN094480.1 | DD402278.1 | DL091207.1 | DD291171.1 |
| GM704215.1 | HC491732.1 | AX241596.1 | DD220609.1 | DL098191.1 | DM074531.1 | DD402246.1 | DL087207.1 | DD319810.1 |
| GM704162.1 | HC481975.1 | AX241564.1 | CQ787254.1 | DL092053.1 | DM078967.1 | DD402214.1 | DL102100.1 | CS365226.1 |
| GM657652.1 | HC481596.1 | AX241532.1 | CQ786565.1 | DL091989.1 | DM077891.1 | DD402182.1 | DL102068.1 | CS376414.1 |
| GM648642.1 | HC491457.1 | AX241500.1 | CQ784687.1 | FB342711.1 | GM704172.1 | DD402118.1 | DL102036.1 | CS376121.1 |
| GM648610.1 | HC472292.1 | AX241468.1 | CQ784621.1 | FB299249.1 | GM622449.1 | DD402086.1 | DL102004.1 | CS362660.1 |
| GM634458.1 | HC471190.1 | AX241436.1 | CQ778516.1 | DL047577.1 | GM622417.1 | DD402054.1 | DL101972.1 | CS360558.1 |
| GM634394.1 | FV533426.1 | AX241129.1 | CQ778394.1 | DL047545.1 | GM622385.1 | DD405774.1 | DL097526.1 | CS359734.1 |
| GM629471.1 | FV531719.1 | AX241097.1 | CQ774722.1 | DL039682.1 | GM657539.1 | DD405742.1 | DL097494.1 | CS359595.1 |

| | | | | | | | | |
|---|---|---|---|---|---|---|---|---|
| GM629429.1 | FV531687.1 | AX241065.1 | CQ756687.1 | DL039650.1 | GM657507.1 | DD405710.1 | DL097462.1 | BD269135.1 |
| GM629397.1 | FV531373.1 | AX241033.1 | CQ755355.1 | DL039618.1 | GM657475.1 | DD405678.1 | DL101887.1 | BD268156.1 |
| GM629365.1 | FV522825.1 | AX241001.1 | CQ754273.1 | DL039586.1 | GM657443.1 | DD405646.1 | DL101823.1 | BD268070.1 |
| GM629333.1 | FV530200.1 | AX240969.1 | AX962890.1 | DL039554.1 | GM657411.1 | DD405594.1 | DL101791.1 | BD267539.1 |
| GM629301.1 | FV529001.1 | AX240937.1 | HC321184.1 | DL039522.1 | GM657379.1 | DD405530.1 | DL101759.1 | BD265605.1 |
| GM648532.1 | FV528637.1 | AX239703.1 | HC356758.1 | DL039490.1 | GM657347.1 | DD405498.1 | DL101727.1 | BD264364.1 |
| GM648500.1 | FV534474.1 | AX235237.1 | HC325498.1 | DL043462.1 | GM650155.1 | BD453622.1 | DL097217.1 | BD263715.1 |
| GM629192.1 | FV534055.1 | AX225237.1 | HC325084.1 | DL043430.1 | GM650123.1 | BD453585.1 | DL097185.1 | BD263546.1 |
| GM629160.1 | FV533991.1 | AX214224.1 | HC325024.1 | DL043398.1 | GM650091.1 | BD493257.1 | DL097153.1 | BD263445.1 |
| GM629128.1 | HC460436.1 | AX208071.1 | HC307877.1 | DL043366.1 | GM643122.1 | BD453572.1 | DL095229.1 | CQ831241.1 |
| GM662971.1 | HC456290.1 | AX207295.1 | HC307808.1 | DL043334.1 | GM643090.1 | BD453540.1 | DL095197.1 | CQ829256.1 |
| GM648354.1 | HC453652.1 | AX205130.1 | HC312269.1 | DL043270.1 | GM643058.1 | BD453508.1 | DL095165.1 | CQ829218.1 |
| GM648322.1 | AF254671.1 | AX203116.1 | HC089467.1 | DL039389.1 | GM643026.1 | BD453476.1 | DL125268.1 | CQ828063.1 |
| GM648290.1 | HC452165.1 | AX191374.1 | HC089435.1 | DL039357.1 | GM636005.1 | BD453444.1 | DL125236.1 | CQ817000.1 |
| GM648258.1 | HC451891.1 | AX180281.1 | HC089402.1 | DL039325.1 | GM635973.1 | BD453412.1 | DL085801.1 | CQ816957.1 |
| GM648226.1 | DM459244.1 | AX174664.1 | HC089370.1 | DL035637.1 | GM657343.1 | BD443121.1 | DL123349.1 | CQ816923.1 |
| GM641423.1 | DM385650.1 | AX175348.1 | HC089338.1 | DL035605.1 | GM622217.1 | BD453235.1 | DL123317.1 | CQ814050.1 |
| GM641391.1 | DM383633.1 | AX172943.1 | HC089305.1 | DL035573.1 | GM622185.1 | BD453203.1 | DL123285.1 | CQ814018.1 |
| GM641327.1 | DM382853.1 | AX172493.1 | HC089273.1 | DL035541.1 | GM622153.1 | BD442910.1 | DL123253.1 | CQ813986.1 |
| GM641295.1 | DM381854.1 | AX172288.1 | HC085250.1 | DL035509.1 | GM631012.1 | BD442212.1 | DL123221.1 | CQ813954.1 |
| GM641263.1 | HC054876.1 | AX167461.1 | AY774971.1 | DL031505.1 | GM630980.1 | BD410209.1 | DL123189.1 | CQ813922.1 |
| GM629040.1 | HC051941.1 | AX167414.1 | AY774923.1 | DL031473.1 | GM630948.1 | BD399073.1 | DL118903.1 | CQ813890.1 |
| GM629008.1 | HC051038.1 | AX155172.1 | AY774874.1 | DL031441.1 | GM630916.1 | BD409475.1 | DL118871.1 | CQ813858.1 |
| GM881522.1 | HC050301.1 | AX151119.1 | AY774758.1 | DL031409.1 | GM630891.1 | BD398709.1 | DL118839.1 | CQ813826.1 |
| GM648193.1 | HC045505.1 | AX149436.1 | AY774699.1 | DL031377.1 | GM635848.1 | BD398633.1 | DL109255.1 | CQ813721.1 |
| GM648161.1 | HC045473.1 | AX146768.1 | AY774644.1 | DL031345.1 | GM635816.1 | BD440944.1 | DL109223.1 | CQ801440.1 |
| DL128430.1 | HC045441.1 | AX145734.1 | AY774586.1 | DL027472.1 | DL086480.1 | BD450800.1 | DL109191.1 | CQ800906.1 |
| DL124672.1 | HC045377.1 | AX145702.1 | AY774478.1 | DL027440.1 | DL086448.1 | BD408110.1 | DL109159.1 | CQ800661.1 |
| DL124640.1 | HC045313.1 | AX145670.1 | AY774415.1 | DL027408.1 | DL086416.1 | BD408078.1 | DL109127.1 | CQ795475.1 |
| DL124608.1 | HC045281.1 | AX145638.1 | AY774306.1 | DL027376.1 | DL121890.1 | BD429796.1 | DL109095.1 | CQ795443.1 |
| DL124576.1 | HC049373.1 | AX145606.1 | AY774198.1 | DL023564.1 | DL121858.1 | BD429575.1 | DL104457.1 | CQ794278.1 |
| DL096911.1 | FW553121.1 | AX145574.1 | AY774068.1 | DL023532.1 | DL121826.1 | BD407506.1 | DL104361.1 | CQ792512.1 |
| DL096847.1 | FW556703.1 | AX145542.1 | AY167892.1 | DL023500.1 | DL121794.1 | BD418442.1 | DL104329.1 | CQ787689.1 |
| DL120311.1 | FW566267.1 | AX145510.1 | HC083741.1 | DL023468.1 | DL117531.1 | BD429034.1 | DL093585.1 | CQ787362.1 |
| DL120279.1 | FW566099.1 | AX145478.1 | DM460865.1 | DL023436.1 | DL117499.1 | BD396347.1 | DL093553.1 | CQ786778.1 |
| DL120247.1 | FW559433.1 | AX145446.1 | HC056024.1 | DL023404.1 | DL117467.1 | BD438177.1 | DL089938.1 | CQ784703.1 |
| DL120215.1 | FW506880.1 | AX145414.1 | DM459941.1 | DL020389.1 | DL117435.1 | BD428088.1 | DL089906.1 | CQ784639.1 |
| DL115812.1 | FW506230.1 | AX145381.1 | DM381878.1 | DL020357.1 | DL112756.1 | BD427916.1 | DL089874.1 | CQ778524.1 |
| DL115780.1 | HI933892.1 | HW260100.1 | DQ408670.1 | DL020325.1 | DL107759.1 | BD437667.1 | DL089842.1 | CQ774817.1 |
| DL115748.1 | HI931708.1 | HW260068.1 | GN030212.1 | DL020293.1 | DL107727.1 | BD395353.1 | DL137586.1 | CQ772354.1 |
| DL111194.1 | HI930645.1 | HW260036.1 | GN029860.1 | DL020261.1 | DL107695.1 | BD459650.1 | DL123134.1 | A01474.1 |
| DL106091.1 | HI929589.1 | HW260004.1 | GN013401.1 | DL020229.1 | DL098318.1 | BD437173.1 | DL123102.1 | A05106.1 |
| DL106059.1 | HI935106.1 | HW259972.1 | GN033560.1 | DL020197.1 | DL098286.1 | BD405245.1 | DL118688.1 | A01418.1 |
| DL106027.1 | HI935032.1 | HW259940.1 | GN033528.1 | DL015794.1 | DL098254.1 | BD446172.1 | DL118656.1 | A00729.1 |
| DL105995.1 | HI929208.1 | HW259895.1 | GN033496.1 | DL015762.1 | DL098222.1 | BD393738.1 | DL118624.1 | M14412.1 |
| DL105963.1 | HI918318.1 | HW259748.1 | GN033464.1 | DL015730.1 | DL098190.1 | BD453353.1 | DL114228.1 | M12618.1 |
| DL101190.1 | HI918268.1 | HW259403.1 | GN033336.1 | DL047252.1 | DL098158.1 | BD453323.1 | DL114196.1 | K00656.1 |

| | | | | | | | | |
|---|---|---|---|---|---|---|---|---|
| DL101158.1 | HI661321.1 | HW259029.1 | GN033304.1 | DL047220.1 | DL092052.1 | BD453291.1 | DL114164.1 | M25027.1 |
| DL101126.1 | HI658877.1 | HW258908.1 | GN033272.1 | DL047188.1 | DL091988.1 | BD444971.1 | DJ044930.1 | M24153.1 |
| DL096616.1 | HI657790.1 | HW258843.1 | GN033240.1 | DL047156.1 | FB342565.1 | BD444957.1 | DJ044889.1 | M60109.1 |
| DL122554.1 | HI657204.1 | HW257327.1 | GN033208.1 | DL047060.1 | FB299248.1 | BD376361.1 | DJ028136.1 | M19562.1 |
| DL122522.1 | FW498192.1 | HW257199.1 | GN033176.1 | DL043238.1 | CS368266.1 | BD376037.1 | DJ030388.1 | AB539797.1 |
| DL118108.1 | FW496477.1 | HW257135.1 | GN033144.1 | DL043206.1 | CS368074.1 | BD375304.1 | DJ029992.1 | AY070236.1 |
| DL118076.1 | FW397872.1 | HW257103.1 | GN033080.1 | DL043174.1 | CS367690.1 | BD373914.1 | DJ029682.1 | LT908469.1 |
| DL118044.1 | FW397574.1 | HW257039.1 | GN033048.1 | DL043142.1 | DL075898.1 | BD364773.1 | DJ026326.1 | AH002286.2 |
| DL118012.1 | FW397487.1 | HW256879.1 | GN033016.1 | DL043110.1 | DL080111.1 | BD353735.1 | CS721636.1 | AH003154.2 |
| DL099022.1 | FW393465.1 | HW256847.1 | GN032984.1 | CS606914.1 | DL075839.1 | AX956811.1 | CS691405.1 | M13381.1 |
| DL098990.1 | FW393019.1 | HW256751.1 | GN032952.1 | CS606840.1 | DL075807.1 | AX952267.1 | CS693899.1 | M19978.1 |
| DL098958.1 | FW396337.1 | HW256719.1 | GN032920.1 | CS606798.1 | DL075762.1 | AX938902.1 | CS693243.1 | DQ250199.1 |
| DL117894.1 | FW395233.1 | HW251217.1 | GN032888.1 | CS606032.1 | DL079747.1 | AX937236.1 | CS682425.1 | AF191635.1 |
| DL117862.1 | FW417601.1 | HW250979.1 | GN032856.1 | CS605021.1 | DL075258.1 | AX935368.1 | CS674194.1 | AF003723.1 |
| DL117830.1 | HH833643.1 | HW250325.1 | GN032824.1 | CS604989.1 | DL079088.1 | AX934338.1 | CS691866.1 | AY270182.1 |
| DL098808.1 | HH833611.1 | HW249716.1 | GN032760.1 | CS604893.1 | DL079048.1 | AX928359.1 | DJ012310.1 | AF283514.1 |
| DL098776.1 | HH833579.1 | HW241204.1 | GN032729.1 | CS604861.1 | DL078964.1 | AX925696.1 | DJ008402.1 | HW390741.1 |
| DL098744.1 | HH833547.1 | HW247845.1 | GN032697.1 | CS604829.1 | DL078932.1 | AX923375.1 | DJ008370.1 | HW399314.1 |
| DL092734.1 | HH833515.1 | HW247805.1 | GN032665.1 | DL128543.1 | DL078901.1 | AX923410.1 | DJ004233.1 | HW408961.1 |
| DL092702.1 | HH833483.1 | HW240839.1 | GN032632.1 | DL125066.1 | DL073099.1 | AX923377.1 | CS450599.1 | HW391267.1 |
| DL088959.1 | HH833451.1 | HW240807.1 | GN032600.1 | DL125034.1 | DL013606.1 | AX840532.1 | DD360975.1 | HW390550.1 |
| DL088927.1 | HH833419.1 | HW240775.1 | GN032568.1 | DL125002.1 | DL020608.1 | AX838976.1 | DD354723.1 | HW390465.1 |
| DL088799.1 | HH833387.1 | HW240743.1 | GN032537.1 | DL124970.1 | DL016205.1 | AX829105.1 | DD359246.1 | HW389556.1 |
| HI642878.1 | FW392844.1 | HW240719.1 | GN032505.1 | DL120817.1 | DL016173.1 | AX826958.1 | DD359129.1 | HW388606.1 |
| HI637691.1 | FW381966.1 | HW240687.1 | GN032473.1 | DL139749.1 | DL016141.1 | AX824451.1 | DD357850.1 | HW387808.1 |
| HI380639.1 | HH931890.1 | HW240623.1 | GN032441.1 | DL124953.1 | DL031641.1 | AX824341.1 | DD357620.1 | HW290901.1 |
| HI378568.1 | HH821864.1 | HW239402.1 | GN032409.1 | DL124923.1 | DL031609.1 | AX823899.1 | DD357434.1 | HW290869.1 |
| HI212993.1 | HH826998.1 | HW239182.1 | GN032377.1 | DL124891.1 | DL031577.1 | AX823774.1 | CS447938.1 | HW290606.1 |
| HI546341.1 | HH822345.1 | HW238892.1 | GN032312.1 | DL120770.1 | DL031545.1 | AX822479.1 | A26307.1 | HW290327.1 |
| HI210987.1 | HH822037.1 | HW238495.1 | GN032280.1 | DL120738.1 | DL023668.1 | AX816387.1 | A12659.1 | HW289987.1 |
| HI210955.1 | FW379679.1 | HW238408.1 | GN032248.1 | DL116207.1 | DL023636.1 | AX816104.1 | A08112.1 | HW289013.1 |
| HI570387.1 | FW379646.1 | HW243003.1 | GN032216.1 | DL116175.1 | DL023604.1 | AX815007.1 | A02321.1 | HW267897.1 |
| HI566018.1 | FW379572.1 | HW237953.1 | GN032184.1 | DL116143.1 | DL015994.1 | AX814474.1 | CS443437.1 | HW267766.1 |
| HI472896.1 | FW378149.1 | HW237898.1 | GN032152.1 | DL116111.1 | DL015962.1 | AX814411.1 | CS441927.1 | HW267690.1 |
| HI209405.1 | FW377968.1 | HW237864.1 | GN032120.1 | DL103759.1 | DL015930.1 | AX809454.1 | CS438973.1 | HW267415.1 |
| HI072380.1 | HC313927.1 | HW248700.1 | GN032088.1 | DL099305.1 | DL015898.1 | AX799693.1 | CS436333.1 | HW266173.1 |
| HI004351.1 | HC318731.1 | HW242510.1 | GN032056.1 | DL093143.1 | DL015866.1 | AX798911.1 | DD349657.1 | HW266058.1 |
| HI004287.1 | HC310216.1 | HW242321.1 | GN032024.1 | DL093111.1 | DL011248.1 | AX798218.1 | CS433053.1 | HW265962.1 |
| HI004223.1 | HC310098.1 | HW242289.1 | GN031992.1 | DL093079.1 | DL011216.1 | AX797714.1 | CS423564.1 | HW263055.1 |
| HI002265.1 | HC310066.1 | HW242257.1 | GN031960.1 | DL089272.1 | DL047640.1 | AX796898.1 | CS417932.1 | HW262843.1 |
| HI002228.1 | HC310034.1 | HW061890.1 | GN031928.1 | DL089240.1 | DL047608.1 | GM623081.1 | CS414530.1 | HW261397.1 |
| HI002131.1 | HC310002.1 | HW061857.1 | GN031896.1 | DL125931.1 | DL047576.1 | GM623049.1 | CS416213.1 | HW261365.1 |
| HI002095.1 | HC309970.1 | HW061825.1 | GN031864.1 | DL125899.1 | DL047544.1 | GM623017.1 | CS415725.1 | HW261333.1 |
| HI000433.1 | HC308609.1 | HW061712.1 | GN031832.1 | DL125867.1 | DL039681.1 | GM622985.1 | CS415540.1 | HW261301.1 |
| HI000394.1 | HC307860.1 | HW065207.1 | GN031799.1 | DL125835.1 | DL039649.1 | GM622953.1 | CS410894.1 | HW261269.1 |
| HI203422.1 | HC307799.1 | DM131110.1 | GN031702.1 | DL125803.1 | DL039617.1 | GM650471.1 | CS410825.1 | HW261237.1 |
| HI179296.1 | FU258271.1 | HB441231.1 | GN031670.1 | DL125771.1 | DL039585.1 | GM650439.1 | CS410620.1 | HW261205.1 |

| | | | | | | | | |
|---|---|---|---|---|---|---|---|---|
| HI553280.1 | FU258207.1 | HB435246.1 | GN031638.1 | DL122561.1 | DL039553.1 | GM650407.1 | CS414840.1 | HW261173.1 |
| HI553208.1 | FU258175.1 | HB426462.1 | GN031606.1 | DL122529.1 | DL039521.1 | GM643599.1 | DD320653.1 | HW261141.1 |
| HI583960.1 | FU260925.1 | HB423119.1 | GN031574.1 | DL118179.1 | DL039489.1 | GM643567.1 | DD327026.1 | HW261109.1 |
| HI565619.1 | FU257899.1 | HB423081.1 | GN031542.1 | DL118115.1 | DL043365.1 | GM643535.1 | DD329899.1 | HW261077.1 |
| HI003874.1 | FU257706.1 | HB416460.1 | GN031510.1 | DL118083.1 | DL043333.1 | GM643503.1 | DD328890.1 | HW261045.1 |
| HI002054.1 | FU263070.1 | HB403498.1 | GN031478.1 | DL118051.1 | DL039388.1 | GM643471.1 | DD321800.1 | HW261013.1 |
| HI001965.1 | FU259886.1 | HB412946.1 | GN031445.1 | DL118019.1 | DL039356.1 | GM643438.1 | DD325735.1 | HW260949.1 |
| HI000297.1 | FU265344.1 | HB396996.1 | GN031413.1 | DL113330.1 | DL039324.1 | GM636449.1 | E03016.1 | HW260917.1 |
| HI000253.1 | FU250461.1 | HB396566.1 | GN031381.1 | DL113298.1 | DL035668.1 | GM636417.1 | E02870.1 | HW260885.1 |
| HI000211.1 | FU267893.1 | HB394813.1 | GN031349.1 | DL113266.1 | DL035604.1 | GM636385.1 | E01554.1 | HW260853.1 |
| HI000153.1 | FU265028.1 | HB394310.1 | BD453298.1 | DL108465.1 | DL035572.1 | GM636353.1 | E01364.1 | HW260821.1 |
| HI000121.1 | FU261565.1 | HB394278.1 | BD453266.1 | DL108433.1 | DL035540.1 | GM631849.1 | E01227.1 | HW260789.1 |
| HI137391.1 | FU261551.1 | HB394246.1 | BD194469.1 | DL108337.1 | DJ061544.1 | GM631817.1 | E01031.1 | HW260757.1 |
| HI204487.1 | FU258331.1 | HB393702.1 | BD187475.1 | DL108305.1 | DJ061496.1 | GM631785.1 | E00128.1 | HW260725.1 |
| HI204208.1 | DM476542.1 | HB397670.1 | AX777601.1 | DL103635.1 | DJ061448.1 | GM622854.1 | DD100277.1 | HW260693.1 |
| HI470706.1 | DM475252.1 | DM114307.1 | AX773258.1 | DL099029.1 | DJ055493.1 | GM622822.1 | DD143959.1 | CS118548.1 |
| HI470590.1 | DM472763.1 | DM115969.1 | AX770158.1 | DL098997.1 | DJ061199.1 | GM657939.1 | DD099333.1 | CS118515.1 |
| HI551659.1 | DM472587.1 | GM668928.1 | AX746407.1 | DL098965.1 | DJ055285.1 | GM657907.1 | DD084818.1 | CS118482.1 |
| HI559249.1 | HC299786.1 | GM038734.1 | BD183742.1 | DL098933.1 | DJ057671.1 | GM657875.1 | DD069877.1 | CS118418.1 |
| HI564739.1 | HC306249.1 | GM000682.1 | BD183124.1 | DL092827.1 | DJ060407.1 | GM657843.1 | DD112548.1 | CS118385.1 |
| HI568901.1 | HC306209.1 | HB388698.1 | BD181091.1 | DL092795.1 | DJ056884.1 | GM657811.1 | DD083447.1 | CS118351.1 |
| HI568843.1 | HC306169.1 | HB387127.1 | BD180842.1 | DL092763.1 | DJ053112.1 | GM657778.1 | DD158427.1 | CS118284.1 |
| HI564243.1 | HC305942.1 | HB386579.1 | AX744009.1 | DL088988.1 | DJ053080.1 | GM657746.1 | DD158371.1 | CS118252.1 |
| FW332128.1 | HC305902.1 | HB385918.1 | AX722076.1 | DL117965.1 | CS810636.1 | GM643387.1 | DD157350.1 | CS118220.1 |
| HC768268.1 | HC305862.1 | DL464554.1 | BD177394.1 | DL117901.1 | CS810527.1 | GM643355.1 | DD154088.1 | CS118153.1 |
| HC757171.1 | HC305822.1 | DL463074.1 | AX683725.1 | DL117869.1 | CS811047.1 | GM643323.1 | DD153643.1 | CS118121.1 |
| HC754646.1 | DM150249.1 | DL476427.1 | AX664354.1 | DL117837.1 | DJ016606.1 | GM643259.1 | DD152589.1 | CS118088.1 |
| HC732134.1 | DM150087.1 | DL462980.1 | AX657683.1 | DL023989.1 | DJ015676.1 | GM643227.1 | DD082125.1 | CS118056.1 |
| HC731668.1 | HB462485.1 | DL469817.1 | HW160604.1 | DL016411.1 | CS803367.1 | GM636238.1 | DD081755.1 | CS118022.1 |
| HC731118.1 | HB455308.1 | DL460980.1 | HW155817.1 | DL016379.1 | FB293606.1 | GM636206.1 | DD149128.1 | CS117986.1 |
| HC471761.1 | HB453862.1 | GM712169.1 | HW155694.1 | DL016347.1 | DJ046993.1 | GM636174.1 | DD148187.1 | CS117954.1 |
| HC471729.1 | HB451933.1 | GM651927.1 | HW150143.1 | DL016315.1 | DJ052311.1 | GM631670.1 | DD147788.1 | AX040759.1 |
| HC471665.1 | HB445374.1 | GM675556.1 | HW150111.1 | DL016283.1 | DD405027.1 | GM631638.1 | DD147756.1 | AX040175.1 |
| HC471633.1 | DM138462.1 | FB509359.1 | HW150047.1 | DL016251.1 | DD404631.1 | GM631574.1 | DD147724.1 | AX037315.1 |
| HC471601.1 | DM138430.1 | FB509261.1 | HW150015.1 | DL028152.1 | DD406805.1 | GM631510.1 | DD147692.1 | AX036011.1 |
| HC729740.1 | DM137118.1 | FB509214.1 | HW149983.1 | DL011735.1 | DD406076.1 | GM622739.1 | DD147134.1 | AX035656.1 |
| HC727365.1 | DM131554.1 | FB509182.1 | HW144832.1 | DL011703.1 | DD402370.1 | GM622707.1 | DD080837.1 | AX032817.1 |
| HC689120.1 | BD263423.1 | FB509150.1 | HW155371.1 | DL011671.1 | DD402338.1 | GM622675.1 | DD138756.1 | AX028796.1 |
| HC688496.1 | BD251964.1 | FB509112.1 | HW155240.1 | DL011639.1 | DD402242.1 | GM704175.1 | DD132352.1 | AX024034.1 |
| HC688419.1 | BD251462.1 | GM709015.1 | HW155208.1 | DL011607.1 | DD402210.1 | GM660771.1 | DD133879.1 | AX023688.1 |
| HC687703.1 | BD251235.1 | GM708798.1 | HW158789.1 | DL048462.1 | BD453898.1 | GM660739.1 | DD132594.1 | AX023605.1 |
| HC490857.1 | BD250270.1 | GM655012.1 | HW154823.1 | DL044845.1 | BD453866.1 | GM653520.1 | DD134592.1 | AX023573.1 |
| HC490825.1 | BD249060.1 | GM654980.1 | HW158530.1 | DL044781.1 | BD453834.1 | GM653456.1 | DD094906.1 | AX019327.1 |
| HC490793.1 | BD247112.1 | GM044475.1 | HW158399.1 | DL044749.1 | BD453802.1 | GM653424.1 | CS075344.1 | AX014770.1 |
| HC490761.1 | BD247010.1 | GM755075.1 | HW154653.1 | DL044717.1 | BD453746.1 | GM653392.1 | CS073671.1 | AX010928.1 |
| HC490729.1 | BD246840.1 | GM043752.1 | HW158234.1 | DL044685.1 | BD453714.1 | GM646393.1 | CS070454.1 | AX008745.1 |
| HC490697.1 | BD244789.1 | GM836926.1 | HW154486.1 | DL044533.1 | BD453682.1 | GM646361.1 | CS063836.1 | AX003023.1 |

| | | | | | | | | |
|---|---|---|---|---|---|---|---|---|
| HC490536.1 | BD243478.1 | GM754518.1 | HW154416.1 | DL044501.1 | BD453650.1 | GM646329.1 | CS061115.1 | AJ286131.1 |
| HC490501.1 | BD243445.1 | GM754230.1 | HW147143.1 | DL044469.1 | BD453618.1 | GM646297.1 | CS059005.1 | A57337.1 |
| HC490366.1 | BD242711.1 | FB511155.1 | HW154030.1 | DL040325.1 | BD453581.1 | GM646265.1 | AY967383.1 | A44217.1 |
| HC472290.1 | BD242460.1 | GM752807.1 | HW153770.1 | DL036605.1 | BD453568.1 | GM646233.1 | AY967351.1 | A25436.1 |
| HC472258.1 | BD240788.1 | GM752449.1 | HW153730.1 | DL036573.1 | BD453536.1 | GM639430.1 | AY967319.1 | A35728.1 |
| HC472226.1 | BD238480.1 | GM643210.1 | HW146404.1 | DL036541.1 | BD453504.1 | GM639398.1 | AY967287.1 | A35304.1 |
| HC471962.1 | BD238038.1 | GM622451.1 | HW145788.1 | DL032242.1 | BD453472.1 | GM639366.1 | AY967255.1 | A29461.1 |
| HC471930.1 | BD237862.1 | GM622419.1 | HW153504.1 | DL032210.1 | BD453440.1 | GM639334.1 | DL123315.1 | A33971.1 |
| HC471898.1 | BD237450.1 | GM622387.1 | HW145523.1 | DL032178.1 | BD453408.1 | GM639302.1 | DL123283.1 | A32460.1 |
| HC471866.1 | BD236837.1 | GM622355.1 | HW145147.1 | DL048448.1 | BD453263.1 | GM639270.1 | DL123251.1 | A30364.1 |
| HB454949.1 | BD235796.1 | GM657541.1 | HW153368.1 | DL048416.1 | BD453231.1 | GM626723.1 | DL123219.1 | A27328.1 |
| HB445182.1 | BD235648.1 | GM657509.1 | HW153323.1 | DL048384.1 | BD453199.1 | GM660568.1 | DL123187.1 | A13706.1 |
| DM131274.1 | BD234874.1 | GM657477.1 | HW151773.1 | DL048352.1 | BD432489.1 | GM660536.1 | DL118901.1 | A25928.1 |
| HB423262.1 | BD233452.1 | GM657445.1 | HW150561.1 | DL048320.1 | BD399492.1 | GM653349.1 | DL118869.1 | A14256.1 |
| HB412736.1 | BD231939.1 | GM657413.1 | HW150529.1 | DL044402.1 | BD399015.1 | GM653317.1 | DL118837.1 | A20089.1 |
| HB394297.1 | BD231168.1 | GM657381.1 | HW150465.1 | DL044370.1 | BD497557.1 | GM653285.1 | DL118807.1 | A18668.1 |
| HB394265.1 | BD231057.1 | GM657349.1 | HW150433.1 | DL044338.1 | BD495661.1 | GM653253.1 | DL109253.1 | A11187.1 |
| HB394233.1 | BD229108.1 | GM650157.1 | HW150401.1 | DL044306.1 | BD451724.1 | GM653221.1 | DL109221.1 | A04789.1 |
| DM114260.1 | BD227236.1 | GM650125.1 | HV988674.1 | DL044274.1 | BD408106.1 | GM653189.1 | DL109189.1 | A00371.1 |
| DM115562.1 | BD227015.1 | GM650093.1 | HV984517.1 | DL040098.1 | BD419032.1 | GM646094.1 | DL109157.1 | A31752.1 |
| DM114855.1 | BD225786.1 | GM643124.1 | HW029009.1 | DL032041.1 | BD429571.1 | GM646062.1 | DL109125.1 | A30244.1 |
| GN368624.1 | BD225754.1 | GM643092.1 | HW028768.1 | DL032009.1 | BD429346.1 | GM646030.1 | DL109093.1 | A29010.1 |
| HB384530.1 | BD225722.1 | GM643060.1 | HV984332.1 | DL031977.1 | BD397114.1 | GM639131.1 | DL104359.1 | A28858.1 |
| HB340026.1 | BD225690.1 | GM643028.1 | HV984240.1 | DJ437233.1 | BD396510.1 | GM639099.1 | DL104327.1 | A25856.1 |
| HA641588.1 | BD225658.1 | GM636007.1 | HV986645.1 | CQ849487.1 | BD438364.1 | GM639067.1 | DL089968.1 | A20016.1 |
| GM645188.1 | BD225626.1 | GM635975.1 | HV986016.1 | CQ849242.1 | BD396025.1 | GM626520.1 | DL089936.1 | A28270.1 |
| GM638133.1 | BD224838.1 | GM635943.1 | HV965124.1 | CQ840687.1 | BD428077.1 | GM626488.1 | DL089904.1 | HW122421.1 |
| GM638101.1 | BD223259.1 | GM635911.1 | HV961300.1 | CQ834934.1 | BD459638.1 | GM626456.1 | DL089872.1 | HW122125.1 |
| GM638069.1 | BD222955.1 | GM635879.1 | HV961004.1 | CQ831462.1 | BD404921.1 | GM626424.1 | DL089840.1 | HW120876.1 |
| AX347270.1 | BD222510.1 | GM622219.1 | HV959519.1 | CQ830722.1 | BD453349.1 | GM626392.1 | DL123132.1 | HW117993.1 |
| AX347234.1 | BD221774.1 | GM622187.1 | HV964050.1 | CQ829829.1 | BD453319.1 | GM626360.1 | DL118686.1 | HW115146.1 |
| AX347200.1 | BD217956.1 | GM622155.1 | HV970077.1 | CQ826847.1 | BD453287.1 | GM626328.1 | DL118654.1 | HW101674.1 |
| AX347164.1 | BD211409.1 | GM631014.1 | HV963872.1 | CQ824422.1 | BD456396.1 | GM660353.1 | DL118622.1 | HW096984.1 |
| AX344908.1 | BD205391.1 | GM630982.1 | HV963680.1 | CQ818939.1 | BD391452.1 | GM653166.1 | DL114226.1 | HW096676.1 |
| AX329476.1 | DD282961.1 | GM630950.1 | HV966195.1 | CQ818563.1 | BD388889.1 | GM653134.1 | DL114194.1 | HW072894.1 |
| AX328973.1 | DD287667.1 | GM630918.1 | HV966161.1 | CQ817013.1 | BD388047.1 | GM653102.1 | DL114162.1 | HW105298.1 |
| AX328150.1 | DD279452.1 | GM630893.1 | HV958157.1 | CQ816974.1 | BD376345.1 | GM653070.1 | DL114130.1 | HW096205.1 |
| AX269000.1 | CS355554.1 | GM635850.1 | HV963323.1 | CQ816934.1 | BD375874.1 | GM653038.1 | DL114098.1 | HW104327.1 |
| AX259251.1 | CS350579.1 | GM635818.1 | HV965991.1 | CQ815743.1 | AX962034.1 | GM653006.1 | DL114066.1 | HW104293.1 |
| AX286173.1 | BD297839.1 | GM635786.1 | HV965959.1 | CQ814528.1 | AX960368.1 | GM639012.1 | DL126206.1 | HW071116.1 |
| AX283496.1 | BD296982.1 | GM635754.1 | HV965489.1 | CQ814093.1 | AX959316.1 | GM638980.1 | DL126174.1 | HW070911.1 |
| AX255413.1 | BD296938.1 | GM635722.1 | HV962276.1 | AX143829.1 | AX954894.1 | GM638948.1 | DL122958.1 | HV694115.1 |
| AX253430.1 | BD296884.1 | GM635690.1 | HV962045.1 | AX143765.1 | AX938889.1 | GM638916.1 | DL122926.1 | HV700008.1 |
| AX244980.1 | BD289752.1 | GM630854.1 | HV965289.1 | AX143701.1 | AX925675.1 | GM638884.1 | DL122894.1 | HV693337.1 |
| AX242314.1 | BD287342.1 | GM630822.1 | HV961793.1 | AX143573.1 | AX923406.1 | GM638852.1 | DL122862.1 | HV689459.1 |
| AX242250.1 | BD276774.1 | GM630790.1 | HV956087.1 | AX143509.1 | AX923372.1 | GM626173.1 | DL122830.1 | HV695881.1 |
| AX242218.1 | BD278972.1 | GM630758.1 | HV951634.1 | AX143445.1 | AX840312.1 | DL049399.1 | DL122798.1 | HV695544.1 |

| | | | | | | | | |
|---|---|---|---|---|---|---|---|---|
| AX242186.1 | BD274145.1 | GM630726.1 | HV951602.1 | AX143381.1 | AX825034.1 | DL049367.1 | DL122766.1 | HV593784.1 |
| AX242154.1 | BD273674.1 | GM630694.1 | HV951570.1 | AX143317.1 | AX824447.1 | DL049335.1 | DL114020.1 | HV585144.1 |
| AX242122.1 | BD273294.1 | GM657320.1 | HV951538.1 | AX143253.1 | AX824337.1 | DL049112.1 | DL041726.1 | HV585112.1 |
| AX241898.1 | BD272235.1 | GM657288.1 | HV950682.1 | AX143189.1 | AX823872.1 | DL012572.1 | DL041694.1 | HV579306.1 |
| AX241866.1 | BD271045.1 | GM661111.1 | HV950603.1 | AX143061.1 | AX816096.1 | DL012540.1 | DL049636.1 | HV592473.1 |
| AX241834.1 | CS279423.1 | GM661079.1 | HV957877.1 | AX142933.1 | AX815001.1 | DL012508.1 | DL049604.1 | HV592441.1 |
| AX241802.1 | DD229945.1 | GM661047.1 | HV949012.1 | AX142869.1 | AX814470.1 | DL012476.1 | DL041557.1 | HV592409.1 |
| AX241738.1 | DD228593.1 | GM661015.1 | HV948764.1 | AX142805.1 | AX802894.1 | DL012444.1 | DL041525.1 | HV585566.1 |
| AX241706.1 | DD228427.1 | GM660983.1 | HV947299.1 | AX142741.1 | AX809450.1 | DL012412.1 | DL037670.1 | HV570630.1 |
| AX241642.1 | DD227387.1 | DL119633.1 | HV947109.1 | AX142677.1 | AX805216.1 | DL045454.1 | DL037638.1 | HV566135.1 |
| AX241610.1 | DD226691.1 | DL114910.1 | HV952900.1 | AX142419.1 | AX799660.1 | DL045422.1 | DL037606.1 | HV573963.1 |
| AX241578.1 | DD225226.1 | DL114878.1 | HV952827.1 | AX142355.1 | AX798843.1 | DL045358.1 | DL037574.1 | HV344902.1 |
| AX241546.1 | DD224659.1 | DL114846.1 | HV952505.1 | AX142291.1 | AX798339.1 | DL041246.1 | DL037542.1 | HV344626.1 |
| AX241482.1 | DD224175.1 | DL119587.1 | HV952264.1 | AX142227.1 | AX798196.1 | DL041214.1 | DL033307.1 | HV339981.1 |
| AX241450.1 | DD223905.1 | DL119555.1 | HV951950.1 | AX142163.1 | AX797702.1 | DL041182.1 | DL033275.1 | HV339949.1 |
| HV945115.1 | DD234122.1 | DL119523.1 | HV951862.1 | AX142033.1 | AX796860.1 | DL041118.1 | DL033243.1 | HV339657.1 |
| HV780051.1 | DD231583.1 | DL119491.1 | DL194519.1 | AX141775.1 | AX796744.1 | DL041086.1 | DL033211.1 | HV344512.1 |
| HV777030.1 | DD231487.1 | DL119459.1 | DL194303.1 | AX141711.1 | AX795620.1 | DL037199.1 | DL033179.1 | HV342187.1 |
| HV775603.1 | DD231443.1 | DL119427.1 | DL194201.1 | AX141583.1 | AX795431.1 | DL037167.1 | DL033147.1 | HV342163.1 |
| HV743718.1 | CS276980.1 | DL123738.1 | DL188833.1 | AX141519.1 | BD185762.1 | DL037135.1 | DL029139.1 | HV322788.1 |
| HW287347.1 | DD219621.1 | DL123706.1 | DL198996.1 | AX139751.1 | BD188844.1 | DL028732.1 | DL029107.1 | HV321736.1 |
| HW302516.1 | DD214728.1 | DL123674.1 | DL196375.1 | AX137108.1 | AX787433.1 | DL028700.1 | DL029075.1 | CS476147.1 |
| HW295690.1 | DD212851.1 | DL123642.1 | DL196318.1 | AX133309.1 | AX785164.1 | DL028668.1 | DL021761.1 | CS467714.1 |
| HW302335.1 | DD214021.1 | DL123610.1 | DL193882.1 | AX127563.1 | AX241431.1 | DL028636.1 | DL021729.1 | CS465363.1 |
| HW302010.1 | DD216642.1 | DL123578.1 | DL196137.1 | AX119971.1 | AX241124.1 | DL028604.1 | DL021697.1 | CS464601.1 |
| HW294998.1 | DD213972.1 | DL119388.1 | DL193837.1 | AX113753.1 | AX241092.1 | DL028572.1 | DL021665.1 | DD400177.1 |
| HW294129.1 | DD213879.1 | DL119356.1 | DL193805.1 | AX111698.1 | AX241060.1 | DL024758.1 | DL021633.1 | DD400144.1 |
| HW294097.1 | DD213847.1 | DL119324.1 | DL193773.1 | AX108259.1 | AX241028.1 | DL024726.1 | DL017395.1 | CS459126.1 |
| HW294020.1 | DD213815.1 | DL114832.1 | DL124159.1 | AX103641.1 | AX240996.1 | DL024694.1 | DL017363.1 | DD361275.1 |
| HW291154.1 | DD213798.1 | DL114800.1 | DL110244.1 | AX100350.1 | AX240964.1 | DL012370.1 | DL017331.1 | DD367535.1 |
| HW291057.1 | DD213766.1 | DL114768.1 | DL110212.1 | AX097517.1 | AX240932.1 | DL048691.1 | DL012942.1 | DD368137.1 |
| HW291025.1 | DD213734.1 | DL114736.1 | DL110180.1 | AX097475.1 | AX236854.1 | DL048659.1 | DL012878.1 | DD361309.1 |
| HW290961.1 | DD213702.1 | DL114704.1 | DL110148.1 | AX093097.1 | AX207894.1 | DL045241.1 | DL041350.1 | CS457344.1 |
| HW290898.1 | DD213670.1 | DL114672.1 | DL110116.1 | AX088790.1 | AX207288.1 | DL045209.1 | DL041286.1 | CS457174.1 |
| HW290866.1 | DD213638.1 | DL135076.1 | DL110084.1 | AX088746.1 | AX205123.1 | DL045177.1 | DL037399.1 | CS456034.1 |
| HW289977.1 | DD213606.1 | DL127148.1 | DL105247.1 | AX088690.1 | AX202548.1 | DL045145.1 | DL037367.1 | CS453772.1 |
| HW298006.1 | DD213588.1 | DL141490.1 | DL105215.1 | AX082944.1 | AX202417.1 | DL038791.1 | DL037335.1 | CS452565.1 |
| DI244367.1 | DD213556.1 | DL123538.1 | DL105183.1 | AX076476.1 | AX196251.1 | DL038759.1 | DL010187.1 | E01488.1 |
| HW267894.1 | DD213507.1 | DL123506.1 | DL105151.1 | AX068355.1 | HH961252.1 | DL038727.1 | DL010171.1 | E01402.1 |
| HW267816.1 | DD216036.1 | DL123474.1 | DL105119.1 | HW328450.1 | HH964351.1 | DL038695.1 | DL010139.1 | E01299.1 |
| HW267763.1 | DD216004.1 | DL123442.1 | DL100705.1 | HW328352.1 | HH982232.1 | DL035071.1 | DL010107.1 | E01177.1 |
| HW267678.1 | DD215972.1 | DL123410.1 | DL100673.1 | HW335694.1 | HH974543.1 | DL035039.1 | DL010075.1 | E00789.1 |
| HW266183.1 | DD218337.1 | DL123378.1 | DL100641.1 | HW335652.1 | HH981878.1 | DL034832.1 | DL010043.1 | DD099262.1 |
| HW266138.1 | GM630885.1 | DL119124.1 | DL100609.1 | HW328207.1 | HH998069.1 | DL030724.1 | DL010011.1 | DD117665.1 |
| HV959601.1 | GM630853.1 | DL119092.1 | DL100545.1 | HW318488.1 | HH998017.1 | DL030692.1 | DL014513.1 | DD158388.1 |
| HV964076.1 | GM630821.1 | DL119060.1 | DL119999.1 | HW335183.1 | HH997957.1 | DL030532.1 | DL014481.1 | DD158350.1 |
| HV958880.1 | GM630789.1 | DL119028.1 | DL119967.1 | HW335140.1 | HH999655.1 | DL026723.1 | DL014449.1 | DD157369.1 |

| | | | | | | | | |
|---|---|---|---|---|---|---|---|---|
| HV963332.1 | GM630757.1 | DL114632.1 | DL119935.1 | HW314204.1 | HH997906.1 | DL026691.1 | DL009863.1 | DD152404.1 |
| HV965631.1 | GM630725.1 | DL114600.1 | DL119903.1 | HW314031.1 | HH997835.1 | DL026659.1 | DL009831.1 | DD082934.1 |
| HV969939.1 | GM630693.1 | DL114568.1 | DL119871.1 | HW312173.1 | HH997769.1 | DL026627.1 | DL009799.1 | DD082063.1 |
| HV965326.1 | GM657319.1 | DL114536.1 | DL119839.1 | HW312053.1 | HH999509.1 | DL026595.1 | DL009767.1 | DD147807.1 |
| HV961767.1 | GM657287.1 | DL114504.1 | DL119807.1 | HW311833.1 | HH999452.1 | DL026563.1 | DL009543.1 | DD147775.1 |
| HV961701.1 | GM657255.1 | DL114472.1 | DL115212.1 | HW311247.1 | HH999414.1 | DL019576.1 | DL009511.1 | DD147743.1 |
| HV956408.1 | GM657223.1 | DL109575.1 | DL115180.1 | HW311074.1 | HH999354.1 | DL019544.1 | DL009479.1 | DD147711.1 |
| HV951144.1 | GM642967.1 | DL109543.1 | DL115148.1 | HW308074.1 | HH997649.1 | DL019512.1 | DL009447.1 | DD147679.1 |
| HV950614.1 | GM642934.1 | DL109511.1 | DL115116.1 | HW307867.1 | HH997616.1 | DL019480.1 | DL009415.1 | DD138007.1 |
| HV947307.1 | GM642902.1 | DL104873.1 | DL115084.1 | HW307835.1 | HH995711.1 | DL019448.1 | DL030140.1 | DD137173.1 |
| HV953006.1 | GM642838.1 | DL104841.1 | DL115052.1 | HW307803.1 | HH999697.1 | DL015013.1 | DL018821.1 | DD135977.1 |
| HV952058.1 | GM635528.1 | DL104809.1 | DL110052.1 | HW316583.1 | HH999247.1 | DL014981.1 | DL034456.1 | DD132318.1 |
| HV939876.1 | GM635496.1 | DL104745.1 | DL110020.1 | HW307733.1 | HH999202.1 | DL014949.1 | DL034328.1 | DD092998.1 |
| HV945600.1 | GM635464.1 | DL095918.1 | DL109988.1 | HW307701.1 | HH999153.1 | DL014917.1 | CS102528.1 | DD092924.1 |
| HV943362.1 | GM630658.1 | DL095886.1 | DL109956.1 | HW315955.1 | HH998119.1 | DL014885.1 | CS102496.1 | DD163901.1 |
| HV942437.1 | GM630626.1 | DL095854.1 | DL109924.1 | HW315600.1 | HH999077.1 | DL010267.1 | CS101489.1 | CS157815.1 |
| HV931340.1 | GM630594.1 | DL095822.1 | DL109892.1 | HW315561.1 | HH999032.1 | DL010235.1 | CS095641.1 | CS157783.1 |
| HV515986.1 | GM621659.1 | DL095790.1 | DL109870.1 | HW315454.1 | HH998971.1 | DL030373.1 | CS080792.1 | CS157944.1 |
| HV515600.1 | GM621627.1 | DL095758.1 | DL109838.1 | HW315168.1 | HH998924.1 | DL030384.1 | CS078826.1 | CS157912.1 |
| HV515568.1 | GM621595.1 | DL094135.1 | DL109806.1 | HW315072.1 | HH997142.1 | DL030344.1 | CS075437.1 | CS157880.1 |
| HV515440.1 | GM621563.1 | DL094103.1 | DL109774.1 | HW314834.1 | HH998869.1 | DL026527.1 | CS073669.1 | CS124701.1 |
| HV515408.1 | FB505558.1 | DL094071.1 | DL109742.1 | HW314778.1 | HH998810.1 | DL026471.1 | CS073056.1 | CS124627.1 |
| HV515376.1 | GM657111.1 | DL090264.1 | DL109710.1 | HW314616.1 | HH998755.1 | DL026439.1 | CS068655.1 | CS119824.1 |
| HV515344.1 | GM657079.1 | DL090232.1 | DL109678.1 | HW121467.1 | HH997095.1 | DL026407.1 | CS063835.1 | CS119529.1 |
| HV515312.1 | GM657047.1 | DL022003.1 | DL105072.1 | HW112771.1 | HH997003.1 | DL026375.1 | AX644612.1 | CS119463.1 |
| HV515280.1 | GM657015.1 | DL025354.1 | DL105040.1 | HW112673.1 | HH996949.1 | DL022563.1 | AY967382.1 | CS119428.1 |
| HV515248.1 | GM656983.1 | DL025322.1 | DL104976.1 | HW112611.1 | HH998704.1 | DL022531.1 | AY967350.1 | CS119363.1 |
| HV514261.1 | GM656951.1 | DL025290.1 | DL104944.1 | HW071780.1 | HH998665.1 | DL019356.1 | AY967318.1 | CS119297.1 |
| HV508675.1 | GM656920.1 | DL025226.1 | DL104912.1 | HW069952.1 | HH998597.1 | DL014650.1 | AY967286.1 | CS119265.1 |
| HV508640.1 | GM656888.1 | DL025194.1 | DL100498.1 | HW069920.1 | HH998542.1 | DJ044892.1 | AY967254.1 | CS119201.1 |
| HV508608.1 | GM656856.1 | DL021950.1 | DL100466.1 | HC688436.1 | HH996921.1 | DJ030499.1 | AY967222.1 | CS119104.1 |
| HV508576.1 | GM656824.1 | DL021918.1 | DL100434.1 | HC490834.1 | HH996817.1 | DJ033846.1 | AY967190.1 | CS119070.1 |
| HV508512.1 | GM656792.1 | DL021886.1 | DL100402.1 | HC490802.1 | HH996765.1 | DJ033541.1 | AY967158.1 | CS119037.1 |
| HV508484.1 | GM649767.1 | DL021822.1 | DL100370.1 | HC490770.1 | HH994556.1 | DJ030004.1 | AY967126.1 | CS119004.1 |
| HV512286.1 | GM649735.1 | DL021790.1 | DL100338.1 | HC490738.1 | HH977636.1 | DJ029930.1 | AY967094.1 | CS118972.1 |
| HH969927.1 | GM649703.1 | DL017424.1 | DL096091.1 | HC490706.1 | HH980636.1 | CS721642.1 | AY967062.1 | CS118873.1 |
| HH980619.1 | GM661590.1 | DL038460.1 | DL096059.1 | HC490674.1 | HH980566.1 | CS693902.1 | AY967030.1 | CS118841.1 |
| HH980515.1 | GM661558.1 | DL038428.1 | DL124151.1 | HC490510.1 | HH980495.1 | CS674199.1 | AY966998.1 | CS118807.1 |
| HH980447.1 | GM661526.1 | DL038396.1 | DL124119.1 | HC490478.1 | HH980354.1 | CS692003.1 | AY966966.1 | CS118775.1 |
| HB475483.1 | GM654532.1 | DL038364.1 | DL124087.1 | HC490407.1 | HH980294.1 | DJ008405.1 | AY966934.1 | CS118742.1 |
| HB469631.1 | GM654468.1 | DL038332.1 | DL124055.1 | DM144906.1 | HH980154.1 | DJ008373.1 | CS055309.1 | CS118710.1 |
| HB469125.1 | GM654436.1 | DL013169.1 | DL124023.1 | HB455304.1 | HH980122.1 | DJ011706.1 | CS054327.1 | CS118678.1 |
| HB468758.1 | GM654404.1 | DL013137.1 | DL123991.1 | HB455024.1 | HH979915.1 | CS671269.1 | CS052383.1 | CS118646.1 |
| DM148277.1 | GM647283.1 | DL013105.1 | DL119801.1 | HB451925.1 | HH979877.1 | CS670994.1 | CS052348.1 | CS118614.1 |
| DM152592.1 | GM647251.1 | DL013073.1 | DL119769.1 | DM143071.1 | HH996703.1 | DD462500.1 | CS052315.1 | CS118581.1 |
| DM150034.1 | GM647219.1 | DL013041.1 | DL119737.1 | DM137620.1 | HH996640.1 | CS647651.1 | BD130466.1 | CS118546.1 |
| HB454849.1 | GM627709.1 | DL013009.1 | DL119705.1 | DM137108.1 | HH996585.1 | CS646652.1 | BD107700.1 | CS118513.1 |

| | | | | | | | | |
|---|---|---|---|---|---|---|---|---|
| DM139718.1 | GM627677.1 | DL042146.1 | DL119673.1 | DM136207.1 | HH996544.1 | CS646199.1 | BD105720.1 | CS118480.1 |
| DM138073.1 | GM627645.1 | DL042114.1 | DL119641.1 | HB426104.1 | HH998401.1 | CS644251.1 | BD090565.1 | CS118416.1 |
| DM137799.1 | GM627585.1 | DL042082.1 | DL119609.1 | HB423115.1 | HH998362.1 | DD460513.1 | BD085725.1 | CS118383.1 |
| DM130831.1 | GM627553.1 | DL033703.1 | DL115014.1 | HB423076.1 | HH999940.1 | DD460153.1 | BD082907.1 | CS118349.1 |
| HB435573.1 | GM627521.1 | DL033671.1 | DL114982.1 | HB416452.1 | HH993945.1 | DD455863.1 | BD081898.1 | CS118315.1 |
| GN082365.1 | GM661494.1 | DL033639.1 | DL114950.1 | HB412673.1 | HH979816.1 | GM635265.1 | BD081711.1 | CS118282.1 |
| GN079251.1 | GM661430.1 | DL029535.1 | DL114918.1 | HB396992.1 | HH996541.1 | GM630459.1 | BD081319.1 | CS118250.1 |
| GN075909.1 | GM661398.1 | DL029503.1 | DL114886.1 | HB395991.1 | HH999820.1 | GM630427.1 | BD080678.1 | CS118184.1 |
| L09143.1 | GM661366.1 | DL029471.1 | DL114854.1 | HB394274.1 | HH999709.1 | GM630395.1 | BD080430.1 | CS118151.1 |
| L08925.1 | GM661334.1 | DL046105.1 | DL123934.1 | HB394242.1 | HH998275.1 | GM630363.1 | BD080139.1 | CS118119.1 |
| L08863.1 | GM654308.1 | CQ898617.1 | DL123902.1 | HB397725.1 | HH998239.1 | GM630331.1 | BD077095.1 | CS118086.1 |
| M27604.1 | GM654276.1 | CQ898585.1 | DL123870.1 | DM118013.1 | HH998191.1 | GM630299.1 | BD074981.1 | CS118054.1 |
| DM058889.1 | GM647187.1 | AX241062.1 | DL123838.1 | DM115614.1 | HH998150.1 | GM621524.1 | BD074946.1 | CS118020.1 |
| DM058809.1 | GM647155.1 | AX241030.1 | DL123806.1 | DM114889.1 | HH993701.1 | GM621492.1 | BD074654.1 | CS114629.1 |
| DM045496.1 | GM647123.1 | AX240998.1 | DL123774.1 | GM038723.1 | HH986632.1 | GM649465.1 | BD073246.1 | HW265959.1 |
| DM045317.1 | GM647091.1 | AX240966.1 | DL119595.1 | GM061289.1 | HH979525.1 | GM649433.1 | BD063668.1 | HW262942.1 |
| DM045052.1 | GM647051.1 | AX240934.1 | DL119563.1 | DM044845.1 | DM114863.1 | GM649401.1 | BD056717.1 | HW261394.1 |
| DM044870.1 | GM627517.1 | AX236998.1 | DL119531.1 | DM044665.1 | GM668917.1 | GM670041.1 | BD017721.1 | HW261362.1 |
| DM060786.1 | GM627485.1 | AX235770.1 | DL119499.1 | DM060706.1 | GM042095.1 | GM656536.1 | BD014227.1 | HW261330.1 |
| DM044752.1 | GM627453.1 | AX235232.1 | DL119467.1 | DM060674.1 | HB388685.1 | GM656504.1 | BD014195.1 | HW261298.1 |
| GN067944.1 | GM627421.1 | AX225201.1 | DL119435.1 | DM059964.1 | HB387467.1 | GM656472.1 | BD014126.1 | HW261266.1 |
| GN067963.1 | GM627389.1 | AX207290.1 | DL123746.1 | GN067962.1 | HA642984.1 | GM649307.1 | E63777.1 | HW261234.1 |
| GN067907.1 | GM627357.1 | AX205126.1 | HC474170.1 | GN065108.1 | HA641602.1 | GM649311.1 | AX481249.1 | HW261202.1 |
| GN067751.1 | GM627325.1 | AX197541.1 | HC491769.1 | GN061110.1 | HA641538.1 | GM649255.1 | AX474740.1 | HW261170.1 |
| GN052368.1 | GM661291.1 | AY043011.1 | HC481601.1 | GN059995.1 | HA641137.1 | GM649223.1 | E51072.1 | HW261138.1 |
| GN059766.1 | GM661259.1 | AX193681.1 | HC481564.1 | GN052382.1 | HA635313.1 | GM649191.1 | AF458111.1 | HW261106.1 |
| GN051246.1 | GM661227.1 | AX188684.1 | HC491462.1 | GN046270.1 | HA639755.1 | GM642582.1 | AX468868.1 | HW261074.1 |
| GN051094.1 | GM661195.1 | AX174630.1 | HC472626.1 | DM019381.1 | HA637714.1 | GM642550.1 | AX468392.1 | CS606867.1 |
| GM656254.1 | GM654137.1 | AX175219.1 | FV533443.1 | DM023387.1 | DM102629.1 | GM642466.1 | AX463657.1 | CS606814.1 |
| GM656222.1 | GM654105.1 | AX172940.1 | FV532170.1 | DM022299.1 | DM109926.1 | GM642453.1 | A23372.1 | CS604781.1 |
| GM656190.1 | GM654073.1 | AX168198.1 | FV531692.1 | DM038571.1 | DM095090.1 | GM642421.1 | AX458646.1 | CS604653.1 |
| GM719322.1 | GM654041.1 | AX167567.1 | FV531410.1 | DM022192.1 | DM094872.1 | GM635239.1 | AX458506.1 | CS592847.1 |
| GM634546.1 | GM654009.1 | AX167411.1 | FV523611.1 | DM021885.1 | GN368616.1 | GM635207.1 | AX456487.1 | CS592359.1 |
| GM634514.1 | GM653977.1 | AX166326.1 | FV530986.1 | DM015975.1 | GN373299.1 | GM635175.1 | AX455874.1 | CS592087.1 |
| GM634482.1 | GM646984.1 | AX155102.1 | FV522830.1 | DM027070.1 | GN360154.1 | GM635111.1 | AX454136.1 | CS589292.1 |
| GM664832.1 | GM646952.1 | AX149423.1 | FV522790.1 | GN045579.1 | GN360090.1 | GM635079.1 | AX452895.1 | CS593469.1 |
| GM655914.1 | GM646920.1 | AX145731.1 | FV530352.1 | GN043674.1 | GN356159.1 | GM630273.1 | AX451590.1 | CS597708.1 |
| GM648729.1 | GM646888.1 | AX145699.1 | FV530205.1 | GN041641.1 | DM045519.1 | GM630241.1 | AX443291.1 | CS585209.1 |
| GM648697.1 | GM646856.1 | AX145667.1 | FV529028.1 | GN040019.1 | DM045383.1 | GM630209.1 | A29534.1 | CS583570.1 |
| GM648665.1 | GM646824.1 | AX145603.1 | FV528650.1 | GN037871.1 | DM045236.1 | GM630177.1 | AX429437.1 | CS582103.1 |
| GM648633.1 | GM640021.1 | AX145571.1 | FV534479.1 | GN037292.1 | DM044939.1 | GM737353.1 | AX428529.1 | CS575765.1 |
| GM648601.1 | GM639989.1 | AX145539.1 | FV534427.1 | GN034782.1 | DM039604.1 | GM698653.1 | AX427946.1 | CS574808.1 |
| GM634449.1 | GM639957.1 | AX145507.1 | FV534387.1 | GN030743.1 | DM044828.1 | GM698557.1 | AX427112.1 | DD222018.1 |
| GM629462.1 | GM639925.1 | AX145475.1 | FV533996.1 | GN030711.1 | DM044663.1 | GM635044.1 | AX420465.1 | CS255455.1 |
| GM619897.1 | GM639893.1 | AX145443.1 | HC465680.1 | GN030647.1 | A08829.1 | GM635012.1 | AX418552.1 | DD212022.1 |
| DL184533.1 | GM627287.1 | AX145411.1 | HB444517.1 | GN030615.1 | A05169.1 | GM634980.1 | AX418286.1 | DD211603.1 |
| DL193892.1 | GM627255.1 | AX145378.1 | DM121080.1 | GN030583.1 | A04054.1 | GM634948.1 | AX417936.1 | DD206906.1 |

| | | | | | | | | |
|---|---|---|---|---|---|---|---|---|
| DL184307.1 | GM627159.1 | AX145314.1 | DM139898.1 | GN030551.1 | A01578.1 | GM634916.1 | HW341711.1 | DD206874.1 |
| DL193713.1 | GM627127.1 | AX145282.1 | DM134515.1 | GN030487.1 | A01339.1 | GM634884.1 | HW341587.1 | DD206842.1 |
| FB571345.1 | HV766042.1 | AX145250.1 | DM138468.1 | GN030409.1 | U12267.1 | GM629950.1 | HW351065.1 | DD206812.1 |
| FB571093.1 | HV766010.1 | AX145218.1 | DM138436.1 | GN030359.1 | K02088.1 | GM629918.1 | HW340484.1 | DD206780.1 |
| FB573116.1 | HV755981.1 | AX145186.1 | DM143379.1 | GN030327.1 | M24953.1 | GM642404.1 | HW350851.1 | DD196870.1 |
| CS696053.1 | HV757518.1 | AX145154.1 | DM138346.1 | GN030295.1 | M13705.1 | GM642238.1 | HW350679.1 | CS119822.1 |
| CS696020.1 | HV758715.1 | AX145122.1 | DM138314.1 | GN030231.1 | M25076.1 | GM642268.1 | HW350645.1 | CS119528.1 |
| CS695988.1 | HV755093.1 | AX145058.1 | DM121667.1 | GN030199.1 | M60084.1 | GM669914.1 | HW340171.1 | CS119495.1 |
| CS695924.1 | HV535862.1 | AX145026.1 | DM137791.1 | GM704169.1 | M19979.1 | GM656335.1 | HW350253.1 | CS119462.1 |
| CS695732.1 | HV537343.1 | AX144994.1 | DM137124.1 | GM622447.1 | MZ890862.1 | GM656303.1 | HW349953.1 | CS119427.1 |
| CS695668.1 | HV535643.1 | AX144962.1 | DM136760.1 | GM622415.1 | MH238439.1 | GM656271.1 | HW347993.1 | CS119395.1 |
| DL176621.1 | HV531938.1 | AX144930.1 | DM131202.1 | GM622383.1 | AB326231.1 | GM887759.1 | HW363676.1 | CS119362.1 |
| DL176471.1 | HV516227.1 | AX144897.1 | DM130795.1 | GM631371.1 | KF664575.1 | GM005808.1 | HW363644.1 | CS119330.1 |
| DL176397.1 | HV515739.1 | AX144865.1 | DM130722.1 | GM657537.1 | AH003156.2 | GM685503.1 | HW355431.1 | CS119232.1 |
| DL175998.1 | HV515583.1 | AX144833.1 | HB436407.1 | GM657505.1 | M19102.1 | FB753776.1 | HW344517.1 | CS119168.1 |
| DL175304.1 | HV515551.1 | AX144801.1 | HB435561.1 | GM657473.1 | K01764.1 | FB728443.1 | HW307419.1 | CS119136.1 |
| DL174441.1 | HV515519.1 | AX144737.1 | HB441323.1 | GM657441.1 | DQ250238.1 | GM618718.1 | HW294142.1 | CS119069.1 |
| DL176738.1 | HV515487.1 | AX144705.1 | HB427181.1 | GM657409.1 | DQ250174.1 | GM040256.1 | HW294077.1 | CS119036.1 |
| DL163229.1 | HV515455.1 | AX144673.1 | HB426471.1 | GM657377.1 | AF440682.1 | FB709033.1 | HW294043.1 | CS119003.1 |
| FB344352.1 | HV515423.1 | AX144641.1 | HB423126.1 | GM657345.1 | AF105012.1 | GM841758.1 | DD159965.1 | CS118906.1 |
| FB343408.1 | HV515391.1 | AX144605.1 | HB423087.1 | GM650121.1 | S71742.1 | GM841722.1 | DD171108.1 | CS118872.1 |
| DL080571.1 | HV515359.1 | AX144541.1 | HB403632.1 | GM650089.1 | HW408445.1 | GM604062.1 | CS118427.1 | CS118840.1 |
| DL075914.1 | HV515295.1 | AX144477.1 | HB416466.1 | GM643120.1 | HW390748.1 | GM603715.1 | CS118394.1 | CS118741.1 |
| DL075238.1 | HV515263.1 | AX144285.1 | HB397693.1 | GM643088.1 | HW399411.1 | GM706684.1 | CS118362.1 | CS118709.1 |
| DL074110.1 | HV515231.1 | AX144155.1 | HB396574.1 | GM643056.1 | HW399321.1 | GM706652.1 | CS118293.1 | CS118677.1 |
| DL046828.1 | HV511230.1 | AX144091.1 | HB394316.1 | GM643024.1 | HW409074.1 | GM706614.1 | CS118261.1 | CS118645.1 |
| DL030706.1 | HV514047.1 | AX143769.1 | HB394284.1 | GM622215.1 | HW408995.1 | GM706387.1 | CS118229.1 | CS118613.1 |
| DL030674.1 | HV510435.1 | AX143641.1 | HB394252.1 | GM622183.1 | HW382168.1 | GM706353.1 | CS118196.1 | CS118580.1 |
| DL014204.1 | HV513360.1 | AX143577.1 | HB393709.1 | GM622151.1 | HW390518.1 | GM704815.1 | CS118098.1 | CS118545.1 |
| DL014172.1 | HV513328.1 | HW124919.1 | HB393657.1 | GM631010.1 | HW154639.1 | GM601149.1 | CS118065.1 | CS118512.1 |
| DL014140.1 | HV509442.1 | HW124810.1 | HB393391.1 | GM630978.1 | HW154472.1 | FB720256.1 | CS117995.1 | CS118479.1 |
| DL042636.1 | HV508623.1 | HW124066.1 | HB403047.1 | GM630946.1 | HW154379.1 | FB717764.1 | CS117963.1 | CS118447.1 |
| DL042572.1 | HV508591.1 | HW123526.1 | HB403014.1 | GM630914.1 | HW158135.1 | FB715075.1 | CS114655.1 | CS118415.1 |
| CS607038.1 | HV508527.1 | HW123462.1 | HB402982.1 | GM630889.1 | HW154105.1 | FB677410.1 | CS109269.1 | CS118348.1 |
| CS606948.1 | HV508501.1 | HW122806.1 | HB397676.1 | FB506785.1 | HW153788.1 | FB676992.1 | CS106399.1 | CS118085.1 |
| CS606813.1 | HV512391.1 | HW122141.1 | DM118345.1 | GM635846.1 | HW153707.1 | GM061147.1 | CS105985.1 | CS118053.1 |
| CS604972.1 | HV512269.1 | HW121446.1 | DM113494.1 | GM635814.1 | HW157493.1 | DL459981.1 | CS104774.1 | CS118018.1 |
| CS592844.1 | HV512237.1 | HW121055.1 | DM116200.1 | GM635782.1 | HW160337.1 | DL262716.1 | CS102911.1 | CS117983.1 |
| CS592086.1 | HV512205.1 | HW120188.1 | HA138175.1 | GM630882.1 | HW151825.1 | DL260062.1 | CS102847.1 | CS108623.1 |
| CS589291.1 | HV512173.1 | HW118620.1 | GM616299.1 | GM647184.1 | HW151511.1 | DL257208.1 | CS102751.1 | CS106692.1 |
| CS585208.1 | HV512141.1 | HW118285.1 | GM691469.1 | GM647152.1 | HW159797.1 | DL241774.1 | CS102687.1 | CS106387.1 |
| HI000020.1 | HV492266.1 | HW117538.1 | GM000650.1 | GM647120.1 | HW125670.1 | DL241344.1 | CS102655.1 | CS104311.1 |
| HI003486.1 | HV504949.1 | HW115652.1 | GM043445.1 | GM647088.1 | HW144449.1 | DL240956.1 | CS102623.1 | CS104200.1 |
| HI001790.1 | HV504917.1 | HW115618.1 | HB388650.1 | GM647048.1 | HW144371.1 | DL240636.1 | CS102591.1 | CS102995.1 |
| HI001753.1 | HV504885.1 | HW087590.1 | HB386666.1 | GM627514.1 | HW144339.1 | FB748883.1 | CS102559.1 | CS102963.1 |
| HI469713.1 | HV504853.1 | HW101691.1 | HB385988.1 | GM661292.1 | HW124891.1 | FB674308.1 | CS102527.1 | CS102931.1 |
| HI575291.1 | HV504821.1 | HW097016.1 | HB385779.1 | GM654102.1 | HW124795.1 | DL233413.1 | CS102495.1 | CS102835.1 |

| | | | | | | | | |
|---|---|---|---|---|---|---|---|---|
| HI465382.1 | HV507451.1 | HW105237.1 | HB342200.1 | GM654070.1 | HW124056.1 | DL232126.1 | CS101487.1 | CS102803.1 |
| HI001633.1 | HV504740.1 | HW072727.1 | HB341965.1 | GM654038.1 | HW120959.1 | DL146282.1 | CS089187.1 | CS102739.1 |
| HI001592.1 | HV504708.1 | HW105044.1 | HB340462.1 | GM654006.1 | HW120092.1 | DL124472.1 | CS082841.1 | CS102675.1 |
| HI001544.1 | HV504676.1 | HW096222.1 | HB339946.1 | GM653974.1 | HV932720.1 | DL124440.1 | CS080791.1 | CS102643.1 |
| HI001499.1 | HV504644.1 | HW086488.1 | HA642298.1 | GM646981.1 | HV932687.1 | DL124408.1 | AX465472.1 | CS102611.1 |
| HI003374.1 | HV504612.1 | HW072442.1 | HA641907.1 | GM646949.1 | HV802939.1 | DL124376.1 | AX463656.1 | CS102579.1 |
| HI003336.1 | HV492401.1 | HW071959.1 | HA641562.1 | GM646917.1 | HV802907.1 | DL120287.1 | A22726.1 | CS102547.1 |
| HI464742.1 | HV504429.1 | HV764771.1 | HA641161.1 | GM646885.1 | HV813696.1 | DL120255.1 | A10289.1 | CS102515.1 |
| HI464710.1 | HV504205.1 | HV774549.1 | GM652130.1 | GM646853.1 | HV818590.1 | DL120223.1 | AX458645.1 | CS102483.1 |
| HI464678.1 | HV455589.1 | HV774480.1 | GM652098.1 | GM646821.1 | HV818564.1 | DL115820.1 | AX458505.1 | A08089.1 |
| HI464646.1 | HV504169.1 | HV761140.1 | GM652066.1 | GM640018.1 | HV817907.1 | DL115788.1 | AX456486.1 | A07633.1 |
| HI504972.1 | HV504137.1 | HV766937.1 | GM652034.1 | GM639986.1 | HV778611.1 | AY658125.1 | AX455873.1 | A06671.1 |
| HI504439.1 | FW557010.1 | HV766777.1 | GM645175.1 | GM639954.1 | HV775754.1 | AY658093.1 | AX454135.1 | A06135.1 |
| HI462581.1 | FW556970.1 | HV766722.1 | GM645143.1 | GM639922.1 | HV770291.1 | AY658061.1 | AX453968.1 | A01414.1 |
| HH713949.1 | FW562765.1 | HV766308.1 | GM645111.1 | GM639890.1 | HV774980.1 | AY658029.1 | AX452893.1 | A00701.1 |
| HH713460.1 | FW562635.1 | HV766090.1 | GM645079.1 | GM627284.1 | HV764702.1 | AY657997.1 | AX452015.1 | M12614.1 |
| HH961365.1 | FW562512.1 | GM649116.1 | GM645047.1 | GM627252.1 | HV764566.1 | AY657965.1 | AX451568.1 | M31016.1 |
| HH961333.1 | FW566802.1 | GM649084.1 | GM645015.1 | GM627220.1 | HV774539.1 | AY657933.1 | AX449256.1 | M15218.1 |
| HH961269.1 | FW566718.1 | GM649020.1 | GM638184.1 | GM627188.1 | HV767337.1 | AY657901.1 | A34702.1 | M15315.1 |
| HH961237.1 | FW505120.1 | GM642153.1 | GM638152.1 | GM627124.1 | HV503145.1 | AY657869.1 | AX443289.1 | M11246.1 |
| HH975431.1 | FW508117.1 | GM642121.1 | GM638120.1 | GM661076.1 | HV503113.1 | AY657837.1 | A29532.1 | AF411595.1 |
| HC325500.1 | FW562494.1 | GM642089.1 | GM638088.1 | GM661044.1 | HV502781.1 | AY657805.1 | AX429420.1 | LT897787.1 |
| HC325090.1 | FW562462.1 | GM642057.1 | GM638056.1 | GM661012.1 | HV502749.1 | AY657773.1 | AX428437.1 | AH002302.2 |
| HC325028.1 | FW560951.1 | GM642025.1 | GM651900.1 | GM660980.1 | HV502717.1 | AY657741.1 | AX427943.1 | AH003160.2 |
| HC314346.1 | FW555401.1 | GM629776.1 | GM651868.1 | GM660948.1 | HV502685.1 | AY657709.1 | AX427222.1 | M12477.1 |
| HC316566.1 | FW553182.1 | GM629744.1 | GM651836.1 | GM653954.1 | HV502653.1 | AY657677.1 | AX427111.1 | M13639.1 |
| HC307882.1 | FW553095.1 | GM629712.1 | GM651804.1 | GM653922.1 | HV502621.1 | AY657645.1 | AX418551.1 | AF162281.1 |
| HC307810.1 | FW556666.1 | GM886352.1 | GM645005.1 | GM653890.1 | HV502589.1 | AY657613.1 | AX418285.1 | AF003718.1 |
| HC307389.1 | FW566095.1 | GM669152.1 | DL043753.1 | GM653858.1 | HV502557.1 | AY657581.1 | AX399828.1 | AF545504.1 |
| HC312271.1 | FW559390.1 | GM656025.1 | DL043721.1 | GM646785.1 | HV502525.1 | AY657549.1 | AX398669.1 | AH003173.2 |
| FU264220.1 | FW559342.1 | GM655993.1 | DL039872.1 | GM646753.1 | HV502493.1 | AY657517.1 | AX395274.1 | HW390737.1 |
| HC011024.1 | FW506878.1 | GM655961.1 | DL039840.1 | GM646721.1 | HV502461.1 | AY657485.1 | AX392415.1 | HW399781.1 |
| HC010668.1 | FW510488.1 | GM648968.1 | DL039808.1 | GM646689.1 | HV492785.1 | AY657453.1 | AX384820.1 | HW399357.1 |
| DM370675.1 | FW506073.1 | GM648936.1 | DL039776.1 | GM646657.1 | HV505300.1 | AY657421.1 | AX384577.1 | GN031321.1 |
| DM370643.1 | HI933994.1 | GM648904.1 | DL039744.1 | GM646625.1 | HV505196.1 | AY657389.1 | AX384033.1 | GN031288.1 |
| DM370611.1 | HI931686.1 | GM648872.1 | DL039712.1 | GM639822.1 | HV492466.1 | AY657357.1 | AX382241.1 | GN031256.1 |
| DM370579.1 | HI930643.1 | GM648840.1 | DL036088.1 | GM639790.1 | HV453605.1 | AY657325.1 | AX382142.1 | GN031224.1 |
| DM370547.1 | HI929586.1 | GM648808.1 | DL036056.1 | GM639758.1 | HV455249.1 | AY657293.1 | AX377965.1 | GN031192.1 |
| DM370515.1 | HI935103.1 | GM634657.1 | DL036024.1 | GM639726.1 | HV451947.1 | AY657261.1 | AX376954.1 | GN031159.1 |
| DM370483.1 | HI935028.1 | GM634625.1 | DL035992.1 | GM639694.1 | HV453162.1 | AY657229.1 | AX370544.1 | GN031127.1 |
| DM205870.1 | HI949790.1 | GM634593.1 | DL035960.1 | GM639662.1 | HV452873.1 | AY657197.1 | AX364561.1 | GN031095.1 |
| DM205256.1 | HI929205.1 | GM634561.1 | DL035928.1 | GM646585.1 | HV449167.1 | AY657133.1 | AX364529.1 | GN031063.1 |
| DM209283.1 | HI918316.1 | GM634529.1 | DL035896.1 | GM646553.1 | HV448875.1 | AY657101.1 | AX364497.1 | GN031031.1 |
| HC003063.1 | HI918266.1 | GM634497.1 | DL031892.1 | GM646521.1 | HV444460.1 | AY657069.1 | AX364465.1 | GN030999.1 |
| HB975558.1 | HI661317.1 | GM629692.1 | DL031860.1 | GM646489.1 | HV437885.1 | AY657037.1 | AX364433.1 | GN030967.1 |
| HB865033.1 | HI657788.1 | GM664878.1 | DL031828.1 | GM646457.1 | HV437538.1 | U05286.1 | AX364401.1 | GN030936.1 |
| HB864971.1 | HC918555.1 | GM664700.1 | DL031796.1 | GM646425.1 | HV349425.1 | M62767.1 | AX364369.1 | GN030904.1 |

| | | | | | | | | |
|---|---|---|---|---|---|---|---|---|
| HB864993.1 | FW334600.1 | GM648774.1 | DL031764.1 | GM639526.1 | HV341637.1 | M61009.1 | AX364337.1 | GN030872.1 |
| HB864937.1 | FW342470.1 | GM648744.1 | DL027859.1 | GM626883.1 | HV341233.1 | J02534.1 | AX364210.1 | GN030840.1 |
| HB864905.1 | FW337391.1 | GM648712.1 | DL027795.1 | GM626851.1 | HV341043.1 | M18042.1 | AX363231.1 | GN030808.1 |
| HB864873.1 | FW336067.1 | GM648680.1 | DL027763.1 | DL100598.1 | DJ339817.1 | M12395.1 | AX359002.1 | CS721919.1 |
| HB864841.1 | HC868203.1 | GM648648.1 | DL023951.1 | DL100566.1 | DJ339753.1 | M20256.1 | AX358638.1 | DJ348048.1 |
| HB864809.1 | HC887872.1 | GM648616.1 | DL023919.1 | DL100534.1 | DJ339721.1 | HV549280.1 | E50932.1 | DJ357731.1 |
| HB864777.1 | HC887347.1 | GM634464.1 | DL023887.1 | DL119988.1 | DJ339657.1 | HV549082.1 | E63236.1 | DJ344586.1 |
| HB864745.1 | HC874430.1 | GM634432.1 | DL023855.1 | DL119956.1 | DJ339592.1 | HV543495.1 | E64775.1 | DJ354950.1 |
| HB866528.1 | DL048090.1 | GM634400.1 | DL023823.1 | DL119924.1 | DJ339560.1 | HV547075.1 | E54541.1 | DJ361565.1 |
| HB977475.1 | DL048058.1 | GM634368.1 | DL023791.1 | DL119892.1 | DJ334567.1 | HV539603.1 | HW155721.1 | DJ361223.1 |
| HB976830.1 | DL048027.1 | GM629477.1 | DL020776.1 | DL119860.1 | DJ334038.1 | HV538860.1 | HW158936.1 | DJ339870.1 |
| HB976626.1 | DL047995.1 | GM629435.1 | DL020744.1 | DL119828.1 | DJ333536.1 | HV538578.1 | HW154985.1 | DJ339838.1 |
| DM193669.1 | DL047963.1 | GM629403.1 | DL020712.1 | DL115201.1 | DJ327043.1 | HV538452.1 | HW158480.1 | DJ339806.1 |
| HB839963.1 | DL047931.1 | DL199852.1 | DL020680.1 | DL115169.1 | DJ327011.1 | HV543370.1 | HW154728.1 | DJ339742.1 |
| HB848022.1 | DL047899.1 | DL199818.1 | DL020648.1 | DL115137.1 | DJ128110.1 | HV542716.1 | HW154640.1 | DJ326902.1 |
| HB839495.1 | DL047867.1 | DL201919.1 | DL020616.1 | DL115105.1 | DJ086518.1 | HV541491.1 | HW154473.1 | DJ127224.1 |
| HB838857.1 | DL040068.1 | DL184327.1 | DL016213.1 | DL115073.1 | DJ086078.1 | HV541034.1 | HW147475.1 | DJ083948.1 |
| HB805435.1 | DL040036.1 | DL198985.1 | DL016181.1 | DL110073.1 | DJ081037.1 | HV514905.1 | HW154303.1 | DJ082656.1 |
| GM640140.1 | DL040004.1 | DL196195.1 | DL016149.1 | DL110041.1 | DJ080753.1 | HV532753.1 | HW158145.1 | DJ071627.1 |
| FB983224.1 | DL039972.1 | DL193795.1 | DL016117.1 | DL110009.1 | DJ069486.1 | HV536510.1 | HW154106.1 | DJ066761.1 |
| FB983172.1 | DL039940.1 | DL193763.1 | DL016085.1 | DL109977.1 | DJ071396.1 | HV534612.1 | HW153755.1 | DJ066398.1 |
| GM681763.1 | DL039908.1 | DL183398.1 | DL016053.1 | DL109763.1 | DD335102.1 | FW420397.1 | HW153708.1 | DJ066365.1 |
| FB753858.1 | DL071366.1 | DL183345.1 | DL011595.1 | DL109731.1 | DD348424.1 | FW503054.1 | HW157494.1 | DJ066333.1 |
| GM751819.1 | DL047823.1 | FB513372.1 | DL011531.1 | DL109699.1 | DD347415.1 | FW496473.1 | HW151826.1 | DJ066299.1 |
| FB727454.1 | DL047791.1 | FB512595.1 | DL011499.1 | DL104965.1 | DD332622.1 | FW496061.1 | HW151512.1 | DJ066265.1 |
| GM618808.1 | DL047759.1 | FB571244.1 | DL011467.1 | DL104933.1 | CS425084.1 | FW504213.1 | DL095374.1 | DJ066211.1 |
| GM842522.1 | DL047727.1 | FB570307.1 | DL011403.1 | DL100487.1 | CS424384.1 | FW503816.1 | DL112253.1 | DJ065247.1 |
| FB725701.1 | DL047695.1 | CS803547.1 | DL031713.1 | DL100391.1 | CS423367.1 | FW499076.1 | DL112221.1 | DJ055698.1 |
| GM840669.1 | DL047663.1 | CS696196.1 | DL031681.1 | DL100327.1 | CS417761.1 | FW504729.1 | DL112189.1 | BD299625.1 |
| CS728629.1 | DL043681.1 | CS696164.1 | DL031649.1 | DL124140.1 | CS421333.1 | FW497089.1 | DL112157.1 | BD295271.1 |
| CS728533.1 | DL039704.1 | CS696132.1 | DL031617.1 | DL124108.1 | CS419011.1 | FW420440.1 | DL112125.1 | BD294882.1 |
| CS727341.1 | DL035920.1 | CS696068.1 | DL031585.1 | DL119790.1 | CS417382.1 | FW420408.1 | DL112093.1 | BD289989.1 |
| CS727258.1 | DL031916.1 | CS696036.1 | DL031553.1 | DL119758.1 | CS412177.1 | FW501741.1 | DL107090.1 | BD277320.1 |
| FB715322.1 | DL031884.1 | CS696003.1 | DL027744.1 | DL119726.1 | CS415529.1 | FW496925.1 | DL107058.1 | BD274293.1 |
| GM949524.1 | DL031852.1 | CS695971.1 | DL027712.1 | DL123859.1 | CS410808.1 | FW420383.1 | DL106994.1 | BD274177.1 |
| GM009436.1 | DL031820.1 | CS695907.1 | DL027680.1 | DL119488.1 | CS414831.1 | FW420351.1 | DL106962.1 | BD272753.1 |
| GM887807.1 | DL031788.1 | CS695843.1 | DL027648.1 | DL119456.1 | DD327021.1 | FW420319.1 | DL106930.1 | BD271650.1 |
| GM773637.1 | DL031756.1 | CS695811.1 | DL027616.1 | DL119424.1 | DD326888.1 | FW496697.1 | DL102187.1 | DD231078.1 |
| GM879303.1 | DL027851.1 | CS695587.1 | DJ361183.1 | DL123671.1 | DD329894.1 | HI653874.1 | DL102157.1 | DD227541.1 |
| GM842700.1 | DL027819.1 | CS695555.1 | DJ354113.1 | DL123607.1 | DD328971.1 | HI653804.1 | DL102125.1 | DD227400.1 |
| FB722584.1 | DL027787.1 | CS695491.1 | DJ339863.1 | DL123575.1 | DD321795.1 | HI653632.1 | DL111924.1 | DD226385.1 |
| DL011780.1 | DL027755.1 | CS695395.1 | DJ339799.1 | DL119385.1 | CS389307.1 | HI653598.1 | DL106857.1 | DD224939.1 |
| DL040370.1 | DL023943.1 | CS695297.1 | DJ339767.1 | GM633339.1 | CS389272.1 | HI653526.1 | DL106825.1 | DD224390.1 |
| DL030703.1 | DL023911.1 | DL176447.1 | DJ339735.1 | GM633307.1 | CS389219.1 | HI653475.1 | DL106793.1 | DD224111.1 |
| DL026670.1 | DL023847.1 | DL176284.1 | DJ339703.1 | GM633275.1 | CS389182.1 | HI653413.1 | DL091267.1 | DD233512.1 |
| DD457035.1 | DL023815.1 | DL164121.1 | DJ339671.1 | GM633252.1 | CS401599.1 | HI653360.1 | DL091235.1 | DD231424.1 |
| DD456099.1 | DL023783.1 | DL176771.1 | DJ339606.1 | GM633220.1 | CS406185.1 | HI653275.1 | DL091203.1 | DD214002.1 |

| | | | | | | | | |
|---|---|---|---|---|---|---|---|---|
| HW112858.1 | DJ044953.1 | DL176672.1 | DJ339574.1 | GM633188.1 | CS406502.1 | HI653212.1 | DL091171.1 | DD218555.1 |
| HW112709.1 | DJ044931.1 | FB316383.1 | DJ339542.1 | GM633156.1 | CS403739.1 | HI651952.1 | DL087235.1 | DD213569.1 |
| HW112628.1 | DJ044890.1 | FB343468.1 | DJ339510.1 | GM633092.1 | CS402446.1 | HI650461.1 | DL087203.1 | DD213537.1 |
| HW112563.1 | DJ030495.1 | DL094180.1 | DJ335798.1 | GM659129.1 | CS401818.1 | HI648664.1 | DL102096.1 | DD220612.1 |
| HW112472.1 | DJ028138.1 | DL090433.1 | DJ333900.1 | GM644785.1 | CS382086.1 | HI646885.1 | DL102064.1 | CQ784691.1 |
| HW112287.1 | DJ030392.1 | DL090401.1 | DJ330227.1 | GM644753.1 | DD308886.1 | HI646165.1 | DL102032.1 | CQ784625.1 |
| HW112197.1 | DJ029998.1 | DL090369.1 | DJ330130.1 | GM644721.1 | BD319435.1 | HH807304.1 | DL101883.1 | CQ778396.1 |
| HV780251.1 | DJ029928.1 | DL086300.1 | DJ330097.1 | GM633059.1 | BD318906.1 | HH806870.1 | DL101819.1 | CQ757564.1 |
| HV780161.1 | DJ026327.1 | DL086268.1 | DJ329298.1 | GM625224.1 | BD318127.1 | HH802864.1 | DL101787.1 | CQ755357.1 |
| HV779812.1 | DJ026280.1 | DL086236.1 | DJ328856.1 | GM625192.1 | BD306493.1 | HH794719.1 | DL101755.1 | AX958948.1 |
| HV779595.1 | CS691407.1 | DL086204.1 | DJ328784.1 | GM625160.1 | BD300353.1 | FW394270.1 | DL101723.1 | AX951553.1 |
| HV778906.1 | CS693900.1 | DL110287.1 | DJ327057.1 | GM624038.1 | BD292599.1 | FW398173.1 | DL097213.1 | AX934533.1 |
| HV778706.1 | CS674197.1 | DL105354.1 | DJ327025.1 | GM624006.1 | BD291542.1 | FW394251.1 | DL114795.1 | AX923422.1 |
| HV778344.1 | CS680818.1 | DL105322.1 | DJ326993.1 | GM623974.1 | BD291309.1 | FW394209.1 | DL114763.1 | AX923389.1 |
| HV777609.1 | CS691867.1 | DL124341.1 | DJ326660.1 | GM623942.1 | BD299636.1 | FW394080.1 | DL114731.1 | AX840832.1 |
| HV777427.1 | DJ012330.1 | DL124309.1 | DJ122010.1 | GM625116.1 | BD298461.1 | FW398090.1 | DL114699.1 | AX840743.1 |
| HV777077.1 | DJ008403.1 | DL110170.1 | DJ088997.1 | GM629914.1 | BD295083.1 | FW397868.1 | DL114667.1 | AX824353.1 |
| HV776006.1 | DJ008371.1 | DL110138.1 | DJ088503.1 | GM656331.1 | BD280248.1 | GM624727.1 | DL127340.1 | AX823787.1 |
| HV770267.1 | DJ004234.1 | DL110106.1 | DJ087186.1 | GM656299.1 | BD274306.1 | GM624695.1 | DL100324.1 | AX814861.1 |
| HV765020.1 | DJ003536.1 | DL105173.1 | DJ086459.1 | GM656267.1 | BD274188.1 | GM624631.1 | DL123469.1 | BD094344.1 |
| HV775005.1 | CS671259.1 | DL105141.1 | DJ082648.1 | GM656235.1 | BD274100.1 | GM633645.1 | DL123437.1 | BD087147.1 |
| HV774962.1 | CS669948.1 | DL105109.1 | DJ082076.1 | GM656203.1 | BD273660.1 | GM633613.1 | DL123405.1 | BD081729.1 |
| HV770211.1 | CS646650.1 | DL104902.1 | DJ088282.1 | GM656171.1 | BD271171.1 | GM633581.1 | DL123373.1 | BD081537.1 |
| HV764780.1 | CS646197.1 | DL104870.1 | DJ080984.1 | GM649146.1 | DD231203.1 | GM633549.1 | DL119119.1 | BD080156.1 |
| HV764748.1 | CS644248.1 | DL104838.1 | DJ080311.1 | GM649114.1 | DD227755.1 | GM633517.1 | DL119087.1 | BD079994.1 |
| HV764716.1 | DD460151.1 | DL104806.1 | DJ079784.1 | GM649082.1 | DD227411.1 | GM633485.1 | DL119055.1 | BD074965.1 |
| HV769886.1 | DD455652.1 | DL095883.1 | DJ069354.1 | GM642151.1 | DD224651.1 | GM638394.1 | DL119023.1 | BD073925.1 |
| HV774645.1 | DD455214.1 | DL095851.1 | DJ066390.1 | GM642119.1 | DD224162.1 | GM638362.1 | DL114627.1 | BD070739.1 |
| HV774558.1 | DD455156.1 | DL095819.1 | DJ066358.1 | GM642087.1 | DD231559.1 | GM659899.1 | DL114595.1 | BD064745.1 |
| HV774494.1 | DD454723.1 | DL095787.1 | DJ066326.1 | GM642055.1 | DD231467.1 | GM659867.1 | DL114531.1 | BD057301.1 |
| HV764423.1 | DD458362.1 | DL094132.1 | DJ066292.1 | GM642023.1 | DD231435.1 | GM659803.1 | DL114499.1 | BD016713.1 |
| HV774128.1 | DD453823.1 | DL094100.1 | DJ066258.1 | GM719711.1 | DD231403.1 | GM659771.1 | DL114467.1 | E50489.1 |
| HV763947.1 | DD457762.1 | DL114434.1 | DJ065986.1 | GM719211.1 | CS260194.1 | GM659739.1 | DL104900.1 | BD014212.1 |
| HV763601.1 | DD456886.1 | DL114402.1 | DJ062772.1 | GM669150.1 | DD214013.1 | GM659703.1 | DL104868.1 | BD014172.1 |
| HV763372.1 | CS631722.1 | DL114370.1 | HV301752.1 | GM656023.1 | DD213996.1 | GM659671.1 | DL104836.1 | E66927.1 |
| HV763277.1 | CS362657.1 | DL114338.1 | HV301618.1 | GM655991.1 | DD213871.1 | GM659639.1 | DL104804.1 | A18211.1 |
| HV762873.1 | CS360737.1 | DL104579.1 | HV306189.1 | GM655959.1 | DD213839.1 | GM659607.1 | DL095881.1 | AX458601.1 |
| HV762448.1 | CS359742.1 | DL104547.1 | HV307807.1 | GM648966.1 | DD213807.1 | GM652350.1 | DL114432.1 | AX457066.1 |
| HV762416.1 | CS359606.1 | DL104515.1 | HV302951.1 | GM648934.1 | DD216477.1 | GM652318.1 | DL114400.1 | AX454041.1 |
| HV760623.1 | BD269698.1 | DL093931.1 | HV302745.1 | GM648902.1 | DD216440.1 | GM652286.1 | DL114368.1 | AX339195.1 |
| HV766554.1 | BD269143.1 | DL093899.1 | HV312748.1 | GM648870.1 | DD213790.1 | GM645391.1 | DL114336.1 | AX329471.1 |
| HV766099.1 | BD268931.1 | DL093867.1 | HV308871.1 | GM648838.1 | AX825002.1 | GM645359.1 | DL109343.1 | AX328296.1 |
| HV766051.1 | BD268164.1 | DL073097.1 | HV304145.1 | GM648806.1 | AX824432.1 | GM645327.1 | DL104577.1 | AX328138.1 |
| HV766019.1 | BD265614.1 | DL013508.1 | HV312315.1 | GM634655.1 | AX824332.1 | GM645295.1 | DL104545.1 | AX259244.1 |
| HV765209.1 | BD264570.1 | DL013476.1 | HV312252.1 | GM634623.1 | AX823824.1 | GM645263.1 | DL104513.1 | HW317124.1 |
| HV765139.1 | BD263421.1 | DL013444.1 | HV311030.1 | GM634591.1 | AX823764.1 | GM645231.1 | DL093929.1 | HW326329.1 |
| HV765097.1 | BD261824.1 | DL013412.1 | HV318269.1 | GM634559.1 | AX817781.1 | GM659377.1 | DL093897.1 | HW338906.1 |

| | | | | | | | | |
|---|---|---|---|---|---|---|---|---|
| HV750376.1 | BD251456.1 | DL013224.1 | HV317243.1 | GM634527.1 | AX816084.1 | GM652153.1 | DL121886.1 | HW338266.1 |
| HV750344.1 | BD251233.1 | DL013192.1 | HI415921.1 | GM634495.1 | AX802889.1 | GM645198.1 | DL121854.1 | HW338010.1 |
| HV755696.1 | BD247529.1 | DL017981.1 | HI415889.1 | GM629690.1 | AX809413.1 | GM645166.1 | DL121822.1 | HW337882.1 |
| HV752879.1 | BD247110.1 | DL017949.1 | HI415857.1 | GM664862.1 | AX805927.1 | GM645134.1 | DL121790.1 | HW337754.1 |
| HV757404.1 | BD246978.1 | DL017917.1 | HI415824.1 | GM664696.1 | AX799568.1 | GM645102.1 | DL112752.1 | HW337498.1 |
| HV755470.1 | BD245263.1 | DL017885.1 | HI415792.1 | GM648742.1 | AX795414.1 | GM645070.1 | DL098314.1 | HW337114.1 |
| HV755438.1 | BD243954.1 | DL017853.1 | HI415760.1 | GM648710.1 | BD192895.1 | GM645038.1 | DL098282.1 | HW321827.1 |
| HV752627.1 | BD242702.1 | DL017821.1 | HI414047.1 | GM648678.1 | BD188832.1 | GM638143.1 | DL098250.1 | HW336858.1 |
| HV504717.1 | BD242458.1 | DL017790.1 | HI414015.1 | GM648646.1 | BD187686.1 | GM638111.1 | DL098218.1 | HW321402.1 |
| HV504685.1 | BD241793.1 | DL017758.1 | HI413773.1 | GM648614.1 | GM827696.1 | GM638079.1 | DL098186.1 | HW320954.1 |
| HV504653.1 | BD240783.1 | DL017694.1 | HI412383.1 | GM634462.1 | GM710113.1 | GM654966.1 | DL098154.1 | HW329270.1 |
| HV504621.1 | BD238478.1 | DL017662.1 | HI410968.1 | GM634430.1 | GM709005.1 | GM640913.1 | CS368002.1 | HW336133.1 |
| HV491626.1 | BD237850.1 | DL022268.1 | HI410848.1 | GM634398.1 | GM650850.1 | GM640881.1 | CS367682.1 | HW328631.1 |
| HV504972.1 | BD235977.1 | DL022236.1 | HI503517.1 | GM634366.1 | GM655002.1 | GM640849.1 | EU124676.1 | HW328418.1 |
| HV504566.1 | DL100599.1 | DL022204.1 | HI516544.1 | DL088086.1 | GM755624.1 | GM628495.1 | DL075894.1 | HW328305.1 |
| HV504534.1 | DL100567.1 | DL029733.1 | HI516090.1 | DL088054.1 | GM649584.1 | GM662108.1 | DL032426.1 | HW335670.1 |
| HV504502.1 | DL100535.1 | DL034058.1 | HI541039.1 | DL088022.1 | GM656527.1 | GM654826.1 | DL016407.1 | HW328252.1 |
| HV504470.1 | DL119989.1 | DL025351.1 | HI547698.1 | DL087990.1 | GM656495.1 | GM654794.1 | DL016375.1 | HW318550.1 |
| HV504438.1 | DL119957.1 | DL025319.1 | HI284309.1 | DL091931.1 | GM656463.1 | GM654762.1 | DL016343.1 | HW328082.1 |
| HV504406.1 | DL119925.1 | DL025287.1 | HI284277.1 | DL091867.1 | GM656431.1 | GM647769.1 | DL016311.1 | HW335164.1 |
| HV504374.1 | DL119893.1 | DL025223.1 | HI571713.1 | DL102892.1 | GM649334.1 | GM647737.1 | DL016247.1 | HW314335.1 |
| HV504342.1 | DL119861.1 | DL021883.1 | HI574530.1 | DL102860.1 | GM649278.1 | GM647705.1 | DL011731.1 | HW314117.1 |
| HV504310.1 | DL119829.1 | DL021851.1 | HI574498.1 | DL102828.1 | GM649246.1 | GM640806.1 | DL011699.1 | HW313631.1 |
| HV504278.1 | DL115234.1 | DL021819.1 | HI574466.1 | DL102764.1 | GM649214.1 | GM628291.1 | DL011667.1 | HW312143.1 |
| HV504246.1 | DL115202.1 | DL021787.1 | HI574434.1 | DL102732.1 | GM635070.1 | GM627967.1 | DL011635.1 | HW308348.1 |
| HV504214.1 | DL115170.1 | DL017421.1 | HI574402.1 | DL107576.1 | GM635035.1 | GM627935.1 | DL011603.1 | HW307849.1 |
| HV455598.1 | DL115138.1 | DL038457.1 | HI571464.1 | DL102583.1 | GM635003.1 | GM661777.1 | DL048458.1 | HW307817.1 |
| HV504178.1 | DL115106.1 | DL038425.1 | HI578559.1 | DL102551.1 | GM634971.1 | GM661745.1 | DL044745.1 | HW307785.1 |
| HV504146.1 | DL115074.1 | DL038393.1 | HI573735.1 | DL102519.1 | GM634939.1 | GM654751.1 | DL044713.1 | HW307747.1 |
| HV504114.1 | DL115042.1 | DL038361.1 | HI593856.1 | DL098041.1 | GM634907.1 | GM654719.1 | DL044681.1 | HW307715.1 |
| HV504082.1 | DL110042.1 | DL013166.1 | HI588389.1 | DL098009.1 | GM634875.1 | GM654687.1 | DL036601.1 | HW307683.1 |
| HV504050.1 | DL110010.1 | DL023142.1 | HI563567.1 | DL097977.1 | GM629973.1 | GM654655.1 | DD164826.1 | HW315841.1 |
| HV504018.1 | DL109978.1 | DL023110.1 | HI636977.1 | DL097945.1 | GM629941.1 | GM654623.1 | CS203954.1 | HW315707.1 |
| HV503986.1 | DL109764.1 | DL023078.1 | HI001402.1 | DL112635.1 | GM629909.1 | GM654591.1 | CS054740.1 | HW315575.1 |
| HV503954.1 | DL109732.1 | DL023046.1 | HI001360.1 | DL112603.1 | GM669847.1 | GM647597.1 | CS052378.1 | HW315542.1 |
| HV503922.1 | DL109700.1 | DL023014.1 | HI001326.1 | DL112571.1 | GM656326.1 | GM647565.1 | CS052344.1 | HV552737.1 |
| HV503890.1 | DL104966.1 | DL010722.1 | HI003209.1 | DL095731.1 | GM656294.1 | GM627894.1 | CS052310.1 | HV550381.1 |
| HV503858.1 | DL104934.1 | DL010690.1 | HI003090.1 | DL095699.1 | GM656262.1 | GM627862.1 | CS048771.1 | AY659385.1 |
| HV503826.1 | DL100392.1 | DL010658.1 | HI003051.1 | DL095667.1 | GM656230.1 | GM627830.1 | CS038930.1 | AY659353.1 |
| HV491236.1 | DL100360.1 | DL010626.1 | HI003010.1 | DL095603.1 | GM656198.1 | GM627798.1 | CS029885.1 | AY659321.1 |
| HV491140.1 | DL100328.1 | DL042940.1 | HI002948.1 | DL095571.1 | GM656166.1 | DL088088.1 | CS026999.1 | AY659289.1 |
| HV503786.1 | DL124141.1 | DL042908.1 | HI002911.1 | DL117408.1 | GM649173.1 | DL088056.1 | CS025486.1 | AY659257.1 |
| HV503754.1 | DL124109.1 | DL042876.1 | HI001276.1 | DL117376.1 | GM642178.1 | DL088024.1 | CS018791.1 | AY659225.1 |
| HV503722.1 | DL046103.1 | DL038963.1 | HI001174.1 | DL091635.1 | GM642146.1 | DL087992.1 | CS018326.1 | AY659193.1 |
| HV503690.1 | DL041957.1 | DL038931.1 | HI001102.1 | DL091603.1 | GM642114.1 | DL091933.1 | CS016576.1 | AY659161.1 |
| HV503626.1 | DL041925.1 | DL031079.1 | HI001084.1 | DL087960.1 | GM642082.1 | DL091869.1 | CS008632.1 | AY659129.1 |
| HV503598.1 | DL041893.1 | DL030954.1 | HI001027.1 | DL087928.1 | GM642050.1 | DL102894.1 | CQ990428.1 | AY659097.1 |

| | | | | | | | | |
|---|---|---|---|---|---|---|---|---|
| HV503566.1 | DL049710.1 | DL027110.1 | HI000994.1 | DL121759.1 | DL098004.1 | DL102862.1 | CQ986626.1 | AY659065.1 |
| HV503534.1 | DL049678.1 | DL027046.1 | HI071007.1 | GN031012.1 | DL097972.1 | DL102766.1 | CQ986592.1 | AY659033.1 |
| HV503502.1 | DL049646.1 | DL027014.1 | HI002850.1 | GN030980.1 | DL097940.1 | DL102734.1 | CQ985789.1 | AY659001.1 |
| HV503470.1 | DL045670.1 | DL035098.1 | HI002818.1 | GN030949.1 | DL112630.1 | DL107578.1 | CQ983180.1 | AY658969.1 |
| HV503406.1 | DL041759.1 | DL030909.1 | HI002762.1 | GN030917.1 | DL112598.1 | DL107486.1 | CQ983146.1 | AY658937.1 |
| HV503374.1 | DL041695.1 | DL030870.1 | HI002718.1 | GN030853.1 | DL112566.1 | DL107454.1 | CQ983097.1 | AY658905.1 |
| DL040976.1 | DL049637.1 | DL030838.1 | HH821177.1 | GN030789.1 | DL095726.1 | DL107390.1 | CQ982999.1 | AY658873.1 |
| DL040912.1 | DL049605.1 | DL030806.1 | HH821118.1 | DM001674.1 | DL095694.1 | DL102585.1 | AX722177.1 | AY658841.1 |
| DL040880.1 | DL041558.1 | DL030774.1 | HH828417.1 | DM001468.1 | DL095662.1 | DL102553.1 | BD177342.1 | AY658809.1 |
| DL037057.1 | DL041526.1 | DL030742.1 | HH827404.1 | DM006944.1 | DL095630.1 | DL102521.1 | AX720170.1 | AY658777.1 |
| DL037025.1 | DL037671.1 | DL026773.1 | HH827372.1 | DM006689.1 | DL095598.1 | DL098011.1 | AX719169.1 | AY658745.1 |
| DL036993.1 | DL037639.1 | DL019754.1 | HH826848.1 | DM000515.1 | DL095566.1 | DL097979.1 | AX699456.1 | AY658713.1 |
| DL036961.1 | DL037607.1 | DL019722.1 | HH826501.1 | DM004998.1 | DL117403.1 | DL097947.1 | AX699424.1 | AY658681.1 |
| DL036929.1 | DL037575.1 | DL015223.1 | HH822366.1 | DM003495.1 | DL117371.1 | DL112637.1 | AX685568.1 | AY658649.1 |
| DL036897.1 | DL037543.1 | DL015191.1 | HH822046.1 | GM983071.1 | DL125474.1 | DL112605.1 | AX684480.1 | AY658617.1 |
| DL032694.1 | DL033308.1 | DL015159.1 | FW379716.1 | GM994299.1 | DL125442.1 | DL112573.1 | AX683880.1 | AY658585.1 |
| DL032662.1 | DL033276.1 | DL015031.1 | FW379684.1 | GM992938.1 | DL125410.1 | DL095733.1 | AX662216.1 | AY658553.1 |
| DL032630.1 | DL033244.1 | DD456356.1 | FW379651.1 | GM992318.1 | DL125378.1 | DL095701.1 | AX659100.1 | AY658521.1 |
| DL032598.1 | DL033212.1 | CS631720.1 | FW374116.1 | GM992095.1 | DL125346.1 | DL095669.1 | AX658680.1 | AY658489.1 |
| DL032566.1 | DL033180.1 | CS642265.1 | FW378154.1 | GM991957.1 | DL102490.1 | DL095637.1 | AX657138.1 | AY658457.1 |
| DL032534.1 | DL033148.1 | CS631289.1 | FW377386.1 | GM989782.1 | DL102458.1 | DL095605.1 | AX657106.1 | AY658425.1 |
| DL028526.1 | DL029140.1 | CS631210.1 | FW377113.1 | GM986644.1 | DL102394.1 | DL095573.1 | BD176073.1 | AY658393.1 |
| DL028494.1 | DL029108.1 | CS631146.1 | FW376189.1 | GM969696.1 | DL104573.1 | DL117410.1 | AX644853.1 | AY658361.1 |
| DL028462.1 | DL029076.1 | CS627845.1 | FW381545.1 | GM969575.1 | DL104541.1 | CQ813996.1 | AX643794.1 | AY658329.1 |
| DL028430.1 | DL024974.1 | CS632289.1 | FW381353.1 | GM980875.1 | DL104509.1 | CQ813964.1 | AX643579.1 | AY658297.1 |
| DL028398.1 | DL021730.1 | CS623707.1 | FW375535.1 | GN007489.1 | DL141054.1 | CQ813932.1 | AX642154.1 | AY658265.1 |
| DL028366.1 | DL021698.1 | CS623653.1 | FW369980.1 | GM984665.1 | DL123341.1 | CQ813900.1 | BD171982.1 | AY658201.1 |
| DL024552.1 | DL021666.1 | CS623589.1 | FW375250.1 | FB986335.1 | DL123309.1 | CQ813868.1 | AX601798.1 | AY658169.1 |
| DL024488.1 | DL021634.1 | GM634030.1 | FW374987.1 | GM631632.1 | DL089969.1 | CQ813836.1 | AX601614.1 | AY658137.1 |
| DL024456.1 | DL017396.1 | GM633998.1 | FW369656.1 | GM631600.1 | DL109151.1 | CQ813804.1 | AX601317.1 | AY658105.1 |
| DL021180.1 | DL029841.1 | GM633934.1 | FW380267.1 | GM704227.1 | DL109119.1 | CQ813731.1 | AX594173.1 | AY658073.1 |
| DL021148.1 | DL029777.1 | GM633902.1 | FW369341.1 | GM622445.1 | DL109087.1 | CQ812796.1 | AX593515.1 | AY658041.1 |
| DL021116.1 | DL029745.1 | GM648358.1 | FW379848.1 | GM657407.1 | DL104353.1 | CQ806527.1 | AX587925.1 | AY658009.1 |
| DL021084.1 | DL026354.1 | GM648326.1 | FW369332.1 | GM657375.1 | DL104321.1 | CQ802130.1 | AX587825.1 | AY657977.1 |
| DL021052.1 | DL018282.1 | GM648294.1 | U17140.1 | GM650119.1 | DL123158.1 | CQ802026.1 | AX306434.1 | AY657945.1 |
| DL021020.1 | DL018250.1 | GM648262.1 | HH736049.1 | GM650087.1 | DL123126.1 | CQ800993.1 | AX262440.1 | AY657913.1 |
| DL016814.1 | DL018218.1 | GM648230.1 | HH735628.1 | GM643118.1 | DL118680.1 | CQ800792.1 | AX258841.1 | AY657881.1 |
| DL016782.1 | DL013816.1 | GM648198.1 | HH755061.1 | GM643086.1 | DL118648.1 | CQ796851.1 | AX300951.1 | AY657849.1 |
| DL016718.1 | DL013784.1 | GM641395.1 | HH734144.1 | GM643022.1 | DL114092.1 | CQ795485.1 | AX286670.1 | AY657817.1 |
| DL016686.1 | DL013752.1 | GM641363.1 | HH733638.1 | GM622213.1 | DL069377.1 | CQ795453.1 | AX282314.1 | AY657785.1 |
| DL016654.1 | DL013720.1 | GM641331.1 | DL038732.1 | GM622181.1 | DL049703.1 | CQ793234.1 | AX281529.1 | AY657753.1 |
| DL016622.1 | DL013688.1 | GM641299.1 | DL038700.1 | GM622149.1 | DL049671.1 | CQ792317.1 | AX278805.1 | AY657721.1 |
| DL012164.1 | DL000625.1 | GM641267.1 | DL035076.1 | FB506783.1 | DL045663.1 | CQ790259.1 | AX278057.1 | AY657689.1 |
| DL012132.1 | DL005818.1 | GM641235.1 | DL035044.1 | GM654823.1 | DL049630.1 | CQ788160.1 | AX256074.1 | AY657657.1 |
| DL012100.1 | DL005437.1 | GM629076.1 | DL035012.1 | GM654791.1 | DL033301.1 | CQ787484.1 | AX253476.1 | AY657625.1 |
| DL012068.1 | DJ493969.1 | GM629044.1 | DL034980.1 | GM654759.1 | DL033269.1 | CQ787452.1 | AX247550.1 | DJ353063.1 |
| DL012036.1 | DL008502.1 | GM629012.1 | DL034948.1 | GM628288.1 | DL033237.1 | CQ787412.1 | AX242322.1 | DJ340966.1 |

| | | | | | | | | |
|---|---|---|---|---|---|---|---|---|
| DL012004.1 | DJ491557.1 | GM881787.1 | DL034916.1 | GM046377.1 | DL033205.1 | CQ787372.1 | AX242258.1 | DJ361219.1 |
| DL016596.1 | DL002522.1 | GM648165.1 | DL034893.1 | GM627932.1 | DL033173.1 | CQ787338.1 | AX242226.1 | DJ339866.1 |
| DL016564.1 | DJ446856.1 | DL200017.1 | DL034861.1 | GM654748.1 | DL033141.1 | CQ787304.1 | AX242194.1 | DJ339802.1 |
| DL016532.1 | DJ446823.1 | DL199985.1 | DL034837.1 | GM654716.1 | DL029133.1 | CQ786936.1 | AX242162.1 | EU020107.1 |
| DL016500.1 | DJ438152.1 | DL195044.1 | DL034797.1 | GM654652.1 | DL029101.1 | CQ784723.1 | AX242130.1 | DJ088336.1 |
| DL016468.1 | DJ433004.1 | DL199752.1 | DL034765.1 | GM647594.1 | DL029069.1 | CQ784659.1 | AX241970.1 | DJ082706.1 |
| DL011914.1 | DJ437352.1 | DL194841.1 | DL034733.1 | GM647562.1 | DL021755.1 | CQ772700.1 | AX241906.1 | DJ080508.1 |
| DL011882.1 | DJ437318.1 | DL202061.1 | DL034701.1 | GM627891.1 | DL021723.1 | CQ771656.1 | AX241874.1 | DJ066361.1 |
| DL011850.1 | DJ437230.1 | DL201579.1 | DL030537.1 | GM627859.1 | DL017389.1 | CQ771624.1 | AX241842.1 | DJ066329.1 |
| DL011818.1 | DJ437173.1 | DL198983.1 | DL026728.1 | GM627827.1 | DL017357.1 | CQ771592.1 | AX241778.1 | DD347391.1 |
| DL024357.1 | DJ437143.1 | DL191616.1 | DL026696.1 | GM627795.1 | DL012936.1 | CQ768815.1 | AX241746.1 | CS423578.1 |
| DL024325.1 | DJ437058.1 | DL196193.1 | DL026664.1 | GM661713.1 | DL041312.1 | CQ766920.1 | AX241714.1 | CS419066.1 |
| DL024293.1 | DJ436984.1 | DL193793.1 | DL026632.1 | GM661681.1 | DL041280.1 | CQ761281.1 | AX241586.1 | CS417163.1 |
| DL024261.1 | DJ436746.1 | DL193761.1 | DL026600.1 | GM661649.1 | DL009310.1 | CQ756622.1 | AX241554.1 | CS416268.1 |
| DL024229.1 | DJ436658.1 | DL184035.1 | DL026568.1 | GM661617.1 | DL009246.1 | CQ754038.1 | AX241458.1 | CS410874.1 |
| DL024197.1 | DJ436504.1 | DL183396.1 | DL022756.1 | GM661585.1 | DL104829.1 | AY220727.1 | AX241426.1 | DD327530.1 |
| DL045057.1 | DJ445587.1 | DL183343.1 | DL022724.1 | GM661553.1 | DL104733.1 | A11897.1 | AX241119.1 | DD329750.1 |
| DL045025.1 | DJ444603.1 | FB513370.1 | DL022692.1 | GM661521.1 | DL095938.1 | A08641.1 | AX235763.1 | DD328078.1 |
| DL044993.1 | DJ444564.1 | FB570919.1 | DL022660.1 | GM647342.1 | DL095906.1 | A06388.1 | AX223897.1 | CS398365.1 |
| DJ380885.1 | DJ434803.1 | CS696162.1 | DL022628.1 | GM647278.1 | DL095874.1 | A04257.1 | AX212312.1 | CS389291.1 |
| DJ380853.1 | DD451211.1 | CS696130.1 | DL022596.1 | GM647246.1 | DL114425.1 | A32070.1 | AX207262.1 | CS389249.1 |
| DJ380833.1 | CS619874.1 | CS696098.1 | DL019581.1 | GM647214.1 | DL114393.1 | A32037.1 | AX205118.1 | CS389202.1 |
| DJ389571.1 | CS617804.1 | CS696034.1 | DL019517.1 | GM870231.1 | DL114361.1 | A30531.1 | AX203106.1 | CS389165.1 |
| DJ388705.1 | CS616855.1 | CS696001.1 | DL019485.1 | GM627614.1 | DL104570.1 | A28879.1 | AX202528.1 | CS401298.1 |
| DJ388663.1 | CS614466.1 | CS695969.1 | DL019453.1 | GM627548.1 | DL104538.1 | A28040.1 | AX194355.1 | CS406460.1 |
| DJ388526.1 | CS613791.1 | CS695905.1 | DL019421.1 | GM661489.1 | DL123338.1 | A26239.1 | AX188573.1 | CS408112.1 |
| DJ388480.1 | CS613388.1 | CS695841.1 | DL014986.1 | GM661457.1 | DL123306.1 | A24786.1 | AX180727.1 | CS402128.1 |
| CS725930.1 | CS611839.1 | CS695809.1 | DL014954.1 | GM661425.1 | DL033298.1 | M23297.1 | AX180269.1 | DD291556.1 |
| CS724992.1 | CS604543.1 | CS695777.1 | DL014922.1 | GM661393.1 | DL033266.1 | A23905.1 | HW307766.1 | DD291028.1 |
| CS724510.1 | CS604511.1 | CS695745.1 | DL014890.1 | GM661361.1 | DL029130.1 | A22087.1 | HW307760.1 | BD300469.1 |
| CS724426.1 | CS604479.1 | CS695617.1 | DL010400.1 | DL097181.1 | DL029098.1 | A21721.1 | HW307728.1 | BD294539.1 |
| CS723753.1 | CS604447.1 | CS695393.1 | DL010336.1 | DL097149.1 | DL029066.1 | A21070.1 | HW307696.1 | BD293157.1 |
| CS721841.1 | CS604415.1 | DL176500.1 | DL010304.1 | DL095225.1 | DL028970.1 | A20271.1 | HW315685.1 | BD295267.1 |
| DJ353061.1 | CS604383.1 | DL176445.1 | DL010272.1 | DL095193.1 | DL021752.1 | A19409.1 | HW315595.1 | BD285367.1 |
| DJ352130.1 | CS604351.1 | DL176355.1 | DL010208.1 | DL095161.1 | DL023657.1 | A21285.1 | HW315556.1 | BD287848.1 |
| DJ357789.1 | CS604319.1 | DL176281.1 | DL034673.1 | DL111835.1 | DL023625.1 | A19396.1 | HW315448.1 | BD277303.1 |
| DJ357757.1 | CS604127.1 | DL176766.1 | DL034641.1 | DL111803.1 | DL023593.1 | A17463.1 | HW315360.1 | BD273455.1 |
| DJ357725.1 | CS603775.1 | DL176669.1 | CS603816.1 | DL111771.1 | DL015919.1 | A16021.1 | DM179444.1 | BD272417.1 |
| DJ357691.1 | CS603743.1 | FB357112.1 | CS603752.1 | DL128886.1 | DL015887.1 | A13639.1 | DM170844.1 | BD271913.1 |
| DJ357627.1 | CS603455.1 | DL086985.1 | CS603464.1 | DL116418.1 | DL015855.1 | A12646.1 | GM637409.1 | DD227527.1 |
| DJ357595.1 | CS603423.1 | DL086953.1 | CS603432.1 | DL116386.1 | DL047629.1 | A12337.1 | GM637377.1 | DD227396.1 |
| DJ340794.1 | CS603391.1 | DL086921.1 | CS603400.1 | DL116354.1 | DL047597.1 | A11910.1 | GM637345.1 | DD227154.1 |
| DJ340962.1 | CS603359.1 | DL086889.1 | CS603368.1 | DL111633.1 | DL047565.1 | A11144.1 | GM637313.1 | DD224931.1 |
| DJ344501.1 | CS603327.1 | DL086857.1 | CS603336.1 | DL111601.1 | DL047533.1 | A06519.1 | GM637281.1 | DD223916.1 |
| DJ355123.1 | CS603295.1 | DL086825.1 | CS603304.1 | DL111537.1 | DL039670.1 | A02229.1 | GM637104.1 | DD231420.1 |
| DJ354943.1 | DD158366.1 | DL086793.1 | CS603272.1 | DL101691.1 | DL039638.1 | A09018.1 | GM637072.1 | A33360.1 |
| DJ361217.1 | DD153642.1 | DL086761.1 | CS603240.1 | DL101659.1 | DL039606.1 | A08179.1 | GM632670.1 | DD213998.1 |

| | | | | | | | | |
|---|---|---|---|---|---|---|---|---|
| DJ361184.1 | DD152588.1 | DL086729.1 | CS603208.1 | DL101627.1 | DL039313.1 | A07246.1 | GM632638.1 | DD213981.1 |
| DJ354116.1 | DD151918.1 | DL086697.1 | CS603176.1 | DL097117.1 | DL023552.1 | A06420.1 | GM741928.1 | AX709607.1 |
| DJ339864.1 | DD096868.1 | DL086665.1 | CS603144.1 | DL097053.1 | DL023520.1 | A05044.1 | GM658448.1 | AX683726.1 |
| DJ339768.1 | DD082124.1 | DL105874.1 | CS603112.1 | DL095129.1 | DL023488.1 | A09813.1 | GM644209.1 | AX670731.1 |
| DJ339736.1 | DD081752.1 | DL105842.1 | CS603080.1 | DL095097.1 | DL023456.1 | A01786.1 | GM636923.1 | AX665513.1 |
| DJ339704.1 | DD149125.1 | DL105810.1 | CS603048.1 | DL095033.1 | CS626248.1 | A01729.1 | GM636891.1 | AX664355.1 |
| DJ339672.1 | DD147787.1 | DL096527.1 | CS603016.1 | DL095001.1 | DD449078.1 | A05194.1 | GM636859.1 | AX662260.1 |
| DJ339607.1 | DD147755.1 | DL096495.1 | CS602982.1 | DD405120.1 | DD437344.1 | A04190.1 | GM670026.1 | AX657123.1 |
| DJ339575.1 | DD147723.1 | DL096463.1 | CS602950.1 | DD408550.1 | CS620287.1 | A01344.1 | GM656525.1 | AX657088.1 |
| DJ339543.1 | DD147691.1 | DL110740.1 | CS602918.1 | DD406797.1 | CS616588.1 | K01476.1 | GM656493.1 | BD174689.1 |
| DJ339093.1 | DD147099.1 | DL110676.1 | CS602886.1 | DD410053.1 | CS614357.1 | M18435.1 | GM656461.1 | BD174406.1 |
| DJ336902.1 | DD080836.1 | DL090674.1 | CS602854.1 | DD402399.1 | CS613340.1 | M10912.1 | GM656429.1 | AX642236.1 |
| DJ335799.1 | DD138537.1 | DL115547.1 | CS602822.1 | DD402367.1 | CS611726.1 | M13106.1 | GM649332.1 | AX642055.1 |
| DJ334687.1 | DD137496.1 | DL115515.1 | CS602790.1 | DD402335.1 | CS604502.1 | M55579.1 | GM649276.1 | AX608828.1 |
| DJ333903.1 | DD132351.1 | DL115483.1 | CS602726.1 | DD402303.1 | CS603638.1 | M60107.1 | GM649244.1 | AX601599.1 |
| DJ330131.1 | DD132330.1 | DL115451.1 | CS602694.1 | DD402271.1 | CS603478.1 | M19081.1 | GM649212.1 | AX601463.1 |
| DJ330098.1 | DD094901.1 | DL105699.1 | CS611208.1 | DD402239.1 | CS603446.1 | LC732343.1 | GM635132.1 | AX601367.1 |
| DJ328857.1 | DD106658.1 | DL105667.1 | CS610141.1 | DD402207.1 | CS603414.1 | MH087226.1 | GM635100.1 | AX601335.1 |
| DJ328825.1 | DD119298.1 | DL105635.1 | CS608388.1 | DD405587.1 | CS603382.1 | M23435.1 | GM635068.1 | AX599953.1 |
| DJ328785.1 | DD118641.1 | DL105603.1 | CS607927.1 | DD405555.1 | CS603350.1 | KX021742.1 | GM634969.1 | AX594148.1 |
| DJ327784.1 | DD061320.1 | DL100798.1 | CS607840.1 | DD405523.1 | CS603286.1 | AH002047.2 | GM656324.1 | AX593489.1 |
| DJ326663.1 | DD091020.1 | DL100766.1 | CS607538.1 | DD405491.1 | CS603254.1 | M22147.1 | GM656292.1 | AX014759.1 |
| DJ121781.1 | DD118101.1 | DL100734.1 | CS600278.1 | DD405459.1 | CS603222.1 | AY569327.1 | GM656260.1 | AX011024.1 |
| DJ122011.1 | DD116171.1 | DL096320.1 | CS607094.1 | DD405427.1 | CS603190.1 | AF463529.1 | GM656228.1 | AX010919.1 |
| DJ089841.1 | DD090304.1 | DL096288.1 | CS607013.1 | DD402021.1 | CS603158.1 | AF105015.1 | GM656196.1 | AX009741.1 |
| DJ087197.1 | DD103253.1 | DL096256.1 | CS606925.1 | DD401989.1 | CS603126.1 | HW071666.1 | GM656164.1 | AX008546.1 |
| DJ082704.1 | DD057939.1 | DL096224.1 | CS605118.1 | DD401928.1 | CS603062.1 | HW104338.1 | GM649171.1 | AX004812.1 |
| DJ088294.1 | DD057907.1 | DL096192.1 | CS605086.1 | DD401864.1 | CS603030.1 | HW104304.1 | GM653154.1 | AX002978.1 |
| DJ081602.1 | DD057875.1 | DL096160.1 | CS605054.1 | DD401832.1 | CS602998.1 | HW070983.1 | GM653122.1 | A41484.1 |
| DJ080991.1 | DD050544.1 | DL120180.1 | CS605027.1 | DD401800.1 | CS602964.1 | E35596.1 | GM653090.1 | A24436.1 |
| DJ079786.1 | DD057222.1 | DL120148.1 | CS604995.1 | DD401624.1 | CS602932.1 | E41211.1 | GM653058.1 | A35719.1 |
| BD222017.1 | DD054758.1 | DL120116.1 | CS604931.1 | DD401592.1 | CS602708.1 | E36138.1 | GM639000.1 | A32097.1 |
| BD218297.1 | CQ787337.1 | DL120084.1 | CS604899.1 | DD401560.1 | DD122482.1 | E06830.1 | GM652834.1 | A29451.1 |
| BD218008.1 | CQ787303.1 | DL120052.1 | CS604835.1 | DD401528.1 | DD138526.1 | E05282.1 | GM638812.1 | A35503.1 |
| BD211616.1 | CQ787271.1 | HV585642.1 | CS407611.1 | DD401496.1 | DD136024.1 | E04461.1 | GM638780.1 | A34242.1 |
| BD205762.1 | CQ787239.1 | HV571047.1 | CS380542.1 | DD401464.1 | DD132321.1 | E03650.1 | GM626105.1 | A32655.1 |
| BD195433.1 | CQ786935.1 | HV570485.1 | CS382738.1 | CS479880.1 | DD106550.1 | E03187.1 | FB766173.1 | A32413.1 |
| DD283609.1 | CQ784721.1 | HV569213.1 | CS376598.1 | DD057220.1 | DD092935.1 | E02861.1 | FB764687.1 | A30349.1 |
| DD283506.1 | CQ784657.1 | HV567935.1 | CS376331.1 | DD053301.1 | DD091011.1 | E02774.1 | FB743888.1 | A31025.1 |
| DD282972.1 | CQ784469.1 | HV573317.1 | DD309002.1 | DD052285.1 | DD117035.1 | E02327.1 | FB743856.1 | A28461.1 |
| DD282294.1 | CQ772689.1 | HV560301.1 | DD298473.1 | DD052227.1 | DD089300.1 | E02181.1 | FB743802.1 | A21441.1 |
| DD281826.1 | CQ771655.1 | HV559947.1 | DD308756.1 | DD051417.1 | DD102593.1 | E01765.1 | FB761823.1 | A21468.1 |
| DD281084.1 | CQ771623.1 | HV559388.1 | DD291756.1 | DD055025.1 | DD057930.1 | E01675.1 | GM036244.1 | A19567.1 |
| DD287638.1 | CQ771591.1 | HV555945.1 | DD291024.1 | DD058544.1 | DD057898.1 | E01548.1 | DL102608.1 | A17136.1 |
| DD279484.1 | CQ768809.1 | HV302071.1 | CS377171.1 | DD041906.1 | DD057866.1 | E01427.1 | DL098002.1 | A15466.1 |
| DD279031.1 | CQ768280.1 | HV038274.1 | CS376991.1 | DD048078.1 | DD057838.1 | E01345.1 | DL097970.1 | A12574.1 |
| DD278875.1 | CQ759504.1 | HV030221.1 | DL115918.1 | DD042876.1 | DD057054.1 | E01019.1 | DL097938.1 | A11034.1 |

| | | | | | | | | |
|---|---|---|---|---|---|---|---|---|
| CS356013.1 | CQ756621.1 | HV037791.1 | DL115886.1 | DD030007.1 | DD053294.1 | E00839.1 | DL095724.1 | A07739.1 |
| CS355627.1 | CQ754680.1 | HV036350.1 | DL115854.1 | DD039028.1 | DD041899.1 | E00371.1 | DL095692.1 | A31397.1 |
| CS349760.1 | CQ754037.1 | HV035752.1 | DL109835.1 | DD037268.1 | DD046369.1 | E00309.1 | DL095660.1 | A30599.1 |
| CS352453.1 | AX960214.1 | FZ435995.1 | DL109803.1 | DD030669.1 | DD038703.1 | DD102375.1 | DL095628.1 | A28928.1 |
| CS351544.1 | AX962033.1 | FZ427621.1 | DL109771.1 | DD026704.1 | DD034263.1 | DD101256.1 | DL095596.1 | GM621603.1 |
| CS283944.1 | AX960411.1 | FZ435780.1 | DL109739.1 | DD025721.1 | DD032564.1 | DD084755.1 | DL095564.1 | GM657119.1 |
| CS283010.1 | AX960221.1 | FZ430206.1 | DL109707.1 | DD023787.1 | CS085661.1 | DD070084.1 | DL117401.1 | GM657087.1 |
| CS277376.1 | AX959235.1 | FZ429942.1 | DL105101.1 | DD028856.1 | CS084255.1 | DD084676.1 | DL117369.1 | GM657055.1 |
| CS276837.1 | AX958115.1 | FZ429753.1 | DL105005.1 | DD028679.1 | CS079781.1 | CS052380.1 | DL125472.1 | GM657023.1 |
| CS274964.1 | AX958012.1 | FZ423908.1 | DL104973.1 | DD019518.1 | CS071948.1 | CS052346.1 | DL125440.1 | GM656991.1 |
| CS273095.1 | AX925674.1 | FZ436949.1 | DL104941.1 | DD017279.1 | AY967405.1 | CS052313.1 | DL125408.1 | GM656959.1 |
| CS272530.1 | AX923405.1 | FZ432326.1 | DL104909.1 | BD412645.1 | AY967373.1 | CS050978.1 | DL125376.1 | GM656928.1 |
| CS265235.1 | AX923371.1 | FZ416170.1 | DL100495.1 | BD495441.1 | AY967341.1 | CS048773.1 | DL110161.1 | GM656864.1 |
| CS265568.1 | AX840309.1 | FZ421419.1 | DL100431.1 | BD454398.1 | AY967309.1 | CS047649.1 | DL110129.1 | GM656832.1 |
| CS260202.1 | AX824446.1 | FZ417032.1 | DL100399.1 | BD453895.1 | AY967277.1 | CS038932.1 | DL110097.1 | GM656800.1 |
| CS257327.1 | AX824336.1 | FZ419851.1 | DL100367.1 | BD453863.1 | AY967245.1 | AY134742.1 | DL105164.1 | GM656768.1 |
| CS255246.1 | AX823863.1 | FZ419818.1 | DL100335.1 | BD453831.1 | AY967213.1 | CS029889.1 | DL105132.1 | GM649775.1 |
| CS253594.1 | AX818056.1 | FZ419786.1 | DL124148.1 | BD453799.1 | AY967181.1 | CS021530.1 | DL119948.1 | GM649743.1 |
| CS253402.1 | AX151112.1 | HH998664.1 | DL124116.1 | BD443678.1 | AY967149.1 | CS018793.1 | DL119916.1 | GM649711.1 |
| CS254323.1 | AX149406.1 | HH998596.1 | DL124084.1 | BD443410.1 | AY967117.1 | CS018330.1 | DL119884.1 | GM649679.1 |
| CS254291.1 | AX147675.1 | HH998541.1 | DL124052.1 | BD453743.1 | AY967085.1 | CS016578.1 | DL119852.1 | GM649647.1 |
| CS253950.1 | AX146316.1 | HH996920.1 | DL123988.1 | BD453711.1 | CQ877623.1 | CS016540.1 | DL119820.1 | GM649615.1 |
| CS254105.1 | AX145728.1 | HH996815.1 | DL119798.1 | BD453679.1 | CQ875504.1 | CS008911.1 | DL115129.1 | GM642807.1 |
| CS254073.1 | AX145696.1 | HH996764.1 | DL119766.1 | BD453647.1 | CQ874861.1 | CQ990430.1 | DL110065.1 | GM642775.1 |
| CS253989.1 | AX145632.1 | HH994555.1 | DL119734.1 | BD453615.1 | CQ873518.1 | CQ987003.1 | DL110001.1 | GM642743.1 |
| CS250655.1 | AX145600.1 | HH980762.1 | DL119702.1 | BD453578.1 | CQ871403.1 | CQ986594.1 | DL109969.1 | GM642711.1 |
| CS248967.1 | DM091309.1 | HH977622.1 | DL119670.1 | BD453565.1 | CQ869281.1 | CQ983148.1 | DL109723.1 | GM642679.1 |
| CS248043.1 | GN087791.1 | HH980635.1 | DL119638.1 | BD453533.1 | CQ868891.1 | CQ983001.1 | DL104957.1 | GM642647.1 |
| CS245136.1 | GN090934.1 | HH980494.1 | DL119606.1 | BD453501.1 | CQ867381.1 | CQ982969.1 | DL104925.1 | GM642615.1 |
| CS244173.1 | GN090007.1 | HH980353.1 | DL115011.1 | BD453469.1 | CQ859631.1 | CQ982818.1 | DL124132.1 | GM635433.1 |
| CS244253.1 | GN089852.1 | HH980293.1 | DL114979.1 | BD453437.1 | CQ857858.1 | CQ982673.1 | DL124100.1 | GM635401.1 |
| CS244221.1 | GN093655.1 | HH980218.1 | DL114947.1 | BD453405.1 | CQ855856.1 | CQ981121.1 | DL024918.1 | GM635369.1 |
| CS244189.1 | GN082889.1 | HH980153.1 | DL114915.1 | BD453260.1 | CQ849445.1 | BD161921.1 | DL017148.1 | GM635337.1 |
| CS243074.1 | GN082818.1 | HH980101.1 | DL114883.1 | BD453228.1 | CQ829253.1 | BD161069.1 | DL017116.1 | GM635305.1 |
| CS239631.1 | GN088790.1 | HH979876.1 | DL114851.1 | BD453196.1 | CQ821003.1 | BD143480.1 | DL028656.1 | GM635273.1 |
| CS236042.1 | GN088035.1 | HH996701.1 | DL127355.1 | BD411238.1 | CQ816997.1 | AX587803.1 | DL028624.1 | GM630467.1 |
| CS230336.1 | GN078175.1 | HH996639.1 | DL123931.1 | BD442627.1 | CQ816954.1 | AX587697.1 | DL028592.1 | GM630435.1 |
| CS228650.1 | L08862.1 | HH996584.1 | DL123899.1 | BD497499.1 | CQ816917.1 | AX587596.1 | DL028560.1 | GM630403.1 |
| CS227878.1 | M74304.1 | HH996543.1 | DL123867.1 | BD451721.1 | CQ814047.1 | AX577546.1 | DL042964.1 | GM630371.1 |
| CS196186.1 | DM059616.1 | HH998398.1 | DL123835.1 | BD130830.1 | CQ814015.1 | AX573870.1 | DL042932.1 | GM630339.1 |
| CS200888.1 | DM058834.1 | HH998361.1 | DL123803.1 | BD130798.1 | CQ813983.1 | AX573549.1 | DL042900.1 | GM630307.1 |
| CS227291.1 | GM659128.1 | HH999992.1 | DL123771.1 | BD129650.1 | CQ813951.1 | AX556853.1 | DL030901.1 | GM621500.1 |
| CS226737.1 | GM644784.1 | HH999936.1 | DL119592.1 | BD105718.1 | CQ813919.1 | AX556819.1 | DL030734.1 | FB505482.1 |
| CS208973.1 | GM644752.1 | HH993944.1 | DL119560.1 | BD105405.1 | CQ813887.1 | AX555188.1 | HC046714.1 | GM653045.1 |
| CS208936.1 | GM644720.1 | HH979815.1 | DL119528.1 | BD081628.1 | CQ813855.1 | AX538882.1 | HC039631.1 | GM653013.1 |
| DD173984.1 | GM644624.1 | HH979653.1 | DL119496.1 | BD080676.1 | CQ813823.1 | AX538625.1 | HC046062.1 | GM652981.1 |
| DD167926.1 | GM633058.1 | HH999862.1 | DL119464.1 | BD080327.1 | CQ813791.1 | AX538293.1 | HC046030.1 | GM645982.1 |

| | | | | | | | | |
|---|---|---|---|---|---|---|---|---|
| DD173112.1 | GM625223.1 | HH999819.1 | DL119432.1 | BD074979.1 | CQ813758.1 | AX536403.1 | DM170842.1 | GM645950.1 |
| DD178499.1 | GM625191.1 | HH999765.1 | DL123743.1 | BD074943.1 | AX053157.1 | AX534750.1 | DM170810.1 | GM645918.1 |
| DD166516.1 | GM625159.1 | HH999708.1 | DL123711.1 | BD070061.1 | AX052938.1 | AX528976.1 | DM187956.1 | GM645886.1 |
| DD166311.1 | GM624037.1 | HH998274.1 | DL123679.1 | BD063666.1 | AX042374.1 | AX528517.1 | DM187859.1 | GM645854.1 |
| DD165452.1 | GM624005.1 | HH998238.1 | DL123647.1 | BD016726.1 | AX040755.1 | AX528099.1 | DM177493.1 | GM645822.1 |
| DD165156.1 | GM623973.1 | HH998190.1 | DL123615.1 | BD015726.1 | AX037313.1 | AX524850.1 | HB491552.1 | GM639019.1 |
| DD165109.1 | GM623941.1 | HH998149.1 | DL123583.1 | BD014628.1 | AX036549.1 | AX523912.1 | HB489352.1 | GM638955.1 |
| DD165042.1 | GM625115.1 | HH993700.1 | DL119393.1 | BD014225.1 | AX028792.1 | AX523630.1 | DM163996.1 | GM638923.1 |
| DD165010.1 | GM625083.1 | HH986631.1 | DL119361.1 | BD014193.1 | AX027609.1 | A30515.1 | DM163963.1 | GM638891.1 |
| DD159572.1 | GM625051.1 | HH979524.1 | DL119329.1 | E54319.1 | AX025094.1 | A33337.1 | DM163580.1 | GM638859.1 |
| DD159189.1 | GM625019.1 | HH975848.1 | DL119297.1 | AX474406.1 | AX023603.1 | AX179433.1 | DM163294.1 | GM626308.1 |
| DD182190.1 | GM624987.1 | HH979628.1 | DL119265.1 | AX471970.1 | AX021518.1 | AX179479.1 | HB488206.1 | GM626244.1 |
| DD170587.1 | GM624955.1 | HH979586.1 | DL119233.1 | E51068.1 | AX019313.1 | AX179410.1 | GN368287.1 | GM626212.1 |
| DD170427.1 | GM623833.1 | HH932379.1 | DL114837.1 | AX468940.1 | AX018719.1 | AX025156.1 | GN360146.1 | GM626180.1 |
| DD175957.1 | GM658916.1 | HH932001.1 | DL114805.1 | AX468388.1 | AX010926.1 | A28416.1 | GN360041.1 | GM626148.1 |
| DD175925.1 | GM658884.1 | AX100679.1 | DL114773.1 | AX465467.1 | AX008698.1 | AX521535.1 | GN360009.1 | GM660315.1 |
| DD175900.1 | GM658852.1 | FW394406.1 | DL114741.1 | AX463655.1 | AX006719.1 | AX521503.1 | GN359977.1 | GM660283.1 |
| DD170110.1 | GM658820.1 | FW394241.1 | DL114709.1 | A22475.1 | AX003645.1 | AF430189.1 | GN359868.1 | GM660251.1 |
| DD053312.1 | GM644605.1 | FW394204.1 | DL114677.1 | AX461659.1 | AX003018.1 | AF430157.1 | GN359804.1 | GM660219.1 |
| DD052466.1 | GM637415.1 | FW393979.1 | DL114645.1 | AX458642.1 | AJ286129.1 | AX505267.1 | GN359708.1 | GM660187.1 |
| DD052238.1 | GM637383.1 | FW397857.1 | DL127154.1 | AX458492.1 | A44215.1 | AX505203.1 | GN359592.1 | GM660155.1 |
| DD052183.1 | GM637351.1 | HH936312.1 | DL141558.1 | AX456485.1 | A25433.1 | AX505139.1 | GN359560.1 | GM652949.1 |
| DD051825.1 | GM637319.1 | HH935095.1 | DL123543.1 | AX455842.1 | A35726.1 | AX497053.1 | GN359496.1 | GM652917.1 |
| DD058670.1 | GM637287.1 | HC307792.1 | DL123479.1 | AX454132.1 | A35301.1 | BD140004.1 | GN365532.1 | GM652885.1 |
| DD042731.1 | GM637255.1 | FU262992.1 | DL123447.1 | AX453964.1 | A29458.1 | BD138023.1 | GN359428.1 | GM652853.1 |
| DD042403.1 | GM623654.1 | FU259870.1 | DL123415.1 | AX451393.1 | A27484.1 | BD136055.1 | GN359332.1 | GM652821.1 |
| DD048089.1 | GM623590.1 | FU265337.1 | DL123383.1 | AX441377.1 | A34517.1 | BD135542.1 | GN365391.1 | GM652788.1 |
| DD047269.1 | GM623558.1 | FU264503.1 | DL119193.1 | AX443286.1 | A33656.1 | BD133978.1 | GN359259.1 | GM645790.1 |
| DD044313.1 | GM658707.1 | HC313118.1 | DL119161.1 | AX427720.1 | A32804.1 | BD131938.1 | GN359227.1 | GM645758.1 |
| DD039055.1 | GM658675.1 | HC313050.1 | DL119129.1 | AX427221.1 | A30362.1 | BD130904.1 | GN359195.1 | GM645726.1 |
| DD038493.1 | GM884097.1 | DM467460.1 | DL119097.1 | AX427107.1 | A13697.1 | HW390411.1 | GN359163.1 | GM645694.1 |
| DD038324.1 | GM648357.1 | HC306164.1 | DL119065.1 | AX418550.1 | A31313.1 | HW389666.1 | GN359131.1 | GM645662.1 |
| DD037081.1 | GM648325.1 | HC306124.1 | DL119033.1 | AX417135.1 | A25920.1 | HW389563.1 | GN343918.1 | GM645630.1 |
| DD031742.1 | GM648293.1 | HC306044.1 | DL114637.1 | HW315558.1 | A12739.1 | HW388672.1 | GN348287.1 | GM638831.1 |
| DD031277.1 | GM648261.1 | HC306004.1 | DL114605.1 | HW315450.1 | A17932.1 | HW388629.1 | GN346571.1 | GM638799.1 |
| DD027437.1 | GM648229.1 | HC302646.1 | DL114573.1 | HW315165.1 | A15719.1 | HI378574.1 | GN346539.1 | GM638767.1 |
| DD026921.1 | GM648197.1 | HC301626.1 | DL114541.1 | HW315069.1 | A11181.1 | HI378094.1 | GN335200.1 | GM638735.1 |
| DD025764.1 | GM641362.1 | HC294302.1 | DL114509.1 | HW314829.1 | A06518.1 | HI376929.1 | GN131878.1 | GM638703.1 |
| DD024706.1 | GM641298.1 | HC294026.1 | DL114477.1 | HW314719.1 | A00363.1 | HI376616.1 | GN116518.1 | GM638671.1 |
| DD029720.1 | GM641266.1 | HC289392.1 | DL114445.1 | HW115424.1 | A29288.1 | HI375919.1 | GM637407.1 | GM626124.1 |
| DD010339.1 | GM641234.1 | HW049316.1 | DL093525.1 | HW112760.1 | A29006.1 | HH735911.1 | GM637375.1 | GM626092.1 |
| DD010165.1 | GM629075.1 | HW049284.1 | DL093493.1 | HW112603.1 | HW155434.1 | HH735611.1 | GM637343.1 | GM626060.1 |
| DD019580.1 | GM629043.1 | HW049252.1 | DL093461.1 | HW069949.1 | HW155300.1 | HH755037.1 | GM637311.1 | GM626028.1 |
| DD017842.1 | GM629011.1 | HW043254.1 | DL093429.1 | HW069877.1 | HW158437.1 | HH733940.1 | GM637279.1 | GM625996.1 |
| DD017422.1 | GM881785.1 | HW041791.1 | DL093365.1 | HW084941.1 | HW153899.1 | HH733846.1 | GM637134.1 | GM625964.1 |
| DD014224.1 | GM648164.1 | HW040789.1 | DL089750.1 | HW089631.1 | HW153741.1 | FW368808.1 | GM637102.1 | GM625932.1 |
| DD009545.1 | GM619118.1 | HW043074.1 | DL089718.1 | HW097735.1 | HW152025.1 | FW368177.1 | GM637070.1 | GM037797.1 |

| | | | | | | | | |
|---|---|---|---|---|---|---|---|---|
| BD495452.1 | GM618940.1 | HW041351.1 | DL089686.1 | HW097693.1 | HW151062.1 | FW363722.1 | GM644207.1 | FB775671.1 |
| BD454043.1 | GM662144.1 | HW054035.1 | DL089654.1 | HW058278.1 | HW159787.1 | FW363522.1 | GM636921.1 | FB774963.1 |
| BD434152.1 | GM640910.1 | HW053986.1 | DL089622.1 | HW047239.1 | HW144714.1 | FW351384.1 | GM636889.1 | FB766243.1 |
| BD453907.1 | GM640878.1 | HW053874.1 | DL089590.1 | HV969573.1 | HW126449.1 | FW351232.1 | GM636857.1 | FB765670.1 |
| BD453874.1 | GM640846.1 | HW040940.1 | DL126110.1 | HV956401.1 | HW125389.1 | FW360406.1 | GM622840.1 | FB764681.1 |
| BD453842.1 | GM628492.1 | HV985963.1 | AX242238.1 | HV951106.1 | HW144439.1 | HD082420.1 | GM741185.1 | FB747076.1 |
| BD453810.1 | GM662105.1 | HV985931.1 | AX242206.1 | HV950000.1 | HW144361.1 | HD112018.1 | GM643110.1 | FB744720.1 |
| BD453776.1 | FB712629.1 | HV985899.1 | AX242110.1 | HV944623.1 | HW144329.1 | HD088243.1 | GM643078.1 | FB744007.1 |
| BD411893.1 | FB707955.1 | HV040124.1 | AX242078.1 | HV779878.1 | HW124861.1 | HD080094.1 | GM643046.1 | FB743940.1 |
| BD453754.1 | FB667093.1 | HV097473.1 | AX242046.1 | HV774718.1 | HV774732.1 | HD080411.1 | GM643014.1 | FB743907.1 |
| BD453722.1 | FB666658.1 | HV200504.1 | AX242014.1 | HV773781.1 | HV774099.1 | HD080149.1 | GM640902.1 | DJ417445.1 |
| BD453690.1 | CS721884.1 | HV182485.1 | AX241918.1 | HV568925.1 | HV767477.1 | HC456315.1 | GM640870.1 | DJ402734.1 |
| BD453658.1 | FB668133.1 | HV182421.1 | AX241886.1 | HW302684.1 | HV761252.1 | HC453740.1 | GM640838.1 | DJ387765.1 |
| BD453626.1 | FB676566.1 | HV182389.1 | AX241822.1 | HW302535.1 | HV767007.1 | HC453676.1 | GM047094.1 | DJ387544.1 |
| BD453594.1 | FB676534.1 | HV220989.1 | AX241790.1 | HV955895.1 | HV760593.1 | HC453644.1 | GM654815.1 | DJ386642.1 |
| BD453544.1 | FB676502.1 | HV232680.1 | AX241758.1 | HV957930.1 | HV752933.1 | AF084397.1 | GM654783.1 | DJ381013.1 |
| BD453512.1 | FB675467.1 | HV038679.1 | AX241726.1 | HV949618.1 | HV758700.1 | HC452099.1 | GM647694.1 | DJ380981.1 |
| BD453480.1 | FB701551.1 | HV038591.1 | AX241694.1 | HV947319.1 | HV755014.1 | HC451825.1 | GM628152.1 | DJ380949.1 |
| BD453448.1 | CS809028.1 | HV182351.1 | AX241662.1 | HV947219.1 | HV745348.1 | FU774064.1 | GM627956.1 | DJ380917.1 |
| BD453416.1 | FB660486.1 | HV182319.1 | AX241630.1 | HV953032.1 | HV117706.1 | FU773362.1 | GM627924.1 | CS597526.1 |
| BD453377.1 | FB660045.1 | HV182287.1 | AX241598.1 | HV940254.1 | HV038666.1 | FU759997.1 | GM715063.1 | CS593094.1 |
| BD453239.1 | FB580692.1 | HV190958.1 | AX241566.1 | HV939886.1 | HV038582.1 | FU759940.1 | GM015127.1 | CS592960.1 |
| BD453207.1 | FB665214.1 | HV190829.1 | AX241502.1 | HV942493.1 | HV202355.1 | FU758129.1 | GM963781.1 | CS592821.1 |
| CQ802140.1 | FB654417.1 | HV228458.1 | AX241470.1 | HV815879.1 | HV219824.1 | FU757632.1 | GM889746.1 | CS592383.1 |
| CQ802039.1 | FB583260.1 | HV037790.1 | AX241438.1 | HV929150.1 | HV029940.1 | FU757600.1 | GM009359.1 | CS592339.1 |
| CQ801232.1 | FB573886.1 | HV035742.1 | AX241099.1 | HV931350.1 | HV037779.1 | HC441565.1 | GM008837.1 | CS589284.1 |
| CQ798735.1 | FB579763.1 | FZ435962.1 | AX241067.1 | HV936810.1 | HV035594.1 | HC358586.1 | FB985017.1 | CS593373.1 |
| CQ797566.1 | DL200016.1 | FZ427620.1 | AX241035.1 | HV930998.1 | GM656255.1 | HC358218.1 | FB983179.1 | CS585119.1 |
| CQ795497.1 | DL199984.1 | FZ435779.1 | AX241003.1 | HV936386.1 | GM656223.1 | HC358088.1 | GM866016.1 | CS583918.1 |
| CQ795461.1 | DL199847.1 | FZ430203.1 | AX240971.1 | HV936354.1 | GM656191.1 | HC358055.1 | FB726888.1 | CS582466.1 |
| CQ794591.1 | DL199719.1 | FZ429921.1 | AX240939.1 | HV930753.1 | GM656159.1 | HC324496.1 | GM864676.1 | CS588635.1 |
| CQ792418.1 | DL194829.1 | FZ423907.1 | AX239707.1 | HV819646.1 | GM719323.1 | HC319210.1 | GM863545.1 | CS581994.1 |
| CQ789694.1 | CQ871411.1 | HI413438.1 | AX236455.1 | HV819464.1 | GM061219.1 | HC317600.1 | FB725712.1 | CS575754.1 |
| CQ788209.1 | CQ871217.1 | HI410677.1 | AX235776.1 | HV803281.1 | DL256296.1 | HC307843.1 | GM881783.1 | CS574928.1 |
| CQ787492.1 | CQ869289.1 | HI516580.1 | AX235241.1 | AY657096.1 | DL241165.1 | DM143061.1 | GM840854.1 | CS574800.1 |
| CQ787460.1 | CQ868899.1 | HI516507.1 | AX225241.1 | AY657064.1 | DL241904.1 | HB441224.1 | FB712023.1 | CS573050.1 |
| CQ787422.1 | CQ867418.1 | HI516109.1 | AX210346.1 | AY657032.1 | DL240250.1 | HB432482.1 | GM723125.1 | CS580001.1 |
| CQ787384.1 | CQ859639.1 | HI516077.1 | AX207297.1 | K03428.1 | FB748904.1 | HB426101.1 | GM041707.1 | CS565819.1 |
| CQ787348.1 | CQ859607.1 | HI554037.1 | AX202556.1 | M36664.1 | FB748852.1 | HB416494.1 | GM040838.1 | CS570752.1 |
| CQ787312.1 | CQ858155.1 | HI547561.1 | AX202431.1 | M12902.1 | DL236211.1 | HB416448.1 | CS729407.1 | CS546318.1 |
| CQ787280.1 | CQ855921.1 | HI284296.1 | AX193725.1 | M38660.1 | DL233595.1 | HB412597.1 | CS728604.1 | CS549245.1 |
| CQ787248.1 | CQ854840.1 | HI571654.1 | AX180283.1 | HV549720.1 | DL233396.1 | HB394806.1 | CS728572.1 | CS549180.1 |
| CQ786944.1 | CQ849461.1 | HI574517.1 | AF017063.1 | HV549075.1 | DL231384.1 | HB394773.1 | CS728540.1 | CS549176.1 |
| CQ785743.1 | CQ840183.1 | HI574485.1 | AX175356.1 | HV543983.1 | FB713749.1 | HB394239.1 | GM836379.1 | CS548913.1 |
| CQ784675.1 | CQ831273.1 | HI574453.1 | AX173377.1 | HV543860.1 | FB671030.1 | HB397653.1 | GM949534.1 | CS566302.1 |
| CQ784478.1 | CQ827719.1 | HI574389.1 | AX172945.1 | HV547337.1 | FB670164.1 | DM118008.1 | DL110159.1 | CS561196.1 |
| CQ778918.1 | CQ827545.1 | HI574366.1 | AX167497.1 | HV543556.1 | FB667455.1 | DM115568.1 | DL110127.1 | CS560468.1 |

| | | | | | | | | |
|---|---|---|---|---|---|---|---|---|
| CQ778510.1 | CQ824417.1 | HI566202.1 | AX167416.1 | HV543486.1 | FB666436.1 | DM114861.1 | DL110095.1 | CS559284.1 |
| CQ774634.1 | CQ821393.1 | HI593988.1 | AX155216.1 | HV538573.1 | FB671496.1 | HB388682.1 | DL105162.1 | DD431474.1 |
| CQ771664.1 | CQ821229.1 | HI592946.1 | AX154613.1 | HV543338.1 | DL119108.1 | HB387465.1 | AY657694.1 | DD420084.1 |
| CQ771632.1 | CQ819075.1 | HI588475.1 | AX146772.1 | HV542743.1 | DL119076.1 | HA642849.1 | AY657662.1 | DD432980.1 |
| CQ771600.1 | CQ817008.1 | HI637014.1 | AX145704.1 | HV542711.1 | DL119044.1 | HA641600.1 | AY657630.1 | DD432685.1 |
| CQ770995.1 | CQ816962.1 | HI636961.1 | AX145672.1 | HV542573.1 | DL119012.1 | HA641536.1 | AY657598.1 | DD421981.1 |
| CQ767248.1 | CQ816928.1 | HI001380.1 | AX145640.1 | HV541777.1 | DL114488.1 | HA641135.1 | AY657566.1 | DD421682.1 |
| CQ766072.1 | CQ815738.1 | HI001347.1 | AX145608.1 | HV514891.1 | DL114456.1 | HA637709.1 | AY657534.1 | DD420782.1 |
| CQ765892.1 | CQ814519.1 | HI001307.1 | AX145576.1 | HV514653.1 | DL104857.1 | HA644355.1 | AY657502.1 | CS541734.1 |
| CQ761208.1 | CQ814056.1 | HI003194.1 | AX145512.1 | HV536505.1 | DL104566.1 | GN032745.1 | AY657470.1 | CS543094.1 |
| CQ760563.1 | CQ814023.1 | HI003134.1 | AX145480.1 | HV535857.1 | DL104534.1 | GN032713.1 | AY657438.1 | CS538875.1 |
| CQ758831.1 | CQ813991.1 | HI003074.1 | AX145448.1 | HV530206.1 | DL049286.1 | GN032649.1 | AY657406.1 | CS538264.1 |
| CQ755282.1 | CQ813959.1 | HI003038.1 | AX145416.1 | HV515578.1 | DL011720.1 | GN032616.1 | AY657374.1 | CS537805.1 |
| CQ754046.1 | CQ813927.1 | HI002997.1 | AX145383.1 | HV515546.1 | DL011688.1 | GN032584.1 | AY657342.1 | CS510545.1 |
| CQ753331.1 | CQ813895.1 | HI002934.1 | AX145351.1 | HV515514.1 | DL011656.1 | GN032553.1 | AY657310.1 | CS503511.1 |
| CQ654953.1 | CQ813863.1 | HI002898.1 | AX145319.1 | HV515482.1 | DL011624.1 | GN032489.1 | AY657278.1 | CS503479.1 |
| CQ654698.1 | CQ813831.1 | HI001261.1 | AX145255.1 | HV515450.1 | DL040374.1 | GN032425.1 | AY657246.1 | CS503446.1 |
| CQ654530.1 | CQ813799.1 | HI001201.1 | AX145223.1 | HV515418.1 | DL028121.1 | GN032393.1 | AY657214.1 | CS502374.1 |
| CQ654393.1 | CQ813766.1 | HI001150.1 | AX145191.1 | HV515354.1 | DL028089.1 | GN032360.1 | AY657182.1 | CS502718.1 |
| CQ654108.1 | CQ812774.1 | HI001049.1 | AX099995.1 | HV504766.1 | DL028057.1 | GN032328.1 | AY657150.1 | CS502684.1 |
| CQ654016.1 | CQ803124.1 | HI001013.1 | AX097524.1 | HV504734.1 | DL028025.1 | GN032296.1 | AY657118.1 | CS502586.1 |
| AX963150.1 | CQ802851.1 | HI000968.1 | AX093170.1 | HV504702.1 | DL027993.1 | GN032264.1 | AY657086.1 | CS502412.1 |
| AX962178.1 | CQ801666.1 | HI002872.1 | AX092996.1 | HV504670.1 | CS628281.1 | GN032232.1 | AY657054.1 | CS502424.1 |
| AX960376.1 | CQ796832.1 | HI002837.1 | AX088759.1 | HV504638.1 | CS627819.1 | GN032200.1 | L23211.1 | CS502212.1 |
| FW302603.1 | CQ795787.1 | HI002799.1 | AX088707.1 | HI637696.1 | CS632193.1 | GN032168.1 | J02556.1 | CS501969.1 |
| FW301166.1 | CQ795480.1 | HI002740.1 | AX085504.1 | HI380389.1 | DD438421.1 | GN032136.1 | M10303.1 | CS501261.1 |
| FW300626.1 | CQ795448.1 | HI002654.1 | AX084286.1 | HI380300.1 | DD438016.1 | GN032104.1 | K00898.1 | CS499327.1 |
| HC470494.1 | AX141509.1 | HI002620.1 | AX081669.1 | HI379173.1 | DD449073.1 | GN032040.1 | M16351.1 | CS499059.1 |
| HC469399.1 | AX141445.1 | HI002581.1 | AX081005.1 | HI376928.1 | DD435537.1 | GN032008.1 | M38482.1 | CS499026.1 |
| HC486454.1 | AX141381.1 | HC869475.1 | AX080798.1 | HI376615.1 | CS616573.1 | GN031976.1 | HV549903.1 | CS498535.1 |
| HC486338.1 | AX141317.1 | FW304461.1 | AX079964.1 | HI375917.1 | CS613292.1 | GN031944.1 | HV549105.1 | CS497256.1 |
| HC467976.1 | AX138450.1 | FW309549.1 | AX078310.1 | HI375883.1 | CS612721.1 | GN031912.1 | HV548886.1 | CS305022.1 |
| HC475387.1 | AX137845.1 | FW308765.1 | AX077299.1 | HI372210.1 | CS611719.1 | GN031880.1 | HV547858.1 | CS302611.1 |
| HC466642.1 | AX134960.1 | FW332545.1 | A28087.1 | HI369621.1 | CS604498.1 | GN031848.1 | HV547463.1 | E63513.1 |
| HC466610.1 | AX134431.1 | HC767801.1 | AX076530.1 | HI369158.1 | CS604338.1 | GN031718.1 | HV543475.1 | CS299444.1 |
| HC492194.1 | AX128459.1 | HC767407.1 | AX074249.1 | HI369056.1 | CS603442.1 | GN031686.1 | HV543199.1 | CS297024.1 |
| HC466286.1 | AX127460.1 | HC757144.1 | AJ296081.1 | HI424507.1 | CS603410.1 | GN031654.1 | HV542108.1 | DD252258.1 |
| HC491765.1 | AX119327.1 | HC754651.1 | AX068384.1 | HI424131.1 | CS603346.1 | GN031622.1 | HV541511.1 | DD252143.1 |
| HC481597.1 | AX113924.1 | HC732139.1 | AX061571.1 | HI424099.1 | CS603282.1 | GN031558.1 | HV541328.1 | DD252111.1 |
| HC491458.1 | AX113542.1 | HC731682.1 | AX060780.1 | HI424067.1 | DD400176.1 | GN031526.1 | HV533390.1 | DD252079.1 |
| HC472338.1 | AX108249.1 | HC471766.1 | AX060090.1 | HI423841.1 | DD400143.1 | GN031494.1 | HV514612.1 | DD252013.1 |
| HC470676.1 | AX107121.1 | HC471734.1 | AX057303.1 | HI423809.1 | DD381343.1 | HW163843.1 | HV537389.1 | DD251311.1 |
| FV533518.1 | AX100345.1 | HC471726.1 | AX056826.1 | HI422961.1 | CS459125.1 | HW163123.1 | FW418986.1 | DD250417.1 |
| FV533437.1 | AX097512.1 | HC471670.1 | AX055858.1 | HH762702.1 | DD367783.1 | HW155678.1 | FW396660.1 | DD248988.1 |
| FV531720.1 | AX097402.1 | HC471638.1 | AX052962.1 | HI415779.1 | DD361274.1 | HW144774.1 | HH835089.1 | DD258252.1 |
| FV531688.1 | AX093092.1 | HC471606.1 | AX050333.1 | HI415747.1 | DD367534.1 | HW155224.1 | HH834393.1 | DD257582.1 |
| FV531379.1 | AX089771.1 | HC921525.1 | AX046224.1 | HI414998.1 | DD368136.1 | HW154873.1 | HH924310.1 | CS287895.1 |

| | | | | | | | | |
|---|---|---|---|---|---|---|---|---|
| FV530954.1 | AX082156.1 | HC920686.1 | AX044424.1 | HI414034.1 | DD361308.1 | HV753425.1 | HH931937.1 | CS287569.1 |
| FV530774.1 | AX078903.1 | HD068877.1 | AX040463.1 | HI413997.1 | CS453771.1 | HV743337.1 | HH925713.1 | DD240944.1 |
| FV522826.1 | AX076584.1 | HD063945.1 | AX039586.1 | HI413593.1 | CS450570.1 | HV743297.1 | HH827047.1 | DD240717.1 |
| FV530201.1 | AX073719.1 | HD063495.1 | AX039327.1 | FW351383.1 | DD357939.1 | HV743258.1 | HH825961.1 | DD240669.1 |
| FV528646.1 | AX063715.1 | HC920305.1 | AX039173.1 | FW351231.1 | DD357905.1 | HV743221.1 | HH822195.1 | DD240613.1 |
| HB836573.1 | AX061288.1 | HD057682.1 | AX037560.1 | FW350955.1 | DD357797.1 | HV744573.1 | FW378487.1 | DD240497.1 |
| HB843809.1 | AX058844.1 | HD061087.1 | AX037088.1 | HD082419.1 | DD357586.1 | HV703163.1 | FW336106.1 | DD240465.1 |
| HB836340.1 | AX057961.1 | M19996.1 | AX036643.1 | HD088242.1 | A18614.1 | HV702431.1 | HC887330.1 | DD239971.1 |
| HB835834.1 | AX056087.1 | M27770.1 | AX036034.1 | HD077952.1 | CS444538.1 | HV701593.1 | HC873813.1 | DD238961.1 |
| HB843138.1 | AX054812.1 | FW341967.1 | AX034871.1 | HD070436.1 | CS446152.1 | HV701239.1 | HC873718.1 | DD238437.1 |
| HB819135.1 | AX052946.1 | HC902356.1 | AX029455.1 | HD070393.1 | CS438912.1 | HV701175.1 | FW310607.1 | DD236648.1 |
| HB806802.1 | AX052911.1 | HC868181.1 | HV437554.1 | HD080148.1 | CS434847.1 | HV701111.1 | FW308822.1 | DD234720.1 |
| HB842036.1 | AX049480.1 | HC887859.1 | HV445412.1 | FW345096.1 | DD335672.1 | HV708547.1 | HC315644.1 | DD247190.1 |
| HB841735.1 | AX046142.1 | HC887340.1 | HV349088.1 | FW345006.1 | DD347405.1 | HV708507.1 | HC314513.1 | DD247123.1 |
| HB841488.1 | AX040771.1 | HC873821.1 | HV341328.1 | FW344974.1 | DD347205.1 | HV708214.1 | HC316594.1 | DD246851.1 |
| HB840913.1 | AX036753.1 | HC288862.1 | HV344006.1 | FW344683.1 | CS425050.1 | HV708177.1 | HC309368.1 | CS283547.1 |
| HB840393.1 | AX036602.1 | HC292535.1 | HV343970.1 | FW343732.1 | DD047835.1 | HV701092.1 | HC307889.1 | AX047764.1 |
| HB650120.1 | AX036021.1 | HC292290.1 | HV343778.1 | FW343536.1 | DD038503.1 | HV701060.1 | HC307814.1 | AX047596.1 |
| HB649984.1 | AX035968.1 | HC295032.1 | HV343586.1 | HC924163.1 | DD037220.1 | HV701028.1 | HC307393.1 | AX046264.1 |
| HB649210.1 | AX034258.1 | HC294852.1 | HV347825.1 | HC923001.1 | DD034057.1 | HV695522.1 | HC306920.1 | AX043904.1 |
| HB645717.1 | HW260986.1 | HC299183.1 | HV347759.1 | HC921526.1 | DD027039.1 | HV695458.1 | FU263223.1 | AX041930.1 |
| HB645685.1 | HW260954.1 | HC299125.1 | HV344770.1 | HC920688.1 | DD023870.1 | HV694803.1 | FU262771.1 | AX040466.1 |
| HB645609.1 | HW260922.1 | HC294612.1 | HV347320.1 | HD068882.1 | DD023579.1 | HV700840.1 | FU267912.1 | AX039596.1 |
| HB850776.1 | HW260890.1 | HC298740.1 | HV339993.1 | HD052874.1 | DD020514.1 | HV694736.1 | HC313231.1 | AX039498.1 |
| HB856019.1 | HW260826.1 | HC290905.1 | HV346971.1 | HD050991.1 | DD028805.1 | HV694672.1 | HC306188.1 | AX039447.1 |
| HB855907.1 | HW260794.1 | HC293493.1 | HV344686.1 | HC920307.1 | DD010175.1 | HV693367.1 | HC306152.1 | AX039257.1 |
| HB855436.1 | HW260762.1 | HC293373.1 | HV339967.1 | HC313081.1 | DD017639.1 | HV699775.1 | HC306032.1 | AX039200.1 |
| DM179544.1 | HW260730.1 | HC293176.1 | FW592639.1 | DM467459.1 | BD434182.1 | HV699492.1 | HC305992.1 | AX037567.1 |
| DM179154.1 | HW260698.1 | HC289695.1 | FW577583.1 | HC306239.1 | BD453852.1 | HV699147.1 | HC302636.1 | AX037304.1 |
| DM178830.1 | HW260666.1 | HC207536.1 | FW576691.1 | HC306199.1 | BD453820.1 | HV698223.1 | HC302246.1 | AX036654.1 |
| DM170826.1 | HW260634.1 | AF306719.1 | FW575889.1 | HC302645.1 | BD453788.1 | HV698139.1 | DM375337.1 | AX035985.1 |
| DM185755.1 | HW260602.1 | HC195333.1 | FW575365.1 | HC305932.1 | BD433652.1 | HV306175.1 | DM370679.1 | HW240596.1 |
| DM185693.1 | HW260570.1 | HC195219.1 | FW571676.1 | HC305892.1 | BD453764.1 | HV302969.1 | DM370647.1 | HW239411.1 |
| DM170615.1 | HW260538.1 | HC193593.1 | FW572725.1 | HC305812.1 | AX144655.1 | HV302937.1 | DM370615.1 | HW238937.1 |
| DM188246.1 | HW260506.1 | HC191375.1 | FW573881.1 | HC305772.1 | AX144623.1 | HV308714.1 | DM370583.1 | HW238539.1 |
| DM188046.1 | HW260474.1 | HC190522.1 | FW573699.1 | HC305732.1 | AX144569.1 | HV226845.1 | DM370551.1 | HW238415.1 |
| DM173724.1 | HW260442.1 | HC187073.1 | FW570229.1 | HC305692.1 | AX144441.1 | HV235790.1 | DM370519.1 | HW238199.1 |
| HB491464.1 | HW260410.1 | HC089577.1 | FW568972.1 | HC305652.1 | AX144313.1 | HV221130.1 | DM370487.1 | HW238011.1 |
| HB488826.1 | A28863.1 | HC089545.1 | FW574729.1 | HC302010.1 | AX144183.1 | HV040123.1 | DM367512.1 | HW242775.1 |
| HB488692.1 | A27719.1 | HC089513.1 | FW572684.1 | HC301625.1 | AX144119.1 | HV200503.1 | DM372000.1 | HW237870.1 |
| DM164207.1 | A25893.1 | HC089481.1 | FW572214.1 | HC294290.1 | AX144055.1 | HV182452.1 | DM371346.1 | HW237696.1 |
| DM164028.1 | A20026.1 | HC089449.1 | FW574654.1 | HC289342.1 | AX143925.1 | HV182420.1 | DM209289.1 | HW237433.1 |
| DM164013.1 | A28466.1 | HC089416.1 | FW574393.1 | HC292972.1 | AX143797.1 | HV182388.1 | HC003070.1 | HW242326.1 |
| DM163979.1 | A26061.1 | HC089384.1 | FW568747.1 | HC296930.1 | AX143733.1 | HV235490.1 | HB865037.1 | HW242294.1 |
| DM163482.1 | A25693.1 | HC089352.1 | FW572088.1 | HC295710.1 | AX143541.1 | HV232679.1 | HB864967.1 | HW242262.1 |
| DM162526.1 | A24412.1 | HC089319.1 | HI967436.1 | HC288863.1 | AX143221.1 | HV038678.1 | HB864997.1 | HW237110.1 |
| DM162375.1 | A25927.1 | HC089287.1 | HI968460.1 | HC295688.1 | AX142901.1 | HV182350.1 | HB864941.1 | HW241546.1 |

| | | | | | | | | |
|---|---|---|---|---|---|---|---|---|
| DL462982.1 | A24545.1 | HC089255.1 | HI979300.1 | HC295556.1 | AX142837.1 | HV182318.1 | HB864909.1 | HW243061.1 |
| DL481634.1 | A23268.1 | AY774993.1 | HI988942.1 | HC292547.1 | AX142773.1 | HV182286.1 | HB864877.1 | HW243029.1 |
| DL460982.1 | A21989.1 | AY774942.1 | HI968579.1 | HC292302.1 | AX142709.1 | HV182050.1 | HB864845.1 | HW241368.1 |
| GM833179.1 | A14587.1 | AY774901.1 | HI987659.1 | HC295044.1 | AX142517.1 | HV190957.1 | HB864813.1 | HW163494.1 |
| GM712171.1 | A12400.1 | AY774839.1 | HI987436.1 | HC294864.1 | AX142451.1 | HV190828.1 | HB864781.1 | HW163441.1 |
| GM831172.1 | A21600.1 | AY774780.1 | HI930648.1 | HC294624.1 | AX142387.1 | HI637011.1 | HB864749.1 | HW163316.1 |
| GM711369.1 | A19012.1 | AY774670.1 | HI949806.1 | HC291102.1 | AX142195.1 | HI636960.1 | HB866537.1 | HW160829.1 |
| FB509327.1 | A20718.1 | AY774616.1 | HI929254.1 | HC290917.1 | AX142131.1 | HI636923.1 | HB866214.1 | HW160635.1 |
| FB509295.1 | A19298.1 | AY774545.1 | HI919632.1 | HC293505.1 | AX142067.1 | HI001378.1 | DD165459.1 | HW160603.1 |
| FB509231.1 | A18748.1 | AY774444.1 | HI918323.1 | HC293385.1 | AX142001.1 | HI001346.1 | CS001506.1 | HW155775.1 |
| GM675060.1 | A18323.1 | AY774381.1 | HI918271.1 | HC296938.1 | AX141871.1 | HI003073.1 | CS157813.1 | HW150142.1 |
| FB509184.1 | A17140.1 | AY774329.1 | HI916904.1 | HC289714.1 | AX141807.1 | HI003037.1 | CS157781.1 | HW150110.1 |
| FB509114.1 | A05515.1 | AY774280.1 | HI923665.1 | HC199258.1 | AX141423.1 | HI002996.1 | CS157942.1 | HW150014.1 |
| GM827554.1 | A14454.1 | AY774224.1 | HI657213.1 | AY255709.1 | AX141359.1 | HI002932.1 | CS157878.1 | HW149982.1 |
| GM709017.1 | A13627.1 | AY774106.1 | FW498195.1 | DM464701.1 | AX133919.1 | HI002891.1 | CS157846.1 | HW144830.1 |
| GM655014.1 | A13203.1 | AY774038.1 | FW496127.1 | HC193934.1 | AX127227.1 | HI001260.1 | CS126034.1 | HW155369.1 |
| GM654982.1 | A12573.1 | AY515503.1 | FW497433.1 | HC193596.1 | HV344620.1 | HI001200.1 | CS124699.1 | HW155207.1 |
| GM755079.1 | A12274.1 | HC083723.1 | FW503884.1 | HC191384.1 | HV339976.1 | HI001149.1 | CS119527.1 | HW041939.1 |
| GM836996.1 | A10619.1 | HC083482.1 | FW499106.1 | HC190523.1 | HV344506.1 | HI001047.1 | CS119494.1 | HW041841.1 |
| FB511157.1 | A08564.1 | GN046257.1 | FW504940.1 | GN030483.1 | HV342157.1 | HI001012.1 | CS119426.1 | HW049469.1 |
| GM752809.1 | A06503.1 | DM010512.1 | FW504766.1 | GN030413.1 | HV342073.1 | HI000966.1 | CS119393.1 | HW049393.1 |
| DL460750.1 | A08526.1 | DM023375.1 | FW503675.1 | GN030323.1 | HV321544.1 | HI002871.1 | CS119361.1 | HW049362.1 |
| DL467586.1 | A08138.1 | DM021625.1 | FW500469.1 | GN030195.1 | HV324799.1 | HI002836.1 | CS119329.1 | HW049330.1 |
| GM660080.1 | A07733.1 | DM037115.1 | FW498994.1 | GN029841.1 | HV323282.1 | HI002798.1 | CS119295.1 | HW049298.1 |
| GM624869.1 | A07227.1 | DM026249.1 | FW420445.1 | GN029809.1 | HV119954.1 | HI002739.1 | CS119231.1 | HW049266.1 |
| GM624831.1 | A06392.1 | GN043370.1 | FW420413.1 | HV931258.1 | HV187357.1 | HI002653.1 | CS119199.1 | HW041805.1 |
| GM632652.1 | A04541.1 | GN042518.1 | FW501794.1 | HV925522.1 | HV195745.1 | HI002619.1 | CS119167.1 | HW041773.1 |
| GM632578.1 | A03843.1 | GN041732.1 | FW501747.1 | HV936351.1 | HV208793.1 | HI002580.1 | CS119135.1 | HW054946.1 |
| GM632612.1 | A00097.1 | GN030706.1 | FW496943.1 | HV930700.1 | HV245448.1 | HI004533.1 | CS119102.1 | HW041552.1 |
| GM658428.1 | M60507.1 | GN030674.1 | FW420388.1 | HV819641.1 | HV214876.1 | HI004469.1 | CS119068.1 | HW047143.1 |
| DD400161.1 | K01247.1 | GN030642.1 | FW420324.1 | HV932911.1 | HV227065.1 | HI002428.1 | CS119002.1 | HW054058.1 |
| DD400129.1 | M31317.1 | GN030610.1 | FW496743.1 | HV932684.1 | HV302958.1 | HI002311.1 | CS118970.1 | HW054017.1 |
| CS460784.1 | HV708213.1 | GN030578.1 | FW496511.1 | HV932652.1 | HI575292.1 | HI000514.1 | CS118937.1 | HW048305.1 |
| CS459108.1 | HV701091.1 | GN030546.1 | HI653886.1 | HV803202.1 | HI465383.1 | HI006799.1 | CS118905.1 | HW053775.1 |
| DD361292.1 | HV701059.1 | GN030514.1 | HI653809.1 | HV814091.1 | HI001635.1 | HI180158.1 | CS118871.1 | HW029709.1 |
| DD367333.1 | HV694834.1 | GN030482.1 | HI653720.1 | HV802936.1 | HI001593.1 | HI202879.1 | CS118839.1 | HV975360.1 |
| DD362228.1 | HV694802.1 | GN030422.1 | HI653637.1 | HV802904.1 | HI003375.1 | HI202830.1 | CS118805.1 | HV985977.1 |
| DD368196.1 | HV699491.1 | GN030354.1 | HI653603.1 | HV818561.1 | HI003337.1 | HI214558.1 | CS118773.1 | HV985945.1 |
| DD361326.1 | HV698138.1 | GN030226.1 | HI653533.1 | HV453159.1 | HI003298.1 | HI214526.1 | CS118708.1 | HV985913.1 |
| CS457944.1 | HV689464.1 | GN030194.1 | HI653492.1 | HV452809.1 | HI464743.1 | HI214460.1 | CS118676.1 | HV985868.1 |
| CS457148.1 | HV695886.1 | GN029840.1 | HI653424.1 | HV450831.1 | HI464711.1 | HI214428.1 | CS118612.1 | HV988673.1 |
| CS456705.1 | HV600332.1 | GN013592.1 | HI653366.1 | HV444809.1 | HI464679.1 | HI179567.1 | CS118579.1 | HV984516.1 |
| CS453661.1 | HV579119.1 | GN010245.1 | HI653284.1 | HV349320.1 | HI464647.1 | HI212997.1 | CS118544.1 | HW028624.1 |
| CS451665.1 | HV577246.1 | GN033542.1 | HI653226.1 | HV348783.1 | HI550133.1 | HI212928.1 | CS118511.1 | HV961283.1 |
| CS451008.1 | HV582879.1 | GN033510.1 | HI651050.1 | HV351533.1 | HI464451.1 | HI203495.1 | CS118478.1 | HV960999.1 |
| DD359133.1 | FW576783.1 | GN033478.1 | HI647560.1 | HV341348.1 | HI504973.1 | HI546345.1 | CS118446.1 | HV960577.1 |
| E01249.1 | FW576748.1 | GN033446.1 | HI002667.1 | HV341230.1 | HI542506.1 | HI546313.1 | CS118414.1 | HV960282.1 |

| | | | | | | | | |
|---|---|---|---|---|---|---|---|---|
| E01038.1 | FW576715.1 | GN033414.1 | HI002633.1 | HV343580.1 | HI462582.1 | AY659268.1 | CS118381.1 | HV960153.1 |
| E00886.1 | FW576675.1 | GN033382.1 | HI002598.1 | HV344908.1 | HH725164.1 | AY659236.1 | CS118347.1 | HV959935.1 |
| E00413.1 | FW573457.1 | GN033350.1 | HI002539.1 | HV347351.1 | HH713461.1 | AY659204.1 | CS118313.1 | HV959487.1 |
| E00321.1 | FW574614.1 | GN033318.1 | HI001491.1 | HV340044.1 | HH961366.1 | AY659172.1 | CS118248.1 | HV964049.1 |
| E00187.1 | FW574313.1 | GN033286.1 | HI004557.1 | HV347067.1 | HH961334.1 | AY659140.1 | CS118216.1 | HV970076.1 |
| DD146378.1 | FW570979.1 | DD227410.1 | HI004429.1 | HV344632.1 | HH961302.1 | AY659108.1 | CS118182.1 | HV963871.1 |
| DD088019.1 | FW571813.1 | DD224650.1 | HI002403.1 | HV339986.1 | HH961270.1 | AY659076.1 | CS118149.1 | HV958699.1 |
| DD146148.1 | FW569611.1 | DD224161.1 | HI002324.1 | HV339922.1 | HH961238.1 | AY659044.1 | CS118117.1 | HV963679.1 |
| DD086312.1 | HI968055.1 | DD233557.1 | HI000530.1 | HV339890.1 | HH964261.1 | AY659012.1 | CS118084.1 | HV966194.1 |
| DD100023.1 | HI987463.1 | DD231554.1 | HI180494.1 | HV344518.1 | HH974921.1 | AY658980.1 | AX143287.1 | HV958156.1 |
| DD143660.1 | HI987423.1 | CQ757648.1 | HI180170.1 | HV342169.1 | HH974526.1 | AY658948.1 | AX143159.1 | HV963322.1 |
| DD069972.1 | HI638974.1 | CS260193.1 | HI178222.1 | HI413995.1 | HH998098.1 | AY658916.1 | AX143095.1 | HV965990.1 |
| DD069881.1 | HI637694.1 | DD214012.1 | HI202842.1 | HI413681.1 | HH998044.1 | AY658884.1 | AX142389.1 | HV965958.1 |
| DD112552.1 | HI380828.1 | DD213995.1 | HI202810.1 | HI413591.1 | HH997991.1 | AY658852.1 | AX142133.1 | HV962275.1 |
| DD143246.1 | HI379169.1 | DD213902.1 | HI177875.1 | HI412697.1 | HH999685.1 | AY658820.1 | AX142069.1 | HV962211.1 |
| DD084334.1 | HI378571.1 | DD213870.1 | HI214570.1 | HI516573.1 | HH999572.1 | AY658788.1 | AX142003.1 | HV561965.1 |
| DD098184.1 | HI378155.1 | DD213838.1 | HI214538.1 | HI516449.1 | HH997800.1 | AY658756.1 | AX141745.1 | HV561867.1 |
| DD132355.1 | HI378123.1 | DD213806.1 | DL099002.1 | HI516107.1 | HH997755.1 | AY658724.1 | AX141617.1 | HV550130.1 |
| DD141439.1 | HI376914.1 | DD216476.1 | DL098970.1 | HI516075.1 | HH999436.1 | AY658692.1 | AX141553.1 | HV550974.1 |
| DD141287.1 | HI375915.1 | DD216439.1 | DL098938.1 | HI585469.1 | HH999394.1 | AY658660.1 | AX139410.1 | HV550377.1 |
| DD152088.1 | HI375881.1 | DD220786.1 | DL092928.1 | HI284294.1 | HH997634.1 | AY658628.1 | AX133920.1 | AY659413.1 |
| DD158435.1 | HI372208.1 | DD213579.1 | DL092896.1 | HI574515.1 | HH997577.1 | AY658596.1 | AF368503.1 | AY659381.1 |
| DD158410.1 | HI370287.1 | DD213547.1 | DL092864.1 | HI574483.1 | HH997509.1 | AY658564.1 | AX112904.1 | FW377039.1 |
| DD157354.1 | HI369619.1 | DD213490.1 | DL092832.1 | HI574451.1 | HH997363.1 | AY658532.1 | AX107111.1 | FW372000.1 |
| DD155783.1 | HI369156.1 | DD215949.1 | DL092800.1 | HI574419.1 | HH999228.1 | AY658500.1 | AX105822.1 | FW371968.1 |
| DD153649.1 | HI369101.1 | DQ354464.1 | DL089153.1 | HI574364.1 | HH999184.1 | AY658468.1 | AX097501.1 | FW371936.1 |
| DD152102.1 | HI368607.1 | DD212258.1 | DL089121.1 | HI578448.1 | HH999139.1 | AY658436.1 | AX093081.1 | FW371904.1 |
| DD096742.1 | HI424129.1 | DD211839.1 | DL089089.1 | HI566198.1 | HH999108.1 | AY658404.1 | AX085931.1 | FW376208.1 |
| DD081759.1 | HI424097.1 | DD192531.1 | DL089057.1 | HI593986.1 | HH999059.1 | AY658372.1 | AX081289.1 | FW381546.1 |
| DD148361.1 | HI423807.1 | DD187321.1 | DL089025.1 | HI637004.1 | HH999001.1 | AY658340.1 | AX080825.1 | FW376316.1 |
| DD147792.1 | HI423775.1 | DD206914.1 | DL088993.1 | HI636959.1 | HH998951.1 | AY658308.1 | AX077718.1 | FW375770.1 |
| DD147760.1 | HI422922.1 | CS016575.1 | DL122352.1 | HI001377.1 | HH997231.1 | AY658276.1 | AX076989.1 | FW381129.1 |
| DD147728.1 | FW366502.1 | CS008525.1 | DL122320.1 | HI001345.1 | HH997160.1 | AY658244.1 | AX074315.1 | FW375538.1 |
| DD147696.1 | FW351381.1 | CQ986625.1 | DL118002.1 | HI001302.1 | HH998903.1 | AY658212.1 | AX068912.1 | FW369981.1 |
| DD147149.1 | FW351228.1 | CQ985787.1 | DL117970.1 | HI003189.1 | HH998845.1 | AY658180.1 | AX061414.1 | HC083884.1 |
| DD095718.1 | FW350132.1 | CQ983144.1 | DL117938.1 | HI003132.1 | HH998794.1 | AY658148.1 | HW381364.1 | HC083706.1 |
| DD137241.1 | HD122421.1 | CQ983063.1 | DL117906.1 | HI003070.1 | HH998735.1 | AY658116.1 | HW381027.1 | HC083529.1 |
| DD134018.1 | HD114570.1 | CQ981118.1 | DL117874.1 | HI003036.1 | HH996973.1 | AY658084.1 | HW350994.1 | DM460860.1 |
| DD132689.1 | HD082416.1 | CQ975456.1 | DL117842.1 | HI002995.1 | HH981100.1 | AY658052.1 | HW350962.1 | HC061849.1 |
| DD132289.1 | HD081917.1 | CQ979567.1 | DL113121.1 | HI002931.1 | BD171809.1 | AY658020.1 | HW350886.1 | HC061380.1 |
| DD093951.1 | HD088210.1 | CQ976604.1 | DL113089.1 | HI002890.1 | AX608832.1 | AY657988.1 | HW340462.1 | HC060623.1 |
| DD106663.1 | HD080675.1 | CQ972390.1 | DL113057.1 | HI001259.1 | AX601772.1 | AY657956.1 | HW350663.1 | HC059789.1 |
| DD092957.1 | FW345050.1 | CQ972358.1 | DL108256.1 | HI001199.1 | AX601601.1 | AY657924.1 | HW350295.1 | HC059666.1 |
| DD106231.1 | FW344681.1 | CQ971116.1 | DL108224.1 | HI001148.1 | AX601465.1 | AY657892.1 | HW350165.1 | HC058883.1 |
| CS061031.1 | AY774991.1 | CQ970791.1 | DL108192.1 | HI001045.1 | AX601369.1 | AY657860.1 | HW339640.1 | HC058737.1 |
| CS059009.1 | AY774939.1 | CQ969936.1 | DL108160.1 | HI001011.1 | AX601337.1 | AY657828.1 | HW266136.1 | HC058657.1 |
| AY967387.1 | AY774899.1 | CQ947530.1 | DL108128.1 | HI000965.1 | AX599991.1 | AY657796.1 | HW266053.1 | HC058544.1 |

| | | | | | | | | |
|---|---|---|---|---|---|---|---|---|
| AY967355.1 | AY774663.1 | CQ947281.1 | DL108096.1 | HI002870.1 | AX597635.1 | AY657764.1 | HW263045.1 | HC058383.1 |
| AY967323.1 | AY774612.1 | CQ947117.1 | DL103490.1 | HI002835.1 | AX593491.1 | AY657732.1 | HW263013.1 | HC058107.1 |
| AY967291.1 | AY774541.1 | AX540475.1 | DL103458.1 | HI002797.1 | AX592526.1 | AY657700.1 | HW262940.1 | HC057818.1 |
| AY967259.1 | AY774377.1 | AX536238.1 | DL103426.1 | HI002738.1 | HW389046.1 | AY657668.1 | HW262838.1 | HC057200.1 |
| AY967227.1 | AY774325.1 | AX528973.1 | DL103394.1 | FW334246.1 | HW388325.1 | AY657636.1 | HW262744.1 | DM459938.1 |
| AY967195.1 | AY774277.1 | AX528463.1 | DL098916.1 | HC767390.1 | HW387492.1 | AY657604.1 | HW261392.1 | DM459754.1 |
| AY967163.1 | AY774158.1 | A30509.1 | DL098884.1 | HC757142.1 | HW380920.1 | AY657572.1 | HW261360.1 | DM383642.1 |
| AY967131.1 | K02125.1 | AX179476.1 | DL098852.1 | HC755770.1 | HW381361.1 | AY657540.1 | HW261328.1 | DM383023.1 |
| AY967099.1 | AF274585.1 | AX164120.1 | DL098820.1 | HC754649.1 | HW381024.1 | AY657508.1 | HW261296.1 | DM381867.1 |
| AY967067.1 | HC070445.1 | AX521671.1 | DL098788.1 | HC741969.1 | HW302049.1 | HH997306.1 | HW261264.1 | HC054886.1 |
| AY967035.1 | GN046252.1 | AX521532.1 | DL098756.1 | HC732137.1 | HW302005.1 | HH997178.1 | HW261232.1 | HC053899.1 |
| AY967003.1 | DM010499.1 | AX521500.1 | DL092714.1 | HC471764.1 | HW294988.1 | HH998917.1 | HW261200.1 | HC053834.1 |
| AY966971.1 | DM023761.1 | AF430186.1 | DL092682.1 | HC471732.1 | HW294005.1 | HH998913.1 | HW261168.1 | HC051950.1 |
| AY966939.1 | DM023371.1 | AF430154.1 | DL092650.1 | HC471724.1 | HW293262.1 | HH998859.1 | HW261104.1 | HC051239.1 |
| CS056287.1 | DM011435.1 | AX505197.1 | DL092618.1 | HC471668.1 | HW293042.1 | HH998803.1 | HC293032.1 | HC050172.1 |
| CS053037.1 | DM021878.1 | AX504967.1 | DL092586.1 | HC471636.1 | HW291181.1 | HH998750.1 | HC289857.1 | HC045510.1 |
| CS052388.1 | DM016240.1 | AX496853.1 | DL088971.1 | HC689128.1 | HW291149.1 | HH997089.1 | HC208008.1 | HC045478.1 |
| CS052320.1 | DM021619.1 | BD140001.1 | DL088939.1 | HC688424.1 | HW291052.1 | HH997035.1 | HC201534.1 | HC045446.1 |
| CS050985.1 | DM026466.1 | BD138395.1 | DL088907.1 | HC037092.1 | HW291020.1 | HH996990.1 | DM462463.1 | HC045414.1 |
| CS047670.1 | DM026246.1 | BD137988.1 | DL103282.1 | HC025528.1 | HW290956.1 | HH996942.1 | HC196680.1 | HC045350.1 |
| CS038824.1 | GN045520.1 | BD136686.1 | DL103250.1 | HC025496.1 | HW290924.1 | HH998699.1 | HC193429.1 | HC045318.1 |
| CS029937.1 | GN041720.1 | BD135144.1 | DL103218.1 | HC025464.1 | HW290893.1 | HH998658.1 | HC187669.1 | HC045286.1 |
| CS025526.1 | GN034656.1 | BD131935.1 | DL107970.1 | HC025432.1 | HW290861.1 | HH998530.1 | HC089565.1 | HC047466.1 |
| CS023848.1 | GN030704.1 | BD131902.1 | DL107938.1 | HC010418.1 | HW287787.1 | HH996916.1 | HC089533.1 | HC047434.1 |
| CS023737.1 | GN030640.1 | BD130795.1 | DL107906.1 | HC007670.1 | DI170075.1 | HH996799.1 | HC089501.1 | HC047402.1 |
| CS022576.1 | GN030608.1 | BD130763.1 | DL092448.1 | HC010679.1 | HW269606.1 | HH996760.1 | HC089469.1 | HC047370.1 |
| CS018551.1 | GN030576.1 | BD130729.1 | DL092416.1 | DM370686.1 | HW267403.1 | HH977558.1 | HC089437.1 | HC047338.1 |
| CS018487.1 | GN030544.1 | BD129647.1 | DL092384.1 | DM370654.1 | HW266133.1 | HH977364.1 | HC089404.1 | HC047306.1 |
| AX601559.1 | GN030512.1 | BD107641.1 | DL088769.1 | DM370622.1 | HW266046.1 | HH980628.1 | HC089372.1 | HC047274.1 |
| AX601359.1 | GN030480.1 | BD105715.1 | DL088737.1 | DM370590.1 | HW265869.1 | HH980531.1 | HC089340.1 | HC047242.1 |
| AX601327.1 | GN030424.1 | BD103159.1 | DL088705.1 | DM370558.1 | HW263042.1 | HH980457.1 | HC089307.1 | HC047210.1 |
| AX599029.1 | GN030384.1 | BD095063.1 | DL088673.1 | AF397139.1 | HW262835.1 | HH980349.1 | GN030870.1 | HC047178.1 |
| AX598983.1 | GN030352.1 | BD084910.1 | DL088641.1 | CS157789.1 | HW262741.1 | HH980289.1 | GN030838.1 | HC047146.1 |
| AX598875.1 | GN030288.1 | BD080673.1 | DL088609.1 | CS157950.1 | HW261389.1 | HH980188.1 | GN030806.1 | HC047114.1 |
| AX598823.1 | GN030224.1 | BD080324.1 | DL112955.1 | CS157918.1 | HW261357.1 | HH980149.1 | GN030774.1 | HC047082.1 |
| AX598773.1 | GN030192.1 | BD076477.1 | DL033937.1 | CS157822.1 | HW261325.1 | HH980097.1 | DM005049.1 | HC047050.1 |
| GM657839.1 | GN030160.1 | BD074976.1 | DL025358.1 | CS124667.1 | HW261293.1 | HH979864.1 | DM004979.1 | HC047018.1 |
| GM657807.1 | GN029838.1 | BD074940.1 | DL025326.1 | CS124633.1 | HW261261.1 | HH996696.1 | DM003480.1 | HC046986.1 |
| GM657774.1 | GN010243.1 | BD057809.1 | DL025294.1 | CS124545.1 | HW261229.1 | HH996635.1 | DL489764.1 | HC046954.1 |
| GM657742.1 | GN033572.1 | BD023616.1 | DL025262.1 | CS119875.1 | HW261165.1 | HH996580.1 | DL483447.1 | HA635423.1 |
| GM643255.1 | GN033540.1 | BD016723.1 | DL025230.1 | CS119502.1 | HW261133.1 | HH998390.1 | GM983735.1 | HA639885.1 |
| GM636202.1 | GN033508.1 | BD015660.1 | DL025166.1 | CS119469.1 | HW261101.1 | HH998357.1 | GM996601.1 | DM106884.1 |
| GM631634.1 | GN033476.1 | A35183.1 | DL021954.1 | CS119402.1 | HW261069.1 | HH999932.1 | GM993553.1 | DM102737.1 |
| GM631602.1 | GN033444.1 | A33976.1 | DL021922.1 | CS119337.1 | HW261005.1 | HH979787.1 | GM992955.1 | DM092261.1 |
| GM631570.1 | GN033380.1 | A32818.1 | DL021858.1 | CS119303.1 | HW260973.1 | HH979704.1 | GM992504.1 | DM105591.1 |
| GM626169.1 | GN033348.1 | A30380.1 | DL021826.1 | CS119271.1 | HW260941.1 | HH999901.1 | GM976350.1 | DM105567.1 |
| GM626137.1 | GN033316.1 | A29911.1 | DL021794.1 | CS119207.1 | HW260909.1 | HH999858.1 | GM989820.1 | DM105521.1 |

| | | | | | | | | |
|---|---|---|---|---|---|---|---|---|
| GM652970.1 | GN033252.1 | A23343.1 | DL017428.1 | CS119143.1 | HW260877.1 | HH999808.1 | GM968464.1 | DM105425.1 |
| GM652938.1 | GN033188.1 | A31645.1 | DL038464.1 | CS119076.1 | HW260845.1 | HH998324.1 | FB986404.1 | DM100607.1 |
| GM652906.1 | GN033156.1 | A26530.1 | DL038432.1 | CS119043.1 | HW260813.1 | HH998270.1 | GM970624.1 | DM095105.1 |
| GM652874.1 | GM630648.1 | A16258.1 | DL038400.1 | CS119011.1 | HW260781.1 | HH998233.1 | GM649267.1 | DM094910.1 |
| GM652842.1 | GM630616.1 | A20099.1 | DL038368.1 | CS118978.1 | HW260749.1 | HH998186.1 | GM649235.1 | DM103656.1 |
| GM652810.1 | GM630584.1 | A10162.1 | DL038336.1 | CS118946.1 | HV187355.1 | HH998135.1 | GM649203.1 | DM093345.1 |
| GM652777.1 | GM621649.1 | A08469.1 | DL013173.1 | CS118913.1 | HV208791.1 | DL107085.1 | GM656315.1 | GN374385.1 |
| GM638788.1 | GM621617.1 | A05022.1 | DL013141.1 | CS118879.1 | HV214874.1 | DL107053.1 | GM656283.1 | GN367627.1 |
| GM638756.1 | GM621585.1 | A02087.1 | DL013109.1 | CS118847.1 | HV035390.1 | DL106989.1 | GM656251.1 | GN367594.1 |
| GM626113.1 | GM621553.1 | A32008.1 | DL013077.1 | CS118814.1 | FZ422168.1 | DL106957.1 | GM736753.1 | GN367554.1 |
| GM626081.1 | GM657133.1 | A31083.1 | DL013045.1 | CS118781.1 | FZ431238.1 | DL106925.1 | GM697138.1 | GN367231.1 |
| GM626049.1 | GM656910.1 | DL101124.1 | DL013013.1 | CS118748.1 | FZ429333.1 | DL102192.1 | FB671023.1 | GN370766.1 |
| GM626017.1 | GM656878.1 | DL096614.1 | DL033908.1 | CS118716.1 | FZ412047.1 | DL109150.1 | FB670944.1 | GN360564.1 |
| GM625985.1 | GM656846.1 | DL096582.1 | DL033876.1 | CS118684.1 | HI987666.1 | DL109118.1 | FB676519.1 | GN360166.1 |
| GM625953.1 | GM670028.1 | DL096550.1 | DL033780.1 | CS118652.1 | HI987447.1 | DL104384.1 | FB701534.1 | GN360134.1 |
| GM038288.1 | GM656462.1 | DL094722.1 | DL033748.1 | CS118620.1 | HC194698.1 | DL104352.1 | FB701797.1 | GN360102.1 |
| FB775380.1 | GM656430.1 | DL094690.1 | DL033716.1 | CS118587.1 | FW512097.1 | DL104320.1 | FB292673.1 | GN356183.1 |
| FB774883.1 | GM649333.1 | DL090947.1 | DL042246.1 | CS118520.1 | FW554697.1 | DL089961.1 | FB660202.1 | GN366481.1 |
| FB766226.1 | GM649277.1 | DL090915.1 | DL042214.1 | CS118486.1 | FW561680.1 | DL123157.1 | FB292232.1 | GN366449.1 |
| FB765892.1 | GM649245.1 | DL086979.1 | DL042150.1 | CS118422.1 | FW510271.1 | DL123125.1 | FB665443.1 | GN366416.1 |
| FB764701.1 | GM649213.1 | DL086947.1 | DL042118.1 | CS118389.1 | HI401250.1 | DL114091.1 | FB654387.1 | GN366384.1 |
| FB764094.1 | GM635133.1 | DL086915.1 | DL042086.1 | CS118355.1 | HI377258.1 | DL114059.1 | FB654897.1 | GN366352.1 |
| FB746533.1 | GM635101.1 | DL105868.1 | DL033707.1 | CS118322.1 | HI375902.1 | DL126231.1 | DL202575.1 | GN360061.1 |
| FB744408.1 | GM635069.1 | DL105836.1 | CS057452.1 | CS118224.1 | HI371024.1 | DL126199.1 | DL188839.1 | GN366327.1 |
| FB743976.1 | GM723190.1 | DL105804.1 | CS056289.1 | CS118190.1 | HI001789.1 | DL126167.1 | DL193777.1 | GN366295.1 |
| FB743928.1 | GM840550.1 | DL096521.1 | CS055786.1 | CS118125.1 | HI001752.1 | DL122951.1 | FB571342.1 | GN366263.1 |
| FB743896.1 | GM040841.1 | DL096489.1 | CS053042.1 | CS118026.1 | HI469698.1 | DL122919.1 | FB572983.1 | GN359856.1 |
| FB743864.1 | GM040269.1 | DL096457.1 | CS052463.1 | CS117990.1 | HI575290.1 | DL114045.1 | CS696114.1 | GN359792.1 |
| FB743822.1 | CS728575.1 | DL115509.1 | CS052390.1 | CS117958.1 | HI001631.1 | DL113917.1 | CS696082.1 | GN359760.1 |
| FB743778.1 | CS728543.1 | DL115477.1 | CS052356.1 | CS111459.1 | HI001588.1 | DL113885.1 | DL023585.1 | GN359728.1 |
| FB761726.1 | CS727353.1 | DL115445.1 | CS052322.1 | CS106394.1 | HI001543.1 | DL113853.1 | DL035718.1 | GN359696.1 |
| FB761228.1 | CS727208.1 | DL105693.1 | CS050987.1 | AX194353.1 | HI003373.1 | DL109052.1 | DJ446696.1 | GN359676.1 |
| FB760331.1 | GM036645.1 | DL105661.1 | CS048829.1 | AX190434.1 | HI003335.1 | DL109020.1 | DJ436697.1 | GN359580.1 |
| GM952617.1 | FB665085.1 | DL105629.1 | CS038867.1 | AX180247.1 | HI464741.1 | DL108988.1 | DJ438375.1 | GN359548.1 |
| GM890113.1 | GM836382.1 | DL105597.1 | CS038826.1 | AX175097.1 | HI464709.1 | DL104286.1 | DJ433800.1 | GN359484.1 |
| GM010644.1 | GM685458.1 | DL029525.1 | CS027202.1 | AX173086.1 | HI464677.1 | DL104254.1 | DJ438300.1 | GN365478.1 |
| GM889431.1 | GM888091.1 | DL029493.1 | CS025542.1 | AX172461.1 | HI464645.1 | DL104222.1 | DJ438231.1 | GN359448.1 |
| GM889318.1 | GM879374.1 | DL029236.1 | CS023743.1 | AX167018.1 | HI550024.1 | DL099584.1 | DJ401280.1 | GN359416.1 |
| GM009369.1 | GM879313.1 | DL069376.1 | CS022578.1 | AY035209.1 | HI464449.1 | DL099552.1 | DJ417449.1 | GN359320.1 |
| GM008857.1 | GM059987.1 | DL049702.1 | CS018555.1 | AX146309.1 | HI504971.1 | DL015688.1 | DJ380837.1 | GN359288.1 |
| GM007130.1 | GM841816.1 | DL049670.1 | CS017169.1 | AX145722.1 | HI504435.1 | DL035403.1 | DJ389575.1 | GN350109.1 |
| GM867485.1 | GM841712.1 | DL045662.1 | CS016476.1 | AX145690.1 | HI462580.1 | DL035371.1 | DJ388710.1 | GN365339.1 |
| GM685453.1 | GM706924.1 | DL049629.1 | CS016119.1 | AX145658.1 | HH725147.1 | DL035339.1 | DD154870.1 | GN359279.1 |
| GM003573.1 | GM838960.1 | DL033300.1 | CQ986637.1 | AX145626.1 | HH713947.1 | DL023362.1 | DD152399.1 | GN033077.1 |
| DL116173.1 | GM602753.1 | DL033268.1 | CQ986603.1 | AX145594.1 | HH961364.1 | DL023330.1 | DD152112.1 | GN033013.1 |
| DL116141.1 | FB715272.1 | DL030713.1 | CQ986571.1 | AX145562.1 | AX242275.1 | DL023298.1 | DD139898.1 | GN032981.1 |
| DL116109.1 | FB676833.1 | DL030681.1 | CQ986537.1 | AX145530.1 | AX242243.1 | DL023266.1 | DD082058.1 | GN032949.1 |

| | | | | | | | | |
|---|---|---|---|---|---|---|---|---|
| DL116077.1 | GM061225.1 | DL026744.1 | CQ986505.1 | AX145498.1 | AX242083.1 | DL023202.1 | DD147802.1 | GN032917.1 |
| DL116045.1 | GM060731.1 | DL026712.1 | CQ983192.1 | AX145466.1 | AX242051.1 | DL019800.1 | DD147770.1 | GN032885.1 |
| DL120569.1 | DL262692.1 | DL026680.1 | CQ983111.1 | AX145434.1 | AX242019.1 | DL010747.1 | DD147738.1 | GN032853.1 |
| DL120537.1 | AX006185.1 | DL026648.1 | CQ982978.1 | AX145402.1 | AX241987.1 | DL010715.1 | DD147706.1 | GN032821.1 |
| DL115910.1 | Y09435.1 | DL026616.1 | CQ982909.1 | AX145369.1 | AX241955.1 | DL010683.1 | DD147674.1 | GN032789.1 |
| DL115878.1 | A57339.1 | DL019501.1 | CQ982840.1 | AX145337.1 | AX241923.1 | DJ433812.1 | DD139285.1 | GN032757.1 |
| DL115846.1 | A35729.1 | DL019469.1 | CQ982613.1 | AX145305.1 | AX241891.1 | DJ438345.1 | DD081039.1 | GN032726.1 |
| DL106456.1 | A34620.1 | DL019437.1 | CQ982191.1 | AX145273.1 | AX241859.1 | DJ438310.1 | DD132331.1 | GN032694.1 |
| DL106424.1 | A34015.1 | DL019405.1 | CQ975370.1 | AX145241.1 | AX241827.1 | DJ438278.1 | DD136988.1 | GN032662.1 |
| DL128470.1 | A33972.1 | DL026492.1 | CQ973141.1 | AX145209.1 | AX241763.1 | DJ402633.1 | DD135822.1 | GN032629.1 |
| DL128435.1 | A32461.1 | DL014506.1 | CQ977472.1 | AX145177.1 | AX241699.1 | DJ402601.1 | DD132313.1 | GN032597.1 |
| DL124677.1 | A30365.1 | DL014474.1 | CQ977099.1 | AX145145.1 | AX241667.1 | DJ402284.1 | DD064462.1 | GN032565.1 |
| DL124645.1 | A27335.1 | CS608350.1 | CQ976299.1 | AX145113.1 | AX241635.1 | DJ416207.1 | DD092359.1 | GN032534.1 |
| DL124613.1 | A13708.1 | CS607883.1 | CQ975976.1 | AX145081.1 | AX241603.1 | DJ400826.1 | DD090068.1 | GN032502.1 |
| DL124581.1 | A25930.1 | CS607661.1 | CQ975796.1 | AX145049.1 | AX241571.1 | DJ400794.1 | DD057922.1 | GN032470.1 |
| DL096916.1 | A14260.1 | CS607060.1 | CQ972466.1 | AX145017.1 | AX241539.1 | DJ391330.1 | DD057890.1 | GN032438.1 |
| DL096884.1 | A20091.1 | CS606963.1 | CQ972338.1 | AX144985.1 | AX241507.1 | DJ417459.1 | DD057858.1 | GN032406.1 |
| DL096852.1 | A18681.1 | CS606820.1 | CQ972306.1 | AX144953.1 | AX241475.1 | DJ380815.1 | DD057238.1 | GN032374.1 |
| DL096756.1 | A11190.1 | CS604851.1 | CQ971140.1 | AX144920.1 | AX241411.1 | DJ389466.1 | DD055735.1 | GN032341.1 |
| DL128107.1 | A09222.1 | CS592101.1 | CQ969083.1 | AX144888.1 | AX234651.1 | CS723809.1 | CS119814.1 | GN032309.1 |
| DL124469.1 | A08194.1 | CS594495.1 | CQ967770.1 | AX144856.1 | AX225280.1 | CS722219.1 | GM640040.1 | GN032277.1 |
| DL124437.1 | A00373.1 | CS593397.1 | CQ963572.1 | AX144824.1 | AX207304.1 | DJ357163.1 | GM627493.1 | GN032245.1 |
| DL124405.1 | A31788.1 | CS597717.1 | CQ957855.1 | AX144792.1 | AX203738.1 | DJ354276.1 | GM627429.1 | GN032213.1 |
| DL124373.1 | A31079.1 | CS585348.1 | CQ955919.1 | AX144760.1 | AX202565.1 | DD408545.1 | GM627397.1 | GN032181.1 |
| DL120284.1 | A30260.1 | CS584357.1 | CQ947452.1 | AX144728.1 | AX202436.1 | DD406768.1 | GM627365.1 | GN032149.1 |
| DL120252.1 | A29668.1 | CS583659.1 | CQ947129.1 | AX144696.1 | AX201610.1 | DD402426.1 | GM627333.1 | GN032117.1 |
| DL120220.1 | A28859.1 | CS575820.1 | CQ944199.1 | AX144664.1 | AX195204.1 | DD402394.1 | GM661313.1 | GN032085.1 |
| DL115817.1 | A27609.1 | CS575292.1 | CQ944071.1 | AX144632.1 | AX193970.1 | DD402330.1 | GM661267.1 | GN032053.1 |
| DL115785.1 | A25887.1 | CS573065.1 | CQ944039.1 | AX144395.1 | AX189528.1 | DD402298.1 | GM661235.1 | GN032021.1 |
| DL115753.1 | A26752.1 | CS576651.1 | CQ944007.1 | AX144137.1 | AF394911.1 | DD402234.1 | GM661203.1 | GN031989.1 |
| DL115721.1 | A26057.1 | CS570789.1 | CQ943975.1 | AX143943.1 | AX172950.1 | DD402202.1 | GM661171.1 | GN031957.1 |
| DL115689.1 | A24314.1 | CS549426.1 | CQ943943.1 | AX143687.1 | AX172311.1 | DD405614.1 | GM661139.1 | GN031925.1 |
| DL115657.1 | A24540.1 | CS459131.1 | CQ943911.1 | AX143559.1 | HV742514.1 | DD405582.1 | GM654145.1 | GN031893.1 |
| DL111231.1 | A14551.1 | DD361280.1 | CQ943879.1 | AX143495.1 | HV704133.1 | DD405550.1 | GM654113.1 | GN031861.1 |
| DL111199.1 | A21831.1 | DD367603.1 | CQ924873.1 | AX143367.1 | HV744630.1 | DD405486.1 | GM654081.1 | GN031829.1 |
| DL111167.1 | A21515.1 | DD363152.1 | CQ918567.1 | AX143239.1 | HV703146.1 | DD401984.1 | GM654049.1 | GN031796.1 |
| DL111103.1 | A20587.1 | DD362896.1 | CQ901656.1 | AX143047.1 | HV701286.1 | DD401923.1 | GM654017.1 | GN031732.1 |
| DL111071.1 | A20088.1 | DD368335.1 | CQ898788.1 | DD236117.1 | HV701128.1 | DD401859.1 | GM653985.1 | GN031699.1 |
| DL103757.1 | A20299.1 | DD361314.1 | CQ898653.1 | E41542.1 | HV708196.1 | DD401827.1 | GM646992.1 | GN031667.1 |
| DL099307.1 | A19275.1 | A10885.1 | CQ898557.1 | E41750.1 | HV708122.1 | DD401795.1 | GM646960.1 | GN031635.1 |
| DL093141.1 | A18720.1 | CS457213.1 | CQ898255.1 | E43914.1 | HV701077.1 | DD401619.1 | GM646928.1 | GN031603.1 |
| DL093077.1 | A17106.1 | CS456725.1 | CQ898102.1 | E37140.1 | HV701045.1 | DD401587.1 | GM646896.1 | GN031571.1 |
| A11908.1 | A16768.1 | CS456085.1 | CQ897031.1 | E35569.1 | HV695202.1 | DD401555.1 | GM646864.1 | GN031539.1 |
| A10360.1 | A05428.1 | CS450653.1 | CQ895646.1 | E33308.1 | HV694930.1 | DD401523.1 | GM646832.1 | GN031507.1 |
| A09219.1 | A14396.1 | DD360942.1 | CQ893710.1 | E06718.1 | HV694721.1 | DD401491.1 | GM639997.1 | GN031475.1 |
| A06517.1 | A13593.1 | DD357833.1 | CQ892577.1 | E04601.1 | HV693214.1 | DD401459.1 | GM639965.1 | GN031442.1 |
| A02228.1 | A12558.1 | DD357594.1 | CQ891352.1 | E04129.1 | HV302954.1 | CS479865.1 | GM639933.1 | GN031410.1 |

| | | | | | | | | |
|---|---|---|---|---|---|---|---|---|
| A08980.1 | A12269.1 | DD355841.1 | CQ890278.1 | E03613.1 | HV308937.1 | CS469265.1 | GM639901.1 | GN031378.1 |
| A07244.1 | A10451.1 | CS447660.1 | CQ889090.1 | E02969.1 | HV217029.1 | CS463949.1 | GM639869.1 | GN031346.1 |
| A06419.1 | A10090.1 | A19548.1 | CQ802136.1 | E02730.1 | HV194208.1 | CS464584.1 | GM639837.1 | GN031314.1 |
| A05985.1 | A08103.1 | A08012.1 | CQ802032.1 | E01741.1 | HV235475.1 | DD400149.1 | GM627295.1 | GN031281.1 |
| A04574.1 | A07725.1 | A08351.1 | CQ801230.1 | E01490.1 | HV190938.1 | DD394230.1 | GM627263.1 | GN031249.1 |
| A08996.1 | A06258.1 | CS438943.1 | CQ800798.1 | E01404.1 | HV190642.1 | DD023880.1 | GM627231.1 | GN031217.1 |
| A01785.1 | A04985.1 | CS436061.1 | CQ798431.1 | E00963.1 | FW420393.1 | DD020530.1 | GM627199.1 | GN031185.1 |
| A01661.1 | A04000.1 | DD350526.1 | CQ797562.1 | E00812.1 | FW420329.1 | DD017651.1 | GM627167.1 | GN031152.1 |
| A05173.1 | A08598.1 | DD347411.1 | CQ796753.1 | DD172708.1 | FW496532.1 | DD012857.1 | GM627135.1 | GN031120.1 |
| A04072.1 | HV988471.1 | DD331009.1 | CQ795493.1 | DD165167.1 | HI653997.1 | BD412633.1 | GM661119.1 | GN031088.1 |
| M22369.1 | HV961480.1 | DD349646.1 | CQ795459.1 | DD163903.1 | HI653907.1 | BD453858.1 | GM661087.1 | GN031056.1 |
| K03427.1 | HV964305.1 | CS427058.1 | CQ794578.1 | CS111754.1 | HI651105.1 | BD453826.1 | GM661055.1 | GN031024.1 |
| M13105.1 | HV959610.1 | CS425081.1 | CQ792369.1 | CS108626.1 | AY145516.1 | CS695799.1 | GM661023.1 | GN030992.1 |
| M60097.1 | HV964126.1 | CS423255.1 | CQ788207.1 | CS106390.1 | HH807101.1 | CS695735.1 | GM660991.1 | GN030961.1 |
| M19080.1 | HV959182.1 | CS421057.1 | CQ787490.1 | CS104883.1 | HH794729.1 | CS695703.1 | GM660959.1 | GN030897.1 |
| MH087225.1 | HV963582.1 | CS417332.1 | CQ787458.1 | CS103265.1 | HH777930.1 | CS695479.1 | GM660927.1 | GN030865.1 |
| M55164.1 | HV963337.1 | BD274847.1 | CQ787420.1 | CS102966.1 | HH819337.1 | DL099580.1 | GM653933.1 | GN030833.1 |
| M34506.1 | HV966002.1 | CS603031.1 | CQ787380.1 | CS102902.1 | HH998686.1 | DL099548.1 | GM653901.1 | GN030801.1 |
| KX431573.1 | HV969947.1 | CS602999.1 | CQ787346.1 | CS102774.1 | HH998624.1 | DL122739.1 | GM653869.1 | GN030769.1 |
| K02249.1 | HV965329.1 | CS602965.1 | CQ787310.1 | CS102742.1 | HH996935.1 | DL122683.1 | GM653837.1 | DM002449.1 |
| M11991.1 | HV961704.1 | CS602933.1 | CQ787278.1 | CS102678.1 | HH996903.1 | DL122651.1 | GM653773.1 | DM002283.1 |
| DQ250240.1 | HV951613.1 | CS602677.1 | CQ787246.1 | CS102646.1 | HH996782.1 | DL122587.1 | GM646764.1 | DM008094.1 |
| DQ250176.1 | HV951581.1 | CS611261.1 | CQ786942.1 | CS102582.1 | HH994642.1 | DL113809.1 | GM646732.1 | DM007532.1 |
| AY569326.1 | HV951549.1 | CS608345.1 | CQ784671.1 | CS102550.1 | HH998519.1 | DL099476.1 | GM646700.1 | DM001480.1 |
| AF336117.1 | HV951168.1 | CS607054.1 | CQ784476.1 | CS102518.1 | HH980613.1 | DL144344.1 | GM646668.1 | DM007032.1 |
| AF105014.1 | HV950617.1 | CS606987.1 | CQ779571.1 | CS102486.1 | HH980509.1 | DL113537.1 | GM639833.1 | DM006880.1 |
| S71747.1 | HV947312.1 | CS606961.1 | CQ778906.1 | CS088909.1 | HH980441.1 | DL035296.1 | GM639801.1 | DM000910.1 |
| AF363774.1 | HV575310.1 | DD087680.1 | CQ775356.1 | CS086371.1 | HH980336.1 | DL010594.1 | GM639769.1 | DM005044.1 |
| HW390750.1 | HV574223.1 | DD069500.1 | CQ772727.1 | CS085660.1 | HH980171.1 | DL010530.1 | GM639737.1 | DM004974.1 |
| HW399432.1 | HV568189.1 | DD157373.1 | GM646527.1 | CS082949.1 | HH980136.1 | DL010498.1 | GM639705.1 | DM003507.1 |
| HW399323.1 | HV566137.1 | DD156974.1 | GM646495.1 | CS079779.1 | HH979891.1 | DL010466.1 | GM639673.1 | DL489748.1 |
| HW408999.1 | HV559784.1 | DD155699.1 | GM646463.1 | CS077858.1 | HH996735.1 | DL010434.1 | GM639641.1 | DM003144.1 |
| HW390413.1 | HV558835.1 | DD097290.1 | GM646431.1 | AY967404.1 | HH996660.1 | DJ436709.1 | GM627062.1 | DM003108.1 |
| HW389669.1 | HV562073.1 | DD083029.1 | GM639532.1 | AY967372.1 | HH996620.1 | DJ439035.1 | GM627054.1 | GM625203.1 |
| HW389565.1 | FW570907.1 | DD082080.1 | GM639446.1 | AY967340.1 | HH996558.1 | DJ433808.1 | GM626998.1 | GM634377.1 |
| HW115615.1 | HV552784.1 | DD139586.1 | GM639492.1 | AY967308.1 | HH998471.1 | DJ438341.1 | GM626966.1 | GM634345.1 |
| HW115549.1 | HV550342.1 | DD147811.1 | GM626889.1 | A35246.1 | HH998377.1 | DJ438306.1 | GM626934.1 | GM634313.1 |
| HW101688.1 | AY659393.1 | DD147779.1 | GM626857.1 | A34184.1 | HH998344.1 | DJ402665.1 | DL183248.1 | GM634281.1 |
| HW087328.1 | HV339658.1 | DD147747.1 | GM626825.1 | AX443266.1 | HH999968.1 | DJ400790.1 | FB571338.1 | GM629443.1 |
| HW097013.1 | HV341788.1 | DD147715.1 | GM653640.1 | A25439.1 | HH999913.1 | DJ398704.1 | FB570345.1 | GM629412.1 |
| HW105140.1 | HV322789.1 | DD147683.1 | GM653608.1 | AX429294.1 | HH979831.1 | DJ391326.1 | CS696142.1 | GM629380.1 |
| HW096455.1 | HV321737.1 | DD122488.1 | GM653576.1 | AX428414.1 | HH979765.1 | DJ417455.1 | CS696110.1 | GM629348.1 |
| HW072719.1 | HV333649.1 | DD138527.1 | GM660773.1 | AX427256.1 | HH999877.1 | DJ380843.1 | CS696078.1 | GM629316.1 |
| HW072666.1 | HV187402.1 | DD132335.1 | GM660741.1 | AX427159.1 | HH999788.1 | DJ380811.1 | CS696013.1 | GM619879.1 |
| HW096217.1 | HV208799.1 | DD136026.1 | GM653522.1 | AX418271.1 | HH999734.1 | DJ389439.1 | CS695917.1 | GM655604.1 |
| HW072446.1 | HV218084.1 | DD106551.1 | DL117255.1 | AX417903.1 | HH998306.1 | DJ388718.1 | CS695885.1 | GM655572.1 |
| HW072224.1 | HV245315.1 | DD092936.1 | DL117223.1 | AX404703.1 | HH998257.1 | CS724444.1 | CS695853.1 | GM648579.1 |

| | | | | | | | | |
|---|---|---|---|---|---|---|---|---|
| HW071720.1 | HV302964.1 | DD104660.1 | DL091642.1 | AX402414.1 | HH998220.1 | CS722091.1 | CS695821.1 | GM648547.1 |
| HW104303.1 | HV302932.1 | DD160151.1 | DL091610.1 | AX399446.1 | HH998168.1 | DJ358848.1 | CS695789.1 | GM648515.1 |
| HW070978.1 | HV312624.1 | DD170657.1 | DL091578.1 | AX397874.1 | HH996493.1 | DJ357669.1 | CS695757.1 | GM648451.1 |
| HW070605.1 | HV305821.1 | DD176019.1 | DL087967.1 | AX392370.1 | HH986614.1 | BD265652.1 | CS695725.1 | GM648419.1 |
| HW069635.1 | HV308611.1 | CS108630.1 | DL087935.1 | AX384568.1 | HW257267.1 | BD265600.1 | CS695661.1 | GM641616.1 |
| HW069221.1 | HI931498.1 | CS106392.1 | DL087903.1 | AX380805.1 | HW257235.1 | BD263859.1 | CS695629.1 | GM641584.1 |
| HW102279.1 | HI930287.1 | CS104885.1 | DL087871.1 | AX375508.1 | HW257203.1 | BD263440.1 | CS695501.1 | GM641552.1 |
| HW067347.1 | HI918254.1 | CS104224.1 | DL087839.1 | AX370284.1 | HW257171.1 | BD263059.1 | CS695469.1 | GM641520.1 |
| HW066622.1 | HI660690.1 | CS103270.1 | DL087807.1 | AX364552.1 | HW257139.1 | BD261179.1 | CS695437.1 | GM641488.1 |
| HW083260.1 | BD453556.1 | CS102968.1 | DL121766.1 | AX364520.1 | HW257107.1 | BD251848.1 | CS695405.1 | GM641456.1 |
| HW097599.1 | BD453524.1 | CS102936.1 | DL121734.1 | AX364488.1 | HW257043.1 | BD250961.1 | CS695339.1 | GM641424.1 |
| HW088264.1 | BD453492.1 | CS102744.1 | DL121702.1 | AX364456.1 | HW257011.1 | BD249688.1 | CS695307.1 | GM629271.1 |
| HW087869.1 | BD453460.1 | CS102712.1 | DL121670.1 | AX364424.1 | HW256947.1 | BD249000.1 | CS791244.1 | GM629239.1 |
| HW081771.1 | BD453428.1 | CS102680.1 | DL121638.1 | AX364392.1 | HW256915.1 | BD248124.1 | DL176531.1 | GM629143.1 |
| HW081687.1 | BD453396.1 | CS102648.1 | DL121606.1 | AX364360.1 | HW256851.1 | BD247032.1 | DL176463.1 | GM629111.1 |
| HW081615.1 | BD453251.1 | CS102616.1 | DL087747.1 | AX364168.1 | HW256755.1 | BD245157.1 | DL176306.1 | GM634041.1 |
| M35281.1 | BD453219.1 | CS102584.1 | DL087715.1 | BD008861.1 | HW254452.1 | BD244541.1 | DL175867.1 | GM634009.1 |
| M60347.1 | BD453148.1 | CS102552.1 | DL087683.1 | E50435.1 | HW251317.1 | BD243795.1 | DL181493.1 | GM633977.1 |
| M12798.1 | BD452218.1 | CS102520.1 | DL087651.1 | E59058.1 | HW251082.1 | BD243427.1 | DL181438.1 | GM633945.1 |
| HV549722.1 | BD493662.1 | CS102488.1 | DL087619.1 | BD002024.1 | HW251026.1 | BD243141.1 | DL174594.1 | GM633913.1 |
| HV549395.1 | BD409233.1 | CS085662.1 | DL087587.1 | E50757.1 | HW250351.1 | BD242445.1 | DL176800.1 | GM633881.1 |
| HV549077.1 | BD451754.1 | CS082067.1 | DL125518.1 | E59810.1 | HW249720.1 | BD238171.1 | DL176720.1 | GM883254.1 |
| HV544279.1 | BD451712.1 | CS079782.1 | DL125454.1 | AX355999.1 | HW247849.1 | BD236961.1 | FB355680.1 | GM662854.1 |
| HV543985.1 | BD450243.1 | CS078852.1 | DL125422.1 | AX354613.1 | HW240939.1 | BD235924.1 | FB357051.1 | GM655522.1 |
| HV547838.1 | BD408023.1 | CS072092.1 | DL125390.1 | AX351515.1 | HW240907.1 | BD235728.1 | FB360900.1 | GM655490.1 |
| HV543882.1 | BD429587.1 | CS068624.1 | DL125358.1 | AX347310.1 | HW240875.1 | BD235445.1 | FB344292.1 | GM655458.1 |
| HV543491.1 | BD429477.1 | CS063742.1 | DL121537.1 | AX347236.1 | HW240843.1 | BD234799.1 | DL030391.1 | GM655426.1 |
| HV546998.1 | BD407580.1 | AY967406.1 | DL121505.1 | AX347166.1 | HW240811.1 | BD234402.1 | DL030351.1 | GM655394.1 |
| HV539590.1 | BD397231.1 | AY967374.1 | DL121473.1 | AX346934.1 | HW240779.1 | BD231153.1 | DL026520.1 | GM655362.1 |
| HV538575.1 | BD439111.1 | AY967342.1 | DL121441.1 | HW262985.1 | HW240747.1 | BD230083.1 | DL026534.1 | GM648369.1 |
| HV538407.1 | BD428734.1 | AY967310.1 | DL121409.1 | HW262950.1 | HW240723.1 | BD226837.1 | DL026478.1 | GM648337.1 |
| HV543340.1 | AX699449.1 | AY967278.1 | DL117197.1 | HW262842.1 | HW240659.1 | BD225384.1 | DL026446.1 | GM648305.1 |
| HV542745.1 | AX699417.1 | AY967246.1 | DL117165.1 | HW262600.1 | HW239410.1 | BD224581.1 | DL026414.1 | GM648273.1 |
| HV542176.1 | AX683828.1 | AY967214.1 | DL117133.1 | HW262562.1 | HW238414.1 | BD223237.1 | DL026382.1 | GM648241.1 |
| HV533480.1 | AX662194.1 | AY967182.1 | DL117101.1 | HW261396.1 | HW238192.1 | BD222641.1 | DL022570.1 | GM648209.1 |
| HV515140.1 | AX659091.1 | AY967150.1 | DL117069.1 | HW261364.1 | HW238010.1 | BD218033.1 | DL022538.1 | GM641406.1 |
| HV514899.1 | AX658024.1 | AY967118.1 | DL117037.1 | HW261332.1 | HW244286.1 | BD211175.1 | DL022458.1 | GM641374.1 |
| HV514655.1 | AX657131.1 | AX203098.1 | DL112417.1 | HW261268.1 | HW237913.1 | BD206112.1 | DL022498.1 | GM641342.1 |
| HV532750.1 | AX657099.1 | AX202482.1 | DL112385.1 | HW261236.1 | HW237869.1 | BD204767.1 | DL022442.1 | GM641310.1 |
| HV536507.1 | BD176064.1 | AX193994.1 | DL112353.1 | HW261172.1 | HW237693.1 | DD283778.1 | DL022410.1 | GM641278.1 |
| HV534609.1 | AX642595.1 | AX190429.1 | DL112321.1 | HW261108.1 | HW237432.1 | DD283205.1 | DL019395.1 | GM641246.1 |
| HV532088.1 | AX615141.1 | AX180722.1 | DL112289.1 | HW261076.1 | HW242325.1 | DD282675.1 | DL019363.1 | GM629087.1 |
| HV530223.1 | BD171822.1 | AX175078.1 | DL107275.1 | HW261044.1 | HW242293.1 | CQ967264.1 | DL019331.1 | GM629055.1 |
| HV516062.1 | BD171401.1 | AX173084.1 | DL107243.1 | DD405140.1 | HW242261.1 | CQ958007.1 | DL019299.1 | GM629023.1 |
| HV515736.1 | AX601511.1 | AX172744.1 | DL107211.1 | DD405108.1 | HW241741.1 | CQ956061.1 | DL019267.1 | GM628991.1 |
| HV515580.1 | AX601375.1 | AX172457.1 | DL107179.1 | DD404559.1 | HW241545.1 | CQ944181.1 | DL019235.1 | GM628959.1 |
| HV515548.1 | AX601343.1 | AF316373.1 | DL107147.1 | DD402419.1 | HW243152.1 | CQ944149.1 | DL019203.1 | GM628927.1 |

| | | | | | | | | |
|---|---|---|---|---|---|---|---|---|
| HV515516.1 | AX600856.1 | AX153893.1 | DL107115.1 | DD402387.1 | HW241339.1 | CQ944117.1 | DL014800.1 | GM655329.1 |
| CS727318.1 | AX598959.1 | AY035207.1 | DL102502.1 | E02271.1 | HV752625.1 | CQ944085.1 | DL014768.1 | GM655297.1 |
| CS727217.1 | AX597698.1 | AX146307.1 | DL102470.1 | E01957.1 | HV755100.1 | CQ944053.1 | DL014736.1 | GM655265.1 |
| GM036650.1 | AX594163.1 | AX145720.1 | DL102438.1 | E01663.1 | HV755036.1 | CQ944021.1 | DL014704.1 | GM655201.1 |
| GM836389.1 | AX593505.1 | AX145688.1 | DL102406.1 | E01486.1 | HV749740.1 | CQ943989.1 | DL014657.1 | GM655169.1 |
| GM888539.1 | BD166157.1 | AX145624.1 | DL102374.1 | E01399.1 | HV748945.1 | CQ943957.1 | DL010182.1 | GM648176.1 |
| GM007172.1 | AX589484.1 | AX145560.1 | DL102342.1 | E00954.1 | HV748489.1 | CQ943925.1 | DL010150.1 | GM648144.1 |
| GM685463.1 | BD144212.1 | AX145528.1 | DL097697.1 | E00265.1 | HV748405.1 | CQ943893.1 | DL010118.1 | GM840660.1 |
| FB728423.1 | AX587734.1 | AX145464.1 | DL097633.1 | DD088123.1 | HV750786.1 | CQ918245.1 | DL010086.1 | FB709678.1 |
| GM680776.1 | AX587632.1 | AX145432.1 | DL097569.1 | DD069491.1 | HV753489.1 | CQ898817.1 | DL010054.1 | GM877912.1 |
| GM879318.1 | AX576409.1 | AX145367.1 | DL097537.1 | DD154003.1 | HV743903.1 | CQ898667.1 | DL010022.1 | CS728624.1 |
| GM680727.1 | AX107114.1 | AX145335.1 | DL095510.1 | DD154874.1 | HV743870.1 | CQ898635.1 | DL019183.1 | CS728592.1 |
| GM618700.1 | AX098685.1 | AX145303.1 | DL095478.1 | DD152115.1 | HV743838.1 | CQ898571.1 | DL019151.1 | CS728560.1 |
| GM721280.1 | AX097927.1 | AX145271.1 | DL095446.1 | DD082061.1 | HV743806.1 | CQ895880.1 | DL019087.1 | CS727590.1 |
| GM060280.1 | AX097504.1 | AX145239.1 | DL095414.1 | DD147805.1 | HV743276.1 | CQ895545.1 | DL019055.1 | CS727436.1 |
| GM841717.1 | AX093084.1 | AX145207.1 | DL095382.1 | DD147773.1 | HV743238.1 | CQ890891.1 | DL019023.1 | CS727336.1 |
| GM604027.1 | AX089415.1 | AX145175.1 | DL095350.1 | DD147741.1 | HV742500.1 | CQ888105.1 | DL014620.1 | FB708987.1 |
| GM603689.1 | AX085319.1 | AX145143.1 | DL091472.1 | DD147709.1 | HV704952.1 | CQ887754.1 | DL014588.1 | GM719140.1 |
| GM706609.1 | AX077845.1 | AX145111.1 | DL091440.1 | DD147677.1 | HV704454.1 | AX720725.1 | DL014556.1 | GM011208.1 |
| DL111197.1 | AX061420.1 | AX145079.1 | DL091408.1 | DD139309.1 | HV744599.1 | AX717717.1 | DL014492.1 | GM888672.1 |
| DL111165.1 | AX068062.1 | AX145047.1 | DL091376.1 | DD081075.1 | HV703533.1 | AX712110.1 | DL014460.1 | GM887787.1 |
| DL106094.1 | AX058592.1 | AX145015.1 | DL087483.1 | DD137353.1 | HV703143.1 | AX708729.1 | DL010002.1 | FB662075.1 |
| DL106062.1 | AX052939.1 | AX144983.1 | DL087451.1 | DD137167.1 | HV702479.1 | AX705314.1 | DL009970.1 | GM869156.1 |
| DL106030.1 | AX040757.1 | CS424099.1 | DL087419.1 | DD132316.1 | HV702367.1 | AX685818.1 | DL009938.1 | GM867513.1 |
| DL105998.1 | AX040173.1 | CS417742.1 | DL112261.1 | DD092992.1 | HV702265.1 | AX670738.1 | DL009906.1 | GM683098.1 |
| DL105966.1 | AX037314.1 | CS417329.1 | DL112229.1 | CS070722.1 | HV702233.1 | AX657963.1 | DL009874.1 | GM680788.1 |
| DL105934.1 | AX033574.1 | CS416937.1 | DL112197.1 | CS059820.1 | HV701283.1 | AX662193.1 | DL009842.1 | GM879359.1 |
| DL105902.1 | AX032763.1 | CS410935.1 | DL112165.1 | AY967400.1 | HV701221.1 | AX659090.1 | DL009778.1 | FB728342.1 |
| DL101193.1 | AX028794.1 | CS410883.1 | DL112133.1 | AY967368.1 | HV453667.1 | AX658018.1 | DL009746.1 | GM842470.1 |
| DL101161.1 | AX026713.1 | CS410602.1 | DL112101.1 | AY967336.1 | HV449721.1 | AX657130.1 | DL009714.1 | FB722575.1 |
| DL101129.1 | AX025185.1 | CS414823.1 | DL112069.1 | AY967304.1 | HV453038.1 | AX657098.1 | DL009682.1 | GM863433.1 |
| DL096715.1 | AX023687.1 | DD327643.1 | DL107066.1 | AY967272.1 | HV445658.1 | BD175890.1 | DL009650.1 | GM863142.1 |
| DL096683.1 | AX023638.1 | DD328870.1 | DL099693.1 | AY967240.1 | HV451133.1 | BD175064.1 | DL009618.1 | GM841833.1 |
| DL096651.1 | AX023572.1 | CS389301.1 | DL099629.1 | AY967208.1 | HV436998.1 | AX642592.1 | DL009586.1 | GM841797.1 |
| DL096619.1 | AX019326.1 | CS389259.1 | DL099565.1 | AY967176.1 | HV436966.1 | BD171821.1 | DL009554.1 | GM841765.1 |
| DL096587.1 | AX018720.1 | CS389213.1 | DD165511.1 | AY967144.1 | HV436895.1 | AF411477.1 | DL009522.1 | FB742688.1 |
| DL096555.1 | AX014769.1 | CS389175.1 | DD165151.1 | AY967112.1 | HV436863.1 | AX212279.1 | DL009490.1 | GM603722.1 |
| DL094727.1 | AX010927.1 | CS399237.1 | DD171163.1 | AY967080.1 | HV444633.1 | AX207615.1 | DL009458.1 | GM706723.1 |
| DL094695.1 | HW338418.1 | CS401718.1 | DD159559.1 | AY967048.1 | HV444349.1 | AX207118.1 | DL009426.1 | GM706659.1 |
| DL094663.1 | HW338162.1 | CS401402.1 | DD182185.1 | AY967016.1 | HV438124.1 | AX205060.1 | DL026342.1 | GM706627.1 |
| DL090952.1 | HW338034.1 | CS406379.1 | DD181786.1 | AY966984.1 | HV443181.1 | AX202476.1 | DL026246.1 | GM970190.1 |
| DL090920.1 | HW337906.1 | AX937794.1 | DD175952.1 | AY966952.1 | HV443092.1 | AX195443.1 | DL026214.1 | FB736414.1 |
| DL086984.1 | HW337650.1 | AX934709.1 | DD175919.1 | AY966920.1 | HV437556.1 | AX193984.1 | DL026182.1 | GM969860.1 |
| DL086952.1 | HW337522.1 | AX925499.1 | CS001489.1 | CS055259.1 | HV437312.1 | AX191690.1 | DL018992.1 | GM969741.1 |
| DL086920.1 | HW337266.1 | AX923394.1 | AF408182.1 | CS052522.1 | HV349090.1 | AX180594.1 | DL018960.1 | FB777912.1 |
| DL086888.1 | HW337138.1 | AX840258.1 | CS157801.1 | CS052402.1 | HV351600.1 | AX179679.1 | DL018928.1 | DL480007.1 |
| DL086856.1 | HW336882.1 | AX839726.1 | CS157962.1 | CS052300.1 | HV344162.1 | AX179258.1 | DL018896.1 | DL479434.1 |

| | | | | | | | | |
|---|---|---|---|---|---|---|---|---|
| DL086824.1 | HW336598.1 | AX838389.1 | CS157930.1 | CS051044.1 | HV343934.1 | AX174842.1 | DL018864.1 | DL465763.1 |
| DL086792.1 | HW329489.1 | AX824358.1 | CS157898.1 | CS050714.1 | HV340906.1 | AX173080.1 | DL018800.1 | DL477848.1 |
| DL086760.1 | HW329404.1 | AX823792.1 | CS157866.1 | CS048042.1 | HV338403.1 | AX172441.1 | DL034467.1 | DL470951.1 |
| DL086728.1 | HW329284.1 | AX816150.1 | CS146883.1 | CS038907.1 | HV347789.1 | AX167809.1 | DL034435.1 | DL477019.1 |
| DL086696.1 | HW328839.1 | AX814428.1 | CS144059.1 | CS024303.1 | HV340621.1 | AF385772.1 | DL034403.1 | DL462985.1 |
| DL086664.1 | HW328546.1 | AX813411.1 | CS126614.1 | AX347268.1 | HV347723.1 | AX153820.1 | DL034371.1 | DL476034.1 |
| DL105873.1 | HW328424.1 | AX805918.1 | CS124723.1 | AX347232.1 | HV347378.1 | AF384425.1 | DL034339.1 | DL481979.1 |
| DL105841.1 | HW328313.1 | AX800140.1 | CS124685.1 | AX347198.1 | HV344926.1 | AX148380.1 | DL034307.1 | FB504545.1 |
| DL105809.1 | HW328264.1 | AX799562.1 | CS124645.1 | AX347162.1 | HV347322.1 | AX146858.1 | DL014403.1 | GM712142.1 |
| DL096526.1 | HW328151.1 | AX798985.1 | CS119548.1 | AX346790.1 | HV339995.1 | AX146303.1 | DL014371.1 | GM651996.1 |
| DL096494.1 | HW318448.1 | AX798169.1 | CS119482.1 | AX344906.1 | DL111934.1 | AX145716.1 | DL014339.1 | GM651964.1 |
| DL096462.1 | HW314142.1 | AX797691.1 | CQ830727.1 | AX329474.1 | DL111902.1 | AX145652.1 | DL014307.1 | GM651932.1 |
| DL038423.1 | HW314001.1 | AX472471.1 | CQ827555.1 | AX328971.1 | DL111890.1 | AX145620.1 | DL014275.1 | GM678493.1 |
| DL038391.1 | HW311235.1 | AX787402.1 | CQ827523.1 | AX328146.1 | DL106867.1 | AX145588.1 | DL014243.1 | GM831281.1 |
| DL038359.1 | HW308549.1 | AX787343.1 | CQ826861.1 | AX306619.1 | DL106835.1 | AX145556.1 | DL009391.1 | GM831178.1 |
| DL013164.1 | HC305682.1 | AX781459.1 | CQ824427.1 | AX259249.1 | DL106803.1 | AX145524.1 | DL009359.1 | GM711476.1 |
| DL013132.1 | HC305642.1 | AX773268.1 | CQ821297.1 | AX300702.1 | DL106771.1 | AX145492.1 | DL009327.1 | FB509366.1 |
| DL013100.1 | HC301946.1 | AX766563.1 | CQ818568.1 | AX278250.1 | DL106739.1 | AX145460.1 | DL009295.1 | FB509330.1 |
| DL013068.1 | HC305037.1 | AX766168.1 | CQ817736.1 | AX255040.1 | DL106707.1 | AX145428.1 | DL009263.1 | FB509298.1 |
| DL013036.1 | HC294194.1 | AX212289.1 | CQ816980.1 | AX253428.1 | DL091341.1 | AX145396.1 | CS603754.1 | FB509266.1 |
| DL033739.1 | HC291342.1 | AX205064.1 | CQ816939.1 | AX250311.1 | DL091277.1 | AX145363.1 | CS603690.1 | GM675063.1 |
| DL042141.1 | HC289378.1 | AX203096.1 | CQ815750.1 | AX242280.1 | DL091213.1 | AX145331.1 | CS603466.1 | FB509219.1 |
| DL042109.1 | HC289334.1 | AX193988.1 | CQ814988.1 | AX242248.1 | DL091181.1 | AX145299.1 | CS603434.1 | FB509155.1 |
| DL042077.1 | HC292876.1 | AX191715.1 | CQ814098.1 | AX242184.1 | DL087373.1 | AX145267.1 | CS603402.1 | FB509117.1 |
| DL029530.1 | HC292744.1 | AX189820.1 | CQ814066.1 | AX242152.1 | DL087341.1 | AX145235.1 | CS603370.1 | GM674174.1 |
| DL029498.1 | HC288855.1 | AX179712.1 | CQ814033.1 | AX242120.1 | DL087309.1 | AX145203.1 | CS603338.1 | FB508990.1 |
| DL029466.1 | HC295620.1 | AX174862.1 | CQ814001.1 | AX242088.1 | DL087277.1 | AX145171.1 | CS603306.1 | FB508958.1 |
| DL038082.1 | HC295540.1 | AX173082.1 | CQ813969.1 | AX242024.1 | DL087245.1 | AX145139.1 | CS603274.1 | GM773244.1 |
| DL046196.1 | HC292446.1 | AX172509.1 | CQ813937.1 | AX241992.1 | DL087213.1 | AX145107.1 | CS603242.1 | GM709020.1 |
| DL046100.1 | HC292206.1 | AX172443.1 | CQ813905.1 | AX241960.1 | DL102106.1 | AX145075.1 | CS603210.1 | GM708988.1 |
| DL041922.1 | HC292194.1 | AF316371.1 | CQ813873.1 | AX241896.1 | DL102074.1 | AX145043.1 | CS603178.1 | GM657912.1 |
| DL041890.1 | HC294768.1 | AX153868.1 | CQ813841.1 | AX241864.1 | DL102042.1 | AX145011.1 | CS603146.1 | GM657848.1 |
| DL045873.1 | HC291762.1 | AY034053.1 | CQ813809.1 | AX241832.1 | DL102010.1 | AX144979.1 | DL049373.1 | GM657816.1 |
| DL049707.1 | HC294540.1 | AX146305.1 | CQ813490.1 | AX241800.1 | DL101978.1 | AX144947.1 | DL049341.1 | GM657783.1 |
| DL049675.1 | HC293966.1 | AX145718.1 | CQ809728.1 | AX241736.1 | DL101946.1 | AX144914.1 | DL049309.1 | GM657751.1 |
| DL023172.1 | HC293409.1 | AX145686.1 | CQ803007.1 | AX241672.1 | DL097532.1 | AX144882.1 | DL049277.1 | GM643392.1 |
| DL023140.1 | HC293289.1 | AX145654.1 | CQ802135.1 | AX241640.1 | DL097500.1 | AX144850.1 | DL049245.1 | GM643360.1 |
| DL023108.1 | HC293092.1 | AX145558.1 | CQ800797.1 | AX241608.1 | DL097468.1 | AX144818.1 | DL049214.1 | GM643328.1 |
| DL023076.1 | HC289862.1 | AX145526.1 | CQ798426.1 | AX241576.1 | DL097436.1 | AX144786.1 | DL049182.1 | GM643296.1 |
| DL023044.1 | HC289679.1 | AX145494.1 | CQ796858.1 | AX241480.1 | DL097404.1 | AX144754.1 | DL049118.1 | GM643264.1 |
| DL023012.1 | HC207398.1 | AX145462.1 | CQ796752.1 | AX241448.1 | DL097372.1 | AX144722.1 | DL010604.1 | GM643232.1 |
| DL022980.1 | DM026461.1 | AX145430.1 | CQ795491.1 | AX241416.1 | DL097340.1 | AX144690.1 | DL047028.1 | GM636243.1 |
| DL010752.1 | DM026235.1 | AX145398.1 | CQ795458.1 | HV957541.1 | DL121295.1 | AX144658.1 | DL046964.1 | GM636211.1 |
| DL010720.1 | GN041498.1 | AX145365.1 | CQ794563.1 | HV945092.1 | DL121231.1 | AX144626.1 | DL046932.1 | GM636179.1 |
| DL010688.1 | GN039771.1 | AX145333.1 | CQ793265.1 | HV764391.1 | DL121199.1 | AX144511.1 | DL046900.1 | GM636147.1 |
| DL042938.1 | GN034807.1 | AX145301.1 | CQ789544.1 | HV775597.1 | DL101893.1 | AX144383.1 | DL046868.1 | GM636115.1 |
| DL042906.1 | GN030731.1 | AX145269.1 | CQ788206.1 | HW302512.1 | DL101833.1 | AX144255.1 | DL043046.1 | GM636083.1 |

| | | | | | | | | |
|---|---|---|---|---|---|---|---|---|
| DL042874.1 | GN030699.1 | AX145237.1 | CQ787621.1 | HW295730.1 | DL101797.1 | AX144125.1 | DL043014.1 | GM631675.1 |
| DL038993.1 | GN030603.1 | AX145205.1 | CQ787489.1 | HW295688.1 | DL101765.1 | AX143867.1 | DL042982.1 | GM631643.1 |
| DL038961.1 | GN030571.1 | AX145173.1 | CQ787457.1 | HC010409.1 | DL101733.1 | AX143803.1 | DL042950.1 | GM631611.1 |
| DL038929.1 | GN030507.1 | AX145141.1 | CQ787419.1 | HC007888.1 | DL097319.1 | AX143739.1 | DL042918.1 | GM631579.1 |
| DL027108.1 | GN030397.1 | AX145109.1 | CQ787379.1 | HB385995.1 | DL097287.1 | AX143675.1 | DL042886.1 | GM631547.1 |
| DL027044.1 | GN030347.1 | AX145077.1 | CQ787345.1 | HB340004.1 | DL097255.1 | AX143611.1 | DL039069.1 | GM631515.1 |
| DL027012.1 | GN030315.1 | AX145045.1 | CQ787309.1 | HA641576.1 | DL097223.1 | AX143291.1 | DL039037.1 | GM631483.1 |
| GN031105.1 | GN030283.1 | AX145013.1 | CQ787277.1 | HA641445.1 | DL097191.1 | HV749319.1 | DL039005.1 | GM622712.1 |
| GN031073.1 | GN030251.1 | AX144981.1 | CQ787245.1 | HA641175.1 | DL097159.1 | HV747489.1 | DL038973.1 | GM622680.1 |
| GN031041.1 | GN029899.1 | AX144949.1 | CQ786941.1 | HA641111.1 | DL095331.1 | HV753515.1 | DL038941.1 | GM622648.1 |
| GN031009.1 | GN029867.1 | AX144916.1 | CQ784631.1 | HA635499.1 | DL095299.1 | HV743329.1 | DL038909.1 | GM622616.1 |
| GN030977.1 | GN013585.1 | AX144884.1 | CQ784669.1 | HA639547.1 | DL095235.1 | HV742533.1 | DL035285.1 | GM622584.1 |
| GN030946.1 | GN033535.1 | AX144852.1 | CQ784475.1 | HA638529.1 | DL095203.1 | HV704234.1 | DL035253.1 | GM622552.1 |
| GN030882.1 | GN033503.1 | AX144820.1 | CQ779570.1 | HA643887.1 | DL095171.1 | HV703881.1 | DL035221.1 | GM740794.1 |
| GN030850.1 | GN033471.1 | AX144788.1 | CQ778507.1 | DM092694.1 | DL125338.1 | HV744554.1 | DL035189.1 | GM704184.1 |
| GN030818.1 | GN033439.1 | AX144756.1 | CQ775038.1 | GN368272.1 | DL125306.1 | HV703155.1 | DL035157.1 | GM657698.1 |
| DM008344.1 | GN033375.1 | AX144724.1 | CQ771661.1 | GN360109.1 | DL125274.1 | HV701231.1 | DL035125.1 | GM657666.1 |
| DM001671.1 | GN033343.1 | AX144692.1 | CQ771629.1 | GN356190.1 | DL125242.1 | HV701199.1 | DL031121.1 | GM657634.1 |
| DM001614.1 | GN033311.1 | AX144660.1 | CQ771597.1 | GN067945.1 | DL125210.1 | HV701167.1 | DL031089.1 | GM657602.1 |
| DM006941.1 | GN033279.1 | AX144628.1 | AX278755.1 | GN067938.1 | DL125178.1 | HV701135.1 | DL031025.1 | GM657570.1 |
| DM000512.1 | GN033247.1 | AX144515.1 | AX278089.1 | GN067874.1 | DL095143.1 | HV701052.1 | DL030993.1 | GM650378.1 |
| DM003492.1 | GN033119.1 | AX144193.1 | AX256339.1 | GN067842.1 | DL121129.1 | HV700919.1 | DL030961.1 | GM622520.1 |
| GM646944.1 | GN033055.1 | AX143999.1 | AX254695.1 | GN067782.1 | DL116598.1 | HV695514.1 | DL030932.1 | GM622488.1 |
| GM646912.1 | GN032927.1 | AX143871.1 | AX251508.1 | GN046077.1 | DL116566.1 | HV695450.1 | DL027120.1 | GM622456.1 |
| GM646880.1 | GN032895.1 | AX143807.1 | AX249903.1 | DM026491.1 | DL116534.1 | HV502738.1 | DL027088.1 | GM622424.1 |
| GM646848.1 | GN032863.1 | AX143743.1 | AX242268.1 | GN043714.1 | DL116502.1 | HV502706.1 | DL027056.1 | GM622392.1 |
| GM646816.1 | GN032831.1 | AX143679.1 | AX242236.1 | GN042356.1 | DL116470.1 | HV502642.1 | DL027024.1 | GM622360.1 |
| GM640013.1 | GN032767.1 | AX143615.1 | AX242076.1 | GN030760.1 | DL116438.1 | HI988964.1 | DL026992.1 | GM631412.1 |
| GM639981.1 | GN032704.1 | AX143231.1 | AX242044.1 | GN030696.1 | DL111845.1 | HI987670.1 | DL026960.1 | GM631348.1 |
| GM639949.1 | GN032672.1 | DL102485.1 | AX242012.1 | GN030632.1 | DL111813.1 | HI987451.1 | DL030919.1 | GM703991.1 |
| GM639917.1 | GN032639.1 | DL102453.1 | AX241980.1 | GN030568.1 | DL111781.1 | HI987250.1 | DL030880.1 | GM657514.1 |
| GM627279.1 | GN032607.1 | DL102421.1 | AX241948.1 | GN030536.1 | DL105912.1 | FW512233.1 | DL030848.1 | GM657482.1 |
| GM627247.1 | GN032575.1 | DL095429.1 | AX241916.1 | GN030504.1 | DL101299.1 | FW552190.1 | DL030816.1 | GM657450.1 |
| GM627215.1 | GN032544.1 | DL095365.1 | AX241884.1 | GN030472.1 | DL101267.1 | FW552151.1 | DL030784.1 | GM657418.1 |
| GM627183.1 | GN032512.1 | DL106953.1 | AX241852.1 | GN030400.1 | DL101235.1 | FW552115.1 | DL030752.1 | GM657386.1 |
| GM627151.1 | GN032480.1 | DL106921.1 | AX241820.1 | FB704818.1 | DL101203.1 | FW561803.1 | DL026943.1 | GM657354.1 |
| GM660975.1 | GN032448.1 | DL106887.1 | AX241788.1 | FB676521.1 | DL101171.1 | FW555614.1 | DL026911.1 | GM650162.1 |
| GM660943.1 | GN032384.1 | DL091226.1 | AX241756.1 | FB705616.1 | DL096725.1 | FW555582.1 | DL026879.1 | GM650130.1 |
| GM646780.1 | GN032351.1 | DL091194.1 | AX241692.1 | CS810552.1 | DL096693.1 | FW563137.1 | DL026847.1 | GM650098.1 |
| GM646748.1 | GN032319.1 | DL101959.1 | AX241660.1 | FB701799.1 | DL096661.1 | FW558674.1 | DL026815.1 | GM643194.1 |
| GM646716.1 | GN032287.1 | DL095216.1 | AX241628.1 | FB292675.1 | DL096629.1 | FW561684.1 | DL026783.1 | GM643129.1 |
| GM646684.1 | GN032223.1 | DL101216.1 | CS644252.1 | FB700925.1 | DL096597.1 | FW561652.1 | DL022971.1 | GM643065.1 |
| GM646652.1 | GN032191.1 | DL094686.1 | DD462053.1 | FB660206.1 | DL096565.1 | FW505238.1 | DL022939.1 | GM643033.1 |
| GM646620.1 | GN032159.1 | DL090943.1 | DD460662.1 | FB573253.1 | DL094737.1 | FW559556.1 | DL022907.1 | GM636012.1 |
| GM639817.1 | GN032127.1 | DL090911.1 | DD455864.1 | DL199706.1 | DL094705.1 | FW553143.1 | DL022875.1 | GM635980.1 |
| GM639785.1 | GN032095.1 | DL086975.1 | DD455217.1 | DL193811.1 | DL094641.1 | FW556557.1 | DL022843.1 | GM635948.1 |
| GM639753.1 | GN032063.1 | DL086943.1 | DD455159.1 | DL193712.1 | DL094609.1 | FW510504.1 | DL022811.1 | DL098019.1 |

| | | | | | | | | |
|---|---|---|---|---|---|---|---|---|
| GM044790.1 | DL095428.1 | DL108374.1 | DD454368.1 | FB571344.1 | DL094577.1 | HC920507.1 | DL019774.1 | DL097987.1 |
| GM646484.1 | DL095396.1 | DL088953.1 | DD457858.1 | FB570993.1 | DL090962.1 | HD068820.1 | DL019788.1 | DL097955.1 |
| GM646452.1 | DL095364.1 | DL088921.1 | DD456893.1 | CS696116.1 | DL090930.1 | HD066674.1 | DL019732.1 | DL112645.1 |
| GM646420.1 | DL106952.1 | DL088623.1 | DD456416.1 | CS695987.1 | DL090898.1 | HD049000.1 | DL019700.1 | DL112613.1 |
| GM626846.1 | DL106920.1 | DL088591.1 | DD456322.1 | CS695923.1 | DL086962.1 | HD033944.1 | DL019668.1 | DL112581.1 |
| GM626814.1 | DL102197.1 | DL092348.1 | CS643161.1 | CS695891.1 | DL086930.1 | HD033692.1 | DL019636.1 | DL112549.1 |
| GM660762.1 | DL106888.1 | DL092316.1 | CS631729.1 | DL029761.1 | DL086898.1 | HD051914.1 | DL019604.1 | DL112517.1 |
| GM660730.1 | DL087353.1 | DL092284.1 | CS631299.1 | DJ493863.1 | DL086866.1 | HD057705.1 | DL015201.1 | DL112485.1 |
| GM653511.1 | DL120272.1 | DL092252.1 | CS631220.1 | DL007684.1 | DL086834.1 | HD057673.1 | DL015137.1 | DL097928.1 |
| GM653479.1 | DL120240.1 | DL092220.1 | CS642197.1 | DJ446698.1 | DL086802.1 | HD062033.1 | DL015105.1 | DL097896.1 |
| GM653447.1 | DL120208.1 | DL092188.1 | CS630761.1 | CS602734.1 | DL086770.1 | M23050.1 | DL015073.1 | DL097864.1 |
| GM653415.1 | DL111091.1 | DL121941.1 | CS627766.1 | CS602670.1 | DL086738.1 | M69286.1 | DL015041.1 | DL097832.1 |
| GM653383.1 | DL101215.1 | DL121909.1 | CS632414.1 | CS607547.1 | DL086706.1 | M19086.1 | DL010583.1 | DL097800.1 |
| GM646384.1 | DL086974.1 | DL035088.1 | CS626882.1 | CS607037.1 | DL086674.1 | AY528410.1 | DL010551.1 | DL097768.1 |
| GM646352.1 | DL086942.1 | DL035056.1 | CS623716.1 | CS606947.1 | DL086610.1 | HC917647.1 | DL010519.1 | DL097736.1 |
| GM646320.1 | DL088952.1 | DL030709.1 | CS626953.1 | CS606857.1 | DL105883.1 | FW337286.1 | DL010487.1 | DL095709.1 |
| GM646288.1 | DL088920.1 | DL030677.1 | CS625898.1 | CS606812.1 | DL041798.1 | FW336959.1 | DL010455.1 | DL095677.1 |
| GM646256.1 | DL049697.1 | DL026740.1 | DD450124.1 | CS604843.1 | DL041766.1 | HC873812.1 | DL010423.1 | DL095645.1 |
| GM646224.1 | DL049665.1 | DL026708.1 | DD438042.1 | CS604779.1 | DL041734.1 | HC880375.1 | DL046820.1 | DL095613.1 |
| GM639389.1 | DL033295.1 | DL026612.1 | DD449093.1 | CS593004.1 | DL041702.1 | HC207396.1 | DL046788.1 | DL095581.1 |
| GM639357.1 | DL033263.1 | DL019497.1 | DD435295.1 | CS596912.1 | DL037879.1 | HC202869.1 | DL046756.1 | DL095549.1 |
| GM639325.1 | GM869165.1 | DL019465.1 | CS613796.1 | CS592085.1 | DL037847.1 | HC201554.1 | DL046692.1 | DL117386.1 |
| GM639293.1 | GM773639.1 | DL019433.1 | CS613764.1 | CS589290.1 | DL037815.1 | HC199498.1 | DL042870.1 | DL117354.1 |
| GM639261.1 | GM680857.1 | DL019401.1 | CS612951.1 | CS593465.1 | DL037783.1 | DM464633.1 | DL042838.1 | DL117322.1 |
| DL104354.1 | GM879366.1 | CS560592.1 | CS612790.1 | CS585207.1 | DL037751.1 | DM462695.1 | DL042806.1 | DL117290.1 |
| DL104322.1 | GM879305.1 | CS560401.1 | CS611854.1 | CS589199.1 | DL037719.1 | HC193432.1 | DL042774.1 | DL117258.1 |
| DL093610.1 | GM680712.1 | DD421991.1 | CS604548.1 | CS575763.1 | DL037687.1 | HC190452.1 | DL042742.1 | DL117226.1 |
| DL123159.1 | FB722586.1 | CS540035.1 | CS604516.1 | CS174080.1 | DL049612.1 | HC089568.1 | DL042710.1 | DL091645.1 |
| DL123127.1 | FB709348.1 | CS539887.1 | CS604484.1 | CS176145.1 | DL049484.1 | HC089536.1 | DL042678.1 | DL091613.1 |
| DL118713.1 | GM600751.1 | CS502508.1 | BD231940.1 | CS172450.1 | DL049452.1 | HC089504.1 | DL038861.1 | DL091581.1 |
| DL118681.1 | FB721284.1 | CS501492.1 | BD231169.1 | CS172304.1 | DL041661.1 | HC089472.1 | DL038829.1 | DL087970.1 |
| DL118649.1 | DL045190.1 | CS500213.1 | BD231136.1 | CS161648.1 | DL041629.1 | HC089440.1 | DL038797.1 | DL087938.1 |
| DL114221.1 | DL030705.1 | CS495898.1 | BD231058.1 | CS159816.1 | DL041597.1 | HC089407.1 | DL038765.1 | DL087906.1 |
| DL114189.1 | DL030673.1 | DD412110.1 | BD229111.1 | CS159784.1 | DL041565.1 | HC089375.1 | DL038733.1 | DL087874.1 |
| DL114157.1 | DL026640.1 | DD411494.1 | BD227422.1 | CS159226.1 | DL041533.1 | HC089343.1 | DL038701.1 | DL087842.1 |
| DL114093.1 | DL019493.1 | CS492170.1 | BD227017.1 | CS148810.1 | DL041501.1 | HC089310.1 | DL035077.1 | DL087810.1 |
| DL114061.1 | DL019461.1 | CS490485.1 | BD226445.1 | CS144403.1 | DL037678.1 | HC089278.1 | DL035045.1 | DL121769.1 |
| DL126233.1 | DL019429.1 | CS486432.1 | BD225787.1 | CS143749.1 | DL037646.1 | HC089098.1 | DL035013.1 | DL121737.1 |
| DL126201.1 | DL019397.1 | CS417740.1 | BD225755.1 | CS141612.1 | DL037614.1 | HC087509.1 | HH758897.1 | DL121705.1 |
| DL126169.1 | CS574806.1 | CS417328.1 | BD225723.1 | CS141544.1 | DL049307.1 | AY774981.1 | HH757473.1 | DL121641.1 |
| DL122953.1 | CS573057.1 | CS416936.1 | BD225691.1 | CS141510.1 | DL049275.1 | AY774931.1 | HH757441.1 | DL121609.1 |
| DL122921.1 | CS543943.1 | CS410882.1 | BD225659.1 | CS134731.1 | DL049243.1 | AY774890.1 | HH756689.1 | DL087782.1 |
| DL113951.1 | CS570710.1 | CS414822.1 | BD225627.1 | CS131722.1 | DL049212.1 | AY774766.1 | HH756401.1 | DL087750.1 |
| DL113919.1 | CS546676.1 | DD326775.1 | BD224839.1 | CS133021.1 | DL049180.1 | AY774653.1 | HH756252.1 | DL087718.1 |
| DL113887.1 | DD420111.1 | CS389300.1 | BD223260.1 | CS122833.1 | DL049116.1 | AY774594.1 | HI401630.1 | DL087686.1 |
| DL113855.1 | DD432993.1 | CS389258.1 | BD222968.1 | CS122300.1 | DL049084.1 | AY774529.1 | HI401097.1 | DL087654.1 |
| DL109054.1 | DD431300.1 | CS389212.1 | BD222513.1 | CS121534.1 | DL049052.1 | AY774427.1 | HI645147.1 | DL087622.1 |

| | | | | | | | | |
|---|---|---|---|---|---|---|---|---|
| DL109022.1 | DD431731.1 | CS389174.1 | BD221775.1 | CS106114.1 | DL012576.1 | AY774364.1 | HI642827.1 | DL087590.1 |
| DL108990.1 | CS537814.1 | CS399168.1 | BD217963.1 | CS106034.1 | DL012544.1 | AY774313.1 | HI642283.1 | DL125521.1 |
| DL104288.1 | CS501441.1 | CS401710.1 | BD211424.1 | CS327364.1 | DL012512.1 | AY774149.1 | HI641739.1 | DL125489.1 |
| DL104256.1 | CS498443.1 | CS401401.1 | BD205392.1 | CS323576.1 | DL012480.1 | AY774013.1 | HI641195.1 | DL125457.1 |
| DL104224.1 | CS495888.1 | CS406378.1 | DD283500.1 | CS330852.1 | DL012416.1 | AY334020.1 | HI640651.1 | DL125425.1 |
| DL099586.1 | DD417886.1 | CS408806.1 | DD279453.1 | CS319843.1 | DL048844.1 | AF271618.1 | HI639894.1 | DL125393.1 |
| DL099554.1 | DD417783.1 | CS382594.1 | CS356001.1 | DD259912.1 | DL045426.1 | HC084789.1 | HI637816.1 | DL125361.1 |
| DL047860.1 | CS490481.1 | DD308878.1 | CS355555.1 | CS302061.1 | DL045394.1 | HC083714.1 | HI380443.1 | DL121572.1 |
| DL040061.1 | CS359725.1 | DD298327.1 | CS350580.1 | E03314.1 | DL045330.1 | HC083537.1 | HI379488.1 | DL121540.1 |
| DL040029.1 | BD269158.1 | DD307924.1 | CS349697.1 | E03062.1 | DL045298.1 | DM460985.1 | HI379309.1 | DL121508.1 |
| DL039997.1 | BD269126.1 | CS376112.1 | CS283938.1 | E02689.1 | DL041250.1 | GM992958.1 | HI379129.1 | DL121476.1 |
| DL047656.1 | BD268882.1 | CS363373.1 | CS272489.1 | E02533.1 | DL041218.1 | GM992507.1 | HI378745.1 | DL121444.1 |
| DL043674.1 | BD265639.1 | CS359729.1 | CS253983.1 | E02239.1 | DL041186.1 | GM992362.1 | FW396261.1 | DL121412.1 |
| CS614387.1 | BD263490.1 | CS359164.1 | CS250285.1 | E02090.1 | DL041122.1 | GM992222.1 | FW395612.1 | DL117200.1 |
| CS613367.1 | BD263436.1 | BD269162.1 | CS247172.1 | E01697.1 | DL037203.1 | GM989958.1 | HH935708.1 | DL117168.1 |
| CS612778.1 | BD263023.1 | BD269130.1 | CS244965.1 | E01387.1 | DL037139.1 | GN000750.1 | HH934830.1 | DL117136.1 |
| CS611822.1 | BD250956.1 | BD268891.1 | CS244167.1 | E00341.1 | DL037107.1 | GM969682.1 | HH834153.1 | DL117104.1 |
| CS604536.1 | BD248726.1 | DD034191.1 | CS244247.1 | DD087930.1 | DL028736.1 | GM969547.1 | HH833647.1 | DL117072.1 |
| CS604504.1 | BD247028.1 | DD023874.1 | CS244183.1 | DD146207.1 | DL028704.1 | GM970627.1 | HH833615.1 | DL117040.1 |
| CS604408.1 | BD246953.1 | DD029346.1 | BD344758.1 | DD145694.1 | DL028672.1 | GM970096.1 | HH833583.1 | DL112452.1 |
| CS603448.1 | BD244509.1 | DD017644.1 | BD342498.1 | DD086378.1 | DL028608.1 | DL465812.1 | HH833551.1 | DL112420.1 |
| CS603416.1 | BD243689.1 | BD412623.1 | BD341871.1 | DD112413.1 | DL028576.1 | DL464619.1 | HH833519.1 | DL112388.1 |
| CS603384.1 | BD242441.1 | BD434319.1 | BD349866.1 | DD142868.1 | DL024762.1 | DL463056.1 | HH833487.1 | DL112356.1 |
| CS603352.1 | BD241014.1 | BD453886.1 | BD339059.1 | CQ869274.1 | DL024730.1 | DL462994.1 | HH833455.1 | DL112324.1 |
| CS603320.1 | BD238521.1 | BD453854.1 | BD314392.1 | CQ868864.1 | DL024698.1 | DL462894.1 | HH833423.1 | DL112292.1 |
| CS603288.1 | BD238072.1 | BD453822.1 | BD313266.1 | CQ858727.1 | DL021358.1 | FB504556.1 | HH833391.1 | DL107278.1 |
| CS603256.1 | BD236956.1 | BD453790.1 | BD312763.1 | CQ858095.1 | DL021326.1 | GM831916.1 | HH833366.1 | DL107246.1 |
| CS603224.1 | BD235724.1 | BD433654.1 | BD319658.1 | CQ855760.1 | DL016928.1 | FB509339.1 | HH833334.1 | DL107214.1 |
| CS603192.1 | BD235407.1 | BD453766.1 | BD319294.1 | CQ854063.1 | DL016896.1 | DL102847.1 | HH832582.1 | DL107182.1 |
| CS603160.1 | BD235252.1 | BD453734.1 | BD318871.1 | CQ832289.1 | DL016832.1 | DL087351.1 | HH832294.1 | DL107150.1 |
| CS603128.1 | BD234795.1 | BD453702.1 | BD317181.1 | CQ829193.1 | DL012374.1 | HW062327.1 | HH836842.1 | DL119497.1 |
| CS603096.1 | BD233774.1 | BD453670.1 | BD308151.1 | CQ816989.1 | DL012342.1 | HW062186.1 | FW392716.1 | DL119465.1 |
| CS603064.1 | BD231754.1 | BD453638.1 | BD307647.1 | CQ816947.1 | DL012310.1 | HW042050.1 | HH928901.1 | DL119433.1 |
| CS603032.1 | BD227086.1 | BD453606.1 | BD300441.1 | CQ815504.1 | DL012278.1 | HW042018.1 | HH931894.1 | DL123744.1 |
| CS603000.1 | BD226833.1 | BD493410.1 | BD294413.1 | CQ814105.1 | DL048695.1 | HW043502.1 | HH925816.1 | DL123712.1 |
| CS602966.1 | BD224577.1 | HI000420.1 | BD293452.1 | CQ814073.1 | DL048663.1 | HW042006.1 | HH821547.1 | DL123680.1 |
| CS602934.1 | BD223029.1 | HI000378.1 | BD292439.1 | CQ814040.1 | DL045245.1 | HW041974.1 | HH828414.1 | DL123648.1 |
| CS602678.1 | BD206025.1 | HI000321.1 | BD292048.1 | CQ814008.1 | DL045213.1 | HW041942.1 | HH827371.1 | DL123616.1 |
| E43938.1 | DD282349.1 | HI203345.1 | BD291383.1 | CQ813976.1 | DL045181.1 | HW049364.1 | HH832134.1 | DL123584.1 |
| E36133.1 | DD286555.1 | HI553274.1 | BD291234.1 | CQ813944.1 | DL045149.1 | HW049333.1 | HH826839.1 | DL119394.1 |
| E06790.1 | AX799557.1 | HI583855.1 | BD299610.1 | CQ813912.1 | DL045117.1 | HW049301.1 | HH826500.1 | DL119362.1 |
| E06031.1 | AX798976.1 | HI002090.1 | BD298678.1 | CQ813880.1 | DL045085.1 | HW043304.1 | HH822363.1 | DL119330.1 |
| E04628.1 | AX796715.1 | HI002043.1 | BD296983.1 | CQ813848.1 | DL040973.1 | HW041808.1 | HH822045.1 | DL119298.1 |
| E04411.1 | AY390769.1 | HI001956.1 | BD296941.1 | CQ813816.1 | DL040941.1 | HW041776.1 | HH821913.1 | DL119266.1 |
| E03936.1 | AX472463.1 | HI000289.1 | BD296885.1 | CQ813695.1 | DL040909.1 | HW041555.1 | FW379683.1 | DL119234.1 |
| E03393.1 | BD187657.1 | HI000247.1 | BD296284.1 | CQ802181.1 | DL037086.1 | HW046686.1 | FW379650.1 | DL114838.1 |
| E02972.1 | AX787194.1 | HI000203.1 | BD285217.1 | CQ800804.1 | DL037054.1 | HV200221.1 | FW379618.1 | DL114806.1 |

| | | | | | | | | |
|---|---|---|---|---|---|---|---|---|
| E02733.1 | AX781269.1 | HI000147.1 | AX003046.1 | CQ800436.1 | DL037022.1 | HV191409.1 | FW379576.1 | DL114774.1 |
| E02568.1 | AX773263.1 | HI000115.1 | AX002968.1 | CQ799256.1 | DL036990.1 | HV217030.1 | FW378153.1 | DL114742.1 |
| E01322.1 | AX766525.1 | HI509228.1 | A48491.1 | CQ795465.1 | DL031583.1 | HV200213.1 | FW377829.1 | DL114710.1 |
| E01208.1 | AX766124.1 | HI204477.1 | A40271.1 | AX398978.1 | DL031551.1 | HV235476.1 | FW377385.1 | DL114678.1 |
| E00699.1 | AX763954.1 | HI201811.1 | A35761.1 | AX395626.1 | DL027742.1 | HV121456.1 | FW377112.1 | DL114646.1 |
| E00304.1 | AX754677.1 | HI552519.1 | A35711.1 | AX392095.1 | DL027710.1 | HV038564.1 | FW377037.1 | A10474.1 |
| DD102365.1 | AX752747.1 | AY774930.1 | A33119.1 | AX377468.1 | DL027678.1 | HV190733.1 | FW376188.1 | A07607.1 |
| DD115471.1 | BD184736.1 | AY774822.1 | A31758.1 | AX367114.1 | DL027646.1 | HV202318.1 | FV534187.1 | A04348.1 |
| DD087681.1 | BD182839.1 | AY774764.1 | A35493.1 | AX364840.1 | DL027614.1 | HV035389.1 | FV533994.1 | A00631.1 |
| DD144781.1 | BD181487.1 | AY774652.1 | A32833.1 | AX364660.1 | DL027582.1 | HV035201.1 | HC460532.1 | A32045.1 |
| DD099486.1 | BD180847.1 | AY774592.1 | A29194.1 | AX364543.1 | DL023770.1 | FZ422164.1 | HC465766.1 | A30559.1 |
| DD099311.1 | AY181085.1 | AY774528.1 | A27479.1 | AX364511.1 | DL023738.1 | FZ437279.1 | AF519543.1 | A29213.1 |
| DD084740.1 | BD177115.1 | AY774488.1 | A19498.1 | AX364447.1 | DL023706.1 | HI204252.1 | AF340222.1 | A26679.1 |
| DD069501.1 | AX286079.1 | AY774423.1 | A16290.1 | AX364415.1 | DL023674.1 | HI551864.1 | HC456293.1 | A22626.1 |
| DD098109.1 | AX283084.1 | AY774312.1 | A14591.1 | AX364383.1 | DL023642.1 | HI559219.1 | HC453871.1 | A27820.1 |
| DD141744.1 | AX279835.1 | AY774264.1 | A11971.1 | AX364224.1 | DL023610.1 | HI568865.1 | HC453687.1 | A25792.1 |
| DD152071.1 | AX253389.1 | AY774208.1 | A10690.1 | BD006207.1 | DL023578.1 | HI564223.1 | HC460197.1 | A24913.1 |
| DD158396.1 | AX249946.1 | AY774147.1 | A10462.1 | BD002012.1 | DL020563.1 | HI564105.1 | HC452250.1 | A23027.1 |
| DD157374.1 | AX242311.1 | AY774076.1 | A07335.1 | E59326.1 | DL020531.1 | HI583088.1 | HC452168.1 | HV757239.1 |
| DD156984.1 | AX242279.1 | AF271233.1 | A02816.1 | AX350303.1 | DL020499.1 | HI544154.1 | HC452110.1 | HV743388.1 |
| DD155700.1 | AX242215.1 | HC084187.1 | A32041.1 | AX347297.1 | DL020467.1 | HI586400.1 | HC452058.1 | HW303245.1 |
| DD152409.1 | AX242183.1 | HC083536.1 | A31181.1 | AX347263.1 | DL020435.1 | HI000018.1 | HC451404.1 | HW302704.1 |
| DD151858.1 | AX242151.1 | DM381888.1 | HW318303.1 | AX347227.1 | A01789.1 | HI001889.1 | HC450599.1 | HW286647.1 |
| DD097291.1 | AX242023.1 | HC055541.1 | HW335187.1 | AX347189.1 | AX443302.1 | HI001787.1 | FU762422.1 | HW295981.1 |
| DD083031.1 | AX241959.1 | HC053847.1 | HW335144.1 | AX347155.1 | AX441326.1 | HI001751.1 | FU764391.1 | HW302488.1 |
| DD082081.1 | AX241895.1 | HC051853.1 | HW314063.1 | AX344885.1 | AX429493.1 | HI469695.1 | FU757699.1 | HW302377.1 |
| DD147874.1 | AX241863.1 | DM155789.1 | HW313985.1 | AX343226.1 | AX429240.1 | HI469520.1 | FU757611.1 | HW295587.1 |
| DD147780.1 | HH979570.1 | HB451958.1 | HW312127.1 | AX339391.1 | AX428229.1 | HI465380.1 | FU756914.1 | HW294980.1 |
| DD147748.1 | HH931974.1 | HB463484.1 | HW312057.1 | AX339193.1 | AX427122.1 | HI465284.1 | FU762037.1 | HW307052.1 |
| DD147716.1 | FW394282.1 | HB447898.1 | HW311837.1 | AX328135.1 | AX419091.1 | HI001630.1 | HC438976.1 | HW294151.1 |
| DD147684.1 | FW394263.1 | DM138072.1 | HW311251.1 | AX316084.1 | AX418391.1 | HI001587.1 | HC438425.1 | HW293171.1 |
| DD139386.1 | FW394189.1 | DM130830.1 | HW311146.1 | HW260875.1 | AX418119.1 | HI001542.1 | HC440479.1 | HW293036.1 |
| DD122490.1 | FW397526.1 | HB435572.1 | HW309423.1 | HW260843.1 | AX404884.1 | HI003372.1 | HC436477.1 | HW292759.1 |
| DD138528.1 | FW396388.1 | HB427369.1 | HW308086.1 | HW260811.1 | AX403364.1 | HI003334.1 | HC435959.1 | HW291175.1 |
| CQ831245.1 | FW396653.1 | HB423189.1 | HW307871.1 | HW260779.1 | AX402394.1 | HI003291.1 | HC358229.1 | HW291078.1 |
| CQ827704.1 | HH834388.1 | HB394259.1 | HW307807.1 | HW260747.1 | AX398678.1 | HI550329.1 | HC358197.1 | HW291046.1 |
| CQ826738.1 | HB645725.1 | HB393664.1 | HW307775.1 | HW260715.1 | AX397809.1 | HI464740.1 | HC358066.1 | HW291014.1 |
| CQ824414.1 | HB645693.1 | HB397689.1 | HW316587.1 | HW260683.1 | AX395585.1 | HI464708.1 | HC357633.1 | HW290982.1 |
| CQ821319.1 | HB645649.1 | DM115190.1 | HW307737.1 | HW260651.1 | AX391497.1 | HI464676.1 | HC357220.1 | HW290950.1 |
| CQ821217.1 | HB855453.1 | DM114648.1 | HW307705.1 | HW260619.1 | AX382515.1 | HI464644.1 | HC325472.1 | HW290918.1 |
| CQ818159.1 | DM179555.1 | HB385916.1 | HW315962.1 | HW260587.1 | AX379583.1 | HI550023.1 | A07560.1 | HW290887.1 |
| CQ817003.1 | DM169954.1 | GN091493.1 | HW315779.1 | HW260555.1 | AX376963.1 | HI464444.1 | HC324977.1 | HW290855.1 |
| CQ816959.1 | DM173775.1 | GN090780.1 | HW315565.1 | HW260523.1 | AX374949.1 | HI504431.1 | HC324507.1 | HW290399.1 |
| CQ816925.1 | HB559420.1 | GN089891.1 | HW315140.1 | HW260491.1 | AX370706.1 | HI462579.1 | HC319647.1 | HW290346.1 |
| CQ814052.1 | HB488911.1 | GN089826.1 | HW315015.1 | HW260459.1 | AX364694.1 | HH713945.1 | GN032019.1 | HW290134.1 |
| CQ814020.1 | DM164036.1 | GN082908.1 | HW314906.1 | HW260427.1 | AX364655.1 | HH961363.1 | GN031987.1 | HW288961.1 |
| CQ813988.1 | DM163988.1 | GN089166.1 | HW314842.1 | HW260395.1 | AX364538.1 | HH961331.1 | GN031955.1 | HW291111.1 |

| | | | | | | | | |
|---|---|---|---|---|---|---|---|---|
| CQ813956.1 | DM163955.1 | GN082805.1 | HW314786.1 | HW260363.1 | AX364506.1 | DM164035.1 | GN031923.1 | HW285155.1 |
| CQ813924.1 | DM163285.1 | GN082363.1 | HW314740.1 | HW260331.1 | AX364474.1 | DM164021.1 | GN031891.1 | HW269947.1 |
| CQ813892.1 | DM016014.1 | L09141.1 | HW151141.1 | HW260299.1 | AX364442.1 | DM163987.1 | GN031859.1 | HW269294.1 |
| CQ813860.1 | DM026401.1 | M26666.1 | HW112797.1 | HW260267.1 | AX145159.1 | DM163953.1 | GN031827.1 | HW269253.1 |
| CQ813828.1 | GN044860.1 | DM059589.1 | HW112685.1 | HW260235.1 | AX145127.1 | DM163284.1 | GN031729.1 | HW267805.1 |
| CQ813796.1 | GN042980.1 | DM058888.1 | HW112616.1 | HW260203.1 | AX145095.1 | DM163073.1 | GN031665.1 | HW266127.1 |
| CQ806488.1 | GN042200.1 | DM045495.1 | HW070020.1 | HW260171.1 | AX145063.1 | HB475681.1 | GN031633.1 | HW265863.1 |
| CQ803119.1 | GN041317.1 | DM045313.1 | HW069956.1 | HW260139.1 | AX145031.1 | HB474891.1 | GN031601.1 | HW263073.1 |
| CQ802836.1 | GN030757.1 | DM045036.1 | HW084948.1 | HW260107.1 | AX144999.1 | HB463823.1 | GN031569.1 | HW263036.1 |
| CQ801658.1 | GN030693.1 | DM039630.1 | HW084909.1 | HW260075.1 | AX144967.1 | HB386675.1 | GN031537.1 | HW262732.1 |
| CQ798786.1 | GN030661.1 | GM631830.1 | HW097742.1 | HW260043.1 | AX144935.1 | HB385991.1 | GN031473.1 | HW262587.1 |
| CQ795606.1 | GN030597.1 | GM631798.1 | HW087994.1 | HW260011.1 | AX144902.1 | HB340483.1 | GN031440.1 | HV819312.1 |
| CQ795477.1 | GN030565.1 | GM622835.1 | HW065057.1 | HW259979.1 | AX144870.1 | HA642537.1 | GN031408.1 | HV803292.1 |
| CQ795445.1 | GN030533.1 | GM046079.1 | HW057915.1 | HW259947.1 | AX144838.1 | HA641568.1 | GN031344.1 | HV932731.1 |
| CQ794290.1 | GN030501.1 | GM715046.1 | HW060211.1 | GN359027.1 | AX144806.1 | HA641504.1 | GN031312.1 | HV932698.1 |
| CQ788636.1 | GN030373.1 | GM647265.1 | HW047623.1 | GM870298.1 | AX144774.1 | HA638525.1 | GN031279.1 | HV932634.1 |
| CQ787444.1 | GN030213.1 | GM647233.1 | HW047248.1 | GM714531.1 | AX144742.1 | HA643883.1 | GN031247.1 | HV929971.1 |
| CQ787364.1 | GN029827.1 | GM647201.1 | HV969610.1 | DL202563.1 | AX144710.1 | GN368120.1 | GN031183.1 | HV932336.1 |
| CQ787328.1 | GN013579.1 | GM661508.1 | HV956154.1 | DL188835.1 | AX144678.1 | GN360137.1 | GN031118.1 | HV803248.1 |
| DL121910.1 | GN033561.1 | GM647169.1 | HV951081.1 | FB571339.1 | AX144646.1 | GN356186.1 | GN031086.1 | HV803184.1 |
| CS367323.1 | GN033529.1 | GM681765.1 | HV957526.1 | CS696175.1 | AX144614.1 | DM045293.1 | GN031054.1 | HV803152.1 |
| CS368370.1 | GN033497.1 | FB753862.1 | HV349486.1 | CS696143.1 | AX144229.1 | DM045011.1 | GN030990.1 | HV803120.1 |
| CS367986.1 | GN033465.1 | GM618812.1 | HV351585.1 | CS696111.1 | AX144165.1 | DM044709.1 | GN030959.1 | HV803088.1 |
| DJ417456.1 | GN033433.1 | GM864203.1 | HV334774.1 | CS696047.1 | AX144037.1 | GN067949.1 | GN030927.1 | HV802950.1 |
| DJ387460.1 | GN033369.1 | GM863540.1 | HV344156.1 | CS696014.1 | AX143971.1 | GN067934.1 | GN030895.1 | HV802918.1 |
| DJ380844.1 | GN033305.1 | FB725704.1 | HV343928.1 | CS695982.1 | AX143907.1 | GN067714.1 | GN030831.1 | HV802886.1 |
| DJ380812.1 | GN033273.1 | GM616028.1 | HV343736.1 | CS695950.1 | AX143843.1 | GN052363.1 | GN030799.1 | HV802845.1 |
| DJ388719.1 | GN033241.1 | FB722375.1 | HV340901.1 | CS695918.1 | AX143715.1 | DM016013.1 | DM001643.1 | HV818374.1 |
| DJ388676.1 | GN033209.1 | GM840671.1 | HV347717.1 | CS695790.1 | AX143651.1 | GN045778.1 | DM001478.1 | HV813039.1 |
| CS724477.1 | GN033177.1 | GM040833.1 | HV344959.1 | CS695758.1 | AX143523.1 | GN044855.1 | DM006965.1 | HV817939.1 |
| CS724445.1 | GN033081.1 | CS729400.1 | HV344920.1 | CS695726.1 | AX143331.1 | GN041963.1 | DM006722.1 | HV822395.1 |
| CS722092.1 | GN033017.1 | CS728631.1 | HV340086.1 | CS695694.1 | AX143267.1 | GN041315.1 | DM004988.1 | HV822303.1 |
| DJ353076.1 | GN032985.1 | CS728535.1 | HV340054.1 | DL176382.1 | AX143203.1 | GN030692.1 | DM004970.1 | HV817655.1 |
| DJ357124.1 | GN032921.1 | CS727343.1 | HV344644.1 | DL175281.1 | AX143139.1 | GN030628.1 | GM652663.1 | HV816794.1 |
| DJ344613.1 | GN032857.1 | CS727260.1 | HV339932.1 | DL181439.1 | AX143075.1 | GN030596.1 | GM652631.1 | HV784510.1 |
| DJ354268.1 | GN032825.1 | FB709019.1 | HV339900.1 | DL174423.1 | AX143011.1 | GN030564.1 | GM652599.1 | HV780280.1 |
| DJ361195.1 | GN032793.1 | FB715326.1 | HV342421.1 | DL176801.1 | AX142819.1 | HH998167.1 | GM645505.1 | HV779809.1 |
| DJ339779.1 | GN032761.1 | GM715443.1 | HV344530.1 | DD435323.1 | AX142755.1 | HH986613.1 | GM645473.1 | HV778903.1 |
| DJ339747.1 | GN032730.1 | GM949529.1 | HV342010.1 | CS616563.1 | AX142627.1 | HH979500.1 | GM645441.1 | HV778701.1 |
| DJ339651.1 | GN032698.1 | GM887854.1 | HV344548.1 | CS612714.1 | AX142563.1 | HH979567.1 | GM746439.1 | HV775037.1 |
| DJ339554.1 | DL111213.1 | GM869485.1 | HV323131.1 | CS611452.1 | AX142369.1 | HH932616.1 | GM638612.1 | HV775002.1 |
| DJ339166.1 | DL127363.1 | BD265578.1 | HV324876.1 | CS604331.1 | AX142241.1 | HH932049.1 | GM638580.1 | HV774955.1 |
| DJ328796.1 | DL114715.1 | BD263465.1 | HV324774.1 | CS603371.1 | AX142177.1 | HH931967.1 | GM638548.1 | HV764745.1 |
| DJ327122.1 | DL127182.1 | BD263433.1 | HV324042.1 | CS603339.1 | AX142049.1 | FW417248.1 | GM638516.1 | HV764713.1 |
| DJ327037.1 | DL141442.1 | BD251788.1 | FW562639.1 | CS602857.1 | AX141853.1 | FW416909.1 | GM638484.1 | HV769881.1 |
| DJ327005.1 | DL021268.1 | BD247911.1 | FW562514.1 | CS602825.1 | AX141789.1 | FW394356.1 | GM638452.1 | HV774491.1 |
| DJ326917.1 | DL039748.1 | BD247122.1 | FW505284.1 | BD242468.1 | AX141341.1 | FW394279.1 | GM633753.1 | HV503595.1 |

| | | | | | | | | |
|---|---|---|---|---|---|---|---|---|
| DJ122189.1 | DL023584.1 | BD246943.1 | FW505126.1 | BD242436.1 | AX141240.1 | FW419785.1 | GM633721.1 | HV503531.1 |
| CS408807.1 | DJ417448.1 | BD244457.1 | FW508119.1 | BD236916.1 | AX138889.1 | FW394260.1 | GM633689.1 | HV503499.1 |
| CS402566.1 | DJ380836.1 | DD181035.1 | FW562496.1 | BD235870.1 | AX137986.1 | FW394186.1 | GM625822.1 | HV503467.1 |
| CS401812.1 | DJ389574.1 | DD161359.1 | FW561001.1 | BD235379.1 | AX137259.1 | FW419004.1 | GM625790.1 | HV503435.1 |
| CS381642.1 | DJ388709.1 | DD165455.1 | FW555403.1 | BD233642.1 | AX133526.1 | FW397522.1 | GM625758.1 | HV503403.1 |
| CS382595.1 | DJ388666.1 | DD182193.1 | FW553184.1 | BD231069.1 | AX119978.1 | FW397441.1 | GM652574.1 | HV503371.1 |
| CS374393.1 | CS724429.1 | CS118442.1 | AY657358.1 | BD229943.1 | AX113818.1 | FW393131.1 | GM624702.1 | HV503339.1 |
| DD308880.1 | CS723756.1 | CS118410.1 | AY657326.1 | BD225173.1 | AX113573.1 | FW418615.1 | GM624670.1 | HV503307.1 |
| DD298328.1 | DJ353064.1 | CS118377.1 | AY657294.1 | BD224572.1 | AX111977.1 | FW418143.1 | GM624638.1 | HV503275.1 |
| DD307925.1 | DJ340975.1 | CS118343.1 | AY657262.1 | BD223672.1 | AX108681.1 | FW396787.1 | GM624606.1 | HV503243.1 |
| DD292501.1 | DJ361562.1 | CS118276.1 | AY657230.1 | BD218009.1 | AX108326.1 | FW396266.1 | GM624574.1 | HV503211.1 |
| CS376403.1 | DJ361220.1 | CS118244.1 | AY657198.1 | BD211656.1 | AX107883.1 | HH944570.1 | GM624542.1 | GM623811.1 |
| CS376113.1 | DJ361187.1 | CS118211.1 | AY657166.1 | BD195437.1 | AX107383.1 | HH937345.1 | GM624509.1 | GM623779.1 |
| CS359730.1 | DJ339803.1 | CS118177.1 | AY657134.1 | DD283507.1 | AX100534.1 | HH934833.1 | GM624477.1 | GM623747.1 |
| CS359188.1 | DJ339739.1 | CS118145.1 | AY657102.1 | CS354069.1 | AX006468.1 | HH934456.1 | GM624445.1 | GM658926.1 |
| BD269163.1 | DD326760.1 | CS118113.1 | AY657070.1 | CS353195.1 | AX004686.1 | HH834785.1 | GM624413.1 | GM658862.1 |
| BD269131.1 | CS389292.1 | CS118080.1 | AY657038.1 | CS283945.1 | AX003187.1 | HH834380.1 | GM624381.1 | GM658830.1 |
| BD268892.1 | CS389203.1 | CS115734.1 | M18932.1 | CS283064.1 | AX002973.1 | HH834166.1 | GM624349.1 | GM658798.1 |
| BD268152.1 | CS389166.1 | CS110176.1 | J02532.1 | CS277377.1 | A13367.1 | HH833955.1 | GM633652.1 | GM651574.1 |
| BD265601.1 | CS401300.1 | CS106382.1 | J02550.1 | CS276838.1 | A49206.1 | HH833710.1 | GM633620.1 | GM651542.1 |
| BD263860.1 | CS406463.1 | CS102894.1 | M10180.1 | CS273096.1 | A35777.1 | HH833650.1 | GM633588.1 | GM651510.1 |
| BD263060.1 | CS403224.1 | CS102766.1 | HV549726.1 | CS265236.1 | A35714.1 | HH833618.1 | GM633556.1 | GM651478.1 |
| BD261180.1 | CS402334.1 | CS102670.1 | HV549688.1 | CS018501.1 | A31765.1 | HH833586.1 | GM633524.1 | GM651446.1 |
| BD250962.1 | CS402129.1 | CS102606.1 | HV549656.1 | CS018359.1 | A35497.1 | HH833554.1 | GM633492.1 | GM651414.1 |
| BD249011.1 | DD292137.1 | CS102574.1 | HV549624.1 | CS016851.1 | A34235.1 | HH833522.1 | GM625714.1 | GM651382.1 |
| BD248130.1 | DD291029.1 | CS102542.1 | HH756189.1 | CQ989090.1 | A33990.1 | HH833490.1 | GM625682.1 | GM644583.1 |
| BD247033.1 | CS376016.1 | CS102478.1 | HI401239.1 | CQ986542.1 | A32836.1 | HH833458.1 | GM625650.1 | GM644551.1 |
| BD245159.1 | CS362757.1 | CS101277.1 | HI401090.1 | CQ980558.1 | A32638.1 | HH833426.1 | GM625586.1 | GM644519.1 |
| BD244549.1 | CS359952.1 | CS097943.1 | HD117900.1 | CQ976307.1 | GM637979.1 | HH833394.1 | GM625554.1 | GM644487.1 |
| BD243428.1 | CS359650.1 | CS091849.1 | HI645013.1 | CQ975806.1 | GM637947.1 | HH833369.1 | GM638433.1 | GM644455.1 |
| BD243143.1 | BD269155.1 | CS086833.1 | HI642810.1 | CQ972375.1 | GM637915.1 | HH833337.1 | GM638401.1 | GM644423.1 |
| BD242607.1 | BD269123.1 | CS082765.1 | HI642266.1 | CQ970085.1 | GM637883.1 | HH832457.1 | GM638369.1 | GM637425.1 |
| BD242446.1 | BD265633.1 | AX468901.1 | HI641722.1 | CQ947134.1 | GM637851.1 | FW390807.1 | GM638337.1 | GM637393.1 |
| BD238551.1 | DL097989.1 | AX467093.1 | HI641178.1 | CQ944204.1 | GM633443.1 | FW383056.1 | GM638305.1 | GM637361.1 |
| BD238172.1 | DL097957.1 | A18207.1 | HI637703.1 | CQ944172.1 | GM633411.1 | HH928904.1 | GM638273.1 | GM637329.1 |
| BD236965.1 | DL112647.1 | AX460841.1 | HI380328.1 | CQ944140.1 | GM633379.1 | HH931897.1 | GM624303.1 | GM637297.1 |
| BD235357.1 | DL112615.1 | AX458590.1 | HI379108.1 | CQ944108.1 | GM633347.1 | HH924994.1 | GM624271.1 | GM637265.1 |
| BD234800.1 | DL112583.1 | AX458213.1 | HI378132.1 | CQ944076.1 | GM633315.1 | HH821552.1 | GM652546.1 | GM623728.1 |
| BD231827.1 | DL112551.1 | AX454009.1 | HI377626.1 | CQ944044.1 | GM633283.1 | HH821180.1 | GM652514.1 | GM623696.1 |
| BD231154.1 | DL112519.1 | AX453568.1 | HI376943.1 | AX657122.1 | GM643894.1 | HH829732.1 | GM652482.1 | GM623664.1 |
| CS172890.1 | DL112487.1 | AX451647.1 | HI375925.1 | AX657087.1 | GM643862.1 | HH828419.1 | GM652450.1 | GM623600.1 |
| CS157785.1 | DL097930.1 | AX443327.1 | HI375013.1 | AX657021.1 | GM636833.1 | HH827919.1 | GM652386.1 | GM623568.1 |
| CS157946.1 | DL097898.1 | AX428405.1 | HI373873.1 | BD174405.1 | GM636801.1 | HH827887.1 | GM659906.1 | GM658717.1 |
| CS157882.1 | DL097866.1 | AX418259.1 | HI371008.1 | AY192160.1 | GM636769.1 | HH827855.1 | GM659874.1 | GM658685.1 |
| CS157850.1 | DL097834.1 | AX402400.1 | HI369413.1 | AX643693.1 | GM636737.1 | HH827823.1 | GM659842.1 | GM658653.1 |
| CS124703.1 | DL097802.1 | AX399385.1 | HI369110.1 | AX642235.1 | GM636705.1 | FU258434.1 | GM659810.1 | GM658621.1 |
| CS119863.1 | DL097770.1 | AX380780.1 | HH987075.1 | AX601462.1 | GM636673.1 | FU258370.1 | GM659778.1 | GM658557.1 |

| | | | | | | | | |
|---|---|---|---|---|---|---|---|---|
| CS119498.1 | DL097738.1 | AX364661.1 | HH957935.1 | AX597564.1 | GM632265.1 | FU258338.1 | GM659746.1 | GM651365.1 |
| CS119465.1 | DL095679.1 | AX364544.1 | HH980359.1 | AX593488.1 | GM632233.1 | HC313324.1 | GM746224.1 | GM651333.1 |
| CS119431.1 | DL095647.1 | AX364512.1 | HH980160.1 | BD160930.1 | GM632201.1 | HC313150.1 | GM659710.1 | GM651301.1 |
| CS119398.1 | DL095615.1 | AX364480.1 | HH980127.1 | AX576326.1 | GM632169.1 | HC312244.1 | GM659678.1 | GM651269.1 |
| CS119365.1 | DL095583.1 | AX364416.1 | HH979882.1 | AX556835.1 | GM632137.1 | HC311312.1 | GM659646.1 | GM651237.1 |
| CS119333.1 | DL095551.1 | AX364384.1 | HH996711.1 | AX555092.1 | GM632105.1 | DM475259.1 | GM659614.1 | GM651205.1 |
| CS119299.1 | DL117388.1 | AX364352.1 | HH996645.1 | AX539580.1 | GM623334.1 | DM466575.1 | GM659582.1 | GM644406.1 |
| CS119235.1 | DL117356.1 | AX364257.1 | HH996605.1 | AX081169.1 | GM623302.1 | DM466015.1 | GM659549.1 | GM644374.1 |
| CS119171.1 | DL117324.1 | AX364192.1 | HH996549.1 | AX077303.1 | GM623270.1 | HC306258.1 | GM652357.1 | GM644342.1 |
| CS119139.1 | DL117292.1 | AX363260.1 | HH998455.1 | AX076538.1 | GM623238.1 | HC306218.1 | GM652325.1 | GM644246.1 |
| CS119106.1 | DL117260.1 | AX358189.1 | HH998406.1 | AX074303.1 | GM623206.1 | HC306178.1 | GM652293.1 | GM637248.1 |
| CS119072.1 | DL117228.1 | AX356725.1 | HH998367.1 | AJ291296.1 | GM623174.1 | HC306142.1 | GM652261.1 | GM637216.1 |
| CS119039.1 | DL091743.1 | BD006208.1 | HH999999.1 | AX057040.1 | GM623142.1 | HC306102.1 | GM652229.1 | GM637184.1 |
| CS119007.1 | DL091679.1 | BD004261.1 | HH999953.1 | AX050416.1 | GM741844.1 | HC306062.1 | GM652197.1 | GM637152.1 |
| CS118941.1 | DL091615.1 | BD002013.1 | HH979821.1 | AX048122.1 | GM741812.1 | HC306022.1 | GM645398.1 | GM637120.1 |
| CS118909.1 | DL091583.1 | DQ250222.1 | HH979715.1 | AX046331.1 | GM658326.1 | HC305982.1 | GM645366.1 | GM637088.1 |
| CS118875.1 | DL087972.1 | AY745248.1 | HH979671.1 | AX044451.1 | GM658294.1 | HC302628.1 | GM645334.1 | GM637056.1 |
| CS118843.1 | DL087940.1 | AF003712.1 | HH976346.1 | AX041932.1 | GM658262.1 | HC305951.1 | GM645270.1 | GM624903.1 |
| CS118777.1 | DL087908.1 | AF518732.1 | HH999868.1 | AX037569.1 | GM658230.1 | HC305911.1 | GM645238.1 | GM624871.1 |
| CS118744.1 | DL087876.1 | KM396323.1 | HH999779.1 | AX035986.1 | GM658198.1 | HC305871.1 | GM638440.1 | GM624829.1 |
| CS118712.1 | DL087844.1 | HW399750.1 | HH999725.1 | DL021405.1 | GM658166.1 | HC305831.1 | GM635084.1 | GM632654.1 |
| CS118680.1 | DL087812.1 | HW399273.1 | HH998281.1 | DL017199.1 | GM650845.1 | HC305791.1 | GM630278.1 | GM635661.1 |
| CS118648.1 | DL121771.1 | HW381564.1 | HH998244.1 | DL017167.1 | GM650813.1 | HC305751.1 | GM630214.1 | GM635629.1 |
| CS118616.1 | DL121739.1 | HW390490.1 | HH998197.1 | DL017135.1 | GM658084.1 | HC305671.1 | GM630182.1 | GM635597.1 |
| CS118583.1 | DL121707.1 | HW390303.1 | HH998155.1 | DL017103.1 | GM658052.1 | HC305631.1 | GM630150.1 | GM635565.1 |
| HW399306.1 | DL121675.1 | HW389045.1 | HH996438.1 | DL017071.1 | GM658020.1 | HC305371.1 | GM630118.1 | GM635533.1 |
| HW408919.1 | DL121643.1 | HW387490.1 | HH979636.1 | DL017039.1 | GM657988.1 | HC301467.1 | GM698662.1 | GM635501.1 |
| HW390497.1 | DL121611.1 | HW155696.1 | HH979596.1 | DL012778.1 | GM657956.1 | HC301172.1 | GM698608.1 | GM635469.1 |
| HW390396.1 | DL087784.1 | HW144838.1 | HH932014.1 | DL012746.1 | GM650764.1 | HC255373.1 | GM698566.1 | GM630663.1 |
| HW388661.1 | DL087752.1 | HW155294.1 | FW394412.1 | DL012714.1 | GM650732.1 | HC251101.1 | GM635049.1 | GM630631.1 |
| HW388329.1 | DL087720.1 | HW155210.1 | FW394271.1 | DL012682.1 | GM650700.1 | HC291234.1 | GM635017.1 | GM630599.1 |
| HW260305.1 | DL087688.1 | HW154655.1 | FW416379.1 | DL012650.1 | GM643804.1 | HC289357.1 | GM634985.1 | GM621728.1 |
| HW260273.1 | DL087656.1 | HW154054.1 | FW394252.1 | DL012618.1 | GM643772.1 | HC289325.1 | GM634953.1 | GM621696.1 |
| HW260241.1 | DL087624.1 | HW153772.1 | FW394210.1 | DL049438.1 | GM643740.1 | HC289187.1 | GM634921.1 | GM621664.1 |
| HW260209.1 | DL087592.1 | HW153732.1 | FW394083.1 | DL049406.1 | GM643708.1 | HC296211.1 | GM634889.1 | GM621632.1 |
| HW260177.1 | DL125491.1 | HW153274.1 | FW398059.1 | DL049374.1 | GM643676.1 | HC295923.1 | GM629955.1 | GM621600.1 |
| HW260145.1 | DL125459.1 | KF036176.1 | FW397870.1 | DL049342.1 | GM643636.1 | HC292636.1 | GM629923.1 | GM621568.1 |
| HW260081.1 | DL125427.1 | HW126575.1 | FW393464.1 | DL049310.1 | GM636647.1 | HC288879.1 | GM642409.1 | FB505799.1 |
| HW260049.1 | DL125363.1 | HW125554.1 | FW392979.1 | DL049278.1 | GM636615.1 | HC295458.1 | GM642233.1 | GM657116.1 |
| HW260017.1 | DL121574.1 | HW125338.1 | FW418570.1 | DL049246.1 | GM636583.1 | HC288712.1 | GM642273.1 | GM657084.1 |
| HW259953.1 | DL121542.1 | HW144398.1 | FW396316.1 | DL049215.1 | GM636519.1 | HC295229.1 | GM656340.1 | GM657052.1 |
| HW259921.1 | DL121510.1 | HW144355.1 | FW395232.1 | DL049183.1 | GM636487.1 | HC292086.1 | GM656308.1 | GM657020.1 |
| HW258856.1 | DL121478.1 | HW144291.1 | HH936660.1 | DL049151.1 | GM636455.1 | HC291918.1 | GM656276.1 | GM656956.1 |
| HW257574.1 | DL121414.1 | HW124843.1 | HH834340.1 | DL049119.1 | GM623116.1 | HC299199.1 | GM656244.1 | GM656925.1 |
| HW257404.1 | DL139916.1 | HW124695.1 | HH833642.1 | DL049087.1 | GM623084.1 | HC299163.1 | GM656212.1 | GM656893.1 |
| HW257340.1 | DL117202.1 | HW117985.1 | HH833610.1 | DL049055.1 | GM623052.1 | HC291654.1 | GM656180.1 | GM656829.1 |
| HW257308.1 | DL117170.1 | HW101664.1 | HH833578.1 | DL012547.1 | GM623020.1 | HC294422.1 | GM649187.1 | GM656797.1 |

| | | | | | | | | |
|---|---|---|---|---|---|---|---|---|
| HW257244.1 | DL117138.1 | DL085938.1 | HH833546.1 | DL012515.1 | GM622988.1 | HC293858.1 | GM649155.1 | GM656765.1 |
| HW257212.1 | DL117106.1 | DL085874.1 | HH833514.1 | DL012483.1 | GM622956.1 | HC293678.1 | GM649123.1 | GM649772.1 |
| HW257180.1 | DL117074.1 | DL085842.1 | HH833482.1 | DL012451.1 | GM650474.1 | HC290725.1 | GM649091.1 | GM649740.1 |
| HW257052.1 | DL117042.1 | DL085810.1 | HH833418.1 | DL012419.1 | GM650442.1 | HC290264.1 | GM649059.1 | GM649708.1 |
| HW256956.1 | DL112454.1 | DL126709.1 | HH833386.1 | DL049039.1 | GM650410.1 | HC290178.1 | GM649027.1 | GM649612.1 |
| HW256796.1 | DL112422.1 | DL126677.1 | HC046155.1 | DL049007.1 | GM643602.1 | HC290146.1 | GM648995.1 | GM642708.1 |
| HW256764.1 | DL112390.1 | DL126645.1 | HC046123.1 | DL048975.1 | GM643538.1 | HC208433.1 | GM642192.1 | GM642676.1 |
| HW256732.1 | DL112358.1 | DL126613.1 | HC046005.1 | DL048943.1 | GM643506.1 | HC289887.1 | GM642160.1 | GM635334.1 |
| HW251044.1 | DL112326.1 | DL123358.1 | HC045973.1 | DL048911.1 | GM643474.1 | HC289853.1 | GM642128.1 | GM635302.1 |
| HW250844.1 | DL112294.1 | DL123326.1 | HC045941.1 | DL048879.1 | GM643441.1 | HC207999.1 | GM642096.1 | GM635270.1 |
| HW240604.1 | DL112658.1 | DL123294.1 | HC045909.1 | DL048847.1 | GM636452.1 | HC207386.1 | GM642064.1 | GM630464.1 |
| HW239291.1 | DL107280.1 | DL123262.1 | HC045877.1 | DL045429.1 | GM636420.1 | HC201493.1 | GM642032.1 | GM630432.1 |
| HW238386.1 | DL107248.1 | DL123230.1 | AX242328.1 | DL045397.1 | GM636388.1 | HC199363.1 | GM634845.1 | GM630400.1 |
| HW238248.1 | DL107216.1 | DL123198.1 | AX242296.1 | DL045365.1 | GM636356.1 | HC198000.1 | GM634813.1 | GM630368.1 |
| HW238024.1 | DL107184.1 | DL123166.1 | AX242264.1 | DL045333.1 | GM631852.1 | DM464480.1 | GM634781.1 | GM630336.1 |
| HW244234.1 | DL107152.1 | DL118976.1 | AX242232.1 | DL045301.1 | GM631820.1 | DM462439.1 | GM629879.1 | GM630304.1 |
| HW237923.1 | DL107120.1 | DL118944.1 | AX242200.1 | DL045269.1 | GM631788.1 | HC196176.1 | GM629847.1 | GM621497.1 |
| HW237878.1 | DL102475.1 | DL118912.1 | AX242136.1 | DL041253.1 | GM631756.1 | HC195241.1 | GM629815.1 | DL088430.1 |
| HW248659.1 | DL102443.1 | DL118880.1 | AX242072.1 | DL041221.1 | GM631724.1 | HC194266.1 | GM629783.1 | DL088398.1 |
| HV579102.1 | DL102411.1 | DL118848.1 | AX242008.1 | DL041189.1 | GM631692.1 | HC193680.1 | GM629751.1 | DL117798.1 |
| HV592438.1 | DL102379.1 | DL118816.1 | AX241976.1 | DL041157.1 | GM622921.1 | HC193421.1 | GM629719.1 | DL117766.1 |
| HV592374.1 | DL102347.1 | DL109264.1 | AX241944.1 | DL041125.1 | GM634265.1 | HC190874.1 | GM718475.1 | DL117734.1 |
| HV570623.1 | DL097734.1 | DL109232.1 | AX241880.1 | DL041093.1 | GM629268.1 | HC190440.1 | GM669625.1 | DL117702.1 |
| HV572472.1 | DL104526.1 | DL109200.1 | AX241848.1 | DL037270.1 | GM629204.1 | DM187986.1 | GM656128.1 | DL117670.1 |
| HV572323.1 | DL100112.1 | DL109168.1 | AX241784.1 | DL037238.1 | GM629172.1 | DM173761.1 | GM656096.1 | DL117638.1 |
| HV560182.1 | DL100080.1 | DL109136.1 | AX241752.1 | DL037206.1 | GM629140.1 | DM187475.1 | GM656064.1 | DL122097.1 |
| HV562069.1 | DL100048.1 | DL109104.1 | AX241720.1 | DL037174.1 | GM629108.1 | DM177084.1 | GM656032.1 | DL122065.1 |
| HV035558.1 | DL100016.1 | DL104498.1 | AX241688.1 | DL037142.1 | GM634038.1 | HB559382.1 | GM656000.1 | DL122033.1 |
| HV035242.1 | DL099984.1 | DL104466.1 | AX241656.1 | DL037110.1 | GM634006.1 | HB491604.1 | GM655968.1 | DL125742.1 |
| FZ435984.1 | DL099952.1 | DL104434.1 | AX241624.1 | DL032907.1 | GM633942.1 | HB489277.1 | GM648975.1 | DL125710.1 |
| FZ431251.1 | DL093910.1 | DL104402.1 | AX241592.1 | DL032875.1 | GM633910.1 | HB488838.1 | GM648911.1 | DL125678.1 |
| FZ426116.1 | DL093878.1 | DL104370.1 | AX241528.1 | DL032843.1 | GM633878.1 | HB488705.1 | GM648879.1 | DL125646.1 |
| FZ437284.1 | DL093814.1 | DL104338.1 | AX241464.1 | DL032811.1 | GM883094.1 | DM165768.1 | GM648847.1 | DL125614.1 |
| FZ428026.1 | DL093782.1 | DL099924.1 | AX241432.1 | DL032779.1 | GM655551.1 | DM161200.1 | GM642012.1 | DL125550.1 |
| FZ422904.1 | DL090103.1 | DL099892.1 | AX241125.1 | DL028739.1 | BD394859.1 | DM164906.1 | GM641980.1 | DL121958.1 |
| FZ421508.1 | DL090071.1 | DL099860.1 | AX241093.1 | DL028707.1 | BD404964.1 | DM164395.1 | GM641948.1 | DL121926.1 |
| FZ421408.1 | DL090039.1 | DL099828.1 | AX241061.1 | DL028675.1 | BD446174.1 | DM164033.1 | GM641916.1 | DL121894.1 |
| FZ417509.1 | DL090007.1 | DL099796.1 | AX241029.1 | DL028611.1 | BD453355.1 | DM167736.1 | GM641884.1 | DL121862.1 |
| FZ411466.1 | DL089975.1 | DL099764.1 | AX240997.1 | DL028579.1 | BD453325.1 | DM164018.1 | GM641852.1 | DL121830.1 |
| FZ419841.1 | DL087035.1 | DL099732.1 | AX240965.1 | DL028547.1 | BD453293.1 | DM163985.1 | GM634664.1 | DL121798.1 |
| FW591760.1 | DL087003.1 | DL093722.1 | AX240933.1 | DL024733.1 | BD456420.1 | DM163951.1 | GM634600.1 | DL031645.1 |
| HI424121.1 | DL124479.1 | DL093690.1 | AX236983.1 | DL024701.1 | BD455083.1 | DM163545.1 | GM634536.1 | DL031613.1 |
| HI424089.1 | DL124447.1 | DL093658.1 | AX235231.1 | DL024669.1 | BD444973.1 | DM163280.1 | GM634504.1 | DL031581.1 |
| HI424057.1 | DL124415.1 | DL093594.1 | AX224747.1 | DL024637.1 | BD444959.1 | DM163069.1 | GM634472.1 | DL031549.1 |
| HI423767.1 | DL124383.1 | DL093562.1 | AX207289.1 | DL024605.1 | BD376363.1 | DM162381.1 | GM629667.1 | DL027740.1 |
| HI422622.1 | DL120390.1 | DL089947.1 | AX202418.1 | DL024573.1 | BD375306.1 | DM162220.1 | GM629635.1 | DL027708.1 |
| HI416740.1 | DL120358.1 | DL089915.1 | AX197540.1 | DL021361.1 | BD374048.1 | HB484360.1 | GM629603.1 | DL027676.1 |

| | | | | | | | | |
|---|---|---|---|---|---|---|---|---|
| HI414024.1 | DL120326.1 | DL089883.1 | AF401227.1 | DL021329.1 | BD366175.1 | HB484327.1 | GM629571.1 | DL027644.1 |
| HI413833.1 | DL120294.1 | DL089851.1 | AX193644.1 | DL022844.1 | BD356470.1 | HB483649.1 | GM629539.1 | DL027612.1 |
| HI413615.1 | DL120262.1 | DL089819.1 | AX188585.1 | DL022812.1 | BD355122.1 | HB483617.1 | GM629507.1 | DL027580.1 |
| HI413580.1 | DL120230.1 | DL085750.1 | AX174629.1 | DL019789.1 | BD359062.1 | HB477213.1 | GM696796.1 | DL023768.1 |
| HI516099.1 | DL120198.1 | DL085718.1 | AX175197.1 | DL019733.1 | BD342506.1 | HB477170.1 | FB711320.1 | DL023736.1 |
| HI284286.1 | DL115795.1 | DL085654.1 | AX172939.1 | DL019701.1 | BD341873.1 | HB477138.1 | GM967131.1 | DL023704.1 |
| HI574507.1 | DL115763.1 | DL137544.1 | AX168187.1 | DL019669.1 | BD349527.1 | HB477100.1 | FB709616.1 | DL023672.1 |
| HI574475.1 | DL115731.1 | DL123143.1 | AX167410.1 | DL019637.1 | DD162628.1 | HB477036.1 | GM040638.1 | DL023640.1 |
| HI574443.1 | DL115699.1 | DL123111.1 | AX167101.1 | DL019605.1 | DD161084.1 | HB477004.1 | GM839360.1 | DL023608.1 |
| HI574411.1 | DL115667.1 | DL123079.1 | AX166325.1 | DL015202.1 | DD178467.1 | HB476910.1 | CS627021.1 | DL023576.1 |
| HI574379.1 | DL115635.1 | DL123047.1 | AX155101.1 | DL015170.1 | DD171929.1 | HB476887.1 | CS625900.1 | DL020561.1 |
| HI574356.1 | DL111241.1 | DL123015.1 | AX149410.1 | DL015138.1 | DD165524.1 | HB476788.1 | DD450360.1 | DL020529.1 |
| HI566246.1 | DL111209.1 | DL122983.1 | AX146641.1 | DL015106.1 | DD165442.1 | HB476550.1 | DD450125.1 | DL020497.1 |
| HI636992.1 | DL111145.1 | DL118793.1 | HW237894.1 | DL015042.1 | DD165152.1 | HB476517.1 | DD449094.1 | DL020465.1 |
| HI001465.1 | DL111113.1 | DL118761.1 | HW248690.1 | DL010584.1 | DD171276.1 | HB485601.1 | DD437088.1 | DL020433.1 |
| HI001369.1 | DL111081.1 | DL118729.1 | HW241646.1 | DL010552.1 | DD170793.1 | HB485258.1 | DD436503.1 | DL015998.1 |
| HI001335.1 | DL106277.1 | DL118697.1 | HW243123.1 | DL010520.1 | DD182186.1 | HB485454.1 | CS617012.1 | DL015966.1 |
| HI003243.1 | DL106245.1 | DL118665.1 | HW163854.1 | DL010488.1 | DD175953.1 | HB475234.1 | CS616548.1 | DL015934.1 |
| HI003172.1 | DL106181.1 | DL118633.1 | HW160674.1 | DL010456.1 | DD175921.1 | HB474889.1 | CS613765.1 | DL015902.1 |
| HI003109.1 | DL106149.1 | DL114237.1 | HW160626.1 | DL010424.1 | CS204327.1 | HB464824.1 | CS612791.1 | DL015870.1 |
| HI003060.1 | DL106117.1 | DL114205.1 | HW160594.1 | DL046821.1 | CS001490.1 | HB463821.1 | CS611857.1 | DL015838.1 |
| HI003024.1 | DL101504.1 | DL114173.1 | HW155902.1 | DL046789.1 | CS001394.1 | HB463636.1 | CS604549.1 | DL011252.1 |
| HI002963.1 | DL101472.1 | DL114141.1 | HW155626.1 | DL046757.1 | CS000441.1 | HB471287.1 | CS604517.1 | DL011220.1 |
| HI002923.1 | DL101440.1 | DL114109.1 | HW150005.1 | DL046725.1 | AF408183.1 | HB469608.1 | CS604485.1 | DL047644.1 |
| HI001287.1 | DL101408.1 | DL114077.1 | HW149973.1 | DL046661.1 | CS161105.1 | HB469113.1 | CS604421.1 | DL047612.1 |
| HI001191.1 | DL101376.1 | DL118608.1 | HW144802.1 | DL042839.1 | CS157802.1 | GM968447.1 | CS604357.1 | DL047580.1 |
| HI001126.1 | DL094745.1 | DL126217.1 | HW155230.1 | DJ011360.1 | CS157770.1 | GM980676.1 | CS604325.1 | DL047548.1 |
| HI001093.1 | DL110910.1 | DL021297.1 | HW159067.1 | DD491859.1 | CS157931.1 | GN007502.1 | CS604197.1 | DL039653.1 |
| HC471628.1 | DL110878.1 | DL021265.1 | HW158779.1 | DD495649.1 | CS157899.1 | GM999361.1 | CS604037.1 | DL039621.1 |
| HC731039.1 | DL106074.1 | DL021233.1 | HW154732.1 | CS671137.1 | CS146885.1 | FB986400.1 | CS603909.1 | DL039589.1 |
| HC728201.1 | DL106042.1 | DL021201.1 | HW154643.1 | CS671001.1 | CS144060.1 | FB741884.1 | CS603813.1 | DL039557.1 |
| AY657593.1 | DL106010.1 | DL016995.1 | HW154476.1 | CS668172.1 | CS126615.1 | GM969706.1 | CS603749.1 | DL039525.1 |
| AY657561.1 | DL105978.1 | DL016963.1 | HW147478.1 | DD467995.1 | CS124724.1 | GM970193.1 | CS603461.1 | DL039493.1 |
| AY657529.1 | DL105946.1 | DL016931.1 | HV940315.1 | DD464411.1 | CS124686.1 | FB736416.1 | CS603429.1 | DL047319.1 |
| AY657497.1 | DL105914.1 | DL016899.1 | HV940043.1 | DD463046.1 | CS124646.1 | GM969862.1 | CS603397.1 | DL047287.1 |
| AY657465.1 | DL101301.1 | DL016867.1 | HV939945.1 | CS646206.1 | CS124602.1 | GM969743.1 | CS603365.1 | DL043465.1 |
| AY657433.1 | DL101269.1 | DL016835.1 | HV815883.1 | CS645255.1 | CS123921.1 | FB778025.1 | CS603333.1 | DL043433.1 |
| AY657401.1 | DL101237.1 | DL012345.1 | HV932239.1 | CS645218.1 | CS119549.1 | FB777906.1 | CS603301.1 | DL043401.1 |
| AY657369.1 | DL101205.1 | DL012313.1 | HV931862.1 | DD309682.1 | CS119483.1 | GM009522.1 | CS603269.1 | DL043369.1 |
| AY657337.1 | DL101173.1 | DL012281.1 | HV937013.1 | DD309044.1 | CS119415.1 | DL480009.1 | CS603237.1 | DL043337.1 |
| AY657305.1 | DL088910.1 | DL012249.1 | HV931269.1 | DD308863.1 | CS119350.1 | DL479436.1 | CS603205.1 | DL043273.1 |
| AY657273.1 | DL088846.1 | DL060846.1 | HV936295.1 | DD308859.1 | CS119318.1 | DL465765.1 | CS603173.1 | DL039456.1 |
| AY657241.1 | DL088814.1 | DL048826.1 | HV803285.1 | DD292657.1 | CS119284.1 | DL477850.1 | CS603141.1 | DL039424.1 |
| AY657209.1 | DL088782.1 | DL048794.1 | HV932625.1 | DD291761.1 | CS119252.1 | DL464782.1 | CS603109.1 | DL039392.1 |
| AY657177.1 | DL103285.1 | DL048730.1 | HV929795.1 | DD291549.1 | CS119188.1 | DL464577.1 | CS603077.1 | DL039360.1 |
| AY657145.1 | DL103253.1 | DL048698.1 | HV932329.1 | DD291026.1 | CS119156.1 | DL464529.1 | CS603045.1 | DL039328.1 |
| AY657113.1 | DL103221.1 | DL048666.1 | HV704989.1 | CS377481.1 | CS119090.1 | DL470989.1 | CS603013.1 | DL039296.1 |

| | | | | | | | | |
|---|---|---|---|---|---|---|---|---|
| AY657081.1 | DL098711.1 | DL045248.1 | HV704662.1 | CS376993.1 | CS119057.1 | DL477038.1 | CS602979.1 | DL035672.1 |
| AY657049.1 | DL098679.1 | DL045216.1 | HV704446.1 | CS376012.1 | CS118959.1 | DL116120.1 | CS602947.1 | DL035640.1 |
| U36598.1 | DL098647.1 | DL045184.1 | HV704185.1 | CS375822.1 | CS118926.1 | DL116088.1 | CS602915.1 | DL035608.1 |
| M24507.1 | DL098615.1 | DL045152.1 | HV704110.1 | CS362754.1 | CS118893.1 | DL116056.1 | CS602883.1 | DL035576.1 |
| K00896.1 | DL098583.1 | DL045120.1 | HV744584.1 | CS359949.1 | CS118860.1 | DL143520.1 | CS602851.1 | DL035544.1 |
| DM092265.1 | DL098551.1 | DL045088.1 | HV703448.1 | CS359647.1 | CS118794.1 | DL120580.1 | CS602787.1 | DL035512.1 |
| DM103492.1 | DL108069.1 | GN359415.1 | HV702437.1 | CS362336.1 | CS118729.1 | DL120548.1 | CS602755.1 | DL031508.1 |
| GN368266.1 | DL107973.1 | GN359383.1 | HV701853.1 | BD269152.1 | CS118633.1 | DL120516.1 | CS602723.1 | DL031444.1 |
| GN373352.1 | DL107941.1 | GN359351.1 | HV701245.1 | BD268979.1 | CS118601.1 | DL120484.1 | AX699466.1 | DL031412.1 |
| GN360138.1 | DL107909.1 | GN359319.1 | HV701213.1 | BD268778.1 | CS118567.1 | DL120452.1 | AX699434.1 | DL031380.1 |
| GN356187.1 | DL092483.1 | GN359287.1 | HV701149.1 | BD268134.1 | CS118533.1 | DL120420.1 | AX682975.1 | DL031348.1 |
| GN360001.1 | DL092451.1 | GN365288.1 | HV701117.1 | BD267698.1 | CS118500.1 | DL116017.1 | AX671502.1 | DL027443.1 |
| GN359905.1 | DL092419.1 | GN364299.1 | HV708712.1 | BD266844.1 | CS118467.1 | DL115985.1 | AX665488.1 | DL027411.1 |
| GN359860.1 | DL092387.1 | GN359278.1 | HV701066.1 | BD265630.1 | CS118402.1 | DL115977.1 | AX664348.1 | DD385289.1 |
| GN359828.1 | DL088772.1 | GN359246.1 | HV701034.1 | BD265529.1 | CS118370.1 | DL115889.1 | AX662236.1 | DD382838.1 |
| GN359764.1 | DL088740.1 | GN359214.1 | HV695528.1 | BD263698.1 | CS118335.1 | DL115857.1 | AX662064.1 | DD400162.1 |
| GN359732.1 | DL088708.1 | GN359150.1 | HV695015.1 | BD263462.1 | CS118301.1 | DL111463.1 | AX657148.1 | DD400130.1 |
| GN359700.1 | DL088676.1 | GN363930.1 | HV694910.1 | BD263430.1 | CS118237.1 | DL111431.1 | AX657116.1 | CS459110.1 |
| GN359680.1 | DL088644.1 | GN358958.1 | DJ433907.1 | BD263348.1 | CS118204.1 | DL111399.1 | AX657074.1 | DD361293.1 |
| GN359584.1 | DL088580.1 | GN363576.1 | DJ438353.1 | BD251243.1 | CS118170.1 | DL111367.1 | BD175983.1 | DD367622.1 |
| GN359420.1 | DL113022.1 | GN363041.1 | DJ438319.1 | BD250820.1 | CS118138.1 | DL111335.1 | BD175651.1 | DD367334.1 |
| GN359388.1 | DL112990.1 | GN362730.1 | DJ438286.1 | BD249420.1 | CS118106.1 | DL111303.1 | BD174680.1 | DD362920.1 |
| GN359324.1 | DL112958.1 | GN346517.1 | DJ438204.1 | BD247881.1 | CS118073.1 | DL111271.1 | BD174396.1 | DD362234.1 |
| GN365382.1 | DL112926.1 | GN346483.1 | DJ427967.1 | BD247022.1 | CS118040.1 | DL106467.1 | AX645183.1 | HV961667.1 |
| GN359091.1 | DL112862.1 | GN344770.1 | DJ402679.1 | BD246896.1 | CS118005.1 | DL106353.1 | AJ005288.1 | HV957837.1 |
| GM831904.1 | DL098507.1 | GN346590.1 | DJ402641.1 | BD244810.1 | CS115043.1 | DL106395.1 | AX642225.1 | HV957337.1 |
| GM744863.1 | DL098475.1 | GN346558.1 | DJ402543.1 | BD243417.1 | CS114663.1 | DL106339.1 | AX601592.1 | HV947291.1 |
| GM658868.1 | DL098443.1 | GN340525.1 | DJ402301.1 | BD243385.1 | CQ888085.1 | DL106307.1 | AX601488.1 | HV953248.1 |
| GM622864.1 | DL098411.1 | GN340098.1 | DJ400834.1 | BD242810.1 | CQ882112.1 | DL136030.1 | AX601455.1 | HV757533.1 |
| FB507557.1 | DL098379.1 | GN339141.1 | DJ400802.1 | BD242566.1 | CQ879660.1 | DL128482.1 | AX599030.1 | HV755399.1 |
| GM640990.1 | DL098347.1 | GN130794.1 | DJ399775.1 | BD242467.1 | CQ877823.1 | DL124752.1 | AX598984.1 | HV750067.1 |
| GM654807.1 | DL092337.1 | GN116537.1 | BD237846.1 | BD242387.1 | CQ877237.1 | DL124720.1 | AX598876.1 | HV758622.1 |
| GM654775.1 | DL092273.1 | GN116505.1 | BD237336.1 | BD238058.1 | CQ876144.1 | DL124688.1 | AX598824.1 | HV749703.1 |
| GM715038.1 | DL092209.1 | GN116472.1 | BD235976.1 | BD237930.1 | CQ875542.1 | DL124656.1 | AX598774.1 | HV752224.1 |
| DL202574.1 | DL092177.1 | GN116440.1 | BD235793.1 | BD237565.1 | CQ874723.1 | DL124624.1 | AX593378.1 | HV749591.1 |
| DL188838.1 | DL088562.1 | GN116408.1 | BD235643.1 | BD236915.1 | CQ873295.1 | DL124592.1 | AX587860.1 | HV748483.1 |
| DL193808.1 | DL088530.1 | GN116376.1 | BD234430.1 | BD235855.1 | CQ871422.1 | DL124560.1 | AX587762.1 | HV747612.1 |
| FB571341.1 | DL088498.1 | GN116344.1 | BD233443.1 | BD235658.1 | CQ869300.1 | DL096927.1 | AX587670.1 | HV747474.1 |
| FB570990.1 | DL088466.1 | GN116312.1 | BD232020.1 | BD235378.1 | CQ868934.1 | DL096895.1 | AX587564.1 | HV750782.1 |
| FB572982.1 | DL088434.1 | GM633262.1 | BD231850.1 | BD234885.1 | CQ868352.1 | DL096863.1 | AX591633.1 | HV753567.1 |
| CS696177.1 | DL117802.1 | GM633230.1 | BD231165.1 | BD234579.1 | CS604797.1 | DL096831.1 | AX591041.1 | HV753485.1 |
| CS696145.1 | DL117770.1 | GM633198.1 | BD229097.1 | BD233595.1 | CS604765.1 | DL096799.1 | BD162056.1 | HV743308.1 |
| CS696113.1 | DL117738.1 | GM633166.1 | BD227216.1 | BD232426.1 | CS604733.1 | DL094939.1 | BD161134.1 | HV743272.1 |
| CS696016.1 | DL117706.1 | GM633134.1 | BD226909.1 | BD231104.1 | CS604701.1 | DL094907.1 | AX587992.1 | HV743233.1 |
| CS695984.1 | DL117674.1 | GM633102.1 | BD226415.1 | BD229941.1 | CS604669.1 | DL094875.1 | AX587912.1 | HV704995.1 |
| CS695888.1 | DL122165.1 | GM625523.1 | BD225783.1 | BD227588.1 | CS604635.1 | DL094843.1 | AX587864.1 | HV704450.1 |
| CS695856.1 | DL122133.1 | GM625491.1 | BD225751.1 | BD227178.1 | CS604603.1 | DL094811.1 | AX587763.1 | HV549123.1 |

| | | | | | | | | |
|---|---|---|---|---|---|---|---|---|
| CS695824.1 | DL122101.1 | GM625459.1 | BD225719.1 | BD227032.1 | CS604571.1 | DL094779.1 | AX587710.1 | HV549085.1 |
| CS695760.1 | DL122037.1 | GM625427.1 | BD225687.1 | BD226662.1 | CS597505.1 | DL094747.1 | AX587609.1 | HV548996.1 |
| CS695728.1 | DL092159.1 | GM625395.1 | BD225655.1 | BD225793.1 | CS592925.1 | DL091132.1 | AX587558.1 | HV544347.1 |
| CS695664.1 | DL125714.1 | GM625363.1 | BD225623.1 | BD225761.1 | CS592802.1 | DL091100.1 | AX577825.1 | HV544106.1 |
| DL176467.1 | DL125682.1 | GM625331.1 | BD224831.1 | BD225729.1 | CS592323.1 | DL091068.1 | AX575309.1 | HV543618.1 |
| DL176385.1 | DL125650.1 | GM659286.1 | BD224127.1 | BD225697.1 | CS598817.1 | DL091036.1 | AX573565.1 | HV543499.1 |
| DL176318.1 | DL125618.1 | GM659254.1 | BD223249.1 | BD225665.1 | CS597732.1 | DL091004.1 | AX103781.1 | HV539680.1 |
| DL175296.1 | DL125586.1 | GM659222.1 | BD221643.1 | BD225633.1 | CS585642.1 | DL090972.1 | AX103646.1 | HV538611.1 |
| DL174426.1 | DL125554.1 | GM659158.1 | BD194682.1 | BD225172.1 | CS584999.1 | DL087164.1 | AX100416.1 | HV538455.1 |
| FB360093.1 | DL121962.1 | GM711169.1 | DD282934.1 | BD223671.1 | CS582429.1 | DL087132.1 | AX099993.1 | HV537607.1 |
| FB342845.1 | DL121930.1 | GM651755.1 | DD280727.1 | DD086347.1 | CS588488.1 | DL087100.1 | AX097522.1 | HV542751.1 |
| CS607543.1 | DL121898.1 | GM651723.1 | DD279442.1 | DD099925.1 | CS574792.1 | DL087068.1 | AX097488.1 | HV542719.1 |
| CS607208.1 | DL121866.1 | GM651691.1 | CS356866.1 | DD069887.1 | CS573118.1 | DL087036.1 | AX093160.1 | HV542687.1 |
| CS607031.1 | DL121834.1 | GM651659.1 | CS355969.1 | DD143283.1 | CS572367.1 | DL087004.1 | AX092950.1 | HV542182.1 |
| CS606942.1 | DL121802.1 | GM651627.1 | CS350543.1 | DD112411.1 | CS546784.1 | DL124544.1 | AX088803.1 | HV541494.1 |
| CS606809.1 | DL117603.1 | GM651594.1 | CS284464.1 | DD098193.1 | CS545393.1 | DL124512.1 | HW291071.1 | AY346129.1 |
| CS605123.1 | DL117571.1 | GM644795.1 | CS283934.1 | DD142050.1 | CS544819.1 | DL124448.1 | HW291039.1 | HV515059.1 |
| CS604968.1 | DL117539.1 | GM644763.1 | CS277024.1 | DD132487.1 | CS561182.1 | DL124416.1 | HW291007.1 | HV533076.1 |
| CS592999.1 | DL117507.1 | GM644731.1 | CS275987.1 | DD141485.1 | CS561007.1 | DL124384.1 | HW290975.1 | HV532756.1 |
| CS589287.1 | DL117475.1 | GM637637.1 | CS253537.1 | DD153590.1 | CS560460.1 | DL120391.1 | HW298875.1 | HV536539.1 |
| CS588641.1 | DL117443.1 | GM637605.1 | CS252553.1 | DD158425.1 | CS559244.1 | DL120359.1 | HW290880.1 | HV534658.1 |
| CS575760.1 | DL112732.1 | GM637573.1 | CS253979.1 | DD157381.1 | DD419841.1 | DL120295.1 | HW290848.1 | HV532476.1 |
| CS574932.1 | DL112700.1 | GM637541.1 | CS249842.1 | DD152128.1 | DD420059.1 | DL126185.1 | HW298195.1 | HV535998.1 |
| CS544234.1 | DL112668.1 | GM637509.1 | CS250643.1 | DD097173.1 | DD421792.1 | DL126153.1 | HW298048.1 | HV532381.1 |
| CS546672.1 | DL107863.1 | GM637477.1 | DD181023.1 | DD082054.1 | DD433777.1 | DL122937.1 | HW289024.1 | HV537346.1 |
| DD420107.1 | DL107831.1 | GM633069.1 | DD163283.1 | DD081765.1 | CS541620.1 | DL122905.1 | HW285406.1 | HV537226.1 |
| DD421551.1 | DL011775.1 | GM633037.1 | DD174347.1 | DD148512.1 | CS542559.1 | DL122841.1 | HW279584.1 | HV531941.1 |
| CS540028.1 | DL011743.1 | GM633005.1 | DD161531.1 | DD147798.1 | CS537971.1 | DL122777.1 | HW267843.1 | HV515586.1 |
| E03811.1 | DL011711.1 | GM632973.1 | DD167021.1 | DD147766.1 | CS534838.1 | DL118555.1 | HW267798.1 | HV515554.1 |
| E03557.1 | DL011679.1 | GM632941.1 | DD173109.1 | DD147734.1 | CS510548.1 | DL118523.1 | HW267601.1 | HV515522.1 |
| E03322.1 | DL011647.1 | GM632909.1 | DD172322.1 | DD147702.1 | CS502906.1 | DL118491.1 | HW265990.1 | HV515490.1 |
| E03074.1 | DL011615.1 | GM632877.1 | DD165146.1 | DD147670.1 | CS502742.1 | DL118459.1 | HW262997.1 | HV515458.1 |
| E02944.1 | DL048598.1 | GM625266.1 | DD171110.1 | CQ871426.1 | CS502710.1 | DL118427.1 | HW262965.1 | HV515426.1 |
| E02690.1 | DL048566.1 | GM625234.1 | DD164868.1 | CQ871372.1 | E01919.1 | DL114031.1 | HW262917.1 | HV515394.1 |
| E02538.1 | DL048534.1 | GM625202.1 | DD163504.1 | CQ871105.1 | E01681.1 | DL113999.1 | HW262874.1 | HV515362.1 |
| E01698.1 | DL048502.1 | GM624112.1 | CS203978.1 | CQ870565.1 | E01239.1 | DL113967.1 | HW262574.1 | HV515330.1 |
| E01388.1 | DL048470.1 | GM624048.1 | CS001388.1 | CQ870527.1 | E01033.1 | DL113935.1 | HW261408.1 | HV515298.1 |
| HI002535.1 | DL044853.1 | GM624016.1 | AF405704.1 | CQ869304.1 | E00872.1 | DL113903.1 | HW261376.1 | HV515266.1 |
| HI001488.1 | DL044821.1 | GM623984.1 | CS157796.1 | CQ869272.1 | DD102383.1 | DL113871.1 | HW261344.1 | HV515234.1 |
| HI004489.1 | DL044789.1 | GM623952.1 | CS157957.1 | CQ868855.1 | DD146374.1 | DL109070.1 | HW261312.1 | HV510389.1 |
| HI004425.1 | DL044757.1 | GM632865.1 | CS157925.1 | CQ868388.1 | DD129364.1 | DL109038.1 | HW261248.1 | HV510357.1 |
| HI002455.1 | DL044725.1 | GM632833.1 | CS157829.1 | CQ867268.1 | DD143580.1 | DL109006.1 | HW261216.1 | HV513363.1 |
| HI002401.1 | DL044693.1 | GM632801.1 | CS143643.1 | CQ858721.1 | DD084820.1 | DL108974.1 | HW261184.1 | HV513331.1 |
| HI002322.1 | DL044637.1 | GM632769.1 | CS126232.1 | CQ858090.1 | DD069879.1 | DL108942.1 | HW261152.1 | HV513079.1 |
| HI000527.1 | DL044605.1 | GM632705.1 | CS124718.1 | CQ857846.1 | DD112550.1 | DL108910.1 | HW261120.1 | HV508594.1 |
| HI183838.1 | DL044573.1 | GM625126.1 | CS124678.1 | CQ857715.1 | DD143238.1 | DL104304.1 | HW261088.1 | HV508562.1 |
| HI178355.1 | DL044541.1 | GM625094.1 | CS124596.1 | CQ857503.1 | DD098771.1 | DL104272.1 | HW261056.1 | HV508530.1 |

| | | | | | | | | |
|---|---|---|---|---|---|---|---|---|
| HI180168.1 | DL044509.1 | GM625062.1 | CS120373.1 | CQ857373.1 | DD084332.1 | DL104240.1 | HW261024.1 | HV508646.1 |
| HI178216.1 | DL044477.1 | GM625030.1 | CS119543.1 | CQ855758.1 | DD098180.1 | DL104208.1 | HW260992.1 | HV508470.1 |
| HI178095.1 | DL040461.1 | GM624998.1 | CS119509.1 | CQ854725.1 | DD083481.1 | DL104176.1 | HW260960.1 | FW577833.1 |
| HI202840.1 | DL040429.1 | GM624966.1 | CS119477.1 | CQ854061.1 | DD141283.1 | DL104144.1 | HW260928.1 | FW577577.1 |
| HI177873.1 | DL040397.1 | GM624934.1 | CS119442.1 | CQ848109.1 | DD158429.1 | DL104112.1 | HW260896.1 | FW576726.1 |
| HI214568.1 | DL040365.1 | GM623908.1 | CS119409.1 | CQ840848.1 | DD158381.1 | DL099698.1 | HW260864.1 | FW576689.1 |
| HI214536.1 | DL040333.1 | GM623876.1 | CS119344.1 | CQ840603.1 | DD157379.1 | DL099666.1 | HW260832.1 | FW576597.1 |
| HI214438.1 | DL040301.1 | GM623844.1 | CS119310.1 | CQ831798.1 | DD157352.1 | DL099634.1 | HW260800.1 | FW575887.1 |
| HI179717.1 | DL036677.1 | GM623812.1 | CS119278.1 | CQ830741.1 | DD155772.1 | DL099602.1 | HW260768.1 | FW575363.1 |
| HI213011.1 | DL036613.1 | GM623780.1 | CS119246.1 | CQ828284.1 | DD153645.1 | DL099570.1 | HW260736.1 | FW571672.1 |
| HI212965.1 | DL036581.1 | GM623748.1 | CS119214.1 | CQ827912.1 | DD152591.1 | DL099538.1 | HW260704.1 | FW572723.1 |
| HI212612.1 | DL036549.1 | GM658927.1 | CS119182.1 | CQ827528.1 | DD152100.1 | DL093528.1 | HW260672.1 | FW573697.1 |
| HI547160.1 | DL036517.1 | GM658895.1 | CS119118.1 | CQ826993.1 | DD096956.1 | DL093496.1 | HW260640.1 | FW568970.1 |
| HI546323.1 | DL032314.1 | GM658863.1 | CS119083.1 | CQ821899.1 | DD096738.1 | DL093432.1 | HW260608.1 | FW574727.1 |
| HI546287.1 | DL032282.1 | GM658831.1 | CS119050.1 | CQ818585.1 | DD081757.1 | DL093400.1 | HW260576.1 | FW572584.1 |
| HI586987.1 | DL032250.1 | GM651575.1 | CS118985.1 | CQ818019.1 | DD148189.1 | DL093368.1 | HW260544.1 | FW573472.1 |
| HI211001.1 | DL032218.1 | GM651543.1 | CS118953.1 | CQ816986.1 | DD147790.1 | DL089753.1 | HW260512.1 | FW572212.1 |
| HI210969.1 | DL032186.1 | GM651511.1 | CS118887.1 | CQ816945.1 | DD147758.1 | DL089721.1 | HV308791.1 | FW572024.1 |
| HI210937.1 | DL032154.1 | GM651479.1 | CS118854.1 | CQ815502.1 | DD147726.1 | DL089689.1 | HV311089.1 | FW571775.1 |
| HI566158.1 | DL048451.1 | GM651447.1 | CS118788.1 | CQ814103.1 | DD147694.1 | DL089657.1 | HV312305.1 | HI968142.1 |
| HI560780.1 | DL048424.1 | GM651415.1 | CS118756.1 | CQ814071.1 | DD147141.1 | DL089625.1 | HV226852.1 | HI967434.1 |
| HI473062.1 | DL048392.1 | GM651383.1 | CS118659.1 | CQ814038.1 | DD138758.1 | DL089593.1 | HV208284.1 | HI968440.1 |
| HI472910.1 | DL048360.1 | GM644584.1 | CS118595.1 | CQ814006.1 | DD137685.1 | DL126145.1 | HV309571.1 | HI979297.1 |
| HI004315.1 | DL048328.1 | GM644552.1 | CS118560.1 | CQ813974.1 | DD132680.1 | DL126113.1 | HV040129.1 | HI989483.1 |
| HI002279.1 | DL044442.1 | GM644520.1 | DD325345.1 | CQ813942.1 | DD107460.1 | DL126081.1 | HV097553.1 | HI989099.1 |
| HI002242.1 | DL044410.1 | GM644488.1 | CS389275.1 | CQ813910.1 | DD107345.1 | DL126049.1 | HV188524.1 | HI002714.1 |
| HI002192.1 | DL044346.1 | GM639671.1 | CS399776.1 | CQ813878.1 | DD093949.1 | DL126017.1 | HV182490.1 | HI002665.1 |
| HI002149.1 | DL044314.1 | GM639639.1 | CS389187.1 | CQ813846.1 | DD106661.1 | DL125985.1 | HV182458.1 | HI002630.1 |
| HI002111.1 | DL044282.1 | GM627060.1 | CS401602.1 | CQ813814.1 | DD092955.1 | DL125953.1 | HV182426.1 | HI002596.1 |
| HI000456.1 | DL040106.1 | GM626996.1 | CS406188.1 | CQ813781.1 | DD106229.1 | DL122761.1 | HV182394.1 | HB864926.1 |
| HI000408.1 | DL032113.1 | GM626964.1 | CS406591.1 | CQ813749.1 | CQ813870.1 | DL122705.1 | HV221025.1 | HB864894.1 |
| HI000366.1 | DL032081.1 | GM626932.1 | CS402169.1 | CQ813693.1 | CQ813838.1 | DL122673.1 | HV117842.1 | HB864862.1 |
| HI000309.1 | DL032049.1 | GM646598.1 | CS402066.1 | CQ813611.1 | CQ813806.1 | DL122641.1 | HV117788.1 | HB864830.1 |
| HC869608.1 | DL032017.1 | GM646562.1 | CS401821.1 | CQ807283.1 | CQ813733.1 | DL122609.1 | HV112791.1 | HB864798.1 |
| HC869544.1 | DL031985.1 | GM646530.1 | CS382102.1 | CQ807219.1 | CQ812867.1 | DL122577.1 | HV038758.1 | HB864250.1 |
| HC869484.1 | DL031953.1 | GM646498.1 | CS380068.1 | CQ807165.1 | CQ806532.1 | DL118387.1 | HV038596.1 | HB866965.1 |
| HC876931.1 | DL028144.1 | GM646466.1 | CS382610.1 | CQ803167.1 | CQ802877.1 | DL118355.1 | HV182356.1 | HB866513.1 |
| HC876893.1 | DL028112.1 | GM646434.1 | CS376545.1 | CQ803017.1 | CQ802132.1 | DL118323.1 | HV182324.1 | HB866098.1 |
| FW304553.1 | DL028048.1 | GM639631.1 | DD298337.1 | CQ802144.1 | CQ802028.1 | DL118291.1 | HV182292.1 | HB981732.1 |
| FW310251.1 | DL028016.1 | GM639599.1 | DD292511.1 | CQ801235.1 | CQ800997.1 | DL118259.1 | HV193487.1 | HC000746.1 |
| FW331839.1 | DL027984.1 | GM639567.1 | DD313243.1 | CQ800802.1 | CQ800794.1 | DL118227.1 | HV202567.1 | HB976603.1 |
| FW309614.1 | DL027952.1 | GM639535.1 | DD290970.1 | CQ800328.1 | CQ795455.1 | DL113831.1 | HV225851.1 | HB976179.1 |
| FW308793.1 | DL036462.1 | GM639503.1 | DD319815.1 | CQ799138.1 | CQ793260.1 | DL113725.1 | HV234590.1 | DM193335.1 |
| FW334257.1 | DL036430.1 | GM639495.1 | CS375990.1 | CQ797798.1 | CQ792319.1 | DL113759.1 | HV225411.1 | DM190419.1 |
| FW333968.1 | DL036398.1 | GM639439.1 | CS375793.1 | AX593487.1 | CQ789541.1 | DL113703.1 | HV312967.1 | DM189854.1 |
| FW307466.1 | DL036366.1 | GM626892.1 | CS366515.1 | AX587876.1 | CQ787486.1 | DL113671.1 | HV302094.1 | HB811486.1 |
| FW306900.1 | DL036334.1 | GM626860.1 | CS360570.1 | AX587782.1 | DL096226.1 | DL108870.1 | HI653871.1 | HB805648.1 |

| | | | | | | | | |
|---|---|---|---|---|---|---|---|---|
| FW306470.1 | DL036302.1 | GM626828.1 | CS359739.1 | AX587688.1 | DL096194.1 | DL108838.1 | HI653803.1 | HB839902.1 |
| FW306110.1 | DL044249.1 | GM626796.1 | BD269140.1 | AX587583.1 | DL096162.1 | DL108806.1 | HI653710.1 | HB847922.1 |
| FW305365.1 | DL044217.1 | GM626764.1 | BD268911.1 | AX592520.1 | DL120182.1 | DL108774.1 | HI653525.1 | HB847374.1 |
| HC769800.1 | DL044185.1 | GM626732.1 | BD268161.1 | AX591144.1 | DL120150.1 | DL108742.1 | HI653469.1 | HB832659.1 |
| HC767818.1 | DD359104.1 | GM653611.1 | BD268118.1 | AX590282.1 | DL120118.1 | DL108710.1 | HI653410.1 | HB838581.1 |
| HC767344.1 | DD357894.1 | GM653579.1 | BD266637.1 | AX589091.1 | DL120086.1 | DL104022.1 | HI653359.1 | HB846504.1 |
| HC755781.1 | DD357637.1 | GM660776.1 | BD265611.1 | BD161768.1 | DL120022.1 | DL104032.1 | HI653274.1 | HB846122.1 |
| HC734406.1 | DD357471.1 | GM660744.1 | BD263450.1 | BD161736.1 | DL094244.1 | DL103976.1 | HI651907.1 | HB838057.1 |
| HC733771.1 | DD355818.1 | GM653525.1 | BD262931.1 | BD161704.1 | DL094212.1 | DL103944.1 | HI651039.1 | HB845640.1 |
| HC733694.1 | DD355436.1 | GM653493.1 | BD261816.1 | BD161672.1 | DL107900.1 | DL103912.1 | HI650459.1 | HB845407.1 |
| HC732148.1 | CS448463.1 | GM653461.1 | BD248924.1 | BD161640.1 | DL092442.1 | DL099498.1 | HI646883.1 | DM001680.1 |
| HC471775.1 | CS448330.1 | GM653429.1 | BD248324.1 | BD161544.1 | DL092410.1 | DL099466.1 | HH807303.1 | DM001474.1 |
| HC471743.1 | CS447058.1 | GM653397.1 | BD247520.1 | BD161163.1 | DL092378.1 | DL099434.1 | HH806869.1 | DM006957.1 |
| HC471703.1 | A15343.1 | GM646398.1 | BD247049.1 | BD160919.1 | DL088603.1 | DL099402.1 | HH802863.1 | DM006540.1 |
| HC471647.1 | A02593.1 | GM646366.1 | BD246975.1 | AX587997.1 | DL113045.1 | DL099338.1 | HH794950.1 | DM004992.1 |
| HC471615.1 | A06139.1 | GM646334.1 | BD245178.1 | AX587964.1 | DL098530.1 | DL093296.1 | HH794718.1 | DM003501.1 |
| HC730940.1 | A04074.1 | GM646302.1 | BD244589.1 | AX587770.1 | DL092328.1 | DL093264.1 | HH794606.1 | DL489247.1 |
| HC045911.1 | CS444194.1 | GM646238.1 | BD243924.1 | AX587668.1 | DL092296.1 | DL093232.1 | HH791544.1 | DL488939.1 |
| HC045879.1 | CS446143.1 | GM639435.1 | BD243473.1 | AX587618.1 | DL092264.1 | DL093200.1 | HH779676.1 | GM998422.1 |
| HC045815.1 | CS439052.1 | GM639403.1 | CS078903.1 | AX587566.1 | DL092232.1 | DL045886.1 | HH777919.1 | GM983617.1 |
| HC045783.1 | CS438579.1 | GM639371.1 | CS070445.1 | AX587411.1 | DL092200.1 | DL042463.1 | HH773137.1 | GM996590.1 |
| HC045751.1 | CS435293.1 | GM639339.1 | CS068654.1 | AX575321.1 | DL092168.1 | DL049720.1 | DD357849.1 | GN010009.1 |
| HC045719.1 | CS435261.1 | GM639307.1 | CS063834.1 | AX573575.1 | DL088489.1 | DL049688.1 | DD357619.1 | GM994764.1 |
| HC045689.1 | CS435229.1 | GM639275.1 | AJ242589.1 | AX573207.1 | DL088457.1 | DL049656.1 | DD357242.1 | GM994319.1 |
| HC045657.1 | CS435197.1 | GM639243.1 | CS060523.1 | AX556834.1 | DL088425.1 | DL045840.1 | CS449741.1 | GM992472.1 |
| HC045625.1 | CS434834.1 | GM626696.1 | AY967381.1 | AX556288.1 | DL088393.1 | DL045776.1 | CS447937.1 | GM991906.1 |
| HC045593.1 | DD349506.1 | GM626664.1 | AY967349.1 | AX555204.1 | DL117793.1 | DL045744.1 | A05965.1 | GM975902.1 |
| HC045561.1 | DD334544.1 | GM626600.1 | AY967317.1 | AX555091.1 | DL117761.1 | DL045680.1 | A09701.1 | GM974808.1 |
| HC045529.1 | DD347835.1 | GM626568.1 | AY967285.1 | AX553570.1 | DL121889.1 | DL041833.1 | A01796.1 | GM969703.1 |
| DM379148.1 | DD347372.1 | GM626536.1 | AY967253.1 | AX551228.1 | DL121857.1 | DL041801.1 | CS443950.1 | GM969396.1 |
| HC035725.1 | DD332707.1 | GM754526.1 | AY967221.1 | AX543894.1 | DL121825.1 | DL041769.1 | CS438968.1 | GM968388.1 |
| HC035623.1 | DD345871.1 | GM660701.1 | AY967189.1 | AX538730.1 | DL121793.1 | DL041737.1 | CS435251.1 | GN007495.1 |
| HC025872.1 | DD347828.1 | GM660669.1 | AY967157.1 | AX538332.1 | DL117530.1 | DL041705.1 | CS435178.1 | GM984678.1 |
| HC025507.1 | DD346214.1 | GM660637.1 | AY967125.1 | AX538273.1 | DL117498.1 | DL037882.1 | DD348428.1 | FB986393.1 |
| HC025475.1 | CS430728.1 | GM660605.1 | AY967093.1 | AX538080.1 | DL117466.1 | DL037850.1 | DD332802.1 | FB985525.1 |
| HC025443.1 | CS426733.1 | GM660573.1 | AY967061.1 | AX537265.1 | DL117434.1 | DL037818.1 | BD449652.1 | GM969081.1 |
| HC025411.1 | CS418600.1 | GM660541.1 | AY967029.1 | AX535871.1 | DL112755.1 | DL037786.1 | BD397417.1 | GM968449.1 |
| HC024898.1 | CS419550.1 | GM653354.1 | AY966997.1 | AX528957.1 | DL107758.1 | DL037754.1 | BD429570.1 | GM970170.1 |
| HC010386.1 | CS419063.1 | GM653322.1 | AY966965.1 | A14259.1 | DL107726.1 | DL037722.1 | BD429345.1 | FB736147.1 |
| HC010183.1 | CS418805.1 | GM653290.1 | AY966933.1 | A01968.1 | DL107694.1 | DL037690.1 | BD429006.1 | GM969736.1 |
| HC009974.1 | CS418709.1 | GM653258.1 | CS057776.1 | A21781.1 | DL098317.1 | DL049615.1 | BD396502.1 | DL465750.1 |
| HC007809.1 | CS418645.1 | GM653226.1 | CS055307.1 | HV702556.1 | DL098285.1 | DL049583.1 | BD396022.1 | DL477843.1 |
| HC007713.1 | CS417160.1 | GM653194.1 | CS054748.1 | HW287338.1 | DL098253.1 | DL049551.1 | BD428050.1 | GM649904.1 |
| DL119731.1 | CS416741.1 | GM646195.1 | CS054326.1 | HW302706.1 | DL098221.1 | DL049519.1 | BD437522.1 | GM642968.1 |
| DL119699.1 | BD404758.1 | GM646163.1 | CS052382.1 | HW302552.1 | DL098189.1 | DL049487.1 | BD437314.1 | GM642935.1 |
| DL119667.1 | BD453359.1 | GM646131.1 | CS052347.1 | HW302382.1 | DL098157.1 | DL049455.1 | BD395087.1 | GM642903.1 |
| DL119635.1 | BD453328.1 | GM646099.1 | CS052314.1 | HW295719.1 | DL092051.1 | DL041664.1 | BD426905.1 | GM642871.1 |

| | | | | | | | | |
|---|---|---|---|---|---|---|---|---|
| DL115040.1 | BD453296.1 | GM646067.1 | CS050979.1 | HW295620.1 | DL091987.1 | DL041632.1 | BD393687.1 | GM642839.1 |
| DL115008.1 | BD453264.1 | GM646035.1 | CS048774.1 | HW295589.1 | DL011247.1 | DL041600.1 | BD453347.1 | GM635529.1 |
| DL114976.1 | BD455628.1 | GM639232.1 | AX773478.1 | HW302045.1 | DL011215.1 | DL041568.1 | BD453318.1 | GM635497.1 |
| DL114912.1 | BD445305.1 | GM639200.1 | AX772846.1 | HW295128.1 | DL047639.1 | DL041536.1 | BD453286.1 | GM635465.1 |
| DL039771.1 | BD444976.1 | GM639168.1 | AX768062.1 | HW306939.1 | DL047607.1 | DL041504.1 | BD391413.1 | GM630659.1 |
| DL039739.1 | BD413627.1 | GM639136.1 | AX766191.1 | HW294088.1 | DL047575.1 | DL037681.1 | BD388888.1 | GM630627.1 |
| DL039707.1 | BD444962.1 | GM639104.1 | AX765943.1 | HW293213.1 | DL047543.1 | DL037649.1 | BD378651.1 | GM630595.1 |
| DL035923.1 | BD389052.1 | GM639072.1 | AX755019.1 | HW293038.1 | DL039680.1 | DL037617.1 | BD376344.1 | GM621660.1 |
| DL031919.1 | BD377942.1 | GM626493.1 | AX751560.1 | HW292969.1 | DL039648.1 | DL037585.1 | BD375872.1 | GM621628.1 |
| DL031887.1 | BD376049.1 | GM626461.1 | BD183006.1 | HW292761.1 | DL039616.1 | DL037553.1 | BD374805.1 | GM621596.1 |
| DL031855.1 | BD375685.1 | GM626429.1 | BD180861.1 | HW291242.1 | DL039584.1 | DL037521.1 | BD350102.1 | GM621564.1 |
| DL031823.1 | BD374945.1 | GM626397.1 | AX743238.1 | HW291144.1 | DL039552.1 | DL037489.1 | BD356423.1 | GM657112.1 |
| DL031791.1 | BD374535.1 | GM626365.1 | AX739853.1 | HW291080.1 | DL039520.1 | DL033254.1 | BD353559.1 | GM657080.1 |
| DL031759.1 | BD374051.1 | GM626333.1 | AX722054.1 | HW291048.1 | DL039488.1 | DL039006.1 | BD359048.1 | GM657048.1 |
| DL027854.1 | BD357343.1 | GM835307.1 | BD177633.1 | HW291016.1 | DL039387.1 | DL038974.1 | BD342477.1 | GM657016.1 |
| DL027822.1 | BD356589.1 | GM660518.1 | AX717576.1 | HW290984.1 | DL039355.1 | DL038942.1 | BD341834.1 | GM656984.1 |
| DL027790.1 | BD350321.1 | GM660486.1 | AX701546.1 | HW290889.1 | DL039323.1 | DL038910.1 | BD323854.1 | GM656952.1 |
| DL027758.1 | BD359224.1 | DL125073.1 | AX699459.1 | HW290857.1 | DL035635.1 | DL035286.1 | BD326222.1 | GM656921.1 |
| DL023946.1 | BD345166.1 | HC083527.1 | AX699427.1 | HW290366.1 | DL035603.1 | DL035254.1 | BD325536.1 | GM656889.1 |
| DL023914.1 | BD341949.1 | HC081834.1 | AX696315.1 | HW290136.1 | DL035571.1 | DL035222.1 | BD313215.1 | GM656857.1 |
| DL023850.1 | BD349532.1 | HC081604.1 | AX685829.1 | HW297809.1 | DL035539.1 | DL035190.1 | BD319538.1 | GM656793.1 |
| DL023818.1 | BD339064.1 | DM460346.1 | AX685574.1 | HW288990.1 | DL035507.1 | DL035158.1 | BD319468.1 | GM656761.1 |
| DL023786.1 | BD338948.1 | HC061924.1 | AX684662.1 | HW287773.1 | DL031503.1 | DL035126.1 | BD308707.1 | GM649768.1 |
| DL020675.1 | BD320179.1 | HC061845.1 | AX683934.1 | HW285157.1 | DL031471.1 | DL031122.1 | CQ979900.1 | GM649736.1 |
| DL020643.1 | BD325570.1 | HC061375.1 | AX675248.1 | HW274691.1 | DL031439.1 | DL031090.1 | CQ972395.1 | GM649704.1 |
| DL020611.1 | BD314207.1 | HC059664.1 | AX664341.1 | HW279402.1 | DL031407.1 | DL031026.1 | CQ972363.1 | GM649672.1 |
| DL016208.1 | BD313629.1 | HC059511.1 | AX658705.1 | HW269951.1 | DL031375.1 | DL030994.1 | CQ972331.1 | GM649640.1 |
| DL016144.1 | BD312798.1 | HC059234.1 | AX657141.1 | HW269731.1 | DL027470.1 | DL030962.1 | CQ971801.1 | GM649608.1 |
| DL031612.1 | BD311488.1 | HC057893.1 | AX657109.1 | HW269592.1 | DL027438.1 | DL030933.1 | CQ971126.1 | GM642704.1 |
| DL031580.1 | BD319299.1 | HC055951.1 | AX657065.1 | HW269257.1 | DL027406.1 | DL027121.1 | CQ970874.1 | GM642672.1 |
| DL031548.1 | BD318881.1 | DM462087.1 | AX658704.1 | HW249164.1 | DL027374.1 | DL027089.1 | CQ968019.1 | GM642640.1 |
| DL027739.1 | BD308197.1 | DM382859.1 | AX657140.1 | HW267861.1 | DL023562.1 | DL027057.1 | CQ964032.1 | GM635330.1 |
| DL027707.1 | BD307652.1 | HC053893.1 | AX657108.1 | HW267807.1 | DL023530.1 | DL027025.1 | CQ955895.1 | GM635298.1 |
| DL027675.1 | BD294519.1 | HC053828.1 | BD175964.1 | HW267394.1 | DL023498.1 | DL026993.1 | CQ947122.1 | GM635266.1 |
| DL027643.1 | BD290004.1 | HC051947.1 | AX648069.1 | HW266129.1 | DL023434.1 | DL026961.1 | CQ944192.1 | GM630460.1 |
| DL027579.1 | BD293457.1 | HC051655.1 | AX643798.1 | HW265999.1 | DL023402.1 | DL030920.1 | CQ944160.1 | DL009038.1 |
| DL023735.1 | BD292450.1 | HC045508.1 | AX642158.1 | HW265865.1 | DL020387.1 | DL030881.1 | CQ944128.1 | DL014222.1 |
| DL023703.1 | BD291397.1 | HC045476.1 | BD171984.1 | HW263075.1 | DL020355.1 | DL030849.1 | CQ944096.1 | DL014190.1 |
| DL023671.1 | BD291257.1 | HC045444.1 | AX608859.1 | HW263038.1 | DL020323.1 | DL030817.1 | CQ944064.1 | DL014158.1 |
| DL023639.1 | BD299787.1 | HC045412.1 | AX608689.1 | HW263006.1 | DL015792.1 | DL030785.1 | CQ944032.1 | DL014126.1 |
| DL023607.1 | BD298752.1 | HC045348.1 | AX601616.1 | HW262974.1 | DL015760.1 | DL030753.1 | CQ944000.1 | DL014094.1 |
| DL023575.1 | BD298155.1 | HC045316.1 | AX601319.1 | HW262737.1 | DL015728.1 | DL026912.1 | CQ943968.1 | DL014062.1 |
| DL020528.1 | BD296948.1 | HC045284.1 | AX598866.1 | HW261417.1 | DL047250.1 | DL026880.1 | CQ943936.1 | DL026152.1 |
| DL020496.1 | BD296890.1 | HC049376.1 | AX598814.1 | HW261385.1 | DL047218.1 | DL026848.1 | CQ943904.1 | DL026120.1 |
| DL020464.1 | BD295264.1 | HC047464.1 | AX593517.1 | HW261353.1 | DL047186.1 | DL026816.1 | CQ943872.1 | DL026088.1 |
| DL020432.1 | BD285362.1 | HC047400.1 | AX587933.1 | HW261321.1 | DL047154.1 | DL026784.1 | CQ904369.1 | DD392045.1 |
| DL015997.1 | BD281336.1 | HC047368.1 | AX587834.1 | HW261289.1 | DL047122.1 | DL022972.1 | CQ903234.1 | DD382836.1 |

| | | | | | | | | |
|---|---|---|---|---|---|---|---|---|
| DL015965.1 | BD276950.1 | HC047336.1 | AX587741.1 | HW261257.1 | DL047090.1 | DL022940.1 | CQ898774.1 | DD400160.1 |
| DL015933.1 | BD279383.1 | HC047304.1 | AX587640.1 | HW261225.1 | CS607530.1 | DL022908.1 | CQ898646.1 | DD400099.1 |
| DL015901.1 | BD275263.1 | HC047272.1 | AX587536.1 | HV767915.1 | CS607461.1 | DL022876.1 | CQ898614.1 | DD393674.1 |
| DL015869.1 | BD274660.1 | HA640317.1 | AX137847.1 | HV766731.1 | CS607082.1 | DJ069355.1 | CQ898582.1 | CS463731.1 |
| DL011251.1 | CQ829239.1 | HB446931.1 | AX134687.1 | HV760622.1 | CS607002.1 | DJ066755.1 | CQ898511.1 | CS462998.1 |
| DL011219.1 | CQ828283.1 | HB445386.1 | AX128609.1 | HV766098.1 | HW069788.1 | DJ066359.1 | CQ896914.1 | CS460160.1 |
| DL047643.1 | CQ826939.1 | HB444515.1 | AX119914.1 | HV753398.1 | HW084944.1 | DJ066327.1 | CQ895562.1 | DD361291.1 |
| DL047611.1 | CQ824762.1 | DM121077.1 | AX114292.1 | HV750375.1 | HW084905.1 | DJ066293.1 | CQ895456.1 | DD367332.1 |
| DL047579.1 | CQ821898.1 | DM139936.1 | AX111695.1 | HV750343.1 | HW097738.1 | DJ068400.1 | CQ893703.1 | DD362227.1 |
| DL047547.1 | CQ818582.1 | DM138466.1 | AX107162.1 | HV752869.1 | HW067062.1 | DJ065241.1 | CQ891001.1 | DD368158.1 |
| DL035383.1 | FW573701.1 | DM138434.1 | AX103761.1 | HV758993.1 | HW087987.1 | DJ055625.1 | CQ888147.1 | DD361325.1 |
| DL035351.1 | FW574731.1 | DM143375.1 | AX103638.1 | HV755504.1 | HV585645.1 | DJ052685.1 | CQ881818.1 | CS458453.1 |
| DL035319.1 | FW573540.1 | DM138344.1 | AX100347.1 | HV755404.1 | HV574028.1 | DJ061654.1 | CQ877782.1 | CS457145.1 |
| DL031315.1 | FW572686.1 | DM138312.1 | AX097514.1 | HV752626.1 | HV573532.1 | DJ061606.1 | CQ875535.1 | CS456702.1 |
| DL031283.1 | FW572654.1 | DM121665.1 | AX093094.1 | HV757358.1 | HV573497.1 | DJ061558.1 | CQ874988.1 | CS453660.1 |
| DL031251.1 | FW572603.1 | DM137789.1 | AX090034.1 | HV746030.1 | HV572398.1 | DJ061510.1 | CQ874707.1 | CS452803.1 |
| DL031219.1 | FW573486.1 | DM137122.1 | AX088021.1 | HV749741.1 | HV560083.1 | DJ055558.1 | CQ873288.1 | CS451660.1 |
| DL031187.1 | FW572094.1 | DM136751.1 | AX082193.1 | HV745871.1 | HV555948.1 | DJ061305.1 | CQ871415.1 | CS451007.1 |
| DL031155.1 | FW570773.1 | DM131195.1 | AX080317.1 | HV749435.1 | HV561987.1 | DJ055294.1 | CQ871221.1 | CS450602.1 |
| DL027346.1 | HI979586.1 | HB441235.1 | AX078905.1 | HV749282.1 | HV553658.1 | DJ055028.1 | CQ869293.1 | CS450523.1 |
| DL027314.1 | HI979303.1 | HB435663.1 | AX077272.1 | HV748490.1 | AY659403.1 | DJ060416.1 | CQ868903.1 | DD361028.1 |
| DL027282.1 | HI969545.1 | HB427179.1 | AX076600.1 | HV748446.1 | AY659371.1 | DJ054299.1 | CQ868749.1 | DD353818.1 |
| DL027250.1 | HI989105.1 | HB426469.1 | AX076393.1 | HV747617.1 | AY659339.1 | DJ060351.1 | CQ866706.1 | DD357924.1 |
| DL027218.1 | HI988946.1 | HB423124.1 | AX063718.1 | HV750798.1 | AY659307.1 | DJ060309.1 | CQ860049.1 | DD357884.1 |
| DL027186.1 | HI988403.1 | HB423085.1 | AX061296.1 | AY657429.1 | AY659275.1 | DJ060275.1 | AX133308.1 | DD357626.1 |
| DL027154.1 | HI968585.1 | HB403630.1 | AX060540.1 | AY657397.1 | AY659243.1 | DJ060229.1 | AX127557.1 | DD357465.1 |
| DL023310.1 | HI987661.1 | HB416464.1 | AX059829.1 | AY657365.1 | AY659211.1 | DJ056901.1 | AX119920.1 | A29480.1 |
| DL023278.1 | HI987438.1 | HB397000.1 | AX058846.1 | AY657333.1 | AY659179.1 | DJ060173.1 | HW380808.1 | A08388.1 |
| DL023246.1 | HC194691.1 | HB396570.1 | AX057967.1 | AY657301.1 | AY659147.1 | DJ053089.1 | HW379425.1 | A04058.1 |
| DL023214.1 | FW508690.1 | HB394784.1 | AX056089.1 | AY657269.1 | AY659115.1 | DJ052906.1 | HW365268.1 | CS443887.1 |
| DL023182.1 | FW512092.1 | HB394726.1 | AX055009.1 | AY657237.1 | AY659083.1 | FB291778.1 | HW381340.1 | CS442256.1 |
| DL020167.1 | HI642504.1 | HB394314.1 | AX052913.1 | AY657205.1 | AY659051.1 | CS810687.1 | HW381039.1 | CS438984.1 |
| DL020135.1 | HI641960.1 | HB394250.1 | AX049484.1 | AY657173.1 | AY659019.1 | CS810645.1 | HW353808.1 | CS435287.1 |
| DL020103.1 | HI641416.1 | HB394172.1 | AX047113.1 | AY657141.1 | AY658987.1 | CS810545.1 | HW260860.1 | CS435255.1 |
| DL020071.1 | HI640872.1 | HB393655.1 | AX046144.1 | AY657109.1 | AY658955.1 | FB292744.1 | HW260828.1 | CS435191.1 |
| DL020039.1 | HI639896.1 | HB403012.1 | AX043769.1 | AY657077.1 | AY658923.1 | CS812655.1 | HW260764.1 | CS430097.1 |
| DL020007.1 | HI637817.1 | DM113492.1 | AX040775.1 | AY657045.1 | AY658891.1 | FB292268.1 | HW260732.1 | CS433056.1 |
| DL015444.1 | HI637631.1 | GM715171.1 | AX038888.1 | U06943.1 | AY658859.1 | DJ017834.1 | HW260700.1 | DD217987.1 |
| DL010922.1 | HI380446.1 | GM616295.1 | AX036755.1 | J03742.1 | AY658827.1 | DJ019843.1 | HW260668.1 | DD217862.1 |
| DL010858.1 | HI380344.1 | GM038806.1 | AX036023.1 | M22554.1 | AY658795.1 | DJ015967.1 | HW260636.1 | CS254886.1 |
| DL010826.1 | HI377771.1 | GM000648.1 | AX035970.1 | K01663.1 | AY658763.1 | DJ015686.1 | HW260604.1 | DD212323.1 |
| DL023147.1 | HI377253.1 | GM043426.1 | AX035426.1 | M34500.1 | AY658731.1 | CS803343.1 | HW260572.1 | DD211905.1 |
| DL023115.1 | HI376816.1 | HB386636.1 | AX032988.1 | HV549875.1 | AY658699.1 | FB295031.1 | HW260540.1 | DD191354.1 |
| DL023083.1 | HI375897.1 | HB385922.1 | AX028966.1 | HV549499.1 | AY658667.1 | FB294180.1 | HW260508.1 | DD206891.1 |
| DL023051.1 | HI373100.1 | HB339938.1 | AX024430.1 | GM627003.1 | AY658635.1 | DJ047315.1 | HW260476.1 | DD206859.1 |
| DL023019.1 | HI371014.1 | HA643426.1 | HW118238.1 | GM626995.1 | AY658603.1 | DJ047022.1 | HW260444.1 | DD206827.1 |
| DL022987.1 | HI370468.1 | GM659512.1 | HW115613.1 | GM626963.1 | AY658571.1 | DJ052351.1 | HW260412.1 | DD206797.1 |

| | | | | | | | | |
|---|---|---|---|---|---|---|---|---|
| DL019812.1 | HI369127.1 | GM659480.1 | HW113815.1 | GM626931.1 | AY658539.1 | DJ052035.1 | HW260380.1 | DD194644.1 |
| DL015409.1 | HI369084.1 | GM659448.1 | HW081515.1 | GM646599.1 | AY658507.1 | DJ045766.1 | HW260348.1 | CS249481.1 |
| HC310032.1 | HI368459.1 | GM659384.1 | HW087321.1 | GM646561.1 | AY658475.1 | DJ050613.1 | HW260316.1 | DD174386.1 |
| HC310000.1 | HI424144.1 | GM659352.1 | HW096777.1 | GM646529.1 | AY658443.1 | DJ050492.1 | HW260284.1 | CS119546.1 |
| HC309968.1 | HI424112.1 | GM652160.1 | HW096431.1 | GM646497.1 | HI380325.1 | DJ049756.1 | HW260252.1 | CS119512.1 |
| HC308576.1 | HI424080.1 | GM652128.1 | HW072717.1 | GM646465.1 | HI379291.1 | DJ049379.1 | HW260188.1 | CS119480.1 |
| HC307858.1 | HI423822.1 | GM652096.1 | HW096215.1 | GM646433.1 | HI378545.1 | DJ048814.1 | HW260156.1 | CS119412.1 |
| FU257897.1 | HI423698.1 | GM652064.1 | HW072217.1 | GM639630.1 | HI378129.1 | DJ048782.1 | HW260124.1 | CS119347.1 |
| FU257865.1 | HI505012.1 | GM652032.1 | HW104301.1 | GM639598.1 | HI378097.1 | CS793631.1 | HW260092.1 | CS119314.1 |
| FU257833.1 | HI464018.1 | GM645205.1 | HW071177.1 | GM639566.1 | HI375922.1 | CS793411.1 | HW260060.1 | CS119281.1 |
| FU263065.1 | HI549584.1 | GM645173.1 | HW070967.1 | GM639534.1 | HI375887.1 | CS800124.1 | HW260028.1 | CS119249.1 |
| FU262848.1 | HI504386.1 | GM645141.1 | HW070558.1 | GM639502.1 | HI373480.1 | CS799998.1 | HW259996.1 | CS119185.1 |
| FU259877.1 | HI463014.1 | GM645109.1 | HW099602.1 | GM639494.1 | HI372214.1 | CS716865.1 | HW259964.1 | CS119153.1 |
| FU265438.1 | HH716027.1 | GM645077.1 | HW099529.1 | GM639438.1 | HI369381.1 | CS716800.1 | HW259863.1 | CS119121.1 |
| FU250456.1 | HH713822.1 | GM645045.1 | HW069455.1 | GM626891.1 | HI369107.1 | CS707355.1 | HW259721.1 | CS119087.1 |
| FU265000.1 | HH961359.1 | GM645013.1 | HW069005.1 | GM626859.1 | HI369067.1 | CS798885.1 | HW259451.1 | CS119053.1 |
| FU264508.1 | HH961327.1 | GM638182.1 | HW102277.1 | GM626827.1 | HH772124.1 | DD401637.1 | HW258942.1 | CS118988.1 |
| FU261563.1 | HH961295.1 | GM638150.1 | HW066587.1 | GM626795.1 | HI424135.1 | DD401605.1 | HW258883.1 | CS118956.1 |
| FU258425.1 | HH961263.1 | GM638118.1 | HW083258.1 | GM626731.1 | HI424103.1 | DD401573.1 | HW258830.1 | CS118923.1 |
| HC313123.1 | HH961231.1 | GM638086.1 | HW083182.1 | GM653610.1 | HI424071.1 | DD401541.1 | HW257383.1 | CS118890.1 |
| DM476467.1 | HH975396.1 | GM651898.1 | HW066114.1 | GM653578.1 | HI423845.1 | DD401509.1 | HW257351.1 | CS118857.1 |
| DM472989.1 | HH975094.1 | GM651866.1 | HW088260.1 | GM660775.1 | HH762710.1 | DD401477.1 | HW257287.1 | CS118824.1 |
| HC306247.1 | HH982452.1 | GM651834.1 | HV579905.1 | GM660743.1 | HI415912.1 | CS480746.1 | HW257191.1 | CS118791.1 |
| HC306207.1 | HH974554.1 | GM651802.1 | HV600338.1 | GM653524.1 | HI415880.1 | CS479596.1 | HW257159.1 | CS118759.1 |
| HC306167.1 | HH998091.1 | GM658342.1 | HV585088.1 | GM653492.1 | HI415783.1 | CS476724.1 | HW256935.1 | CS118598.1 |
| DM144961.1 | HH998028.1 | GM651150.1 | HV588885.1 | GM653428.1 | HI415751.1 | CS475998.1 | HW256903.1 | CS118564.1 |
| HB460440.1 | HH997984.1 | GM651118.1 | HV584761.1 | GM653396.1 | HI414038.1 | CS469234.1 | HW256839.1 | CS118530.1 |
| HB455306.1 | HH999666.1 | GM651086.1 | HV579122.1 | GM646397.1 | HI414001.1 | CS468157.1 | HW256775.1 | CS118497.1 |
| HB455026.1 | HH997932.1 | GM651054.1 | HV579003.1 | GM646365.1 | HI413442.1 | CS467738.1 | HW256711.1 | CS118464.1 |
| HB453859.1 | HH997846.1 | GM651022.1 | HV592486.1 | HW240968.1 | HI412240.1 | CS467653.1 | HW251201.1 | CS118432.1 |
| HB445307.1 | HH997787.1 | GM650990.1 | HV592337.1 | HW240872.1 | HI410709.1 | CS467517.1 | HW250927.1 | CS118399.1 |
| HB444509.1 | HH997748.1 | GM644191.1 | HV578516.1 | HW240808.1 | HI516594.1 | CS466955.1 | HW241286.1 | CS118367.1 |
| DM138460.1 | HH999470.1 | GM644159.1 | HV601496.1 | HW240744.1 | HI516517.1 | CS464638.1 | HW241157.1 | CS118266.1 |
| DM138428.1 | HH999425.1 | GM644127.1 | HV601353.1 | HW240720.1 | HI516113.1 | CS464556.1 | HW247833.1 | CS118234.1 |
| DM143078.1 | HH999375.1 | GM644095.1 | HV586240.1 | HW240656.1 | HI516081.1 | CS464503.1 | HW240959.1 | CS118201.1 |
| DM137698.1 | HH997665.1 | GM644063.1 | HV601074.1 | HW240624.1 | FW304479.1 | CS464126.1 | HW240895.1 | CS118167.1 |
| DM137114.1 | HH997627.1 | GM644031.1 | DL021756.1 | HW247939.1 | FW310246.1 | DD392064.1 | HW240799.1 | CS118135.1 |
| HB441229.1 | HH997570.1 | GM636937.1 | DL021724.1 | HW239405.1 | FW331774.1 | DD391955.1 | HW240767.1 | CS118103.1 |
| HB423117.1 | HH997495.1 | GM636905.1 | DL017390.1 | HW238893.1 | FW309554.1 | DD391752.1 | HW240647.1 | CS118037.1 |
| HB403494.1 | HH997400.1 | GM636873.1 | DL017358.1 | HW238595.1 | FW308319.1 | DD391866.1 | HW240615.1 | CS118001.1 |
| HB396994.1 | HH997356.1 | GM632465.1 | DL017326.1 | HW238496.1 | FW306419.1 | DD391542.1 | HW239315.1 | CS117968.1 |
| HB394811.1 | HH999278.1 | GM632433.1 | DL012937.1 | HW238411.1 | FW332170.1 | DD391631.1 | HW239169.1 | DD178767.1 |
| HB394717.1 | HH999221.1 | GM632401.1 | DL041345.1 | HW238370.1 | HC769897.1 | DD382904.1 | HW238749.1 | DD171566.1 |
| HB394244.1 | HH999169.1 | GM632369.1 | DL041313.1 | HW243004.1 | HC768865.1 | DD400167.1 | HW238558.1 | DD181783.1 |
| HB397667.1 | HH999132.1 | GM632337.1 | DL041281.1 | HW237975.1 | HC757398.1 | DD400135.1 | HW238312.1 | CS070528.1 |
| DM111663.1 | HH999091.1 | GM632305.1 | DL021485.1 | HW237899.1 | HC742883.1 | DD393795.1 | HW242995.1 | CS068661.1 |
| DM118017.1 | HH999052.1 | GM623534.1 | DL021453.1 | HW237866.1 | HC754717.1 | DD393707.1 | HW242950.1 | CS064605.1 |

| | | | | | | | | |
|---|---|---|---|---|---|---|---|---|
| DM114891.1 | HH998991.1 | GM623502.1 | DL021421.1 | HW248706.1 | HC732143.1 | CS461262.1 | HV347747.1 | CS063839.1 |
| GM038732.1 | HH998937.1 | GM623470.1 | DL021389.1 | HW237429.1 | HC471770.1 | CS459921.1 | HV344950.1 | AY967385.1 |
| GM000680.1 | HH997215.1 | GM623438.1 | DL017183.1 | HW242322.1 | HC471738.1 | CS459117.1 | HV347356.1 | AY967353.1 |
| HB386984.1 | HH997153.1 | GM623406.1 | DL017151.1 | HW242290.1 | HC471698.1 | DD367712.1 | HV340015.1 | AY967321.1 |
| HB386576.1 | HH970111.1 | GM623374.1 | DL017119.1 | HW242258.1 | HC471642.1 | DD361266.1 | HV347087.1 | AY967289.1 |
| GN029847.1 | HH998890.1 | GM623342.1 | DL049422.1 | HW243435.1 | HC471610.1 | DD367343.1 | HV344674.1 | AY967257.1 |
| GN029815.1 | HH998830.1 | GM643893.1 | DL049390.1 | HW236729.1 | HC729139.1 | DD373640.1 | HV339989.1 | AY967225.1 |
| GN013376.1 | HH998787.1 | GM643861.1 | DL049358.1 | HW248263.1 | HC729057.1 | DD362925.1 | HV339957.1 | AY967193.1 |
| GN010219.1 | HH997136.1 | GM643829.1 | DL049326.1 | HW243149.1 | HC728986.1 | DD362509.1 | HV339666.1 | AY967161.1 |
| GN033549.1 | HH997073.1 | GM636832.1 | DL030715.1 | HW243057.1 | HC727673.1 | DD370039.1 | HV341943.1 | AY967129.1 |
| GN033517.1 | HH997014.1 | GM636800.1 | DL030683.1 | HW163859.1 | HC727390.1 | DD368057.1 | HV341797.1 | AY967097.1 |
| GN033485.1 | HH996964.1 | GM636768.1 | DL026746.1 | HW163490.1 | DL119353.1 | DD361300.1 | HV335503.1 | AY967065.1 |
| GN033453.1 | HH996938.1 | GM636736.1 | DL026714.1 | HW163429.1 | DL119321.1 | CS457872.1 | HV322259.1 | AY967033.1 |
| GN033421.1 | HH998608.1 | GM636704.1 | DL026682.1 | HW161420.1 | DL114829.1 | CS457748.1 | HV321826.1 | AY967001.1 |
| GN033357.1 | HH996932.1 | GM636672.1 | DL026650.1 | HW160704.1 | DL114797.1 | CS457297.1 | HV334352.1 | AY966969.1 |
| GN033325.1 | HH996900.1 | GM632264.1 | DL019503.1 | HW160631.1 | DL114765.1 | CS457165.1 | HV325839.1 | AY966937.1 |
| GN033293.1 | AX960344.1 | GM632200.1 | DL019471.1 | HW160599.1 | DL114733.1 | CS453694.1 | HV325349.1 | CS057795.1 |
| GN033261.1 | AX958652.1 | GM632168.1 | DL019439.1 | HW155911.1 | DL114701.1 | CS451675.1 | HV323577.1 | CS055385.1 |
| GN033229.1 | AX957833.1 | GM632136.1 | DL019407.1 | HW155755.1 | DL114669.1 | CS450929.1 | HV313388.1 | CS053033.1 |
| GN033197.1 | BD010843.1 | GM632104.1 | DL014908.1 | HW150138.1 | DL130734.1 | CS450621.1 | HV187470.1 | CS052438.1 |
| GN033165.1 | AX957205.1 | GM623333.1 | DL014876.1 | HW150106.1 | DL032364.1 | DD360888.1 | HV187236.1 | CS052386.1 |
| GN033133.1 | AX952273.1 | GM623301.1 | DL014844.1 | HW150074.1 | DL016409.1 | DD359257.1 | HV189864.1 | CS052352.1 |
| GN033069.1 | AX938906.1 | GM623269.1 | DL030460.1 | HW150042.1 | DL016377.1 | DD359192.1 | HV227382.1 | CS052318.1 |
| GN033037.1 | AX934512.1 | GM623237.1 | DL026504.1 | HW150010.1 | DL016345.1 | DD359106.1 | HV247803.1 | CS047668.1 |
| GN033005.1 | AX928623.1 | GM741843.1 | DL026494.1 | HW149978.1 | DL016313.1 | DD357895.1 | HV247162.1 | CS037088.1 |
| GN032973.1 | AX925801.1 | GM741811.1 | DL022474.1 | HW144821.1 | DL016281.1 | DD357638.1 | HV301791.1 | CS023731.1 |
| GN032941.1 | HW363653.1 | GM658325.1 | DL014508.1 | HW155534.1 | DL016249.1 | DD357217.1 | HH757462.1 | CS022574.1 |
| GN032909.1 | HW344769.1 | GM658293.1 | DL014476.1 | HV963660.1 | DL011733.1 | CS448466.1 | HH757430.1 | CS021415.1 |
| GN032877.1 | HW344745.1 | GM658261.1 | DL014444.1 | HV966112.1 | DL011701.1 | CS448331.1 | HI401122.1 | AX608808.1 |
| GN032845.1 | HW344566.1 | GM658229.1 | CS608439.1 | HV966018.1 | DL011669.1 | A15511.1 | HI642572.1 | AX601557.1 |
| GN032813.1 | HW344515.1 | GM658197.1 | CS608353.1 | HV965954.1 | DL011637.1 | A02595.1 | HI637697.1 | AX601389.1 |
| GN032781.1 | HW317108.1 | GM658165.1 | CS607664.1 | HV962411.1 | DL011605.1 | A06280.1 | HI380392.1 | AX601357.1 |
| GN032750.1 | HW326520.1 | GM650844.1 | CS607065.1 | HV962271.1 | DL048556.1 | A04076.1 | HI380301.1 | AX601325.1 |
| GN032718.1 | HW326322.1 | GM650812.1 | CS606966.1 | HV951629.1 | DL048460.1 | CS446144.1 | CS157856.1 | AX599027.1 |
| GN032686.1 | HW326281.1 | GM650781.1 | CS606904.1 | HV951565.1 | DL044747.1 | CS439053.1 | CS141617.1 | AX598873.1 |
| GN032621.1 | HW338882.1 | GM658115.1 | CS606822.1 | HV951533.1 | DL044715.1 | DD103279.1 | CS124709.1 | AX598771.1 |
| GN032589.1 | HW338626.1 | GM658083.1 | CS593078.1 | HV957870.1 | DL044683.1 | DD103204.1 | CS124635.1 | AX598350.1 |
| GN032557.1 | HW338370.1 | GM658051.1 | CS597720.1 | HV957589.1 | DL044531.1 | DD102584.1 | CS124194.1 | AX593522.1 |
| GN032494.1 | HW338242.1 | GM658019.1 | CS585352.1 | HV947292.1 | DL044499.1 | DD102451.1 | CS119878.1 | AX592804.1 |
| GN032430.1 | HW338114.1 | GM657987.1 | CS584891.1 | HV953249.1 | DL044467.1 | DD102419.1 | CS119538.1 | AX591628.1 |
| GN032398.1 | HW337858.1 | GM657955.1 | CS583661.1 | HV952888.1 | DL036603.1 | DD052031.1 | CS119471.1 | AX589681.1 |
| GN032366.1 | HW337730.1 | GM650763.1 | CS575300.1 | HV951945.1 | DL036571.1 | DD057918.1 | CS119404.1 | BD161075.1 |
| GN032333.1 | HW337474.1 | GM650731.1 | CS574127.1 | HV951808.1 | DL036539.1 | DD057886.1 | CS119371.1 | BD160755.1 |
| GN032301.1 | HW337346.1 | GM650699.1 | CS573068.1 | HV946791.1 | DL032240.1 | DD057854.1 | CS119339.1 | AX587907.1 |
| GN032269.1 | HW337218.1 | GM629633.1 | CS570793.1 | HV946277.1 | DL032208.1 | DD057234.1 | CS119305.1 | AX575306.1 |
| GN032237.1 | HW336962.1 | GM629601.1 | CS549428.1 | HV940048.1 | DL032176.1 | DD056823.1 | CS119273.1 | AX573161.1 |
| GN032205.1 | HW321653.1 | GM629569.1 | CS561267.1 | HV945936.1 | DL048446.1 | DD055248.1 | CS119241.1 | AX556824.1 |

| | | | | | | | | |
|---|---|---|---|---|---|---|---|---|
| GN032173.1 | HW321203.1 | GM629537.1 | CS561172.1 | HV943546.1 | DL048414.1 | DD052484.1 | CS119209.1 | AX554282.1 |
| GN032141.1 | HW336470.1 | GM629505.1 | CS559087.1 | HV943280.1 | DL048382.1 | DD052240.1 | CS119177.1 | AX543884.1 |
| GN032077.1 | HW321162.1 | GM696776.1 | DD373502.1 | HV942954.1 | DL048350.1 | DD051843.1 | CS119145.1 | AX542213.1 |
| GN032045.1 | HW320832.1 | GM664729.1 | DD362899.1 | HV815182.1 | DL044336.1 | DD058871.1 | CS119078.1 | AX538887.1 |
| GN032013.1 | HW329374.1 | GM655934.1 | DD368337.1 | HV931271.1 | DL044272.1 | DD042737.1 | CS119013.1 | AX538323.1 |
| GN031917.1 | HW320672.1 | GM655870.1 | DD368144.1 | HV925495.1 | DL040096.1 | DD048097.1 | CS118948.1 | AX538265.1 |
| GN031885.1 | HW336364.1 | GM655766.1 | A10881.1 | HV936360.1 | DJ446855.1 | DD044325.1 | CS118882.1 | AX537598.1 |
| GN031853.1 | HW336127.1 | GM648749.1 | CS457187.1 | HV930400.1 | DJ438151.1 | DD030108.1 | CS118816.1 | AX536408.1 |
| HC889202.1 | HW335929.1 | GM648717.1 | CS456727.1 | HV803287.1 | DJ437312.1 | DD039102.1 | CS118783.1 | AX534958.1 |
| HC877930.1 | HW328824.1 | GM648685.1 | CS456089.1 | HV782089.1 | DJ437224.1 | DD038834.1 | CS118751.1 | AX529073.1 |
| HC869947.1 | HW328698.1 | GM648653.1 | CS453958.1 | HV932661.1 | DJ437105.1 | DD038495.1 | CS118718.1 | AX528949.1 |
| HC869480.1 | HW328606.1 | GM648621.1 | CS450655.1 | HV930031.1 | DJ436980.1 | DD038331.1 | CS118686.1 | AX528654.1 |
| HC876938.1 | HW319234.1 | GM641818.1 | CS450262.1 | HV929923.1 | DJ436827.1 | DD037407.1 | CS118654.1 | AX528111.1 |
| FW304497.1 | HW328360.1 | GM641786.1 | DD359445.1 | HV932331.1 | DJ436744.1 | DD037147.1 | CS118589.1 | AX526822.1 |
| FW310247.1 | HW328288.1 | GM641754.1 | DD357915.1 | HV803147.1 | DJ436657.1 | DD033927.1 | CS118554.1 | AX523917.1 |
| FW309559.1 | HW335765.1 | GM641722.1 | DD357836.1 | HV803115.1 | DJ436503.1 | DD031279.1 | CS118522.1 | AX523635.1 |
| FW309121.1 | HW328240.1 | GM641690.1 | DD357600.1 | HV781638.1 | DJ445571.1 | DD030776.1 | CS118488.1 | A32039.1 |
| FW308789.1 | HW328096.1 | GM641657.1 | DD355903.1 | HV802945.1 | DJ444602.1 | DD030697.1 | CS118456.1 | A31164.1 |
| FW308320.1 | HW318507.1 | GM641625.1 | CS447692.1 | HV549281.1 | DJ444563.1 | DD026923.1 | CS118424.1 | A29113.1 |
| FW334253.1 | HW335150.1 | GM634437.1 | A19557.1 | HV549083.1 | DJ434335.1 | DD026052.1 | CS118391.1 | A28115.1 |
| FW333833.1 | HW314323.1 | GM634405.1 | A12248.1 | HV548994.1 | DJ438352.1 | DD025702.1 | CS118358.1 | A20261.1 |
| FW306425.1 | HW314087.1 | GM634373.1 | A08586.1 | HV547842.1 | DJ438285.1 | DD023852.1 | CS118324.1 | A06569.1 |
| FW306106.1 | HW312136.1 | GM634341.1 | CS444612.1 | HV547407.1 | DJ438203.1 | DD023431.1 | CS118226.1 | A25740.1 |
| FW332182.1 | HW312079.1 | GM634309.1 | CS446335.1 | HV543496.1 | DJ427945.1 | DD020885.1 | CS118193.1 | A24791.1 |
| HC769916.1 | HW311846.1 | GM634277.1 | CS441594.1 | HV547077.1 | DJ402640.1 | DD029744.1 | CS118159.1 | A22362.1 |
| HC769792.1 | HW311291.1 | GM629447.1 | CS438953.1 | HV539676.1 | DJ402608.1 | DD029075.1 | CS118127.1 | A22624.1 |
| HC767810.1 | HW311163.1 | GM629450.1 | CS436067.1 | HV538933.1 | DJ402542.1 | DD027692.1 | CS118095.1 | A22089.1 |
| HC767416.1 | HV962273.1 | GM629408.1 | DD335101.1 | HV538579.1 | DJ400833.1 | DD010341.1 | CS118062.1 | A16264.1 |
| HC757181.1 | HV962201.1 | GM629376.1 | DD347413.1 | HV538453.1 | DJ400801.1 | DD010167.1 | CS118028.1 | A21887.1 |
| HC755777.1 | HV961756.1 | GM629344.1 | DD349653.1 | HV543375.1 | DJ399774.1 | DD019582.1 | CS117992.1 | A21726.1 |
| HC754720.1 | HV961722.1 | CS118334.1 | DD332620.1 | HV543289.1 | DJ393076.1 | DD017889.1 | CS117960.1 | A20275.1 |
| HC733690.1 | HV961690.1 | CS118300.1 | CS430158.1 | HV542717.1 | DJ419220.1 | DD017619.1 | CS114719.1 | A19436.1 |
| HC732144.1 | HV951599.1 | CS118268.1 | CS425083.1 | HV542180.1 | DJ417430.1 | DD014262.1 | CS106396.1 | A03819.1 |
| HC731693.1 | HV951567.1 | CS118236.1 | CS424378.1 | HV541804.1 | DJ402723.1 | BD495454.1 | CS104694.1 | A18337.1 |
| HC057879.1 | HV951535.1 | CS118203.1 | CS423365.1 | HV541492.1 | DJ386554.1 | BD454131.1 | A19336.1 | A16023.1 |
| HC057171.1 | HV950600.1 | CS118169.1 | CS421300.1 | HV541035.1 | DJ381034.1 | BD434155.1 | A20747.1 | A14610.1 |
| DM459964.1 | HV957874.1 | CS118137.1 | CS417381.1 | HV514913.1 | DJ381002.1 | BD453909.1 | A18325.1 | A13269.1 |
| DM459789.1 | HV949009.1 | CS118105.1 | CS412175.1 | HV533064.1 | DJ380970.1 | BD453876.1 | A05517.1 | A12679.1 |
| DM385646.1 | HV954451.1 | CS118072.1 | CS415527.1 | HV532754.1 | DJ380938.1 | BD453844.1 | A15639.1 | A11166.1 |
| DM383910.1 | HV947906.1 | CS118039.1 | CS410888.1 | HV536511.1 | DJ380906.1 | BD453812.1 | A14581.1 | A10364.1 |
| DM381539.1 | HV947296.1 | CS118004.1 | BD271170.1 | HV534645.1 | DJ380874.1 | BD453778.1 | A13628.1 | A09358.1 |
| HC054869.1 | HV953252.1 | CS114662.1 | DD231202.1 | HV532288.1 | DJ380822.1 | BD433591.1 | A12582.1 | A02231.1 |
| HC053878.1 | HV951947.1 | CS114318.1 | BD274185.1 | HV537344.1 | DJ388689.1 | BD453756.1 | A11126.1 | A09020.1 |
| HC051641.1 | HV951811.1 | CS112344.1 | BD273649.1 | HV535646.1 | DJ388448.1 | BD453724.1 | A10256.1 | A08193.1 |
| HC050948.1 | HV946793.1 | CS111524.1 | BD273234.1 | HV531986.1 | CS724939.1 | BD453692.1 | A08566.1 | A06424.1 |
| HC050283.1 | HV940465.1 | CS110922.1 | BD273098.1 | HV531939.1 | DD346815.1 | BD453660.1 | A16710.1 | A05991.1 |
| HC049744.1 | HV946279.1 | CS103349.1 | BD271971.1 | HV516230.1 | DD349794.1 | BD453628.1 | A08534.1 | A05048.1 |

| | | | | | | | | |
|---|---|---|---|---|---|---|---|---|
| HC045501.1 | HV940062.1 | CS105996.1 | BD271162.1 | HV528048.1 | CS433052.1 | BD453596.1 | A07738.1 | GM621520.1 |
| HC045437.1 | HV945799.1 | CS103432.1 | BD271066.1 | HV515584.1 | CS421355.1 | BD493220.1 | A07228.1 | GM656646.1 |
| HC045405.1 | HV944549.1 | CS103331.1 | DD231200.1 | HV515552.1 | CS415724.1 | BD453546.1 | A04543.1 | GM649461.1 |
| HC045373.1 | HV943282.1 | CS103297.1 | DD230436.1 | HV515520.1 | CS415539.1 | BD453514.1 | A03848.1 | GM649429.1 |
| HC045341.1 | HV942961.1 | CS102982.1 | DD229862.1 | HV515488.1 | CS414838.1 | BD453482.1 | A01166.1 | GM649397.1 |
| HC045277.1 | HV942064.1 | CS102918.1 | DD227724.1 | HV515456.1 | DD320526.1 | BD453450.1 | M29480.1 | GM670037.1 |
| HC049369.1 | HV932150.1 | CS102630.1 | DD227408.1 | HV515424.1 | DD327196.1 | BD453418.1 | M14393.1 | GM656532.1 |
| HC042010.1 | HV929264.1 | CS102598.1 | DD226571.1 | HV515360.1 | DD327025.1 | BD453379.1 | K01210.1 | GM656500.1 |
| HC047457.1 | HV937099.1 | CS102566.1 | DD224454.1 | HV515296.1 | DD326892.1 | BD453241.1 | J02530.1 | GM656468.1 |
| HC047425.1 | HV931869.1 | CS102534.1 | DD224159.1 | HV515264.1 | DD329898.1 | BD453209.1 | M25074.1 | GM656436.1 |
| HC047393.1 | HV931458.1 | CS102502.1 | DD223497.1 | HV515232.1 | DD329034.1 | BD453122.1 | M60080.1 | GM649339.1 |
| HC047361.1 | HV815188.1 | CS101215.1 | DD233399.1 | HV514048.1 | DD321799.1 | BD453019.1 | M19568.1 | GM649251.1 |
| HC047329.1 | HV925502.1 | CS091385.1 | DD231548.1 | HV510673.1 | DD325361.1 | BD442219.1 | OK586149.1 | GM649219.1 |
| HC047297.1 | HV939107.1 | AX573171.1 | DD231400.1 | HV510355.1 | CS389278.1 | BD432365.1 | LT908474.1 | GM642470.1 |
| HC047265.1 | HV936400.1 | AX556827.1 | CQ757644.1 | HV510322.1 | CS389224.1 | BD399671.1 | AH003159.2 | GM635235.1 |
| HC047041.1 | HV936365.1 | AX555196.1 | A02532.1 | HV513361.1 | CS389188.1 | BD399187.1 | M19103.1 | GM635203.1 |
| HC047009.1 | HV936315.1 | AX554977.1 | CS259577.1 | HV513329.1 | CS401603.1 | CS054358.1 | DQ250204.1 | GM635171.1 |
| HC046977.1 | HV925257.1 | AX551075.1 | DD214010.1 | HV513077.1 | DL094969.1 | CS053086.1 | DQ250172.1 | GM635139.1 |
| HC046945.1 | HV343856.1 | AX544471.1 | DD213993.1 | HV508659.1 | DL106655.1 | CS052956.1 | AF422193.1 | GM635107.1 |
| HC046913.1 | HV343664.1 | AX539574.1 | DD213900.1 | HI988928.1 | DL106559.1 | CS052879.1 | U72062.1 | GM635075.1 |
| HC046885.1 | HV343520.1 | AX538268.1 | DD213868.1 | HI987432.1 | DL106527.1 | CS052395.1 | M24231.1 | GM630269.1 |
| HC046853.1 | HV340905.1 | AX537936.1 | DD213836.1 | FW511795.1 | DL128657.1 | CS052360.1 | HW391099.1 | GM630237.1 |
| HC046821.1 | CS597736.1 | AX537250.1 | DD216437.1 | FW559257.1 | DL125062.1 | CS052326.1 | HW408634.1 | GM630205.1 |
| HC046789.1 | CS585653.1 | AX528952.1 | DD213577.1 | FW552302.1 | DL125030.1 | CS052293.1 | HW390746.1 | GM698702.1 |
| HC046757.1 | CS585069.1 | AX528812.1 | DD213545.1 | FW552268.1 | DL124998.1 | CS050991.1 | HW405830.1 | GM736997.1 |
| HC046725.1 | CS584685.1 | AX528114.1 | DD215947.1 | FW552231.1 | DL124966.1 | CS050956.1 | HW399319.1 | GM635040.1 |
| HC046693.1 | CS588491.1 | AX524910.1 | DD222032.1 | FW562041.1 | DL124951.1 | CS048069.1 | HW399217.1 | GM635008.1 |
| HC046676.1 | CS581925.1 | AX523920.1 | DD212248.1 | FW552170.1 | DL124919.1 | CS047743.1 | HW408982.1 | GM634976.1 |
| HC046644.1 | CS574846.1 | AX523638.1 | DD211834.1 | FW552135.1 | DL124887.1 | CS047265.1 | HW238556.1 | GM634944.1 |
| HC046612.1 | CS574795.1 | AX179495.1 | DD192527.1 | FW561784.1 | DL113546.1 | CS046587.1 | HW238396.1 | GM634880.1 |
| HC046580.1 | CS573131.1 | AX179437.1 | DD182427.1 | FW557297.1 | DL113514.1 | CS039317.1 | HW238266.1 | FB764089.1 |
| HC046548.1 | CS572374.1 | AX027964.1 | AX548050.1 | FW561669.1 | DL113450.1 | CS039285.1 | HW244248.1 | FB746527.1 |
| HC046516.1 | CS546790.1 | A09547.1 | AX544094.1 | FW557011.1 | DL093139.1 | CS039253.1 | HW237888.1 | FB743975.1 |
| HC046484.1 | CS570592.1 | AX521543.1 | AX540473.1 | FW566745.1 | DL093075.1 | CS039221.1 | HW248677.1 | FB743927.1 |
| HC046452.1 | CS155789.1 | AX521511.1 | AX528971.1 | FW505123.1 | DL089300.1 | CS039175.1 | HW237376.1 | FB743895.1 |
| HC046388.1 | CS148772.1 | AX513489.1 | AX528459.1 | FW508118.1 | DL089268.1 | CS038883.1 | HW242311.1 | FB743863.1 |
| HC046356.1 | CS145671.1 | AF430197.1 | AX525463.1 | FW555402.1 | DL089236.1 | CS038834.1 | HW243424.1 | FB761582.1 |
| HC046324.1 | CS144356.1 | AF430165.1 | A30505.1 | FW553183.1 | DL136981.1 | CS037904.1 | HW243087.1 | FB761227.1 |
| DM038572.1 | CS141584.1 | AX511456.1 | AX179471.1 | FW553104.1 | DL125927.1 | CS037245.1 | HW241448.1 | FB760064.1 |
| DM022149.1 | CS141531.1 | AX505219.1 | AX164111.1 | FW556667.1 | DL125895.1 | CS030147.1 | GM638751.1 | GM720576.1 |
| DM015976.1 | CS141499.1 | AX505155.1 | AX027983.1 | FW559391.1 | DL122557.1 | CS030019.1 | FB583257.1 | GM035173.1 |
| DM027072.1 | CS141140.1 | AX498431.1 | A28405.1 | FW559343.1 | DL122525.1 | CS027609.1 | FB573329.1 | GM890112.1 |
| DM026273.1 | CS140725.1 | AX497109.1 | AX521668.1 | FW506879.1 | DL118079.1 | CS027368.1 | DL200045.1 | GM889317.1 |
| GN045758.1 | CS132060.1 | AX493022.1 | AX521530.1 | FW510489.1 | DL118047.1 | CS025566.1 | DL195026.1 | GM009368.1 |
| GN041642.1 | CS129755.1 | BD140192.1 | AX521498.1 | HI002109.1 | DL118015.1 | CS023768.1 | DL196189.1 | GM008854.1 |
| GN037872.1 | CS123570.1 | BD139560.1 | AX512777.1 | HI000454.1 | DL103631.1 | CS022582.1 | DL193789.1 | GM007129.1 |
| GN037293.1 | CS123349.1 | BD138750.1 | AF430152.1 | HI000406.1 | DL099025.1 | CS021424.1 | DL193757.1 | GM868810.1 |

| | | | | | | | | |
|---|---|---|---|---|---|---|---|---|
| GN034785.1 | CS122622.1 | BD138408.1 | AX505193.1 | HI000364.1 | DL098993.1 | CS018499.1 | DL183798.1 | GM867484.1 |
| GN030744.1 | CS122258.1 | BD137460.1 | AX504296.1 | HI553292.1 | DL098961.1 | CS018357.1 | DL183391.1 | GM685440.1 |
| GN030712.1 | CS106087.1 | BD137146.1 | AX496851.1 | HI553188.1 | DL088984.1 | CS017236.1 | FB513954.1 | GM003572.1 |
| GN030680.1 | CS179759.1 | BD135872.1 | BD139977.1 | HI201812.1 | DL021456.1 | CS016849.1 | FB513364.1 | FB983565.1 |
| GN030648.1 | CS179192.1 | BD134011.1 | BD138871.1 | HI583972.1 | DL021424.1 | CS016629.1 | FB571354.1 | FB983208.1 |
| GN030616.1 | CS326353.1 | BD133199.1 | BD138733.1 | HI583875.1 | DL021392.1 | CS016484.1 | FB571298.1 | FB983191.1 |
| GN030584.1 | CS323680.1 | BD130843.1 | BD138552.1 | HI568989.1 | DL017186.1 | CS016123.1 | FB571238.1 | FB754265.1 |
| GN030552.1 | CS323598.1 | BD130806.1 | BD137962.1 | HI207670.1 | DL017154.1 | CS016051.1 | FB570900.1 | FB753867.1 |
| GN030520.1 | CS330149.1 | BD130774.1 | BD137418.1 | HI003700.1 | DL017122.1 | CQ986641.1 | CS696190.1 | GM865637.1 |
| GN030488.1 | CS329671.1 | BD130708.1 | BD136675.1 | HV966040.1 | DL017090.1 | CQ986607.1 | CS696158.1 | GM751799.1 |
| GN030328.1 | CS319462.1 | BD130628.1 | BD136047.1 | HV958118.1 | DL017058.1 | CQ986575.1 | CS696062.1 | FB727123.1 |
| GN030232.1 | CS318712.1 | BD105803.1 | BD135139.1 | HV969961.1 | DL017026.1 | CQ986541.1 | CS696030.1 | FB726022.1 |
| GN030200.1 | CS322149.1 | BD090572.1 | BD134644.1 | HV962267.1 | DL049425.1 | CQ986509.1 | CS695965.1 | FB725816.1 |
| GN030136.1 | DD270941.1 | BD087675.1 | BD131932.1 | HV961662.1 | DL049393.1 | CQ983161.1 | CS695837.1 | GM842425.1 |
| DL108996.1 | DD261016.1 | BD085731.1 | BD130825.1 | HV951625.1 | DL049361.1 | CQ796796.1 | CS695805.1 | FB723048.1 |
| DL104294.1 | DD259172.1 | BD081942.1 | BD130793.1 | HV951561.1 | DL049329.1 | CQ795499.1 | CS695773.1 | GM731840.1 |
| DL104262.1 | DD265467.1 | BD081515.1 | BD130761.1 | HV951529.1 | DL068457.1 | CQ794833.1 | CS695709.1 | GM879665.1 |
| DL104230.1 | CS302590.1 | BD080684.1 | BD130727.1 | HV950642.1 | DL041080.1 | CQ792422.1 | CS695517.1 | GM723333.1 |
| DL099592.1 | CS302519.1 | BD080145.1 | BD130695.1 | HV950453.1 | DL037129.1 | CQ789698.1 | CS695389.1 | FB712064.1 |
| DL099560.1 | CS299523.1 | BD078590.1 | BD130598.1 | HV950192.1 | DL028662.1 | CQ788512.1 | AX047299.1 | GM041774.1 |
| DL093454.1 | CS300491.1 | AX009470.1 | BD129645.1 | HV957585.1 | DL028630.1 | CQ787494.1 | DL176486.1 | GM040848.1 |
| DL093422.1 | DD252138.1 | AX006974.1 | BD105713.1 | HV952255.1 | DL028598.1 | CQ787462.1 | DL176349.1 | GM840198.1 |
| DL093390.1 | DD251996.1 | AX006466.1 | BD103157.1 | HV951893.1 | DL028566.1 | CQ787426.1 | DL176275.1 | FB709180.1 |
| DL093358.1 | DD251606.1 | AX004123.1 | BD084151.1 | HV946433.1 | DL015157.1 | CQ787386.1 | DL181651.1 | CS728645.1 |
| DL126135.1 | DD248874.1 | AX003073.1 | BD082038.1 | HV945919.1 | DL010539.1 | CQ787350.1 | DL107958.1 | DL106858.1 |
| DL126103.1 | DD258247.1 | AX002971.1 | BD080777.1 | HV939950.1 | DL010507.1 | CQ787314.1 | DL107926.1 | DL106826.1 |
| DL122751.1 | DD257968.1 | A48495.1 | BD080663.1 | HV943381.1 | DL010475.1 | CQ787282.1 | DL107894.1 | DL106794.1 |
| DL122663.1 | DD257323.1 | A40272.1 | BD080132.1 | HV942055.1 | DL010443.1 | CQ787250.1 | DL088597.1 | DL106762.1 |
| DL122567.1 | DD253719.1 | A35772.1 | BD074974.1 | HV932242.1 | DL010411.1 | CQ786958.1 | DL113039.1 | DL106730.1 |
| DL118377.1 | CS287700.1 | A35712.1 | BD074938.1 | HV929187.1 | DL038785.1 | CQ785944.1 | DL092354.1 | DL106698.1 |
| DL118345.1 | DD240759.1 | A34724.1 | BD070056.1 | HH779677.1 | DL038753.1 | CQ784679.1 | DL092322.1 | DL091268.1 |
| DL017364.1 | DD240662.1 | A31760.1 | BD069443.1 | HH777920.1 | DL038721.1 | GM654986.1 | DL092290.1 | DL091236.1 |
| DL017332.1 | DD240492.1 | A32834.1 | HW350969.1 | HH773139.1 | DL035097.1 | GM694857.1 | DL092226.1 | DL091204.1 |
| DL012975.1 | DD240460.1 | A30340.1 | HW350894.1 | HH820334.1 | DL035065.1 | GM694787.1 | DL092194.1 | DL087236.1 |
| DL012943.1 | CS254891.1 | A06234.1 | HW340470.1 | HH759211.1 | DL035033.1 | GM044490.1 | DL092162.1 | DL087204.1 |
| DL041351.1 | DD212333.1 | A29202.1 | HW350670.1 | HH759179.1 | DL030718.1 | GM043810.1 | DL088419.1 | DL102097.1 |
| DL041287.1 | DD212211.1 | HW145133.1 | HW340206.1 | HH759147.1 | DL030686.1 | GM837004.1 | DL088387.1 | DL102065.1 |
| DL037400.1 | DD192242.1 | HW153263.1 | HW340161.1 | HH759083.1 | DL026749.1 | GM694180.1 | DL121787.1 | DL102033.1 |
| DL037368.1 | DD191811.1 | HW152856.1 | HW339896.1 | HH759051.1 | DL026717.1 | GM752862.1 | DL108085.1 | DL097523.1 |
| DL037336.1 | DD209218.1 | HW151683.1 | HW349933.1 | HH759019.1 | DL026685.1 | GM752575.1 | DL037158.1 | DL097491.1 |
| DL021491.1 | DD208696.1 | HW144663.1 | HW347914.1 | HH758987.1 | DL026653.1 | GM659956.1 | DL037126.1 | DL097459.1 |
| DL021459.1 | DD206861.1 | HW126547.1 | HW326536.1 | HH758955.1 | DL026621.1 | GM652761.1 | DL028659.1 | DL095342.1 |
| DL021427.1 | DD206829.1 | HW144378.1 | HW326449.1 | HH758923.1 | DL026589.1 | GM652729.1 | DL028627.1 | DL101884.1 |
| DL021395.1 | DD206799.1 | HW144346.1 | HW338946.1 | HH758891.1 | DL026557.1 | GM652697.1 | DL028595.1 | DL101820.1 |
| DL017189.1 | DD205872.1 | HW144314.1 | HW338690.1 | HH757467.1 | DL019570.1 | GM652665.1 | DL028563.1 | DL101788.1 |
| DL017157.1 | DD163495.1 | HW124955.1 | HW338562.1 | HH757435.1 | DL019538.1 | GM652633.1 | DL048682.1 | DL101756.1 |
| DL017125.1 | DD181028.1 | HW124816.1 | HW338434.1 | HH756188.1 | DL019506.1 | GM652601.1 | DL009538.1 | DL101724.1 |

| | | | | | | | | |
|---|---|---|---|---|---|---|---|---|
| DL017093.1 | DD163291.1 | HW124657.1 | HW338306.1 | HI401458.1 | DL019474.1 | GM630665.1 | DL009506.1 | DL097214.1 |
| DL017061.1 | CS119381.1 | HW124524.1 | M27311.1 | HI401089.1 | DL019442.1 | GM630633.1 | DL009474.1 | DL097182.1 |
| DL017029.1 | CS119349.1 | HV964042.1 | K03392.1 | HI645012.1 | DL019410.1 | GM630601.1 | DL009442.1 | DL097150.1 |
| DL049428.1 | CS119316.1 | HV970070.1 | AY575070.1 | HI642589.1 | DL014911.1 | GM621730.1 | DL034323.1 | DL095226.1 |
| DL049396.1 | CS119283.1 | HV963865.1 | HC918491.1 | HI642045.1 | DL014879.1 | GM621698.1 | DL009343.1 | DL095194.1 |
| DL049364.1 | CS119219.1 | HV963657.1 | HC917435.1 | HI641501.1 | DL014847.1 | GM621666.1 | DL009311.1 | DL095162.1 |
| DL049332.1 | CS119187.1 | HV966042.1 | FW341964.1 | HI640957.1 | DL026497.1 | GM621634.1 | DL009279.1 | AX240993.1 |
| DL012569.1 | CS119155.1 | HV958129.1 | FW337029.1 | HI637702.1 | DD410615.1 | GM621602.1 | DL009247.1 | AX240961.1 |
| DL012505.1 | CS119123.1 | HV966016.1 | FW340849.1 | HI380402.1 | CS492095.1 | GM621570.1 | DL009215.1 | AX240929.1 |
| DL012473.1 | CS119089.1 | HV965984.1 | HC868149.1 | HI380327.1 | CS493058.1 | GM657118.1 | DL018705.1 | AX235765.1 |
| DL045451.1 | CS119056.1 | HV962269.1 | HC873818.1 | HH998021.1 | CS490616.1 | GM657086.1 | DL018641.1 | AX224249.1 |
| DL045419.1 | CS119023.1 | HV965369.1 | HC880382.1 | HH997961.1 | CS489141.1 | GM657054.1 | DL018609.1 | AX214166.1 |
| DL045387.1 | CS118990.1 | HV961718.1 | FU262481.1 | HH999659.1 | CS486621.1 | GM657022.1 | DL014213.1 | AX207744.1 |
| DL045355.1 | CS118958.1 | HV951627.1 | FU264492.1 | HH997911.1 | CS482999.1 | GM656958.1 | DL014181.1 | AX207278.1 |
| DL031080.1 | CS118859.1 | HV951595.1 | FU258415.1 | HH997839.1 | CS482963.1 | GM656927.1 | DL014149.1 | AX202538.1 |
| DL030955.1 | CS118826.1 | HV951531.1 | HC313237.1 | HH997777.1 | CS482928.1 | GM656895.1 | DL014117.1 | AF401223.1 |
| DL027143.1 | CS118761.1 | HV957836.1 | HC306234.1 | HH999513.1 | CS482883.1 | GM656863.1 | DL014085.1 | AX188575.1 |
| DL027111.1 | CS118728.1 | HV949003.1 | HC306194.1 | HH999461.1 | CS482747.1 | GM656831.1 | DL029970.1 | AX180741.1 |
| DL027079.1 | CS118696.1 | HV957587.1 | HC305998.1 | HH999418.1 | DD401433.1 | GM656799.1 | DD449172.1 | AX172935.1 |
| DL027047.1 | CS118664.1 | HV957322.1 | HC302641.1 | HH999368.1 | DD401401.1 | GM656767.1 | DD449082.1 | HW326545.1 |
| DL027015.1 | CS118632.1 | HV947290.1 | HC302381.1 | HH997654.1 | DD405216.1 | GM649774.1 | DD441638.1 | HW326499.1 |
| DL030910.1 | CS118600.1 | HV946439.1 | HC305927.1 | HH997620.1 | DD405184.1 | GM649742.1 | DD436060.1 | HW326305.1 |
| DL030871.1 | CS118566.1 | HV945923.1 | HC305887.1 | HH997556.1 | DD405152.1 | GM649710.1 | CS620301.1 | HW338582.1 |
| DL030839.1 | CS118532.1 | HV939989.1 | HC305847.1 | HH997468.1 | HV515322.1 | GM649614.1 | CS616491.1 | HW338326.1 |
| DL030807.1 | CS118499.1 | HV945704.1 | HC305807.1 | HH999702.1 | HV515290.1 | GM642806.1 | CS614439.1 | HW338198.1 |
| DL030775.1 | CS118466.1 | HV942760.1 | HC305767.1 | HH999258.1 | HV515258.1 | GM642774.1 | CS613377.1 | HW338070.1 |
| DL030743.1 | CS118434.1 | HV942057.1 | HC305727.1 | HH999157.1 | HV515226.1 | GM642742.1 | CS604538.1 | HW337430.1 |
| DL026774.1 | CS118401.1 | HV816123.1 | HC305687.1 | HH998124.1 | HV511156.1 | GM642710.1 | CS604506.1 | HW337302.1 |
| DL019755.1 | AX593371.1 | HV815895.1 | HC305647.1 | HH999081.1 | HV507928.1 | GM642678.1 | CS604474.1 | HW337174.1 |
| DL019723.1 | AX587858.1 | HV932248.1 | HC294242.1 | HH999040.1 | FW580718.1 | GM642614.1 | CS604410.1 | HW337046.1 |
| DL015224.1 | AX587561.1 | HV932146.1 | HC289382.1 | HH998975.1 | FW591561.1 | GM635432.1 | CS604378.1 | HW321852.1 |
| DL015192.1 | AX589683.1 | HV929191.1 | HC289338.1 | HH998930.1 | FW590726.1 | GM635400.1 | CS604346.1 | HW336918.1 |
| DL015160.1 | BD161128.1 | HV937062.1 | HC292924.1 | HH997146.1 | FW590298.1 | GM635368.1 | CS604314.1 | HW336634.1 |
| DL015032.1 | BD160782.1 | HV931449.1 | HC295987.1 | HH970049.1 | FW589156.1 | GM635336.1 | CS604282.1 | HW336554.1 |
| DL010542.1 | AX587990.1 | HV931378.1 | HC295704.1 | HH998883.1 | FW593441.1 | GM635304.1 | CS604250.1 | HW336388.1 |
| DL010510.1 | AX587957.1 | HV815183.1 | HC288859.1 | HH998823.1 | FW592629.1 | GM635272.1 | CS604058.1 | HW329557.1 |
| DL010478.1 | AX587814.1 | HV112801.1 | HC292494.1 | HH998761.1 | FW585095.1 | GM630466.1 | CS603994.1 | HW329441.1 |
| DL010446.1 | AX587708.1 | HV038761.1 | HC292254.1 | HH997056.1 | FW577641.1 | GM630434.1 | CS603930.1 | HW329074.1 |
| DL010414.1 | AX587607.1 | HV038659.1 | HC294996.1 | HH997007.1 | FW576752.1 | GM630402.1 | CS603482.1 | HW335973.1 |
| DL038788.1 | AX587557.1 | HV038599.1 | HC294816.1 | HH996953.1 | FW576718.1 | GM630370.1 | CS603450.1 | HW328741.1 |
| DL038756.1 | AX577824.1 | HV182327.1 | HC299179.1 | HH998708.1 | FW576679.1 | GM630338.1 | CS603418.1 | HW328560.1 |
| DL038724.1 | AX556826.1 | HV182295.1 | HC294576.1 | HH998671.1 | FW576640.1 | GM630306.1 | CS603386.1 | HW328486.1 |
| DL038692.1 | AX555195.1 | HV193546.1 | HC298735.1 | HH998601.1 | FW575879.1 | GM621531.1 | CS603354.1 | HW328447.1 |
| DL035068.1 | AX554439.1 | HV182361.1 | HC291054.1 | HH998548.1 | FW573830.1 | GM621499.1 | CS603322.1 | HW328339.1 |
| DL034829.1 | AX544469.1 | HV199755.1 | HC290869.1 | HH996925.1 | FW573666.1 | GM739529.1 | CS603290.1 | HW335648.1 |
| DL030721.1 | AX538889.1 | HV234615.1 | HC293457.1 | HH996769.1 | FW573460.1 | GM738888.1 | A12565.1 | HW328162.1 |
| DL030689.1 | AX538325.1 | HV234444.1 | HC293337.1 | HH977648.1 | FW569788.1 | GM698720.1 | A12271.1 | HW318476.1 |

| | | | | | | | | |
|---|---|---|---|---|---|---|---|---|
| DL026752.1 | AX537702.1 | HV312977.1 | HC293140.1 | HH962749.1 | FW571816.1 | GM656721.1 | A06471.1 | HW335180.1 |
| DL026720.1 | AX537200.1 | HV302116.1 | HC289866.1 | HH980570.1 | FW571746.1 | GM656689.1 | A15475.1 | HW335058.1 |
| DL026688.1 | AX528951.1 | HV030249.1 | HC289690.1 | HH980499.1 | HI001310.1 | GM656657.1 | A08113.1 | HW314152.1 |
| DL026656.1 | AX528810.1 | HV036228.1 | HC207402.1 | HH980431.1 | HI003197.1 | GM656625.1 | A07731.1 | HW314025.1 |
| DL026624.1 | AX528113.1 | HV031612.1 | HC199625.1 | HH957934.1 | HI003135.1 | GM656593.1 | A07220.1 | HW311064.1 |
| DL026592.1 | AX524909.1 | FZ435970.1 | HC198907.1 | HH980358.1 | HI003075.1 | GM656561.1 | A05953.1 | HW307938.1 |
| DL026560.1 | AX523637.1 | FZ435806.1 | M62894.1 | HH980298.1 | HI003039.1 | GM649568.1 | A04847.1 | HW307864.1 |
| DJ051987.1 | A32490.1 | FZ425939.1 | DM464644.1 | HH980223.1 | HI002998.1 | GM649536.1 | A02741.1 | HW307832.1 |
| DJ050213.1 | A33403.1 | FZ437388.1 | HB394298.1 | HH980159.1 | HI002899.1 | GM649504.1 | A05108.1 | HW307800.1 |
| DJ048836.1 | AX179429.1 | FZ424104.1 | HB394266.1 | HH980126.1 | HI001262.1 | GM649472.1 | A02712.1 | HW307768.1 |
| DJ048804.1 | AX027963.1 | FZ423930.1 | HB394234.1 | HH979881.1 | HI001202.1 | GM649440.1 | A00764.1 | HW307762.1 |
| CS793897.1 | A28426.1 | FZ432340.1 | DM115563.1 | HH996710.1 | HI001151.1 | GM649408.1 | A00095.1 | HW307730.1 |
| CS721585.1 | A27781.1 | FZ428241.1 | DM114856.1 | HH996644.1 | HI001050.1 | GM670048.1 | HV784204.1 | HW307698.1 |
| CS800088.1 | A26375.1 | FZ431980.1 | GM061279.1 | HH996597.1 | HI001014.1 | GM656543.1 | HV780446.1 | HW315943.1 |
| CS707437.1 | A25744.1 | FZ422766.1 | HB387457.1 | HH996548.1 | HI000969.1 | GM656511.1 | HV778811.1 | HW081766.1 |
| CS716776.1 | A22989.1 | FZ422534.1 | HB384534.1 | GM647250.1 | HI002873.1 | GM656479.1 | HV778018.1 | HW081677.1 |
| CS796339.1 | A22094.1 | FZ413359.1 | HB338860.1 | GM647218.1 | HI002838.1 | GM656447.1 | HV777559.1 | HW085071.1 |
| CS715285.1 | A21889.1 | FZ421716.1 | HA642840.1 | GM627584.1 | HI002800.1 | GM656415.1 | HV775696.1 | HW103378.1 |
| CS792478.1 | A21730.1 | HI214567.1 | HA641590.1 | GM627552.1 | HI002741.1 | GM656383.1 | HV775021.1 | HW098198.1 |
| DJ044952.1 | A21422.1 | HI214535.1 | HA641267.1 | GM627520.1 | HI002693.1 | GM656351.1 | HV764729.1 | HW102319.1 |
| HC306087.1 | A20279.1 | HI214437.1 | HA638542.1 | GM661493.1 | HI002621.1 | GM649358.1 | HV769969.1 | HW061849.1 |
| HC306047.1 | A19443.1 | HI213770.1 | HA637701.1 | GM661461.1 | HI002584.1 | GM649300.1 | HV763994.1 | HW058815.1 |
| HC306007.1 | A12708.1 | HI213010.1 | HA643915.1 | GM661429.1 | HI004537.1 | GM649318.1 | HV774116.1 | HW065231.1 |
| HC305967.1 | A19410.1 | HI212963.1 | GN368296.1 | GM661397.1 | HI004409.1 | GM649262.1 | HV766847.1 | HW064872.1 |
| HC302013.1 | A18339.1 | HI212611.1 | GM987030.1 | GM661365.1 | HI002435.1 | GM649230.1 | HV760600.1 | HW064833.1 |
| HC301628.1 | A17475.1 | HI553352.1 | GM981438.1 | GM661333.1 | HI002313.1 | GM649198.1 | HV766282.1 | HW061118.1 |
| HC294326.1 | A15718.1 | HI546322.1 | GM984659.1 | GM654339.1 | HI000516.1 | GM642589.1 | HV766032.1 | HW056843.1 |
| HC251377.1 | A14630.1 | HI546284.1 | FB740450.1 | GM654307.1 | HI006839.1 | GM642557.1 | HV755859.1 | HW056525.1 |
| HC294050.1 | A13735.1 | HI586986.1 | GM596705.1 | GM654243.1 | HI180160.1 | GM642525.1 | HV750177.1 | HW064729.1 |
| HC289410.1 | A13364.1 | HI211000.1 | GM970126.1 | GM647186.1 | HI178192.1 | GM642517.1 | HV504762.1 | HW064655.1 |
| HC289345.1 | A12697.1 | HI210968.1 | GM969724.1 | GM647154.1 | HI202832.1 | GM642460.1 | HV504730.1 | HW060700.1 |
| HC289313.1 | A11183.1 | HI210936.1 | FB777934.1 | GM647122.1 | HI214560.1 | GM642428.1 | HV504698.1 | HW062400.1 |
| HC296933.1 | A09360.1 | HI570412.1 | GM009600.1 | GM647090.1 | HI214528.1 | GM635246.1 | HV504666.1 | HW060516.1 |
| HC295717.1 | A06533.1 | HI560778.1 | DL477930.1 | CS695812.1 | HI200846.1 | GM635214.1 | HV504634.1 | HW060449.1 |
| HC288867.1 | A16719.1 | HI473033.1 | DL477726.1 | CS695716.1 | HI214430.1 | GM635182.1 | HV504602.1 | HW062375.1 |
| HC288830.1 | A07924.1 | HI472909.1 | DL464542.1 | CS695652.1 | HI212999.1 | GM635150.1 | HV504985.1 | HW056170.1 |
| HC292588.1 | A07273.1 | HI004377.1 | DL476943.1 | CS695620.1 | HI212947.1 | GM635118.1 | HV504227.1 | HW042066.1 |
| HC292338.1 | A05995.1 | HI004313.1 | DL476691.1 | CS695524.1 | HI203497.1 | DL039396.1 | HV455720.1 | HW042034.1 |
| HC294900.1 | A05141.1 | HI004249.1 | DL463062.1 | CS695492.1 | HI546347.1 | DL039364.1 | HV503807.1 | HW043564.1 |
| HC299187.1 | A01050.1 | HI002278.1 | DL470101.1 | CS695460.1 | HI546315.1 | DL039332.1 | HV491262.1 | HW041990.1 |
| HC299129.1 | A07983.1 | HI002241.1 | DL462900.1 | CS695428.1 | HI546145.1 | DL039300.1 | HV503204.1 | HW041926.1 |
| HC294660.1 | A01733.1 | HI002191.1 | DL482391.1 | CS695396.1 | HI210993.1 | DL035676.1 | HV503172.1 | HW041864.1 |
| HB485559.1 | A05199.1 | DL088936.1 | DL461717.1 | DL176507.1 | HI210961.1 | DL035644.1 | HV503140.1 | HW041828.1 |
| HB475846.1 | A04254.1 | DL088904.1 | DL475553.1 | DL176448.1 | HI573196.1 | DL035612.1 | HV503108.1 | HW049412.1 |
| HB469151.1 | A01348.1 | DL103279.1 | DL461257.1 | DL176285.1 | HI570399.1 | DL035580.1 | HV503076.1 | HW049380.1 |
| HB468032.1 | A00200.1 | DL103247.1 | DL461184.1 | DL174623.1 | HC877784.1 | DL035548.1 | HV502807.1 | HW049349.1 |
| DM148873.1 | M24177.1 | DL103215.1 | FB504563.1 | DL164122.1 | FW304462.1 | DL035516.1 | HV502776.1 | HW049285.1 |

| | | | | | | | | |
|---|---|---|---|---|---|---|---|---|
| DM148778.1 | M17413.1 | DL107967.1 | GM659577.1 | DL176772.1 | FW310243.1 | DL031448.1 | HV502744.1 | HW049253.1 |
| DM152689.1 | M12680.1 | DL107935.1 | GM828148.1 | DL176684.1 | FW331770.1 | DL031416.1 | HV502712.1 | HI372211.1 |
| DM152537.1 | M13110.1 | DL107903.1 | FB509202.1 | FB344503.1 | FW309550.1 | DL031384.1 | HV502648.1 | HI369624.1 |
| DM156192.1 | M19415.1 | DL092445.1 | GM742039.1 | FB342768.1 | FW308905.1 | DL031352.1 | HV502552.1 | HI369311.1 |
| DM144904.1 | M12689.1 | DL092413.1 | GM709003.1 | DL088089.1 | FW308767.1 | DL027543.1 | HV502456.1 | HI369159.1 |
| HB455508.1 | M19083.1 | DL092381.1 | GM627574.1 | DL088057.1 | FW334249.1 | DL027511.1 | HV505368.1 | HI369104.1 |
| HB455303.1 | LC733661.1 | DL088606.1 | GM661483.1 | DL088025.1 | FW332547.1 | DL027447.1 | HV505289.1 | HI369063.1 |
| DM134732.1 | MF989993.1 | DL112952.1 | HI657458.1 | DL087993.1 | FW306410.1 | DL027415.1 | HH804809.1 | HI368997.1 |
| DM143065.1 | L35893.1 | DL098533.1 | FW503726.1 | DL091934.1 | FW332166.1 | DL027383.1 | HH794941.1 | HI368091.1 |
| DM137619.1 | AY071819.1 | DL092331.1 | FW420460.1 | DL091902.1 | HC769894.1 | DL023571.1 | HH794709.1 | HI424132.1 |
| DM137107.1 | AH002281.2 | DL092299.1 | FW420371.1 | DL091870.1 | HC757177.1 | DL023539.1 | HH793122.1 | HI424100.1 |
| GN346473.1 | M30600.1 | DL092267.1 | FW420339.1 | DL091838.1 | HC757145.1 | DL023507.1 | HH792578.1 | HI424068.1 |
| GN343929.1 | M30040.1 | DL088556.1 | FW496638.1 | DL091774.1 | HC742874.1 | DL023475.1 | HH779965.1 | DL028131.1 |
| GN342150.1 | DQ250245.1 | DL088524.1 | HI653555.1 | DL102895.1 | HC755773.1 | DL023443.1 | HH779667.1 | DL028099.1 |
| GN346580.1 | AY569034.1 | DL088492.1 | HI653393.1 | DL102863.1 | HC046640.1 | DL023411.1 | HH774425.1 | DL028067.1 |
| GN346548.1 | AY061816.1 | DL088460.1 | HI651015.1 | DL102799.1 | HC046608.1 | DL023379.1 | HH759309.1 | DL028035.1 |
| GN131007.1 | U89686.1 | DL088428.1 | HI002648.1 | DL102767.1 | HC046576.1 | DL020364.1 | HI401263.1 | DL028003.1 |
| GN131887.1 | S43266.1 | DL117796.1 | HI002614.1 | DL102735.1 | HC046544.1 | DL020332.1 | HI401117.1 | DL027971.1 |
| GN116527.1 | KP784699.1 | DL117764.1 | HI002575.1 | DL107579.1 | HC046512.1 | DL020300.1 | HI643101.1 | DL044236.1 |
| GN116495.1 | KJ598132.1 | DL117732.1 | HI004523.1 | DL107487.1 | HC046480.1 | DL020268.1 | HI505032.1 | DL044204.1 |
| GN116462.1 | HW408611.1 | DL117700.1 | HI002487.1 | DL107455.1 | HC046448.1 | DL020236.1 | HI462679.1 | DL044172.1 |
| GN116430.1 | HW158846.1 | DL117668.1 | HI002419.1 | DL107391.1 | HC046416.1 | DJ443503.1 | HH725220.1 | DL048245.1 |
| GN116398.1 | HW158782.1 | DL117636.1 | HI002306.1 | DL102586.1 | HC046384.1 | DJ443471.1 | HH716005.1 | DL048213.1 |
| GN116366.1 | HW158487.1 | DL122095.1 | HI000508.1 | DL102554.1 | HC046352.1 | DJ438368.1 | HH714038.1 | DL048181.1 |
| GN116334.1 | HW154646.1 | DL125740.1 | HI180153.1 | DL102522.1 | HC046069.1 | DJ438295.1 | HH713504.1 | DL048149.1 |
| GN116302.1 | HW154479.1 | DL125708.1 | HI178171.1 | DL098012.1 | HC046037.1 | DJ427991.1 | HH961374.1 | DL048053.1 |
| GN115066.1 | HW154447.1 | DL125676.1 | HI202825.1 | DL097980.1 | HC045999.1 | DJ402688.1 | HH961342.1 | DL047862.1 |
| GN094509.1 | HW147481.1 | DL125644.1 | HI214585.1 | DL097948.1 | HC045903.1 | DJ402652.1 | HH961310.1 | CS803329.1 |
| GN094477.1 | HW147437.1 | DL125612.1 | HI214553.1 | DL112638.1 | HC045839.1 | DJ402292.1 | HH961278.1 | DJ052639.1 |
| DM078964.1 | HW147122.1 | DL125580.1 | HI214520.1 | DL112606.1 | AX356491.1 | DJ416099.1 | HH961246.1 | DJ046983.1 |
| DM078721.1 | HW154023.1 | DL125548.1 | HI179966.1 | DL112574.1 | AX353676.1 | DJ401099.1 | HH975449.1 | DJ045450.1 |
| DM069071.1 | HW153797.1 | DL070829.1 | HI214455.1 | DL112542.1 | AX350622.1 | DJ400811.1 | HH982026.1 | DJ050209.1 |
| GM983103.1 | HW153761.1 | DL031643.1 | HI214423.1 | DL112510.1 | AX349183.1 | DJ393227.1 | HH974537.1 | DJ049443.1 |
| GM996585.1 | HW145660.1 | DL031611.1 | HI212988.1 | DL112478.1 | AX348856.1 | DJ419933.1 | HH998106.1 | DJ048832.1 |
| GM996489.1 | FZ415919.1 | DL031579.1 | HI546340.1 | DL119926.1 | AX347416.1 | DJ415905.1 | HH998055.1 | CS704144.1 |
| GN010002.1 | FZ421436.1 | DL031547.1 | HI546308.1 | DL119894.1 | AX347318.1 | DJ398325.1 | HH998000.1 | CS721581.1 |
| GM993010.1 | FZ421345.1 | DL027738.1 | HI210986.1 | DL119862.1 | AX347242.1 | DJ418072.1 | HH997940.1 | CS800053.1 |
| GM992390.1 | FZ418102.1 | DL027706.1 | HI210954.1 | DL119830.1 | AX347210.1 | DJ418808.1 | HH999649.1 | CS720081.1 |
| GM977072.1 | FZ417834.1 | DL027674.1 | HI472895.1 | DL115235.1 | AX347172.1 | DJ418649.1 | HH997900.1 | CS717364.1 |
| GM992097.1 | FZ420788.1 | DL027642.1 | HI580541.1 | DL115203.1 | AX346994.1 | DJ417444.1 | HH997829.1 | CS798837.1 |
| GM991959.1 | FZ416707.1 | DL027610.1 | HI209404.1 | DL115171.1 | AX345012.1 | DJ402733.1 | HH997763.1 | CS716768.1 |
| GM976409.1 | FZ419860.1 | DL027578.1 | HI004349.1 | DL115139.1 | AX343728.1 | DJ386641.1 | HH999498.1 | CS716108.1 |
| GM990848.1 | FZ419824.1 | DL023670.1 | HI004285.1 | DL115107.1 | AX339638.1 | DJ381012.1 | HH999444.1 | CS796334.1 |
| GM989488.1 | FZ419792.1 | DL023638.1 | HI002264.1 | DL115075.1 | AX338480.1 | DJ380980.1 | HH999316.1 | CS715273.1 |
| GN000575.1 | FZ416678.1 | DL023606.1 | HI002227.1 | DL115043.1 | AX329382.1 | DJ380948.1 | HH997642.1 | CS805311.1 |
| GN000322.1 | FZ416526.1 | DL023574.1 | HI002175.1 | DL110043.1 | AX328276.1 | DJ380916.1 | HH997377.1 | CS680728.1 |
| GM969576.1 | FW582555.1 | DL015996.1 | HI002130.1 | DL110011.1 | AX323102.1 | DJ380884.1 | HH999691.1 | CS792474.1 |

| | | | | | | | | |
|---|---|---|---|---|---|---|---|---|
| GM980876.1 | HI003361.1 | DL015964.1 | HI002094.1 | DL109979.1 | AX319363.1 | DJ380852.1 | HH999238.1 | CS791969.1 |
| FB986339.1 | HI003324.1 | DL015932.1 | HI000432.1 | DL109765.1 | HW158473.1 | DJ380832.1 | HH999196.1 | DJ044926.1 |
| GM970135.1 | HI071577.1 | DL015900.1 | HI000393.1 | DL109733.1 | HW154723.1 | DJ380370.1 | HH999147.1 | DJ044885.1 |
| FB736135.1 | HI464730.1 | DL015868.1 | HI179291.1 | DL109701.1 | HW154637.1 | DJ389570.1 | HH998110.1 | DJ028180.1 |
| GM969731.1 | HI464698.1 | DL011250.1 | HI553279.1 | DL104967.1 | HW154470.1 | DJ389315.1 | DL102488.1 | DJ030376.1 |
| GM715850.1 | HI464666.1 | DL011218.1 | HI553207.1 | DL104935.1 | HW154435.1 | DJ389232.1 | DL102456.1 | DJ029983.1 |
| DL479997.1 | HI464624.1 | DL047642.1 | HI553175.1 | DL100489.1 | HW154377.1 | DJ388704.1 | DL102424.1 | DJ026322.1 |
| DL476965.1 | HI464423.1 | DL047610.1 | HC485137.1 | DL100393.1 | HW158133.1 | DJ388662.1 | DL097587.1 | CS721632.1 |
| DL463069.1 | HI542547.1 | DL047578.1 | HC467116.1 | DL100361.1 | HW153786.1 | DJ388479.1 | DL097555.1 | CS691345.1 |
| DL462975.1 | HI504380.1 | DL047546.1 | HC466626.1 | DL100329.1 | HW157491.1 | CS726744.1 | DL095432.1 | CS693536.1 |
| DL469766.1 | HI462699.1 | DL039683.1 | HC474628.1 | DL124142.1 | HW151817.1 | CS725929.1 | DL095400.1 | CS682284.1 |
| DL475567.1 | HI503821.1 | DL039651.1 | HC491781.1 | DL124110.1 | HW151268.1 | CS724509.1 | DL095368.1 | CS674189.1 |
| DL481624.1 | HH713802.1 | DL039619.1 | HC491749.1 | DL119792.1 | HW159795.1 | CS724425.1 | DL107084.1 | CS691851.1 |
| GM832151.1 | HH961353.1 | DL039587.1 | HC491641.1 | DL119760.1 | HW159752.1 | CS723752.1 | DL107052.1 | DJ012061.1 |
| GM712164.1 | HH961321.1 | DL039555.1 | HC481630.1 | DL119728.1 | HW144739.1 | CS721840.1 | DL106988.1 | CS254952.1 |
| GM675493.1 | HH961289.1 | DL039523.1 | HC491076.1 | DL119696.1 | HW125662.1 | DJ352129.1 | DL106956.1 | CS252549.1 |
| GM657011.1 | HH961257.1 | DL039491.1 | FV531704.1 | DL119664.1 | HW144447.1 | DJ357788.1 | DL106924.1 | CS249832.1 |
| GM656979.1 | HH975360.1 | DL043463.1 | FV531672.1 | DL119632.1 | HW144369.1 | DJ357756.1 | DL106884.1 | CS250639.1 |
| GM656947.1 | HH974650.1 | DL043431.1 | FV523705.1 | DL114909.1 | HW144305.1 | DJ357724.1 | DL106851.1 | CS247226.1 |
| GM656916.1 | HH974548.1 | DL043399.1 | FV531004.1 | DL114877.1 | HW124885.1 | DJ357690.1 | DL106819.1 | CS245378.1 |
| GM656884.1 | HH963813.1 | DL043367.1 | FV522842.1 | DL114845.1 | HW124421.1 | DJ357658.1 | DL106787.1 | CS244159.1 |
| GM656852.1 | HH998075.1 | DL043335.1 | FV522802.1 | DJ066777.1 | HW124051.1 | DJ357626.1 | DL091229.1 | CS244239.1 |
| GM656788.1 | HH998022.1 | DL043271.1 | FV530131.1 | DJ066381.1 | HW123499.1 | DJ357594.1 | DL091197.1 | CS244207.1 |
| GM656756.1 | HH997963.1 | DL039454.1 | FV528438.1 | DJ066349.1 | HW123420.1 | DJ340793.1 | DL087229.1 | CS243163.1 |
| GM635357.1 | HH999660.1 | DL039422.1 | FV534509.1 | DJ066316.1 | HW122427.1 | DJ340960.1 | DL087197.1 | CS239692.1 |
| GM630487.1 | HH997916.1 | DL039390.1 | FV534405.1 | DJ066282.1 | HW120956.1 | DJ350147.1 | DL113540.1 | CS237725.1 |
| HI423842.1 | HH997840.1 | DL039358.1 | FV534079.1 | DJ066248.1 | HW120090.1 | DJ362394.1 | DL125921.1 | CS229988.1 |
| HI423810.1 | HH997778.1 | DL039326.1 | HC465622.1 | DJ065934.1 | HV932913.1 | DJ354942.1 | DL125889.1 | CS203493.1 |
| HI422962.1 | HH999514.1 | DL039294.1 | HC460862.1 | DJ067994.1 | HV932685.1 | DJ361250.1 | DL118105.1 | CS227331.1 |
| HH762704.1 | HH999462.1 | DL035670.1 | AY181092.1 | DJ067982.1 | HV802905.1 | DJ361216.1 | DL118073.1 | CS227248.1 |
| HI415780.1 | HH999419.1 | DL035606.1 | AF394662.1 | DJ055492.1 | HV818562.1 | CS602183.1 | DL118041.1 | CS205999.1 |
| HI414035.1 | HH999369.1 | DL035574.1 | HC456307.1 | DJ055284.1 | HV778609.1 | CS611210.1 | DL108371.1 | CS208249.1 |
| HI413998.1 | HH997655.1 | DL035542.1 | HC453701.1 | DJ060406.1 | HV777569.1 | CS611087.1 | DL025215.1 | CS207914.1 |
| HI413439.1 | HH997621.1 | DL035510.1 | HC453669.1 | DJ053435.1 | HV775752.1 | CS610143.1 | DL013126.1 | CS189621.1 |
| HH760625.1 | HH997564.1 | DL031506.1 | HC460364.1 | DJ053111.1 | HV764764.1 | CS608389.1 | DL013094.1 | CS193222.1 |
| HI516585.1 | HH997474.1 | DL031442.1 | HC452132.1 | DJ053079.1 | HV764551.1 | CS607928.1 | DL013062.1 | CS187878.1 |
| HI516510.1 | HH999705.1 | DL031410.1 | HB412735.1 | CS810635.1 | HV774525.1 | CS607760.1 | DL013030.1 | CS185174.1 |
| HI516110.1 | HH999260.1 | DL031378.1 | HB394328.1 | CS810525.1 | HV763997.1 | CS607539.1 | DL012998.1 | CS177183.1 |
| HI516078.1 | HH999158.1 | DJ055287.1 | DM115561.1 | CS813030.1 | HV560566.1 | CS600680.1 | DL038126.1 | CS174651.1 |
| HI547562.1 | HH998125.1 | DJ055205.1 | DM114854.1 | FB292234.1 | HV554551.1 | CS607480.1 | DL029524.1 | CS172472.1 |
| HI571660.1 | HH999082.1 | DJ057676.1 | GM061277.1 | CS810877.1 | HV553176.1 | CS600279.1 | DL029492.1 | CS172328.1 |
| HI574518.1 | HH999042.1 | DJ060409.1 | HB385791.1 | DJ016605.1 | HV551404.1 | CS607096.1 | DL047456.1 | CS166436.1 |
| HI574486.1 | HH998985.1 | DJ056888.1 | GN368623.1 | CS803333.1 | HV550396.1 | CS599888.1 | DL039314.1 | CS159796.1 |
| HI574454.1 | HH998931.1 | DJ053500.1 | HB340024.1 | FB293491.1 | AY659398.1 | CQ854060.1 | DL023553.1 | CS159369.1 |
| HI574422.1 | HH997209.1 | DJ053214.1 | HB338854.1 | DD405590.1 | AY659366.1 | CQ849425.1 | DL023521.1 | CS159183.1 |
| HI574390.1 | HH997147.1 | DJ053082.1 | HA641586.1 | DD405558.1 | AY659334.1 | CQ848447.1 | DL023489.1 | CS150837.1 |
| HI574367.1 | HH970050.1 | CS810638.1 | HA641522.1 | DD405526.1 | AY659302.1 | AX073451.1 | DL023457.1 | CS146541.1 |

| | | | | | | | | |
|---|---|---|---|---|---|---|---|---|
| HI566204.1 | HH998884.1 | CS810531.1 | HA640511.1 | DD405494.1 | AY659270.1 | CQ840846.1 | DL023425.1 | CS144309.1 |
| HI540736.1 | HH998824.1 | CS812642.1 | HA639660.1 | DD405462.1 | AY659238.1 | CQ840586.1 | DL023393.1 | CS141713.1 |
| HI593989.1 | HH998762.1 | DJ020647.1 | HA638534.1 | DD405430.1 | AY659206.1 | CQ831797.1 | DL020378.1 | CS141567.1 |
| HI593016.1 | HH997057.1 | DJ015678.1 | DM102621.1 | DD402024.1 | AY659174.1 | AX539578.1 | DL020346.1 | CS141522.1 |
| HI637015.1 | HH997008.1 | CS803369.1 | GM709159.1 | DD401992.1 | AY659142.1 | AX538729.1 | DL020314.1 | CS141490.1 |
| HI636962.1 | HH996954.1 | CS803336.1 | GM709001.1 | DD401943.1 | AY659110.1 | AX538331.1 | DL039218.1 | CS140821.1 |
| HI636925.1 | HH998711.1 | FB295833.1 | GM655030.1 | DD401899.1 | AY659078.1 | AX537264.1 | DL035402.1 | CS123423.1 |
| HI001444.1 | HH998672.1 | DJ047000.1 | GM654998.1 | DD401867.1 | AY659046.1 | AX529538.1 | DL035370.1 | CS122938.1 |
| HI001382.1 | HH998602.1 | DJ052314.1 | GM044795.1 | DD409185.1 | AY659014.1 | AX528956.1 | DL035338.1 | CS122407.1 |
| HI001348.1 | HH998549.1 | DJ052001.1 | GM755055.1 | DD401627.1 | AY658982.1 | AX528120.1 | DL035306.1 | CS250437.1 |
| HC500983.1 | HH996926.1 | DJ050216.1 | GM834847.1 | DD401595.1 | AY658950.1 | AX527660.1 | DJ387469.1 | DD181019.1 |
| HC500239.1 | HH996826.1 | DJ048839.1 | DL480711.1 | DD401563.1 | AY658918.1 | AX524914.1 | DJ381180.1 | DD161415.1 |
| HC499859.1 | HH996770.1 | CS793959.1 | DL467555.1 | DD401531.1 | AY658886.1 | AX523924.1 | DJ380814.1 | DD167017.1 |
| FW300536.1 | HH977649.1 | CS800108.1 | HW307887.1 | DD401499.1 | AY658854.1 | AX523715.1 | DJ388678.1 | DD172930.1 |
| FW298764.1 | HH977273.1 | CS720120.1 | HW307791.1 | DD401467.1 | AY658822.1 | AX521950.1 | CS724447.1 | DD166218.1 |
| HC488030.1 | HH980500.1 | CS799870.1 | HW307753.1 | CS479224.1 | AY658790.1 | A33441.1 | CS723808.1 | DD159733.1 |
| HC486501.1 | HH980432.1 | CS716780.1 | HW307721.1 | CS470241.1 | AY658758.1 | A33873.1 | CS722217.1 | DD165142.1 |
| HC486436.1 | FU257802.1 | CS798094.1 | HW307689.1 | CS468997.1 | AY658726.1 | AX179532.1 | DJ353078.1 | CS080789.1 |
| HC485946.1 | FU257705.1 | CS796352.1 | HW315870.1 | CS467563.1 | AY658694.1 | AX179502.1 | DJ357738.1 | HI472898.1 |
| HC475378.1 | FU263069.1 | CS715290.1 | HW315397.1 | CS466057.1 | AY658662.1 | AX179444.1 | DJ357706.1 | HI580544.1 |
| HC474951.1 | FU262849.1 | CS806051.1 | HW315156.1 | CS464783.1 | AY658630.1 | AX076858.1 | DJ357640.1 | HI210620.1 |
| HC491788.1 | FU259882.1 | CS809312.1 | HW315092.1 | DD392028.1 | AY658598.1 | AX521547.1 | DJ357451.1 | HI072382.1 |
| HC491756.1 | FU250457.1 | CS790633.1 | HW314762.1 | DD391914.1 | AY658566.1 | AX521515.1 | DJ357162.1 | HI004355.1 |
| HC491724.1 | FU265001.1 | CS792481.1 | HV758701.1 | DD382833.1 | AY658534.1 | AX513509.1 | DJ340479.1 | HI002267.1 |
| HC481583.1 | FU261564.1 | DJ042529.1 | HV745349.1 | DD400157.1 | AY658502.1 | AF430201.1 | DJ339845.1 | HI002230.1 |
| HC473412.1 | FU258394.1 | DJ044955.1 | HV749320.1 | CS463696.1 | AY658470.1 | AF430169.1 | DJ339781.1 | HI002180.1 |
| HC491011.1 | FU258330.1 | DJ044933.1 | HV750964.1 | CS462158.1 | AY658438.1 | AX511468.1 | DJ339653.1 | HI002133.1 |
| FV531711.1 | HC313124.1 | CS570733.1 | HV753741.1 | CS459029.1 | AY658406.1 | AX511246.1 | DJ339620.1 | HI002097.1 |
| FV531679.1 | DM476469.1 | CS546785.1 | HV753516.1 | DD361288.1 | AY658374.1 | AX505227.1 | DJ339588.1 | HI000436.1 |
| FV523752.1 | DM473013.1 | CS570227.1 | HV743330.1 | DD367616.1 | AY658342.1 | AX503694.1 | DJ339556.1 | HI000396.1 |
| FV522809.1 | HC299785.1 | CS544821.1 | HV743249.1 | DD367320.1 | AY658310.1 | AX497563.1 | DJ339168.1 | HI000338.1 |
| FV530191.1 | HC306168.1 | CS561183.1 | HV742536.1 | DD362915.1 | AY658278.1 | AX496672.1 | DJ327358.1 | HI203429.1 |
| FV528447.1 | HC306132.1 | CS560461.1 | HV226915.1 | DD362179.1 | AY658246.1 | BD140202.1 | DJ327039.1 | HI553282.1 |
| FV534536.1 | HC306052.1 | DD419954.1 | HV235769.1 | DD368154.1 | AY658214.1 | BD139565.1 | DJ327007.1 | HI553210.1 |
| FV534414.1 | HC305972.1 | DD420062.1 | HV305475.1 | DD361322.1 | AY658182.1 | BD138754.1 | DJ086514.1 | HI553178.1 |
| FV534093.1 | HC301632.1 | CS542560.1 | HV188410.1 | CS457838.1 | DM370526.1 | BD138718.1 | BD224590.1 | HI553022.1 |
| HC461933.1 | HC301309.1 | CS538093.1 | HV200482.1 | CS457449.1 | DM370494.1 | BD138412.1 | BD223825.1 | HI471783.1 |
| HC461379.1 | HC291414.1 | CS510550.1 | HV182445.1 | CS457128.1 | DM370462.1 | BD138377.1 | BD222644.1 | HI583962.1 |
| HC465808.1 | HC294098.1 | CS502907.1 | HV203118.1 | CS453655.1 | DM367519.1 | BD137470.1 | BD218048.1 | HI568953.1 |
| AF533145.1 | HC289415.1 | CS502777.1 | HV117707.1 | DD106659.1 | DM205453.1 | BD137168.1 | BD204959.1 | HI003900.1 |
| HC003092.1 | AX142681.1 | CS502743.1 | HV112886.1 | DD092953.1 | HC003084.1 | BD135073.1 | DD282678.1 | HI002056.1 |
| HC002696.1 | AX142617.1 | CS502711.1 | HV038641.1 | DD119306.1 | HC001398.1 | BD134015.1 | DD282386.1 | HI001968.1 |
| HB865569.1 | AX142553.1 | CS502547.1 | HV038583.1 | DD092150.1 | HB865044.1 | BD133203.1 | DD281306.1 | HI000299.1 |
| HB865047.1 | AX142487.1 | CS502405.1 | HV190785.1 | DD118644.1 | HB864960.1 | BD131917.1 | DD279235.1 | HI000255.1 |
| HB865015.1 | AX141973.1 | CS502483.1 | HV202356.1 | DD091021.1 | HB865004.1 | BD131358.1 | CS353467.1 | HI000217.1 |
| HB865007.1 | AX141909.1 | CS501246.1 | HV029941.1 | DD118103.1 | HB864948.1 | BD130847.1 | CS353175.1 | HI000157.1 |
| HB864951.1 | AX141843.1 | CS498528.1 | HV037780.1 | DD090844.1 | HB864916.1 | BD130810.1 | CS352516.1 | HI000123.1 |

| | | | | | | | | |
|---|---|---|---|---|---|---|---|---|
| HB864919.1 | AX141779.1 | CS498428.1 | HV035595.1 | DD090409.1 | HB864884.1 | BD130778.1 | CS352395.1 | HI204489.1 |
| HB864887.1 | AX141715.1 | DD418615.1 | FW505324.1 | DD116229.1 | HB864852.1 | BD130680.1 | CS283925.1 | HI203117.1 |
| HB864855.1 | AX139905.1 | DD418580.1 | HI931369.1 | DD103115.1 | HB864820.1 | BD130501.1 | CS279165.1 | HI470710.1 |
| HB864823.1 | AX137112.1 | DD412331.1 | HI930283.1 | DD057940.1 | HB864788.1 | BD106886.1 | CS275481.1 | HI470592.1 |
| HB864791.1 | AX134788.1 | DD412067.1 | HI924541.1 | DD057908.1 | HB864756.1 | BD105695.1 | CS272543.1 | HI551662.1 |
| HB864759.1 | AX133311.1 | DD411583.1 | HI658629.1 | DD057876.1 | HB859737.1 | BD096864.1 | CS274783.1 | HI559255.1 |
| HB859740.1 | AX127574.1 | DD411473.1 | FW420816.1 | DD057844.1 | HB866550.1 | BD095822.1 | CS265610.1 | HI568905.1 |
| HB866939.1 | AX119973.1 | DD414320.1 | FW503716.1 | DD053304.1 | HB866486.1 | BD090918.1 | CS263126.1 | HI568845.1 |
| HB866625.1 | AX114303.1 | DD410770.1 | FW420458.1 | DD052288.1 | HC000343.1 | BD087733.1 | CS252529.1 | HI564247.1 |
| HB866497.1 | AX113775.1 | DD417725.1 | FW420426.1 | DD052230.1 | HB999699.1 | BD085736.1 | CS249798.1 | HI564118.1 |
| HC000349.1 | AX113551.1 | DD410183.1 | FW420369.1 | DD059292.1 | HB976734.1 | BD081957.1 | GM639381.1 | HI563642.1 |
| HB999772.1 | AX108265.1 | CS482969.1 | FW420337.1 | DD058550.1 | HB976487.1 | BD080712.1 | GM639349.1 | HC461202.1 |
| HB999316.1 | AX107007.1 | CS482934.1 | FW420156.1 | DD041984.1 | DM197979.1 | BD080515.1 | GM639082.1 | AY305395.1 |
| HB976740.1 | AX103771.1 | DD240598.1 | FW496634.1 | DD048081.1 | DM201541.1 | BD080149.1 | DL176622.1 | HC456311.1 |
| DM195293.1 | AX100360.1 | DD240490.1 | HI654044.1 | DD042879.1 | DM193214.1 | BD077991.1 | DL176472.1 | HC453672.1 |
| HB817103.1 | AX097519.1 | DD240458.1 | HI653915.1 | DD032319.1 | GM659924.1 | BD077107.1 | DL176400.1 | HC460382.1 |
| HB840197.1 | AX097485.1 | DD240096.1 | HI653829.1 | DD027320.1 | GM659892.1 | BD075751.1 | DL176328.1 | AF073900.1 |
| HB848130.1 | AX093099.1 | DD236634.1 | HI653553.1 | DD026765.1 | GM659860.1 | BD075061.1 | DL176011.1 | HC442312.1 |
| HB847860.1 | AX088792.1 | DD234706.1 | HI653391.1 | DD023889.1 | GM659828.1 | BD074956.1 | DL174446.1 | HC452135.1 |
| HB839678.1 | AX088748.1 | DD246844.1 | HH980144.1 | DD023827.1 | GM659796.1 | BD070712.1 | DL176739.1 | HC451941.1 |
| HB847607.1 | AX088692.1 | E41524.1 | HH980076.1 | DD029701.1 | GM659764.1 | BD062683.1 | DL163230.1 | HC449851.1 |
| HB847299.1 | AX085492.1 | E44269.1 | HH979854.1 | DD010448.1 | GM659732.1 | A41481.1 | FB344353.1 | FU756128.1 |
| HB827787.1 | AX082964.1 | E35602.1 | HH996691.1 | DD013402.1 | GM712210.1 | A35716.1 | DL097997.1 | FU774503.1 |
| HB846836.1 | AX077283.1 | E33920.1 | HH996628.1 | BD495444.1 | GM652343.1 | A29447.1 | DL097965.1 | FU774060.1 |
| HB838534.1 | AX076478.1 | E41219.1 | HH996568.1 | CS118231.1 | GM652311.1 | A32640.1 | DL112527.1 | FU971688.1 |
| HB846373.1 | AX067255.1 | E37590.1 | HH998516.1 | CS118198.1 | GM652279.1 | A32254.1 | DL101214.1 | FU773358.1 |
| HB846074.1 | HW335698.1 | E06922.1 | HH998385.1 | CS118164.1 | GM645384.1 | A30344.1 | DL094230.1 | FU759993.1 |
| HB809887.1 | HW328209.1 | E06558.1 | HH998352.1 | DD405493.1 | GM645352.1 | A23370.1 | DL094198.1 | FU759936.1 |
| HB826601.1 | HW328090.1 | E05671.1 | HH999923.1 | DD405461.1 | GM645320.1 | A06239.1 | DL094166.1 | FU764212.1 |
| HB837576.1 | HW318494.1 | E03527.1 | HH979778.1 | DD405429.1 | GM645288.1 | A30428.1 | DL110156.1 | FU757628.1 |
| HB845148.1 | HW318286.1 | E03213.1 | HH979691.1 | DD402023.1 | GM645256.1 | A21080.1 | DL110124.1 | HC441400.1 |
| HB844416.1 | HW335185.1 | E02874.1 | HH999890.1 | DD401991.1 | GM645224.1 | A19558.1 | DL110092.1 | HC436124.1 |
| CS070405.1 | HW335142.1 | E02641.1 | HH999849.1 | DD401898.1 | GM645191.1 | A17078.1 | DL105255.1 | HC358402.1 |
| A04347.1 | HW314230.1 | E02484.1 | HH999745.1 | DD401866.1 | GM645159.1 | A14930.1 | DL088951.1 | HC358214.1 |
| CS059931.1 | HW314060.1 | E02337.1 | HH998316.1 | DD401834.1 | GM645127.1 | A12324.1 | DL088919.1 | HC358083.1 |
| CS059001.1 | HW313983.1 | E01773.1 | HH998265.1 | DD401802.1 | GM645095.1 | A10838.1 | DL088621.1 | HC321442.1 |
| AY967379.1 | HW312125.1 | E01245.1 | HH998228.1 | DD401626.1 | GM645063.1 | A10498.1 | DL088589.1 | HC324862.1 |
| AY967347.1 | HW312055.1 | E01034.1 | HH996540.1 | DD401594.1 | GM645031.1 | A07609.1 | DL092346.1 | HC324492.1 |
| AY967315.1 | HW311835.1 | E00004.1 | HH993664.1 | DD401562.1 | GM638072.1 | A04571.1 | DL092314.1 | HC319621.1 |
| AY967283.1 | HW311249.1 | DD146375.1 | HH986622.1 | DD401530.1 | GM633334.1 | A30562.1 | DL092282.1 | HC318841.1 |
| AY967251.1 | HW311133.1 | DD088016.1 | HH979509.1 | DD401498.1 | GM633302.1 | A30164.1 | DL092250.1 | HC318700.1 |
| AY967219.1 | HW308081.1 | DD129366.1 | HH979619.1 | DD401466.1 | GM633247.1 | A28155.1 | DL092218.1 | HC316834.1 |
| AY967187.1 | HW307869.1 | DD100022.1 | HH979577.1 | CS470224.1 | GM633215.1 | A26680.1 | DL092186.1 | HA641087.1 |
| AY967155.1 | HW307837.1 | DD143659.1 | HH931980.1 | CS287622.1 | GM633183.1 | A22627.1 | DL121939.1 | HA638543.1 |
| AY967123.1 | HW307805.1 | DD069880.1 | FW394382.1 | DD240751.1 | GM629159.1 | A28163.1 | DL042637.1 | HA637702.1 |
| AY967091.1 | HW316585.1 | DD112551.1 | FW394288.1 | DD240654.1 | GM629127.1 | A25801.1 | DL042605.1 | DM102624.1 |
| AY967059.1 | HW307735.1 | DD143239.1 | FW394231.1 | DD240486.1 | GM619871.1 | A25155.1 | DL042573.1 | DM094737.1 |

| | | | | | | | | |
|---|---|---|---|---|---|---|---|---|
| AY967027.1 | HW307703.1 | DD098830.1 | FW394195.1 | DD240454.1 | GM884059.1 | A23308.1 | DL029763.1 | GN368297.1 |
| AY966995.1 | HW315958.1 | DD084333.1 | FW418988.1 | DD237945.1 | GM662970.1 | A24689.1 | DJ493871.1 | GN360085.1 |
| AY966963.1 | HW315602.1 | DD098181.1 | FW396285.1 | DD235104.1 | GM648353.1 | HW099664.1 | DJ492740.1 | GN360044.1 |
| AY966931.1 | HW315563.1 | DD141438.1 | HH936038.1 | DD234698.1 | GM648321.1 | HW099557.1 | DJ491543.1 | GN360012.1 |
| CS057768.1 | HW315074.1 | DD141284.1 | HH834394.1 | DD247146.1 | GM648289.1 | HW089226.1 | DJ446700.1 | GN359980.1 |
| CS054744.1 | HW314783.1 | DD152082.1 | HH833980.1 | E49677.1 | GM648257.1 | HW083516.1 | BD269160.1 | GN359807.1 |
| CS052379.1 | HW314728.1 | DD158434.1 | HH924312.1 | E41520.1 | GM648225.1 | HW088843.1 | BD269128.1 | GN359775.1 |
| CS052311.1 | HW314682.1 | DL125173.1 | FU262772.1 | E43961.1 | GM641390.1 | HW083270.1 | BD268884.1 | GN359743.1 |
| CS051911.1 | HW121469.1 | DL116593.1 | HC313232.1 | E35598.1 | GM641358.1 | HW083238.1 | BD267982.1 | GN359711.1 |
| CS050942.1 | HW112788.1 | DL116561.1 | HC306229.1 | E36140.1 | GM641326.1 | HW083206.1 | BD265647.1 | GN359659.1 |
| CS048772.1 | HW112682.1 | DL111840.1 | HC306189.1 | E05667.1 | GM641294.1 | HW088684.1 | BD265598.1 | GN359595.1 |
| CS038853.1 | HW112613.1 | DL111808.1 | HC302637.1 | E05285.1 | GM641262.1 | HW081798.1 | BD263803.1 | GN359499.1 |
| CS027001.1 | HW069954.1 | DL111776.1 | HC302247.1 | E04465.1 | GM641230.1 | HW099469.1 | BD263438.1 | GN359399.1 |
| BD192897.1 | HW069922.1 | DL128891.1 | HC305922.1 | E03478.1 | GM629039.1 | HW103540.1 | BD263055.1 | GN359335.1 |
| BD188834.1 | HW069826.1 | DL128857.1 | HC305882.1 | E03207.1 | GM629007.1 | HW084530.1 | BD251316.1 | GN365396.1 |
| BD187687.1 | HW069790.1 | DL128792.1 | HC305842.1 | E03008.1 | GM648192.1 | HW103428.1 | BD250959.1 | GM712158.1 |
| AX787415.1 | HW069758.1 | DL128759.1 | HC305802.1 | E02867.1 | GM648160.1 | HW102877.1 | BD249653.1 | GM827694.1 |
| AX787365.1 | HW084946.1 | DL116391.1 | HC305762.1 | E02776.1 | GM716036.1 | HW084158.1 | BD248733.1 | FB509203.1 |
| AX773474.1 | HW084907.1 | DL116359.1 | HC305722.1 | E02470.1 | GM640906.1 | HW058485.1 | BD248118.1 | GM772914.1 |
| AX769752.1 | HW097740.1 | DL111638.1 | BD271156.1 | E02046.1 | GM640874.1 | HW061940.1 | BD247030.1 | GM709004.1 |
| AX765916.1 | HW067064.1 | DL111606.1 | DD231092.1 | E01551.1 | GM640842.1 | HW061861.1 | BD246955.1 | GM708973.1 |
| AX755004.1 | HW065055.1 | DL111574.1 | DD230118.1 | E01350.1 | GM628488.1 | HW061829.1 | BD245074.1 | GM655033.1 |
| AX752634.1 | HW057913.1 | DL111542.1 | DD227402.1 | E01215.1 | DL193756.1 | HW061815.1 | BD243425.1 | GM655001.1 |
| AX751527.1 | HW043964.1 | DL111510.1 | DD224954.1 | E00867.1 | DL183797.1 | HW061749.1 | BD243011.1 | GM755620.1 |
| BD185263.1 | HW047621.1 | DL111478.1 | DD224448.1 | E00604.1 | FB513363.1 | HW061716.1 | BD242443.1 | CS106061.1 |
| BD180859.1 | HV969578.1 | DL101696.1 | DD224116.1 | E00126.1 | FB571353.1 | HV701189.1 | BD238536.1 | CS179696.1 |
| AX743926.1 | HV745657.1 | DL101664.1 | DD231426.1 | DD088003.1 | FB571297.1 | HV701157.1 | BD238169.1 | CS179211.1 |
| AX734393.1 | HV748685.1 | DL101600.1 | DD231394.1 | DD087815.1 | FB571237.1 | HV701125.1 | BD235726.1 | CS327370.1 |
| AX722015.1 | HV748436.1 | DL097122.1 | A06167.1 | DD101258.1 | FB570898.1 | HV708781.1 | BD235441.1 | CS326336.1 |
| BD177612.1 | HV751132.1 | DL097090.1 | DD214004.1 | DD084815.1 | CS696157.1 | HV708192.1 | BD234797.1 | CS323582.1 |
| AX710269.1 | HV750901.1 | DL097026.1 | DD218567.1 | DD069876.1 | CS696125.1 | HV701106.1 | BD234400.1 | CS323282.1 |
| AX709083.1 | HV750779.1 | DL095134.1 | DD213571.1 | DD112547.1 | CS696029.1 | HV701074.1 | BD233778.1 | DD261103.1 |
| AX704557.1 | HV753563.1 | DL095102.1 | DD213539.1 | DD070092.1 | CS695964.1 | HV695536.1 | BD230044.1 | DD259335.1 |
| AX699457.1 | HV753482.1 | DL108338.1 | CS038908.1 | DD084329.1 | CS695932.1 | HV695392.1 | BD227107.1 | CS297036.1 |
| AX699425.1 | HV743344.1 | DL108306.1 | CS027637.1 | DD152078.1 | CS695900.1 | HV695103.1 | BD226835.1 | DD253215.1 |
| AX685569.1 | HV743304.1 | DL103636.1 | CS024304.1 | CS124717.1 | CS695868.1 | HV695071.1 | BD224579.1 | DD251646.1 |
| AX684487.1 | HV743267.1 | DL099030.1 | CS018515.1 | CS124639.1 | CS695836.1 | HV694927.1 | BD222637.1 | DD248660.1 |
| AX675246.1 | HV743229.1 | DL098998.1 | CS018390.1 | CS124198.1 | CS695804.1 | HV694852.1 | BD211170.1 | CS288141.1 |
| AX670747.1 | HV704415.1 | DL098966.1 | CS007991.1 | CS120368.1 | CS695772.1 | HV694785.1 | DD283704.1 | CS287613.1 |
| AX664339.1 | HV704111.1 | DL098934.1 | CQ986649.1 | CS119542.1 | CS695740.1 | HV700706.1 | DD282357.1 | DD240735.1 |
| AX662217.1 | HV744589.1 | DL092828.1 | CQ986583.1 | CS119508.1 | CS695516.1 | HV694718.1 | CS088906.1 | DD240578.1 |
| AX659101.1 | HV702884.1 | DL092796.1 | CQ986517.1 | CS119441.1 | HI415742.1 | HV700635.1 | CS082047.1 | DD247530.1 |
| AX658681.1 | HV702350.1 | DL092764.1 | CQ981105.1 | CS119408.1 | HI414097.1 | HV700228.1 | CS075315.1 | BD276212.1 |
| AX657139.1 | HV701854.1 | DL089021.1 | CQ975015.1 | CS119343.1 | HI414029.1 | HV698622.1 | CS070723.1 | BD274845.1 |
| AX078904.1 | HV701246.1 | DL088989.1 | AX799559.1 | CS119309.1 | HI413992.1 | HV698557.1 | AY967401.1 | BD274299.1 |
| AX076599.1 | HV701214.1 | DL122316.1 | AX798982.1 | CS119277.1 | HI413621.1 | HV690930.1 | AY967369.1 | BD274095.1 |
| AX076392.1 | HC728701.1 | DL117966.1 | AX798493.1 | CS119245.1 | HI412447.1 | HV697912.1 | AY967337.1 | BD271969.1 |

| | | | | | | | | |
|---|---|---|---|---|---|---|---|---|
| AX074107.1 | HC727680.1 | DL117934.1 | AX796717.1 | CS119213.1 | HH760614.1 | HV689963.1 | AY967305.1 | DD231176.1 |
| AX073720.1 | HC688440.1 | DL117902.1 | AX472468.1 | CS119181.1 | HI503569.1 | HV689029.1 | AY967273.1 | DD227722.1 |
| AX063717.1 | HC686911.1 | DL117870.1 | BD190818.1 | CS119149.1 | HI516104.1 | HV695814.1 | AY967241.1 | DD227406.1 |
| AX061289.1 | HC490836.1 | DL117838.1 | AX787399.1 | CS119082.1 | HI516072.1 | HV688832.1 | AY967209.1 | DD224452.1 |
| AX060538.1 | HC490804.1 | DL108156.1 | AX787204.1 | CS119049.1 | HI284349.1 | HV600543.1 | AY967177.1 | DD224157.1 |
| AX058845.1 | HC490772.1 | DL108124.1 | AX773265.1 | CS119017.1 | HI284291.1 | HV585540.1 | AY967145.1 | DD223495.1 |
| AX057962.1 | HC490708.1 | DL108092.1 | AX766138.1 | CS118984.1 | HI574512.1 | HV579861.1 | AY967113.1 | DD231542.1 |
| AX056088.1 | HC490676.1 | DL103486.1 | AX752750.1 | CS118919.1 | HI574480.1 | HV585132.1 | AY967081.1 | DD231398.1 |
| AX052912.1 | HC490547.1 | DL103454.1 | AX752504.1 | CS118853.1 | HI574448.1 | HV585100.1 | AY967049.1 | CQ757640.1 |
| AX046143.1 | HC490512.1 | DL103422.1 | BD182886.1 | CS118820.1 | HI574416.1 | HV584976.1 | AY967017.1 | DD214008.1 |
| AX043753.1 | HC490480.1 | DL103390.1 | BD181489.1 | CS118787.1 | HI574384.1 | HV579204.1 | AY966985.1 | DD213991.1 |
| AX040773.1 | HC686463.1 | DL098784.1 | BD180849.1 | CS118722.1 | HI283457.1 | HV579086.1 | AY966953.1 | DD213575.1 |
| AX040184.1 | HC490409.1 | DL098752.1 | BD180738.1 | CS118690.1 | HI574361.1 | HV588569.1 | AY966921.1 | DD213543.1 |
| AX039522.1 | HC490248.1 | DL092742.1 | AY181087.1 | CS118658.1 | HB835444.1 | HV578981.1 | CS055262.1 | DD218339.1 |
| AX037322.1 | HC490216.1 | DL092710.1 | BD177187.1 | CS118626.1 | HB807550.1 | HV592533.1 | CS052523.1 | DD215945.1 |
| AX036754.1 | DM164009.1 | DL092678.1 | AX717715.1 | CS118593.1 | HB842896.1 | HV592461.1 | CS052403.1 | DD222028.1 |
| AX036603.1 | DM163975.1 | DL092646.1 | AX716791.1 | CS118558.1 | HB842337.1 | HV221454.1 | GM831913.1 | DD212238.1 |
| AX036022.1 | DM163307.1 | DL092614.1 | AX708727.1 | CS118493.1 | HB842313.1 | HV217829.1 | GM709026.1 | DD212038.1 |
| AX035969.1 | HB476995.1 | DL092582.1 | AX283494.1 | CS118460.1 | HB841895.1 | HV235902.1 | GM708994.1 | DD211831.1 |
| AX035265.1 | HB476399.1 | DL088967.1 | AX280099.1 | CS118428.1 | HB841705.1 | HV316810.1 | GM652300.1 | CS029875.1 |
| AX023646.1 | HB485574.1 | DL088935.1 | AX278251.1 | CS118395.1 | HB841243.1 | HV310801.1 | GM659292.1 | CS018728.1 |
| AX023613.1 | HB475864.1 | DL088903.1 | AX255411.1 | CS118363.1 | HB840632.1 | HV309546.1 | GM658660.1 | CS017701.1 |
| AX023580.1 | HB475517.1 | DL103310.1 | AX250488.1 | CS118328.1 | HB823257.1 | HV304098.1 | GM650546.1 | CS016571.1 |
| AX019441.1 | HB474829.1 | DL103278.1 | AX244968.1 | CS118294.1 | HB647043.1 | HV221078.1 | GM650482.1 | CS016533.1 |
| AX015789.1 | HB463659.1 | DL103246.1 | AX242313.1 | CS118262.1 | HB645702.1 | HV203316.1 | GM631860.1 | CS008048.1 |
| AX002866.1 | HB463594.1 | DL103214.1 | AX242281.1 | CS118230.1 | HB645448.1 | HV197644.1 | GM631828.1 | CQ990423.1 |
| AX011548.1 | HB469096.1 | DL107966.1 | AX242249.1 | CS118131.1 | HB855975.1 | HV191355.1 | GM631796.1 | CQ986587.1 |
| AX011472.1 | HB468035.1 | DL107934.1 | AX242185.1 | CS118099.1 | DM170843.1 | HV182466.1 | GM643334.1 | CQ986555.1 |
| AX011408.1 | DM152692.1 | DL107902.1 | AX242153.1 | CS118066.1 | DM170811.1 | HV182434.1 | GM646404.1 | CQ981109.1 |
| AX011375.1 | DM152550.1 | DL092444.1 | AX242089.1 | CS118032.1 | DM177495.1 | HV182402.1 | GM653232.1 | CQ975032.1 |
| AX011343.1 | GM659672.1 | DL092412.1 | AX241993.1 | CS117996.1 | HB489353.1 | HV182370.1 | GM639110.1 | CQ973471.1 |
| AX010937.1 | GM659640.1 | DL047792.1 | AX241961.1 | CS117964.1 | DM160454.1 | HV246859.1 | GM639078.1 | CQ979563.1 |
| AX010514.1 | GM659608.1 | DL047760.1 | AX241929.1 | CS115023.1 | DM163997.1 | HV121628.1 | DL136648.1 | CQ972386.1 |
| AX009277.1 | GM652351.1 | DL047728.1 | AX241865.1 | HW260829.1 | DM163964.1 | HV117906.1 | DL102511.1 | CQ972354.1 |
| AX008211.1 | GM652319.1 | DL047696.1 | AX241833.1 | HW260797.1 | DM163583.1 | HV117801.1 | DL095423.1 | CQ970339.1 |
| AX006909.1 | GM652287.1 | DL043682.1 | AX241801.1 | HW260765.1 | DM163295.1 | HV117764.1 | DL095391.1 | AX797692.1 |
| AX003038.1 | GM652255.1 | DL039769.1 | AX241769.1 | HW260733.1 | HB488207.1 | HV117504.1 | DL095359.1 | AX472474.1 |
| AX002818.1 | GM645392.1 | DL039737.1 | AX241705.1 | HW260669.1 | GM652678.1 | HV038766.1 | DL115120.1 | AX787353.1 |
| HV538750.1 | GM645360.1 | DL039705.1 | AX241673.1 | HW260637.1 | GM645488.1 | HV116993.1 | DL114716.1 | AX781461.1 |
| HI002652.1 | GM645328.1 | DL035921.1 | AX241641.1 | HW260605.1 | GM645456.1 | HV112426.1 | DL114684.1 | AX773269.1 |
| HI002618.1 | GM645264.1 | DL031917.1 | AX241609.1 | HW260573.1 | GM645424.1 | HV038604.1 | DL114652.1 | AX772012.1 |
| HI002579.1 | GM645232.1 | DL031885.1 | AX241577.1 | HW260541.1 | GM638531.1 | HV038529.1 | DL023544.1 | AX770674.1 |
| HI004531.1 | GM659378.1 | DL031853.1 | AX241545.1 | HW260509.1 | GM638499.1 | HV182332.1 | CS724470.1 | AX766565.1 |
| HI004467.1 | GM659346.1 | DL031821.1 | AX241513.1 | HW260477.1 | GM638467.1 | HV182300.1 | DJ347805.1 | AX752754.1 |
| HI004403.1 | GM652154.1 | DL031789.1 | AX241481.1 | HW260445.1 | GM633736.1 | HV202767.1 | DJ340875.1 | AX752607.1 |
| HI002427.1 | GM645199.1 | DL031757.1 | AX241417.1 | HW260413.1 | GM633704.1 | HV187863.1 | DJ340976.1 | AX746474.1 |
| HI002310.1 | GM645167.1 | DL027852.1 | AF411476.1 | HW260381.1 | GM625837.1 | HV199776.1 | DJ344584.1 | BD184759.1 |

| | | | | | | | | |
|---|---|---|---|---|---|---|---|---|
| HI000512.1 | GM645135.1 | DL027820.1 | AX212278.1 | HW260349.1 | GM625805.1 | HV187606.1 | DJ361221.1 | BD181493.1 |
| HI006497.1 | GM645103.1 | DL027788.1 | AX207117.1 | HW260317.1 | GM624717.1 | HV234370.1 | DJ361188.1 | BD181217.1 |
| HI180157.1 | GM645071.1 | DL027756.1 | AX205052.1 | HW260285.1 | GM633667.1 | HV302189.1 | DJ339868.1 | BD180853.1 |
| HI178183.1 | GM638144.1 | DL023944.1 | AX201769.1 | HW260253.1 | GM659889.1 | HV312989.1 | DJ339836.1 | AX743756.1 |
| HI202829.1 | GM638112.1 | DL023912.1 | AX195438.1 | HW260221.1 | GM659857.1 | HV038138.1 | DJ339804.1 | BD177545.1 |
| HI214557.1 | GM638080.1 | DL023880.1 | AX193982.1 | HW260189.1 | GM659825.1 | HV037713.1 | DJ339740.1 | BD177331.1 |
| HI214525.1 | GM638038.1 | DL023848.1 | AX180553.1 | HW260157.1 | GM659793.1 | HV037260.1 | DJ082708.1 | AX708748.1 |
| HI214459.1 | GM633470.1 | DL023816.1 | AX179678.1 | HW260125.1 | GM652340.1 | HV035181.1 | DJ082654.1 | AX705329.1 |
| HI214427.1 | GM633406.1 | DL023784.1 | AX172980.1 | HW260093.1 | GM652308.1 | GN030832.1 | DJ080510.1 | AX703579.1 |
| HI213916.1 | GM633374.1 | DL020673.1 | AX167806.1 | HW260061.1 | GM652276.1 | GN030800.1 | DJ071361.1 | AX699451.1 |
| HI185524.1 | GM633342.1 | DL020641.1 | AF385771.1 | HW260029.1 | GM652212.1 | GN030768.1 | DJ066759.1 | AX699419.1 |
| HI213367.1 | GM633310.1 | DL020609.1 | AX153817.1 | HW259965.1 | GM645381.1 | DM002017.1 | DJ066363.1 | AX687260.1 |
| HI212996.1 | GM633278.1 | DL016174.1 | AF384424.1 | HW259933.1 | GM645349.1 | DM001908.1 | DJ066331.1 | AX145174.1 |
| HI212750.1 | GM633223.1 | DL114340.1 | AX146300.1 | HW259731.1 | GM645317.1 | DM001840.1 | DJ066297.1 | AX145142.1 |
| HI546344.1 | GM633191.1 | DL104581.1 | AX145715.1 | HW258943.1 | GM645285.1 | DM007529.1 | DJ066263.1 | AX145110.1 |
| HI546312.1 | GM633159.1 | DL104549.1 | AX145683.1 | HW258894.1 | GM645253.1 | DM001479.1 | DD218015.1 | AX145078.1 |
| HI546142.1 | GM633127.1 | DL104517.1 | AX145651.1 | HW258832.1 | GM645221.1 | DM006723.1 | CS254929.1 | AX145046.1 |
| HI210990.1 | GM633095.1 | DL093933.1 | AX145619.1 | HW257320.1 | GM648189.1 | DM004987.1 | DD211572.1 | AX145014.1 |
| HI210958.1 | GM625388.1 | DL093901.1 | AX145587.1 | HW257288.1 | GM628698.1 | DM004973.1 | DD210738.1 | AX144982.1 |
| HI573167.1 | GM625356.1 | DL093869.1 | HW237521.1 | HW257256.1 | GM716008.1 | DM003552.1 | DD206902.1 | AX144950.1 |
| HI472899.1 | GM659132.1 | DL090062.1 | HW237035.1 | HW257064.1 | GM640903.1 | DM003506.1 | DD206870.1 | AX144917.1 |
| HI580545.1 | GM651684.1 | DL090030.1 | HW241804.1 | HW257032.1 | GM640871.1 | DL489047.1 | DD206838.1 | AX144885.1 |
| HI577377.1 | GM634658.1 | DL033743.1 | HW241617.1 | HV514893.1 | GM640839.1 | DM002635.1 | DD206808.1 | AX144853.1 |
| HI072383.1 | GM634626.1 | DL033713.1 | HW243069.1 | HV514654.1 | GM628485.1 | DM010298.1 | DD206776.1 | AX144821.1 |
| HI004357.1 | GM634594.1 | DL042145.1 | HW241508.1 | HV536506.1 | GM047095.1 | GM998434.1 | DD196866.1 | AX144789.1 |
| HI004229.1 | GM634530.1 | DL042113.1 | AX241831.1 | HV531981.1 | GM654816.1 | GM996595.1 | CS236430.1 | AX144757.1 |
| HI002268.1 | GM634498.1 | DL042081.1 | AX241799.1 | HV530222.1 | GM654784.1 | GM996249.1 | DD181001.1 | AX144725.1 |
| HI002231.1 | GM629693.1 | DL033702.1 | AX241767.1 | HV515733.1 | GM647695.1 | GM980121.1 | DD165456.1 | AX144693.1 |
| HI002181.1 | GM664881.1 | DL033670.1 | AX241735.1 | HV515579.1 | GM627957.1 | GM994466.1 | DD159275.1 | AX144661.1 |
| HI002134.1 | GM664725.1 | DL029534.1 | AX241703.1 | HV515547.1 | GM627925.1 | GM994345.1 | DD182194.1 | AX144629.1 |
| HI002098.1 | GM655866.1 | DL029502.1 | AX241639.1 | HV515515.1 | GM715070.1 | GM992645.1 | CS204335.1 | DL109146.1 |
| HI000437.1 | GM648773.1 | DL029470.1 | AX241607.1 | HV515483.1 | FB660222.1 | GM992484.1 | CS001503.1 | DL109114.1 |
| HI000397.1 | GM648745.1 | DL046104.1 | AX241575.1 | HV515451.1 | FB659907.1 | GM992342.1 | CS157810.1 | DL109082.1 |
| HI000342.1 | GM648713.1 | DL041958.1 | AX241543.1 | HV515419.1 | FB580677.1 | GM992173.1 | CS157778.1 | DL123153.1 |
| HI203432.1 | GM648681.1 | DL041926.1 | AX241511.1 | HV515355.1 | FB654403.1 | GM992047.1 | CS157939.1 | DL114119.1 |
| HI553211.1 | GM648617.1 | DL041894.1 | AX241447.1 | HV515323.1 | FB654860.1 | GM991915.1 | CS157843.1 | DL114087.1 |
| HI553179.1 | GM634465.1 | DL069450.1 | AX241415.1 | HV515291.1 | DL199808.1 | GM976286.1 | CS144325.1 | DL114055.1 |
| HI553026.1 | GM634433.1 | DL049711.1 | AY044147.1 | HV515259.1 | DL199712.1 | GM990789.1 | CQ859626.1 | DL126227.1 |
| HI583963.1 | GM634401.1 | DL049679.1 | AX229850.1 | HV515227.1 | DL202053.1 | GM989806.1 | CQ859594.1 | DL126195.1 |
| HI568955.1 | GM634369.1 | DL045671.1 | AX214419.1 | HV511157.1 | DL196185.1 | GM989064.1 | CQ858100.1 | DL126163.1 |
| FU757822.1 | GM629478.1 | DL041696.1 | AX207311.1 | HV510350.1 | DL193817.1 | GM644456.1 | CQ857608.1 | DL122947.1 |
| FU757629.1 | GM629436.1 | DL037809.1 | AX207115.1 | HV510317.1 | DL183794.1 | GM644424.1 | CQ854065.1 | DL122915.1 |
| HC441402.1 | GM629404.1 | DL035508.1 | AX202638.1 | HV513324.1 | FB513360.1 | GM637426.1 | CQ840609.1 | DL021062.1 |
| HC358403.1 | GM629372.1 | DL031504.1 | AX202440.1 | HV513292.1 | FB571350.1 | GM637394.1 | CQ824250.1 | DL016632.1 |
| HC358215.1 | GM629308.1 | DL031440.1 | AX193978.1 | HV508702.1 | FB570824.1 | GM637362.1 | CQ821306.1 | DL011892.1 |
| HC358084.1 | GM664440.1 | DL031408.1 | AX181920.1 | HV508587.1 | CS696186.1 | GM637330.1 | CQ820509.1 | DL023687.1 |
| HC321443.1 | GM648571.1 | DL031376.1 | AX180413.1 | HV508555.1 | CS696058.1 | GM637298.1 | CQ816991.1 | DL023655.1 |

| | | | | | | | | |
|---|---|---|---|---|---|---|---|---|
| HC356961.1 | GM648539.1 | DL027471.1 | AX179667.1 | HV508523.1 | HV512159.1 | GM637266.1 | CQ816949.1 | DL023623.1 |
| HC324493.1 | GM648507.1 | DL027439.1 | AX174837.1 | HV508495.1 | HV512127.1 | GM623729.1 | CQ814075.1 | DL015917.1 |
| HC314735.1 | GM641608.1 | DL027407.1 | AX172954.1 | HV508463.1 | HV504726.1 | GM623697.1 | CQ814042.1 | DL015885.1 |
| HC319622.1 | GM629263.1 | DL027375.1 | AX167798.1 | HV512573.1 | HV504694.1 | GM623665.1 | CQ814010.1 | DL015853.1 |
| HC318842.1 | GM629231.1 | DL023563.1 | AX151026.1 | HV512297.1 | HV504662.1 | GM623633.1 | CQ813978.1 | DL047627.1 |
| HC318703.1 | GM629199.1 | DL023531.1 | AX153811.1 | HV512265.1 | HV504630.1 | GM623601.1 | CQ813946.1 | DL047595.1 |
| HC310088.1 | GM629167.1 | DL023499.1 | AX148296.1 | HV512233.1 | HV504598.1 | GM623569.1 | CQ813914.1 | DL047531.1 |
| HC310056.1 | GM629135.1 | DL023467.1 | AX144928.1 | HV512169.1 | HV504981.1 | GM658718.1 | CQ813882.1 | DL047366.1 |
| HC309992.1 | GM629103.1 | DL023435.1 | HW237921.1 | HV512137.1 | HV455615.1 | GM658654.1 | CQ813850.1 | DL039343.1 |
| HC309960.1 | GM634065.1 | DL023403.1 | HW237706.1 | JA660266.1 | HV503835.1 | GM658622.1 | CQ813818.1 | DJ043831.1 |
| HC307840.1 | GM634033.1 | DL020388.1 | HW237456.1 | HV492197.1 | HV500900.1 | GM658558.1 | CQ812173.1 | CS721670.1 |
| HC307789.1 | GM634001.1 | DL020356.1 | HW242300.1 | HV504945.1 | HV502804.1 | GM651366.1 | CQ807301.1 | CS693520.1 |
| FU260909.1 | GM633969.1 | DL020324.1 | HW165040.1 | HV504913.1 | HV502772.1 | GM651334.1 | CQ802184.1 | CS688538.1 |
| FU259852.1 | GM633937.1 | DL015793.1 | HW241386.1 | HV504881.1 | HV502740.1 | GM651302.1 | CQ800898.1 | DJ008422.1 |
| FU262613.1 | GM633905.1 | DL015761.1 | HW161287.1 | HV504849.1 | HV502708.1 | GM651270.1 | CQ800639.1 | DJ008390.1 |
| FU265310.1 | GM633873.1 | DL015729.1 | HW155699.1 | HV504768.1 | HV502676.1 | GM651238.1 | CQ797808.1 | DJ008358.1 |
| FU265324.1 | GM716542.1 | DL047251.1 | HW155497.1 | HV504736.1 | HV502644.1 | GM651206.1 | CQ795504.1 | DJ008018.1 |
| DM188066.1 | GM655514.1 | DL047219.1 | HW155431.1 | HV504704.1 | HV502548.1 | GM644407.1 | CQ795467.1 | DJ004221.1 |
| DM187870.1 | GM655482.1 | DL047187.1 | HW155297.1 | HV504672.1 | HV502516.1 | GM644375.1 | CQ795428.1 | DJ003331.1 |
| DM187702.1 | GM655450.1 | DL047155.1 | HW158406.1 | HV504640.1 | HV502484.1 | GM644343.1 | CQ788566.1 | DD491216.1 |
| HB489481.1 | GM648329.1 | DL047091.1 | HW154658.1 | HV492396.1 | HV502452.1 | GM644279.1 | CQ787681.1 | CS671011.1 |
| HB489175.1 | GM648297.1 | DL047059.1 | HW154491.1 | HI516588.1 | HI002731.1 | GM644247.1 | A13392.1 | CS669863.1 |
| DM164000.1 | FB667101.1 | DL043077.1 | HW101669.1 | HI516111.1 | HI002646.1 | GM644215.1 | DM077896.1 | CS673612.1 |
| DM163967.1 | FB666790.1 | DL039100.1 | HW096652.1 | HI516079.1 | HI002612.1 | GM637217.1 | DM070631.1 | DD463392.1 |
| DM163589.1 | CS721895.1 | DL035476.1 | HW072887.1 | HI284298.1 | HI002573.1 | GM637185.1 | DM069110.1 | CS647384.1 |
| DM163253.1 | FB676538.1 | DL035444.1 | HW105058.1 | HI571673.1 | HI004455.1 | GM637153.1 | DM068818.1 | CS646216.1 |
| DM162307.1 | FB705903.1 | DL035412.1 | HW096197.1 | HI574487.1 | HI002417.1 | GM637121.1 | GN087807.1 | CS646178.1 |
| HB485503.1 | FB675471.1 | DL035380.1 | HW071105.1 | HI574455.1 | HI002303.1 | GM637089.1 | GN087445.1 | DD459654.1 |
| HB464061.1 | FB701521.1 | DL035348.1 | HW070886.1 | HI574423.1 | HI000506.1 | GM637057.1 | GN087206.1 | DD458769.1 |
| DM114273.1 | CS809040.1 | DL035316.1 | HW103940.1 | HI574391.1 | HI006491.1 | GM624904.1 | GN094113.1 | DD453517.1 |
| DM120692.1 | FB701041.1 | DL031312.1 | HW099516.1 | HI574368.1 | HI202823.1 | GM624872.1 | GN089870.1 | DD456640.1 |
| DM117990.1 | FB660049.1 | DL031184.1 | HW068955.1 | HI581411.1 | HI214689.1 | GM624828.1 | GN089806.1 | DD451638.1 |
| DM115565.1 | FB665226.1 | DL031152.1 | HW102124.1 | HI573711.1 | HI214583.1 | GM632655.1 | GN082898.1 | CS631745.1 |
| DM114858.1 | FB653142.1 | DL027343.1 | HW066924.1 | HI540737.1 | HI214551.1 | GM632575.1 | GN089213.1 | CS631252.1 |
| GM668912.1 | FB654876.1 | DL027311.1 | HW083277.1 | HI589612.1 | HI214518.1 | GM632615.1 | GN082827.1 | CS631188.1 |
| GM061281.1 | FB580279.1 | DL027279.1 | HW083213.1 | HI001445.1 | HI179927.1 | GM632559.1 | GN088990.1 | CS642127.1 |
| HB387461.1 | FB653501.1 | DL027247.1 | HW081632.1 | HI001383.1 | HI214453.1 | GM632527.1 | GN079337.1 | CS627823.1 |
| HB384543.1 | FB580186.1 | DL027215.1 | HW099476.1 | HI001349.1 | HI214421.1 | GM632495.1 | GN079081.1 | DD236734.1 |
| HB338867.1 | FB580122.1 | DL027183.1 | HV704735.1 | HI001311.1 | HI213826.1 | GM674138.1 | DL233271.1 | DD234208.1 |
| HA642843.1 | FB573728.1 | DL023371.1 | HV704297.1 | AX352725.1 | HI185515.1 | GM658505.1 | GN078435.1 | E41541.1 |
| HA641594.1 | DL200020.1 | DL023307.1 | HV703152.1 | AX349185.1 | HI212986.1 | GM658425.1 | GN031793.1 | E33307.1 |
| HA641530.1 | DL199988.1 | DL023275.1 | HV701290.1 | AX349103.1 | HI546338.1 | GM658465.1 | GN031664.1 | E40308.1 |
| HA641271.1 | DL199853.1 | DL023243.1 | HV701196.1 | AX348864.1 | HI546306.1 | GM658409.1 | GN031632.1 | E06717.1 |
| HA641129.1 | DL199819.1 | DL023211.1 | HV701164.1 | AX348465.1 | HI586948.1 | GM658377.1 | GN031568.1 | E06006.1 |
| HC083540.1 | DL199723.1 | DL023179.1 | HV708200.1 | AX347584.1 | HI210984.1 | GM658345.1 | GN031536.1 | E05608.1 |
| DM460994.1 | DL201952.1 | DL020100.1 | HV708127.1 | AX347420.1 | HI210952.1 | GM651153.1 | GN031504.1 | E05086.1 |
| K00764.1 | DL184469.1 | DL020068.1 | HV701081.1 | AX347320.1 | HI570381.1 | GM651121.1 | GN031472.1 | E04121.1 |

| | | | | | | | | |
|---|---|---|---|---|---|---|---|---|
| HC061769.1 | DL193914.1 | DL015441.1 | HV701049.1 | AX347280.1 | HI473087.1 | GM651089.1 | GN031439.1 | E03342.1 |
| HC056941.1 | DL191630.1 | DL010951.1 | HV695511.1 | AX347244.1 | HI472925.1 | GM651057.1 | GN031375.1 | E03110.1 |
| DM384316.1 | DL196196.1 | DL010919.1 | HV695206.1 | AX347212.1 | HI210928.1 | GM651025.1 | GN031311.1 | E02725.1 |
| DM381905.1 | DL193796.1 | DL010855.1 | HV694824.1 | AX347174.1 | HI580539.1 | GM650993.1 | GN031278.1 | E01740.1 |
| HC055549.1 | DL193764.1 | DL010823.1 | HV694792.1 | AX347004.1 | HI209402.1 | GM644194.1 | GN031214.1 | E01489.1 |
| HC053257.1 | DL183399.1 | CS603073.1 | HV694656.1 | AX345054.1 | HI072377.1 | GM644162.1 | GN031182.1 | E01403.1 |
| HC051893.1 | DL183346.1 | CS603041.1 | HV694081.1 | AX344528.1 | HI004281.1 | GM644130.1 | GN031117.1 | E01300.1 |
| HC045301.1 | FB513373.1 | CS603009.1 | HV693314.1 | AX343857.1 | HI002262.1 | GM644098.1 | GN031085.1 | E01178.1 |
| HC047477.1 | FB512596.1 | CS602975.1 | HV689454.1 | AX339640.1 | HI002219.1 | GM644066.1 | GN031053.1 | DD088221.1 |
| HB488071.1 | FB571329.1 | CS602943.1 | HV688887.1 | AX338527.1 | HI002128.1 | GM644034.1 | GN031021.1 | DD087671.1 |
| HB475490.1 | FB571245.1 | CS602911.1 | HV585548.1 | AX329384.1 | HI002092.1 | GM637004.1 | GN030989.1 | DD098981.1 |
| HB475314.1 | CS803576.1 | CS602879.1 | HV585139.1 | AX320694.1 | HI000428.1 | GM636972.1 | GN030958.1 | DD112524.1 |
| HB475017.1 | CS696165.1 | CS602847.1 | HV029532.1 | AX317528.1 | HI000391.1 | GM636940.1 | GN030926.1 | DD117668.1 |
| HB474751.1 | CS696133.1 | CS602815.1 | HV035552.1 | AX317308.1 | DM380856.1 | GM636908.1 | GN030894.1 | DD152067.1 |
| HB470609.1 | CS696069.1 | CS602751.1 | FZ435982.1 | AX306687.1 | HC021037.1 | DL106570.1 | GN030862.1 | DD158389.1 |
| HB469131.1 | CS696037.1 | CS602719.1 | FZ422185.1 | AX306469.1 | HC025522.1 | DL106538.1 | GN030830.1 | DD157370.1 |
| HB468761.1 | CS695972.1 | CS602687.1 | FZ417010.1 | HW352263.1 | HC025490.1 | DL106506.1 | GN030798.1 | DD156971.1 |
| DM152632.1 | CS695940.1 | CS602399.1 | FW590745.1 | HW351580.1 | HC025458.1 | DL128669.1 | GN030766.1 | DD154928.1 |
| DM069061.1 | CS695908.1 | CS608450.1 | FW590143.1 | HW350675.1 | HC025426.1 | DL128635.1 | DM002280.1 | DD152405.1 |
| GN084707.1 | CS695876.1 | AX528533.1 | FW589134.1 | HW350643.1 | HC025061.1 | DL109354.1 | DM002015.1 | DD152120.1 |
| GN090095.1 | CS695844.1 | AX523631.1 | FW577607.1 | HW339916.1 | HC010661.1 | DL109322.1 | DM001477.1 | DD147808.1 |
| GN089834.1 | DL123505.1 | A24065.1 | FW577149.1 | HW349951.1 | HC010412.1 | DL109290.1 | DM006964.1 | CS038910.1 |
| GN082912.1 | DL123473.1 | A33338.1 | FW570996.1 | HW348595.1 | HC009022.1 | DL104556.1 | DM006721.1 | CS037845.1 |
| GN089177.1 | DL123441.1 | AX179415.1 | HI001091.1 | HW348495.1 | HC010673.1 | DL104524.1 | DM004989.1 | CS024306.1 |
| GN082809.1 | DL123409.1 | AX025157.1 | HI001035.1 | HW347989.1 | DM375338.1 | DL093940.1 | DL489823.1 | CS023578.1 |
| GN088025.1 | DL123377.1 | A04365.1 | HI001001.1 | HW363674.1 | DM371081.1 | DL093876.1 | DM003550.1 | CS017699.1 |
| L09145.1 | DL119123.1 | AX521536.1 | HI000954.1 | HW363642.1 | DM370680.1 | DL093844.1 | DM003504.1 | CS016531.1 |
| L08928.1 | DL119091.1 | AX521504.1 | HI002825.1 | HW363497.1 | DM370648.1 | DL093812.1 | DL488954.1 | CQ986651.1 |
| M94249.1 | DL119059.1 | AF430190.1 | HI002787.1 | HW306969.1 | DM370616.1 | DL093780.1 | DM002633.1 | CQ985743.1 |
| DM059607.1 | DL119027.1 | AF430158.1 | HI002727.1 | HW294140.1 | DM370584.1 | DL090037.1 | DL488543.1 | CQ981107.1 |
| DM058894.1 | DL114631.1 | AX511343.1 | HI002680.1 | HW294041.1 | DM370552.1 | DL090005.1 | DM010296.1 | CQ975427.1 |
| DM058816.1 | DL114599.1 | AX505205.1 | CS631739.1 | HW293110.1 | DM370520.1 | DL089973.1 | GM997935.1 | CQ975017.1 |
| DM056870.1 | DL114567.1 | AX497055.1 | CS631240.1 | HW292748.1 | DM370488.1 | DL085904.1 | GM997546.1 | CQ973469.1 |
| DM045506.1 | DL114535.1 | BD140005.1 | CS631176.1 | HW291283.1 | DM367513.1 | DL085872.1 | GM983649.1 | CQ977701.1 |
| DM045340.1 | DL114503.1 | BD138558.1 | CS627811.1 | HW291164.1 | DM372001.1 | DL126611.1 | GM996593.1 | CQ972352.1 |
| DM045087.1 | DL114471.1 | BD138400.1 | CS626239.1 | HW291122.1 | DM371347.1 | DL123356.1 | GM996497.1 | CQ971104.1 |
| DM044879.1 | DL109574.1 | BD138054.1 | DD438012.1 | HW291035.1 | HC003071.1 | DL123292.1 | GM994843.1 | DJ380841.1 |
| DM039595.1 | DL104872.1 | BD136874.1 | CS616569.1 | HW291003.1 | HB865038.1 | DL123260.1 | GM994331.1 | DJ388715.1 |
| DM060858.1 | DL104840.1 | BD135548.1 | CS613018.1 | HW290939.1 | HB864966.1 | DL123228.1 | GM992947.1 | DJ388672.1 |
| DM044778.1 | DL104808.1 | BD131906.1 | CS612806.1 | HW298871.1 | HB864998.1 | DL123196.1 | GM992641.1 | CS724397.1 |
| GN069090.1 | DL095917.1 | BD131150.1 | CS612717.1 | HW290908.1 | HB864942.1 | DL123164.1 | GM992480.1 | DJ358846.1 |
| GN067941.1 | DL095885.1 | BD130799.1 | CS603726.1 | HW290876.1 | HB864910.1 | DL118910.1 | GM992408.1 | CS424092.1 |
| GN068384.1 | DL095853.1 | BD130767.1 | CS603374.1 | HW290844.1 | HB864878.1 | DL118878.1 | GM992268.1 | CS422553.1 |
| GN075432.1 | DL095821.1 | BD130606.1 | CS174738.1 | HW290108.1 | HB864846.1 | DL118846.1 | GM992198.1 | CS417191.1 |
| GM645283.1 | DL095789.1 | BD129668.1 | CS176143.1 | HW298191.1 | HB864814.1 | DL118814.1 | GM992044.1 | CS411018.1 |
| GM645251.1 | DL094134.1 | BD107676.1 | CS172447.1 | HW267794.1 | HB864782.1 | DL109262.1 | GM991911.1 | CS410929.1 |
| GM645219.1 | DL094102.1 | BD105719.1 | CS172300.1 | HW267436.1 | HB864750.1 | DL109230.1 | GM709018.1 | CS414820.1 |

| | | | | | | | | |
|---|---|---|---|---|---|---|---|---|
| GM638131.1 | DL094070.1 | BD085700.1 | CS165976.1 | HW267191.1 | HB866539.1 | DL109198.1 | GM708986.1 | DD323521.1 |
| GM638099.1 | DL086194.1 | BD081897.1 | CS161637.1 | HW266204.1 | HB866218.1 | DL109166.1 | GM708966.1 | DD327011.1 |
| GM638067.1 | DL086162.1 | BD081629.1 | CS159814.1 | HW262993.1 | HB999689.1 | DL109134.1 | GM655015.1 | DD326769.1 |
| GM637778.1 | DL086130.1 | BD081304.1 | CS159782.1 | HW262913.1 | GM991937.1 | DL109102.1 | GM654983.1 | CS398393.1 |
| GM633242.1 | DL086098.1 | BD080677.1 | CS159593.1 | HW262870.1 | GM989961.1 | DL104496.1 | GM044480.1 | CS389297.1 |
| GM633210.1 | DL086066.1 | BD080330.1 | CS141602.1 | HW262570.1 | GM987025.1 | DL104432.1 | GM836868.1 | CS389256.1 |
| GM633178.1 | DL086034.1 | BD080138.1 | CS141540.1 | HW261404.1 | CS297030.1 | DL104400.1 | FB511158.1 | CS389209.1 |
| GM651767.1 | DL086002.1 | BD077093.1 | CS141508.1 | HW261372.1 | DD258732.1 | DL104368.1 | GM835769.1 | CS389171.1 |
| GM642952.1 | DL114436.1 | BD074980.1 | CS141288.1 | HW261340.1 | CQ854067.1 | DL104336.1 | GM752810.1 | CS401399.1 |
| GM630644.1 | DL114404.1 | BD074945.1 | CS134729.1 | HW261308.1 | CQ849439.1 | DL099922.1 | GM752475.1 | CS408138.1 |
| GM630612.1 | DL114372.1 | BD074653.1 | CS138160.1 | HW261276.1 | CQ830289.1 | DL099890.1 | DL467587.1 | CS401809.1 |
| GM630580.1 | DL031915.1 | BD073245.1 | CS133017.1 | HW261244.1 | CQ824367.1 | DL099858.1 | GM712962.1 | CS383390.1 |
| GM621645.1 | DL031883.1 | BD069449.1 | CS122830.1 | HW261212.1 | CQ818778.1 | DL099826.1 | GM652758.1 | CS382582.1 |
| GM621581.1 | DL031851.1 | BD063667.1 | CS122289.1 | HW261180.1 | CQ816993.1 | DL099794.1 | GM652726.1 | DD291062.1 |
| GM657129.1 | DL031819.1 | BD017649.1 | CS106028.1 | HW261148.1 | CQ816951.1 | DL099762.1 | GM652694.1 | CS376110.1 |
| GM642721.1 | DL031787.1 | BD014226.1 | CS179870.1 | HW261116.1 | CQ816913.1 | DL099730.1 | GM652662.1 | CS375830.1 |
| GM739605.1 | DL031755.1 | BD014194.1 | CS333957.1 | HW261084.1 | CQ816252.1 | DL093720.1 | GM652630.1 | BD428728.1 |
| GM656636.1 | DL027850.1 | E63776.1 | CS326662.1 | HW261052.1 | CQ814077.1 | DL093688.1 | GM652598.1 | BD426701.1 |
| GM670023.1 | DL027818.1 | AX481248.1 | CS332078.1 | HW261020.1 | CQ814044.1 | DL093656.1 | GM645504.1 | BD404622.1 |
| GM656522.1 | DL027786.1 | AX474736.1 | CS323607.1 | HW260988.1 | CQ814012.1 | DL093592.1 | GM645472.1 | BD445853.1 |
| GM656490.1 | DL027754.1 | AX471973.1 | CS323574.1 | HW260956.1 | CQ813980.1 | DL093560.1 | GM645440.1 | BD453369.1 |
| GM656426.1 | DL023846.1 | E51071.1 | CS330850.1 | HW260924.1 | CQ813948.1 | DL089945.1 | GM746438.1 | BD453333.1 |
| GM649273.1 | DL023814.1 | HW374804.1 | CS320130.1 | HW260892.1 | CQ813916.1 | DL089913.1 | GM638611.1 | BD453305.1 |
| GM649241.1 | DL023782.1 | HW375064.1 | DD266011.1 | HV767071.1 | CQ813884.1 | DL089849.1 | GM638579.1 | BD453273.1 |
| GM649209.1 | DL016172.1 | HW381040.1 | DD272442.1 | HV760606.1 | CQ813852.1 | DL089785.1 | GM638547.1 | BD402327.1 |
| DL262682.1 | DL016140.1 | HW353809.1 | CS297028.1 | HV766069.1 | CQ813820.1 | DL085780.1 | GM638515.1 | BD413430.1 |
| DL258517.1 | DL031640.1 | HW353592.1 | CS102607.1 | HV750193.1 | CQ813787.1 | DL085748.1 | GM638483.1 | BD388659.1 |
| DL256303.1 | DL031608.1 | HW353461.1 | CS102575.1 | HV758710.1 | CQ802187.1 | DL085716.1 | GM638451.1 | BD376129.1 |
| DL241167.1 | DL031576.1 | HW256712.1 | CS102543.1 | HV758646.1 | CQ800900.1 | DL085684.1 | GM622857.1 | BD374164.1 |
| DL240252.1 | DL031544.1 | HW251202.1 | CS102511.1 | HV755152.1 | CQ797811.1 | DL085652.1 | GM622825.1 | BD356838.1 |
| FB748854.1 | DL023667.1 | HW250928.1 | CS102479.1 | HV745531.1 | CQ795507.1 | DL123141.1 | FB507961.1 | BD356503.1 |
| FB674294.1 | DL023635.1 | HW249704.1 | CS101278.1 | HV749414.1 | CQ795469.1 | DL123109.1 | GM704481.1 | BD350553.1 |
| DL236213.1 | DL023603.1 | AB775805.1 | CS088902.1 | HV748639.1 | CQ789709.1 | DL123045.1 | GM657910.1 | BD341800.1 |
| DL227076.1 | DL015993.1 | HW247835.1 | CS086834.1 | HV748517.1 | CQ788572.1 | DL123013.1 | GM657878.1 | BD325691.1 |
| FB720134.1 | DL015961.1 | HW240928.1 | CS085415.1 | HV748471.1 | AX014765.1 | DL122981.1 | GM657846.1 | BD325486.1 |
| FB713815.1 | DL015929.1 | HW240896.1 | CS084380.1 | HV747500.1 | AX010923.1 | DL118791.1 | GM657781.1 | BD314343.1 |
| FB713751.1 | DL015897.1 | HW240832.1 | CS070587.1 | HV753777.1 | AF187979.1 | DL118759.1 | GM657749.1 | BD319275.1 |
| FB713719.1 | DL015865.1 | HW240736.1 | CS070146.1 | HV753552.1 | U49850.1 | DL118727.1 | GM643390.1 | BD317780.1 |
| FB713452.1 | DJ419895.1 | HW240648.1 | CS067356.1 | HV753429.1 | A41840.1 | DL118695.1 | GM643358.1 | BD300559.1 |
| FB712617.1 | DJ419222.1 | HW239328.1 | CS063868.1 | HV509412.1 | A25422.1 | DL118663.1 | GM643326.1 | BD300253.1 |
| FB674473.1 | DJ417434.1 | HW238752.1 | CS061699.1 | HV508685.1 | A35723.1 | DL118631.1 | GM643294.1 | BD293466.1 |
| FB671076.1 | DJ402724.1 | HW238559.1 | AY967397.1 | HV508618.1 | A35297.1 | DL025264.1 | GM643262.1 | BD291406.1 |
| FB670504.1 | DJ387697.1 | HW238400.1 | AY967365.1 | HV508586.1 | A34512.1 | DL025232.1 | GM643230.1 | BD291284.1 |
| FB667475.1 | DJ381035.1 | HW242996.1 | AY967333.1 | HV508554.1 | A33450.1 | DL025200.1 | GM636241.1 | BD299627.1 |
| FB666440.1 | DJ381003.1 | HW242951.1 | AY967301.1 | HV508522.1 | A32420.1 | DL025168.1 | GM636209.1 | CQ849443.1 |
| FB671498.1 | DJ380971.1 | HW248920.1 | AY967269.1 | CQ986627.1 | A30353.1 | DL021956.1 | GM636177.1 | CQ840714.1 |
| FB704833.1 | DJ380939.1 | HW244251.1 | AY967237.1 | CQ986593.1 | A26136.1 | DL021924.1 | GM636145.1 | AY324098.1 |

| | | | | | | | | |
|---|---|---|---|---|---|---|---|---|
| FB676525.1 | DJ380907.1 | HW237891.1 | AY967205.1 | CQ985858.1 | A06249.1 | DL021892.1 | GM636113.1 | CQ830293.1 |
| FB705631.1 | DJ380875.1 | HW237850.1 | AY967173.1 | CQ983065.1 | A04445.1 | DL021860.1 | GM636081.1 | CQ829250.1 |
| DL109178.1 | DJ380823.1 | HW248686.1 | AY967141.1 | CQ982931.1 | A11820.1 | DL021796.1 | GM631673.1 | CQ821310.1 |
| AY657853.1 | DJ388691.1 | HW237381.1 | AY967109.1 | CQ982899.1 | A19973.1 | DL017430.1 | GM631641.1 | CQ821001.1 |
| AY657821.1 | CS724659.1 | HW242314.1 | AY967077.1 | CQ981120.1 | A12648.1 | DL038466.1 | GM631609.1 | CQ816996.1 |
| AY657789.1 | CS724489.1 | HW237204.1 | AY967045.1 | CQ975458.1 | A11125.1 | DL038434.1 | GM631577.1 | CQ816953.1 |
| AY657757.1 | CS724456.1 | HW241640.1 | AY967013.1 | CQ973478.1 | A06512.1 | DL038402.1 | GM631545.1 | CQ816915.1 |
| AY657725.1 | DJ353117.1 | HW241452.1 | AY966981.1 | CQ976608.1 | A07762.1 | DL038370.1 | GM631513.1 | CQ814468.1 |
| AY657693.1 | DJ352789.1 | HW163851.1 | AY966949.1 | CQ972360.1 | A04774.1 | DL038338.1 | GM622742.1 | CQ814046.1 |
| AY657661.1 | DJ359584.1 | HW161204.1 | CS052399.1 | CQ971120.1 | A00125.1 | DL038306.1 | GM622710.1 | CQ814014.1 |
| AY657629.1 | DJ357681.1 | HV694841.1 | CS052330.1 | CQ970842.1 | A31639.1 | DL013175.1 | GM622678.1 | CQ813982.1 |
| AY657597.1 | DJ357649.1 | HV694806.1 | CS052297.1 | CQ970040.1 | A28934.1 | DL013143.1 | GM740789.1 | CQ813950.1 |
| AY657565.1 | DJ357617.1 | HV694764.1 | CS051000.1 | CQ967273.1 | A28847.1 | DL013111.1 | GM704180.1 | CQ813918.1 |
| AY657533.1 | DJ357585.1 | HV700695.1 | AX112901.1 | CQ947532.1 | A23083.1 | DL013079.1 | GM650376.1 | CQ813886.1 |
| AY657501.1 | DJ341132.1 | HV694707.1 | AX107891.1 | CQ947164.1 | A28241.1 | DL013047.1 | GM622454.1 | CQ813854.1 |
| AY657469.1 | DJ363051.1 | HV694675.1 | AX107108.1 | CQ947119.1 | A26533.1 | DL013015.1 | GM622422.1 | CQ813822.1 |
| AY657437.1 | DJ362240.1 | HV694146.1 | AX105619.1 | CQ945503.1 | A25478.1 | DL033910.1 | GM622390.1 | CQ813790.1 |
| AY657405.1 | DJ354542.1 | HV693378.1 | AX097498.1 | CQ944189.1 | A23318.1 | DL033878.1 | GM622358.1 | CQ812760.1 |
| AY657373.1 | DJ361207.1 | HV699150.1 | AX093078.1 | CQ944157.1 | A23657.1 | DL033782.1 | GM631346.1 | CQ803225.1 |
| AY657341.1 | DJ354205.1 | HV698456.1 | AX088392.1 | CQ944125.1 | A20313.1 | DL033750.1 | GM631314.1 | CQ802190.1 |
| AY657309.1 | DJ360421.1 | HV698275.1 | AX085625.1 | CQ944093.1 | A14494.1 | AX108703.1 | GM703824.1 | CQ801296.1 |
| AY657277.1 | DJ339854.1 | HV698142.1 | AX084730.1 | CQ944061.1 | A21814.1 | AX108335.1 | GM657512.1 | CQ800902.1 |
| AY657245.1 | DJ339758.1 | HV690771.1 | AX083959.1 | CQ944029.1 | A21467.1 | AX107884.1 | GM657480.1 | CQ800651.1 |
| AY657213.1 | DJ339726.1 | HV579906.1 | AX081281.1 | AB084112.1 | A16533.1 | AX107384.1 | GM657448.1 | CQ795471.1 |
| AY657181.1 | DJ339662.1 | HV585089.1 | AX080817.1 | AX962030.1 | A18947.1 | AX100597.1 | GM657416.1 | CQ795439.1 |
| AY657149.1 | DJ339565.1 | HW163850.1 | AX077351.1 | AX960209.1 | A18006.1 | AX100166.1 | GM657384.1 | CQ788610.1 |
| AY657117.1 | DJ334065.1 | HW163125.1 | AX076542.1 | AX959225.1 | A16051.1 | AX099996.1 | GM657352.1 | CQ787322.1 |
| AY657085.1 | CS389357.1 | HW161200.1 | AX073548.1 | AX958630.1 | A16112.1 | AX097525.1 | GM650160.1 | CQ787290.1 |
| AY657053.1 | CS389279.1 | HW155720.1 | AX068019.1 | AX958112.1 | A15907.1 | AX093404.1 | GM650128.1 | CQ786569.1 |
| AF409120.1 | CS389225.1 | HW155680.1 | AX058582.1 | AX958065.1 | A12464.1 | AX088806.1 | GM650096.1 | CQ784695.1 |
| M27885.1 | CS389189.1 | HW155512.1 | AX057310.1 | AX952692.1 | A12109.1 | AX088708.1 | GM643127.1 | CQ784629.1 |
| M15804.1 | CS400829.1 | HW155226.1 | AX057046.1 | AX937802.1 | A11523.1 | AX088370.1 | GM643095.1 | CQ778520.1 |
| M26402.1 | CS398634.1 | HW154984.1 | AX052933.1 | AX931825.1 | A10906.1 | AX085505.1 | GM643063.1 | CQ778398.1 |
| M64645.1 | CS401604.1 | HW154875.1 | AX052817.1 | AX924206.1 | HV753228.1 | AX084287.1 | GM643031.1 | AX010925.1 |
| M22211.1 | CS406190.1 | HW158475.1 | AX046620.1 | AX923402.1 | HV758999.1 | AX081670.1 | GM636010.1 | AX003644.1 |
| HC889146.1 | CS403495.1 | HW154726.1 | AX041938.1 | AX840303.1 | HV758601.1 | AX081148.1 | GM635978.1 | AX003016.1 |
| HC870092.1 | CS402172.1 | HV743339.1 | AX039931.1 | AX825028.1 | HV755011.1 | AX077300.1 | GM635946.1 | A57331.1 |
| FW310606.1 | CS402069.1 | HV743299.1 | AY658935.1 | AX824437.1 | HV753620.1 | AX074252.1 | GM635914.1 | A41849.1 |
| FW308821.1 | CS382122.1 | HV743223.1 | AY658903.1 | AX824333.1 | HV753496.1 | AX073932.1 | GM635882.1 | A25426.1 |
| FW334317.1 | CS380096.1 | HV704410.1 | AY658871.1 | AX823765.1 | HV743102.1 | AJ296082.1 | GM631093.1 | A35725.1 |
| FW332940.1 | CS382614.1 | HV704250.1 | AY658839.1 | AX814466.1 | HV509309.1 | AX068404.1 | GM635333.1 | A29457.1 |
| FW332331.1 | CS376549.1 | HV704177.1 | AY658807.1 | AX814403.1 | HV508637.1 | AX068159.1 | GM635301.1 | A33452.1 |
| FW306356.1 | DD298339.1 | HV744579.1 | AY658775.1 | AX814198.1 | HV508573.1 | AX061388.1 | GM635269.1 | A06775.1 |
| FW305533.1 | DD308736.1 | HV703165.1 | AY658743.1 | AX802890.1 | HV508541.1 | AX060782.1 | GM630431.1 | A04006.1 |
| FW310636.1 | DD292513.1 | HV702433.1 | AY658711.1 | AX799569.1 | HV508509.1 | AX060092.1 | GM630399.1 | A07610.1 |
| AF491939.1 | DD291678.1 | HV701835.1 | AY658679.1 | AX798335.1 | HV508481.1 | AX057304.1 | GM630367.1 | A06131.1 |
| HC754641.1 | DD290972.1 | HV701241.1 | AY658647.1 | AX797698.1 | HV512283.1 | AX055859.1 | GM630335.1 | A05165.1 |

| | | | | | | | | |
|---|---|---|---|---|---|---|---|---|
| HC732129.1 | DD319819.1 | HV701209.1 | AY658615.1 | AX795426.1 | HV512251.1 | AX052963.1 | GM630303.1 | A04834.1 |
| HC731619.1 | CS365238.1 | HV701177.1 | AY658583.1 | AX144465.1 | HV512219.1 | AX050338.1 | GM621528.1 | A02815.1 |
| HC471756.1 | CS366520.1 | HV701145.1 | AY658551.1 | AX144401.1 | HV512187.1 | AX047567.1 | GM621496.1 | A08349.1 |
| FU263222.1 | CS362653.1 | HV701113.1 | AY658519.1 | AX144337.1 | HV512123.1 | AX046225.1 | GM739526.1 | A00382.1 |
| FU265390.1 | CS359741.1 | HV708696.1 | AY658487.1 | AX144273.1 | HV504786.1 | AX044425.1 | GM702854.1 | A02086.1 |
| FU265358.1 | CS359605.1 | HV701094.1 | AY658455.1 | AX144207.1 | HV504722.1 | AX043877.1 | GM656590.1 | A01412.1 |
| FU264293.1 | DQ459358.1 | HV701062.1 | AY658423.1 | AX144079.1 | HV504690.1 | AX041926.1 | GM656558.1 | A00372.1 |
| DM474173.1 | BD269572.1 | HV695524.1 | AY658391.1 | AX143885.1 | HV504658.1 | AX040464.1 | GM649533.1 | A01318.1 |
| HC306227.1 | BD269142.1 | HV695187.1 | AY658359.1 | AX143821.1 | HV504626.1 | AX039592.1 | GM649501.1 | A00499.1 |
| HC306187.1 | BD268163.1 | HV695123.1 | AY658327.1 | AX143629.1 | HV491631.1 | HW309716.1 | GM649469.1 | M12612.1 |
| HC306151.1 | BD267566.1 | HV694840.1 | AY658295.1 | AX143501.1 | HV504977.1 | HW308095.1 | GM649437.1 | M12053.1 |
| HC302635.1 | BD266725.1 | HV694805.1 | AY658263.1 | AX143437.1 | HV455603.1 | HW307875.1 | GM649405.1 | M18420.1 |
| HC305919.1 | BD265613.1 | HV700726.1 | AY658231.1 | AX143053.1 | AX286077.1 | HW307843.1 | GM670045.1 | M31881.1 |
| HC305879.1 | BD264412.1 | HV700694.1 | AY658199.1 | AX142989.1 | AX283083.1 | HW307811.1 | GM656540.1 | M10046.1 |
| HC305839.1 | BD263452.1 | HV694674.1 | AY658167.1 | AX142925.1 | AX279821.1 | HW307779.1 | GM656508.1 | J02557.1 |
| HC305799.1 | BD263338.1 | HV694123.1 | AY658135.1 | AX142797.1 | AX256450.1 | HW316591.1 | GM656476.1 | AY005803.1 |
| HC305759.1 | BD262933.1 | HV693869.1 | AY658103.1 | AX142283.1 | AX254832.1 | HW307741.1 | GM656412.1 | GN044355.1 |
| HC305719.1 | HC037102.1 | HV699149.1 | AY658071.1 | AX142155.1 | AX242310.1 | HW307709.1 | GM656380.1 | AH002300.2 |
| HC305679.1 | HC036996.1 | HV698608.1 | AY658039.1 | AX142091.1 | AX242246.1 | HW315972.1 | GM656348.1 | AH003654.2 |
| HC305639.1 | HC022631.1 | HV698274.1 | AY658007.1 | AX141961.1 | AX242182.1 | HW315646.1 | GM649303.1 | K03395.1 |
| HC291594.1 | HC025573.1 | HV698141.1 | AY657975.1 | AX141831.1 | AX242150.1 | HW315569.1 | GM649315.1 | DQ250193.1 |
| HC251208.1 | HC025501.1 | HV690770.1 | AY657943.1 | AX141703.1 | AX242086.1 | HW315536.1 | GM649259.1 | AF156935.1 |
| HC289366.1 | HC025469.1 | HV302971.1 | AY657911.1 | AX141639.1 | AX241990.1 | HW315176.1 | GM649227.1 | AF003715.1 |
| HC289332.1 | HC025437.1 | HV302939.1 | AY657879.1 | AX141511.1 | AX241958.1 | HW315080.1 | GM642586.1 | HW408861.1 |
| HC292852.1 | HC025405.1 | HV308717.1 | AY657847.1 | AX141383.1 | AX241926.1 | HW315024.1 | GM642554.1 | HW390735.1 |
| HC296929.1 | HC010423.1 | HV308618.1 | AY657815.1 | AX137846.1 | AX241894.1 | HW314958.1 | GM642522.1 | HW399775.1 |
| HC296241.1 | HC010332.1 | HV317188.1 | AY657783.1 | AX134961.1 | AX241862.1 | HW314910.1 | GM642457.1 | HW399355.1 |
| HC292720.1 | HC008340.1 | HV226847.1 | AY657751.1 | AX134686.1 | AX241830.1 | HW314849.1 | GM642425.1 | HW381659.1 |
| HC295616.1 | HC020909.1 | HV313067.1 | AY657719.1 | AX128561.1 | AX241798.1 | HW314642.1 | GM635243.1 | HW390544.1 |
| HC295472.1 | HC007675.1 | HV309534.1 | AY657687.1 | AX127538.1 | AX241734.1 | HW144957.1 | GM635211.1 | HW390495.1 |
| HC292422.1 | HC007643.1 | HV194841.1 | AY657655.1 | AX119840.1 | AX241702.1 | HW115435.1 | GM635179.1 | HW390394.1 |
| HC295313.1 | HC007615.1 | HV040125.1 | AY657623.1 | AF364062.1 | AX241670.1 | HW115400.1 | GM635147.1 | HW389052.1 |
| HC292170.1 | HC007583.1 | HV097474.1 | AY657591.1 | AX111694.1 | AX241638.1 | HW113244.1 | GM635115.1 | HW388327.1 |
| HC299171.1 | HC007551.1 | HV200505.1 | AY657559.1 | AX108251.1 | AX241606.1 | HW112829.1 | GM635083.1 | HW387496.1 |
| HC294516.1 | HC007519.1 | HV182486.1 | AY657527.1 | AX107122.1 | AX241574.1 | HW112705.1 | DL023705.1 | HW154060.1 |
| HC293942.1 | HC010684.1 | HV182454.1 | AY657495.1 | AX103358.1 | HW328632.1 | HW112624.1 | DL023673.1 | HW153891.1 |
| HC293762.1 | DM375844.1 | HV182422.1 | AY657463.1 | AX097513.1 | HW328419.1 | HW112369.1 | DL023641.1 | HW153775.1 |
| HC290809.1 | DM371127.1 | HV182390.1 | AY657431.1 | AX097405.1 | HW328307.1 | HW072754.1 | DL023609.1 | HW153511.1 |
| HC293265.1 | DM370659.1 | HV221006.1 | AY657399.1 | AX093093.1 | HW328253.1 | HW070088.1 | DL023577.1 | HW145242.1 |
| HC293068.1 | DM370627.1 | HV216735.1 | AY657367.1 | AX090033.1 | HW328107.1 | HW069960.1 | DL020530.1 | HW153373.1 |
| DM016034.1 | DM370595.1 | HV232681.1 | AY657335.1 | AX088786.1 | HW318552.1 | HW069796.1 | DL020498.1 | HW150976.1 |
| DM026458.1 | DM370563.1 | HV039523.1 | AY657303.1 | AX088012.1 | HW335586.1 | HW069764.1 | DL020466.1 | HW126584.1 |
| GN042357.1 | DM370531.1 | HV038721.1 | AY657271.1 | AX082191.1 | HW314336.1 | HW084952.1 | DL020434.1 | HW125557.1 |
| GN041489.1 | DM370499.1 | HV038680.1 | AY657239.1 | AX081492.1 | HW314118.1 | HW097746.1 | DL015999.1 | HW125371.1 |
| GN034802.1 | DM370467.1 | HV038652.1 | AY657207.1 | HW380807.1 | HW312144.1 | HW088947.1 | DL015967.1 | HW144436.1 |
| GN030761.1 | DM367524.1 | HV038592.1 | AY657175.1 | HW374970.1 | HW312090.1 | HW097631.1 | DL015935.1 | HW144358.1 |
| GN030697.1 | DM205458.1 | HV182352.1 | AY657143.1 | HW373213.1 | HW311868.1 | HW065061.1 | DL015871.1 | HW144294.1 |

| | | | | | | | | |
|---|---|---|---|---|---|---|---|---|
| GN030633.1 | HC003094.1 | HV182320.1 | AF377318.1 | HW352454.1 | HW307882.1 | HW060596.1 | DL015839.1 | HW124775.1 |
| GN030601.1 | HC002973.1 | HV190835.1 | HC453691.1 | HW329326.1 | HW307850.1 | HW050529.1 | DL011253.1 | HW123003.1 |
| GN030569.1 | HB865831.1 | HV228459.1 | U94951.1 | HW335972.1 | HW307818.1 | HV969755.1 | DL011221.1 | HW122041.1 |
| GN030505.1 | HB865049.1 | FW496916.1 | FU761086.1 | HW328740.1 | HW307748.1 | HV956158.1 | DL047645.1 | HW118450.1 |
| GN030377.1 | GM636930.1 | FW420346.1 | FU772852.1 | GM641688.1 | HW307716.1 | HV951085.1 | DL047613.1 | HW117988.1 |
| GN030249.1 | GM636898.1 | FW496665.1 | FU764204.1 | GM655003.1 | HW307684.1 | HV957530.1 | DL047581.1 | HV951956.1 |
| GN013583.1 | GM636866.1 | FW503285.1 | FU763330.1 | GM640495.1 | HW315842.1 | HV957461.1 | DL047549.1 | HV951868.1 |
| GN010358.1 | GM623527.1 | HI653799.1 | FU762511.1 | GM044199.1 | HW315653.1 | HV940358.1 | DL047517.1 | HV939873.1 |
| GN033533.1 | GM623495.1 | HI653517.1 | HC358389.1 | GM043999.1 | HW315576.1 | HV944850.1 | DL047485.1 | HV925510.1 |
| HI000419.1 | GM623431.1 | HI653355.1 | HC358201.1 | GM755060.1 | HW315543.1 | HV778433.1 | DL039654.1 | HV936329.1 |
| HI000377.1 | GM623399.1 | HI651901.1 | HC358070.1 | GM712944.1 | HW315151.1 | HW089225.1 | DL031253.1 | HV933097.1 |
| HI000320.1 | GM623367.1 | HI650449.1 | HC321183.1 | GM652746.1 | HW160818.1 | HW067535.1 | DL031221.1 | HV932706.1 |
| HI553273.1 | GM643854.1 | HH806506.1 | HC356757.1 | GM652714.1 | HW069967.1 | HW102116.1 | DL031189.1 | HV929979.1 |
| HI521616.1 | GM643822.1 | HH802666.1 | HC325497.1 | GM652682.1 | HW069935.1 | HW067359.1 | DL031157.1 | HV585107.1 |
| HI577095.1 | GM636825.1 | HH794946.1 | HC325069.1 | GM652650.1 | HW069803.1 | HW083511.1 | DL027348.1 | HV592436.1 |
| HI003855.1 | GM636793.1 | HH794714.1 | HC324124.1 | GM652618.1 | HW084920.1 | HW083237.1 | DL027316.1 | HV592372.1 |
| HI002089.1 | GM636761.1 | HH794193.1 | HC319664.1 | GM652586.1 | HW097753.1 | HW083205.1 | DL027284.1 | HV586128.1 |
| HI002039.1 | GM632257.1 | HH791199.1 | HC308998.1 | GM645492.1 | HW065068.1 | HW082743.1 | DL027252.1 | HV600961.1 |
| HI001955.1 | GM632225.1 | HH774430.1 | HC307876.1 | GM645460.1 | HW064756.1 | HW081795.1 | DL027220.1 | HV585553.1 |
| HI000287.1 | GM623262.1 | HH773129.1 | DM130799.1 | GM645428.1 | HW060676.1 | HW081718.1 | DL027188.1 | HV570618.1 |
| HI000246.1 | GM623230.1 | HH820910.1 | HB427183.1 | GM657404.1 | HW062418.1 | HW085246.1 | DL027156.1 | HV572458.1 |
| HI000202.1 | GM741836.1 | HH759206.1 | HB426587.1 | GM650148.1 | HW062386.1 | HW099468.1 | DL023344.1 | HV572319.1 |
| HI000146.1 | GM658286.1 | HH759174.1 | HB403638.1 | GM650116.1 | HW060495.1 | HW103539.1 | DL023312.1 | HV560180.1 |
| HI000114.1 | GM658254.1 | HH759110.1 | HB396576.1 | GM650084.1 | HW056334.1 | HW103427.1 | DL023280.1 | HV226904.1 |
| HI204476.1 | GM658222.1 | HH759078.1 | HB397678.1 | GM643083.1 | HW056249.1 | HW102905.1 | DL023248.1 | HV305443.1 |
| HI552479.1 | GM658190.1 | HH759046.1 | GM691505.1 | GM643051.1 | HW064460.1 | HW102876.1 | DL023216.1 | HV197646.1 |
| HI551899.1 | GM658158.1 | HH759014.1 | GM047919.1 | GM643019.1 | HW062361.1 | HC501800.1 | DL023184.1 | HV200446.1 |
| HI470583.1 | GM650870.1 | HH758982.1 | HB386672.1 | GM635870.1 | HW062329.1 | HC500771.1 | DL020169.1 | HV182441.1 |
| HI551643.1 | GM658076.1 | HH758950.1 | HA641243.1 | FB506778.1 | HW062233.1 | HC499871.1 | DL020137.1 | HV200216.1 |
| HI538373.1 | GM658044.1 | HC732140.1 | DM069115.1 | GM642991.1 | HW042052.1 | FW303973.1 | DL020105.1 | HV235479.1 |
| HI559228.1 | GM658012.1 | HC731683.1 | GN089880.1 | GM642958.1 | HW043510.1 | FW302605.1 | DL020073.1 | HV038662.1 |
| HI564723.1 | GM657980.1 | DD133697.1 | GN089816.1 | GM642926.1 | HW042008.1 | FW301618.1 | DL020041.1 | HV190739.1 |
| HI564229.1 | GM657948.1 | DD132326.1 | GN082903.1 | GM630650.1 | HW041976.1 | FW301170.1 | DL020009.1 | HV190483.1 |
| HI564108.1 | GM650756.1 | DD107338.1 | GN082832.1 | GM630618.1 | HW041912.1 | FW300629.1 | DL015446.1 | HV225733.1 |
| HI583096.1 | GM650692.1 | DD106654.1 | GN082356.1 | GM630586.1 | HW041850.1 | FW299455.1 | DL010924.1 | HI654026.1 |
| HI563633.1 | GM643700.1 | DD118908.1 | L08958.1 | GM635036.1 | HW049510.1 | FW299340.1 | DL010892.1 | HI653911.1 |
| HI563601.1 | GM643668.1 | DD091016.1 | L08918.1 | GM635004.1 | HW049366.1 | FW299308.1 | DL010860.1 | HI651881.1 |
| HI557535.1 | GM636543.1 | DD104229.1 | L08783.1 | GM634972.1 | HW049335.1 | FW299276.1 | DL010828.1 | AY145508.1 |
| HI508516.1 | GM636511.1 | DD112770.1 | M33420.1 | GM634940.1 | HW049303.1 | FW299244.1 | DL039087.1 | HH797408.1 |
| HI000021.1 | GM636479.1 | DD090296.1 | DM057732.1 | GM634908.1 | HW049271.1 | FW298557.1 | DL023149.1 | HH794733.1 |
| HI003487.1 | GM632071.1 | DD103428.1 | DM045542.1 | GM634876.1 | HW049239.1 | HC469463.1 | DL023117.1 | HH777934.1 |
| HI001754.1 | GM623076.1 | DD103243.1 | DM045285.1 | GM629974.1 | HW043310.1 | HC469401.1 | DL023085.1 | HI004337.1 |
| HI469731.1 | GM653216.1 | CS102972.1 | DM045008.1 | GM629942.1 | HW041810.1 | HC486609.1 | DL023053.1 | HI004273.1 |
| HI544119.1 | GM646089.1 | CS102908.1 | AX241410.1 | GM629910.1 | HW041778.1 | HC486458.1 | DL023021.1 | HI002258.1 |
| FU267906.1 | GM646057.1 | CS102812.1 | AX225275.1 | GM669848.1 | HW055332.1 | HC486340.1 | DL022989.1 | HI002206.1 |
| HC306224.1 | GM646025.1 | CS102716.1 | AF408180.1 | GM656327.1 | HW046688.1 | HC492551.1 | DL015411.1 | HI002164.1 |
| HC306184.1 | GM626387.1 | CS102684.1 | AX207303.1 | GM656295.1 | HV217032.1 | HC475389.1 | DL015379.1 | HI002124.1 |

| | | | | | | | | |
|---|---|---|---|---|---|---|---|---|
| HC302633.1 | GM626355.1 | CS102652.1 | AX203737.1 | GM656263.1 | HV190941.1 | HC466644.1 | DL015347.1 | HI000496.1 |
| HC305957.1 | GM660476.1 | CS102620.1 | AX202435.1 | GM656231.1 | HV190736.1 | HC466612.1 | DL015315.1 | HW257241.1 |
| HC305917.1 | GM660380.1 | CS102588.1 | AX195203.1 | GM656199.1 | HV035211.1 | HC466580.1 | DL015283.1 | HW257017.1 |
| HC305877.1 | GM660348.1 | CS102556.1 | AX193968.1 | GM656167.1 | FZ422184.1 | HC492341.1 | DL010793.1 | HV455602.1 |
| HC305837.1 | GM653161.1 | CS102524.1 | AX189501.1 | GM649174.1 | FW561681.1 | HC491799.1 | DL010761.1 | HV502799.1 |
| HC305797.1 | GM653129.1 | CS102492.1 | AF394910.1 | GM642179.1 | FW561649.1 | HC491767.1 | DL010729.1 | HV502767.1 |
| HC305757.1 | GM653097.1 | CS088958.1 | AX172949.1 | GM642147.1 | FW507408.1 | HC481599.1 | DL010697.1 | HV502735.1 |
| HC305717.1 | GM653065.1 | CS085787.1 | AX167420.1 | GM642115.1 | D88393.1 | HC491460.1 | DL010665.1 | HV502703.1 |
| HC305677.1 | GM653033.1 | CS079802.1 | AX163791.1 | GM642083.1 | HI936528.1 | HC472609.1 | DL010633.1 | HV502639.1 |
| HC305637.1 | GM653001.1 | CS078822.1 | AX145708.1 | GM642051.1 | HI930656.1 | HC479088.1 | DL047057.1 | HV502607.1 |
| HC291570.1 | GM639007.1 | AY967378.1 | AX145676.1 | GM719207.1 | HI930273.1 | HC488128.1 | DL047025.1 | HV502543.1 |
| HC291294.1 | GM638975.1 | AY967346.1 | AX145644.1 | GM648962.1 | M23201.1 | HC470678.1 | DL046961.1 | HV502511.1 |
| HC289364.1 | GM638943.1 | AY967314.1 | AX145612.1 | GM648930.1 | HI918279.1 | FV533520.1 | DL046929.1 | HV502447.1 |
| HC289330.1 | GM638911.1 | AY967282.1 | AX145580.1 | GM648898.1 | HI661449.1 | FV532168.1 | DL046897.1 | HV449932.1 |
| HC292828.1 | GM638879.1 | AY967250.1 | AX145548.1 | GM648866.1 | HI660612.1 | FV531722.1 | DL046865.1 | FW570989.1 |
| HC292696.1 | GM626168.1 | AY967218.1 | AX145516.1 | GM648834.1 | HI284283.1 | FV531690.1 | DL043043.1 | FW568061.1 |
| HC295612.1 | GM652969.1 | AY967186.1 | AX145452.1 | GM648802.1 | HI574504.1 | FV531397.1 | DL043011.1 | FW571801.1 |
| HC292398.1 | GM652937.1 | AY967154.1 | AX145420.1 | GM634523.1 | HI574472.1 | FV523609.1 | DL042979.1 | HI967458.1 |
| HC295289.1 | GM652905.1 | AY967122.1 | AX145387.1 | GM634491.1 | HI574440.1 | FV530958.1 | DL042947.1 | HI987667.1 |
| HC292146.1 | GM652873.1 | AY967090.1 | AX145323.1 | GM629686.1 | HI574408.1 | FV530776.1 | DL042915.1 | HI987448.1 |
| HC291966.1 | GM652841.1 | AY967058.1 | AX145291.1 | GM641821.1 | HI580822.1 | FV522828.1 | DL042883.1 | FW555579.1 |
| HC299169.1 | GM652809.1 | AY967026.1 | AX145259.1 | GM664845.1 | HI584655.1 | FV530203.1 | DL039066.1 | HH777933.1 |
| HC291714.1 | GM652776.1 | AY966994.1 | AX145227.1 | GM648738.1 | HI636989.1 | FV528648.1 | DL039034.1 | HH820931.1 |
| HC293738.1 | HV588886.1 | AY966962.1 | AX145195.1 | GM648706.1 | HI636948.1 | FV534569.1 | DL039002.1 | HH819342.1 |
| HC290785.1 | HV584763.1 | AY966930.1 | AX145163.1 | GM648674.1 | HI001462.1 | FV534477.1 | DL038970.1 | HI401251.1 |
| HC293236.1 | HV579123.1 | CS057766.1 | AX145131.1 | DL125475.1 | HI001419.1 | FV534425.1 | DL038938.1 | HI401104.1 |
| GN368269.1 | HV579004.1 | CS055301.1 | AX145099.1 | DL125443.1 | HI001366.1 | HB819139.1 | DL038906.1 | HI637683.1 |
| GN360108.1 | HV592338.1 | AX587733.1 | AX145067.1 | DL125411.1 | HI001332.1 | HB818773.1 | DL035122.1 | HI378112.1 |
| GN356189.1 | HV578517.1 | AX587635.1 | AX145035.1 | DL125379.1 | HI003215.1 | HB842659.1 | DL031086.1 | HI375903.1 |
| GN359971.1 | HV582924.1 | AX587532.1 | AX145003.1 | DL125347.1 | HI003169.1 | HB806853.1 | DL031054.1 | HI371025.1 |
| GN359862.1 | HV601497.1 | BD161915.1 | AX144971.1 | DL117186.1 | HI003103.1 | HB842134.1 | DL031022.1 | HI369138.1 |
| GN359798.1 | HV575508.1 | BD145080.1 | AX144939.1 | DL117122.1 | HI003057.1 | HB835194.1 | DL030990.1 | HI369090.1 |
| GN359766.1 | HV601354.1 | BD143212.1 | AX144906.1 | DL102491.1 | HI003019.1 | HB841541.1 | DL027151.1 | HI424279.1 |
| GN359734.1 | HV601075.1 | AX587898.1 | AX144874.1 | DL102459.1 | HI002955.1 | HB840939.1 | DL030929.1 | HI424118.1 |
| GN359618.1 | HV574870.1 | AX587799.1 | AX144842.1 | DL102395.1 | HI002920.1 | HB840440.1 | DL027117.1 | HI424086.1 |
| GN359586.1 | HV200506.1 | AX556851.1 | AX144810.1 | DL097622.1 | HI001283.1 | HB650452.1 | DL027085.1 | HI424054.1 |
| GN359422.1 | HV182487.1 | AX556817.1 | AX144778.1 | DL097590.1 | HI001234.1 | HB650232.1 | DL027053.1 | HI423828.1 |
| GN359390.1 | HV182455.1 | AX528974.1 | AX144746.1 | DL097558.1 | HI001184.1 | HB649986.1 | DL027021.1 | HI423796.1 |
| GN365384.1 | HV182391.1 | AX528464.1 | AX144714.1 | DL107087.1 | HI001122.1 | HB649254.1 | DL026989.1 | HI423764.1 |
| GN359125.1 | HV221007.1 | AX523910.1 | AX144682.1 | DL107055.1 | HI001090.1 | HB646994.1 | DL026957.1 | HI422893.1 |
| GN359063.1 | HV038723.1 | AX179477.1 | AX144650.1 | DL106991.1 | HI001034.1 | HB645719.1 | HV040137.1 | HI414053.1 |
| GN346503.1 | HV038681.1 | AX164121.1 | HW321166.1 | DL106959.1 | HI001000.1 | HB645613.1 | HV197640.1 | HI414021.1 |
| GM674293.1 | HV038593.1 | AX521533.1 | HW320838.1 | DL106927.1 | HI000953.1 | HB645426.1 | HV191321.1 | HI413612.1 |
| GM709027.1 | HV182353.1 | AX521501.1 | HW329377.1 | DL106895.1 | HI002856.1 | HB858566.1 | HV182462.1 | HI413576.1 |
| GM708995.1 | HV182321.1 | AX513180.1 | HW320683.1 | DL102190.1 | HI002824.1 | HB856835.1 | HV182366.1 | HI503523.1 |
| GM043988.1 | HV182289.1 | AF430187.1 | HW320447.1 | DL100730.1 | HI002786.1 | HB856023.1 | HV220382.1 | HI516555.1 |
| GM645481.1 | HV182066.1 | AF430155.1 | HW329244.1 | DL096316.1 | HI002726.1 | DM179547.1 | HV220009.1 | HI516096.1 |

| | | | | | | | | |
|---|---|---|---|---|---|---|---|---|
| GM645449.1 | HV228150.1 | AX505263.1 | HW320364.1 | DL096284.1 | HI002679.1 | DM179068.1 | HV117892.1 | HI003153.1 |
| GM645417.1 | HV030222.1 | AX505199.1 | HW320300.1 | DL096252.1 | HI002641.1 | DM178832.1 | HV117760.1 | FW361533.1 |
| GM739600.1 | HV036351.1 | AX505135.1 | HW336130.1 | DL120176.1 | HI002607.1 | DM170828.1 | HV112803.1 | HD122561.1 |
| GM649268.1 | HV035753.1 | AX496854.1 | HW335938.1 | DL120144.1 | HI002560.1 | DM187982.1 | HV116979.1 | HD079713.1 |
| GM649236.1 | FZ435996.1 | BD140613.1 | HW328827.1 | DL120112.1 | HI002494.1 | DM169882.1 | HV038661.1 | FW345544.1 |
| GM649204.1 | FZ435964.1 | BD140002.1 | HW328609.1 | DL120080.1 | HI004573.1 | DM176838.1 | HV038600.1 | FW345148.1 |
| GM045840.1 | FZ430240.1 | BD137989.1 | HW319239.1 | DL120048.1 | HI004509.1 | HB492052.1 | HV038522.1 | FW343704.1 |
| GM647264.1 | FZ429465.1 | HW391379.1 | HW328461.1 | DL120016.1 | HI002334.1 | HB491538.1 | HV182328.1 | FW343510.1 |
| GM647232.1 | FZ437343.1 | HW382148.1 | HW328399.1 | DL094238.1 | HI002293.1 | HB489260.1 | HV182296.1 | HC922350.1 |
| GM647168.1 | FZ423909.1 | HW390565.1 | HW328302.1 | DL094206.1 | HI000501.1 | HB488831.1 | HV187833.1 | HC920501.1 |
| GM646869.1 | FZ436950.1 | HW390512.1 | HW335770.1 | HB844121.1 | DM164037.1 | HB488779.1 | HV234619.1 | HD064836.1 |
| GM646837.1 | HI002394.1 | HW389561.1 | HW335667.1 | HB843549.1 | DM163989.1 | HB488700.1 | HV234232.1 | HD053213.1 |
| GM646805.1 | HI002314.1 | HW389138.1 | HW328243.1 | HB807554.1 | DM163956.1 | DM161010.1 | HV312979.1 | HD033689.1 |
| DL193810.1 | HI000517.1 | HW388626.1 | HW328103.1 | HB818093.1 | DM163286.1 | DM164030.1 | HV302120.1 | HD051874.1 |
| DL193778.1 | HI006854.1 | HW380842.1 | HW318511.1 | HB842363.1 | HB471314.1 | DM167733.1 | HV030300.1 | HD057702.1 |
| AY658552.1 | HI183831.1 | HW368617.1 | HW318376.1 | HB850372.1 | HB468755.1 | DM164015.1 | HV037741.1 | HD057670.1 |
| AY658520.1 | HI180161.1 | HW381037.1 | HW335157.1 | GM645490.1 | DM152589.1 | DM163981.1 | HV037245.1 | HD062030.1 |
| AY658488.1 | HI178195.1 | HW353901.1 | HW314326.1 | GM645458.1 | DM147632.1 | DM163948.1 | HV036229.1 | HC679165.1 |
| AY658456.1 | HI202833.1 | HW352389.1 | HW311850.1 | GM645426.1 | DM074510.1 | DM163540.1 | HI933569.1 | HC678803.1 |
| AY658424.1 | HI214561.1 | HW351468.1 | HW311386.1 | GM638533.1 | DM064152.1 | DM162560.1 | HI930646.1 | HC510438.1 |
| AY658392.1 | HI214529.1 | HW350673.1 | HW309897.1 | GM638501.1 | DM070850.1 | HB483687.1 | HI930265.1 | HC504668.1 |
| AY658360.1 | HI201516.1 | HW350639.1 | HW309504.1 | GM638469.1 | GN091353.1 | HB483614.1 | HI935041.1 | HC494823.1 |
| AY658328.1 | HI202545.1 | HW340227.1 | HW309438.1 | GM633738.1 | GN090913.1 | HB477472.1 | HI949804.1 | HC502380.1 |
| AY658296.1 | HI214431.1 | HW340164.1 | HW309381.1 | GM633706.1 | GN090776.1 | GM655936.1 | HI934079.1 | HC499878.1 |
| AY658264.1 | HI213000.1 | HW339914.1 | HW307878.1 | GM633674.1 | GN089887.1 | GM655904.1 | HI929210.1 | HC499845.1 |
| AY658232.1 | HI212948.1 | HW350249.1 | HW307846.1 | GM625839.1 | GN089822.1 | GM655872.1 | HI918319.1 | FW300639.1 |
| AY658200.1 | HI203499.1 | HW349949.1 | HW307814.1 | GM625807.1 | GN082906.1 | GM655840.1 | HI918269.1 | FW304276.1 |
| AY658168.1 | HI546348.1 | HW348593.1 | HW307782.1 | GM624719.1 | GN082835.1 | GM655808.1 | HI656214.1 | HC487994.1 |
| AY658136.1 | HI546316.1 | HW348493.1 | HW316594.1 | GM624687.1 | GN082359.1 | GM655768.1 | FW503021.1 | HC486537.1 |
| AY658104.1 | HI210994.1 | HW363672.1 | HW307744.1 | GM633669.1 | GN075915.1 | GM648751.1 | FW496482.1 | HC475396.1 |
| AY658072.1 | HI210962.1 | HW363640.1 | HW307712.1 | GM659923.1 | L08959.1 | GM648719.1 | FW504222.1 | HC491774.1 |
| AY658040.1 | HI573197.1 | HW363495.1 | HW307680.1 | GM659891.1 | L08915.1 | GM648687.1 | FW503857.1 | HC491742.1 |
| AY658008.1 | HI566050.1 | HW295862.1 | HW307589.1 | GM659859.1 | M74490.1 | DL096924.1 | FW499104.1 | HC306067.1 |
| AY657976.1 | HI473105.1 | HW302533.1 | HW315742.1 | GM659827.1 | DM059587.1 | DL096892.1 | FW504764.1 | HC306027.1 |
| AY657944.1 | HI472903.1 | HW285973.1 | HW315649.1 | GM659795.1 | DM058881.1 | DL096860.1 | FW504732.1 | HC305987.1 |
| AY657912.1 | HI004365.1 | HW295574.1 | HW315572.1 | GM659927.1 | DM058805.1 | DL096796.1 | FW497146.1 | HC302632.1 |
| AY657880.1 | HI004301.1 | HW295047.1 | HW315340.1 | GM652342.1 | DM048644.1 | DL096764.1 | FW420475.1 | HC305756.1 |
| AY657848.1 | HI004237.1 | HI002650.1 | HW315179.1 | GM652310.1 | DM045299.1 | DL094840.1 | FW420443.1 | HC305375.1 |
| AY657816.1 | HI002272.1 | HI002616.1 | HW315147.1 | GM652278.1 | DM045028.1 | DL094808.1 | FW420411.1 | HC291558.1 |
| AY657784.1 | HI002235.1 | HI002577.1 | HW315083.1 | GM645351.1 | GM601025.1 | DL094776.1 | FW501792.1 | HC291282.1 |
| AY657752.1 | HI002185.1 | HI004655.1 | HW314914.1 | GM645319.1 | FB721280.1 | DL091129.1 | FW501745.1 | HC289361.1 |
| AY657720.1 | HI002138.1 | HI004527.1 | HW314854.1 | GM645287.1 | FB715258.1 | DL091097.1 | FW496939.1 | HC289329.1 |
| AY657688.1 | HI002104.1 | HI004463.1 | HW314659.1 | GM645255.1 | GM060563.1 | DL087097.1 | FW420386.1 | HC292816.1 |
| AY657656.1 | HI000449.1 | HI002424.1 | HW160799.1 | GM645223.1 | DL257743.1 | DL087065.1 | FW420354.1 | HC296215.1 |
| AY657624.1 | HI000401.1 | HI002308.1 | HW106142.1 | GM648736.1 | FB748848.1 | DL087033.1 | FW420322.1 | HC292684.1 |
| AY657592.1 | HI000346.1 | HI000510.1 | HW115438.1 | GM648704.1 | DL236094.1 | DL087001.1 | FW496737.1 | HC295277.1 |
| AY657560.1 | HI553287.1 | HI180155.1 | HW114533.1 | GM648672.1 | DL233425.1 | DL124477.1 | FW496509.1 | HC292134.1 |

| | | | | | | | | |
|---|---|---|---|---|---|---|---|---|
| AY657528.1 | HI553215.1 | HI202827.1 | HW113129.1 | GM648640.1 | DL233392.1 | DL124445.1 | HI653963.1 | HC291954.1 |
| AY657496.1 | HI553183.1 | HI214587.1 | HW112991.1 | GM648608.1 | DL206337.1 | DL124413.1 | HI653878.1 | HC291702.1 |
| AY657464.1 | HI583967.1 | HI214555.1 | GM635599.1 | GM634456.1 | FB713777.1 | DL124381.1 | HI653807.1 | HC293906.1 |
| AY657432.1 | HI568960.1 | HI214523.1 | GM635567.1 | GM634392.1 | FB713713.1 | DL120292.1 | HI653717.1 | HC293726.1 |
| AY657400.1 | HI207660.1 | HI214457.1 | GM635535.1 | GM629469.1 | FB711047.1 | DL120260.1 | HI653635.1 | HC290773.1 |
| AY657368.1 | HI003692.1 | HI214425.1 | GM635503.1 | GM629286.1 | FB708238.1 | DL120228.1 | HI653601.1 | HC293224.1 |
| AY657336.1 | HI002063.1 | HI202304.1 | GM635471.1 | GM629126.1 | CS368356.1 | DL115825.1 | HI653530.1 | HC289967.1 |
| AY657304.1 | HI002019.1 | HI212994.1 | GM626123.1 | GM662969.1 | CS368228.1 | DL115793.1 | HI653418.1 | AX181148.1 |
| AY657272.1 | HI001936.1 | HI546342.1 | GM626091.1 | GM648352.1 | CS368100.1 | DL115761.1 | HI653363.1 | AF394909.1 |
| AY657240.1 | HI000268.1 | HI210988.1 | GM626059.1 | GM648320.1 | CS367972.1 | DL115729.1 | HI653282.1 | AX175387.1 |
| AY657208.1 | HI000223.1 | HI210956.1 | GM626027.1 | GM648288.1 | CS367908.1 | DL115697.1 | FW368390.1 | AX173381.1 |
| AY657176.1 | HI000172.1 | HI566020.1 | GM625963.1 | GM648256.1 | CS367716.1 | DL115665.1 | FW363793.1 | AX172498.1 |
| AY657144.1 | HI000128.1 | HI472897.1 | GM625931.1 | GM648224.1 | CS367588.1 | DL115633.1 | FW363357.1 | AX167419.1 |
| AY657112.1 | HB839691.1 | HI580543.1 | GM037796.1 | GM641389.1 | CS367524.1 | DL111239.1 | FW362967.1 | AX155551.1 |
| AY657080.1 | HB847609.1 | HI209406.1 | FB775670.1 | GM641357.1 | DL080568.1 | DL111207.1 | FW362769.1 | AX148124.1 |
| AY657048.1 | HB847301.1 | HI072381.1 | FB774962.1 | GM641325.1 | DL075911.1 | DL111143.1 | FW362662.1 | AX146808.1 |
| U25666.1 | HB827958.1 | HI004289.1 | FB766242.1 | GM641293.1 | CS588643.1 | DL111111.1 | FW349832.1 | AX145707.1 |
| M22043.1 | HB846838.1 | HI002266.1 | FB764907.1 | GM641261.1 | CS575761.1 | DL111079.1 | FW366342.1 | AX145643.1 |
| K00895.1 | HB838536.1 | HI002229.1 | FB764680.1 | GM629070.1 | CS574934.1 | DL106275.1 | FW366210.1 | AX145579.1 |
| M13242.1 | HB846375.1 | HI002132.1 | FB747071.1 | GM629038.1 | CS574804.1 | DL106243.1 | FW351245.1 | AX145547.1 |
| K00801.1 | HB846076.1 | HI002096.1 | FB744816.1 | GM629006.1 | CS570699.1 | DL106179.1 | FW351186.1 | AX145515.1 |
| K00991.1 | HB826603.1 | HI000435.1 | FB744719.1 | GM880295.1 | CS546674.1 | DL106147.1 | FW360408.1 | AX145483.1 |
| HV549331.1 | HB838045.1 | HI000395.1 | FB763090.1 | GM648191.1 | CS558717.1 | DL106115.1 | HD121599.1 | AX145451.1 |
| HV549097.1 | HB837627.1 | HI000334.1 | FB744006.1 | DL241711.1 | CS560560.1 | DL101406.1 | HD114109.1 | AX145419.1 |
| HV549017.1 | HB845150.1 | HI203426.1 | FB743939.1 | DL241171.1 | DL089489.1 | DL101374.1 | HD113175.1 | AX145386.1 |
| HV544368.1 | HB844880.1 | HI553241.1 | FB743906.1 | DL241026.1 | DL089425.1 | DL101342.1 | HD082720.1 | AX145354.1 |
| HV544172.1 | HB844418.1 | HI553209.1 | FB743874.1 | DL240792.1 | DL089393.1 | DL086990.1 | HD082436.1 | AX145322.1 |
| HV543513.1 | HB844147.1 | HI553177.1 | FB743842.1 | FB674298.1 | DL122566.1 | DL110940.1 | HD088253.1 | AX145290.1 |
| HV538619.1 | HB836502.1 | HC490826.1 | FB743788.1 | DL233402.1 | DL113623.1 | DL110908.1 | HD070445.1 | AX145258.1 |
| HV538463.1 | GM621653.1 | HC490794.1 | FB761762.1 | DL231408.1 | DL113591.1 | DL110876.1 | FW345125.1 | AX145226.1 |
| HV543321.1 | GM621621.1 | HC490762.1 | FB761604.1 | DL206277.1 | DL113559.1 | DL106072.1 | FW344983.1 | AX145194.1 |
| HV543189.1 | GM661712.1 | HC490730.1 | FB742910.1 | FB713819.1 | DL113527.1 | DL106040.1 | FW344849.1 | AX145162.1 |
| HV541504.1 | GM661648.1 | HC490698.1 | FB761493.1 | FB713787.1 | DL113495.1 | DL106008.1 | FW344192.1 | AX145130.1 |
| FW351257.1 | GM661616.1 | HC490537.1 | FB760127.1 | FB713723.1 | DL113463.1 | DL105976.1 | FW344000.1 | AX145098.1 |
| FW361530.1 | GM661584.1 | HC490367.1 | FB708869.1 | FB713689.1 | DL108662.1 | DL105944.1 | FW343682.1 | AX145066.1 |
| HD122294.1 | GM661552.1 | HC490271.1 | GM877102.1 | FB712621.1 | DL108598.1 | DL105676.1 | FW343618.1 | AX145034.1 |
| HD084321.1 | GM661520.1 | HC472291.1 | GM036122.1 | FB674504.1 | DL108566.1 | DL105644.1 | FW343554.1 | AX145002.1 |
| FW345542.1 | GM647277.1 | HC472259.1 | GM952836.1 | FB667083.1 | DL108502.1 | DL105612.1 | FW342974.1 | AX144970.1 |
| FW344991.1 | GM647245.1 | HC472227.1 | GM894239.1 | FB666589.1 | DL103896.1 | DL105580.1 | HC923154.1 | AX144938.1 |
| FW344227.1 | GM647213.1 | HC472195.1 | GM893722.1 | FB704844.1 | DL103864.1 | DL105548.1 | HC922741.1 | AX144905.1 |
| FW343700.1 | GM627615.1 | HC471963.1 | GM890130.1 | FB676593.1 | DL103832.1 | DL105516.1 | HC921635.1 | AX144873.1 |
| FW343570.1 | GM627547.1 | HC471931.1 | GM963585.1 | FB705653.1 | DL103800.1 | DL100903.1 | HC920703.1 | AX144809.1 |
| HC923252.1 | GM714477.1 | HC471899.1 | GM889455.1 | FB705952.1 | DL103768.1 | DL100871.1 | HD068951.1 | AX144777.1 |
| HC922894.1 | GM661488.1 | HC471867.1 | GM889329.1 | FB701815.1 | DL103736.1 | DL100839.1 | HD068770.1 | AX144745.1 |
| HC920497.1 | GM661456.1 | HC678821.1 | GM888717.1 | FB707076.1 | DL103713.1 | DL100807.1 | HC729199.1 | AX144681.1 |
| HD067661.1 | GM661424.1 | HC678755.1 | GM008873.1 | FB701005.1 | DL099226.1 | DL100775.1 | HC728996.1 | AX144649.1 |
| HD051867.1 | GM661392.1 | HC511349.1 | GM888521.1 | FB580681.1 | DL099194.1 | DL100743.1 | DL106208.1 | AX144617.1 |

| | | | | | | | | |
|---|---|---|---|---|---|---|---|---|
| HD057700.1 | GM661360.1 | HC504629.1 | GM008193.1 | DL128529.1 | DL099162.1 | DL096329.1 | DL106176.1 | AX144557.1 |
| HD057668.1 | GM661328.1 | HC504260.1 | GM872375.1 | DL128497.1 | DL093152.1 | DL096297.1 | DL101403.1 | AX144365.1 |
| DM380123.1 | GM654334.1 | HC471036.1 | GM006311.1 | DL128461.1 | DL093120.1 | DL096233.1 | DL101371.1 | AX144107.1 |
| HC025933.1 | GM654302.1 | HC500968.1 | GM005940.1 | DL120245.1 | DL093088.1 | DL096201.1 | DL101339.1 | AX144043.1 |
| HC025515.1 | GM654270.1 | HC499854.1 | GM867495.1 | DL120213.1 | DL093056.1 | DL096169.1 | DL106069.1 | AX143913.1 |
| HC025483.1 | GM654238.1 | FW301302.1 | FB984709.1 | DL115810.1 | DL093024.1 | DL120189.1 | DL106037.1 | AX143849.1 |
| HC025451.1 | GM647181.1 | FW300531.1 | FB983621.1 | DL115778.1 | DL092992.1 | DL120157.1 | DL106005.1 | AX143785.1 |
| HB847771.1 | GM647149.1 | FW298759.1 | FB983582.1 | DL101487.1 | DL092960.1 | DL120125.1 | DL105973.1 | AX143721.1 |
| HB839491.1 | GM647117.1 | HC486491.1 | FB983219.1 | DL101220.1 | DL089345.1 | DL120093.1 | DL105909.1 | AX143657.1 |
| HB838853.1 | GM647085.1 | HC485142.1 | GM682680.1 | DL101188.1 | DL089313.1 | DL120061.1 | DL101296.1 | AX143529.1 |
| HB805431.1 | GM647077.1 | HC474871.1 | FB983167.1 | DL104958.1 | DL089281.1 | DL120029.1 | DL101264.1 | AX143401.1 |
| HB847032.1 | GM627511.1 | HC037088.1 | GM681758.1 | DL104926.1 | DL089249.1 | DL094546.1 | DL101232.1 | AX143337.1 |
| HB838742.1 | GM838923.1 | HC025526.1 | FB753635.1 | DL124133.1 | DL089185.1 | DL094315.1 | DL101200.1 | AX143273.1 |
| HB838156.1 | GM661295.1 | HC025494.1 | GM680849.1 | DL124101.1 | DL125940.1 | DL094283.1 | DL101168.1 | AX143209.1 |
| HB846179.1 | GM654131.1 | HC025462.1 | GM865388.1 | DL119481.1 | DL125908.1 | DL094251.1 | DL101136.1 | AX143145.1 |
| HB837835.1 | GM654099.1 | HC025430.1 | GM968366.1 | DL119449.1 | DL125876.1 | DL094219.1 | DL096722.1 | AX142761.1 |
| HB845500.1 | GM654067.1 | HC025398.1 | GM618794.1 | GN346572.1 | DL125844.1 | DL094187.1 | DL096690.1 | AX142569.1 |
| HB837333.1 | GM654035.1 | HC010665.1 | FB726125.1 | GN346540.1 | DL125812.1 | DL094155.1 | DL096658.1 | AX142439.1 |
| HB845245.1 | GM654003.1 | HC010309.1 | FB725964.1 | GN116519.1 | DL125780.1 | DL090540.1 | DL096626.1 | AX142375.1 |
| HB844594.1 | GM653971.1 | HC009027.1 | FB724508.1 | GN116487.1 | DL144197.1 | DL090508.1 | DL096594.1 | AX142183.1 |
| HB844340.1 | GM646946.1 | HC007668.1 | GM731776.1 | GN116454.1 | DL122538.1 | DL090472.1 | DL096562.1 | AX142119.1 |
| HW375257.1 | GM646914.1 | HC010677.1 | FB711630.1 | GN116422.1 | DL122506.1 | DL090440.1 | DL094734.1 | AX141989.1 |
| HW375074.1 | GM646882.1 | HC005709.1 | DL106394.1 | GN116390.1 | DL122474.1 | DL090408.1 | DL094702.1 | HW355240.1 |
| HW375049.1 | GM646850.1 | HC005047.1 | DL106338.1 | DM075118.1 | DL122410.1 | DL090376.1 | DL094670.1 | HW344777.1 |
| HW374883.1 | GM646818.1 | DM370684.1 | DL106306.1 | DM064177.1 | DL122378.1 | DL086371.1 | DL090959.1 | HW344704.1 |
| HW374644.1 | DL111536.1 | DM370652.1 | DL136024.1 | DM077789.1 | DL118124.1 | DL086339.1 | DL090927.1 | HW344753.1 |
| HW374470.1 | DL111504.1 | DM370620.1 | DL128514.1 | DM077436.1 | DL118092.1 | DL086307.1 | DL090895.1 | HW344558.1 |
| FW348491.1 | DL101690.1 | DM370588.1 | DL128481.1 | GM712537.1 | DL118060.1 | DL086243.1 | DL086959.1 | HW344731.1 |
| HW381358.1 | DL101658.1 | DM370556.1 | DL124751.1 | GM745631.1 | DL118028.1 | DL086211.1 | DL086927.1 | HW344507.1 |
| HW381053.1 | DL101626.1 | DM370524.1 | DL124719.1 | GM711150.1 | DL113435.1 | DL110454.1 | DL086895.1 | HW326522.1 |
| HW381021.1 | DL097116.1 | DM370492.1 | DL124687.1 | FB509201.1 | DL113403.1 | DL110422.1 | DL086863.1 | HW326430.1 |
| HW344418.1 | DL097084.1 | DM370460.1 | DL124655.1 | FB509133.1 | DL113371.1 | DL110390.1 | DL086831.1 | HW326325.1 |
| HW344354.1 | DL097052.1 | HC003079.1 | DL124623.1 | GM709160.1 | DL113339.1 | DL110358.1 | DL086799.1 | HW326284.1 |
| HW344322.1 | DL106654.1 | HB865042.1 | DL124591.1 | GM709002.1 | DL113275.1 | DL110326.1 | DL086767.1 | HW338962.1 |
| HW344290.1 | DL106558.1 | HB864962.1 | DL124559.1 | GM655031.1 | DL108442.1 | DL110294.1 | DL086735.1 | HW338890.1 |
| HW344258.1 | DL106526.1 | HB865002.1 | DL096926.1 | GM654999.1 | DL108410.1 | DL105489.1 | DL086703.1 | HW338634.1 |
| HW353886.1 | DL128691.1 | HB864946.1 | DL096894.1 | GM694756.1 | DL108402.1 | DL105457.1 | DL086671.1 | HW338506.1 |
| HW353854.1 | DL128623.1 | HB864914.1 | DL096862.1 | GM755056.1 | DL108346.1 | DL105425.1 | DL086639.1 | HW338378.1 |
| HW353822.1 | DL128591.1 | HB864882.1 | DL096830.1 | DL480712.1 | DL108314.1 | DL009455.1 | DL086607.1 | HW338250.1 |
| HW353790.1 | DL128537.1 | HB864850.1 | DL096798.1 | GM652742.1 | DL103644.1 | DL030148.1 | DL105880.1 | HW338122.1 |
| HW353758.1 | DL125093.1 | HB864818.1 | DL096766.1 | GM652710.1 | DL103580.1 | DL026339.1 | DL105848.1 | HW337994.1 |
| HW353726.1 | DL125061.1 | HB864786.1 | DL094938.1 | GM630646.1 | DL103548.1 | DL026275.1 | DL105816.1 | HW337866.1 |
| HW344181.1 | DL125029.1 | HB864754.1 | DL094906.1 | GM630614.1 | DL103516.1 | DL026243.1 | DL114880.1 | HW337738.1 |
| HW344149.1 | DL124997.1 | HB859735.1 | DL094874.1 | GM630582.1 | DL099102.1 | DL026179.1 | DL114848.1 | HW337610.1 |
| HW344117.1 | DL124965.1 | HB866933.1 | DL094842.1 | GM621647.1 | DL099070.1 | DL018989.1 | DL127348.1 | HW337482.1 |
| HW344053.1 | DL124950.1 | HB866545.1 | DL094810.1 | GM621615.1 | DL099038.1 | DL018957.1 | DL123928.1 | HW337354.1 |
| HW353637.1 | DL124918.1 | HC000339.1 | DL094778.1 | GM621583.1 | DL099006.1 | DL018925.1 | DL123896.1 | HW337226.1 |

| | | | | | | | | |
|---|---|---|---|---|---|---|---|---|
| HW353605.1 | DL116170.1 | HB999695.1 | DL094746.1 | GM621551.1 | DL098974.1 | DL018893.1 | DL123864.1 | HW337098.1 |
| HW353573.1 | DL116138.1 | HB977615.1 | DL091131.1 | GM657131.1 | DL098942.1 | DL018861.1 | DL123832.1 | HW321932.1 |
| HW344028.1 | DL116106.1 | HB977093.1 | DL091099.1 | GM656908.1 | DL092932.1 | DL018829.1 | DL123800.1 | HW336970.1 |
| HW353474.1 | DL116074.1 | HB976704.1 | DL091067.1 | GM656876.1 | DL092900.1 | DL034336.1 | DL123768.1 | HW336842.1 |
| HW339232.1 | DL116042.1 | GM659922.1 | DL091035.1 | GM656844.1 | DL092868.1 | DL018799.1 | DL119589.1 | HW336686.1 |
| HW352821.1 | DL120566.1 | GM659890.1 | DL090971.1 | GM642723.1 | DL092836.1 | DL014304.1 | DL119557.1 | HW321273.1 |
| HW352757.1 | DL120534.1 | GM659858.1 | DL087099.1 | GM656460.1 | DL092804.1 | DL014272.1 | DL119525.1 | HW336478.1 |
| HW352661.1 | DL115907.1 | GM659826.1 | DL087067.1 | GM656428.1 | DL092772.1 | DL014240.1 | DL119493.1 | HW321165.1 |
| HW352629.1 | DL115875.1 | GM659794.1 | DL044153.1 | GM649331.1 | DL089157.1 | DL009356.1 | DL119461.1 | HW320836.1 |
| HW352565.1 | DL115843.1 | GM659926.1 | DL044121.1 | GM649275.1 | DL089125.1 | DL009324.1 | DL119429.1 | HW329376.1 |
| HW352469.1 | DL124674.1 | GM652341.1 | DL044089.1 | GM649243.1 | DL089093.1 | DL009292.1 | DL123740.1 | HW329243.1 |
| HW352341.1 | DL107960.1 | GM652309.1 | DL071612.1 | GM649211.1 | DL089061.1 | DL009260.1 | DL123708.1 | HW336370.1 |
| HW352277.1 | DL107928.1 | GM652277.1 | DL048226.1 | GM635131.1 | DL089029.1 | DL009228.1 | DL123676.1 | HW336129.1 |
| HW341541.1 | DL107896.1 | GM645382.1 | DL048194.1 | GM646348.1 | DL033047.1 | DL018686.1 | DL123644.1 | HV568024.1 |
| HW341303.1 | DL088599.1 | GM645350.1 | DL048162.1 | GM646316.1 | DL033015.1 | DL018654.1 | DL123612.1 | HV567992.1 |
| HW351626.1 | DL113041.1 | GM645318.1 | DL048130.1 | GM639385.1 | DL032983.1 | DL018622.1 | DL123580.1 | HV567904.1 |
| HW351594.1 | DL092356.1 | GM645286.1 | DL046578.1 | GM639353.1 | DL032951.1 | DL009074.1 | DL119390.1 | HV566998.1 |
| HW341246.1 | DL092324.1 | GM645254.1 | DL046546.1 | GM626710.1 | DL032919.1 | DL009042.1 | DL119358.1 | HV566115.1 |
| HW351484.1 | DL092292.1 | GM645222.1 | DL046514.1 | GM646081.1 | DL028751.1 | DL014226.1 | DL119326.1 | HV565825.1 |
| HW351420.1 | DL092260.1 | GM652112.1 | DL046482.1 | GM646049.1 | DL024937.1 | DL014194.1 | DL114834.1 | HV573508.1 |
| HW351289.1 | DL092228.1 | GM645189.1 | DL042660.1 | GM646017.1 | DL024905.1 | DL014162.1 | DL114802.1 | HV572439.1 |
| HV701108.1 | DL092196.1 | GM638134.1 | DL042628.1 | GM645930.1 | DL024873.1 | DL014130.1 | DL114770.1 | HV572129.1 |
| HV701076.1 | DL092164.1 | GM638102.1 | DL042596.1 | GM638999.1 | DL024841.1 | DL014098.1 | DL114738.1 | HV571426.1 |
| HV701044.1 | DL088421.1 | GM638070.1 | DL042564.1 | GM752371.1 | DL024809.1 | DL014066.1 | DL114706.1 | HV558757.1 |
| HV695538.1 | DL088389.1 | GM633364.1 | DL042532.1 | DL241169.1 | DL024777.1 | DL014034.1 | DL114674.1 | HV555476.1 |
| HV695474.1 | DL129178.1 | GM640840.1 | DL042500.1 | DL241024.1 | DL021565.1 | DL026124.1 | DL127932.1 | HV190571.1 |
| HV695336.1 | DL121885.1 | GM047096.1 | DL038672.1 | FB674296.1 | DL021533.1 | DL026092.1 | DL127150.1 | HV202314.1 |
| HV695297.1 | DL121853.1 | GM654817.1 | DL038640.1 | DL236215.1 | DL021501.1 | DL026060.1 | DL127021.1 | HV187608.1 |
| HV694993.1 | DL121821.1 | GM654785.1 | DL038608.1 | DL231400.1 | DL021469.1 | DL026028.1 | DL141493.1 | HV225538.1 |
| HV694929.1 | DL121789.1 | GM647760.1 | DL034303.1 | DL206273.1 | DL021437.1 | DL025996.1 | DL123540.1 | HV234373.1 |
| HV694787.1 | DL041079.1 | GM647696.1 | DL034271.1 | FB713785.1 | DL044345.1 | DL025964.1 | DL123508.1 | HV234304.1 |
| HV700708.1 | DL037128.1 | GM627958.1 | DL034239.1 | FB713721.1 | DL044313.1 | DL018541.1 | DL123476.1 | HV302191.1 |
| HV694688.1 | DL028661.1 | GM627926.1 | DL034207.1 | FB713671.1 | DL044281.1 | DL018509.1 | DL123444.1 | HV312992.1 |
| HV694549.1 | DL028629.1 | GM715072.1 | DL034175.1 | FB712619.1 | DL032112.1 | DL018477.1 | DL123412.1 | HV120331.1 |
| HV700637.1 | DL028597.1 | GM654742.1 | DL034143.1 | FB674502.1 | DL032080.1 | DL018445.1 | DL123380.1 | HV037714.1 |
| HV694226.1 | GM638504.1 | GM647428.1 | DL034111.1 | FB671078.1 | DL032048.1 | DL018413.1 | DL119094.1 | HV036672.1 |
| HV700233.1 | GM638472.1 | GM647240.1 | DL029914.1 | FB667612.1 | DL032016.1 | DL014011.1 | DL119062.1 | HV036299.1 |
| HV693213.1 | GM633741.1 | GM647208.1 | DL029850.1 | FB667079.1 | DL031984.1 | DL013979.1 | DL119030.1 | HV035182.1 |
| HV699164.1 | GM633709.1 | FB713818.1 | DL029818.1 | FB704839.1 | DL031952.1 | DL013947.1 | DL114634.1 | FZ435976.1 |
| HV699094.1 | GM633677.1 | FB713722.1 | DL029786.1 | FB676591.1 | DL028143.1 | DL013915.1 | DL114602.1 | FZ435939.1 |
| HV698570.1 | GM625842.1 | FB713672.1 | DL029754.1 | FB676559.1 | DL028111.1 | DL013883.1 | DL114570.1 | FZ422122.1 |
| HV690933.1 | GM625810.1 | FB712620.1 | DL025944.1 | FB705925.1 | DL028079.1 | DL013847.1 | DL114538.1 | FZ435655.1 |
| HV698289.1 | GM625778.1 | FB674503.1 | DL025912.1 | FB701813.1 | DL028047.1 | DL030111.1 | DL114506.1 | FZ434959.1 |
| HV698241.1 | GM625746.1 | FB667081.1 | DL025880.1 | FB701003.1 | DL028015.1 | DJ355102.1 | DL114474.1 | FZ426104.1 |
| HV698182.1 | GM624658.1 | FB666588.1 | DL025848.1 | DL120275.1 | DL027983.1 | DJ354938.1 | DL109577.1 | FZ429561.1 |
| HV698118.1 | GM625606.1 | FB704841.1 | DL025816.1 | DL120243.1 | DL027951.1 | DJ361248.1 | DL109545.1 | FZ429271.1 |
| HV697914.1 | GM642992.1 | FB676592.1 | DL025784.1 | DL120211.1 | DL036461.1 | DJ361214.1 | DL109513.1 | FZ437403.1 |

| | | | | | | | | |
|---|---|---|---|---|---|---|---|---|
| HV689278.1 | GM642959.1 | FB676560.1 | DL069495.1 | DL094234.1 | DL036429.1 | DJ354100.1 | DL104875.1 | FZ437356.1 |
| HV695630.1 | GM642927.1 | FB705951.1 | DL018387.1 | DL094202.1 | DL036397.1 | DJ339861.1 | DL104843.1 | FZ436704.1 |
| HV695598.1 | GM630651.1 | FB701814.1 | DL018355.1 | DL094170.1 | DL036365.1 | DJ339797.1 | DL104811.1 | FZ423463.1 |
| HV688838.1 | GM630619.1 | FB701004.1 | DL018323.1 | DL090487.1 | DL044248.1 | DJ339765.1 | DL095920.1 | FZ423224.1 |
| HV600545.1 | GM630587.1 | FB580679.1 | DL018291.1 | DL086290.1 | DL044216.1 | DJ339733.1 | DL095888.1 | FZ431986.1 |
| HV585542.1 | GM621652.1 | FB654405.1 | DL018259.1 | DL086258.1 | DL044184.1 | DJ339701.1 | DL095856.1 | FZ422784.1 |
| HV593586.1 | GM621620.1 | FB654862.1 | DL018227.1 | DL086226.1 | DL044152.1 | DJ339669.1 | DL095824.1 | FZ416439.1 |
| HV579924.1 | GM621588.1 | FB652952.1 | DL013825.1 | DL110160.1 | DL044120.1 | DJ339604.1 | DL095792.1 | FZ419485.1 |
| HV579863.1 | GM621556.1 | FB573757.1 | DL013793.1 | DL110128.1 | DL044088.1 | DJ339572.1 | DL095760.1 | FZ418824.1 |
| HV600394.1 | GM657136.1 | DL119480.1 | DL013761.1 | DL110096.1 | DL071611.1 | DJ339540.1 | DL094137.1 | FZ421985.1 |
| HV585134.1 | GM657104.1 | DL119448.1 | DL013729.1 | DL105163.1 | DL048225.1 | DJ333883.1 | DL094105.1 | FZ415425.1 |
| HV585102.1 | GM657072.1 | DL119416.1 | DL013697.1 | DL105131.1 | DL048193.1 | DJ329296.1 | DL094073.1 | FZ421400.1 |
| HV585002.1 | GM657040.1 | DL123663.1 | DL013665.1 | DL119947.1 | DL048161.1 | DJ328782.1 | DL090266.1 | FZ421220.1 |
| HV579277.1 | GM657008.1 | DL114821.1 | DL000100.1 | DL119915.1 | DL048129.1 | DJ327055.1 | DL090234.1 | FZ415247.1 |
| HV579090.1 | GM656913.1 | DL114789.1 | DL000686.1 | DL119883.1 | DL048097.1 | DJ327023.1 | DL090202.1 | FZ421008.1 |
| HV588591.1 | GM656881.1 | DL114757.1 | DL000612.1 | DL119851.1 | DL048065.1 | DJ326990.1 | DL090170.1 | FZ420813.1 |
| HV578983.1 | GM656849.1 | DL114725.1 | DL000548.1 | DL119819.1 | DL048034.1 | DJ326630.1 | DL086165.1 | FZ420748.1 |
| HV592611.1 | GM656817.1 | DL114693.1 | DL005239.1 | DL115128.1 | DL048002.1 | DJ122514.1 | DL086133.1 | FZ414806.1 |
| HV592536.1 | GM656785.1 | DL114661.1 | DJ493836.1 | DL110064.1 | DL047970.1 | DJ122008.1 | DL086101.1 | HI377254.1 |
| HV592431.1 | GM656753.1 | DL127301.1 | DJ492714.1 | DL110032.1 | DL047938.1 | DJ088993.1 | DL086069.1 | HI376817.1 |
| HV578497.1 | GM649600.1 | DL141461.1 | DL003041.1 | DL110000.1 | DL047906.1 | DJ082645.1 | DL025356.1 | HI375898.1 |
| HV591574.1 | GM635418.1 | DL123463.1 | DL002942.1 | DL109968.1 | DL047874.1 | DJ080931.1 | DL025324.1 | HI375664.1 |
| HV598626.1 | GM630484.1 | DL123431.1 | DJ491598.1 | DL028655.1 | DL044020.1 | DJ081435.1 | DL025292.1 | HI375534.1 |
| HV586973.1 | GM621549.1 | DL123399.1 | DJ491426.1 | DL028623.1 | DL043988.1 | DJ079782.1 | DL025228.1 | HI373895.1 |
| HV582316.1 | GM621485.1 | DL123367.1 | DJ491070.1 | DL028591.1 | DL043956.1 | DJ071526.1 | DL021952.1 | HI373101.1 |
| HV344347.1 | FB505209.1 | DL119113.1 | DJ446833.1 | DL028559.1 | DL043924.1 | DJ066388.1 | DL021920.1 | HI370486.1 |
| HV344309.1 | GM009365.1 | DL119081.1 | DJ474321.1 | DL048678.1 | DL043892.1 | DJ066356.1 | DL021856.1 | HI369128.1 |
| GM650849.1 | GM008850.1 | DL119049.1 | DJ442350.1 | DL047952.1 | DL040075.1 | DJ066324.1 | DL021824.1 | HI369085.1 |
| GM650785.1 | GM007126.1 | DL119017.1 | DJ437328.1 | DL040057.1 | DL040043.1 | DJ066290.1 | DL021792.1 | HI369029.1 |
| GM658120.1 | GM867481.1 | DL114621.1 | DJ437280.1 | DL040025.1 | DL040011.1 | DJ066256.1 | DL017426.1 | HI424145.1 |
| GM658088.1 | FB983185.1 | DL114589.1 | DJ437152.1 | DL039993.1 | DL039979.1 | DJ065984.1 | DL038462.1 | HI424113.1 |
| GM658056.1 | FB727103.1 | DL114557.1 | DJ437115.1 | DL043670.1 | DL039947.1 | DJ068025.1 | DL038430.1 | HI424081.1 |
| GM658024.1 | GM864065.1 | DL114525.1 | DJ437083.1 | DL027840.1 | DL039915.1 | DJ067042.1 | DL038398.1 | HI423791.1 |
| GM657992.1 | FB725751.1 | DL114493.1 | DJ436997.1 | DL027808.1 | DL036195.1 | DJ061649.1 | DL038366.1 | HI423358.1 |
| GM657960.1 | GM616086.1 | DL114461.1 | DJ436770.1 | DL027776.1 | DL036163.1 | DJ061505.1 | DL038334.1 | HI416367.1 |
| GM650768.1 | GM879662.1 | DL104894.1 | DJ440917.1 | DL023868.1 | DL036131.1 | DJ061290.1 | DL013171.1 | HI416163.1 |
| GM650736.1 | GM723330.1 | DL104830.1 | DJ431134.1 | DL023836.1 | DL036099.1 | DJ055291.1 | DL013139.1 | HI415922.1 |
| GM650704.1 | FB712050.1 | DL095907.1 | DJ444575.1 | DL023804.1 | DL043866.1 | DJ061128.1 | DL013107.1 | HI415890.1 |
| GM650672.1 | FB709962.1 | DL095875.1 | DJ439638.1 | DJ339844.1 | DL047830.1 | DJ057712.1 | DL013075.1 | HI415858.1 |
| GM650640.1 | GM041771.1 | DL137866.1 | DJ434400.1 | DJ339812.1 | DL047798.1 | DJ060413.1 | DL013043.1 | HI415825.1 |
| GM650608.1 | GM840186.1 | DL114426.1 | CS459116.1 | DJ339780.1 | DL047766.1 | DJ054278.1 | DL013011.1 | HI415761.1 |
| GM643808.1 | FB709153.1 | DL114394.1 | DD361297.1 | DJ339748.1 | DL047734.1 | DJ060348.1 | DL033906.1 | HI414048.1 |
| GM643776.1 | CS728674.1 | DL114362.1 | DD361265.1 | DJ339652.1 | DL047702.1 | DJ060306.1 | DL033874.1 | HI414016.1 |
| GM643712.1 | CS728610.1 | DL009372.1 | DD367342.1 | DJ339587.1 | DL047670.1 | DJ060272.1 | DL033842.1 | HI413607.1 |
| GM643680.1 | CS728578.1 | DL009340.1 | DD362924.1 | DJ339555.1 | DL043720.1 | DD401842.1 | DL033810.1 | HI413571.1 |
| GM643640.1 | CS727215.1 | DL009308.1 | DD362380.1 | DJ331380.1 | DL043688.1 | DD401810.1 | DL033778.1 | HI412384.1 |
| GM636651.1 | GM036648.1 | DL009244.1 | DD370038.1 | DJ327303.1 | DL039871.1 | DD401746.1 | DL033746.1 | HI410973.1 |

| | | | | | | | | |
|---|---|---|---|---|---|---|---|---|
| GM636619.1 | GM836387.1 | DL018702.1 | DD368056.1 | DJ122190.1 | DL039839.1 | DD401714.1 | DL033714.1 | HI410849.1 |
| GM636587.1 | GM888608.1 | DL018670.1 | DD361299.1 | DJ082716.1 | DL039807.1 | DD401682.1 | DL042148.1 | HI503518.1 |
| GM636555.1 | GM007133.1 | AF042785.1 | CS457871.1 | DJ082664.1 | DL039775.1 | DD401650.1 | DL042116.1 | HI541344.1 |
| GM636523.1 | GM868792.1 | HC452133.1 | CS457747.1 | DJ084886.1 | DL039743.1 | DD405396.1 | DL042084.1 | HI516091.1 |
| GM636491.1 | GM680774.1 | HC449837.1 | CS457296.1 | DJ080726.1 | DL039711.1 | DD405364.1 | DL046271.1 | HI541096.1 |
| GM636459.1 | GM879316.1 | FU756126.1 | CS457162.1 | DJ080507.1 | DL036087.1 | DD405332.1 | DL033705.1 | HI284310.1 |
| GM632051.1 | GM618696.1 | FU773356.1 | CS456710.1 | DJ069481.1 | DL034609.1 | DD405300.1 | DL033673.1 | HI284278.1 |
| GM632019.1 | GM840929.1 | FU759934.1 | CS455665.1 | DJ066404.1 | DL034577.1 | DD405268.1 | DL033641.1 | HI283482.1 |
| GM631987.1 | GM721268.1 | FU764208.1 | CS453693.1 | DJ066371.1 | DL034545.1 | DD401634.1 | DL029537.1 | HI574531.1 |
| GM631955.1 | GM841715.1 | FU757783.1 | CS450927.1 | DJ066339.1 | DL034513.1 | DD401570.1 | DL029505.1 | HI574499.1 |
| GM631923.1 | DL116073.1 | FU757299.1 | CS450619.1 | DJ066306.1 | DL030389.1 | DD401506.1 | DL029473.1 | HI574467.1 |
| GM631891.1 | DL116041.1 | HC441396.1 | CS450542.1 | DJ066272.1 | DL026532.1 | DD401474.1 | DL029441.1 | HI574403.1 |
| GM623120.1 | DL120595.1 | HC359262.1 | DD361046.1 | DJ066238.1 | DL026476.1 | CS480734.1 | DL029409.1 | HI571467.1 |
| GM623088.1 | DL120565.1 | HC358400.1 | DD353947.1 | DJ061576.1 | DL026444.1 | CS476471.1 | DL029377.1 | HI283370.1 |
| GM623056.1 | DL115906.1 | HC358212.1 | AX017672.1 | DJ061407.1 | DL026412.1 | CS475818.1 | DL029345.1 | HI578560.1 |
| GM623024.1 | DL115874.1 | HC358081.1 | AX014756.1 | DJ061342.1 | DL026380.1 | CS469222.1 | DL046107.1 | HI573737.1 |
| GM622992.1 | DL115842.1 | A00765.1 | AX002762.1 | DJ055187.1 | DL022568.1 | CS467514.1 | DL046075.1 | HI573577.1 |
| GM622960.1 | DL136134.1 | HC324490.1 | AX011485.1 | DJ060185.1 | DL022536.1 | CS464635.1 | DL041961.1 | HI566230.1 |
| GM650574.1 | DL128464.1 | GN033314.1 | AX011453.1 | CS417330.1 | DL022496.1 | CS464703.1 | DL041929.1 | FW375263.1 |
| GM650542.1 | DL128431.1 | GN033282.1 | AX011421.1 | CS416938.1 | DL022440.1 | DD392051.1 | DL041897.1 | HC046653.1 |
| GM650510.1 | DL124737.1 | GN033250.1 | AX011388.1 | CS410937.1 | DL022408.1 | DD392053.1 | DL038010.1 | HC046621.1 |
| GM650478.1 | DL124673.1 | GN033218.1 | AX011356.1 | CS410884.1 | DL019393.1 | DD391745.1 | DJ326987.1 | HC046589.1 |
| GM650446.1 | DL124641.1 | GN033186.1 | AX011324.1 | CS414824.1 | DL019361.1 | DD391855.1 | DJ326601.1 | HC046557.1 |
| GM650414.1 | DL124609.1 | GN033154.1 | AX010960.1 | DD327644.1 | DL019329.1 | DD382840.1 | DJ128272.1 | HC046525.1 |
| GM643606.1 | DL124577.1 | GN033058.1 | AX010384.1 | DD327015.1 | DL019297.1 | DD400164.1 | DJ122501.1 | HC046493.1 |
| GM643574.1 | DL096912.1 | GN033026.1 | AX009700.1 | DD329888.1 | DL019265.1 | BD263427.1 | DJ088990.1 | HC046461.1 |
| GM643542.1 | DL096880.1 | GN032994.1 | AX007118.1 | CS389260.1 | DL019233.1 | BD263345.1 | DJ088497.1 | HC046429.1 |
| GM643510.1 | DL096848.1 | GN032962.1 | AX006472.1 | CS389214.1 | DL019201.1 | BD262205.1 | DJ086527.1 | HC046397.1 |
| GM643478.1 | DL120280.1 | GN032930.1 | AX004799.1 | CS389176.1 | DL014655.1 | BD251477.1 | DJ082694.1 | HC046365.1 |
| GM643446.1 | DL120248.1 | GN032898.1 | AX003195.1 | CS399239.1 | DL010180.1 | BD251240.1 | DJ081981.1 | HC046333.1 |
| GM643413.1 | DL120216.1 | GN032866.1 | AX002975.1 | CS408808.1 | DL010148.1 | BD249281.1 | DJ086710.1 | HC046082.1 |
| GM636424.1 | DL115813.1 | GN032834.1 | A76773.1 | CS402567.1 | DL010116.1 | BD247116.1 | DJ080259.1 | HC046050.1 |
| GM636392.1 | DL093927.1 | GN032802.1 | U34759.1 | CS401813.1 | DL010084.1 | BD246860.1 | DJ081430.1 | HC046018.1 |
| GM636360.1 | DL093895.1 | GN032770.1 | HW163493.1 | CS376531.1 | DL010052.1 | BD243450.1 | DJ066781.1 | HC046290.1 |
| GM636328.1 | DL141056.1 | GN032739.1 | HW163438.1 | CS374394.1 | DL010020.1 | BD243414.1 | DJ071359.1 | HC046258.1 |
| GM636264.1 | DL123343.1 | GN032707.1 | HW161249.1 | DD308881.1 | DL014618.1 | BD242464.1 | DJ066385.1 | HC046226.1 |
| GM631856.1 | DL123311.1 | GN032675.1 | HW160828.1 | DD298329.1 | DD213820.1 | BD238484.1 | DJ066320.1 | HC046194.1 |
| GM631824.1 | DL109153.1 | GN032610.1 | HW160634.1 | DD307926.1 | DD216453.1 | BD237510.1 | DJ066287.1 | HC046162.1 |
| GM631792.1 | DL109121.1 | GN032578.1 | HW160602.1 | DD292503.1 | DD216421.1 | BD235825.1 | DJ066253.1 | HC046130.1 |
| GM631760.1 | DL109089.1 | GN032547.1 | HW155692.1 | DD291066.1 | DD213771.1 | BD235375.1 | DJ068021.1 | HC046098.1 |
| GM631728.1 | DL104355.1 | GN032483.1 | HW155655.1 | CS365220.1 | DD213739.1 | BD234881.1 | DJ067013.1 | HC045980.1 |
| GM631696.1 | AX490813.1 | GN032419.1 | HW150141.1 | CS376404.1 | DD213707.1 | BD234569.1 | DJ061645.1 | HC045948.1 |
| GM622925.1 | AX474390.1 | GN032387.1 | HW150045.1 | CS363420.1 | DD213675.1 | BD234389.1 | DJ055584.1 | HC045884.1 |
| GM622893.1 | AF458106.1 | GN032354.1 | HW149981.1 | CS362627.1 | DD213643.1 | BD232106.1 | DJ061236.1 | HC045852.1 |
| GM622861.1 | AX468382.1 | GN032322.1 | HW144829.1 | CS359731.1 | DD213611.1 | BD231946.1 | DJ057691.1 | HC045820.1 |
| GM622829.1 | AX468161.1 | GN032290.1 | HW155206.1 | CS359589.1 | DD220744.1 | BD231218.1 | DJ060410.1 | HC045788.1 |
| GM622797.1 | AX463621.1 | GN032258.1 | HW158883.1 | BD269164.1 | DD213593.1 | BD231061.1 | DJ056889.1 | HC045756.1 |

| | | | | | | | | |
|---|---|---|---|---|---|---|---|---|
| GM622765.1 | AX458629.1 | GN032226.1 | HW158757.1 | BD269132.1 | DD213561.1 | BD227580.1 | DJ053215.1 | HC045724.1 |
| GM657914.1 | AX458479.1 | GN032162.1 | HW158387.1 | BD268153.1 | DD216041.1 | BD226448.1 | DJ053083.1 | HC045694.1 |
| GM657850.1 | AX456482.1 | GN032130.1 | HW154651.1 | BD267536.1 | DD216009.1 | BD225790.1 | DJ052885.1 | HC045630.1 |
| GM657818.1 | AX455512.1 | GN032098.1 | HW154484.1 | DD192519.1 | DD215977.1 | BD225758.1 | CS810639.1 | HC045598.1 |
| GM657785.1 | AX454127.1 | GN032034.1 | HW154452.1 | DD182413.1 | DD215931.1 | BD225726.1 | CS810533.1 | HC045566.1 |
| GM657753.1 | AX451980.1 | GN032002.1 | HW154409.1 | DD206910.1 | DD215899.1 | BD225694.1 | CS811058.1 | HC045534.1 |
| GM643395.1 | AX451340.1 | GN031970.1 | HW154354.1 | DD206878.1 | DD215867.1 | BD225662.1 | DJ020648.1 | DM380119.1 |
| GM627593.1 | A34193.1 | GN031938.1 | HW154318.1 | DD196874.1 | DD215835.1 | BD225630.1 | DJ017820.1 | HC035740.1 |
| GM627561.1 | AX443278.1 | GN031906.1 | HW147141.1 | CS247806.1 | DD215803.1 | BD224846.1 | DJ016611.1 | HC035631.1 |
| GM627529.1 | AX427696.1 | GN031874.1 | HW146971.1 | DD174306.1 | DD215771.1 | BD224245.1 | DJ015680.1 | HC037063.1 |
| GM714494.1 | AX427212.1 | GN031842.1 | HW153727.1 | DD178541.1 | DD215741.1 | BD223543.1 | CS803370.1 | HC036950.1 |
| GM661502.1 | AX420106.1 | GN031810.1 | HW145786.1 | DD166211.1 | DD215709.1 | BD222521.1 | DL117252.1 | HC025512.1 |
| GM661470.1 | AX417924.1 | GN031777.1 | HW145521.1 | DD171383.1 | DD215677.1 | BD211427.1 | DL117220.1 | HC025480.1 |
| GM661438.1 | AX407348.1 | GN031745.1 | HW145143.1 | DD165168.1 | CS254894.1 | BD209869.1 | DL091639.1 | HC025448.1 |
| GM661406.1 | AX404735.1 | GN031712.1 | HW153268.1 | DD165136.1 | DD211197.1 | BD205759.1 | DL091607.1 | HC025416.1 |
| GM661374.1 | AX398665.1 | GN031680.1 | HW152949.1 | DD164758.1 | DD191816.1 | DD283600.1 | DL091575.1 | HC010651.1 |
| GM661342.1 | AX395267.1 | GN031648.1 | HW151769.1 | DD176018.1 | DD200277.1 | DD283503.1 | DL087964.1 | HC010192.1 |
| GM654348.1 | AX394152.1 | GN031616.1 | HV577531.1 | DD163904.1 | DD199980.1 | DD281823.1 | DL087932.1 | HC010115.1 |
| GM654316.1 | AX392381.1 | GN031552.1 | HV591564.1 | L07338.1 | DD206864.1 | DD287676.1 | DL121763.1 | HC008557.1 |
| GM654284.1 | AX391691.1 | GN031520.1 | HV582306.1 | CS157786.1 | DD206832.1 | DD280155.1 | DL121731.1 | HC007814.1 |
| GM654188.1 | AX384573.1 | GN031488.1 | HV582163.1 | CS157947.1 | DD206802.1 | DD279456.1 | DL121699.1 | HC007782.1 |
| GM647195.1 | AX377926.1 | GN031423.1 | HV586593.1 | CS127012.1 | DD212660.1 | DD279028.1 | DL121635.1 | HC007750.1 |
| GM647163.1 | AX376950.1 | GN031391.1 | HV594615.1 | CS124704.1 | CS236394.1 | DD278741.1 | DL121603.1 | HC007686.1 |
| GM647131.1 | AX375739.1 | GN031327.1 | HV586272.1 | CS124664.1 | DD163498.1 | CS356010.1 | DL087776.1 | HC007654.1 |
| GM647099.1 | AX370402.1 | GN031294.1 | HV601131.1 | CS124630.1 | DD163307.1 | CS353191.1 | DL087744.1 | HC007626.1 |
| GM647032.1 | AX364557.1 | GN031262.1 | HV574888.1 | CS124582.1 | DD163195.1 | CS352450.1 | DL087712.1 | HC007594.1 |
| GM647059.1 | AX364525.1 | GN031230.1 | HV574489.1 | CS124542.1 | CQ876214.1 | CS351530.1 | DL087680.1 | HC007562.1 |
| GM647006.1 | AX364493.1 | GN031198.1 | HV585762.1 | CS119867.1 | CQ876182.1 | CS283941.1 | DL087648.1 | HC007530.1 |
| GM640200.1 | AX364461.1 | GN031166.1 | HV585749.1 | HW381025.1 | CQ876147.1 | CS142499.1 | DL087616.1 | HC010769.1 |
| GM640168.1 | AX364397.1 | GN031133.1 | HV574125.1 | HW350992.1 | CQ848456.1 | CS141588.1 | DL128941.1 | DM374723.1 |
| GM640136.1 | AX364365.1 | GN031101.1 | HV570604.1 | HW350884.1 | CQ873297.1 | CS141533.1 | DL125515.1 | HC005492.1 |
| GM640104.1 | AX364270.1 | GN031069.1 | HV569503.1 | HW340460.1 | CQ871424.1 | CS141501.1 | DL125483.1 | HC003694.1 |
| GM640072.1 | AX364173.1 | GN031037.1 | HV569043.1 | HW350661.1 | CQ871369.1 | CS141163.1 | DL125451.1 | DM371004.1 |
| CS408106.1 | AX360312.1 | GN031005.1 | HV568111.1 | HV945088.1 | CQ869302.1 | CS139083.1 | DL125419.1 | DM370972.1 |
| CS402512.1 | AX358359.1 | GN030973.1 | HV568023.1 | HV778461.1 | CQ868936.1 | CS136262.1 | DL125387.1 | DM370940.1 |
| CS407638.1 | HW336622.1 | GN030942.1 | HV567903.1 | HV775404.1 | CQ868386.1 | CS132064.1 | DL125355.1 | DM370837.1 |
| CS401801.1 | HW336542.1 | GN030910.1 | HV566106.1 | HV757326.1 | CQ867176.1 | CS131078.1 | DL121566.1 | DM370803.1 |
| CS380592.1 | HW321087.1 | GN030878.1 | HV565824.1 | HV702583.1 | CQ861179.1 | CS123352.1 | DL121534.1 | DM370768.1 |
| CS382742.1 | HW329412.1 | GN030846.1 | HV573541.1 | HW295686.1 | CQ859652.1 | CS122635.1 | DL121502.1 | DM370670.1 |
| CS376600.1 | HW336157.1 | GN030814.1 | HV572436.1 | HW294989.1 | CQ859316.1 | CS106187.1 | DL121470.1 | DM370638.1 |
| DD288562.1 | HW328679.1 | GN030782.1 | HV572128.1 | HW294006.1 | CQ858715.1 | CS106093.1 | DL121438.1 | DM370606.1 |
| BD399089.1 | HW328552.1 | DM001667.1 | HV571425.1 | HW293263.1 | CQ858085.1 | CS179767.1 | DL121406.1 | DM370574.1 |
| BD441342.1 | HW328439.1 | DM006935.1 | HV560165.1 | HW293043.1 | CQ857501.1 | CS326355.1 | DL090200.1 | DM370542.1 |
| BD409482.1 | HW328336.1 | DM005041.1 | HV558756.1 | HW291307.1 | CQ855977.1 | CS323707.1 | DL086195.1 | DM370510.1 |
| BD451965.1 | HW328157.1 | DL125439.1 | HV561865.1 | HW291182.1 | CQ855756.1 | CS323600.1 | DL086163.1 | GN052392.1 |
| BD398718.1 | HW318647.1 | DL125407.1 | HV550129.1 | HW291150.1 | CQ854058.1 | CS329673.1 | DL086131.1 | GN059750.1 |
| BD398639.1 | HW318470.1 | DL125375.1 | HV554338.1 | HW291053.1 | CQ849421.1 | CS320114.1 | DL086099.1 | GN051181.1 |

| | | | | | | | | |
|---|---|---|---|---|---|---|---|---|
| BD451734.1 | HW335177.1 | DL102455.1 | HV552732.1 | HW291021.1 | CQ840699.1 | CS318714.1 | DL086067.1 | GN048971.1 |
| BD451684.1 | HW314148.1 | DL102423.1 | HV550973.1 | HW290957.1 | CQ832131.1 | DD261033.1 | DL086035.1 | GN046490.1 |
| BD398165.1 | HW314021.1 | DL095431.1 | HV550376.1 | HW290925.1 | CQ831795.1 | DD259888.1 | DL086003.1 | GN045898.1 |
| BD408123.1 | HW312166.1 | DL095367.1 | AY659412.1 | HW290862.1 | CQ829238.1 | DD175897.1 | DL114437.1 | DM014245.1 |
| BD450230.1 | HW312101.1 | DL107083.1 | AY659380.1 | HW290094.1 | CQ828194.1 | CS001395.1 | DL114405.1 | DM012886.1 |
| BD439820.1 | HW307764.1 | DL107051.1 | AY659348.1 | HW289971.1 | CQ827910.1 | AF408184.1 | DL114373.1 | DM023678.1 |
| BD408013.1 | HW308586.1 | DL106987.1 | AY659316.1 | HW287788.1 | CQ827558.1 | CS157803.1 | DL114341.1 | DM023580.1 |
| BD449663.1 | HW307893.1 | DL106955.1 | AY659284.1 | DI170076.1 | CQ819840.1 | CS157932.1 | DL109348.1 | DM028086.1 |
| BD449627.1 | HW307829.1 | CS119234.1 | AY659252.1 | HV961473.1 | CQ818017.1 | CS157900.1 | DL109316.1 | DM022397.1 |
| BD419019.1 | HW307765.1 | CS119202.1 | AY659220.1 | HV960585.1 | CQ816983.1 | CS157868.1 | DL109284.1 | DM022321.1 |
| BD449397.1 | HW307759.1 | CS119170.1 | AY659188.1 | HV964072.1 | CQ816943.1 | CS146887.1 | DL104582.1 | DM038581.1 |
| BD418811.1 | HW307727.1 | CS119105.1 | FW577839.1 | HV958300.1 | CQ815109.1 | CS128722.1 | DL104550.1 | DM022280.1 |
| BD429363.1 | HW307695.1 | CS119038.1 | FW577599.1 | HV963328.1 | CQ814101.1 | CS124725.1 | DL104518.1 | DM022214.1 |
| BD418545.1 | HW315590.1 | CS119005.1 | FW577118.1 | HV969829.1 | CQ814069.1 | CS124687.1 | DL093934.1 | DM022158.1 |
| BD396647.1 | HW315447.1 | CS118973.1 | FW576960.1 | HV965305.1 | CQ814036.1 | CS124647.1 | DL093902.1 | DM032828.1 |
| BD437799.1 | HW315098.1 | CS118908.1 | FW576807.1 | HV956029.1 | CQ814004.1 | CS124603.1 | DL093870.1 | DM015997.1 |
| BD437705.1 | HW315066.1 | CS118874.1 | FW576695.1 | HV951132.1 | CQ813972.1 | CS124565.1 | DL090095.1 | DM015953.1 |
| BD437673.1 | HW314824.1 | CS118842.1 | FW575891.1 | HV950607.1 | CQ813940.1 | CS124525.1 | DL090063.1 | GN045775.1 |
| BD459659.1 | HW314770.1 | CS118808.1 | FW575452.1 | HV947303.1 | CQ813908.1 | CS120791.1 | DL090031.1 | GN041690.1 |
| BD437126.1 | HW314716.1 | CS118776.1 | FW572727.1 | HV947134.1 | CQ813876.1 | CS119516.1 | DL089999.1 | GN039843.1 |
| BD394869.1 | HW065228.1 | CS118711.1 | HV313437.1 | HV951955.1 | CQ813844.1 | CS119484.1 | DL126669.1 | GN037326.1 |
| BD404971.1 | HW058486.1 | CS118615.1 | HV317234.1 | HV946895.1 | CQ813812.1 | CS119416.1 | DL123350.1 | GN034794.1 |
| BD446181.1 | HW064902.1 | CS118582.1 | HV226857.1 | HV945872.1 | CQ813691.1 | CS119383.1 | DL123318.1 | GN030753.1 |
| BD393746.1 | HW064869.1 | CS118547.1 | HV221451.1 | HV939872.1 | CQ813607.1 | CS119351.1 | DL123286.1 | GN030625.1 |
| BD453360.1 | HW056522.1 | CS118514.1 | HV214622.1 | HV815575.1 | CQ809735.1 | CS119319.1 | DL123254.1 | GN030593.1 |
| BD453329.1 | HW060696.1 | CS118449.1 | HV304094.1 | AY658426.1 | CQ807215.1 | CS119285.1 | DL123222.1 | GM661202.1 |
| BD453297.1 | HW062397.1 | CS118417.1 | HV217625.1 | AY658394.1 | CQ807163.1 | CS119253.1 | DL123190.1 | GM654144.1 |
| BD453265.1 | HW060509.1 | CS118384.1 | HV191506.1 | AY658362.1 | AX497562.1 | CS119221.1 | DL118904.1 | GM654112.1 |
| BD455629.1 | HW064439.1 | CS118251.1 | HV197641.1 | AY658330.1 | AX494201.1 | CS086827.1 | DL118872.1 | DL241156.1 |
| BD455582.1 | HW062372.1 | CS118185.1 | HV191322.1 | AY658298.1 | BD139563.1 | CS086063.1 | DL118840.1 | FB748693.1 |
| BD444977.1 | HW042063.1 | CS118120.1 | HV182463.1 | AY658266.1 | BD138716.1 | CS084272.1 | DL118808.1 | FB742061.1 |
| BD413659.1 | HW042031.1 | CS118087.1 | HV182431.1 | AY658234.1 | BD138411.1 | CS083812.1 | DL109256.1 | FB748892.1 |
| BD444963.1 | HW043561.1 | CS118021.1 | HV182399.1 | AY658202.1 | BD138376.1 | CS082355.1 | DL109224.1 | FB748754.1 |
| BD388115.1 | HW041987.1 | CS117283.1 | HV182367.1 | AY658170.1 | BD136247.1 | CS080404.1 | DL109192.1 | FB748139.1 |
| BD378120.1 | HW041955.1 | CS108625.1 | HV220401.1 | AY658138.1 | BD135886.1 | CS079166.1 | DL109160.1 | DL233420.1 |
| BD375686.1 | HW041923.1 | CS106694.1 | HV226357.1 | AY658106.1 | BD134014.1 | CS077805.1 | DL109128.1 | DL233386.1 |
| BD374946.1 | HW041825.1 | CS106389.1 | HV039586.1 | AY658074.1 | BD131357.1 | CS075524.1 | DL109096.1 | DL232101.1 |
| BD374052.1 | HW049409.1 | CS104202.1 | HV117793.1 | AY658042.1 | BD130809.1 | CS073942.1 | DL104362.1 | DL207312.1 |
| BD360145.1 | HW049377.1 | CS102997.1 | HV117225.1 | AY658010.1 | BD130777.1 | CS072179.1 | DL093746.1 | DL213344.1 |
| BD357347.1 | HW049346.1 | CS102901.1 | HV038763.1 | AY657978.1 | BD130744.1 | CS070577.1 | DL093714.1 | DL207431.1 |
| BD356596.1 | HW049250.1 | CS102805.1 | HI177804.1 | AY657946.1 | BD128325.1 | CS070114.1 | DL093682.1 | DL206828.1 |
| BD356481.1 | HW046212.1 | CS102773.1 | HI214439.1 | AY657914.1 | BD106885.1 | CS067278.1 | DL093586.1 | DL206664.1 |
| BD350531.1 | HW043252.1 | CS102741.1 | HI213012.1 | AY657882.1 | HI990055.1 | CS062959.1 | DL093554.1 | DL220150.1 |
| BD359225.1 | HW041789.1 | CS102645.1 | HI212969.1 | AY657850.1 | HI988936.1 | CS061608.1 | DL089939.1 | DL219991.1 |
| BD345189.1 | HW043071.1 | CS102613.1 | HI547161.1 | AY657818.1 | HI987433.1 | CS061035.1 | DL089907.1 | DL219909.1 |
| BD342578.1 | HW054033.1 | CS102581.1 | HI000500.1 | AY657786.1 | FW559258.1 | CS059013.1 | DL089875.1 | DL219507.1 |
| BD340627.1 | HV985993.1 | CS102549.1 | HI546324.1 | AY657754.1 | FW562042.1 | AY967390.1 | DL089843.1 | DL206310.1 |

| | | | | | | | | |
|---|---|---|---|---|---|---|---|---|
| BD349533.1 | HI001825.1 | CS102517.1 | HI546290.1 | AY657722.1 | FW552171.1 | AY967358.1 | DL041353.1 | DL230376.1 |
| BD339098.1 | HI001765.1 | CS102485.1 | HI211003.1 | AY657690.1 | FW552136.1 | AY967326.1 | DL041289.1 | DL230341.1 |
| BD338950.1 | HI469553.1 | CS101385.1 | HI210970.1 | AY657658.1 | FW555986.1 | AY967294.1 | DL037402.1 | FB714131.1 |
| BD325666.1 | HI544128.1 | CS098076.1 | HI210938.1 | AY657626.1 | FW557302.1 | AY967262.1 | DL037370.1 | FB713964.1 |
| BD325573.1 | HI001609.1 | A07200.1 | HI570414.1 | AY657594.1 | FW561702.1 | AY967230.1 | DL037338.1 | FB713804.1 |
| BD314208.1 | HI001562.1 | A02301.1 | HI473063.1 | AY657562.1 | FW561670.1 | AY967198.1 | DL021493.1 | FB713772.1 |
| BD313630.1 | HI001509.1 | A02701.1 | HI472911.1 | AY657530.1 | FW557012.1 | AY967166.1 | DL021461.1 | FB713740.1 |
| BD312799.1 | HI003389.1 | A01416.1 | HI071984.1 | AY657498.1 | FW562704.1 | AY967134.1 | DL021429.1 | FB713577.1 |
| CQ982982.1 | HI003350.1 | A00705.1 | HI004381.1 | AY657466.1 | HI564709.1 | AY967102.1 | DL021397.1 | FB712998.1 |
| CQ982945.1 | HI003309.1 | M12616.1 | HI004253.1 | AY657434.1 | HI568856.1 | AY967070.1 | DL017191.1 | FB708589.1 |
| CQ982913.1 | HI550419.1 | M63418.1 | HI002280.1 | AY657402.1 | HI559127.1 | AY967038.1 | DL017159.1 | FB708163.1 |
| CQ982785.1 | HI464688.1 | M31472.1 | HI002243.1 | AY657370.1 | HI554957.1 | AY967006.1 | DL017127.1 | FB672304.1 |
| CQ982620.1 | HI464656.1 | M29404.1 | HI002193.1 | AY657338.1 | HI564205.1 | AY966974.1 | DL017095.1 | FB669115.1 |
| CQ982547.1 | HI464608.1 | M11248.1 | HI002112.1 | AY657306.1 | HI564096.1 | AY966942.1 | DL017063.1 | FB667427.1 |
| CQ975406.1 | HI549482.1 | M19560.1 | HI000457.1 | AY657274.1 | HI563621.1 | CS056290.1 | DL017031.1 | FB667352.1 |
| CQ975374.1 | HI462680.1 | AF411597.1 | HI000409.1 | AY657242.1 | HI563588.1 | CS055982.1 | DL049430.1 | FB666806.1 |
| CQ973145.1 | HH714039.1 | LT897789.1 | HI000367.1 | AY657210.1 | HI551353.1 | CS055787.1 | DL049366.1 | FB721081.1 |
| CQ977197.1 | HH713505.1 | AH002284.2 | HI000310.1 | AY657178.1 | HI544145.1 | CS054778.1 | CS607532.1 | FB704339.1 |
| CQ976305.1 | HH961375.1 | M12532.1 | HI553263.1 | AY657146.1 | HI508246.1 | CS053081.1 | CS600623.1 | FB676578.1 |
| CQ975805.1 | HH961311.1 | M19976.1 | HI553191.1 | AY657114.1 | HI000050.1 | CS052506.1 | CS607464.1 | FB676546.1 |
| CQ972470.1 | HH961279.1 | DQ250197.1 | HI545337.1 | AY657082.1 | HI000008.1 | CS052391.1 | CS607195.1 | AB470101.1 |
| CQ972438.1 | HH961247.1 | AY781342.1 | HI583939.1 | AY657050.1 | HI003595.1 | CS052357.1 | CS607085.1 | FB701529.1 |
| CQ972374.1 | HH975166.1 | AF003720.1 | HI583844.1 | U39467.1 | HI001838.1 | CS052323.1 | CS001390.1 | CS810728.1 |
| CQ972310.1 | HH964276.1 | AY255626.1 | HI520629.1 | M27084.1 | HI001778.1 | CS052258.1 | AF408177.1 | FB701785.1 |
| CQ971887.1 | HH982027.1 | S78584.1 | HI207676.1 | M17743.1 | HI001740.1 | CS050988.1 | CS161096.1 | FB702559.1 |
| CQ971605.1 | HH974538.1 | HW390739.1 | HI207612.1 | K02757.1 | HI465731.1 | CS048859.1 | CS157798.1 | FB660759.1 |
| CQ970079.1 | HH998107.1 | HW399359.1 | HI003703.1 | M10308.1 | HI574582.1 | CS039112.1 | CS157959.1 | FB660718.1 |
| CQ969212.1 | HH998059.1 | HW399311.1 | HI002078.1 | M35111.1 | HI001679.1 | CS038830.1 | CS157927.1 | FB581876.1 |
| CQ967778.1 | HH998003.1 | HW408957.1 | HI002027.1 | M34000.1 | HI001620.1 | CS037898.1 | CS146877.1 | FB665242.1 |
| CQ964479.1 | HH997941.1 | HW382550.1 | HI001944.1 | HV549333.1 | HI001578.1 | CQ840583.1 | CS126236.1 | DL116909.1 |
| CQ963846.1 | HH999650.1 | HW381663.1 | HI000276.1 | HV549099.1 | HI003362.1 | CQ830729.1 | CS124720.1 | DL116877.1 |
| CQ963581.1 | HH997901.1 | HW390548.1 | HI000236.1 | HV544370.1 | HI003325.1 | CQ828193.1 | CS124680.1 | DL116845.1 |
| CQ955625.1 | HH997830.1 | HV952059.1 | HI000192.1 | HV543465.1 | HI071578.1 | CQ827557.1 | CS124558.1 | DL116817.1 |
| CQ947458.1 | HH997764.1 | HV939877.1 | HI000136.1 | HV538621.1 | HI550387.1 | CQ827525.1 | CS121849.1 | DL116785.1 |
| CQ947218.1 | HH999499.1 | HV943363.1 | HI205883.1 | CS106205.1 | HI464731.1 | CQ826865.1 | CS120450.1 | DL116753.1 |
| CQ947133.1 | HH999445.1 | HV942438.1 | HI180717.1 | CS106024.1 | HI464699.1 | CQ821854.1 | CS119545.1 | DL116721.1 |
| CQ945875.1 | HH999404.1 | HV929306.1 | HI204238.1 | CS179868.1 | HI464667.1 | CQ821299.1 | CS119511.1 | DL116689.1 |
| CQ944203.1 | HH999317.1 | HV931341.1 | HI470573.1 | CQ924201.1 | HI464635.1 | CQ818666.1 | CS119445.1 | DL116657.1 |
| CQ944171.1 | HH997643.1 | HV931252.1 | HI544680.1 | A32691.1 | HI528871.1 | CQ816982.1 | CS119378.1 | DL116625.1 |
| DL034981.1 | HH997533.1 | HV925516.1 | HI559198.1 | CQ898802.1 | HI464424.1 | CQ816941.1 | CS119346.1 | DL112032.1 |
| DL034949.1 | HH997425.1 | HV936377.1 | HI564711.1 | CQ898660.1 | HI542548.1 | CQ815752.1 | CS119248.1 | DL112000.1 |
| DL034917.1 | HH997380.1 | HV929983.1 | FW335709.1 | CQ898628.1 | HI462736.1 | CQ815107.1 | CS119152.1 | DL111968.1 |
| CS693498.1 | HH999692.1 | HV813903.1 | HC868222.1 | CQ898596.1 | HI503822.1 | CQ814068.1 | CS119120.1 | DL111936.1 |
| CS680848.1 | HH999148.1 | HV802930.1 | HC868076.1 | CQ898262.1 | HH961354.1 | CQ814035.1 | CS119085.1 | DL111904.1 |
| CS677752.1 | HH998111.1 | HV802898.1 | HC868020.1 | CQ895671.1 | HH961322.1 | CQ814003.1 | CS119052.1 | DL111892.1 |
| DJ008411.1 | HH999072.1 | AY659391.1 | HC887351.1 | CQ891376.1 | HH961290.1 | CQ813971.1 | CS119020.1 | DL106869.1 |
| DJ008379.1 | HH999027.1 | AY659359.1 | HC867645.1 | CQ888094.1 | HH961258.1 | CQ813939.1 | CS118987.1 | DL106837.1 |

| | | | | | | | | |
|---|---|---|---|---|---|---|---|---|
| DJ011749.1 | HH998960.1 | AY659327.1 | HC867554.1 | CQ877371.1 | HH964841.1 | CQ813907.1 | CS118955.1 | DL106805.1 |
| DD491858.1 | HC083721.1 | AY659295.1 | HC872741.1 | CQ875002.1 | HH975381.1 | CQ813875.1 | CS118922.1 | DL106773.1 |
| DD495648.1 | U26408.1 | AY659263.1 | HC856029.1 | CQ871397.1 | HH975089.1 | CQ813843.1 | CS118889.1 | DL106741.1 |
| DD495144.1 | HC057021.1 | AY659231.1 | HC880551.1 | CQ869275.1 | HH964366.1 | CQ813811.1 | CS118856.1 | DL106709.1 |
| CS669793.1 | DM381914.1 | AY659199.1 | HC889123.1 | CQ857849.1 | HH974549.1 | CQ813778.1 | CS118823.1 | DL091343.1 |
| CS667969.1 | DM381313.1 | AY659167.1 | HC876970.1 | CQ857588.1 | HH981890.1 | CQ813746.1 | CS118790.1 | DL091311.1 |
| DD467994.1 | HC054855.1 | AY659135.1 | HC876933.1 | CQ854064.1 | HH998076.1 | CQ813606.1 | CS118758.1 | DL091247.1 |
| DD464406.1 | HC053861.1 | AY659103.1 | HC876894.1 | AX781234.1 | HH998023.1 | CQ809732.1 | CS118725.1 | DL091215.1 |
| DD463045.1 | HC051926.1 | AY659071.1 | FW304570.1 | AX773260.1 | HH997965.1 | CQ807276.1 | CS118693.1 | DL087375.1 |
| CS647275.1 | HC051577.1 | AY659039.1 | FW331840.1 | AX766949.1 | HH999661.1 | DD407004.1 | CS118661.1 | DL087343.1 |
| CS646767.1 | HC049892.1 | AY658975.1 | FW309615.1 | AX746410.1 | HH997919.1 | DD406131.1 | CS118597.1 | DL087311.1 |
| CS646205.1 | HC045493.1 | AY658943.1 | FW307469.1 | BD183209.1 | HH997841.1 | DD402586.1 | CS118563.1 | DL087279.1 |
| CS645217.1 | HC045461.1 | AY658911.1 | FW306901.1 | BD182780.1 | HH997779.1 | DD405836.1 | CS118496.1 | DL087247.1 |
| CS644552.1 | HC045429.1 | AY658879.1 | HC471913.1 | BD181479.1 | FV529000.1 | DD405804.1 | CS118463.1 | DL087215.1 |
| DD462114.1 | HC045397.1 | HW261042.1 | HC471881.1 | BD180844.1 | FV534648.1 | DD402408.1 | CS118431.1 | DL102108.1 |
| DD460835.1 | HC045365.1 | HW261010.1 | HC678784.1 | AX744020.1 | FV534473.1 | DD402376.1 | CS118398.1 | DL102076.1 |
| DD460531.1 | HC045333.1 | HW260978.1 | HC678460.1 | BD177399.1 | HC460435.1 | DD402344.1 | CS118366.1 | DL102044.1 |
| DD460159.1 | HC045269.1 | HW260946.1 | HC668137.1 | AX709608.1 | HC456289.1 | DD402312.1 | CS118331.1 | DL102012.1 |
| DD459946.1 | HC047033.1 | HW260914.1 | HC508387.1 | AX708672.1 | HC453683.1 | DD402280.1 | CS118297.1 | DL101948.1 |
| DD455164.1 | HC047001.1 | HW260882.1 | HC504648.1 | AX703496.1 | HC453651.1 | DD402248.1 | CS118265.1 | DL097534.1 |
| DD458756.1 | HC046969.1 | HW260850.1 | HC494557.1 | AX683727.1 | HC460189.1 | DD402216.1 | CS118233.1 | DL097502.1 |
| DD453831.1 | GN090927.1 | HW260818.1 | HC494424.1 | AX664356.1 | HC452240.1 | DD402184.1 | CS118200.1 | DL097470.1 |
| DD451438.1 | GN089842.1 | HW260786.1 | HC502326.1 | AX659120.1 | HC452162.1 | DD402152.1 | CS118166.1 | DL097438.1 |
| CS642385.1 | GN089186.1 | HW260754.1 | HC501572.1 | AX657124.1 | HC452106.1 | DD402120.1 | CS118134.1 | DL097406.1 |
| CS642214.1 | GN088780.1 | HW260722.1 | HC500692.1 | AX657089.1 | HC452046.1 | DD402088.1 | CS118102.1 | DL097374.1 |
| CS631230.1 | GN088030.1 | HW260690.1 | HC499869.1 | AX144049.1 | HC451966.1 | DD402056.1 | AX348943.1 | DL097342.1 |
| CS627912.1 | L08948.1 | HW260658.1 | GM641609.1 | AX143919.1 | HC451772.1 | DD405776.1 | AX348470.1 | DL121297.1 |
| CS627779.1 | L08937.1 | HW260626.1 | GM629264.1 | AX143855.1 | HC450530.1 | DD405744.1 | AX347325.1 | DL121233.1 |
| CS632851.1 | L08856.1 | HW260594.1 | GM629232.1 | AX143791.1 | FU761018.1 | DD405712.1 | AX347287.1 | DL121201.1 |
| CS632819.1 | M94405.1 | HW260562.1 | GM629200.1 | AX143727.1 | FU760024.1 | DD405680.1 | AX347249.1 | DL101895.1 |
| CS632618.1 | DM059611.1 | HW260530.1 | GM629136.1 | AX143151.1 | FU756849.1 | DD405648.1 | AX347217.1 | DL101831.1 |
| CS632184.1 | DM063452.1 | HW260498.1 | GM629104.1 | AX143023.1 | HC439414.1 | DD405596.1 | AX347179.1 | DL199730.1 |
| CS627081.1 | DM058823.1 | HW260466.1 | GM634066.1 | AX142831.1 | HC438922.1 | DD405564.1 | AX347039.1 | DL199698.1 |
| CS623615.1 | DM045513.1 | HW260434.1 | GM634034.1 | AX142575.1 | HC438421.1 | DD405500.1 | AX345073.1 | DL194549.1 |
| DD450369.1 | DM045361.1 | HW260306.1 | GM634002.1 | AX142509.1 | HC360365.1 | DD405436.1 | AX344535.1 | DL194517.1 |
| DD450300.1 | DM045099.1 | HW260274.1 | GM633874.1 | AX142381.1 | HC435806.1 | DD402030.1 | AX343929.1 | DL194199.1 |
| CS416228.1 | DM044911.1 | HW260242.1 | GM716543.1 | AX142125.1 | HC358225.1 | DD401998.1 | AX342802.1 | DL188830.1 |
| CS415775.1 | DM039599.1 | HW260210.1 | GM655515.1 | AX142061.1 | HC358193.1 | DD401966.1 | AX338546.1 | DL198994.1 |
| CS410713.1 | DM044794.1 | HW260178.1 | GM655483.1 | AX141995.1 | HC358062.1 | DD401905.1 | AX327957.1 | DL124446.1 |
| CS414856.1 | DM060667.1 | HW260146.1 | GM655451.1 | AX141931.1 | HC357540.1 | CS409993.1 | AX319612.1 | DL124414.1 |
| CS414784.1 | GN067914.1 | HW260114.1 | GM648362.1 | AX141865.1 | HC434783.1 | CS403046.1 | AX316546.1 | DL124382.1 |
| DD321004.1 | GN067726.1 | HW260082.1 | GM648330.1 | AX141737.1 | HC325460.1 | CS408094.1 | AX306563.1 | DL120293.1 |
| DD327515.1 | GN067694.1 | HW260050.1 | GM648298.1 | AX141673.1 | HC324888.1 | CS402196.1 | AX304907.1 | DL120261.1 |
| DD323494.1 | GN067598.1 | HW260018.1 | GM648266.1 | AX141417.1 | HC324503.1 | CS402111.1 | AX266989.1 | DL120229.1 |
| DD326902.1 | GN062878.1 | HW259986.1 | GM648234.1 | AX141353.1 | HC324081.1 | CS401797.1 | AX259986.1 | DL115826.1 |
| DD329920.1 | GM636537.1 | HW259954.1 | GM648202.1 | AX133916.1 | HC042320.1 | CS380540.1 | AX301008.1 | DL115794.1 |
| DD329747.1 | GM636505.1 | HW259922.1 | GM641399.1 | AX113864.1 | HC047460.1 | CS382625.1 | AX297551.1 | DL115762.1 |

| | | | | | | | | |
|---|---|---|---|---|---|---|---|---|
| DD326846.1 | GM636473.1 | HW258857.1 | GM641367.1 | AX107890.1 | HC047396.1 | CS376596.1 | AX287016.1 | DL115698.1 |
| DD321767.1 | GM650460.1 | HW258817.1 | GM641335.1 | AX097496.1 | GM642060.1 | DD309774.1 | AX283687.1 | DL115666.1 |
| CS390500.1 | GM643492.1 | HW257309.1 | GM641303.1 | AX088391.1 | GM642028.1 | DD309000.1 | AX283221.1 | DL115634.1 |
| CS398358.1 | GM643427.1 | HW257213.1 | GM641271.1 | AX085623.1 | GM634841.1 | DD292522.1 | AX282868.1 | DL111240.1 |
| CS389321.1 | GM631838.1 | HW257181.1 | GM641239.1 | AX083745.1 | GM634809.1 | DD291491.1 | AX282187.1 | DL111208.1 |
| CS389288.1 | GM631806.1 | HW257085.1 | GM629080.1 | AX081280.1 | GM634777.1 | DD291022.1 | AX279949.1 | DL111144.1 |
| CS389199.1 | AX253456.1 | HW256989.1 | GM629048.1 | AX077349.1 | GM629747.1 | CS365146.1 | AX279674.1 | DL111080.1 |
| CS389161.1 | AX247519.1 | HW256765.1 | GM629016.1 | HW097751.1 | GM629715.1 | CS376006.1 | AX278750.1 | DL106276.1 |
| CS388973.1 | AX242254.1 | HW256733.1 | GM628984.1 | HW097646.1 | GM736707.1 | CS362750.1 | AX256405.1 | DL106244.1 |
| CS401626.1 | AX242222.1 | HW250847.1 | GM628952.1 | HW065066.1 | GM887868.1 | CS378191.1 | AX256336.1 | DL106212.1 |
| CS406686.1 | AX242190.1 | HV349276.1 | GM881896.1 | HW049573.1 | GM655996.1 | CS359747.1 | AX253577.1 | DL106180.1 |
| CS406451.1 | AX242158.1 | HV344938.1 | GM655226.1 | HV969764.1 | GM655964.1 | CS359643.1 | AX242329.1 | DL106148.1 |
| CS406004.1 | AX242126.1 | HV344662.1 | GM655194.1 | HV778442.1 | GM648971.1 | BD269148.1 | AX242297.1 | DL106116.1 |
| CS410006.1 | AX242062.1 | HV339979.1 | GM655162.1 | HV777009.1 | GM648939.1 | BD268936.1 | AX242265.1 | DL101407.1 |
| CS403591.1 | AX242030.1 | HV339915.1 | GM648169.1 | HV757487.1 | GM648907.1 | BD268665.1 | AX242233.1 | DL101375.1 |
| CS408105.1 | AX241966.1 | HV342038.1 | GM641174.1 | HV757324.1 | GM648875.1 | BD266839.1 | AX242169.1 | DL090964.1 |
| CS402205.1 | AX241902.1 | HV322669.1 | GM641142.1 | HV743701.1 | GM648843.1 | BD265620.1 | AX242137.1 | DL110941.1 |
| CS407637.1 | AX241870.1 | HV321921.1 | GM641078.1 | HW302004.1 | GM648811.1 | BD265522.1 | AX242105.1 | DL110909.1 |
| CS401800.1 | AX241806.1 | HV321734.1 | GM641046.1 | HW294987.1 | GM642008.1 | BD263458.1 | AX242073.1 | DL110877.1 |
| CS380543.1 | AX241774.1 | HV325329.1 | GM628886.1 | HW293921.1 | GM641912.1 | BD262204.1 | AX241945.1 | DL106073.1 |
| CS382739.1 | AX241710.1 | FW420457.1 | GM628854.1 | HW293261.1 | GM641848.1 | BD252164.1 | AX241913.1 | DL106041.1 |
| CS376599.1 | AX241678.1 | FW420368.1 | GM628822.1 | HW293041.1 | GM634660.1 | BD251469.1 | AX241881.1 | DL106009.1 |
| DD288561.1 | AX241646.1 | FW420336.1 | GM628790.1 | HW292764.1 | GM634628.1 | BD251239.1 | AX241785.1 | DL105977.1 |
| DD309681.1 | AX241614.1 | FW420113.1 | FB761590.1 | HW291180.1 | GM652942.1 | BD250354.1 | DL099423.1 | DL105945.1 |
| DD309003.1 | AX241582.1 | FW496631.1 | FB761231.1 | HW291148.1 | GM652910.1 | BD249176.1 | DL089510.1 | DL105913.1 |
| DD308862.1 | AX241518.1 | HI653914.1 | GM035737.1 | HW291051.1 | GM652878.1 | BD247115.1 | DL089478.1 | DL101300.1 |
| DD308757.1 | AX241454.1 | HI653552.1 | GM035204.1 | HW291019.1 | GM652846.1 | BD246859.1 | DL089446.1 | DL101268.1 |
| DD292656.1 | AX235565.1 | HI653389.1 | GM017388.1 | HW290923.1 | GM652814.1 | BD243449.1 | DL089414.1 | DL101236.1 |
| DD291759.1 | AX223880.1 | HI653034.1 | GM952620.1 | HW290892.1 | GM652781.1 | BD243413.1 | DL089382.1 | DL101204.1 |
| DD291497.1 | HW338814.1 | HI646747.1 | GM949518.1 | HW290860.1 | GM645687.1 | BD242718.1 | DL113548.1 | DL101172.1 |
| DD291025.1 | HW338558.1 | AY145513.1 | GM890118.1 | HW298502.1 | GM645655.1 | BD242561.1 | DL113516.1 | DL101140.1 |
| DD289088.1 | HW338430.1 | HH804677.1 | GM889442.1 | DI213359.1 | GM645623.1 | BD242463.1 | DL113452.1 | DL096726.1 |
| CS377288.1 | HW338302.1 | HH797411.1 | GM889321.1 | HW104658.1 | GM638824.1 | BD242383.1 | DL014154.1 | DL096694.1 |
| CS376992.1 | HW338174.1 | HH794936.1 | GM888710.1 | HW099566.1 | GM638792.1 | BD240799.1 | DL014122.1 | DL096662.1 |
| CS364648.1 | HW337918.1 | HH779775.1 | GM008862.1 | HW099513.1 | GM638760.1 | BD238483.1 | DL014003.1 | DL096630.1 |
| CS376253.1 | HW337790.1 | HH997758.1 | GM888440.1 | HW066778.1 | GM626117.1 | BD238041.1 | DL013971.1 | DL096598.1 |
| CS376219.1 | HW337278.1 | HH999492.1 | GM868917.1 | HW083274.1 | GM626085.1 | BD235806.1 | DL013939.1 | DL096566.1 |
| CS375821.1 | HW336894.1 | HH999439.1 | GM003576.1 | HW083242.1 | GM626053.1 | BD234878.1 | DL030039.1 | DL094738.1 |
| CS362753.1 | HW336610.1 | HH997682.1 | FB754275.1 | HW083210.1 | GM626021.1 | BD232353.1 | DL030007.1 | DL094706.1 |
| CS367237.1 | HW329287.1 | HH997637.1 | FB753532.1 | HW099473.1 | GM625989.1 | BD232026.1 | DL029975.1 | DL094674.1 |
| CS276303.1 | HW336154.1 | HH997419.1 | GM618780.1 | GN359451.1 | GM625957.1 | BD231138.1 | DL046632.1 | DL094642.1 |
| CS359939.1 | HW335966.1 | HH997366.1 | GM864832.1 | GN359419.1 | GM652774.1 | BD229933.1 | DL046600.1 | DL094610.1 |
| CS359646.1 | HW328844.1 | HH999314.1 | FB726030.1 | GN359387.1 | FB776238.1 | CS318713.1 | DL046568.1 | DL094578.1 |
| CS230199.1 | HW328735.1 | HH999231.1 | FB725957.1 | GN359355.1 | FB775384.1 | CS322150.1 | DL046536.1 | DL090963.1 |
| BD269151.1 | HW328663.1 | HH999188.1 | GM842465.1 | GN359291.1 | FB774890.1 | DD261135.1 | DL042650.1 | DL090931.1 |
| HV958687.1 | HW328549.1 | HH999142.1 | GM616333.1 | GN359250.1 | FB766230.1 | DD259351.1 | DL042618.1 | DL090899.1 |
| HV963320.1 | HW328436.1 | HH999111.1 | GM731848.1 | GN343886.1 | FB764707.1 | DD259174.1 | DL042586.1 | DL086963.1 |

| | | | | | | | | |
|---|---|---|---|---|---|---|---|---|
| HV560161.1 | HW328154.1 | HH999062.1 | GM879669.1 | GN349490.1 | FB764669.1 | DD265468.1 | DL042670.1 | DL086931.1 |
| HV558755.1 | HW318464.1 | HH999015.1 | FB711259.1 | GN340544.1 | FB747040.1 | DD272120.1 | DL038662.1 | DL086899.1 |
| HV561863.1 | HW314145.1 | HH998954.1 | GM840414.1 | GN336552.1 | FB744747.1 | CS305316.1 | DL029840.1 | DL086867.1 |
| HV550128.1 | HW312156.1 | HH997263.1 | GM041778.1 | GN333944.1 | FB762774.1 | CS302592.1 | DL029744.1 | DL086835.1 |
| HV552567.1 | HW312098.1 | HH997163.1 | GM721294.1 | GN112582.1 | FB743932.1 | CS299546.1 | DL018281.1 | DL086803.1 |
| HV550972.1 | HW311238.1 | HH978163.1 | GM040292.1 | GM659290.1 | FB743900.1 | CS299417.1 | DL018249.1 | DL086771.1 |
| AY659411.1 | HW120880.1 | HH998906.1 | CS728553.1 | GM741850.1 | FB743868.1 | DD252139.1 | DL018217.1 | DL086739.1 |
| AY659379.1 | HW117995.1 | HH998848.1 | CS727225.1 | FB507556.1 | FB743836.1 | DD251997.1 | DL013783.1 | DL086707.1 |
| AY659347.1 | HW081478.1 | HH998797.1 | FB708966.1 | GM622524.1 | FB743782.1 | DD251607.1 | DL013751.1 | DL086675.1 |
| AY659315.1 | HW101676.1 | HH998738.1 | DL087208.1 | FB506764.1 | FB761734.1 | DD248877.1 | DL013719.1 | DL086643.1 |
| AY659283.1 | HW096986.1 | HH997029.1 | DL102101.1 | GM654806.1 | FB760335.1 | DD258248.1 | DL013687.1 | DL086611.1 |
| AY659251.1 | HW096722.1 | HH996979.1 | DL102069.1 | GM654774.1 | GM035211.1 | DD257324.1 | DL000624.1 | DL105884.1 |
| AY659219.1 | HW072897.1 | HH981106.1 | DL102037.1 | GM715036.1 | GM017389.1 | DD253720.1 | DL005727.1 | DL105852.1 |
| AY659187.1 | HW096349.1 | HH998693.1 | DL102005.1 | GM870324.1 | GM952642.1 | CS287701.1 | DL005436.1 | DL105820.1 |
| AY659155.1 | HW096207.1 | HH998652.1 | DL101973.1 | CS696112.1 | GM952243.1 | CS287565.1 | DJ493968.1 | DL101015.1 |
| AY659123.1 | HW071361.1 | HH998524.1 | DL097527.1 | CS696015.1 | GM890119.1 | AX766734.1 | DL008705.1 | DL100983.1 |
| AY659091.1 | HW104295.1 | HH996910.1 | DL097495.1 | CS695983.1 | GM889443.1 | AX765961.1 | DL008501.1 | DL096537.1 |
| AY659059.1 | HW071228.1 | HH996789.1 | DL097463.1 | CS695919.1 | GM889322.1 | AX764646.1 | DJ491588.1 | DL096505.1 |
| AY658995.1 | HW070914.1 | HH996754.1 | DL101888.1 | CS695887.1 | GM888711.1 | AX764614.1 | CS582401.1 | DL096473.1 |
| AY658963.1 | HW104026.1 | HH977452.1 | DL101824.1 | CS695855.1 | GM008865.1 | AX764582.1 | CS588482.1 | DL092834.1 |
| AY658931.1 | HW103745.1 | HC727360.1 | DL101792.1 | CS695823.1 | GM888441.1 | AF430166.1 | CS576393.1 | DL092802.1 |
| AY658899.1 | HW099581.1 | HC688471.1 | DL101760.1 | CS695759.1 | GM007154.1 | AX505221.1 | CS576675.1 | DL092770.1 |
| AY658867.1 | HV693339.1 | HC688408.1 | DL101728.1 | CS695663.1 | GM867489.1 | AX505157.1 | CS565544.1 | DL089155.1 |
| AY658835.1 | HV699143.1 | HC687674.1 | DL097218.1 | DL176603.1 | GM685522.1 | AX498499.1 | CS569625.1 | DL089123.1 |
| AY658803.1 | HV698135.1 | HC490852.1 | DL097186.1 | DL176384.1 | GM003577.1 | AX497560.1 | CS545383.1 | DL089091.1 |
| AY658771.1 | HV689461.1 | HC490820.1 | DL097154.1 | DL176310.1 | FB983615.1 | BD140197.1 | CS560777.1 | DL089059.1 |
| AY658739.1 | HV695883.1 | HC490788.1 | DL095230.1 | DL175873.1 | FB983213.1 | BD139561.1 | CS560457.1 | DL089027.1 |
| AY658707.1 | HV695578.1 | HC490756.1 | DL095198.1 | DL175282.1 | FB754798.1 | BD138751.1 | CS559099.1 | DL088995.1 |
| AY658675.1 | HV695546.1 | HC490692.1 | DL095166.1 | DL163556.1 | FB754120.1 | BD138409.1 | DD419822.1 | DL122354.1 |
| AY658643.1 | HV593623.1 | HC490528.1 | DL125269.1 | DL176730.1 | FB753533.1 | BD138374.1 | DD420051.1 | DL122322.1 |
| AY658611.1 | HV585146.1 | HC490496.1 | DL125237.1 | FB360092.1 | CS695622.1 | BD137147.1 | CS541288.1 | DL118004.1 |
| AY658579.1 | HV585114.1 | HC679513.1 | DL125205.1 | FB342843.1 | CS695494.1 | BD134012.1 | CS539268.1 | DL117972.1 |
| AY658547.1 | HV585082.1 | HC678809.1 | A06421.1 | DL102661.1 | CS695430.1 | BD131914.1 | CS537868.1 | DL117908.1 |
| AY658515.1 | HV592476.1 | HC504671.1 | A05046.1 | DL097991.1 | CS695398.1 | BD130807.1 | CS534835.1 | DL117876.1 |
| AY658483.1 | HV592411.1 | HC504136.1 | A04745.1 | DL026668.1 | DL176509.1 | BD130775.1 | CS502395.1 | DL117844.1 |
| AY658451.1 | HV592274.1 | HC502258.1 | A09814.1 | DJ446836.1 | DL176366.1 | BD130742.1 | CS502479.1 | DL113251.1 |
| AY658419.1 | HV575311.1 | HC500962.1 | A01730.1 | DJ432944.1 | DL176289.1 | BD130709.1 | CS498515.1 | DL113219.1 |
| AY658387.1 | HV570870.1 | HC499848.1 | A05196.1 | DJ436676.1 | DL182004.1 | BD130629.1 | DD418611.1 | DL113187.1 |
| AY658355.1 | HV570479.1 | FW303061.1 | A01949.1 | DJ434486.1 | DL181367.1 | BD130496.1 | DD418576.1 | DL113155.1 |
| AY658323.1 | HV566138.1 | FW300643.1 | A01345.1 | DJ438371.1 | DL174625.1 | BD090579.1 | DD412327.1 | DL113091.1 |
| AY658291.1 | HV573965.1 | FW304280.1 | M22371.1 | DJ433798.1 | DL164124.1 | BD087719.1 | DD412046.1 | DL113059.1 |
| AY658259.1 | HV571319.1 | HB423191.1 | M12467.1 | DJ438298.1 | DL176774.1 | BD081516.1 | DD411469.1 | DL108258.1 |
| AY658227.1 | HC873817.1 | HB403656.1 | M18433.1 | DJ438219.1 | FB355449.1 | BD080709.1 | DD410177.1 | DL108226.1 |
| AY658195.1 | HC880381.1 | HB395732.1 | M10913.1 | DJ428001.1 | FB344522.1 | BD080146.1 | CS493062.1 | DL108194.1 |
| AY658163.1 | FW304433.1 | HB397690.1 | M13107.1 | DJ402657.1 | FB343474.1 | BD078610.1 | BD292427.1 | DL108162.1 |
| AY658131.1 | FW308826.1 | DM115191.1 | J02560.1 | DJ401942.1 | FB359894.1 | BD075036.1 | BD291579.1 | DL108130.1 |
| AY658099.1 | FW308595.1 | DM114850.1 | M24158.1 | DJ401278.1 | FB342770.1 | BD074953.1 | BD291223.1 | DL108098.1 |

| | | | | | | | | |
|---|---|---|---|---|---|---|---|---|
| AY658067.1 | FW332115.1 | HB386901.1 | M19082.1 | DJ417447.1 | DL088283.1 | BD073588.1 | BD299605.1 | DL103492.1 |
| AY658035.1 | FW307796.1 | HB340006.1 | LC732344.1 | DJ402736.1 | DL088091.1 | BD070700.1 | BD298091.1 | DL103460.1 |
| AY658003.1 | FW332945.1 | HA641578.1 | MH087227.1 | DJ389573.1 | DL088059.1 | BD063684.1 | BD297644.1 | DL103428.1 |
| AY657971.1 | FW332374.1 | HA641383.1 | DQ156168.1 | CS467740.1 | DL088027.1 | BD062454.1 | BD296228.1 | DL103396.1 |
| AY657939.1 | FV534081.1 | HA641177.1 | AH002276.2 | DD400169.1 | DL087995.1 | BD016703.1 | BD295199.1 | DL098918.1 |
| AY657907.1 | HC465647.1 | HA640322.1 | M64480.1 | DD400137.1 | DL091936.1 | BD015183.1 | BD280099.1 | DL098886.1 |
| AY657875.1 | HC460863.1 | HA639653.1 | AF463530.1 | CS459119.1 | DL091904.1 | BD014235.1 | BD289748.1 | DL098854.1 |
| AY657843.1 | AY278220.1 | HA638530.1 | AF092842.1 | DD367714.1 | DM039677.1 | BD014202.1 | BD280562.1 | DL098822.1 |
| AY657811.1 | AF424784.1 | HA637678.1 | S52233.1 | DD361268.1 | DM044959.1 | BD014162.1 | BD277256.1 | DL098790.1 |
| AY657779.1 | HC456308.1 | HA643896.1 | LC002203.1 | DD362927.1 | DM039467.1 | BD013384.1 | BD279916.1 | DL098758.1 |
| AY657747.1 | HC453670.1 | DM102617.1 | HW408576.1 | DD368130.1 | DM055893.1 | AX478054.1 | BD278912.1 | DL092748.1 |
| AY657715.1 | HC460365.1 | DM092361.1 | HW408512.1 | DD361302.1 | GN069325.1 | E61335.1 | BD274104.1 | DL092716.1 |
| AY657683.1 | HC292900.1 | GN032189.1 | HW408480.1 | CS458323.1 | GN075486.1 | E64481.1 | BD273145.1 | DL092684.1 |
| HV038603.1 | HC295953.1 | GN032157.1 | HW408449.1 | CS457167.1 | GN067924.1 | AX468888.1 | BD272138.1 | DL092652.1 |
| HV038528.1 | HC292768.1 | GN031997.1 | HW408417.1 | CS456713.1 | GN067828.1 | AX468461.1 | BD271073.1 | DL092588.1 |
| HV182299.1 | HC288857.1 | GN031965.1 | HW408385.1 | CS450623.1 | GN067796.1 | AX468328.1 | BD271041.1 | DL088973.1 |
| HV202709.1 | HC295544.1 | GN031933.1 | HW390752.1 | DD361086.1 | GN067736.1 | AX466459.1 | DD231222.1 | DL088941.1 |
| HV187862.1 | HC292230.1 | GN031901.1 | HW399451.1 | DD359109.1 | GN052460.1 | AX460509.1 | DD230443.1 | DL088909.1 |
| HV199775.1 | HC294972.1 | GN031869.1 | HW399325.1 | DD357933.1 | GN052385.1 | AX458655.1 | DD229869.1 | DL088877.1 |
| HV187580.1 | HC294792.1 | GN031837.1 | HW409010.1 | DD357897.1 | GN047808.1 | AX458573.1 | DD228419.1 | DL088845.1 |
| HV302188.1 | HC299210.1 | GN031804.1 | HW391425.1 | DD357481.1 | GN046273.1 | AX456524.1 | DD227769.1 | DL088813.1 |
| HV312987.1 | HC299177.1 | GN031675.1 | HW390663.1 | DD355440.1 | GN045829.1 | AX454151.1 | DD227383.1 | DL088781.1 |
| HV120329.1 | HC291786.1 | GN031643.1 | HW390524.1 | A03652.1 | DM014183.1 | AX453998.1 | DD224655.1 | DL103284.1 |
| HV038136.1 | HC298732.1 | GN031611.1 | HW390415.1 | A06662.1 | DM019510.1 | AX453434.1 | DD231571.1 | DL103252.1 |
| HV037765.1 | HC291030.1 | GN031579.1 | HW389567.1 | CS446146.1 | DM022271.1 | AX232675.1 | DD231475.1 | DL103220.1 |
| HV037712.1 | HC290845.1 | GN031547.1 | HW389025.1 | CS434837.1 | DM022195.1 | AX225239.1 | DD231439.1 | DL098614.1 |
| HV037259.1 | HC293433.1 | GN031515.1 | HW388635.1 | BD408125.1 | GM658921.1 | AX214301.1 | DD231407.1 | DL098582.1 |
| HV036293.1 | HC293313.1 | GN031483.1 | HW081519.1 | BD408086.1 | GM658889.1 | AX209893.1 | CS273080.1 | DL098550.1 |
| HV031542.1 | HC293116.1 | GN031451.1 | HW101690.1 | BD450233.1 | GM658857.1 | AX207296.1 | A02470.1 | DL107972.1 |
| FZ435974.1 | HC289864.1 | GN031418.1 | HW097015.1 | BD429579.1 | GM658825.1 | AX205156.1 | FW559383.1 | DL107940.1 |
| FZ435886.1 | HC289686.1 | GN031386.1 | HW105234.1 | BD428607.1 | GM658793.1 | AX203361.1 | FW510483.1 | DL107908.1 |
| FZ425972.1 | HC207400.1 | GN031354.1 | HW105068.1 | BD396356.1 | GM651537.1 | AX202430.1 | FW506212.1 | DL092450.1 |
| FZ429322.1 | HC199623.1 | GN031322.1 | HW072673.1 | BD394871.1 | GM651505.1 | AX195147.1 | HI933977.1 | DL092418.1 |
| FZ437394.1 | GN041517.1 | GN031289.1 | HW096219.1 | BD453362.1 | GM651473.1 | AX193710.1 | HI936419.1 | DL092386.1 |
| FZ424176.1 | GN039776.1 | GN031225.1 | HW086487.1 | BD453331.1 | GM651441.1 | AX191375.1 | HI930638.1 | DL088771.1 |
| FZ428791.1 | GN037857.1 | GN031193.1 | HW072441.1 | BD453299.1 | GM651409.1 | AX180282.1 | HI929430.1 | DL088739.1 |
| FZ423885.1 | GN034809.1 | GN031160.1 | HW072140.1 | BD453267.1 | GM644610.1 | AX175349.1 | HI935097.1 | DL088707.1 |
| FZ436788.1 | GN030701.1 | GN031128.1 | HW071722.1 | BD413667.1 | GM637420.1 | AX173362.1 | HI661307.1 | DL088643.1 |
| FZ423436.1 | GN030573.1 | GN031096.1 | HW104339.1 | BD388119.1 | GM637388.1 | AX172944.1 | HI660937.1 | DL088579.1 |
| FZ431984.1 | GN030541.1 | GN031064.1 | HW104305.1 | BD356598.1 | GM637356.1 | AX172494.1 | HI923491.1 | DL112957.1 |
| FZ422778.1 | GN030509.1 | GN031032.1 | HW104273.1 | BD356483.1 | GM637324.1 | AX167577.1 | HI657184.1 | DL098506.1 |
| FZ422542.1 | GN030395.1 | GN031000.1 | HW071190.1 | BD350536.1 | GM637292.1 | AX167462.1 | FW420951.1 | DL098474.1 |
| FZ419483.1 | GN030317.1 | GN030968.1 | HW070628.1 | BD341686.1 | GM637260.1 | AX167415.1 | FW503009.1 | DL098442.1 |
| FZ415423.1 | GN030285.1 | GN030873.1 | HW099609.1 | BD338966.1 | GM623723.1 | AX167191.1 | FW498006.1 | DL098410.1 |
| FZ421398.1 | GN030253.1 | GN030841.1 | HW069652.1 | BD325579.1 | GM623691.1 | AX155213.1 | FW496023.1 | DL037440.1 |
| FZ412041.1 | GN030221.1 | GN030809.1 | HW067352.1 | BD319527.1 | GM623659.1 | AX145735.1 | FW504756.1 | DL037408.1 |
| FZ421006.1 | GN029835.1 | GN030777.1 | HW083198.1 | OQ693855.1 | GM623627.1 | AX145671.1 | FW500537.1 | DL037376.1 |

| | | | | | | | | |
|---|---|---|---|---|---|---|---|---|
| HH759025.1 | GN012526.1 | DM000220.1 | HW088286.1 | MK674485.1 | GM623595.1 | AX145607.1 | FW420467.1 | DL037312.1 |
| HH758993.1 | GN033537.1 | DM005064.1 | HW087889.1 | AF069380.1 | GM623563.1 | AX145575.1 | FW420435.1 | DL033109.1 |
| HH758961.1 | GN033505.1 | DM004982.1 | HW081777.1 | GN051234.1 | GM658712.1 | AX145543.1 | FW420403.1 | DL033077.1 |
| HH758929.1 | GN033473.1 | DM003483.1 | HW081689.1 | GN041646.1 | GM658680.1 | AX145479.1 | FW501721.1 | DL033045.1 |
| DL125037.1 | GN033441.1 | DL489776.1 | HW103525.1 | AH002295.2 | GM658648.1 | AX145447.1 | HH833906.1 | DL033013.1 |
| DL125005.1 | GN033409.1 | GM996604.1 | HV585092.1 | AF509586.1 | GM658616.1 | AX145415.1 | FW383155.1 | DL032981.1 |
| DL124973.1 | GN033377.1 | GM993556.1 | HV588895.1 | M18417.1 | GM658552.1 | AX145382.1 | HH932408.1 | DL032949.1 |
| DL139752.1 | GN033345.1 | DL114116.1 | HV584788.1 | AY589054.1 | GM651360.1 | AX145350.1 | HH931883.1 | DL024935.1 |
| DL124926.1 | GN033313.1 | DL114084.1 | HV579126.1 | AY157618.1 | GM651328.1 | AX145318.1 | HH925734.1 | DL024903.1 |
| DL124894.1 | GN033281.1 | DL114052.1 | HV579007.1 | AF003710.1 | GM651296.1 | AX145286.1 | HH821857.1 | DL024871.1 |
| DL120773.1 | GN033217.1 | DL108949.1 | HV599526.1 | HW408837.1 | GM644401.1 | AX145254.1 | HH822012.1 | DL024839.1 |
| DL120741.1 | GN033185.1 | DL099609.1 | HV592595.1 | HW408805.1 | GM644369.1 | AX145222.1 | HH821970.1 | DL024807.1 |
| DL116210.1 | GN033057.1 | DL099577.1 | HV588517.1 | HW408773.1 | GM644337.1 | AX145190.1 | FW379607.1 | DL024775.1 |
| DL116178.1 | GN033025.1 | DL099545.1 | HV592490.1 | HW408741.1 | GM644305.1 | AX145158.1 | FW379261.1 | DL021499.1 |
| DL116146.1 | GN032897.1 | DL028618.1 | HV592421.1 | HW408709.1 | GM644273.1 | AX145126.1 | FW377942.1 | DL021467.1 |
| DL116114.1 | GN032865.1 | DL027867.1 | HV577341.1 | HW408677.1 | GM644241.1 | AX145094.1 | FW376126.1 | DL021435.1 |
| DL116050.1 | GN032833.1 | DL027835.1 | HV601507.1 | HW408618.1 | GM637243.1 | AX145062.1 | FW381288.1 | DL021403.1 |
| DL120574.1 | GN032801.1 | DL027803.1 | HV581982.1 | HW408586.1 | GM637211.1 | AX145030.1 | FW375462.1 | DL017197.1 |
| DL120542.1 | GN032769.1 | DL027771.1 | HV601357.1 | HW408554.1 | GM637179.1 | AX144998.1 | FW375236.1 | DL017165.1 |
| DL116011.1 | GN032738.1 | DL023588.1 | HV601080.1 | HW408522.1 | GM637147.1 | AX144966.1 | FW380416.1 | DL017133.1 |
| DL115979.1 | GN032706.1 | DL015882.1 | HV589981.1 | HW408490.1 | GM637115.1 | AX144934.1 | FW369630.1 | DL017101.1 |
| DL115915.1 | GN032674.1 | DL015850.1 | HV585648.1 | HW408427.1 | GM637083.1 | GM008869.1 | FW379805.1 | DL017069.1 |
| DL115883.1 | GN032641.1 | DL047624.1 | HV570593.1 | HW408395.1 | GM637051.1 | GM005890.1 | HH736085.1 | DL017037.1 |
| DL115851.1 | GN032609.1 | DJ003328.1 | HV569026.1 | HW408363.1 | GM624898.1 | GM685528.1 | FW345117.1 | DL012776.1 |
| DL106493.1 | GN032577.1 | DD491139.1 | HV565952.1 | HW408331.1 | GM624866.1 | GM003579.1 | FW344696.1 | DL012744.1 |
| DL106461.1 | GN032482.1 | CS673595.1 | HV573877.1 | HW408299.1 | GM632649.1 | FB983215.1 | HC924196.1 | DL012712.1 |
| DL106429.1 | GN032450.1 | E00910.1 | HV560150.1 | HW408235.1 | GM632581.1 | FB983163.1 | HC923002.1 | DL012680.1 |
| DL106359.1 | GN032418.1 | E00253.1 | HV030246.1 | HW408203.1 | GM632609.1 | FB754284.1 | HC921527.1 | DL012648.1 |
| DL124682.1 | GN032386.1 | E00055.1 | HV037881.1 | HW408171.1 | GM658459.1 | GM618786.1 | HC920689.1 | DL012616.1 |
| DL124650.1 | GN032353.1 | DD087931.1 | HV037705.1 | HW390762.1 | GM658339.1 | FB726113.1 | HD063947.1 | DL049436.1 |
| DL124618.1 | GN032321.1 | DD100894.1 | HV037673.1 | HW390730.1 | GM651147.1 | FB725960.1 | HD063516.1 | DL049404.1 |
| DL124586.1 | GN032289.1 | DD112414.1 | HV031609.1 | HW399712.1 | GM651115.1 | CS695302.1 | HD052875.1 | DL049372.1 |
| DL096921.1 | GN032257.1 | DD142882.1 | FZ435999.1 | HW399342.1 | GM651083.1 | DL176590.1 | HD050992.1 | DL049340.1 |
| DL096889.1 | GN032225.1 | DD132509.1 | FZ435784.1 | HW399263.1 | GM651051.1 | DL176515.1 | HC920309.1 | DL049308.1 |
| DL096793.1 | GN032193.1 | DD158424.1 | FZ430263.1 | HW392232.1 | GM651019.1 | DL176454.1 | HD065418.1 | DL049276.1 |
| DL096761.1 | GN032161.1 | DD154867.1 | FZ429837.1 | HW391549.1 | GM650987.1 | DL176370.1 | HD058861.1 | DL049244.1 |
| DL108736.1 | GN032129.1 | DD152398.1 | FZ437385.1 | HW408869.1 | GM644156.1 | DL176297.1 | HD057684.1 | DL049213.1 |
| DL108704.1 | GN032097.1 | DD097013.1 | FZ423912.1 | HW382532.1 | GM644124.1 | DL181465.1 | HD062423.1 | DL049149.1 |
| DL104058.1 | GN032065.1 | DD139897.1 | GM660350.1 | HW390488.1 | GM644092.1 | DL181433.1 | HD055946.1 | DL049117.1 |
| DL099524.1 | GN032033.1 | DD082057.1 | GM653163.1 | HW390447.1 | GM644060.1 | DL181372.1 | M24508.1 | DL049085.1 |
| DL099492.1 | GN032001.1 | DD081768.1 | GM653131.1 | HW390301.1 | GM644028.1 | DL174627.1 | M81126.1 | DL049053.1 |
| DL099460.1 | GN031969.1 | DD147801.1 | GM653099.1 | HW389600.1 | GM636902.1 | DL174589.1 | M60420.1 | DL012577.1 |
| DL099428.1 | GN031937.1 | DD147769.1 | GM653067.1 | HW389515.1 | GM636870.1 | DL164126.1 | HC918540.1 | DL012545.1 |
| DL099396.1 | GN031905.1 | DD147737.1 | GM653035.1 | HW388654.1 | GM632462.1 | DL176780.1 | HC917448.1 | HW261319.1 |
| DL099364.1 | GN031873.1 | DD147705.1 | GM653003.1 | HW388308.1 | GM632430.1 | DL176700.1 | FW335024.1 | HW261287.1 |
| DL099332.1 | GN031841.1 | DD147673.1 | GM639009.1 | HW387486.1 | GM632398.1 | FB355786.1 | FW341969.1 | HW261255.1 |
| DL093322.1 | DL101811.1 | DD080966.1 | GM638977.1 | HW387417.1 | GM653957.1 | FB344633.1 | FW337199.1 | HW261223.1 |

| | | | | | | | | |
|---|---|---|---|---|---|---|---|---|
| DL093290.1 | DL095217.1 | DD138776.1 | GM638945.1 | HW387385.1 | GM653925.1 | FB359897.1 | FW341149.1 | HW261159.1 |
| DL089547.1 | DL111859.1 | DD137754.1 | GM638913.1 | HW387299.1 | GM653893.1 | FB342772.1 | FW335996.1 | HW261127.1 |
| DL089515.1 | DL111827.1 | DD136987.1 | GM638881.1 | HW387267.1 | GM653861.1 | DL088093.1 | HC889767.1 | HW261095.1 |
| DL089483.1 | DL111795.1 | DD132312.1 | GM626170.1 | HW387143.1 | GM653785.1 | DL088061.1 | HC868186.1 | HW261031.1 |
| DL089451.1 | DL111731.1 | DD080705.1 | GM652939.1 | HW386633.1 | GM646756.1 | DL088029.1 | HC887861.1 | HW260999.1 |
| DL089387.1 | DL128878.1 | DD064461.1 | GM652907.1 | HW386601.1 | GM646724.1 | DL087997.1 | HC887342.1 | HW260967.1 |
| DL093153.1 | DL128843.1 | AX528129.1 | GM652875.1 | HW386569.1 | GM646692.1 | DL091938.1 | HC883724.1 | HW260903.1 |
| DL113617.1 | DL128746.1 | AX525184.1 | GM652843.1 | HW386537.1 | GM646660.1 | DL091906.1 | HB648676.1 | HW260871.1 |
| DL113585.1 | DL111657.1 | AX523928.1 | GM652811.1 | HW386505.1 | GM639825.1 | DL091874.1 | HB647049.1 | HW260839.1 |
| DL113553.1 | DL111593.1 | AX179510.1 | GM652778.1 | HW386473.1 | GM639793.1 | DL091842.1 | HB645740.1 | HW260807.1 |
| DL113521.1 | DL097045.1 | AX179450.1 | GM638789.1 | HW386377.1 | GM639761.1 | DL091810.1 | HB645708.1 | HW260775.1 |
| DL113457.1 | DL092349.1 | AX164059.1 | GM638757.1 | HW386313.1 | GM639729.1 | DL091778.1 | HB645674.1 | HW260743.1 |
| DL108560.1 | DL092317.1 | AX028004.1 | GM626114.1 | HW386281.1 | GM639697.1 | DL102899.1 | HB855096.1 | HW260711.1 |
| DL103762.1 | DL092285.1 | A28441.1 | GM626082.1 | HW386249.1 | HV760610.1 | DL102867.1 | HB858481.1 | HW260647.1 |
| DL103730.1 | DL092253.1 | AF430205.1 | GM626050.1 | HW386153.1 | HV766089.1 | DL102803.1 | HB856465.1 | HW260615.1 |
| DL093146.1 | DL092189.1 | AF430173.1 | GM626018.1 | HW386089.1 | HV766041.1 | DL102771.1 | HB855984.1 | HW260583.1 |
| DL093114.1 | DL088414.1 | AX512213.1 | GM625986.1 | HW386057.1 | HV509439.1 | DL102739.1 | HB855891.1 | HW260551.1 |
| DL093082.1 | DL088382.1 | AX497567.1 | GM625954.1 | HW385993.1 | HV508622.1 | DL107615.1 | DM179531.1 | HW260487.1 |
| DL089275.1 | GN031967.1 | AX496839.1 | GM625928.1 | HW385961.1 | HV508590.1 | DL107519.1 | DM178928.1 | HW260455.1 |
| DL089243.1 | GN031935.1 | BD140206.1 | GM037633.1 | HW383110.1 | HV508558.1 | DL107491.1 | DM170850.1 | HW260423.1 |
| DL125934.1 | GN031903.1 | BD137654.1 | FB775381.1 | HV758692.1 | HV508466.1 | DL107459.1 | DM170817.1 | HW260391.1 |
| DL125902.1 | GN031871.1 | BD135957.1 | FB765894.1 | HV758660.1 | HV512604.1 | DL107395.1 | DM178264.1 | HW260359.1 |
| DL125870.1 | GN031839.1 | BD133465.1 | FB764703.1 | HV755102.1 | HV512268.1 | DL102590.1 | DM188007.1 | HW260327.1 |
| DL125838.1 | GN031807.1 | BD131921.1 | FB764101.1 | HV745872.1 | HV512204.1 | DL102558.1 | DM187969.1 | HW260295.1 |
| DL125806.1 | GN031742.1 | BD131385.1 | FB747029.1 | HV752291.1 | HV512172.1 | DL102526.1 | DM187711.1 | HW260263.1 |
| DL125774.1 | GN031709.1 | BD130782.1 | FB744532.1 | HV748580.1 | HV512140.1 | DL098016.1 | DM177655.1 | HW260231.1 |
| DL122564.1 | GN031677.1 | BD130749.1 | FB743977.1 | HV748491.1 | HV504948.1 | DL097984.1 | HB491785.1 | HW260199.1 |
| DL122532.1 | GN031645.1 | BD130716.1 | DL119791.1 | HV748447.1 | HV504916.1 | DL097952.1 | HB489484.1 | HW260167.1 |
| DL118118.1 | GN031613.1 | BD128431.1 | DL119759.1 | HV756585.1 | HV504884.1 | DL112642.1 | HB488752.1 | HW260135.1 |
| DL118086.1 | GN031549.1 | BD091313.1 | DL119727.1 | HV753829.1 | HV504852.1 | DL112610.1 | DM164071.1 | HW260071.1 |
| DL118054.1 | GN031517.1 | BD083864.1 | DL119695.1 | HV747703.1 | HV504820.1 | DL112578.1 | DM164003.1 | HW259975.1 |
| DL118022.1 | GN031485.1 | BD081726.1 | DL119663.1 | HV747618.1 | HV504739.1 | DL112546.1 | DM163970.1 | HW259943.1 |
| DL113333.1 | GN031420.1 | BD081530.1 | DL119631.1 | HV747480.1 | HV504707.1 | DL112514.1 | DM163257.1 | HW259911.1 |
| DL113301.1 | GN031388.1 | BD080153.1 | DL123892.1 | HV750799.1 | HV504675.1 | DL112482.1 | DM162316.1 | HW259774.1 |
| DL108468.1 | GN031356.1 | BD074962.1 | DL119489.1 | HV753608.1 | HV504643.1 | DL097829.1 | DM162150.1 | HW259473.1 |
| DL108436.1 | GN031324.1 | BD074814.1 | DL119457.1 | HV756022.1 | HV504611.1 | DL097765.1 | AX772725.1 | HW259045.1 |
| DL108358.1 | GN031291.1 | BD070724.1 | DL119425.1 | HV755990.1 | HV492399.1 | DL095738.1 | AX770681.1 | HW258912.1 |
| DL108340.1 | GN031259.1 | BD069510.1 | DL123736.1 | HV753491.1 | HV504204.1 | DL095706.1 | AX769740.1 | HW258846.1 |
| DL108308.1 | GN031227.1 | BD016710.1 | DL123704.1 | HV708096.1 | HV504168.1 | DL095674.1 | AX766185.1 | HW257426.1 |
| DL103670.1 | GN031195.1 | E50486.1 | DL123672.1 | HV744178.1 | HV504136.1 | DL095642.1 | AX765771.1 | HW257330.1 |
| DL103638.1 | GN031164.1 | BD014209.1 | DL123640.1 | HV744143.1 | HV504104.1 | DL095610.1 | AX754997.1 | HW257298.1 |
| DL045627.1 | GN031130.1 | BD014169.1 | DL123608.1 | HV743907.1 | HV504072.1 | DL095578.1 | AX752631.1 | HW257266.1 |
| DL045595.1 | GN031098.1 | BD013763.1 | DL123576.1 | HV743872.1 | HV504040.1 | DL095546.1 | AX751524.1 | HW257234.1 |
| DL045563.1 | GN031066.1 | HW336132.1 | DL119386.1 | HV743318.1 | HV504008.1 | DL117383.1 | BD185258.1 | HW257202.1 |
| DL041355.1 | GN031034.1 | HW328829.1 | DL119354.1 | HV743278.1 | HV503976.1 | DL117351.1 | AX743923.1 | HW257042.1 |
| DL041323.1 | GN031002.1 | HW328611.1 | DL119322.1 | HV743240.1 | HV503944.1 | DL117319.1 | AX175108.1 | HW257010.1 |
| DL041291.1 | GN030970.1 | HW328463.1 | DL114830.1 | HV742503.1 | HV503912.1 | DL117287.1 | AX173088.1 | HW256882.1 |

| | | | | | | | | |
|---|---|---|---|---|---|---|---|---|
| DL037404.1 | GN030939.1 | HW328304.1 | DL114798.1 | HV705074.1 | HV503880.1 | DL110015.1 | AX172870.1 | HW256850.1 |
| DL037372.1 | GN030907.1 | HW319167.1 | DL114766.1 | HV704623.1 | HV503816.1 | DL109983.1 | AX172469.1 | HW256818.1 |
| DL037340.1 | GN030875.1 | HW335669.1 | DL114734.1 | HV704456.1 | HV501009.1 | DL109951.1 | AX168174.1 | HW256786.1 |
| DL021495.1 | GN030843.1 | HW328251.1 | DL114702.1 | HV744628.1 | HV491273.1 | DL109769.1 | AX167075.1 | HW256754.1 |
| DL021463.1 | GN030811.1 | HW318523.1 | DL114670.1 | HV703547.1 | HV491104.1 | DL109705.1 | AX166287.1 | HW256722.1 |
| DL021431.1 | GN030779.1 | HW314334.1 | DL130737.1 | DD228691.1 | HV503776.1 | DL105099.1 | AX155095.1 | HW251081.1 |
| DL017193.1 | DM008013.1 | HW314116.1 | DL114643.1 | DD228658.1 | HV503648.1 | DL105003.1 | AY035211.1 | HW251025.1 |
| DL017129.1 | DM000224.1 | HW313630.1 | DL123472.1 | DD228602.1 | HV503524.1 | DL104971.1 | AX148745.1 | HW250350.1 |
| DL017097.1 | DM005066.1 | HW311852.1 | DL123440.1 | DD228450.1 | HV503492.1 | DL104939.1 | AX147413.1 | HW249719.1 |
| DL017065.1 | DM004984.1 | HW311226.1 | DL123408.1 | DD227394.1 | HV503460.1 | DL104907.1 | AX146311.1 | HW241207.1 |
| AX796864.1 | DM003485.1 | HW308347.1 | DL123376.1 | DD227152.1 | HV503428.1 | DL100493.1 | AX145692.1 | HW247848.1 |
| AX796750.1 | GM993558.1 | HW307816.1 | DL119122.1 | DD225903.1 | HV503396.1 | DL100461.1 | AX145660.1 | HW240906.1 |
| AX795624.1 | GM992366.1 | HW307746.1 | DL119090.1 | DD224772.1 | HV503364.1 | DL100429.1 | AX145628.1 | HW042077.1 |
| AX795439.1 | FB985401.1 | HW307714.1 | DL119058.1 | DD224218.1 | HV503332.1 | DL100397.1 | AX145596.1 | HW042045.1 |
| BD189468.1 | FB983150.1 | HW307682.1 | DL119026.1 | DD223914.1 | HV503300.1 | DL100365.1 | AX145564.1 | HW042016.1 |
| BD188127.1 | GM879308.1 | HW315840.1 | DL114630.1 | DD234131.1 | HV503268.1 | DL100333.1 | AX145532.1 | HW041969.1 |
| AX794206.1 | GM680716.1 | HW315574.1 | DL114598.1 | DD233506.1 | HV503236.1 | DL124146.1 | AX145500.1 | HW041937.1 |
| AX786787.1 | FB722589.1 | HW315541.1 | DL114566.1 | DD232716.1 | HV455444.1 | DL124114.1 | AX145468.1 | HW041898.1 |
| AX786755.1 | GM042052.1 | HW315181.1 | DL114534.1 | DD231506.1 | HV503181.1 | DL124082.1 | AX145436.1 | HW041839.1 |
| AX768201.1 | FB745015.1 | HW315149.1 | DL114502.1 | DD231450.1 | HV503149.1 | DL124050.1 | AX145404.1 | HW049463.1 |
| AX766880.1 | GM603467.1 | HW069965.1 | DL114470.1 | DD231418.1 | HV503085.1 | DL123986.1 | AX145339.1 | HW049391.1 |
| AX766730.1 | GM601058.1 | HW069801.1 | DL109573.1 | CS277355.1 | HV503053.1 | DL119796.1 | AX145307.1 | HW049360.1 |
| AX766382.1 | FB721294.1 | HW084957.1 | DL104903.1 | A05963.1 | HV503021.1 | DL119764.1 | AX145275.1 | HW049296.1 |
| AX765959.1 | FB717571.1 | HW084918.1 | DL104871.1 | A02514.1 | HV502989.1 | DL119732.1 | AX145243.1 | HW049264.1 |
| AX239701.1 | FB702164.1 | HV986647.1 | DL104839.1 | DD217684.1 | HV502957.1 | DL119700.1 | AX145211.1 | HW049178.1 |
| AX236449.1 | FB677463.1 | HV963694.1 | DL104807.1 | DD219546.1 | HV502925.1 | DL119668.1 | AX145179.1 | HW041803.1 |
| AX235773.1 | GM061220.1 | HV963326.1 | DL104743.1 | DD221392.1 | HV502893.1 | DL119636.1 | AX145147.1 | HW041771.1 |
| AX235236.1 | DL258516.1 | HV965291.1 | DL095884.1 | DD220929.1 | HV502861.1 | AX363238.1 | AX145115.1 | HW054938.1 |
| AX225209.1 | DL257815.1 | HV956091.1 | DL095852.1 | DD213979.1 | HV502829.1 | AX359930.1 | AX145051.1 | HW041550.1 |
| AX207292.1 | DL256300.1 | HV951637.1 | DL095820.1 | DD213854.1 | HH756185.1 | AX358426.1 | AX145019.1 | HW047141.1 |
| AX203115.1 | DL241166.1 | HV951122.1 | DL095788.1 | DD213822.1 | HI401457.1 | AX357298.1 | AX144987.1 | HW054015.1 |
| AX202428.1 | DL240251.1 | HV950684.1 | DL094133.1 | BD137474.1 | HI401088.1 | AX356672.1 | AX144955.1 | HW048302.1 |
| AX195141.1 | FB748853.1 | A28333.1 | DL094101.1 | BD137172.1 | HI642912.1 | BD006811.1 | AX144922.1 | HV975312.1 |
| AX193704.1 | FB674293.1 | A26931.1 | DL094069.1 | BD136250.1 | HI641824.1 | BD002003.1 | AX144890.1 | HV985975.1 |
| AX180280.1 | DL236212.1 | A22630.1 | DL011596.1 | BD135896.1 | HI641280.1 | E54570.1 | AX144858.1 | HV985943.1 |
| AX172942.1 | DL233596.1 | A28187.1 | DL047822.1 | BD135074.1 | HI640736.1 | E55383.1 | AX144826.1 | HV985911.1 |
| AX172287.1 | DL233397.1 | A25195.1 | DL047790.1 | BD134107.1 | HI639837.1 | BD000192.1 | AX144794.1 | HV985866.1 |
| AX167413.1 | DL231386.1 | A23311.1 | DL047758.1 | BD131918.1 | HI637701.1 | E58975.1 | AX144762.1 | HV984424.1 |
| AX167189.1 | DL028684.1 | A23132.1 | DL047726.1 | BD131378.1 | HI380399.1 | AX356509.1 | AX144730.1 | HW028889.1 |
| AX166329.1 | DL028620.1 | A22411.1 | DL047694.1 | BD130848.1 | HI380326.1 | AX354692.1 | AX144698.1 | HW028622.1 |
| AX154602.1 | DL028556.1 | A18477.1 | DL047662.1 | BD130811.1 | HI379435.1 | AX353942.1 | AX144666.1 | HV986641.1 |
| AX149435.1 | DL016631.1 | A16629.1 | DL043680.1 | BD130779.1 | HI378930.1 | AX352746.1 | AX144527.1 | HV969324.1 |
| AX146696.1 | DL031394.1 | A14486.1 | DL039703.1 | BD130746.1 | HI376941.1 | AX351101.1 | AX144335.1 | HV960807.1 |
| AX145733.1 | DL020342.1 | A01970.1 | DL035919.1 | BD130713.1 | HI375923.1 | AX349125.1 | AX144271.1 | HV960572.1 |
| AX145669.1 | DL020310.1 | A21790.1 | FW368434.1 | BD130560.1 | HI375888.1 | AX348962.1 | AX144077.1 | HV960213.1 |
| AX145637.1 | DJ493876.1 | A21447.1 | FW363642.1 | BD106887.1 | HI375007.1 | AX348475.1 | AX144011.1 | HV959933.1 |
| AX145605.1 | DJ491576.1 | A20469.1 | FW363394.1 | BD105696.1 | HI370358.1 | AX347452.1 | AX143947.1 | HV959697.1 |

| | | | | | | | | |
|---|---|---|---|---|---|---|---|---|
| AX145573.1 | DL007691.1 | A19551.1 | FW367427.1 | BD096865.1 | HI369890.1 | AX347362.1 | AX143883.1 | HV964047.1 |
| AX145541.1 | DJ446843.1 | HV932702.1 | FW362935.1 | BD090944.1 | HI574577.1 | AX347290.1 | AX143819.1 | HV963869.1 |
| AX145509.1 | DJ446701.1 | HV816929.1 | FW362241.1 | BD090592.1 | HI465368.1 | AX347252.1 | AX143563.1 | GM645364.1 |
| AX145445.1 | DJ436707.1 | HV778907.1 | FW366205.1 | BD090497.1 | HI001676.1 | AX347182.1 | AX143499.1 | GM645332.1 |
| AX145380.1 | DJ445556.1 | HV778345.1 | FW351387.1 | BD085739.1 | HI001617.1 | AX345346.1 | AX143307.1 | GM645236.1 |
| AX145348.1 | DJ444639.1 | HV777430.1 | FW351175.1 | BD083025.1 | HI001575.1 | AX344642.1 | AX143243.1 | GM678659.1 |
| AX145316.1 | DJ439031.1 | HV774646.1 | FV531714.1 | BD081958.1 | HI003402.1 | AX344123.1 | AX142923.1 | GM659382.1 |
| AX145284.1 | DJ433807.1 | HV763962.1 | FV531682.1 | BD081723.1 | HI003359.1 | AX342816.1 | AX142859.1 | GM659350.1 |
| AX145252.1 | DJ438340.1 | AY657111.1 | FV531019.1 | BD081521.1 | HI003322.1 | AX342198.1 | AX142795.1 | GM652158.1 |
| AX145220.1 | DJ438305.1 | AY657079.1 | FV534868.1 | BD080713.1 | HI003283.1 | AX338574.1 | AX142539.1 | GM652126.1 |
| AX145188.1 | DJ438273.1 | AY657047.1 | FV534449.1 | BD079307.1 | HI464728.1 | AX328286.1 | AX142409.1 | GM652094.1 |
| AX145156.1 | DJ402664.1 | U25665.1 | FV534417.1 | BD077996.1 | HI464696.1 | AX327961.1 | AX142345.1 | GM652062.1 |
| AX145124.1 | DJ402628.1 | L07491.1 | AY270183.1 | BD077115.1 | HI464664.1 | AX326761.1 | AX142281.1 | GM652030.1 |
| AX145092.1 | DJ416202.1 | M21835.1 | HC456285.1 | BD075752.1 | HI463845.1 | AX323386.1 | AX141893.1 | GM645203.1 |
| AX145060.1 | DJ400821.1 | M28018.1 | HC453679.1 | BD075070.1 | HI542545.1 | AX322068.1 | AX141701.1 | GM645171.1 |
| AX145028.1 | DJ400789.1 | J02555.1 | AF156670.1 | BD074957.1 | HI463154.1 | AX306763.1 | HV955870.1 | GM645139.1 |
| AX144996.1 | DD401918.1 | J02537.1 | HC442326.1 | BD074810.1 | HI462688.1 | AX268742.1 | HV950635.1 | GM645107.1 |
| AX144964.1 | DD401886.1 | M61898.1 | HC452154.1 | BD073864.1 | HI503814.1 | AX268695.1 | HV949616.1 | GM645075.1 |
| AX144932.1 | DD401822.1 | HV549330.1 | HC448958.1 | BD070713.1 | HH732009.1 | AX258934.1 | HV947317.1 | GM645043.1 |
| AX144899.1 | DD401550.1 | HV549096.1 | FU772332.1 | BD069461.1 | HV932131.1 | AX304312.1 | HV947217.1 | GM645011.1 |
| AX144867.1 | DD401518.1 | HV549016.1 | FU761013.1 | BD062850.1 | HV936913.1 | AX299855.1 | HV940417.1 | GM638180.1 |
| AX144835.1 | DD401486.1 | HV544362.1 | FU773365.1 | BD062684.1 | HV931352.1 | AX297658.1 | HV940251.1 | GM638148.1 |
| AX144803.1 | DD401454.1 | HV543511.1 | FU759945.1 | BD057283.1 | HV936388.1 | AX283690.1 | HV945912.1 | GM638116.1 |
| AX144771.1 | CS477994.1 | HV538618.1 | FU757603.1 | BD016707.1 | HV936356.1 | AX282874.1 | HV939884.1 | GM638084.1 |
| AX144739.1 | DL105369.1 | HV538462.1 | FU756839.1 | BD015881.1 | HV930760.1 | AX282190.1 | HV936909.1 | GM651896.1 |
| AX144707.1 | DL115121.1 | HV543188.1 | HC358221.1 | BD014239.1 | HV819466.1 | AX280226.1 | HV930748.1 | GM651864.1 |
| AX144675.1 | DL109747.1 | HV541503.1 | HC358091.1 | BD014206.1 | HV803283.1 | AX279953.1 | HV936242.1 | GM651832.1 |
| AX144643.1 | DL109715.1 | HV535590.1 | HC358058.1 | BD014166.1 | HV932722.1 | AX279737.1 | HV819642.1 | GM651800.1 |
| AX144609.1 | DL109683.1 | HV516276.1 | DM164083.1 | BD013751.1 | HV932689.1 | A01178.1 | HV510347.1 | GM645001.1 |
| AX144481.1 | DL045306.1 | HV515775.1 | DM159694.1 | AX474094.1 | HV932617.1 | A05359.1 | HV510282.1 | GM644969.1 |
| AX144353.1 | DL032720.1 | HV515593.1 | DM164007.1 | AX472655.1 | HV510286.1 | A01736.1 | HV509348.1 | GM644937.1 |
| AX144223.1 | DL028616.1 | HV515561.1 | DM163973.1 | E61351.1 | HV513325.1 | A05200.1 | HV508584.1 | GM644905.1 |
| AX144031.1 | DL021270.1 | HV515465.1 | DM163304.1 | E64485.1 | HV509435.1 | A01349.1 | HV508552.1 | GM644873.1 |
| AX143901.1 | DL035298.1 | HV515433.1 | DM162321.1 | AX469475.1 | HV508703.1 | A00830.1 | HV508520.1 | GM644841.1 |
| AX143837.1 | DJ493860.1 | HV515401.1 | DM162155.1 | AX468469.1 | HV508620.1 | A00252.1 | HV512294.1 | GM638042.1 |
| AX143389.1 | DL007683.1 | HV515369.1 | HB477378.1 | AX466979.1 | HV508588.1 | M67486.1 | HV512262.1 | GM638010.1 |
| AX143133.1 | DJ446839.1 | HV515337.1 | HB476993.1 | AX464626.1 | HV508556.1 | M64439.1 | HV512230.1 | GM637978.1 |
| HW265840.1 | DJ436700.1 | HV515305.1 | HB486507.1 | AX460810.1 | HV508524.1 | M60894.1 | HV512198.1 | GM637946.1 |
| HW263751.1 | DJ445548.1 | HV515241.1 | HB486003.1 | AX458659.1 | HV508496.1 | M16782.1 | HV512166.1 | GM637914.1 |
| HW263032.1 | DJ438376.1 | FW390808.1 | HB476378.1 | AX458587.1 | HV508464.1 | M33419.1 | HV512134.1 | GM637882.1 |
| HW263000.1 | DJ433801.1 | HH928905.1 | HB474826.1 | AX458208.1 | HV512577.1 | M16239.1 | HV504765.1 | GM637850.1 |
| HW262968.1 | DJ438336.1 | HH827032.1 | HB469153.1 | AX456812.1 | HV512266.1 | LC733662.1 | DM371119.1 | GM633442.1 |
| HW262920.1 | DJ438301.1 | HH822056.1 | HB468033.1 | AX454159.1 | HV512234.1 | MF989994.1 | DM370687.1 | GM633410.1 |
| HW262583.1 | DJ438247.1 | HH821917.1 | DM152690.1 | AX454003.1 | HV512202.1 | LT799419.1 | DM370655.1 | GM633378.1 |
| HW261379.1 | DJ401879.1 | FW369983.1 | DM152548.1 | AX451642.1 | HV512170.1 | AH002283.2 | DM370623.1 | GM633346.1 |
| HW261347.1 | DJ400817.1 | FW375267.1 | HB388687.1 | AX449466.1 | HV512138.1 | K01155.1 | DM370591.1 | GM633314.1 |
| HW261315.1 | DJ400785.1 | FW375015.1 | HB387493.1 | AX449423.1 | JA660269.1 | M33731.1 | DM370559.1 | GM633282.1 |

| | | | | | | | | |
|---|---|---|---|---|---|---|---|---|
| HW261283.1 | CS450627.1 | U84737.1 | HB386574.1 | A34771.1 | HV504946.1 | AY521451.1 | DM370527.1 | GM637827.1 |
| HW261251.1 | DD359811.1 | HH755067.1 | HB385913.1 | AX443308.1 | HV504914.1 | AY061817.1 | DM370495.1 | GM637795.1 |
| HW261219.1 | DD357936.1 | FW362729.1 | HB385769.1 | AX441329.1 | HV504882.1 | AF003704.1 | DM370463.1 | GM637763.1 |
| HW261187.1 | DD355448.1 | FW349852.1 | HB384565.1 | AX440872.1 | HV504850.1 | AF003703.1 | DM367520.1 | GM637731.1 |
| HW261155.1 | A05960.1 | FW351255.1 | HA641606.1 | AX429806.1 | HV504818.1 | KP784700.1 | GM624034.1 | GM637699.1 |
| HW261123.1 | A04086.1 | HD122558.1 | HA641542.1 | AX428237.1 | HV504769.1 | JX912274.1 | GM624002.1 | GM633259.1 |
| HW261091.1 | CS446149.1 | HD122296.1 | HA641347.1 | A20466.1 | HV504737.1 | HW408612.1 | GM623970.1 | GM633227.1 |
| HW261059.1 | CS438909.1 | HD122272.1 | HA641283.1 | A15657.1 | HV504705.1 | HW408580.1 | GM623938.1 | GM633195.1 |
| HW261027.1 | CS434840.1 | FW345541.1 | HA641141.1 | A18390.1 | HV504673.1 | HW408548.1 | GM625048.1 | GM633163.1 |
| HW260963.1 | DD347397.1 | HC295241.1 | HA641062.1 | A17042.1 | HV504641.1 | HW408484.1 | GM625016.1 | GM633131.1 |
| HW260931.1 | DD353762.1 | HC292098.1 | DM110294.1 | A16031.1 | HV504609.1 | HW408453.1 | GM658913.1 | GM633099.1 |
| HW260867.1 | DD349187.1 | HC291929.1 | DM109928.1 | DL094981.1 | HV492397.1 | HW408357.1 | GM658881.1 | GM625520.1 |
| HW260835.1 | CS424574.1 | HC291666.1 | DM094881.1 | DL094949.1 | HV504202.1 | HW390756.1 | GM658849.1 | GM625488.1 |
| HW260803.1 | CS410877.1 | HC293870.1 | DM103434.1 | DL106667.1 | HV455586.1 | HW390724.1 | GM658817.1 | GM625456.1 |
| HW260771.1 | CS414816.1 | HC293690.1 | DM102892.1 | DL106635.1 | HV504166.1 | HW399543.1 | GM637412.1 | GM625424.1 |
| HW260739.1 | CS410148.1 | HC290737.1 | GN368413.1 | DL106603.1 | HV504134.1 | HW399335.1 | GM637380.1 | GM625392.1 |
| DL010141.1 | DD327008.1 | HC293188.1 | GN367653.1 | DL106571.1 | HV504102.1 | HW399234.1 | GM637316.1 | GM625360.1 |
| DL010109.1 | DD326764.1 | HC292996.1 | GN367115.1 | DL106539.1 | HV504070.1 | HW382299.1 | GM637284.1 | GM711148.1 |
| DL010077.1 | AX962907.1 | HC289854.1 | GN360092.1 | DL106507.1 | HV504038.1 | HW381545.1 | GM637252.1 | GM651752.1 |
| DL010045.1 | AX951545.1 | HC202652.1 | GN356161.1 | DL128704.1 | HV504006.1 | HW390528.1 | GM623651.1 | GM651720.1 |
| DL010013.1 | AX938919.1 | HC196662.1 | GN360051.1 | DL128670.1 | HV503942.1 | HW390482.1 | GM623619.1 | GM651688.1 |
| DL014515.1 | AX934532.1 | HC194271.1 | GM658159.1 | DL128636.1 | HV503878.1 | HW390419.1 | GM623587.1 | GM651656.1 |
| DL014483.1 | AX923421.1 | HC193426.1 | GM658077.1 | DL128604.1 | HV503846.1 | HW390339.1 | GM623555.1 | GM644792.1 |
| DL014451.1 | AX923388.1 | HC187665.1 | GM658045.1 | DL128551.1 | HV503814.1 | HW389509.1 | GM644233.1 | GM644760.1 |
| DL009865.1 | AX839013.1 | HC089562.1 | GM658013.1 | DL125074.1 | HV501006.1 | HW388917.1 | GM637139.1 | GM644728.1 |
| DL009833.1 | AX824352.1 | HC089530.1 | GM657981.1 | DL125042.1 | HV491271.1 | HW387859.1 | GM637107.1 | GM637602.1 |
| DL009801.1 | AX823786.1 | HC089498.1 | GM657949.1 | DL125010.1 | HV503774.1 | HW387474.1 | GM637075.1 | GM637570.1 |
| DL009769.1 | AX814944.1 | HC089466.1 | GM650757.1 | DL124978.1 | HV503710.1 | HW387047.1 | GM632673.1 | GM642424.1 |
| DL009545.1 | AX814422.1 | HC089434.1 | GM650725.1 | DL120953.1 | HV503678.1 | HV312311.1 | GM632641.1 | GM635242.1 |
| DL009513.1 | AX814390.1 | HC089401.1 | GM650693.1 | DL120921.1 | HV503646.1 | HV317227.1 | GM741931.1 | GM635210.1 |
| DL009481.1 | AX805909.1 | HC089369.1 | GM643701.1 | DL120889.1 | HV503522.1 | HV226856.1 | GM658451.1 | GM635178.1 |
| DL009449.1 | AX799556.1 | HC089337.1 | GM643669.1 | DL120857.1 | HV503458.1 | HV214563.1 | GM619002.1 | GM635114.1 |
| DL009417.1 | AX798080.1 | HC089304.1 | GM636640.1 | DL120825.1 | HV503426.1 | HV217622.1 | GM047655.1 | GM635082.1 |
| DL030142.1 | AX797346.1 | HC089272.1 | GM636608.1 | DL139757.1 | HV503394.1 | DL095791.1 | GM640907.1 | GM630276.1 |
| DL018983.1 | AX663062.1 | HC087855.1 | GM636576.1 | DL124931.1 | HV503362.1 | DL094136.1 | GM640875.1 | GM630212.1 |
| DL018951.1 | AX472453.1 | HC086296.1 | GM636544.1 | DL124899.1 | HV503330.1 | DL094104.1 | GM640843.1 | GM630180.1 |
| DL018919.1 | BD194473.1 | AY774969.1 | GM636512.1 | DL124867.1 | HV503298.1 | DL094072.1 | GM628489.1 | GM630148.1 |
| DL018887.1 | BD188790.1 | AY774922.1 | GM636480.1 | DL124835.1 | HV503266.1 | DL090265.1 | GM047101.1 | GM630116.1 |
| DL018855.1 | BD187487.1 | DL101949.1 | GM632072.1 | DL124803.1 | HV455364.1 | DL090233.1 | GM654788.1 | GM737383.1 |
| DL018823.1 | AX241510.1 | DL097503.1 | GM631912.1 | DL124771.1 | HV503179.1 | DL090201.1 | GM627961.1 | GM635047.1 |
| DL034330.1 | AX241478.1 | DL097439.1 | GM643499.1 | DL120778.1 | HV503147.1 | DL090169.1 | GM627929.1 | GM635015.1 |
| DL009382.1 | AX241446.1 | DL097407.1 | GM643467.1 | DL120746.1 | HV503115.1 | DL086164.1 | GM654745.1 | GM634983.1 |
| DL009350.1 | AY044146.1 | DL097375.1 | GM643434.1 | DL116215.1 | HV503083.1 | DL086132.1 | FB775375.1 | GM634951.1 |
| DL009318.1 | AX211860.1 | DL097343.1 | GM636445.1 | DL116183.1 | HV503051.1 | DL086100.1 | FB766217.1 | GM634919.1 |
| DL009254.1 | AX207310.1 | DL121362.1 | GM631845.1 | DL116151.1 | HI002901.1 | DL086068.1 | FB764691.1 | GM634887.1 |
| DL009222.1 | AX207114.1 | DL121330.1 | GM631813.1 | DL116119.1 | HI001264.1 | DL086036.1 | FB764062.1 | GM629953.1 |
| DL018680.1 | AX202582.1 | DL121298.1 | GM631781.1 | DL116087.1 | HI001206.1 | DL086004.1 | FB743970.1 | GM629921.1 |

| | | | | | | | | |
|---|---|---|---|---|---|---|---|---|
| DL018648.1 | AX202439.1 | DL121266.1 | GM622850.1 | DL116055.1 | HI001153.1 | DL114438.1 | FB743923.1 | GM642407.1 |
| DL009164.1 | AX193976.1 | DL121234.1 | GM657903.1 | DL143519.1 | HI001053.1 | DL114406.1 | FB743891.1 | GM642235.1 |
| DL014220.1 | AX181916.1 | DL121202.1 | GM657871.1 | DL120579.1 | HI001016.1 | DL114374.1 | FB743859.1 | GM642271.1 |
| DL014188.1 | AX179624.1 | DL101896.1 | HV515484.1 | DL120515.1 | HI000974.1 | DL114342.1 | FB743805.1 | GM656338.1 |
| DL014156.1 | AX174833.1 | DL123618.1 | HV515452.1 | DL120483.1 | HI002875.1 | DL109349.1 | FB761837.1 | GM656306.1 |
| DL014124.1 | AX172953.1 | DL123586.1 | HV515420.1 | DL120451.1 | HI002840.1 | DL109317.1 | FB730014.1 | GM656274.1 |
| DL014092.1 | AX151024.1 | DL119396.1 | HV515356.1 | DL120419.1 | HI002802.1 | DL109285.1 | GM036248.1 | GM656210.1 |
| CS574791.1 | AX148295.1 | DL119364.1 | HV515324.1 | DL116016.1 | HI002743.1 | DL104551.1 | GM715397.1 | GM656178.1 |
| CS573095.1 | AX145712.1 | DL119332.1 | HV515292.1 | DL115984.1 | HI002707.1 | DL104519.1 | GM889349.1 | HI415917.1 |
| CS572124.1 | AX145680.1 | DL119300.1 | HV515260.1 | DL115920.1 | HI002659.1 | DL100041.1 | GM008849.1 | HI415885.1 |
| CS576685.1 | AX145648.1 | DL119268.1 | HV515228.1 | DL115888.1 | HI002623.1 | DL093935.1 | GM867630.1 | HI415853.1 |
| CS569630.1 | AX145616.1 | DL119236.1 | HV514404.1 | DL115856.1 | HI002586.1 | DL093903.1 | FB983605.1 | HI415788.1 |
| CS545389.1 | AX145584.1 | DL114840.1 | HV511158.1 | DL106466.1 | HI002525.1 | DL093871.1 | FB983184.1 | HI415756.1 |
| CS544818.1 | AX145552.1 | DL114808.1 | HV514044.1 | DL106434.1 | HI001478.1 | DL090064.1 | GM865632.1 | HI414043.1 |
| CS561181.1 | AX145488.1 | DL114776.1 | HV510351.1 | DL106354.1 | HI004541.1 | DL090032.1 | FB727100.1 | HI414011.1 |
| CS561006.1 | AX145456.1 | DL114744.1 | HV510318.1 | DL125907.1 | HI004413.1 | DL090000.1 | FB725750.1 | HI413448.1 |
| CS560459.1 | AX145424.1 | DL114712.1 | FW496673.1 | DL125875.1 | HI002395.1 | DL042147.1 | GM968177.1 | HI516610.1 |
| CS559242.1 | AX145391.1 | DL114680.1 | FW503287.1 | DL125843.1 | HI002315.1 | DL042115.1 | GM616085.1 | HI516538.1 |
| DD419840.1 | AX145359.1 | DL114648.1 | HI653931.1 | DL125811.1 | HI000518.1 | DL042083.1 | GM731832.1 | HI516132.1 |
| DD420057.1 | AX145327.1 | DL127160.1 | HI653869.1 | DL125779.1 | HI066249.1 | DL038138.1 | GM879661.1 | HI516086.1 |
| DD433776.1 | AX145295.1 | DL141561.1 | HI653801.1 | DL122537.1 | HI180162.1 | DL033704.1 | GM723329.1 | HI547618.1 |
| CS542030.1 | AX145263.1 | DL123514.1 | HI653708.1 | DL122505.1 | HI178198.1 | DL033672.1 | FB712048.1 | HI284305.1 |
| CS538844.1 | AX145231.1 | DL123482.1 | HI653521.1 | DL122473.1 | HI202834.1 | DL033640.1 | GM723192.1 | HI284273.1 |
| CS537968.1 | AX145199.1 | DL123450.1 | HI653467.1 | DL122441.1 | HI214562.1 | DL029536.1 | DL096584.1 | HI574526.1 |
| CS534837.1 | AX145167.1 | DL123418.1 | HI653406.1 | DL122409.1 | HI214530.1 | DL029504.1 | DL096552.1 | HI574494.1 |
| CS502741.1 | AX145135.1 | DL123386.1 | HI653357.1 | DL122377.1 | HI214432.1 | DL029472.1 | DL094724.1 | HI574462.1 |
| CS502709.1 | AX145103.1 | DL119196.1 | HI651903.1 | DL118123.1 | HI213001.1 | DL046106.1 | DL094692.1 | HI574430.1 |
| CS502673.1 | AX145071.1 | DL119164.1 | HI650455.1 | DL118091.1 | HI212949.1 | DL041960.1 | DL090949.1 | HI574398.1 |
| CS502609.1 | AX145039.1 | DL119132.1 | HH807301.1 | DL118059.1 | HI203500.1 | DL041928.1 | DL090917.1 | HI571452.1 |
| CS502403.1 | AX145007.1 | DL119100.1 | HH802826.1 | DL118027.1 | HI210995.1 | DL041896.1 | DL086981.1 | HI561761.1 |
| CS502365.1 | GN067951.1 | DL119068.1 | HH794948.1 | DL113338.1 | HI210963.1 | DL046007.1 | DL086949.1 | HI574375.1 |
| CS501242.1 | GN067932.1 | DL119036.1 | HH794716.1 | DL113306.1 | HI565886.1 | DL045975.1 | DL086917.1 | HI573727.1 |
| CS500060.1 | GN067868.1 | DL114640.1 | HH794199.1 | DL113274.1 | HI473008.1 | DL049713.1 | DL105870.1 | HI553828.1 |
| CS498526.1 | GN067804.1 | DL114608.1 | HH791208.1 | DL108473.1 | HI472904.1 | DL049681.1 | DL105838.1 | HI585324.1 |
| DD418613.1 | GN067776.1 | DL114576.1 | HH779674.1 | DL108441.1 | FW343873.1 | DL045673.1 | DL105806.1 | HI588360.1 |
| DD418578.1 | GN067744.1 | DL114544.1 | HH777917.1 | DL108353.1 | FW343606.1 | DL041858.1 | DL096523.1 | HI284314.1 |
| DD412329.1 | GN067680.1 | DL114512.1 | HH773133.1 | DL108401.1 | FW343718.1 | DL041826.1 | DL096491.1 | HI636972.1 |
| DD411471.1 | GN067616.1 | DL114480.1 | HH820912.1 | DL108345.1 | FW342867.1 | DL041794.1 | DL096459.1 | HI636933.1 |
| DD414318.1 | GN063780.1 | DL114448.1 | HH820314.1 | DL108313.1 | HC924245.1 | DL041698.1 | DL115511.1 | HI001452.1 |
| DD410759.1 | GN060844.1 | DL109551.1 | HH759208.1 | DL103643.1 | HC923008.1 | DL037779.1 | DL115479.1 | HI001397.1 |
| DD417722.1 | GN052436.1 | DL109519.1 | HH759176.1 | DL099101.1 | HC921538.1 | DL037747.1 | DL115447.1 | HI001356.1 |
| CS493064.1 | GN060527.1 | DL109487.1 | HH759112.1 | DL099069.1 | HC920693.1 | DL049608.1 | DL105695.1 | HI001322.1 |
| CS491166.1 | GN060090.1 | DL104881.1 | HH759080.1 | DL099037.1 | HD063949.1 | DL045113.1 | DL105663.1 | HI003205.1 |
| CS490626.1 | GN052393.1 | DL104849.1 | HH759048.1 | DL099005.1 | HD052877.1 | DL045081.1 | DL105631.1 | HI003143.1 |
| CS489157.1 | GN059752.1 | DL104817.1 | HH759016.1 | DL098973.1 | HD050994.1 | DL037082.1 | DL105599.1 | HI003086.1 |
| DD153644.1 | GN051182.1 | DL104785.1 | HH758984.1 | DL098941.1 | HD048630.1 | DL037050.1 | DL100794.1 | HI003047.1 |
| DD152590.1 | GN046492.1 | DL104753.1 | HH758952.1 | DL092899.1 | HC920314.1 | DL037018.1 | DL100762.1 | HI003006.1 |

| | | | | | | | | |
|---|---|---|---|---|---|---|---|---|
| DD151834.1 | DM014246.1 | DL104721.1 | HC877786.1 | DL092867.1 | HD033287.1 | DL024545.1 | CQ890959.1 | HI002944.1 |
| DD096955.1 | DM025627.1 | DL095926.1 | FW310245.1 | DL092835.1 | HD065420.1 | DL024513.1 | CQ888117.1 | HI002907.1 |
| DD096737.1 | DM025549.1 | DL095894.1 | FW331773.1 | DL092803.1 | HD058870.1 | DL024481.1 | CQ875530.1 | HI001270.1 |
| DD081756.1 | DM025288.1 | DL095862.1 | FW308787.1 | DL092771.1 | HD057686.1 | DL024449.1 | CQ874248.1 | HI001217.1 |
| DD149168.1 | DM019672.1 | DL095830.1 | FW308318.1 | DL089156.1 | HD057654.1 | DL024417.1 | CQ872968.1 | HI001170.1 |
| DD148188.1 | DM024283.1 | DL095798.1 | FW334251.1 | DL089124.1 | HD062429.1 | DL024385.1 | CQ871410.1 | HI001098.1 |
| DD147789.1 | DM012887.1 | DL095766.1 | FW307218.1 | DL089092.1 | M24889.1 | DL021173.1 | CQ868898.1 | HI001080.1 |
| DD147757.1 | DM024249.1 | DL100313.1 | FW332549.1 | DL089060.1 | M20840.1 | DL021141.1 | CQ868490.1 | HI001022.1 |
| DD147725.1 | DM023679.1 | DL100281.1 | FW306418.1 | DL089028.1 | M13815.1 | DL021109.1 | CQ867417.1 | HI000990.1 |
| DD147693.1 | DM023583.1 | DL094143.1 | FW332169.1 | DL088996.1 | HC918549.1 | DL021077.1 | CQ859606.1 | HI002884.1 |
| DD147140.1 | DM028087.1 | DL094111.1 | HC769896.1 | DL122355.1 | FW334597.1 | DL021045.1 | CQ858050.1 | HI002846.1 |
| DD140146.1 | DM022398.1 | DL094079.1 | HC768864.1 | DL118005.1 | FW341507.1 | DL021013.1 | CQ857389.1 | HI002813.1 |
| DD080838.1 | DM022322.1 | DL094047.1 | HC742878.1 | DL117909.1 | HC889606.1 | DL012189.1 | CQ854811.1 | HI002754.1 |
| DD138757.1 | DM038582.1 | DL093983.1 | HC732142.1 | DL117877.1 | HC887869.1 | DL012157.1 | CQ831271.1 | FW392845.1 |
| DD107344.1 | DM022281.1 | DL093951.1 | FW298766.1 | DL117845.1 | HC883726.1 | DL012125.1 | CQ830536.1 | FW381967.1 |
| DD106660.1 | DM022215.1 | DL090336.1 | HC468965.1 | DL117813.1 | HC880535.1 | DL012093.1 | CQ828071.1 | HH931891.1 |
| DD092954.1 | DM022159.1 | DL090304.1 | HC486505.1 | DL113220.1 | HC291138.1 | DL012061.1 | CQ827718.1 | HH821483.1 |
| DD118645.1 | DM021901.1 | DL090240.1 | HC486440.1 | AY659156.1 | HC290958.1 | DL012029.1 | AX144793.1 | HH821865.1 |
| DD118104.1 | DM032830.1 | DL090208.1 | HC484165.1 | AY659124.1 | HC293541.1 | DL011997.1 | AX144729.1 | HH827400.1 |
| DD090410.1 | DM016010.1 | DL090176.1 | HC475380.1 | AY659092.1 | HC290581.1 | DL011939.1 | AX144697.1 | HH826999.1 |
| DD116276.1 | DM015954.1 | DL086171.1 | HC466635.1 | AY659060.1 | HC289875.1 | DL011907.1 | AX144665.1 | HH826497.1 |
| DD090306.1 | DM027051.1 | DL086139.1 | HC491790.1 | AY659028.1 | HC289841.1 | DL011875.1 | AX144633.1 | HH826425.1 |
| DL014187.1 | DM026398.1 | DL086107.1 | HC491758.1 | AY658996.1 | HC207646.1 | DL011843.1 | AX144589.1 | HH822352.1 |
| DL014155.1 | GN045776.1 | DL086075.1 | HC491726.1 | AY658964.1 | HC203586.1 | DL011811.1 | AX144525.1 | HH822038.1 |
| DL014123.1 | GN043702.1 | DL086043.1 | HC491017.1 | AY658932.1 | HC201650.1 | DL024286.1 | AX144461.1 | HH821978.1 |
| DL014091.1 | GN041957.1 | DL086011.1 | FV533144.1 | AY658900.1 | HC200109.1 | DL024254.1 | AX144333.1 | FW379712.1 |
| DL014004.1 | GN041701.1 | DL114413.1 | FV531713.1 | AY658868.1 | HC199275.1 | DL024222.1 | AX144139.1 | FW379680.1 |
| DL013972.1 | GN039844.1 | DL114381.1 | FV531681.1 | AY658836.1 | DM464842.1 | DL024190.1 | AX144075.1 | FW379647.1 |
| DL013940.1 | GN038623.1 | DL114349.1 | FV522811.1 | AY658804.1 | DM463598.1 | DL040874.1 | AX143945.1 | FW379615.1 |
| DL030040.1 | GN037625.1 | DL114317.1 | FV530193.1 | AY658772.1 | HC195229.1 | DL040842.1 | AX143881.1 | FW379573.1 |
| DL030008.1 | GN034795.1 | DL114285.1 | FV528456.1 | AY658740.1 | HC194031.1 | DL040810.1 | AX143817.1 | FW378150.1 |
| DL029976.1 | GN030754.1 | DL114253.1 | FV534416.1 | AY658708.1 | HC190673.1 | DL040778.1 | AX143497.1 | FW377969.1 |
| DL046633.1 | GN030722.1 | DL109452.1 | HC456317.1 | AY658676.1 | HC187044.1 | DL032496.1 | AX143369.1 | FW377382.1 |
| DL046601.1 | GN030690.1 | DL109420.1 | HC456046.1 | AY658644.1 | HC089581.1 | DL014007.1 | AX143305.1 | FW377096.1 |
| DL046569.1 | GN030658.1 | DL109388.1 | HC453678.1 | AY658612.1 | HC089549.1 | DL013975.1 | AX143241.1 | FW376185.1 |
| DL046537.1 | GN030626.1 | DL109356.1 | HC046482.1 | AY658580.1 | HC089517.1 | DL013943.1 | AX143177.1 | FW381119.1 |
| DL042651.1 | GM625461.1 | DL109324.1 | HC046450.1 | AY658548.1 | HC089485.1 | DL013911.1 | AX143113.1 | FW380263.1 |
| DL042619.1 | GM625397.1 | DL109292.1 | HC046418.1 | AY658516.1 | HC089453.1 | DL013879.1 | AX143049.1 | FW369305.1 |
| DL042587.1 | GM625365.1 | DL104686.1 | HC046386.1 | AY658484.1 | HC089421.1 | DL030043.1 | AX142857.1 | HH736041.1 |
| DL042671.1 | GM625333.1 | DL104590.1 | HC046354.1 | AY658452.1 | HC089388.1 | HV573501.1 | AX142793.1 | HH735919.1 |
| DL038663.1 | GM659321.1 | DL104558.1 | HC046071.1 | AY658420.1 | HC089356.1 | HV572102.1 | AX142601.1 | HH735624.1 |
| DL038631.1 | GM659288.1 | DL075848.1 | HC046039.1 | AY658388.1 | HC089323.1 | HV560154.1 | AX142537.1 | HH734125.1 |
| CS613792.1 | GM659256.1 | DL075816.1 | HC046247.1 | AY658356.1 | HC089291.1 | HV562134.1 | AX142343.1 | HD119616.1 |
| CS612923.1 | GM659224.1 | DL079954.1 | HC046215.1 | AY658324.1 | HC089259.1 | FW590869.1 | AX142021.1 | FW359983.1 |
| CS611842.1 | GM659192.1 | DL074552.1 | HC046151.1 | AY658292.1 | HC085651.1 | FW590732.1 | AX141891.1 | FW359899.1 |
| CS604544.1 | GM659160.1 | DL079057.1 | HC046001.1 | AY658260.1 | AY774946.1 | FW590099.1 | AX141827.1 | FW359839.1 |
| CS604512.1 | GM659109.1 | DL079025.1 | HC045969.1 | AY658228.1 | AY774848.1 | FW588674.1 | AX141443.1 | FW359753.1 |

| | | | | | | | | |
|---|---|---|---|---|---|---|---|---|
| CS604480.1 | GM659077.1 | DL078941.1 | HC045937.1 | AY658196.1 | AY774784.1 | FW593450.1 | AX141379.1 | FW359669.1 |
| CS604448.1 | GM659045.1 | DL078909.1 | HC045905.1 | AY658164.1 | AY774732.1 | FW586442.1 | AX141315.1 | FW368816.1 |
| CS604416.1 | GM659013.1 | DL072411.1 | HC045841.1 | AY658132.1 | AY774675.1 | FW592635.1 | AX139459.1 | FW368718.1 |
| CS604384.1 | GM658981.1 | DL072241.1 | HC045809.1 | AY658100.1 | AY774621.1 | FW577827.1 | AX134430.1 | FU262644.1 |
| CS604352.1 | GM658949.1 | DL081498.1 | HC045745.1 | AY658068.1 | AY774552.1 | FW576793.1 | AX108247.1 | FU250462.1 |
| CS604320.1 | GM651757.1 | DL013615.1 | HC045713.1 | AY658036.1 | AY774449.1 | FW576758.1 | AX107120.1 | FU261566.1 |
| CS604032.1 | GM651725.1 | DL013583.1 | HC045683.1 | AY658004.1 | AY774336.1 | FW576724.1 | AX101292.1 | FU269624.1 |
| CS603808.1 | GM651693.1 | DL013551.1 | DM378908.1 | AY657972.1 | AY774286.1 | FW576340.1 | AX100344.1 | HC313138.1 |
| CS603776.1 | GM651661.1 | DL013519.1 | HC035667.1 | AY657940.1 | AY774228.1 | FW575885.1 | AX097511.1 | DM476543.1 |
| CS603744.1 | GM651629.1 | DL013487.1 | DL028565.1 | AY657908.1 | AY774116.1 | FW575361.1 | AX097401.1 | DM475253.1 |
| CS603648.1 | GM651596.1 | DL013455.1 | DL012363.1 | AY657876.1 | AY645670.1 | FW571632.1 | AX093091.1 | HC299787.1 |
| CS603456.1 | GM644797.1 | DL013423.1 | DL048684.1 | AY657844.1 | HC070451.1 | FW572721.1 | AX089769.1 | HC306134.1 |
| CS603424.1 | GM644765.1 | DL013391.1 | DL045138.1 | AY657812.1 | HC070441.1 | FW568968.1 | AX088784.1 | HC306094.1 |
| CS603392.1 | GM644733.1 | DL018178.1 | DL037075.1 | AY657780.1 | HC070101.1 | FW573470.1 | AX088684.1 | HC306054.1 |
| CS603360.1 | GM644701.1 | DL018146.1 | DL037043.1 | AY657748.1 | HC083550.1 | FW572210.1 | HW126607.1 | GM626141.1 |
| CS603328.1 | GM644669.1 | DL018114.1 | DL015989.1 | AY657716.1 | HC083518.1 | FW574380.1 | HW144446.1 | DL116594.1 |
| CS603296.1 | GM644637.1 | DL018082.1 | DL015925.1 | AY657684.1 | HC069865.1 | FW568743.1 | HW144368.1 | DL116562.1 |
| CS603264.1 | GM637639.1 | DL018018.1 | DL015893.1 | AY657652.1 | AF080094.1 | FW572007.1 | HW144304.1 | DL111841.1 |
| CS603232.1 | GM637575.1 | DL013363.1 | DL015861.1 | AY657620.1 | GN032491.1 | FW571880.1 | HW124788.1 | DL111809.1 |
| CS603200.1 | GM637543.1 | DL013331.1 | DL011243.1 | AY657588.1 | GN032459.1 | HI967923.1 | HW124420.1 | DL111777.1 |
| CS603168.1 | GM637511.1 | DL013299.1 | DL011211.1 | AY657556.1 | GN032427.1 | HI967432.1 | HW123975.1 | DL128858.1 |
| CS603136.1 | GM637479.1 | DL013267.1 | DL047635.1 | AY657524.1 | GN032395.1 | HI979294.1 | HW122426.1 | DL128825.1 |
| CS603104.1 | GM633071.1 | DL013235.1 | DL047603.1 | AY657492.1 | GN032363.1 | HI001020.1 | HW118004.1 | DL128793.1 |
| CS603072.1 | GM633039.1 | DL013203.1 | DL047539.1 | AY657460.1 | GN032330.1 | HI000978.1 | HW106639.1 | DL128760.1 |
| CS603040.1 | GM633007.1 | DL017992.1 | DL039676.1 | AY657428.1 | GN032266.1 | HI002882.1 | HW115644.1 | DL107093.1 |
| CS603008.1 | GM632975.1 | DL017960.1 | DL039644.1 | AY657396.1 | GN032234.1 | HI002844.1 | HW113809.1 | DL116392.1 |
| CS602974.1 | GM632943.1 | DL017928.1 | DL039612.1 | AY657364.1 | GN032202.1 | HI002808.1 | HW113649.1 | DL116360.1 |
| CS602942.1 | GM632879.1 | DL017896.1 | DL035892.1 | AY657332.1 | GN032170.1 | HI002751.1 | HW101683.1 | DL111639.1 |
| CS602910.1 | GM625268.1 | DL017864.1 | DL039383.1 | AY657300.1 | GN032138.1 | HI002712.1 | HW087310.1 | DL111575.1 |
| CS602878.1 | GM625236.1 | DL017801.1 | DL039351.1 | AY657268.1 | GN032106.1 | HI002663.1 | HW097004.1 | DL111543.1 |
| CS602846.1 | GM625204.1 | DL017769.1 | DL039319.1 | AY657236.1 | GN032074.1 | HI002627.1 | HW096747.1 | DL111511.1 |
| CS602782.1 | GM625172.1 | DL017737.1 | DL035663.1 | AY657204.1 | BD243436.1 | HI002594.1 | HW072902.1 | DL111479.1 |
| CS602174.1 | GM625140.1 | DL017705.1 | DL035631.1 | AY657172.1 | BD242454.1 | HI002532.1 | HW096373.1 | DL101697.1 |
| CS610255.1 | GM624114.1 | DL017673.1 | DL023558.1 | AY657140.1 | BD241787.1 | HI001484.1 | HW072713.1 | DL101665.1 |
| CS323675.1 | GM624082.1 | DL017641.1 | DL023526.1 | AY657108.1 | BD238580.1 | HI004549.1 | HW072676.1 | DL101601.1 |
| CS323593.1 | GM624050.1 | DL022375.1 | DL023494.1 | AY657076.1 | BD238474.1 | HI004485.1 | HV778076.1 | DL097123.1 |
| CS330144.1 | GM624018.1 | DL022343.1 | DL023462.1 | AY657044.1 | BD235958.1 | HI004421.1 | HV777568.1 | DL097091.1 |
| CS329666.1 | GM623986.1 | DL022311.1 | DL023430.1 | M30828.1 | BD235639.1 | HI002399.1 | HV776436.1 | DL097027.1 |
| CS322141.1 | GM623954.1 | DL022279.1 | DL023398.1 | M13162.1 | BD234852.1 | HI002319.1 | HV775701.1 | DL095135.1 |
| DD271413.1 | GM632867.1 | DL022247.1 | DL020383.1 | K02755.1 | BD234412.1 | HI000525.1 | HV764763.1 | DL095103.1 |
| DD261130.1 | GM632835.1 | DL022215.1 | DL020351.1 | M28667.1 | BD232687.1 | HI183836.1 | HV764731.1 | DL095039.1 |
| DD259345.1 | GM632803.1 | DL022183.1 | DL020319.1 | J02535.1 | BD232286.1 | HI180166.1 | HV764699.1 | DL094975.1 |
| DD266538.1 | GM632771.1 | DL025730.1 | DL015820.1 | M34499.1 | BD231842.1 | HI202838.1 | HV764550.1 | DL106661.1 |
| CS302576.1 | GM632739.1 | DL025698.1 | DL015788.1 | HV549872.1 | BD231162.1 | HI202806.1 | HV763996.1 | DL106565.1 |
| CS297375.1 | GM632707.1 | DL025666.1 | DL015756.1 | HV549734.1 | BD231127.1 | HI214566.1 | HV760603.1 | DL106533.1 |
| DD251989.1 | GM625128.1 | DL025602.1 | DL015724.1 | HV549694.1 | BD231050.1 | HI214534.1 | HV766448.1 | DL106501.1 |
| DD251593.1 | GM625096.1 | DL025570.1 | CS601864.1 | HV549662.1 | BD227156.1 | HI214436.1 | HV766066.1 | DL125152.1 |

| | | | | | | | | |
|---|---|---|---|---|---|---|---|---|
| DD248718.1 | GM625064.1 | DL029712.1 | CS608441.1 | HV549630.1 | BD225780.1 | HI213764.1 | HV766034.1 | DL128698.1 |
| DD253699.1 | GM625032.1 | DL029680.1 | CS608356.1 | HV549598.1 | BD225748.1 | HI213007.1 | HV752946.1 | DL128664.1 |
| CS287623.1 | GM625000.1 | DL029648.1 | CS607913.1 | HV549500.1 | BD225716.1 | HI212962.1 | HV750190.1 | DL128545.1 |
| CS287531.1 | GM624968.1 | DL029616.1 | CS607666.1 | HV549301.1 | BD225684.1 | HI546321.1 | HV221129.1 | DL125068.1 |
| DD240752.1 | GM624936.1 | DL029584.1 | CS600905.1 | HV549191.1 | BD225652.1 | HI546226.1 | HV040122.1 | DL125036.1 |
| DD240703.1 | GM623910.1 | DL029552.1 | CS607174.1 | HV549089.1 | BD225620.1 | HI586985.1 | HV200502.1 | DL125004.1 |
| DD240655.1 | GM623878.1 | DL025511.1 | CS607073.1 | HV544359.1 | BD224826.1 | HI210999.1 | M17936.1 | DL124972.1 |
| DD240592.1 | GM623846.1 | DL025479.1 | CS606993.1 | HV544156.1 | BD224008.1 | HI210967.1 | HV549751.1 | DL124925.1 |
| DD240487.1 | GM623814.1 | DL025447.1 | CS606970.1 | HV543733.1 | BD222156.1 | HI210935.1 | HV549111.1 | DL124893.1 |
| DD240455.1 | GM623782.1 | DL025415.1 | CS606907.1 | HV543504.1 | CS157922.1 | HI566071.1 | HV549069.1 | DL120772.1 |
| DD238408.1 | GM623750.1 | DL025383.1 | CS606824.1 | FW508359.1 | CS157890.1 | HI210931.1 | HV548898.1 | DL120740.1 |
| DD234700.1 | GM658929.1 | DL034069.1 | CS605015.1 | FW508317.1 | CS124671.1 | HI473028.1 | HV544233.1 | DL116209.1 |
| DD247147.1 | GM658897.1 | DL034037.1 | CS604951.1 | FW508264.1 | CS124549.1 | HI472908.1 | HV547919.1 | DL116177.1 |
| E44250.1 | GM658865.1 | DL034005.1 | CS604887.1 | FW508220.1 | CS120364.1 | HI004375.1 | HV547832.1 | DL116145.1 |
| E41521.1 | GM658833.1 | DL033973.1 | CS593080.1 | FW559265.1 | CS119540.1 | HI004247.1 | HV543845.1 | DL116113.1 |
| E43962.1 | GM658801.1 | DL033941.1 | CS592772.1 | FW552308.1 | CS119506.1 | HI002277.1 | HV543482.1 | DL116081.1 |
| E35599.1 | GM658769.1 | DL021982.1 | CS597723.1 | FW552274.1 | CS119473.1 | HI002240.1 | HV538749.1 | DL116049.1 |
| E33537.1 | GM651545.1 | DL025330.1 | CS584923.1 | FW552237.1 | CS119406.1 | HI002190.1 | HV538569.1 | DL120573.1 |
| E06060.1 | GM651513.1 | DL025298.1 | CS576390.1 | FW552178.1 | CS119373.1 | HC920700.1 | HV543334.1 | DL120541.1 |
| E05792.1 | GM651481.1 | DL025266.1 | CS575308.1 | FW552102.1 | CS119341.1 | HD068942.1 | HV542739.1 | DL093145.1 |
| E05299.1 | GM651449.1 | DL025234.1 | CS544063.1 | FW558940.1 | CS119307.1 | HD068764.1 | HV542707.1 | DL093113.1 |
| E04742.1 | GM651417.1 | DL025202.1 | CS565540.1 | FW555605.1 | CS119275.1 | HD067802.1 | HV541663.1 | DL093081.1 |
| E04466.1 | GM651385.1 | DL025170.1 | CS570723.1 | FW560384.1 | CS119243.1 | HD064432.1 | HV514645.1 | DL089274.1 |
| E04183.1 | GM644586.1 | DL021958.1 | CS569604.1 | GM639867.1 | CS119211.1 | HD063554.1 | HV532566.1 | DL089242.1 |
| E03976.1 | GM644554.1 | DL021926.1 | CS561174.1 | GM639835.1 | CS119179.1 | HD052881.1 | HV537396.1 | DL125933.1 |
| E03208.1 | GM644522.1 | DL021894.1 | CS559089.1 | GM627293.1 | CS119115.1 | HD052106.1 | HV515728.1 | DL125901.1 |
| CQ814061.1 | GM644490.1 | DL021862.1 | DD421785.1 | GM627261.1 | CS119047.1 | HC920319.1 | HV515574.1 | DL125869.1 |
| CQ814028.1 | GM644458.1 | DL031829.1 | CS539994.1 | GM627229.1 | CS119015.1 | HD057690.1 | HV515510.1 | DL125837.1 |
| HB486001.1 | GM644426.1 | DL031797.1 | CS106073.1 | GM627197.1 | CS118982.1 | HD057658.1 | HV515478.1 | DL125805.1 |
| HB476373.1 | GM637428.1 | DL031765.1 | CS179720.1 | GM627165.1 | CS118950.1 | HD062477.1 | HV515414.1 | DL125773.1 |
| DM023383.1 | GM637396.1 | DL031733.1 | CS179475.1 | GM627133.1 | CS118917.1 | HD061335.1 | HV515382.1 | DL122563.1 |
| DM017757.1 | GM637364.1 | DL027924.1 | CS326343.1 | GM661117.1 | CS118884.1 | M28853.1 | HV515350.1 | DL122531.1 |
| DM010744.1 | GM637332.1 | DL027892.1 | CS323671.1 | GM661085.1 | CS118851.1 | M13566.1 | HV515318.1 | DL118117.1 |
| DM016248.1 | GM637300.1 | DL027860.1 | CS323589.1 | GM661053.1 | CS118785.1 | M65080.1 | HV515286.1 | DL118085.1 |
| DM015974.1 | GM637268.1 | DL027828.1 | CS330140.1 | GM661021.1 | CS118753.1 | FW334361.1 | HV515254.1 | DL118053.1 |
| GN045578.1 | GM621990.1 | DL027796.1 | CS329662.1 | GM660989.1 | CS118688.1 | FW334601.1 | HV511497.1 | DL118021.1 |
| GN042694.1 | GM621958.1 | DL027764.1 | DD271407.1 | GM660957.1 | CS118656.1 | FW342471.1 | HI378122.1 | DL113332.1 |
| GN041761.1 | FB506762.1 | DL023952.1 | DD272636.1 | GM660925.1 | CS118624.1 | FW337536.1 | HI376907.1 | DL113300.1 |
| GN041640.1 | GM635825.1 | DL023888.1 | CS299496.1 | GM653931.1 | CS118556.1 | FW337422.1 | HI376609.1 | DL108467.1 |
| GN040018.1 | GM635793.1 | DL023856.1 | CS297366.1 | GM653899.1 | CS118491.1 | FW341205.1 | HI375914.1 | DL108359.1 |
| GN037291.1 | GM635761.1 | DL023824.1 | DD251656.1 | GM653867.1 | CS118458.1 | FW336068.1 | HI375880.1 | DL108339.1 |
| GN030710.1 | GM635729.1 | DL023792.1 | DD251585.1 | GM653779.1 | CS118393.1 | FW335699.1 | HI369618.1 | DL108307.1 |
| GN030646.1 | GM635697.1 | DL020777.1 | DD253790.1 | GM653827.1 | CS118326.1 | HC889614.1 | HI369155.1 | DL103637.1 |
| GN030614.1 | GM635665.1 | DL020745.1 | CS287619.1 | GM646762.1 | CS118292.1 | HC868207.1 | HI369100.1 | DL099031.1 |
| GN030550.1 | GM630861.1 | DL020713.1 | CS287402.1 | GM646730.1 | CS118260.1 | HC867639.1 | HI424432.1 | DL098999.1 |
| GN030518.1 | GM630829.1 | DL020681.1 | DD240745.1 | GM646698.1 | CS118228.1 | DJ052208.1 | HI424128.1 | DL098935.1 |
| GN030486.1 | GM630797.1 | DL020649.1 | DD240584.1 | GM646666.1 | CS118195.1 | DJ051991.1 | HI424096.1 | DL092829.1 |

| | | | | | | | | |
|---|---|---|---|---|---|---|---|---|
| GN030410.1 | GM630765.1 | DL020617.1 | DD238903.1 | GM646634.1 | CS118129.1 | DJ050214.1 | HI424064.1 | DL092797.1 |
| GN030390.1 | GM630733.1 | DL016214.1 | DD237941.1 | GM639831.1 | CS118030.1 | CS793909.1 | HI423838.1 | DL092765.1 |
| GN030326.1 | GM630701.1 | DL016182.1 | DD235036.1 | GM639799.1 | CS117994.1 | CS721586.1 | HI422920.1 | DL089022.1 |
| GN030294.1 | GM621926.1 | E41531.1 | DD234582.1 | GM639767.1 | CS117962.1 | CS800090.1 | HI415744.1 | DL088990.1 |
| GN030230.1 | GM621894.1 | E49892.1 | E41552.1 | GM639735.1 | CS114801.1 | CS720118.1 | HI414151.1 | DL122317.1 |
| GN030198.1 | GM621830.1 | E49314.1 | E41131.1 | GM639703.1 | CS114654.1 | CS716784.1 | HI414031.1 | DL117967.1 |
| GN030166.1 | GM621798.1 | E38007.1 | E43947.1 | DL107118.1 | CS106398.1 | CS716778.1 | HI413994.1 | DL117935.1 |
| GN029942.1 | GM621766.1 | E40591.1 | E35595.1 | DL102505.1 | CS105930.1 | CS715286.1 | HI413679.1 | DL117903.1 |
| GN029812.1 | GM657327.1 | E33208.1 | E36137.1 | DL102473.1 | CS102974.1 | CS714323.1 | HI413588.1 | DL117871.1 |
| GN013596.1 | GM657295.1 | E33086.1 | CQ943997.1 | DL102441.1 | CS102942.1 | CS810201.1 | HI412696.1 | DL117839.1 |
| GN013373.1 | GM657263.1 | E06934.1 | CQ943965.1 | DL102409.1 | AX399666.1 | CS792479.1 | HI516572.1 | DL113118.1 |
| GN010216.1 | GM657231.1 | E06606.1 | CQ943933.1 | DL102377.1 | AX395273.1 | CS466059.1 | HI516106.1 | DL113086.1 |
| GN033546.1 | GM657199.1 | E05858.1 | CQ943901.1 | DL102345.1 | AX384806.1 | CS464787.1 | HI516074.1 | DL113054.1 |
| GN033514.1 | GM657167.1 | E05461.1 | CQ943869.1 | DL097732.1 | AX384576.1 | DD392037.1 | HI284351.1 | DL108157.1 |
| GN033450.1 | GM649975.1 | E04533.1 | A02367.1 | DL097700.1 | AX382226.1 | DD391921.1 | HI284293.1 | DL108125.1 |
| GN033418.1 | GM649943.1 | E04047.1 | HW261403.1 | DL097668.1 | AX376953.1 | DD382834.1 | HI574482.1 | DL108093.1 |
| GN033386.1 | GM649911.1 | E03793.1 | HW261371.1 | DL097636.1 | AX364528.1 | DD400158.1 | HI574450.1 | DL103487.1 |
| GN033354.1 | GM649879.1 | E03311.1 | HW261339.1 | DL097604.1 | AX364496.1 | DD400094.1 | HI574418.1 | DL017064.1 |
| GN033322.1 | GM649847.1 | E02926.1 | HW261307.1 | DL097572.1 | AX364464.1 | CS463697.1 | HI574386.1 | DL017032.1 |
| GN033290.1 | GM649815.1 | E02683.1 | HW261275.1 | DL097540.1 | AX364432.1 | CS459099.1 | HI574363.1 | DL049431.1 |
| GN033226.1 | GM642975.1 | E02388.1 | HW261243.1 | DL095513.1 | AX364400.1 | DD361289.1 | HI578440.1 | AX608810.1 |
| GN033194.1 | GM642942.1 | E01381.1 | HW261211.1 | DL095449.1 | AX364368.1 | DD367617.1 | HI566196.1 | AX599028.1 |
| GN033130.1 | GM642910.1 | E00636.1 | HW261179.1 | DL095417.1 | AX364326.1 | DD367329.1 | HC767389.1 | AX598874.1 |
| GN033066.1 | GM642878.1 | E00033.1 | HW261115.1 | DL095353.1 | AX364209.1 | DD373564.1 | HC757141.1 | AX598772.1 |
| GN033034.1 | GM642846.1 | DD146391.1 | HW261083.1 | DL091539.1 | AX364176.1 | DD368156.1 | HC755769.1 | AX593346.1 |
| GN033002.1 | GM642814.1 | DD115369.1 | HW261051.1 | DL091507.1 | AX363230.1 | DD361323.1 | HC754648.1 | AY183361.1 |
| GN032970.1 | GM635632.1 | DD087911.1 | HW261019.1 | DL091475.1 | AX359001.1 | CS458451.1 | HC732136.1 | AX592805.1 |
| GN032938.1 | GM635600.1 | DD146259.1 | HW260987.1 | DL091443.1 | AX358636.1 | CS457839.1 | HC471763.1 | AX591630.1 |
| GN032906.1 | GM635568.1 | DD146204.1 | HW260955.1 | DL091379.1 | AX358391.1 | CS457450.1 | HC471731.1 | BD162045.1 |
| GN032874.1 | GM635536.1 | DD144850.1 | HW260923.1 | DL087486.1 | E50931.1 | CS457130.1 | HC471667.1 | BD160756.1 |
| GN032810.1 | GM635504.1 | DD216455.1 | HW260891.1 | DL087454.1 | E54538.1 | CS456700.1 | HC471635.1 | BD143511.1 |
| GN032778.1 | GM635472.1 | DD216423.1 | HW260859.1 | DL087422.1 | BD000711.1 | CS453996.1 | HC471603.1 | AX587989.1 |
| GN032747.1 | GM630666.1 | DD213773.1 | HW260827.1 | DL087390.1 | E59831.1 | CS453656.1 | HC688423.1 | AX587956.1 |
| GN032715.1 | GM630634.1 | DD213741.1 | HW260795.1 | DL112232.1 | E58874.1 | CS452794.1 | HC490827.1 | AX587861.1 |
| GN032683.1 | GM630602.1 | DD213709.1 | HW260763.1 | DL112200.1 | AX356495.1 | CS451652.1 | HC490795.1 | AX587813.1 |
| GN032651.1 | GM630570.1 | DD213677.1 | HW260699.1 | DL112136.1 | AX353934.1 | DD360980.1 | HC490763.1 | AX587706.1 |
| GN032586.1 | GM630538.1 | DD213645.1 | HW260667.1 | DL112104.1 | AX353682.1 | DD357922.1 | HC490731.1 | HW387043.1 |
| GM627892.1 | GM630506.1 | DD213613.1 | HW260635.1 | DL112072.1 | HI424130.1 | DD357436.1 | HC490699.1 | HW386497.1 |
| GM627860.1 | GM621731.1 | DD220746.1 | HW260603.1 | DL107069.1 | HI424098.1 | CS449745.1 | HC490538.1 | HW386305.1 |
| GM627828.1 | GM621699.1 | DD213595.1 | HW260571.1 | DL107005.1 | HI424066.1 | CS447939.1 | HC490503.1 | HW386273.1 |
| GM627796.1 | GM621667.1 | DD213563.1 | HW260539.1 | DL106973.1 | HI423808.1 | A32912.1 | HC686446.1 | HW386241.1 |
| GM661714.1 | GM621635.1 | DD213531.1 | HW260507.1 | DL106941.1 | HI423776.1 | A08143.1 | HC490368.1 | HW386177.1 |
| GM661650.1 | DL016150.1 | DD216043.1 | HW260475.1 | DL106909.1 | HI422924.1 | A02841.1 | HC490239.1 | HW386017.1 |
| GM661618.1 | DL016118.1 | DD216011.1 | HW260443.1 | DL102296.1 | HI416194.1 | A03623.1 | HC472293.1 | HW385921.1 |
| GM661586.1 | DL016086.1 | DD215979.1 | HW260411.1 | DL102264.1 | HI415746.1 | CS443438.1 | HC472260.1 | HW380895.1 |
| GM661554.1 | DL016054.1 | DD220371.1 | HW260379.1 | DL102176.1 | HI414033.1 | CS442149.1 | HC472196.1 | HW380818.1 |
| GM661522.1 | DL016022.1 | DD215933.1 | HW260347.1 | DL102224.1 | HI413996.1 | CS438978.1 | HC472164.1 | HW376182.1 |

| | | | | | | | | |
|---|---|---|---|---|---|---|---|---|
| GM654528.1 | DL011532.1 | DD215901.1 | HW260283.1 | DL102168.1 | HI413683.1 | CS435285.1 | HC471964.1 | HW375237.1 |
| GM654496.1 | DL011500.1 | DD215869.1 | HW260251.1 | DL102136.1 | HI413592.1 | CS435221.1 | HC471932.1 | HW364671.1 |
| GM654464.1 | DL011468.1 | DD215837.1 | HW260219.1 | DL117004.1 | HI413437.1 | CS435187.1 | HC471900.1 | HW381045.1 |
| GM654368.1 | DL011404.1 | DD215805.1 | HW260187.1 | DL116972.1 | HI516506.1 | DD332631.1 | HC678823.1 | HW353846.1 |
| GM647279.1 | DL031714.1 | DD215773.1 | HW260155.1 | DL116940.1 | HI516108.1 | DD346816.1 | HC511397.1 | HW353498.1 |
| GM647247.1 | DL031682.1 | DD217994.1 | HW260091.1 | DL116908.1 | HI516076.1 | CS101550.1 | HB842329.1 | HW352461.1 |
| GM647215.1 | DL031650.1 | DD215743.1 | HW260059.1 | DL116876.1 | HI540889.1 | CS101207.1 | HB841961.1 | HW293162.1 |
| GM640284.1 | DL031586.1 | DD215711.1 | HW260027.1 | DL116844.1 | HI553984.1 | CS091374.1 | HB841709.1 | HW293031.1 |
| GM870240.1 | DL031554.1 | DD215679.1 | HW259995.1 | DL116816.1 | HI547560.1 | CS088871.1 | HB841247.1 | HW292754.1 |
| GM627613.1 | DL027745.1 | DD213052.1 | HW259963.1 | DL116784.1 | HI284295.1 | CS088083.1 | HB828561.1 | HW291170.1 |
| GM627581.1 | DL027713.1 | DD221798.1 | HW259931.1 | DL116752.1 | HI571653.1 | CS085798.1 | HB648625.1 | HW291128.1 |
| GM627549.1 | DL027681.1 | CS254896.1 | HW259854.1 | DL112031.1 | HI574516.1 | CS083077.1 | HB645704.1 | HW291073.1 |
| GM661490.1 | DL027649.1 | DD212547.1 | HW258941.1 | DL111999.1 | HI574452.1 | CS078828.1 | HC190458.1 | HW291041.1 |
| GM661458.1 | DL027617.1 | DD212000.1 | HW258829.1 | DL111935.1 | HI574420.1 | CS077634.1 | HC089574.1 | HW291009.1 |
| GM661426.1 | DL027585.1 | DD211315.1 | HW257350.1 | DL111903.1 | HI574388.1 | CS075440.1 | HC089542.1 | HW290977.1 |
| GM661394.1 | DL027553.1 | DD210927.1 | HW257318.1 | DL111891.1 | HI574365.1 | CS070527.1 | HC089510.1 | HW290945.1 |
| GM661362.1 | DL023741.1 | DD209233.1 | HW257254.1 | DL106868.1 | HI566200.1 | CS068659.1 | HC089478.1 | HW290913.1 |
| GM661330.1 | DL023709.1 | DD200279.1 | AY658791.1 | DL106836.1 | HI593987.1 | CS063837.1 | HC089446.1 | HW298792.1 |
| GM654336.1 | DL023677.1 | DD200043.1 | AY658759.1 | DL106804.1 | HI592945.1 | AY967384.1 | HC089413.1 | HW290882.1 |
| GM654304.1 | DL023645.1 | DD187299.1 | AY658727.1 | DL106772.1 | HC880421.1 | AY967352.1 | HC089381.1 | HW290850.1 |
| GM654272.1 | DL023613.1 | DD206898.1 | AY658695.1 | DL106740.1 | HC877780.1 | AY967320.1 | HC089349.1 | HW290341.1 |
| GM654240.1 | DL023581.1 | DD206866.1 | AY658663.1 | DL106708.1 | FW304459.1 | AY967288.1 | HC089316.1 | HW298197.1 |
| GM647183.1 | DL020534.1 | DD206834.1 | AY658631.1 | DL091342.1 | FW310241.1 | AY967256.1 | HC089284.1 | HW269102.1 |
| GM647151.1 | DL020502.1 | DD206804.1 | AY658599.1 | DL091310.1 | FW331768.1 | AY967224.1 | HC089252.1 | HW269070.1 |
| GM647119.1 | DL020470.1 | DD206772.1 | AY658567.1 | DL091278.1 | FW309548.1 | AY967192.1 | AY774989.1 | HW267800.1 |
| GM647087.1 | DL020438.1 | DD196862.1 | AY658535.1 | DL091246.1 | FW332153.1 | AY967160.1 | AY774898.1 | HW267616.1 |
| GM647079.1 | DL016003.1 | DD212671.1 | AY658503.1 | DL091214.1 | HC767797.1 | AY967128.1 | AY774836.1 | HW263068.1 |
| GM647044.1 | DL015971.1 | CS236401.1 | AY658471.1 | DL091182.1 | HC767394.1 | AY967096.1 | AY774775.1 | HW263031.1 |
| GM627513.1 | DL015939.1 | DD169477.1 | AY658439.1 | DL087374.1 | HC757393.1 | AY967064.1 | AY774720.1 | HW262999.1 |
| GM661293.1 | DL015907.1 | DD163500.1 | AY658407.1 | AX282195.1 | HC755803.1 | AY967032.1 | AY774611.1 | HW262967.1 |
| GM654133.1 | DL015875.1 | DD169215.1 | AY658375.1 | AX280356.1 | HC465804.1 | AY967000.1 | AY774495.1 | HW262919.1 |
| GM654101.1 | DL015843.1 | DD163310.1 | AY658343.1 | AX279962.1 | AY305397.1 | AY966968.1 | AY774441.1 | HW262882.1 |
| GM654069.1 | DL011385.1 | DD163197.1 | AY658311.1 | AX279767.1 | HC456313.1 | AY966936.1 | AY774323.1 | HW262578.1 |
| GM654037.1 | DL011353.1 | DD168805.1 | AY658279.1 | AX278764.1 | HC456037.1 | CS057794.1 | AY774276.1 | HW261410.1 |
| DL013938.1 | DL011321.1 | DD168390.1 | AY658247.1 | AX278097.1 | HC453706.1 | CS056284.1 | AY774097.1 | HW261378.1 |
| DL030038.1 | DL011289.1 | AX419097.1 | AY658215.1 | AX256280.1 | HC453642.1 | CS055383.1 | AY181197.1 | HW261346.1 |
| DL029974.1 | DL011257.1 | AX418396.1 | AY658183.1 | AX254825.1 | HC442316.1 | CS053031.1 | K01435.1 | HW261282.1 |
| DL046631.1 | DL011225.1 | AX418198.1 | AY658151.1 | AX253026.1 | HC452097.1 | CS052834.1 | AF274976.1 | HW261218.1 |
| DL046599.1 | DL047649.1 | AX411558.1 | AY658119.1 | AX251540.1 | FU774062.1 | CS052436.1 | HC083543.1 | HW261186.1 |
| DL046567.1 | DL047617.1 | AX405106.1 | AY658087.1 | AX249910.1 | FU761005.1 | CS052385.1 | HC083463.1 | HW261154.1 |
| DL046535.1 | DL047585.1 | AX403672.1 | AY658055.1 | AX242335.1 | FU971690.1 | CS050982.1 | DM461155.1 | HW261122.1 |
| DL042649.1 | DL047553.1 | AX402397.1 | AY658023.1 | AX242271.1 | FU773360.1 | CS047667.1 | U26407.1 | HW261090.1 |
| DL042617.1 | DL047521.1 | AX398973.1 | AY657991.1 | AX242207.1 | FU759995.1 | BD085727.1 | HC057020.1 | HW261026.1 |
| DL042585.1 | DL047489.1 | AX397845.1 | AY657959.1 | AX242175.1 | FU757920.1 | BD082915.1 | GN030735.1 | HW260930.1 |
| DL042669.1 | DL047457.1 | AX395622.1 | AY657927.1 | AX242143.1 | FU757630.1 | BD081900.1 | GN030639.1 | HW260898.1 |
| DL038661.1 | DL039658.1 | AX392857.1 | AY657895.1 | AX242111.1 | HC441562.1 | BD080680.1 | GN030607.1 | HW260866.1 |
| DL038629.1 | DL039626.1 | AX392091.1 | AY657863.1 | AX242079.1 | HC358404.1 | BD080432.1 | GN030575.1 | HW260834.1 |

| | | | | | | | | |
|---|---|---|---|---|---|---|---|---|
| DD405588.1 | DL039594.1 | AX391501.1 | AY657831.1 | AX242047.1 | HC358216.1 | BD080141.1 | GN030543.1 | HW260802.1 |
| DD405556.1 | DL039562.1 | AX384698.1 | AY657799.1 | AX241951.1 | HC358085.1 | BD077097.1 | GN030511.1 | HW260770.1 |
| DD405492.1 | DL039498.1 | AX384391.1 | AY657767.1 | AX241919.1 | HC435690.1 | BD075588.1 | GN030479.1 | HW260738.1 |
| DD405460.1 | DL035714.1 | AX382518.1 | AY657735.1 | AX241887.1 | A00965.1 | BD075357.1 | GN030393.1 | HW260706.1 |
| DD405428.1 | DL000549.1 | AX377466.1 | AY657703.1 | AX241855.1 | HC324494.1 | GM648264.1 | GN030351.1 | HW260674.1 |
| DD402022.1 | DJ493839.1 | AX367108.1 | AY657671.1 | AX241823.1 | HC313542.1 | GM648232.1 | GN030319.1 | HW260642.1 |
| DD401990.1 | DJ492715.1 | AX364626.1 | AY657639.1 | AX241759.1 | HC318843.1 | GM648200.1 | GN030255.1 | HW260610.1 |
| DD401929.1 | DL008512.1 | AX364541.1 | AY657607.1 | AX241727.1 | HC318706.1 | GM641397.1 | GN030191.1 | HW260578.1 |
| DD401897.1 | DL003042.1 | AX364509.1 | AY657575.1 | AX241695.1 | HC317598.1 | GM641365.1 | GN029935.1 | HW260546.1 |
| DD401865.1 | DL002943.1 | GM625171.1 | AY657543.1 | AX241663.1 | DM121639.1 | GM641333.1 | GN029837.1 | HW260514.1 |
| DD401833.1 | DJ491567.1 | GM625139.1 | AY657511.1 | AX241631.1 | HB442585.1 | GM641301.1 | GN029805.1 | HW260482.1 |
| DD401801.1 | DL002533.1 | GM624113.1 | AY657479.1 | AX241599.1 | HB441222.1 | GM641269.1 | GN013589.1 | HW260450.1 |
| DD401769.1 | DJ446834.1 | GM624081.1 | AY657447.1 | AX241567.1 | HB434956.1 | GM641237.1 | GN010242.1 | HW260418.1 |
| DD401625.1 | DJ474322.1 | GM624049.1 | AY657415.1 | AX241535.1 | HB432480.1 | GM629078.1 | GN033539.1 | HW260386.1 |
| DD401593.1 | DJ438111.1 | GM624017.1 | AY657383.1 | AX241503.1 | HB416446.1 | GM629046.1 | GN033507.1 | HW260354.1 |
| DD401561.1 | DJ442352.1 | GM623985.1 | AY657351.1 | AX241471.1 | HB413049.1 | GM629014.1 | GN033475.1 | HW260322.1 |
| DD401529.1 | DJ437329.1 | GM623953.1 | AY657319.1 | AX241100.1 | HB412594.1 | GM628950.1 | GN033443.1 | HV344566.1 |
| DD401497.1 | DJ437281.1 | GM632866.1 | AY657287.1 | AX241068.1 | HB394301.1 | GM628918.1 | GN033411.1 | HV339963.1 |
| DD401465.1 | DJ437183.1 | GM632834.1 | AY657255.1 | AX241036.1 | HB394269.1 | GM655192.1 | GN033347.1 | HV339931.1 |
| CS479222.1 | DJ437153.1 | GM632802.1 | AY657223.1 | AX241004.1 | HB394237.1 | GM655160.1 | GN033315.1 | HV339867.1 |
| CS476354.1 | DJ437084.1 | GM632770.1 | AY657191.1 | AX240972.1 | HB403381.1 | GM648167.1 | GN033283.1 | HV342418.1 |
| CS468419.1 | DJ437000.1 | GM632738.1 | HW285864.1 | AX240940.1 | HB397715.1 | GM641172.1 | GN033251.1 | HV344452.1 |
| CS467758.1 | DJ436924.1 | GM625127.1 | HW294137.1 | AX239709.1 | DM120693.1 | GM641140.1 | GN033219.1 | HV335549.1 |
| CS466053.1 | DJ436858.1 | GM625095.1 | HW294073.1 | AX236457.1 | DM118004.1 | DL206285.1 | GN033091.1 | HV341998.1 |
| DD382831.1 | DJ436771.1 | GM625063.1 | HW294039.1 | AX235777.1 | DM115566.1 | FB714900.1 | GN033059.1 | HV323127.1 |
| DD400155.1 | DJ436674.1 | GM625031.1 | HW293098.1 | AX235247.1 | DM114859.1 | FB713987.1 | GN032899.1 | HV322265.1 |
| CS463679.1 | DJ431072.1 | GM624967.1 | HW292746.1 | AX225272.1 | GM668914.1 | FB713763.1 | GN032867.1 | BD130488.1 |
| DD361286.1 | DJ444576.1 | GM624935.1 | HW291162.1 | AX210347.1 | HB387462.1 | FB713731.1 | GN032835.1 | BD107707.1 |
| DD367614.1 | DJ434404.1 | GM623909.1 | HW291065.1 | AX207298.1 | HB386546.1 | FB712764.1 | GN032803.1 | BD105787.1 |
| DD367318.1 | DJ443472.1 | GM623877.1 | HW291033.1 | HW390759.1 | HB384546.1 | FB708008.1 | GN032771.1 | BD105666.1 |
| DD362903.1 | DJ438369.1 | GM623845.1 | HW291001.1 | HW399702.1 | HB338868.1 | FB675126.1 | GN032740.1 | BD087391.1 |
| DD362143.1 | DJ438296.1 | GM623813.1 | HW290937.1 | HW399339.1 | HA642846.1 | FB672284.1 | GN032708.1 | BD085728.1 |
| DD368152.1 | DJ427992.1 | GM623781.1 | HW290906.1 | HW399257.1 | HA642257.1 | CS721893.1 | GN032676.1 | BD082916.1 |
| DD361320.1 | DJ430184.1 | GM623749.1 | HW290874.1 | HW408898.1 | HA641596.1 | FB676569.1 | GN032643.1 | BD081472.1 |
| CS457447.1 | DJ402655.1 | GM658896.1 | HW290842.1 | HW408866.1 | HA641532.1 | FB676537.1 | GN032579.1 | BD080433.1 |
| CS457193.1 | DJ402619.1 | GM658864.1 | HW290106.1 | HW382302.1 | HA641131.1 | FB705690.1 | GN032548.1 | BD080142.1 |
| BD495442.1 | DJ402587.1 | GM658832.1 | HW289995.1 | HW390536.1 | GM711161.1 | FB675470.1 | GN032516.1 | BD078579.1 |
| BD453896.1 | DJ402555.1 | GM658768.1 | HW289018.1 | HW390444.1 | GM828151.1 | FB701520.1 | GN032484.1 | BD077098.1 |
| BD453864.1 | DJ416281.1 | GM651576.1 | HW285400.1 | HW390374.1 | GM742771.1 | CS809039.1 | GN032452.1 | BD075600.1 |
| BD453832.1 | DJ416100.1 | GM651544.1 | HW267902.1 | HW388646.1 | GM710184.1 | FB701039.1 | GN032420.1 | BD074949.1 |
| BD453800.1 | DJ401116.1 | GM651512.1 | HW267771.1 | HW387870.1 | GM772918.1 | FB660048.1 | GN032355.1 | BD074800.1 |
| BD453744.1 | DJ400812.1 | GM651480.1 | HW267433.1 | HW387480.1 | GM709006.1 | FB660133.1 | GN032323.1 | BD073563.1 |
| BD453712.1 | DJ400780.1 | GM651448.1 | HW266175.1 | HW386566.1 | GM708974.1 | FB665225.1 | GN032291.1 | BD063672.1 |
| BD453680.1 | DJ393684.1 | GM651416.1 | HW266202.1 | HW386310.1 | AY171560.1 | FB654423.1 | GN032259.1 | BD061600.1 |
| BD453648.1 | DJ393230.1 | GM651384.1 | HW266146.1 | HW386278.1 | HH804838.1 | FB653048.1 | GN032227.1 | BD057143.1 |
| BD453616.1 | DJ415906.1 | GM644585.1 | HW263023.1 | HW386246.1 | HH802659.1 | FB583263.1 | GN032195.1 | BD016699.1 |
| BD453579.1 | DJ391697.1 | GM644553.1 | HW262991.1 | HW386214.1 | HH794942.1 | FB580218.1 | GN032163.1 | BD015797.1 |

| | | | | | | | | |
|---|---|---|---|---|---|---|---|---|
| BD493235.1 | DJ418073.1 | GM644521.1 | HW262959.1 | HW386054.1 | HH794710.1 | FB580185.1 | GN032131.1 | BD014231.1 |
| BD453566.1 | AX364477.1 | GM644489.1 | HW262911.1 | HW386022.1 | HH794185.1 | FB580121.1 | GN032099.1 | BD014198.1 |
| BD453534.1 | AX364445.1 | GM644457.1 | HW262854.1 | HW385990.1 | HH774426.1 | FB573727.1 | GN032067.1 | BD014158.1 |
| BD453502.1 | AX364413.1 | GM644425.1 | HW262568.1 | HW380914.1 | FW375403.1 | DL200051.1 | GN032035.1 | E63780.1 |
| BD453470.1 | AX364381.1 | GM637427.1 | HW261402.1 | HW380823.1 | FW375227.1 | DL200019.1 | GN032003.1 | AX482607.1 |
| BD453438.1 | AX364349.1 | GM637395.1 | HW261370.1 | HW368542.1 | FW369621.1 | DL199987.1 | GN031971.1 | AX481570.1 |
| BD453406.1 | AX364254.1 | GM637363.1 | HW261338.1 | HW366481.1 | FW369392.1 | DL124470.1 | GN031939.1 | AX473141.1 |
| BD453261.1 | AX364222.1 | GM637331.1 | HW261306.1 | HW375068.1 | HH736081.1 | DL124438.1 | GN031907.1 | E61321.1 |
| BD453229.1 | AX364189.1 | GM637299.1 | HW261274.1 | HW365540.1 | HH733740.1 | DL124406.1 | GN031875.1 | AX468884.1 |
| BD453197.1 | AX363243.1 | GM637267.1 | HW261242.1 | HW364429.1 | HD119648.1 | DL124374.1 | GN031843.1 | AX468319.1 |
| BD442632.1 | AX361067.1 | GM623730.1 | HW261210.1 | HW373305.1 | FW368173.1 | DL120285.1 | GN031811.1 | AX463660.1 |
| BD399406.1 | AX357317.1 | GM623698.1 | HW261178.1 | HW381355.1 | FW367819.1 | DL120253.1 | GN031778.1 | A23991.1 |
| BD398994.1 | AX356677.1 | GM623666.1 | HW261114.1 | HW381050.1 | FW362405.1 | DL120221.1 | GN031713.1 | A10402.1 |
| BD497500.1 | BD011667.1 | GM623602.1 | HW261082.1 | HW344319.1 | FW351380.1 | DL115818.1 | GN031681.1 | AX458651.1 |
| BD451779.1 | BD006818.1 | GM623570.1 | AY657286.1 | HW344287.1 | FW351227.1 | DL115786.1 | GN031649.1 | AX458535.1 |
| BD450794.1 | BD006203.1 | GM827580.1 | AY657254.1 | HW240810.1 | FW361584.1 | DL115754.1 | DL193904.1 | HW347996.1 |
| BD408104.1 | E54577.1 | GM658719.1 | AY657222.1 | HW240722.1 | HD115809.1 | DL115690.1 | DL193786.1 | HW347635.1 |
| BD419030.1 | E59176.1 | GM658687.1 | AY657190.1 | HW247941.1 | HD082409.1 | DL115658.1 | DL193754.1 | HW363679.1 |
| BD429569.1 | AX354712.1 | GM658655.1 | AY657158.1 | HW239409.1 | HC873819.1 | DL111232.1 | CQ986522.1 | HW363502.1 |
| BD397104.1 | AX353445.1 | GM658623.1 | AY657126.1 | HW239187.1 | HC880389.1 | DL111200.1 | CQ981110.1 | HW355180.1 |
| BD429005.1 | AX351216.1 | GM658591.1 | AY657094.1 | HW238895.1 | FW310239.1 | DL111168.1 | CQ973472.1 | HW344687.1 |
| BD406433.1 | AX351106.1 | GM658559.1 | AY657062.1 | HW238597.1 | FW308597.1 | DL111104.1 | CQ979564.1 | HW317053.1 |
| BD396021.1 | AX350134.1 | GM651367.1 | AY499198.1 | HW238413.1 | FW332376.1 | DL111072.1 | CQ972387.1 | HW326508.1 |
| BD395084.1 | AX349047.1 | GM651335.1 | K01681.1 | HW238375.1 | FW306080.1 | DL101399.1 | CQ972355.1 | HW326422.1 |
| BD426881.1 | AX348529.1 | GM651303.1 | M18257.1 | HW238191.1 | FW332139.1 | DL106097.1 | CQ971110.1 | HW326310.1 |
| BD445721.1 | AX347459.1 | GM651271.1 | M12663.1 | HW242968.1 | HC768813.1 | DL106065.1 | CQ970415.1 | HW338858.1 |
| BD453346.1 | AX347377.1 | GM651239.1 | M74306.1 | HW238009.1 | HC307902.1 | DL106033.1 | CQ969377.1 | HW338602.1 |
| BD453317.1 | AX347295.1 | GM644376.1 | M17937.1 | HW244285.1 | HC307835.1 | DL105969.1 | CQ947153.1 | HW338474.1 |
| BD453285.1 | AX347257.1 | GM644344.1 | HV549073.1 | HW237912.1 | HC307788.1 | DL105937.1 | CQ947114.1 | HW338346.1 |
| BD455506.1 | AX347225.1 | GM644280.1 | HV544266.1 | HW237868.1 | FU262777.1 | DL105905.1 | CQ944184.1 | HW338090.1 |
| BD391412.1 | AX347187.1 | GM644216.1 | HV547834.1 | HW237691.1 | FU259851.1 | DL101196.1 | CQ944152.1 | HW337706.1 |
| CQ898772.1 | AX345617.1 | GM637218.1 | HV543858.1 | HW237431.1 | FU265397.1 | DL101164.1 | CQ944120.1 | HW337450.1 |
| CQ898645.1 | AX344875.1 | GM637186.1 | HV543553.1 | HW242324.1 | FU262612.1 | DL101132.1 | CQ944088.1 | HW337322.1 |
| CQ898613.1 | AX344137.1 | GM637154.1 | HV543484.1 | HW242292.1 | FU265323.1 | DL096718.1 | CQ944056.1 | HW337194.1 |
| CQ898581.1 | AX343222.1 | GM637122.1 | HV538571.1 | HW242260.1 | FU264493.1 | DL096686.1 | CQ944024.1 | HW337066.1 |
| CQ898510.1 | AX339385.1 | GM637090.1 | HV543336.1 | GM660634.1 | HC313238.1 | DL096654.1 | CQ943992.1 | HW336938.1 |
| CQ895561.1 | AX339191.1 | GM637058.1 | HV543209.1 | GM660602.1 | HC299772.1 | DL096622.1 | CQ943960.1 | HW321644.1 |
| CQ895455.1 | AX329440.1 | GM624905.1 | HV542741.1 | GM660570.1 | HC306119.1 | DL096590.1 | CQ943928.1 | HW336654.1 |
| CQ893702.1 | AX329153.1 | GM624873.1 | HV542605.1 | GM660538.1 | HC306079.1 | DL096558.1 | CQ943896.1 | HW329647.1 |
| CQ892398.1 | AX328291.1 | GM632656.1 | HV541665.1 | GM653351.1 | HC306039.1 | DL094730.1 | CQ943864.1 | HW329332.1 |
| DL105137.1 | AX328132.1 | GM632574.1 | HV540055.1 | GM653319.1 | HC305999.1 | DL094698.1 | CQ898670.1 | HW335978.1 |
| DL105105.1 | AX327575.1 | GM632616.1 | HV515098.1 | GM653287.1 | HC302642.1 | DL094666.1 | CQ898638.1 | HW328682.1 |
| DL119985.1 | AX323807.1 | GM632560.1 | HV536485.1 | GM653255.1 | HC302382.1 | DL090955.1 | CQ898606.1 | HW328354.1 |
| DL119953.1 | AX322858.1 | GM632528.1 | HV537554.1 | GM653223.1 | HC305768.1 | DL086987.1 | CQ898574.1 | HW062405.1 |
| DL119889.1 | AX319652.1 | GM632496.1 | HV537399.1 | GM653191.1 | HC301622.1 | DL086955.1 | CQ897148.1 | HW064447.1 |
| DL119857.1 | AX141795.1 | GM741946.1 | HV515730.1 | GM646192.1 | HC305964.1 | DL086923.1 | CQ895921.1 | HW062380.1 |
| DL119825.1 | AX141539.1 | GM674387.1 | HV515576.1 | GM646160.1 | HC294254.1 | DL086891.1 | CQ895548.1 | HW062316.1 |

| | | | | | | | | |
|---|---|---|---|---|---|---|---|---|
| DL115134.1 | AX141411.1 | GM655770.1 | HV515512.1 | GM646128.1 | HC291402.1 | DL108336.1 | CQ891334.1 | HW056011.1 |
| DL110070.1 | AX141283.1 | GM648753.1 | HV515480.1 | GM646096.1 | HC289383.1 | DL108304.1 | CQ890951.1 | HW042071.1 |
| DL110038.1 | AX138001.1 | GM648721.1 | HV515416.1 | GM646032.1 | HC289339.1 | DL103634.1 | CQ887983.1 | HW042039.1 |
| DL110006.1 | AX137606.1 | GM648689.1 | HV515352.1 | GM639229.1 | DM370525.1 | DL099028.1 | A25449.1 | HW043569.1 |
| DL109974.1 | AX135831.1 | GM648657.1 | HV515320.1 | GM639197.1 | DM370493.1 | DL098996.1 | AX427667.1 | HW041963.1 |
| DL109728.1 | AX135076.1 | GM648625.1 | HV515288.1 | GM639165.1 | DM370461.1 | DL098964.1 | AX427203.1 | HW041931.1 |
| DL109696.1 | AX127714.1 | GM648593.1 | HV515256.1 | GM639133.1 | DM367518.1 | DL092826.1 | AX419823.1 | HW041833.1 |
| DL104962.1 | AX127188.1 | GM641790.1 | HV515224.1 | GM639101.1 | HC003082.1 | DL092794.1 | AX418541.1 | HW049385.1 |
| DL104930.1 | AX114855.1 | GM641758.1 | HV511154.1 | GM639069.1 | HB865557.1 | DL092762.1 | AX418274.1 | HW049354.1 |
| DL100388.1 | AX113853.1 | GM641726.1 | HV507921.1 | GM626522.1 | HB865043.1 | DL089019.1 | AX417907.1 | HW049322.1 |
| DL100356.1 | AX113666.1 | GM641694.1 | HV514027.1 | GM626490.1 | HB864961.1 | DL088987.1 | AX399448.1 | HW049290.1 |
| DL021070.1 | AX112056.1 | GM641661.1 | HH834133.1 | GM626458.1 | HB865003.1 | DL117868.1 | AX398662.1 | HW049258.1 |
| DL021038.1 | AX111605.1 | GM641629.1 | HH833904.1 | GM626426.1 | HB864947.1 | DL117836.1 | AX392375.1 | HW041797.1 |
| DL016736.1 | AX109083.1 | GM634441.1 | FW392871.1 | GM626394.1 | HB864915.1 | DL108154.1 | AX391604.1 | HW041765.1 |
| DL012182.1 | AX109019.1 | GM634409.1 | HH930550.1 | GM626362.1 | HB864883.1 | DL108122.1 | AX384570.1 | HW041429.1 |
| DL012150.1 | AX108716.1 | GM706360.1 | HH821151.1 | GM626330.1 | HB864851.1 | DL108090.1 | AX375510.1 | HW045682.1 |
| DL012118.1 | AX107886.1 | FB742342.1 | HH821900.1 | GM660355.1 | HB864819.1 | DL103484.1 | AX374850.1 | HW054041.1 |
| DL012086.1 | AX107386.1 | GM706321.1 | FW379605.1 | GM653168.1 | HB864787.1 | DL103452.1 | AX370390.1 | HW044047.1 |
| DL011932.1 | AX107104.1 | GM601156.1 | FW378089.1 | GM653136.1 | HB864755.1 | DL103420.1 | AX364671.1 | HV985969.1 |
| DL011900.1 | AX103651.1 | FB720270.1 | FW377076.1 | GM653104.1 | HB859736.1 | DL098814.1 | AX364554.1 | HV985937.1 |
| DL024279.1 | AX100796.1 | FB719726.1 | FW381496.1 | GM653072.1 | HB866935.1 | DL098782.1 | AX364490.1 | HV985905.1 |
| DL024247.1 | AX100173.1 | FB717780.1 | FW376112.1 | GM653040.1 | HB866548.1 | DL098750.1 | AX364458.1 | HV985860.1 |
| DL024215.1 | AX100014.1 | FB715248.1 | FW375408.1 | GM652976.1 | HC000341.1 | DL092708.1 | AX364426.1 | HW028883.1 |
| DL016406.1 | AX097531.1 | FB706329.1 | FW380414.1 | GM639014.1 | HB999696.1 | DL088965.1 | AX364394.1 | HV984391.1 |
| DL016342.1 | AX093433.1 | FB677540.1 | HC754650.1 | GM638982.1 | HB977616.1 | DL088933.1 | AX364267.1 | HV984267.1 |
| DL016310.1 | AX093021.1 | FB677443.1 | HC741973.1 | GM638950.1 | HB977096.1 | DL107964.1 | AX364235.1 | HV984231.1 |
| DL016278.1 | AX088808.1 | DL460033.1 | HC732138.1 | GM638918.1 | HB976706.1 | DL107932.1 | AX364203.1 | HV961492.1 |
| DL016246.1 | AX088762.1 | DL262833.1 | HV985961.1 | GM638886.1 | DM201540.1 | DL041083.1 | AX358355.1 | HV969520.1 |
| DL011730.1 | AX088710.1 | DL260643.1 | HV985929.1 | GM638854.1 | DM193213.1 | DL037132.1 | AX357907.1 | HV960875.1 |
| DL011698.1 | AX088387.1 | DL260149.1 | HV985897.1 | GM626143.1 | DM189754.1 | DL028665.1 | BD008863.1 | AY658221.1 |
| DL011666.1 | AX085615.1 | DL260069.1 | HV974818.1 | GM660310.1 | HB840189.1 | DL028633.1 | BD006311.1 | AY658189.1 |
| DL011634.1 | AX084354.1 | DL259350.1 | HV335489.1 | GM660278.1 | HB848042.1 | DL028601.1 | E59060.1 | AY658157.1 |
| DL011602.1 | AX083739.1 | DL258280.1 | HV322832.1 | GM660246.1 | HB839620.1 | DL028569.1 | E51994.1 | DL117378.1 |
| DJ402637.1 | AX082495.1 | DL257633.1 | HV321822.1 | GM660214.1 | HB847599.1 | DL048688.1 | E54525.1 | DL117346.1 |
| DJ402288.1 | AX081678.1 | DL241389.1 | HV333669.1 | GM660182.1 | HB847291.1 | DL048656.1 | E59812.1 | DL117314.1 |
| DJ416228.1 | AX081160.1 | DL096727.1 | HV333568.1 | GM660150.1 | HB838877.1 | DL045206.1 | AX356078.1 | DL117282.1 |
| DJ400830.1 | AX080809.1 | DL096695.1 | HV325791.1 | GM660327.1 | HB832415.1 | DL045174.1 | AX354615.1 | DL117250.1 |
| DJ400798.1 | AX080577.1 | DL096663.1 | HV325338.1 | GM652944.1 | HB846753.1 | DL045142.1 | AX353664.1 | DL117218.1 |
| DJ399771.1 | AX078393.1 | DL096631.1 | HV323570.1 | GM652912.1 | HB838307.1 | DL045110.1 | AX347574.1 | DL091637.1 |
| DJ391347.1 | AX077302.1 | DL096599.1 | HV313355.1 | GM652880.1 | HB846316.1 | DL037079.1 | AX347312.1 | DL091605.1 |
| DJ417999.1 | AX074258.1 | DL096567.1 | HV187418.1 | GM652848.1 | HB846066.1 | DL037047.1 | AX347274.1 | DL087962.1 |
| DJ390558.1 | AX073940.1 | DL094739.1 | HV192770.1 | GM652816.1 | HB837979.1 | DL037015.1 | AX347238.1 | DL087930.1 |
| DJ402720.1 | AJ296084.1 | DL094707.1 | HV245469.1 | DL105674.1 | HB845942.1 | DL028356.1 | AX347204.1 | DL121761.1 |
| DJ381031.1 | AX068437.1 | DL094675.1 | HV227294.1 | DL105610.1 | HB845524.1 | DL021170.1 | AX347168.1 | DL121697.1 |
| DJ380999.1 | AX068161.1 | DL094643.1 | HV236082.1 | DL100901.1 | HB826498.1 | DL021138.1 | AX346966.1 | DL121665.1 |
| DJ380967.1 | AX063435.1 | DL094611.1 | HV306174.1 | DL100869.1 | HB845948.1 | DL021106.1 | HW262564.1 | DL121601.1 |
| DJ380935.1 | AX061579.1 | DL094579.1 | HV302968.1 | DL100837.1 | HB845140.1 | DL021074.1 | HW261398.1 | DL087710.1 |

| | | | | | | | | |
|---|---|---|---|---|---|---|---|---|
| DJ380903.1 | AX060262.1 | DL094547.1 | HV302936.1 | DL100805.1 | HB844799.1 | DL021042.1 | HW261366.1 | DL125449.1 |
| DJ380871.1 | AX067781.1 | DL090932.1 | HV312692.1 | DL100773.1 | HB844364.1 | DL021010.1 | HW261334.1 | DL125417.1 |
| DJ380819.1 | AX058577.1 | DL090900.1 | HV308615.1 | DL100741.1 | DL119417.1 | DL019573.1 | HW261302.1 | DL125385.1 |
| DJ389470.1 | AX057306.1 | DL086964.1 | HV235785.1 | DL096327.1 | DL114822.1 | DL019541.1 | HW261270.1 | DL125353.1 |
| CS726879.1 | AX056838.1 | DL086932.1 | HI504571.1 | DL096295.1 | DL114790.1 | DL019509.1 | HW261238.1 | DL113821.1 |
| CS724485.1 | AX055862.1 | DL086900.1 | HI462681.1 | DL096231.1 | DL114758.1 | DL019477.1 | HW261206.1 | DL113789.1 |
| CS724452.1 | AX052965.1 | DL086868.1 | HH716009.1 | DL096199.1 | DL114726.1 | DL019445.1 | HW261174.1 | DL113749.1 |
| CS724407.1 | DL041220.1 | DL086836.1 | HH961344.1 | DL096167.1 | DL114694.1 | DL019413.1 | HW261142.1 | DL113693.1 |
| CS723817.1 | DL041188.1 | DL086804.1 | HH961280.1 | DL120187.1 | DL114662.1 | DL014914.1 | HW261110.1 | DL113661.1 |
| DJ357677.1 | DL041156.1 | DL086772.1 | HH975168.1 | DL120155.1 | DL123464.1 | DL014882.1 | HW261078.1 | DL108860.1 |
| DJ357613.1 | DL041124.1 | DL086740.1 | HH964277.1 | DL120123.1 | DL123400.1 | DL026468.1 | HW261046.1 | DL108796.1 |
| DJ357505.1 | DL041092.1 | DL086708.1 | HH982028.1 | DL120091.1 | DL123368.1 | DL026436.1 | HW261014.1 | DL108764.1 |
| DJ361203.1 | DL037269.1 | DL086676.1 | HH974539.1 | DL120059.1 | DL119114.1 | DL026372.1 | HW260982.1 | DL108732.1 |
| DJ360415.1 | DL037237.1 | DL086612.1 | HH998108.1 | DL120027.1 | DL119082.1 | DL022560.1 | HW260950.1 | DL108700.1 |
| DJ339850.1 | DL037205.1 | DL105885.1 | HH998065.1 | DL094345.1 | DL119050.1 | DL022528.1 | HW260918.1 | DL104054.1 |
| DJ339818.1 | DL037173.1 | DL105853.1 | HH998004.1 | DL094313.1 | DL119018.1 | DL014647.1 | HW260886.1 | DL099520.1 |
| DJ339754.1 | DL037141.1 | DL105821.1 | HH997943.1 | DL094281.1 | DL114622.1 | DL010172.1 | HW260854.1 | DL099488.1 |
| DJ339722.1 | DL037109.1 | DL105789.1 | HH999651.1 | DL094249.1 | DL114590.1 | DL010140.1 | HW260822.1 | DL099456.1 |
| DJ339658.1 | DL032874.1 | DL105757.1 | HH997902.1 | DL094217.1 | DL114558.1 | DL010108.1 | HW260790.1 | DL099424.1 |
| DD327022.1 | DL032842.1 | DL105725.1 | HH997765.1 | DL094185.1 | DL114526.1 | DL010076.1 | HW260758.1 | DL089543.1 |
| DD326926.1 | DL032810.1 | DL101016.1 | HH999505.1 | DL094153.1 | DL114494.1 | DL010044.1 | HW260726.1 | DL089511.1 |
| DD321796.1 | DL032778.1 | DL100984.1 | HH999447.1 | DL090470.1 | DL114462.1 | DL010012.1 | HW260694.1 | DL089479.1 |
| DD325703.1 | DL032746.1 | DL100952.1 | HH999318.1 | DL090438.1 | DL011726.1 | DL014514.1 | HW260662.1 | DL089447.1 |
| CS389308.1 | DL028738.1 | DL096538.1 | HH997644.1 | DL090406.1 | DL011694.1 | DL014482.1 | HW260630.1 | DL089415.1 |
| CS389273.1 | DL028706.1 | DL096506.1 | HH997592.1 | DL090374.1 | DL011662.1 | DL014450.1 | CS625503.1 | DL089383.1 |
| CS389222.1 | DL028674.1 | DL096474.1 | HH997426.1 | DL086369.1 | DL011630.1 | DL009864.1 | DD438019.1 | DL113645.1 |
| CS389183.1 | DL028610.1 | DL096442.1 | HH997382.1 | DL086337.1 | DL011598.1 | DL009832.1 | DD449077.1 | DL113613.1 |
| CS399238.1 | DL028578.1 | DL096410.1 | HH999693.1 | DL086305.1 | DL048453.1 | DL009800.1 | DD437118.1 | DL113581.1 |
| CS401600.1 | DL028546.1 | DL096378.1 | HH999149.1 | DL086273.1 | DL044740.1 | DL009768.1 | DD435634.1 | DL113549.1 |
| CS406512.1 | DL024700.1 | DL096346.1 | HH998115.1 | DL086241.1 | DL044708.1 | DL009544.1 | CS620285.1 | DL113517.1 |
| CS403742.1 | DL024668.1 | DL094518.1 | HH999073.1 | DL086209.1 | DL044492.1 | DL009512.1 | CS616587.1 | DL103758.1 |
| CS403483.1 | DL024636.1 | DL094486.1 | HH999028.1 | DL110452.1 | DL044265.1 | DL009480.1 | CS614354.1 | DL099306.1 |
| CS402167.1 | DL024604.1 | DL094454.1 | HH998961.1 | DL110420.1 | DL040089.1 | DL009448.1 | CS611724.1 | DL093142.1 |
| CS382089.1 | DL024572.1 | DL094422.1 | HH998919.1 | DL110388.1 | DL032032.1 | DL009416.1 | CQ757638.1 | DL093110.1 |
| CS380030.1 | DL021360.1 | DL094390.1 | HH997179.1 | DL110356.1 | DL032000.1 | CS503210.1 | DD214007.1 | DL093078.1 |
| CS382607.1 | DL021328.1 | DL094358.1 | HH997138.1 | DL110324.1 | DL031968.1 | CS502740.1 | DD213990.1 | DL089271.1 |
| CS376541.1 | DL021296.1 | DL110847.1 | HH998914.1 | DL110292.1 | DL028127.1 | CS502708.1 | DD213574.1 | DL089239.1 |
| DD298335.1 | DL021264.1 | DL110815.1 | HH998861.1 | DL105487.1 | DL028095.1 | CS502608.1 | DD218338.1 | DL125930.1 |
| DD292509.1 | DL021232.1 | DL110783.1 | HH998804.1 | DL105455.1 | DL028063.1 | CS502364.1 | DD215944.1 | DL125898.1 |
| DD291441.1 | DL016930.1 | DL110751.1 | HH998751.1 | DL105423.1 | DL027999.1 | CS502266.1 | DD222023.1 | DL125866.1 |
| DD319813.1 | DL016898.1 | DL110687.1 | HH997091.1 | DL105391.1 | DL027967.1 | CS501241.1 | DD212237.1 | DL125834.1 |
| CS375983.1 | DL016834.1 | DL090749.1 | HH996992.1 | HW350685.1 | DL044232.1 | CS500050.1 | DD182410.1 | DL125802.1 |
| CS375791.1 | DL012376.1 | DL090717.1 | HH996943.1 | HW350651.1 | DL044200.1 | CS498516.1 | DD206909.1 | DL125770.1 |
| CS362712.1 | DL012344.1 | DL090685.1 | HH998700.1 | HW350556.1 | DL044168.1 | CS498385.1 | DD206877.1 | DL122560.1 |
| CS360562.1 | DL012312.1 | DL090653.1 | HH998659.1 | HW340186.1 | DL048241.1 | DD418612.1 | DD206845.1 | DL122528.1 |
| CS359737.1 | DL012280.1 | DL090621.1 | HH998535.1 | HW350341.1 | DL048209.1 | DD418577.1 | DD206783.1 | DL118114.1 |
| HI935093.1 | DL060845.1 | DL090589.1 | HH996917.1 | HW339556.1 | DL048177.1 | DD412328.1 | DD196873.1 | DL118082.1 |

| | | | | | | | | |
|---|---|---|---|---|---|---|---|---|
| HC920304.1 | DL048825.1 | DL086584.1 | HH996761.1 | HW349544.1 | DL048145.1 | DD411519.1 | CS247805.1 | DL118018.1 |
| HD057681.1 | DL048793.1 | DL086552.1 | HH977560.1 | HW348560.1 | CS810627.1 | DD411470.1 | DD181009.1 | DL012186.1 |
| HD061086.1 | DL048761.1 | DL086520.1 | HH980629.1 | HW347999.1 | CS812989.1 | DD414317.1 | AY967276.1 | DL012154.1 |
| U34829.1 | DL048729.1 | DL086456.1 | HH980537.1 | HW347798.1 | FB292419.1 | DD410178.1 | AY967244.1 | DL012122.1 |
| M36415.1 | DL048697.1 | DL086424.1 | HH980459.1 | HW347766.1 | CS811264.1 | CS491152.1 | AY967212.1 | DL012090.1 |
| M54790.1 | DL048665.1 | DL115622.1 | HH987328.1 | HW347605.1 | DJ021066.1 | CS483005.1 | AY967180.1 | DL012058.1 |
| HC918497.1 | DL045247.1 | DL115558.1 | HH980350.1 | HW347573.1 | CS803358.1 | CS482966.1 | AY967148.1 | DL012026.1 |
| HC917439.1 | DL045215.1 | DL115526.1 | HH980290.1 | HW363619.1 | CS803325.1 | CS482931.1 | AY967116.1 | DL011904.1 |
| FW341966.1 | DL045183.1 | DL115494.1 | HH980215.1 | HW347336.1 | FB293312.1 | CS482891.1 | AY967084.1 | DL011872.1 |
| FW337321.1 | DL045151.1 | DL115462.1 | HH980150.1 | HW355195.1 | DJ052627.1 | DD401436.1 | AY967052.1 | DL024283.1 |
| HC887858.1 | DL045119.1 | DL115437.1 | HH980098.1 | HW317056.1 | DJ046976.1 | DD401404.1 | AY967020.1 | DL024251.1 |
| HC887339.1 | DL045087.1 | DL115405.1 | HH979865.1 | HW326517.1 | DJ050558.1 | DD401181.1 | AY966988.1 | DL024219.1 |
| HC873820.1 | DL040975.1 | DL115373.1 | HH996697.1 | HW338870.1 | DJ049439.1 | DD405187.1 | AY966956.1 | DL016410.1 |
| HC880390.1 | DL040943.1 | DL115341.1 | HH996636.1 | HW338742.1 | DJ048828.1 | DD405155.1 | AY966924.1 | DL016378.1 |
| HC870899.1 | DL040911.1 | DL115309.1 | HH996581.1 | HW338614.1 | DJ048796.1 | DD405123.1 | CS052527.1 | DL016346.1 |
| HC191141.1 | DL040879.1 | DL115277.1 | HH998443.1 | HV550407.1 | CS793495.1 | DD405059.1 | CS052406.1 | DL016250.1 |
| HC190465.1 | DL037056.1 | DL115245.1 | HH998392.1 | AY659408.1 | CS701572.1 | DM060736.1 | CS052372.1 | DL028151.1 |
| HC089418.1 | DL037024.1 | DL110643.1 | HH998358.1 | AY659376.1 | CS800049.1 | DM060704.1 | CS052304.1 | DL011734.1 |
| HC089576.1 | DL036992.1 | DL110611.1 | HH999987.1 | AY659344.1 | CS707373.1 | DM060672.1 | CS048046.1 | DL011702.1 |
| HC089544.1 | DL036960.1 | DL110579.1 | HH999933.1 | AY659312.1 | CS798833.1 | GN067919.1 | CS047276.1 | DL011670.1 |
| HC089512.1 | DL036928.1 | DL105678.1 | HH993941.1 | AY659280.1 | CS716760.1 | GN067855.1 | CS039430.1 | DL011638.1 |
| HC089480.1 | DL036896.1 | DL105646.1 | HH979792.1 | AY659248.1 | CS806181.1 | GN065104.1 | CS038912.1 | DL011606.1 |
| HC089448.1 | DL032693.1 | DL105614.1 | HH979706.1 | AY659216.1 | CS796320.1 | GN060300.1 | CS036959.1 | DL044812.1 |
| HC089415.1 | DL032661.1 | DL105582.1 | GN360118.1 | AY659184.1 | CS675436.1 | GN059993.1 | CS023785.1 | DL044780.1 |
| HC089383.1 | DL034894.1 | DL105550.1 | GN360086.1 | AY659152.1 | CS680723.1 | GN051712.1 | CS023579.1 | DL044748.1 |
| HC089351.1 | DL034862.1 | DL105518.1 | GN366857.1 | AY659120.1 | CS791409.1 | GN047431.1 | CS018314.1 | DL044716.1 |
| HC089318.1 | DL034838.1 | DL100905.1 | GN360045.1 | AY659088.1 | CS792470.1 | DM036360.1 | CS017700.1 | DL044684.1 |
| HC089286.1 | DL034798.1 | DL089720.1 | GN359981.1 | AY659056.1 | CS791946.1 | DM017755.1 | CS016570.1 | DL034329.1 |
| HC089254.1 | DL034766.1 | DL089688.1 | GN359949.1 | AY659024.1 | DJ031566.1 | DM010743.1 | AF430181.1 | DL009381.1 |
| AY774940.1 | DL034734.1 | DL089656.1 | GN359840.1 | AY658992.1 | DJ031175.1 | DM022180.1 | AF430149.1 | DL009349.1 |
| AY774900.1 | DL034702.1 | DL089624.1 | GN359808.1 | AY658960.1 | DJ044922.1 | DM015973.1 | AX505251.1 | DL009317.1 |
| AY774838.1 | DL030698.1 | DL089592.1 | GN359660.1 | AY658928.1 | DJ044797.1 | DM026252.1 | AX505187.1 | DL009285.1 |
| AY774725.1 | DL030538.1 | DL126112.1 | GN359564.1 | AY658896.1 | DJ033725.1 | GN044856.1 | AX498156.1 | DL009253.1 |
| AY774615.1 | DL026729.1 | DL126080.1 | DL018638.1 | AY658864.1 | DJ029508.1 | GN043667.1 | AX496848.1 | DL009221.1 |
| AY774443.1 | DL026697.1 | DL126048.1 | DL018606.1 | AY658832.1 | CS691329.1 | GN041741.1 | BD140317.1 | DL018679.1 |
| AY774326.1 | DL026665.1 | DL126016.1 | DL014178.1 | AY658800.1 | CS275485.1 | GN041621.1 | BD137885.1 | DL018647.1 |
| AY774160.1 | DL026633.1 | DL125984.1 | DL014146.1 | AY658768.1 | CS274784.1 | GN040015.1 | BD137233.1 | DL018615.1 |
| AY774037.1 | DL026601.1 | DL125952.1 | DL042642.1 | AY658736.1 | CS263127.1 | GN037276.1 | BD135128.1 | DL014219.1 |
| AY515502.1 | DL026569.1 | DL122760.1 | DL042578.1 | AY658704.1 | CS252530.1 | GN030741.1 | BD133622.1 | HI003321.1 |
| M22196.1 | DL022757.1 | DL122704.1 | DL038686.1 | AY658672.1 | CS249799.1 | GN030645.1 | BD131929.1 | HI550491.1 |
| HC083552.1 | DL022725.1 | DL122672.1 | DL034125.1 | AY658640.1 | CS248876.1 | GN030613.1 | BD130790.1 | HI464727.1 |
| HC070446.1 | DL022693.1 | DL122640.1 | DL018305.1 | AY658608.1 | CS246000.1 | GN030549.1 | BD130758.1 | HI464695.1 |
| HC083722.1 | DL022661.1 | DL122608.1 | DL018273.1 | AY658576.1 | CS245370.1 | GN030517.1 | BD130692.1 | HI464663.1 |
| HC083545.1 | DL022629.1 | DL118386.1 | DL018241.1 | AY658544.1 | CS244203.1 | GN030325.1 | BD130594.1 | HI505458.1 |
| U26409.1 | DL022597.1 | DL118354.1 | DL018209.1 | AY658512.1 | CS243765.1 | GM656022.1 | BD129642.1 | HI549986.1 |
| HC059642.1 | DL019582.1 | DL118322.1 | DD401618.1 | AY658480.1 | CS237712.1 | GM655990.1 | BD084032.1 | HI542544.1 |
| HC057022.1 | DL019550.1 | DL118290.1 | DD401586.1 | AY658448.1 | CS229854.1 | GM655958.1 | BD081980.1 | HI503813.1 |

| | | | | | | | | |
|---|---|---|---|---|---|---|---|---|
| HB469141.1 | DL019518.1 | DL118258.1 | DD401554.1 | AY658416.1 | CS228038.1 | GM648965.1 | BD080660.1 | HH716017.1 |
| HB468766.1 | DL019486.1 | DL118226.1 | DD401522.1 | AY658384.1 | CS203483.1 | GM648933.1 | BD080225.1 | HH961382.1 |
| HB461513.1 | DL019454.1 | DL113830.1 | DD401490.1 | AY658352.1 | CS227321.1 | GM648901.1 | BD075326.1 | HH961350.1 |
| HB455018.1 | DL019422.1 | DL113798.1 | DD401458.1 | AY658320.1 | CS208886.1 | GM648869.1 | BD074970.1 | HH961318.1 |
| HB451913.1 | DL015019.1 | DL113726.1 | CS479864.1 | AY658288.1 | CS207910.1 | GM648837.1 | BD074935.1 | HH961286.1 |
| DM143443.1 | DL014987.1 | DL113758.1 | CS476261.1 | AY658256.1 | CS189617.1 | GM648805.1 | BD023240.1 | HH961254.1 |
| DM121638.1 | DL014955.1 | DL113702.1 | CS463947.1 | AY658224.1 | CS193218.1 | GM634654.1 | BD016718.1 | HH961136.1 |
| DM136186.1 | DL014923.1 | DL113670.1 | CS464583.1 | AY658192.1 | CS174647.1 | GM634622.1 | BD014472.1 | HH975198.1 |
| HB434955.1 | DL014891.1 | DL108869.1 | DD400148.1 | AY658160.1 | CS163780.1 | GM634590.1 | BD014217.1 | HC049368.1 |
| HB416488.1 | DL014859.1 | DL108837.1 | DD394229.1 | AY658128.1 | CS159792.1 | GM634526.1 | BD014182.1 | HC041926.1 |
| HB416445.1 | DL010401.1 | DL108805.1 | CS462149.1 | AY658096.1 | CS159028.1 | GM634494.1 | BD014022.1 | HC047456.1 |
| HB412586.1 | DL010337.1 | DL108773.1 | CS460061.1 | AY658064.1 | CS155782.1 | GM629689.1 | AX490798.1 | HC047424.1 |
| HB394300.1 | DL010305.1 | DL108741.1 | DD361279.1 | AY658032.1 | CS146530.1 | GM664861.1 | AX474385.1 | HC047392.1 |
| HB394268.1 | HI002195.1 | DL108709.1 | DD367602.1 | AY658000.1 | CS144305.1 | GM664694.1 | E58952.1 | HC047360.1 |
| HB394236.1 | HI002152.1 | DL104023.1 | DD363151.1 | AY657968.1 | CS141705.1 | GM648741.1 | AX468156.1 | HC047328.1 |
| GN031426.1 | HI002114.1 | DL104031.1 | DD361661.1 | AY657936.1 | CS141518.1 | GM648709.1 | AX464729.1 | HC047296.1 |
| GN031394.1 | HI000459.1 | DL103975.1 | A10882.1 | AY657904.1 | CS141486.1 | GM648677.1 | AX463610.1 | HC047264.1 |
| GN031362.1 | HI000411.1 | DL103943.1 | CS456724.1 | AY657872.1 | DD165138.1 | GM648645.1 | A22054.1 | HC047040.1 |
| GN031297.1 | HI000369.1 | DL103911.1 | CS450652.1 | AY657840.1 | DD159532.1 | GM648613.1 | AX458727.1 | HC047008.1 |
| GN031265.1 | HI000312.1 | DL099497.1 | DD360941.1 | AY657808.1 | DD176020.1 | GM634461.1 | AX458616.1 | HC046976.1 |
| GN031233.1 | HI553318.1 | DL099465.1 | DD357912.1 | AY657776.1 | CS231035.1 | GM634429.1 | AX454050.1 | HC046944.1 |
| GN031201.1 | HI553265.1 | DL099433.1 | CS447657.1 | AY657744.1 | CS188243.1 | GM634397.1 | HV777035.1 | HC046912.1 |
| GN031169.1 | HI553193.1 | DL099401.1 | A16530.1 | AY657712.1 | AF397138.1 | GM634365.1 | HV775605.1 | HC046884.1 |
| GN031136.1 | HI521565.1 | DL099369.1 | BD325608.1 | AY657680.1 | CS157788.1 | GM629432.1 | HW287349.1 | HC046852.1 |
| GN031104.1 | HI539187.1 | DL099337.1 | BD314352.1 | AY657648.1 | CS157949.1 | GM629336.1 | HW302522.1 | HC046820.1 |
| GN031072.1 | HI207707.1 | DL093327.1 | BD312676.1 | AY657616.1 | CS157917.1 | GM648567.1 | HW295692.1 | HC046788.1 |
| GN031040.1 | HI583945.1 | DL093295.1 | BD319280.1 | AY657584.1 | CS157885.1 | GM648535.1 | HW302340.1 | HC046756.1 |
| GN031008.1 | HI520871.1 | DL089552.1 | BD307808.1 | AY657552.1 | CS157853.1 | GM648503.1 | HW302012.1 | HC046724.1 |
| GN030976.1 | HI520489.1 | DL089520.1 | BD306307.1 | AY657520.1 | CS124666.1 | GM629163.1 | HW295006.1 | HC046692.1 |
| GN030881.1 | HI207616.1 | DL089488.1 | BD300348.1 | AY657488.1 | CS124632.1 | GM629131.1 | HW285858.1 | HC046675.1 |
| GN030817.1 | HI003705.1 | DL089424.1 | BD291305.1 | AY657456.1 | CS124544.1 | GM629099.1 | FZ435953.1 | HC046643.1 |
| DM008343.1 | HI002081.1 | DL089392.1 | BD299632.1 | AY657424.1 | CS124044.1 | GM634061.1 | FZ437285.1 | HC046611.1 |
| DM001975.1 | HI002029.1 | DL118210.1 | BD295287.1 | AY657392.1 | CS119872.1 | CS696161.1 | FZ422905.1 | HC046579.1 |
| DM001670.1 | HI001946.1 | DL113622.1 | BD277108.1 | AY657360.1 | CS119501.1 | CS696129.1 | FZ415471.1 | HC046547.1 |
| DM006938.1 | HI000278.1 | DL113590.1 | BD276247.1 | AY657328.1 | CS119468.1 | CS696065.1 | HI401108.1 | HC046515.1 |
| DM000511.1 | HI000238.1 | DL113558.1 | BD274096.1 | AY657296.1 | CS119434.1 | CS696000.1 | HI637687.1 | HC046483.1 |
| DM005002.1 | HI000194.1 | DL113526.1 | BD273642.1 | AY657264.1 | CS119401.1 | CS695936.1 | HI380627.1 | HC046419.1 |
| DM003491.1 | HI000138.1 | DL113494.1 | BD273228.1 | AY657232.1 | CS119368.1 | CS695872.1 | HI177964.1 | HC046387.1 |
| GM997883.1 | HI205889.1 | DL113462.1 | BD272913.1 | AY657200.1 | CS119302.1 | CS695840.1 | HI214582.1 | HC046355.1 |
| GM996195.1 | HI471212.1 | DL108565.1 | BD271970.1 | AY657168.1 | CS119238.1 | CS695808.1 | HI214550.1 | HC046323.1 |
| GM995278.1 | HI180966.1 | DL108533.1 | DD231197.1 | AY657136.1 | CS119174.1 | CS695776.1 | HI214452.1 | HC046072.1 |
| GM995115.1 | HI539013.1 | DL103895.1 | DD229821.1 | AY657104.1 | CS119142.1 | CS695744.1 | HI214420.1 | HC046248.1 |
| GM992934.1 | HI470575.1 | DL103863.1 | DD227723.1 | AY657072.1 | CS119110.1 | CS695712.1 | HI185495.1 | HC046216.1 |
| GM992450.1 | HI544684.1 | DL103831.1 | DD227407.1 | AY657040.1 | CS119075.1 | CS695616.1 | HI212985.1 | HC046152.1 |
| GM992240.1 | HI564714.1 | DL103799.1 | DD224453.1 | U05289.1 | CS119042.1 | CS695552.1 | HI546337.1 | HC046120.1 |
| GM987032.1 | HI568935.1 | DL103767.1 | DD223496.1 | M19553.1 | CQ947154.1 | CS695520.1 | HI546305.1 | HC046002.1 |
| GN000541.1 | HI568860.1 | DL103735.1 | DD233349.1 | M11516.1 | CQ947115.1 | CS695424.1 | HI587864.1 | HC045970.1 |

| | | | | | | | | |
|---|---|---|---|---|---|---|---|---|
| GM642990.1 | HI559131.1 | DL103712.1 | DD231463.1 | AX468324.1 | CQ944185.1 | CS695392.1 | HI210983.1 | HC045938.1 |
| GM642957.1 | HI564213.1 | DL099225.1 | CQ757642.1 | AX466455.1 | CQ944121.1 | DL176499.1 | HI210951.1 | HC045906.1 |
| GM642925.1 | HC769805.1 | DL099193.1 | A10473.1 | AX463662.1 | CQ944089.1 | DL176354.1 | HI570427.1 | HC045874.1 |
| GM630649.1 | HC767574.1 | DL099161.1 | BD182993.1 | AX458653.1 | CQ944057.1 | DL176280.1 | HI473086.1 | HC045810.1 |
| GM630617.1 | HC767347.1 | DL093151.1 | AF548059.1 | AX458539.1 | CQ944025.1 | DL174619.1 | HI472924.1 | HC045778.1 |
| A27630.1 | HC755784.1 | DL093119.1 | BD181608.1 | AX456520.1 | CQ943993.1 | DL182939.1 | HI210927.1 | HC045746.1 |
| A20021.1 | HC734609.1 | DL093087.1 | BD180854.1 | HW347604.1 | CQ943961.1 | DL176765.1 | HI580538.1 | HC045714.1 |
| A26778.1 | HC733774.1 | DL093055.1 | BD180766.1 | HW363681.1 | CQ943929.1 | DL176668.1 | HI209401.1 | HC045684.1 |
| A26058.1 | HC732413.1 | DL089344.1 | AX743757.1 | HW363505.1 | CQ943897.1 | FB355742.1 | HI004343.1 | HC045652.1 |
| A24315.1 | HC731349.1 | DL089312.1 | AX742977.1 | HW347337.1 | CQ943865.1 | DL088341.1 | HI002261.1 | HC045620.1 |
| A25917.1 | HC731317.1 | DL089280.1 | BD177332.1 | HW355232.1 | CQ898671.1 | DL088085.1 | HI002217.1 | HC045588.1 |
| A24542.1 | HC731285.1 | DL089248.1 | AX703580.1 | HW355182.1 | CQ898639.1 | DL088053.1 | HI002169.1 | HC045556.1 |
| HW068962.1 | HC731253.1 | DL089184.1 | AX699452.1 | HW317055.1 | CQ898607.1 | DL088021.1 | HI002127.1 | HC045524.1 |
| HW067103.1 | HC471778.1 | DL125939.1 | AX699420.1 | HW326510.1 | CQ898575.1 | DL087989.1 | HI000499.1 | DM378909.1 |
| HW088905.1 | HC471746.1 | DL049086.1 | AX685822.1 | HW338610.1 | CQ898273.1 | DL091930.1 | HI000426.1 | HC035668.1 |
| HW083284.1 | HC471684.1 | DL049054.1 | AX664948.1 | HW338098.1 | CQ897149.1 | DL091898.1 | HI000390.1 | HC037104.1 |
| HW083220.1 | HC471706.1 | DL012578.1 | AX664073.1 | HW337842.1 | CQ895549.1 | DL091866.1 | HI203404.1 | HC037003.1 |
| HW081981.1 | HC471650.1 | DL012546.1 | AX662197.1 | HW337714.1 | CQ890955.1 | DL102859.1 | HI553276.1 | HC022640.1 |
| HW081603.1 | HC471618.1 | DL012514.1 | AX657134.1 | HW337330.1 | CQ878658.1 | DL102827.1 | HI583956.1 | HC025502.1 |
| HW099483.1 | HC471504.1 | DL012482.1 | AX657102.1 | HW337202.1 | CQ875528.1 | DL102795.1 | HI564917.1 | HC025470.1 |
| HW103587.1 | HC471564.1 | DL012418.1 | BD176069.1 | HW337074.1 | CQ871856.1 | DL102763.1 | HI207638.1 | HC025438.1 |
| HW084544.1 | HC729289.1 | DL048846.1 | AX648054.1 | HW321815.1 | CQ868896.1 | DL102731.1 | HI003858.1 | HC025406.1 |
| HW084510.1 | HC728805.1 | DL045428.1 | AX643786.1 | HW336946.1 | CQ868458.1 | HB477363.1 | HI002047.1 | HC010424.1 |
| HW102795.1 | HC727692.1 | DL045396.1 | AX615147.1 | HW336818.1 | CQ867413.1 | HB486504.1 | HI001958.1 | HC020910.1 |
| HW102762.1 | HC726070.1 | DL045332.1 | BD171489.1 | HW321647.1 | CQ859636.1 | GM651013.1 | HI000292.1 | GM644398.1 |
| HW084256.1 | HC688452.1 | DL045300.1 | AX601794.1 | HW336662.1 | CQ859604.1 | GM650981.1 | HI000249.1 | GM644366.1 |
| HW102297.1 | HC491010.1 | DL041252.1 | AX601610.1 | HW321190.1 | CQ858045.1 | GM644150.1 | HI000205.1 | GM644238.1 |
| HW065770.1 | HC490978.1 | DL127155.1 | AX328159.1 | HW336454.1 | CQ857387.1 | GM644118.1 | HI000149.1 | GM637208.1 |
| HW065225.1 | HC490946.1 | DL127026.1 | AX318281.1 | HW321149.1 | CQ855866.1 | GM636928.1 | HI000117.1 | GM637144.1 |
| HW058482.1 | HC490914.1 | DL141559.1 | AX306657.1 | HW329466.1 | AX242287.1 | GM636896.1 | HI137489.1 | GM637112.1 |
| HW064827.1 | HC490882.1 | DL141436.1 | AX269004.1 | HW329334.1 | AX242255.1 | GM636864.1 | HI204483.1 | GM637080.1 |
| HW055565.1 | HC686949.1 | DL123544.1 | AX258732.1 | HW335980.1 | AX242223.1 | GM623525.1 | HI203062.1 | GM624927.1 |
| HW056519.1 | HC490842.1 | DL123480.1 | AX303556.1 | HW328819.1 | AX242159.1 | GM623493.1 | HI552629.1 | GM624895.1 |
| HW064723.1 | HC490810.1 | DL123448.1 | AX286662.1 | HW328687.1 | AX242127.1 | GM623461.1 | HB819465.1 | GM624863.1 |
| HW060691.1 | HC490778.1 | DL123384.1 | AX286180.1 | HW328602.1 | AX242063.1 | GM623429.1 | HB845285.1 | GM632646.1 |
| HW062429.1 | HC490746.1 | DL119194.1 | AX283531.1 | HW328356.1 | AX241967.1 | GM623397.1 | HB844787.1 | GM632584.1 |
| HW060503.1 | HC490714.1 | DL119162.1 | AX278264.1 | HW328284.1 | AX241903.1 | GM623365.1 | HB844352.1 | GM632606.1 |
| HW056298.1 | HC490682.1 | DL119130.1 | AX278053.1 | HW335747.1 | AX241839.1 | GM643852.1 | HB836748.1 | GM658456.1 |
| HW064468.1 | HC490650.1 | DL119066.1 | AX255838.1 | HW335659.1 | AX241807.1 | GM643820.1 | HB844032.1 | GM658368.1 |
| HW062369.1 | HC490618.1 | DL119034.1 | BD000461.1 | HW328092.1 | AX241775.1 | GM636823.1 | HB843537.1 | GM651176.1 |
| HW062337.1 | HC490586.1 | DL114638.1 | E59806.1 | HW318496.1 | AX241711.1 | GM636791.1 | HB836172.1 | GM651112.1 |
| HW062241.1 | HC490554.1 | DL114606.1 | AX351223.1 | HW123534.1 | AX241679.1 | GM636759.1 | HB843257.1 | GM651080.1 |
| HW060404.1 | HC490518.1 | DL114574.1 | HW319184.1 | HW123466.1 | AX241647.1 | GM632255.1 | HB843027.1 | GM651048.1 |
| HI564116.1 | HC490486.1 | DL114542.1 | HW328261.1 | HW122705.1 | AX241615.1 | GM632223.1 | HB835383.1 | GM651016.1 |
| HI583119.1 | HC490447.1 | DL114510.1 | HW328109.1 | HW122143.1 | AX241487.1 | GM741834.1 | HB842868.1 | GM650984.1 |
| HI563640.1 | HC490415.1 | DL114478.1 | HW318443.1 | HW121449.1 | AX241455.1 | GM741802.1 | HB842249.1 | GM644153.1 |
| HI563608.1 | HC490383.1 | DL114446.1 | HW314120.1 | HW120356.1 | AX235760.1 | GM658284.1 | HB841887.1 | GM644121.1 |

| | | | | | | | | |
|---|---|---|---|---|---|---|---|---|
| HI563573.1 | HC490351.1 | DL109581.1 | HW312092.1 | HW115956.1 | AX212301.1 | GM658252.1 | HB841666.1 | GM636899.1 |
| HI508526.1 | HC490319.1 | DL109549.1 | HW311870.1 | HW115050.1 | AX203099.1 | GM658220.1 | HB841197.1 | GM636867.1 |
| HI575507.1 | HC490287.1 | DL109517.1 | HW311230.1 | HW072729.1 | AX202483.1 | GM658188.1 | HB841097.1 | GM623528.1 |
| HI538010.1 | HC490254.1 | DL109485.1 | HW307852.1 | HW105046.1 | AX193996.1 | GM658156.1 | HB840563.1 | GM623496.1 |
| HI508385.1 | HC490222.1 | DL104879.1 | HW307820.1 | HW072143.1 | AX190431.1 | GM658106.1 | HB650525.1 | GM623464.1 |
| HI000030.1 | HC472308.1 | DL104847.1 | HW307750.1 | HW071966.1 | AX180723.1 | GM658074.1 | HB647014.1 | GM623432.1 |
| HI003669.1 | HC472275.1 | DL104815.1 | HW307718.1 | HW071536.1 | AX179402.1 | GM658042.1 | HB645730.1 | GM623400.1 |
| HI003621.1 | HC472243.1 | DL095924.1 | HW307686.1 | HW104454.1 | AX175095.1 | GM658010.1 | HB645698.1 | GM623368.1 |
| HI003582.1 | HC472211.1 | DL095892.1 | HW315864.1 | HW104342.1 | AX172747.1 | GM657978.1 | HB856266.1 | GM643887.1 |
| HI003544.1 | HC472179.1 | DL095860.1 | HW315749.1 | HW104308.1 | AX167017.1 | GM657946.1 | HB855969.1 | GM643855.1 |
| HI001763.1 | HC471947.1 | DL095828.1 | HW315655.1 | HW104276.1 | AX164601.1 | GM650754.1 | HB855728.1 | GM643823.1 |
| HI544126.1 | HC471915.1 | DL095796.1 | HW315579.1 | HW071080.1 | AY035208.1 | GM650722.1 | DM170839.1 | GM636826.1 |
| HI001603.1 | HC471883.1 | DL094109.1 | HW315546.1 | HV931278.1 | AX146308.1 | GM650690.1 | DM170807.1 | GM636794.1 |
| HI001560.1 | HC471828.1 | DL094077.1 | HW315185.1 | HV925496.1 | AX145721.1 | GM643698.1 | DM177477.1 | GM636762.1 |
| HI003382.1 | HC471796.1 | DL094045.1 | HW315153.1 | HV936361.1 | AY145518.1 | GM643662.1 | HB559433.1 | GM632226.1 |
| HI003347.1 | HB476884.1 | DL093981.1 | HW315089.1 | HV936302.1 | HH804679.1 | GM636541.1 | HB491549.1 | GM623263.1 |
| HI003306.1 | HB475870.1 | DL090366.1 | HW314697.1 | HV930401.1 | HH797416.1 | GM636509.1 | HB489349.1 | GM623231.1 |
| HI550417.1 | HB475736.1 | DL090334.1 | HW115412.1 | HV814580.1 | HH794938.1 | GM636477.1 | HB488936.1 | GM741869.1 |
| HI464792.1 | HB475458.1 | DL090302.1 | HW115377.1 | HV819212.1 | HH794706.1 | FB579756.1 | DM163993.1 | GM741837.1 |
| HI464718.1 | GM623949.1 | DL090270.1 | HW069937.1 | HV803288.1 | HH793102.1 | DL199983.1 | DM163960.1 | GM741805.1 |
| HI464686.1 | GM632766.1 | DL090238.1 | HW069865.1 | HV932630.1 | HH779961.1 | DL195042.1 | DM163577.1 | GM658319.1 |
| HI464654.1 | GM632734.1 | DL090206.1 | HW069805.1 | HV932590.1 | HH774422.1 | DL199846.1 | DM163290.1 | GM658287.1 |
| HI464597.1 | GM632702.1 | DL090174.1 | HW084927.1 | HV929934.1 | FW381490.1 | DL194816.1 | GM645184.1 | GM658255.1 |
| HI549465.1 | GM625123.1 | DL086169.1 | HW068045.1 | HV932332.1 | FW374779.1 | DL198980.1 | GM625180.1 | GM658223.1 |
| HI542523.1 | GM625091.1 | DL086137.1 | HW049577.1 | HV803148.1 | HH736077.1 | DL193909.1 | GM625148.1 | GM658191.1 |
| HI463142.1 | GM625059.1 | DL086105.1 | HV745330.1 | HV803116.1 | FW368418.1 | DL196191.1 | GM623962.1 | FB983192.1 |
| HI462678.1 | GM625027.1 | DL086073.1 | HV750934.1 | HV781641.1 | FW367507.1 | DL193823.1 | GM637340.1 | FB754403.1 |
| HH725215.1 | GM624995.1 | DL086009.1 | HV747487.1 | HV802946.1 | FW367073.1 | DL193759.1 | GM637308.1 | FB754271.1 |
| HH716004.1 | GM624963.1 | DL114443.1 | HV753621.1 | HV802914.1 | FW361318.1 | DL183880.1 | GM637276.1 | FB753868.1 |
| HH714037.1 | GM624931.1 | DL114411.1 | HV753497.1 | HV802882.1 | FW360602.1 | DL183341.1 | GM658568.1 | GM865638.1 |
| HH961373.1 | GM623905.1 | DL114379.1 | HV705102.1 | HV822842.1 | HD122353.1 | FB511516.1 | GM651312.1 | GM751803.1 |
| HH961341.1 | GM623873.1 | DL114347.1 | HV704318.1 | HV812983.1 | HD122359.1 | FB513368.1 | GM649207.1 | FB727241.1 |
| HH961309.1 | GM623841.1 | DL109450.1 | HV704226.1 | HV822253.1 | HD115791.1 | FB571356.1 | GM635127.1 | FB726024.1 |
| HH961277.1 | GM623809.1 | DL109386.1 | HV704152.1 | HV784497.1 | HD085076.1 | FB571300.1 | GM635095.1 | FB725817.1 |
| HH961245.1 | GM623777.1 | DL099400.1 | HV744534.1 | HV778759.1 | HD084538.1 | FB571240.1 | GM634964.1 | GM842426.1 |
| HH975447.1 | GM623745.1 | DL099368.1 | HV703153.1 | HV778230.1 | HD113813.1 | CS696192.1 | GM656287.1 | FB723056.1 |
| HH964273.1 | GM658924.1 | DL099336.1 | HV701197.1 | HV775033.1 | FW337010.1 | CS696160.1 | DD147707.1 | GM841606.1 |
| HH974536.1 | GM658892.1 | DL093326.1 | HV701165.1 | HV774951.1 | FW336108.1 | CS696032.1 | DD147675.1 | GM879666.1 |
| HH998105.1 | GM658860.1 | DL093294.1 | HV701050.1 | HV764773.1 | HC887332.1 | CS695999.1 | DD081040.1 | GM731609.1 |
| HH997999.1 | GM658828.1 | DL093262.1 | HV700915.1 | HV764741.1 | HC873815.1 | CS695967.1 | DD132332.1 | FB712065.1 |
| HH997939.1 | GM658796.1 | DL089551.1 | HV695512.1 | HV764709.1 | HC873720.1 | CS695935.1 | DD135341.1 | FB711256.1 |
| HH999648.1 | GM651540.1 | DL089519.1 | HV695175.1 | HV566061.1 | FW310609.1 | CS695903.1 | DD132314.1 | GM040849.1 |
| HH997899.1 | GM651508.1 | DL089487.1 | HV502845.1 | HV573536.1 | FW310089.1 | CS695839.1 | DD093861.1 | GM840210.1 |
| HH997828.1 | GM651476.1 | DL089423.1 | HV502801.1 | HV571420.1 | FW308824.1 | CS695807.1 | DD175997.1 | FB709182.1 |
| HH997762.1 | GM651444.1 | DL089391.1 | HV502769.1 | HV560155.1 | FW332943.1 | CS695775.1 | CS204336.1 | CS728646.1 |
| HH999496.1 | GM651412.1 | DL113648.1 | HV502737.1 | HV558749.1 | HC471928.1 | CS695743.1 | CS001504.1 | CS728582.1 |
| HH999443.1 | GM651380.1 | DL113621.1 | HV502705.1 | HV555952.1 | HC471896.1 | CS695711.1 | CS157811.1 | CS727320.1 |

| | | | | | | | | |
|---|---|---|---|---|---|---|---|---|
| HH997703.1 | GM644581.1 | DL113589.1 | HV502545.1 | HV561850.1 | HC471864.1 | CS695583.1 | CS157779.1 | GM036652.1 |
| HH997641.1 | GM644549.1 | DL113557.1 | HV502513.1 | HV554017.1 | HC678815.1 | CS695519.1 | CS157940.1 | GM887747.1 |
| HH997585.1 | GM644517.1 | DL113525.1 | HV452051.1 | HV551421.1 | HC678748.1 | CS695423.1 | CS157908.1 | GM685465.1 |
| HH997531.1 | GM644485.1 | DL113461.1 | HV449936.1 | HV550406.1 | HC510457.1 | CS695391.1 | CS145720.1 | FB983738.1 |
| HH997423.1 | GM644453.1 | DL108564.1 | HV453148.1 | AY659407.1 | HC504674.1 | DL176496.1 | CS126030.1 | FB728858.1 |
| HH997373.1 | GM644421.1 | DL108532.1 | HV449384.1 | AY659375.1 | HC471030.1 | DL176278.1 | CS119816.1 | FB728431.1 |
| HH999690.1 | GM637423.1 | DL108500.1 | HV444134.1 | HC504640.1 | HC500965.1 | DL176763.1 | CS119525.1 | GM680778.1 |
| HH999237.1 | GM637391.1 | DL103894.1 | HV444028.1 | HC494421.1 | HC499893.1 | DL176662.1 | CS119492.1 | GM680729.1 |
| HC047031.1 | GM637359.1 | DL103862.1 | FW562437.1 | HC500312.1 | HC499851.1 | DL124137.1 | CS119459.1 | GM618712.1 |
| HC046999.1 | GM637327.1 | DL103830.1 | FW555358.1 | HC499866.1 | FW299289.1 | DL124105.1 | CS119391.1 | FB709602.1 |
| HC046967.1 | GM637295.1 | DL103798.1 | FW553142.1 | FW301503.1 | HC488006.1 | DL119787.1 | CS119359.1 | GM841787.1 |
| DL088955.1 | GM637263.1 | DL103766.1 | EU363766.1 | FW300962.1 | HC486546.1 | DL119755.1 | CS119327.1 | GM841719.1 |
| DL088923.1 | GM623726.1 | DL103734.1 | HI930658.1 | FW300623.1 | HC486485.1 | DL119723.1 | CS119261.1 | GM604041.1 |
| DL088625.1 | GM623694.1 | DL103711.1 | HI930277.1 | FW299061.1 | HC484122.1 | DL142372.1 | CS119229.1 | GM706611.1 |
| DL088593.1 | GM623662.1 | DL099224.1 | HI935081.1 | FW298770.1 | HC474626.1 | DL119485.1 | CS119197.1 | GM706384.1 |
| DL092350.1 | GM623630.1 | DL099192.1 | AF353576.1 | HC488037.1 | HC491640.1 | DL119453.1 | CS119165.1 | GM603496.1 |
| DL092318.1 | GM623566.1 | DL099160.1 | HI918281.1 | HB855992.1 | HC488313.1 | DL119421.1 | CS119133.1 | FB742332.1 |
| DL092286.1 | GM658715.1 | DL093150.1 | HI574474.1 | DM179151.1 | FV533471.1 | DL146013.1 | CS119066.1 | DL086542.1 |
| DL092254.1 | GM658683.1 | DL093118.1 | HI574442.1 | DM186024.1 | FV531703.1 | DL123668.1 | CS119033.1 | DL086510.1 |
| DL092222.1 | GM658651.1 | DL093086.1 | HI574410.1 | DM178815.1 | FV530917.1 | DL123636.1 | CS119000.1 | DL086478.1 |
| DL092190.1 | GM658619.1 | DL093054.1 | HI574378.1 | DM170823.1 | FV522841.1 | DL123604.1 | CS118935.1 | DL086446.1 |
| DL088415.1 | GM658555.1 | DL089279.1 | HI581381.1 | DM187975.1 | FV522801.1 | DL123572.1 | CS118903.1 | DL086414.1 |
| DL088383.1 | GM651363.1 | DL089247.1 | HI566244.1 | DM177661.1 | FV522618.1 | DL119382.1 | CS118836.1 | DL115548.1 |
| DL121911.1 | GM651331.1 | DL089183.1 | HI636991.1 | HB491792.1 | FV530129.1 | DL119350.1 | CS118803.1 | DL115516.1 |
| DL023424.1 | GM651299.1 | DL125938.1 | HI636950.1 | HB488823.1 | FV529649.1 | DL114826.1 | CS118771.1 | DL115484.1 |
| DL020377.1 | GM644404.1 | DL125906.1 | HI001464.1 | DM164025.1 | DM370681.1 | DL114794.1 | CS118706.1 | DL115452.1 |
| DL020345.1 | GM644372.1 | DL125874.1 | HI001368.1 | DM163976.1 | DM370649.1 | DL114762.1 | CS118642.1 | DL105668.1 |
| DL020313.1 | GM644340.1 | DL125842.1 | HI001334.1 | DM163308.1 | DM370617.1 | DL114730.1 | CS118610.1 | DL105636.1 |
| DL035401.1 | GM644244.1 | DL125810.1 | HI003105.1 | DM163268.1 | DM370585.1 | DL114698.1 | CS118577.1 | DL105604.1 |
| DL035369.1 | GM637246.1 | DL125778.1 | HI003059.1 | DM162327.1 | DM370553.1 | DL114666.1 | CS118542.1 | DL100799.1 |
| DJ444659.1 | GM637214.1 | DL144185.1 | HI003023.1 | DM162212.1 | DM370521.1 | DL095943.1 | CS118509.1 | DL100767.1 |
| DJ444548.1 | GM637182.1 | DL122536.1 | HI002961.1 | HB486512.1 | DM370489.1 | DL123468.1 | CS118476.1 | DL100735.1 |
| DJ434780.1 | GM637150.1 | DL122504.1 | HI002922.1 | HB484915.1 | DM370197.1 | DL123436.1 | CS118379.1 | DL096289.1 |
| DJ433810.1 | GM637086.1 | DL122440.1 | HI001286.1 | HB432572.1 | DM367514.1 | DL123404.1 | CS118345.1 | DL096257.1 |
| DJ438343.1 | GM637054.1 | DL122408.1 | HI001190.1 | HB423118.1 | DM371919.1 | DL123372.1 | HW399756.1 | DL096225.1 |
| DJ438308.1 | GM624901.1 | DL122376.1 | HI001125.1 | HB423080.1 | DM204373.1 | DL119118.1 | HW399354.1 | DL096193.1 |
| DJ438276.1 | GM627972.1 | DL118122.1 | HI001092.1 | HB416459.1 | HC003074.1 | DL119086.1 | HW399280.1 | DL096161.1 |
| DJ402282.1 | GM627940.1 | DL118090.1 | HI001036.1 | HB403496.1 | HC002146.1 | DL119054.1 | HW381566.1 | DL120181.1 |
| DJ400824.1 | GM715475.1 | DL118058.1 | HI001002.1 | HB412944.1 | HB865549.1 | DL119022.1 | HW390494.1 | DL120117.1 |
| DJ400792.1 | GM661750.1 | DL118026.1 | HI000955.1 | HB396995.1 | HB865039.1 | DL114626.1 | HW389047.1 | DL120085.1 |
| DJ393037.1 | GM654756.1 | DL113337.1 | HI002826.1 | HB396565.1 | HB864965.1 | DL114594.1 | HW388658.1 | DL120053.1 |
| DJ391328.1 | GM654724.1 | DL113305.1 | HI002788.1 | HB394812.1 | HB864999.1 | DL114530.1 | HW388326.1 | DL120021.1 |
| DJ418259.1 | GM654692.1 | DL113273.1 | HI002728.1 | HB394277.1 | HB864943.1 | DL114498.1 | HW387494.1 | DL094243.1 |
| DJ387461.1 | GM654660.1 | DL108472.1 | HI002686.1 | HB394245.1 | HB864911.1 | DL114466.1 | HW380921.1 | DL094211.1 |
| DJ380813.1 | GM654628.1 | DL108440.1 | HI002643.1 | HB393701.1 | HB864879.1 | DL104899.1 | HW350607.1 | DL090464.1 |
| CS724401.1 | GM654596.1 | DL108354.1 | HH996624.1 | HB397669.1 | HB864847.1 | DL104867.1 | HW350513.1 | DL090432.1 |
| CS722216.1 | GM654563.1 | DL108344.1 | HH996563.1 | DM114306.1 | HB864815.1 | DL104835.1 | HW339863.1 | DL090368.1 |

| | | | | | | | | |
|---|---|---|---|---|---|---|---|---|
| DJ357125.1 | GM647570.1 | DL108312.1 | HH998512.1 | DM111998.1 | HB864783.1 | DL104803.1 | HW339632.1 | DL086299.1 |
| DD400147.1 | GM627899.1 | DL103642.1 | HH998433.1 | DM118022.1 | HB864751.1 | DL095912.1 | HW355323.1 | DL086267.1 |
| DD394228.1 | GM627867.1 | DL099100.1 | HH998381.1 | DM115807.1 | HB866540.1 | DL095880.1 | HW326330.1 | DL086203.1 |
| CS462148.1 | GM627803.1 | DL099068.1 | HH998348.1 | DM114892.1 | HB866222.1 | DL026748.1 | HW338910.1 | DL110318.1 |
| CS460060.1 | GM871608.1 | DL099036.1 | HH999972.1 | GM668925.1 | HB999690.1 | DL026716.1 | HW338398.1 | DL110286.1 |
| CS459129.1 | GM870419.1 | DL099004.1 | HH999919.1 | GM038733.1 | HB976959.1 | DL026684.1 | HW337758.1 | DL124340.1 |
| DD367949.1 | GM661689.1 | DL098972.1 | HH979837.1 | GM000681.1 | DM193206.1 | DL026652.1 | HW337630.1 | DL124308.1 |
| DD361278.1 | GM661657.1 | DL098940.1 | HH979769.1 | HB388697.1 | DM194715.1 | DL026620.1 | HW337502.1 | DL110169.1 |
| DD367601.1 | GM661625.1 | DL092930.1 | HH979687.1 | HB386991.1 | HB840181.1 | DL026588.1 | HW337118.1 | DL110105.1 |
| DD362657.1 | GM661593.1 | DL092898.1 | HH999886.1 | HB386578.1 | HB848034.1 | DL026556.1 | HW336990.1 | DL105172.1 |
| DD361312.1 | GM661561.1 | DL092866.1 | HH999792.1 | HB385917.1 | HB839612.1 | DL019505.1 | HW321828.1 | DL105140.1 |
| AM235741.1 | GM661529.1 | DL024323.1 | HH999740.1 | HB385772.1 | HB847218.1 | DL019473.1 | HW336706.1 | DL105108.1 |
| CS457177.1 | GM654535.1 | DL024291.1 | HH998310.1 | HB338916.1 | HB838869.1 | DL019441.1 | HW336498.1 | DL013134.1 |
| CS450651.1 | GM654503.1 | DL024227.1 | HH998261.1 | HA643289.1 | HB847133.1 | DL019409.1 | HW321174.1 | DL013102.1 |
| DD360939.1 | GM654471.1 | DL024195.1 | HH998224.1 | HA641612.1 | HB846690.1 | DL014910.1 | HW329271.1 | DL013070.1 |
| DD357911.1 | GM654439.1 | DL045055.1 | HH998177.1 | HA635318.1 | HB838240.1 | DL014878.1 | HW336134.1 | DL013038.1 |
| DD355839.1 | GM654407.1 | DL045023.1 | HH996536.1 | HA639791.1 | HB846303.1 | DL014846.1 | HW335958.1 | DL033741.1 |
| A18868.1 | GM654375.1 | DL044991.1 | HH993647.1 | HA637803.1 | HB837560.1 | DL026496.1 | HW328724.1 | DL029735.1 |
| CS444595.1 | GM647350.1 | DL044959.1 | HH986618.1 | DM102658.1 | HB845852.1 | DL022472.1 | HW125556.1 | DL042143.1 |
| CS438933.1 | GM647318.1 | DL044927.1 | HH979572.1 | GM633439.1 | HB845516.1 | DL014510.1 | HW125370.1 | DL042111.1 |
| CS436045.1 | GM647286.1 | DL040847.1 | FW394284.1 | GM633407.1 | DJ331362.1 | DL014478.1 | HW144357.1 | DL042079.1 |
| CS434851.1 | GM647254.1 | DL040815.1 | FW394191.1 | GM633375.1 | DJ326916.1 | DL014446.1 | HW144325.1 | DL029532.1 |
| DD335093.1 | GM647222.1 | DL040783.1 | FW396390.1 | GM633343.1 | DJ082662.1 | DL009860.1 | HW144293.1 | DL029500.1 |
| DD347409.1 | GM627712.1 | DL036864.1 | FW396276.1 | GM633311.1 | DJ083906.1 | DL009828.1 | HW122876.1 | DL029468.1 |
| DD345399.1 | GM627680.1 | DL036832.1 | FW396657.1 | GM633279.1 | DJ069479.1 | DL009796.1 | HW122040.1 | DL046198.1 |
| DD347269.1 | DL106177.1 | DL032437.1 | FW417623.1 | GM637824.1 | DJ071389.1 | DL009764.1 | HW117987.1 | DL046102.1 |
| DD332614.1 | DL101404.1 | DL032405.1 | GU583855.1 | GM633256.1 | DJ066765.1 | DJ008398.1 | HW101668.1 | DL041956.1 |
| CS425079.1 | DL101372.1 | DL032373.1 | HH834756.1 | GM633224.1 | DJ066402.1 | DJ008366.1 | HW096634.1 | DL041924.1 |
| CS417743.1 | DL101340.1 | DL032341.1 | HH834390.1 | GM633192.1 | DJ066369.1 | DJ008045.1 | HW072880.1 | DL041892.1 |
| DD032249.1 | DL114603.1 | DL024156.1 | HC046088.1 | GM633160.1 | DJ066304.1 | DJ004229.1 | HV763966.1 | DL069435.1 |
| DD030661.1 | DL114571.1 | DL024124.1 | DM380134.1 | GM633128.1 | DJ066270.1 | CS671087.1 | HV766685.1 | DL049709.1 |
| DD020527.1 | DL114539.1 | DL024092.1 | HC025518.1 | GM625389.1 | DJ062856.1 | DD468054.1 | HV766321.1 | DL049677.1 |
| DD009908.1 | DL114507.1 | DL024060.1 | HC025486.1 | GM625357.1 | DJ061477.1 | CS646993.1 | HV753223.1 | DL045669.1 |
| BD412627.1 | DL114475.1 | DL024028.1 | HC025454.1 | GM637647.1 | DJ061405.1 | CS646224.1 | HV570615.1 | DL032039.1 |
| BD453888.1 | DL109578.1 | DL023996.1 | HC025422.1 | GM659133.1 | DJ055185.1 | CS646192.1 | HV569660.1 | DL032007.1 |
| BD453856.1 | DL109546.1 | DL016418.1 | HC010657.1 | GM644789.1 | DJ054380.1 | DD461896.1 | HV567022.1 | DL031975.1 |
| BD453824.1 | DL109514.1 | DL016386.1 | HC010408.1 | GM644757.1 | CS283954.1 | DD458777.1 | HV573933.1 | DL028134.1 |
| BD453792.1 | DL109482.1 | DL016354.1 | HC010669.1 | GM644725.1 | CS283849.1 | DD453897.1 | HV572457.1 | DL028102.1 |
| BD433656.1 | DL104876.1 | DL016322.1 | DM370676.1 | GM637599.1 | CS283076.1 | DD456717.1 | HV572318.1 | DL028070.1 |
| BD453768.1 | DL104844.1 | DL016290.1 | DM370644.1 | GM637567.1 | CS276858.1 | CS631717.1 | HV560175.1 | DL028038.1 |
| BD453736.1 | DL104812.1 | DL016258.1 | DM370612.1 | GM637535.1 | CS275478.1 | CS631283.1 | HV558762.1 | DL028006.1 |
| BD453704.1 | DL095889.1 | DL016226.1 | DM370580.1 | GM637503.1 | CS266080.1 | CS631204.1 | HV565518.1 | DL027974.1 |
| BD453672.1 | DL095857.1 | DL011774.1 | DM370548.1 | GM637471.1 | CS265607.1 | CS643944.1 | HH979610.1 | DL036420.1 |
| BD453640.1 | DL095825.1 | DL011742.1 | DM370516.1 | GM633063.1 | CS249789.1 | CS627839.1 | HH979568.1 | DL044207.1 |
| BD453608.1 | DL095793.1 | DL011710.1 | DM370484.1 | GM633031.1 | CS244151.1 | CS627198.1 | FW394280.1 | DL044175.1 |
| BD453558.1 | DL094138.1 | DL011678.1 | DM209284.1 | GM632999.1 | CS244231.1 | CS632203.1 | FW394265.1 | DL048248.1 |
| BD453526.1 | DL094106.1 | DL011646.1 | HC003064.1 | GM632967.1 | CS244199.1 | CS623704.1 | FW394261.1 | DL048184.1 |

| | | | | | | | | |
|---|---|---|---|---|---|---|---|---|
| BD453494.1 | DL094074.1 | DL011614.1 | HB865034.1 | GM632935.1 | CS243761.1 | CS623650.1 | FW394219.1 | DL048152.1 |
| BD453462.1 | DL094042.1 | DL048629.1 | HB864970.1 | GM632903.1 | CS243152.1 | DD437994.1 | FW394187.1 | DL048088.1 |
| BD453430.1 | DL094010.1 | DL048597.1 | HB864994.1 | GM625292.1 | CS230950.1 | BD243823.1 | FW393134.1 | DL048025.1 |
| BD453398.1 | DL093978.1 | DL048565.1 | HB864938.1 | GM625260.1 | CS228092.1 | BD243471.1 | FW396804.1 | DL047993.1 |
| BD453253.1 | DL090267.1 | DL048533.1 | HB864906.1 | GM625228.1 | CS228034.1 | BD243435.1 | FW396269.1 | DL047961.1 |
| BD453221.1 | DL090235.1 | DL048501.1 | HB864874.1 | GM625196.1 | CS210666.1 | BD242673.1 | HC056023.1 | DL047929.1 |
| BD453151.1 | DL090203.1 | DL048469.1 | DM021869.1 | GM625164.1 | CS204053.1 | BD242511.1 | DM381877.1 | DL047897.1 |
| BD432174.1 | DL090171.1 | DL044852.1 | DM016017.1 | GM625132.1 | CS208080.1 | BD242453.1 | HC052002.1 | DL047865.1 |
| BD399359.1 | DL086166.1 | DL044820.1 | DM026455.1 | GM624042.1 | CS207906.1 | BD241785.1 | HB475680.1 | DL043883.1 |
| BD451756.1 | DL086134.1 | DL044788.1 | GN042231.1 | GM624010.1 | CS189613.1 | BD238558.1 | HB474890.1 | DL040066.1 |
| BD451714.1 | DL086102.1 | DL044756.1 | GN030759.1 | GM623978.1 | CS193200.1 | BD237285.1 | HB464859.1 | DL040034.1 |
| BD440407.1 | DL086070.1 | DL044724.1 | GN030695.1 | GM623946.1 | CS175714.1 | BD235957.1 | HB463822.1 | DL040002.1 |
| BD450264.1 | DL086038.1 | DL044692.1 | GN030663.1 | DL014005.1 | CS172460.1 | BD235632.1 | DM155735.1 | DL039970.1 |
| BD429589.1 | DL086006.1 | DL044604.1 | GN030631.1 | DL013973.1 | CS172312.1 | BD235364.1 | HB463479.1 | DL039938.1 |
| BD429479.1 | DL114440.1 | DL044572.1 | GN030599.1 | DL013941.1 | CS162776.1 | BD232003.1 | HB447885.1 | DL039906.1 |
| BD407585.1 | DL114408.1 | DL044540.1 | GN030567.1 | DL013909.1 | CS159788.1 | BD231841.1 | DM139905.1 | DL036282.1 |
| BD439124.1 | DL114376.1 | DL044508.1 | GN030535.1 | DL013877.1 | BD294892.1 | BD231161.1 | GN343723.1 | DL047853.1 |
| AX708830.1 | DL114344.1 | DL044476.1 | GN030503.1 | DL030041.1 | BD283885.1 | BD231123.1 | GN349489.1 | DL047821.1 |
| AX708725.1 | DL109447.1 | DL040460.1 | GN030375.1 | DL030009.1 | BD276192.1 | BD228709.1 | GN340543.1 | DL047789.1 |
| AX703500.1 | DL109415.1 | DL040428.1 | GN030279.1 | DL029977.1 | BD274297.1 | BD227155.1 | GN335375.1 | DL047757.1 |
| AX701745.1 | DL109383.1 | DL040396.1 | GN030247.1 | DL046634.1 | BD272758.1 | BD226750.1 | GN112659.1 | DL047725.1 |
| AX700473.1 | DL109351.1 | DL040364.1 | GN029863.1 | DL046602.1 | BD271967.1 | BD225779.1 | GN113554.1 | DL047693.1 |
| AX657128.1 | DL109319.1 | DL040332.1 | GN013581.1 | DL046570.1 | BD271158.1 | BD225747.1 | DM064147.1 | DL047661.1 |
| AX657096.1 | DL109287.1 | DL040300.1 | GN010351.1 | DL046538.1 | DD227718.1 | BD225715.1 | GM997567.1 | DL043679.1 |
| BD176056.1 | DL104553.1 | DL036676.1 | GN033563.1 | DL042652.1 | DD227404.1 | BD225683.1 | GM996598.1 | DL039702.1 |
| BD175044.1 | DL104521.1 | DL036644.1 | GN033531.1 | DL042620.1 | DD224450.1 | BD225651.1 | GN009692.1 | DL036046.1 |
| AX642253.1 | DL100043.1 | DL036612.1 | GN033499.1 | DL042588.1 | DD224141.1 | BD225619.1 | GM992499.1 | DL035918.1 |
| AX058588.1 | DL093937.1 | DL036580.1 | GN033467.1 | DL038664.1 | DD233178.1 | BD224823.1 | GM992051.1 | DL031914.1 |
| AX045145.1 | DL093905.1 | DL036548.1 | GN033371.1 | DL038632.1 | DD231428.1 | BD224007.1 | GM986847.1 | DL031882.1 |
| AX037311.1 | DL093873.1 | DL036516.1 | GN033339.1 | DL029906.1 | DD231396.1 | BD223245.1 | GN000744.1 | DL031818.1 |
| AX036001.1 | DL090098.1 | DL032313.1 | GN033307.1 | DL029874.1 | CQ757636.1 | BD222155.1 | GM999539.1 | DL031754.1 |
| AX028788.1 | DL090066.1 | DL032281.1 | GN033275.1 | DL029842.1 | A08065.1 | DD288455.1 | FB986075.1 | DL027849.1 |
| AX025092.1 | DL090034.1 | DL027286.1 | GN033211.1 | DL029778.1 | DD221619.1 | DD278893.1 | GM970616.1 | DL027817.1 |
| AX023635.1 | DL090002.1 | DL027254.1 | GN033179.1 | DL029746.1 | DD214006.1 | CS355326.1 | GM970194.1 | DL027785.1 |
| AX023601.1 | DL089970.1 | DL027222.1 | GN033083.1 | DL026355.1 | DD213989.1 | GM619040.1 | DL464530.1 | DL027753.1 |
| AX019311.1 | DL123353.1 | DL027190.1 | GN032987.1 | DL018283.1 | DD218616.1 | GM716046.1 | DL470990.1 | DL023877.1 |
| AX018712.1 | DL123321.1 | DL027158.1 | GN032955.1 | DL018251.1 | DD213573.1 | GM662142.1 | DL462988.1 | DL023845.1 |
| AX018248.1 | DL123289.1 | DL023346.1 | GN032923.1 | DL018219.1 | DD213541.1 | GM640908.1 | GM831910.1 | DL023813.1 |
| AX010924.1 | DL098883.1 | DL023314.1 | GN032891.1 | DL013785.1 | DD215943.1 | GM640876.1 | GM692565.1 | CS466055.1 |
| AX010414.1 | DL098851.1 | GM739527.1 | GN032859.1 | DL013753.1 | DD222022.1 | GM640844.1 | GM711805.1 | CS464782.1 |
| AX004545.1 | DL098819.1 | GM698716.1 | GN032795.1 | DL013721.1 | CQ840717.1 | GM628490.1 | CS368478.1 | DD391913.1 |
| AX003015.1 | DL098787.1 | GM656623.1 | GN032700.1 | DL013689.1 | CQ830503.1 | GM047136.1 | CS368286.1 | DD382832.1 |
| AF072575.1 | DL098755.1 | GM656591.1 | GN032668.1 | DL000626.1 | CQ829216.1 | GM603683.1 | CS368158.1 | DD400156.1 |
| A41844.1 | DL092745.1 | GM656559.1 | GN032635.1 | DJ493665.1 | CQ821035.1 | GM603492.1 | CS368030.1 | CS463680.1 |
| A35724.1 | DL092681.1 | GM649566.1 | GN032603.1 | DL008503.1 | CQ816998.1 | GM601142.1 | CS367902.1 | CS462157.1 |
| A35298.1 | DL092649.1 | GM649534.1 | GN032571.1 | DJ491816.1 | CQ816955.1 | FB677309.1 | CS367710.1 | CS459027.1 |
| A33451.1 | DL092617.1 | GM649502.1 | GN032540.1 | DJ491590.1 | CQ816918.1 | FB676839.1 | DL079980.1 | DD361287.1 |

| | | | | | | | | |
|---|---|---|---|---|---|---|---|---|
| A30354.1 | DL092585.1 | GM649470.1 | GN032508.1 | DJ446857.1 | CQ814470.1 | GM061231.1 | DJ327059.1 | DD367615.1 |
| A26144.1 | DL088970.1 | GM649438.1 | GN032476.1 | DJ446824.1 | CQ814048.1 | GM061132.1 | DJ327027.1 | DD367319.1 |
| A06250.1 | DL088938.1 | GM649406.1 | GN032444.1 | DJ446683.1 | CQ814016.1 | DL260535.1 | DJ326995.1 | DD368153.1 |
| A31179.1 | DL088906.1 | GM670046.1 | GN032412.1 | DJ438153.1 | CQ813984.1 | DL241174.1 | DJ082705.1 | DD361321.1 |
| A04448.1 | DL103281.1 | GM656541.1 | AX144975.1 | CS482967.1 | CQ813952.1 | DL240795.1 | DJ082651.1 | CS457837.1 |
| A25385.1 | DL103249.1 | GM656509.1 | AX144943.1 | CS482932.1 | CQ813920.1 | FB741976.1 | DJ080157.1 | CS457194.1 |
| A19975.1 | DL103217.1 | GM656477.1 | AX144910.1 | CS482892.1 | CQ813888.1 | FB748873.1 | DJ079787.1 | CS450598.1 |
| A15711.1 | DL107969.1 | GM656445.1 | AX144878.1 | CS484791.1 | CQ813856.1 | FB674301.1 | DJ066392.1 | DD359179.1 |
| A12678.1 | DL107937.1 | GM656413.1 | AX144846.1 | DD401437.1 | CQ813824.1 | DL233405.1 | DJ066360.1 | DD357920.1 |
| A11140.1 | DL107905.1 | GM656381.1 | AX144814.1 | DD401405.1 | CQ813792.1 | DL231663.1 | DJ066328.1 | DL049634.1 |
| A07765.1 | DL092447.1 | GM656349.1 | AX144750.1 | DD405188.1 | CQ812762.1 | FB713982.1 | DJ066294.1 | DL049602.1 |
| A00329.1 | DL092415.1 | GM649302.1 | AX144718.1 | DD405156.1 | CQ801298.1 | FB713854.1 | DJ066260.1 | DL041555.1 |
| HW155498.1 | DL092383.1 | GM649316.1 | AX144686.1 | DD405124.1 | CQ800904.1 | DL110165.1 | DJ065242.1 | DL041523.1 |
| HW155432.1 | DL088768.1 | GM649260.1 | AX144654.1 | DD405092.1 | CQ800659.1 | DL110133.1 | DJ055295.1 | DL033305.1 |
| HW155298.1 | DL088736.1 | GM649228.1 | AX144622.1 | DD405060.1 | CQ795473.1 | DL110101.1 | DJ054311.1 | DL033241.1 |
| HW154895.1 | DL088704.1 | GM649196.1 | AX144439.1 | DD410094.1 | CQ793556.1 | DL105168.1 | CS810646.1 | DL033209.1 |
| HW154659.1 | DL088672.1 | GM642587.1 | AX144375.1 | DD402371.1 | CQ788625.1 | DL105136.1 | CS810547.1 | DL033177.1 |
| HW154492.1 | DL088640.1 | GM642555.1 | AX144247.1 | DD402339.1 | CQ787472.1 | DL105104.1 | DD468098.1 | DL029137.1 |
| HW158026.1 | DL088608.1 | GM642523.1 | AX144181.1 | DD402307.1 | CQ784699.1 | DL119984.1 | DJ017899.1 | DL029105.1 |
| HW154091.1 | DL112954.1 | GM642515.1 | AX143923.1 | DD402275.1 | CQ784635.1 | DL119952.1 | DJ019844.1 | DL029073.1 |
| HW153893.1 | DL092358.1 | GM642458.1 | HV694823.1 | DD402243.1 | CQ778522.1 | DL119920.1 | DD080703.1 | DL021759.1 |
| HW145338.1 | DL098503.1 | GM642426.1 | HV694791.1 | DD402211.1 | CQ778400.1 | DL119856.1 | DD107479.1 | DL021727.1 |
| HW152023.1 | DL098471.1 | GM635244.1 | HV693717.1 | DD402179.1 | CQ774811.1 | DL119824.1 | DD092356.1 | DL021695.1 |
| HW144711.1 | DL092333.1 | GM635212.1 | HV693313.1 | DD402147.1 | AX255414.1 | DL115133.1 | DD090691.1 | DL021663.1 |
| HW126585.1 | DL092301.1 | GM635180.1 | HV699123.1 | DD402083.1 | AX253431.1 | DL110069.1 | DD090320.1 | DL021631.1 |
| HW125560.1 | DL092269.1 | GM635148.1 | HV698193.1 | DD402051.1 | AX250538.1 | DL110005.1 | DD102585.1 | DL017361.1 |
| HW125383.1 | DL092173.1 | GM635116.1 | HV697826.1 | DD405771.1 | DL047560.1 | DL109973.1 | DD052036.1 | DL017329.1 |
| HW144437.1 | DL088558.1 | HW123469.1 | HV689282.1 | DD405739.1 | DL047528.1 | DL109727.1 | DD057919.1 | DL012940.1 |
| HW144359.1 | DL088526.1 | HW122726.1 | HV695570.1 | DD405707.1 | DL031392.1 | DL109695.1 | DD057887.1 | DL012876.1 |
| HW144327.1 | DL088494.1 | HW122160.1 | HV688884.1 | DD405675.1 | DL020340.1 | DL104961.1 | DD057855.1 | DL041348.1 |
| HV931478.1 | DL088462.1 | HW120067.1 | HV585138.1 | DD405643.1 | DL020308.1 | DL104929.1 | DD055422.1 | DL041284.1 |
| HV925511.1 | GM630428.1 | HW118289.1 | HV585106.1 | DD405591.1 | DL070415.1 | DL100483.1 | DD053315.1 | DL037397.1 |
| HV929980.1 | GM630396.1 | HW117910.1 | HV592504.1 | DD405559.1 | DJ327065.1 | DL100387.1 | DD055117.1 | DL037365.1 |
| HV819038.1 | GM630332.1 | HW115073.1 | HV592435.1 | DD405495.1 | DJ327033.1 | DL100355.1 | DD038496.1 | DL037333.1 |
| HV802927.1 | GM630300.1 | HW081524.1 | HV594373.1 | DD402025.1 | DJ327001.1 | DL124136.1 | DD026980.1 | DL022471.1 |
| HV802895.1 | GM621525.1 | HW087594.1 | HV586033.1 | DD401993.1 | DJ326903.1 | DL124104.1 | DD029745.1 | DL014511.1 |
| HV778938.1 | GM699206.1 | HW087216.1 | HV600960.1 | DD401961.1 | DJ082657.1 | DL119786.1 | DD159573.1 | DL014479.1 |
| HV777978.1 | GM698709.1 | HW105244.1 | HB847059.1 | DD401900.1 | DJ071628.1 | DL119754.1 | DD159201.1 | DL014447.1 |
| HV566130.1 | GM656619.1 | HW072783.1 | HB838746.1 | DD401868.1 | DJ066762.1 | DL142371.1 | DD182191.1 | DL009861.1 |
| HV572469.1 | GM656587.1 | HW072690.1 | HB838160.1 | DD401836.1 | DJ066366.1 | DL119484.1 | CS204332.1 | DL009829.1 |
| HV572322.1 | GM656555.1 | HW105048.1 | HB846183.1 | DD401804.1 | DJ066334.1 | DL119452.1 | CS157807.1 | DL009797.1 |
| HV560181.1 | GM649562.1 | HW104473.1 | HB837839.1 | DD409186.1 | DJ066300.1 | DL119420.1 | CS157775.1 | DL009765.1 |
| HV550141.1 | GM649466.1 | HW071972.1 | HB845786.1 | DD401628.1 | DJ066266.1 | DL123667.1 | CS157936.1 | DL009541.1 |
| HV554541.1 | GM649434.1 | HW104456.1 | HB845504.1 | DD401596.1 | DJ066212.1 | DL123635.1 | CS119751.1 | DL009509.1 |
| AY659388.1 | GM649402.1 | HW104424.1 | HB845277.1 | DD401564.1 | DJ065248.1 | DL123603.1 | CS119488.1 | DL009477.1 |
| AY659356.1 | GM670042.1 | HW104344.1 | HB845057.1 | DD401532.1 | DJ055699.1 | DL123571.1 | CS119455.1 | DL009445.1 |
| AY659324.1 | GM656537.1 | HW104311.1 | HB844636.1 | DD401500.1 | DJ061664.1 | DL119381.1 | CS119420.1 | DL009413.1 |

| | | | | | | | | |
|---|---|---|---|---|---|---|---|---|
| AY659292.1 | GM656505.1 | HW104278.1 | HB844344.1 | DD401468.1 | DJ061616.1 | DL049673.1 | CS119355.1 | DL034326.1 |
| AY659260.1 | GM656473.1 | HW071085.1 | HB836740.1 | CS470297.1 | DJ061568.1 | DL045665.1 | DL016000.1 | DL009410.1 |
| AY659228.1 | GM656441.1 | HW104236.1 | HB843972.1 | CS467566.1 | DJ061472.1 | DL041690.1 | DL015968.1 | DL009378.1 |
| AY659196.1 | GM649306.1 | HW104196.1 | HB843529.1 | BD299642.1 | DJ055611.1 | DL049632.1 | DL015904.1 | DL009346.1 |
| AY659164.1 | GM649312.1 | HW104156.1 | HB835916.1 | BD298095.1 | DJ055301.1 | DL049600.1 | DL015872.1 | DL009314.1 |
| AY659132.1 | GM649256.1 | HW099980.1 | HB843220.1 | BD297646.1 | DJ055181.1 | DL045658.1 | DL015840.1 | DL009282.1 |
| AY659100.1 | GM649224.1 | HV753568.1 | HB842931.1 | BD296980.1 | DJ054362.1 | DL033303.1 | DL011254.1 | DL009250.1 |
| AY659068.1 | FB677411.1 | HV753486.1 | HB835271.1 | BD296936.1 | FB291802.1 | DL033239.1 | DL011222.1 | DL009218.1 |
| AY659036.1 | GM061149.1 | HV743435.1 | HB842806.1 | BD296882.1 | CS810620.1 | DL033207.1 | DL047646.1 | DL018708.1 |
| AY659004.1 | DL460024.1 | HV743311.1 | HB842181.1 | BD296232.1 | FB292588.1 | DL033175.1 | DL047614.1 | DL018676.1 |
| AY658972.1 | DL459982.1 | HV743273.1 | HB841879.1 | BD295214.1 | FB292329.1 | DL033143.1 | DL047582.1 | DL018644.1 |
| AY658940.1 | DL262719.1 | HV743234.1 | HB841608.1 | BD280120.1 | CS803351.1 | DL029135.1 | DL047550.1 | DL018612.1 |
| AY658908.1 | DL260586.1 | HV704948.1 | HB840489.1 | BD289750.1 | CS802276.1 | DL029103.1 | DL047518.1 | DD458977.1 |
| AY658876.1 | DL260063.1 | HV704451.1 | HB645726.1 | BD287712.1 | CS389255.1 | DL029071.1 | DL047486.1 | DD453755.1 |
| AY658844.1 | DL257209.1 | HV704419.1 | HB645694.1 | BD287340.1 | CS389207.1 | DL021757.1 | DL039655.1 | DD456718.1 |
| AY658812.1 | DL241776.1 | HV704264.1 | HB645650.1 | BD274143.1 | CS389170.1 | DL021725.1 | DL039623.1 | CS631718.1 |
| AY658780.1 | DL241345.1 | HV704199.1 | HB855454.1 | BD273292.1 | CS406475.1 | DL021693.1 | DL039591.1 | CS631285.1 |
| AY658748.1 | DL241047.1 | HV744595.1 | DM187948.1 | BD273167.1 | CS408137.1 | DL021661.1 | DL039559.1 | CS631206.1 |
| AY658716.1 | DL240957.1 | HV703524.1 | DM173881.1 | HV187247.1 | CS402146.1 | DL021629.1 | DL039527.1 | CS642168.1 |
| AY658684.1 | DL240638.1 | HV703088.1 | HB559422.1 | HV192777.1 | CS383385.1 | DL017391.1 | DL039495.1 | CS640891.1 |
| AY658652.1 | FB674309.1 | HV702357.1 | HB491545.1 | HV218234.1 | CS382581.1 | DL017359.1 | DL047321.1 | CS627841.1 |
| AY658620.1 | DL238832.1 | HV702262.1 | HB488916.1 | HV221669.1 | CS376394.1 | DL017327.1 | DL047289.1 | CS627317.1 |
| AY658588.1 | DL233414.1 | HV702025.1 | DM165788.1 | HV234155.1 | CS362761.1 | DL012938.1 | DL043467.1 | CS632678.1 |
| A13050.1 | DL232127.1 | HV701276.1 | GM624750.1 | HV301799.1 | CS359726.1 | DL012874.1 | DL043403.1 | CS632204.1 |
| A10376.1 | DL213310.1 | HV701218.1 | GM623182.1 | HV302943.1 | BD269159.1 | DL041314.1 | DL043371.1 | CS624427.1 |
| A06767.1 | DL206820.1 | HV701186.1 | GM741852.1 | HI931685.1 | BD269127.1 | DL041282.1 | DL043339.1 | CS623705.1 |
| A08449.1 | DL212466.1 | HV701154.1 | GM741820.1 | HI930642.1 | BD265640.1 | DJ389469.1 | DL043275.1 | CS623651.1 |
| A08083.1 | DL227049.1 | HV701122.1 | GM618983.1 | HI929440.1 | BD265597.1 | DJ388683.1 | DL039458.1 | CS626367.1 |
| A06621.1 | DL206295.1 | HV708775.1 | GM047077.1 | HI935102.1 | BD263799.1 | CS726875.1 | DL039426.1 | DD437995.1 |
| A05161.1 | FB714978.1 | HV708346.1 | GM654808.1 | HI935026.1 | BD263524.1 | CS724451.1 | DL039394.1 | CS616539.1 |
| A08331.1 | FB714485.1 | HV701071.1 | CS695985.1 | HI918315.1 | BD263437.1 | CS724406.1 | DL039362.1 | CS614442.1 |
| A01410.1 | FB714402.1 | HV701039.1 | CS695953.1 | HI918265.1 | BD263394.1 | CS723812.1 | DL039330.1 | CS611833.1 |
| A01099.1 | FB713958.1 | HV694849.1 | CS695921.1 | HI923634.1 | BD263047.1 | DJ353082.1 | DL039298.1 | CS604541.1 |
| M29831.1 | FB713798.1 | HV700735.1 | CS695889.1 | HI657188.1 | BD250958.1 | DJ359621.1 | DL035674.1 | CS604509.1 |
| M12054.1 | FB713766.1 | HV694715.1 | CS695793.1 | FW420983.1 | BD249440.1 | DJ357676.1 | DL035642.1 | CS604477.1 |
| M22127.1 | FB708016.1 | HV694683.1 | CS695697.1 | FW503017.1 | BD248732.1 | DJ357612.1 | DL035610.1 | CS604445.1 |
| M12442.1 | FB672290.1 | HV515491.1 | CS695665.1 | FW496471.1 | BD248116.1 | DJ344661.1 | DL035578.1 | CS604413.1 |
| M24331.1 | FB667105.1 | HV515459.1 | DL176468.1 | FW503818.1 | BD247029.1 | DJ361202.1 | DL035546.1 | CS604381.1 |
| M23996.1 | FB666794.1 | HV515427.1 | DL176388.1 | FW499028.1 | BD246954.1 | DJ360414.1 | DL035514.1 | CS604349.1 |
| M35459.1 | DL115173.1 | HV515395.1 | DL176319.1 | FW420743.1 | BD244512.1 | DJ339849.1 | DL031510.1 | CS604317.1 |
| MK967457.1 | DL115141.1 | HV515363.1 | DL175297.1 | FW500541.1 | BD243691.1 | DL009214.1 | DL031478.1 | CS604221.1 |
| AF069383.1 | DL115109.1 | HV515331.1 | DL174427.1 | FW420471.1 | BD243460.1 | DL018704.1 | DL031446.1 | CS604189.1 |
| AH002298.2 | DL115077.1 | HV515299.1 | FB360096.1 | FW420439.1 | BD243424.1 | DL018672.1 | DL031414.1 | CS604029.1 |
| M10323.1 | DL115045.1 | HV515267.1 | DL097993.1 | FW420407.1 | BD243010.1 | DL018640.1 | DL031382.1 | CS603997.1 |
| DQ250255.1 | DL110045.1 | HV515235.1 | DL045188.1 | FW496924.1 | BD242442.1 | DL018608.1 | DL031350.1 | CS603869.1 |
| AY774231.1 | DL110013.1 | HW380820.1 | DL032602.1 | FW420382.1 | BD238535.1 | DL009156.1 | DL027445.1 | CS603837.1 |
| AY145833.1 | DL109981.1 | HW376099.1 | DJ054749.1 | FW420350.1 | BD238168.1 | DL014180.1 | DL027413.1 | CS603805.1 |

| | | | | | | | | |
|---|---|---|---|---|---|---|---|---|
| HW399755.1 | DL109767.1 | HW366476.1 | DJ054339.1 | FW420318.1 | BD236957.1 | DL014148.1 | DL027381.1 | CS603773.1 |
| HW399278.1 | DL109703.1 | HW375250.1 | FB291797.1 | FW496696.1 | BD235725.1 | DL014116.1 | DL023569.1 | CS603741.1 |
| HW381565.1 | DL104969.1 | HW374807.1 | CS810617.1 | FW496681.1 | BD235408.1 | DL014084.1 | DL023537.1 | CS603485.1 |
| HW390542.1 | DL104937.1 | HW374493.1 | FB292585.1 | FW503293.1 | CS018513.1 | DL042644.1 | DL023505.1 | DD251658.1 |
| HW117986.1 | DL104905.1 | HW381352.1 | DJ020594.1 | FW368812.1 | CS018388.1 | DL042612.1 | DL023473.1 | DD248691.1 |
| HW101665.1 | DL100395.1 | HW353720.1 | CS802219.1 | FW368674.1 | CS016566.1 | DL042580.1 | DL023441.1 | DD257953.1 |
| HW072877.1 | DL100363.1 | HW353500.1 | DJ052233.1 | FW368458.1 | CS008500.1 | DL034127.1 | DL010794.1 | DD253797.1 |
| HW072854.1 | DL100331.1 | HW353468.1 | DJ049393.1 | FW368143.1 | CQ990292.1 | DL029834.1 | DL010762.1 | CS288537.1 |
| HW105056.1 | DL124144.1 | HW341643.1 | CS793703.1 | FW363305.1 | CQ986516.1 | DL029738.1 | DL010730.1 | CS287620.1 |
| HW104625.1 | DL124112.1 | HW338486.1 | CS716737.1 | FW362626.1 | CQ981104.1 | DL018307.1 | DL010698.1 | CS287403.1 |
| HW071648.1 | DL119794.1 | HW338358.1 | AY657238.1 | FW366590.1 | CQ975014.1 | DL018275.1 | DL010666.1 | DD235037.1 |
| HW071103.1 | DL119762.1 | HW338102.1 | AY657206.1 | FW366206.1 | CQ973427.1 | DL018243.1 | DL010634.1 | DD247144.1 |
| HW070702.1 | DL119730.1 | HW337974.1 | AY657174.1 | FW351239.1 | CQ972381.1 | DL018211.1 | DL010602.1 | E41518.1 |
| HW103936.1 | DL119698.1 | HW337846.1 | AY657142.1 | FW351176.1 | CQ972349.1 | CS497132.1 | DL047026.1 | E43955.1 |
| HW099568.1 | DL119666.1 | HW337718.1 | AY657110.1 | FW351016.1 | CQ972317.1 | DD418605.1 | DL046994.1 | E49203.1 |
| HW099514.1 | DL119634.1 | HW337334.1 | AY657078.1 | FW360645.1 | CQ971098.1 | DD417819.1 | DL046962.1 | DL023662.1 |
| HW068953.1 | DL114911.1 | HW337206.1 | AY657046.1 | HD121591.1 | CQ970242.1 | DD410171.1 | DL046930.1 | DL023630.1 |
| HW067601.1 | DL114879.1 | HW337078.1 | K02994.1 | HD114098.1 | CQ967261.1 | CS492974.1 | DL046898.1 | DL023598.1 |
| HW066803.1 | DL114847.1 | HW336950.1 | M34295.1 | HD082428.1 | CQ956050.1 | CS490587.1 | DL046866.1 | DL016020.1 |
| HW083275.1 | DL127347.1 | HW321649.1 | K01478.1 | HD082298.1 | CQ947242.1 | CS489137.1 | DL043044.1 | DL015956.1 |
| HW083243.1 | DL119588.1 | HW321191.1 | M20874.1 | HD088248.1 | CQ947140.1 | CS486440.1 | DL043012.1 | DL015924.1 |
| HW083211.1 | DL119556.1 | HW336458.1 | J02536.1 | HD077383.1 | CQ947021.1 | CS482921.1 | DL042980.1 | DL015892.1 |
| HW081888.1 | DL119524.1 | HW329371.1 | M34501.1 | FW344979.1 | CQ944178.1 | CS482877.1 | DL042948.1 | DL015860.1 |
| HV743099.1 | DL119492.1 | HW336342.1 | HV549876.1 | FW344732.1 | CQ944146.1 | DD401429.1 | DL042916.1 | DL011274.1 |
| HV704841.1 | DL119460.1 | HW328820.1 | HV549696.1 | FW344186.1 | CQ944114.1 | DD401397.1 | DL042884.1 | DL011242.1 |
| HV704144.1 | DL119428.1 | HW328688.1 | HV549664.1 | FW343962.1 | CQ944082.1 | DD405212.1 | DL039067.1 | DL011210.1 |
| HV703779.1 | DL123739.1 | HW328603.1 | HV549600.1 | FW343610.1 | CQ944050.1 | DD405180.1 | DL039035.1 | DL047602.1 |
| HV703150.1 | DL123707.1 | HW328357.1 | HV549553.1 | FW343546.1 | CQ944018.1 | DD405148.1 | DL039003.1 | DL047570.1 |
| HV701288.1 | DL123675.1 | HW328285.1 | HV549329.1 | FW343421.1 | CQ943986.1 | DD405116.1 | DL038971.1 | DL047538.1 |
| HV701162.1 | DL123643.1 | HW328093.1 | HV549257.1 | HC452155.1 | CQ943954.1 | DD406769.1 | DL038939.1 | DL039675.1 |
| HV701130.1 | DL123611.1 | HW318501.1 | HV549193.1 | HC451881.1 | CQ943922.1 | DD402427.1 | DL038907.1 | DL039643.1 |
| HV708198.1 | DL123579.1 | HW313986.1 | HV549091.1 | HC450069.1 | CQ943890.1 | DD402395.1 | DL035123.1 | DL039611.1 |
| HV701079.1 | DL119389.1 | HW313617.1 | HV549015.1 | HC449524.1 | CQ943858.1 | DD402363.1 | DL031119.1 | DJ066412.1 |
| HV701047.1 | DL119357.1 | HW311252.1 | HV544361.1 | FU761014.1 | CQ898811.1 | DD402331.1 | DL031087.1 | DJ066376.1 |
| HV695541.1 | DL119325.1 | HW309710.1 | HV544170.1 | FU757636.1 | CQ898664.1 | DD402299.1 | DL031055.1 | DJ066344.1 |
| HV694858.1 | DL119293.1 | HW309424.1 | HV547850.1 | FU757604.1 | CQ898632.1 | DD402267.1 | DL031023.1 | DJ066311.1 |
| HV694822.1 | DL114833.1 | HW307872.1 | HV543739.1 | FU756842.1 | CQ898600.1 | DD402235.1 | DL030991.1 | DJ066277.1 |
| HV693716.1 | GN031821.1 | HW307840.1 | HV547280.1 | HC358222.1 | CQ898568.1 | DD402203.1 | DL027150.1 | DJ066243.1 |
| HV693312.1 | GN031723.1 | HW307808.1 | HV547415.1 | HC358092.1 | CQ898266.1 | DD405615.1 | DL027118.1 | DJ068738.1 |
| HV698292.1 | GN031691.1 | HW307776.1 | HV543506.1 | HC358059.1 | A30919.1 | DD405583.1 | DL027086.1 | DJ061583.1 |
| HV697825.1 | GN031659.1 | HW316588.1 | HV547230.1 | HC357537.1 | A29002.1 | DD405551.1 | DL027054.1 | DJ061487.1 |
| HV689281.1 | GN031627.1 | HW307738.1 | HV539717.1 | HC434780.1 | A27227.1 | DD405519.1 | DL027022.1 | DJ055196.1 |
| HV688880.1 | GN031531.1 | HW307706.1 | HV539535.1 | HC434748.1 | A25124.1 | DD405487.1 | DL026990.1 | DJ057591.1 |
| HV585546.1 | GN031499.1 | HW315696.1 | HV538617.1 | HC325456.1 | A28243.1 | DD401985.1 | DL026958.1 | DJ060401.1 |
| FW590743.1 | GN031467.1 | HW315626.1 | HV538461.1 | A07174.1 | A26536.1 | DD401924.1 | DL030917.1 | DJ060190.1 |
| FW577605.1 | GN031434.1 | HW315566.1 | HV221080.1 | HC324882.1 | A26052.1 | DD401892.1 | DL030878.1 | DJ053397.1 |
| FW577147.1 | GN031402.1 | HW315532.1 | HV040143.1 | HC324500.1 | A23319.1 | DD401860.1 | DL030846.1 | DJ056342.1 |

| | | | | | | | | |
|---|---|---|---|---|---|---|---|---|
| FW575896.1 | GN031370.1 | HW315173.1 | HV114417.1 | HC315439.1 | A23259.1 | DD401828.1 | DL030814.1 | DJ053106.1 |
| FW571121.1 | GN031338.1 | HW315141.1 | HV191915.1 | HC313695.1 | A22420.1 | DD401796.1 | DL030750.1 | DJ053074.1 |
| FW570988.1 | GN031306.1 | HW315109.1 | HV200766.1 | HC318724.1 | A21002.1 | BD319282.1 | DL022841.1 | CS810630.1 |
| FW568060.1 | GN031273.1 | HW315077.1 | HV203345.1 | HC308498.1 | A21488.1 | BD318904.1 | DL019786.1 | CS812997.1 |
| FW571800.1 | GN031241.1 | HW315018.1 | HV182468.1 | HC307851.1 | A20574.1 | BD318125.1 | DL019730.1 | CS812772.1 |
| HI967449.1 | GN031209.1 | HW314953.1 | HV182436.1 | HC307796.1 | A19915.1 | BD307822.1 | DL019698.1 | DJ021069.1 |
| HI369137.1 | GN031177.1 | HW314844.1 | HV182404.1 | HB474827.1 | A18685.1 | BD306473.1 | DL019666.1 | DJ052637.1 |
| HI369089.1 | GN031144.1 | HW314787.1 | HV182372.1 | HB463658.1 | A16282.1 | BD300351.1 | CS376990.1 | DJ045449.1 |
| HH772340.1 | GN031112.1 | HW314742.1 | HV217028.1 | HB469155.1 | A16130.1 | BD294176.1 | CS376251.1 | DJ050561.1 |
| HI424117.1 | GN031080.1 | HW314622.1 | HV191100.1 | HB469094.1 | A15949.1 | BD293577.1 | CS376215.1 | DJ050208.1 |
| HI424085.1 | GN031048.1 | HW145135.1 | HV246861.1 | HB468034.1 | A15345.1 | BD293182.1 | CS376009.1 | DJ048831.1 |
| HI423827.1 | GN031016.1 | HW153312.1 | HV191097.1 | DM152691.1 | A14315.1 | BD292421.1 | CS362751.1 | CS800052.1 |
| HI423763.1 | GN030984.1 | HW153264.1 | HV039662.1 | DM152549.1 | A13503.1 | BD291413.1 | CS367227.1 | CS720075.1 |
| HI422892.1 | GN030953.1 | HW152006.1 | HV117916.1 | HB455305.1 | A13177.1 | BD291307.1 | CS359929.1 | CS798836.1 |
| HI415322.1 | GN030921.1 | HW151685.1 | HV117803.1 | HB455025.1 | A12465.1 | BD299995.1 | CS359644.1 | CS716767.1 |
| HI414020.1 | GN030889.1 | HW150555.1 | HV117766.1 | HB453856.1 | A10907.1 | BD298659.1 | BD269149.1 | CS807356.1 |
| HI413611.1 | GN030825.1 | HW144749.1 | HV117506.1 | DM137691.1 | A10414.1 | BD298440.1 | BD265621.1 | CS716107.1 |
| HI413575.1 | GN030793.1 | HW144665.1 | HV112732.1 | DM137113.1 | A04665.1 | BD294901.1 | BD265524.1 | DD291440.1 |
| HI503522.1 | DM008362.1 | HW144626.1 | HV116995.1 | GN032365.1 | A08088.1 | BD277615.1 | DD246848.1 | CS376417.1 |
| HI516095.1 | GM648265.1 | HW144503.1 | HV112587.1 | GN032332.1 | A07612.1 | BD279840.1 | E49065.1 | CS364005.1 |
| HI002736.1 | GM648233.1 | HW126550.1 | HV112445.1 | GN032300.1 | A06133.1 | BD274848.1 | E41528.1 | CS362711.1 |
| HI284334.1 | GM648201.1 | HW125525.1 | HV038606.1 | GN032268.1 | A04836.1 | BD273657.1 | E46949.1 | CS359736.1 |
| HI284282.1 | GM641398.1 | HW144379.1 | HV038553.1 | GN032236.1 | A02298.1 | BD273235.1 | E38336.1 | CS359597.1 |
| HI574503.1 | GM641366.1 | HW144347.1 | HV182334.1 | GN032204.1 | A02331.1 | BD273138.1 | E32004.1 | BD269137.1 |
| HI574471.1 | GM641334.1 | HW144315.1 | HV182302.1 | HB864953.1 | A01413.1 | BD271972.1 | E05438.1 | BD268158.1 |
| HI574439.1 | GM641302.1 | HW124956.1 | HV182143.1 | HB864921.1 | A00700.1 | BD271163.1 | E03274.1 | BD268115.1 |
| HI574407.1 | GM641270.1 | HW124819.1 | HV182111.1 | HB864889.1 | A00029.1 | DD231201.1 | E02502.1 | BD266618.1 |
| HI636987.1 | GM641238.1 | HW124658.1 | HV182079.1 | HB864857.1 | K01687.1 | DD230437.1 | E02217.1 | BD265607.1 |
| HI636947.1 | GM629079.1 | HW123468.1 | HV191034.1 | HB864825.1 | M12613.1 | DD227725.1 | E02064.1 | BD263553.1 |
| BD319529.1 | GM629047.1 | HW121450.1 | HV190602.1 | HB864793.1 | M10713.1 | DD227409.1 | E01800.1 | BD263447.1 |
| BD300479.1 | GM629015.1 | HW120100.1 | HV202315.1 | HB864761.1 | M16571.1 | DD226573.1 | E01252.1 | BD251210.1 |
| BD294540.1 | GM628983.1 | HW118288.1 | HV187616.1 | HB883859.1 | M30778.1 | DD224547.1 | E00891.1 | BD248300.1 |
| BD290008.1 | GM628951.1 | HW115072.1 | HV225539.1 | HB866943.1 | M11210.1 | DD224160.1 | E00425.1 | BD247505.1 |
| BD295268.1 | GM628919.1 | HW081523.1 | HV234305.1 | HB866661.1 | MT000723.1 | DD223498.1 | E00326.1 | BD247046.1 |
| BD289850.1 | GM881895.1 | HV985908.1 | HV302192.1 | HB866500.1 | AF411594.1 | DD233556.1 | E00023.1 | BD246969.1 |
| BD287854.1 | GM648168.1 | HV985863.1 | HV306785.1 | HC000353.1 | LT897786.1 | DD231465.1 | DD146384.1 | BD245170.1 |
| BD287414.1 | GM641205.1 | HW029144.1 | HV306753.1 | HB999858.1 | GN044358.1 | CQ757646.1 | DD146288.1 | BD244577.1 |
| BD277317.1 | GM641173.1 | HV984396.1 | HV306721.1 | HB977426.1 | AH002301.2 | CS259583.1 | DD146256.1 | BD243820.1 |
| BD274171.1 | GM641141.1 | HV984234.1 | HV306689.1 | HB976746.1 | AH003162.2 | DD214011.1 | DD146178.1 | BD243434.1 |
| BD273544.1 | GM641109.1 | HV986638.1 | HV312016.1 | DM193329.1 | M17628.1 | DD213994.1 | DD101522.1 | BD242452.1 |
| BD273184.1 | GM641077.1 | HV960534.1 | HV316659.1 | DM195295.1 | DQ250226.1 | DD213901.1 | DD086341.1 | BD241783.1 |
| BD272419.1 | GM641045.1 | HV960148.1 | HV037179.1 | DM194972.1 | AF170445.1 | BD138553.1 | DD069884.1 | BD238557.1 |
| BD271916.1 | GM716095.1 | HV959643.1 | HV037770.1 | DM203201.1 | AF003717.1 | BD137963.1 | DD112585.1 | BD238402.1 |
| DD231074.1 | GM655122.1 | HV963866.1 | HV037715.1 | DM189838.1 | AH003161.2 | BD137424.1 | DD143257.1 | BD235956.1 |
| CS016562.1 | GM654967.1 | HV966110.1 | HV036673.1 | HB848134.1 | HW124855.1 | BD136685.1 | DD098190.1 | BD235772.1 |
| CQ981081.1 | GM640914.1 | HV958130.1 | HV035387.1 | HB847864.1 | HW124776.1 | BD136049.1 | DD142047.1 | BD235577.1 |
| CQ975377.1 | GM640882.1 | HV966017.1 | HV035191.1 | HB839741.1 | HW117989.1 | DL128846.1 | DD132358.1 | BD234843.1 |

| | | | | | | | | |
|---|---|---|---|---|---|---|---|---|
| CQ972377.1 | GM640850.1 | HV965985.1 | HV031552.1 | HB847638.1 | HW081454.1 | DL118106.1 | DD141442.1 | BD234410.1 |
| CQ972313.1 | GM628496.1 | HV965953.1 | FZ435940.1 | HB847313.1 | HW101670.1 | DL118074.1 | DD141291.1 | BD231839.1 |
| CQ947136.1 | GM662109.1 | HV962270.1 | FZ422123.1 | HB827960.1 | HW096672.1 | DL118042.1 | DD158441.1 | BD231160.1 |
| CQ945944.1 | GM654827.1 | HV962119.1 | FZ435656.1 | HB846883.1 | HW072889.1 | DL108370.1 | DD158417.1 | BD228708.1 |
| CQ944174.1 | GM654795.1 | DL015377.1 | FZ429859.1 | HB838538.1 | HW096198.1 | DL099020.1 | DD157782.1 | BD227124.1 |
| CQ944142.1 | GM654763.1 | DL015345.1 | FZ429803.1 | HB846078.1 | HW072072.1 | DL098988.1 | DD152125.1 | BD226844.1 |
| CQ944110.1 | GM647770.1 | DL015313.1 | FZ429735.1 | HB815199.1 | HW062420.1 | DL098956.1 | DD152105.1 | BD226738.1 |
| CQ944078.1 | GM647738.1 | DL015281.1 | FZ429654.1 | HB826896.1 | HW062388.1 | DL117892.1 | DD096754.1 | BD225778.1 |
| CQ944046.1 | GM647706.1 | DL010791.1 | FZ429586.1 | HB837665.1 | HW056336.1 | DL117860.1 | DD081762.1 | BD225746.1 |
| CQ944014.1 | GM640807.1 | DL010759.1 | FZ429434.1 | HB845578.1 | HW056259.1 | DL117828.1 | DD081689.1 | BD225714.1 |
| CQ943982.1 | GM640774.1 | DL010727.1 | FZ429326.1 | HB827349.1 | HW062363.1 | DL092732.1 | DD148365.1 | BD225682.1 |
| CQ943950.1 | GM640742.1 | DL010695.1 | FZ429272.1 | HB837071.1 | HW062331.1 | DL092700.1 | DD147795.1 | BD225650.1 |
| CQ943918.1 | GM652972.1 | DL010663.1 | FZ429216.1 | HB845173.1 | HW043767.1 | DL033236.1 | DD147763.1 | BD225618.1 |
| CQ943886.1 | GM652940.1 | DL010631.1 | FZ429184.1 | HB844882.1 | HW042086.1 | DL033204.1 | DD147731.1 | BD224822.1 |
| CQ943854.1 | GM652908.1 | DL047055.1 | HI414017.1 | HB844420.1 | HW042054.1 | DL029132.1 | DD147699.1 | BD223244.1 |
| E59415.1 | GM652876.1 | DL047023.1 | HI413817.1 | HB844225.1 | HW043552.1 | DL029100.1 | DD147277.1 | BD222122.1 |
| AX354730.1 | GM652844.1 | DL046991.1 | HI413608.1 | HB836523.1 | HW042010.1 | DL021754.1 | DD081444.1 | BD218880.1 |
| AX354090.1 | GM652812.1 | DL046959.1 | HI413572.1 | HB843681.1 | HW041978.1 | DL017388.1 | DD030762.1 | AF397141.1 |
| AX350314.1 | GM652779.1 | DL046927.1 | HI412396.1 | HB835801.1 | HW041946.1 | DL017356.1 | DD030689.1 | CS157791.1 |
| AX347298.1 | GM638822.1 | DL046895.1 | HI412290.1 | HB843105.1 | HW041914.1 | DL012967.1 | DD027436.1 | CS157952.1 |
| AX347264.1 | GM638790.1 | DL046863.1 | HI410850.1 | HB835523.1 | HW049368.1 | DL041375.1 | DD023844.1 | CS157920.1 |
| AX347228.1 | GM638758.1 | DL043041.1 | HI503519.1 | HB834881.1 | HW049337.1 | DL041343.1 | DD023385.1 | DL048210.1 |
| AX347158.1 | GM626115.1 | DL043009.1 | HI516547.1 | HB842483.1 | HW049305.1 | DL041311.1 | DD023250.1 | DL048178.1 |
| AX346278.1 | GM626083.1 | DL042977.1 | HI541345.1 | HB841973.1 | HW049273.1 | DL041279.1 | DD020789.1 | DL047859.1 |
| AX344902.1 | GM626051.1 | DL042945.1 | HI516092.1 | HB841721.1 | HW049241.1 | DL021483.1 | DD010256.1 | DL040060.1 |
| AX339194.1 | GM626019.1 | DL042913.1 | HI541101.1 | HB841305.1 | HW041812.1 | DL021451.1 | DD010164.1 | DL040028.1 |
| AX329470.1 | GM625987.1 | DL042881.1 | HI547702.1 | HB840820.1 | HW041780.1 | DL021419.1 | DD019577.1 | DL039996.1 |
| AX328294.1 | GM625955.1 | DL038968.1 | HI284279.1 | HB648683.1 | HV961475.1 | DL017181.1 | DD009544.1 | DL043673.1 |
| AX328137.1 | GM037683.1 | DL038936.1 | HI567240.1 | HB647051.1 | HV969138.1 | DL017149.1 | BD495451.1 | DL036040.1 |
| AX268994.1 | FB775382.1 | DL035120.1 | HI574500.1 | GM827443.1 | AY658236.1 | DL017117.1 | BD412570.1 | CS716087.1 |
| AX262246.1 | FB774887.1 | DL031084.1 | HI574436.1 | GM772966.1 | AY658204.1 | DL049420.1 | BD434145.1 | CS796322.1 |
| AX259243.1 | FB766228.1 | DL031052.1 | HI574404.1 | GM709010.1 | AY658172.1 | DL049388.1 | BD453906.1 | CS805534.1 |
| AX303191.1 | FB765898.1 | DL031020.1 | HI571470.1 | GM708978.1 | AY658140.1 | DL049356.1 | BD453873.1 | CS675437.1 |
| AX300158.1 | FB764704.1 | DL030988.1 | HI283371.1 | GM655007.1 | AY658108.1 | DL049324.1 | BD453841.1 | CS791410.1 |
| AY044148.1 | FB764667.1 | DL030959.1 | HI582124.1 | GM654975.1 | AY658076.1 | DJ361198.1 | BD453809.1 | CS791952.1 |
| AX279802.1 | FB747037.1 | DL027147.1 | HI582092.1 | GM044432.1 | AY658044.1 | DJ339846.1 | BD453775.1 | DJ031577.1 |
| AX254830.1 | FB762751.1 | DL027115.1 | HI582028.1 | GM755063.1 | AY658012.1 | DJ339814.1 | BD453753.1 | DJ045216.1 |
| AX242308.1 | FB743930.1 | DL027083.1 | HI581965.1 | DL467564.1 | AY657980.1 | DJ339718.1 | BD453721.1 | DJ044923.1 |
| AX242276.1 | FB743898.1 | CS800117.1 | HI581933.1 | GM712948.1 | AY657948.1 | DJ339654.1 | BD453689.1 | DJ044882.1 |
| AX242212.1 | FB743866.1 | CS720121.1 | HI581901.1 | GM652750.1 | AY657916.1 | DJ339621.1 | BD453657.1 | DJ029510.1 |
| AX242148.1 | FB743834.1 | CS799871.1 | HI581869.1 | GM652718.1 | AY657884.1 | DJ339589.1 | BD453625.1 | CS682279.1 |
| AX242116.1 | FB743780.1 | CS716781.1 | HI581837.1 | GM652654.1 | AY657852.1 | DJ339557.1 | BD453593.1 | CS674186.1 |
| AX242084.1 | FB761588.1 | CS798095.1 | HI578561.1 | GM652622.1 | AY657820.1 | DJ333995.1 | BD453543.1 | DD490727.1 |
| AX241988.1 | DL114132.1 | CS806053.1 | HI581373.1 | GM645496.1 | AY657788.1 | DJ327359.1 | BD453511.1 | DJ008395.1 |
| AX241924.1 | DL114100.1 | CS805337.1 | HI573739.1 | GM645464.1 | AY657756.1 | DJ122214.1 | BD453479.1 | DJ008363.1 |
| AX241892.1 | DL114068.1 | CS680868.1 | HI573670.1 | GM645432.1 | AY657724.1 | DJ086032.1 | BD453447.1 | DJ008331.1 |
| AX241860.1 | DL144483.1 | CS809313.1 | HI577673.1 | GM638539.1 | AY657692.1 | DJ082678.1 | BD453415.1 | DJ003340.1 |

| | | | | | | | | |
|---|---|---|---|---|---|---|---|---|
| AX241764.1 | DL126208.1 | CS790634.1 | HI593860.1 | GM638507.1 | AY657660.1 | DJ082549.1 | BD453376.1 | CS671201.1 |
| HW350958.1 | DL126176.1 | CS792482.1 | HI636981.1 | GM638475.1 | AY657628.1 | DJ084894.1 | BD453238.1 | CS671084.1 |
| HW350605.1 | DL122960.1 | CS792036.1 | HI001458.1 | GM638443.1 | AY657596.1 | DJ080728.1 | BD453206.1 | CS669875.1 |
| HW350291.1 | DL122928.1 | DJ044956.1 | HI001362.1 | GM633744.1 | AY657564.1 | DJ069483.1 | BD453119.1 | CS673652.1 |
| HW339574.1 | DL122896.1 | DJ028594.1 | HI001328.1 | GM633712.1 | AY657532.1 | DJ066373.1 | BD442215.1 | CS647414.1 |
| HW349610.1 | DL122864.1 | DJ044934.1 | HI003211.1 | GM633680.1 | AY657500.1 | DJ066341.1 | BD432361.1 | CS646221.1 |
| HW363627.1 | DL122832.1 | DJ044893.1 | HI003162.1 | GM643498.1 | AY657468.1 | DJ066308.1 | BD399076.1 | CS646189.1 |
| HW355321.1 | DL122800.1 | DJ030500.1 | HI003094.1 | GM643466.1 | AY657436.1 | DJ066274.1 | BD441333.1 | CS440787.1 |
| HW317123.1 | DL122768.1 | DJ028141.1 | HI003053.1 | GM643433.1 | AY657404.1 | DJ066240.1 | BD497776.1 | CS438948.1 |
| HW338974.1 | DL114022.1 | DJ033847.1 | HI003012.1 | GM636444.1 | AY657372.1 | DJ061421.1 | BD398715.1 | CS436062.1 |
| HW338902.1 | DL113990.1 | DJ030170.1 | HI002950.1 | GM631844.1 | AY657340.1 | DJ055572.1 | BD398636.1 | DD335880.1 |
| HW338518.1 | DL113958.1 | DJ030007.1 | HI002913.1 | GM631812.1 | AY657308.1 | DJ055189.1 | BD451731.1 | DD348422.1 |
| HW338134.1 | DL113926.1 | DJ029932.1 | HI001279.1 | GM631780.1 | AY657276.1 | DJ060396.1 | BD408120.1 | DD331010.1 |
| HW338006.1 | DL113894.1 | CS691574.1 | HI001230.1 | GM622849.1 | AY657244.1 | DJ056338.1 | BD408081.1 | DD349647.1 |
| HW337750.1 | DL113862.1 | CS693488.1 | HI001178.1 | GM657934.1 | AY657212.1 | DJ053103.1 | BD450224.1 | CS427059.1 |
| HW337622.1 | DL109061.1 | CS693247.1 | HI001106.1 | GM657902.1 | AY657180.1 | DJ053071.1 | BD439813.1 | CS425082.1 |
| HW337238.1 | DL017190.1 | CS674200.1 | HI001086.1 | GM657870.1 | AY657148.1 | CS810703.1 | BD408010.1 | CS422866.1 |
| HW336982.1 | DL017158.1 | CS692006.1 | HI001030.1 | GM657838.1 | AY657116.1 | CS416940.1 | BD449379.1 | CS421111.1 |
| HW336854.1 | DL017126.1 | DJ008406.1 | HI000996.1 | GM657806.1 | AY657084.1 | CS410886.1 | BD429353.1 | CS419002.1 |
| HW321334.1 | DL017094.1 | DJ008374.1 | HI000949.1 | GM657773.1 | AY657052.1 | CS414826.1 | BD418445.1 | CS415526.1 |
| HW336490.1 | DL017062.1 | DJ011712.1 | HI002852.1 | GM657741.1 | AF160759.1 | DD327646.1 | BD429042.1 | CS410887.1 |
| HW320842.1 | DL017030.1 | DJ008088.1 | HI002820.1 | GM643254.1 | M27881.1 | DD329891.1 | BD396351.1 | CS410806.1 |
| HW329379.1 | DL049429.1 | DJ011490.1 | HI002771.1 | GM631633.1 | M16806.1 | CS389262.1 | BD438217.1 | CS414829.1 |
| HW320773.1 | DL049397.1 | DJ004237.1 | HI002720.1 | GM631601.1 | M10301.1 | CS389216.1 | BD437748.1 | DD327186.1 |
| HW320479.1 | DL049365.1 | DJ010969.1 | HI004911.1 | GM622606.1 | M35115.1 | CS389178.1 | BD437670.1 | DD329892.1 |
| HW055441.1 | DL049333.1 | CS001392.1 | HI002675.1 | GM622446.1 | M13493.1 | CS406182.1 | BD459655.1 | DD328878.1 |
| HW064746.1 | DL049110.1 | AF408181.1 | HI002637.1 | GM622414.1 | M12708.1 | CS406499.1 | CS119189.1 | CS389263.1 |
| HW064681.1 | DL049046.1 | CS157961.1 | HI002602.1 | GM657408.1 | HV549900.1 | CS402783.1 | CS119157.1 | CS389217.1 |
| HW060674.1 | DL012570.1 | CS157929.1 | HC491770.1 | GM657376.1 | HV549606.1 | CS402578.1 | CS119125.1 | CS389180.1 |
| HW062416.1 | DL012538.1 | CS146881.1 | HC491738.1 | GM650120.1 | HV549101.1 | CS402157.1 | CS119058.1 | CS401406.1 |
| HW060585.1 | DL012506.1 | CS126601.1 | HC481738.1 | AY657224.1 | HV544377.1 | CS401815.1 | CS119025.1 | CS406500.1 |
| HW056332.1 | DL012474.1 | CS124722.1 | HC481602.1 | AY657192.1 | HV544217.1 | CS382072.1 | CS118992.1 | CS401816.1 |
| HW056244.1 | DL045452.1 | CS124684.1 | HC481565.1 | AY657160.1 | HV547856.1 | CS376535.1 | CS118927.1 | CS382084.1 |
| HW064458.1 | DL045420.1 | CS124644.1 | HC491463.1 | AY657128.1 | HV547442.1 | CS374666.1 | CS118894.1 | DD213837.1 |
| HW062359.1 | DL045356.1 | CS124600.1 | HC491064.1 | HI473104.1 | HV543472.1 | DD308883.1 | CS118861.1 | DD220785.1 |
| AX041934.1 | DL041084.1 | CS119547.1 | FV533619.1 | HI472902.1 | HV539726.1 | DD298331.1 | CS118828.1 | DD213578.1 |
| AX039925.1 | DL037197.1 | CS119513.1 | FV533444.1 | HI580550.1 | HV538623.1 | DD307930.1 | CS118795.1 | DD213546.1 |
| AX037648.1 | DL037133.1 | CS119481.1 | FV532171.1 | HI072386.1 | HV543195.1 | DD292505.1 | CS118763.1 | DD213485.1 |
| AX035987.1 | DL028730.1 | CS119447.1 | FV531693.1 | HI004363.1 | HV542595.1 | DD313219.1 | CS118730.1 | DD215948.1 |
| AX034876.1 | DL028698.1 | CS119413.1 | FV531411.1 | HI004235.1 | HV542031.1 | DD319731.1 | CS118698.1 | DD222033.1 |
| AX028778.1 | DL028666.1 | CS119380.1 | FV523612.1 | HI002271.1 | HV541509.1 | CS365224.1 | CS118666.1 | DD215569.1 |
| AX027441.1 | DL028634.1 | CS119348.1 | FV530987.1 | HI002234.1 | HV541310.1 | CS376409.1 | CS118634.1 | DD212257.1 |
| HW294986.1 | DL028602.1 | CS119315.1 | FV530859.1 | HI002184.1 | HV515083.1 | CS362659.1 | CS118602.1 | DD212127.1 |
| HW294122.1 | DL028570.1 | CS119282.1 | FV522831.1 | HI002137.1 | HV533247.1 | CS360548.1 | CS118568.1 | DD211835.1 |
| HW293920.1 | DL024756.1 | CS119250.1 | FV522791.1 | HI002102.1 | HV534819.1 | CS359733.1 | CS118534.1 | DD192529.1 |
| HW293260.1 | DL024724.1 | CS119218.1 | FV530353.1 | HI000448.1 | FV530906.1 | CS359594.1 | CS118436.1 | DD182428.1 |
| HW293040.1 | DL024692.1 | CS119186.1 | FV530206.1 | HI000400.1 | FV522837.1 | BD269134.1 | CS118404.1 | DD210749.1 |

| | | | | | | | | |
|---|---|---|---|---|---|---|---|---|
| HW292763.1 | DL012336.1 | CS119154.1 | FV529032.1 | HI000345.1 | FV522797.1 | BD268155.1 | CS118336.1 | DD200076.1 |
| HW291179.1 | DL037088.1 | CS119122.1 | FV528651.1 | HI553246.1 | FV522614.1 | BD268069.1 | CS118303.1 | DD187320.1 |
| HW291147.1 | DL038789.1 | CS119055.1 | FV534655.1 | HI553214.1 | FV534828.1 | BD267538.1 | CS118270.1 | DD206913.1 |
| HW291050.1 | DL038757.1 | CS119022.1 | FV534480.1 | HI553182.1 | HC465612.1 | BD263539.1 | CS118238.1 | DD206881.1 |
| HW291018.1 | DL038725.1 | CS118989.1 | FV534428.1 | HI583966.1 | HC461775.1 | CS119010.1 | CS118171.1 | DD206849.1 |
| HW290986.1 | DL038693.1 | CS118924.1 | FV534190.1 | HI583867.1 | HC453696.1 | AY967053.1 | CS118139.1 | DD206817.1 |
| HW290954.1 | DL035069.1 | CS118891.1 | FV533998.1 | HI568959.1 | HC453664.1 | AY967021.1 | CS118107.1 | CS250102.1 |
| HW290891.1 | DL035037.1 | CS118858.1 | HC465683.1 | HI207658.1 | AF030353.1 | AY966989.1 | CS118074.1 | DD181013.1 |
| HW290859.1 | DL034830.1 | CS118825.1 | HC465494.1 | HI002062.1 | HC449483.1 | AY966957.1 | CS118041.1 | AX838562.1 |
| HW298501.1 | DL030722.1 | CS118792.1 | HC462313.1 | HI002018.1 | FU756120.1 | AY966925.1 | CS118006.1 | AX824330.1 |
| HW269779.1 | DL030690.1 | HV226854.1 | HC461721.1 | HI001935.1 | FU758386.1 | CS054232.1 | CS117972.1 | AX823796.1 |
| HW266131.1 | DL026753.1 | HV221448.1 | HC460536.1 | HI000267.1 | FU757777.1 | CS052777.1 | CS115723.1 | AX817769.1 |
| HW263040.1 | DL026721.1 | HV221392.1 | AF527487.1 | HI000222.1 | HC358394.1 | CS052373.1 | CS114664.1 | AX814432.1 |
| HW262976.1 | DL026689.1 | HV214540.1 | AF335989.1 | HI000168.1 | HC358206.1 | CS052339.1 | CS113509.1 | AX805924.1 |
| HW262896.1 | DL026657.1 | HV304088.1 | HC456296.1 | HI000127.1 | HC358075.1 | CS052305.1 | CS113477.1 | AX799566.1 |
| HW262833.1 | DL026625.1 | HV217619.1 | HC453958.1 | HI470596.1 | HB837906.1 | CS048047.1 | CS113445.1 | AX798990.1 |
| HW262739.1 | DL026593.1 | HV040131.1 | HC453722.1 | HI551677.1 | HB845790.1 | CS047277.1 | CS113411.1 | AX798506.1 |
| HW261419.1 | DL026561.1 | HV097584.1 | HC453690.1 | HI544513.1 | HB845508.1 | CS038913.1 | CS113347.1 | AX798332.1 |
| HW261387.1 | DL019574.1 | HV188537.1 | HC453658.1 | HI560307.1 | HB837369.1 | CS038847.1 | CS113315.1 | AX798177.1 |
| HW261355.1 | DL019542.1 | HV197645.1 | HC458406.1 | HI564698.1 | HB845281.1 | AX555171.1 | CS112347.1 | AX797695.1 |
| HW261323.1 | DL019510.1 | HV191315.1 | U94950.1 | HI568913.1 | HB844707.1 | AX544013.1 | CS110952.1 | AX642397.1 |
| HW261291.1 | DL019478.1 | HV182460.1 | AF490554.1 | HI568849.1 | HB844348.1 | AX528455.1 | CS109284.1 | BD194483.1 |
| HW261227.1 | DL019446.1 | HV182428.1 | HC442354.1 | HI559001.1 | HB836744.1 | AX523905.1 | CS103351.1 | BD187681.1 |
| HW261195.1 | DL019414.1 | HV182396.1 | HC442282.1 | HI564255.1 | HB843997.1 | A30503.1 | CS106376.1 | AX787359.1 |
| HW261163.1 | DL014915.1 | HV182364.1 | HC452177.1 | HI564154.1 | HB836116.1 | A33933.1 | CS105798.1 | AX781524.1 |
| HW261131.1 | DL014883.1 | HV039821.1 | HC452067.1 | HI563646.1 | HB843238.1 | AX179528.1 | CS103435.1 | AX773272.1 |
| HW261099.1 | DL026501.1 | HV117758.1 | HC451979.1 | HI563614.1 | HB843023.1 | AX164105.1 | CS103333.1 | AX770677.1 |
| HW261003.1 | DL026469.1 | HV112798.1 | HC450989.1 | HI563579.1 | HB835316.1 | AX028013.1 | CS103301.1 | HH993684.1 |
| HW260971.1 | DL026437.1 | HV038760.1 | HC450956.1 | HI558031.1 | HB842843.1 | A28085.1 | CS102984.1 | HH986627.1 |
| HW260907.1 | DL026405.1 | HV112560.1 | HC450924.1 | HI508549.1 | HB842217.1 | AX521666.1 | CS102952.1 | HH979553.1 |
| HV502796.1 | DL026373.1 | HV038598.1 | HC450602.1 | HI551326.1 | HB841883.1 | AX512660.1 | CS102888.1 | HH979624.1 |
| HV502764.1 | DL022561.1 | HV182358.1 | FU772469.1 | HI544138.1 | HB841651.1 | AF430182.1 | CS102664.1 | HH979582.1 |
| HV502732.1 | DL022529.1 | HV182326.1 | FU773344.1 | HI508397.1 | HB841193.1 | AF430150.1 | CS102600.1 | FW394400.1 |
| HV502700.1 | DJ358885.1 | HV182294.1 | FU759959.1 | HI000038.1 | HB840493.1 | AX505189.1 | CS102568.1 | FW394236.1 |
| HV502636.1 | DJ347465.1 | HV182360.1 | FU759559.1 | HC295056.1 | HB647009.1 | AX496849.1 | CS102536.1 | FW394200.1 |
| HV502572.1 | DJ352790.1 | HV234613.1 | FU758618.1 | HC294876.1 | HB645728.1 | BD138731.1 | CS102504.1 | FW398044.1 |
| HV502540.1 | DJ359585.1 | HV234193.1 | FU764203.1 | HC299220.1 | HB855724.1 | BD138550.1 | CS101250.1 | FW397553.1 |
| HV502508.1 | DJ357682.1 | HV312975.1 | FU763329.1 | HC299185.1 | DM170837.1 | BD137362.1 | CS091387.1 | FW393343.1 |
| HV502444.1 | DJ357650.1 | HV302110.1 | DM194617.1 | HC299127.1 | DM170805.1 | BD136522.1 | CQ958264.1 | FW396877.1 |
| HV453670.1 | DJ357618.1 | HV030248.1 | DM203092.1 | HC294636.1 | HB559430.1 | BD135129.1 | CQ947453.1 | FW396422.1 |
| HV449927.1 | DJ357586.1 | HV037884.1 | HB828291.1 | HC291114.1 | HB491547.1 | BD133700.1 | CQ947130.1 | FW396666.1 |
| HV453066.1 | DJ363052.1 | FW392843.1 | HB810682.1 | HC290934.1 | HB489347.1 | BD131930.1 | CQ944200.1 | FW396504.1 |
| HV449439.1 | DJ362241.1 | HH931889.1 | HB839916.1 | HC290557.1 | GN359831.1 | BD130823.1 | CQ944168.1 | DM143442.1 |
| HV451136.1 | DJ354544.1 | HH821165.1 | HB847936.1 | HC296939.1 | GN359735.1 | BD130791.1 | CQ944136.1 | HB434953.1 |
| HV448647.1 | DJ361208.1 | HH832114.1 | HB847730.1 | HC289873.1 | AX512214.1 | BD130759.1 | CQ944104.1 | HB423266.1 |
| HV437504.1 | DJ354216.1 | HH822344.1 | HB839487.1 | HC289839.1 | AX497568.1 | BD130693.1 | CQ944072.1 | HB416486.1 |
| HV341333.1 | DJ360422.1 | HH822031.1 | HB847468.1 | HC200107.1 | AX496840.1 | BD130595.1 | CQ944040.1 | HB412535.1 |

| | | | | | | | | |
|---|---|---|---|---|---|---|---|---|
| HI401248.1 | DJ339855.1 | HH821976.1 | HB839216.1 | HC199264.1 | BD135966.1 | BD129643.1 | CQ944008.1 | HB394802.1 |
| HI638015.1 | DJ339823.1 | HH821908.1 | HB838782.1 | L07339.1 | BD134117.1 | BD107328.1 | CQ943976.1 | HB394267.1 |
| HI378141.1 | A32071.1 | FW379678.1 | HB805291.1 | HC193936.1 | BD133466.1 | BD105711.1 | CQ943944.1 | HB394235.1 |
| HI377256.1 | A32038.1 | FW379645.1 | HB847007.1 | HC193597.1 | BD131922.1 | BD103155.1 | CQ943912.1 | HB403370.1 |
| HI375900.1 | A31163.1 | FW379571.1 | HB838738.1 | HC190669.1 | BD130783.1 | BD087157.1 | CQ943880.1 | HB397712.1 |
| HI000016.1 | A30534.1 | FW379270.1 | HB832283.1 | HC188465.1 | BD130717.1 | BD084102.1 | CQ924874.1 | DM114272.1 |
| HI001872.1 | A28881.1 | FW378148.1 | HB846175.1 | HC187412.1 | AX019522.1 | BD081981.1 | A06159.1 | DM115564.1 |
| HI001786.1 | A28114.1 | FW377357.1 | HB804374.1 | HC089579.1 | AX011045.1 | BD081594.1 | CQ901658.1 | DM114857.1 |
| HI001750.1 | A26253.1 | FW376183.1 | HB815019.1 | HC089547.1 | AX010921.1 | BD080661.1 | CQ898654.1 | HB387459.1 |
| HI469668.1 | A20259.1 | FW381511.1 | HB809136.1 | HC089515.1 | AX008548.1 | BD080231.1 | CQ898622.1 | HB338861.1 |
| HI465283.1 | A27463.1 | FW375481.1 | HB808939.1 | HC089483.1 | AX003259.1 | BD080130.1 | CQ898590.1 | HA642841.1 |
| HI001628.1 | A26072.1 | FW369641.1 | HB845724.1 | HC089451.1 | AX002980.1 | BD076412.1 | CQ898558.1 | HA641592.1 |
| HI001586.1 | A24787.1 | FW380261.1 | HB845496.1 | HC089419.1 | U49848.1 | BD075327.1 | CQ898256.1 | DL486606.1 |
| HI003371.1 | A22313.1 | FW369302.1 | HB837311.1 | HC089386.1 | A25419.1 | BD074971.1 | CQ893711.1 | GM983985.1 |
| HI003333.1 | A16257.1 | HH736037.1 | HB813995.1 | HC089354.1 | A35721.1 | HW243082.1 | CQ891353.1 | GM996194.1 |
| HI464739.1 | A21722.1 | HH755049.1 | HB845206.1 | HC089321.1 | A32100.1 | HW241438.1 | CQ890279.1 | GM978856.1 |
| HI464707.1 | A19430.1 | FW359742.1 | HB844992.1 | HC089289.1 | A29453.1 | HW161327.1 | CQ888179.1 | GM993563.1 |
| HI464675.1 | A19397.1 | HC452045.1 | HB844567.1 | HC089257.1 | A35507.1 | HW161106.1 | CQ882113.1 | GM991947.1 |
| HI464643.1 | A18791.1 | HC451965.1 | HB844287.1 | AY775002.1 | A34510.1 | HW160496.1 | CQ877824.1 | GM976379.1 |
| HI528994.1 | A16022.1 | HC451771.1 | HB836732.1 | AY774944.1 | A33365.1 | HW155705.1 | A30342.1 | GM987031.1 |
| HI462578.1 | A14595.1 | HC450526.1 | HB843945.1 | AY774903.1 | A32418.1 | HW155503.1 | A23368.1 | GN000540.1 |
| HH713943.1 | A13654.1 | HC449018.1 | HB843521.1 | AY774844.1 | A30351.1 | HW154837.1 | A06237.1 | GM984660.1 |
| HH961362.1 | A13266.1 | FU772148.1 | HB835908.1 | AY774782.1 | A06247.1 | HW158459.1 | A30317.1 | FB740452.1 |
| HH961330.1 | A12677.1 | FU761017.1 | HB843188.1 | AY774618.1 | A31087.1 | HW154716.1 | A28235.1 | GM596707.1 |
| HH961298.1 | A11970.1 | FU759948.1 | HB835679.1 | AY774504.1 | A28467.1 | HW153781.1 | A19552.1 | GM969725.1 |
| HH961266.1 | HW158148.1 | FU757606.1 | HB829779.1 | AY774447.1 | A22258.1 | HW153297.1 | A14633.1 | FB777935.1 |
| HH961234.1 | HW154123.1 | HC439413.1 | HB813472.1 | AY774282.1 | A17374.1 | HW157486.1 | A12288.1 | DL479248.1 |
| HH975400.1 | HW153794.1 | HC438921.1 | HB807321.1 | AY774226.1 | A11056.1 | HW160312.1 | A10759.1 | DL477728.1 |
| HH975097.1 | HW153718.1 | HC438420.1 | HB842798.1 | AY774114.1 | A06501.1 | HV940079.1 | A10471.1 | DL464543.1 |
| HH974676.1 | HW145127.1 | HC360364.1 | HB806979.1 | AY774040.1 | A07756.1 | HV939879.1 | A07378.1 | DL476958.1 |
| HH974557.1 | HW153308.1 | HC358224.1 | HB842140.1 | AY645668.1 | A00062.1 | HV943367.1 | A04013.1 | DL476692.1 |
| HH974409.1 | HW153260.1 | HC358094.1 | HB841803.1 | J02541.1 | A31610.1 | HV942440.1 | A00484.1 | DL463063.1 |
| HH998094.1 | HW152275.1 | HC358061.1 | HB841600.1 | AF283768.1 | A30601.1 | HV936878.1 | A32044.1 | DL470104.1 |
| HH998033.1 | HW151829.1 | HC357539.1 | HB834070.1 | HC070099.1 | A29270.1 | HV931343.1 | A31312.1 | DL462901.1 |
| HH997987.1 | HW144745.1 | HC434782.1 | HB840945.1 | HC083548.1 | A28932.1 | HV936743.1 | A30558.1 | DL482392.1 |
| HH999672.1 | HW126527.1 | HC434750.1 | HB840446.1 | HC083516.1 | A22632.1 | HV925518.1 | A28889.1 | DL475867.1 |
| HH999526.1 | HW144375.1 | HC325459.1 | HB650455.1 | HH998327.1 | A25197.1 | HV939036.1 | A26619.1 | DL475554.1 |
| HH997858.1 | HW144311.1 | A07178.1 | HB650237.1 | HH998273.1 | A23313.1 | HV936379.1 | A22625.1 | DL461258.1 |
| HH997790.1 | HW124807.1 | HC324886.1 | HB649642.1 | GN007487.1 | A23256.1 | HV925332.1 | A27817.1 | FB504564.1 |
| HH997751.1 | HW124520.1 | HC324502.1 | HB649416.1 | FB740462.1 | A22413.1 | HV932680.1 | A26381.1 | GM832058.1 |
| HH999475.1 | HW124065.1 | HC315508.1 | HB648569.1 | GM970523.1 | A01972.1 | HV929985.1 | A25750.1 | GM648159.1 |
| GM629349.1 | HW123525.1 | HC313925.1 | HB648537.1 | GM970132.1 | A21812.1 | HV802932.1 | A22889.1 | GM641196.1 |
| GM629317.1 | HW123460.1 | HC318729.1 | HB648503.1 | GM969778.1 | A21460.1 | HV802900.1 | A22257.1 | GM628700.1 |
| GM655733.1 | HW122793.1 | HC308577.1 | HB648471.1 | GM969728.1 | A19568.1 | HV547829.1 | A18248.1 | GM640905.1 |
| GM655701.1 | HW120096.1 | HC307859.1 | HB645722.1 | DL479994.1 | A16246.1 | HV547732.1 | A16332.1 | GM640873.1 |
| GM655669.1 | HW118281.1 | HC307369.1 | HB645690.1 | DL477749.1 | A17973.1 | HV543478.1 | A21986.1 | GM640841.1 |
| GM655637.1 | HW115617.1 | FU257898.1 | HB645619.1 | DL464699.1 | A16043.1 | HV539098.1 | A21425.1 | GM628487.1 |

| | | | | | | | | |
|---|---|---|---|---|---|---|---|---|
| GM655605.1 | HW048258.1 | FU257866.1 | HB645581.1 | DL464546.1 | HW260685.1 | HV538670.1 | A21209.1 | GM047098.1 |
| GM655573.1 | HV975527.1 | DM091558.1 | HB645549.1 | DL476961.1 | HW260653.1 | HV538540.1 | A19477.1 | GM654818.1 |
| GM648580.1 | HV985968.1 | DM094903.1 | HB645429.1 | DL483005.1 | HW260621.1 | HV542848.1 | A13671.1 | GM654786.1 |
| GM648548.1 | HV985936.1 | DM102932.1 | HB851007.1 | DL463066.1 | HW260557.1 | HV541548.1 | A18344.1 | GM647697.1 |
| GM648516.1 | HV985904.1 | GN367741.1 | HB858832.1 | DL470132.1 | HW260525.1 | HV514617.1 | A17585.1 | GM046234.1 |
| GM648452.1 | HV985859.1 | GN367620.1 | HB856838.1 | DL462972.1 | HW260493.1 | HV534839.1 | A15380.1 | GM627959.1 |
| GM648420.1 | HW028882.1 | GN373336.1 | HB855912.1 | DL482398.1 | HW260461.1 | HV535835.1 | A16028.1 | GM627927.1 |
| GM641617.1 | HV984390.1 | GN361587.1 | DM179550.1 | DL462044.1 | HW260429.1 | HV515989.1 | A12752.1 | GM639387.1 |
| GM641585.1 | HV984266.1 | GN360159.1 | DM179159.1 | DL475564.1 | HW260397.1 | HV515724.1 | A12358.1 | GM639355.1 |
| GM641553.1 | HV986520.1 | GN360095.1 | DM179082.1 | FB504567.1 | HW260365.1 | HV515571.1 | A12059.1 | GM646083.1 |
| GM641521.1 | HV961491.1 | GN356164.1 | DM178844.1 | GM832061.1 | HW260333.1 | HV515507.1 | A10832.1 | GM646051.1 |
| GM641489.1 | HV960526.1 | GN360022.1 | DM170831.1 | GM712718.1 | HW260301.1 | DL035335.1 | A02584.1 | GM660374.1 |
| GM641457.1 | HV960203.1 | GN359894.1 | DM170792.1 | GM712161.1 | HW260269.1 | DL027170.1 | A09228.1 | GM660342.1 |
| GM641425.1 | HV960144.1 | GN366320.1 | DM185698.1 | GM827700.1 | HW260237.1 | DJ060183.1 | A08272.1 | GM653155.1 |
| GM634237.1 | HV959469.1 | GN366288.1 | DM185654.1 | GM742773.1 | HW260205.1 | DJ056272.1 | A07334.1 | GM653123.1 |
| GM634205.1 | HV963862.1 | GN366256.1 | DM170653.1 | FB509174.1 | HW260173.1 | CS810623.1 | A06034.1 | GM653091.1 |
| GM634141.1 | HV966039.1 | A10352.1 | DM048638.1 | GM774138.1 | HW260141.1 | FB296094.1 | A05145.1 | GM653059.1 |
| GM634109.1 | HV958117.1 | A07981.1 | DM045541.1 | GM710188.1 | HW260109.1 | CS812756.1 | A04928.1 | GM639001.1 |
| GM634077.1 | HV969960.1 | A01731.1 | DM056760.1 | GM709007.1 | HW260077.1 | DD468180.1 | A01182.1 | GM638813.1 |
| GM629272.1 | HV965365.1 | A05197.1 | DM045282.1 | GM708975.1 | HW260045.1 | CS803354.1 | A07986.1 | GM638781.1 |
| GM629208.1 | HV969805.1 | A04239.1 | DM045005.1 | GM655004.1 | HW260013.1 | CS802329.1 | A05362.1 | DL125473.1 |
| GM889330.1 | HV961661.1 | A01346.1 | DM039617.1 | GM044930.1 | HW259981.1 | DJ047078.1 | A01738.1 | DL125441.1 |
| GM889256.1 | HV950446.1 | M21207.1 | DM039584.1 | GM719208.1 | HW259949.1 | DJ046961.1 | HW341237.1 | DL125409.1 |
| GM828398.1 | HV948978.1 | M18436.1 | DM055991.1 | GM717970.1 | HW259917.1 | DJ050549.1 | HW351352.1 | DL125377.1 |
| GM888718.1 | HV957584.1 | M60892.1 | DM044703.1 | GM669147.1 | HW259520.1 | DJ049435.1 | HW350984.1 | DL125345.1 |
| GM008874.1 | HV947286.1 | M13108.1 | DM060717.1 | GM648963.1 | HW258919.1 | DJ048824.1 | HW350943.1 | DL102457.1 |
| GM888522.1 | HV953239.1 | J02539.1 | DM044566.1 | GM648931.1 | HW258852.1 | CS793882.1 | HW350698.1 | DL102425.1 |
| GM872376.1 | HV554500.1 | M13356.1 | GN069432.1 | GM648899.1 | HW257540.1 | CS720808.1 | HW350653.1 | DL102393.1 |
| FB661306.1 | HV553659.1 | M19084.1 | FW572261.1 | GM648867.1 | HW257400.1 | CS799085.1 | HW350558.1 | DL097620.1 |
| GM005941.1 | HV552825.1 | LC732345.1 | FW571111.1 | GM648835.1 | HW257304.1 | CS716821.1 | HW350349.1 | DL097588.1 |
| GM867496.1 | HV551418.1 | MF989991.1 | FW568762.1 | GM648803.1 | HW257240.1 | CS716750.1 | HW349937.1 | DL097556.1 |
| FB984710.1 | AY659404.1 | L35896.1 | FW573275.1 | GM634524.1 | HW257208.1 | CS716073.1 | HW349595.1 | DL095369.1 |
| FB983220.1 | AY659372.1 | DQ158862.1 | FW570981.1 | GM634492.1 | HW257144.1 | CS714153.1 | HW347800.1 | DL102914.1 |
| GM682915.1 | AY659340.1 | M15659.1 | FW420448.1 | GM629687.1 | HW257080.1 | CS675432.1 | HW347768.1 | CS141553.1 |
| GM681834.1 | AY659308.1 | K01192.1 | FW420416.1 | GM648739.1 | HW256984.1 | CS680688.1 | HW347640.1 | CS141515.1 |
| FB983168.1 | AY659276.1 | AF074852.1 | FW503331.1 | GM648707.1 | HW256952.1 | CS790263.1 | HW347607.1 | CS141483.1 |
| FB753636.1 | AY659244.1 | LC002204.1 | FW501750.1 | GM648675.1 | HW256760.1 | CS791086.1 | HW347479.1 | CS138716.1 |
| GM680850.1 | AY659212.1 | HW104340.1 | FW497039.1 | GM648643.1 | HW254483.1 | CS791899.1 | HW363722.1 | CS122926.1 |
| GM865370.1 | AY659180.1 | HW104306.1 | FW420391.1 | GM648611.1 | HW251032.1 | DJ044909.1 | HW363621.1 | CS122363.1 |
| GM618795.1 | AY659148.1 | HW071191.1 | FW420359.1 | GM634459.1 | HW240848.1 | DJ028124.1 | HW355199.1 | CS106157.1 |
| GM618670.1 | AY659116.1 | HW104192.1 | FW420327.1 | GM634427.1 | HW240600.1 | DJ027950.1 | HW355121.1 | CS106060.1 |
| FB725965.1 | AY659084.1 | HW103994.1 | EU363764.1 | GM634395.1 | HW239415.1 | DD212024.1 | HW344658.1 | CS179692.1 |
| GM864117.1 | AY659052.1 | HW099610.1 | FW498482.1 | GM629430.1 | HW238941.1 | DD182409.1 | HW344707.1 | CS179210.1 |
| GM842495.1 | AY659020.1 | HW099534.1 | FW496530.1 | GM629398.1 | HW238382.1 | DD206908.1 | HW344519.1 | CS327369.1 |
| FB725649.1 | AY658988.1 | HW069229.1 | FW501574.1 | GM629366.1 | HW238241.1 | DD206876.1 | HW344643.1 | CS323614.1 |
| GM618447.1 | AY658956.1 | HW089216.1 | HI653995.1 | GM629334.1 | HW238016.1 | DD206844.1 | HW317099.1 | CS329387.1 |
| GM615889.1 | AY658924.1 | HW067353.1 | HI653812.1 | GM629302.1 | HV502798.1 | DD206782.1 | HW326519.1 | CS329262.1 |

| | | | | | | | | |
|---|---|---|---|---|---|---|---|---|
| FB712546.1 | AY658892.1 | HW066708.1 | HI653641.1 | GM648533.1 | HV502766.1 | DD196872.1 | HW326395.1 | BD325701.1 |
| GM731778.1 | AY658860.1 | HW083263.1 | HI653606.1 | GM648501.1 | HV502734.1 | CS247803.1 | HW326280.1 | BD325603.1 |
| GM614671.1 | AY658828.1 | HW083231.1 | HI653536.1 | GM629161.1 | HV502702.1 | DD181008.1 | HW332578.1 | BD325499.1 |
| GM840811.1 | AY658796.1 | HW088012.1 | HI653495.1 | GM629129.1 | CQ973179.1 | CS082049.1 | HW338622.1 | BD314346.1 |
| FB711635.1 | AY658764.1 | HW081779.1 | HI653427.1 | GM619874.1 | CQ977674.1 | CS079778.1 | HW338494.1 | BD312674.1 |
| GM967134.1 | AY658732.1 | HW081690.1 | HI653375.1 | FB571355.1 | CQ976308.1 | CS075319.1 | HW338238.1 | BD319278.1 |
| GM041237.1 | AY658700.1 | HW099462.1 | HI653287.1 | FB571239.1 | CQ970097.1 | AY967403.1 | HW337982.1 | BD319398.1 |
| GM840280.1 | AY658668.1 | HW103533.1 | HI653229.1 | CS696159.1 | CQ965261.1 | AY967371.1 | HW337854.1 | BD300041.1 |
| GM040641.1 | GN013605.1 | HW103415.1 | HI647563.1 | CS696063.1 | CQ947135.1 | AY967339.1 | HW290917.1 | BD300346.1 |
| GM040445.1 | GN013375.1 | HW059857.1 | HI646929.1 | CS695998.1 | CQ944205.1 | AY967307.1 | HW290886.1 | BD293534.1 |
| GM839371.1 | GN010218.1 | HW061887.1 | AY145519.1 | CS695966.1 | CQ944173.1 | AY967275.1 | HW290854.1 | BD291303.1 |
| CS728657.1 | GN033548.1 | HW061854.1 | HH806357.1 | CS695902.1 | CQ944141.1 | AY967243.1 | HW290398.1 | BD299398.1 |
| CS728561.1 | GN033516.1 | HW058820.1 | HH806325.1 | CS695838.1 | CQ944109.1 | AY967211.1 | HW290133.1 | BD298371.1 |
| CS727437.1 | GN033452.1 | HW065237.1 | HH804191.1 | CS695806.1 | CQ944077.1 | AY967179.1 | HW298238.1 | BD294893.1 |
| CS727337.1 | GN033388.1 | HW061393.1 | HH793040.1 | CS695774.1 | CQ944045.1 | AY967147.1 | HW288960.1 | BD283886.1 |
| CS727235.1 | GN033356.1 | HW058494.1 | HH792763.1 | CS695742.1 | CQ944013.1 | AY967115.1 | HW268857.1 | BD283568.1 |
| GM011227.1 | GN033324.1 | HW064838.1 | HH791754.1 | CS695710.1 | CQ943981.1 | AY967083.1 | HW285154.1 | BD276193.1 |
| GM888673.1 | GN033292.1 | HW061123.1 | HH791633.1 | CS695582.1 | CQ943949.1 | AY967051.1 | HW269946.1 | BD274844.1 |
| GM888577.1 | GN033260.1 | HW056752.1 | HH779718.1 | CS695454.1 | CQ943917.1 | AY967019.1 | HW269325.1 | BD272770.1 |
| GM887791.1 | GN033228.1 | HW056580.1 | HH777928.1 | CS695422.1 | CQ943885.1 | AY966987.1 | HW269074.1 | BD271968.1 |
| GM683207.1 | GN033164.1 | HW064602.1 | HH820925.1 | DL176495.1 | CQ943853.1 | AY966955.1 | HW267855.1 | BD271159.1 |
| FB754174.1 | GN033132.1 | HW062549.1 | HH759579.1 | DL176350.1 | CQ924200.1 | AY966923.1 | HW267631.1 | DD230433.1 |
| GM888025.1 | GN033068.1 | HV694768.1 | HH759219.1 | DL181652.1 | A16320.1 | CS054196.1 | HW266272.1 | DD227719.1 |
| GM879381.1 | GN033004.1 | HV700699.1 | HH759187.1 | DL174617.1 | CQ898659.1 | CS052526.1 | HW265996.1 | DD227405.1 |
| GM680851.1 | GN032972.1 | HV694679.1 | HH759155.1 | DL174505.1 | CQ898595.1 | CS052405.1 | HW265862.1 | DD224451.1 |
| GM879360.1 | GN032940.1 | HV694642.1 | HH759123.1 | DL182873.1 | CQ898563.1 | CS052371.1 | FV531721.1 | DD231461.1 |
| FB728350.1 | GN032908.1 | HV700251.1 | HH759091.1 | DL176762.1 | CQ888092.1 | CS052337.1 | FV531689.1 | AX754695.1 |
| GM842473.1 | GN032876.1 | HV693191.1 | HH759059.1 | DL176659.1 | CQ871428.1 | CS052303.1 | FV531381.1 | AX752753.1 |
| GM731698.1 | GN032844.1 | HV699082.1 | HH759027.1 | FB343458.1 | CQ871396.1 | CS051047.1 | FV522827.1 | AX746472.1 |
| GM040761.1 | GN032812.1 | HV690018.1 | HH758995.1 | DL088083.1 | BD130813.1 | CS048045.1 | FV522787.1 | BD184758.1 |
| GM863434.1 | GN032780.1 | HV695921.1 | HH758963.1 | DL088051.1 | BD130781.1 | CS047275.1 | FV530202.1 | BD182991.1 |
| GM863143.1 | GN032749.1 | HV695653.1 | HH758931.1 | DL091928.1 | BD130572.1 | CS039427.1 | FV528647.1 | BD181492.1 |
| GM841834.1 | GN032717.1 | HV688794.1 | HH758899.1 | DL091896.1 | BD091308.1 | AX145364.1 | FV534424.1 | BD181215.1 |
| DL120890.1 | GN032685.1 | HV600528.1 | HH758867.1 | DL091864.1 | BD081529.1 | AX145332.1 | FV534057.1 | BD180852.1 |
| DL120858.1 | GN032653.1 | HV579910.1 | HH937344.1 | DL102889.1 | BD074961.1 | AX145300.1 | FV533993.1 | BD180764.1 |
| DL120826.1 | GN032620.1 | HV579746.1 | HH935169.1 | DL102857.1 | BD073866.1 | AX145268.1 | HC465489.1 | AX743755.1 |
| DL124932.1 | GN032588.1 | HV585093.1 | HH934832.1 | DL102825.1 | BD069509.1 | AX145236.1 | HC456292.1 | AY181089.1 |
| DL124900.1 | GN032525.1 | HV584789.1 | HH934455.1 | DL102793.1 | BD016709.1 | AX145204.1 | HC453686.1 | BD177541.1 |
| DL124868.1 | GN032493.1 | HV579127.1 | HH834794.1 | DL107573.1 | BD014241.1 | AX145172.1 | HC453654.1 | AX708746.1 |
| DL124836.1 | GN032429.1 | HV579008.1 | HH834163.1 | DL119349.1 | BD014208.1 | AX145140.1 | HC460196.1 | AX705320.1 |
| DL124804.1 | GN032397.1 | HV588518.1 | HH833953.1 | DL119317.1 | BD014168.1 | AX145108.1 | HC458386.1 | AX703574.1 |
| DL124772.1 | GM643057.1 | HV592422.1 | HH833709.1 | DL114825.1 | BD013762.1 | AX145076.1 | HC442346.1 | AX699450.1 |
| DL120779.1 | GM643025.1 | HV578488.1 | HH833649.1 | CS444601.1 | AX482021.1 | AX145044.1 | HC452249.1 | AX699418.1 |
| DL120747.1 | GM636036.1 | HV577342.1 | HH833617.1 | DD089927.1 | AX472662.1 | AX145012.1 | HC452167.1 | AX664935.1 |
| DL116216.1 | GM636004.1 | HV582980.1 | HH833585.1 | DD102596.1 | E64487.1 | AX144980.1 | HC452109.1 | AX662195.1 |
| DL116184.1 | GM622216.1 | HV581983.1 | HH833553.1 | DD057933.1 | AX469479.1 | AX144948.1 | HC451403.1 | AX657132.1 |
| DL116152.1 | GM622184.1 | HV575479.1 | HH833521.1 | DD057901.1 | AX467092.1 | AX144915.1 | HC449536.1 | AX657100.1 |

| | | | | | | | | |
|---|---|---|---|---|---|---|---|---|
| DL033222.1 | GM622152.1 | HV601187.1 | HH833489.1 | DD057869.1 | AX460840.1 | AX144883.1 | FU265347.1 | BD176065.1 |
| DL033190.1 | GM631011.1 | HV601081.1 | HH833457.1 | DD057841.1 | AX458589.1 | AX144851.1 | FU262652.1 | BD174759.1 |
| DL033126.1 | GM630979.1 | HV574880.1 | HH833425.1 | DD057125.1 | AX458210.1 | AX144787.1 | FU250477.1 | AX644226.1 |
| DL029118.1 | GM630947.1 | HV574374.1 | HH833393.1 | DD053910.1 | AX456891.1 | AX144755.1 | FU262464.1 | AX615143.1 |
| DL029086.1 | GM630915.1 | HV589985.1 | HH833368.1 | DD053297.1 | AX454007.1 | AX144723.1 | FU267897.1 | BD171474.1 |
| DL029054.1 | GM630890.1 | HV585649.1 | HH833336.1 | DD052281.1 | AX453567.1 | AX144691.1 | FU265031.1 | AX601608.1 |
| DL029022.1 | GM635847.1 | HV570594.1 | HH837055.1 | DD051920.1 | AX451645.1 | AX144659.1 | FU264928.1 | AX601576.1 |
| DL028990.1 | GM635815.1 | HV331763.1 | FW392852.1 | DD056470.1 | AX443326.1 | AX144627.1 | FU253488.1 | A26692.1 |
| DL028958.1 | GM635783.1 | HV324594.1 | FW383055.1 | DD058905.1 | AX430069.1 | AX144513.1 | FU261570.1 | A26056.1 |
| DL025144.1 | GM630883.1 | HV323865.1 | HH928903.1 | DD058531.1 | AX427127.1 | AX144449.1 | FU269785.1 | A25583.1 |
| DL025112.1 | GM642965.1 | HV111844.1 | HH931896.1 | DD041902.1 | AX418258.1 | AX144257.1 | HC313141.1 | A24313.1 |
| DL025080.1 | GM642932.1 | HV098613.1 | HH821550.1 | DD046593.1 | HW350989.1 | AX144127.1 | DM475255.1 | A25384.1 |
| DL025016.1 | GM630656.1 | HV119924.1 | HH821432.1 | DD038716.1 | HW350954.1 | AX143997.1 | DM465821.1 | A23997.1 |
| DL024984.1 | GM630624.1 | HV192788.1 | HH821179.1 | DD037263.1 | HW350658.1 | AX143869.1 | HC299789.1 | A23263.1 |
| DL021772.1 | GM630592.1 | HV218540.1 | HH821119.1 | DD032569.1 | HW350290.1 | AX143805.1 | HC306137.1 | A22470.1 |
| DL021740.1 | GM621657.1 | HV208757.1 | HH827437.1 | DD032252.1 | HW339571.1 | AX143741.1 | HC306097.1 | A21096.1 |
| DL021708.1 | GM621593.1 | HV218247.1 | HH827373.1 | DD030665.1 | HW349890.1 | AX143677.1 | HC306057.1 | A11924.1 |
| DL021676.1 | GM621561.1 | HV236001.1 | HH827010.1 | DD017702.1 | HW355320.1 | HH998649.1 | HC306017.1 | A21830.1 |
| DL021612.1 | GM657141.1 | HV247167.1 | HH826849.1 | DD012858.1 | HW315573.1 | HH998575.1 | HC305977.1 | A21493.1 |
| DL017406.1 | GM657109.1 | HV301800.1 | HH826529.1 | BD412637.1 | HW315540.1 | HH998521.1 | HC305906.1 | A16544.1 |
| DL017374.1 | GM657077.1 | HV301579.1 | HH822049.1 | BD434083.1 | HW315180.1 | HH996907.1 | HC305866.1 | A20193.1 |
| DL017342.1 | GM657045.1 | HV306182.1 | HH821915.1 | BD453859.1 | HW315148.1 | HH996786.1 | HC301315.1 | A17101.1 |
| DL017310.1 | GM657013.1 | HV303088.1 | FW379760.1 | BD453827.1 | HW084956.1 | HH996751.1 | HC301148.1 | A05426.1 |
| DL017278.1 | GM656949.1 | HV302944.1 | FW379717.1 | BD453795.1 | HW097750.1 | HH994524.1 | HC301024.1 | A15450.1 |
| DL017246.1 | GM656918.1 | HV312636.1 | FW379685.1 | BD433659.1 | HW097643.1 | HH980708.1 | HC254431.1 | A13189.1 |
| DL012953.1 | GM656886.1 | HV308792.1 | FW379652.1 | BD453771.1 | HW065065.1 | GN337633.1 | HC291462.1 | A12552.1 |
| DL012921.1 | GM656854.1 | HV304171.1 | FW379620.1 | BD453739.1 | HW050536.1 | GN336557.1 | HC294146.1 | A11055.1 |
| DL012889.1 | GM870690.1 | HV221447.1 | FW374117.1 | BD453707.1 | HV969763.1 | GN112640.1 | HC289557.1 | A10443.1 |
| DL012825.1 | GM661716.1 | HV221391.1 | FW379578.1 | BD453675.1 | HV951089.1 | DM064153.1 | HC289353.1 | A09994.1 |
| DL012793.1 | GM661652.1 | HV309572.1 | FW379546.1 | BD453643.1 | HV778441.1 | DM077411.1 | HC289321.1 | A08462.1 |
| DL045633.1 | GM661620.1 | HV312019.1 | FW378258.1 | BD453611.1 | HV757323.1 | GN090915.1 | HC289183.1 | A08094.1 |
| DL045601.1 | GM661588.1 | HV217618.1 | FW378155.1 | BD493415.1 | HV743700.1 | GM993554.1 | HC296062.1 | A07724.1 |
| DL045569.1 | GM661556.1 | HV040130.1 | FW377974.1 | BD453574.1 | HW286183.1 | GM992505.1 | HC295908.1 | A02736.1 |
| DL045537.1 | GM661524.1 | HV097554.1 | FW377114.1 | BD453561.1 | HW154654.1 | GM976351.1 | HC289001.1 | A01473.1 |
| DL045505.1 | GM654530.1 | HV188535.1 | DD405036.1 | BD453529.1 | HW125553.1 | GM989822.1 | HC288842.1 | A05105.1 |
| DL045473.1 | GM654498.1 | HV200539.1 | DD408895.1 | BD453497.1 | HW125337.1 | GM987013.1 | HC295592.1 | A02708.1 |
| DL041361.1 | GM654466.1 | HV182491.1 | DD404686.1 | BD453465.1 | HW144354.1 | GN000748.1 | HC295450.1 | A01417.1 |
| DL041329.1 | GM654370.1 | HV182459.1 | DD407074.1 | BD453433.1 | HW144322.1 | GM970625.1 | HC288708.1 | A00728.1 |
| DL041297.1 | GM647281.1 | HV182427.1 | DD409749.1 | BD453401.1 | HW124840.1 | GM970092.1 | HC292038.1 | M25763.1 |
| DL037442.1 | GM647249.1 | HV182395.1 | DD405839.1 | BD453256.1 | HW124694.1 | DL479491.1 | HC291870.1 | HV508641.1 |
| DL037410.1 | GM647217.1 | HV182363.1 | DD405807.1 | BD453224.1 | HW117984.1 | DL464534.1 | HC299195.1 | HV508577.1 |
| DL037378.1 | GM640286.1 | HV216679.1 | DD402411.1 | BD453192.1 | HW096547.1 | DL463054.1 | HC299137.1 | HV508513.1 |
| DL037314.1 | GM870242.1 | HI574395.1 | DD402347.1 | BD411232.1 | HW105486.1 | DL462992.1 | HC291606.1 | HV508485.1 |
| DL033111.1 | GM627583.1 | HI573721.1 | DD402315.1 | BD442386.1 | HW105054.1 | DL461709.1 | HC294374.1 | FW390816.1 |
| DL033079.1 | GM627551.1 | HI573557.1 | DD402283.1 | BD180856.1 | HW103889.1 | FB504554.1 | HC293810.1 | FW392862.1 |
| DL023252.1 | GM627519.1 | HI553807.1 | DD402251.1 | AX743759.1 | HW099563.1 | GM831914.1 | HC290677.1 | HH925714.1 |
| DL023220.1 | GM661492.1 | HI593064.1 | DD402219.1 | AX722157.1 | HW068951.1 | GM712216.1 | HC290174.1 | HH827052.1 |

| | | | | | | | | |
|---|---|---|---|---|---|---|---|---|
| DL023188.1 | GM661428.1 | HI592174.1 | DD402187.1 | AX721037.1 | HW067599.1 | GM619893.1 | HC208416.1 | HH826949.1 |
| DL020173.1 | GM661396.1 | HI637151.1 | DD402155.1 | BD177334.1 | HW066742.1 | GM648182.1 | HC289883.1 | HH822197.1 |
| DL020141.1 | GM661364.1 | HI636968.1 | DD402123.1 | AX720089.1 | HW083273.1 | GM639378.1 | HC289849.1 | FW381565.1 |
| DL020077.1 | GM661332.1 | HI636930.1 | DD402091.1 | AX719157.1 | HW083241.1 | GM639346.1 | HC207915.1 | FW375275.1 |
| DL020045.1 | GM654338.1 | HI001449.1 | DD402059.1 | AX712180.1 | HV766819.1 | FB743945.1 | HC207350.1 | HH736076.1 |
| DL020013.1 | GM654306.1 | HI001390.1 | DD405779.1 | AX710072.1 | HV760624.1 | FB743912.1 | HC201452.1 | HD033946.1 |
| DL015546.1 | GM654274.1 | HI001353.1 | DD405747.1 | AX704452.1 | HV752892.1 | FB743880.1 | HC199339.1 | HD033701.1 |
| DL015514.1 | GM654242.1 | HI001319.1 | DD405715.1 | AX699454.1 | HV749443.1 | FB743848.1 | DM171098.1 | HD057707.1 |
| DL015482.1 | GM654178.1 | HI003202.1 | DD405683.1 | AX699422.1 | HV753733.1 | FB743794.1 | DM179155.1 | HD057675.1 |
| DL015450.1 | GM647185.1 | HI003084.1 | DD405651.1 | AX685557.1 | HV703145.1 | FB761768.1 | DM179060.1 | HD062035.1 |
| DL010992.1 | GM647153.1 | HI003044.1 | DD405619.1 | AX675243.1 | HV702377.1 | FB742922.1 | DM178831.1 | U19100.1 |
| DL010960.1 | GM647121.1 | HI003003.1 | DD405599.1 | AX670744.1 | HV702267.1 | FB708875.1 | DM170827.1 | M81900.1 |
| DL010928.1 | GM647089.1 | HI002941.1 | DD405567.1 | AX657136.1 | HV702235.1 | FB676985.1 | DM185775.1 | AY569171.1 |
| DL010896.1 | GM647049.1 | HI002904.1 | DD405503.1 | AX657104.1 | HV701285.1 | GM036182.1 | DM185694.1 | HC917651.1 |
| DL010864.1 | HC201652.1 | HI001267.1 | DD405471.1 | BD176071.1 | HV701223.1 | GM894297.1 | DM170616.1 | FW337650.1 |
| DL010832.1 | HC200110.1 | HI001213.1 | DD405439.1 | BD171515.1 | HV701191.1 | GM890141.1 | DM188047.1 | FW336998.1 |
| DL070193.1 | HC199286.1 | HI001156.1 | DD402033.1 | AX614791.1 | HV701159.1 | GM963593.1 | DM169879.1 | HC887331.1 |
| DL023153.1 | DM464955.1 | HI001058.1 | DD402001.1 | AX601796.1 | HV701127.1 | GM889530.1 | DM176836.1 | HC873814.1 |
| DL023121.1 | DM463599.1 | HI001019.1 | DD401969.1 | AX601612.1 | HV708785.1 | FB983173.1 | HB491537.1 | HC873719.1 |
| DL023089.1 | HC195397.1 | HI000977.1 | DD401908.1 | AX601516.1 | HV543310.1 | FB753859.1 | HB489259.1 | HC880378.1 |
| DL023057.1 | HC195230.1 | HI002881.1 | DD401876.1 | AX601315.1 | HV542761.1 | GM751823.1 | HB488827.1 | HC879675.1 |
| DL023025.1 | HC187066.1 | HI002843.1 | DD401844.1 | AX598856.1 | HV542725.1 | GM618810.1 | HB488777.1 | HC727362.1 |
| DL022993.1 | HC089582.1 | HI002806.1 | DD401812.1 | AX036015.1 | HV542693.1 | FB725703.1 | DM164826.1 | HC688475.1 |
| DL019946.1 | HC089550.1 | HI002746.1 | DD401748.1 | AX033857.1 | HV542188.1 | FB722369.1 | DM159988.1 | HC688412.1 |
| DL019914.1 | HC089518.1 | HI002711.1 | DD401716.1 | AX028811.1 | HV541501.1 | GM731820.1 | DM164029.1 | HC687679.1 |
| DL019818.1 | HC089486.1 | HI003282.1 | DD401684.1 | AX027866.1 | HV527898.1 | GM840826.1 | DM164014.1 | HC490854.1 |
| DL015415.1 | HC089454.1 | HI002662.1 | DD401652.1 | AX024044.1 | HV515077.1 | GM040832.1 | DM163980.1 | HC490822.1 |
| DL015383.1 | HC089422.1 | HI002626.1 | DD405398.1 | AX023693.1 | HV533134.1 | GM840288.1 | DM163945.1 | HC490790.1 |
| DL015351.1 | HC089389.1 | HI002592.1 | DD405366.1 | AX023643.1 | HV536614.1 | CS728630.1 | DM163539.1 | HC490726.1 |
| DL015319.1 | HC089357.1 | HI002531.1 | DD405334.1 | AX023610.1 | HV536326.1 | CS728534.1 | DM163275.1 | HC490694.1 |
| DL015287.1 | HC089324.1 | HI001483.1 | DD405302.1 | AX023577.1 | HV534621.1 | CS727342.1 | DM162559.1 | HC490530.1 |
| DL015255.1 | HC089292.1 | HI004547.1 | DD405270.1 | AX021717.1 | HV532503.1 | DD491128.1 | HH736035.1 | HC490498.1 |
| DL010797.1 | GN366317.1 | HI004419.1 | DD405238.1 | AX019969.1 | HV537436.1 | CS671006.1 | HH735916.1 | HC490266.1 |
| DL010765.1 | GN366285.1 | HI002398.1 | DD401636.1 | AX019754.1 | HV535886.1 | CS673573.1 | HH733593.1 | HC472255.1 |
| DL010733.1 | GN359846.1 | HI002318.1 | DD401604.1 | AX002863.1 | HV537352.1 | CS646211.1 | HD119602.1 | HC472223.1 |
| DL010701.1 | GN359814.1 | HI000521.1 | DD401572.1 | AX010932.1 | HV537251.1 | CS645464.1 | FW368813.1 | HC472191.1 |
| DL010669.1 | GN359750.1 | HD121593.1 | DD401540.1 | AX010240.1 | HV535451.1 | CS645127.1 | FW368459.1 | HC471959.1 |
| DL010637.1 | GN359666.1 | HD114100.1 | DD401508.1 | AX008952.1 | HV528237.1 | DD460078.1 | FW367555.1 | HC471927.1 |
| DL010605.1 | GN359570.1 | HD082431.1 | DD401476.1 | AX008152.1 | HV515592.1 | DD453837.1 | FW367125.1 | HC471895.1 |
| DL047029.1 | GN359506.1 | HD082299.1 | CS480617.1 | AX006873.1 | HV515560.1 | DD453492.1 | FW362659.1 | HC678813.1 |
| DL046997.1 | GN365271.1 | HD088605.1 | CS479593.1 | AX006188.1 | HV515528.1 | DD456100.1 | FW366592.1 | HC504673.1 |
| DL046965.1 | GN359269.1 | HD077384.1 | CS476723.1 | M58162.1 | HV515496.1 | CS631740.1 | FW366207.1 | HC471017.1 |
| DL046933.1 | GN359205.1 | HD070399.1 | CS469228.1 | A57345.1 | HV515464.1 | CS642687.1 | FW351389.1 | HC502262.1 |
| DL046901.1 | GN359173.1 | HD069940.1 | CS467835.1 | A38670.1 | HV515432.1 | CS631242.1 | FW351183.1 | HC500964.1 |
| DL046869.1 | GN364166.1 | FW345122.1 | CS467591.1 | A35732.1 | HV515400.1 | CS631178.1 | FW366031.1 | HC499891.1 |
| DL043047.1 | GN363359.1 | FW344980.1 | CS467516.1 | A35524.1 | HV515368.1 | CS627813.1 | FW351017.1 | HC499850.1 |
| DL043015.1 | GN346508.1 | FW344735.1 | CS466954.1 | A29487.1 | HV515336.1 | CS632190.1 | FW360647.1 | FW303070.1 |

| | | | | | | | | |
|---|---|---|---|---|---|---|---|---|
| DL042983.1 | GN346474.1 | FW343968.1 | CS464637.1 | A35018.1 | HV515304.1 | CS632096.1 | FW349999.1 | FW301870.1 |
| DL042951.1 | GN348297.1 | FW343612.1 | CS464555.1 | FW351167.1 | HV515272.1 | CS626240.1 | HC472302.1 | FW298751.1 |
| DL042919.1 | GN346828.1 | FW343548.1 | CS464125.1 | FW361583.1 | HV515240.1 | DD438339.1 | HC472269.1 | FW298082.1 |
| DL042887.1 | GN346581.1 | FW343423.1 | DD392063.1 | HD122371.1 | HV511362.1 | DD438013.1 | HC472237.1 | HB475486.1 |
| DL039070.1 | GN346549.1 | FW342969.1 | DD391751.1 | HD115797.1 | HV511256.1 | DD449070.1 | HC472205.1 | HB475015.1 |
| DL039038.1 | GN131888.1 | HC920699.1 | DD391865.1 | HD084549.1 | HV511013.1 | DD435327.1 | HC472173.1 | HB474749.1 |
| AX240974.1 | GN116496.1 | HD068761.1 | DD391740.1 | HD082408.1 | HV514127.1 | CS617228.1 | HC471909.1 | HB469129.1 |
| AX240942.1 | GN116463.1 | HD067801.1 | DD391630.1 | HD081915.1 | HV510412.1 | CS616570.1 | HC471877.1 | DM152631.1 |
| AX240910.1 | GN116431.1 | HD066577.1 | DD385337.1 | HD088205.1 | HV513405.1 | E03094.1 | HC678841.1 | HB461475.1 |
| AX240108.1 | GN116399.1 | HD063960.1 | DD382899.1 | HD071342.1 | HV510363.1 | E02963.1 | HC678776.1 | HB447905.1 |
| AX239715.1 | GN116367.1 | HD063549.1 | DD400166.1 | FW345362.1 | HV510330.1 | E02692.1 | HC509929.1 | HB441215.1 |
| AX235902.1 | GN116335.1 | HD052880.1 | DD400134.1 | FW345034.1 | HV510266.1 | E02269.1 | HC504592.1 | GN088024.1 |
| AX235258.1 | GN116303.1 | HC920317.1 | DD382119.1 | FW345002.1 | HV510070.1 | E00952.1 | HC494420.1 | L09144.1 |
| AX234580.1 | GN095151.1 | HD033291.1 | DD393794.1 | FW344679.1 | HV513369.1 | DD101030.1 | HC502592.1 | L08927.1 |
| AX225274.1 | GN094510.1 | HD065423.1 | CS463246.1 | FW343724.1 | HV513273.1 | DD112416.1 | HC500311.1 | M88477.1 |
| AX214409.1 | GN094478.1 | HD058880.1 | CS461227.1 | FW343592.1 | HV513241.1 | DD142884.1 | HC499865.1 | DM059606.1 |
| AF408179.1 | GM659802.1 | HD057689.1 | DD137311.1 | FW343528.1 | HV509842.1 | DD084275.1 | FW301502.1 | DM058892.1 |
| AX207302.1 | GM659770.1 | HD057657.1 | DD136473.1 | HC922995.1 | HV509294.1 | DD152062.1 | FW300961.1 | DM058815.1 |
| AX205196.1 | GM659738.1 | HD062475.1 | DD135768.1 | HC921522.1 | HV508667.1 | DD082059.1 | FW298769.1 | DM045498.1 |
| AX202563.1 | GM659702.1 | HD061138.1 | DD135335.1 | HC920517.1 | HV508632.1 | DD081770.1 | HC488036.1 | DM045337.1 |
| AX202434.1 | GM659670.1 | M26974.1 | DD134024.1 | HD068872.1 | HV508568.1 | DD147803.1 | HC477800.1 | DM045084.1 |
| AX201440.1 | GM659638.1 | M17215.1 | DD133029.1 | HD065541.1 | HV508536.1 | DD147771.1 | HC486515.1 | DM044876.1 |
| AX193964.1 | GM659606.1 | HC324501.1 | DD132294.1 | HD063942.1 | HV508504.1 | DD147739.1 | HC486446.1 | DM039594.1 |
| AX191641.1 | GM652317.1 | HC315507.1 | DD107153.1 | HB469137.1 | HV508476.1 | GN030900.1 | HC475383.1 | DM044760.1 |
| HW344084.1 | GM652285.1 | HC313697.1 | DD093919.1 | HB468764.1 | HV508402.1 | GN030868.1 | HC474962.1 | GN067942.1 |
| HW353668.1 | GM645390.1 | HC319270.1 | DD093887.1 | DM148863.1 | HV512278.1 | GN030804.1 | HC466606.1 | GN075431.1 |
| HW353572.1 | GM645358.1 | HC318727.1 | DD093855.1 | DM152635.1 | HV512246.1 | DM005047.1 | HC466281.1 | GN067753.1 |
| HW353540.1 | GM645294.1 | HC310064.1 | DD092968.1 | HB461487.1 | HV512214.1 | DM004977.1 | HC491793.1 | GN052370.1 |
| HW353508.1 | GM645262.1 | AY658636.1 | DD092351.1 | HB455479.1 | HV512150.1 | DM003478.1 | HC491761.1 | GN059770.1 |
| HW344027.1 | GM645230.1 | AY658604.1 | DD119266.1 | HB454950.1 | HV512118.1 | GM997864.1 | HC491729.1 | DM023752.1 |
| HW353473.1 | GM659376.1 | AY658572.1 | DD091560.1 | HB445183.1 | HV512076.1 | GM996599.1 | HC481952.1 | DM016036.1 |
| HW339217.1 | GM652152.1 | AY658540.1 | DD061430.1 | DM138187.1 | HV511839.1 | GN009693.1 | HC481593.1 | GM741825.1 |
| HW352852.1 | GM645197.1 | AY658508.1 | DD118656.1 | DM121636.1 | JA660238.1 | GM994612.1 | FV531716.1 | GM658307.1 |
| HW352404.1 | GM645165.1 | AY658476.1 | DD091313.1 | HB441219.1 | HV492190.1 | GM993551.1 | FV531684.1 | GM658275.1 |
| HW341673.1 | GM645133.1 | AY658444.1 | DD061340.1 | HB434952.1 | HV504958.1 | GM992502.1 | HB338914.1 | GM658243.1 |
| HW341540.1 | GM645101.1 | AY658348.1 | DD061284.1 | HB432423.1 | HV504926.1 | GM992212.1 | HA643288.1 | GM658211.1 |
| HW341302.1 | GM645069.1 | AY658316.1 | DD061234.1 | HB423264.1 | HV504894.1 | GM992052.1 | HA642069.1 | GM641434.1 |
| HW351625.1 | GM645037.1 | AY658284.1 | DD118415.1 | HB416484.1 | HV504862.1 | GM989816.1 | HA635317.1 | GM641384.1 |
| HW341244.1 | GM638110.1 | AY658252.1 | DD091165.1 | HB394801.1 | HV504830.1 | GN000745.1 | HA639790.1 | GM641352.1 |
| HW351387.1 | GM638078.1 | AY658220.1 | DD118112.1 | GN031617.1 | HV504798.1 | DL101951.1 | DM110296.1 | GM629065.1 |
| HW351192.1 | GM638036.1 | AY658188.1 | AX145161.1 | GN031585.1 | HV504781.1 | DL115891.1 | DM105621.1 | GM629033.1 |
| HW350987.1 | GM633468.1 | AY658156.1 | AX145129.1 | GN031553.1 | HV504749.1 | DL111273.1 | DM109948.1 | GM629001.1 |
| HW350949.1 | GM633404.1 | AY658124.1 | AX145097.1 | GN031521.1 | HI641754.1 | DL115150.1 | DM100522.1 | GM638808.1 |
| HW340495.1 | GM633372.1 | AY658092.1 | AX145065.1 | GN031489.1 | HI641650.1 | DL088945.1 | DM103444.1 | GM638776.1 |
| HW350874.1 | GM633340.1 | AY658060.1 | AX145033.1 | GN031424.1 | HI641534.1 | DL037176.1 | DM102931.1 | GM638744.1 |
| HW350656.1 | GM633308.1 | AY658028.1 | AX145001.1 | GN031392.1 | HI641430.1 | DL043280.1 | GN368736.1 | HV694795.1 |
| HW350357.1 | GM633276.1 | AY657996.1 | HW321931.1 | GN031360.1 | HI641314.1 | DJ066261.1 | GN368415.1 | FZ421509.1 |

| | | | | | | | | |
|---|---|---|---|---|---|---|---|---|
| HW350288.1 | GM633221.1 | AY657964.1 | HW336966.1 | GN031328.1 | HI641210.1 | DJ065243.1 | GN367740.1 | FZ421409.1 |
| HW339569.1 | GM633189.1 | AY657932.1 | HW336838.1 | GN031295.1 | HI641106.1 | DJ055667.1 | GN367584.1 | FZ421032.1 |
| HW349604.1 | GM633157.1 | AY657900.1 | HW321680.1 | GN031263.1 | HI640990.1 | DJ055045.1 | GN367543.1 | FZ417510.1 |
| HW348275.1 | GM633125.1 | AY657868.1 | HW336682.1 | GN031231.1 | HI640886.1 | DJ054322.1 | GN373316.1 | FZ411467.1 |
| HW347803.1 | GM633093.1 | AY657836.1 | HW321216.1 | GN031199.1 | HI640770.1 | CS810549.1 | GN370256.1 | FZ413939.1 |
| HW347739.1 | GM625386.1 | AY657804.1 | HW336474.1 | GN031134.1 | HI640666.1 | FB292270.1 | GN360158.1 | FZ419842.1 |
| HW347707.1 | GM625354.1 | AY657772.1 | HW329654.1 | GN031102.1 | HI640562.1 | CS811176.1 | GN360126.1 | FZ419808.1 |
| HW347675.1 | GM659130.1 | AY657740.1 | HW320834.1 | GN031070.1 | HI640446.1 | DD468099.1 | GN356163.1 | FW590748.1 |
| HW347643.1 | GM648903.1 | AY657708.1 | HW329375.1 | GN031038.1 | HI639902.1 | DJ019847.1 | GN359989.1 | FW592738.1 |
| HW347610.1 | GM648871.1 | AY657676.1 | HW320675.1 | GN031006.1 | HI638010.1 | CS803538.1 | GN359893.1 | FW577213.1 |
| HW347578.1 | GM648839.1 | AY657644.1 | HW335930.1 | GN030974.1 | HI637633.1 | CS803346.1 | GN366319.1 | FW576090.1 |
| HW347510.1 | GM648807.1 | AY657612.1 | HW328825.1 | GN030911.1 | HI380350.1 | FB295422.1 | GN366287.1 | HI378116.1 |
| HW347482.1 | GM634656.1 | AY657580.1 | HW328607.1 | GN030847.1 | HI379154.1 | DJ052354.1 | GM043757.1 | HI375907.1 |
| HW347450.1 | GM634624.1 | AY657548.1 | HW328459.1 | GN030815.1 | HI378748.1 | DJ045852.1 | DL465746.1 | HI369147.1 |
| HW363655.1 | GM634592.1 | AY657516.1 | HW328362.1 | GN030783.1 | HI378716.1 | DJ049387.1 | DL464721.1 | HI369094.1 |
| HW363624.1 | GM634560.1 | AY657484.1 | HW328300.1 | DM008341.1 | HC310040.1 | DJ048784.1 | DL464552.1 | HI424338.1 |
| HW347331.1 | GM634528.1 | AY657452.1 | HW319069.1 | DM001668.1 | HC309976.1 | CS800126.1 | DL463787.1 | HI424122.1 |
| HW158527.1 | GM634496.1 | AY657420.1 | HW335665.1 | DM006936.1 | HC308991.1 | CS800000.1 | DL470917.1 | HI424058.1 |
| HW158398.1 | GM629691.1 | AY657388.1 | HW328241.1 | DM005042.1 | HC307874.1 | CS707358.1 | DL476972.1 | HI423832.1 |
| HW154652.1 | GM664698.1 | AY657356.1 | HW328097.1 | DM003489.1 | HC307805.1 | CS716027.1 | DL463072.1 | HI422642.1 |
| HW154485.1 | DD090298.1 | AY657324.1 | HW318509.1 | GM996193.1 | HC307382.1 | CS713109.1 | DL476425.1 | HI416741.1 |
| HW154453.1 | DD103430.1 | AY657292.1 | HW335151.1 | GM995276.1 | HC312266.1 | CS675414.1 | DL462978.1 | HI414058.1 |
| HW154414.1 | DD103248.1 | AY657260.1 | HW314324.1 | GN009991.1 | FU258245.1 | DD132508.1 | DL469778.1 | HI414025.1 |
| HW147443.1 | DD057937.1 | AY657228.1 | HW312137.1 | GM991945.1 | FU258213.1 | DD158423.1 | DL475570.1 | HI413616.1 |
| HW154319.1 | DD057905.1 | AY657196.1 | HW312080.1 | GM976376.1 | FU258149.1 | DD155251.1 | DL481629.1 | HI516561.1 |
| HW147142.1 | DD057873.1 | AY657164.1 | HW311847.1 | GM741187.1 | FU258093.1 | DD152378.1 | DL460978.1 | HI548054.1 |
| HW154029.1 | DD167018.1 | AY657132.1 | HW311292.1 | GM657799.1 | FU260736.1 | DD152110.1 | GM832157.1 | HI516100.1 |
| HW153728.1 | DD172931.1 | AY657100.1 | HW311169.1 | GM657766.1 | FU257905.1 | DD139896.1 | GM712167.1 | HI284287.1 |
| HW145787.1 | DD159952.1 | AY657068.1 | HW309502.1 | GM643112.1 | FU257841.1 | DD082056.1 | GM831164.1 | HI574508.1 |
| HW145522.1 | DD165143.1 | AY657036.1 | HW309434.1 | GM643080.1 | FU257712.1 | DD081767.1 | GM675545.1 | HI574476.1 |
| HW145145.1 | DD171107.1 | J02552.1 | HW309379.1 | GM643048.1 | FU271878.1 | DD147800.1 | FB509357.1 | HI574444.1 |
| HW153367.1 | DD163600.1 | J02559.1 | HW307876.1 | GM643016.1 | FU262708.1 | DD147768.1 | FB509323.1 | HI574412.1 |
| HW153269.1 | AF405701.1 | M29236.1 | HW307844.1 | GM662968.1 | FU262667.1 | DD147736.1 | FB509259.1 | HI574380.1 |
| HW152951.1 | CS160259.1 | M74313.1 | HW307812.1 | GM648351.1 | FU250483.1 | DD147704.1 | FB509148.1 | FW307721.1 |
| HW151771.1 | CS157793.1 | M12533.1 | HW307780.1 | GM648319.1 | FU262467.1 | DD147672.1 | GM772974.1 | FW332333.1 |
| HW150560.1 | CS157954.1 | M19974.1 | HW316592.1 | GM648287.1 | FU267900.1 | DD139283.1 | GM709013.1 | FW306357.1 |
| HW150528.1 | CQ975459.1 | HI369108.1 | HW307742.1 | GM648255.1 | FU264931.1 | DD080965.1 | GM708981.1 | FW305536.1 |
| HW150496.1 | CQ973480.1 | HH772203.1 | HW307710.1 | GM648223.1 | FU262196.1 | DD137698.1 | CS406189.1 | FW310640.1 |
| HW150464.1 | CQ979573.1 | HI424136.1 | HW315647.1 | GM641388.1 | FU262076.1 | DD133061.1 | CS406593.1 | HC767599.1 |
| HW150400.1 | CQ976610.1 | HI424104.1 | HW315570.1 | GM641356.1 | FU269788.1 | DD132297.1 | CS403494.1 | HC767365.1 |
| HW150368.1 | CQ975999.1 | HI423846.1 | HW315537.1 | GM641324.1 | FU258433.1 | DD080704.1 | CS402930.1 | AF491940.1 |
| HW144671.1 | CQ972489.1 | HI423814.1 | HW315145.1 | GM641292.1 | FU258369.1 | DD107482.1 | CS402067.1 | HC754642.1 |
| HW144631.1 | CQ972393.1 | HI423782.1 | HW315113.1 | GM641260.1 | FU258337.1 | DD092357.1 | CS401822.1 | HC732130.1 |
| HW126565.1 | CQ972361.1 | HH762712.1 | HW315081.1 | GM641228.1 | HC313147.1 | DD089914.1 | CS382110.1 | HC731620.1 |
| HW125551.1 | CQ972329.1 | HI416148.1 | HW314959.1 | GM629069.1 | DM475258.1 | DD090066.1 | CS380093.1 | HC471757.1 |
| HW144352.1 | CQ971122.1 | HI415881.1 | HW314911.1 | GM629037.1 | DM474082.1 | DD052042.1 | CS382613.1 | HC471661.1 |
| HW144320.1 | CQ970843.1 | HI415816.1 | HW314850.1 | GM629005.1 | HC306257.1 | DD057920.1 | CS376547.1 | HC471629.1 |

| | | | | | | | | |
|---|---|---|---|---|---|---|---|---|
| HW144288.1 | CQ947165.1 | HI415752.1 | HW314794.1 | GM880294.1 | HC306217.1 | DD057888.1 | DD290971.1 | HC731040.1 |
| HW124834.1 | CQ947120.1 | HI414039.1 | HW314652.1 | GM648190.1 | HC306177.1 | CQ787428.1 | DD053303.1 | HC727361.1 |
| HW124692.1 | CQ945505.1 | HI414002.1 | HW160797.1 | GM628699.1 | HC302627.1 | CQ786543.1 | DD052287.1 | HC689104.1 |
| HW124603.1 | CQ944190.1 | HI413443.1 | HW106140.1 | GM716009.1 | HC305949.1 | CQ784683.1 | DD052229.1 | HC688474.1 |
| HW123473.1 | CQ944158.1 | HI516597.1 | HW115436.1 | GM662138.1 | HC305909.1 | CQ778514.1 | DD059291.1 | HC688411.1 |
| HW122341.1 | CQ944126.1 | HI516521.1 | HW114529.1 | GM640904.1 | HC305869.1 | CQ774657.1 | DD058546.1 | HC687678.1 |
| HW121254.1 | CQ944094.1 | HI516114.1 | HW112706.1 | GM640872.1 | HC305829.1 | CQ771553.1 | DD041941.1 | HC490853.1 |
| HW083239.1 | CQ944062.1 | HI516082.1 | HW112625.1 | DL114088.1 | HC305789.1 | CQ756685.1 | DD046707.1 | HC490821.1 |
| HW083207.1 | CQ944030.1 | HI547684.1 | HW112548.1 | DL114056.1 | HC305749.1 | CQ754050.1 | DD042878.1 | HC490725.1 |
| HW097477.1 | CQ943998.1 | HI547612.1 | HW112465.1 | DL126228.1 | HC305709.1 | AX963308.1 | DD030010.1 | HC490693.1 |
| HW097349.1 | CQ943966.1 | HI284301.1 | HV984515.1 | DL126196.1 | HC305669.1 | AX962888.1 | DD037273.1 | HC490529.1 |
| HW081872.1 | CQ943934.1 | HI571690.1 | HV986642.1 | DL126164.1 | HC305629.1 | AX958747.1 | DD030671.1 | HC490497.1 |
| HW081720.1 | CQ943902.1 | HI574522.1 | HV965178.1 | DL122948.1 | HC305370.1 | AX938913.1 | DD026764.1 | HB461467.1 |
| HW081626.1 | CQ943870.1 | HI574490.1 | HV961282.1 | DL122916.1 | HC301366.1 | AX937437.1 | DD023826.1 | HB454850.1 |
| HW081590.1 | CQ904363.1 | HI574458.1 | HV969325.1 | DL099549.1 | HC301030.1 | AX927078.1 | DD023181.1 | HB451966.1 |
| HW103541.1 | CQ903051.1 | HI574426.1 | HV960808.1 | DL122684.1 | HC254833.1 | AX923418.1 | DD029433.1 | HB463487.1 |
| HW103429.1 | A06171.1 | HI574394.1 | HV960573.1 | DL122652.1 | HC291498.1 | AX923385.1 | DD028785.1 | DM141119.1 |
| HW102878.1 | CQ898612.1 | HI553789.1 | HV960281.1 | DL122588.1 | HC294350.1 | AX832723.1 | DD010447.1 | HB435574.1 |
| HW061941.1 | CQ898580.1 | HI593060.1 | HV959934.1 | DL028622.1 | HC291222.1 | AX824349.1 | DD013401.1 | HB433979.1 |
| HW061862.1 | CQ898505.1 | HI637150.1 | HV959872.1 | DL028558.1 | HC289356.1 | AX268993.1 | BD495443.1 | HB423193.1 |
| HW061830.1 | CQ897880.1 | HI636967.1 | HV964048.1 | DL048677.1 | HC289324.1 | AX259242.1 | BD453897.1 | HB403659.1 |
| HW059113.1 | CQ895554.1 | HI001448.1 | HV963870.1 | DL016633.1 | HC289186.1 | AX304427.1 | BD453865.1 | HB394261.1 |
| HW062177.1 | CQ895454.1 | HI001389.1 | HV958688.1 | DL039637.1 | GN359183.1 | AX303189.1 | BD453833.1 | HB394229.1 |
| HW061784.1 | CQ893701.1 | HI001352.1 | HV963678.1 | DL039605.1 | GN359151.1 | AY043010.1 | BD453801.1 | HB397697.1 |
| HW061750.1 | CQ891882.1 | HI001318.1 | HV966193.1 | DL023551.1 | GN359119.1 | AX279799.1 | BD443473.1 | DM115549.1 |
| HW058575.1 | CQ890965.1 | HI003201.1 | HV958150.1 | DL023519.1 | GN359087.1 | AX254829.1 | BD453745.1 | DM114851.1 |
| HW065310.1 | CQ890048.1 | HI003139.1 | HV965989.1 | DL023487.1 | GN363931.1 | AX253171.1 | BD453713.1 | HB389714.1 |
| HW065212.1 | CQ888121.1 | HI003083.1 | HV965957.1 | DL023455.1 | GN359057.1 | AX251579.1 | BD453681.1 | GN368627.1 |
| HW065102.1 | CQ877780.1 | HI003043.1 | HV965487.1 | DL023423.1 | GN358962.1 | AX249919.1 | BD453649.1 | DM000223.1 |
| HW064886.1 | CQ877137.1 | HI003002.1 | HV962274.1 | DL020376.1 | GN363042.1 | DM162216.1 | BD453617.1 | DM005065.1 |
| HW064846.1 | CQ875533.1 | HI002903.1 | HV962206.1 | DL020344.1 | GN362731.1 | HB484356.1 | BD453580.1 | DM004983.1 |
| HW061083.1 | CQ874986.1 | HI001266.1 | HV965428.1 | DL020312.1 | GN362487.1 | HB477471.1 | BD493236.1 | DM003484.1 |
| HW055434.1 | CQ874305.1 | HI001210.1 | HV961791.1 | DL035400.1 | GN346518.1 | HB475735.1 | BD453567.1 | GM996650.1 |
| HW060946.1 | CQ872973.1 | HI001155.1 | HV951632.1 | DL035368.1 | GN346484.1 | GN373340.1 | BD453535.1 | GM996509.1 |
| HW056588.1 | CQ871219.1 | GM627515.1 | HV957875.1 | DL035336.1 | GN344771.1 | GN370763.1 | BD453503.1 | GM993557.1 |
| HW056556.1 | AX258843.1 | GM654135.1 | HV948915.1 | CS603477.1 | GN343719.1 | GN360163.1 | BD453471.1 | GM992517.1 |
| HW056406.1 | AX300959.1 | GM654103.1 | HV947907.1 | CS603445.1 | GN349479.1 | GN360131.1 | BD453439.1 | GM992434.1 |
| HW064742.1 | AX288129.1 | GM654071.1 | HV947297.1 | CS603413.1 | GN346591.1 | GN356168.1 | BD453407.1 | GM992364.1 |
| HW064669.1 | AX286672.1 | GM654007.1 | HV952897.1 | CS608407.1 | GN346559.1 | GN366510.1 | BD453262.1 | GM992294.1 |
| HW064633.1 | AX282342.1 | GM653975.1 | HV951948.1 | CS607557.1 | GN340527.1 | GN366478.1 | BD453230.1 | GM976370.1 |
| HW062571.1 | AX281531.1 | GM646982.1 | HV951812.1 | CS607044.1 | GN340248.1 | GN366446.1 | BD453198.1 | GM989960.1 |
| HW056324.1 | AX279942.1 | GM646950.1 | HV946794.1 | CS606984.1 | GN340100.1 | GN366413.1 | BD411260.1 | GM989758.1 |
| HW056238.1 | AX279609.1 | GM646918.1 | HV538615.1 | CS606952.1 | GN339657.1 | GN366381.1 | BD442633.1 | GM987024.1 |
| HW064455.1 | AX278271.1 | GM646886.1 | HV538459.1 | CS606817.1 | GN339179.1 | GN366349.1 | BD432488.1 | GN000751.1 |
| HW062356.1 | AX278069.1 | GM646854.1 | HV537716.1 | CS501493.1 | GN130840.1 | GN360026.1 | BD399490.1 | GM969683.1 |
| HW062260.1 | AX256076.1 | GM646822.1 | HV543304.1 | CS500215.1 | GN335373.1 | GN359994.1 | BD399013.1 | GM969548.1 |
| HW060295.1 | AX253482.1 | GM640019.1 | HC471829.1 | CS498448.1 | GN334578.1 | GN359930.1 | BD497501.1 | GM970628.1 |

| | | | | | | | | |
|---|---|---|---|---|---|---|---|---|
| HW055645.1 | AX252394.1 | GM639987.1 | HC471797.1 | CS495910.1 | GN333938.1 | GN359898.1 | BD451723.1 | GM970097.1 |
| HW042047.1 | AX247554.1 | GM639955.1 | HC679434.1 | DD418601.1 | GN116538.1 | GN366324.1 | BD450795.1 | FB987374.1 |
| HW043499.1 | AX242324.1 | GM639923.1 | HC678986.1 | DD412283.1 | GN116506.1 | GN366292.1 | BD419031.1 | DL465908.1 |
| HW042003.1 | AX242292.1 | GM639891.1 | HC668004.1 | DD411497.1 | GN116473.1 | GN366260.1 | AX717577.1 | GM639413.1 |
| HW041971.1 | AX242260.1 | GM627285.1 | HC510273.1 | DD417800.1 | GN116441.1 | GN359853.1 | AX699460.1 | FV531698.1 |
| HV514108.1 | AX242228.1 | GM627253.1 | HC508644.1 | CS492171.1 | GN116409.1 | GN359693.1 | AX699428.1 | FV530905.1 |
| HV510411.1 | AX242196.1 | GM627221.1 | HC504659.1 | CS492959.1 | GN116345.1 | GN359641.1 | AX685575.1 | FV522836.1 |
| HV510362.1 | AX242132.1 | GM627189.1 | HC504602.1 | CS490556.1 | GN116313.1 | GN359513.1 | AX684664.1 | FV522796.1 |
| HV510297.1 | AX241972.1 | DL121468.1 | HC494563.1 | CS488845.1 | GN115556.1 | GN359445.1 | AX683936.1 | FV522613.1 |
| HV513368.1 | AX241940.1 | DL121436.1 | HC493111.1 | CS486433.1 | GN112657.1 | GN359317.1 | AX675249.1 | FV534228.1 |
| HV513336.1 | AX241908.1 | DL121404.1 | HC504198.1 | DD401425.1 | GN114941.1 | GN359285.1 | AX664342.1 | AY125353.1 |
| HV513272.1 | AX241844.1 | DL117128.1 | HC501817.1 | DD405176.1 | GN094585.1 | GN365284.1 | AX662221.1 | HC453695.1 |
| HV509836.1 | AX241812.1 | DL102497.1 | HC500844.1 | DD405144.1 | GN094552.1 | GN364297.1 | AX661181.1 | HC453663.1 |
| HV509293.1 | AX241780.1 | DL102433.1 | HC499872.1 | DD405112.1 | GN094520.1 | GN359276.1 | AX659104.1 | AF029884.1 |
| HV508666.1 | AX241716.1 | DL102401.1 | FW302606.1 | DD402423.1 | GN094488.1 | GN359244.1 | AX658712.1 | HB855455.1 |
| HV508631.1 | AX241652.1 | DL102369.1 | FW301619.1 | DD402391.1 | GN094456.1 | GN359212.1 | AX657142.1 | DM173882.1 |
| HV508599.1 | AX241620.1 | DL102337.1 | FW301194.1 | DD402359.1 | GN113552.1 | GN359180.1 | AX657110.1 | HB559428.1 |
| HV508567.1 | AX241588.1 | DL097628.1 | FW299456.1 | DD402327.1 | DM065368.1 | GN359148.1 | AX657066.1 | HB491546.1 |
| HV508535.1 | AX241556.1 | DL097596.1 | FW299341.1 | DD402295.1 | DM065002.1 | GN359116.1 | BD176077.1 | HB488921.1 |
| HV508500.1 | AX241524.1 | DL097564.1 | FW299309.1 | DD402263.1 | DM064087.1 | GN358849.1 | BD175968.1 | DM165789.1 |
| HV508475.1 | AX241492.1 | DL095537.1 | FW299277.1 | DD402231.1 | DM070478.1 | GN362484.1 | BD174659.1 | DM164040.1 |
| HV512418.1 | AX241428.1 | DL095505.1 | FW299245.1 | DD402199.1 | DM475256.1 | GN346515.1 | AX648075.1 | DM163990.1 |
| HV512277.1 | AX241121.1 | DL095409.1 | FW298787.1 | DD405611.1 | DM465927.1 | GN346481.1 | AX643802.1 | DM163957.1 |
| HV512245.1 | AX241089.1 | DL095377.1 | HC469955.1 | DD405579.1 | HC299796.1 | GN344168.1 | AX642216.1 | HB475831.1 |
| HV512213.1 | AX241057.1 | DL095345.1 | HC469467.1 | DD405515.1 | HC306254.1 | GN346901.1 | AX616533.1 | GN090778.1 |
| HV512149.1 | AX241025.1 | DL091467.1 | HC469039.1 | DD405483.1 | HC306214.1 | GN346588.1 | AX601618.1 | GN089889.1 |
| HV512117.1 | HW314827.1 | DL087510.1 | HC486610.1 | DD401981.1 | HC306174.1 | GN346556.1 | AX601522.1 | GN089824.1 |
| HV492189.1 | HW314717.1 | DL087478.1 | HC486526.1 | DD401920.1 | HC302625.1 | GN340523.1 | AX601482.1 | GN082804.1 |
| HV504957.1 | HW069843.1 | DL087414.1 | HC486460.1 | E00693.1 | HC305947.1 | GN340245.1 | AX601321.1 | GN082361.1 |
| HV504925.1 | HW084939.1 | DL112256.1 | HC475390.1 | E00283.1 | HC305907.1 | GN339925.1 | AX598818.1 | GN079249.1 |
| HV504893.1 | HW097733.1 | DL112224.1 | HC474969.1 | DD087672.1 | HC305867.1 | GN339112.1 | AX594178.1 | L09140.1 |
| HV504829.1 | HW097689.1 | DL112192.1 | HC466645.1 | DD101244.1 | HC305827.1 | GN337038.1 | AX593519.1 | L08916.1 |
| HV504797.1 | HW064974.1 | DL112160.1 | DM185696.1 | DD144778.1 | HC305787.1 | GN130792.1 | AX587941.1 | M80356.1 |
| HV504780.1 | HW058258.1 | DL112128.1 | DM170651.1 | DD069497.1 | HC305747.1 | GN335366.1 | AX587840.1 | DM059588.1 |
| HV504748.1 | HW046277.1 | DL112096.1 | DM187983.1 | DD117672.1 | HC305707.1 | GN116535.1 | AX587745.1 | DM058883.1 |
| HV504716.1 | HV956361.1 | DL112064.1 | DM169886.1 | DD158390.1 | HC305667.1 | GN116503.1 | AX587542.1 | DM058807.1 |
| HV504684.1 | HV951104.1 | DL107061.1 | DM173758.1 | DD157371.1 | HC305627.1 | GN042720.1 | AX592801.1 | DM057736.1 |
| HV504652.1 | HV949989.1 | DL107029.1 | DM176978.1 | DD152121.1 | HC305222.1 | GN041681.1 | AX014746.1 | DM048649.1 |
| HV504620.1 | HV953058.1 | DL106997.1 | HB492054.1 | DD083026.1 | HC301316.1 | GN037323.1 | AX012309.1 | DM045309.1 |
| HV491625.1 | HV946243.1 | DL106965.1 | HB491539.1 | DD139584.1 | GN359318.1 | GN034791.1 | AX011475.1 | DM045032.1 |
| HV504971.1 | HV944608.1 | DL106933.1 | HB488833.1 | DD147809.1 | GN359286.1 | GN030750.1 | AX011443.1 | DM044735.1 |
| HV504565.1 | HV943099.1 | DL106901.1 | HB488782.1 | DD147777.1 | GN350107.1 | GN030718.1 | AX011411.1 | GN067946.1 |
| HV504533.1 | HV779876.1 | DL102216.1 | HB488702.1 | DD147745.1 | GN365287.1 | GN030686.1 | AX011378.1 | GN067937.1 |
| HV504501.1 | HV774716.1 | DL102160.1 | DM164031.1 | DD147713.1 | GN364298.1 | GN030654.1 | HI380324.1 | GN067873.1 |
| HV504469.1 | HV773779.1 | DL102128.1 | DM164016.1 | DD147681.1 | GN359277.1 | GN030622.1 | HI376933.1 | GN052366.1 |
| HV504437.1 | HW118005.1 | DL116808.1 | DM163983.1 | DD139313.1 | GN359245.1 | GN030590.1 | HI376223.1 | GN059762.1 |
| HV504405.1 | HW106667.1 | DL116776.1 | DM163949.1 | DD122281.1 | GN359213.1 | GN030427.1 | HI375921.1 | GN051231.1 |

| | | | | | | | | |
|---|---|---|---|---|---|---|---|---|
| HV504373.1 | HW115645.1 | DL116744.1 | DM163541.1 | DD138525.1 | GN359181.1 | GN030439.1 | HI372213.1 | GN046514.1 |
| HV504341.1 | HW113650.1 | DL111927.1 | DM163278.1 | DD137181.1 | GN359149.1 | GN030366.1 | HI370982.1 | GN046076.1 |
| HV504309.1 | HW081504.1 | DL106860.1 | DM162218.1 | DD135987.1 | GN359117.1 | GN030334.1 | HH833510.1 | DL233393.1 |
| HV504277.1 | HW087582.1 | DL106828.1 | DM161494.1 | DD132320.1 | GN359085.1 | GN030238.1 | HH833478.1 | FB712612.1 |
| HV504245.1 | HW101684.1 | DL106796.1 | HB484272.1 | DD106549.1 | GN363928.1 | GN030174.1 | HH833446.1 | FB708239.1 |
| HV504213.1 | HW097005.1 | DL106764.1 | HB477136.1 | DD092934.1 | GN362485.1 | GN030142.1 | HH833414.1 | FB671024.1 |
| HV455597.1 | HW096748.1 | DL106732.1 | HB477034.1 | CS008047.1 | GN346516.1 | GN030110.1 | HH833382.1 | FB666219.1 |
| HV504177.1 | HW072903.1 | DL106700.1 | HB485587.1 | CQ990422.1 | GN346482.1 | GN030078.1 | FW381655.1 | FB671031.1 |
| HV504145.1 | HW096372.1 | DL089937.1 | HB475226.1 | CQ986586.1 | GN344169.1 | GN030014.1 | HH931885.1 | FB676584.1 |
| HV504113.1 | HW072714.1 | DL089905.1 | GN112656.1 | CQ986554.1 | GN346902.1 | GN029982.1 | HH827426.1 | FB675359.1 |
| HV504081.1 | HW072677.1 | DL089873.1 | GN114940.1 | CQ981108.1 | GN346589.1 | GN029950.1 | HH822331.1 | FB705606.1 |
| HV504049.1 | HW096213.1 | DL089841.1 | GN094584.1 | CQ975429.1 | GN346557.1 | GN029853.1 | HH821972.1 | FB701535.1 |
| HV504017.1 | HW072084.1 | DL089777.1 | GN094551.1 | CQ973470.1 | GN340524.1 | GN013881.1 | FW379738.1 | FB701798.1 |
| HV503953.1 | HW104299.1 | DL137578.1 | GN094519.1 | DL095214.1 | GN339926.1 | GN013381.1 | FW379641.1 | FB292674.1 |
| HV503921.1 | HW071174.1 | DL123133.1 | GN094487.1 | DL128412.1 | GN339124.1 | GN010224.1 | FW379609.1 | FB660205.1 |
| HV503889.1 | HW070943.1 | DL118655.1 | GN094455.1 | DL120271.1 | GN130793.1 | GN033554.1 | FW379263.1 | FB654898.1 |
| HV503857.1 | HW070544.1 | DL118623.1 | GN113551.1 | DL120239.1 | GN334574.1 | GN033522.1 | FW378144.1 | DL109237.1 |
| HV503825.1 | HW099592.1 | DL114227.1 | DM065367.1 | DL120207.1 | GN116536.1 | GN033490.1 | FW381580.1 | DL092279.1 |
| HV501018.1 | HW068972.1 | DL114195.1 | DM064977.1 | DL106190.1 | GN116504.1 | GN033458.1 | FW381109.1 | DL092215.1 |
| HV491139.1 | HW066585.1 | DL114163.1 | DM064086.1 | DL115124.1 | GN116471.1 | GN033426.1 | FW375464.1 | DL092183.1 |
| HV503785.1 | HW083288.1 | DL114131.1 | DM070841.1 | DL110060.1 | GN116439.1 | GN033394.1 | FW369634.1 | DL046826.1 |
| HV503753.1 | HW083166.1 | DL114099.1 | DM070583.1 | DL110028.1 | GN116375.1 | GN033362.1 | FW380257.1 | DL030704.1 |
| HV503721.1 | HV766851.1 | DL114067.1 | DM069112.1 | DL109996.1 | GN116343.1 | GN033298.1 | HH736087.1 | DJ327063.1 |
| HV503689.1 | HV766752.1 | DL126207.1 | DM091333.1 | DL109964.1 | GN116311.1 | GN033266.1 | HH735985.1 | DJ327031.1 |
| HV503625.1 | HV760604.1 | DL126175.1 | DM068820.1 | DL131446.1 | GN112577.1 | GN033234.1 | HH735913.1 | DJ326999.1 |
| HV503597.1 | HV766449.1 | DL122959.1 | GN087810.1 | DL109750.1 | GN112655.1 | GN033170.1 | HH735613.1 | DJ326901.1 |
| HV503533.1 | HV766035.1 | DL122927.1 | GN087209.1 | DL109718.1 | GN115075.1 | GN033106.1 | HC057875.1 | DJ127225.1 |
| HV503501.1 | HV752947.1 | DL122895.1 | GN089874.1 | DL109686.1 | GN094583.1 | GN033074.1 | DM459962.1 | DJ082709.1 |
| HI967439.1 | HV755054.1 | DL122863.1 | GN089810.1 | DL123999.1 | GN094550.1 | GN033042.1 | DM459787.1 | DJ082655.1 |
| HI979667.1 | HV745392.1 | DL122831.1 | GN082900.1 | DL131239.1 | GN094518.1 | GN033010.1 | DM381533.1 | DJ071626.1 |
| HI979304.1 | HV748429.1 | DL122799.1 | GN082829.1 | DL119475.1 | GN094486.1 | GN032978.1 | HC053872.1 | DJ080519.1 |
| HI986185.1 | HV750991.1 | DL122767.1 | GN079366.1 | DL119443.1 | GN094454.1 | GN032946.1 | HC053484.1 | DJ066760.1 |
| HI990090.1 | HV747498.1 | DL114021.1 | GN082001.1 | DL119411.1 | DM074459.1 | GN032914.1 | HC051935.1 | DJ066394.1 |
| HI989106.1 | HV753773.1 | DL113989.1 | GN079083.1 | DL114816.1 | DM065363.1 | GN032850.1 | HC050946.1 | DJ066364.1 |
| HI988404.1 | HV753632.1 | DL113957.1 | DL233318.1 | DL114784.1 | DM064976.1 | GN032818.1 | HC045499.1 | DJ066332.1 |
| HI987439.1 | HV340797.1 | DL113925.1 | DL232595.1 | DL114752.1 | DM064085.1 | GN032786.1 | HC045467.1 | DJ066298.1 |
| HC194692.1 | HV345272.1 | DL113893.1 | GN078437.1 | DL114720.1 | DM070840.1 | GN032723.1 | HC045403.1 | DJ066264.1 |
| FW508691.1 | HV344974.1 | DL113861.1 | GN075927.1 | DL114688.1 | DM070632.1 | GN032691.1 | HC045371.1 | DJ066210.1 |
| FW512093.1 | HV344794.1 | DL109060.1 | GN078785.1 | DL114656.1 | DM070582.1 | GN032659.1 | HC045339.1 | DJ062839.1 |
| FW511921.1 | HV347352.1 | DL109028.1 | L08956.1 | DL127193.1 | DL460984.1 | GN032626.1 | HC045275.1 | DJ065246.1 |
| FW508360.1 | HV347073.1 | AY658506.1 | L08912.1 | DL141575.1 | FB504543.1 | GN032531.1 | HC047455.1 | DJ055685.1 |
| FW508318.1 | HV344710.1 | AY658474.1 | L08787.1 | DJ054368.1 | GM746232.1 | GN032499.1 | HC047423.1 | DJ061661.1 |
| FW508265.1 | HV322833.1 | AY658442.1 | DM059631.1 | DJ056268.1 | GM712173.1 | GN032467.1 | HC047391.1 | DJ061613.1 |
| FW508221.1 | HV322203.1 | AY658410.1 | DM058857.1 | FB291820.1 | GM712086.1 | GN032435.1 | HC047359.1 | DJ061565.1 |
| FW552401.1 | HV321823.1 | AY658378.1 | GM987269.1 | CS810621.1 | GM651995.1 | GN032403.1 | HC047327.1 | DJ055299.1 |
| FW552309.1 | HV325339.1 | AY658346.1 | GM999532.1 | FB292589.1 | GM651963.1 | GN032371.1 | HC047295.1 | DJ057543.1 |
| FW552276.1 | HV325272.1 | AY658314.1 | GM969673.1 | CS812751.1 | GM651931.1 | GN032306.1 | HC047263.1 | DJ054347.1 |

| | | | | | | | | |
|---|---|---|---|---|---|---|---|---|
| FW552238.1 | HV323575.1 | AY658282.1 | GM969401.1 | DD468171.1 | GM831176.1 | GN032274.1 | HC047039.1 | DJ055997.1 |
| FW552179.1 | HV187420.1 | AY658250.1 | GM968444.1 | CS803352.1 | GM711474.1 | GN032242.1 | HC047007.1 | FB291799.1 |
| FW552143.1 | HV201906.1 | AY658218.1 | GM980643.1 | CS802327.1 | GM676154.1 | GN032210.1 | HC046975.1 | CS810618.1 |
| FW552103.1 | HV192771.1 | AY658186.1 | FB986059.1 | DJ047074.1 | FB509365.1 | GN032178.1 | HC046943.1 | FB292586.1 |
| FW558941.1 | HV227378.1 | AY658154.1 | GM970613.1 | DJ050547.1 | FB509329.1 | GN032146.1 | HC046911.1 | DD468128.1 |
| FW563216.1 | HV247750.1 | AY658122.1 | DJ043650.1 | DJ049405.1 | FB509297.1 | GN032114.1 | HC046883.1 | DJ020622.1 |
| FW555606.1 | HV228444.1 | AY658090.1 | DJ043618.1 | CS793878.1 | FB509265.1 | GN032082.1 | HC046851.1 | CS803349.1 |
| FW553309.1 | HV302068.1 | AY658058.1 | CS691625.1 | CS716743.1 | GM675062.1 | GN032050.1 | HC046819.1 | CS802238.1 |
| FW560401.1 | HV035739.1 | AY658026.1 | CS686960.1 | CS716061.1 | FB509218.1 | GN032018.1 | HC046787.1 | BD453881.1 |
| FW563129.1 | FZ427595.1 | AY657994.1 | CS688503.1 | CS796914.1 | FB509116.1 | GN031986.1 | HC046755.1 | BD453849.1 |
| FW558554.1 | FZ435778.1 | AY657962.1 | CS674204.1 | CS806145.1 | GM827558.1 | GN031954.1 | HC046723.1 | BD453817.1 |
| FW557320.1 | FZ430200.1 | AY657930.1 | CS677748.1 | CS714149.1 | FB508989.1 | GN031922.1 | HC046691.1 | BD453785.1 |
| FW565826.1 | FZ436947.1 | AY657898.1 | DJ008410.1 | CS675430.1 | FB508957.1 | GN031890.1 | HC046642.1 | BD433649.1 |
| FW557018.1 | FZ428191.1 | AY657866.1 | DJ011748.1 | CS680679.1 | FB508925.1 | GN031858.1 | HC046610.1 | BD453761.1 |
| FW562718.1 | FZ436103.1 | AY657834.1 | DJ004243.1 | DD401422.1 | GM773243.1 | GN031826.1 | HC046578.1 | BD453729.1 |
| FW560093.1 | FZ416168.1 | AY657802.1 | DJ003146.1 | DD401390.1 | GM709019.1 | GM633491.1 | HC046546.1 | BD453697.1 |
| FW560061.1 | FZ421417.1 | AY657770.1 | DJ010406.1 | DD405205.1 | GM708987.1 | GM625713.1 | HC046514.1 | BD453665.1 |
| FW560029.1 | FZ421069.1 | AY657738.1 | DD495647.1 | DD405173.1 | GM708967.1 | GM625681.1 | GN090935.1 | BD453633.1 |
| FW559964.1 | FZ416909.1 | AY657706.1 | CS670999.1 | DD405141.1 | GM654984.1 | GM625649.1 | GN087193.1 | BD453601.1 |
| FW559924.1 | FW553152.1 | AY657674.1 | DD464401.1 | DD405109.1 | GM694855.1 | GM625617.1 | GN089854.1 | BD453551.1 |
| FW563955.1 | FW556659.1 | AY657642.1 | DD462940.1 | DD404560.1 | GM044486.1 | GM625585.1 | GN089790.1 | BD453519.1 |
| FW559903.1 | FW559379.1 | AY657610.1 | CS499322.1 | DD406601.1 | GM837000.1 | GM625553.1 | GN089192.1 | BD453487.1 |
| FW505290.1 | FW510513.1 | AY657578.1 | CS498533.1 | DD402420.1 | GM835770.1 | GM638432.1 | GN082819.1 | BD453455.1 |
| FW505217.1 | FW510169.1 | AY657546.1 | CS495626.1 | DD402388.1 | GM752857.1 | GM638400.1 | GN088792.1 | BD453423.1 |
| FW505144.1 | HI936415.1 | AY657514.1 | DD413371.1 | DD401978.1 | GM833954.1 | GM638368.1 | GN088509.1 | BD453391.1 |
| FW508125.1 | HI930636.1 | AY657482.1 | DD418805.1 | DD401917.1 | DL467588.1 | GM624334.1 | DL232983.1 | BD453246.1 |
| HI563558.1 | HI935282.1 | AY657450.1 | DD418588.1 | DD401885.1 | GM659954.1 | GM624302.1 | M27939.1 | BD453214.1 |
| HI568947.1 | HI935094.1 | AY657418.1 | DD417839.1 | DD401821.1 | BD242461.1 | GM624270.1 | L08870.1 | BD450237.1 |
| HI583086.1 | HI918291.1 | AY657386.1 | DD411478.1 | DD401549.1 | BD242381.1 | GM652545.1 | L09134.1 | BD408018.1 |
| HI575436.1 | HI918259.1 | AY657354.1 | DD417771.1 | DD401517.1 | BD240791.1 | GM652513.1 | DM063532.1 | BD397192.1 |
| HI551165.1 | HI660935.1 | AY657322.1 | DD414033.1 | DD401485.1 | BD238039.1 | GM652481.1 | DM058841.1 | BD429300.1 |
| HI544151.1 | HI923015.1 | AY657290.1 | CS491857.1 | DD401453.1 | BD237486.1 | GM652449.1 | DM045520.1 | BD428453.1 |
| HI508270.1 | FB730012.1 | AY657258.1 | CS491740.1 | CS479846.1 | BD236844.1 | GM652417.1 | DM045239.1 | BD394874.1 |
| HI585907.1 | GM720760.1 | AY657226.1 | CS490680.1 | CS476144.1 | BD235649.1 | GM652385.1 | DM044942.1 | BD426666.1 |
| HI000014.1 | GM017428.1 | AY657194.1 | CS488274.1 | CS468169.1 | BD234875.1 | GM659905.1 | DM039605.1 | BD453365.1 |
| HI003653.1 | DL112262.1 | AY657162.1 | CS487196.1 | DD082062.1 | BD234566.1 | GM659873.1 | DM044831.1 | BD453358.1 |
| HI003602.1 | DL120563.1 | AY657130.1 | CS486790.1 | DD147806.1 | BD232349.1 | GM659841.1 | DM044664.1 | BD453302.1 |
| HI003569.1 | DL128429.1 | AY657098.1 | CS484403.1 | DD147774.1 | BD232024.1 | GM659809.1 | DM060705.1 | BD453270.1 |
| HI003518.1 | DL096846.1 | AY657066.1 | CS482976.1 | DD147742.1 | DD231584.1 | GM659777.1 | GN067856.1 | BD445339.1 |
| HI001844.1 | DL110163.1 | AY657034.1 | CS482941.1 | DD147710.1 | DD231488.1 | GM659745.1 | GN067792.1 | BD413838.1 |
| HI001784.1 | DL110131.1 | M17338.1 | CS482903.1 | DD147678.1 | DD231412.1 | GM746210.1 | GN065105.1 | BD376114.1 |
| HI001748.1 | DL110099.1 | HI369106.1 | CS482857.1 | DD139310.1 | CS276981.1 | GM659709.1 | GN061109.1 | AX642242.1 |
| HI469625.1 | DL105166.1 | HI425016.1 | CS482816.1 | DD081077.1 | DD219539.1 | GM659677.1 | GN052411.1 | AX642074.1 |
| HI469500.1 | DL105134.1 | HI424134.1 | CS482775.1 | DD137172.1 | DD221329.1 | GM659645.1 | GN059994.1 | AX616677.1 |
| HI508180.1 | DL119950.1 | HI424102.1 | CS482302.1 | DD132317.1 | DD212853.1 | GM659613.1 | GN051732.1 | AX601466.1 |
| HI465604.1 | DL119918.1 | HI424070.1 | CS485113.1 | DD092993.1 | DD214022.1 | GM659581.1 | DM036361.1 | AX600169.1 |
| HI575286.1 | DL119854.1 | HI423844.1 | DD404995.1 | DD090737.1 | DD216643.1 | GM659548.1 | GM645010.1 | AX599992.1 |

| | | | | | | | | |
|---|---|---|---|---|---|---|---|---|
| HI465377.1 | DL119822.1 | HI423001.1 | DD404963.1 | DD117022.1 | DD213880.1 | GM652356.1 | GM644834.1 | AX594155.1 |
| HI001626.1 | DL115131.1 | HH762708.1 | DD404931.1 | DD089920.1 | DD213848.1 | GM652324.1 | GM638035.1 | AX590495.1 |
| HI001584.1 | DL110067.1 | HI415911.1 | DD401444.1 | DD102589.1 | DD213816.1 | GM652292.1 | DL121695.1 | BD160934.1 |
| HI003369.1 | DL110003.1 | HI415847.1 | DD401412.1 | DD057926.1 | DD216449.1 | GM652260.1 | DL121663.1 | AX587461.1 |
| HI003331.1 | DL109971.1 | HI415814.1 | DD401199.1 | DD057894.1 | DD213767.1 | GM645397.1 | DL121599.1 | AX573585.1 |
| HI505601.1 | DL109725.1 | HI415750.1 | DD405195.1 | DD057862.1 | DD213735.1 | GM645365.1 | DL089967.1 | AX573313.1 |
| HI464737.1 | DL104959.1 | HI414037.1 | DD405163.1 | DD057834.1 | DD213703.1 | GM645333.1 | DL089935.1 | AX556839.1 |
| HI464705.1 | DL104927.1 | HI414000.1 | DD405131.1 | DD056934.1 | DD213671.1 | GM645301.1 | DL089903.1 | AX555844.1 |
| HI464673.1 | DL100385.1 | HI413596.1 | DD405099.1 | DD053290.1 | DD213639.1 | GM645269.1 | DL089871.1 | AX555215.1 |
| HI464641.1 | DL100353.1 | HI413441.1 | DD405067.1 | DD051869.1 | DD213607.1 | GM645237.1 | DL089839.1 | AX555098.1 |
| HI464078.1 | DL124134.1 | HI412337.1 | DD405035.1 | DD041851.1 | DD213589.1 | GM638439.1 | DL089775.1 | AX546500.1 |
| HI549592.1 | DL124102.1 | HI516514.1 | DD404685.1 | CQ755435.1 | DD213525.1 | GM659383.1 | DL123131.1 | AX543905.1 |
| HI504387.1 | DL119482.1 | HI516112.1 | DD407072.1 | CQ753997.1 | DD216037.1 | GM659351.1 | DL118685.1 | AX539584.1 |
| HI463294.1 | DL119450.1 | HI516080.1 | DD403971.1 | AX958969.1 | DD216005.1 | GM652159.1 | DL118653.1 | AX537272.1 |
| HI463015.1 | DL119418.1 | HI547609.1 | DD405838.1 | AX934540.1 | DD215973.1 | GM652127.1 | DL118621.1 | AX528833.1 |
| HI462576.1 | DL092225.1 | HI284299.1 | DD405806.1 | AX923391.1 | DD215927.1 | GM652095.1 | DL114225.1 | A12645.1 |
| HH713835.1 | DL092193.1 | HI571675.1 | DD402378.1 | AX824355.1 | DD215895.1 | GM652063.1 | DL114193.1 | A11116.1 |
| HH732171.1 | DL092161.1 | HI574520.1 | DD402346.1 | AX823789.1 | DD215863.1 | GM652031.1 | DL114161.1 | A06510.1 |
| HH961360.1 | DL088418.1 | HI574488.1 | DD402314.1 | AX823755.1 | DD215831.1 | GM645204.1 | DL114129.1 | A07759.1 |
| HH961328.1 | DL088386.1 | HI574424.1 | DD402282.1 | GM621640.1 | DD215799.1 | GM645172.1 | DL114097.1 | A00077.1 |
| HH961296.1 | DL103314.1 | HI574392.1 | DD402250.1 | GM654809.1 | DD215767.1 | GM645140.1 | DL114065.1 | A28933.1 |
| HH961264.1 | DL098214.1 | HI574369.1 | DD402218.1 | GM654777.1 | CS254889.1 | GM645076.1 | DL126205.1 | A28844.1 |
| HH961232.1 | DL098182.1 | HI581413.1 | DD402186.1 | GM647784.1 | DD212332.1 | GM645044.1 | DL126173.1 | A22873.1 |
| HH975397.1 | DL098150.1 | HI581356.1 | DD402154.1 | GM715044.1 | DD212207.1 | GM645012.1 | DL122957.1 | A26532.1 |
| BD271894.1 | FB316733.1 | HI573640.1 | DD402090.1 | GM638676.1 | DD187282.1 | GM638213.1 | DL122925.1 | A26050.1 |
| BD271051.1 | FJ154954.1 | HI566208.1 | DD402058.1 | GM037897.1 | DD206892.1 | GM638181.1 | DL122893.1 | A25198.1 |
| BD270863.1 | CS368314.1 | HI540754.1 | DD405778.1 | GM036863.1 | DD206860.1 | GM638149.1 | DL122861.1 | A23317.1 |
| BD270577.1 | CS368250.1 | HI593058.1 | DD405746.1 | FB766249.1 | DD206798.1 | GM638117.1 | DL122829.1 | A23257.1 |
| BD270197.1 | CS367994.1 | HI636927.1 | DD405714.1 | FB747857.1 | DD205871.1 | GM638085.1 | DL114019.1 | A22416.1 |
| DD231067.1 | DL028830.1 | HI001446.1 | DD405682.1 | FB744636.1 | CS249482.1 | GM651897.1 | DL113987.1 | A16645.1 |
| DD228724.1 | DL024856.1 | HI001385.1 | DD405650.1 | DL111619.1 | DD163494.1 | GM651865.1 | DL113955.1 | A21813.1 |
| DD228601.1 | DL021484.1 | HI001350.1 | DD405618.1 | DL111587.1 | A09546.1 | GM651833.1 | DL113923.1 | A21465.1 |
| DD228443.1 | DL021452.1 | HI001315.1 | DD405598.1 | DL136395.1 | AX521542.1 | GM651801.1 | DL113891.1 | A20495.1 |
| DD227393.1 | DL021420.1 | HI003199.1 | DD405566.1 | DL110693.1 | AX521510.1 | GM645002.1 | DL113859.1 | A16532.1 |
| DD225902.1 | DL017150.1 | HI003078.1 | DD405534.1 | DL120037.1 | AX513488.1 | GM644970.1 | DL109058.1 | A18946.1 |
| DD224771.1 | DL017118.1 | HI003041.1 | DD405502.1 | DL108947.1 | AF430196.1 | GM644938.1 | DL109026.1 | A18678.1 |
| DD224217.1 | DL049421.1 | HI003000.1 | DD405470.1 | DL098829.1 | AF430164.1 | GM644906.1 | DL108994.1 | A17981.1 |
| DD223913.1 | DL049389.1 | FU263022.1 | DD405438.1 | CS368294.1 | AX511455.1 | GM644874.1 | DL104292.1 | A16050.1 |
| DD234128.1 | DL049357.1 | FU259872.1 | DD402032.1 | CS368230.1 | AX505217.1 | GM644842.1 | DL104260.1 | A16111.1 |
| DD233505.1 | DL049325.1 | FU264505.1 | DD402000.1 | CS368166.1 | AX505153.1 | GM638043.1 | DL104228.1 | A15906.1 |
| DD232715.1 | DL012466.1 | FU258457.1 | DD401968.1 | CS368038.1 | AX498430.1 | GM638011.1 | DL099590.1 | A15274.1 |
| DD231505.1 | DL048958.1 | HC313120.1 | DD401907.1 | CS367974.1 | AX497100.1 | DL100605.1 | DL099558.1 | A14312.1 |
| DD231449.1 | DL037125.1 | HC313052.1 | DD401875.1 | CS367910.1 | BD140182.1 | DL100573.1 | DL093452.1 | A12463.1 |
| DD217683.1 | DL028658.1 | DM475558.1 | DD401843.1 | CS367654.1 | BD139559.1 | DL100541.1 | DL093420.1 | A12097.1 |
| DD221370.1 | DL028626.1 | HC299780.1 | DD401811.1 | DL080569.1 | BD138747.1 | DL119995.1 | DL093388.1 | A09758.1 |
| DD220928.1 | DL028594.1 | HC306127.1 | DD401779.1 | DL075912.1 | BD137459.1 | DL119963.1 | DL093356.1 | A06770.1 |
| DD216684.1 | DL028562.1 | HB836458.1 | DD401747.1 | DL027865.1 | BD135860.1 | DL119931.1 | DL126101.1 | A03764.1 |

| | | | | | | | | |
|---|---|---|---|---|---|---|---|---|
| DD213978.1 | DL026505.1 | HB843557.1 | DD401715.1 | DL027833.1 | BD131912.1 | DL119899.1 | DL122749.1 | A10480.1 |
| DD213885.1 | DL026493.1 | HB836253.1 | BD453120.1 | DL027801.1 | BD131217.1 | DL119867.1 | DL122693.1 | A08450.1 |
| DD213853.1 | DL014507.1 | HB835776.1 | BD432669.1 | DL027769.1 | BD130805.1 | DL119835.1 | DL122661.1 | A07608.1 |
| DD213821.1 | DL014475.1 | HB843101.1 | BD432362.1 | DL027590.1 | BD130773.1 | DL119803.1 | DL038962.1 | A06625.1 |
| DD216504.1 | DL014443.1 | HB835519.1 | BD399669.1 | DL023586.1 | BD130625.1 | DL115208.1 | DL038930.1 | A06129.1 |
| DD213772.1 | DL009537.1 | HB824077.1 | BD399144.1 | DL015848.1 | BD124160.1 | DL115176.1 | DL027141.1 | A05164.1 |
| DD213740.1 | DL009505.1 | HB842479.1 | BD399083.1 | CS607036.1 | BD105789.1 | DL115112.1 | DL027109.1 | A06146.1 |
| DD213708.1 | DL009473.1 | HB850381.1 | BD441338.1 | CS606946.1 | BD087487.1 | DL115080.1 | DL027045.1 | A02021.1 |
| DD213676.1 | DL009441.1 | HB841969.1 | BD495992.1 | CS592840.1 | BD085730.1 | DL115048.1 | DL027013.1 | A01411.1 |
| DD213644.1 | DL009342.1 | HB841717.1 | BD398637.1 | CS592084.1 | BD081484.1 | DL110048.1 | DL030908.1 | A01317.1 |
| DD213612.1 | DJ380964.1 | HB841301.1 | BD451732.1 | CS589289.1 | BD080435.1 | DL110016.1 | DL030869.1 | M13693.1 |
| DD220745.1 | DJ380932.1 | HB840791.1 | HB475222.1 | CS593378.1 | BD080144.1 | DL109984.1 | DL030837.1 | M12611.1 |
| DD213594.1 | DJ380900.1 | HB828766.1 | HB464821.1 | CS582100.1 | BD075616.1 | DL109952.1 | DL030805.1 | M17097.1 |
| DD213562.1 | DJ380868.1 | GM633742.1 | HB463818.1 | CS575762.1 | BD074951.1 | DL109866.1 | DL030773.1 | M11421.1 |
| DD216042.1 | DJ380816.1 | GM633710.1 | HB463600.1 | CS574805.1 | BD074802.1 | DL109834.1 | DL030741.1 | M12443.1 |
| DD216010.1 | DJ389625.1 | GM633678.1 | HB469605.1 | CS573056.1 | BD073583.1 | DL109802.1 | DL026772.1 | M10045.1 |
| DD215978.1 | DJ389467.1 | GM625843.1 | HB468041.1 | CS544571.1 | BD070611.1 | DL109770.1 | DL022800.1 | M11209.1 |
| DD215932.1 | DJ388680.1 | GM625811.1 | DM148840.1 | CS543942.1 | BD069455.1 | DL109738.1 | DL019753.1 | MN911270.1 |
| DD215900.1 | CS724537.1 | GM625779.1 | DM152710.1 | CS546675.1 | BD063674.1 | DL109706.1 | DL019721.1 | AF069384.1 |
| DD215868.1 | CS724449.1 | GM625747.1 | DM152556.1 | DD420110.1 | BD057158.1 | DL105100.1 | DL015222.1 | LT897784.1 |
| DD215836.1 | CS723810.1 | GM624723.1 | DM147322.1 | DD431299.1 | BD017745.1 | DL105068.1 | DL015190.1 | AH002299.2 |
| DD215804.1 | CS722266.1 | GM624691.1 | DM155854.1 | DD432052.1 | BD016701.1 | DL105036.1 | DL015158.1 | AF395832.1 |
| DD215772.1 | DJ353080.1 | GM659895.1 | DM146510.1 | CS540030.1 | BD014233.1 | DL105004.1 | DL015030.1 | M10911.1 |
| DD217993.1 | DJ359481.1 | GM659863.1 | DM146478.1 | CS537813.1 | BD014200.1 | DL104972.1 | DL010540.1 | DQ250224.1 |
| DD215742.1 | DJ357179.1 | GM659831.1 | DM146445.1 | CS244258.1 | BD014160.1 | DL104940.1 | DL010508.1 | AY774561.1 |
| DD215710.1 | DJ354278.1 | GM659799.1 | DM155728.1 | CS244226.1 | AX481600.1 | DL104908.1 | DL010476.1 | AF003714.1 |
| DD215678.1 | DJ361199.1 | GM659767.1 | DM150255.1 | CS244696.1 | AX477730.1 | DL100494.1 | DL010444.1 | HV534668.1 |
| DD213051.1 | DJ339847.1 | GM659735.1 | DM149928.1 | CS239817.1 | AX473166.1 | DL100430.1 | DL010412.1 | HV532702.1 |
| DD221797.1 | DJ339815.1 | GM692462.1 | HB455312.1 | CS227302.1 | AX472343.1 | DL100398.1 | DL038786.1 | HV515805.1 |
| DD211999.1 | DJ339783.1 | GM652346.1 | HB455090.1 | CS207862.1 | E61326.1 | DL100366.1 | DL038754.1 | HV515596.1 |
| DD211199.1 | DJ339751.1 | GM652314.1 | GN065521.1 | CS189608.1 | E64479.1 | DL100334.1 | DL038722.1 | HV515500.1 |
| DD210926.1 | DJ339719.1 | GM652282.1 | GN063775.1 | CS194009.1 | AX468886.1 | DL095991.1 | DL035066.1 | HV515468.1 |
| DD209232.1 | DJ339655.1 | GM645387.1 | GN053498.1 | CS191574.1 | AX468459.1 | DL124147.1 | DL035034.1 | HV515436.1 |
| CQ947456.1 | DJ339622.1 | GM645355.1 | GN053184.1 | CS174739.1 | DJ380846.1 | DL124115.1 | DL030719.1 | HV515404.1 |
| CQ947132.1 | DJ339590.1 | GM645323.1 | GN052433.1 | CS174079.1 | DJ380826.1 | DL124083.1 | DL030687.1 | HV515372.1 |
| CQ944170.1 | DJ339558.1 | GM645291.1 | GN052358.1 | CS176144.1 | DJ389529.1 | DL124051.1 | DL026750.1 | HV515340.1 |
| CQ944138.1 | DJ327360.1 | GM645259.1 | GN059746.1 | CS172302.1 | DJ388696.1 | DL124019.1 | DL026718.1 | HV515308.1 |
| CQ944106.1 | DJ327041.1 | GM645227.1 | GN048965.1 | CS165977.1 | DJ388656.1 | DL123987.1 | DL026686.1 | HV515276.1 |
| CQ944074.1 | AX664097.1 | GM652149.1 | DM025465.1 | CS161639.1 | CS726026.1 | DL119797.1 | DL026654.1 | HV515244.1 |
| CQ944042.1 | AX662207.1 | GM645194.1 | DM024280.1 | CS159815.1 | CS724492.1 | DL119765.1 | CS534833.1 | HV509869.1 |
| CQ944010.1 | AX658672.1 | GM645162.1 | DM012884.1 | CS159783.1 | CS724418.1 | DL119733.1 | CS502394.1 | HV508636.1 |
| CQ943978.1 | AX657135.1 | GM645130.1 | DM023668.1 | CS143748.1 | DJ358887.1 | DL119701.1 | CS502638.1 | HV508572.1 |
| CQ943946.1 | AX657103.1 | GM645098.1 | DM023578.1 | CS141611.1 | DJ359588.1 | DL119669.1 | CS500046.1 | HV508540.1 |
| CQ943914.1 | BD176070.1 | GM645066.1 | DM022393.1 | CS141542.1 | CS423567.1 | DL119637.1 | CS498365.1 | HV508508.1 |
| CQ943882.1 | AX643788.1 | GM645034.1 | DM022309.1 | CS141509.1 | CS419506.1 | DL119605.1 | CS497137.1 | HV508480.1 |
| CQ943850.1 | AX642084.1 | GM621589.1 | DM022210.1 | CS141289.1 | CS419038.1 | DL115010.1 | DD418610.1 | HV508445.1 |
| CQ918577.1 | AX615150.1 | GM621557.1 | DM022156.1 | CS134730.1 | CS414533.1 | DL114978.1 | DD418575.1 | HV512282.1 |

| | | | | | | | | |
|---|---|---|---|---|---|---|---|---|
| CQ898656.1 | BD171490.1 | GM657137.1 | DM021893.1 | CS131705.1 | CS417154.1 | DL114946.1 | DD412325.1 | HV512250.1 |
| CQ898624.1 | AX614787.1 | GM657105.1 | DM021806.1 | CS122831.1 | CS416481.1 | DL114914.1 | DD412045.1 | HV512218.1 |
| CQ898592.1 | AX601795.1 | GM657073.1 | DM015995.1 | CS106112.1 | CS416217.1 | DL114882.1 | DD410616.1 | HV512122.1 |
| CQ898560.1 | AX601611.1 | GM657041.1 | DM015951.1 | CS106030.1 | CS415769.1 | DL114850.1 | DD410176.1 | HV504785.1 |
| CQ898258.1 | AX601379.1 | GM657009.1 | DM027045.1 | CS333959.1 | CS410901.1 | DL123930.1 | CS493061.1 | HV504721.1 |
| CQ895508.1 | AX601347.1 | GM656977.1 | DM026364.1 | CS118246.1 | CS414843.1 | DL123898.1 | CS491150.1 | HV504689.1 |
| CQ891355.1 | AX598855.1 | GM656945.1 | GN045772.1 | CS118214.1 | CS414778.1 | DL123866.1 | CS490619.1 | HV504625.1 |
| CQ890282.1 | AX594168.1 | GM656914.1 | GN041682.1 | CS118180.1 | DD320990.1 | DL099526.1 | CS489142.1 | HV491630.1 |
| CQ888306.1 | AX587626.1 | GM656882.1 | GN034293.1 | CS118147.1 | DD320064.1 | DL099494.1 | HH794715.1 | HV504976.1 |
| CQ888088.1 | BD166163.1 | GM656850.1 | GN038617.1 | CS118082.1 | DD326993.1 | DL099462.1 | HH793452.1 | FW394283.1 |
| CQ882117.1 | BD161891.1 | GM656818.1 | GN037324.1 | CS118049.1 | DD326896.1 | DL099430.1 | HH779673.1 | FW394264.1 |
| CQ879663.1 | BD161052.1 | GM656786.1 | GN034792.1 | CS118014.1 | DD326840.1 | DL099398.1 | HH774431.1 | FW394226.1 |
| CQ877367.1 | BD143208.1 | GM656754.1 | GN030751.1 | CS117980.1 | DD321803.1 | DL099366.1 | HH773131.1 | FW394190.1 |
| CQ876151.1 | AX576926.1 | GM630485.1 | GN030623.1 | CS116765.1 | CS389364.1 | DL099334.1 | HH820911.1 | FW396389.1 |
| CQ874998.1 | AX565738.1 | GM621486.1 | GN030591.1 | CS114516.1 | CS389282.1 | DL093324.1 | HH820313.1 | FW396275.1 |
| CQ871425.1 | AX556848.1 | FB505210.1 | GN030559.1 | CS113517.1 | CS389237.1 | DL093292.1 | HH759207.1 | FW396654.1 |
| CQ871370.1 | AX556814.1 | GM649459.1 | GN030527.1 | CS106384.1 | CS389192.1 | DL093260.1 | HH759175.1 | FW396452.1 |
| CQ869303.1 | AX554102.1 | GM649427.1 | GN030495.1 | CS102960.1 | CS389086.1 | DL089549.1 | HH759143.1 | GU583854.1 |
| CQ869271.1 | AX093089.1 | GM649395.1 | GN030463.1 | CS102928.1 | CS398659.1 | DL089517.1 | HH759111.1 | HH834389.1 |
| CQ867177.1 | AX088782.1 | GM656530.1 | GN030426.1 | CS102896.1 | CS401607.1 | DL089485.1 | HH759079.1 | HH928929.1 |
| CQ861180.1 | AX088724.1 | GM656498.1 | GN030367.1 | CS102768.1 | CS406674.1 | DL089453.1 | HH759047.1 | HH931926.1 |
| CQ859621.1 | AX088682.1 | GM656466.1 | GN030303.1 | CS102736.1 | CS409987.1 | DL089389.1 | HH759015.1 | HH827039.1 |
| CQ859318.1 | AX085345.1 | GM656434.1 | GN030239.1 | CS102672.1 | CS403523.1 | DL103909.1 | HH758983.1 | HH826940.1 |
| CQ858718.1 | AX081485.1 | GM649337.1 | GN030207.1 | CS102608.1 | CS408087.1 | DL113619.1 | HH758951.1 | FW310589.1 |
| CQ857845.1 | AX078900.1 | GM649249.1 | GN030175.1 | CS102576.1 | CS402505.1 | DL113587.1 | HH757463.1 | FW308818.1 |
| CQ857714.1 | AX077852.1 | GM649217.1 | GN030111.1 | GM652666.1 | CS402178.1 | DL113555.1 | HH757431.1 | FW332327.1 |
| CQ857502.1 | AX077108.1 | GM635233.1 | GN030047.1 | GM652634.1 | CS402099.1 | DL113523.1 | HI401441.1 | FW305530.1 |
| CQ855979.1 | AX076578.1 | GM635169.1 | GN029951.1 | GM652602.1 | CS401794.1 | DL113459.1 | HI401209.1 | FW310631.1 |
| CQ855757.1 | AX068976.1 | GM635105.1 | GN029919.1 | GM645604.1 | CS380441.1 | DL108562.1 | HI401085.1 | HC757689.1 |
| CQ854724.1 | AX063581.1 | GM635073.1 | GN029821.1 | GM645540.1 | CS382619.1 | DL108530.1 | HI643126.1 | HC731036.1 |
| GM648655.1 | AX061428.1 | GM630267.1 | GN029789.1 | GM645508.1 | CS376593.1 | DL108498.1 | HI642793.1 | HC729393.1 |
| GM648623.1 | AX059668.1 | GM630235.1 | GN014037.1 | GM645476.1 | DD298468.1 | DL103892.1 | HI642249.1 | HC728193.1 |
| GM641820.1 | AX054571.1 | GM630203.1 | GN013383.1 | GM645444.1 | DD308741.1 | DL103860.1 | FW351385.1 | HC688466.1 |
| GM641788.1 | AX052943.1 | GM635038.1 | GN010225.1 | GM645412.1 | DD292516.1 | DL103828.1 | FW351173.1 | HC687204.1 |
| GM641756.1 | AX045808.1 | GM635006.1 | GN033491.1 | GM638615.1 | DD291019.1 | DL103796.1 | FW350964.1 | HC490849.1 |
| GM641724.1 | AX040180.1 | GM634974.1 | GN033459.1 | GM638583.1 | CS365141.1 | DL103764.1 | FW349891.1 | HC490817.1 |
| GM641692.1 | AX039518.1 | GM634942.1 | GN033395.1 | GM638551.1 | CS375760.1 | DL103732.1 | HD122463.1 | HC490721.1 |
| GM641659.1 | AX038742.1 | FB674302.1 | GN033363.1 | GM638519.1 | CS362719.1 | DL093148.1 | HD114092.1 | HC490689.1 |
| GM641627.1 | AX036014.1 | DL233406.1 | GN033331.1 | GM638487.1 | CS378184.1 | DL093116.1 | HD082423.1 | HC089275.1 |
| GM634439.1 | AX035964.1 | DL206480.1 | GM632972.1 | GM638455.1 | CS359744.1 | DL093084.1 | HD112021.1 | HC088942.1 |
| GM634407.1 | AX028809.1 | FB714456.1 | GM632940.1 | GM633852.1 | CS359640.1 | DL089277.1 | HD088244.1 | HC087496.1 |
| GM634375.1 | AX027711.1 | FB713983.1 | GM632908.1 | GM633820.1 | BD438176.1 | DL089245.1 | FW345008.1 | AY774928.1 |
| GM634343.1 | AX024042.1 | FB713855.1 | GM625265.1 | GM633788.1 | BD396028.1 | DL125936.1 | FW343736.1 | AY774880.1 |
| GM634279.1 | AX023692.1 | FB713791.1 | GM625233.1 | GM633756.1 | BD427909.1 | DL125904.1 | FW343257.1 | AY774816.1 |
| GM629445.1 | AX023642.1 | FB713727.1 | GM625201.1 | GM633724.1 | BD437666.1 | DL125872.1 | HC924197.1 | AY774760.1 |
| GM629410.1 | AX023609.1 | FB712628.1 | GM625169.1 | GM633692.1 | BD437171.1 | DL125840.1 | HC923005.1 | AY774650.1 |
| GM629378.1 | AX023576.1 | FB707954.1 | GM624047.1 | GM833887.1 | BD394856.1 | DL125808.1 | HC921531.1 | AY774589.1 |

| | | | | | | | | |
|---|---|---|---|---|---|---|---|---|
| GM629346.1 | AX019954.1 | FB674508.1 | GM624015.1 | GM625921.1 | BD446171.1 | DL044957.1 | HC920691.1 | AY774526.1 |
| GM629314.1 | AX002862.1 | FB667091.1 | GM623983.1 | GM625889.1 | BD393737.1 | DL044925.1 | HD063948.1 | AY774485.1 |
| GM648591.1 | AX010931.1 | FB666657.1 | GM623951.1 | GM625857.1 | BD453352.1 | DL044893.1 | HD052876.1 | AY774358.1 |
| GM655602.1 | AX010479.1 | CS721883.1 | GM632864.1 | GM625825.1 | BD453322.1 | DL040877.1 | HD050993.1 | AY774259.1 |
| GM655570.1 | AX010239.1 | FB666097.1 | GM632832.1 | GM625793.1 | BD453290.1 | DL040845.1 | HC920312.1 | AY774203.1 |
| GM648577.1 | AX008951.1 | HI000126.1 | GM632800.1 | GM625761.1 | BD391455.1 | DL040813.1 | HD033237.1 | AY774139.1 |
| GM648545.1 | AX008151.1 | HI203125.1 | GM632768.1 | GM625729.1 | BD376349.1 | DL040781.1 | HD065419.1 | AY774073.1 |
| GM648513.1 | AX006872.1 | HI180945.1 | GM632704.1 | GM624705.1 | BD375679.1 | DL036862.1 | HD057685.1 | AY181093.1 |
| GM648481.1 | AX006187.1 | HI470716.1 | GM625125.1 | GM624673.1 | BD362617.1 | DL036830.1 | HD053599.1 | AF267910.1 |
| GM648449.1 | AX003034.1 | HI564697.1 | GM625093.1 | GM624641.1 | BD356564.1 | DL016416.1 | DM467461.1 | HC084775.1 |
| GM648417.1 | HW289992.1 | HI568911.1 | GM625029.1 | GM624609.1 | BD356466.1 | DL016384.1 | HC299779.1 | HC083743.1 |
| GM641614.1 | HW289015.1 | HI568848.1 | GM624997.1 | GM624577.1 | BD353734.1 | DL016352.1 | HC306242.1 | HC083711.1 |
| GM641550.1 | HW288935.1 | HI572744.1 | GM624965.1 | GM624545.1 | BD342497.1 | DL016320.1 | HC306202.1 | HC083534.1 |
| GM634266.1 | HW267768.1 | HI564253.1 | GM624933.1 | GM624512.1 | BD341870.1 | DL016288.1 | HC302729.1 | HC061758.1 |
| GM629269.1 | HW267423.1 | HI564153.1 | GM623907.1 | GM624480.1 | BD349865.1 | DL016256.1 | HC302647.1 | HC056138.1 |
| GM629237.1 | HW267115.1 | HI583190.1 | GM623875.1 | GM624448.1 | BD339058.1 | DL016224.1 | HC305934.1 | DM459943.1 |
| GM629205.1 | HW263020.1 | HI563645.1 | GM623843.1 | GM624416.1 | BD320104.1 | DL028157.1 | HC305894.1 | DM191200.1 |
| GM629173.1 | HW262956.1 | HI563613.1 | HI001218.1 | GM624384.1 | BD325648.1 | DL011772.1 | HC305854.1 | GM970023.1 |
| GM629141.1 | HW262845.1 | HI563578.1 | HI001171.1 | GM624352.1 | BD325552.1 | DL011740.1 | HC305814.1 | DL463053.1 |
| GM629109.1 | HW262752.1 | HI508548.1 | HI001099.1 | GM633655.1 | BD314391.1 | DL011708.1 | HC305774.1 | DL462991.1 |
| GM634039.1 | HW261399.1 | HI551325.1 | HI001081.1 | GM633623.1 | BD313774.1 | DL011676.1 | HC305734.1 | DL461708.1 |
| GM634007.1 | HW261367.1 | HI544137.1 | HI001023.1 | GM633591.1 | BD312762.1 | DL011644.1 | HC305694.1 | FB504553.1 |
| GM633975.1 | HW261303.1 | HI508395.1 | HI000991.1 | GM633559.1 | BD311557.1 | DL011612.1 | HC305654.1 | GN030532.1 |
| GM633943.1 | HW261271.1 | HI000037.1 | HI002885.1 | GM633527.1 | BD319292.1 | DL048627.1 | HC302012.1 | GN030500.1 |
| GM633911.1 | HW261239.1 | HI003674.1 | HI002847.1 | GM633495.1 | BD318870.1 | DL048595.1 | HC301627.1 | GN030340.1 |
| GM633879.1 | HW261207.1 | HI003629.1 | HI002814.1 | GM625717.1 | BD300440.1 | DL048563.1 | HC294314.1 | GM626700.1 |
| GM662839.1 | HW261175.1 | HI003587.1 | HI002757.1 | GM625685.1 | BD294412.1 | DL048531.1 | HC294038.1 | GM639076.1 |
| GM655520.1 | HW261111.1 | HI001830.1 | HI002715.1 | GM625653.1 | BD293451.1 | DL048499.1 | HC289396.1 | GM600701.1 |
| GM655488.1 | HW261079.1 | HI001769.1 | HI002666.1 | GM625621.1 | BD293053.1 | DL048467.1 | HC289344.1 | FB715254.1 |
| GM655456.1 | HW261047.1 | HI465485.1 | HI002632.1 | GM625589.1 | BD292437.1 | DL044850.1 | HC289312.1 | GM708731.1 |
| GM655392.1 | HW261015.1 | HI465304.1 | HI002597.1 | GM625557.1 | BD292047.1 | DL044818.1 | HC296932.1 | GM061167.1 |
| GM648367.1 | HW260983.1 | HI001612.1 | HI002536.1 | GM638436.1 | BD291382.1 | DL044786.1 | HC288865.1 | FB748844.1 |
| GM648335.1 | HW260951.1 | HI003395.1 | HI001489.1 | GM638404.1 | BD299655.1 | DL044722.1 | HC288829.1 | DL236089.1 |
| GM648303.1 | HW260919.1 | HI003353.1 | HI004491.1 | GM638372.1 | BD299609.1 | DL044690.1 | HC295560.1 | DL233423.1 |
| GM648271.1 | HW260887.1 | HI003313.1 | HI002460.1 | GM638340.1 | BD298097.1 | DL040610.1 | HC295496.1 | DL233390.1 |
| GM648239.1 | HW260855.1 | HI505480.1 | HI002402.1 | GM638308.1 | CQ854364.1 | DL044634.1 | HC292576.1 | DL206331.1 |
| GM648207.1 | HW260791.1 | HI464723.1 | HI002323.1 | GM638276.1 | CQ849508.1 | DL044538.1 | HC292326.1 | FB714195.1 |
| GM641404.1 | HW260759.1 | HI464691.1 | HI000528.1 | GM638244.1 | CQ849411.1 | DL044506.1 | HC295073.1 | DL122643.1 |
| GM641372.1 | HW260727.1 | HI464659.1 | HI180491.1 | GM662086.1 | CQ840576.1 | DL044474.1 | HC294888.1 | DL029088.1 |
| GM641340.1 | HW260695.1 | HI505326.1 | HI201337.1 | GM662054.1 | FW580411.1 | DL040458.1 | HC299221.1 | DL023583.1 |
| GM641308.1 | HW260663.1 | HI505038.1 | HI180169.1 | GM662022.1 | FW582542.1 | DL040426.1 | HC294648.1 | DL013984.1 |
| GM641276.1 | HW260631.1 | HI549552.1 | HI178219.1 | GM661990.1 | FW582850.1 | DL040394.1 | HC291126.1 | DJ493847.1 |
| GM641244.1 | HW260599.1 | HI542540.1 | HI202841.1 | GM661958.1 | FW582516.1 | DL040362.1 | HC290946.1 | DJ492734.1 |
| GM629053.1 | HW260567.1 | HI462683.1 | HI202809.1 | GM661926.1 | FW591958.1 | DL040330.1 | HC293529.1 | CS603372.1 |
| GM629021.1 | DL019746.1 | HI503809.1 | HI177874.1 | GM654932.1 | FW590867.1 | DL036642.1 | HC290569.1 | CS603340.1 |
| GM628989.1 | DL019714.1 | HC490702.1 | HI214569.1 | GM654900.1 | FW590731.1 | DL039622.1 | HC296940.1 | CS603276.1 |
| GM628957.1 | DL010533.1 | HC490670.1 | HI214537.1 | GM654868.1 | FW589164.1 | DL039590.1 | HC289874.1 | CS602858.1 |

| | | | | | | | | |
|---|---|---|---|---|---|---|---|---|
| GM628925.1 | DL010501.1 | HC490541.1 | HH997567.1 | GM654836.1 | FW588673.1 | DL039526.1 | HC289840.1 | CS602826.1 |
| GM646333.1 | DL010469.1 | HC490506.1 | HH997492.1 | GM654804.1 | FW593449.1 | DL047320.1 | HC201645.1 | CS602698.1 |
| GM646301.1 | DL010437.1 | HC490371.1 | HH997396.1 | GM654772.1 | FW586441.1 | DL047288.1 | HC200108.1 | CS611006.1 |
| GM646269.1 | DL010405.1 | HC490210.1 | HH997353.1 | GM647779.1 | FW592966.1 | DL043466.1 | HC199268.1 | CS607542.1 |
| GM646237.1 | DL038779.1 | HC472263.1 | HH999266.1 | GM647747.1 | FW592666.1 | DL043402.1 | HC195359.1 | CS607030.1 |
| GM639434.1 | DL038747.1 | HC472231.1 | HH999164.1 | GM647715.1 | FW592634.1 | DL043370.1 | HC195228.1 | CS606936.1 |
| GM639402.1 | DL038715.1 | HC472167.1 | HH998128.1 | GM647683.1 | FW577826.1 | DL043338.1 | HC194024.1 | CS592997.1 |
| GM639370.1 | DL035091.1 | HC471967.1 | HH999086.1 | GM647651.1 | FW577438.1 | DL043274.1 | HC193939.1 | CS592823.1 |
| GM639338.1 | DL035059.1 | HC471935.1 | HH999049.1 | GM647619.1 | FW576792.1 | DL039457.1 | HC190671.1 | DD057856.1 |
| GM639306.1 | DL030712.1 | HC471903.1 | HH998988.1 | GM640816.1 | FW576685.1 | DL039425.1 | HC187598.1 | DD055573.1 |
| GM639274.1 | DL030680.1 | HC471871.1 | HH998934.1 | GM640783.1 | FW575884.1 | DL039393.1 | HC089580.1 | DD053316.1 |
| GM639242.1 | DL026743.1 | HC678829.1 | HH997212.1 | GM640751.1 | FW575360.1 | DL039361.1 | HC089548.1 | DD050206.1 |
| GM626695.1 | DL026711.1 | HC511400.1 | HH997150.1 | GM640719.1 | FW571615.1 | DL039329.1 | HC089516.1 | DD038497.1 |
| GM626663.1 | DL019468.1 | HC509335.1 | HH970098.1 | GM640655.1 | FW572720.1 | DL039297.1 | HC089484.1 | DD026981.1 |
| GM626631.1 | DL019436.1 | HC508170.1 | HH998887.1 | GM628301.1 | HI539005.1 | DL035673.1 | DL105632.1 | DD017628.1 |
| GM626599.1 | DL019404.1 | HC504633.1 | HH998827.1 | GM628269.1 | HI470728.1 | DL035641.1 | DL105600.1 | BD434170.1 |
| GM626567.1 | CS604658.1 | HC500972.1 | HH998784.1 | GM628237.1 | HI470601.1 | DL035609.1 | DL100795.1 | BD454141.1 |
| GM626535.1 | CS592364.1 | HC500238.1 | HH997103.1 | GM628205.1 | HI564706.1 | DL035577.1 | DL100763.1 | BD434157.1 |
| GM754525.1 | CS592092.1 | HC499858.1 | HH997070.1 | GM628173.1 | HI568923.1 | DL035545.1 | DL100731.1 | BD453911.1 |
| GM660700.1 | CS589305.1 | FW303078.1 | HH997011.1 | GM628141.1 | HI568854.1 | DL035513.1 | DL096285.1 | BD453878.1 |
| GM660668.1 | CS597715.1 | FW300947.1 | HH996959.1 | GM628109.1 | HI559125.1 | DL031509.1 | DL096253.1 | BD453846.1 |
| GM660636.1 | CS585257.1 | FW300535.1 | HH998715.1 | GM628105.1 | HI554954.1 | DL031477.1 | DL120177.1 | BD453814.1 |
| GM660604.1 | CS584092.1 | FW298763.1 | HH998680.1 | GM628073.1 | HI564201.1 | DL031445.1 | DL120145.1 | BD453782.1 |
| GM660572.1 | CS583658.1 | HC486499.1 | HH998605.1 | GM628041.1 | HI564094.1 | DL031413.1 | DL120113.1 | BD453758.1 |
| GM660540.1 | CS574813.1 | HC486434.1 | HH998555.1 | GM628009.1 | HI563584.1 | DL031381.1 | DL120081.1 | BD453726.1 |
| GM653353.1 | CS573064.1 | HC485944.1 | HH996929.1 | GM627977.1 | HI575629.1 | DL031349.1 | DL120049.1 | BD453694.1 |
| GM653321.1 | CS570787.1 | HC475377.1 | HH996857.1 | GM627945.1 | HI551343.1 | DL027444.1 | DL120017.1 | BD453662.1 |
| GM653289.1 | CS546698.1 | HC466632.1 | HH996774.1 | GM715522.1 | HI544143.1 | DL027412.1 | DL094239.1 | BD453630.1 |
| GM653257.1 | DD420248.1 | HC491755.1 | HH994567.1 | GM661915.1 | HI508240.1 | DL027380.1 | DL094207.1 | BD453598.1 |
| GM653225.1 | DD431748.1 | HC025498.1 | HH980991.1 | GM661883.1 | HI000044.1 | DL023568.1 | DL094175.1 | BD453548.1 |
| GM653193.1 | CS539919.1 | HC025402.1 | HH954688.1 | GM661851.1 | HI000006.1 | DL023536.1 | DL090496.1 | BD453516.1 |
| GM646194.1 | CS531814.1 | HC024829.1 | HH962760.1 | CS001398.1 | HI003645.1 | DL023504.1 | DL086295.1 | BD453484.1 |
| GM646162.1 | CS503963.1 | HC007736.1 | HH980574.1 | CS000599.1 | HI003593.1 | DL023472.1 | DL086263.1 | BD453452.1 |
| GM646130.1 | CS501497.1 | HC007672.1 | HH980503.1 | CS000340.1 | HI003558.1 | DL023440.1 | DL086199.1 | BD453420.1 |
| GM646098.1 | CS500219.1 | HC007548.1 | HH980435.1 | CS176012.1 | HI003508.1 | DL023408.1 | DL089474.1 | BD453388.1 |
| GM646034.1 | CS446328.1 | HC007516.1 | HH957938.1 | CS157806.1 | HI001836.1 | DL023376.1 | DL089442.1 | BD453243.1 |
| DJ056896.1 | CS442970.1 | HC010681.1 | HH980362.1 | CS157774.1 | HI001776.1 | DL020361.1 | DL113544.1 | BD453211.1 |
| DJ056358.1 | CS438938.1 | DM375841.1 | HH980311.1 | CS157935.1 | HI001738.1 | DL020329.1 | DL093137.1 | BD497806.1 |
| CS623609.1 | CS436047.1 | DM375468.1 | HH980227.1 | CS157903.1 | HI465706.1 | DL020297.1 | DL093105.1 | BD496002.1 |
| CS623539.1 | CS434856.1 | DM370688.1 | HH980164.1 | CS146895.1 | HI506051.1 | DL020265.1 | DL122555.1 | AX823783.1 |
| DD450362.1 | DD335677.1 | DM370656.1 | HH980130.1 | CS144321.1 | HI465517.1 | DL020233.1 | DL122523.1 | AX816460.1 |
| DD450330.1 | DD335094.1 | DM370624.1 | HH980059.1 | CS126755.1 | HI574578.1 | DL020201.1 | DL118109.1 | AX814387.1 |
| DD450266.1 | DD347270.1 | DM370592.1 | HH979885.1 | CS124736.1 | HI465369.1 | DL015798.1 | DL118077.1 | AX805576.1 |
| DD450234.1 | DD332616.1 | DM370560.1 | HH996716.1 | CS124692.1 | HI001677.1 | DL015766.1 | DL118045.1 | AX799799.1 |
| DD450161.1 | CS426822.1 | DM370528.1 | HH996648.1 | CS124650.1 | HI001618.1 | DL011180.1 | DL118013.1 | AX799438.1 |
| DD450127.1 | CS425080.1 | DM370496.1 | HH996608.1 | CS123989.1 | HI001576.1 | DL047256.1 | DL103629.1 | AX798938.1 |
| DD438045.1 | CS423200.1 | DM370464.1 | HH996552.1 | CS120794.1 | HI001520.1 | DL047224.1 | DL103565.1 | AX798477.1 |

| | | | | | | | | |
|---|---|---|---|---|---|---|---|---|
| DD449096.1 | CS422858.1 | DM367521.1 | HH998465.1 | CS119750.1 | HI003405.1 | DL047192.1 | DL099023.1 | AX798042.1 |
| DD441740.1 | CS417331.1 | HC003088.1 | HH998409.1 | CS119519.1 | HI003360.1 | DL047160.1 | DL098991.1 | AX797331.1 |
| DD437092.1 | CS416939.1 | HB865046.1 | HH998371.1 | CS119419.1 | HI003323.1 | DL047128.1 | DL098959.1 | AX796949.1 |
| DD436740.1 | CS410885.1 | HB864958.1 | HH998338.1 | CS119386.1 | HI464729.1 | DL047096.1 | DL088982.1 | BD194470.1 |
| CS619949.1 | CS414825.1 | HB865006.1 | HH999956.1 | CS119354.1 | HI464697.1 | DL047064.1 | DL021486.1 | BD187476.1 |
| CS622383.1 | DD327645.1 | HB864950.1 | HH999907.1 | CS119288.1 | HI464665.1 | DL043242.1 | DL021454.1 | AX773259.1 |
| CS617089.1 | CS389261.1 | HB864918.1 | HH979824.1 | CS119256.1 | HI464623.1 | DD361294.1 | DL021422.1 | AX766424.1 |
| CS616550.1 | CS389215.1 | HB864886.1 | HH979674.1 | CS119224.1 | HI528776.1 | DD367626.1 | DL021390.1 | BD181478.1 |
| CS613799.1 | CS389177.1 | HB864854.1 | HH999871.1 | CS119192.1 | HI505067.1 | DD367335.1 | DL017184.1 | BD180843.1 |
| CS612967.1 | CS406498.1 | HB864822.1 | HH999782.1 | CS119160.1 | HI528731.1 | DD363426.1 | DL017152.1 | AX722077.1 |
| CS612793.1 | CS402568.1 | HB864790.1 | HH999728.1 | CS119128.1 | HI463846.1 | DD362921.1 | DL017120.1 | BD177395.1 |
| CS612710.1 | BD453190.1 | HB864758.1 | HH998296.1 | CS119094.1 | HI542546.1 | DD362268.1 | DL017088.1 | AX135946.1 |
| CS604551.1 | BD411213.1 | HB859739.1 | HH998247.1 | CS119061.1 | HI504379.1 | DD370034.1 | DL017056.1 | AX127757.1 |
| CS604519.1 | BD399362.1 | HB866938.1 | HH998204.1 | CS119028.1 | HI503819.1 | CS458464.1 | DL017024.1 | AX113862.1 |
| CS604487.1 | BD451715.1 | HB866562.1 | HH998161.1 | CS118995.1 | HH716019.1 | CS458068.1 | DL049423.1 | AX107106.1 |
| CS604455.1 | BD440500.1 | HB866495.1 | HH993617.1 | CS118930.1 | HH713771.1 | CS457494.1 | DL049391.1 | AX097495.1 |
| CS604423.1 | BD440062.1 | HC000347.1 | HH979536.1 | CS118897.1 | HH961320.1 | CS457157.1 | DL049359.1 | AX088390.1 |
| CS604391.1 | BD429590.1 | HB999701.1 | HH979640.1 | CS118831.1 | HH961288.1 | CS456707.1 | DL049327.1 | AX085621.1 |
| CS604359.1 | BD429480.1 | HB980246.1 | HH932018.1 | CS118798.1 | HH961256.1 | CS451671.1 | DL037127.1 | AX083744.1 |
| CS604327.1 | BD407586.1 | HB977105.1 | FW417369.1 | CS118733.1 | HH961148.1 | CS451040.1 | DL028660.1 | AX077304.1 |
| CS604071.1 | BD428773.1 | HB976739.1 | FW416840.1 | CS118701.1 | HH975201.1 | DD360884.1 | DL028628.1 | AX074305.1 |
| CS604007.1 | BD427309.1 | DM193326.1 | FW394274.1 | CS118669.1 | HH982237.1 | DD353829.1 | DL028596.1 | AX073546.1 |
| CS603783.1 | BD395073.1 | DM195292.1 | FW416386.1 | CS118637.1 | HH974649.1 | DD359090.1 | DL028564.1 | AX061404.1 |
| CS603751.1 | BD427096.1 | DM045377.1 | HB394815.1 | CS118605.1 | HH974547.1 | DD357927.1 | DL026495.1 | AX067853.1 |
| CS603559.1 | BD426874.1 | DM045221.1 | HB394724.1 | CS118572.1 | HH998073.1 | DD357631.1 | DL022473.1 | AX057042.1 |
| CS603527.1 | BD426735.1 | DM039602.1 | HB394280.1 | CS118537.1 | FV530948.1 | BD227174.1 | DL014509.1 | AX050483.1 |
| CS603495.1 | BD394367.1 | DM044813.1 | HB389892.1 | CS118439.1 | FV530770.1 | BD225789.1 | DL014477.1 | AX048138.1 |
| CS603463.1 | BD393888.1 | DM044661.1 | HB397672.1 | CS118407.1 | FV522822.1 | BD225757.1 | DL014445.1 | AX046332.1 |
| CS603431.1 | BD453374.1 | DM060734.1 | DM118035.1 | CS118340.1 | FV534451.1 | BD225725.1 | DL009859.1 | DD259352.1 |
| CS603399.1 | BD453310.1 | DM060670.1 | GM715167.1 | CS118306.1 | FV534419.1 | BD225693.1 | DL009827.1 | DD259175.1 |
| CS603367.1 | BD453278.1 | GN068783.1 | GM604351.1 | CS118273.1 | HC460422.1 | BD225661.1 | DL009795.1 | DD258823.1 |
| CS603335.1 | BD445479.1 | GN068391.1 | GM038798.1 | CS118241.1 | AF275953.1 | BD225629.1 | DL009763.1 | DD265477.1 |
| CS603303.1 | BD392648.1 | GN067917.1 | HB386582.1 | CS118208.1 | HC456287.1 | BD224843.1 | DL009539.1 | CS304869.1 |
| CS603271.1 | BD413457.1 | GN067665.1 | HB341591.1 | CS118174.1 | HC453760.1 | BD224182.1 | DL009507.1 | CS302594.1 |
| CS603239.1 | BD388699.1 | GN067601.1 | HB338922.1 | CS118142.1 | HC453681.1 | BD222982.1 | DL009475.1 | CS302521.1 |
| CS603207.1 | BD356557.1 | GN065092.1 | HA643424.1 | CS118110.1 | AF159242.1 | BD222520.1 | DL009443.1 | CS299547.1 |
| CS603175.1 | BD356379.1 | GN052410.1 | GN033457.1 | CS118077.1 | HC452156.1 | BD217965.1 | DL034324.1 | CS299418.1 |
| CS603143.1 | BD359720.1 | GN052378.1 | GN033425.1 | CS118044.1 | HC452104.1 | BD211426.1 | DL009344.1 | CS299056.1 |
| CS603111.1 | BD342399.1 | GN051263.1 | GN033361.1 | CS117975.1 | FU772335.1 | BD209847.1 | DL009312.1 | DD252246.1 |
| CS603079.1 | BD341812.1 | DM010522.1 | GN033297.1 | CS114667.1 | FU761016.1 | BD205622.1 | DL009280.1 | DD252140.1 |
| CS603047.1 | BD349653.1 | DM015971.1 | GN033233.1 | CS113512.1 | FU760881.1 | BD195023.1 | DL009248.1 | DD251998.1 |
| CS603015.1 | BD325706.1 | DM037116.1 | GN033169.1 | CS113480.1 | FU759947.1 | DD283502.1 | DL009216.1 | DD251618.1 |
| CS602981.1 | CQ877111.1 | DM026250.1 | GN033137.1 | CS113448.1 | FU757637.1 | DD281822.1 | CS603483.1 | DD251308.1 |
| CS602949.1 | CQ875527.1 | GN045523.1 | GN033105.1 | CS113382.1 | FU757605.1 | DD279455.1 | CS603451.1 | DD250291.1 |
| CS602917.1 | CQ871407.1 | GN043371.1 | GN033041.1 | CS113350.1 | FU756843.1 | DD279027.1 | CS603419.1 | DD248957.1 |
| CS602885.1 | CQ868895.1 | GN042578.1 | GN033009.1 | CS113318.1 | FB761586.1 | CS356009.1 | CS603387.1 | DD258249.1 |
| CS602853.1 | CQ868457.1 | GN041607.1 | GN032945.1 | CS112762.1 | FB761229.1 | CS353033.1 | CS603355.1 | DD258157.1 |

| | | | | | | | | |
|---|---|---|---|---|---|---|---|---|
| CS602821.1 | CQ861367.1 | GN030739.1 | GN032913.1 | CS110956.1 | FB760332.1 | CS352715.1 | CS603323.1 | DD240763.1 |
| CS602789.1 | CQ861198.1 | GN030707.1 | GN032881.1 | CS109339.1 | FB760066.1 | CS352449.1 | CS603227.1 | DD240666.1 |
| CS602725.1 | CQ859603.1 | GN030675.1 | GN032785.1 | CS103354.1 | GM872478.1 | CS284622.1 | CS603195.1 | DD240494.1 |
| CS602693.1 | DL105130.1 | GN030611.1 | GN032754.1 | CS106379.1 | GM952618.1 | CS283940.1 | CS603163.1 | DD240110.1 |
| DD401411.1 | DL119946.1 | GN030579.1 | GN032722.1 | CS105945.1 | GM952044.1 | CS277158.1 | CS603131.1 | DD238416.1 |
| DD405194.1 | DL119914.1 | GN030547.1 | GN032690.1 | AX826089.1 | GM949481.1 | CS272521.1 | CS603099.1 | DD236642.1 |
| DD405162.1 | DL119882.1 | GN030515.1 | GN032625.1 | AX826007.1 | GM890116.1 | CS272562.1 | CS603067.1 | DD235491.1 |
| DD405130.1 | DL119850.1 | GM741833.1 | GN032593.1 | AX825911.1 | GM889319.1 | CS273643.1 | CS603035.1 | DD234714.1 |
| DD405098.1 | DL119818.1 | GM741791.1 | GN032561.1 | AX825301.1 | GM009460.1 | CS265874.1 | CS603003.1 | DD248262.1 |
| DD405066.1 | DL115191.1 | GM658315.1 | GN032530.1 | AX824458.1 | GM008858.1 | CS252560.1 | CS602969.1 | DD247187.1 |
| DD405034.1 | DL115127.1 | GM658283.1 | GN032498.1 | AX824347.1 | GM003574.1 | CS254287.1 | CS602937.1 | BD437267.1 |
| DD403970.1 | DL110063.1 | GM658251.1 | GN032434.1 | AX823781.1 | FB983193.1 | CS254101.1 | CS602905.1 | BD437178.1 |
| DD409747.1 | DL109999.1 | GM658219.1 | GN032402.1 | AX822405.1 | FB754404.1 | CS254069.1 | CS602809.1 | BD437119.1 |
| DD403011.1 | DL109967.1 | GM658187.1 | GN032370.1 | AX821573.1 | FB754272.1 | CS253985.1 | CS602713.1 | BD404714.1 |
| DD405837.1 | DL109721.1 | GM658155.1 | GN032337.1 | AX816454.1 | FB753872.1 | CS250649.1 | CS608440.1 | BD446175.1 |
| DD402409.1 | DL109689.1 | GM658105.1 | GN032305.1 | AX816114.1 | FB753528.1 | CS250617.1 | CS608355.1 | BD453356.1 |
| DD402345.1 | DL100381.1 | GM658073.1 | GN032273.1 | AX815016.1 | GM751807.1 | CS250287.1 | CS607946.1 | BD453326.1 |
| DD402313.1 | DL124130.1 | GM658041.1 | GN032241.1 | AX814384.1 | FB727251.1 | CS244967.1 | CS607898.1 | BD453294.1 |
| DD402281.1 | DL025213.1 | GM658009.1 | GN032209.1 | AX811487.1 | FB726026.1 | CS244169.1 | CS607665.1 | BD452694.1 |
| DD402249.1 | DL029522.1 | GM657977.1 | GN032177.1 | AX802172.1 | GM864107.1 | CS244217.1 | CS607068.1 | BD455483.1 |
| DD402217.1 | DL029490.1 | GM657945.1 | GN032145.1 | AX805540.1 | GM842442.1 | CS244185.1 | AX348791.1 | BD444974.1 |
| DD402185.1 | DL041882.1 | GM650753.1 | GN032113.1 | AX800441.1 | GM616329.1 | CS243070.1 | AX348104.1 | BD413600.1 |
| DD402153.1 | DL048404.1 | GM650721.1 | GN032081.1 | AX800024.1 | GM731844.1 | CS237812.1 | AX347575.1 | BD444960.1 |
| DD402121.1 | DL048308.1 | GM650689.1 | GN032049.1 | AX799751.1 | GM841607.1 | BD453478.1 | AX347313.1 | BD376364.1 |
| DD402089.1 | DL032029.1 | GM643697.1 | GN032017.1 | AX798918.1 | FB711257.1 | BD453446.1 | AX347275.1 | BD376047.1 |
| DD402057.1 | DL031997.1 | GM636540.1 | GN031985.1 | AX798429.1 | GM040850.1 | BD453414.1 | AX347239.1 | BD374049.1 |
| DD405777.1 | DL031965.1 | GM636508.1 | GN031921.1 | AX797993.1 | GM840216.1 | BD443171.1 | AX347205.1 | BD356472.1 |
| DD405745.1 | DL028124.1 | GM636476.1 | GN031889.1 | AX797170.1 | CS728679.1 | BD453237.1 | AX347169.1 | BD345066.1 |
| DD405713.1 | DL028092.1 | GM643495.1 | GN031857.1 | AX797138.1 | CS728647.1 | BD453205.1 | AX346967.1 | BD342518.1 |
| DD405681.1 | DL028060.1 | GM643463.1 | GN031825.1 | AX796904.1 | CS727323.1 | BD442920.1 | AX328161.1 | BD341874.1 |
| DD405649.1 | DL028028.1 | GM643430.1 | GN031792.1 | AX796870.1 | DL097056.1 | BD442739.1 | AX306430.1 | BD349913.1 |
| DD402046.1 | DL027996.1 | GM636441.1 | GN031695.1 | AX796838.1 | DL095132.1 | BD432660.1 | AX269154.1 | BD339127.1 |
| DD405597.1 | DL027964.1 | GM631841.1 | GN031663.1 | AX795867.1 | DL095100.1 | BD442214.1 | AX258827.1 | BD338945.1 |
| DD405565.1 | DL036410.1 | GM631809.1 | GN031631.1 | AX795448.1 | DL095036.1 | BD432342.1 | AX303559.1 | BD320152.1 |
| DD405501.1 | DL044261.1 | GM631777.1 | GN031599.1 | AX149553.1 | DL095004.1 | BD441331.1 | AX300942.1 | BD325564.1 |
| DD405469.1 | DL044229.1 | GM622846.1 | GN031567.1 | BD194455.1 | DL094972.1 | BD409478.1 | AX299240.1 | BD314447.1 |
| DD405437.1 | DL044197.1 | GM657803.1 | GN031535.1 | BD190493.1 | DL106690.1 | BD450813.1 | AX286663.1 | BD314194.1 |
| DD402031.1 | DL044165.1 | GM657770.1 | GN031471.1 | DL109709.1 | DL106658.1 | BD408119.1 | AX279930.1 | BD313691.1 |
| DD401999.1 | DL047855.1 | GM714475.1 | GN031438.1 | DL109677.1 | DL106562.1 | BD408080.1 | AX278265.1 | BD313627.1 |
| DD401967.1 | DL040024.1 | GM661487.1 | GN031406.1 | DL105071.1 | DL106530.1 | BD450223.1 | AX278054.1 | BD311525.1 |
| DD401906.1 | DL039992.1 | GM661455.1 | GN031374.1 | DL107799.1 | DL106498.1 | BD429798.1 | AX242319.1 | BD319297.1 |
| DD401874.1 | DL043669.1 | GM661423.1 | GN031342.1 | DL107767.1 | DL128695.1 | BD408009.1 | M18741.1 | BD318876.1 |
| HV689945.1 | CS798790.1 | GM661391.1 | GN031310.1 | DL107735.1 | DL128661.1 | BD449623.1 | J02525.1 | BD308194.1 |
| HV689020.1 | CS716753.1 | GM661359.1 | GN031277.1 | DL107703.1 | DL128595.1 | BD449378.1 | K01245.1 | BD300456.1 |
| HV695804.1 | CS716075.1 | GM661327.1 | GN031213.1 | DL103099.1 | DL125065.1 | BD407508.1 | M25029.1 | BD293415.1 |
| HV695626.1 | CS714155.1 | GM654333.1 | GN031181.1 | DL103067.1 | DL125033.1 | BD429352.1 | M21348.1 | BD292448.1 |
| HV688824.1 | CS675433.1 | GM654301.1 | GN031148.1 | DL103035.1 | DL125001.1 | BD418444.1 | M60112.1 | BD291395.1 |

| | | | | | | | | |
|---|---|---|---|---|---|---|---|---|
| HV600537.1 | CS680720.1 | GM654269.1 | GN031116.1 | DL102971.1 | DL124969.1 | BD429040.1 | M19564.1 | BD291240.1 |
| HV585130.1 | CS791361.1 | GM647148.1 | GN031084.1 | DL098326.1 | DL124922.1 | BD396350.1 | KU665646.1 | BD299785.1 |
| HV585098.1 | CS791087.1 | GM647116.1 | GN031052.1 | DL098294.1 | DL124890.1 | BD438216.1 | LT908471.1 | BD298689.1 |
| HV584866.1 | CS791904.1 | GM647084.1 | GN030988.1 | DL098262.1 | DL120769.1 | BD428094.1 | AH002288.2 | BD298148.1 |
| HV579190.1 | DJ031172.1 | GM647076.1 | GN030957.1 | DL098230.1 | DL120737.1 | BD437746.1 | AH003167.2 | BD296986.1 |
| HV579084.1 | DJ044919.1 | GM627510.1 | GN030925.1 | DL098198.1 | DL116174.1 | BD437701.1 | M30905.1 | BD296944.1 |
| HV579013.1 | DJ028125.1 | GM838922.1 | GN030861.1 | DL098166.1 | DL116142.1 | BD437669.1 | K01302.1 | BD296888.1 |
| HV578526.1 | DJ027952.1 | GM661296.1 | GN030829.1 | DL092156.1 | DL116110.1 | BD437637.1 | AY255830.1 | BD296846.1 |
| HV512146.1 | DJ043832.1 | GM654130.1 | GN030797.1 | DL092124.1 | DL116078.1 | BD395361.1 | AF283516.1 | BD295246.1 |
| JA660229.1 | DJ008423.1 | GM654098.1 | DM002279.1 | DL092092.1 | DL116046.1 | BD459653.1 | KJ608139.1 | BD285357.1 |
| HV492168.1 | DJ008391.1 | GM654066.1 | DM001476.1 | DL092060.1 | DL120570.1 | BD437266.1 | HW390743.1 | BD283721.1 |
| HV504954.1 | DJ008359.1 | GM654034.1 | DM006959.1 | DL092028.1 | DL120538.1 | BD437118.1 | HW405818.1 | BD287820.1 |
| HV504922.1 | CS671012.1 | GM654002.1 | GM637506.1 | DL091964.1 | DL115911.1 | AX278758.1 | HW399316.1 | DD211187.1 |
| HV504890.1 | DD401856.1 | GM653970.1 | GM637474.1 | FB332232.1 | DL115879.1 | AX278090.1 | HW408965.1 | DD208698.1 |
| HV504858.1 | DD401824.1 | GM646945.1 | GM633066.1 | CS368282.1 | DL115847.1 | AX256411.1 | HW381729.1 | DD206895.1 |
| HV504794.1 | DD401552.1 | GM646913.1 | GM633034.1 | CS368218.1 | DL106489.1 | AX256340.1 | HW390562.1 | DD206863.1 |
| HV504777.1 | DD401520.1 | GM646881.1 | GM633002.1 | CS368090.1 | DL106457.1 | AX254823.1 | HW390507.1 | DD206831.1 |
| HV504745.1 | DD401488.1 | CS119112.1 | GM632970.1 | DL080913.1 | DL128505.1 | AX253010.1 | HW390467.1 | DD206801.1 |
| HV504713.1 | DD401456.1 | CS119044.1 | GM632938.1 | DL076152.1 | DL128471.1 | AX251527.1 | HW390358.1 | DD212658.1 |
| HV504681.1 | CS476252.1 | CS119012.1 | GM632906.1 | DL075906.1 | DL128436.1 | AX249906.1 | HW389653.1 | CS236393.1 |
| HV504649.1 | CS469262.1 | CS118979.1 | GM625327.1 | DL075874.1 | DL124678.1 | AX242333.1 | HW389558.1 | DD181030.1 |
| HV504617.1 | DD400180.1 | CS118947.1 | GM625263.1 | DL075847.1 | DL124646.1 | AX242301.1 | HW389133.1 | DD163296.1 |
| HV491622.1 | DD400146.1 | CS118914.1 | GM625231.1 | DL075815.1 | DL124614.1 | AX242269.1 | HW387844.1 | DD162629.1 |
| HV504968.1 | CS460035.1 | CS118880.1 | GM625199.1 | DL075770.1 | DL124582.1 | AX242237.1 | HV939881.1 | DD161086.1 |
| HV504562.1 | CS459128.1 | CS118848.1 | GM625167.1 | DL079946.1 | DL096917.1 | AX242173.1 | HV810456.1 | DD178793.1 |
| HV504530.1 | DD367948.1 | CQ754499.1 | GM625135.1 | DL074583.1 | DL096885.1 | AX242141.1 | HV936896.1 | DD178468.1 |
| HV504498.1 | DD361277.1 | CQ754032.1 | GM624045.1 | DL079056.1 | DL096853.1 | AX242109.1 | HV925520.1 | DD166307.1 |
| HV504466.1 | DD367599.1 | D88390.1 | GM624013.1 | DL079024.1 | DL096789.1 | AX242077.1 | HV936381.1 | DD165525.1 |
| HV504434.1 | DD363149.1 | AX962028.1 | GM623981.1 | DL078940.1 | DL096757.1 | AX242045.1 | HV819631.1 | DD165443.1 |
| HV504210.1 | DD362655.1 | AX960394.1 | GM627648.1 | DL074428.1 | FB342564.1 | AX242013.1 | HV819459.1 | DD165153.1 |
| HV504174.1 | DD361630.1 | AX960362.1 | GM870257.1 | DL073615.1 | FB299247.1 | AX241981.1 | HV932682.1 | DD171277.1 |
| HV504142.1 | DD361311.1 | AX959102.1 | GM647200.1 | DL072410.1 | CS368264.1 | AX241949.1 | HV813908.1 | DD159561.1 |
| HV504110.1 | CS457256.1 | AX958102.1 | GM627588.1 | DL072289.1 | CS368136.1 | AX241917.1 | HV802934.1 | DD182187.1 |
| HV504078.1 | CS457176.1 | AX952428.1 | GM627556.1 | DL081495.1 | CS368008.1 | AX241885.1 | HV802902.1 | DD164316.1 |
| HV504046.1 | CS456722.1 | AX937800.1 | GM661497.1 | DL013614.1 | CS367816.1 | AX241853.1 | HV818559.1 | HI547694.1 |
| HV504014.1 | CS453774.1 | AX924202.1 | GM661465.1 | DL013582.1 | CS367688.1 | AX241789.1 | HV758673.1 | HI284307.1 |
| HV503982.1 | CS450650.1 | AX923399.1 | GM661433.1 | DL013550.1 | DL075897.1 | AX241757.1 | HV755115.1 | HI284275.1 |
| HV503950.1 | CS450213.1 | AX923146.1 | GM661401.1 | DL013518.1 | DL080109.1 | AX241725.1 | HV745892.1 | HI571710.1 |
| HV503854.1 | DD360254.1 | AX838563.1 | GM661369.1 | DL013486.1 | DL079087.1 | AX241661.1 | HV745364.1 | HI574528.1 |
| HV503822.1 | BD453493.1 | AX824331.1 | GM661337.1 | DL013454.1 | DL079047.1 | AX241629.1 | HV750970.1 | HI574496.1 |
| HV491136.1 | BD453461.1 | AX823763.1 | GM654343.1 | DL013422.1 | DL078931.1 | AX241565.1 | HV747494.1 | HI571458.1 |
| HV503782.1 | BD453429.1 | AX817770.1 | GM654311.1 | DL013390.1 | DL078900.1 | AX241501.1 | HV753747.1 | HI561764.1 |
| HV503750.1 | BD453397.1 | AX816040.1 | GM654279.1 | DL018177.1 | DL073098.1 | AX241469.1 | HV753524.1 | HI573731.1 |
| HV503718.1 | BD453252.1 | AX814433.1 | GM654247.1 | DL018145.1 | DL081135.1 | AX241437.1 | HV743293.1 | HI573568.1 |
| HV503686.1 | BD453220.1 | AX802888.1 | GM647190.1 | DL018113.1 | DL013509.1 | AX241405.1 | HV743218.1 | HI553878.1 |
| HV503654.1 | BD453150.1 | AX809412.1 | GM647158.1 | DL018081.1 | DL013477.1 | AX241098.1 | HV704241.1 | HI594011.1 |
| HV503594.1 | BD432172.1 | AX800153.1 | GM647126.1 | DL018049.1 | DL013445.1 | AX241066.1 | HV704026.1 | HI547153.1 |

| | | | | | | | | |
|---|---|---|---|---|---|---|---|---|
| HV503562.1 | BD452219.1 | AX799567.1 | GM647094.1 | DL018017.1 | DL013413.1 | AX241034.1 | HV701444.1 | HI636974.1 |
| HV503498.1 | BD451755.1 | AX798991.1 | GM647054.1 | DL013362.1 | DL013257.1 | AX241002.1 | HV701236.1 | HI636937.1 |
| HV503466.1 | BD440406.1 | AX357918.1 | GM627488.1 | DL013330.1 | DL013225.1 | AX240970.1 | FZ428182.1 | HI001454.1 |
| HV503434.1 | BD408095.1 | AX357199.1 | GM627456.1 | DL013298.1 | DL013193.1 | AX240938.1 | FZ423018.1 | HI001400.1 |
| HV503402.1 | BD450244.1 | BD006317.1 | GM627424.1 | DL013266.1 | DL098156.1 | AX239705.1 | FZ415476.1 | HI001358.1 |
| HV503370.1 | BD408024.1 | E50925.1 | DL260588.1 | DL013234.1 | FB299246.1 | AX236453.1 | FZ415405.1 | HI001324.1 |
| HV503338.1 | BD429588.1 | E64591.1 | DL260145.1 | DL013202.1 | CS368326.1 | AX235775.1 | FZ421519.1 | HI003207.1 |
| HV503306.1 | BD429478.1 | E62960.1 | DL260065.1 | DL017991.1 | CS368262.1 | AX235240.1 | FZ417515.1 | HI003146.1 |
| HV503274.1 | BD407581.1 | E54529.1 | DL259357.1 | DL017959.1 | CS368198.1 | AX142625.1 | FZ416891.1 | HI003088.1 |
| HV503242.1 | BD080159.1 | BD000703.1 | DL258276.1 | DL017927.1 | CS368134.1 | AX142561.1 | FZ416679.1 | HI003049.1 |
| HV503210.1 | BD074968.1 | E59815.1 | DL257212.1 | DL017895.1 | CS368006.1 | AX142239.1 | FZ419813.1 | HI003008.1 |
| HV503187.1 | BD074906.1 | E50986.1 | DL241347.1 | DL017863.1 | CS367942.1 | AX142175.1 | FW584013.1 | HI002909.1 |
| HV503155.1 | BD072839.1 | AX354618.1 | DL241049.1 | DL017831.1 | CS367878.1 | AX142047.1 | FW582242.1 | HI001272.1 |
| HV503123.1 | BD021839.1 | AX353931.1 | DL240965.1 | DL048066.1 | CS367686.1 | AX141981.1 | FW590755.1 | HI001226.1 |
| HV503091.1 | BD016716.1 | AX353673.1 | DL240640.1 | DL048035.1 | CS367622.1 | AX141917.1 | FW590721.1 | HI001172.1 |
| HV503059.1 | BD015988.1 | AX352678.1 | FB748886.1 | DL048003.1 | EU099579.1 | AX141723.1 | FW590293.1 | HI001100.1 |
| HV503027.1 | BD015615.1 | AX348855.1 | FB674311.1 | DL047971.1 | DL075896.1 | AX141595.1 | FW589145.1 | HI001082.1 |
| HV502995.1 | BD014447.1 | AX348246.1 | DL236442.1 | DL047939.1 | DL080108.1 | AX141339.1 | FW593332.1 | HI001025.1 |
| HV502963.1 | BD014215.1 | AX347317.1 | DL233416.1 | DL047907.1 | DL079086.1 | AX140365.1 | HI415743.1 | HI000992.1 |
| HV502931.1 | BD014175.1 | AX347241.1 | DL206823.1 | DL047875.1 | DL078962.1 | AX137985.1 | HI414030.1 | HI002886.1 |
| HV502899.1 | AX470111.1 | AX347171.1 | DL220937.1 | DL044053.1 | DL078930.1 | AX135823.1 | HI413993.1 | HI002848.1 |
| HV502867.1 | AX468915.1 | AX346969.1 | DL206306.1 | DL044021.1 | DL078899.1 | AX127631.1 | HI413587.1 | HI002816.1 |
| HV502835.1 | AX468153.1 | AX344925.1 | DL213602.1 | DL043989.1 | DL016203.1 | AX119977.1 | HI516570.1 | HI002758.1 |
| HV502791.1 | AX464652.1 | AX344525.1 | FB714503.1 | DL043957.1 | DL016171.1 | AX113558.1 | HI516105.1 | HI002716.1 |
| HV502759.1 | AX463604.1 | AX343666.1 | FB714404.1 | DL043925.1 | DL016139.1 | AX111976.1 | HI516073.1 | HI464734.1 |
| HV502695.1 | AX458612.1 | AX339347.1 | FB713960.1 | DL043893.1 | DL070795.1 | AX108619.1 | HI284350.1 | HI464702.1 |
| HV502631.1 | HW390738.1 | AX323101.1 | FB713768.1 | DL040076.1 | DL031639.1 | AX108289.1 | HI284292.1 | HI464670.1 |
| HV502599.1 | HW399358.1 | AX319351.1 | FB713736.1 | DL040044.1 | DL031607.1 | AX107881.1 | HI574513.1 | HI464638.1 |
| HV502567.1 | HW399309.1 | AX306660.1 | FB713704.1 | DL040012.1 | DL031575.1 | AX107290.1 | HI574481.1 | HI464429.1 |
| HV502535.1 | HW399193.1 | AX306433.1 | FB712963.1 | DL039980.1 | DL031543.1 | AX103782.1 | HI574449.1 | HI463855.1 |
| HV502503.1 | HW408956.1 | AX258830.1 | FB670837.1 | DL039948.1 | DL023666.1 | AX103647.1 | HI574417.1 | HI549880.1 |
| HV502471.1 | HW390462.1 | AX286669.1 | FB667396.1 | DL039884.1 | DL023634.1 | AX100459.1 | HI574385.1 | HI549575.1 |
| HV497273.1 | HW390397.1 | AX283598.1 | FB667109.1 | DL036196.1 | DL023602.1 | AX099994.1 | HI574362.1 | HI542559.1 |
| HV505350.1 | HW389553.1 | AX282313.1 | FB666798.1 | DL036164.1 | DL015992.1 | AX097523.1 | HI573621.1 | HI542345.1 |
| HV505225.1 | HW389100.1 | AX281528.1 | CS724667.1 | DL036132.1 | DL015960.1 | AX097489.1 | HI566194.1 | HI463009.1 |
| HV317235.1 | HW388330.1 | AX278268.1 | FB676510.1 | DL036100.1 | DL015928.1 | AX093169.1 | HI593984.1 | HI462573.1 |
| HV226858.1 | HW387805.1 | AX278056.1 | FB705969.1 | DL047854.1 | DL015896.1 | AX092951.1 | HI588456.1 | HH716025.1 |
| HV221452.1 | HW380925.1 | AX256073.1 | DL115916.1 | DL047831.1 | DL015864.1 | AX088804.1 | HI637001.1 | HH715691.1 |
| HV214664.1 | HW380834.1 | AX253474.1 | DL115884.1 | DL047799.1 | DL011246.1 | AX088752.1 | HI636957.1 | HH961357.1 |
| HV247105.1 | HW366559.1 | AX247549.1 | DL115852.1 | DL047767.1 | DL011214.1 | AX088696.1 | HI001475.1 | HH961325.1 |
| HV304096.1 | HW318447.1 | AX242321.1 | DL106494.1 | DL047735.1 | DL047638.1 | AX088363.1 | HI001375.1 | HH961293.1 |
| HV217626.1 | HW335170.1 | AX242289.1 | DL106462.1 | DL047703.1 | DL047606.1 | AX085503.1 | HI001343.1 | HH961261.1 |
| HV040139.1 | HW314141.1 | AX242225.1 | DL106430.1 | DL047671.1 | DL047542.1 | AX084142.1 | HI001300.1 | HH961229.1 |
| HV197642.1 | HW312094.1 | AX242129.1 | DL106358.1 | BD180839.1 | DL039679.1 | AX083700.1 | HI003187.1 | HH975394.1 |
| HV191327.1 | HW311234.1 | HW350672.1 | DL128476.1 | AX744006.1 | DL039647.1 | AX081668.1 | HI003130.1 | HH975092.1 |
| HV182464.1 | HW307886.1 | HW340226.1 | DL128441.1 | AX741080.1 | DL039615.1 | AX080979.1 | HI003067.1 | HH982378.1 |
| HV182400.1 | HW307854.1 | HW340163.1 | DL124747.1 | AX739987.1 | DL039583.1 | AX080469.1 | HI003033.1 | HH974552.1 |

| | | | | | | | | |
|---|---|---|---|---|---|---|---|---|
| HV182368.1 | HW307822.1 | HW339913.1 | DL124715.1 | AX722068.1 | DL039551.1 | AX079963.1 | HI002993.1 | HH998089.1 |
| HH981029.1 | HW307790.1 | HW349935.1 | DL124683.1 | AX721701.1 | DL039519.1 | AX077298.1 | HI002929.1 | HH998026.1 |
| HH980681.1 | HW307752.1 | HW348492.1 | DL124651.1 | BD178186.1 | DL039487.1 | AX074215.1 | HI001294.1 | HH997982.1 |
| HH980578.1 | HW307720.1 | HW363671.1 | DL124619.1 | BD177391.1 | DL039386.1 | AX073928.1 | HI001257.1 | HH999664.1 |
| HH980504.1 | HW307688.1 | HW363639.1 | DL124587.1 | AX718408.1 | DL039354.1 | AJ286132.1 | HI001197.1 | HH997929.1 |
| HH980436.1 | HW315866.1 | HW363494.1 | DL139206.1 | AX710316.1 | DL039322.1 | AX068381.1 | HI001146.1 | HH997784.1 |
| HH957939.1 | HW315581.1 | HW355218.1 | DL096922.1 | AX709555.1 | CS724488.1 | AX068083.1 | HI001043.1 | HH997746.1 |
| HH980363.1 | HW315548.1 | HW338954.1 | DL096890.1 | AX708531.1 | CS724455.1 | AX061570.1 | HI001009.1 | HH999466.1 |
| HH980313.1 | HW315091.1 | HW338698.1 | DL096858.1 | AX705398.1 | CS723903.1 | AX060089.1 | HI000963.1 | HH999423.1 |
| HH980228.1 | HW070165.1 | HW338314.1 | DL096794.1 | AX705189.1 | CS722282.1 | AX058566.1 | HI002867.1 | HH999373.1 |
| HH980165.1 | HW069939.1 | HW338186.1 | DL096762.1 | AX701655.1 | DJ352788.1 | AX057302.1 | HI002833.1 | HH997662.1 |
| HH980131.1 | HW069907.1 | HW338058.1 | DL094838.1 | AX701063.1 | DJ357648.1 | AX056825.1 | HI002795.1 | HH997625.1 |
| HH980060.1 | HW069807.1 | HW337930.1 | DL094806.1 | AX699437.1 | DJ357616.1 | AX055850.1 | HI002735.1 | HH997568.1 |
| HH979886.1 | HW084929.1 | HW337802.1 | DL094774.1 | AX685160.1 | DJ357584.1 | AX050245.1 | FW310238.1 | HH997493.1 |
| HH996717.1 | HW068047.1 | HW337674.1 | DL124475.1 | AX684115.1 | DJ341128.1 | AX047501.1 | FW331765.1 | HH997398.1 |
| HH996649.1 | HW097664.1 | HW337418.1 | DL124443.1 | AX674835.1 | DJ363050.1 | HW313618.1 | FW308596.1 | HH997354.1 |
| HH996609.1 | HV963293.1 | HW337290.1 | DL124411.1 | AX665509.1 | DJ362239.1 | HW312129.1 | FW333831.1 | HH999267.1 |
| FW368150.1 | HV956196.1 | HW321805.1 | DL124379.1 | AX664351.1 | DJ354541.1 | HW312077.1 | FW334243.1 | HH999167.1 |
| FW363360.1 | HW122419.1 | HW336906.1 | DL120290.1 | AX662240.1 | DJ361206.1 | DL047935.1 | FW332375.1 | HH999116.1 |
| FW363203.1 | HW117991.1 | HW155589.1 | DL120258.1 | AX659115.1 | DJ354204.1 | DL047903.1 | FW306079.1 | HH999087.1 |
| FW363001.1 | HW115142.1 | HW155509.1 | DL120226.1 | AX658896.1 | DJ360420.1 | DL047871.1 | FW332138.1 | HH999050.1 |
| FW362969.1 | HW081459.1 | HW155223.1 | DL115823.1 | AX657678.1 | DJ348058.1 | DL044049.1 | HC754647.1 | HH998989.1 |
| FW367132.1 | HW101672.1 | HW155041.1 | DL115791.1 | AX657119.1 | DJ339853.1 | DL044017.1 | HC732135.1 | HH998935.1 |
| FW362771.1 | HW096982.1 | HW158472.1 | DL115759.1 | AX657084.1 | DJ339821.1 | DL043985.1 | HC731670.1 | HH997213.1 |
| FW362664.1 | HW096674.1 | HW154722.1 | DL115727.1 | BD176102.1 | DJ339789.1 | DL043953.1 | HC731227.1 | HH997151.1 |
| FW349836.1 | HW096200.1 | HW154636.1 | DL115695.1 | BD176041.1 | DJ339757.1 | DL043921.1 | HC471762.1 | HH998888.1 |
| FW366344.1 | HW104358.1 | HW158132.1 | DL115663.1 | BD175654.1 | DJ339725.1 | DL043889.1 | HC471730.1 | HH998828.1 |
| FW351298.1 | HW064464.1 | HW153785.1 | DL115631.1 | BD175614.1 | DJ339661.1 | DL040072.1 | HC471666.1 | HH998785.1 |
| FW351248.1 | HW062333.1 | HW153751.1 | DL111237.1 | BD174684.1 | DJ334571.1 | DL040040.1 | HC471634.1 | HH997134.1 |
| FW351188.1 | HW056100.1 | HW157490.1 | DL111141.1 | BD174400.1 | DJ334055.1 | DL040008.1 | HC727366.1 | HH997071.1 |
| FW366100.1 | HW056002.1 | HW151816.1 | DL111109.1 | AX645660.1 | DJ327047.1 | DL039976.1 | HC688497.1 | HH997012.1 |
| FW361493.1 | HW043769.1 | HW159794.1 | DL111077.1 | AJ005291.1 | DJ327015.1 | DL039944.1 | HC688420.1 | HH996960.1 |
| HD121587.1 | HW042056.1 | HW096212.1 | DL106273.1 | AX642230.1 | DJ326593.1 | DL039912.1 | DM383668.1 | HH998716.1 |
| HD114845.1 | HW042024.1 | HW104298.1 | DL106241.1 | AX641920.1 | DJ122451.1 | DL031923.1 | DM381911.1 | HH998681.1 |
| HD084087.1 | HW043554.1 | HW070934.1 | DL106209.1 | BD173323.1 | DJ090092.1 | DL047827.1 | HC055555.1 | HH998606.1 |
| HD082723.1 | HW042012.1 | HW070537.1 | GM653425.1 | BD172292.1 | DJ088713.1 | DL047795.1 | HC053858.1 | HH998566.1 |
| HD082440.1 | HW041980.1 | HW099584.1 | GM653393.1 | BD171633.1 | DJ082566.1 | DL047763.1 | HC050237.1 | HH996930.1 |
| HD082369.1 | HW041948.1 | HW068970.1 | GM646394.1 | AX614974.1 | DJ081972.1 | DL047699.1 | HC045492.1 | HH996861.1 |
| HD086806.1 | HW041916.1 | HW067342.1 | GM646362.1 | AX608820.1 | DJ086338.1 | DL047667.1 | HC045460.1 | HH996775.1 |
| HD077996.1 | HW041818.1 | HW066551.1 | GM646298.1 | AX601531.1 | DJ080759.1 | DL039772.1 | HC045428.1 | HH994569.1 |
| HD077396.1 | HW049402.1 | HW083223.1 | GM646266.1 | AX601363.1 | DJ081396.1 | DL039740.1 | HC045396.1 | DL122901.1 |
| HD070415.1 | HW049370.1 | HW088257.1 | GM646234.1 | AX601331.1 | DJ069490.1 | DL039708.1 | HC045332.1 | DL122869.1 |
| HD069952.1 | HW049339.1 | HW087840.1 | GM639431.1 | AX600113.1 | DJ066416.1 | DL035924.1 | HC045268.1 | DL122837.1 |
| FW345127.1 | HW049307.1 | HW081652.1 | GM639399.1 | AX599039.1 | DJ066348.1 | DL031920.1 | HC047032.1 | DL122805.1 |
| FW345017.1 | HW049275.1 | HW081606.1 | GM639367.1 | HW353787.1 | DJ066315.1 | DL031888.1 | HC047000.1 | DL122773.1 |
| FW344904.1 | HW049243.1 | HW081534.1 | GM639335.1 | HW353723.1 | DJ066247.1 | DL031856.1 | HC046968.1 | DL113995.1 |
| FW344002.1 | HW041814.1 | HW099486.1 | GM639303.1 | HW344025.1 | DJ065933.1 | DL031824.1 | HC046936.1 | DL113963.1 |

| | | | | | | | | |
|---|---|---|---|---|---|---|---|---|
| FW343686.1 | HV984548.1 | HW103590.1 | GM639271.1 | HW353471.1 | DJ067993.1 | DL031792.1 | HC046904.1 | DL113931.1 |
| FW343558.1 | HW040420.1 | HW103518.1 | GM639239.1 | HW352306.1 | DD281070.1 | DL031760.1 | HC046876.1 | DL113899.1 |
| FW343433.1 | HV961477.1 | HW102891.1 | GM694173.1 | HW351126.1 | DD278998.1 | DL027855.1 | HC046844.1 | DL113867.1 |
| FW342978.1 | HV964298.1 | HW102798.1 | GM660569.1 | HW350985.1 | CS350542.1 | DL027791.1 | HC046812.1 | DL109066.1 |
| HC923227.1 | AY658046.1 | HW102766.1 | GM660537.1 | HW350654.1 | CS283933.1 | DL027759.1 | HC046780.1 | DL109034.1 |
| HC920707.1 | AY658014.1 | HW059078.1 | GM653350.1 | HW350585.1 | CS279313.1 | DL023947.1 | HC046748.1 | DL109002.1 |
| HD068961.1 | AY657982.1 | AY658981.1 | GM653318.1 | HW340189.1 | CS277022.1 | DL023915.1 | HC046716.1 | DL108970.1 |
| HD068776.1 | AY657950.1 | AY658949.1 | GM653286.1 | HW349936.1 | CS272551.1 | DL023883.1 | HC039690.1 | DL108938.1 |
| HD068610.1 | AY657918.1 | AY658917.1 | GM653254.1 | HW349886.1 | CS273349.1 | DL023851.1 | HC046064.1 | DL108906.1 |
| HD067821.1 | AY657886.1 | AY658885.1 | GM653222.1 | HW349772.1 | CS256591.1 | DL023819.1 | HC046032.1 | DL104300.1 |
| HD067643.1 | AY657854.1 | AY658853.1 | GM653190.1 | HW349598.1 | GN030165.1 | DL023787.1 | HC045708.1 | DL104268.1 |
| HD066662.1 | AY657822.1 | AY658821.1 | GM646191.1 | HW348272.1 | GN029941.1 | DL020676.1 | HC045676.1 | DL104204.1 |
| HD064618.1 | AY657790.1 | AY658789.1 | GM646159.1 | HW347769.1 | GN029843.1 | DL020644.1 | DM057121.1 | DL104172.1 |
| HD063597.1 | AY657758.1 | AY658757.1 | GM646127.1 | HW347737.1 | GN013371.1 | DL020612.1 | DM045512.1 | DL104140.1 |
| HD053124.1 | AY657726.1 | AY658725.1 | GM646095.1 | HW347673.1 | GN033545.1 | DL016209.1 | DM045358.1 | DL099726.1 |
| HD052974.1 | GN033501.1 | AY658693.1 | GM646063.1 | HW347641.1 | GN033513.1 | DL016177.1 | DM045096.1 | DL099694.1 |
| HD052886.1 | GN033469.1 | AY658661.1 | GM646031.1 | HW347576.1 | GN033481.1 | DL016145.1 | DM044907.1 | DL099662.1 |
| HC869642.1 | GN033437.1 | AY658629.1 | GM639228.1 | HW347512.1 | GN033449.1 | DJ436668.1 | DM039598.1 | DL099566.1 |
| HC869578.1 | GN033373.1 | AY658597.1 | GM639196.1 | HW347480.1 | GN033417.1 | DJ436509.1 | DM060874.1 | DL099534.1 |
| HC869546.1 | GN033341.1 | AY658565.1 | GM639164.1 | HW363723.1 | GN033353.1 | DJ445694.1 | DM044790.1 | DL093524.1 |
| HC876971.1 | GN033213.1 | AY658533.1 | GM639132.1 | HW120103.1 | GN033321.1 | DJ431037.1 | DM060762.1 | DL093492.1 |
| HC869518.1 | GN033117.1 | AY658501.1 | GM639100.1 | HW118926.1 | GN033289.1 | DJ444571.1 | DM060730.1 | DL093428.1 |
| HC876934.1 | GN033053.1 | AY658469.1 | DL086240.1 | HW118894.1 | GN033257.1 | DJ439617.1 | DM060666.1 | DL093396.1 |
| HC876895.1 | GN033021.1 | AY658437.1 | DL086208.1 | HW118442.1 | GN033225.1 | DJ443599.1 | GN067913.1 | DL093364.1 |
| HC868494.1 | GN032957.1 | AY658405.1 | DL110323.1 | HW115559.1 | GN033161.1 | DJ434344.1 | GN052374.1 | DL089749.1 |
| FW304571.1 | GN032893.1 | AY658373.1 | DL110291.1 | HW115123.1 | GN033129.1 | DJ438364.1 | GN046251.1 | DL089717.1 |
| FW310253.1 | GN032861.1 | AY658341.1 | DL105486.1 | HW114438.1 | GN033097.1 | DJ438358.1 | DM010492.1 | DL089685.1 |
| FW331843.1 | GN032829.1 | AY658309.1 | DL105454.1 | HW117663.1 | GN033065.1 | DJ438291.1 | DM023760.1 | DL089653.1 |
| FW309616.1 | GN032765.1 | AY658277.1 | DL105422.1 | HW101697.1 | GN033033.1 | DJ438210.1 | DM011434.1 | DL089621.1 |
| FW308795.1 | GN032734.1 | AY658245.1 | DL105390.1 | HW087219.1 | GN032905.1 | DJ427983.1 | DM021877.1 | DL089589.1 |
| FW334259.1 | GN032702.1 | AY658213.1 | DL105358.1 | HW105472.1 | GN032873.1 | DJ428141.1 | DM016058.1 | DL126141.1 |
| FW333475.1 | GN032670.1 | AY658181.1 | DL105326.1 | HW105246.1 | GN032841.1 | DJ402684.1 | DM026465.1 | DL126109.1 |
| FW307547.1 | GN032637.1 | AY658149.1 | DL124345.1 | HW072789.1 | GN032809.1 | DJ402614.1 | CS802335.1 | DL126077.1 |
| FW332860.1 | GN032605.1 | AY658117.1 | DL124313.1 | HW105050.1 | GN032746.1 | DJ402548.1 | DJ052622.1 | DL126045.1 |
| FW306906.1 | GN032573.1 | AY658085.1 | DL110174.1 | HW104684.1 | GN032714.1 | DJ402296.1 | DJ051976.1 | DL126013.1 |
| FW332694.1 | GN032542.1 | AY658053.1 | DL110142.1 | HW104515.1 | GN032682.1 | DJ400807.1 | DJ050557.1 | DL125981.1 |
| HC769804.1 | GN032510.1 | AY658021.1 | DL110110.1 | HW086399.1 | GN032650.1 | DJ393091.1 | DJ048827.1 | DL125949.1 |
| HC757155.1 | GN032478.1 | AY657989.1 | DL110078.1 | HW104426.1 | GN032617.1 | DJ415869.1 | DJ048795.1 | DL122757.1 |
| HC755783.1 | GN032446.1 | AY657957.1 | DL105177.1 | HW104380.1 | GN032585.1 | DJ418645.1 | CS793450.1 | DL122701.1 |
| HC733773.1 | GN032414.1 | AY657925.1 | DL105145.1 | HW104346.1 | GN032554.1 | DJ417439.1 | CS800048.1 | DL122669.1 |
| HC742012.1 | GN032382.1 | AY657893.1 | DL105113.1 | HW104280.1 | GN032458.1 | DJ402729.1 | CS798832.1 | DL122605.1 |
| HC732412.1 | GN032349.1 | AY657861.1 | DL100667.1 | HW071197.1 | GN032426.1 | DJ381549.1 | CS716759.1 | DL118383.1 |
| HC731348.1 | GN032317.1 | AY657829.1 | DL100635.1 | HW071092.1 | GN032394.1 | DJ381040.1 | CS203480.1 | DL118351.1 |
| HC731316.1 | GN032285.1 | AY657797.1 | DL100603.1 | HW104270.1 | GN032361.1 | DJ381008.1 | CS210558.1 | DL118319.1 |
| HC731284.1 | GN032253.1 | AY657765.1 | DL100571.1 | HW104198.1 | GN032329.1 | DJ380976.1 | CS208083.1 | DL118287.1 |
| HC731252.1 | GN032221.1 | AY657733.1 | DL100539.1 | HW104075.1 | GN032297.1 | DJ380944.1 | CS189616.1 | DL118255.1 |
| HC471777.1 | DL095426.1 | AY657701.1 | DL119993.1 | HW103883.1 | GN032265.1 | DJ380912.1 | CS193217.1 | DL118223.1 |

| | | | | | | | | |
|---|---|---|---|---|---|---|---|---|
| HC471745.1 | DL095394.1 | AY657669.1 | DL119961.1 | HW099621.1 | GN032233.1 | DJ380880.1 | CS187390.1 | DL113827.1 |
| HC471685.1 | DL095362.1 | AY657637.1 | DL119929.1 | HW099554.1 | GN032201.1 | DJ380848.1 | CS174646.1 | DL113795.1 |
| HC471705.1 | DL106950.1 | AY657605.1 | DL119897.1 | A02589.1 | GN032169.1 | DJ380828.1 | CS172318.1 | DL113729.1 |
| HC471617.1 | DL106918.1 | AY657573.1 | DL119865.1 | A06035.1 | GN032137.1 | DJ389545.1 | CS159791.1 | DL113755.1 |
| HC471503.1 | DL112063.1 | AY657541.1 | DL119833.1 | CS439050.1 | GN032105.1 | DJ388699.1 | CS159026.1 | DL113699.1 |
| HC471505.1 | DL116665.1 | AY657509.1 | DL115238.1 | CS438577.1 | GN032073.1 | DJ388658.1 | CS155781.1 | DL113667.1 |
| HC471563.1 | DL106189.1 | AY657477.1 | DL115206.1 | CS436518.1 | GN032041.1 | CS726031.1 | CS144290.1 | DL108866.1 |
| DL086833.1 | DL106082.1 | AY657445.1 | DL115174.1 | CS435291.1 | GN032009.1 | CS724503.1 | CS141682.1 | DL108834.1 |
| DL086801.1 | DL101213.1 | AY657413.1 | DL115142.1 | CS435227.1 | GN031977.1 | CS724421.1 | CS141557.1 | DL108802.1 |
| DL086769.1 | DL104565.1 | AY657381.1 | DL115110.1 | CS435195.1 | GN031913.1 | CS723029.1 | CS141517.1 | DL108770.1 |
| DL086737.1 | DL104533.1 | AY657349.1 | DL115078.1 | CS434829.1 | GN031881.1 | DJ359596.1 | CS141485.1 | DL108738.1 |
| DL086705.1 | DL100055.1 | AY657317.1 | DL115046.1 | DD347359.1 | GN031849.1 | DJ357816.1 | CS143467.1 | DL108706.1 |
| DL086673.1 | DL123333.1 | AY657285.1 | DL110046.1 | DD345868.1 | GN031817.1 | DJ357784.1 | CS122931.1 | DL104026.1 |
| DL086641.1 | DL104313.1 | AY657253.1 | DL110014.1 | DD346211.1 | GN031784.1 | DJ357720.1 | CS122375.1 | DL104028.1 |
| DL086609.1 | DL088620.1 | AY657221.1 | DL109982.1 | CS430556.1 | GN031719.1 | DJ357686.1 | CS106063.1 | DL098534.1 |
| DL105882.1 | DL088588.1 | AY657189.1 | DL109950.1 | CS426730.1 | GN031687.1 | DD401441.1 | CS179700.1 | DL098472.1 |
| DL105850.1 | DL092345.1 | AY657157.1 | DL109768.1 | CS433138.1 | GN031655.1 | DD401409.1 | CS179212.1 | DL098440.1 |
| DL105818.1 | DL092313.1 | AY657125.1 | DL109736.1 | CS418598.1 | GN031623.1 | DD401196.1 | CS327371.1 | DL092334.1 |
| DL123834.1 | DL092281.1 | AY657093.1 | DL109704.1 | CS422431.1 | GN031559.1 | DD405192.1 | CS326338.1 | DL092302.1 |
| DL123802.1 | DL092217.1 | AY499193.1 | DL105098.1 | CS419061.1 | GN031527.1 | DD405160.1 | CS323616.1 | DL092270.1 |
| DL123770.1 | DL092185.1 | M16210.1 | DL105066.1 | CS274809.1 | GN031495.1 | DD405128.1 | CS323583.1 | DL092206.1 |
| DL119591.1 | DL121906.1 | M18897.1 | DL105034.1 | CS273064.1 | GN031463.1 | DD405096.1 | CS323284.1 | DL092174.1 |
| DL119559.1 | FB316579.1 | M20979.1 | DL105002.1 | CS265875.1 | GN031430.1 | DD405064.1 | CS329497.1 | DL088559.1 |
| DL119527.1 | DL029762.1 | M18801.1 | DL104970.1 | CS254288.1 | GN031398.1 | DD405032.1 | DD271399.1 | DL088527.1 |
| DL119495.1 | DJ493868.1 | M34596.1 | DL104938.1 | CS253947.1 | GN031366.1 | CS414844.1 | AY967086.1 | DL088495.1 |
| DL119463.1 | DJ491606.1 | M59863.1 | DL104906.1 | CS254102.1 | GN031334.1 | CS414779.1 | AY967054.1 | DL088463.1 |
| DL119431.1 | DJ491574.1 | HV549072.1 | DL100492.1 | CS254070.1 | GN031269.1 | DD320996.1 | AY967022.1 | DL088431.1 |
| DL123742.1 | DL007685.1 | HV543854.1 | DL100460.1 | CS253986.1 | GN031237.1 | DD320073.1 | AY966958.1 | DL088399.1 |
| DL123710.1 | DJ445551.1 | HV543483.1 | DL100428.1 | CS249741.1 | GN031205.1 | DD326841.1 | AY966926.1 | DL117799.1 |
| DL123678.1 | DJ433803.1 | HA635590.1 | DL100396.1 | CS250618.1 | GN031173.1 | DD321804.1 | CS052778.1 | DL117767.1 |
| DL123646.1 | DJ438338.1 | HA637703.1 | DL100364.1 | CS250289.1 | GN031140.1 | DD325739.1 | CS052408.1 | DL117735.1 |
| DL123614.1 | DJ438303.1 | DM102625.1 | DL100332.1 | CS247201.1 | GN031108.1 | CS390530.1 | CS052374.1 | DL117703.1 |
| DL123582.1 | DJ438267.1 | DM109920.1 | DL096085.1 | CS244968.1 | GN031076.1 | CS389365.1 | CS052340.1 | DL117671.1 |
| DL119392.1 | DJ402715.1 | DM094830.1 | DL124145.1 | CS244170.1 | GM661329.1 | CS389283.1 | CS052306.1 | DL117639.1 |
| DL119360.1 | DJ401325.1 | GN368435.1 | DL124113.1 | CS244250.1 | GM654335.1 | CS389240.1 | CS051801.1 | DL122098.1 |
| DL119328.1 | DJ400819.1 | GN368299.1 | DL124081.1 | CS244218.1 | GM654303.1 | CS389193.1 | CS048767.1 | DL122066.1 |
| DL114836.1 | DJ400787.1 | GN030641.1 | DL124049.1 | CS244186.1 | GM654271.1 | CS389088.1 | CS047278.1 | DL122034.1 |
| DL114804.1 | CS244260.1 | GN030609.1 | DL124017.1 | CS243071.1 | GM654239.1 | DD118650.1 | CS038919.1 | DL122002.1 |
| DL114772.1 | CS244196.1 | GN030577.1 | DL123985.1 | CS237887.1 | GM647150.1 | DD091025.1 | CS038848.1 | DL125743.1 |
| DL114740.1 | CS244706.1 | GN030545.1 | DL119795.1 | CS228596.1 | GM647118.1 | DD118107.1 | CS029876.1 | DL125711.1 |
| DL114708.1 | CS243147.1 | GN030513.1 | DL119763.1 | CS235459.1 | GM647086.1 | DD090507.1 | CS021442.1 | DL125679.1 |
| DL114676.1 | CS237270.1 | GN030481.1 | DL114801.1 | CS200918.1 | GM647078.1 | DD117209.1 | CS020249.1 | DL125647.1 |
| DL114644.1 | CS228031.1 | GN030415.1 | DL114769.1 | CS227280.1 | GM627512.1 | DD116350.1 | CS018737.1 | DL125615.1 |
| DL127024.1 | CS204007.1 | GN030423.1 | DL114737.1 | CS226732.1 | GM661294.1 | DD089594.1 | CS016572.1 | DL125583.1 |
| DL141556.1 | CS207903.1 | GN030353.1 | DL114705.1 | CS208933.1 | GM654132.1 | DD102576.1 | CS016534.1 | DL125551.1 |
| DL141510.1 | CS207864.1 | GN030321.1 | DL114673.1 | CS208836.1 | GM654100.1 | DD052071.1 | CS016270.1 | DL121959.1 |
| DL123542.1 | CS189610.1 | GN030257.1 | DL135078.1 | CS193245.1 | GM654068.1 | DD057944.1 | CQ990424.1 | BD276889.1 |

| | | | | | | | | |
|---|---|---|---|---|---|---|---|---|
| DL123510.1 | CS194011.1 | GN030225.1 | DL127149.1 | CS188629.1 | GM654036.1 | DD057912.1 | AX598964.1 | BD279380.1 |
| DL123478.1 | CS174639.1 | GN030193.1 | DL141545.1 | CS186207.1 | GM654004.1 | DD057880.1 | AX598854.1 | BD277777.1 |
| DL123446.1 | CS172454.1 | GN030161.1 | DL141492.1 | CS177738.1 | GM653972.1 | DD057848.1 | AX594166.1 | BD274657.1 |
| DL123382.1 | CS172306.1 | GN029937.1 | DL141431.1 | CS174035.1 | GM646947.1 | DD054772.1 | BD166162.1 | BD274224.1 |
| DL119192.1 | CS161649.1 | GN029839.1 | DL123539.1 | CS173158.1 | GM646915.1 | DD054041.1 | BD161792.1 | BD273177.1 |
| DL119160.1 | CS159785.1 | GN013591.1 | DL123507.1 | CS159807.1 | GM646883.1 | DD053308.1 | BD161051.1 | BD272248.1 |
| DL119128.1 | CS144404.1 | GN010244.1 | DL123475.1 | CS159618.1 | GM646851.1 | DD052458.1 | BD143207.1 | BD271875.1 |
| DL119096.1 | CS141613.1 | GN033541.1 | DL123443.1 | CS159208.1 | GM646819.1 | DD052234.1 | AX587892.1 | BD271080.1 |
| DL119064.1 | CS141546.1 | GN033509.1 | DL123411.1 | CS148781.1 | GM639984.1 | DD059304.1 | AX587738.1 | BD271048.1 |
| DL119032.1 | CS141511.1 | GN033477.1 | DL123379.1 | CS145676.1 | GM639952.1 | DD042130.1 | AX573784.1 | DD228687.1 |
| DL114636.1 | CS134732.1 | GN033445.1 | DL049512.1 | CS144362.1 | GM639920.1 | DD048085.1 | AX573496.1 | DD228654.1 |
| DL114604.1 | CS131723.1 | GN033413.1 | DL049480.1 | DD122267.1 | GM627282.1 | DD030076.1 | AX565637.1 | DD227514.1 |
| DL114572.1 | CS133025.1 | GN033349.1 | DL041657.1 | DD137693.1 | GM627250.1 | DD038489.1 | AX556847.1 | DD227390.1 |
| DL114540.1 | CS122868.1 | GN033317.1 | DL041625.1 | DD137244.1 | GM627218.1 | DD038307.1 | AX556813.1 | DD226722.1 |
| DL114508.1 | CS121536.1 | GN033253.1 | DL041593.1 | DD136426.1 | GM627186.1 | DD037391.1 | AX555172.1 | DD225899.1 |
| DL114476.1 | CS106115.1 | GN033221.1 | DL041561.1 | DD135766.1 | GM627154.1 | DD032527.1 | AX553988.1 | DD224214.1 |
| DL109579.1 | CS106035.1 | GN033189.1 | DL041529.1 | DD135714.1 | A20768.1 | DD030752.1 | AX546841.1 | DD223908.1 |
| DL109547.1 | CS179676.1 | GN033157.1 | DL037674.1 | DD134022.1 | A18777.1 | DD027328.1 | AX544014.1 | DD233502.1 |
| DL109515.1 | CS327365.1 | GN033125.1 | DL037642.1 | DD132292.1 | A18328.1 | DD026784.1 | AX540472.1 | DD231494.1 |
| DL109483.1 | CS326318.1 | GN033093.1 | DL037610.1 | DD107358.1 | A17373.1 | DD025947.1 | AX528970.1 | DD231446.1 |
| DL104877.1 | DD166562.1 | GN033029.1 | DL037578.1 | DD093917.1 | HW049148.1 | DD024027.1 | AX528456.1 | CS112864.1 |
| DL104845.1 | AX141867.1 | GN032997.1 | DL037546.1 | DD093885.1 | HW043075.1 | DD023831.1 | AX527688.1 | CS112346.1 |
| DL104813.1 | AX141675.1 | GN032965.1 | DL037514.1 | DD093853.1 | HW050601.1 | DD023380.1 | AX525456.1 | CS103350.1 |
| DL095922.1 | AX141291.1 | GN032933.1 | DL033311.1 | DD092325.1 | HW044237.1 | DD010249.1 | A33934.1 | CS106375.1 |
| DL095890.1 | HW099474.1 | GN032901.1 | DL033279.1 | DD119264.1 | HW054036.1 | DD017751.1 | AX402341.1 | CS103434.1 |
| DL095858.1 | HW103152.1 | GN032869.1 | DL033247.1 | DD061480.1 | HW053987.1 | DD009541.1 | AX179470.1 | CS103332.1 |
| DL095826.1 | HW089638.1 | GN032837.1 | DL033215.1 | DD118653.1 | HW040942.1 | BD495448.1 | A28084.1 | CS102919.1 |
| DL095794.1 | HW102818.1 | GN032805.1 | DL033183.1 | DD061282.1 | HV985964.1 | BD495245.1 | AX521667.1 | CS102823.1 |
| DL095762.1 | HW084178.1 | GN032773.1 | DL033151.1 | DD118407.1 | HV985932.1 | BD454039.1 | AX512774.1 | CS102727.1 |
| DL094107.1 | HW062181.1 | GN032710.1 | DL033119.1 | DD091163.1 | HV985900.1 | BD434142.1 | AF430183.1 | CS102695.1 |
| DL094075.1 | HW061820.1 | GN032646.1 | DL029111.1 | DD118110.1 | HW028875.1 | BD453903.1 | AF430151.1 | CS102631.1 |
| DL094043.1 | HW065249.1 | GN032613.1 | DL029079.1 | DD116397.1 | HV984386.1 | BD453870.1 | AX505255.1 | CS102599.1 |
| DL093979.1 | HW065216.1 | GN032550.1 | DL029047.1 | DD103198.1 | HV984262.1 | BD453838.1 | AX505191.1 | CS102567.1 |
| DL090268.1 | HW065184.1 | GN032518.1 | DL029015.1 | DD102581.1 | HV961487.1 | BD453806.1 | AX504295.1 | CS102535.1 |
| DL090236.1 | HW058473.1 | GN032486.1 | DL028983.1 | DD102448.1 | HV960464.1 | BD453750.1 | HW390742.1 | CS102503.1 |
| DL090204.1 | HW064850.1 | GN032454.1 | DL028951.1 | DD102416.1 | HV959628.1 | BD453718.1 | HW399806.1 | CS101217.1 |
| DL090172.1 | HW064714.1 | GN032422.1 | DL024977.1 | DD052023.1 | HV959240.1 | BD453686.1 | HW399404.1 | CS091386.1 |
| DL086167.1 | HW064682.1 | GN032390.1 | DL021765.1 | DD057915.1 | HV965941.1 | BD453654.1 | HW399315.1 | CS086826.1 |
| DL086135.1 | HW060675.1 | GN032357.1 | DL021733.1 | DD057883.1 | HV969954.1 | CS112343.1 | HW408964.1 | CS086005.1 |
| DL086103.1 | HW062417.1 | GN032325.1 | DL021701.1 | DD057851.1 | HV962087.1 | CS111523.1 | HW391270.1 | CS082417.1 |
| DL086071.1 | HW060586.1 | GN032293.1 | DL021669.1 | DD054860.1 | HV969801.1 | CS110904.1 | HW390554.1 | CS082282.1 |
| DL086007.1 | HW056333.1 | GN032261.1 | DL021637.1 | DD053311.1 | HV961654.1 | CS105995.1 | HW390506.1 | CS080403.1 |
| DL114441.1 | HW056246.1 | GN032197.1 | DL017399.1 | DD052237.1 | HV560885.1 | CS103431.1 | HW390466.1 | CS079155.1 |
| DL114409.1 | HW062360.1 | GN032165.1 | DL017367.1 | DD051781.1 | HV549979.1 | CS103330.1 | HW389650.1 | CS078833.1 |
| DL114377.1 | HW062328.1 | GN032133.1 | DL017335.1 | DD054995.1 | HV553637.1 | CS103296.1 | HW389557.1 | CS077804.1 |
| DL114345.1 | HW055837.1 | GN032101.1 | DL045626.1 | DD058669.1 | HV552791.1 | CS103072.1 | HW387809.1 | CS075514.1 |
| DL118693.1 | HW042051.1 | GN032069.1 | DL045594.1 | DD042730.1 | HV551413.1 | CS102949.1 | HW365744.1 | CS070576.1 |

| | | | | | | | | |
|---|---|---|---|---|---|---|---|---|
| DL118661.1 | HW042019.1 | GN032037.1 | DL045562.1 | DD042402.1 | HV550399.1 | CS102917.1 | HW373010.1 | CS070061.1 |
| DL118629.1 | HW043504.1 | GN032005.1 | DL041354.1 | DD048088.1 | HV550349.1 | CS102853.1 | HW381033.1 | CS062957.1 |
| DL114233.1 | HW042007.1 | GN031973.1 | DL041322.1 | DD030079.1 | AY659400.1 | CS102789.1 | HV775607.1 | CS061204.1 |
| DL114201.1 | HW041975.1 | GN031941.1 | DL041290.1 | DD037402.1 | AY659368.1 | CS102757.1 | HV743728.1 | CS061034.1 |
| DL114169.1 | HW041943.1 | GN031877.1 | DL037403.1 | DD037080.1 | AY659336.1 | CS102693.1 | HW287351.1 | CS059012.1 |
| DL114137.1 | HW041911.1 | GN031845.1 | DL037371.1 | DD032536.1 | AY659304.1 | CS102661.1 | HW295846.1 | AY967389.1 |
| DL114105.1 | HW049503.1 | GN031813.1 | DL037339.1 | BD287346.1 | AY659272.1 | CS102629.1 | HW302527.1 | AY967357.1 |
| DL114073.1 | HW049397.1 | GN031715.1 | DL021494.1 | BD277265.1 | AY659240.1 | CS102597.1 | HW295694.1 | AY967325.1 |
| DL099727.1 | HW049365.1 | GN031683.1 | DL021462.1 | GN045893.1 | AY659208.1 | CS102565.1 | HW302343.1 | AY967293.1 |
| DL126213.1 | HW049334.1 | GN031651.1 | DL021398.1 | DM024193.1 | AY659176.1 | CS102533.1 | HW285860.1 | AX934481.1 |
| DL126181.1 | HW049302.1 | GN031619.1 | DL017192.1 | DM023667.1 | AY659144.1 | CS102501.1 | HW294024.1 | AX924038.1 |
| DL126149.1 | HW049270.1 | GN031555.1 | DL017128.1 | DM022392.1 | AY659112.1 | CS101214.1 | HW291158.1 | AX923412.1 |
| DL122933.1 | HW043309.1 | GN031523.1 | DL010175.1 | DM022308.1 | AY659080.1 | CS095855.1 | HW291107.1 | AX923379.1 |
| HW262585.1 | HW041809.1 | GM653124.1 | DL010143.1 | DM038578.1 | AY659048.1 | CS091384.1 | HW291061.1 | AX840797.1 |
| HW261413.1 | HW041777.1 | GM653092.1 | DL010111.1 | DM022277.1 | AY659016.1 | CS088890.1 | HW291029.1 | AX840534.1 |
| HW261381.1 | FW367659.1 | GM653060.1 | DL010079.1 | DM022209.1 | AY658984.1 | CS083135.1 | HW290965.1 | AX839986.1 |
| HW261349.1 | FW367274.1 | GM639002.1 | DL010047.1 | DM022155.1 | AY658952.1 | CS082413.1 | HW290902.1 | AX829279.1 |
| HW261317.1 | FW361531.1 | GM638970.1 | DL014517.1 | DM021897.1 | AY658920.1 | CS080390.1 | HW290870.1 | AX825989.1 |
| HW261285.1 | HD079297.1 | GM652836.1 | DL014485.1 | DM021805.1 | AY658888.1 | CS073744.1 | HW289988.1 | AX824453.1 |
| HW261253.1 | FW345543.1 | GM652804.1 | DL014453.1 | DM015950.1 | AY658856.1 | CS072154.1 | HW289014.1 | AX824343.1 |
| HW261189.1 | FW345144.1 | GM638814.1 | DL009995.1 | DM027044.1 | AY658824.1 | CS070535.1 | HW288886.1 | AX823906.1 |
| HW261157.1 | FW344992.1 | GM638782.1 | DL009963.1 | GN045771.1 | AY658792.1 | CS070058.1 | HW285585.1 | AX823777.1 |
| HW261125.1 | FW344920.1 | GM638750.1 | DL009931.1 | GM976284.1 | AY658760.1 | CS064607.1 | HW277186.1 | AX821554.1 |
| HW261093.1 | HC889140.1 | GM626107.1 | DL009899.1 | GM974815.1 | AY658728.1 | CS063841.1 | HW267898.1 | AX816390.1 |
| HW261061.1 | FW308817.1 | GM037555.1 | DL009867.1 | GM990787.1 | AY658696.1 | CS061197.1 | HW267767.1 | AX816106.1 |
| HW261029.1 | FW307700.1 | FB775374.1 | DL009835.1 | GM987392.1 | AY658632.1 | AX755249.1 | HW267694.1 | AX815010.1 |
| HW260997.1 | FW332325.1 | FB774871.1 | DL009803.1 | GM986817.1 | AY658600.1 | AX752656.1 | HW267422.1 | AX814829.1 |
| HW260965.1 | FW305529.1 | FB766191.1 | DL009771.1 | GM969707.1 | AY658568.1 | AX752095.1 | HW267058.1 | AX814413.1 |
| HW260933.1 | HC757686.1 | FB764690.1 | DL009547.1 | GM969671.1 | AY658536.1 | BD183108.1 | HW266179.1 | AX803560.1 |
| HW260901.1 | HC728191.1 | FB764059.1 | DL009515.1 | GM969399.1 | AY658504.1 | AX746346.1 | HW266174.1 | AX577475.1 |
| HW260869.1 | HC727356.1 | FB743922.1 | DL009451.1 | GM969935.1 | AY658472.1 | DL101010.1 | HW265964.1 | AX805985.1 |
| HW260837.1 | HC689094.1 | FB743890.1 | DL030144.1 | GM968442.1 | AY658440.1 | DL100978.1 | HW263019.1 | AX799696.1 |
| HW260805.1 | HC687203.1 | FB743858.1 | DL026175.1 | GN007498.1 | AY658408.1 | DL100946.1 | HW262844.1 | AX798914.1 |
| HW260773.1 | HC490848.1 | FB743804.1 | DL018985.1 | FB986396.1 | AY658376.1 | DL096532.1 | HW262751.1 | AX798345.1 |
| HW260741.1 | HC490784.1 | FB761827.1 | DL018953.1 | GM969084.1 | AY658344.1 | DL096500.1 | AY657666.1 | AX797134.1 |
| HW260709.1 | HC490752.1 | DJ327009.1 | DL018921.1 | GM970611.1 | AY658312.1 | DL096468.1 | AY657634.1 | AX796900.1 |
| HW260677.1 | HC490720.1 | DJ122228.1 | DL018889.1 | GM970173.1 | AY658280.1 | DL094544.1 | AY657602.1 | AX796866.1 |
| HW260645.1 | HC490688.1 | DJ090070.1 | DL018857.1 | FB736151.1 | AY658248.1 | DL094512.1 | AY657570.1 | AX796753.1 |
| HW260613.1 | HC679162.1 | DJ086034.1 | DL018825.1 | FB780317.1 | AY658216.1 | DL094480.1 | AY657538.1 | AX795561.1 |
| HW260581.1 | HC678801.1 | DJ082727.1 | DL034332.1 | DL480005.1 | AY658184.1 | DL110713.1 | AY657506.1 | AX795442.1 |
| HW260549.1 | HC510436.1 | DJ089120.1 | DL014236.1 | DL477846.1 | AY658152.1 | DL090743.1 | AY657474.1 | BD194403.1 |
| HW260517.1 | HC508657.1 | DJ080729.1 | DL009384.1 | DL470949.1 | AY658120.1 | DL090711.1 | AY657442.1 | AX794228.1 |
| HW260485.1 | HC504667.1 | DD144075.1 | DL009288.1 | DL483316.1 | AY658088.1 | DL086578.1 | AY657410.1 | AX787387.1 |
| HW260453.1 | HC494822.1 | DD099312.1 | DL009256.1 | DL462983.1 | AY658056.1 | DL086546.1 | AY657378.1 | AX786789.1 |
| HW260421.1 | HC503544.1 | DD084741.1 | DL009224.1 | DL481642.1 | AY658024.1 | DL086514.1 | AY657346.1 | AX786757.1 |
| HW260389.1 | HC502250.1 | DD084619.1 | DL018682.1 | DL460983.1 | AY657992.1 | DL086482.1 | AY657314.1 | AX785118.1 |
| HW260357.1 | HC499877.1 | DD069502.1 | DL018650.1 | GM833180.1 | AY657960.1 | DL086450.1 | AY657282.1 | AX777470.1 |

| | | | | | | | | |
|---|---|---|---|---|---|---|---|---|
| HW260325.1 | HC499844.1 | DD098134.1 | DD069878.1 | GM712172.1 | AY657928.1 | DL086418.1 | AY657250.1 | AX773253.1 |
| HW260293.1 | HC056137.1 | DD141745.1 | DD112549.1 | GM651930.1 | AY657896.1 | DL115520.1 | AY657218.1 | AX771048.1 |
| HW260229.1 | DM381883.1 | DD158397.1 | DD098770.1 | GM831174.1 | AY657864.1 | DL115488.1 | AY657186.1 | AX769828.1 |
| HW260197.1 | DQ408671.1 | DD157375.1 | DD084331.1 | GM711387.1 | AY657832.1 | DL115456.1 | AY657154.1 | AX768203.1 |
| HW260165.1 | DM380128.1 | DD156986.1 | DD083744.1 | FB509364.1 | AY657800.1 | DL121892.1 | AY657122.1 | AX766884.1 |
| HW260133.1 | HC025516.1 | DD155701.1 | DD083478.1 | FB509328.1 | AY657768.1 | DL121860.1 | AY657090.1 | HV571422.1 |
| HW260101.1 | HC025484.1 | DD154046.1 | DD141281.1 | FB509296.1 | AY657736.1 | DL121828.1 | AY657058.1 | HV560157.1 |
| HW260069.1 | HC025452.1 | DD151859.1 | DD152080.1 | FB509264.1 | AY657704.1 | DL121796.1 | U05273.1 | HV558753.1 |
| HW260037.1 | HC025420.1 | DD097296.1 | DD158428.1 | FB509185.1 | AY657672.1 | DL117533.1 | M13924.1 | HV555463.1 |
| HW260005.1 | HC010655.1 | DD082108.1 | DD158380.1 | FB509115.1 | AY657640.1 | DL117501.1 | M11279.1 | HV561860.1 |
| HW259973.1 | HC010406.1 | DD147875.1 | DD157351.1 | GM827556.1 | AY657608.1 | DL117469.1 | M16871.1 | HV554505.1 |
| HW259941.1 | HC008575.1 | DD147781.1 | CQ972397.1 | GM637828.1 | AY657576.1 | DL117437.1 | HV549109.1 | HV554297.1 |
| HW259770.1 | HC007842.1 | DD147749.1 | CQ972365.1 | GM637796.1 | AY657544.1 | DL112758.1 | HV547830.1 | HV552565.1 |
| HW259030.1 | HC011023.1 | DD147717.1 | CQ972333.1 | GM637764.1 | AY657512.1 | DL112726.1 | HV547733.1 | HV550408.1 |
| HW258909.1 | DM370674.1 | DD147685.1 | CQ971803.1 | GM637732.1 | AY657480.1 | DL112694.1 | HV543479.1 | AY659409.1 |
| HW258844.1 | DM370642.1 | DD122502.1 | CQ971130.1 | GM637700.1 | AY657448.1 | DL112662.1 | HV539099.1 | AY659377.1 |
| HW257360.1 | DM370610.1 | DD138529.1 | CQ971060.1 | GM633260.1 | AY657416.1 | DL107761.1 | HV538567.1 | AY659345.1 |
| HW257264.1 | DM370578.1 | DD138173.1 | CQ970876.1 | GM633228.1 | AY657384.1 | DL107729.1 | HV542737.1 | AY659313.1 |
| HW257200.1 | DM370546.1 | DD132341.1 | CQ970045.1 | GM633196.1 | AY657352.1 | DL107697.1 | HV541614.1 | AY659281.1 |
| HW257008.1 | DM370514.1 | DD133437.1 | CQ969053.1 | GM633164.1 | AY657320.1 | DL098320.1 | HV515089.1 | AY659249.1 |
| HW256912.1 | DM370482.1 | DD107442.1 | AX708836.1 | GM633132.1 | AY657288.1 | DL098288.1 | HV533418.1 | AY659217.1 |
| HW256880.1 | DM367507.1 | DD107336.1 | CQ964034.1 | GM633100.1 | AY657256.1 | DL098256.1 | HV534840.1 | AY659185.1 |
| HW256848.1 | DM209282.1 | DD104227.1 | CQ963550.1 | GM625521.1 | CQ772677.1 | DL098224.1 | HV532526.1 | AY659153.1 |
| HW256784.1 | HC003062.1 | DD117039.1 | CQ957174.1 | GM625489.1 | CQ771652.1 | DL098192.1 | HV537393.1 | AY659121.1 |
| HW256752.1 | GN349491.1 | DD090338.1 | CQ955897.1 | GM625457.1 | CQ771620.1 | DL098160.1 | HV515990.1 | AY659089.1 |
| HW256720.1 | GN337629.1 | DD173365.1 | CQ947173.1 | GM625425.1 | CQ756618.1 | DL092150.1 | HV515726.1 | AY659057.1 |
| HW254444.1 | GN336555.1 | DD178544.1 | CQ947124.1 | GM625393.1 | CQ754501.1 | DL092118.1 | HV515508.1 | AY659025.1 |
| HW251227.1 | HB836736.1 | DD159646.1 | CQ944194.1 | GM625361.1 | CQ754034.1 | DL092086.1 | HV515476.1 | AY658993.1 |
| HW251074.1 | HB843949.1 | DD165139.1 | CQ944162.1 | GM625329.1 | A30798.1 | DL092054.1 | HV515444.1 | AY658961.1 |
| HW250980.1 | HB843525.1 | DD159535.1 | CQ944130.1 | GM711155.1 | A29918.1 | DL092022.1 | HV515412.1 | AY658929.1 |
| HW250326.1 | HB835912.1 | DD176021.1 | CQ944098.1 | GM651753.1 | A23354.1 | DL091990.1 | HV515380.1 | AY658897.1 |
| HW249717.1 | HB843192.1 | CS057760.1 | CQ944066.1 | GM651721.1 | A32009.1 | FB299251.1 | HV515348.1 | AY658865.1 |
| HW249676.1 | HB835749.1 | CS052804.1 | CQ944034.1 | GM651689.1 | A26693.1 | CS368462.1 | HI376859.1 | AY658833.1 |
| HW247846.1 | HB835255.1 | CS052376.1 | CQ944002.1 | GM651657.1 | A16269.1 | CS368398.1 | HI375911.1 | AY658801.1 |
| HW247806.1 | HB842802.1 | CS052342.1 | CQ943970.1 | GM644793.1 | A20135.1 | CS368270.1 | HI369601.1 | AY658769.1 |
| HV936363.1 | HB842144.1 | CS052308.1 | CQ943938.1 | GM644761.1 | A19538.1 | CS368206.1 | HI369153.1 | AY658737.1 |
| HV819682.1 | HB841836.1 | CS051896.1 | CQ943906.1 | GM644729.1 | A15381.1 | CS368142.1 | HI369098.1 | AY658705.1 |
| HV814629.1 | HB841604.1 | CS048769.1 | CQ943874.1 | GM637635.1 | A11604.1 | CS368014.1 | HI424126.1 | AY658673.1 |
| HV819310.1 | HB834770.1 | CS047280.1 | CQ918490.1 | GM637603.1 | A10403.1 | CS367950.1 | HI424094.1 | AY658641.1 |
| HV803290.1 | HB840470.1 | CS046372.1 | CQ898778.1 | GM637571.1 | A08527.1 | CS367886.1 | HI424062.1 | AY658609.1 |
| HV782093.1 | HB645724.1 | CS025480.1 | CQ898648.1 | GM637539.1 | A05230.1 | CS367694.1 | HI423804.1 | AY658577.1 |
| HV930206.1 | HB645692.1 | CS023790.1 | CQ898616.1 | GM637507.1 | A32019.1 | CS367630.1 | HI422918.1 | AY658545.1 |
| HV932696.1 | HB645648.1 | CS018741.1 | CQ898584.1 | GM637475.1 | A29089.1 | DL076095.1 | HI422685.1 | AY658513.1 |
| HV803150.1 | HB855452.1 | CS017041.1 | CQ897004.1 | GM633067.1 | A25895.1 | DL075900.1 | HI415774.1 | AY658481.1 |
| HV803118.1 | DM179554.1 | CS016574.1 | CQ895564.1 | GM633035.1 | A20030.1 | DL075841.1 | CS695993.1 | AY658449.1 |
| HV803086.1 | DM179355.1 | CS008522.1 | CQ895473.1 | GM633003.1 | U26413.1 | DL075809.1 | CS695929.1 | AY658417.1 |
| HV802948.1 | DM173774.1 | CQ990426.1 | CQ893705.1 | GM632971.1 | A26067.1 | DL075764.1 | CS695897.1 | AY658385.1 |

| | | | | | | | | |
|---|---|---|---|---|---|---|---|---|
| HV802916.1 | HB559419.1 | CQ986624.1 | CQ891010.1 | GM632939.1 | A24418.1 | DL079090.1 | CS695865.1 | AY658353.1 |
| HV802884.1 | GM970617.1 | CQ874982.1 | CQ890052.1 | GM632907.1 | A25931.1 | DL079050.1 | CS695833.1 | AY658321.1 |
| HV818966.1 | GM970196.1 | CQ874247.1 | CQ889050.1 | GM632875.1 | A24547.1 | DL078934.1 | CS695705.1 | AY658289.1 |
| HV802843.1 | GM970002.1 | CQ872311.1 | CQ881867.1 | GM625264.1 | A23897.1 | DL078903.1 | CS695545.1 | AY658257.1 |
| HV822844.1 | DL477915.1 | CQ871409.1 | CQ879655.1 | GM625232.1 | A23623.1 | DL073101.1 | CS695513.1 | AY658225.1 |
| HV813008.1 | DL464579.1 | CQ868897.1 | CQ876127.1 | GM625200.1 | A22075.1 | DL013608.1 | DL022291.1 | AY658193.1 |
| HV822299.1 | DL464531.1 | CQ868489.1 | CQ875537.1 | GM624046.1 | A16155.1 | DL013576.1 | DL022259.1 | AY658161.1 |
| HV817124.1 | DL470991.1 | CQ867414.1 | CQ874718.1 | GM624014.1 | A12738.1 | DL013544.1 | DL022227.1 | AY658129.1 |
| HV816787.1 | DL462989.1 | CQ861200.1 | CQ873290.1 | GM623982.1 | A21624.1 | DL013512.1 | DL025310.1 | AY658097.1 |
| HV780156.1 | GM752129.1 | CQ859605.1 | CQ871417.1 | GM623950.1 | A20726.1 | DL013480.1 | DL025214.1 | AY658065.1 |
| HV779731.1 | GM831911.1 | CQ855868.1 | CQ871223.1 | GM632767.1 | A20766.1 | DL013448.1 | DL016240.1 | AY658033.1 |
| HV778771.1 | GM828036.1 | CQ849457.1 | CQ869295.1 | GM632735.1 | A18756.1 | DL013416.1 | DL011724.1 | AY658001.1 |
| HV778232.1 | GM674218.1 | CQ840180.1 | CQ868905.1 | GM632703.1 | A18326.1 | DL022986.1 | DL011692.1 | AY657969.1 |
| HV775035.1 | GM709024.1 | CQ827705.1 | CQ867573.1 | GM625124.1 | A05519.1 | DL019811.1 | DL011660.1 | AY657937.1 |
| HV774953.1 | GM708992.1 | CQ826739.1 | CQ866776.1 | DL036427.1 | A16017.1 | DL010790.1 | DL011628.1 | AY657905.1 |
| HV764775.1 | GM708972.1 | CQ824415.1 | CQ861210.1 | DL036395.1 | A14583.1 | DL010758.1 | DL044738.1 | AY657873.1 |
| HV764743.1 | DL121773.1 | CQ821376.1 | CQ859613.1 | DL036363.1 | A13629.1 | DL010726.1 | DL044706.1 | HV344120.1 |
| HV769879.1 | DL121645.1 | CQ821218.1 | CQ858161.1 | DL036331.1 | A13242.1 | DL010694.1 | DL032030.1 | HV343892.1 |
| HV572125.1 | DL128737.1 | CQ817005.1 | AX016801.1 | DL036299.1 | A12584.1 | DL010662.1 | DL031966.1 | HV343700.1 |
| DM022153.1 | DL090871.1 | CQ816960.1 | AX014748.1 | DL044246.1 | A11128.1 | DL010630.1 | DL028125.1 | HV343556.1 |
| DM038366.1 | DL141441.1 | CQ814493.1 | AX011477.1 | DL044214.1 | A09215.1 | DL047054.1 | DL028093.1 | HV347681.1 |
| DM015980.1 | CS368288.1 | CQ814054.1 | AX011445.1 | DL044182.1 | A08585.1 | DL047022.1 | DL028061.1 | HV340088.1 |
| GM633649.1 | CS368032.1 | CQ814021.1 | AX011413.1 | DL044118.1 | A07741.1 | DL046990.1 | DL028029.1 | HV340056.1 |
| GM633617.1 | CS367648.1 | CQ813989.1 | AX011380.1 | DL044086.1 | A06406.1 | DL046958.1 | DL027997.1 | HV346969.1 |
| GM633585.1 | DL079982.1 | CQ813957.1 | AX011348.1 | DL048223.1 | A05167.1 | DL046926.1 | DL027965.1 | HV344608.1 |
| GM633553.1 | DD458759.1 | CQ813925.1 | AX010942.1 | DL048191.1 | A03893.1 | DL046894.1 | DL044262.1 | HV339934.1 |
| GM633521.1 | DD453458.1 | CQ813893.1 | AX010618.1 | DL048159.1 | A01571.1 | DL046862.1 | DL044198.1 | HV339870.1 |
| GM633489.1 | DD452060.1 | CQ813861.1 | AX008289.1 | DL048127.1 | A01338.1 | DL043040.1 | DL044166.1 | HV344494.1 |
| GM625583.1 | DD456097.1 | CQ813829.1 | AX006407.1 | DL048063.1 | J02558.1 | DL043008.1 | DD401426.1 | HV335557.1 |
| GM625551.1 | CS631737.1 | CQ813797.1 | AX004119.1 | DL048032.1 | M25046.1 | DL042976.1 | DD401394.1 | HV342145.1 |
| GM638398.1 | CS631236.1 | CQ803121.1 | AX002910.1 | DL048000.1 | M60948.1 | DL042944.1 | DD405209.1 | HV342061.1 |
| GM624332.1 | CS631172.1 | CQ800785.1 | A48487.1 | DL047968.1 | J02531.1 | DL042912.1 | DD405177.1 | HV341806.1 |
| GM624300.1 | CS627087.1 | CQ798787.1 | A38678.1 | DL047936.1 | M25075.1 | DL042880.1 | DD405145.1 | HV323139.1 |
| GM624268.1 | DD449055.1 | HW399317.1 | A35742.1 | DL047904.1 | M60082.1 | DL038967.1 | DD405113.1 | HV332145.1 |
| GM652543.1 | DD435324.1 | HW408968.1 | A35706.1 | DL047872.1 | OK586151.1 | DL038935.1 | DD400924.1 | HV332102.1 |
| GM652511.1 | CS620451.1 | HW381730.1 | A33044.1 | DL044050.1 | MF521566.1 | DL035119.1 | DD406757.1 | HV331807.1 |
| GM652479.1 | CS618334.1 | HW390563.1 | A29523.1 | DL044018.1 | LT908475.1 | CQ787454.1 | DD402392.1 | HV324880.1 |
| GM652447.1 | CS616564.1 | HW390510.1 | A34041.1 | DL043986.1 | HV984385.1 | CQ787340.1 | DD402360.1 | HV324044.1 |
| GM652415.1 | CS612798.1 | HW389657.1 | A33984.1 | DL043954.1 | HV984261.1 | CQ787306.1 | DD402328.1 | HV313397.1 |
| GM652383.1 | CS612715.1 | HW389559.1 | A32632.1 | DL043922.1 | HV969352.1 | CQ787274.1 | DD402296.1 | HV043014.1 |
| GM659903.1 | CS611453.1 | HW389134.1 | A31579.1 | DL043890.1 | HV960463.1 | CQ786938.1 | DD402264.1 | FW572213.1 |
| GM659871.1 | CS604364.1 | HH999931.1 | A23362.1 | DL040073.1 | HV959627.1 | CQ784663.1 | DD402232.1 | FW572025.1 |
| GM659839.1 | CS362756.1 | HH993782.1 | A32029.1 | DL040041.1 | HV964132.1 | CQ772702.1 | DD402200.1 | FW571901.1 |
| GM659807.1 | CS359649.1 | HH979785.1 | A28922.1 | DL040009.1 | HV959239.1 | CQ771658.1 | DD405612.1 | HI967435.1 |
| GM659775.1 | BD269154.1 | HH979696.1 | A27460.1 | DL039977.1 | HV963638.1 | CQ771626.1 | DD405580.1 | HI968459.1 |
| GM659743.1 | BD266846.1 | HH999900.1 | A16441.1 | DL039945.1 | HV965938.1 | CQ771594.1 | DD405516.1 | HI990062.1 |
| GM659707.1 | BD265632.1 | HH999857.1 | A20282.1 | DL039913.1 | HV962322.1 | CQ768335.1 | DD405484.1 | HI989484.1 |

| | | | | | | | | |
|---|---|---|---|---|---|---|---|---|
| GM659675.1 | BD263734.1 | HH999753.1 | A19013.1 | DL036093.1 | HV962086.1 | CQ767894.1 | DD401982.1 | HI988940.1 |
| GM659643.1 | BD251783.1 | HH998321.1 | A15395.1 | DL047828.1 | HV961776.1 | CQ766995.1 | DD401921.1 | HI968571.1 |
| GM659611.1 | BD248941.1 | HH998269.1 | A14308.1 | DL047796.1 | HV961651.1 | CQ766066.1 | DD401889.1 | HI987435.1 |
| GM659579.1 | BD247121.1 | HH998232.1 | A11907.1 | DL047764.1 | HV951619.1 | CQ764839.1 | DD401649.1 | FW512089.1 |
| GM659546.1 | BD247024.1 | HH998185.1 | A08830.1 | DL047732.1 | HV951587.1 | CQ761198.1 | DD401617.1 | FW508356.1 |
| GM652354.1 | BD246941.1 | HH998131.1 | A06905.1 | DL047700.1 | HV951555.1 | CQ759551.1 | DD401585.1 | FW552271.1 |
| GM652322.1 | BD244449.1 | HH993680.1 | FW496465.1 | DL047668.1 | HV955894.1 | CQ758820.1 | DD401553.1 | FW552234.1 |
| GM652290.1 | BD242976.1 | HH986626.1 | FW420739.1 | DL030845.1 | HV950636.1 | CQ754040.1 | DD401521.1 | FW562056.1 |
| GM652258.1 | BD242437.1 | HH979516.1 | FW504759.1 | DL030813.1 | HV957929.1 | CQ753250.1 | DD401489.1 | FW552173.1 |
| GM645395.1 | BD238500.1 | HH979552.1 | FW498891.1 | DL030749.1 | HV949617.1 | AY220729.1 | DD401457.1 | FW552138.1 |
| GM645363.1 | BD236917.1 | HH979623.1 | FW420470.1 | DL022840.1 | HV947318.1 | AX963142.1 | CS479856.1 | FW552099.1 |
| GM645331.1 | BD235872.1 | HH979581.1 | FW420438.1 | DL019777.1 | HV947218.1 | AX962036.1 | CS476260.1 | HC452166.1 |
| GM645299.1 | BD235385.1 | HH931984.1 | FW420406.1 | DL019785.1 | HV940419.1 | AX960554.1 | CS468362.1 | HC449021.1 |
| GM645267.1 | BD234581.1 | FW394391.1 | FW501787.1 | DL015198.1 | HV939885.1 | AX960370.1 | DD400182.1 | FU772390.1 |
| GM645235.1 | BD233664.1 | FW394235.1 | FW420381.1 | DL015166.1 | HV504606.1 | AX960329.1 | BD427094.1 | FU759954.1 |
| GM678655.1 | BD224573.1 | FW394199.1 | FW420349.1 | DL015134.1 | HV504989.1 | AX058997.1 | BD426723.1 | FU764050.1 |
| GM659381.1 | BD223673.1 | FW418234.1 | FW420317.1 | DL015102.1 | HV504199.1 | AX058557.1 | BD394366.1 | FU757609.1 |
| GM659349.1 | BD218516.1 | FW396875.1 | FW496688.1 | DL015070.1 | HV455583.1 | AX057298.1 | BD453373.1 | FU756912.1 |
| GM652157.1 | BD218010.1 | FW396417.1 | FW496680.1 | DL010548.1 | HV504163.1 | AX056657.1 | BD453337.1 | HC438960.1 |
| GM652125.1 | BD211657.1 | FW396295.1 | FW503292.1 | DL010516.1 | HV504131.1 | AX055491.1 | BD453309.1 | HC438423.1 |
| GM652093.1 | DD220607.1 | HH936046.1 | HI653934.1 | DL010484.1 | HV504099.1 | AX052918.1 | BD453277.1 | HC360367.1 |
| GM652061.1 | DD213165.1 | HH935023.1 | HI653870.1 | DL010452.1 | HV504067.1 | AX047140.1 | BD393236.1 | HC435956.1 |
| GM652029.1 | DD206868.1 | HH834113.1 | HI653802.1 | DL010420.1 | HV504003.1 | AX046151.1 | BD392647.1 | HC358227.1 |
| GM645202.1 | DD206836.1 | FW390823.1 | HI653709.1 | DL046849.1 | HV503939.1 | AX044416.1 | BD388698.1 | HC358195.1 |
| GM645170.1 | DD206806.1 | FW392866.1 | HI653523.1 | DL046817.1 | HV503875.1 | AX040785.1 | BD356556.1 | HC358064.1 |
| GM645138.1 | DD206774.1 | HC466628.1 | HI653468.1 | DL046785.1 | HV503843.1 | AX039322.1 | BD356280.1 | HC357628.1 |
| GM645106.1 | CS236411.1 | HC466415.1 | HI653408.1 | DL046753.1 | HV503811.1 | AX038930.1 | BD359714.1 | HC434753.1 |
| GM645074.1 | DD180999.1 | HC491783.1 | HI653358.1 | DL042867.1 | HV491268.1 | AX036887.1 | BD341811.1 | HC357072.1 |
| GM638243.1 | CS052397.1 | HC491751.1 | HI651904.1 | DL042803.1 | CQ795479.1 | AX036028.1 | BD325505.1 | HC320713.1 |
| GM638211.1 | CS052295.1 | HC491643.1 | HI651038.1 | DL042771.1 | CQ795447.1 | AX035976.1 | BD314347.1 | HC356171.1 |
| GM638179.1 | CS050958.1 | HC481633.1 | HI650457.1 | DL042739.1 | CQ794296.1 | AX035433.1 | BD312675.1 | HC325466.1 |
| GM638147.1 | CS023959.1 | HC481578.1 | HI647275.1 | DL042707.1 | CQ788650.1 | AX033071.1 | BD319279.1 | HC324505.1 |
| GM638115.1 | CS018503.1 | HC479550.1 | HI646163.1 | DL042675.1 | CQ788132.1 | AX029300.1 | BD319399.1 | HC324083.1 |
| GM638083.1 | CS728686.1 | HC478535.1 | HH807302.1 | DL038794.1 | CQ787510.1 | AX028758.1 | BD300109.1 | HC315543.1 |
| GM651895.1 | CS728590.1 | FV533476.1 | HH806868.1 | DL038762.1 | CQ787406.1 | AX027721.1 | BD306273.1 | DM370968.1 |
| GM651863.1 | CS727620.1 | FV531706.1 | HH802827.1 | DL038730.1 | CQ787298.1 | AX024595.1 | BD300347.1 | DM370936.1 |
| GM651831.1 | CS727434.1 | FV531674.1 | HH796561.1 | DL038698.1 | CQ787266.1 | AX023767.1 | BD293535.1 | DM370904.1 |
| GM651799.1 | CS727334.1 | FV523709.1 | HH794949.1 | DL035042.1 | CQ787234.1 | AX023652.1 | BD291304.1 | DM370833.1 |
| GM645000.1 | GM835948.1 | FV522844.1 | FW351015.1 | DL034896.1 | CQ786930.1 | AX023620.1 | BD299631.1 | DM370764.1 |
| GM644968.1 | GM014332.1 | FV522804.1 | FW349921.1 | DL034835.1 | CQ784711.1 | AX023240.1 | BD295286.1 | DM370666.1 |
| GM644936.1 | GM011206.1 | FV528440.1 | HD122544.1 | DL030727.1 | CQ784647.1 | AX019177.1 | BD287868.1 | DM370634.1 |
| GM644904.1 | GM887779.1 | FV534511.1 | HD121589.1 | DL030535.1 | CQ779400.1 | AX016803.1 | CQ759691.1 | DM370602.1 |
| GM644872.1 | FB660874.1 | FV534407.1 | HD114096.1 | DL026726.1 | CQ778528.1 | AX014749.1 | CQ754028.1 | DM370570.1 |
| GM644840.1 | FB984995.1 | FV534083.1 | HD082297.1 | DL026694.1 | CQ774406.1 | AX011478.1 | AX962024.1 | DM370538.1 |
| GM638041.1 | GM003680.1 | HC465650.1 | HD088603.1 | DL026662.1 | CQ772588.1 | AX011446.1 | AX937795.1 | DM370506.1 |
| GM637977.1 | GM680786.1 | AF398463.1 | HD088247.1 | DL026630.1 | CQ757696.1 | AX011414.1 | AX925520.1 | DM370474.1 |
| GM637945.1 | GM680702.1 | AF310191.1 | HD080101.1 | DL026598.1 | HW380841.1 | AX011381.1 | AX924083.1 | DM367582.1 |

| | | | | | | | | |
|---|---|---|---|---|---|---|---|---|
| GM637913.1 | FB725694.1 | HC453671.1 | HD077381.1 | DL026566.1 | HW380801.1 | AX011349.1 | AX923395.1 | DM367494.1 |
| GM637881.1 | FB722371.1 | HC460381.1 | HD070440.1 | DL022722.1 | HW368618.1 | AX011210.1 | AX838399.1 | DM371969.1 |
| GM637849.1 | GM615425.1 | AF087957.1 | FW345010.1 | DL022690.1 | HW364602.1 | AX010943.1 | AX828670.1 | DM204760.1 |
| GM631091.1 | FB712133.1 | HC442310.1 | FW344729.1 | DL022658.1 | HW373208.1 | AX010621.1 | GM969684.1 | DM204166.1 |
| GM622188.1 | GM841727.1 | HC452134.1 | FW343960.1 | DL022594.1 | HW381036.1 | AX010103.1 | GM968684.1 | HC001592.1 |
| GM622156.1 | GM603720.1 | HC451940.1 | FW343608.1 | DL019579.1 | HW353900.1 | AX002921.1 | GM996537.1 | HB865859.1 |
| GM631015.1 | GM706721.1 | FU756127.1 | FW343544.1 | DL019515.1 | HW297856.1 | A64187.1 | GM970631.1 | HB865369.1 |
| GM630983.1 | GM706689.1 | FU760999.1 | FW343416.1 | DL019483.1 | HW289017.1 | A48488.1 | GM009561.1 | HB865024.1 |
| GM630951.1 | GM706657.1 | DM193210.1 | HC924246.1 | DL019451.1 | HW288943.1 | A38679.1 | DL464791.1 | HB864980.1 |
| GM630919.1 | GM706393.1 | DM199592.1 | HC923064.1 | DL019419.1 | HW288889.1 | A35709.1 | DL464621.1 | HB864984.1 |
| GM630894.1 | GM706358.1 | DM189629.1 | HC921546.1 | DL014984.1 | HW285399.1 | A33046.1 | DL463058.1 | HB864928.1 |
| GM635851.1 | FB742340.1 | HB848653.1 | HC920696.1 | DL014952.1 | HW277191.1 | U87267.1 | DL462996.1 | HB864896.1 |
| GM635819.1 | GM704825.1 | HB847793.1 | HD068938.1 | DL014920.1 | HW267901.1 | A32829.1 | DL462896.1 | HB864864.1 |
| GM635787.1 | GM601154.1 | HB839618.1 | HD068696.1 | DL014888.1 | HW267770.1 | A30331.1 | DL461713.1 | HB864832.1 |
| GM635755.1 | GM600491.1 | HB847597.1 | HD063953.1 | DL014856.1 | HW267706.1 | A23363.1 | GM678825.1 | HB864800.1 |
| GM635723.1 | FB720266.1 | HB847289.1 | HD052878.1 | DL010334.1 | HW267426.1 | U57436.1 | FB509198.1 | HB864768.1 |
| GM635691.1 | FB718514.1 | HB838875.1 | HC920315.1 | DL018288.1 | HW262958.1 | A27462.1 | FB509166.1 | HB866975.1 |
| GM630887.1 | FB718248.1 | HB846712.1 | HD065421.1 | DL018256.1 | HW262853.1 | A16631.1 | GM640932.1 | HB859186.1 |
| GM630855.1 | FB717769.1 | HB838305.1 | HD058871.1 | DL018224.1 | HW262754.1 | A15417.1 | GM047091.1 | HB866515.1 |
| GM630791.1 | FB715242.1 | HB846314.1 | HD057687.1 | DL013822.1 | HW261401.1 | A14316.1 | GM654813.1 | HB866102.1 |
| GM630759.1 | FB677536.1 | HB845990.1 | HD057655.1 | FU265030.1 | HW261337.1 | A07206.1 | GM654781.1 | HC000749.1 |
| GM630695.1 | FB677439.1 | HB845917.1 | HD062469.1 | FU253467.1 | HW261305.1 | A32072.1 | GM647724.1 | HB999503.1 |
| GM621856.1 | GM061157.1 | HB845522.1 | HC306204.1 | FU261569.1 | HW261273.1 | HW249713.1 | FB708879.1 | HB977962.1 |
| GM621824.1 | DL460028.1 | HB826496.1 | HC305968.1 | FU269784.1 | HW261209.1 | HW249641.1 | GM890145.1 | HB976760.1 |
| GM657321.1 | DL459986.1 | HB845344.1 | HC305937.1 | FU258429.1 | HW261177.1 | HW241191.1 | GM963600.1 | HB976610.1 |
| HC473823.1 | DL260590.1 | HB844797.1 | HC305897.1 | FU258365.1 | HW261145.1 | HW247842.1 | GM011262.1 | HB976181.1 |
| FW345511.1 | DL260147.1 | HB844362.1 | HC305857.1 | FU258333.1 | HW261113.1 | HW240740.1 | GM747083.1 | DM193637.1 |
| FW345124.1 | DL260067.1 | HB844105.1 | HC305817.1 | HC311275.1 | HW261081.1 | HW240716.1 | GM008833.1 | DM193343.1 |
| FW344752.1 | HW347574.1 | HB843547.1 | HC305777.1 | DM475254.1 | HW261049.1 | HW241317.1 | GM869523.1 | DM191865.1 |
| FW343999.1 | HW347514.1 | HB836214.1 | HC305737.1 | DM472767.1 | HW261017.1 | HW239395.1 | FB985013.1 | DM189859.1 |
| FW343680.1 | HW347478.1 | HB825657.1 | HC305697.1 | HC299788.1 | HW260921.1 | HW239177.1 | FB983674.1 | DM194479.1 |
| FW343552.1 | HW363683.1 | HB843091.1 | HC305657.1 | HC306252.1 | HW260889.1 | HW238756.1 | FB754306.1 | HB806115.1 |
| FW342638.1 | HW355236.1 | HB835446.1 | HC302014.1 | HC306172.1 | HW260857.1 | HW238563.1 | FB753865.1 | HB821846.1 |
| FW342973.1 | HW355198.1 | HB842898.1 | HC301629.1 | HC302623.1 | HW260825.1 | HW242959.1 | GM865615.1 | HB805744.1 |
| HC921633.1 | HW344737.1 | HB842361.1 | HC294338.1 | HC305864.1 | HW260793.1 | HW237950.1 | FB727478.1 | HB839906.1 |
| HC920701.1 | HW344654.1 | HB842327.1 | HC294062.1 | HC305824.1 | HW260761.1 | HW237895.1 | GM863543.1 | HB848186.1 |
| HD068943.1 | HW344703.1 | HB841707.1 | HC289412.1 | HC305784.1 | HW260729.1 | HW237854.1 | FB725709.1 | HB847926.1 |
| HD068767.1 | HW344523.1 | HB840691.1 | HC289346.1 | HC305744.1 | HW260697.1 | HW248692.1 | GM839523.1 | HB839755.1 |
| HD066658.1 | HW317160.1 | HB828559.1 | HC289314.1 | HC305704.1 | HW260665.1 | HW242506.1 | CS729404.1 | HB839371.1 |
| HD064433.1 | HW317098.1 | HB648624.1 | HC289079.1 | HC305664.1 | HW260633.1 | HW242254.1 | CS728634.1 | HB847378.1 |
| HD063559.1 | HW326518.1 | HB647044.1 | HC296934.1 | HC305621.1 | HW260569.1 | HW243431.1 | CS727346.1 | HB839067.1 |
| HD053020.1 | HW332577.1 | HB645735.1 | HC296007.1 | HA641618.1 | HW260537.1 | HW236725.1 | CS727264.1 | HB832883.1 |
| HD052883.1 | HW338874.1 | HB645703.1 | HC295882.1 | HA641490.1 | HW260505.1 | DJ437107.1 | FB715332.1 | HB846997.1 |
| HD052108.1 | HW338746.1 | HB858335.1 | HC288868.1 | HA635679.1 | HW260473.1 | DJ437064.1 | GM720295.1 | HB838585.1 |
| HC920322.1 | HW338618.1 | HB856359.1 | HC288831.1 | HA639219.1 | HW260441.1 | DJ436986.1 | GM949532.1 | HB840303.1 |
| HD033387.1 | HW338490.1 | HB855781.1 | HC295564.1 | DM102729.1 | HW260377.1 | DJ436833.1 | GM869488.1 | HB846165.1 |
| HD065425.1 | HW338234.1 | DM179946.1 | HC292350.1 | DM092117.1 | HW260345.1 | DJ436664.1 | DL104567.1 | HB837766.1 |

| | | | | | | | | |
|---|---|---|---|---|---|---|---|---|
| HD057691.1 | HW338106.1 | HH998268.1 | HC294912.1 | DM105585.1 | HW260313.1 | DJ436505.1 | DL104535.1 | HB808929.1 |
| HD057659.1 | HW337978.1 | HH998231.1 | HC299295.1 | DM091575.1 | HW260281.1 | DJ444604.1 | DL104315.1 | HB845654.1 |
| HD062500.1 | HW337850.1 | HH998184.1 | HC294672.1 | DM095101.1 | HW260249.1 | DJ443530.1 | DL085791.1 | HB845411.1 |
| HD061464.1 | HW337722.1 | HH998130.1 | HC291150.1 | GN367623.1 | HW260153.1 | DJ438360.1 | DL033950.1 | HB837207.1 |
| M24888.1 | HW337210.1 | HH993676.1 | HC290970.1 | GN367550.1 | HW260121.1 | DJ438354.1 | DL025211.1 | HB813985.1 |
| K02873.1 | HW337082.1 | HH986625.1 | HC293553.1 | GN370762.1 | HW260089.1 | DJ438287.1 | DL029520.1 | HB844532.1 |
| M35591.1 | HW336954.1 | HH979515.1 | HC290593.1 | GN374413.1 | HW260057.1 | DJ438205.1 | DL029488.1 | HB844277.1 |
| HC918557.1 | HW321650.1 | HH979551.1 | HC289876.1 | GN360098.1 | HW260025.1 | DJ427970.1 | DL041880.1 | DM091324.1 |
| FW334602.1 | HW321193.1 | HH979622.1 | HC289842.1 | GN356167.1 | HW259993.1 | DJ402680.1 | DL028122.1 | DM068817.1 |
| FW342472.1 | HW336462.1 | HH979580.1 | HC207905.1 | GN366509.1 | HW259961.1 | DJ402642.1 | DL028090.1 | GN087806.1 |
| HC305782.1 | HW329372.1 | HH931983.1 | HC207650.1 | GN366444.1 | HW259850.1 | DJ402544.1 | DL028058.1 | GN087443.1 |
| HC305702.1 | HW336349.1 | FW394390.1 | HC204107.1 | GN366380.1 | HW258938.1 | DJ402300.1 | DL028026.1 | GN094074.1 |
| HC305662.1 | HW335982.1 | FW394291.1 | DL111836.1 | GN366348.1 | HW258827.1 | DJ393081.1 | DL027994.1 | GN089868.1 |
| HC301310.1 | HW328693.1 | FW394234.1 | DL111804.1 | GN360025.1 | HW257412.1 | DJ419969.1 | DL027962.1 | GN089804.1 |
| HC291426.1 | HW328604.1 | FW394198.1 | DL111772.1 | GN359993.1 | HW257380.1 | DJ419223.1 | DL044259.1 | GN082897.1 |
| HC294110.1 | HW328358.1 | FW397845.1 | DL128887.1 | GN359961.1 | HV601489.1 | DJ417435.1 | DL044227.1 | GN089199.1 |
| HC289417.1 | HW328286.1 | FW418627.1 | DL128820.1 | GN359929.1 | HV574858.1 | DJ402725.1 | DL044195.1 | GN082826.1 |
| HC289350.1 | HW328094.1 | FW418233.1 | DL128755.1 | GN359897.1 | HV571038.1 | DJ387698.1 | DL044163.1 | GM635659.1 |
| HC289318.1 | HW318503.1 | FW396873.1 | DL116419.1 | GN366323.1 | HV573968.1 | DJ381285.1 | DD491147.1 | GM635627.1 |
| HC289117.1 | HW335189.1 | HC294230.1 | DL116387.1 | GN366259.1 | HV573158.1 | DJ381036.1 | CS671010.1 | GM635595.1 |
| HC292792.1 | HW314068.1 | HC291378.1 | DL116355.1 | GN359788.1 | HV559926.1 | DJ381004.1 | CS669861.1 | GM635563.1 |
| HC296033.1 | HW313987.1 | HC289381.1 | DL111634.1 | GN359756.1 | HV555942.1 | DJ380972.1 | CS673606.1 | GM635531.1 |
| HC288872.1 | HW159072.1 | HC289337.1 | DL111602.1 | GN359724.1 | HV561912.1 | DJ380940.1 | DD463391.1 | GM635499.1 |
| HC288839.1 | HW154648.1 | HC292780.1 | DL111538.1 | DM006720.1 | HV554550.1 | DJ380908.1 | CS647381.1 | GM635467.1 |
| HC295586.1 | HW154481.1 | HC292482.1 | DL111506.1 | DM004968.1 | HV553175.1 | DJ380876.1 | CS646215.1 | GM630661.1 |
| HC295508.1 | HW154449.1 | HC292242.1 | DL101692.1 | DM003503.1 | HV551402.1 | DJ386143.1 | CS644284.1 | GM630629.1 |
| HC295444.1 | HW147483.1 | HC294984.1 | DL101660.1 | DL489800.1 | HV550395.1 | DJ380824.1 | DD459653.1 | GM630597.1 |
| HC288601.1 | HW147439.1 | HC294804.1 | DL118048.1 | DL489743.1 | HV550346.1 | DJ389524.1 | DD458768.1 | GM621662.1 |
| HC295145.1 | HW154313.1 | HC294564.1 | DL118016.1 | DM003086.1 | AY659397.1 | DJ389306.1 | DD453841.1 | GM621630.1 |
| HC292002.1 | HW154025.1 | HC298733.1 | DL113359.1 | DM008736.1 | AY659365.1 | DJ388693.1 | DD453511.1 | GM621598.1 |
| HC291834.1 | HW146915.1 | HC291042.1 | DL103632.1 | GM997934.1 | AY659333.1 | DJ388554.1 | DD456638.1 | GM621566.1 |
| HC291198.1 | HW153763.1 | HC290857.1 | DL099026.1 | GM996592.1 | AY659301.1 | DJ388512.1 | DD451634.1 | GM657114.1 |
| HC298111.1 | HW153723.1 | HC293445.1 | DL098994.1 | GM994842.1 | AY659269.1 | DJ388464.1 | CS631744.1 | GM657082.1 |
| HC293589.1 | HW153475.1 | HC293325.1 | DL098962.1 | GM994327.1 | AY659237.1 | CS725991.1 | CS631250.1 | GM657050.1 |
| HC290641.1 | HW145518.1 | HC293128.1 | DL089017.1 | GM992639.1 | DL097939.1 | CS724457.1 | CS631186.1 | GM657018.1 |
| HC290171.1 | HW145137.1 | HC289865.1 | DL088985.1 | GM992336.1 | DL112629.1 | CS723021.1 | CS628282.1 | GM656986.1 |
| HC289880.1 | HW153265.1 | HC289688.1 | DL117898.1 | GM992266.1 | DL112597.1 | CS176634.1 | CS627821.1 | GM656954.1 |
| HC289846.1 | HW157345.1 | HC207401.1 | DL117866.1 | GM992111.1 | DL112565.1 | CS172834.1 | CS632194.1 | GM656923.1 |
| HC207910.1 | HW152007.1 | HC199624.1 | DL117834.1 | GM991909.1 | DL095725.1 | CS166441.1 | CS626244.1 | GM656859.1 |
| HC207184.1 | GN030860.1 | DM464642.1 | DL108152.1 | GM976279.1 | DL095693.1 | CS159801.1 | DD450045.1 | GM656827.1 |
| HC201310.1 | GN030828.1 | DM462711.1 | DL108088.1 | GM974814.1 | DL095661.1 | CS159574.1 | DD438017.1 | GM656795.1 |
| HC199334.1 | GN030796.1 | HC193437.1 | DL098780.1 | GM990786.1 | DL095629.1 | CS159198.1 | DD449164.1 | GM656763.1 |
| DM464965.1 | GN031163.1 | HC190457.1 | DL098748.1 | GM986816.1 | DL095597.1 | CS158044.1 | CS618399.1 | GM649770.1 |
| HC196127.1 | DM008307.1 | HC089573.1 | DL092738.1 | GN000633.1 | DL095565.1 | CS155636.1 | CS616583.1 | GM649738.1 |
| HC194240.1 | DM002013.1 | HC089541.1 | DL092706.1 | GM969705.1 | DL117402.1 | CS148559.1 | CS612722.1 | GM649706.1 |
| HC193963.1 | DM001475.1 | HC089509.1 | DL088963.1 | GM969589.1 | DL104895.1 | CS144348.1 | CS611722.1 | GM649674.1 |
| HC190865.1 | DM006958.1 | HC089477.1 | DL088931.1 | GM969398.1 | DL104831.1 | CS141576.1 | CS604499.1 | GM649642.1 |

| | | | | | | | | |
|---|---|---|---|---|---|---|---|---|
| HC187501.1 | DM006719.1 | HC089445.1 | DL021728.1 | GM969934.1 | DL095908.1 | CS141527.1 | CS243760.1 | GM649610.1 |
| HC187070.1 | DM004991.1 | HC089412.1 | DL021696.1 | GN007497.1 | DL095876.1 | CS141495.1 | CS243149.1 | GM642706.1 |
| HC089586.1 | DM004967.1 | HC089380.1 | DL021664.1 | FB986395.1 | DL114427.1 | CS141126.1 | CS242342.1 | GM642674.1 |
| HC089554.1 | DM003502.1 | HC089348.1 | DL021632.1 | FB985978.1 | DL114395.1 | CS140534.1 | CS240018.1 | GM642642.1 |
| HC089522.1 | DL489798.1 | HC089315.1 | DL017330.1 | GM969083.1 | DL114363.1 | CS132038.1 | CS227312.1 | GM635332.1 |
| HC089490.1 | DM003085.1 | HC089283.1 | DL012941.1 | GM968451.1 | DL104572.1 | CS132439.1 | CS208079.1 | GM635300.1 |
| HC089458.1 | DL488940.1 | HC089251.1 | DL041349.1 | GM970609.1 | DL104540.1 | CS123553.1 | CS207905.1 | GM635268.1 |
| HC089426.1 | DL488541.1 | HC087739.1 | DL041285.1 | GM970172.1 | DL104508.1 | CS122493.1 | CS189612.1 | GM630462.1 |
| HC089393.1 | GM998424.1 | HC086500.1 | DL037398.1 | FB736150.1 | DL126659.1 | CS179740.1 | CS193199.1 | GM630430.1 |
| HC089361.1 | GM997933.1 | AY774937.1 | DL037366.1 | GM969738.1 | DL123340.1 | CS323676.1 | FW420419.1 | GM630398.1 |
| HC089328.1 | GM983618.1 | AY774897.1 | DL037334.1 | GM009517.1 | DL123308.1 | CS323594.1 | FW420394.1 | GM630334.1 |
| HC089296.1 | GM996591.1 | AY774835.1 | DL021489.1 | DL480004.1 | DL017623.1 | CS330145.1 | FW420362.1 | GM630302.1 |
| HC089264.1 | GM995266.1 | AY774774.1 | DL021457.1 | DL465754.1 | DL022293.1 | CS329667.1 | FW420330.1 | GM621495.1 |
| HC085664.1 | GM994841.1 | AY774719.1 | DL021425.1 | DL477845.1 | DL022261.1 | CS322801.1 | FW496533.1 | GM702762.1 |
| AY774914.1 | GM992856.1 | AY774375.1 | DL017187.1 | DL464725.1 | DL022229.1 | CS322142.1 | HI653998.1 | GM656621.1 |
| AY774689.1 | GM992474.1 | AY774321.1 | DL017155.1 | DL464563.1 | DL025216.1 | DD259346.1 | HI653735.1 | GM656589.1 |
| GN359670.1 | GM992404.1 | AY774273.1 | DL017123.1 | DL470948.1 | DL038354.1 | DD266548.1 | HI646935.1 | GM656557.1 |
| GN359638.1 | GM623405.1 | AY774218.1 | DL017091.1 | DL470401.1 | DL038322.1 | DD265346.1 | AY145515.1 | GM649564.1 |
| GN359606.1 | GM623373.1 | AY774155.1 | DL017059.1 | GM622453.1 | DL013159.1 | DQ387204.1 | HH807103.1 | GM649468.1 |
| GN359510.1 | GM623341.1 | GN052373.1 | DL017027.1 | GM622421.1 | DL013127.1 | CS297376.1 | HH979569.1 | GM649436.1 |
| GN359410.1 | GM643892.1 | GN051255.1 | DL049426.1 | GM622389.1 | DL013095.1 | DD251992.1 | FW394281.1 | GM649404.1 |
| GN359282.1 | GM643860.1 | DM010491.1 | DL049362.1 | GM622357.1 | DL013031.1 | DD257964.1 | FW394262.1 | GM670044.1 |
| GN365279.1 | GM636831.1 | DM023755.1 | DL049330.1 | GM631345.1 | DL012999.1 | DD161532.1 | FW394224.1 | GM656539.1 |
| GN359273.1 | GM636799.1 | DM011433.1 | DL039969.1 | GM631313.1 | DL042072.1 | DD161461.1 | FW394188.1 | GM656507.1 |
| GN359177.1 | GM636767.1 | DM021913.1 | DL039937.1 | GM703823.1 | DL042965.1 | DD167022.1 | FW396805.1 | GM656475.1 |
| GN359145.1 | GM632263.1 | DM021895.1 | DL039905.1 | GM657511.1 | DL042933.1 | DD166740.1 | FW396384.1 | GM656443.1 |
| GN359113.1 | GM632199.1 | DM026464.1 | DL047852.1 | GM634372.1 | DL042901.1 | DD165147.1 | HH933766.1 | GM656411.1 |
| GN359081.1 | GM632167.1 | DM026244.1 | DL047820.1 | GM634340.1 | DL030902.1 | DD182267.1 | HH928906.1 | GM656379.1 |
| GN359051.1 | GM632135.1 | GN041713.1 | DL047788.1 | GM634308.1 | DL030735.1 | DD159555.1 | HH931924.1 | GM649304.1 |
| GN363569.1 | GM632103.1 | GN041518.1 | DL047756.1 | GM634276.1 | DL022954.1 | DD181658.1 | HH827035.1 | GM649314.1 |
| GN363032.1 | GM623332.1 | GN037858.1 | DL047724.1 | GM629448.1 | DL019747.1 | DD163505.1 | HH826938.1 | GM649258.1 |
| DD038306.1 | GM623300.1 | GN034811.1 | DL047692.1 | GM629449.1 | DL019715.1 | CS000420.1 | HC679160.1 | GM649226.1 |
| DD037076.1 | GM623268.1 | GN030702.1 | DL047660.1 | GM629407.1 | DL010534.1 | AF408176.1 | HC678799.1 | GM649194.1 |
| DD032625.1 | GM623236.1 | GN030638.1 | DL043678.1 | GM629375.1 | DL010502.1 | CS157797.1 | HC510434.1 | GM642585.1 |
| DD032488.1 | GM741842.1 | GN030606.1 | DL039701.1 | GM629343.1 | DL010470.1 | CS157958.1 | HC508656.1 | GM642553.1 |
| DD031271.1 | GM741810.1 | GN030574.1 | DL035917.1 | GM629311.1 | DL010438.1 | CS157926.1 | HC494795.1 | GM642521.1 |
| DD030747.1 | GM658324.1 | GN030542.1 | DL027848.1 | GM655631.1 | DL010406.1 | CS157894.1 | HC502245.1 | GM642456.1 |
| DD026780.1 | GM658292.1 | GN030510.1 | DL027816.1 | GM655599.1 | DL038780.1 | CS157862.1 | HC499876.1 | GM652783.1 |
| DD025946.1 | GM658260.1 | GN030455.1 | DL027784.1 | GM655567.1 | DL038748.1 | CS146875.1 | HC499843.1 | GM645689.1 |
| DD024026.1 | GM658228.1 | GN030286.1 | DL027752.1 | GM648574.1 | DL038716.1 | CS124719.1 | FW300634.1 | GM645657.1 |
| DD023830.1 | GM658196.1 | GN030254.1 | DL023844.1 | GM648542.1 | DL035092.1 | CS124679.1 | FW298891.1 | GM645625.1 |
| DD023371.1 | GM658164.1 | GN030222.1 | DL023812.1 | GM639068.1 | DL034964.1 | CQ857739.1 | FW304267.1 | GM638826.1 |
| DD029704.1 | GM658114.1 | GN030190.1 | DL016202.1 | GM626521.1 | DD432222.1 | CQ856032.1 | HC469547.1 | GM638794.1 |
| DD028790.1 | GM658082.1 | GN033538.1 | DL016170.1 | GM626489.1 | DD431752.1 | CQ855927.1 | HC486530.1 | GM638762.1 |
| DD010451.1 | GM658050.1 | GN033506.1 | DL016138.1 | GM626457.1 | CS531815.1 | CQ854354.1 | HC475394.1 | GM626119.1 |
| DD010248.1 | GM658018.1 | GN033474.1 | DL031638.1 | GM626393.1 | CS537858.1 | CQ849499.1 | HC491772.1 | GM626087.1 |
| DD019565.1 | GM657986.1 | GN033442.1 | DL031606.1 | GM626361.1 | CS502632.1 | CQ849243.1 | HC491740.1 | GM626055.1 |

| | | | | | | | | |
|---|---|---|---|---|---|---|---|---|
| DD017742.1 | GM657954.1 | GN033410.1 | DL031574.1 | GM626329.1 | CS502504.1 | CQ834936.1 | DM156351.1 | GM626023.1 |
| DD017403.1 | GM650762.1 | GN033346.1 | DL031542.1 | GM660418.1 | CS501512.1 | AX593518.1 | DM155736.1 | GM625991.1 |
| DD009540.1 | GM650730.1 | GM643111.1 | DL023665.1 | GM660354.1 | CS500248.1 | AX587938.1 | HB463480.1 | GM625959.1 |
| BD495447.1 | GM650698.1 | GM643047.1 | DL023633.1 | GM653167.1 | CS498455.1 | AX587643.1 | HB444717.1 | GM037323.1 |
| BD495241.1 | GM650602.1 | GM643015.1 | DL015991.1 | GM653135.1 | DD417814.1 | BD162040.1 | DM121738.1 | FB776240.1 |
| BD454038.1 | GM643706.1 | FB506774.1 | DL015959.1 | GM653103.1 | CS490586.1 | BD161070.1 | DM137794.1 | FB774894.1 |
| BD434096.1 | GM643674.1 | AY658977.1 | DJ400832.1 | GM653071.1 | CS489136.1 | BD160749.1 | HB427184.1 | FB766234.1 |
| BD453901.1 | GM643634.1 | AY658945.1 | DJ400800.1 | GM653039.1 | CS486439.1 | BD143481.1 | HB426588.1 | FB764672.1 |
| BD453869.1 | GM636645.1 | AY658913.1 | DJ399773.1 | GM653007.1 | CS482993.1 | AX587902.1 | HB403643.1 | FB743934.1 |
| BD453837.1 | GM636613.1 | AY658881.1 | DJ391349.1 | GM639013.1 | CS482958.1 | AX587805.1 | HB396579.1 | FB743902.1 |
| BD453805.1 | GM636581.1 | AY658849.1 | DJ390560.1 | GM638981.1 | CS482876.1 | AX587698.1 | DM119571.1 | FB743870.1 |
| BD453749.1 | GM636549.1 | AY658817.1 | DJ402722.1 | GM638949.1 | DD401428.1 | AX587598.1 | GM833981.1 | FB743838.1 |
| BD453717.1 | GM636517.1 | AY658785.1 | DJ381525.1 | GM638917.1 | DD401396.1 | AX573872.1 | GM624418.1 | FB743784.1 |
| BD453685.1 | GM636485.1 | AY658753.1 | DJ386553.1 | GM638885.1 | DD405211.1 | AX572937.1 | GM624244.1 | FB761596.1 |
| BD453653.1 | GM632077.1 | AY658721.1 | DJ381033.1 | GM638853.1 | DD405179.1 | AX556854.1 | GM741818.1 | GM720301.1 |
| BD453621.1 | GM623114.1 | AY658689.1 | DJ381001.1 | GM626174.1 | DD405147.1 | AX556820.1 | GM623058.1 | GM036117.1 |
| BD453584.1 | GM623082.1 | AY658657.1 | DJ380969.1 | GM626142.1 | DD405115.1 | AX555189.1 | FB508045.1 | GM035292.1 |
| BD453571.1 | GM623050.1 | AY658625.1 | DJ380937.1 | GM660181.1 | CS123416.1 | AX554278.1 | FB708236.1 | GM952644.1 |
| BD453539.1 | GM623018.1 | AY658593.1 | DL036569.1 | GM660149.1 | CS122932.1 | AX544165.1 | FB667746.1 | GM952298.1 |
| BD453507.1 | GM622986.1 | AY658561.1 | DL036537.1 | GM652975.1 | CS122385.1 | AX538883.1 | FB670942.1 | GM951785.1 |
| BD453475.1 | GM622954.1 | AY658529.1 | DL032238.1 | GM652943.1 | CS106160.1 | AX538319.1 | FB706178.1 | GM890250.1 |
| BD453443.1 | GM650472.1 | AY658497.1 | DL032174.1 | GM652911.1 | CS106067.1 | AX538261.1 | FB705602.1 | GM963580.1 |
| BD453411.1 | GM650440.1 | AY658465.1 | DL048444.1 | GM652879.1 | CS179704.1 | AX536404.1 | FB701532.1 | GM889324.1 |
| BD442994.1 | GM650408.1 | AY658433.1 | DL048412.1 | GM652847.1 | CS179213.1 | AX534752.1 | FB701791.1 | DL119604.1 |
| BD453234.1 | GM643600.1 | AY658401.1 | DL048380.1 | GM652815.1 | CS327372.1 | AF302189.1 | FB292671.1 | DL115009.1 |
| BD453202.1 | GM643536.1 | AY658369.1 | DL048348.1 | GM652782.1 | CS326339.1 | AF105013.1 | FB665384.1 | DL114977.1 |
| BD442863.1 | GM643504.1 | AY658337.1 | DL044270.1 | GM645688.1 | CS323584.1 | S71745.1 | FB654383.1 | DL114913.1 |
| BD399950.1 | GM643472.1 | AY658305.1 | DL014216.1 | GM645624.1 | CS323286.1 | AF359363.1 | FB654895.1 | DL114881.1 |
| BD410200.1 | GM643439.1 | AY658273.1 | DL014184.1 | GM638825.1 | CS330526.1 | HW408477.1 | FB584254.1 | DL114849.1 |
| BD410027.1 | GM636450.1 | AY658241.1 | DL014152.1 | GM638793.1 | CS329498.1 | HW408446.1 | FB583305.1 | DL123929.1 |
| BD497562.1 | GM636418.1 | AY658209.1 | DL014120.1 | GM638761.1 | DD261105.1 | HW390749.1 | DL202571.1 | DL123897.1 |
| BD495912.1 | GM636354.1 | AY658177.1 | DL014001.1 | GM626118.1 | DD259337.1 | HW399322.1 | DL188836.1 | DL123865.1 |
| BD430791.1 | GM631850.1 | AY658145.1 | DL013969.1 | GM626086.1 | DD272522.1 | HW399224.1 | DL193807.1 | DL123801.1 |
| BD450799.1 | GM654438.1 | AY658113.1 | DL013937.1 | GM626054.1 | CS302650.1 | HW408996.1 | DL193775.1 | DL123769.1 |
| BD408109.1 | GM654406.1 | AY658081.1 | DL030037.1 | GM626022.1 | CS297211.1 | HW389147.1 | FB571340.1 | DL119590.1 |
| BD408077.1 | GM647349.1 | AY658049.1 | DL029973.1 | GM625990.1 | DD251648.1 | HW388972.1 | CS585196.1 | DL119558.1 |
| BD429795.1 | GM647317.1 | AY658017.1 | DL046630.1 | GM625958.1 | CS287615.1 | HW388632.1 | CS588639.1 | DL119526.1 |
| BD449656.1 | GM647285.1 | AY657985.1 | DL046598.1 | GM660128.1 | DD240739.1 | HW387446.1 | CS574931.1 | DL119494.1 |
| BD429574.1 | GM647253.1 | AY657953.1 | DL046566.1 | GM037785.1 | DD247579.1 | HW380845.1 | CS573053.1 | DL119462.1 |
| BD407505.1 | GM647221.1 | AY657921.1 | DL046534.1 | GM037322.1 | DD246913.1 | HW379426.1 | CS570690.1 | DL119430.1 |
| AX798910.1 | GM627711.1 | AY657889.1 | DL042648.1 | FB776239.1 | AX417904.1 | HW104302.1 | CS546670.1 | DL123741.1 |
| AX798216.1 | GM627679.1 | AY657857.1 | DL042616.1 | FB775619.1 | AX407059.1 | HW071178.1 | CS540027.1 | DL123709.1 |
| AX797705.1 | GM627647.1 | AY657825.1 | DL042584.1 | FB774892.1 | AX402420.1 | HW070970.1 | CS541738.1 | DL123677.1 |
| AX796930.1 | GM870256.1 | AY657793.1 | DL042668.1 | FB764709.1 | AX399447.1 | HW104220.1 | CS538928.1 | DL123645.1 |
| AX796749.1 | GM627555.1 | AY657761.1 | DL038660.1 | FB764671.1 | AX398661.1 | HW104108.1 | CS498543.1 | DL123613.1 |
| AX795590.1 | DL017033.1 | AY657729.1 | DL034131.1 | FB747041.1 | AX397875.1 | HW070572.1 | CS498439.1 | DL123581.1 |
| AX795558.1 | DL049432.1 | AY657697.1 | DL029838.1 | FB744811.1 | AX395206.1 | HW099603.1 | CS495828.1 | DL119391.1 |

| | | | | | | | | |
|---|---|---|---|---|---|---|---|---|
| AX795437.1 | DL049400.1 | AY657665.1 | DL029742.1 | FB743933.1 | AX392371.1 | HW069634.1 | DD418592.1 | DL119359.1 |
| BD182680.1 | DL049368.1 | AY657633.1 | DL038483.1 | FB743869.1 | AX384569.1 | HW066588.1 | DD236704.1 | DL119327.1 |
| BD189024.1 | DL049336.1 | AY657601.1 | DL018279.1 | FB743837.1 | AX380806.1 | HW066158.1 | DD246853.1 | DL114835.1 |
| AX777464.1 | DL049081.1 | AY657569.1 | DL018247.1 | FB743783.1 | AX375509.1 | HW081769.1 | DD241171.1 | DL114803.1 |
| AX774561.1 | DL049049.1 | AY657537.1 | DL018215.1 | FB761736.1 | AX370389.1 | HW081678.1 | E41533.1 | DL114771.1 |
| AX768200.1 | DL012573.1 | AY657505.1 | DL013781.1 | FB761594.1 | AX364553.1 | HW085168.1 | E39971.1 | DL114739.1 |
| AX766875.1 | DL012541.1 | AY657473.1 | DL013749.1 | FB761252.1 | AX364521.1 | HW099458.1 | E40758.1 | DL114707.1 |
| AX766728.1 | DL012509.1 | AY657441.1 | DL013717.1 | FB760406.1 | AX364489.1 | HW103522.1 | E38472.1 | DL114675.1 |
| AX766380.1 | DL012477.1 | AY657409.1 | DL013685.1 | DL102103.1 | AX364457.1 | HW098199.1 | E33088.1 | DL127933.1 |
| AX765958.1 | DL012413.1 | AY657377.1 | DL005434.1 | DL102071.1 | HW380837.1 | HW102320.1 | E05860.1 | DL141501.1 |
| AX752655.1 | DL045455.1 | AY657345.1 | DL008702.1 | DL102039.1 | HW375063.1 | HW061883.1 | E05529.1 | DL141433.1 |
| AX752386.1 | DL045423.1 | AY657313.1 | DL008499.1 | DL102007.1 | HW381519.1 | HW061850.1 | E05010.1 | DL123541.1 |
| AX752035.1 | DL045391.1 | AY657281.1 | CS603453.1 | DL101975.1 | HW381032.1 | HW065298.1 | E04536.1 | DL123509.1 |
| BD180804.1 | DL045359.1 | AY657249.1 | CS603421.1 | DL101943.1 | HW352832.1 | HW065265.1 | E04200.1 | DL123477.1 |
| AX744001.1 | DL041247.1 | AY657217.1 | CS603389.1 | DL097529.1 | HW352480.1 | HW064873.1 | E03810.1 | DL123445.1 |
| AX742849.1 | DL041215.1 | AY657185.1 | CS603357.1 | DL097497.1 | HW151065.1 | HW064834.1 | GM741846.1 | DL123413.1 |
| AX739887.1 | DL041183.1 | AY657153.1 | CS603325.1 | DL097465.1 | HW159790.1 | HW061119.1 | GM741814.1 | DL123381.1 |
| AX721696.1 | DL041151.1 | AY657121.1 | CS603293.1 | DL121292.1 | HW155982.1 | HW058212.1 | GM658328.1 | DL119191.1 |
| BD178181.1 | DL041119.1 | AY657089.1 | CS603261.1 | DL121260.1 | HW144717.1 | HV504201.1 | GM658296.1 | DL119159.1 |
| AX720804.1 | DL037200.1 | AY657057.1 | CS603229.1 | DL121228.1 | HW126600.1 | HV455585.1 | GM658232.1 | DL119127.1 |
| AX717588.1 | DL037168.1 | M15940.1 | CS603197.1 | DL121196.1 | HW126453.1 | HV504165.1 | GM658200.1 | DL119095.1 |
| AX708503.1 | DL037136.1 | M10650.1 | CS603165.1 | DL101890.1 | HW125395.1 | HV504133.1 | GM658168.1 | DL119063.1 |
| AX145540.1 | DL018382.1 | M16227.1 | CS603133.1 | DL101826.1 | HW144364.1 | HV504101.1 | GM650847.1 | DL119031.1 |
| AX145508.1 | DL018350.1 | M12688.1 | CS603101.1 | DL101794.1 | HW144300.1 | HV504069.1 | GM650783.1 | DL114635.1 |
| AX145476.1 | DL018318.1 | M16485.1 | CS603069.1 | DL101762.1 | HW124784.1 | HV504037.1 | GM658086.1 | DL041563.1 |
| AX145444.1 | DL018286.1 | HV549912.1 | CS603037.1 | DL101730.1 | HW122422.1 | HV504005.1 | GM658054.1 | DL041531.1 |
| AX145412.1 | DL018254.1 | HV549747.1 | CS603005.1 | DL097220.1 | HW120878.1 | HV503973.1 | GM658022.1 | DL041499.1 |
| AX145379.1 | DL018222.1 | HV549108.1 | CS602971.1 | DL097188.1 | HW117994.1 | HV503941.1 | GM657990.1 | DL037676.1 |
| AX145347.1 | DL013820.1 | HV544227.1 | CS602939.1 | DL097156.1 | HW115611.1 | HV503877.1 | GM657958.1 | DL037644.1 |
| AX145315.1 | DL013788.1 | FW308825.1 | CS602907.1 | DL095328.1 | HW081475.1 | HV503773.1 | GM650766.1 | DL037612.1 |
| AX145283.1 | DL013756.1 | FW307792.1 | CS602875.1 | DL095296.1 | HW101675.1 | HV503677.1 | GM650734.1 | DL037580.1 |
| AX145251.1 | DL013724.1 | FW332373.1 | CS602843.1 | DL095232.1 | HW096985.1 | HV503645.1 | GM650702.1 | DL037548.1 |
| AX145219.1 | DL013692.1 | FW310644.1 | CS602811.1 | GM657256.1 | HV757226.1 | HV503178.1 | GM650670.1 | DL037516.1 |
| AX145187.1 | DL000815.1 | HC768263.1 | CS602779.1 | GM657224.1 | HV758703.1 | HV503146.1 | GM650638.1 | DL033313.1 |
| AX145155.1 | DL000575.1 | HC767369.1 | CS602747.1 | GM657192.1 | HV745360.1 | HV503114.1 | GM650606.1 | DL033281.1 |
| AX145123.1 | DJ493973.1 | HC757170.1 | CS602715.1 | GM641272.1 | HV747492.1 | HV503082.1 | GM643774.1 | DL033249.1 |
| AX145091.1 | DJ493815.1 | HC757138.1 | CS602683.1 | GM641240.1 | HV753518.1 | HV502782.1 | GM643742.1 | DL033217.1 |
| HV450039.1 | DL008721.1 | HC755798.1 | CS602171.1 | GM629049.1 | HV743216.1 | HV502750.1 | GM643710.1 | DL033185.1 |
| HV451951.1 | DL008506.1 | HC754645.1 | CS608444.1 | GM629017.1 | HV704363.1 | HV502718.1 | GM643678.1 | DL033153.1 |
| HV449867.1 | DJ491888.1 | HC741958.1 | CS607948.1 | GM628985.1 | HV704239.1 | HV502686.1 | GM643638.1 | DL033121.1 |
| HV452996.1 | DJ491561.1 | HC732133.1 | CS607668.1 | GM628953.1 | HV704162.1 | HV502654.1 | GM636649.1 | DL029113.1 |
| HV452881.1 | DL002526.1 | HC731666.1 | CS607527.1 | GM628921.1 | HV703914.1 | HV502622.1 | GM636617.1 | DL029081.1 |
| HV445485.1 | DJ446860.1 | HC471760.1 | CS607457.1 | GM881898.1 | HV703158.1 | HV502590.1 | GM636585.1 | DL029049.1 |
| HV448961.1 | DJ446827.1 | HC471728.1 | CS607178.1 | GM655227.1 | FZ430304.1 | HV502526.1 | GM636553.1 | DL029017.1 |
| HV450926.1 | DJ438105.1 | HC471664.1 | CS607074.1 | GM655195.1 | FZ430133.1 | HV502494.1 | GM636521.1 | DL028985.1 |
| HV443959.1 | DJ437356.1 | HC471632.1 | CS606971.1 | GM655163.1 | FZ437139.1 | HV505301.1 | GM636489.1 | DL028953.1 |
| HV350012.1 | DJ437275.1 | DM381907.1 | CS606908.1 | GM648170.1 | FZ422934.1 | HV505198.1 | GM636457.1 | DL021767.1 |

| | | | | | | | | |
|---|---|---|---|---|---|---|---|---|
| HV437583.1 | DJ437177.1 | HC055551.1 | CS606835.1 | GM641207.1 | FZ415474.1 | HV494719.1 | GM632049.1 | DL021735.1 |
| HV437542.1 | DJ437147.1 | HC045300.1 | CS605016.1 | GM641143.1 | FZ415403.1 | HV452150.1 | GM632017.1 | DL021703.1 |
| HV341322.1 | DJ436990.1 | HC047030.1 | CS604728.1 | GM641079.1 | FZ421517.1 | HV453606.1 | GM631985.1 | DL021671.1 |
| HV343886.1 | DJ436841.1 | HC046998.1 | CS593081.1 | GM641047.1 | FZ417513.1 | HV450073.1 | GM631953.1 | DL021639.1 |
| HV343694.1 | DJ436508.1 | HC046966.1 | CS589756.1 | GM628887.1 | FZ419845.1 | HV455252.1 | GM631921.1 | DL017401.1 |
| HV341054.1 | DJ431118.1 | HC046934.1 | DD158364.1 | GM628855.1 | FZ419811.1 | HV448935.1 | GM631889.1 | DL017369.1 |
| HV343550.1 | DJ445074.1 | HC046902.1 | DD157347.1 | GM628823.1 | FW582231.1 | HH794947.1 | GM623118.1 | DL017337.1 |
| HV347675.1 | DJ444568.1 | HC046874.1 | DD155705.1 | GM628791.1 | FW590753.1 | HC045807.1 | GM623086.1 | DL012980.1 |
| HV344878.1 | DJ443533.1 | HC046842.1 | DD152586.1 | GM628679.1 | FW589143.1 | HC045743.1 | GM623054.1 | DL045628.1 |
| HI001057.1 | DJ434340.1 | HC046810.1 | DD151916.1 | GM628647.1 | HI642657.1 | HC045711.1 | GM623022.1 | DL045596.1 |
| HI001018.1 | DJ438363.1 | HC046778.1 | DD083068.1 | GM628615.1 | HI637690.1 | HC045681.1 | GM622990.1 | DL045564.1 |
| HI000976.1 | DJ438357.1 | HC046746.1 | DD096866.1 | GM628583.1 | HI380635.1 | DM378906.1 | GM622958.1 | DL045532.1 |
| HI002880.1 | DJ438290.1 | DL092315.1 | DD082122.1 | GM628551.1 | HI379165.1 | HC035663.1 | GM650540.1 | DL045468.1 |
| HI002842.1 | DJ427981.1 | DL092283.1 | DD081750.1 | GM628519.1 | HI377950.1 | HC037098.1 | GM650476.1 | DL041356.1 |
| HI002804.1 | DJ428071.1 | DL092251.1 | DD147753.1 | GM716099.1 | HI376187.1 | HC036994.1 | GM650444.1 | DL041324.1 |
| HI002745.1 | DJ402683.1 | DL092219.1 | DD147721.1 | GM662547.1 | HI375910.1 | HC022622.1 | GM650412.1 | DL041292.1 |
| HI002710.1 | DJ402547.1 | DL092187.1 | DD147689.1 | GM654969.1 | HI373236.1 | HC025499.1 | GM643604.1 | DL037405.1 |
| HI003264.1 | DJ402297.1 | DL121908.1 | DD147097.1 | GM640916.1 | HI369150.1 | HC025467.1 | GM643572.1 | DL037373.1 |
| HI002661.1 | DJ400838.1 | FB316584.1 | DD139493.1 | GM640884.1 | HI369097.1 | HC025403.1 | GM643540.1 | DL037341.1 |
| HI002625.1 | DJ400806.1 | FB332443.1 | DD080832.1 | GM640852.1 | HI424125.1 | HC010421.1 | GM643508.1 | DL021496.1 |
| HI002590.1 | DJ419977.1 | CS368430.1 | DD138753.1 | GM628498.1 | HI424093.1 | HC010328.1 | GM643476.1 | DL021464.1 |
| HI002530.1 | DJ415868.1 | CS368302.1 | DD132347.1 | GM662111.1 | HI424061.1 | HC007705.1 | GM643443.1 | DL021432.1 |
| HI001480.1 | DJ419611.1 | CS368174.1 | DD132328.1 | GM654829.1 | HI423803.1 | HC007673.1 | GM643411.1 | DL021400.1 |
| HI004545.1 | CS502450.1 | DJ327003.1 | DD104233.1 | GM654797.1 | HH996577.1 | HC007641.1 | GM636422.1 | DL017194.1 |
| HI004417.1 | CS502370.1 | DJ326915.1 | A57351.1 | GM654765.1 | HH956541.1 | HC007549.1 | GM636390.1 | DL017162.1 |
| HI002397.1 | CS501249.1 | DJ082661.1 | A46504.1 | GM647772.1 | HH998387.1 | HC007517.1 | GM636358.1 | DL017130.1 |
| HI002317.1 | CS511301.1 | DJ081675.1 | A38673.1 | GM647740.1 | HH998354.1 | HC010682.1 | GM631854.1 | DL017066.1 |
| HI000520.1 | CS498529.1 | DJ080165.1 | A35737.1 | GM647708.1 | HH999929.1 | DM375842.1 | GM631822.1 | DL017034.1 |
| HI183834.1 | CS498429.1 | DJ080429.1 | A35536.1 | GM640809.1 | HH986868.1 | DM370690.1 | GM631790.1 | DL012773.1 |
| HI183555.1 | DD418616.1 | DJ069478.1 | A29492.1 | GM640776.1 | HH979782.1 | DM370657.1 | GM631758.1 | DJ055289.1 |
| HI180164.1 | DD418581.1 | DJ066401.1 | A33978.1 | GM640744.1 | HH979693.1 | DM370625.1 | GM631726.1 | DJ061122.1 |
| HI178204.1 | DD412332.1 | DJ066368.1 | HW257126.1 | GM640712.1 | HH999893.1 | DM370593.1 | GM631694.1 | DJ057692.1 |
| HI202836.1 | DD411474.1 | DJ066336.1 | HW256934.1 | GM640680.1 | HH999855.1 | DM370561.1 | GM622923.1 | DJ060390.1 |
| HI214564.1 | DD410771.1 | DJ066303.1 | HW256806.1 | GM640648.1 | HH999751.1 | DM370529.1 | GM622891.1 | DJ060346.1 |
| HI214532.1 | DD417726.1 | DJ066269.1 | HW256710.1 | GM628294.1 | HH998318.1 | DM370497.1 | GM622859.1 | CS545488.1 |
| HI202789.1 | DD410184.1 | DJ065250.1 | HW251200.1 | DL095576.1 | HH998267.1 | DM370465.1 | GM622827.1 | CS544824.1 |
| HI214434.1 | CS487191.1 | DJ055702.1 | HW250891.1 | DL095544.1 | HH998230.1 | GM997886.1 | GM622795.1 | CS561191.1 |
| HI185962.1 | CS486786.1 | DJ061667.1 | HW247832.1 | DL117381.1 | HH998183.1 | GM983070.1 | GM622763.1 | CS560463.1 |
| HI213756.1 | CS483416.1 | DJ061619.1 | HW240894.1 | DL117349.1 | HH998518.1 | GM996538.1 | FB507967.1 | DD420074.1 |
| HI213004.1 | CS482970.1 | DJ061571.1 | HW240798.1 | DL117317.1 | HH993672.1 | GM994650.1 | GM628503.1 | DD434538.1 |
| HI203564.1 | CS482935.1 | DJ061523.1 | HW240766.1 | DL117285.1 | HH986624.1 | GM994297.1 | GM628471.1 | DD421795.1 |
| HI546319.1 | CS482895.1 | DJ055184.1 | HW240678.1 | DL117253.1 | HH979514.1 | GM992937.1 | GM628439.1 | DD433780.1 |
| HI546219.1 | DD401440.1 | DJ054374.1 | HW239308.1 | DL117221.1 | HH979550.1 | GM992456.1 | GM628407.1 | DD421665.1 |
| HI210997.1 | DD405191.1 | DJ053291.1 | HW239165.1 | DL091640.1 | HH979621.1 | GM992386.1 | GM628375.1 | DD420770.1 |
| HI210965.1 | DD405159.1 | EU233428.1 | HW238747.1 | DL091608.1 | HH979579.1 | GM992246.1 | GM628343.1 | CS541724.1 |
| HI210933.1 | DD405127.1 | CS810622.1 | HW238557.1 | DL091576.1 | HH931982.1 | GM992093.1 | GM628311.1 | CS541822.1 |
| HI566066.1 | DD405095.1 | CS812980.1 | HW242994.1 | DL087965.1 | DL069810.1 | GM991955.1 | GM640827.1 | CS543083.1 |

| | | | | | | | | |
|---|---|---|---|---|---|---|---|---|
| HI473010.1 | DD405063.1 | CS811216.1 | HW242949.1 | DL087933.1 | DL046830.1 | GM976399.1 | GM662084.1 | CS542562.1 |
| HI472906.1 | DD405031.1 | DD468179.1 | HW244249.1 | DL121764.1 | DL035055.1 | GM990842.1 | GM662052.1 | CS538097.1 |
| HI635494.1 | DD410098.1 | CS803353.1 | HW237889.1 | DL121732.1 | DL030708.1 | GM973570.1 | GM662020.1 | CS537441.1 |
| HC923065.1 | DD402580.1 | CS802328.1 | HW237848.1 | DL121700.1 | DL030676.1 | GM989462.1 | GM661988.1 | CS537182.1 |
| HC920698.1 | DD455216.1 | DJ047077.1 | HW242312.1 | DL121668.1 | DL030580.1 | GN000573.1 | GM661956.1 | CS502914.1 |
| HD068939.1 | DD455158.1 | DJ046960.1 | HW237201.1 | DL121636.1 | DL026739.1 | GM969574.1 | GM661924.1 | CS502713.1 |
| HD068721.1 | DD454728.1 | DJ050548.1 | HW237055.1 | DL121604.1 | DL026707.1 | GM981501.1 | GM654930.1 | CS502645.1 |
| HD066576.1 | DD458367.1 | DJ048823.1 | HW243425.1 | DL087777.1 | DL019496.1 | GM980874.1 | GM654898.1 | CS502549.1 |
| HD063954.1 | DD453825.1 | DD231459.1 | HW241638.1 | DL087745.1 | DL019464.1 | FB986331.1 | GM654866.1 | CS502407.1 |
| HD052879.1 | DD457792.1 | AX960226.1 | HW243088.1 | DL087713.1 | DL019432.1 | GM596733.1 | GM654834.1 | CS502451.1 |
| HC920316.1 | DD456892.1 | A06169.1 | HW243015.1 | DL087649.1 | DL019400.1 | GM970525.1 | GM654802.1 | CS502371.1 |
| HD033290.1 | DD456415.1 | DD214005.1 | HW163849.1 | DL087617.1 | CS604654.1 | FB736133.1 | GM654770.1 | CS501250.1 |
| HD065422.1 | CS643160.1 | DD213988.1 | HW163124.1 | DL128942.1 | CS592886.1 | GM969779.1 | GM647777.1 | CS498530.1 |
| HD058879.1 | CS631724.1 | DD218578.1 | HW161199.1 | DL125516.1 | CS592360.1 | GM969729.1 | GM647745.1 | CS498430.1 |
| HD057688.1 | CS642269.1 | DD213572.1 | HV778079.1 | DL125452.1 | CS592088.1 | DL479995.1 | GM647713.1 | CS497207.1 |
| HD057656.1 | CS631297.1 | DD213540.1 | HV777570.1 | DL125420.1 | CS598022.1 | DL479252.1 | GM647681.1 | CS494726.1 |
| HD062471.1 | CS631218.1 | DD222019.1 | HV775753.1 | DL125388.1 | CS597709.1 | DL476962.1 | GM647649.1 | DD418617.1 |
| M26695.1 | CS642196.1 | DD212023.1 | HV770290.1 | DL125356.1 | CS585210.1 | DL483006.1 | GM640814.1 | DD412387.1 |
| M14992.1 | CS161432.1 | DD211604.1 | HV775025.1 | DL121535.1 | CS583650.1 | DL463067.1 | GM640781.1 | DD412069.1 |
| HC916595.1 | CS159803.1 | DD182408.1 | HV764733.1 | DL121503.1 | CS574809.1 | DL470136.1 | GM640749.1 | DD411596.1 |
| FW334357.1 | CS159577.1 | DD206907.1 | HV764701.1 | DL121471.1 | CS573060.1 | DL462973.1 | GM640717.1 | DD411475.1 |
| FW337524.1 | CS159200.1 | DD206875.1 | HV764627.1 | DL121439.1 | CS543990.1 | DL482399.1 | DL090441.1 | DD414322.1 |
| FW337390.1 | CS155787.1 | DD206843.1 | HV764565.1 | DL121407.1 | CS546681.1 | DL462062.1 | DL090409.1 | DD410772.1 |
| FW336066.1 | CS148769.1 | DD206781.1 | HV774538.1 | DL117195.1 | CS560591.1 | DL475565.1 | DL090377.1 | DD417729.1 |
| HC889610.1 | CS144953.1 | DD196871.1 | HV767336.1 | DL117163.1 | DD421990.1 | DL481572.1 | DL086372.1 | CS491465.1 |
| HC887871.1 | CS141580.1 | CS240010.1 | HV767069.1 | DL117131.1 | DD431734.1 | FB504568.1 | DL086340.1 | CS490677.1 |
| HC887346.1 | CS141529.1 | DD181007.1 | HV766450.1 | DL117099.1 | CS540034.1 | GM832063.1 | DL086308.1 | CS487192.1 |
| HC867636.1 | CS141497.1 | AX958000.1 | HV766068.1 | DL117067.1 | CS538436.1 | GM712720.1 | DL086276.1 | CS482972.1 |
| HC880538.1 | CS141134.1 | AX937658.1 | HV755975.1 | DL117035.1 | CS537817.1 | GM712162.1 | DL086244.1 | CS482936.1 |
| HC046041.1 | CS136253.1 | AX934549.1 | HV444620.1 | DL112415.1 | BD231152.1 | GM711970.1 | DL086212.1 | CS482899.1 |
| HC046009.1 | CS131835.1 | AX923392.1 | HV444459.1 | DL112383.1 | BD230045.1 | GM653342.1 | DL110455.1 | BD437745.1 |
| HC046249.1 | CS130980.1 | AX840835.1 | HV444101.1 | DL112351.1 | BD226836.1 | GM646087.1 | DL110423.1 | BD395360.1 |
| HC046217.1 | CS129687.1 | AX824356.1 | HV437578.1 | DL112319.1 | BD225373.1 | GM646055.1 | DL110391.1 | BD405246.1 |
| HC046185.1 | CS122512.1 | AX823790.1 | HV349322.1 | DL112287.1 | BD223791.1 | GM646023.1 | DL110359.1 | BD437114.1 |
| HC046153.1 | CS106179.1 | AX823756.1 | HV341354.1 | DL143736.1 | BD223236.1 | GM626385.1 | DL110327.1 | BD404960.1 |
| HC046121.1 | CS106084.1 | AX814963.1 | HV341232.1 | DL107273.1 | BD222638.1 | GM626353.1 | DL110295.1 | BD404712.1 |
| HC046003.1 | CS181484.1 | AX805915.1 | HV343544.1 | DL107241.1 | BD218700.1 | GM660346.1 | DL105490.1 | BD446173.1 |
| HC045971.1 | CS179751.1 | AX799560.1 | HV344872.1 | DL107209.1 | BD211174.1 | GM653159.1 | DL105458.1 | BD453354.1 |
| HC045907.1 | CS326351.1 | AX798983.1 | HV340046.1 | GM658079.1 | BD206049.1 | GM653127.1 | DL105426.1 | BD453324.1 |
| HC045875.1 | CS323678.1 | AX796718.1 | HV347079.1 | GM658047.1 | DD283761.1 | GM653095.1 | DL105394.1 | BD453292.1 |
| HC045843.1 | CS322967.1 | AX472469.1 | HV344596.1 | GM658015.1 | DD286564.1 | GM653063.1 | DL098378.1 | BD456417.1 |
| HC045811.1 | CS330147.1 | BD194477.1 | HV339956.1 | GM657983.1 | CS283953.1 | GM639037.1 | DL098346.1 | BD444972.1 |
| HC045779.1 | CS318710.1 | BD191691.1 | HV339924.1 | GM657951.1 | CS283848.1 | GM639005.1 | DL092336.1 | BD444958.1 |
| A00267.1 | DD271419.1 | AX781449.1 | HV339664.1 | GM650759.1 | CS283075.1 | GM638973.1 | DL092304.1 | BD376362.1 |
| DD212841.1 | DD259348.1 | AX773266.1 | HV344482.1 | GM650727.1 | CS276857.1 | GM626166.1 | DL092272.1 | BD373915.1 |
| DD214017.1 | DD265352.1 | AF430211.1 | HV342133.1 | GM650695.1 | CS266079.1 | GM626134.1 | DL092240.1 | BD366174.1 |
| DD216628.1 | CS305187.1 | AF430179.1 | HV342049.1 | GM643703.1 | CS265602.1 | GM652839.1 | DL092208.1 | BD353797.1 |

| | | | | | | | | |
|---|---|---|---|---|---|---|---|---|
| DD213907.1 | CS299520.1 | AX512476.1 | FW561968.1 | GM643671.1 | CS249788.1 | GM652807.1 | DL092176.1 | BD342505.1 |
| DD213875.1 | DD251994.1 | AX505183.1 | FW552163.1 | GM643631.1 | CS244150.1 | GM638817.1 | DL088561.1 | BD341872.1 |
| DD213843.1 | DD250794.1 | AX496846.1 | FW552125.1 | GM636642.1 | CS244230.1 | GM638785.1 | DL088529.1 | BD349909.1 |
| DD213811.1 | DD249854.1 | BD137870.1 | FW561714.1 | GM636610.1 | CS244198.1 | GM638753.1 | DL088497.1 | BD349526.1 |
| DD216486.1 | DD248868.1 | BD135118.1 | FW555592.1 | GM636578.1 | BD341801.1 | GM038282.1 | DL088465.1 | BD339060.1 |
| DD213794.1 | DD258238.1 | BD133477.1 | FW556530.1 | GM636546.1 | BD339009.1 | DL125477.1 | DL088433.1 | BD325655.1 |
| DD213762.1 | DD257966.1 | BD131927.1 | FW561694.1 | GM636514.1 | BD325598.1 | DL125445.1 | DL088401.1 | BD325558.1 |
| DD213730.1 | DD253702.1 | BD130820.1 | FW561662.1 | GM636482.1 | BD325491.1 | DL125413.1 | DL117801.1 | BD314394.1 |
| DD213698.1 | CS287626.1 | BD130788.1 | FW562744.1 | GM632074.1 | BD314344.1 | DL125381.1 | DL117769.1 | BD314172.1 |
| DD213666.1 | DD240757.1 | BD130756.1 | FW565212.1 | GM623111.1 | BD312820.1 | DL125349.1 | DL117737.1 | BD312784.1 |
| DD213634.1 | DD240660.1 | BD130690.1 | FW562598.1 | GM623079.1 | BD319276.1 | DL117188.1 | DL117705.1 | BD311523.1 |
| DD213602.1 | DD161541.1 | BD130592.1 | FW561181.1 | GM643501.1 | BD317793.1 | DL117124.1 | DL117673.1 | BD319295.1 |
| DD213584.1 | DD161078.1 | BD129640.1 | FW508154.1 | GM643469.1 | BD308640.1 | DL143641.1 | DL117641.1 | BD318872.1 |
| DD213552.1 | DD178781.1 | BD107264.1 | FW508111.1 | GM643436.1 | BD300560.1 | AY658150.1 | DL122164.1 | BD300452.1 |
| DD220649.1 | DD171820.1 | BD094356.1 | FW562448.1 | GM636447.1 | BD300344.1 | AY658118.1 | DL122132.1 | BD294448.1 |
| DD220594.1 | DD165509.1 | BD087154.1 | FW553167.1 | GM631847.1 | BD291407.1 | AY658086.1 | DL122100.1 | BD293453.1 |
| BD161071.1 | DD165149.1 | BD080746.1 | FW556660.1 | GM631815.1 | BD291301.1 | AY658054.1 | DL122036.1 | BD292829.1 |
| BD160750.1 | DD164876.1 | BD080658.1 | FW559382.1 | GM631783.1 | BD299329.1 | AY658022.1 | DL122004.1 | BD292446.1 |
| AX587807.1 | DD182183.1 | A32773.1 | FW506211.1 | GM622852.1 | BD294891.1 | AY657990.1 | DL125713.1 | BD291235.1 |
| AX577647.1 | DD175950.1 | A13696.1 | HI931580.1 | GM622820.1 | BD276165.1 | AY657958.1 | DL125681.1 | BD298687.1 |
| AX573895.1 | DD175917.1 | A31182.1 | HI936417.1 | GM657905.1 | BD272756.1 | AY657926.1 | DL125649.1 | BD296984.1 |
| AX573106.1 | CS204324.1 | A25918.1 | HI929429.1 | GM657873.1 | BD271966.1 | AY657894.1 | DL125617.1 | BD296942.1 |
| AX556855.1 | CS001423.1 | A19977.1 | HI935095.1 | GM657841.1 | BD271157.1 | AY657862.1 | DL125585.1 | BD296886.1 |
| AX556821.1 | AF408178.1 | A12680.1 | HI934979.1 | GM657809.1 | DD230119.1 | AY657830.1 | DL125553.1 | BD296829.1 |
| AX555190.1 | CS157799.1 | A04780.1 | HI929196.1 | GM657776.1 | DD227403.1 | AY657798.1 | DL121961.1 | AX364653.1 |
| AX554279.1 | CS157960.1 | A00351.1 | HI918292.1 | GM657744.1 | DD224449.1 | AY657766.1 | DL121897.1 | AX364536.1 |
| AX544166.1 | CS157928.1 | A31644.1 | HI918260.1 | GM643257.1 | CQ812761.1 | AY657734.1 | DL121865.1 | AX364504.1 |
| AX538884.1 | CS146879.1 | A30922.1 | HI000266.1 | GM643225.1 | CQ802456.1 | AY657702.1 | DL121833.1 | AX364472.1 |
| AX538639.1 | CS144057.1 | A29004.1 | HI000220.1 | GM636236.1 | CQ801297.1 | AY657670.1 | DL121801.1 | AX364440.1 |
| AX538262.1 | CS126252.1 | A28855.1 | HI000164.1 | GM631668.1 | CQ800903.1 | AY657638.1 | DL117538.1 | AX364408.1 |
| AX536405.1 | CS124721.1 | A25128.1 | HB845966.1 | GM631636.1 | CQ800658.1 | AY657606.1 | DL117506.1 | AX364376.1 |
| AX534755.1 | CS124681.1 | A20000.1 | HB845144.1 | GM631604.1 | CQ796820.1 | AY657574.1 | DL117474.1 | AX364344.1 |
| AX528978.1 | CS124643.1 | A28244.1 | HB844851.1 | GM631572.1 | CQ795472.1 | AY657542.1 | DL117442.1 | AX364249.1 |
| AX528946.1 | CS124599.1 | A26540.1 | HB844412.1 | GM653636.1 | CQ795440.1 | AY657510.1 | DL112731.1 | AX364217.1 |
| AX528535.1 | AX704649.1 | A26053.1 | HB836891.1 | GM653604.1 | CQ789904.1 | AY657478.1 | DL112699.1 | AX364184.1 |
| AX524852.1 | AX701590.1 | A25580.1 | HB836454.1 | GM653572.1 | CQ788611.1 | AY657446.1 | DL112667.1 | GM641655.1 |
| AX523914.1 | AX699464.1 | A23320.1 | HB836238.1 | GM660769.1 | CQ787291.1 | AY657414.1 | DL107862.1 | GM641623.1 |
| AX523632.1 | AX699432.1 | A23260.1 | HB830438.1 | GM660737.1 | CQ784697.1 | AY657382.1 | DL107830.1 | GM634435.1 |
| A24069.1 | AX685836.1 | A11919.1 | HB843097.1 | GM653518.1 | CQ784633.1 | AY657350.1 | DL107798.1 | GM634403.1 |
| A33386.1 | AX685146.1 | A21489.1 | HB835515.1 | GM653486.1 | CQ778521.1 | AY657318.1 | DL107766.1 | GM634371.1 |
| AX179483.1 | AX682973.1 | A19974.1 | HB807558.1 | GM653454.1 | CQ778399.1 | HI544136.1 | DL107734.1 | GM634339.1 |
| AX179416.1 | AX671499.1 | A18689.1 | HB824017.1 | GM653390.1 | CQ774808.1 | HI000036.1 | DL107702.1 | GM634307.1 |
| AX027990.1 | AX670722.1 | A18010.1 | HB842367.1 | GM646391.1 | CQ771675.1 | HI003673.1 | DL103098.1 | GM634275.1 |
| AX521537.1 | AX665486.1 | A16285.1 | HB850377.1 | GM646359.1 | CQ771199.1 | HI003626.1 | DL103066.1 | GM629480.1 |
| AX521505.1 | AX664346.1 | A15950.1 | HB842331.1 | GM646295.1 | CQ759688.1 | HI003586.1 | DL103034.1 | GM629438.1 |
| AF430191.1 | AX662228.1 | A15353.1 | HB841965.1 | GM646263.1 | CQ756596.1 | HI003548.1 | DL098325.1 | GM629406.1 |
| AF430159.1 | AX662053.1 | A14318.1 | HB841713.1 | GM646231.1 | CQ755436.1 | HI001768.1 | DL098293.1 | GM629374.1 |

| | | | | | | | | |
|---|---|---|---|---|---|---|---|---|
| AX511344.1 | AX657146.1 | A13504.1 | HB841297.1 | GM639428.1 | CQ754025.1 | HI001731.1 | DL098261.1 | GM629310.1 |
| AX505271.1 | AX657114.1 | A12493.1 | HB840697.1 | GM639396.1 | AX962915.1 | HI544130.1 | DL098229.1 | GM619878.1 |
| AX505207.1 | AX657070.1 | A11671.1 | GM645192.1 | GM639364.1 | AX454048.1 | HI465479.1 | DL098197.1 | GM648573.1 |
| AX505143.1 | BD176081.1 | A10998.1 | GM645160.1 | GM639332.1 | A35244.1 | HI567466.1 | DL098165.1 | GM648541.1 |
| BD140159.1 | BD174394.1 | HV961476.1 | GM645128.1 | GM639300.1 | A34182.1 | HI001611.1 | DL092155.1 | GM648509.1 |
| BD138559.1 | AX644917.1 | HV960817.1 | GM645096.1 | GM639268.1 | AX923423.1 | HI001511.1 | DL092123.1 | GM648477.1 |
| BD138055.1 | AX643979.1 | HV964075.1 | GM645064.1 | GM626721.1 | AX923390.1 | HI003393.1 | DL092027.1 | GM648445.1 |
| BD135698.1 | AX642222.1 | HV959178.1 | GM645032.1 | GM660566.1 | AX840833.1 | HI003352.1 | DL091963.1 | GM648413.1 |
| BD131181.1 | AX641834.1 | HV958862.1 | GM638137.1 | GM660534.1 | AX825360.1 | HI003312.1 | FB329191.1 | GM641610.1 |
| BD130800.1 | BD172074.1 | HV963331.1 | GM638105.1 | GM653347.1 | AX824354.1 | HI505470.1 | FB299285.1 | GM629265.1 |
| BD130734.1 | HH820955.1 | HV965612.1 | GM638073.1 | GM653315.1 | AX823788.1 | HI464690.1 | CS368344.1 | GM629233.1 |
| HW389148.1 | HH820914.1 | HV969892.1 | GM644926.1 | GM653283.1 | AX805912.1 | HI464658.1 | CS368280.1 | GM629201.1 |
| HW388633.1 | HH820332.1 | HV965325.1 | GM633335.1 | GM653251.1 | AX799558.1 | HI505290.1 | CS368088.1 | GM629169.1 |
| HW387852.1 | HH759321.1 | HV956046.1 | GM633303.1 | GM653219.1 | AX797517.1 | HI542539.1 | CS368024.1 | GM629137.1 |
| HW379427.1 | HH759210.1 | HV951609.1 | GM633248.1 | GM653187.1 | AX796716.1 | HI462682.1 | CS367404.1 | GM629105.1 |
| FW349644.1 | HH759178.1 | HV951545.1 | GM633216.1 | GM646092.1 | AX794344.1 | HH716010.1 | CS367340.1 | GM634067.1 |
| HW376166.1 | HH759146.1 | HV951143.1 | GM633184.1 | GM646060.1 | AX472467.1 | HH714041.1 | DL032249.1 | GM634035.1 |
| HW376088.1 | HH759114.1 | HV950689.1 | GM633152.1 | GM646028.1 | BD194475.1 | HH961377.1 | DL032217.1 | GM634003.1 |
| HW365414.1 | HH759082.1 | HV950610.1 | GM633120.1 | GM626518.1 | AX773264.1 | HH961345.1 | DL032185.1 | GM633939.1 |
| HW365416.1 | HH759050.1 | HV947137.1 | GM651773.1 | GM626486.1 | AX179520.1 | HH961313.1 | DL032153.1 | GM633907.1 |
| FW348554.1 | HH759018.1 | HV953005.1 | GM644781.1 | GM626454.1 | AX179460.1 | HH961281.1 | DL048423.1 | GM633875.1 |
| HW381041.1 | HH758986.1 | HV554542.1 | GM644749.1 | GM626422.1 | AX028008.1 | HH961249.1 | DL048391.1 | GM655516.1 |
| HW317048.1 | HH758954.1 | HV550385.1 | GM644717.1 | GM626390.1 | A33489.1 | HH975455.1 | DL048359.1 | GM655484.1 |
| HW326501.1 | HH758922.1 | AY659389.1 | GM625188.1 | GM626358.1 | AF430209.1 | HH975170.1 | DL048327.1 | GM655452.1 |
| HW338846.1 | HH758890.1 | AY659357.1 | GM625156.1 | GM836071.1 | AF430177.1 | HH964278.1 | DL044441.1 | GM648363.1 |
| HW338590.1 | HH757466.1 | AY659325.1 | GM621651.1 | GM660383.1 | AX512474.1 | HH982168.1 | DL044409.1 | GM648331.1 |
| HW338334.1 | HH757434.1 | AY659293.1 | GM621619.1 | GM660351.1 | AX505243.1 | HH974540.1 | DL044377.1 | GM648299.1 |
| HW338206.1 | HC471771.1 | AY659261.1 | GM621587.1 | GM653164.1 | AX505179.1 | HH963746.1 | DL014586.1 | GM648267.1 |
| HW338078.1 | HC471739.1 | AY659229.1 | GM621555.1 | GM653132.1 | AX497901.1 | HH998109.1 | DL014554.1 | GM648235.1 |
| HW337566.1 | HC471699.1 | AY659197.1 | GM657135.1 | GM653100.1 | AX496844.1 | HH998066.1 | DL014522.1 | GM648203.1 |
| HW337438.1 | HC471643.1 | AY659165.1 | GM657103.1 | GM653068.1 | BD140256.1 | HH998005.1 | DL014458.1 | GM641400.1 |
| HW337182.1 | HC471611.1 | AY659133.1 | GM657071.1 | GM653036.1 | BD138726.1 | HH997947.1 | DL010000.1 | GM641368.1 |
| HW321858.1 | HC729059.1 | AY659101.1 | GM657039.1 | GM653004.1 | BD137863.1 | HH999652.1 | DL009968.1 | GM641336.1 |
| HW336798.1 | HC728988.1 | AY659069.1 | GM657007.1 | GM638978.1 | BD135100.1 | HH997903.1 | DL009936.1 | GM618782.1 |
| HW336434.1 | HC728650.1 | AY659037.1 | GM656912.1 | GM638946.1 | BD131778.1 | HH997766.1 | DL009904.1 | GM864921.1 |
| HW329579.1 | HC727675.1 | AY659005.1 | GM656880.1 | GM638914.1 | BD130818.1 | HH999506.1 | DL009872.1 | FB725958.1 |
| HW329443.1 | HC727392.1 | AY658973.1 | GM656848.1 | GM638882.1 | BD130786.1 | HH999448.1 | DL009840.1 | GM842466.1 |
| HW329329.1 | HC688439.1 | AY658941.1 | GM656816.1 | GM638850.1 | BD130754.1 | HH999329.1 | DL009808.1 | GM616335.1 |
| HW320435.1 | HC686909.1 | AY658909.1 | GM656784.1 | GM626171.1 | BD130720.1 | HH997645.1 | DL009776.1 | GM841615.1 |
| HW336063.1 | HC490835.1 | AY658877.1 | GM656752.1 | GM626139.1 | BD130688.1 | HH997613.1 | DL009744.1 | GM731764.1 |
| HW335975.1 | HC490771.1 | AY658845.1 | GM630483.1 | DL116079.1 | BD130586.1 | HH997431.1 | DL009712.1 | FB711260.1 |
| HW328743.1 | HC490739.1 | AY658813.1 | GM621548.1 | DL116047.1 | BD094345.1 | HH997383.1 | DL009680.1 | GM840417.1 |
| HW328449.1 | HC490707.1 | AY658781.1 | GM621484.1 | DL143511.1 | BD087152.1 | HH999694.1 | DL009648.1 | GM041779.1 |
| HW328341.1 | HC490675.1 | AY658749.1 | FB505208.1 | DL120571.1 | BD084024.1 | HH999244.1 | DL009616.1 | GM721319.1 |
| HW335693.1 | HC490479.1 | AY658717.1 | GM649553.1 | GN031304.1 | BD081730.1 | HH999150.1 | DL009584.1 | CS728554.1 |
| HW335650.1 | HC490408.1 | AY658685.1 | GM670031.1 | GN031271.1 | BD081543.1 | HH998116.1 | DL009552.1 | CS727616.1 |
| HW318486.1 | HC472301.1 | AY658653.1 | GM656528.1 | GN031239.1 | BD080723.1 | HH999074.1 | DL009520.1 | CS727226.1 |

| | | | | | | | | |
|---|---|---|---|---|---|---|---|---|
| HW318156.1 | HC472268.1 | AY658621.1 | GM656496.1 | GN031207.1 | BD080157.1 | HH999029.1 | DL009488.1 | GM835944.1 |
| HW335182.1 | HC472236.1 | AY658589.1 | GM656464.1 | GN031175.1 | BD080125.1 | HH998963.1 | DL009456.1 | GM005809.1 |
| DL015927.1 | HC472204.1 | AY658557.1 | GM656432.1 | GN031142.1 | BD074966.1 | HH998920.1 | DL009424.1 | GM685504.1 |
| DL015895.1 | HC472172.1 | AY658525.1 | GM649335.1 | GN031110.1 | BD074818.1 | HH997181.1 | DL026340.1 | FB753777.1 |
| DL015863.1 | HC471940.1 | AY658493.1 | GM649247.1 | GN031078.1 | BD071145.1 | HH998915.1 | DL026308.1 | FB728449.1 |
| DL011245.1 | HC471908.1 | AY658461.1 | GM649215.1 | GN031046.1 | BD070002.1 | HH998862.1 | DL026244.1 | GM680782.1 |
| DL011213.1 | HC471876.1 | AY658429.1 | GM642474.1 | GN030982.1 | BD064949.1 | HH998805.1 | DL026212.1 | FB727449.1 |
| DL047637.1 | HC509928.1 | AY658397.1 | GM635103.1 | GN030951.1 | BD057304.1 | HB485504.1 | DL026180.1 | GM618720.1 |
| DL047605.1 | HC494545.1 | AY658365.1 | GM635071.1 | GN030887.1 | BD016714.1 | HB474816.1 | DL018990.1 | FB712562.1 |
| DL047573.1 | HC494419.1 | AY658333.1 | GM738863.1 | GM648327.1 | BD015529.1 | HB464062.1 | DL018958.1 | GM040585.1 |
| DL047541.1 | HC500299.1 | AY658301.1 | GM840184.1 | GM648295.1 | E50524.1 | HB469143.1 | DL018926.1 | FB709037.1 |
| DL039678.1 | HC499864.1 | AY658269.1 | CS728609.1 | GM648263.1 | BD014213.1 | HB468767.1 | DL018894.1 | GM049545.1 |
| DL039646.1 | FW302102.1 | AY658237.1 | CS728577.1 | GM648231.1 | BD014173.1 | HB468027.1 | DL018830.1 | GM604063.1 |
| DL039614.1 | FW301501.1 | AY658205.1 | CS727421.1 | GM648199.1 | AX490790.1 | DM152672.1 | DL034497.1 | GM603716.1 |
| DL039582.1 | FW300958.1 | AY658173.1 | CS727214.1 | GM641396.1 | AX478551.1 | DM156106.1 | DL034465.1 | GM706685.1 |
| DL039550.1 | HB864827.1 | AY658141.1 | GM036647.1 | GM641364.1 | E66892.1 | HB461514.1 | DL034433.1 | GM706388.1 |
| DL039518.1 | HB864795.1 | AY658109.1 | GM836384.1 | GM641332.1 | E66928.1 | HB455145.1 | DL034401.1 | GM706354.1 |
| DL039486.1 | HB864763.1 | AY658077.1 | GM007120.1 | GM641300.1 | AX468905.1 | GN087186.1 | DL034369.1 | GM704817.1 |
| DL039385.1 | HB866945.1 | AY658045.1 | GM685460.1 | GM641268.1 | A22047.1 | GN089991.1 | DL034337.1 | FB722181.1 |
| DL039353.1 | HB866504.1 | AY658013.1 | FB983714.1 | GM641236.1 | AX458602.1 | GN089846.1 | DL026155.1 | FB720258.1 |
| DL039321.1 | HB981727.1 | AY657981.1 | GM680773.1 | GM629077.1 | HW251039.1 | GN093652.1 | DJ088311.1 | FB718244.1 |
| DL035665.1 | HB999865.1 | AY657949.1 | GM879315.1 | GM629045.1 | HW250843.1 | GN082856.1 | DJ080934.1 | FB717765.1 |
| DL035633.1 | HB977439.1 | AY657917.1 | FB712553.1 | GM629013.1 | HW241223.1 | GN089188.1 | DJ080309.1 | FB715076.1 |
| DL035601.1 | HB976600.1 | AY657885.1 | GM840928.1 | GM881893.1 | HW247776.1 | GN082815.1 | DJ081438.1 | DL119093.1 |
| DL035569.1 | DM201829.1 | HW261072.1 | GM721266.1 | GM648166.1 | HW240819.1 | GN088784.1 | DJ069353.1 | DL119061.1 |
| DL035537.1 | DM201551.1 | HW261040.1 | GM059989.1 | GM716077.1 | HW240635.1 | GN088032.1 | DJ066389.1 | DL119029.1 |
| DL035505.1 | DM193331.1 | HW261008.1 | GM841714.1 | GM655120.1 | HW240603.1 | DL232854.1 | DJ066325.1 | DL114633.1 |
| DL031501.1 | DM195297.1 | HW260976.1 | GM603973.1 | GM640912.1 | HW239421.1 | GN078164.1 | DJ066291.1 | DL114601.1 |
| DL031469.1 | HB840241.1 | HW260944.1 | GM841211.1 | GM640880.1 | HW239282.1 | L08954.1 | DJ066257.1 | DL114569.1 |
| DL031437.1 | HB848138.1 | HW260880.1 | GM839081.1 | GM640848.1 | HW238546.1 | L08939.1 | DJ068026.1 | DL114537.1 |
| DL031405.1 | HB847887.1 | HW260848.1 | GM601141.1 | GM628494.1 | HW238385.1 | L08859.1 | DJ067043.1 | DL114505.1 |
| DL031373.1 | HB839319.1 | HW260816.1 | FB715276.1 | GM662107.1 | HW238246.1 | M94407.1 | DJ052725.1 | DL114473.1 |
| DL023560.1 | HB847345.1 | HW260784.1 | FB676837.1 | GM654825.1 | HW244308.1 | DM063454.1 | DJ055588.1 | DL109576.1 |
| DL023528.1 | HB839057.1 | HW260720.1 | GM061228.1 | GM654793.1 | HW237922.1 | DM058828.1 | DJ061291.1 | DL109544.1 |
| DL023496.1 | HB832562.1 | HW260688.1 | DL241173.1 | GM654761.1 | HW237877.1 | DM045374.1 | DJ061131.1 | DL109512.1 |
| DL023432.1 | HB846923.1 | HW260656.1 | DL241031.1 | GM647768.1 | HW237707.1 | DM045218.1 | DJ060414.1 | DL109480.1 |
| DL023400.1 | HB838542.1 | HW260624.1 | HW387846.1 | GM647736.1 | HW248658.1 | DM044925.1 | DJ054280.1 | DL104874.1 |
| DL020385.1 | HB846431.1 | HW260592.1 | HW380840.1 | GM647704.1 | HW242373.1 | DM039601.1 | DJ060349.1 | DL104842.1 |
| DL020353.1 | HB846082.1 | HW260560.1 | HW368619.1 | CS696002.1 | HW242301.1 | DM044809.1 | DJ060307.1 | DL104810.1 |
| DL020321.1 | HB826940.1 | HW260528.1 | HW364598.1 | CS695938.1 | HW241803.1 | DM044660.1 | DJ060273.1 | DL095855.1 |
| DL015790.1 | HB845619.1 | HW260496.1 | HW373275.1 | CS695810.1 | HW241603.1 | DM060669.1 | DJ056897.1 | DL095823.1 |
| DL015758.1 | HB845378.1 | HW260464.1 | HW381331.1 | CS695778.1 | HW165041.1 | GN068782.1 | DJ053508.1 | AX145059.1 |
| DL015726.1 | HB837098.1 | HW260432.1 | HW381035.1 | CS695746.1 | HW243068.1 | GN068390.1 | DJ056359.1 | AX145027.1 |
| CS608445.1 | HB813975.1 | HW260400.1 | HW154871.1 | CS695586.1 | HW241387.1 | GN067916.1 | DJ053219.1 | AX144995.1 |
| CS608363.1 | HB845186.1 | HW260336.1 | HW145900.1 | CS695554.1 | HW163333.1 | GN065091.1 | DJ053087.1 | AX144963.1 |
| CS607915.1 | HB844886.1 | HW260304.1 | HW154720.1 | CS695490.1 | HW155766.1 | GN060114.1 | DJ052893.1 | AX144931.1 |
| CS607670.1 | HB844424.1 | HW260272.1 | HW154468.1 | CS695458.1 | HW155700.1 | GN052377.1 | CS810643.1 | AX144898.1 |

| | | | | | | | | |
|---|---|---|---|---|---|---|---|---|
| CS607528.1 | HB844267.1 | HW260240.1 | HW158131.1 | CS695394.1 | HV437566.1 | GM651043.1 | FB292722.1 | AX144866.1 |
| CS607458.1 | HB836527.1 | HW260208.1 | HW154017.1 | DL176446.1 | HV437521.1 | GM651011.1 | FB292245.1 | AX144834.1 |
| CS607076.1 | HB843770.1 | HW260176.1 | HW153784.1 | DL176357.1 | HV341338.1 | GM644212.1 | DD468059.1 | AX144802.1 |
| CS606997.1 | HB835824.1 | HW260144.1 | HW153604.1 | DL176283.1 | HV344782.1 | GM644180.1 | DJ017824.1 | AX144770.1 |
| CS606972.1 | HB835196.1 | HW260112.1 | HW157489.1 | DL174621.1 | HV344698.1 | GM644148.1 | DJ019825.1 | AX144738.1 |
| CS606909.1 | HB835170.1 | HW260080.1 | HW151801.1 | DL183112.1 | HV339977.1 | GM644116.1 | DJ015684.1 | AX144706.1 |
| CS606836.1 | HB842568.1 | HW260048.1 | HW151135.1 | DL176770.1 | HV339913.1 | GM636926.1 | DJ021139.1 | AX144674.1 |
| CS605993.1 | HB806719.1 | HW260016.1 | HW159793.1 | DL176671.1 | HV322597.1 | GM636862.1 | CS802103.1 | AX144642.1 |
| CS604953.1 | HB842026.1 | HW259984.1 | HW155987.1 | FB357118.1 | HV321545.1 | GM623523.1 | FB295398.1 | AX144543.1 |
| CS604889.1 | HB841725.1 | HW259952.1 | HW144737.1 | DL088087.1 | FW570997.1 | GM623491.1 | DJ047018.1 | AX144479.1 |
| CS589838.1 | HB841425.1 | HW259920.1 | HW144445.1 | DL088055.1 | FW568097.1 | GM623427.1 | DJ052329.1 | AX144157.1 |
| CS593429.1 | HB840824.1 | HW258855.1 | HW144367.1 | DL088023.1 | CS102544.1 | GM623395.1 | DJ052029.1 | AX143899.1 |
| CS597726.1 | HB650194.1 | HW257307.1 | HW144303.1 | DL087991.1 | CS102512.1 | GM623363.1 | DJ045755.1 | AX143835.1 |
| CS593361.1 | HB648685.1 | HW257211.1 | HW124879.1 | DL091932.1 | CS102480.1 | GM643882.1 | DJ050609.1 | AX143643.1 |
| CS585634.1 | HB645712.1 | HW257115.1 | HW124787.1 | DL091900.1 | CS097954.1 | GM643850.1 | DJ050489.1 | AX143579.1 |
| CS588481.1 | HB645680.1 | HW257051.1 | HW123955.1 | DL091868.1 | CS084382.1 | GM643818.1 | DJ049693.1 | AX143323.1 |
| CS576392.1 | HI465491.1 | HW257019.1 | HW123102.1 | DL102861.1 | AX773262.1 | GM632221.1 | DJ048845.1 | AX143195.1 |
| CS576673.1 | HI465365.1 | HW256795.1 | HW122425.1 | DL102829.1 | AX771247.1 | GM741832.1 | CS793392.1 | AX143131.1 |
| CS565543.1 | HI001614.1 | HW256763.1 | HW120952.1 | DL102797.1 | AX766523.1 | GM658314.1 | CS800122.1 | AX143003.1 |
| CS570725.1 | HI003398.1 | HW256699.1 | HV767830.1 | DL102765.1 | AX766123.1 | GM658282.1 | CS799996.1 | AX142875.1 |
| CS545382.1 | HI003355.1 | HV537387.1 | HV766848.1 | DL102733.1 | AX754676.1 | GM658250.1 | CS716861.1 | AX142811.1 |
| CS560776.1 | HI003317.1 | HV515807.1 | HV760602.1 | DL107577.1 | AX746414.1 | GM658218.1 | CS707352.1 | AX142683.1 |
| CS560456.1 | HI550424.1 | HV515598.1 | HV766033.1 | DL107485.1 | BD184732.1 | GM658186.1 | DD401683.1 | AX142555.1 |
| CS559091.1 | HI464725.1 | HV515566.1 | HV755828.1 | DL107453.1 | BD183211.1 | GM658104.1 | DD401651.1 | AX142489.1 |
| DD420050.1 | HI464693.1 | HV515438.1 | HV750178.1 | DL107389.1 | BD182838.1 | GM658072.1 | DD405397.1 | AX142425.1 |
| DD421788.1 | HI464661.1 | HV515406.1 | HV758674.1 | DL102584.1 | BD181486.1 | GM658040.1 | DD405365.1 | AX142297.1 |
| DD431868.1 | HI505332.1 | HV515374.1 | HV745366.1 | DL102552.1 | BD180846.1 | GM658008.1 | DD405333.1 | AX141845.1 |
| CS543248.1 | HI505040.1 | HV515342.1 | HV749404.1 | DL102520.1 | AX741739.1 | GM657976.1 | DD405301.1 | AX141717.1 |
| CS531843.1 | HI549558.1 | HV515310.1 | HV750971.1 | DL098042.1 | BD177401.1 | GM657944.1 | DD405269.1 | AX141525.1 |
| CS537867.1 | HI542542.1 | HV515278.1 | HV747495.1 | DL098010.1 | AX716729.1 | GM650752.1 | DD401635.1 | AX141333.1 |
| BD376343.1 | HI462685.1 | HV515246.1 | HV507919.1 | DL097978.1 | AX711157.1 | GM650720.1 | DD401603.1 | AX133313.1 |
| BD375298.1 | HI503811.1 | HV511336.1 | HV509338.1 | DL097946.1 | AX709612.1 | GM650688.1 | DD401571.1 | AX119974.1 |
| BD374746.1 | HH716015.1 | HV507843.1 | HV508681.1 | DL112636.1 | AX708721.1 | GM643696.1 | DD401507.1 | AX113776.1 |
| BD356422.1 | HH713510.1 | HV513562.1 | HV508614.1 | DL112604.1 | AX701741.1 | GM636539.1 | DD401475.1 | AX113552.1 |
| BD353558.1 | HH961380.1 | HV510208.1 | HV508582.1 | DL112572.1 | AX686822.1 | GM636507.1 | CS480613.1 | AX111912.1 |
| BD359047.1 | HH961348.1 | HV508673.1 | HV508550.1 | DL127142.1 | AX684262.1 | GM636475.1 | CS479314.1 | HW242319.1 |
| BD342470.1 | HH961316.1 | HV508638.1 | HV508490.1 | DL141541.1 | AX683734.1 | FB741912.1 | CS475886.1 | HW242287.1 |
| BD341833.1 | HC010330.1 | HV508574.1 | HV512292.1 | DL123471.1 | AX664358.1 | FB674300.1 | CS469223.1 | HW242255.1 |
| BD349739.1 | HC009744.1 | HV508542.1 | HV512260.1 | DL123439.1 | AX663012.1 | DL233404.1 | CS466953.1 | HW243432.1 |
| BD349500.1 | HC008042.1 | HV508510.1 | HV512228.1 | DL123407.1 | AX657126.1 | FB713981.1 | CS464636.1 | HW236726.1 |
| BD326089.1 | HC007770.1 | HV512284.1 | HV512164.1 | DL123375.1 | AX657091.1 | FB713853.1 | DD392052.1 | HW241474.1 |
| BD325627.1 | HC007674.1 | HV512252.1 | HV512132.1 | DL119121.1 | AX143859.1 | FB712623.1 | DD392054.1 | HW163856.1 |
| BD325533.1 | HC007582.1 | HV512188.1 | HV504763.1 | DL119089.1 | AX143795.1 | FB707628.1 | DD391746.1 | HW163487.1 |
| BD324949.1 | HC007550.1 | HV512156.1 | HV504731.1 | DL119057.1 | AX143731.1 | FB674506.1 | DD385336.1 | HW160676.1 |
| BD314371.1 | HC007518.1 | HV512124.1 | HV504699.1 | DL119025.1 | AX143603.1 | FB671082.1 | DD382841.1 | HW160628.1 |
| BD313214.1 | HC010683.1 | HV504787.1 | HV504667.1 | DL114629.1 | AX143475.1 | FB670800.1 | DD400165.1 | HW160596.1 |
| BD319290.1 | DM375843.1 | HV504755.1 | HV504635.1 | DL114597.1 | AX143283.1 | FB667087.1 | DD400133.1 | HW155686.1 |

| | | | | | | | | |
|---|---|---|---|---|---|---|---|---|
| BD319464.1 | DM371126.1 | HV504723.1 | HV504603.1 | DL114533.1 | AX143027.1 | FB666655.1 | CS461669.1 | HW155634.1 |
| BD318829.1 | DM370658.1 | HV504691.1 | HV504986.1 | DL114501.1 | AX142899.1 | FB676499.1 | CS463243.1 | HW150135.1 |
| BD308702.1 | DM370626.1 | HV504627.1 | HV504228.1 | DL114469.1 | AX142579.1 | FB705959.1 | CS461224.1 | HW150103.1 |
| BD307641.1 | DM370594.1 | HV504595.1 | HV504196.1 | DL201916.1 | AX142513.1 | FB701820.1 | CS459838.1 | HW150007.1 |
| BD293376.1 | DM370562.1 | HV504978.1 | HV503840.1 | DL201578.1 | AX142449.1 | FB665209.1 | CS459115.1 | HW149975.1 |
| BD292426.1 | DM370530.1 | HI375905.1 | HV491263.1 | DL198981.1 | AX142385.1 | DL111662.1 | DD361296.1 | HW144812.1 |
| BD291333.1 | DM370498.1 | HI369145.1 | HV503800.1 | DL191614.1 | AX142257.1 | DL111630.1 | DD361264.1 | HW154992.1 |
| BD298672.1 | DM370466.1 | HI369092.1 | HV503736.1 | DL196192.1 | AX142065.1 | DL111598.1 | DD367340.1 | HW158486.1 |
| BD297643.1 | HC003093.1 | HI424335.1 | HV503704.1 | DL193792.1 | AX141869.1 | DL111566.1 | DD363463.1 | HW154645.1 |
| BD296226.1 | HB865572.1 | HI424120.1 | HV503672.1 | DL193760.1 | AX141805.1 | DL111534.1 | DD362923.1 | HW154478.1 |
| BD295198.1 | HB865048.1 | HI424056.1 | HV503640.1 | DL184034.1 | AX141741.1 | DL111502.1 | DD370037.1 | HW147480.1 |
| BD280093.1 | HB865016.1 | HI423798.1 | HV190826.1 | DL183395.1 | AX141421.1 | DL101688.1 | DD361298.1 | HW147436.1 |
| BD280365.1 | HB865008.1 | HI414056.1 | HV216285.1 | DL183342.1 | AX141293.1 | DL101656.1 | CS457295.1 | HW147120.1 |
| BD277255.1 | HB864952.1 | HI414023.1 | HV228436.1 | DL183130.1 | AX133918.1 | DL101624.1 | CS457161.1 | HW153796.1 |
| BD274884.1 | HB864920.1 | HI413614.1 | HV306506.1 | FB571241.1 | AX112902.1 | DL106556.1 | CS456709.1 | HV751069.1 |
| BD274313.1 | HB864888.1 | HI516558.1 | HV302065.1 | FB570915.1 | AX107892.1 | DL106524.1 | CS451673.1 | HV752845.1 |
| BD274103.1 | HB864856.1 | HI516098.1 | HV030036.1 | DL105170.1 | AX105806.1 | DL128722.1 | CS450926.1 | HV757521.1 |
| BD273143.1 | HB864824.1 | HI547823.1 | HV037787.1 | DL105138.1 | AX097671.1 | DL128689.1 | CS450618.1 | HV755572.1 |
| BD272106.1 | HB864792.1 | HI284285.1 | FZ435959.1 | DL105106.1 | AX097499.1 | DL120564.1 | DD359190.1 | HV750065.1 |
| DD231213.1 | HB864760.1 | HI574506.1 | FZ427592.1 | DL100596.1 | AX093079.1 | DL120532.1 | DD359137.1 | HV755030.1 |
| AB105219.1 | HB859741.1 | HD115784.1 | FZ435776.1 | DL100564.1 | AX088393.1 | DL115905.1 | DD357929.1 | HV748886.1 |
| AX962032.1 | HB866942.1 | HD114372.1 | FZ430368.1 | DL100532.1 | AX083962.1 | DL115873.1 | DD357893.1 | HV751430.1 |
| AX960366.1 | HB866627.1 | HD079723.1 | FZ430139.1 | DL119986.1 | AX081285.1 | DL115841.1 | DD357634.1 | HV751133.1 |
| AX960219.1 | HB866498.1 | FW345546.1 | FZ429158.1 | DL119954.1 | AX077353.1 | DL095942.1 | DD355757.1 | HV750780.1 |
| AX959233.1 | HC000351.1 | FW345150.1 | FZ437144.1 | DL119922.1 | AX076987.1 | DL095910.1 | CS449871.1 | HV753565.1 |
| AX958637.1 | HB999773.1 | FW344995.1 | FZ423023.1 | DL119890.1 | AX074312.1 | CS227246.1 | A15341.1 | HV753483.1 |
| AX952725.1 | HB999330.1 | FW343708.1 | FZ413140.1 | DL119858.1 | HW256793.1 | CS207912.1 | DD259889.1 | HV743432.1 |
| AX938884.1 | HB976597.1 | FW343191.1 | FZ421415.1 | DL119826.1 | HW256729.1 | CS189619.1 | DD259353.1 | HV743305.1 |
| AX933333.1 | HB975656.1 | HC923350.1 | FZ421065.1 | DL115199.1 | HW254484.1 | CS193220.1 | DD259180.1 | HV743268.1 |
| AX925668.1 | DM201548.1 | HC922963.1 | HH756157.1 | DL115167.1 | HW250836.1 | CS183148.1 | CS305320.1 | HV743230.1 |
| AX924297.1 | DM193328.1 | CS673976.1 | HI401427.1 | DL115135.1 | HW240913.1 | CS177181.1 | CS305016.1 | HV704943.1 |
| AX923404.1 | DM195294.1 | DJ004217.1 | HI401118.1 | DL110071.1 | HW240785.1 | CS174649.1 | CS302596.1 | HV579009.1 |
| AX923370.1 | DM194971.1 | DD491138.1 | HI642776.1 | DL110039.1 | HW239416.1 | CS176597.1 | CS302524.1 | HV588536.1 |
| AX824445.1 | DM203200.1 | CS671007.1 | HI638970.1 | DL110007.1 | HW239194.1 | CS172467.1 | CS299563.1 | HV592455.1 |
| AX824335.1 | HB840199.1 | CS673592.1 | HI637693.1 | DL109975.1 | HW238946.1 | CS166428.1 | DD253306.1 | HV577343.1 |
| AX823862.1 | HB847862.1 | CS667533.1 | HI380642.1 | DL109729.1 | HW238604.1 | CS163802.1 | DD252141.1 | HV582986.1 |
| AX814468.1 | FB740507.1 | DD463380.1 | HI378570.1 | DL109697.1 | HW238383.1 | CS159605.1 | DD251999.1 | HV581984.1 |
| AX814405.1 | GM970527.1 | CS646212.1 | HI378378.1 | DL104963.1 | HW238242.1 | CS150831.1 | DD251309.1 | HV575480.1 |
| AX814282.1 | GM969730.1 | CS645469.1 | HI546343.1 | DL104931.1 | HW238017.1 | CS146539.1 | DD250296.1 | HV601360.1 |
| AX802892.1 | GM009612.1 | CS644659.1 | HI210989.1 | DL100485.1 | HW244294.1 | CS141709.1 | DD248958.1 | HV601083.1 |
| AX809442.1 | DL479996.1 | DD458763.1 | HI210957.1 | DL100389.1 | HW237875.1 | CS141520.1 | DD258250.1 | HV574882.1 |
| AX800000.1 | DL464548.1 | DD453494.1 | HI573166.1 | DL100357.1 | HW237705.1 | CS141488.1 | DD258158.1 | HV585651.1 |
| AX799571.1 | DL476964.1 | DD456101.1 | HW260535.1 | DL100325.1 | HW248654.1 | CS140206.1 | DD253731.1 | HV570595.1 |
| AX796741.1 | DL463068.1 | CS631741.1 | HW260503.1 | DL124138.1 | HW237453.1 | BD194484.1 | CS287712.1 | HV569268.1 |
| AX795428.1 | DL462974.1 | DD152401.1 | HW260471.1 | DL124106.1 | HW242299.1 | BD192893.1 | DD240764.1 | HV568017.1 |
| BD188840.1 | DL462063.1 | DD152114.1 | HW260439.1 | DL119788.1 | HW243066.1 | BD188830.1 | DD240715.1 | HV566060.1 |
| BD187689.1 | DL475566.1 | DD139995.1 | HW260407.1 | DL119756.1 | HW241378.1 | BD187685.1 | DD240667.1 | HV573535.1 |

| | | | | | | | | |
|---|---|---|---|---|---|---|---|---|
| AX787367.1 | DL481573.1 | DD082060.1 | HW260375.1 | DL119724.1 | HW161286.1 | AX787407.1 | DD240609.1 | GM635784.1 |
| AX144211.1 | GM712727.1 | DD147804.1 | HW260343.1 | DL119486.1 | HV925509.1 | AX787360.1 | DD240495.1 | GM647050.1 |
| AX144147.1 | GM712163.1 | DD147772.1 | HW260311.1 | DL119454.1 | HV936328.1 | AX781571.1 | DD239965.1 | GM627516.1 |
| AX144083.1 | GM711971.1 | DD147740.1 | HW260279.1 | DL119422.1 | HV933096.1 | AX773273.1 | DD236644.1 | GM627324.1 |
| AX144017.1 | GM675317.1 | DD147708.1 | HW260247.1 | DL123669.1 | HV932750.1 | AX772723.1 | DD234716.1 | GM654136.1 |
| AX143953.1 | GM827594.1 | DD147676.1 | HW260215.1 | DL123637.1 | HV932358.1 | AX770680.1 | DD248263.1 | GM654104.1 |
| AX143825.1 | GM774149.1 | DD139308.1 | HW260183.1 | DL123605.1 | HV929706.1 | AX766573.1 | DD246849.1 | GM654072.1 |
| AX143761.1 | GM709009.1 | DD081074.1 | HW260151.1 | DL123573.1 | HV803191.1 | AX766183.1 | E49078.1 | GM654040.1 |
| AX143633.1 | GM708977.1 | DD135344.1 | HW260119.1 | DL119383.1 | HV819035.1 | AX752769.1 | E41529.1 | GM654008.1 |
| AX143569.1 | GM643136.1 | DD132315.1 | HW260087.1 | DL119351.1 | HV802957.1 | AX751523.1 | E49299.1 | GM653976.1 |
| AX143505.1 | GM655006.1 | DD117020.1 | HW260055.1 | DL119319.1 | HV802925.1 | BD185257.1 | E40899.1 | GM646983.1 |
| AX143377.1 | GM647801.1 | DD090327.1 | HW260023.1 | DL114827.1 | HV802893.1 | BD181631.1 | E38337.1 | GM646951.1 |
| AX143249.1 | GM044932.1 | DD089286.1 | HW259991.1 | DL016822.1 | HV817778.1 | BD180857.1 | E49569.1 | GM646919.1 |
| AX143057.1 | GM658317.1 | DD102587.1 | HW259959.1 | DL048685.1 | HV777437.1 | AX743922.1 | E32005.1 | GM646887.1 |
| AX142929.1 | GM658285.1 | DD057924.1 | HW259927.1 | DL037076.1 | HV119953.1 | AX722176.1 | E06930.1 | GM646855.1 |
| AX142865.1 | GM658253.1 | DD057892.1 | HW259833.1 | DL037044.1 | HV187356.1 | AX721038.1 | E05828.1 | GM646823.1 |
| AX142673.1 | GM658221.1 | DD057860.1 | HW259679.1 | DL028353.1 | HV195744.1 | BD177605.1 | E05676.1 | GM640020.1 |
| AX142609.1 | GM658189.1 | DD056195.1 | AY659171.1 | DL021039.1 | HW313626.1 | AX720119.1 | E05439.1 | GM639988.1 |
| AX142479.1 | GM658157.1 | DD053288.1 | AY659139.1 | DL012183.1 | HW311851.1 | AX719159.1 | E04045.1 | GM639956.1 |
| AX142223.1 | GM658107.1 | DD161377.1 | AY659107.1 | DL012151.1 | HW308346.1 | AX712182.1 | E03550.1 | GM639924.1 |
| AX142095.1 | GM658075.1 | DD166379.1 | AY659075.1 | DL012119.1 | HW307847.1 | AX710223.1 | E03275.1 | GM639892.1 |
| AX141771.1 | GM658043.1 | DD175999.1 | AY659043.1 | DL012087.1 | HW307815.1 | AX704453.1 | E02924.1 | GM627313.1 |
| AX141707.1 | GN031462.1 | CS204338.1 | AY659011.1 | DL012055.1 | HW316595.1 | AX699455.1 | E02675.1 | GM627286.1 |
| AX141643.1 | GN031429.1 | CS157812.1 | AY658979.1 | DL012023.1 | HW307745.1 | AX699423.1 | E02218.1 | GM627254.1 |
| AX141323.1 | GN031397.1 | CS157780.1 | AY658947.1 | DL011933.1 | HW307713.1 | AX685559.1 | E02078.1 | GM627222.1 |
| AX137848.1 | GN031365.1 | CS157941.1 | AY658915.1 | DL011901.1 | HW307681.1 | AX684479.1 | E01801.1 | GM627190.1 |
| AX133307.1 | GN031333.1 | CS157877.1 | AY658883.1 | DL024280.1 | HW307590.1 | AX683879.1 | E01263.1 | GM627126.1 |
| AX119917.1 | GN031301.1 | CS126033.1 | AY658851.1 | DL024248.1 | HV947300.1 | AX675244.1 | E01112.1 | GM661110.1 |
| AX114294.1 | GN031268.1 | CS119526.1 | AY658819.1 | DL008238.1 | HV947110.1 | AX662212.1 | E00892.1 | GM661078.1 |
| AX113730.1 | GN031236.1 | CS119460.1 | AY658787.1 | DJ491586.1 | HV951952.1 | AX088008.1 | E00758.1 | GM661046.1 |
| AX113546.1 | GN031204.1 | CS119425.1 | AY658755.1 | DJ446853.1 | HV951863.1 | AX082155.1 | E00634.1 | GM661014.1 |
| AX111696.1 | GN031172.1 | CS119392.1 | AY658723.1 | DJ446820.1 | HV940066.1 | AX081489.1 | E00327.1 | GM660982.1 |
| AX103639.1 | GN031139.1 | CS119360.1 | AY658691.1 | DJ438130.1 | HV943219.1 | AX078902.1 | E00208.1 | GM660950.1 |
| AX100348.1 | GN031107.1 | CS119328.1 | AY658659.1 | DJ437310.1 | HV925506.1 | AX078101.1 | DD221367.1 | GM653956.1 |
| AX097515.1 | GN031075.1 | CS119294.1 | AY658627.1 | DJ437220.1 | HV585543.1 | AX077111.1 | DD220927.1 | GM653924.1 |
| AX097408.1 | GN031043.1 | CS119262.1 | AY658595.1 | DJ436978.1 | HV592501.1 | AX076582.1 | DD216682.1 | GM653892.1 |
| AX093095.1 | GN031011.1 | CS119198.1 | AY658563.1 | DJ436740.1 | HV592368.1 | AX076208.1 | DD213977.1 | GM653860.1 |
| AX090035.1 | GN030979.1 | CS119134.1 | AY658531.1 | DJ430773.1 | HV600952.1 | AX063682.1 | DD213884.1 | GM646755.1 |
| AX088788.1 | GN030948.1 | CS119101.1 | AY658499.1 | DJ434333.1 | HV570610.1 | AX060528.1 | DD213852.1 | GM646723.1 |
| AX088744.1 | GN030916.1 | CS119034.1 | AY658467.1 | DJ438350.1 | HV565829.1 | AX059770.1 | HV221453.1 | GM646691.1 |
| AX082942.1 | GN030852.1 | CS119001.1 | AY658435.1 | DJ438283.1 | HV572452.1 | AX058843.1 | HV214665.1 | GM646659.1 |
| AX079103.1 | GN030820.1 | CS118969.1 | AY658403.1 | DJ438200.1 | HV560170.1 | AX057960.1 | HV235900.1 | GM646627.1 |
| AX077276.1 | GN030788.1 | CS118870.1 | AY658371.1 | DJ427847.1 | HV321540.1 | AX056086.1 | HV316807.1 | GM639824.1 |
| HW089633.1 | DM008346.1 | CS118838.1 | AY658339.1 | DJ402676.1 | HV325257.1 | AX052945.1 | HV304097.1 | GM639792.1 |
| HW097736.1 | DM001467.1 | CS118804.1 | AY658307.1 | DJ402638.1 | HV187353.1 | AX052910.1 | HV040140.1 | GM639760.1 |
| HW097695.1 | DM006943.1 | CS118772.1 | AY658275.1 | DJ402540.1 | HV195741.1 | AX049399.1 | HV191508.1 | GM639728.1 |
| HW058279.1 | DM006688.1 | CS118739.1 | AY658243.1 | DJ400831.1 | HV208789.1 | AX038744.1 | HV200690.1 | GM639696.1 |

| | | | | | | | | |
|---|---|---|---|---|---|---|---|---|
| HW046280.1 | DM000514.1 | CS118707.1 | AY658211.1 | DJ400799.1 | HV214872.1 | AX036016.1 | HV203315.1 | GM627085.1 |
| HW047240.1 | DM004999.1 | CS118675.1 | AY658179.1 | DJ399772.1 | FZ426108.1 | AX035967.1 | HV197643.1 | GM646587.1 |
| HV956402.1 | DM003494.1 | CS118643.1 | AY658147.1 | DJ399087.1 | FZ437278.1 | AX034257.1 | HV191334.1 | GM646555.1 |
| HV950005.1 | GM640909.1 | CS118611.1 | AY658115.1 | DJ391348.1 | FZ414809.1 | AX028813.1 | HV182465.1 | GM646523.1 |
| HV944624.1 | GM640877.1 | CS118578.1 | AY658083.1 | DJ390559.1 | FZ417006.1 | AX027867.1 | HV182433.1 | GM646491.1 |
| HV779879.1 | GM640845.1 | CS118510.1 | AY658051.1 | DJ402721.1 | HI930653.1 | AX027713.1 | HV182401.1 | GM646459.1 |
| HV774719.1 | GM628491.1 | CS118445.1 | AY658019.1 | DJ381524.1 | HI661446.1 | AX024046.1 | HV182369.1 | GM646427.1 |
| HV773782.1 | GM047137.1 | CS118413.1 | AY657987.1 | DJ386429.1 | HI660602.1 | AX023644.1 | HV232799.1 | GM639528.1 |
| HV743659.1 | GM662104.1 | CS118380.1 | AY657955.1 | DJ381032.1 | FW498203.1 | AX023611.1 | HV039590.1 | GM626885.1 |
| HW302685.1 | GM654790.1 | CS118312.1 | AY657923.1 | DL023810.1 | FW420450.1 | AX023578.1 | HV038765.1 | GM626853.1 |
| HW286514.1 | GM654758.1 | CS118279.1 | AY657891.1 | DL031540.1 | FW420418.1 | AX019620.1 | HV116992.1 | GM626821.1 |
| HW295057.1 | GM647701.1 | CS118215.1 | AY657859.1 | DL023663.1 | HI369130.1 | AX015733.1 | FW497155.1 | DL101400.1 |
| HW307398.1 | GM628287.1 | CS118116.1 | AY657827.1 | DL023631.1 | HI369087.1 | AX002864.1 | FW420446.1 | DL106066.1 |
| HW294141.1 | GM627963.1 | CS118083.1 | AY657795.1 | DL034130.1 | HI424147.1 | AX010933.1 | FW420414.1 | DL106034.1 |
| HW294076.1 | GM627931.1 | CS118050.1 | AY657763.1 | DL029837.1 | HI424115.1 | AX010292.1 | FW503324.1 | DL106002.1 |
| HW294042.1 | GM654747.1 | CS118015.1 | AY657731.1 | DL029741.1 | HI424083.1 | AX009252.1 | FW501748.1 | DL105970.1 |
| HW293115.1 | GM654715.1 | CS117981.1 | AY657699.1 | DL018278.1 | HI423857.1 | AX008206.1 | FW497024.1 | DL105938.1 |
| HW292749.1 | GM654683.1 | CS114517.1 | AY657667.1 | DL018246.1 | HI423825.1 | AX006888.1 | FW420389.1 | DL105906.1 |
| HW291284.1 | GM654651.1 | CS106385.1 | AY657635.1 | DL018214.1 | HI423793.1 | AX006189.1 | FW420357.1 | DL101197.1 |
| HW291165.1 | GM647593.1 | CS103516.1 | AY657603.1 | DL005433.1 | HI423761.1 | AX003726.1 | FW420325.1 | DL101165.1 |
| HW291036.1 | FB775377.1 | CS103100.1 | AY657571.1 | DJ491553.1 | HI422890.1 | M58161.1 | FW496852.1 | DL096719.1 |
| HW291004.1 | FB766220.1 | CS102993.1 | AY657539.1 | DJ446852.1 | HI414050.1 | A57347.1 | EU880350.1 | DL096655.1 |
| HW290972.1 | FB765830.1 | CS102961.1 | AY657507.1 | DJ446819.1 | HI414018.1 | A38671.1 | HI653887.1 | DL096623.1 |
| HW290909.1 | FB764694.1 | CS102929.1 | AY657475.1 | DJ446678.1 | HI413609.1 | A35735.1 | HI653810.1 | DL096591.1 |
| HW290877.1 | FB764082.1 | CS102865.1 | AY657443.1 | DJ437219.1 | HI503520.1 | A35525.1 | HI653721.1 | DL096559.1 |
| HW290845.1 | FB743973.1 | CS102833.1 | AY657411.1 | DJ437169.1 | HI516093.1 | HV939883.1 | HI653638.1 | DL094731.1 |
| HW290109.1 | FB743925.1 | CS102769.1 | AY657379.1 | DJ437139.1 | HI541102.1 | HV943477.1 | HI653604.1 | DL094699.1 |
| HW298192.1 | FB743893.1 | CS102737.1 | AY657347.1 | DJ437038.1 | HI284312.1 | HV943373.1 | HI653534.1 | DL094667.1 |
| HW298045.1 | FB743861.1 | CS102673.1 | AY657315.1 | DJ436818.1 | HI284280.1 | HV810463.1 | HI653493.1 | DL090956.1 |
| HW289021.1 | FB743819.1 | CS102641.1 | AY657283.1 | DJ436738.1 | HI283484.1 | HV931347.1 | HI653425.1 | DL090924.1 |
| HW285403.1 | FB761198.1 | CS102609.1 | AY657251.1 | DJ434795.1 | HI574533.1 | HV936769.1 | HI653367.1 | DL086988.1 |
| HW279581.1 | FB730018.1 | CS102577.1 | AY657219.1 | DJ434332.1 | HI574501.1 | DL176485.1 | HI653285.1 | DL086956.1 |
| HV778082.1 | GM036250.1 | CS102545.1 | AY657187.1 | DJ438349.1 | HI574469.1 | DL176439.1 | HI653227.1 | DL086924.1 |
| HV764735.1 | GM952594.1 | CS102513.1 | AY657155.1 | DJ438282.1 | HI574437.1 | DL176348.1 | HI652913.1 | DL086892.1 |
| HV764703.1 | GM952003.1 | A10398.1 | AY657123.1 | DJ402675.1 | HI574405.1 | DL176274.1 | HI650593.1 | DL086860.1 |
| HV764567.1 | GM890109.1 | A09771.1 | AY657091.1 | CS457189.1 | HI566234.1 | DL174615.1 | HI648741.1 | DL086828.1 |
| HV774540.1 | GM747114.1 | CQ816950.1 | AY657059.1 | CS450657.1 | HI580814.1 | DL174459.1 | HI647561.1 | DL086796.1 |
| HV766856.1 | GM009454.1 | CQ816909.1 | M12010.1 | CS450512.1 | HI636970.1 | DL182871.1 | HI646923.1 | DL086764.1 |
| HV760607.1 | GM009366.1 | CQ815811.1 | M10337.1 | DD359157.1 | HI636982.1 | DL176760.1 | AY145505.1 | DL086732.1 |
| HV766070.1 | GM008852.1 | AX179515.1 | M21614.1 | DD359125.1 | HI001410.1 | DL091926.1 | HH807086.1 | DL086700.1 |
| HV766038.1 | GM888420.1 | AX179455.1 | M10213.1 | DD357838.1 | HI001363.1 | DL091862.1 | HH806421.1 | DL086668.1 |
| HV750238.1 | GM007127.1 | A28443.1 | K03391.1 | DD357613.1 | HC878856.1 | DL102855.1 | HH797238.1 | DL105877.1 |
| AY658601.1 | GM867482.1 | A20356.1 | M17938.1 | DD357239.1 | HC889136.1 | DL102823.1 | HH796569.1 | DL105845.1 |
| AY658569.1 | GM003570.1 | AX513603.1 | HV549749.1 | DD355905.1 | FW310434.1 | DL102791.1 | HH794725.1 | DL105813.1 |
| AY658537.1 | FB983206.1 | AF430207.1 | HV549375.1 | A19563.1 | FW331631.1 | DL107571.1 | HH793038.1 | DL096530.1 |
| AY658505.1 | FB983187.1 | AF430175.1 | HV549110.1 | CS443398.1 | FW307698.1 | DL117893.1 | HH791752.1 | DL096498.1 |
| AY658473.1 | GM751795.1 | AX512472.1 | HV548897.1 | CS441613.1 | FW332322.1 | DL117861.1 | HH791629.1 | DL096466.1 |

| | | | | | | | | |
|---|---|---|---|---|---|---|---|---|
| AY658441.1 | FB727105.1 | AX496842.1 | HV543844.1 | CS437394.1 | FW305527.1 | DL117829.1 | HH779683.1 | DL110711.1 |
| AY658409.1 | FB726018.1 | BD140208.1 | CQ855864.1 | DD332623.1 | HC757683.1 | DL098807.1 | HH777926.1 | DL110679.1 |
| AY658377.1 | GM731836.1 | BD138724.1 | CQ846365.1 | CS425085.1 | HC307385.1 | DL098775.1 | HH774441.1 | DL090677.1 |
| AY658345.1 | GM879663.1 | BD135984.1 | CQ831243.1 | CS424385.1 | HC312268.1 | DL098743.1 | HH774157.1 | DL086576.1 |
| AY658313.1 | GM840869.1 | BD134118.1 | CQ829257.1 | CS422871.1 | FU262760.1 | DL092733.1 | HH820965.1 | DL086544.1 |
| AY658281.1 | FB709970.1 | BD131923.1 | CQ828064.1 | CS417762.1 | DM475285.1 | DL092701.1 | HH820923.1 | DL086512.1 |
| AY658249.1 | GM041772.1 | BD131694.1 | CQ824413.1 | CS419022.1 | DM474067.1 | DL088958.1 | HH759336.1 | HC045747.1 |
| AY658217.1 | GM840190.1 | BD130816.1 | CQ821160.1 | CS412179.1 | HC306143.1 | DL088926.1 | HH759217.1 | HC045715.1 |
| AY658185.1 | DL120178.1 | BD130784.1 | CQ817001.1 | CS415530.1 | HC302629.1 | DL103301.1 | HH759185.1 | HC045685.1 |
| AY658153.1 | DL120146.1 | BD130686.1 | CQ816924.1 | CS410890.1 | HC305372.1 | DL107957.1 | HH759153.1 | HC045653.1 |
| AY658121.1 | DL120114.1 | BD130584.1 | CQ814473.1 | CS410809.1 | HC291522.1 | DL107925.1 | HH759121.1 | HC045621.1 |
| AY658089.1 | DL120082.1 | BD128434.1 | CQ814051.1 | CS410498.1 | HC291246.1 | DL107893.1 | HH759089.1 | HC045589.1 |
| AY658057.1 | DL120050.1 | BD083927.1 | CQ814019.1 | CS414834.1 | HC289358.1 | DL088596.1 | HH759057.1 | HC045557.1 |
| AY658025.1 | DL120018.1 | BD081728.1 | CQ813987.1 | DD320523.1 | HC289326.1 | DL092321.1 | HD082729.1 | HC045525.1 |
| AY657993.1 | DL094240.1 | BD081536.1 | CQ813955.1 | DD028675.1 | HC292648.1 | DL092289.1 | HD082445.1 | DM378910.1 |
| AY657961.1 | DL094208.1 | BD080721.1 | CQ813923.1 | DD017278.1 | HC288847.1 | DL092257.1 | HD082370.1 | HC035670.1 |
| AY657929.1 | DL094176.1 | BD080155.1 | CQ813891.1 | BD412643.1 | HB649417.1 | DL039444.1 | HD081496.1 | HC037052.1 |
| AY657897.1 | DL090497.1 | BD074964.1 | CQ813859.1 | BD495437.1 | HB645647.1 | DL039316.1 | HD086807.1 | HC022641.1 |
| AY657865.1 | DL086296.1 | BD073869.1 | CQ813827.1 | BD453894.1 | HB856029.1 | DL035692.1 | HD077397.1 | HC025575.1 |
| AY657833.1 | DL086264.1 | BD070738.1 | CQ813795.1 | BD453862.1 | HB855451.1 | DL035660.1 | FW345130.1 | HC025503.1 |
| AY657801.1 | DL086232.1 | BD069700.1 | CQ813762.1 | BD453830.1 | DM179553.1 | DL035628.1 | FW345018.1 | HC025471.1 |
| AY657769.1 | DL086200.1 | BD064738.1 | CQ803117.1 | BD453798.1 | DM179354.1 | DL023555.1 | FW344906.1 | HC025439.1 |
| AY657737.1 | DL110347.1 | BD057300.1 | AX496850.1 | BD443676.1 | DM173762.1 | DL023523.1 | FW344477.1 | HC025407.1 |
| AY657705.1 | DL110283.1 | BD016712.1 | BD138780.1 | BD453710.1 | GM642034.1 | DL023491.1 | FW344003.1 | HC010425.1 |
| AY657673.1 | DL110166.1 | E63271.1 | BD138551.1 | BD453678.1 | GM634847.1 | DL023459.1 | FW343688.1 | HC008344.1 |
| AY657641.1 | DL110134.1 | E50488.1 | BD137363.1 | BD453646.1 | GM634815.1 | DL023427.1 | FW343560.1 | HC020911.1 |
| AY657609.1 | DL110102.1 | BD014211.1 | BD136044.1 | BD453614.1 | GM634783.1 | DL023395.1 | FW343436.1 | HC007805.1 |
| AY657577.1 | DL105169.1 | BD014171.1 | BD134643.1 | BD453577.1 | GM634751.1 | DL020380.1 | HC924277.1 | HC007677.1 |
| AY657545.1 | GM649458.1 | AX490770.1 | BD133701.1 | BD493233.1 | GM634719.1 | DL020348.1 | HC923228.1 | HC007585.1 |
| AY657513.1 | GM649426.1 | E64423.1 | BD131931.1 | BD453564.1 | GM634686.1 | DL020316.1 | HC921644.1 | HC010686.1 |
| AY657481.1 | GM649394.1 | E66925.1 | BD130824.1 | BD453532.1 | GM629881.1 | DL039220.1 | HC920709.1 | DM375846.1 |
| AY657449.1 | GM670032.1 | AX468903.1 | BD130792.1 | BD453500.1 | GM629849.1 | DL035404.1 | HD068974.1 | DM371131.1 |
| AY657417.1 | GM656529.1 | AX467180.1 | BD130694.1 | BD453468.1 | GM629817.1 | DL035340.1 | HD067644.1 | DM370759.1 |
| AY657385.1 | GM656497.1 | HW267812.1 | BD130597.1 | BD453436.1 | GM629753.1 | DL023363.1 | HD066663.1 | DM370661.1 |
| AY657353.1 | GM656465.1 | HW267405.1 | BD129644.1 | BD453404.1 | GM629721.1 | CS593077.1 | HD064619.1 | DM370629.1 |
| AY657321.1 | GM656433.1 | HW266047.1 | BD107331.1 | BD453259.1 | GM634666.1 | CS593398.1 | HD063598.1 | DM370597.1 |
| AY657289.1 | GM649336.1 | HW265870.1 | BD105712.1 | BD453227.1 | GM697108.1 | CS597718.1 | HD053199.1 | DM370565.1 |
| AY657257.1 | GM649248.1 | HW263043.1 | BD103156.1 | BD453195.1 | GM669378.1 | CS585350.1 | HD052975.1 | DM370533.1 |
| AY657225.1 | GM649216.1 | HW262836.1 | BD087160.1 | BD411237.1 | GM656130.1 | CS583660.1 | HD052887.1 | DM370501.1 |
| AY657193.1 | GM635232.1 | HW262742.1 | BD084103.1 | BD410670.1 | GM656098.1 | CS575830.1 | HD048921.1 | DM370469.1 |
| AY657161.1 | GM635168.1 | HW261390.1 | BD082037.1 | BD399404.1 | GM656066.1 | CS574815.1 | HD033610.1 | DM372755.1 |
| AY657129.1 | GM635104.1 | HW261358.1 | BD080776.1 | BD398981.1 | GM656034.1 | CS574126.1 | HD023505.1 | DM209378.1 |
| AY657097.1 | GM635072.1 | HW261326.1 | BD080662.1 | BD497489.1 | GM656002.1 | CS573066.1 | HD057695.1 | HC003096.1 |
| AY657065.1 | GM630266.1 | HW261294.1 | BD080131.1 | BD451762.1 | GM655970.1 | CS569086.1 | HD057663.1 | HB865051.1 |
| AY657033.1 | GM630234.1 | HW261262.1 | BD076413.1 | BD451720.1 | GM648977.1 | CS561171.1 | HD057649.1 | HB865019.1 |
| U05280.1 | GM630202.1 | HW261230.1 | BD075328.1 | BD450790.1 | GM648945.1 | CS559084.1 | GM643410.1 | HB865011.1 |
| M29750.1 | GM635037.1 | HW261198.1 | BD074973.1 | BD408102.1 | GM648913.1 | DD420154.1 | GM636421.1 | HB864955.1 |

| | | | | | | | | |
|---|---|---|---|---|---|---|---|---|
| M36665.1 | GM635005.1 | HW261166.1 | BD074937.1 | BD449814.1 | GM648881.1 | DD431796.1 | GM636389.1 | HB864923.1 |
| M14422.1 | GM634973.1 | HW261134.1 | BD070055.1 | BD449683.1 | GM648849.1 | CS543168.1 | GM636357.1 | HB864891.1 |
| M31350.1 | GM634941.1 | HW261070.1 | BD016720.1 | BD397354.1 | GM642014.1 | CS537859.1 | GM631853.1 | HB864859.1 |
| M17479.1 | GM634909.1 | HW261038.1 | BD014474.1 | BD429543.1 | GM641982.1 | CS503967.1 | GM631821.1 | GM638037.1 |
| M68968.1 | GM634877.1 | HW261006.1 | BD014184.1 | BD407496.1 | GM641950.1 | CS502537.1 | GM631789.1 | GM633469.1 |
| HV549783.1 | GM629975.1 | HW260974.1 | BD014037.1 | BD429342.1 | GM641918.1 | CS501513.1 | GM631757.1 | GM633437.1 |
| HV549721.1 | GM629943.1 | HW260942.1 | AX474387.1 | BD397083.1 | GM641886.1 | CS500249.1 | GM631725.1 | GM633405.1 |
| HV549076.1 | GM629911.1 | HW260910.1 | E59474.1 | BD396313.1 | GM641854.1 | CS189618.1 | GM631693.1 | GM633373.1 |
| HV544276.1 | GM669849.1 | HW260878.1 | AX468158.1 | BD406400.1 | GM641822.1 | CS193219.1 | GM622922.1 | GM633341.1 |
| HV543984.1 | GM656328.1 | HW260846.1 | AX463617.1 | BD428045.1 | GM634634.1 | CS177180.1 | GM622890.1 | GM633309.1 |
| HV543861.1 | GM656296.1 | HW260814.1 | AX459627.1 | BD437691.1 | GM634602.1 | CS174648.1 | GM622858.1 | GM633277.1 |
| HV543557.1 | GM656264.1 | HW260782.1 | AX458620.1 | BD437152.1 | GM634538.1 | CS172466.1 | GM622826.1 | GM633222.1 |
| HV538574.1 | GM656200.1 | HW260750.1 | AX457969.1 | BD445621.1 | GM634506.1 | CS163801.1 | FB507966.1 | GM633158.1 |
| HV538392.1 | GM656168.1 | HW260718.1 | AX454120.1 | BD453344.1 | GM634474.1 | CS159793.1 | GM657911.1 | GM633094.1 |
| HV543339.1 | GM649143.1 | HW260686.1 | AX451312.1 | BD453315.1 | GM629669.1 | CS159350.1 | GM657879.1 | GM625387.1 |
| HV542894.1 | GM649111.1 | HW260654.1 | A34188.1 | GM639554.1 | GM629637.1 | CS159054.1 | GM657847.1 | GM625355.1 |
| HV542744.1 | GM642180.1 | HW260622.1 | AX443535.1 | GM639522.1 | GM629605.1 | CS150653.1 | GM657815.1 | GM625328.1 |
| HV542574.1 | GM642148.1 | HW260558.1 | AX443271.1 | GM626879.1 | GM629573.1 | CS146538.1 | GM657782.1 | GM659131.1 |
| HV542175.1 | GM642116.1 | HW260526.1 | HW353706.1 | GM626847.1 | GM629541.1 | CS144306.1 | GM657750.1 | GM644787.1 |
| HH961284.1 | GM642084.1 | HW260462.1 | HW353617.1 | GM626815.1 | GM629509.1 | CS141707.1 | GM643391.1 | GM644755.1 |
| CS350539.1 | GM642052.1 | HW260430.1 | HW350968.1 | GM660763.1 | GM884205.1 | CS141561.1 | GM643359.1 | GM644723.1 |
| CS348910.1 | DL098006.1 | HW260398.1 | HW350893.1 | GM660731.1 | GM664820.1 | CS141519.1 | GM643327.1 | GM633061.1 |
| CS353261.1 | DL097974.1 | HW260366.1 | HW350669.1 | GM653512.1 | GM655938.1 | CS141487.1 | GM643295.1 | GM625290.1 |
| CS352970.1 | DL097942.1 | HW260334.1 | HW339891.1 | GM653480.1 | GM655906.1 | CS123417.1 | GM643263.1 | GM625258.1 |
| CS283930.1 | DL112632.1 | HW260302.1 | HW349932.1 | GM653448.1 | GM655874.1 | CS122935.1 | GM643231.1 | GM625226.1 |
| CS273346.1 | DL112600.1 | HW260270.1 | HW240825.1 | GM653416.1 | GM655842.1 | CS122404.1 | GM636242.1 | GM625194.1 |
| CS263131.1 | DL112568.1 | HW260238.1 | HW240761.1 | GM653384.1 | GM655810.1 | CS106069.1 | GM636210.1 | GM625162.1 |
| DD159546.1 | DL095728.1 | HW260206.1 | HW240609.1 | GM646385.1 | GM648765.1 | CS179708.1 | GM636178.1 | GM625130.1 |
| DD164827.1 | DL095696.1 | HW260174.1 | HW239296.1 | GM646353.1 | FB654441.1 | CS179214.1 | GM636146.1 | GM624040.1 |
| DD181653.1 | DL095664.1 | HW260142.1 | HW239149.1 | GM646321.1 | FB654377.1 | CS327373.1 | GM636114.1 | GM623976.1 |
| CS203955.1 | DL095632.1 | HW260110.1 | HW238612.1 | GM646289.1 | FB656264.1 | CS326340.1 | GM636082.1 | GM623944.1 |
| AF405700.1 | DL095600.1 | HW260078.1 | HW238258.1 | GM646257.1 | FB654884.1 | CS323586.1 | GM631674.1 | GM625118.1 |
| CS157953.1 | DL095568.1 | HW260046.1 | HW237930.1 | GM646225.1 | FB573599.1 | CS330804.1 | GM631610.1 | GM625086.1 |
| CS157921.1 | DL117405.1 | HW260014.1 | HW237884.1 | GM639390.1 | FB582147.1 | CS329499.1 | GM631578.1 | GM625054.1 |
| CS124670.1 | DL117373.1 | HW259982.1 | HW248668.1 | GM639358.1 | FB580238.1 | DD259338.1 | GM631546.1 | GM625022.1 |
| CS124548.1 | DL117245.1 | HW259950.1 | HW237372.1 | GM639326.1 | FB580194.1 | DD163501.1 | GM631514.1 | GM624990.1 |
| CS124195.1 | DL087957.1 | HW259521.1 | HW242307.1 | GM639294.1 | FB580162.1 | A02319.1 | GM622711.1 | GM624958.1 |
| CS119539.1 | DL087925.1 | HW258920.1 | HW241633.1 | GM639262.1 | FB580130.1 | HW294995.1 | GM622679.1 | GM629371.1 |
| CS119505.1 | DL121756.1 | HW258853.1 | HW243083.1 | GM626715.1 | FB580092.1 | HW285855.1 | GM622647.1 | GM629307.1 |
| CS119472.1 | DL121724.1 | HW257571.1 | HW163839.1 | GM646086.1 | DL200113.1 | HW294128.1 | GM622615.1 | GM648570.1 |
| CS119405.1 | DL125476.1 | HW257337.1 | HW161471.1 | GM646054.1 | DL200077.1 | HW294019.1 | GM622583.1 | GM648538.1 |
| CS119372.1 | DL125444.1 | HW257305.1 | HW155706.1 | GM626448.1 | DL200028.1 | HW102757.1 | GM622551.1 | GM648506.1 |
| CS119340.1 | DL125412.1 | GN112583.1 | HW155504.1 | GM626384.1 | DL189559.1 | HW089259.1 | GM740793.1 | GM641607.1 |
| CS119306.1 | DL125380.1 | GN113556.1 | HW042060.1 | GM626352.1 | DL189462.1 | HW061871.1 | GM657729.1 | GM629262.1 |
| CS119274.1 | DL125348.1 | DM065116.1 | HW042028.1 | GM660377.1 | DL199827.1 | HW061838.1 | GM657697.1 | GM629230.1 |
| CS119242.1 | DL117187.1 | DM064151.1 | HW043558.1 | GM660345.1 | DL199795.1 | HW065696.1 | GM657665.1 | GM629198.1 |
| CS119210.1 | DL117155.1 | DM070849.1 | HW041952.1 | GM653158.1 | DL199731.1 | HW065431.1 | GM657633.1 | GM629166.1 |

| | | | | | | | | |
|---|---|---|---|---|---|---|---|---|
| CS119146.1 | DL117123.1 | GM997865.1 | HW041920.1 | GM653126.1 | DL199699.1 | HW061792.1 | GM657601.1 | GM629134.1 |
| CS119114.1 | DL143640.1 | GM983727.1 | HW041822.1 | GM653094.1 | DL194518.1 | HW065286.1 | GM657569.1 | GM629102.1 |
| CS119079.1 | DL102492.1 | GM996600.1 | HW049406.1 | GM653062.1 | DL201729.1 | HW065253.1 | GM650377.1 | GM634064.1 |
| CS119046.1 | DL102460.1 | GM992954.1 | HW049374.1 | DL101753.1 | DL194298.1 | HW065188.1 | GM661264.1 | GM634032.1 |
| CS119014.1 | DL102428.1 | GM992503.1 | HW049343.1 | DL101721.1 | DL188832.1 | HW064894.1 | GM661232.1 | GM634000.1 |
| CS118981.1 | DL102396.1 | GM992284.1 | HW049311.1 | DL097211.1 | DL198995.1 | HW064860.1 | GM661200.1 | GM633872.1 |
| CS118916.1 | DL097623.1 | GM989818.1 | HW049279.1 | DL097179.1 | DL196374.1 | HW055557.1 | GM654142.1 | GM716519.1 |
| CS118850.1 | DL117863.1 | GM968462.1 | AX144517.1 | DL097147.1 | DL196206.1 | HW064686.1 | GM654078.1 | GM655513.1 |
| CS118817.1 | DL117831.1 | FB986403.1 | AX144325.1 | DL095223.1 | DL193836.1 | HW060679.1 | GM654046.1 | GM655481.1 |
| CS118784.1 | DL108149.1 | GM970621.1 | AX144195.1 | DL121117.1 | DL193804.1 | HW062421.1 | GM654014.1 | GM655449.1 |
| CS118752.1 | DL098809.1 | GM970197.1 | AX144131.1 | DL111833.1 | DL193772.1 | HW062389.1 | GM653982.1 | GM648328.1 |
| CS118719.1 | DL098777.1 | DL462990.1 | AX143937.1 | DL111769.1 | DL193704.1 | HW056337.1 | GM646989.1 | GM648296.1 |
| CS118687.1 | DL092735.1 | GM752130.1 | AX143681.1 | DL128817.1 | DL183247.1 | HW056260.1 | GM646957.1 | AF401226.1 |
| CS118655.1 | DL092703.1 | FB504552.1 | AX143361.1 | DL128752.1 | FB513474.1 | HW062364.1 | GM646925.1 | AX193629.1 |
| AX474397.1 | DL088960.1 | GM831912.1 | AX143297.1 | DL116416.1 | FB513122.1 | HW062203.1 | GM646893.1 | AX191257.1 |
| AX472867.1 | DL088928.1 | GM711810.1 | AX142977.1 | DL116384.1 | FB571337.1 | HW056099.1 | GM646861.1 | AX188584.1 |
| AX470374.1 | DL103303.1 | FB747855.1 | AX142913.1 | DL116352.1 | FB571849.1 | HW055994.1 | GM646829.1 | AX174628.1 |
| E51067.1 | DL107959.1 | FB763993.1 | AX142849.1 | DL111663.1 | CS696141.1 | HW043768.1 | GM640026.1 | AX175196.1 |
| AX468386.1 | DL107927.1 | FB743943.1 | AX142657.1 | DL111631.1 | CS696109.1 | HW042055.1 | GM639994.1 | AX167096.1 |
| AX463654.1 | DL107895.1 | FB743910.1 | AX142593.1 | DL111599.1 | CS696077.1 | HW042023.1 | GM639962.1 | AX166324.1 |
| A22070.1 | DL088598.1 | FB743878.1 | AX142529.1 | DL111567.1 | CS696045.1 | HW043553.1 | GM639930.1 | AX146317.1 |
| AX459920.1 | DL092323.1 | FB743846.1 | AX142463.1 | DL111535.1 | CS696012.1 | HW042011.1 | GM639898.1 | AX145729.1 |
| AX458641.1 | DL092291.1 | FB743792.1 | AX142079.1 | DL101689.1 | CS695980.1 | HW041979.1 | GM639866.1 | AX145697.1 |
| AX458491.1 | DL092259.1 | FB742917.1 | AX142013.1 | DL101625.1 | CS695916.1 | HW041947.1 | GM627260.1 | AX145633.1 |
| AX456484.1 | DL092195.1 | FB708873.1 | AX141949.1 | DL097115.1 | CS695852.1 | HW041915.1 | GM627228.1 | AX145601.1 |
| AX456137.1 | DL092163.1 | GM036137.1 | AX141627.1 | DL097051.1 | CS695820.1 | HW049513.1 | GM627196.1 | AX145569.1 |
| AX455841.1 | DL027845.1 | GM963590.1 | AX141563.1 | DL097019.1 | CS695788.1 | HW049401.1 | GM627164.1 | AX145537.1 |
| AX454131.1 | DL027813.1 | GM889512.1 | AX141499.1 | DL106557.1 | CS695756.1 | HW049369.1 | GM627132.1 | AX145505.1 |
| AX451982.1 | DL027781.1 | GM003698.1 | AX141371.1 | DL106525.1 | CS695724.1 | HW049338.1 | GM661116.1 | AX145473.1 |
| AX451342.1 | DL023873.1 | FB983171.1 | AX141307.1 | DL106496.1 | HW340190.1 | HW049274.1 | GM661084.1 | AX145441.1 |
| AX449254.1 | DL023841.1 | FB753844.1 | AX136890.1 | DL128536.1 | HW350356.1 | HW049242.1 | GM661052.1 | AX145409.1 |
| AX443283.1 | DL023809.1 | GM751815.1 | AX127336.1 | DL120971.1 | HW350018.1 | HW041813.1 | GM661020.1 | AX145376.1 |
| A29528.1 | DL031539.1 | GM618728.1 | AX118835.1 | DL088420.1 | HW339568.1 | HW041781.1 | GM660988.1 | AX145344.1 |
| AX427218.1 | DL027602.1 | GM842513.1 | AX113707.1 | DL088388.1 | HW349773.1 | HV188407.1 | GM660956.1 | AX145312.1 |
| AX418282.1 | HI546143.1 | GM840668.1 | AX113384.1 | DL121884.1 | HW348273.1 | HV182443.1 | GM660924.1 | AX145280.1 |
| AX417134.1 | HI586965.1 | CS498545.1 | AX106732.1 | DL121820.1 | HW347802.1 | HV182411.1 | GM653930.1 | AX145248.1 |
| AX399665.1 | HI210991.1 | CS498440.1 | AX100915.1 | DL121788.1 | HW347706.1 | HV182379.1 | GM653898.1 | AX145216.1 |
| AX395271.1 | HI210959.1 | DD418593.1 | HW070168.1 | DL108086.1 | HW347674.1 | HV217111.1 | GM653866.1 | AX145184.1 |
| AX394162.1 | HC307841.1 | CS490478.1 | HW069814.1 | DL098216.1 | HW347609.1 | HV235481.1 | GM653826.1 | AX145152.1 |
| AX392385.1 | HC307790.1 | CS485316.1 | HW084934.1 | DL098184.1 | HW347577.1 | HV038665.1 | GM646761.1 | AX145120.1 |
| AX391693.1 | FU260910.1 | DD401449.1 | HW068050.1 | DL098152.1 | HW347481.1 | HV038633.1 | GM646729.1 | AX145088.1 |
| AX384792.1 | FU262990.1 | DD401417.1 | HW057894.1 | FB317767.1 | HW347449.1 | HV190743.1 | GM646697.1 | AX145024.1 |
| AX384575.1 | FU259864.1 | DD401223.1 | HW043950.1 | CS368446.1 | HW363740.1 | HV202354.1 | GM646665.1 | AX144992.1 |
| AX384027.1 | FU265431.1 | DD406594.1 | HV951099.1 | CS368382.1 | HW363623.1 | HV031066.1 | GM646633.1 | AX144960.1 |
| AX382031.1 | FU265335.1 | BD242438.1 | HV949971.1 | CS368190.1 | HW347332.1 | HV037778.1 | GM639830.1 | AX144927.1 |
| AX364559.1 | FU262615.1 | BD237933.1 | HV957479.1 | CS368062.1 | HW347368.1 | HI651883.1 | GM639798.1 | AX144895.1 |
| AX364527.1 | FU250449.1 | BD235391.1 | HV942843.1 | CS367998.1 | HW355239.1 | HI651011.1 | GM639766.1 | AX144863.1 |

| | | | | | | | | |
|---|---|---|---|---|---|---|---|---|
| AX364495.1 | FU264495.1 | BD233665.1 | HV780054.1 | CS367678.1 | HW355123.1 | HI646743.1 | GM639734.1 | AX144831.1 |
| AX364463.1 | HC313240.1 | BD231145.1 | HV777036.1 | DL076386.1 | HW344773.1 | AY145511.1 | GM639702.1 | AX144799.1 |
| AX364431.1 | HC299774.1 | BD229960.1 | HV774711.1 | DL075892.1 | HW344609.1 | HH804525.1 | GM639670.1 | AX144767.1 |
| AX364399.1 | HC306162.1 | BD225287.1 | HV775606.1 | DL032424.1 | HW344666.1 | HH797410.1 | GM639638.1 | AX144735.1 |
| AX364367.1 | HC306122.1 | BD223022.1 | HW287350.1 | DL020808.1 | HW344562.1 | HH794935.1 | DL031475.1 | AX144703.1 |
| AX364272.1 | HC306042.1 | DD283509.1 | HW295842.1 | DL016405.1 | HW317109.1 | HH777936.1 | DL031443.1 | AX144671.1 |
| AX364240.1 | HC306002.1 | CS354130.1 | HW302341.1 | DL016373.1 | HW326521.1 | HH980374.1 | DL031411.1 | AX144639.1 |
| AX364208.1 | HC302644.1 | CS283947.1 | HW302135.1 | DL016341.1 | HW326397.1 | HH980342.1 | DL031379.1 | AX144601.1 |
| AX364175.1 | HC302387.1 | CS283066.1 | HW295140.1 | DL016309.1 | HW326283.1 | HH980282.1 | DL031347.1 | AX144537.1 |
| AX358716.1 | HC294278.1 | CS277041.1 | HW295007.1 | DL016277.1 | HW332580.1 | HH980180.1 | DL027442.1 | AX144409.1 |
| AX358635.1 | HC294002.1 | CS276844.1 | HW285859.1 | DL016245.1 | HW338886.1 | HH980142.1 | DL027410.1 | AX144345.1 |
| AX358385.1 | HC289385.1 | CS272331.1 | HW294023.1 | DL011729.1 | HW338502.1 | HH996689.1 | DL027378.1 | AX144023.1 |
| E50927.1 | HC289341.1 | CS272573.1 | HW293050.1 | DL011697.1 | HW338246.1 | HH996626.1 | DL023566.1 | AX143957.1 |
| HW069784.1 | GN359104.1 | CS265238.1 | HW291199.1 | DL011665.1 | HW337990.1 | HH996566.1 | DL023534.1 | HW060703.1 |
| HW084940.1 | GN363011.1 | CS254294.1 | HW291157.1 | DL011633.1 | HW337862.1 | HH998514.1 | DL023502.1 | HW062403.1 |
| HW089629.1 | GN342135.1 | DD228612.1 | HW291060.1 | DL011601.1 | HW337478.1 | HH998435.1 | DL023470.1 | HW060454.1 |
| HW097734.1 | GN348292.1 | DD227397.1 | HW291028.1 | DL048456.1 | HW337350.1 | HH998383.1 | DL023438.1 | HW062378.1 |
| HW097691.1 | GN346712.1 | DD227155.1 | HW065322.1 | DL044743.1 | HV946280.1 | HH998350.1 | DL023406.1 | HW062314.1 |
| HW058261.1 | GN346576.1 | DD226080.1 | HW065224.1 | DL044711.1 | HV940063.1 | HH999974.1 | DL023374.1 | HW056009.1 |
| HW043957.1 | GN346544.1 | DD224935.1 | HW065192.1 | DL044679.1 | HV945979.1 | HH999921.1 | DL020359.1 | HW065987.1 |
| HW046278.1 | GN337975.1 | DD224370.1 | HW064826.1 | DL036599.1 | HV945832.1 | HH979846.1 | DL020327.1 | HW042069.1 |
| HW047238.1 | GN335342.1 | DD223917.1 | HW056518.1 | AF430185.1 | HV944671.1 | HH979771.1 | DL020295.1 | HW042037.1 |
| HV969572.1 | GN116523.1 | DD231453.1 | HW064785.1 | AF430153.1 | HV943062.1 | HH979689.1 | DL020263.1 | HW043567.1 |
| HV956362.1 | GN116458.1 | A01563.1 | HW060690.1 | AX505195.1 | HV942066.1 | HH999888.1 | DL020231.1 | HW041831.1 |
| HV951105.1 | GN116426.1 | CS254928.1 | HW062428.1 | AX504297.1 | HV932151.1 | HH999844.1 | DL020199.1 | HW049415.1 |
| HV957485.1 | GN116394.1 | DD206901.1 | HW060502.1 | AX496852.1 | HV931958.1 | HH999795.1 | DL015796.1 | HW049383.1 |
| BD135143.1 | GN116362.1 | DD206869.1 | HW062368.1 | BD140000.1 | HV929265.1 | HH999742.1 | DL015764.1 | HW049352.1 |
| BD134834.1 | GN116330.1 | DD206837.1 | HW062336.1 | A12386.1 | HV931865.1 | HH998312.1 | DL011178.1 | HW049320.1 |
| BD133733.1 | GN116298.1 | DD206807.1 | HW062207.1 | A21576.1 | HV925765.1 | HH998263.1 | DL047254.1 | HW049256.1 |
| BD131934.1 | GN112541.1 | DD206775.1 | HW042059.1 | A18409.1 | HV931460.1 | HH998226.1 | DL047222.1 | HW043284.1 |
| BD130794.1 | GN094473.1 | DD196865.1 | HW042027.1 | A19288.1 | HV925593.1 | HH996538.1 | DL047190.1 | HW041795.1 |
| BD130762.1 | DM078717.1 | CS236412.1 | HW043557.1 | A18746.1 | HV936710.1 | HH993656.1 | DL047158.1 | HW041763.1 |
| BD130728.1 | GM997884.1 | CS077385.1 | HW042015.1 | A17134.1 | HV936403.1 | HH986620.1 | DL047062.1 | HW041420.1 |
| BD130696.1 | GM996517.1 | CS070586.1 | HW041983.1 | A15513.1 | HV936316.1 | HH979507.1 | DL043240.1 | HW045677.1 |
| BD130601.1 | GM996196.1 | CS061695.1 | HW041951.1 | A14452.1 | HV925258.1 | HH979574.1 | DL043208.1 | HW053745.1 |
| BD129646.1 | GM995279.1 | CS059511.1 | HW041919.1 | A11843.1 | HV819313.1 | HH931978.1 | DL043176.1 | HV975522.1 |
| BD107351.1 | GN009996.1 | AY967396.1 | HW041821.1 | A11120.1 | HV803293.1 | FW394229.1 | DL043144.1 | HV985935.1 |
| BD105714.1 | GM978858.1 | AY967364.1 | HW049405.1 | A08557.1 | HV930265.1 | FW394193.1 | DL043112.1 | HV985903.1 |
| BD103158.1 | GM991951.1 | AY967332.1 | HW049373.1 | A06500.1 | HV932699.1 | FW418985.1 | DL043080.1 | HV975189.1 |
| BD084676.1 | GM976385.1 | AY967300.1 | HW049342.1 | A08523.1 | HV932667.1 | FW396280.1 | DL039263.1 | HW028878.1 |
| BD080234.1 | GM990840.1 | AY967268.1 | HW049278.1 | A08137.1 | HV932595.1 | HH834392.1 | DL039231.1 | HV984389.1 |
| BD076465.1 | GM987033.1 | AY967236.1 | HW043243.1 | A07221.1 | HV929581.1 | DM459949.1 | DL039199.1 | HV984265.1 |
| BD074975.1 | GN000559.1 | AY967204.1 | HW041785.1 | A06387.1 | HV803249.1 | DM384085.1 | DL039167.1 | HW030093.1 |
| BD074939.1 | GM969572.1 | AY967172.1 | HW047222.1 | A05973.1 | HV803217.1 | HW302331.1 | DL039135.1 | AY658411.1 |
| BD070057.1 | GM981442.1 | AY967140.1 | HV985989.1 | A04374.1 | HV803185.1 | HW302008.1 | DL039103.1 | AY658379.1 |
| BD057806.1 | FB740457.1 | AY967108.1 | HV985957.1 | A08630.1 | HV803153.1 | HW294993.1 | DL035447.1 | AY658347.1 |
| BD023525.1 | GM596713.1 | AY967076.1 | HV985925.1 | A07205.1 | HV803121.1 | HW294127.1 | DL018858.1 | AY658315.1 |

| | | | | | | | | |
|---|---|---|---|---|---|---|---|---|
| BD014221.1 | GM970521.1 | AY967044.1 | HV985893.1 | A05109.1 | HV803089.1 | HW293265.1 | DL018826.1 | AY658283.1 |
| BD014186.1 | GM969777.1 | CS483999.1 | AY659361.1 | A01559.1 | HV802951.1 | HW293045.1 | DL034333.1 | AY658251.1 |
| BD014040.1 | GM009609.1 | DD408901.1 | AY659329.1 | A01054.1 | HV802919.1 | HW291152.1 | DL014237.1 | AY658219.1 |
| AX490812.1 | DL477747.1 | DD406592.1 | AY659297.1 | A00096.1 | HV802887.1 | HW291055.1 | DL009385.1 | AY658187.1 |
| AX478582.1 | DL464698.1 | CS122286.1 | AY659265.1 | M13648.1 | HV802878.1 | HW291023.1 | DL009353.1 | AY658155.1 |
| AX474389.1 | DL464545.1 | CS106196.1 | AY659233.1 | K01246.1 | HV781270.1 | HW290991.1 | DL009321.1 | AY658123.1 |
| AX468160.1 | DL476960.1 | CS106106.1 | AY659201.1 | M25030.1 | HV813441.1 | HW290864.1 | DL009289.1 | AY658091.1 |
| AX463620.1 | DL463065.1 | AX958363.1 | AY659169.1 | M60029.1 | HV818508.1 | HW290482.1 | DL009257.1 | AY658059.1 |
| AX459689.1 | DL470110.1 | DQ789393.1 | AY659137.1 | LT908472.1 | HV813040.1 | HW289975.1 | DL009225.1 | AY658027.1 |
| AX458628.1 | DL462971.1 | CS326365.1 | AY659105.1 | AH002289.2 | HV817940.1 | HW249426.1 | DL018683.1 | AY657995.1 |
| AX457971.1 | DL482397.1 | CS323604.1 | AY659073.1 | AH003149.2 | HV822525.1 | HW267892.1 | DL018651.1 | AY657963.1 |
| AX456481.1 | DL475874.1 | CS330847.1 | AY659041.1 | M10493.1 | HV335689.1 | HW267814.1 | DL018619.1 | AY657931.1 |
| AX456125.1 | DL475563.1 | DD271427.1 | AY659009.1 | M12925.1 | HV342019.1 | HW071106.1 | DL009071.1 | AY657899.1 |
| AX454124.1 | DL481568.1 | DD259900.1 | HW335172.1 | DQ250234.1 | HV344554.1 | HW070888.1 | DL009039.1 | AY657867.1 |
| AX451979.1 | DL481427.1 | DD272183.1 | HW314143.1 | DQ250202.1 | HV344179.1 | HW103941.1 | DL014223.1 | AY657835.1 |
| AX451339.1 | FB504566.1 | CS305251.1 | HW158458.1 | DQ250170.1 | HV322367.1 | HW066925.1 | DL014191.1 | AY657803.1 |
| A34190.1 | GM832060.1 | CS302617.1 | HW154714.1 | AY569320.1 | HV321459.1 | HW083278.1 | DL014159.1 | AY657771.1 |
| AX443277.1 | GM712712.1 | CS301584.1 | HW158120.1 | AF003702.1 | HV333165.1 | HW083246.1 | DL014127.1 | AY657739.1 |
| A25453.1 | GM712160.1 | CS298522.1 | HW154013.1 | AY239610.1 | HV332399.1 | HW081894.1 | DL014063.1 | DL107575.1 |
| HV568921.1 | GM692461.1 | CS297025.1 | HW153780.1 | AF283517.1 | HV332154.1 | HW081597.1 | DL026153.1 | DL102582.1 |
| HW295854.1 | GM043849.1 | DD249006.1 | HW153746.1 | HW408632.1 | HV325767.1 | HW085257.1 | DL026121.1 | DL102550.1 |
| HW302531.1 | GM653212.1 | DD258388.1 | HW153543.1 | HW390744.1 | HV324888.1 | HW084538.1 | DL026089.1 | DL102518.1 |
| HW302347.1 | GM646085.1 | CS287896.1 | HW157485.1 | HW239300.1 | HV324784.1 | HW102821.1 | DL026057.1 | DL098008.1 |
| HW295572.1 | GM646053.1 | DD240969.1 | HW144716.1 | HW238614.1 | HV313400.1 | HW102756.1 | DL026025.1 | DL097976.1 |
| HW295460.1 | GM646021.1 | DD246852.1 | HW126679.1 | HW238554.1 | HV042403.1 | HW084182.1 | DL025993.1 | DL113954.1 |
| HW302087.1 | GM626383.1 | E41532.1 | HW126451.1 | HW238262.1 | HV111867.1 | HW089253.1 | DL025961.1 | DL113922.1 |
| HW307554.1 | GM626351.1 | E40757.1 | HW125394.1 | HW237936.1 | HV041734.1 | HW061870.1 | DL014008.1 | DL113890.1 |
| HW295045.1 | GM660344.1 | E38467.1 | HW144441.1 | HW237886.1 | HV120188.1 | HW061837.1 | DL013976.1 | DL113858.1 |
| HW285862.1 | GM653157.1 | E33087.1 | HW144331.1 | HW237845.1 | HV187350.1 | HW065694.1 | DL013944.1 | DL109057.1 |
| HW306958.1 | GM653125.1 | CS082036.1 | HW144299.1 | HW248675.1 | HV189881.1 | HW065429.1 | DL013912.1 | DL109025.1 |
| HW294135.1 | GM653093.1 | CS079575.1 | HW124867.1 | HW242433.1 | HV218624.1 | HW065252.1 | DL013880.1 | DL104291.1 |
| HW294103.1 | GM653061.1 | CS077809.1 | HW124783.1 | HW237374.1 | HV227970.1 | HW065219.1 | DL030108.1 | DL104259.1 |
| HW293279.1 | GM639035.1 | CS059018.1 | HV936378.1 | HW242309.1 | HV245433.1 | HW058476.1 | DL030076.1 | DL104227.1 |
| HW293067.1 | GM639003.1 | AY967394.1 | HV936341.1 | HW248377.1 | HV214869.1 | HW064821.1 | DL030044.1 | DL099589.1 |
| HW291257.1 | GM638971.1 | AY967362.1 | HV819456.1 | HW243422.1 | HV247206.1 | HW055555.1 | DL030012.1 | DL099557.1 |
| HW291160.1 | GM626196.1 | AY967330.1 | HV932712.1 | HW243085.1 | DL105330.1 | HW064717.1 | DL029980.1 | DL093451.1 |
| HW291114.1 | GM652837.1 | AY967298.1 | HV932679.1 | HW163841.1 | DL124349.1 | HW064685.1 | DL046637.1 | DL093419.1 |
| HW291063.1 | GM652805.1 | AY967266.1 | HV929984.1 | HW161159.1 | DL124317.1 | HW060678.1 | DL046605.1 | DL093387.1 |
| HW291031.1 | GM638815.1 | AY967234.1 | HV813904.1 | HW155709.1 | DL124285.1 | HV117704.1 | DL046573.1 | DL093355.1 |
| HW290967.1 | GM638783.1 | AY967202.1 | HV802931.1 | HW155222.1 | DL124253.1 | HV038664.1 | DL046541.1 | DL126132.1 |
| HW290935.1 | HH999071.1 | AY967170.1 | HV818556.1 | HW106638.1 | DL124221.1 | HV190741.1 | DL046509.1 | DL126100.1 |
| HW290904.1 | HH999026.1 | AY967138.1 | HV786534.1 | HW113808.1 | DL124189.1 | HV202353.1 | DL046477.1 | DL122692.1 |
| HW290872.1 | HH998959.1 | AY967106.1 | HV784200.1 | HW097001.1 | DL105302.1 | HV029533.1 | DL042655.1 | DL122660.1 |
| HW290635.1 | HH997297.1 | AY967074.1 | HV777984.1 | HW096746.1 | DL110242.1 | HV037776.1 | DL042623.1 | DL118406.1 |
| HW289993.1 | HH997137.1 | AY967042.1 | HV777544.1 | HW072900.1 | DL110210.1 | HV035553.1 | DL042591.1 | DL118374.1 |
| HW298014.1 | HH998912.1 | AY967010.1 | HV777181.1 | HW096399.1 | DL110178.1 | HV035241.1 | DL038667.1 | DL118342.1 |
| HW297851.1 | HH998858.1 | AY966978.1 | HV775687.1 | HW072653.1 | DL110146.1 | FZ435951.1 | DL038635.1 | DL113746.1 |

| | | | | | | | | |
|---|---|---|---|---|---|---|---|---|
| HW289016.1 | HH998802.1 | AY966946.1 | HV775018.1 | HW096209.1 | DL110114.1 | FZ431250.1 | DL034500.1 | DL108857.1 |
| HW288888.1 | HH998749.1 | CS052516.1 | HV764726.1 | HW104297.1 | DL110082.1 | FZ437283.1 | DL029909.1 | DL108825.1 |
| HW267769.1 | HH997088.1 | CS052396.1 | HV774735.1 | HW071232.1 | DL105149.1 | FZ428023.1 | DL029877.1 | DL108793.1 |
| HW267425.1 | HH996984.1 | CS052327.1 | HV502849.1 | HW071163.1 | DL105117.1 | FZ422903.1 | DL029845.1 | DL108761.1 |
| HW266177.1 | HH996940.1 | CS052294.1 | HV502805.1 | HW070925.1 | DL100703.1 | FW394285.1 | DL029781.1 | DL108729.1 |
| HW266200.1 | HH998657.1 | CS050957.1 | HV502773.1 | HW103756.1 | DL100671.1 | FW394228.1 | DL029749.1 | DL108697.1 |
| HW263021.1 | HH998529.1 | CS038886.1 | HV502741.1 | HW099583.1 | DL100639.1 | FW394192.1 | CS605023.1 | DL099517.1 |
| HW262989.1 | HH996915.1 | AX537266.1 | HV502709.1 | HW102181.1 | DL100607.1 | FW396658.1 | CS604991.1 | DL099485.1 |
| HW262957.1 | HH996798.1 | AX529542.1 | HV502677.1 | HV753525.1 | DL100543.1 | HH834391.1 | CS604959.1 | DL099453.1 |
| HW262846.1 | HH996759.1 | AX523926.1 | HV502645.1 | HV753423.1 | DL119997.1 | FW390813.1 | CS604927.1 | DL099421.1 |
| HW261400.1 | HH994544.1 | AX179536.1 | HV502613.1 | HV743255.1 | DL119965.1 | HH931932.1 | GM639665.1 | DL089540.1 |
| HW261336.1 | HH977457.1 | AX179508.1 | HV502581.1 | HV743219.1 | DL119933.1 | HH830452.1 | GM627086.1 | DL089508.1 |
| HW261304.1 | HH977363.1 | AX179448.1 | HV502549.1 | HV704028.1 | DL119901.1 | HH826942.1 | GM646556.1 | DL089476.1 |
| HW261272.1 | HH980627.1 | AX081416.1 | HV445627.1 | HV703161.1 | DL119869.1 | HH822191.1 | GM646524.1 | DL089444.1 |
| AY657476.1 | HH980526.1 | A28439.1 | HV453154.1 | HV702429.1 | DL119837.1 | FW308820.1 | GM646492.1 | DL040094.1 |
| AY657444.1 | HH980456.1 | AF430203.1 | HV449103.1 | HV701590.1 | DL119805.1 | FW307709.1 | GM646460.1 | DL032037.1 |
| AY657412.1 | HH980383.1 | AF430171.1 | HV448769.1 | HV701237.1 | DL115210.1 | FW332330.1 | GM646428.1 | DL032005.1 |
| AY657380.1 | HH980348.1 | AX511561.1 | HV444452.1 | HV701173.1 | DL115178.1 | FW305532.1 | GM639529.1 | DL031973.1 |
| AY657348.1 | HH980288.1 | AX511249.1 | HV444160.1 | HV701141.1 | DL115146.1 | FW310635.1 | GM639449.1 | DL028132.1 |
| AY657316.1 | HH980187.1 | AX497565.1 | HV349285.1 | HV701109.1 | DL115114.1 | HC757702.1 | GM626854.1 | DL028100.1 |
| AY657284.1 | HH980148.1 | BD138720.1 | FW574608.1 | HV708141.1 | DL115082.1 | HC757133.1 | GM626822.1 | DL028068.1 |
| AY657252.1 | HH980096.1 | BD137475.1 | FW574303.1 | HV701090.1 | DL115050.1 | HC754640.1 | GM653637.1 | DL028036.1 |
| AY657220.1 | HH979862.1 | BD135917.1 | FW571734.1 | HV701058.1 | DL110050.1 | HC731038.1 | GM653605.1 | DL028004.1 |
| AY657188.1 | HH996695.1 | BD134112.1 | HI967746.1 | HV695520.1 | DL110018.1 | HC728199.1 | GM660770.1 | DL027972.1 |
| AY657156.1 | HH996634.1 | BD131919.1 | HI988970.1 | HV695183.1 | DL109986.1 | HC727359.1 | GM653519.1 | DL044237.1 |
| AY657124.1 | HH996579.1 | BD131379.1 | HI987255.1 | HV695119.1 | DL109954.1 | HC688470.1 | GM653487.1 | DL044205.1 |
| AY657092.1 | HH957842.1 | BD130812.1 | FW552193.1 | HV694670.1 | DL109922.1 | HC688407.1 | GM653455.1 | DL044173.1 |
| AY657060.1 | HH998441.1 | BD130747.1 | FW552155.1 | GM661451.1 | DL109890.1 | HC679667.1 | GM653423.1 | DL048246.1 |
| M30296.1 | HH998389.1 | BD130571.1 | FW555624.1 | GM661419.1 | DL109868.1 | HC490851.1 | GM653391.1 | DL048214.1 |
| M14413.1 | HH998356.1 | BD128400.1 | FW555585.1 | GM661387.1 | DL109836.1 | HC490819.1 | GM646392.1 | DL048182.1 |
| M12489.1 | DM170812.1 | BD106888.1 | FW563140.1 | GM661323.1 | DL109804.1 | HC490787.1 | GM646360.1 | DL048150.1 |
| M14030.1 | DM185715.1 | BD081959.1 | FW561687.1 | GM647176.1 | DL109772.1 | HC490755.1 | GM646328.1 | DL048118.1 |
| HW260598.1 | DM187861.1 | BD081528.1 | FW561655.1 | GM647144.1 | DL109740.1 | HC490723.1 | GM646296.1 | DL048086.1 |
| HW260566.1 | DM177461.1 | BD079936.1 | HI378150.1 | GM647112.1 | DL109708.1 | HC490691.1 | GM646264.1 | DL040064.1 |
| HW260534.1 | HB559453.1 | BD077997.1 | HI373235.1 | GM647080.1 | DL105102.1 | HC490527.1 | GM646232.1 | DL040032.1 |
| HW260470.1 | HB489354.1 | BD075753.1 | HI370251.1 | GM647019.1 | DL105070.1 | HC490495.1 | GM639429.1 | DL040000.1 |
| HW260406.1 | DM160524.1 | BD074960.1 | HI369149.1 | GM661300.1 | DL105038.1 | HC047473.1 | GM639397.1 | DL039968.1 |
| HW260374.1 | DM163998.1 | BD070714.1 | HI369096.1 | GM646877.1 | DL104974.1 | HC046869.1 | GM639365.1 | DL039936.1 |
| HW260342.1 | DM163965.1 | BD016708.1 | HI424343.1 | GM646845.1 | DL104942.1 | HC046837.1 | GM639333.1 | DL039904.1 |
| HW260310.1 | DM163584.1 | BD014240.1 | HI424124.1 | GM646813.1 | DL104910.1 | HC046805.1 | GM639301.1 | DL036280.1 |
| HW260278.1 | DM163251.1 | BD014207.1 | HI424092.1 | GM640010.1 | DL100496.1 | HC046773.1 | GM639269.1 | DL047659.1 |
| HW260246.1 | HB485496.1 | BD014167.1 | HI424060.1 | GM639946.1 | DL100464.1 | HC046741.1 | GM626658.1 | DL043677.1 |
| HW260214.1 | HB475496.1 | BD013757.1 | HI423802.1 | GM639914.1 | DL100432.1 | HC046089.1 | GM660567.1 | DL036044.1 |
| HW260182.1 | HB475025.1 | HW381012.1 | HI416743.1 | GM639850.1 | DL100400.1 | DM380270.1 | GM660535.1 | DL027847.1 |
| HW260150.1 | DM074520.1 | HW380980.1 | HI416185.1 | GM627244.1 | DL100368.1 | HC035644.1 | GM653348.1 | DL027815.1 |
| HW260118.1 | DM078714.1 | HW380917.1 | HI416047.1 | GM627180.1 | DL100336.1 | HC021034.1 | GM653316.1 | DL027783.1 |
| HW260086.1 | DM077795.1 | HW380826.1 | HI415740.1 | GM627148.1 | DL124149.1 | HC025519.1 | GM653284.1 | DJ066245.1 |

| | | | | | | | | |
|---|---|---|---|---|---|---|---|---|
| HW260054.1 | DM077437.1 | HW369043.1 | HI414027.1 | GM660972.1 | DL124117.1 | HC025487.1 | GM653252.1 | DJ061634.1 |
| HW260022.1 | GN090926.1 | HW368973.1 | HI413584.1 | GM660940.1 | DL124085.1 | HC010658.1 | GM653220.1 | DJ061586.1 |
| HW259990.1 | GN089988.1 | HW368549.1 | HI503567.1 | GM646777.1 | DL124053.1 | GN031255.1 | GM653188.1 | DJ061538.1 |
| HW259958.1 | GN089840.1 | HW367138.1 | HI516564.1 | GM646745.1 | DL124021.1 | GN031223.1 | GM646093.1 | DJ061490.1 |
| HW259828.1 | GN089184.1 | HW366945.1 | HI516102.1 | GM646713.1 | DL123989.1 | GN031191.1 | GM646061.1 | DJ061443.1 |
| HW258934.1 | GN082812.1 | HW376134.1 | HI516070.1 | GM646681.1 | DL119799.1 | GN031158.1 | DL095038.1 | DJ055281.1 |
| HW258824.1 | GN088778.1 | HW366486.1 | HI284347.1 | GM646649.1 | DL119767.1 | GN031126.1 | DL095006.1 | DJ055198.1 |
| HW257377.1 | GN088028.1 | DL105501.1 | HI284289.1 | GM646617.1 | DL119735.1 | GN031094.1 | DL094974.1 | DJ054969.1 |
| HW257281.1 | L09155.1 | DL094318.1 | HI574510.1 | DL120276.1 | DL119703.1 | GN031062.1 | DL106660.1 | DJ060403.1 |
| HW256993.1 | L08934.1 | DL094286.1 | HI574478.1 | DL120244.1 | DL119671.1 | GN031030.1 | DL106564.1 | DJ053432.1 |
| HW256961.1 | L08857.1 | DL094254.1 | HI574446.1 | DL120212.1 | DL119639.1 | GN030998.1 | DL106532.1 | DJ056348.1 |
| HW256865.1 | M94404.1 | DL094222.1 | HI574414.1 | DL115809.1 | DL119607.1 | GN030966.1 | DL106500.1 | DJ053108.1 |
| HW256833.1 | DM059610.1 | DL094190.1 | HI574382.1 | DL115777.1 | DL115012.1 | GN030935.1 | DL128697.1 | DJ053076.1 |
| HW256801.1 | DM063451.1 | DL094158.1 | AY774318.1 | DL101219.1 | DL114980.1 | GN030903.1 | DL128663.1 | CS810632.1 |
| HW256737.1 | GM633245.1 | DL090543.1 | AY774153.1 | DL094689.1 | DL114948.1 | GN030871.1 | DL128629.1 | CS813005.1 |
| HW254335.1 | GM633213.1 | DL090511.1 | AY774083.1 | DL090946.1 | DL114916.1 | GN030839.1 | DL128597.1 | CS812776.1 |
| HW241233.1 | GM633181.1 | DL090475.1 | AY774021.1 | DL090914.1 | DL114884.1 | GN030807.1 | DL128544.1 | DJ016602.1 |
| HV344866.1 | GM677243.1 | DL090443.1 | AY360475.1 | DL086978.1 | DL114852.1 | DM005050.1 | DL125067.1 | DJ015725.1 |
| HV347035.1 | GM651770.1 | DL090411.1 | HC084825.1 | DL086946.1 | DL127356.1 | DM004980.1 | DL125035.1 | CS803363.1 |
| HV344590.1 | GM644810.1 | DL090379.1 | HV704237.1 | DL105867.1 | DL123932.1 | DM003481.1 | DL125003.1 | CS803330.1 |
| HV339983.1 | GM625185.1 | DL086374.1 | HV704160.1 | DL105835.1 | DL123900.1 | GM983736.1 | DL124971.1 | FB294049.1 |
| HV339951.1 | GM625153.1 | DL086342.1 | HV703904.1 | DL105803.1 | DL123868.1 | GM996602.1 | DL139683.1 | DJ052643.1 |
| HV339659.1 | GM624031.1 | DL086310.1 | HV703156.1 | DL096520.1 | DL123836.1 | CS502723.1 | DL124924.1 | DJ046985.1 |
| HV344476.1 | GM623999.1 | DL086278.1 | HV701232.1 | DL096488.1 | DL123804.1 | CS498713.1 | DL124892.1 | DJ051984.1 |
| HV342127.1 | GM623967.1 | DL086246.1 | HV701200.1 | DL114090.1 | DL123772.1 | CS498442.1 | DL120771.1 | HI214424.1 |
| HV342043.1 | GM658910.1 | DL086214.1 | HV701168.1 | DL114058.1 | DL119593.1 | CS495887.1 | DL120739.1 | HI213910.1 |
| HV341789.1 | GM658878.1 | DL110457.1 | HV701136.1 | DL126230.1 | DL119561.1 | DD418595.1 | DL116208.1 | J01773.1 |
| HV322180.1 | GM658846.1 | DL110425.1 | HV701085.1 | DL126198.1 | DL119529.1 | CS492348.1 | DL116176.1 | HC084829.1 |
| HV321738.1 | GM658814.1 | DL110393.1 | HV701053.1 | DL126166.1 | DL029220.1 | CS485214.1 | DL116144.1 | HC083719.1 |
| HV333650.1 | GM648959.1 | DL110361.1 | HV700920.1 | DL122950.1 | DL029188.1 | DD401419.1 | DL116112.1 | HC083462.1 |
| HI968052.1 | GM641996.1 | DL110329.1 | HV695210.1 | DL122918.1 | DL046044.1 | DD401225.1 | DL116080.1 | U26406.1 |
| HI979338.1 | GM634552.1 | DL110297.1 | HV694828.1 | DL113916.1 | DL046012.1 | DD406596.1 | DL116048.1 | HC058504.1 |
| HI987457.1 | GM634520.1 | DL105492.1 | HV694761.1 | DL113884.1 | DL045980.1 | CS327363.1 | DL120572.1 | DM383667.1 |
| HI987420.1 | GM634488.1 | DL105460.1 | HV694664.1 | DL113852.1 | DL045948.1 | CS332243.1 | DL120540.1 | DM381909.1 |
| FW552195.1 | GM629683.1 | DL105428.1 | HV693336.1 | DL109051.1 | DL045916.1 | CS323575.1 | DL116009.1 | HC055553.1 |
| FW552157.1 | GM664840.1 | DL105396.1 | HV698252.1 | DL109019.1 | DL045884.1 | CS330851.1 | DL115913.1 | HC053855.1 |
| FW561810.1 | GM664581.1 | DL105364.1 | HV698198.1 | DL108987.1 | DL069482.1 | DD259907.1 | DL115881.1 | HC053461.1 |
| FW555652.1 | GM648735.1 | DL105332.1 | HV689458.1 | DL104285.1 | DL049718.1 | DD272443.1 | DL115849.1 | HA641187.1 |
| FW555587.1 | GM648703.1 | DL124351.1 | HV695543.1 | DL104253.1 | DL049686.1 | CS298526.1 | DL106491.1 | HA640546.1 |
| FW563142.1 | GM648671.1 | DL124319.1 | HV593783.1 | DL104221.1 | DL049654.1 | CS297029.1 | DL106459.1 | HA638536.1 |
| FW561657.1 | GM648639.1 | DL124287.1 | HV585143.1 | DL099583.1 | DL045678.1 | DD258731.1 | DL106427.1 | HA643914.1 |
| FW565207.1 | GM648607.1 | DL124255.1 | HV585111.1 | DL122686.1 | GM661371.1 | CS287606.1 | DL128507.1 | DM102622.1 |
| HI578402.1 | GM634455.1 | DL124223.1 | HV579304.1 | DL122654.1 | GM661339.1 | DD236707.1 | DL128473.1 | GN374414.1 |
| HI573696.1 | GM634423.1 | DL124191.1 | HV579104.1 | DL122590.1 | GM654345.1 | DD234186.1 | DL128438.1 | GN368288.1 |
| HI593983.1 | GM634391.1 | DL088563.1 | HI987453.1 | DL048679.1 | GM654281.1 | DD246574.1 | DL124680.1 | GN360115.1 |
| HI588455.1 | GM629468.1 | DL088531.1 | HI987254.1 | DL012049.1 | GM654249.1 | E41536.1 | DL124648.1 | GN360074.1 |
| HI636999.1 | BD396796.1 | DL088499.1 | FW552192.1 | DL012017.1 | GM654217.1 | E41742.1 | DL124616.1 | GN359773.1 |

| | | | | | | | | |
|---|---|---|---|---|---|---|---|---|
| HI636956.1 | BD428750.1 | DL088467.1 | FW552153.1 | DL027809.1 | GM654185.1 | E49739.1 | DL124584.1 | GN359709.1 |
| HI001474.1 | BD427989.1 | DL088435.1 | FW552117.1 | DL027777.1 | GM647160.1 | E41515.1 | DL096919.1 | GN359657.1 |
| HI001374.1 | BD437485.1 | DL117803.1 | FW561806.1 | DL023869.1 | GM647128.1 | E35612.1 | DL096887.1 | GN359593.1 |
| HI003186.1 | AX364425.1 | DL117771.1 | FW555584.1 | DL023837.1 | GM647096.1 | E35158.1 | DL096791.1 | GN359561.1 |
| HI003129.1 | AX364393.1 | DL117707.1 | FW563139.1 | DL023805.1 | GM647035.1 | E33638.1 | DL096759.1 | GN359529.1 |
| HI003065.1 | AX364361.1 | DL117675.1 | FW561686.1 | DL031535.1 | GM647056.1 | E06637.1 | DL123135.1 | GN359497.1 |
| HI003032.1 | AX364266.1 | DL122166.1 | FW562480.1 | DL023690.1 | GM647003.1 | E05894.1 | DL123103.1 | GN359461.1 |
| HI002992.1 | AX364202.1 | DL122134.1 | FW562440.1 | DL023658.1 | GM627490.1 | E05702.1 | DL123039.1 | GN359429.1 |
| HI002928.1 | AX364169.1 | DL122102.1 | FW555361.1 | DL023626.1 | GM627458.1 | E04546.1 | DL118657.1 | GN089987.1 |
| HI001293.1 | AX358344.1 | DL122070.1 | FW553145.1 | DL023594.1 | GM627426.1 | E03586.1 | DL118625.1 | GN089838.1 |
| HI001247.1 | BD008862.1 | DL122038.1 | FW556592.1 | DL015920.1 | GM627362.1 | DD181037.1 | DL114229.1 | GN082914.1 |
| HI001196.1 | BD006310.1 | DL122006.1 | HI424123.1 | DL015888.1 | GM838697.1 | DD163209.1 | DL114197.1 | GN089182.1 |
| HI001145.1 | E59059.1 | DL098335.1 | HI424059.1 | DL015856.1 | GM645627.1 | DD161375.1 | DL114165.1 | GN082811.1 |
| HI001042.1 | E51988.1 | DL125715.1 | HI423801.1 | DL047630.1 | GM638828.1 | DD166378.1 | DL114133.1 | GN088774.1 |
| HI001008.1 | E50758.1 | DL125683.1 | HI422914.1 | DL047598.1 | GM626121.1 | DD165457.1 | DL114101.1 | GN088027.1 |
| HI000962.1 | E59811.1 | DL125651.1 | HI416742.1 | DL047534.1 | GM626089.1 | DD182195.1 | DL114069.1 | L08931.1 |
| HI002865.1 | AX354614.1 | DL125619.1 | HI416046.1 | DL043616.1 | GM626057.1 | CS018384.1 | DL010078.1 | L08853.1 |
| HI002832.1 | AX347311.1 | DL125555.1 | HI414026.1 | DL039671.1 | GM626025.1 | CS017675.1 | DL010046.1 | DM059609.1 |
| HI002794.1 | AX347237.1 | DL121963.1 | HI413617.1 | DL039639.1 | GM625993.1 | CS016564.1 | DL010014.1 | DM063425.1 |
| HI002734.1 | AX347167.1 | DL121931.1 | HI413583.1 | DL039607.1 | GM625961.1 | CS007962.1 | DL014516.1 | DM058821.1 |
| HI002649.1 | AX346935.1 | DL121867.1 | HI516101.1 | CS603447.1 | GM625929.1 | CQ986580.1 | DL014484.1 | DM057114.1 |
| HI002615.1 | AX344911.1 | DL121835.1 | HI284343.1 | CS603415.1 | FB775657.1 | CQ986548.1 | DL014452.1 | DM056876.1 |
| HI002576.1 | AX344505.1 | DL121803.1 | HI284288.1 | CS603383.1 | FB774898.1 | CQ985695.1 | DL009866.1 | DM045511.1 |
| HI004525.1 | AX342770.1 | DL117604.1 | HI574509.1 | CS603351.1 | FB766239.1 | CQ981102.1 | DL009834.1 | DM045355.1 |
| HI004461.1 | AX337980.1 | DL117572.1 | HI574477.1 | CS603319.1 | FB766119.1 | CQ972475.1 | DL009802.1 | DM045093.1 |
| HI002488.1 | AX328156.1 | DL117540.1 | HI574445.1 | CS603287.1 | FB764712.1 | CQ972379.1 | DL009770.1 | DM044903.1 |
| HI002423.1 | AX258729.1 | DL117508.1 | HI574413.1 | CS603255.1 | FB764676.1 | CQ972347.1 | DL009546.1 | DM039597.1 |
| HI002359.1 | AX303607.1 | DL117476.1 | HI574381.1 | CS603223.1 | FB747046.1 | CQ971094.1 | DL009514.1 | DM060871.1 |
| HI002307.1 | AX283499.1 | DL117444.1 | HI574358.1 | CS603191.1 | FB744717.1 | CQ970954.1 | DL009482.1 | DM044786.1 |
| HI000509.1 | AX280171.1 | DL136828.1 | HI588452.1 | CS603159.1 | FB743936.1 | CQ970239.1 | DL009450.1 | GN067848.1 |
| HI006494.1 | AX278052.1 | DL112733.1 | HI636994.1 | CS603127.1 | FB743904.1 | CQ967259.1 | DL009418.1 | GN067816.1 |
| HI180154.1 | HW337786.1 | DL112701.1 | HI001471.1 | CS603095.1 | FB743872.1 | CQ956046.1 | DL030143.1 | GM658447.1 |
| HI178174.1 | HW337658.1 | DL112669.1 | HI001371.1 | CS603063.1 | FB743840.1 | CQ947138.1 | DJ418061.1 | GM644208.1 |
| HI214586.1 | HW337530.1 | DL107864.1 | HI001338.1 | AX142975.1 | FB743786.1 | CQ944176.1 | DJ417436.1 | GM636922.1 |
| HI214554.1 | HW337402.1 | DL107832.1 | HI003260.1 | AX142911.1 | FB761600.1 | CQ944144.1 | DJ402726.1 | GM741828.1 |
| HI214522.1 | HW337274.1 | DL107800.1 | HI003115.1 | AX142847.1 | FB761489.1 | CQ944080.1 | DJ387699.1 | GM741786.1 |
| HI179969.1 | HW337146.1 | DL107768.1 | HI003062.1 | AX142783.1 | GM720306.1 | CQ944048.1 | DJ381037.1 | GM658310.1 |
| HI214456.1 | HW337018.1 | DL107736.1 | HI003026.1 | HV963335.1 | GM036119.1 | CQ944016.1 | DJ381005.1 | GM658278.1 |
| DM102619.1 | HW336734.1 | DL107704.1 | HI002925.1 | HV965969.1 | GM952834.1 | CQ943984.1 | DJ380973.1 | GM658246.1 |
| GM647268.1 | HW329286.1 | DL103100.1 | HI001289.1 | HV969942.1 | GM894236.1 | CQ943952.1 | DJ380941.1 | GM658214.1 |
| GM647204.1 | HW336153.1 | DL103068.1 | HI001244.1 | HV965328.1 | GM893716.1 | CQ943920.1 | DJ380909.1 | GM658100.1 |
| GM647172.1 | HW328841.1 | DL103036.1 | HI001193.1 | HV961910.1 | GM890252.1 | CQ943888.1 | DJ380877.1 | GM658036.1 |
| GM647140.1 | HW328662.1 | DL102972.1 | HI001129.1 | HV961703.1 | GM890124.1 | CQ943856.1 | DJ380845.1 | GM658004.1 |
| GM647108.1 | HW328548.1 | DL098327.1 | HI001095.1 | HV951146.1 | GM963583.1 | CQ902717.1 | DJ380825.1 | GM657972.1 |
| GM647015.1 | HW328426.1 | DL098295.1 | HI001039.1 | HV947311.1 | GM888715.1 | CQ898662.1 | DJ389525.1 | GM650780.1 |
| GM646873.1 | HW328153.1 | DL098263.1 | HI001005.1 | HV953008.1 | GM008871.1 | AX348534.1 | DJ388695.1 | GM650748.1 |
| GM646841.1 | HW318462.1 | DL098231.1 | HI000958.1 | HV940075.1 | GM008186.1 | AX347302.1 | DJ388655.1 | GM650716.1 |

| | | | | | | | | |
|---|---|---|---|---|---|---|---|---|
| GM646809.1 | HW335173.1 | DL098199.1 | HI002861.1 | HV939878.1 | GM872373.1 | AX347230.1 | CS726025.1 | GM650684.1 |
| GM627176.1 | HW314144.1 | DL098167.1 | HI002829.1 | HV943366.1 | GM005938.1 | AX347192.1 | CS724491.1 | GM638811.1 |
| GM653846.1 | HW314004.1 | DL092157.1 | HI002791.1 | HV942439.1 | FB984707.1 | AX347160.1 | CS724458.1 | GM638779.1 |
| DL102849.1 | HW312155.1 | DL092125.1 | FW310608.1 | HV936874.1 | GM003581.1 | AX344904.1 | CS724416.1 | GM638747.1 |
| DL097998.1 | HW311769.1 | DL092029.1 | FW308823.1 | HV931342.1 | FB983217.1 | AX329472.1 | CS723906.1 | FB774865.1 |
| DL097966.1 | HW311237.1 | DL091965.1 | FW307727.1 | HV925517.1 | FB983165.1 | AX328141.1 | CS723025.1 | FB766167.1 |
| DL106645.1 | HW308551.1 | FB329193.1 | FW332334.1 | HV572326.1 | GM681700.1 | AX259245.1 | DJ358886.1 | FB764686.1 |
| DL128715.1 | HW307889.1 | FB341641.1 | FW306358.1 | HV560185.1 | FB753633.1 | AX300166.1 | DJ352791.1 | FB764049.1 |
| DL128582.1 | HW307825.1 | FB299288.1 | FW305539.1 | HV559781.1 | GM968340.1 | DM189902.1 | DJ357781.1 | FB746164.1 |
| DL128492.1 | HW307793.1 | CS368476.1 | FW310641.1 | HV562072.1 | GM618790.1 | HB828504.1 | DJ357683.1 | FB743919.1 |
| DL128423.1 | HW307755.1 | CS368348.1 | HC767600.1 | FW570905.1 | FB725962.1 | HB848020.1 | DJ357651.1 | FB743887.1 |
| DL110157.1 | HW307632.1 | CS368156.1 | HC767367.1 | HV551397.1 | GM842489.1 | HB839493.1 | DJ357619.1 | FB743855.1 |
| DL110125.1 | HW315872.1 | CS368028.1 | AY155577.1 | HV550390.1 | DL176592.1 | HB839222.1 | DJ357587.1 | FB743801.1 |
| DL115125.1 | HW315660.1 | CQ944139.1 | HC757136.1 | AY659392.1 | DL176518.1 | HB838855.1 | DJ363053.1 | FB761790.1 |
| DL110061.1 | HW315399.1 | CQ944107.1 | HC755796.1 | AY659360.1 | DL176460.1 | HB805433.1 | DJ361209.1 | FB708882.1 |
| DL110029.1 | HW315126.1 | CQ944075.1 | HC754643.1 | AY659328.1 | DL176373.1 | HB847057.1 | DJ354220.1 | GM036232.1 |
| DL109997.1 | HW315094.1 | CQ944043.1 | HC732131.1 | AY659296.1 | DL176299.1 | HB838744.1 | DJ360462.1 | GM963794.1 |
| DL109965.1 | HW315062.1 | CQ944011.1 | HC471758.1 | AY659264.1 | DL181467.1 | HB838158.1 | DJ339856.1 | GM889747.1 |
| DL109751.1 | HW314764.1 | CQ943979.1 | HC471630.1 | AY659232.1 | DL174591.1 | HB846181.1 | DJ339824.1 | GM008838.1 |
| DL109719.1 | HW115417.1 | CQ943947.1 | HC449498.1 | AY659200.1 | DL174344.1 | HB837837.1 | DJ339760.1 | GM888379.1 |
| DL109687.1 | HV786598.1 | CQ943915.1 | FU756123.1 | AY659168.1 | FB357050.1 | HB845784.1 | DJ339728.1 | FB660835.1 |
| DL100379.1 | HV784201.1 | CQ943883.1 | FU772121.1 | AY659136.1 | FB357047.1 | HB845502.1 | DJ339696.1 | FB985068.1 |
| DL027869.1 | HV778943.1 | CQ943851.1 | FU759984.1 | AY659104.1 | FB344635.1 | HB844634.1 | DJ339567.1 | FB983601.1 |
| DL027837.1 | HV778011.1 | CQ924120.1 | FU757780.1 | AY659072.1 | FB359901.1 | HB844342.1 | DD359248.1 | FB983180.1 |
| DL027805.1 | HV777184.1 | CQ898657.1 | FU757623.1 | AY659040.1 | DL088351.1 | HB836738.1 | DD357923.1 | GM866017.1 |
| DL027773.1 | HV775688.1 | CQ898593.1 | HC000329.1 | AY659008.1 | DL088319.1 | HB843970.1 | CS449747.1 | GM865625.1 |
| DL023865.1 | HV764759.1 | CQ898561.1 | HB999688.1 | AY658976.1 | DL088287.1 | HB835914.1 | CS448060.1 | GM751777.1 |
| DL023833.1 | HV764695.1 | CQ898259.1 | HB976923.1 | AY658944.1 | DL088255.1 | HB843214.1 | A34698.1 | GM864060.1 |
| DL023801.1 | HV774736.1 | CQ898106.1 | HB976631.1 | AY658912.1 | DL088223.1 | HB842929.1 | A08216.1 | FB725714.1 |
| DL023686.1 | HV764473.1 | CQ897933.1 | DM193129.1 | AY658880.1 | DL088191.1 | HB835269.1 | A04115.1 | DL104893.1 |
| DL023654.1 | HV763988.1 | CQ897087.1 | DM194706.1 | AY658848.1 | DL088159.1 | HB842804.1 | A04056.1 | HC316658.1 |
| DL023622.1 | HV767491.1 | CQ895668.1 | HB848030.1 | AY658816.1 | DL088127.1 | HB842158.1 | CS438983.1 | HC309776.1 |
| DL023590.1 | HV767061.1 | CQ895509.1 | HB847783.1 | AY658784.1 | DL088095.1 | HB841838.1 | CS436337.1 | HC307894.1 |
| DL015916.1 | HV766838.1 | CQ894313.1 | HB847587.1 | AY658752.1 | DL088063.1 | HB841606.1 | CS435188.1 | HC307395.1 |
| DL015852.1 | HV760598.1 | CQ892566.1 | HB847214.1 | AY658720.1 | DL088031.1 | HB841162.1 | DD347349.1 | FU265425.1 |
| DL047626.1 | HV766427.1 | CQ891356.1 | HB838865.1 | AY658688.1 | DL087999.1 | HB840487.1 | CS430544.1 | FU264480.1 |
| DL047594.1 | HV766279.1 | CQ891034.1 | HB847067.1 | AY658656.1 | DL091940.1 | GM045836.1 | CS433055.1 | HC306154.1 |
| DL047562.1 | HV750172.1 | CQ890352.1 | HB846666.1 | AY658624.1 | DL091908.1 | GM870325.1 | CS419505.1 | HC306074.1 |
| DL047530.1 | HV453155.1 | CQ888315.1 | HB846299.1 | AY658592.1 | DL091876.1 | GM647102.1 | CS414532.1 | HC306034.1 |
| CS501453.1 | HV449107.1 | CQ888089.1 | HB837556.1 | AY658560.1 | DL091844.1 | DL236090.1 | CS416479.1 | HC305994.1 |
| CS500211.1 | HV448770.1 | CQ882126.1 | HB845848.1 | AY658528.1 | DL091780.1 | DL233424.1 | CS416215.1 | HC302638.1 |
| CS499470.1 | HV444697.1 | CQ879664.1 | GM645484.1 | AY658496.1 | DL102901.1 | DL233391.1 | CS415768.1 | HC305966.1 |
| CS498446.1 | HV444453.1 | CQ877916.1 | GM645452.1 | AY658464.1 | DL102869.1 | DL206333.1 | CS415549.1 | HC301947.1 |
| CS495897.1 | HV444092.1 | CQ877368.1 | GM645420.1 | AY658432.1 | DL102837.1 | FB711045.1 | CS414842.1 | HB836752.1 |
| DD419542.1 | HV437531.1 | CQ876248.1 | GM633732.1 | AY658400.1 | DL102805.1 | FB708237.1 | CS414768.1 | HB844055.1 |
| DD418599.1 | HV349288.1 | CQ876216.1 | GM633700.1 | AY658368.1 | DL102773.1 | FB729822.1 | DD320063.1 | HB843541.1 |
| DD411493.1 | HV348725.1 | CQ876184.1 | GM659917.1 | AY658336.1 | DL102741.1 | FB670943.1 | DD327030.1 | HB836195.1 |

| | | | | | | | | |
|---|---|---|---|---|---|---|---|---|
| CS492952.1 | HV341344.1 | CQ874999.1 | GM659885.1 | AY658304.1 | AX027726.1 | FB706639.1 | DD326970.1 | HB843281.1 |
| CS490484.1 | HV341221.1 | CQ874734.1 | GM659853.1 | AY658272.1 | AX025599.1 | FB701533.1 | DD329902.1 | HB843031.1 |
| DD401423.1 | HV348192.1 | AX742876.1 | GM659821.1 | AY658240.1 | AX024906.1 | FB701793.1 | DD326839.1 | HB835420.1 |
| DD401391.1 | HV344968.1 | AX741272.1 | GM659789.1 | AY658208.1 | AX023657.1 | FB292672.1 | DD328895.1 | HB842886.1 |
| DD405206.1 | HV344788.1 | AX739989.1 | GM712196.1 | AY658176.1 | AX023591.1 | FB660201.1 | DD321802.1 | HB842309.1 |
| DD405174.1 | M12617.1 | AX722075.1 | GM622126.1 | AY658144.1 | AX020249.1 | FB655218.1 | DD325737.1 | HB841891.1 |
| DD405142.1 | M10227.1 | AX721703.1 | FB506770.1 | AY658112.1 | AX019714.1 | FB665442.1 | CS389363.1 | HB841670.1 |
| DD400871.1 | M20290.1 | BD177393.1 | GM629064.1 | AY658080.1 | AX018591.1 | FB654455.1 | CS389281.1 | HB841201.1 |
| DD406602.1 | M29405.1 | BD177105.1 | GM629000.1 | AY658048.1 | AX014754.1 | FB654385.1 | BD493558.1 | HB840605.1 |
| DD402421.1 | M59864.1 | AX720713.1 | GM655338.1 | AY658016.1 | AX002754.1 | FB654896.1 | BD453748.1 | HB647016.1 |
| DD402389.1 | M12947.1 | AX719255.1 | GM715995.1 | AY657984.1 | AX011483.1 | FB583309.1 | BD453716.1 | HB645732.1 |
| DD259332.1 | M19561.1 | AX718410.1 | GM047090.1 | AY657920.1 | AX011451.1 | CS616568.1 | BD453684.1 | HB645700.1 |
| CS298534.1 | AF434709.1 | AX710591.1 | GM654812.1 | AY657888.1 | AX011419.1 | CS612799.1 | BD453652.1 | HB645446.1 |
| CS297033.1 | LT897790.1 | AX709559.1 | GM654780.1 | HI179930.1 | AX011386.1 | CS612716.1 | BD453620.1 | HB855971.1 |
| DD253212.1 | AH002285.2 | AX705400.1 | GM647723.1 | HI214454.1 | AX011354.1 | CS603245.1 | BD453583.1 | HB855732.1 |
| DD251643.1 | AH003153.2 | AX703433.1 | GM638807.1 | HI214422.1 | AX011274.1 | CS602891.1 | BD453570.1 | DM170959.1 |
| DD248478.1 | M23355.1 | AX702157.1 | GM638775.1 | HI202301.1 | AX010958.1 | CS602859.1 | BD453538.1 | DM170841.1 |
| DD257721.1 | M19977.1 | AX699439.1 | GM037908.1 | HI185517.1 | AX010812.1 | CS602827.1 | BD453506.1 | DM170809.1 |
| CS287610.1 | AY260966.1 | AX699049.1 | FB774857.1 | HI212987.1 | AX010628.1 | CS453712.1 | BD453474.1 | DM188005.1 |
| DD236733.1 | AF265665.1 | AX697547.1 | FB766152.1 | HI546339.1 | AX010382.1 | CS450624.1 | BD453442.1 | HB559438.1 |
| DD234740.1 | HV764472.1 | AX685809.1 | FB747997.1 | HI546307.1 | AX009538.1 | DD361087.1 | BD453410.1 | HB491551.1 |
| E41746.1 | HV763985.1 | AX684117.1 | FB764038.1 | HI586959.1 | AX007102.1 | DD359110.1 | BD442993.1 | HB489351.1 |
| E41631.1 | HV767479.1 | AX683015.1 | FB743948.1 | HI210985.1 | HW386277.1 | DD357934.1 | DM039676.1 | DM163995.1 |
| E43909.1 | HV767009.1 | AX674839.1 | FB743915.1 | HI210953.1 | HW386245.1 | DD357898.1 | DM044956.1 | DM163962.1 |
| E40785.1 | HV766837.1 | AX670729.1 | FB743883.1 | HI473088.1 | HW386117.1 | CS446147.1 | DM039466.1 | DM163579.1 |
| E35567.1 | HV760595.1 | AX665511.1 | FB743851.1 | HI472926.1 | HW385957.1 | CS434838.1 | DM044851.1 | DM163293.1 |
| E33243.1 | HV766278.1 | AX664353.1 | FB743797.1 | HI210930.1 | HW385925.1 | CS419067.1 | DM044667.1 | GM625182.1 |
| E39153.1 | HV750171.1 | AX662250.1 | FB761771.1 | HI580540.1 | HW380899.1 | CS417183.1 | DM060740.1 | GM625150.1 |
| E06689.1 | HV758702.1 | AX662176.1 | FB742928.1 | HI209403.1 | HW380822.1 | CS410924.1 | DM060676.1 | GM624124.1 |
| E04245.1 | HV745359.1 | AX659117.1 | FB730280.1 | HI004347.1 | HW368540.1 | CS410875.1 | DM039091.1 | GM637406.1 |
| E03601.1 | AY657856.1 | AX658988.1 | FB708878.1 | HI004283.1 | HW376104.1 | DD092358.1 | GM644788.1 | GM637374.1 |
| E03341.1 | AY657824.1 | AX657682.1 | FB708634.1 | HI002263.1 | HW366479.1 | DD090697.1 | GM644756.1 | GM637342.1 |
| E02435.1 | AY657792.1 | AX657121.1 | GM894300.1 | HI002225.1 | HW365458.1 | DD090322.1 | GM644724.1 | GM637310.1 |
| E02273.1 | AY657760.1 | AX657086.1 | GM890144.1 | HI002129.1 | HW374969.1 | DD090067.1 | GM637598.1 | GM637278.1 |
| E01826.1 | AY657728.1 | AX657018.1 | GM011261.1 | HI002093.1 | HW364711.1 | DD057921.1 | GM637566.1 | GM631834.1 |
| DD178520.1 | AY657696.1 | BD175782.1 | GM747069.1 | HI000431.1 | HW374641.1 | DD057889.1 | GM637534.1 | GM631802.1 |
| DD172704.1 | AY657664.1 | BD175616.1 | GM008629.1 | HI000392.1 | HW364249.1 | DD057857.1 | GM637502.1 | GM622839.1 |
| DD165468.1 | AY657632.1 | BD174687.1 | GM869491.1 | HI000330.1 | HW381354.1 | DD057237.1 | GM637470.1 | FB508282.1 |
| DD159281.1 | AY657600.1 | BD174403.1 | FB983673.1 | HI179268.1 | HW381049.1 | DD055574.1 | GM633062.1 | GM646874.1 |
| DD182208.1 | AY657568.1 | AX645738.1 | FB983196.1 | DL029127.1 | HW353850.1 | DD050207.1 | GM625323.1 | GM646842.1 |
| DD176005.1 | AY657536.1 | AJ005293.1 | FB983176.1 | DL029095.1 | HW353786.1 | DD044360.1 | GM625291.1 | GM646810.1 |
| CQ890889.1 | AY657504.1 | AX642233.1 | FB754302.1 | DL021781.1 | HW353722.1 | DD032544.1 | GM625259.1 | GM627177.1 |
| CQ888104.1 | AY657472.1 | AX641981.1 | FB753864.1 | DL021749.1 | HW353601.1 | DD030809.1 | GM625227.1 | GM627145.1 |
| CQ878305.1 | AY657440.1 | AX616606.1 | GM866013.1 | DL021717.1 | HW352785.1 | CS204334.1 | GM625195.1 | GM660969.1 |
| GM646807.1 | AY657408.1 | BD173335.1 | GM865613.1 | DL014437.1 | HW341645.1 | CS157809.1 | GM625163.1 | GM660937.1 |
| GM627174.1 | AY657376.1 | BD172990.1 | DL121907.1 | DJ419140.1 | HW112703.1 | CS157777.1 | GM625131.1 | GM646774.1 |
| GM627142.1 | AY657344.1 | BD172300.1 | DL121843.1 | DJ417454.1 | HW072749.1 | CS157938.1 | GM624041.1 | GM646742.1 |

| | | | | | | | | |
|---|---|---|---|---|---|---|---|---|
| FB713812.1 | AY657312.1 | BD171635.1 | FB316582.1 | DJ380842.1 | HW070119.1 | CS157906.1 | GM623977.1 | GM646710.1 |
| FB712614.1 | AY657280.1 | AX614976.1 | CS368300.1 | DJ389438.1 | HW069959.1 | CS144324.1 | GM623945.1 | GM646678.1 |
| FB671029.1 | AY657248.1 | AX608924.1 | DL080572.1 | DJ388717.1 | HW069887.1 | CS125849.1 | GM625119.1 | GM646646.1 |
| FB670159.1 | AY657216.1 | AX608824.1 | DL075915.1 | DJ388673.1 | HW069795.1 | CS119812.1 | GM625087.1 | GM646614.1 |
| FB671495.1 | AY657184.1 | AX601768.1 | DL075239.1 | CS724443.1 | HW084951.1 | CS119523.1 | GM625055.1 | GM627022.1 |
| FB704821.1 | AY657152.1 | AX601533.1 | DL079388.1 | CS723804.1 | HW084912.1 | CS119457.1 | GM625023.1 | GM646478.1 |
| FB705618.1 | AY657120.1 | AX601493.1 | DL074230.1 | CS722058.1 | HW097745.1 | CS119422.1 | GM624991.1 | GM646446.1 |
| FB701801.1 | AY657088.1 | AX601461.1 | DL023420.1 | DJ359611.1 | HW067069.1 | CS119389.1 | GM624959.1 | GM646414.1 |
| FB292676.1 | AY657056.1 | AX601365.1 | DL020373.1 | DJ357734.1 | HW065060.1 | CS119325.1 | GM623933.1 | GM626872.1 |
| FB700926.1 | L22059.1 | AX601333.1 | DL020341.1 | DJ357702.1 | HW060594.1 | CS119291.1 | GM623901.1 | GM626840.1 |
| FB654393.1 | M57709.1 | AX600120.1 | DL020309.1 | DJ357668.1 | HW050523.1 | CS119227.1 | GM623869.1 | GM653537.1 |
| FB654854.1 | M12707.1 | AX599041.1 | DL031201.1 | DJ357636.1 | HV969754.1 | CS119195.1 | GM623837.1 | DL111858.1 |
| DL110155.1 | M12203.1 | AX598989.1 | DL027168.1 | DJ340887.1 | HV956157.1 | CS119131.1 | GM623805.1 | DL111826.1 |
| DL110123.1 | M12541.1 | AX598881.1 | DJ436705.1 | DJ341032.1 | HV951084.1 | CS119098.1 | GM623773.1 | DL111794.1 |
| DL115123.1 | M16484.1 | AX598781.1 | DJ445552.1 | DJ354254.1 | HV956448.1 | GM624874.1 | GM623741.1 | DL128877.1 |
| DL110059.1 | HV549107.1 | AX597794.1 | DJ444638.1 | DJ361193.1 | HV940331.1 | GM624842.1 | GM827624.1 | DL128842.1 |
| DL110027.1 | HV549063.1 | AX597482.1 | DJ433804.1 | DJ339841.1 | HV944849.1 | GM624810.1 | GM658920.1 | DL111656.1 |
| DL109995.1 | HV548892.1 | AX593883.1 | DJ438339.1 | DJ339809.1 | HV780039.1 | GM624778.1 | GM658888.1 | DL111624.1 |
| DL109963.1 | HV547828.1 | AX189499.1 | DJ438304.1 | DJ339777.1 | HV778432.1 | GM624746.1 | GM658856.1 | DL111592.1 |
| DL109749.1 | HV538647.1 | BD268978.1 | DJ438272.1 | DJ339649.1 | HV777003.1 | GM632657.1 | GM658824.1 | DL111560.1 |
| DL109717.1 | HV538539.1 | BD268772.1 | DJ402663.1 | DJ339584.1 | HV776315.1 | GM632573.1 | GM651536.1 | DL097044.1 |
| DL109685.1 | HV543201.1 | BD267697.1 | DJ402563.1 | DJ335763.1 | HV745926.1 | GM632617.1 | GM651504.1 | DL125149.1 |
| DL036525.1 | HV542841.1 | BD265623.1 | DJ402716.1 | DJ331361.1 | HV743685.1 | GM632561.1 | GM651472.1 | DL115937.1 |
| DL032162.1 | HV541513.1 | BD265528.1 | DJ416201.1 | DJ328834.1 | HV743387.1 | GM632529.1 | GM651440.1 | DL112679.1 |
| DL028120.1 | HV533395.1 | BD263697.1 | DJ401326.1 | DJ327067.1 | HW287335.1 | GM632497.1 | GM651408.1 | CS367984.1 |
| DL028088.1 | HV514616.1 | BD263461.1 | DJ400820.1 | DJ327035.1 | HW302703.1 | GM741947.1 | GM644609.1 | DL080574.1 |
| DL028056.1 | HV516644.1 | BD263429.1 | DJ400788.1 | DD117026.1 | HW286646.1 | GM674388.1 | GM637419.1 | DL078919.1 |
| DL028024.1 | HV515988.1 | BD263347.1 | DJ391733.1 | DD116969.1 | HW302546.1 | GM658507.1 | GM637387.1 | DL028557.1 |
| DL027992.1 | HV515723.1 | BD263004.1 | DJ419139.1 | DD089295.1 | HW302369.1 | GM658423.1 | GM637355.1 | DL048708.1 |
| DL036406.1 | HH997583.1 | BD262207.1 | DJ418082.1 | DD102590.1 | HW295586.1 | GM658467.1 | GM637323.1 | DL048676.1 |
| DJ050499.1 | HH997518.1 | BD251749.1 | DJ417453.1 | DD057927.1 | HW295157.1 | GM658411.1 | GM637291.1 | DL023422.1 |
| DJ049403.1 | HH997421.1 | DD029721.1 | CS119491.1 | DD057895.1 | HW294978.1 | GM658379.1 | GM637259.1 | DL020375.1 |
| CS793877.1 | HH997368.1 | DD028796.1 | CS119458.1 | DD057863.1 | HW307047.1 | GM658347.1 | GM623690.1 | DL020343.1 |
| CS720189.1 | HH999688.1 | DD010340.1 | CS119423.1 | DD057835.1 | HW294150.1 | GM651155.1 | GM623658.1 | DL020311.1 |
| CS716741.1 | HH999234.1 | DD010166.1 | CS119358.1 | DD056936.1 | HW294085.1 | GM651123.1 | GM623626.1 | DL035399.1 |
| CS716032.1 | HH999193.1 | DD019581.1 | CS119326.1 | DD053291.1 | HW294051.1 | GM651091.1 | GM623594.1 | DL035367.1 |
| CS675429.1 | HH999144.1 | DD017423.1 | CS119292.1 | DD051870.1 | HW293170.1 | GM651059.1 | GM623562.1 | HH961332.1 |
| CS680678.1 | HH999113.1 | DD014246.1 | CS119260.1 | DD041896.1 | HW293035.1 | GM651027.1 | GM827422.1 | HH961300.1 |
| CS790691.1 | HH999067.1 | BD495453.1 | CS119228.1 | DD047836.1 | HW292758.1 | GM644196.1 | GM658711.1 | HH961268.1 |
| DJ042543.1 | HH999023.1 | BD412573.1 | CS119196.1 | DD046365.1 | HW291216.1 | GM644164.1 | GM658679.1 | HH961236.1 |
| DJ045315.1 | HH998956.1 | BD454044.1 | CS119132.1 | DD034131.1 | HW291174.1 | GM644132.1 | GM658647.1 | HH975403.1 |
| DJ044906.1 | HH997275.1 | BD453875.1 | CS119099.1 | DD032556.1 | HW291077.1 | GM644100.1 | GM658551.1 | HH964094.1 |
| DJ028155.1 | HH997165.1 | BD453843.1 | CS119065.1 | DD027224.1 | HW291045.1 | GM644068.1 | GM651359.1 | HH974559.1 |
| DJ027943.1 | HH978177.1 | BD453811.1 | CS119032.1 | DD023621.1 | HW291013.1 | GM644036.1 | GM651327.1 | HH974426.1 |
| DJ040569.1 | HH998908.1 | BD453777.1 | CS118999.1 | DD020515.1 | HW290981.1 | GM637038.1 | GM651295.1 | HH998096.1 |
| CS721664.1 | HH998852.1 | BD453755.1 | CS118967.1 | DD017641.1 | HW243150.1 | GM637006.1 | GM644400.1 | HH998040.1 |
| CS684777.1 | HH998799.1 | BD453723.1 | CS118934.1 | BD412621.1 | HW241478.1 | GM636974.1 | GM644368.1 | HH997989.1 |

| | | | | | | | | |
|---|---|---|---|---|---|---|---|---|
| CS693394.1 | HH998740.1 | BD453691.1 | CS118902.1 | BD434183.1 | HW163860.1 | GM636942.1 | GM628292.1 | HH999674.1 |
| CS692921.1 | HH997031.1 | BD453659.1 | CS118868.1 | BD453853.1 | HW163794.1 | GM636910.1 | GM627968.1 | HH999562.1 |
| DD490655.1 | HH996981.1 | BD453627.1 | CS118835.1 | BD453821.1 | HW163491.1 | GM636878.1 | GM661746.1 | HH997867.1 |
| CS323577.1 | HH981110.1 | BD453595.1 | CS118802.1 | BD453789.1 | HW163432.1 | GM632470.1 | GM654752.1 | HH997798.1 |
| CS330853.1 | HH998695.1 | BD453545.1 | CS118770.1 | BD433653.1 | HW160632.1 | GM632438.1 | GM654720.1 | HH997753.1 |
| CS319844.1 | HH998654.1 | BD453513.1 | CS118673.1 | BD453765.1 | HW160600.1 | GM632406.1 | GM654688.1 | HH999478.1 |
| CS318802.1 | HH998582.1 | BD453481.1 | CS118609.1 | BD453733.1 | GM656127.1 | GM632374.1 | GM654624.1 | HH999432.1 |
| DD271470.1 | HH998526.1 | BD453449.1 | CS118576.1 | BD453701.1 | GM656095.1 | GM632310.1 | GM654592.1 | HH997675.1 |
| CS305131.1 | HH996912.1 | BD453417.1 | CS118541.1 | BD453669.1 | GM656063.1 | GM632278.1 | GM654559.1 | HH997632.1 |
| CS298528.1 | HH996793.1 | BD453378.1 | CS118475.1 | CS016530.1 | GM656031.1 | GM623507.1 | GM647566.1 | HH997575.1 |
| CS297031.1 | HH996756.1 | BD453240.1 | CS118443.1 | CS008510.1 | GM655999.1 | GM623475.1 | GM627895.1 | HH997361.1 |
| DD258733.1 | HH994534.1 | BD453208.1 | CS118411.1 | CQ990420.1 | GM655967.1 | GM623443.1 | GM627863.1 | HH999226.1 |
| DD238316.1 | HH977454.1 | BD453121.1 | CS118378.1 | CQ986616.1 | GM648974.1 | GM623411.1 | GM627831.1 | HH999182.1 |
| DD236709.1 | HH977344.1 | BD453018.1 | CS118277.1 | CQ985742.1 | GM648942.1 | GM623379.1 | GM627799.1 | HH999137.1 |
| DD234188.1 | HH980622.1 | BD432670.1 | CS118213.1 | CQ981106.1 | GM648910.1 | GM623347.1 | GM870691.1 | HH999106.1 |
| DD246862.1 | HH980521.1 | BD442218.1 | CS118178.1 | CQ973468.1 | GM648878.1 | GM643995.1 | GM661717.1 | HH999057.1 |
| E41538.1 | HH980450.1 | BD432364.1 | CS118114.1 | CQ977700.1 | GM648846.1 | GM643963.1 | GM661653.1 | HH998997.1 |
| E41744.1 | HH968270.1 | BD399670.1 | CS118081.1 | CQ977391.1 | GM648814.1 | GM643931.1 | GM661621.1 | HH998949.1 |
| E39297.1 | HH980378.1 | BD410314.1 | CS118048.1 | CQ972479.1 | GM641915.1 | GM643899.1 | GM661589.1 | HH997229.1 |
| E40780.1 | HH980285.1 | BD399156.1 | CS118013.1 | CQ972351.1 | GM641883.1 | GM643866.1 | GM661557.1 | HH997158.1 |
| E36564.1 | HH980183.1 | BD399086.1 | CS117979.1 | CQ971102.1 | GM634599.1 | GM636837.1 | GM661525.1 | HH978150.1 |
| E35565.1 | HH980145.1 | BD441340.1 | CS116764.1 | CQ970333.1 | GM634567.1 | GM636805.1 | GM654531.1 | HH998901.1 |
| E38117.1 | HH980077.1 | BD409481.1 | CS114515.1 | CQ956054.1 | GM634535.1 | GM636773.1 | GM654499.1 | HH998843.1 |
| E06648.1 | HH979856.1 | BD497801.1 | CS106383.1 | CQ947144.1 | GM634503.1 | GM636741.1 | GM654467.1 | HH998792.1 |
| E05543.1 | HH996692.1 | BD495993.1 | CS102991.1 | CQ947027.1 | GM634471.1 | GM636709.1 | GM654435.1 | HH998733.1 |
| E05061.1 | HH996629.1 | BD451733.1 | CS102959.1 | CQ944180.1 | GM629666.1 | GM636677.1 | GM654403.1 | HH997078.1 |
| E04054.1 | HH996572.1 | BD451675.1 | CS102895.1 | CQ944148.1 | GM629634.1 | GM632269.1 | GM654371.1 | HH997024.1 |
| E03594.1 | HH998517.1 | BD398164.1 | CS102799.1 | CQ944116.1 | GM629602.1 | GM632237.1 | GM647282.1 | HH996970.1 |
| E03107.1 | HH998438.1 | BD408122.1 | CS102767.1 | CQ944084.1 | GM629570.1 | GM632205.1 | DL021041.1 | HH998636.1 |
| E02965.1 | HH998386.1 | BD408083.1 | CS102735.1 | CQ944052.1 | GM629538.1 | GM632173.1 | DL021009.1 | HH998573.1 |
| E02694.1 | HH998353.1 | BD439819.1 | CS102703.1 | CQ944020.1 | GM664730.1 | GM632141.1 | DL012185.1 | HH996937.1 |
| E02433.1 | HH999977.1 | BD408012.1 | AX151758.1 | CQ943988.1 | GM655935.1 | GM656722.1 | DL012153.1 | HH996905.1 |
| CS118016.1 | HH999928.1 | BD449626.1 | AX145679.1 | CQ943956.1 | GM655903.1 | GM656690.1 | DL012121.1 | HH996784.1 |
| CS117982.1 | HH979780.1 | BD449396.1 | AX145647.1 | CQ943924.1 | GM655871.1 | GM656658.1 | DL012089.1 | DM130823.1 |
| CS114614.1 | HH979692.1 | BD418810.1 | AX145615.1 | CQ943892.1 | GM655839.1 | GM656626.1 | DL012057.1 | HB427190.1 |
| CS106691.1 | HH999892.1 | BD418447.1 | AX145583.1 | CQ943860.1 | GM655807.1 | GM656594.1 | DL012025.1 | HB403646.1 |
| CS106386.1 | HH999850.1 | BD429173.1 | AX145551.1 | CQ918244.1 | GM648768.1 | GM656562.1 | DL011993.1 | HB396582.1 |
| CS104310.1 | HH999749.1 | BD396895.1 | AX145519.1 | CQ898815.1 | GM655767.1 | GM649569.1 | DL011935.1 | HB398947.1 |
| CS103519.1 | HH998317.1 | BD396646.1 | AX145487.1 | CQ898666.1 | GM648750.1 | GM649537.1 | DL011871.1 | HB386678.1 |
| CS102994.1 | HH998266.1 | BD428592.1 | AX145455.1 | CQ898634.1 | GM648686.1 | GM649505.1 | DL024282.1 | HB385992.1 |
| CS102930.1 | HH998229.1 | BD459658.1 | AX145423.1 | CQ898602.1 | GM648654.1 | GM649473.1 | DL024250.1 | GN368630.1 |
| CS102898.1 | HH998182.1 | BD437269.1 | AX145390.1 | CQ898268.1 | GM648622.1 | GM649441.1 | DL010541.1 | HB340484.1 |
| CS102802.1 | HH996738.1 | BD437122.1 | AX145358.1 | CQ895878.1 | GM641819.1 | GM649409.1 | DL010509.1 | HA641570.1 |
| CS102738.1 | HH993668.1 | DD171941.1 | AX145326.1 | CQ895537.1 | GM641787.1 | GM670049.1 | DL010477.1 | HA641506.1 |
| CS102642.1 | HH986623.1 | DD165451.1 | AX145294.1 | AX347308.1 | GM641755.1 | GM656544.1 | DL010445.1 | HA641169.1 |
| CS102610.1 | HH979549.1 | DD165093.1 | AX145262.1 | CS583568.1 | GM641723.1 | GM656512.1 | DL010413.1 | HA635490.1 |
| CS102578.1 | HH979620.1 | DD159568.1 | AX145230.1 | CS575764.1 | GM641691.1 | GM656480.1 | DL038787.1 | HA640062.1 |

| | | | | | | | | |
|---|---|---|---|---|---|---|---|---|
| CS102546.1 | HH979578.1 | DD170880.1 | AX145198.1 | CS575285.1 | GM641658.1 | GM656448.1 | DL038755.1 | HA643884.1 |
| CS102514.1 | HH836759.1 | DD182189.1 | AX145166.1 | CS573058.1 | GM641626.1 | GM656416.1 | DL038723.1 | GM741819.1 |
| CS102482.1 | FW394383.1 | DD175956.1 | AX145134.1 | CS543944.1 | GM634438.1 | GM656384.1 | DL035067.1 | GM643134.1 |
| HW338126.1 | FW394289.1 | DD175924.1 | AX145102.1 | CS546679.1 | GM634406.1 | GM656352.1 | DL035035.1 | GM037886.1 |
| HW337998.1 | FW394232.1 | DD175899.1 | AX145070.1 | DD420113.1 | GM634374.1 | GM649359.1 | DL030720.1 | FB766247.1 |
| HW337742.1 | FW394196.1 | CS204330.1 | AX145038.1 | DD431305.1 | GM634342.1 | GM649263.1 | DL030688.1 | FB766140.1 |
| HW337614.1 | GN359495.1 | CS001493.1 | AX145006.1 | DD431732.1 | GM634310.1 | GM649231.1 | DL026751.1 | FB765676.1 |
| HW337486.1 | GN365531.1 | CS001429.1 | AX144974.1 | CS540032.1 | GM634278.1 | GM649199.1 | DL026719.1 | DL117009.1 |
| HW337358.1 | GN359363.1 | CS001397.1 | AX144942.1 | BD235253.1 | GM629446.1 | GM642590.1 | DL026687.1 | DL131956.1 |
| HW337102.1 | GN359331.1 | CS175875.1 | AX144909.1 | BD233775.1 | GM629451.1 | GM642558.1 | DL026655.1 | DL109009.1 |
| HW336846.1 | GN365389.1 | CS161111.1 | AX144877.1 | BD231755.1 | GM629409.1 | GM642526.1 | DL026623.1 | DL137140.1 |
| HW321321.1 | GN359194.1 | CS157805.1 | AX144845.1 | BD227088.1 | GM629377.1 | GM642518.1 | DL026591.1 | DL122413.1 |
| HW336482.1 | GN359162.1 | CS157773.1 | AX144813.1 | BD226834.1 | GM629345.1 | GM642461.1 | DL026559.1 | DL033944.1 |
| HV945711.1 | GN359036.1 | CS157934.1 | AX144781.1 | BD224578.1 | GM629313.1 | GM642429.1 | DL019572.1 | DL029450.1 |
| HV944900.1 | GN362988.1 | CS157902.1 | AX144749.1 | BD223710.1 | GM655601.1 | GM635247.1 | DL019540.1 | DL028020.1 |
| HV943437.1 | GN348286.1 | CS157870.1 | AX144717.1 | BD222636.1 | GM655569.1 | GM635215.1 | DL019508.1 | DL027988.1 |
| HV943218.1 | GN346705.1 | CS146893.1 | AX144685.1 | BD218698.1 | GM648576.1 | GM635183.1 | DL019476.1 | DJ493852.1 |
| HV942211.1 | GN346570.1 | CS131903.1 | AX144653.1 | BD211169.1 | GM648544.1 | GM635151.1 | DL019444.1 | DJ432945.1 |
| HV816133.1 | GN346538.1 | CS126734.1 | AX144621.1 | BD206027.1 | GM648512.1 | GM635119.1 | DL019412.1 | DJ438372.1 |
| HV815904.1 | GN131877.1 | CS124735.1 | AX144565.1 | BD204764.1 | GM648480.1 | GM635087.1 | DL014913.1 | DJ433799.1 |
| HV820273.1 | GN116517.1 | CS124689.1 | AX144501.1 | DD282356.1 | GM648448.1 | GM640685.1 | DL014881.1 | DJ438299.1 |
| HV815383.1 | GN116485.1 | CS124649.1 | AX144245.1 | CS353169.1 | GM648416.1 | GM640653.1 | DL030500.1 | DJ402658.1 |
| HV929321.1 | GN116452.1 | CS124527.1 | AX144115.1 | CS352389.1 | GM641613.1 | GM628299.1 | DL026499.1 | CS479727.1 |
| HV932153.1 | GN116388.1 | CS123988.1 | AX143985.1 | CS283951.1 | GM646632.1 | GM628267.1 | DL026467.1 | CS476729.1 |
| HV937159.1 | DM075108.1 | CS120464.1 | AX143921.1 | CS272581.1 | GM639829.1 | GM628235.1 | DL026435.1 | CS469242.1 |
| HV925771.1 | DM073116.1 | CS106094.1 | AX143793.1 | CS266077.1 | GM639797.1 | GM628203.1 | DL026403.1 | CS467715.1 |
| HV936714.1 | DM064158.1 | CS181890.1 | AX143729.1 | CS265594.1 | GM639765.1 | GM628171.1 | DL026371.1 | CS465351.1 |
| HV931230.1 | DM077787.1 | CS179785.1 | AX143601.1 | CS259464.1 | GM639733.1 | GM628139.1 | DL022469.1 | CS464566.1 |
| HV925505.1 | DM077434.1 | CS179628.1 | AX143345.1 | DD224447.1 | GM639701.1 | GM046521.1 | DJ061637.1 | DD400170.1 |
| HV939186.1 | GM741826.1 | CS179195.1 | AX143089.1 | DD224114.1 | GM639669.1 | GM627975.1 | DJ061589.1 | DD400138.1 |
| HV819790.1 | GM658308.1 | CS328406.1 | AX143025.1 | DD231457.1 | GM639637.1 | GM627943.1 | DJ061541.1 | CS459120.1 |
| HV925261.1 | GM658244.1 | CS323758.1 | AX142961.1 | AX960222.1 | GM627090.1 | GM715482.1 | DJ061493.1 | DD367715.1 |
| HV803709.1 | GM658212.1 | CS323601.1 | AX142577.1 | DD214003.1 | GM627004.1 | GM661913.1 | DJ061445.1 | DD361269.1 |
| HV782456.1 | GM658098.1 | CS330844.1 | AX142447.1 | DD213986.1 | GM626962.1 | GM661881.1 | DJ055491.1 | DD362512.1 |
| HV930430.1 | GM658066.1 | CS329674.1 | AX142383.1 | DD218559.1 | GM626930.1 | GM661849.1 | DJ055283.1 | DD368131.1 |
| HV930334.1 | GM658034.1 | CS329309.1 | AX142063.1 | DD213538.1 | GM646600.1 | GM661817.1 | DJ057653.1 | DD361303.1 |
| HV819501.1 | GM658002.1 | CS320116.1 | AX141933.1 | DD220635.1 | GM646560.1 | GM661785.1 | DJ060405.1 | CS179945.1 |
| HV819418.1 | GM657970.1 | CS318715.1 | AY967012.1 | DD221986.1 | GM646528.1 | GM661753.1 | DJ053434.1 | CS326611.1 |
| HV819315.1 | GM653505.1 | CS204329.1 | AY966980.1 | DD211594.1 | GM646496.1 | GM661721.1 | DJ056350.1 | CS332077.1 |
| HV803295.1 | GM653473.1 | CS000445.1 | AY966948.1 | DD182401.1 | GM646464.1 | GM654727.1 | DJ053110.1 | CS323606.1 |
| HV782098.1 | GM653441.1 | AF408185.1 | CS055058.1 | DD210741.1 | GM646432.1 | GM654695.1 | CS810634.1 | CS323573.1 |
| HV782034.1 | GM653409.1 | CS157804.1 | CS052518.1 | DD206905.1 | GM639629.1 | GM654663.1 | CS810523.1 | CS330110.1 |
| HV932734.1 | GM653377.1 | CS157772.1 | CS052398.1 | DD206873.1 | GM639533.1 | GM654631.1 | CS813009.1 | GN094548.1 |
| HV932701.1 | GM646314.1 | CS157933.1 | CS052329.1 | DD206841.1 | GM639445.1 | GM654599.1 | FB292095.1 | GN094516.1 |
| HV932346.1 | GM639415.1 | CS157901.1 | CS050999.1 | DD206779.1 | GM639493.1 | GM654566.1 | FB292231.1 | GN094484.1 |
| HV929583.1 | GM639383.1 | CS146889.1 | CS050959.1 | DD196869.1 | GM626890.1 | GM647573.1 | DJ016604.1 | GN094452.1 |
| HV803251.1 | GM639351.1 | CS144062.1 | BD171805.1 | CS236438.1 | GM626858.1 | GM640610.1 | FB294855.1 | GN113450.1 |

| | | | | | | | | |
|---|---|---|---|---|---|---|---|---|
| HV803219.1 | GM626708.1 | CS131898.1 | AX601464.1 | DD181039.1 | GM626826.1 | GM640578.1 | DJ051986.1 | DM074118.1 |
| HV803187.1 | FB705921.1 | CS126620.1 | AX599955.1 | CQ897112.1 | GM626794.1 | GM640546.1 | DJ050212.1 | DM078971.1 |
| HV803155.1 | FB701540.1 | CS124734.1 | AX597567.1 | CQ879682.1 | GM653641.1 | GM627902.1 | DJ048835.1 | DM064968.1 |
| HV803123.1 | FB701811.1 | CS124688.1 | AX594149.1 | CQ877616.1 | GM653609.1 | GM627838.1 | DJ048803.1 | DM070630.1 |
| HV803091.1 | FB707036.1 | CS124648.1 | AX593490.1 | CQ875502.1 | GM653577.1 | GM627806.1 | CS793895.1 | GM643707.1 |
| HV819023.1 | FB660220.1 | CS123927.1 | AX587695.1 | CQ875006.1 | GM660870.1 | GM627774.1 | CS721584.1 | GM643675.1 |
| HV802953.1 | FB654399.1 | CS120792.1 | BD160932.1 | CQ874760.1 | GM660774.1 | GM627742.1 | CS800056.1 | GM643635.1 |
| HV802921.1 | DL199774.1 | CS119743.1 | BD143613.1 | CQ873406.1 | GM660742.1 | GM627717.1 | CS716774.1 | GM636646.1 |
| HV802889.1 | DL193783.1 | CS119517.1 | AX576374.1 | CQ871401.1 | GM653523.1 | GM870422.1 | CS805314.1 | GM636614.1 |
| HV802875.1 | FB513355.1 | CS119451.1 | AX573311.1 | CQ869382.1 | GM653491.1 | GM661692.1 | CS491151.1 | GM636582.1 |
| HV781300.1 | FB571348.1 | CS119417.1 | AX557106.1 | CQ868889.1 | GM653459.1 | GM661660.1 | CS490620.1 | GM636550.1 |
| HV813458.1 | FB571292.1 | CS119384.1 | AX556837.1 | CQ867377.1 | GM653427.1 | GM661628.1 | CS489145.1 | GM636518.1 |
| HV822642.1 | CS696184.1 | CS119352.1 | AX555842.1 | CQ859833.1 | DL033675.1 | GM661596.1 | CS483004.1 | GM636486.1 |
| HV822604.1 | CS696088.1 | CS119320.1 | AX555094.1 | CQ857855.1 | DL033643.1 | GM661564.1 | CS482965.1 | GM636454.1 |
| HV817954.1 | CS696056.1 | CS119286.1 | AX543901.1 | CQ855852.1 | DL033611.1 | GM661532.1 | CS482930.1 | GM623115.1 |
| HV822533.1 | CS695991.1 | CS119222.1 | AX539582.1 | CQ854111.1 | DL033579.1 | GM654538.1 | DD401435.1 | GM623083.1 |
| HV822398.1 | CS695959.1 | CS119190.1 | AX537268.1 | CQ848455.1 | DL033547.1 | GM654506.1 | DD401403.1 | GM623051.1 |
| HV822309.1 | CS052369.1 | CS119158.1 | AX076541.1 | CQ830291.1 | DL029507.1 | GM654474.1 | DD405218.1 | GM623019.1 |
| HV817131.1 | CS052301.1 | CS119126.1 | AX074306.1 | CQ820999.1 | DL029475.1 | GM654442.1 | DD405186.1 | GM622987.1 |
| HV816797.1 | CS051045.1 | CS119092.1 | AX073547.1 | CQ816994.1 | DL029443.1 | GM654410.1 | DD405154.1 | GM622955.1 |
| HV821236.1 | CS050716.1 | CS119059.1 | AX068005.1 | CQ816952.1 | DL029379.1 | GM654378.1 | DD405122.1 | GM650441.1 |
| HV780283.1 | CS048043.1 | CS119026.1 | AX052968.1 | CQ816914.1 | DL029347.1 | GM647385.1 | DD406802.1 | GM650409.1 |
| HI002084.1 | AX375489.1 | CS118993.1 | AX052542.1 | CQ816253.1 | DL046109.1 | GM647353.1 | DD406007.1 | GM643601.1 |
| HI002032.1 | AX364634.1 | CS118961.1 | AX041936.1 | CQ814467.1 | DL046077.1 | GM647321.1 | DD402433.1 | GM643569.1 |
| HI001949.1 | AX364549.1 | CS118928.1 | AX037307.1 | CQ814078.1 | DL041963.1 | GM647289.1 | DD402401.1 | GM643537.1 |
| HI000281.1 | AX364517.1 | CS118862.1 | AX035988.1 | CQ814045.1 | DL041931.1 | GM647257.1 | DD402369.1 | GM643505.1 |
| HI000241.1 | AX364485.1 | CS118829.1 | AX028780.1 | CQ814013.1 | DL041899.1 | GM647225.1 | DD402337.1 | GM643473.1 |
| HI000197.1 | AX364453.1 | CS118796.1 | AX026037.1 | CQ813981.1 | DL038012.1 | GM640422.1 | DD402305.1 | GM643440.1 |
| HI000141.1 | AX364421.1 | CS118731.1 | AX023631.1 | CQ813949.1 | DL041357.1 | GM640390.1 | DD402273.1 | GM636451.1 |
| HI000109.1 | AX364357.1 | CS118699.1 | AX023597.1 | CQ813917.1 | DL041325.1 | GM640358.1 | DD402241.1 | GM636419.1 |
| HI471217.1 | AX364230.1 | CS118667.1 | AX023564.1 | AF411474.1 | DL041293.1 | DL196204.1 | DD402209.1 | GM636387.1 |
| HI201578.1 | AX364197.1 | CS118635.1 | AX019517.1 | AX207313.1 | DL037406.1 | DL193835.1 | DD405589.1 | GM636355.1 |
| HI180972.1 | AX361215.1 | CS118603.1 | AX019230.1 | AX207116.1 | DL037374.1 | DL183246.1 | DD405557.1 | GM631851.1 |
| HI552293.1 | AX358537.1 | CS118569.1 | AX011044.1 | AX203052.1 | DL037342.1 | FB513473.1 | DD405525.1 | GM631819.1 |
| HI539017.1 | AX358332.1 | CS118502.1 | AX010920.1 | AX202441.1 | DL024933.1 | FB513120.1 | CS114656.1 | GM631787.1 |
| HI470924.1 | AX357128.1 | CS118469.1 | AX009742.1 | AX201768.1 | DL024901.1 | FB571336.1 | CS106400.1 | GM622856.1 |
| HI470756.1 | BD008064.1 | CS118437.1 | AX008547.1 | AX193980.1 | DL021497.1 | FB571280.1 | CS105986.1 | GM622824.1 |
| HI551360.1 | BD006215.1 | CS118405.1 | AX004815.1 | AX182217.1 | DL021465.1 | FB570201.1 | CS102976.1 | FB507960.1 |
| HI538345.1 | E64037.1 | CS118372.1 | AX002979.1 | AX179670.1 | DL021433.1 | FB571847.1 | CS102912.1 | GM657909.1 |
| HI564718.1 | E54592.1 | CS118337.1 | U49847.1 | AX172419.1 | DL017195.1 | CS696204.1 | CS102752.1 | GM657877.1 |
| HI568863.1 | BD000466.1 | CS118304.1 | A41832.1 | AX167799.1 | DL017163.1 | CS696172.1 | CS102688.1 | GM657845.1 |
| HI568807.1 | E59807.1 | CS118271.1 | A25418.1 | AX164089.1 | DL017131.1 | CS696108.1 | AX395277.1 | GM657813.1 |
| HI559134.1 | AX349066.1 | CS118239.1 | A35720.1 | AX153815.1 | DL017099.1 | CS696076.1 | AX392798.1 | GM657780.1 |
| HI558658.1 | AX347269.1 | CS118206.1 | A32098.1 | AX145394.1 | DL017067.1 | CS696044.1 | AX384850.1 | GM657748.1 |
| HI564219.1 | AX347233.1 | CS118172.1 | A35506.1 | AX145714.1 | DL017035.1 | CS696011.1 | AX384578.1 | GM643389.1 |
| HI564103.1 | AX347199.1 | CS118140.1 | A34509.1 | AX145650.1 | DL012774.1 | CS695979.1 | AX382242.1 | GM643357.1 |
| HI563703.1 | AX347163.1 | CS118108.1 | A33246.1 | AX145618.1 | DL012742.1 | CS695947.1 | AX377966.1 | GM643325.1 |

| | | | | | | | | |
|---|---|---|---|---|---|---|---|---|
| HI558090.1 | AX346791.1 | CS118075.1 | A32656.1 | AX145586.1 | DL049434.1 | CS695883.1 | AX376955.1 | GM643261.1 |
| HI568110.1 | AX344907.1 | CS118042.1 | A32414.1 | AX145554.1 | DL049402.1 | CS695787.1 | AX370546.1 | GM643229.1 |
| HI563628.1 | AX339312.1 | CS118007.1 | A06245.1 | AX145522.1 | DL049370.1 | CS695755.1 | AX364679.1 | GM636240.1 |
| HI563595.1 | AX329475.1 | CS117973.1 | A31028.1 | AX145490.1 | DL049338.1 | CS695723.1 | AX364562.1 | GM636208.1 |
| HI563559.1 | AX328972.1 | CS114665.1 | HW256983.1 | DL019428.1 | DL049306.1 | CS695691.1 | AX364530.1 | GM636176.1 |
| HI508503.1 | AX328149.1 | CS114361.1 | HW256887.1 | DL030516.1 | DL049274.1 | CS695595.1 | AX364498.1 | GM636144.1 |
| HI575437.1 | AX306620.1 | CS113412.1 | HW256823.1 | CS613023.1 | DL049242.1 | CS695563.1 | AX364466.1 | GM636112.1 |
| HI544152.1 | AX268999.1 | CS113380.1 | HW256791.1 | CS612807.1 | DL049211.1 | CS695531.1 | AX364434.1 | GM636080.1 |
| HI582567.1 | AX268715.1 | CS113348.1 | HW256759.1 | CS612718.1 | DL049147.1 | CS695499.1 | AX364402.1 | GM631672.1 |
| DL047131.1 | AX259250.1 | CS113316.1 | HW256727.1 | CS604399.1 | DL049115.1 | CS695467.1 | AX364370.1 | GM631608.1 |
| DL047099.1 | AX300709.1 | CS112349.1 | HW251169.1 | CS608401.1 | DL049083.1 | CS695305.1 | AX364243.1 | GM631576.1 |
| DL047067.1 | AX286167.1 | CS111537.1 | HW251031.1 | DD401546.1 | DL049051.1 | CS791219.1 | AX364178.1 | GM631544.1 |
| DL043245.1 | HW326334.1 | AX923414.1 | HW250730.1 | DD401514.1 | DL012575.1 | DL176593.1 | AX359855.1 | GM631512.1 |
| DL043213.1 | HW338154.1 | AX923381.1 | HW240943.1 | DD401482.1 | DL012543.1 | DL176529.1 | AX358639.1 | GM622741.1 |
| DL043181.1 | HW337898.1 | AX840536.1 | HW240815.1 | DD401451.1 | DL012511.1 | DL176461.1 | E50933.1 | GM622709.1 |
| DL043149.1 | HW337386.1 | AX840014.1 | HW240631.1 | CS469249.1 | DL012479.1 | DL176300.1 | BD001836.1 | GM622677.1 |
| DL043117.1 | HW337130.1 | AX832719.1 | HW238940.1 | CS468138.1 | DL012447.1 | DL182009.1 | E54542.1 | GM704178.1 |
| DL043085.1 | HW337002.1 | AX826996.1 | HW238602.1 | CS464515.1 | DL012415.1 | DL181468.1 | E55069.1 | GM650375.1 |
| DL039268.1 | HW336718.1 | AX825991.1 | HW238230.1 | DD400140.1 | DL045425.1 | DL181377.1 | E64642.1 | GM628987.1 |
| DL039236.1 | HW336510.1 | AX824455.1 | HW237917.1 | CS459122.1 | DL045393.1 | DL174592.1 | E58876.1 | GM628955.1 |
| DL039204.1 | HW328834.1 | AX824345.1 | HW237873.1 | DD367717.1 | DL045361.1 | DL174374.1 | AX354623.1 | GM628923.1 |
| DL039172.1 | HW328544.1 | AX823779.1 | HW237699.1 | DD361271.1 | DL045329.1 | DL176789.1 | AX353936.1 | GM655229.1 |
| DL039140.1 | HW328422.1 | AX823535.1 | HW248649.1 | DD364146.1 | DL045297.1 | CS694392.1 | AX352731.1 | GM655197.1 |
| DL039108.1 | HW319193.1 | AX816448.1 | HW237438.1 | DD368133.1 | DL041249.1 | FB357048.1 | AX350854.1 | GM655165.1 |
| DL035484.1 | HW335785.1 | AX816110.1 | HW242297.1 | DD361305.1 | DL041217.1 | FB344636.1 | AX349189.1 | GM648172.1 |
| DL035452.1 | HW328262.1 | AX814831.1 | HW242265.1 | CS457892.1 | DL041185.1 | FB344287.1 | AX348906.1 | GM641209.1 |
| DL035420.1 | HW328135.1 | AX814415.1 | HW241554.1 | CS457340.1 | DL041153.1 | HD062023.1 | AX348467.1 | GM641145.1 |
| DL035388.1 | HW318562.1 | AX814381.1 | HV856239.1 | CS453768.1 | DL041121.1 | M15419.1 | AX347591.1 | GM641081.1 |
| DL035356.1 | HW314121.1 | AX811305.1 | HV926197.1 | DD092986.1 | DL041089.1 | M60606.1 | AX347322.1 | GM628889.1 |
| DL035324.1 | HW311231.1 | AX802756.1 | HV936725.1 | DD116979.1 | DL037202.1 | HC916999.1 | AX347284.1 | GM628857.1 |
| DL031320.1 | HW309923.1 | AX806011.1 | HV925507.1 | DD089285.1 | DL037170.1 | HC918581.1 | AX347246.1 | GM628825.1 |
| DL031288.1 | HW307885.1 | AX088751.1 | HV932703.1 | DD057923.1 | DL037138.1 | FW334415.1 | AX347214.1 | GM628793.1 |
| DL031256.1 | HW307853.1 | AX088695.1 | HV929976.1 | DD057891.1 | DL037106.1 | FW338097.1 | AX347176.1 | GM618948.1 |
| DL031224.1 | HW307821.1 | AX083535.1 | HV932356.1 | DD057859.1 | DL028735.1 | FW337544.1 | AX347028.1 | GM628681.1 |
| DL031160.1 | HW307789.1 | AX081667.1 | HV802955.1 | DD057239.1 | DL036578.1 | FW341716.1 | AX344530.1 | GM628649.1 |
| DL031128.1 | HW307751.1 | AX080955.1 | HV802923.1 | DD056922.1 | DL036546.1 | FW341645.1 | AX343862.1 | GM628617.1 |
| DL027319.1 | HW307719.1 | AX080783.1 | HV802891.1 | DD056007.1 | DL032311.1 | HC868224.1 | AX339352.1 | GM628585.1 |
| DL027287.1 | HW307687.1 | AX080468.1 | HV817776.1 | DD053287.1 | DL032279.1 | HC868085.1 | AX338529.1 | GM628553.1 |
| DL027255.1 | HW315865.1 | AX079170.1 | HV816931.1 | DD047304.1 | DL032247.1 | HC047048.1 | AX328280.1 | GM628521.1 |
| DL027223.1 | HW315090.1 | AX073926.1 | HV780163.1 | DD046351.1 | DL032215.1 | HC047016.1 | AX327950.1 | GM716102.1 |
| DL027191.1 | HV802896.1 | AJ286128.1 | HV779814.1 | DD023572.1 | DL032183.1 | HC046984.1 | AX320708.1 | GM654971.1 |
| DL027159.1 | HV817775.1 | AX068361.1 | AY658616.1 | DD028802.1 | DL032151.1 | HC046952.1 | AX319583.1 | GM641014.1 |
| DL023347.1 | HV778939.1 | AX063430.1 | AY658584.1 | DD017633.1 | DL036491.1 | HC046920.1 | AX317312.1 | GM640982.1 |
| DL023315.1 | HV777980.1 | AX060078.1 | FW344918.1 | BD495459.1 | DL048421.1 | HC040011.1 | AX306484.1 | GM640950.1 |
| DL023283.1 | HV763969.1 | AX058562.1 | FW343698.1 | BD434178.1 | DL048389.1 | HC046860.1 | AX277032.1 | GM640918.1 |
| DL023251.1 | HV774096.1 | AX058374.1 | HC923239.1 | DD227399.1 | DL048357.1 | HC046828.1 | AX266974.1 | GM640886.1 |
| DL020172.1 | HV767476.1 | AX057301.1 | HC922882.1 | DD227170.1 | DL048325.1 | HC046796.1 | HW315167.1 | GM640854.1 |

| | | | | | | | | |
|---|---|---|---|---|---|---|---|---|
| DL020140.1 | HV766833.1 | AX056823.1 | HC921427.1 | DD226384.1 | DL044439.1 | HC046764.1 | HW315135.1 | GM628500.1 |
| DL020108.1 | HV760566.1 | AX055833.1 | HC920496.1 | DD224937.1 | DL044407.1 | HC046732.1 | HW315071.1 | DL086832.1 |
| DL020076.1 | HV766324.1 | AX052960.1 | HD067660.1 | DD224389.1 | DL044375.1 | HC046700.1 | HW314831.1 | DL086800.1 |
| DL020044.1 | HV508575.1 | AX052921.1 | HD064807.1 | DD231455.1 | DL044343.1 | HC046683.1 | HW314777.1 | DL086768.1 |
| DL020012.1 | HV508511.1 | AX050244.1 | HD048986.1 | AX960210.1 | DL044311.1 | HC046651.1 | HW314725.1 | DL086736.1 |
| DL015449.1 | HV508483.1 | AX047489.1 | HD057699.1 | DD214001.1 | DL044279.1 | HC046587.1 | HW106130.1 | DL086704.1 |
| DL010991.1 | HV512285.1 | AX046155.1 | HD057667.1 | DD213984.1 | DL032110.1 | HC046555.1 | HW112769.1 | DL086672.1 |
| DL010959.1 | HV512253.1 | AX044422.1 | HD057653.1 | DD213600.1 | DL032078.1 | HC046523.1 | HW112666.1 | DL086640.1 |
| DL010927.1 | HV512221.1 | AX040863.1 | M27213.1 | DD213568.1 | DL032014.1 | HC046491.1 | HW112610.1 | DL086608.1 |
| DL010895.1 | HV512157.1 | AX039584.1 | U69259.1 | CS254930.1 | DL031950.1 | HC046459.1 | HW071379.1 | DL105881.1 |
| DL010863.1 | HV504788.1 | AX039153.1 | FW342478.1 | DD211573.1 | DL028141.1 | HC046427.1 | HW070015.1 | DL105849.1 |
| DL010831.1 | HV504756.1 | AX037085.1 | FW341731.1 | DD210739.1 | DL028109.1 | HC046395.1 | HW069951.1 | DL105817.1 |
| DL023152.1 | HV504724.1 | AX036616.1 | FW340695.1 | DD206903.1 | DL028077.1 | HC046363.1 | HW069787.1 | DL105721.1 |
| DL023120.1 | HV504692.1 | AX036031.1 | HC874690.1 | DD206871.1 | DL028045.1 | HC046331.1 | HW084943.1 | DL101012.1 |
| DL023088.1 | HV504660.1 | AX035979.1 | HC880624.1 | DD206839.1 | DL028013.1 | HC046048.1 | HW084902.1 | DL100980.1 |
| DL023056.1 | HV504628.1 | AX034869.1 | DM380122.1 | DD206809.1 | DL027981.1 | HC046016.1 | HW097737.1 | DL100948.1 |
| DL022992.1 | HV504596.1 | AX033317.1 | HC021029.1 | DD206777.1 | DL027949.1 | HC046288.1 | HW067061.1 | DL096534.1 |
| DL019977.1 | HV504979.1 | AX029308.1 | HC025932.1 | DD196867.1 | DL036459.1 | HC046256.1 | HW058316.1 | DL096502.1 |
| DL019945.1 | HV455605.1 | AX028764.1 | HC025514.1 | CQ814076.1 | HV533081.1 | HC046224.1 | HW047618.1 | DL096470.1 |
| DL019913.1 | HV503833.1 | AX027724.1 | HC025482.1 | CQ814043.1 | HV536540.1 | HC046160.1 | HV963993.1 | DL094514.1 |
| DL019881.1 | HV502846.1 | AX024787.1 | HC025450.1 | CQ814011.1 | HV534659.1 | HC046128.1 | HV956403.1 | DL094482.1 |
| DL019849.1 | HV502802.1 | AX023655.1 | HC025418.1 | CQ813947.1 | HV532477.1 | HC046096.1 | HV951112.1 | DL110715.1 |
| DL019817.1 | HV502770.1 | AX023589.1 | HC010653.1 | CQ813915.1 | HV532382.1 | HC045978.1 | HV950456.1 | DL090745.1 |
| DL015414.1 | DL110122.1 | AX023334.1 | HC008573.1 | CQ813883.1 | HV537227.1 | HC045946.1 | HV944625.1 | DL090713.1 |
| DL015382.1 | DL110090.1 | AX020389.1 | HC007628.1 | CQ813851.1 | HV531942.1 | HC045914.1 | HV780471.1 | DL086580.1 |
| DL015318.1 | DL092248.1 | AX002735.1 | HC011019.1 | CQ813819.1 | HV528087.1 | HC045882.1 | HV779880.1 | DL086548.1 |
| DL015286.1 | DL092216.1 | AX011481.1 | DM377196.1 | CQ813786.1 | HV515587.1 | HC045850.1 | HV774720.1 | DL086484.1 |
| DL015254.1 | DL092184.1 | AX011449.1 | DM370672.1 | CQ813754.1 | HV515555.1 | HC045818.1 | HV773783.1 | DL086452.1 |
| DL010796.1 | DL029124.1 | AX011417.1 | DM370640.1 | CQ802186.1 | HV515523.1 | HC045786.1 | HW302690.1 | DL086420.1 |
| DL010764.1 | DL021778.1 | AX011384.1 | DM370608.1 | CQ800899.1 | FW503672.1 | HC045754.1 | HW286515.1 | DL115522.1 |
| DL010732.1 | DL021746.1 | AX011352.1 | DM370576.1 | CQ800645.1 | FW497150.1 | HC045722.1 | HW295878.1 | DL115490.1 |
| DL010700.1 | DL021714.1 | AX011248.1 | DM370544.1 | CQ795468.1 | FW420444.1 | HC045692.1 | HW302537.1 | DL115458.1 |
| DL010668.1 | DL031199.1 | AX010956.1 | DM370512.1 | CQ788569.1 | FW420412.1 | HC045660.1 | HW302417.1 | DL117535.1 |
| DL010636.1 | DJ055300.1 | AX010733.1 | DM370480.1 | CQ787431.1 | FW501793.1 | HC045628.1 | HW285987.1 | DL117503.1 |
| DD450236.1 | DJ055180.1 | AX010626.1 | DM371277.1 | CQ787287.1 | FW501746.1 | HC045596.1 | HV949949.1 | DL117471.1 |
| DD450163.1 | DJ054355.1 | HW291172.1 | GN032759.1 | CQ786995.1 | FW496942.1 | HC045564.1 | HV947321.1 | DL117439.1 |
| DD450129.1 | DJ056266.1 | HW291075.1 | GN032728.1 | CQ786566.1 | FW420387.1 | HC045532.1 | HV947221.1 | DL112760.1 |
| DD438047.1 | DJ055998.1 | HW291043.1 | GN032664.1 | CQ784689.1 | FW420355.1 | HC035628.1 | HV953034.1 | DL112696.1 |
| DD438007.1 | FB291800.1 | HW291011.1 | GN032631.1 | CQ784623.1 | FW420323.1 | HC037061.1 | HV939915.1 | DL112664.1 |
| DD449098.1 | CS810619.1 | HW290979.1 | GN032599.1 | CQ778517.1 | FW496742.1 | AB526354.1 | HV816111.1 | DL107731.1 |
| DD448962.1 | FB292587.1 | HW290947.1 | GN032567.1 | CQ778395.1 | HI653964.1 | HC021025.1 | DL120249.1 | DL107699.1 |
| DD441523.1 | FB292328.1 | HW290915.1 | GN032536.1 | CQ774304.1 | HI653879.1 | HC025510.1 | DL120217.1 | DL103095.1 |
| DD435320.1 | CS803350.1 | HW290884.1 | GN032472.1 | CQ771671.1 | HI653808.1 | HC025478.1 | DL115814.1 | DL103063.1 |
| CS619954.1 | CS802270.1 | HW290852.1 | GN032440.1 | CQ761095.1 | HI653718.1 | HC025446.1 | DL115782.1 | DL103031.1 |
| CS622385.1 | DJ050498.1 | HW290131.1 | GN032408.1 | CQ759684.1 | HI653636.1 | HC025414.1 | DL115750.1 | DL098322.1 |
| CS617093.1 | DJ048820.1 | HW298236.1 | GN032376.1 | CQ758861.1 | HI653602.1 | HC010649.1 | DL115718.1 | DL098290.1 |
| CS616560.1 | CS793783.1 | HW285133.1 | GN032343.1 | CQ755356.1 | HI653532.1 | HC010188.1 | DL115686.1 | DL098258.1 |

| | | | | | | | | |
|---|---|---|---|---|---|---|---|---|
| CS613801.1 | CS716740.1 | HW268481.1 | GN032311.1 | AX060344.1 | HI653419.1 | HC010109.1 | DL115654.1 | DL098226.1 |
| CS612975.1 | CS796911.1 | HW267851.1 | GN032279.1 | AX057311.1 | HI653364.1 | HC008501.1 | DL111196.1 | DL098194.1 |
| CS612712.1 | CS714145.1 | HW267802.1 | GN032247.1 | AX052985.1 | HI653283.1 | HC007836.1 | DL111164.1 | DL098162.1 |
| CS604521.1 | CS675428.1 | HW266124.1 | GN032215.1 | AX052899.1 | HI653225.1 | HC007812.1 | DL106093.1 | DL092152.1 |
| CS604489.1 | CS680675.1 | HW264478.1 | GN032183.1 | AX041940.1 | HI652911.1 | HC007748.1 | DL106061.1 | DL092120.1 |
| CS604457.1 | CS790646.1 | HW263070.1 | GN032119.1 | AX039934.1 | HI651049.1 | HC007716.1 | DL106029.1 | DL092088.1 |
| CS604425.1 | CS353168.1 | HW263033.1 | GN032087.1 | AX037309.1 | HI650591.1 | HC007684.1 | DL105997.1 | DL092056.1 |
| CS604361.1 | CS352384.1 | HW263001.1 | GN032055.1 | AX035998.1 | HI648679.1 | HC007652.1 | DL105965.1 | DL092024.1 |
| CS604329.1 | CS283950.1 | HW262584.1 | GN032023.1 | AX034880.1 | HI646712.1 | HC007592.1 | DL101192.1 | DL091992.1 |
| CS604297.1 | CS283072.1 | HW261412.1 | GN031991.1 | AX030369.1 | AF044078.1 | HC007560.1 | DL101160.1 | DL098734.1 |
| CS604137.1 | CS276847.1 | HW261380.1 | GN031927.1 | CS070144.1 | HH807077.1 | HC007528.1 | DL101128.1 | FB299281.1 |
| CS604105.1 | CS272579.1 | HW261348.1 | GN031895.1 | CS067352.1 | HH796599.1 | HC010767.1 | DL096714.1 | CS368402.1 |
| CS604009.1 | CS265593.1 | HW261316.1 | GN031863.1 | CS063866.1 | HH794988.1 | DM377064.1 | DL096682.1 | CS368082.1 |
| CS603785.1 | CS254297.1 | HW261284.1 | GN031831.1 | AY967395.1 | HH794723.1 | DM375508.1 | DL096650.1 | CS368018.1 |
| CS603593.1 | CS244259.1 | HW261252.1 | GN031669.1 | AY967363.1 | HH779681.1 | HC005761.1 | DL096618.1 | CS367570.1 |
| AX796759.1 | CS244227.1 | HW261220.1 | GN031637.1 | AY967331.1 | HH777924.1 | HC005250.1 | DL096586.1 | DL076098.1 |
| AX795864.1 | CS244195.1 | HW261188.1 | GN031605.1 | AY967299.1 | HH774439.1 | DM371002.1 | DL096554.1 | DL080447.1 |
| AX795564.1 | CS244697.1 | HW261124.1 | GN031573.1 | AY967267.1 | FW393022.1 | DM370970.1 | DL094726.1 | DL075902.1 |
| AX795446.1 | CS243146.1 | HW261060.1 | GN031509.1 | AY967235.1 | FW395235.1 | DM370938.1 | DL094694.1 | DL075843.1 |
| BD194454.1 | CS239839.1 | HW261028.1 | GN031477.1 | AY967203.1 | FW395608.1 | DM370801.1 | DL094662.1 | DL075811.1 |
| BD190491.1 | CS237266.1 | HW260996.1 | GN031412.1 | AY967171.1 | HH935703.1 | DM069111.1 | DL122691.1 | DL075766.1 |
| AX785121.1 | CS228705.1 | HW260964.1 | GN031348.1 | AY967139.1 | HH934828.1 | DM076040.1 | DL122659.1 | DL079052.1 |
| AX777597.1 | CS227305.1 | HW260932.1 | GN031316.1 | AY967107.1 | HH934451.1 | DM068819.1 | DL118405.1 | DL078968.1 |
| AX774567.1 | CS207863.1 | HW260900.1 | GN031283.1 | AY967075.1 | HH833947.1 | GN087808.1 | DL118373.1 | DL078936.1 |
| AX773256.1 | CS194010.1 | HW260868.1 | GN031251.1 | AY967043.1 | HH833645.1 | GN087447.1 | DL118341.1 | DL073471.1 |
| AX771051.1 | CS254931.1 | HW260836.1 | GN031219.1 | AY967011.1 | HH833613.1 | GN087207.1 | DL108856.1 | DL072285.1 |
| AX770156.1 | DD212006.1 | HW260804.1 | GN031187.1 | AY966979.1 | HH833581.1 | GN089872.1 | DL108792.1 | DL072228.1 |
| AX769458.1 | DD211578.1 | HW260772.1 | GN031154.1 | AY966947.1 | HH833549.1 | GN089808.1 | DL108760.1 | DL013610.1 |
| AX767522.1 | DD182400.1 | HW260740.1 | GN031122.1 | CS055057.1 | HH833517.1 | GN082899.1 | DL108728.1 | DL013578.1 |
| AX766890.1 | DD210740.1 | HW260708.1 | GN031090.1 | CS052517.1 | HH833485.1 | GN089214.1 | DL108696.1 | DL013546.1 |
| AX766740.1 | DD206904.1 | HW260676.1 | GN031058.1 | AX167421.1 | HH833453.1 | GN082828.1 | DL099516.1 | DL013514.1 |
| AX765969.1 | DD206872.1 | HW260644.1 | GN030994.1 | AX145709.1 | HH833421.1 | GN093111.1 | DL099484.1 | DL013482.1 |
| AX764649.1 | DD206840.1 | HW260612.1 | GN030963.1 | AX145677.1 | HH833389.1 | CS604863.1 | DL099452.1 | DL013450.1 |
| AX764617.1 | DD206810.1 | HW260580.1 | GN030931.1 | AX145645.1 | FW392848.1 | CS604831.1 | DL099420.1 | DL013418.1 |
| AX764585.1 | DD206778.1 | HW260548.1 | GN030899.1 | AX145613.1 | HH928772.1 | CS604799.1 | DL089539.1 | DL013386.1 |
| AX764553.1 | DD196868.1 | HW260516.1 | GN030867.1 | AX145581.1 | HH931892.1 | CS604767.1 | DL089507.1 | DL018109.1 |
| AX756517.1 | CS236432.1 | HW260484.1 | GN030835.1 | AX145549.1 | HH821172.1 | CS604735.1 | DL089475.1 | DL018077.1 |
| BD183725.1 | DD181003.1 | HC306014.1 | GN030803.1 | AX145517.1 | HH821866.1 | CS604703.1 | DL089443.1 | DL018045.1 |
| BD183122.1 | CS038903.1 | HC305974.1 | GN030771.1 | AX145485.1 | HH827002.1 | CS604671.1 | DL113545.1 | DL036298.1 |
| BD181840.1 | CS029246.1 | HC302622.1 | DM005046.1 | AX145453.1 | HH826833.1 | CS604637.1 | DL113513.1 | DL044245.1 |
| BD181068.1 | CS024022.1 | HC305619.1 | DM004976.1 | AX145421.1 | HH826498.1 | CS604605.1 | DL093138.1 | DL044213.1 |
| BD180840.1 | CS018386.1 | HC301311.1 | DM003477.1 | AX145388.1 | HH826427.1 | CS604573.1 | DL093106.1 | DL044181.1 |
| AX744007.1 | CS016565.1 | HC301020.1 | CS510546.1 | AX145356.1 | HH822353.1 | CS597517.1 | DL093074.1 | DL044149.1 |
| AX743518.1 | CS016527.1 | HC291438.1 | CS501970.1 | AX145324.1 | HH822041.1 | CS593088.1 | DL122556.1 | DL044117.1 |
| AX742859.1 | CQ986613.1 | HC294122.1 | CS495827.1 | AX145292.1 | HH821911.1 | CS592811.1 | DL122524.1 | DL044085.1 |
| AX741270.1 | CQ986549.1 | HC289510.1 | DD418591.1 | AX145260.1 | FW379648.1 | CS598819.1 | DL029529.1 | DL048222.1 |
| AX739988.1 | CQ985696.1 | HC289351.1 | CS489537.1 | AX145228.1 | FW379616.1 | CS589274.1 | DL029497.1 | DL048190.1 |

| | | | | | | | | |
|---|---|---|---|---|---|---|---|---|
| AX721702.1 | CQ981103.1 | HC289319.1 | CS486987.1 | AX145196.1 | FW379574.1 | CS597735.1 | DL029465.1 | DL048158.1 |
| BD178187.1 | AX587727.1 | HC289119.1 | CS485304.1 | AX145164.1 | FW379273.1 | CS585652.1 | DL046099.1 | DL048126.1 |
| BD177392.1 | AX587462.1 | HC288873.1 | HV344270.1 | AX145132.1 | FW378151.1 | CS585014.1 | DL041953.1 | DL048094.1 |
| BD177104.1 | AX576405.1 | HC295588.1 | HV344232.1 | AX145100.1 | FW377097.1 | CS588490.1 | DL041921.1 | DL048062.1 |
| AX720712.1 | AX573315.1 | HC295446.1 | HV344194.1 | AX145068.1 | HC310100.1 | CS575466.1 | DL041889.1 | DL048031.1 |
| AX718409.1 | AX556840.1 | HC288604.1 | HV341876.1 | AX145036.1 | HC310068.1 | CS574844.1 | DL045872.1 | DL047999.1 |
| AX709558.1 | AX556793.1 | HC295157.1 | HV344557.1 | AX145004.1 | HC310036.1 | CS574794.1 | DL049706.1 | DL047967.1 |
| AX699438.1 | AX555165.1 | HC292014.1 | HV335369.1 | AX144972.1 | HC310004.1 | CS573130.1 | DL049674.1 | AX143711.1 |
| AX698952.1 | AX543908.1 | HC291846.1 | HV322467.1 | AX144940.1 | HC309972.1 | CS572373.1 | DL045666.1 | AX143583.1 |
| AX697544.1 | AX537305.1 | HC299193.1 | HV321877.1 | AX144907.1 | HC309745.1 | CS546788.1 | DL041691.1 | AX143455.1 |
| AX696341.1 | AX528834.1 | HC294732.1 | HV334674.1 | AX144875.1 | HC308770.1 | CS570228.1 | DL049633.1 | AX143327.1 |
| AX685193.1 | AX525276.1 | HC293786.1 | HV333167.1 | AX144843.1 | HC307866.1 | CS545477.1 | DL049601.1 | AX143263.1 |
| AX684116.1 | AX523795.1 | HC293606.1 | HV332179.1 | AX144811.1 | HC307372.1 | CS561184.1 | DL041554.1 | AX143135.1 |
| AX683014.1 | AX179516.1 | HC290172.1 | HV325416.1 | AX144779.1 | HC312262.1 | CS560462.1 | DL041522.1 | AX143071.1 |
| AX674837.1 | AX179456.1 | HC297101.1 | HV331842.1 | AX144747.1 | FU258273.1 | DD420073.1 | DL033304.1 | AX143007.1 |
| K02390.1 | AX164065.1 | HC289881.1 | HV324787.1 | AX144715.1 | FU258241.1 | DD434536.1 | DL033272.1 | AX142879.1 |
| M31705.1 | AX028007.1 | HC289847.1 | HV324313.1 | AX144683.1 | FU258145.1 | DD431724.1 | DL033208.1 | AX142751.1 |
| K02998.1 | A20358.1 | HC207911.1 | HV323891.1 | AX144651.1 | FU257933.1 | CS541722.1 | DL033176.1 | AX142687.1 |
| M19991.1 | AF430208.1 | HB977961.1 | HV313316.1 | AX144619.1 | FU257901.1 | CS541769.1 | DL033144.1 | AX142623.1 |
| M31026.1 | AF430176.1 | HB977445.1 | HV111877.1 | AX144497.1 | FU257837.1 | CS542561.1 | DL029136.1 | AX142559.1 |
| M33417.1 | AX512473.1 | HB976605.1 | HV041736.1 | AX144433.1 | FU257740.1 | CS538094.1 | DL029104.1 | AX142365.1 |
| M55326.1 | AX496843.1 | HB976180.1 | HV120192.1 | AX144239.1 | FU260836.1 | CS537181.1 | DL029072.1 | AX142301.1 |
| OM981241.1 | BD140255.1 | DM193385.1 | HV187352.1 | AX144175.1 | FU262645.1 | CS510551.1 | DL035407.1 | AX142045.1 |
| MK327980.1 | BD137859.1 | DM193338.1 | HV189792.1 | AX144111.1 | FU267896.1 | CS502910.1 | DL035375.1 | AX141915.1 |
| U21238.1 | BD135985.1 | DM189855.1 | HV189735.1 | AX144047.1 | HW058497.1 | CS502712.1 | DL035343.1 | AX141785.1 |
| GN041322.1 | BD135089.1 | DM189264.1 | HV218626.1 | AX143981.1 | HW064881.1 | CS502644.1 | DL031179.1 | AX141657.1 |
| AH002293.2 | BD131702.1 | HB816499.1 | HV218467.1 | AX143853.1 | HW064841.1 | CS502406.1 | DL031147.1 | AX141529.1 |
| K02540.1 | BD130817.1 | HB805650.1 | HV221705.1 | AX143725.1 | HW064809.1 | CS243275.1 | DL027242.1 | AX141401.1 |
| M35114.1 | BD130785.1 | HB848184.1 | HV245435.1 | AX143149.1 | HW057648.1 | CS235773.1 | DL027210.1 | AX140364.1 |
| AY599500.1 | HI001417.1 | HB839353.1 | HV214871.1 | AX142957.1 | HW056501.1 | CS228475.1 | DL027178.1 | AX135821.1 |
| AY331974.1 | HI001365.1 | HB847376.1 | HV233845.1 | AX142829.1 | HW056401.1 | CS231719.1 | DL023366.1 | AX133514.1 |
| AF003708.1 | HI001331.1 | HB832800.1 | HV227053.1 | AX142573.1 | HW064663.1 | CS200912.1 | DL023334.1 | AX127630.1 |
| S72666.1 | HI003167.1 | HB846995.1 | HV236035.1 | AX142507.1 | HW064605.1 | CS227257.1 | DL023302.1 | AX119976.1 |
| KJ668651.1 | HI003101.1 | HB838583.1 | HV247240.1 | AX142443.1 | HW062558.1 | CS226729.1 | DL023270.1 | AX114524.1 |
| HW408835.1 | HI003056.1 | HB840281.1 | HV247208.1 | AX142315.1 | HW062408.1 | CS208832.1 | DL023238.1 | AX113816.1 |
| HW408739.1 | HI003016.1 | HB846124.1 | HV247176.1 | AX142187.1 | HW060577.1 | CS208782.1 | DL023206.1 | AX113554.1 |
| HW408707.1 | HC291690.1 | HB838112.1 | HV247144.1 | AX141929.1 | HW060526.1 | CS203530.1 | DL023171.1 | AX111975.1 |
| HW408616.1 | HC294463.1 | HB837764.1 | HV301853.1 | HW043303.1 | HW062383.1 | CS186196.1 | DL023139.1 | AX108618.1 |
| HW408584.1 | HC293894.1 | HB845642.1 | HV301758.1 | HW041807.1 | HW062319.1 | CS182271.1 | DL023107.1 | AX108288.1 |
| HW408552.1 | HC293714.1 | HB845409.1 | HV301588.1 | HW041775.1 | HW062287.1 | CS183875.1 | DL023075.1 | HW151143.1 |
| HW408520.1 | HC290761.1 | HB837185.1 | HV301556.1 | HW041554.1 | HF586457.1 | CS178999.1 | DL023043.1 | HW113221.1 |
| HW408488.1 | HC293212.1 | HB844952.1 | HV306191.1 | HW044193.1 | HW042074.1 | CS174580.1 | DL023011.1 | HW112618.1 |
| HW408457.1 | HC289966.1 | HB844500.1 | HV306138.1 | HV986646.1 | HW042042.1 | CS174660.1 | DL010751.1 | HW072120.1 |
| HW408425.1 | HC293020.1 | HB844275.1 | HV304360.1 | HV960811.1 | HW043572.1 | CS203501.1 | DL010719.1 | HW070117.1 |
| HW408393.1 | HC289856.1 | HB836571.1 | HV302953.1 | HV960579.1 | HW041998.1 | CS172389.1 | DL010687.1 | HW069957.1 |
| HW408361.1 | HC201531.1 | HB843807.1 | HV312757.1 | HV958292.1 | HW041966.1 | CS159804.1 | DJ339593.1 | HW069893.1 |
| HW399704.1 | HC198626.1 | HB836318.1 | HV308920.1 | HV963324.1 | HW041934.1 | CS159578.1 | HV743220.1 | HW069793.1 |

| | | | | | | | | |
|---|---|---|---|---|---|---|---|---|
| HW399258.1 | HC195244.1 | HB835832.1 | HV302696.1 | HV956090.1 | HW041836.1 | CS159201.1 | HV704030.1 | HW069761.1 |
| HW392230.1 | HC194969.1 | HB843136.1 | HV311112.1 | DQ377844.1 | HW049388.1 | CS155788.1 | HV744572.1 | HW084949.1 |
| HW391547.1 | HC190443.1 | HB835667.1 | HV312317.1 | AY659383.1 | HW049357.1 | CS145021.1 | HV703162.1 | HW084910.1 |
| HW408899.1 | HC089564.1 | HB819133.1 | HV310966.1 | AY659351.1 | HW049325.1 | CS144355.1 | HV702430.1 | HW097743.1 |
| HW408867.1 | HC089532.1 | HB829373.1 | HV313449.1 | AY659319.1 | HW049293.1 | CS141582.1 | HV701206.1 | HW065058.1 |
| HW390486.1 | HC089500.1 | HB842616.1 | HV318331.1 | AY659287.1 | HW049261.1 | CS141530.1 | HV701174.1 | HW056086.1 |
| HW390445.1 | HC089468.1 | HB842034.1 | HV317252.1 | AY659255.1 | HW041800.1 | CS141498.1 | HV701110.1 | HW047321.1 |
| HW390297.1 | HC089436.1 | HB840911.1 | HV226869.1 | AY659223.1 | HW041768.1 | CS141138.1 | HV504764.1 | HV969611.1 |
| HW389598.1 | HC089403.1 | HB840366.1 | HV217831.1 | AY659191.1 | HW054085.1 | CS140716.1 | HV504732.1 | HV956155.1 |
| HW387482.1 | HC089371.1 | HB649983.1 | HV247109.1 | AY659159.1 | HW054007.1 | CS136254.1 | HV504700.1 | HV951082.1 |
| HW386599.1 | HC089339.1 | HB648973.1 | HV235906.1 | AY659127.1 | HW044179.1 | CS132044.1 | HV504668.1 | HV956420.1 |
| HW386535.1 | HC089306.1 | HB645748.1 | HV316813.1 | AY659095.1 | HV985995.1 | CS131836.1 | HV504636.1 | HV944653.1 |
| HW386503.1 | HC089274.1 | HB645716.1 | HI463025.1 | AY659063.1 | HV985972.1 | CS130981.1 | HV504604.1 | HV941991.1 |
| HW386247.1 | HC086303.1 | HB645684.1 | HI462577.1 | AY659031.1 | HV985940.1 | CS129688.1 | HV504987.1 | HV779886.1 |
| HW386215.1 | HC085251.1 | HB645607.1 | HH724934.1 | AY658999.1 | HV507702.1 | CS123347.1 | HV504229.1 | HV778426.1 |
| HW386183.1 | AY774927.1 | HB850790.1 | HH716029.1 | AY658967.1 | HV453663.1 | CS122557.1 | HV504197.1 | HV773799.1 |
| HW386119.1 | AY774876.1 | GN031984.1 | HH715950.1 | DM044706.1 | HV449913.1 | CS122253.1 | HV503841.1 | HW287333.1 |
| HW258914.1 | AY774815.1 | GN031952.1 | HH713836.1 | DM044575.1 | HV445632.1 | CS106181.1 | HV491264.1 | HW286521.1 |
| HW258847.1 | AY774759.1 | GN031920.1 | HH713451.1 | GN067950.1 | HV445494.1 | CS106085.1 | HV503801.1 | HW286488.1 |
| HW257427.1 | AY774701.1 | GN031888.1 | HH961361.1 | GN067901.1 | HV448965.1 | CS181485.1 | HV503769.1 | HW302365.1 |
| HW257395.1 | AY774480.1 | GN031856.1 | HH961329.1 | GN067837.1 | HV448482.1 | CS179755.1 | HV503705.1 | HW295615.1 |
| HV346973.1 | AY774254.1 | GN031791.1 | HH961297.1 | GN067681.1 | HV444629.1 | CS179620.1 | HV503673.1 | HW295584.1 |
| HV344650.1 | AY774072.1 | GN031726.1 | HH961265.1 | FB360901.1 | HV444468.1 | CS179184.1 | HV455295.1 | HW307493.1 |
| HV339937.1 | AY258148.1 | GN031630.1 | HH961233.1 | FB360087.1 | HV444187.1 | CS326352.1 | HV503174.1 | HW307045.1 |
| HV339905.1 | AF267909.1 | GN031566.1 | HH975399.1 | DL114713.1 | HV444110.1 | CS323679.1 | HV503142.1 | HW294148.1 |
| HV344536.1 | HC083742.1 | GN031534.1 | HH975096.1 | DL127180.1 | HV444002.1 | CQ892567.1 | HV503110.1 | HW294115.1 |
| HD048922.1 | HC083710.1 | GN031470.1 | HH964375.1 | DL069498.1 | HV438466.1 | CQ890274.1 | HV503078.1 | HW294083.1 |
| HD052440.1 | HC083533.1 | GN031437.1 | HH982478.1 | DJ493844.1 | HV437636.1 | CQ889053.1 | HV502778.1 | HW294049.1 |
| HD057696.1 | GM709025.1 | GN031405.1 | HH974556.1 | DJ446835.1 | HV443088.1 | CQ888175.1 | HV502714.1 | HW293166.1 |
| HD057664.1 | GM708993.1 | GN031373.1 | HH974393.1 | DJ446693.1 | HV437546.1 | CQ881960.1 | HV502682.1 | HW293033.1 |
| HD062729.1 | GM655022.1 | GN031341.1 | HH998093.1 | DJ436675.1 | HV351586.1 | CQ877817.1 | HV502618.1 | HW292756.1 |
| HD057650.1 | GM833983.1 | GN031309.1 | HH998030.1 | DJ436421.1 | HV334775.1 | CQ877235.1 | HV502586.1 | HW291214.1 |
| M10170.1 | GM712985.1 | GN031276.1 | HH997986.1 | DJ434484.1 | HV344042.1 | CQ876142.1 | HV502554.1 | HW072730.1 |
| M35049.1 | GM638490.1 | GN031244.1 | HH999669.1 | DJ438370.1 | HV343814.1 | CQ875540.1 | HV502522.1 | HW086492.1 |
| M62900.1 | GM642102.1 | GN031180.1 | HH999515.1 | DJ433797.1 | HV343622.1 | CQ874721.1 | HV502490.1 | HW071971.1 |
| U53587.1 | GM697136.1 | GN031147.1 | HH997852.1 | DJ438297.1 | HV343478.1 | CQ873293.1 | HV492743.1 | HW104455.1 |
| HC918582.1 | GM634542.1 | GN031115.1 | HH997789.1 | DJ427993.1 | HV340902.1 | CQ871226.1 | HV505297.1 | HW104343.1 |
| FW334416.1 | GM634510.1 | GN031083.1 | HH997750.1 | DJ430190.1 | HV338395.1 | CQ868815.1 | HV492444.1 | HW104309.1 |
| FW340293.1 | GM634478.1 | GN031051.1 | HH999472.1 | CS479603.1 | HV347861.1 | DL018075.1 | HV453588.1 | HW104277.1 |
| FW342475.1 | GM664826.1 | GN030987.1 | HH999427.1 | CS469235.1 | HV347603.1 | DL018043.1 | HV455246.1 | HW104195.1 |
| FW337615.1 | GM655878.1 | GN030956.1 | HH999379.1 | CS468158.1 | HV344986.1 | DL018011.1 | HH794943.1 | HW104115.1 |
| FW341717.1 | DL088133.1 | GN030924.1 | HH997667.1 | DD400168.1 | HV344806.1 | DL013260.1 | HH794711.1 | HW070676.1 |
| FW341649.1 | DL128835.1 | GN030892.1 | HH997629.1 | DD400136.1 | HV344722.1 | DL013228.1 | HH793153.1 | HW099613.1 |
| FW341214.1 | DL128738.1 | GM627523.1 | HH997572.1 | CS459118.1 | HI401093.1 | DL013196.1 | HH787614.1 | HW099549.1 |
| FW336093.1 | DL120035.1 | GM661496.1 | HH997497.1 | DD361267.1 | HD117976.1 | DL017985.1 | HH779669.1 | HW069670.1 |
| FW335846.1 | DL115119.1 | GM661464.1 | HH997402.1 | DD367407.1 | HI642708.1 | DL017953.1 | HH774427.1 | HW069241.1 |
| HC868033.1 | DL100373.1 | GM661432.1 | HH997358.1 | DD362926.1 | HI642164.1 | DL017921.1 | HH820947.1 | HW089222.1 |

| | | | | | | | | |
|---|---|---|---|---|---|---|---|---|
| HC887354.1 | DL098827.1 | GM661400.1 | HH999290.1 | DD362510.1 | HI641620.1 | DL017889.1 | HI401430.1 | HW067531.1 |
| HC874653.1 | CS368162.1 | GM661368.1 | HH999223.1 | DD368129.1 | HI641076.1 | DL017857.1 | HI642997.1 | HW089008.1 |
| HC867478.1 | CS368034.1 | GM661336.1 | HH999176.1 | E05859.1 | HI637705.1 | DL017825.1 | HI642453.1 | HW083502.1 |
| HC867390.1 | CS367714.1 | GM654310.1 | HH999134.1 | E04534.1 | HI380409.1 | DL017794.1 | HI002057.1 | HW088825.1 |
| HC872744.1 | DL075910.1 | GM647189.1 | HH999095.1 | E03798.1 | HI380330.1 | DL017698.1 | HI001970.1 | HW083202.1 |
| HC872700.1 | DL073709.1 | GM647157.1 | HH999054.1 | E03313.1 | HI379455.1 | DL017666.1 | HI001895.1 | HW082731.1 |
| HC880621.1 | DL046824.1 | GM647125.1 | HH998993.1 | E02531.1 | HI379117.1 | DL017634.1 | HI000256.1 | HW088155.1 |
| DM383639.1 | DL030702.1 | GM647093.1 | HH998946.1 | E01696.1 | HI378390.1 | DL022272.1 | HI000218.1 | HW081696.1 |
| DM462110.1 | DD491118.1 | GM647053.1 | HH997219.1 | E01386.1 | HI378134.1 | DL022208.1 | HI000160.1 | HW081621.1 |
| DM383014.1 | CS646209.1 | GM640066.1 | HH997155.1 | E01122.1 | HI378102.1 | DL025755.1 | HI000124.1 | HW081585.1 |
| DM382861.1 | CS645037.1 | GM627487.1 | HH970256.1 | E00907.1 | HI376117.1 | DL025723.1 | HI552120.1 | HW099465.1 |
| DM381865.1 | DD461199.1 | GM627455.1 | HH998893.1 | E00644.1 | HI375019.1 | DL025691.1 | HI470712.1 | HW103536.1 |
| HC054884.1 | DD460015.1 | GM627423.1 | HH998832.1 | DD146393.1 | HI371010.1 | DL025595.1 | HI470593.1 | HV703087.1 |
| HC053896.1 | DD459054.1 | GM627391.1 | HH998789.1 | DD146206.1 | HI370369.1 | DL025563.1 | HI544506.1 | FW393466.1 |
| HC053831.1 | DD458760.1 | GM627359.1 | HH998723.1 | DD145693.1 | HI369899.1 | DL029705.1 | HI559258.1 | FW393021.1 |
| HC051949.1 | DD453835.1 | GM627327.1 | HH997075.1 | DD144851.1 | HI369415.1 | DL029673.1 | HI568907.1 | FW396363.1 |
| HC051657.1 | DD453487.1 | GM661261.1 | HH997018.1 | DD069828.1 | HI369123.1 | DL025536.1 | HI568846.1 | FW395234.1 |
| HC050667.1 | DD457034.1 | GM661229.1 | HH996966.1 | DD112412.1 | HI369080.1 | DL025504.1 | HI564249.1 | FW417602.1 |
| HC045509.1 | DD452061.1 | GM661197.1 | HH981089.1 | DD142082.1 | HI369023.1 | DL025355.1 | HI564119.1 | HH934827.1 |
| HC045477.1 | DD456098.1 | GM654139.1 | HH998685.1 | DD152366.1 | HI424140.1 | DL025323.1 | HI563643.1 | HH834809.1 |
| HC045445.1 | CS631738.1 | GM654075.1 | HH998612.1 | DD139895.1 | HI201814.1 | DL025291.1 | HI563611.1 | HH834342.1 |
| HC045413.1 | CS631238.1 | GM654043.1 | HH996934.1 | DD082055.1 | HI553298.1 | DL025227.1 | HI563576.1 | HH833644.1 |
| HC045381.1 | CS632188.1 | GM654011.1 | HH996902.1 | DD081766.1 | HI553262.1 | DL021951.1 | HI558017.1 | HH833612.1 |
| HC045349.1 | CS627089.1 | GM653979.1 | HH996781.1 | DD147799.1 | HI553190.1 | DL021919.1 | HI551323.1 | HH833580.1 |
| HC045317.1 | DD435325.1 | GM646986.1 | HH994575.1 | DD147767.1 | HI586922.1 | DL021887.1 | HI000035.1 | HH833548.1 |
| HC045285.1 | BD356487.1 | GM646954.1 | HH987446.1 | DD147735.1 | HI539184.1 | DL021855.1 | HI003672.1 | HH833516.1 |
| HC049378.1 | BD350537.1 | GM646922.1 | HH954761.1 | DD147703.1 | HI583974.1 | DL021823.1 | HI003624.1 | HH833484.1 |
| HC047465.1 | BD359230.1 | GM646890.1 | HH954283.1 | DD147671.1 | HI583879.1 | DL021791.1 | HI003585.1 | HH833452.1 |
| HC047433.1 | BD358105.1 | GM646858.1 | HH980612.1 | DD080952.1 | HI583843.1 | DL017425.1 | HI001828.1 | HH833420.1 |
| HC047401.1 | BD341687.1 | GM646826.1 | HH980508.1 | DD138772.1 | HI207674.1 | DL038461.1 | HI001767.1 | HH833388.1 |
| HC047369.1 | BD325676.1 | GM640023.1 | HH980440.1 | DD137697.1 | DL102468.1 | DL038429.1 | HI001730.1 | DL102866.1 |
| HC047337.1 | BD325582.1 | GM639991.1 | HH987167.1 | DD136721.1 | DL102436.1 | DL038397.1 | HI544129.1 | DL102834.1 |
| HC047305.1 | BD325163.1 | GM639959.1 | HH957943.1 | DD132296.1 | DL102404.1 | DL038365.1 | HI465474.1 | DL102802.1 |
| HC047273.1 | BD312805.1 | GM639927.1 | HH980367.1 | CQ816946.1 | DL102372.1 | DL038333.1 | HI465454.1 | DL102770.1 |
| HC047049.1 | BD319674.1 | GM639895.1 | HH980320.1 | CQ815503.1 | DL102340.1 | DL013170.1 | HI001610.1 | DL102738.1 |
| HC047017.1 | GM630277.1 | GM639863.1 | HH980170.1 | CQ814104.1 | DL097695.1 | DL013138.1 | HI001510.1 | DL107518.1 |
| HC046985.1 | GM630245.1 | GM627316.1 | HH980135.1 | CQ814072.1 | DL097567.1 | DL013106.1 | HI003392.1 | DL107490.1 |
| HC046953.1 | GM630213.1 | GM627289.1 | HH980064.1 | CQ814039.1 | DL095540.1 | DL013042.1 | HI003351.1 | DL107458.1 |
| HC046921.1 | GM630181.1 | GM627257.1 | HH979890.1 | CQ814007.1 | DL095508.1 | DL013010.1 | HI003310.1 | DL107394.1 |
| HC046861.1 | GM630149.1 | GM627225.1 | HH996731.1 | CQ813975.1 | DL095476.1 | DL033905.1 | HI464689.1 | DL102589.1 |
| HC046829.1 | GM630117.1 | GM627193.1 | HH996659.1 | CQ813943.1 | DL095444.1 | DL033873.1 | HI464657.1 | DL102557.1 |
| HC046797.1 | GM737384.1 | GM627161.1 | HH996619.1 | CQ813911.1 | DL095412.1 | DL033841.1 | HI549489.1 | DL102525.1 |
| HC046765.1 | GM698660.1 | GM627129.1 | HH996557.1 | CQ813879.1 | DL095380.1 | DL033809.1 | HI542538.1 | DL098015.1 |
| HC046733.1 | GM698606.1 | GM661113.1 | HH998470.1 | CQ813847.1 | DL095348.1 | DL033777.1 | HC490828.1 | DL097983.1 |
| HC046701.1 | GM698564.1 | GM661081.1 | HH998425.1 | CQ813815.1 | DL091470.1 | DL033745.1 | HC490796.1 | DL097951.1 |
| HC039666.1 | GM635048.1 | GM661049.1 | HH998376.1 | CQ813694.1 | DL091438.1 | DL031346.1 | HC490764.1 | DL112609.1 |
| HC046684.1 | GM635016.1 | GM661017.1 | HH998343.1 | CQ802180.1 | DL091406.1 | DL027473.1 | HC490732.1 | DL112577.1 |

| | | | | | | | | |
|---|---|---|---|---|---|---|---|---|
| HC046652.1 | GM634984.1 | GM660985.1 | HH999912.1 | CQ795500.1 | DL091374.1 | DL027441.1 | HC490700.1 | DL112545.1 |
| HC046620.1 | GM634952.1 | GM660953.1 | HH979829.1 | CQ792425.1 | DL087481.1 | DL027409.1 | HC490668.1 | DL112513.1 |
| HC046588.1 | GM634920.1 | GM653959.1 | HH979764.1 | CQ785945.1 | DL087449.1 | DL027377.1 | HC490504.1 | DL112481.1 |
| HC046556.1 | GM634888.1 | GM653927.1 | HH979723.1 | CQ784681.1 | DL087417.1 | DL023565.1 | HC490369.1 | DL097828.1 |
| HC046524.1 | GM629954.1 | GM653863.1 | HH979679.1 | CQ778923.1 | DL112259.1 | DL023533.1 | HC472294.1 | DL097796.1 |
| HC046492.1 | GM642408.1 | GM653783.1 | HH976531.1 | CQ778513.1 | DL112227.1 | DL023501.1 | DL010503.1 | DL097764.1 |
| HC046460.1 | GM642234.1 | DL212467.1 | HH999876.1 | CQ771002.1 | DL112195.1 | DL023469.1 | DL010471.1 | DL095737.1 |
| HC046428.1 | GM642272.1 | DL206302.1 | HH999833.1 | CQ768121.1 | DL123351.1 | DL023437.1 | DL010439.1 | DL095673.1 |
| HC046396.1 | GM656339.1 | DL220816.1 | HC869582.1 | CQ759680.1 | DL123319.1 | DL023405.1 | DL010407.1 | DL095609.1 |
| HC046364.1 | GM656307.1 | FB714981.1 | HC869550.1 | CQ757745.1 | DL123287.1 | DL020390.1 | DL038781.1 | DL095577.1 |
| HC046332.1 | GM656275.1 | FB714486.1 | HC877360.1 | CQ756684.1 | DL123255.1 | DL020358.1 | DL038749.1 | DL095545.1 |
| HC046081.1 | GM656243.1 | FB714403.1 | HC876978.1 | CQ754049.1 | DL123223.1 | DL020326.1 | DL038717.1 | DL117382.1 |
| HC046049.1 | GM656211.1 | FB714091.1 | HC869458.1 | AX241922.1 | DL123191.1 | DL020294.1 | DL035093.1 | DL117350.1 |
| HC046017.1 | GM656179.1 | FB713959.1 | HC869426.1 | AX241890.1 | DL118905.1 | DL020262.1 | DL034878.1 | DL117318.1 |
| HC046289.1 | GM649186.1 | FB713735.1 | HC876899.1 | AX241858.1 | DL118873.1 | DL020230.1 | DL034809.1 | DL117286.1 |
| HC046257.1 | GM649154.1 | FB708544.1 | HC876864.1 | AX241826.1 | DL118841.1 | DL020198.1 | DL030714.1 | DL117254.1 |
| HC046225.1 | GM649122.1 | FB708020.1 | HC868277.1 | AX241794.1 | DL118809.1 | DL015795.1 | DL030682.1 | DL117222.1 |
| HC046193.1 | GM649090.1 | FB675138.1 | FW310433.1 | AX241762.1 | DL109257.1 | DL015763.1 | DL026745.1 | DL091641.1 |
| HC046161.1 | GM649026.1 | FB668145.1 | FW310141.1 | AX241730.1 | DL109225.1 | DL015731.1 | DL026713.1 | DL091609.1 |
| HC046129.1 | GM648994.1 | FB667107.1 | FW331859.1 | AX241634.1 | DL109193.1 | DL047253.1 | DL026681.1 | DL091577.1 |
| HC046097.1 | GM642191.1 | FB666796.1 | FW309716.1 | AX241602.1 | DL109161.1 | DL047221.1 | DL019470.1 | DL087966.1 |
| HC045979.1 | GM642159.1 | CS724665.1 | FW308443.1 | AX241506.1 | DL109129.1 | DL047189.1 | DL019438.1 | DL087934.1 |
| HC045947.1 | GM641015.1 | FB676541.1 | FW334264.1 | DL017894.1 | DL109097.1 | DL047157.1 | DL019406.1 | DL121765.1 |
| HC045915.1 | GM640983.1 | FB676509.1 | FW334225.1 | DL017830.1 | DL104363.1 | DL047125.1 | DL014907.1 | DL121733.1 |
| HC045883.1 | GM640919.1 | FB705963.1 | FW333479.1 | DL017799.1 | DL104331.1 | DL047093.1 | DL014875.1 | DL121701.1 |
| HC045851.1 | GM640887.1 | FB704838.1 | FW307697.1 | DL017767.1 | DL099789.1 | DL047061.1 | DL014843.1 | DL121637.1 |
| HC045819.1 | GM640855.1 | FB701524.1 | FW306955.1 | DL017735.1 | DL099757.1 | DL043239.1 | CS603481.1 | DL121605.1 |
| HC045787.1 | GM628501.1 | FB701775.1 | FW332698.1 | DL017703.1 | DL093747.1 | DL043207.1 | CS603449.1 | DL087746.1 |
| HC045755.1 | GM628469.1 | FB701085.1 | FW332321.1 | DL017671.1 | DL093715.1 | DL043175.1 | CS603417.1 | DL087714.1 |
| HC045723.1 | GM628437.1 | FB660513.1 | FW305436.1 | DL017639.1 | DL093683.1 | DL043143.1 | CS603385.1 | DL087682.1 |
| HC045693.1 | GM628405.1 | FB580559.1 | FW304697.1 | DL022373.1 | DL093587.1 | DL043111.1 | CS603353.1 | DL087650.1 |
| HC045661.1 | GM628373.1 | FB665233.1 | HC769812.1 | DL022341.1 | DL093555.1 | DL043079.1 | CS603321.1 | DL087618.1 |
| HC045629.1 | GM628341.1 | FB654431.1 | HC767350.1 | DL022309.1 | DL089940.1 | DL039262.1 | CS603289.1 | DL128973.1 |
| HC045597.1 | GM628309.1 | FB656254.1 | HC757682.1 | DL022277.1 | DL089908.1 | DL039230.1 | CS603257.1 | DL125517.1 |
| HC045565.1 | GM662114.1 | FB654879.1 | HC757127.1 | DL022245.1 | DL089876.1 | DL039198.1 | CS603193.1 | DL125453.1 |
| DM058795.1 | GM662082.1 | FB580286.1 | HC733784.1 | DL022213.1 | DL089844.1 | DL039166.1 | CS603161.1 | DL125421.1 |
| DM063077.1 | GM662050.1 | FB653544.1 | HC733777.1 | DL022181.1 | DL123136.1 | DL039134.1 | CS603129.1 | DL125389.1 |
| DM057727.1 | GM662018.1 | FB580189.1 | HC733700.1 | DL025728.1 | DL123104.1 | DL039102.1 | CS603097.1 | DL125357.1 |
| DM041713.1 | GM661986.1 | FB580125.1 | HC732457.1 | DL025696.1 | DL118690.1 | DL035478.1 | CS603065.1 | DL121568.1 |
| DM056743.1 | GM661954.1 | FB580077.1 | HC731569.1 | DL025664.1 | DL118658.1 | DL035446.1 | CS603033.1 | DL121536.1 |
| DM045271.1 | GM661922.1 | DL202493.1 | HC731352.1 | DL025632.1 | DL118626.1 | DL035414.1 | CS603001.1 | DL121504.1 |
| DM044993.1 | GM654832.1 | DL189522.1 | HC731320.1 | DL025600.1 | DL114230.1 | DL035382.1 | CS602967.1 | DL121472.1 |
| DM039582.1 | GM654800.1 | DL106566.1 | HC731288.1 | DL025568.1 | DL114198.1 | DL035350.1 | CS602935.1 | DL121440.1 |
| DM044674.1 | GM654768.1 | DL106534.1 | HC731256.1 | DL029710.1 | DL114166.1 | DL035318.1 | CS608438.1 | DL121408.1 |
| DM060715.1 | GM647775.1 | DL106502.1 | HC471781.1 | DL029678.1 | DL114134.1 | HI002074.1 | CS608351.1 | DL136605.1 |
| GN075527.1 | GM647743.1 | DL128699.1 | HC471749.1 | DL029646.1 | DL114102.1 | HI002024.1 | CS607884.1 | DL117196.1 |
| GN067802.1 | GM647711.1 | DL128631.1 | HC471681.1 | DL029614.1 | DL114070.1 | HI001941.1 | CS607662.1 | DL117164.1 |

| | | | | | | | | |
|---|---|---|---|---|---|---|---|---|
| GN067774.1 | GM647679.1 | DL128546.1 | HC471709.1 | DL029582.1 | DL144485.1 | HI000273.1 | CS607062.1 | DL117132.1 |
| GN067614.1 | GM647647.1 | DL125069.1 | HC471653.1 | DL025509.1 | DL126210.1 | HI000233.1 | CS606989.1 | DL117100.1 |
| GN060837.1 | GM647615.1 | FW499103.1 | HC471621.1 | DL025477.1 | DL126178.1 | HI000189.1 | CS606964.1 | DL117068.1 |
| GN060522.1 | GM640812.1 | FW499031.1 | HC471509.1 | DL025445.1 | DL122962.1 | HI000133.1 | CS606903.1 | DL117036.1 |
| GN052391.1 | GM640779.1 | FW504969.1 | HC471567.1 | DL025413.1 | DL122930.1 | HI071763.1 | CS606821.1 | DL099064.1 |
| GN052359.1 | GM640747.1 | FW504934.1 | HC729387.1 | DL025381.1 | DL122898.1 | HI180954.1 | BD224818.1 | DL099032.1 |
| GN059748.1 | GM640715.1 | FW497142.1 | HC729079.1 | DL033939.1 | DL122834.1 | HI635304.1 | BD223242.1 | DL099000.1 |
| GN051180.1 | GM640683.1 | FW420474.1 | HC728819.1 | DL025328.1 | DL122802.1 | HI470604.1 | BD222646.1 | DL098968.1 |
| GN048968.1 | GM640651.1 | FW420442.1 | HC728146.1 | DL025296.1 | DL122770.1 | HI564707.1 | BD218828.1 | DL098936.1 |
| DM014244.1 | GM628297.1 | FW420410.1 | HC727412.1 | DJ381552.1 | DL114024.1 | HI568925.1 | BD218163.1 | DL092830.1 |
| DM024281.1 | GM627973.1 | FW496933.1 | HC726157.1 | DJ381394.1 | DL113992.1 | HI568855.1 | BD206187.1 | DL092798.1 |
| DM022396.1 | GM627941.1 | FW420385.1 | HC689090.1 | DJ381043.1 | DL113960.1 | HI554956.1 | BD204978.1 | DL092766.1 |
| DM022320.1 | GM715477.1 | FW420353.1 | HC688458.1 | DJ381011.1 | DL113928.1 | HI564203.1 | DD282405.1 | DL089151.1 |
| DM038580.1 | GM661911.1 | FW420321.1 | HC688342.1 | DJ380979.1 | DL113896.1 | HI564095.1 | DD288436.1 | DL089119.1 |
| DM022211.1 | GM661879.1 | FW496736.1 | HC687942.1 | DJ380947.1 | DL113864.1 | HI563620.1 | DD286606.1 | DL089087.1 |
| DM022157.1 | GM661847.1 | HI653876.1 | HC679621.1 | DJ380915.1 | DL109063.1 | HI563585.1 | CS352953.1 | DL089055.1 |
| DM015996.1 | GM661815.1 | HI653806.1 | HC490981.1 | DJ380883.1 | DL109031.1 | HI508555.1 | CS352518.1 | DL089023.1 |
| DM015952.1 | GM661783.1 | HI653634.1 | HC490949.1 | DJ380851.1 | DL108999.1 | HI575630.1 | CS352401.1 | DL088991.1 |
| DM027082.1 | GM661751.1 | HI653600.1 | HC490917.1 | DJ380831.1 | DL104297.1 | HI551344.1 | CS284249.1 | DL122350.1 |
| DM027046.1 | GM661719.1 | HI653528.1 | HC490885.1 | DJ379274.1 | DL036392.1 | HI544144.1 | CS283927.1 | BD180805.1 |
| DM026366.1 | GM654725.1 | HI653415.1 | HC687197.1 | DJ389569.1 | DL036360.1 | HI508245.1 | CS274785.1 | AX742850.1 |
| GN045774.1 | GM654693.1 | HI653362.1 | HC490845.1 | DJ389231.1 | DL036328.1 | HI000049.1 | CS252533.1 | AX739968.1 |
| GN042730.1 | GM654661.1 | HI653281.1 | HC021028.1 | DJ388661.1 | DL036296.1 | HI000007.1 | CS250526.1 | AX722063.1 |
| GN041689.1 | GM654629.1 | HI653223.1 | HC025896.1 | DJ388524.1 | DL044243.1 | HI003646.1 | CS248881.1 | BD178182.1 |
| GN034311.1 | GM654597.1 | HI652909.1 | HC025513.1 | CS726742.1 | DL044211.1 | HI003594.1 | CS245371.1 | BD177353.1 |
| DL259355.1 | GM654564.1 | HI648668.1 | HC025481.1 | CS724424.1 | DL044179.1 | HI003560.1 | CS244156.1 | AX718180.1 |
| DL258278.1 | GM647571.1 | HI647557.1 | HC025449.1 | DJ352128.1 | DL044147.1 | HI003510.1 | CS244236.1 | AX717589.1 |
| DL257631.1 | GM627900.1 | HI646889.1 | HC010652.1 | DJ357787.1 | DL044115.1 | HI001837.1 | CS244204.1 | AX710312.1 |
| DL241372.1 | GM627804.1 | HH807335.1 | HC010194.1 | DJ357755.1 | DL044083.1 | HI001777.1 | CS243766.1 | AX709548.1 |
| DL241786.1 | GM871610.1 | HH807044.1 | HC010118.1 | DJ357723.1 | DL048220.1 | HI001739.1 | CS239686.1 | AX708509.1 |
| DL240642.1 | GM870420.1 | HH804172.1 | HC008558.1 | DJ357689.1 | DL048188.1 | HI465710.1 | CS231149.1 | AX705041.1 |
| FB748395.1 | GM661690.1 | HH794953.1 | HC007839.1 | DJ357657.1 | DL048156.1 | HI465521.1 | CS203485.1 | AX701591.1 |
| FB742059.1 | GM661658.1 | HH779679.1 | HC007815.1 | DJ357625.1 | DL048124.1 | HI574581.1 | CS227324.1 | AX699465.1 |
| FB748890.1 | GM661626.1 | HH777922.1 | HC007783.1 | DJ357593.1 | DL048092.1 | HI001619.1 | BD420445.1 | AX699433.1 |
| DL233418.1 | GM661594.1 | HC471943.1 | HC007751.1 | DJ357391.1 | DL048060.1 | HI001577.1 | BD497462.1 | AX696328.1 |
| DL207299.1 | GM661562.1 | HC471879.1 | HC007719.1 | DJ363059.1 | DL048029.1 | HC486448.1 | BD451759.1 | AX686643.1 |
| DL213334.1 | GM661530.1 | HC678951.1 | HC007687.1 | DJ362319.1 | HC045813.1 | HC486324.1 | BD451717.1 | AX685803.1 |
| DL206825.1 | GM654536.1 | HC678780.1 | HC007655.1 | DJ355105.1 | HC045781.1 | HC475384.1 | BD398291.1 | AX685147.1 |
| DL219581.1 | GM654504.1 | HC678458.1 | HC007627.1 | DJ361249.1 | HC045749.1 | HC466639.1 | BD440512.1 | AX682974.1 |
| DL219501.1 | GM654472.1 | HC668134.1 | HC007595.1 | DJ361215.1 | HC045717.1 | HC466607.1 | BD429492.1 | AX671501.1 |
| DL220941.1 | GM654440.1 | HC521218.1 | HC007563.1 | DJ361182.1 | HC045687.1 | HC474329.1 | BD406397.1 | AX665487.1 |
| DL206308.1 | DJ053503.1 | HC504597.1 | HC007531.1 | DJ354102.1 | HC045655.1 | HC474156.1 | BD460609.1 | AX664347.1 |
| DL230371.1 | DJ053217.1 | HC494422.1 | HC010770.1 | DJ360289.1 | HC045623.1 | HC466282.1 | BD427996.1 | AX662233.1 |
| DL220821.1 | DJ053085.1 | HC500313.1 | DM377195.1 | DJ339862.1 | HC045591.1 | HC491794.1 | BD395647.1 | AX657147.1 |
| FB714507.1 | DJ052890.1 | HC499867.1 | DM374886.1 | DJ339830.1 | HC045559.1 | HC491762.1 | BD427311.1 | AX657115.1 |
| FB713802.1 | CS810641.1 | FW301505.1 | HC006523.1 | DJ339798.1 | HC045527.1 | HC481959.1 | BD395075.1 | AX657073.1 |
| FB713770.1 | CS810537.1 | FW300963.1 | HC005538.1 | DJ339766.1 | DM379015.1 | HC481594.1 | BD426876.1 | BD176082.1 |

| | | | | | | | | |
|---|---|---|---|---|---|---|---|---|
| FB713738.1 | FB292243.1 | FW299062.1 | HC004350.1 | DJ339734.1 | HC035620.1 | HC491455.1 | BD394369.1 | BD174395.1 |
| FB712994.1 | CS811062.1 | FW298771.1 | DM371005.1 | DJ339702.1 | HC037054.1 | HC491052.1 | BD453340.1 | AX645182.1 |
| FB708584.1 | DJ020650.1 | HC470491.1 | DM370973.1 | DJ339605.1 | HC022643.1 | FV533205.1 | BD453312.1 | AJ005287.1 |
| FB672298.1 | DJ017822.1 | HC488038.1 | DM370941.1 | DJ339573.1 | HC025505.1 | FV531717.1 | BD453280.1 | AX642224.1 |
| FB670957.1 | DJ019821.1 | HC477909.1 | DM370909.1 | DJ339541.1 | HC025473.1 | FV531685.1 | BD388745.1 | AX616538.1 |
| FB668773.1 | DJ015682.1 | HC486450.1 | DM370838.1 | DJ339085.1 | HC025441.1 | FV530771.1 | BD350055.1 | AX608812.1 |
| FB667423.1 | CS803339.1 | HC486336.1 | DM370804.1 | DJ330228.1 | HC025409.1 | FV522823.1 | BD356389.1 | HW408579.1 |
| FB667113.1 | FB295838.1 | HC492537.1 | DM370769.1 | DJ330129.1 | HC024865.1 | FV522725.1 | BD353553.1 | HW408547.1 |
| FB666802.1 | FB295157.1 | HC475385.1 | DM370671.1 | DJ329297.1 | HC010427.1 | FV534647.1 | BD359743.1 | HW408483.1 |
| FB676544.1 | FB294173.1 | HC466640.1 | DM370639.1 | DJ328783.1 | HC009864.1 | FV534472.1 | BD342953.1 | HW408420.1 |
| FB676512.1 | DJ047311.1 | HC466608.1 | DM370607.1 | DJ327775.1 | HC020913.1 | HC460423.1 | BD341814.1 | HW390755.1 |
| FB705977.1 | DJ047006.1 | HC474330.1 | DM370575.1 | DJ326631.1 | HC007807.1 | AF504292.1 | AX145689.1 | HW399542.1 |
| FB704843.1 | DJ052214.1 | HC466284.1 | DM370543.1 | DJ088502.1 | HC007775.1 | AF287353.1 | AX145657.1 | HW392026.1 |
| FB701758.1 | DJ052017.1 | HC491795.1 | DM370511.1 | DJ086458.1 | HC007619.1 | HC456288.1 | AX145625.1 | HW390527.1 |
| FB701527.1 | DJ045578.1 | HC491763.1 | DM370479.1 | DJ083898.1 | HC007523.1 | HC460188.1 | AX145593.1 | HW390418.1 |
| FB701783.1 | DJ050483.1 | HC481595.1 | DM367502.1 | DJ082702.1 | HC010736.1 | AF195002.1 | AX145561.1 | HW389033.1 |
| FB701107.1 | DJ049476.1 | HC481556.1 | DM205204.1 | DD060704.1 | DM370761.1 | HC452239.1 | AX145529.1 | HW388641.1 |
| DL102223.1 | DJ048842.1 | HC491456.1 | DM371901.1 | DD116398.1 | DM370663.1 | HC452161.1 | AX145497.1 | HW387472.1 |
| DL102167.1 | CS793985.1 | HC491023.1 | DM365915.1 | DD089598.1 | DM370631.1 | HB474831.1 | AX145465.1 | HW387046.1 |
| DL102135.1 | CS800858.1 | FV533516.1 | DM204360.1 | DD102582.1 | DM370599.1 | HB463660.1 | AX145433.1 | HW386242.1 |
| DL117003.1 | CS800119.1 | FV533425.1 | HC003053.1 | DD102449.1 | DM370567.1 | HB471255.1 | AX145401.1 | HW386178.1 |
| DL116971.1 | CS720125.1 | FV531718.1 | HB865993.1 | DD102417.1 | DM370535.1 | HB469591.1 | AX145368.1 | HW386114.1 |
| DL116939.1 | CS799994.1 | FV531686.1 | HB865029.1 | DD052029.1 | DM370503.1 | HB469098.1 | AX145336.1 | HW385922.1 |
| DL116907.1 | CS667953.1 | FV531372.1 | HB864975.1 | DD057916.1 | DM370471.1 | HB468036.1 | AX145304.1 | HW385908.1 |
| DL116875.1 | CS646202.1 | FV530772.1 | HB864989.1 | DD057884.1 | DM371857.1 | DM152551.1 | AX145272.1 | HW380896.1 |
| DL116843.1 | CS645214.1 | FV522824.1 | HB864933.1 | DD057852.1 | HC001554.1 | DM150248.1 | AX145240.1 | HW380819.1 |
| DL116815.1 | CS644547.1 | FV522726.1 | HB864901.1 | DD056821.1 | HB865021.1 | HB460454.1 | AX145208.1 | HW367116.1 |
| DL116783.1 | DD462107.1 | DM162328.1 | HB864869.1 | DD055234.1 | HB865013.1 | HB455307.1 | AX145176.1 | HW366921.1 |
| DL116751.1 | DD460664.1 | HB486513.1 | HB864837.1 | GM654833.1 | HB864957.1 | HB453860.1 | AX145144.1 | HW376186.1 |
| DL112062.1 | DD460516.1 | HB476401.1 | HB864805.1 | GM654801.1 | HB864925.1 | HB445369.1 | AX145112.1 | HW376097.1 |
| DL112030.1 | DD455161.1 | HB485578.1 | HB864773.1 | GM654769.1 | HB864893.1 | HB444510.1 | AX145080.1 | HW366474.1 |
| DL111998.1 | DD459039.1 | HB463661.1 | HB866992.1 | GM647776.1 | HB864861.1 | DM139931.1 | AX145048.1 | HW375241.1 |
| DL111966.1 | DD458753.1 | HB463629.1 | HB859501.1 | GM647744.1 | HB864829.1 | DM138461.1 | AX145016.1 | HW364675.1 |
| DL019634.1 | DD457861.1 | HB471266.1 | HB866112.1 | GM647712.1 | HB864797.1 | DM138429.1 | HW337026.1 | HV956419.1 |
| DL019602.1 | DD456324.1 | HB469339.1 | HC001102.1 | GM647680.1 | HB864765.1 | DM143079.1 | HW321845.1 | HV944652.1 |
| DL015199.1 | CS631731.1 | HB469099.1 | HC000285.1 | GM647648.1 | HB886341.1 | DM137713.1 | HW321736.1 | HV941167.1 |
| DL015167.1 | CS631224.1 | HB468037.1 | HB999517.1 | GM640813.1 | HB866963.1 | DM131552.1 | HW336742.1 | HV779885.1 |
| DL015135.1 | CS627770.1 | DM152698.1 | HB977969.1 | GM640780.1 | FB509149.1 | DM131109.1 | HW336614.1 | HV778423.1 |
| DL015103.1 | CS632687.1 | DM152552.1 | HB977471.1 | GM640748.1 | GM772976.1 | GN082823.1 | HW336534.1 | HV774725.1 |
| DL015071.1 | CS632428.1 | DM146441.1 | DM197917.1 | GM640716.1 | GM709014.1 | GN088986.1 | HW335968.1 | HV773798.1 |
| DL015039.1 | CS632181.1 | HB340405.1 | DM197885.1 | GM640684.1 | GM708982.1 | GN081976.1 | HW329277.1 | HV743384.1 |
| DL010549.1 | CS626886.1 | HB338918.1 | DM197716.1 | GM640652.1 | GM708635.1 | DL232987.1 | HW328736.1 | HV700898.1 |
| DL010517.1 | CS623724.1 | HA643290.1 | DM196035.1 | GM628298.1 | GM655011.1 | U49852.1 | HW328664.1 | HW287332.1 |
| DL010485.1 | CS623660.1 | HA641614.1 | DM190473.1 | GM046520.1 | GM654979.1 | L08947.1 | HW328550.1 | HW302700.1 |
| DL010453.1 | CS623538.1 | HA641550.1 | DM195143.1 | GM627974.1 | GM837742.1 | M74307.1 | HW328437.1 | HW286520.1 |
| DL010421.1 | DD450329.1 | HA641227.1 | DM190209.1 | GM627942.1 | GM755067.1 | DM059624.1 | HW328155.1 | HW286485.1 |
| DL046850.1 | DD450297.1 | HA641149.1 | DM189897.1 | GM715480.1 | GM043751.1 | DM058846.1 | HW328083.1 | HW295583.1 |

| | | | | | | | | |
|---|---|---|---|---|---|---|---|---|
| DL046818.1 | DD450265.1 | HA635319.1 | DM194649.1 | GM661912.1 | GM836860.1 | DM042459.1 | HW318466.1 | HW307488.1 |
| DL046786.1 | DD450233.1 | HA639793.1 | DM148843.1 | HW408390.1 | GM754517.1 | DM045533.1 | HW335175.1 | HW294147.1 |
| DL042868.1 | DD450160.1 | HA639031.1 | DM148399.1 | HW408358.1 | GM754226.1 | DM045427.1 | HW314387.1 | HW294048.1 |
| DL042836.1 | DD450126.1 | HA638499.1 | DM152834.1 | HW390757.1 | FB511152.1 | BD453233.1 | HW314146.1 | HW306883.1 |
| DL042804.1 | DD438004.1 | DM094904.1 | DM152573.1 | HW399252.1 | GM752448.1 | BD453201.1 | HW314018.1 | HW293163.1 |
| DL042772.1 | DD449095.1 | GN374326.1 | DM147330.1 | HW382300.1 | GM712953.1 | BD442862.1 | HW312157.1 | HW293032.1 |
| DL042740.1 | DD436721.1 | GN367621.1 | DM155880.1 | HW390529.1 | GM652754.1 | BD410026.1 | HW312099.1 | HW292755.1 |
| DL042708.1 | CS620445.1 | GN367586.1 | DM146353.1 | HW390483.1 | GM652722.1 | BD399052.1 | HW311239.1 | HW291171.1 |
| DL042676.1 | CS613798.1 | CS322144.1 | DM155731.1 | HW389595.1 | GM652690.1 | BD441302.1 | HW308556.1 | HW291074.1 |
| DL038795.1 | CS613766.1 | DD271417.1 | DM150117.1 | HW388643.1 | GM652658.1 | BD497560.1 | HW307827.1 | HW291042.1 |
| DL038763.1 | CS612792.1 | DD265348.1 | DM149952.1 | HW387860.1 | GM652626.1 | BD430790.1 | HW307757.1 | HW291010.1 |
| DL038731.1 | CS611860.1 | CS302581.1 | HB461820.1 | HW387476.1 | GM652594.1 | BD450798.1 | HW307725.1 | HW290978.1 |
| DJ444610.1 | CS604550.1 | CS299519.1 | HB455315.1 | HW387380.1 | GM645500.1 | BD408076.1 | HW307693.1 | HW290946.1 |
| DJ444573.1 | CS604518.1 | CS300485.1 | HB455098.1 | HW386596.1 | GM645468.1 | BD439933.1 | HW315901.1 | HW290883.1 |
| DJ439620.1 | CS604486.1 | DD258236.1 | HB453826.1 | HW386564.1 | GM645436.1 | BD419034.1 | HW315401.1 | HW290851.1 |
| DJ434392.1 | CS604422.1 | CS287625.1 | HB463474.1 | HW386532.1 | GM638607.1 | BD429573.1 | HW315353.1 | HW290130.1 |
| DJ443469.1 | CS604358.1 | CS287556.1 | HB444490.1 | HW386500.1 | GM638575.1 | BD407504.1 | HW315160.1 | HW298235.1 |
| DJ438366.1 | CS604326.1 | DD240705.1 | HB444518.1 | HW386276.1 | GM638543.1 | BD429348.1 | HW315096.1 | HW287767.1 |
| DJ438293.1 | CS604230.1 | DD240657.1 | DM121081.1 | HW386244.1 | GM638511.1 | BD429009.1 | HW314822.1 | HW267801.1 |
| DJ438212.1 | CS501253.1 | DD240594.1 | DM138469.1 | HW386116.1 | GM638479.1 | BD438367.1 | HW314768.1 | HW267617.1 |
| DJ427989.1 | CS500180.1 | DD240489.1 | DM143380.1 | HW386020.1 | GM638447.1 | BD396027.1 | HW115419.1 | HW064449.1 |
| DJ402550.1 | CS498531.1 | DD234704.1 | DM138347.1 | HW385956.1 | GM633748.1 | BD428086.1 | HV543481.1 | HW062318.1 |
| DJ402294.1 | CS498431.1 | DD247162.1 | DM138315.1 | HW380898.1 | GM633716.1 | BD395337.1 | HV539452.1 | HW056013.1 |
| DJ400841.1 | CS494727.1 | DD246843.1 | DM121668.1 | HW380821.1 | GM633684.1 | BD437168.1 | HV539100.1 | HW042073.1 |
| DJ400809.1 | DD418586.1 | E49779.1 | DM137792.1 | FW349607.1 | GM625817.1 | BD446170.1 | HV538744.1 | HW043571.1 |
| DJ400489.1 | DD412070.1 | E41523.1 | DM137127.1 | HW376103.1 | GM625753.1 | BD393736.1 | HV538568.1 | DL031115.1 |
| DJ399401.1 | DD411603.1 | E49208.1 | DM136761.1 | HW366478.1 | GM624729.1 | BD453351.1 | HV543206.1 | DL031083.1 |
| DJ419982.1 | DD411476.1 | E33648.1 | DM131008.1 | HW375251.1 | GM624697.1 | BD453321.1 | HV542738.1 | DL031051.1 |
| DJ419910.1 | DD410773.1 | E39197.1 | DM130797.1 | HW374529.1 | GM624633.1 | BD453289.1 | HV542706.1 | DL031019.1 |
| DJ419614.1 | CS491852.1 | E06875.1 | DM130723.1 | HW381353.1 | GM635910.1 | BD456398.1 | HV515090.1 | DL030987.1 |
| DJ398298.1 | CS489527.1 | E06160.1 | HB436408.1 | HW062191.1 | GM635878.1 | BD391454.1 | HV533421.1 | DL030958.1 |
| DJ418070.1 | CS487193.1 | E05305.1 | HB435562.1 | HF586458.1 | GM622218.1 | BD388965.1 | HV514644.1 | DL027146.1 |
| DJ418647.1 | CS486788.1 | E04908.1 | HB441678.1 | HW042075.1 | GM622186.1 | BD376348.1 | HV534841.1 | DL027114.1 |
| DJ417441.1 | CS483418.1 | E04468.1 | HB441492.1 | HW042043.1 | GM622154.1 | BD375876.1 | HV536482.1 | DL027082.1 |
| DJ381042.1 | CS482974.1 | E04185.1 | HB440989.1 | HW043592.1 | GM631013.1 | BD375678.1 | HV532564.1 | DL027050.1 |
| DJ381010.1 | CS482937.1 | E03524.1 | HB427182.1 | HW043448.1 | GM630981.1 | BD375302.1 | HV515727.1 | DL027018.1 |
| DJ380978.1 | CS482855.1 | E03212.1 | HB426472.1 | HW041878.1 | GM630949.1 | BD373912.1 | HV515477.1 | DL026986.1 |
| DJ380946.1 | CS482814.1 | E02616.1 | HB423135.1 | HW041837.1 | GM630917.1 | BD362582.1 | HV515445.1 | DL026954.1 |
| DJ380914.1 | CS482773.1 | E02473.1 | HB423088.1 | HW049461.1 | GM630892.1 | CS118035.1 | HV515413.1 | DL030913.1 |
| DJ380882.1 | DD401442.1 | E02336.1 | HB403633.1 | HW049389.1 | GM635849.1 | CS117999.1 | HV515381.1 | DL030874.1 |
| DJ380850.1 | DD401410.1 | BD356965.1 | HB416467.1 | HW049358.1 | GM635817.1 | CS117967.1 | HV515349.1 | DL030842.1 |
| DJ380830.1 | DD405193.1 | BD356563.1 | HB413955.1 | HW049326.1 | GM635785.1 | CS114659.1 | HV515285.1 | DL030810.1 |
| DJ389568.1 | DD405161.1 | BD356460.1 | HB393672.1 | HW049294.1 | BD235646.1 | CS114293.1 | HV515253.1 | DL030778.1 |
| DJ389313.1 | DD405129.1 | BD353566.1 | HB396575.1 | HW049262.1 | BD234860.1 | CS106598.1 | HV511724.1 | DL019780.1 |
| DJ388660.1 | DD405097.1 | BD342482.1 | HB394317.1 | HW047588.1 | BD234464.1 | CS105993.1 | HV511496.1 | DL019782.1 |
| CS724423.1 | DD405065.1 | BD341869.1 | HB394285.1 | HW041801.1 | BD232021.1 | CS102979.1 | HV507918.1 | DL019726.1 |
| CS723369.1 | DD405033.1 | BD349856.1 | HB394253.1 | HW041769.1 | BD231934.1 | CS102947.1 | HV510978.1 | DL015195.1 |

| | | | | | | | | |
|---|---|---|---|---|---|---|---|---|
| CS721834.1 | HW065239.1 | BD339057.1 | HB393658.1 | HW043128.1 | BD231166.1 | CS102883.1 | HV509337.1 | DL015163.1 |
| DJ357786.1 | HW065079.1 | BD325642.1 | HB403048.1 | HW041546.1 | BD231054.1 | CS102787.1 | HV508680.1 | DL015131.1 |
| DJ357754.1 | HW064880.1 | BD325549.1 | HB403015.1 | HW047139.1 | BD229102.1 | CS102755.1 | HV508645.1 | DL015099.1 |
| DJ357722.1 | HW064840.1 | BD314384.1 | GN030594.1 | HW048291.1 | BD227217.1 | CS102723.1 | HV508613.1 | DL015067.1 |
| DJ357688.1 | HW061073.1 | BD311556.1 | GN030562.1 | HW044180.1 | BD226910.1 | CS102691.1 | HV508581.1 | DL015035.1 |
| DJ357656.1 | HW057647.1 | BD319654.1 | GN030530.1 | HW029683.1 | BD225752.1 | CS102659.1 | HV508489.1 | DL010545.1 |
| DJ357624.1 | HW056582.1 | BD319472.1 | GN030498.1 | HV975356.1 | BD225688.1 | CS102627.1 | HV512291.1 | DL010513.1 |
| DJ357592.1 | HW056550.1 | BD318833.1 | GN030406.1 | HV985973.1 | BD225656.1 | CS102595.1 | HV512259.1 | DL010481.1 |
| DJ357288.1 | HW056500.1 | BD294337.1 | GN030443.1 | HV985909.1 | BD225624.1 | CS102563.1 | HV512227.1 | DL010449.1 |
| DJ363058.1 | HW062557.1 | BD293450.1 | GN030370.1 | HV985864.1 | BD224834.1 | CS102531.1 | HV512195.1 | DL010417.1 |
| DJ362318.1 | HW062407.1 | BD293409.1 | GN030306.1 | HV974796.1 | BD224128.1 | CS102499.1 | HV512163.1 | CS602881.1 |
| DJ053218.1 | HW056318.1 | BD293049.1 | GN030274.1 | HW029151.1 | BD222495.1 | CS101551.1 | HV512131.1 | CS602849.1 |
| DJ053086.1 | HV503915.1 | BD291625.1 | GN030242.1 | HV965119.1 | DD282953.1 | CS101209.1 | FU971685.1 | CS602785.1 |
| DJ052891.1 | HV503883.1 | BD291381.1 | GN030210.1 | HV960149.1 | DD280734.1 | CS095706.1 | FU759992.1 | CS602753.1 |
| CS810642.1 | HV503819.1 | BD291230.1 | GN030178.1 | HV959480.1 | DD215828.1 | CS091379.1 | FU759935.1 | CS602689.1 |
| CS810539.1 | HV491107.1 | BD299643.1 | GN030114.1 | HV963867.1 | DD215796.1 | CS088888.1 | FU764211.1 | CS602241.1 |
| FB292244.1 | HV503779.1 | BD299608.1 | GN030082.1 | HV958611.1 | DD215764.1 | CS085918.1 | FU757820.1 | CS602083.1 |
| CS811064.1 | HV503747.1 | BD298676.1 | GN030050.1 | HV111853.1 | DD217860.1 | CS083119.1 | BD453306.1 | CS608379.1 |
| DJ020689.1 | HV503715.1 | BD297647.1 | GN030018.1 | HV041728.1 | DD212313.1 | CS082473.1 | BD453274.1 | CS607920.1 |
| CS803340.1 | HV503651.1 | BD296234.1 | GN029986.1 | HV189878.1 | DD211112.1 | CS082345.1 | BD402328.1 | CS600631.1 |
| FB295851.1 | HV503527.1 | BD295215.1 | GN029954.1 | HV218554.1 | DD210005.1 | CS077636.1 | BD413434.1 | CS607086.1 |
| FB295384.1 | HV503495.1 | BD289751.1 | GN029922.1 | HV227959.1 | DD208691.1 | CS075441.1 | BD388660.1 | CS607005.1 |
| FB294174.1 | HV503463.1 | BD287341.1 | GN029890.1 | HV234140.1 | DD206889.1 | CS075348.1 | BD378212.1 | CS599810.1 |
| DJ047312.1 | HV503431.1 | BD274220.1 | GN029792.1 | HV245430.1 | DD206857.1 | AX766726.1 | BD376144.1 | CS605022.1 |
| DJ047017.1 | HV503335.1 | BD273673.1 | GN014133.1 | HV214866.1 | DD206795.1 | AX766361.1 | AY967402.1 | CS604990.1 |
| DJ052322.1 | HV503303.1 | BD273293.1 | GN013386.1 | HV234163.1 | CS249479.1 | AX752032.1 | AY967370.1 | CS604926.1 |
| DJ052215.1 | HV503271.1 | BD272230.1 | GN034224.1 | HV236018.1 | CS236378.1 | BD185652.1 | AY967338.1 | CS604894.1 |
| DJ052019.1 | HV503239.1 | BD271849.1 | GN010228.1 | HV301808.1 | CS120440.1 | BD180803.1 | AY967306.1 | CS604862.1 |
| DJ045754.1 | HV503207.1 | BD271076.1 | GN033558.1 | HV306397.1 | CS119510.1 | AX744000.1 | AY967274.1 | CS604830.1 |
| DJ050484.1 | HV503152.1 | BD271044.1 | GN033526.1 | HV306186.1 | CS119478.1 | AX743503.1 | AY967242.1 | CS604798.1 |
| DJ048843.1 | HV503120.1 | BD270834.1 | GN033494.1 | HV302948.1 | CS119443.1 | AX742848.1 | AY967210.1 | CS604766.1 |
| CS800859.1 | HV503088.1 | DD231225.1 | GN033462.1 | HV302723.1 | CS119410.1 | AX739885.1 | AY967178.1 | CS604734.1 |
| CS800120.1 | HV503056.1 | DD230482.1 | GN033430.1 | HV308863.1 | CS119312.1 | AX721695.1 | AY967146.1 | CS604702.1 |
| CS720126.1 | HV503024.1 | DD230448.1 | GN033398.1 | HV311098.1 | CS119279.1 | BD178180.1 | AY967114.1 | CS604670.1 |
| CS799995.1 | HV502992.1 | DD228576.1 | GN033334.1 | HV803117.1 | CS119247.1 | AX720271.1 | AY967082.1 | CS604636.1 |
| CS707348.1 | HV502960.1 | DD228422.1 | GN033302.1 | HV803085.1 | CS119215.1 | AX717586.1 | AY967050.1 | CS604604.1 |
| CS798882.1 | HV502928.1 | DD227386.1 | GN033270.1 | HV802947.1 | CS119183.1 | AX704648.1 | AY967018.1 | CS604572.1 |
| CS798098.1 | HV502896.1 | DD226688.1 | GN033238.1 | HV802915.1 | CS119151.1 | AX699463.1 | AY966986.1 | CS593087.1 |
| CS806651.1 | HV502832.1 | DD225224.1 | GN033206.1 | HV802883.1 | CS119084.1 | AX699431.1 | AY966954.1 | CS592807.1 |
| GM655519.1 | HV492860.1 | AX179486.1 | GN033174.1 | HV818965.1 | CS119051.1 | AX698627.1 | AY966922.1 | CS592324.1 |
| GM655487.1 | HV502788.1 | AX179423.1 | GN033142.1 | HV512413.1 | CS119019.1 | AX685748.1 | CS055265.1 | CS592114.1 |
| GM655455.1 | HV502756.1 | AX027956.1 | GN033110.1 | HV512272.1 | CS118986.1 | AX684667.1 | CS052524.1 | CS598818.1 |
| GM648366.1 | HV502724.1 | A09538.1 | GN033078.1 | HV512240.1 | CS118954.1 | AX682969.1 | CS052370.1 | CS593439.1 |
| GM648334.1 | HV502692.1 | AX521540.1 | GN033014.1 | HV512176.1 | CS118888.1 | AX670753.1 | CS052335.1 | CS597733.1 |
| GM648302.1 | HV502660.1 | AX521508.1 | GN032918.1 | HV512144.1 | CS118855.1 | AX665483.1 | CS052302.1 | CS585643.1 |
| GM648270.1 | HV502628.1 | AF430194.1 | GN032886.1 | HV511833.1 | CS118822.1 | AX664345.1 | CS051046.1 | CS585011.1 |
| GM648238.1 | HV502596.1 | AX505213.1 | GN032854.1 | HV492312.1 | CS118789.1 | AX662227.1 | CS050719.1 | CS584681.1 |

| | | | | | | | | |
|---|---|---|---|---|---|---|---|---|
| GM648206.1 | HV502532.1 | AX505149.1 | GN032822.1 | HV504952.1 | CS118757.1 | AX662046.1 | CS048044.1 | CS582430.1 |
| GM641403.1 | HV502500.1 | AX498427.1 | GN032790.1 | HV504888.1 | CS118724.1 | AX657145.1 | CS038909.1 | CS588489.1 |
| GM641371.1 | HV492755.1 | AX497097.1 | GN032758.1 | HV504856.1 | CS118692.1 | AX657113.1 | CS029699.1 | CS575460.1 |
| GM641339.1 | HV505319.1 | BD139554.1 | GN032727.1 | HV507454.1 | CS118660.1 | AX657069.1 | CS024305.1 | CS574793.1 |
| GM641307.1 | HV505213.1 | BD138745.1 | GN032695.1 | HV504775.1 | CS118628.1 | BD176080.1 | CS023783.1 | CS572368.1 |
| GM641275.1 | HV507622.1 | BD138637.1 | GN032663.1 | HV504743.1 | CS118561.1 | BD174671.1 | AX143099.1 | DD096739.1 |
| GM641243.1 | HV504990.1 | BD138405.1 | GN032630.1 | HV504711.1 | CS118528.1 | AX644914.1 | AX142971.1 | DD081758.1 |
| GM629052.1 | HV453662.1 | BD135804.1 | GN032598.1 | HV504679.1 | CS118495.1 | AX643977.1 | AX142907.1 | DD081607.1 |
| GM629020.1 | HV450042.1 | BD131910.1 | GN032566.1 | HV504647.1 | CS118462.1 | AX642220.1 | AX142779.1 | DD147791.1 |
| GM628988.1 | HV451954.1 | BD130840.1 | GN032535.1 | HV504615.1 | CS118430.1 | AX615149.1 | AX142715.1 | DD147759.1 |
| GM628956.1 | HV449909.1 | BD130803.1 | GN032503.1 | HV491620.1 | CS118397.1 | AX456490.1 | AX142651.1 | DD147727.1 |
| GM628924.1 | HV453001.1 | BD130771.1 | GN032471.1 | HV504592.1 | CS118365.1 | AX455877.1 | AX142587.1 | DD147695.1 |
| GM716139.1 | HV448964.1 | BD130737.1 | GN032439.1 | HV504560.1 | CS118330.1 | AX454143.1 | AX142457.1 | DD081137.1 |
| GM655230.1 | HV448481.1 | BD130617.1 | GN032407.1 | HV504528.1 | CS118296.1 | AX453994.1 | AX142201.1 | DD138759.1 |
| GM655198.1 | HV445037.1 | GM970168.1 | GN032375.1 | HV504496.1 | CS118264.1 | AX452031.1 | AX142137.1 | DD137686.1 |
| GM655166.1 | HV444467.1 | GM969734.1 | GN032342.1 | HV504464.1 | CS118232.1 | AX451628.1 | AX142007.1 | DD137240.1 |
| GM648173.1 | HV444109.1 | GM697230.1 | GN032310.1 | HV504432.1 | CS118199.1 | AX441398.1 | AX141943.1 | DD132688.1 |
| GM641210.1 | HV438463.1 | GM644336.1 | GN032278.1 | HV504208.1 | CS118165.1 | AX443297.1 | AX141813.1 | DD132288.1 |
| GM641178.1 | HV437633.1 | GM644272.1 | GN032246.1 | HV504172.1 | CS118133.1 | AX441274.1 | AX141685.1 | DD093950.1 |
| GM641146.1 | HH773143.1 | GM644240.1 | GN032214.1 | HV504108.1 | CS118068.1 | AX429440.1 | AX141621.1 | DD106662.1 |
| GM641082.1 | HH820959.1 | GM637242.1 | GN032182.1 | HV504076.1 | CS118034.1 | AX428581.1 | AX141557.1 | DD092956.1 |
| GM628890.1 | HH820550.1 | GM637210.1 | GN032150.1 | HV504012.1 | CS117998.1 | AX427232.1 | AX141365.1 | DD105863.1 |
| GM628858.1 | HH759324.1 | GM637146.1 | GN032118.1 | HV503980.1 | CS117966.1 | AX418555.1 | AX133940.1 | DD118648.1 |
| GM628794.1 | HH759213.1 | GM637114.1 | GN032086.1 | HV503948.1 | CS114658.1 | AX411459.1 | AX127333.1 | DD061226.1 |
| GM628730.1 | HH759181.1 | GM637082.1 | GN032054.1 | HV503916.1 | CS113405.1 | AX404814.1 | AX112907.1 | DD118106.1 |
| GM618949.1 | HH759149.1 | GM624929.1 | GN032022.1 | HV503884.1 | CS111526.1 | AX403257.1 | AX108996.1 | DD090412.1 |
| GM628682.1 | HH759117.1 | GM624897.1 | GN031990.1 | HV503852.1 | CS105934.1 | AX402389.1 | AX107113.1 | DD117208.1 |
| GM628618.1 | HH759085.1 | GM624865.1 | GN031958.1 | HV503820.1 | CS102978.1 | AX398673.1 | AX100872.1 | DD116346.1 |
| GM628586.1 | HH759053.1 | GM632648.1 | GN031926.1 | HV501013.1 | CS102946.1 | AX395281.1 | AX098682.1 | DD090308.1 |
| GM628554.1 | HH759021.1 | GM632582.1 | GN031894.1 | HV491108.1 | CS102786.1 | AX391843.1 | AX097926.1 | DD089593.1 |
| GM654972.1 | HH758989.1 | GM632608.1 | GN031862.1 | HV503780.1 | CS102754.1 | AX391155.1 | AX097503.1 | DD103263.1 |
| GM647287.1 | HH758957.1 | GM741938.1 | GN031830.1 | HV503748.1 | CS102722.1 | AX384581.1 | AX093083.1 | HW321142.1 |
| GM647255.1 | HH758925.1 | GM658458.1 | GN031700.1 | HV503652.1 | CS102690.1 | AX382507.1 | AX085935.1 | HW329465.1 |
| GM647223.1 | HH758893.1 | GM658370.1 | GN031668.1 | HV503592.1 | CS102594.1 | AX378797.1 | AX080848.1 | HW329333.1 |
| GM640420.1 | HH757501.1 | GM651178.1 | GN031636.1 | HV503560.1 | CS102562.1 | AF469487.1 | AX074060.1 | HW336340.1 |
| GM627713.1 | HH757469.1 | GM651114.1 | GN031604.1 | HV503528.1 | CS102530.1 | AX364533.1 | AX061418.1 | HW328686.1 |
| GM627681.1 | HH757437.1 | GM651082.1 | GN031572.1 | HV503464.1 | CS102498.1 | AX364501.1 | AX057563.1 | HW328601.1 |
| GM627649.1 | HH756190.1 | GM651050.1 | GN031540.1 | HV503432.1 | AX287013.1 | AX364437.1 | HW363664.1 | HW328355.1 |
| GM654359.1 | HI401240.1 | GM651018.1 | GN031508.1 | HV503400.1 | AX283686.1 | AX364405.1 | HW338998.1 | HW335701.1 |
| GM627589.1 | HI401091.1 | GM650986.1 | GN031476.1 | HV503368.1 | AX282117.1 | AX364373.1 | HW338798.1 | HW335658.1 |
| GM627557.1 | HI643031.1 | GM644155.1 | GN031443.1 | HV503304.1 | AX279948.1 | AX364341.1 | HW338670.1 | HW328091.1 |
| GM627525.1 | HI642487.1 | GM644123.1 | GN031411.1 | HV503272.1 | AX279663.1 | AX364246.1 | HW338414.1 | HW318495.1 |
| GM661498.1 | HI641943.1 | GM644091.1 | GN031379.1 | HV503240.1 | AX278747.1 | AX364214.1 | HW338158.1 | HW318293.1 |
| GM661466.1 | HI641399.1 | GM644059.1 | GN031347.1 | HV503208.1 | AX278275.1 | AX364181.1 | HW337902.1 | HW335186.1 |
| GM661434.1 | HI640855.1 | GM644027.1 | GN031315.1 | HV503185.1 | AX278076.1 | AX363235.1 | HW337774.1 | HW314061.1 |
| GM661402.1 | HI380406.1 | GM636933.1 | GN031282.1 | HV503153.1 | AX256403.1 | AX358642.1 | HW337390.1 | HW313984.1 |
| GM661370.1 | HI380329.1 | GM636901.1 | GN031250.1 | HV503121.1 | AX253575.1 | AX358422.1 | HW337006.1 | HW311836.1 |

| | | | | | | | | |
|---|---|---|---|---|---|---|---|---|
| GM661338.1 | HI379301.1 | GM636869.1 | GN031186.1 | HV503057.1 | AX247885.1 | BD006783.1 | HW336878.1 | HW311250.1 |
| GM654344.1 | HI379116.1 | GM623530.1 | GN031153.1 | HV503025.1 | HV455587.1 | E50936.1 | HW336722.1 | HW311137.1 |
| GM654280.1 | HD113174.1 | GM623498.1 | GN031121.1 | HV502993.1 | HV504167.1 | E63240.1 | HW329487.1 | HW309705.1 |
| GM654248.1 | HD082435.1 | GM623466.1 | GN031089.1 | HV502961.1 | HV504135.1 | BD001998.1 | HW329403.1 | HW309418.1 |
| GM647191.1 | HD082301.1 | GM623434.1 | GN031057.1 | HV502929.1 | HV504103.1 | E54557.1 | HW329275.1 | HW308083.1 |
| GM647159.1 | HD088252.1 | GM623402.1 | GN031025.1 | HV502897.1 | HV504039.1 | E55082.1 | HW328838.1 | HW307870.1 |
| GM647127.1 | DL030011.1 | GM623370.1 | GN030993.1 | HV502865.1 | HV504007.1 | E58966.1 | HW328545.1 | HW307806.1 |
| GM647095.1 | DL029979.1 | GM643889.1 | GN030898.1 | HV502833.1 | HV503975.1 | AX356506.1 | HW328423.1 | HW316586.1 |
| GM647055.1 | DL046636.1 | GM643857.1 | GN030866.1 | HV502789.1 | HV503943.1 | AX354680.1 | HW328263.1 | HW307736.1 |
| GM647002.1 | DL046604.1 | GM643825.1 | GN030834.1 | HV502757.1 | HV503911.1 | AX353939.1 | HW328136.1 | HW307704.1 |
| GM627489.1 | DL046572.1 | GM636828.1 | GN030802.1 | HV502725.1 | HV503879.1 | AX352741.1 | HW239292.1 | HW315961.1 |
| GM627457.1 | DL046540.1 | GM636796.1 | GN030770.1 | HV502693.1 | HV503847.1 | AX352340.1 | HW238608.1 | HW315564.1 |
| GM627425.1 | DL042654.1 | GM636764.1 | DM008095.1 | HV502661.1 | HV503815.1 | HW364617.1 | HW238388.1 | HW315171.1 |
| GM627393.1 | DL042622.1 | GM632260.1 | DM001910.1 | HV502629.1 | HV491103.1 | HW373285.1 | HW238250.1 | HW315075.1 |
| GM627361.1 | DL042590.1 | GM632228.1 | DM001842.1 | HV502565.1 | HV503775.1 | HW353717.1 | HW244310.1 | HV985938.1 |
| GM627329.1 | DL042558.1 | GM623329.1 | DM001685.1 | HV502533.1 | HV503743.1 | HW352652.1 | HW237924.1 | HV985906.1 |
| GM661263.1 | DL038666.1 | GM623297.1 | DM001652.1 | HV502501.1 | HV503711.1 | HW351216.1 | HW237879.1 | HV985861.1 |
| GM661231.1 | DL038634.1 | GM623265.1 | DM001481.1 | HV502469.1 | HV503679.1 | HW350682.1 | HW248662.1 | HW028884.1 |
| GM661199.1 | DL038506.1 | GM623233.1 | DM001391.1 | HV497321.1 | HV503647.1 | HW350648.1 | HW237523.1 | HV984268.1 |
| GM654141.1 | DL034499.1 | GM741839.1 | DM007033.1 | HI653224.1 | HV503523.1 | HW350520.1 | HW237036.1 | HV984232.1 |
| GM654109.1 | DL029908.1 | GM741807.1 | DM006883.1 | HI652910.1 | HV503491.1 | HW350254.1 | HW241618.1 | HV986633.1 |
| GM654077.1 | DL029876.1 | GM658321.1 | DM000911.1 | HI647558.1 | HV503459.1 | HW349958.1 | HW243070.1 | HV961493.1 |
| GM654045.1 | DL029844.1 | GM658289.1 | DM006547.1 | HI646891.1 | HV503427.1 | HV956407.1 | HW241509.1 | HV969524.1 |
| GM654013.1 | DL029780.1 | GM658257.1 | DM005080.1 | HI646229.1 | HV503395.1 | HV956152.1 | HW241412.1 | HV960528.1 |
| GM653981.1 | DL026357.1 | GM658225.1 | DM005045.1 | HH807336.1 | HV503363.1 | HV951079.1 | HW161295.1 | HV960205.1 |
| GM646988.1 | DL018381.1 | GM658193.1 | DM004975.1 | HH807045.1 | HV503331.1 | HV957524.1 | HW161083.1 | HV960146.1 |
| GM646956.1 | DL018349.1 | GM658161.1 | DM003554.1 | HH796566.1 | HV503299.1 | HV951770.1 | HW160400.1 | HV964030.1 |
| GM646892.1 | DL018317.1 | FB754274.1 | DM003508.1 | HH794954.1 | HV503267.1 | HV780578.1 | HW155789.1 | HV963864.1 |
| GM646860.1 | DL018285.1 | FB753873.1 | DM002697.1 | HH793035.1 | HV455370.1 | HV779883.1 | HW155768.1 | HV966041.1 |
| GM646828.1 | DL018253.1 | FB753529.1 | DM002605.1 | HH779680.1 | HV503180.1 | HV778421.1 | HW155702.1 | HV963606.1 |
| GM640025.1 | DL018221.1 | FB727276.1 | DM010301.1 | HH777923.1 | HV503116.1 | HV774723.1 | HW155670.1 | HV958119.1 |
| GM639993.1 | DL013819.1 | FB726028.1 | DM008705.1 | HH774438.1 | HV503020.1 | HV773792.1 | HW155555.1 | HV966015.1 |
| GM639961.1 | DL013787.1 | FB725956.1 | GM998436.1 | HH820961.1 | HV502956.1 | HV700896.1 | HV936740.1 | HV965983.1 |
| GM639929.1 | DL013755.1 | GM864108.1 | GM997939.1 | HH820919.1 | HV502924.1 | HW302697.1 | HV925515.1 | HV969963.1 |
| GM639897.1 | DL013723.1 | GM842455.1 | GM996597.1 | HH820551.1 | HV502892.1 | HW286518.1 | HV819454.1 | HV962268.1 |
| GM639865.1 | DL013691.1 | GM616332.1 | GM996501.1 | HH759214.1 | HV502860.1 | HW302540.1 | HV929982.1 | HV965367.1 |
| GM627318.1 | DL000628.1 | GM879668.1 | GM996251.1 | HH759182.1 | HV502828.1 | HW295581.1 | HV802929.1 | HV950469.1 |
| GM627291.1 | DL005848.1 | GM731759.1 | GM980125.1 | HH759150.1 | HV502784.1 | HW307424.1 | HV802897.1 | HV949002.1 |
| GM627259.1 | DJ493782.1 | FB711258.1 | GN009671.1 | HH759118.1 | HV502752.1 | HW294145.1 | HV781105.1 | HV947289.1 |
| GM627227.1 | DJ491887.1 | GM840411.1 | GM994902.1 | HH759086.1 | HV502720.1 | HW294046.1 | HV784689.1 | HV953244.1 |
| GM627195.1 | DJ491592.1 | GM041777.1 | GM994610.1 | HH759054.1 | HV502688.1 | HW293159.1 | HV778940.1 | HV704194.1 |
| GM627163.1 | DL002525.1 | GM040851.1 | GM978595.1 | HH759022.1 | HV502656.1 | HW293030.1 | HV778381.1 | HV704118.1 |
| GM627131.1 | DJ446859.1 | GM840218.1 | GM978381.1 | HH758990.1 | HV502624.1 | HW292753.1 | HV777982.1 | HV744590.1 |
| GM661115.1 | DJ446826.1 | GM721206.1 | GM977880.1 | HH758958.1 | HV502560.1 | HW291292.1 | HV776125.1 | HV703499.1 |
| GM661083.1 | DJ446685.1 | GM040291.1 | GM992647.1 | HH758926.1 | HV502528.1 | HW291169.1 | HV694663.1 | HV702439.1 |
| DL087620.1 | EU529743.1 | CS728680.1 | GM992498.1 | HH758894.1 | HV502464.1 | HW291072.1 | HV693721.1 | HV702351.1 |
| DL087588.1 | DJ438099.1 | CS728648.1 | GM992278.1 | HH757470.1 | HV505311.1 | HW291040.1 | HV693335.1 | HV702259.1 |

| | | | | | | | | |
|---|---|---|---|---|---|---|---|---|
| DL125519.1 | DJ437322.1 | CS727328.1 | GM992175.1 | HH757438.1 | HV505203.1 | HW291008.1 | HV698251.1 | HV701868.1 |
| DL125487.1 | DJ437274.1 | GM887755.1 | GM992050.1 | HH756191.1 | HV494722.1 | HW290976.1 | HV698197.1 | HV701215.1 |
| DL125455.1 | DJ437235.1 | FB753597.1 | GM991919.1 | HI071597.1 | HV452179.1 | HW290944.1 | HV689457.1 | HV701183.1 |
| DL125423.1 | DJ437176.1 | FB728439.1 | GM991763.1 | HI464732.1 | HV453655.1 | HW290912.1 | HV695879.1 | HV701151.1 |
| DL125391.1 | DJ437146.1 | GM680731.1 | GM976309.1 | HI464700.1 | HV451950.1 | HW298791.1 | HV585110.1 | HV701119.1 |
| DL125359.1 | CS482894.1 | GM618716.1 | GM975136.1 | HI464668.1 | HV449814.1 | HW290881.1 | HV592439.1 | HV708717.1 |
| DL121570.1 | DD401439.1 | FB709030.1 | GM990791.1 | HI464636.1 | HV452994.1 | HW290849.1 | HV592375.1 | HV701068.1 |
| DL121538.1 | DD401407.1 | GM060269.1 | GM989810.1 | HI464425.1 | HV452879.1 | HW298196.1 | HV580625.1 | HV701036.1 |
| DL121506.1 | DD401184.1 | GM841757.1 | GM989190.1 | HI542549.1 | HV448953.1 | HW289025.1 | HV585556.1 | HV695530.1 |
| DL121474.1 | DD405190.1 | GM841721.1 | GM986846.1 | HI462737.1 | HV450924.1 | HW279585.1 | HV570625.1 | HV695434.1 |
| DL121442.1 | DD405158.1 | GM604047.1 | GM999536.1 | HI503862.1 | HV444624.1 | HV960527.1 | HV503834.1 | HV694921.1 |
| DL121410.1 | DD405126.1 | GM603714.1 | GM969675.1 | HH961355.1 | HI066256.1 | HV960204.1 | HV502803.1 | HV694846.1 |
| DL117198.1 | DD405094.1 | GM706715.1 | GM969403.1 | HH961323.1 | HI180163.1 | HV960145.1 | HV502771.1 | HV694811.1 |
| DL117166.1 | DD405062.1 | GM706683.1 | GM632109.1 | HH961291.1 | HI202835.1 | HV964000.1 | HV502739.1 | HV694769.1 |
| DL117134.1 | DD405030.1 | GM706651.1 | GM623338.1 | HH961259.1 | HI214563.1 | HV963863.1 | HV502707.1 | HV700732.1 |
| DL117102.1 | DD406925.1 | GM706613.1 | GM623306.1 | HH964842.1 | HI214531.1 | HH961351.1 | HV502643.1 | HV700700.1 |
| DL117070.1 | DD410097.1 | GM706386.1 | GM623274.1 | HH975392.1 | HI214433.1 | HH961319.1 | HV502611.1 | HV694680.1 |
| DL117038.1 | DD402405.1 | GM706352.1 | GM623242.1 | HH975090.1 | HI213002.1 | HH961287.1 | HV502579.1 | HV694643.1 |
| DL112450.1 | DD402373.1 | GM603498.1 | GM623210.1 | HH964367.1 | HI203501.1 | HH961255.1 | HV502547.1 | HV700216.1 |
| DL112418.1 | DD402341.1 | GM704813.1 | GM623178.1 | HH982251.1 | HI546318.1 | HH961137.1 | HV502515.1 | HV699502.1 |
| DL112386.1 | DD402309.1 | HV347335.1 | GM623146.1 | HH974550.1 | HI546215.1 | HH975200.1 | HV450362.1 | HV699156.1 |
| DL112354.1 | DD402277.1 | HV347034.1 | GM741848.1 | HH981892.1 | HI210996.1 | HH964363.1 | HV453324.1 | HV699083.1 |
| DL112322.1 | DD402245.1 | HV344704.1 | GM741816.1 | HH998077.1 | HI210964.1 | HH982236.1 | HV453150.1 | HV690118.1 |
| DL112290.1 | DD402213.1 | AY145517.1 | GM658330.1 | HH998024.1 | HI210932.1 | HH974648.1 | HV451238.1 | HV698610.1 |
| DL128667.1 | DD402181.1 | HH804680.1 | GM658298.1 | HH997966.1 | HI565888.1 | HH974546.1 | HV448766.1 | HV695943.1 |
| DL128601.1 | DD402149.1 | HH797417.1 | GM658234.1 | HH999662.1 | HI473009.1 | HH981883.1 | HV445107.1 | HV688797.1 |
| DL128548.1 | DD402085.1 | HH794939.1 | GM658202.1 | HH997926.1 | HI472905.1 | HH998020.1 | HV444450.1 | HV600529.1 |
| DL125071.1 | DD405773.1 | HH794707.1 | GM658170.1 | HH997842.1 | HI635493.1 | HH997960.1 | HV444138.1 | HV585531.1 |
| DL125039.1 | DD405741.1 | HH793104.1 | GM650978.1 | HH997780.1 | HI004369.1 | HH999658.1 | HV444032.1 | HV579747.1 |
| DL125007.1 | DD405709.1 | HH792572.1 | GM650946.1 | HH978406.1 | HI004241.1 | HH997909.1 | FW505244.1 | HV585094.1 |
| DL124975.1 | DD405677.1 | HH779963.1 | GM650914.1 | HH999464.1 | HI002274.1 | HH997838.1 | FW555360.1 | HV588905.1 |
| DL120886.1 | DD405645.1 | HH779665.1 | GM650882.1 | HH999421.1 | HI002237.1 | HH997775.1 | FW553144.1 | HV584908.1 |
| DL120854.1 | DD405593.1 | HH774423.1 | CS367708.1 | HH999371.1 | HI002187.1 | HH999512.1 | FW556588.1 | HV584810.1 |
| DL120822.1 | DD405497.1 | HH820984.1 | CS367446.1 | HH997659.1 | HI002144.1 | HH999460.1 | EU363768.1 | HI933671.1 |
| DL124928.1 | DD405465.1 | HI401111.1 | CS367344.1 | HH997623.1 | HI002106.1 | HH999417.1 | HI930281.1 | HI930644.1 |
| DL124896.1 | DD405433.1 | HI464791.1 | DL081050.1 | HH997566.1 | HI000451.1 | HH999367.1 | HI935083.1 | HI929588.1 |
| DL124864.1 | DD401995.1 | HI464717.1 | DL081018.1 | HH997491.1 | HI000403.1 | HH997653.1 | HI918283.1 | HI935105.1 |
| DL124832.1 | DD401963.1 | HI464685.1 | DL080986.1 | HH997352.1 | HI553289.1 | HH997619.1 | HI918251.1 | HW243053.1 |
| DL124800.1 | DD401902.1 | HI464653.1 | DL080954.1 | HH999265.1 | HI553249.1 | HH997553.1 | HI924540.1 | HW241473.1 |
| DL124768.1 | DD401870.1 | HI464596.1 | DL048329.1 | HH999209.1 | HI553217.1 | HH999701.1 | HI658613.1 | HW163855.1 |
| DL120775.1 | DD401838.1 | HI464457.1 | DL044443.1 | HH999163.1 | HI553185.1 | HH999250.1 | FW495950.1 | HW160675.1 |
| DL120743.1 | DD401806.1 | HI549608.1 | DL044411.1 | HH998127.1 | HI000301.1 | HH999205.1 | HI538381.1 | HW160627.1 |
| DL116212.1 | DD409244.1 | HI463141.1 | DL044379.1 | HH999085.1 | HI521199.1 | HH999156.1 | HI559237.1 | HW160595.1 |
| DL116180.1 | DD401630.1 | HI462677.1 | DL044315.1 | HH999044.1 | HI539083.1 | HH998123.1 | HI564735.1 | HW155903.1 |
| DL116148.1 | DD401598.1 | HH725201.1 | DL044283.1 | HH998987.1 | HI583969.1 | HH999080.1 | HI564235.1 | HW155685.1 |
| DL116116.1 | DD401566.1 | HH714036.1 | DL040267.1 | HH998933.1 | HI568962.1 | HH999039.1 | HI564111.1 | HW155627.1 |
| DL116052.1 | DD401534.1 | HH961372.1 | DL040235.1 | HH997211.1 | HI207664.1 | HH998974.1 | HI583099.1 | HV573887.1 |

| | | | | | | | | |
|---|---|---|---|---|---|---|---|---|
| DL143516.1 | DD401502.1 | HH961340.1 | DL040203.1 | HH997149.1 | HI003697.1 | HH998929.1 | HI563636.1 | HV572400.1 |
| DL120576.1 | DD401470.1 | HH961308.1 | DL040171.1 | HH998886.1 | HI002069.1 | HH997191.1 | HI563604.1 | HV560151.1 |
| DL120544.1 | CS480599.1 | HH961276.1 | DL040139.1 | HH998826.1 | HI002021.1 | HH997145.1 | HI563569.1 | HV555950.1 |
| DL116013.1 | CS479227.1 | HH961244.1 | DL040107.1 | HH998783.1 | HI001938.1 | HH998882.1 | HI557538.1 | HV553660.1 |
| DL115981.1 | CS476463.1 | HH964272.1 | DL032114.1 | HH997102.1 | HI000270.1 | HH998822.1 | HI544254.1 | HV552826.1 |
| DL115917.1 | CS469007.1 | HH974535.1 | DL032082.1 | HH997069.1 | HI000226.1 | HH998760.1 | HI000026.1 | HV551419.1 |
| DL115885.1 | CS467731.1 | HH998104.1 | DL032050.1 | HH997010.1 | HI000176.1 | HH997098.1 | HI001757.1 | HV550404.1 |
| DL115853.1 | CS467569.1 | HH997997.1 | DL032018.1 | HH996957.1 | HW041830.1 | HH997006.1 | HI001723.1 | HV550355.1 |
| DL106495.1 | CS464828.1 | HH997938.1 | DL031986.1 | HH998713.1 | HW049414.1 | HH996952.1 | HI469534.1 | AY659405.1 |
| DL106463.1 | CS464549.1 | HH999647.1 | DL031954.1 | HH998674.1 | HW049382.1 | HH998707.1 | HI544122.1 | AY659373.1 |
| DL106357.1 | DD044205.1 | HH997898.1 | DL028145.1 | HH998604.1 | HW049319.1 | HH998670.1 | HI575295.1 | AY659341.1 |
| DL106303.1 | DD038488.1 | HH997819.1 | DL028113.1 | HH996928.1 | HW049287.1 | HH998600.1 | HI001639.1 | AY659309.1 |
| DL128477.1 | HC057877.1 | HH997761.1 | DL028081.1 | HH996773.1 | HW049255.1 | HH998546.1 | HI001599.1 | AY659277.1 |
| DL128442.1 | HC057170.1 | HH999495.1 | DL028049.1 | HH994566.1 | HW043281.1 | HH996924.1 | HI001556.1 | AY659245.1 |
| DL124748.1 | DM459788.1 | HH999442.1 | DL028017.1 | HH980988.1 | HW041794.1 | HH996823.1 | HI001503.1 | AY659213.1 |
| DL124716.1 | DM382808.1 | HH997640.1 | DL027985.1 | HH954655.1 | HW041762.1 | HH996768.1 | HI003378.1 | AY659181.1 |
| DL124684.1 | DM381534.1 | HH997584.1 | DL027953.1 | HH980573.1 | HW050627.1 | HH977639.1 | HI003340.1 | AY659149.1 |
| DL124652.1 | HC053875.1 | HH997520.1 | DL036463.1 | HH980502.1 | HV302941.1 | HH962748.1 | HI003301.1 | AY659117.1 |
| DL124620.1 | HC051936.1 | HH997372.1 | DL036431.1 | HH980434.1 | HV308789.1 | HC290168.1 | HI464746.1 | AY659085.1 |
| DL124588.1 | HC049743.1 | HH999689.1 | DL036399.1 | HH957937.1 | HV308532.1 | HC290136.1 | HI464714.1 | AY659053.1 |
| DL124556.1 | HC045500.1 | HH999235.1 | DL036367.1 | HH980361.1 | HV304168.1 | HC289877.1 | HI464682.1 | AY659021.1 |
| DL096923.1 | HC045468.1 | HH999194.1 | DL036335.1 | HH980310.1 | HV311085.1 | HC289843.1 | HI464650.1 | AY658989.1 |
| DL096891.1 | HC045436.1 | HH999145.1 | DL044250.1 | HH980226.1 | HV040127.1 | HC207906.1 | HI550140.1 | AY658957.1 |
| DL096859.1 | HC045404.1 | HH999114.1 | DL044218.1 | HH980162.1 | HV188522.1 | HC207654.1 | HI464454.1 | AY658925.1 |
| DL096795.1 | HC045372.1 | HH999068.1 | DL044186.1 | HC202638.1 | HV200507.1 | HC199291.1 | HI505026.1 | AY658893.1 |
| DL096763.1 | HC045340.1 | HH999024.1 | DL044154.1 | HC201311.1 | HV182488.1 | DM464956.1 | HI504981.1 | AY658861.1 |
| DL094839.1 | HC045276.1 | HH998957.1 | DL044122.1 | HC199335.1 | HV182456.1 | DM463602.1 | HI463138.1 | AY658829.1 |
| DL094775.1 | DM078965.1 | HH997290.1 | DL044090.1 | HC206744.1 | HV182424.1 | HC196124.1 | HI462585.1 | AY658797.1 |
| DL091160.1 | DM078731.1 | HH997169.1 | DL071613.1 | HC196128.1 | HV182392.1 | HC195231.1 | HH714033.1 | AY658765.1 |
| DL091128.1 | GN087796.1 | HH978181.1 | DL048227.1 | HC193965.1 | HV221010.1 | HC193945.1 | HH961369.1 | AY658733.1 |
| DL091096.1 | GN090936.1 | HH998909.1 | DL048195.1 | HC187071.1 | HV182322.1 | HC190739.1 | HH961337.1 | AY658701.1 |
| DL110325.1 | GN087194.1 | HH998855.1 | DL048163.1 | HC089959.1 | HV182290.1 | HC187067.1 | HH961305.1 | AY658669.1 |
| DL110293.1 | GN089856.1 | HH998800.1 | DL048131.1 | HC089555.1 | HV234189.1 | HC089583.1 | HH961273.1 | AY658637.1 |
| DL105488.1 | GN089792.1 | HH998741.1 | DL048099.1 | HC089523.1 | FW591893.1 | HC089551.1 | HH961241.1 | AY658605.1 |
| DL105456.1 | GN082891.1 | HH997086.1 | DL048067.1 | HC089491.1 | FW590728.1 | HC089519.1 | HH964264.1 | AY658573.1 |
| DL105424.1 | GN088510.1 | HH997032.1 | DL048036.1 | HC089459.1 | FW589161.1 | HC089487.1 | HH974590.1 | AY658541.1 |
| DL105392.1 | DL232984.1 | HH996982.1 | DL048004.1 | HC089427.1 | FW593445.1 | HC089455.1 | HH974532.1 | AY658509.1 |
| DL105360.1 | GN077118.1 | HH981128.1 | DL047972.1 | HC089394.1 | FW586438.1 | HC089423.1 | HH998101.1 | AY658477.1 |
| DL105328.1 | L08946.1 | HH998696.1 | DL047940.1 | HC089362.1 | FW592631.1 | HC089390.1 | HH998048.1 | AY658445.1 |
| DL124347.1 | L08871.1 | HH998655.1 | DL047908.1 | HC089329.1 | FW577821.1 | HC089358.1 | HH997994.1 | AY658413.1 |
| DL124315.1 | L09135.1 | HH998583.1 | DL047876.1 | HC089297.1 | FW576789.1 | HC089325.1 | HH997935.1 | AY658381.1 |
| DL124251.1 | DM059618.1 | HH998527.1 | DL043990.1 | HC089265.1 | FW576754.1 | HC089293.1 | HH999588.1 | AY658349.1 |
| DL110176.1 | DM058842.1 | HH996913.1 | DL043958.1 | HC087843.1 | FW576720.1 | HC089261.1 | HH997895.1 | AY658317.1 |
| DL110144.1 | DM045399.1 | HH996794.1 | DL043926.1 | HC086269.1 | FW576645.1 | HC086185.1 | HH997807.1 | AY658285.1 |
| DL110112.1 | DM045242.1 | HH996757.1 | DL043894.1 | HC085669.1 | FW576336.1 | HC085661.1 | AY774654.1 | AY658253.1 |
| DL110080.1 | DM044945.1 | HH994539.1 | DL040077.1 | AY775014.1 | FW575881.1 | AY775008.1 | AY774596.1 | HV117846.1 |
| DL105179.1 | GM752445.1 | HH980741.1 | DL040045.1 | DL120186.1 | FW575357.1 | AY774909.1 | AY774532.1 | HV117789.1 |

| | | | | | | | | |
|---|---|---|---|---|---|---|---|---|
| DL105147.1 | DL480731.1 | HH977455.1 | DL040013.1 | DL120154.1 | FW573832.1 | AY774853.1 | AY774490.1 | HV117477.1 |
| DL105115.1 | DL467566.1 | HH977345.1 | DL039981.1 | DL120122.1 | FW568959.1 | AY774683.1 | AY774430.1 | HV112795.1 |
| DL100669.1 | GM712949.1 | HH980623.1 | DL039949.1 | DL120090.1 | FW571844.1 | AY774623.1 | AY774366.1 | HV112557.1 |
| DL100637.1 | GM652751.1 | HH980523.1 | DL039917.1 | DL120058.1 | FW571750.1 | AY774558.1 | AY774315.1 | HV038657.1 |
| DL043210.1 | GM652719.1 | HH980452.1 | DL039885.1 | DL120026.1 | HI968133.1 | AY774508.1 | AY774268.1 | HV038597.1 |
| DL043178.1 | GM652687.1 | HH980379.1 | DL036229.1 | DL094344.1 | HI635479.1 | AY774456.1 | AY774211.1 | HV182325.1 |
| DL043146.1 | GM652655.1 | HH980346.1 | DL036197.1 | DL094312.1 | HI004367.1 | AY774395.1 | AY366933.1 | HV182293.1 |
| DL043114.1 | GM652623.1 | HH980286.1 | DL036165.1 | DL094248.1 | HI004239.1 | AY774288.1 | AF305422.1 | HV193543.1 |
| DL043082.1 | GM652591.1 | HH980184.1 | DL036133.1 | DL094216.1 | HI002273.1 | AY774234.1 | HC084790.1 | HV182359.1 |
| DL039265.1 | GM645497.1 | HH980146.1 | DL036101.1 | DL094184.1 | HI002236.1 | AY774119.1 | HC083715.1 | HV234612.1 |
| DL039233.1 | GM645465.1 | HH980094.1 | DL071192.1 | DL094152.1 | HI002186.1 | AY774047.1 | HC083538.1 | HV222957.1 |
| DL039201.1 | GM645433.1 | HH996693.1 | DL047832.1 | DL090469.1 | HI002143.1 | AF284213.1 | DM459950.1 | HV312973.1 |
| DL039169.1 | GM638540.1 | HH996632.1 | DL047800.1 | DL090437.1 | HI002105.1 | HC070453.1 | DM381896.1 | HV302108.1 |
| DL039137.1 | GM638508.1 | GN116486.1 | DL047768.1 | DL090405.1 | HI000450.1 | HC070103.1 | HC055545.1 | HV031121.1 |
| DL039105.1 | GM638476.1 | GN116453.1 | DL047736.1 | DL090373.1 | HI000402.1 | HC083729.1 | HC053255.1 | HV030247.1 |
| DL035449.1 | GM638444.1 | GN116421.1 | DL047704.1 | DL086368.1 | HI553288.1 | HC083553.1 | HC051855.1 | HV037882.1 |
| DL035385.1 | GM633745.1 | GN116389.1 | DL047672.1 | DL086336.1 | HI553216.1 | HC083520.1 | HC045303.1 | HV037706.1 |
| DL035353.1 | GM633713.1 | DM075117.1 | DL043850.1 | DL086304.1 | HI553184.1 | HC069867.1 | HA641580.1 | HV037674.1 |
| DL035321.1 | GM633681.1 | DM073117.1 | DL043818.1 | DL086272.1 | HI546055.1 | HC081526.1 | HA641179.1 | HV037238.1 |
| DL031317.1 | GM625814.1 | DM064159.1 | DL043786.1 | DL088607.1 | HI539523.1 | AF301133.1 | HA641113.1 | HV036157.1 |
| DL031285.1 | GM625782.1 | DM077788.1 | DL043754.1 | DL098470.1 | HI583968.1 | HC059760.1 | HA637681.1 | HV035378.1 |
| DL010302.1 | GM625750.1 | DM077435.1 | DL043722.1 | DL092332.1 | HI568961.1 | HH979920.1 | HA643897.1 | HV031610.1 |
| DL010270.1 | GM624726.1 | GN089986.1 | DL043690.1 | DL092300.1 | HI207662.1 | HH979879.1 | DM102618.1 | FZ436000.1 |
| DL010238.1 | GM624662.1 | GN089836.1 | DL039873.1 | DL092268.1 | HI003907.1 | HH996705.1 | GN368276.1 | FZ435968.1 |
| DL010206.1 | GM624630.1 | GN082913.1 | DL039841.1 | DL092236.1 | HI003696.1 | HH996642.1 | GN356192.1 | FZ435803.1 |
| DL034671.1 | GM638393.1 | GN082810.1 | DL039809.1 | DL092204.1 | HI002064.1 | HH996594.1 | GN360070.1 | FZ430282.1 |
| DL034639.1 | GM638361.1 | GN088026.1 | DL039777.1 | DL092172.1 | HI002020.1 | HH996546.1 | GN360038.1 | FZ429609.1 |
| DL030387.1 | GM659898.1 | GN000754.1 | DL039745.1 | DL088557.1 | HI001937.1 | HH998403.1 | GN359974.1 | FZ437386.1 |
| DL030347.1 | GM659866.1 | GM969686.1 | DL039713.1 | DL088525.1 | HI000269.1 | HH998364.1 | GN359942.1 | FZ423913.1 |
| DL026524.1 | GM656533.1 | GM968966.1 | DL036089.1 | DL088493.1 | HI000224.1 | HH999996.1 | GN359801.1 | FZ423867.1 |
| DL026530.1 | GM656501.1 | GM983622.1 | DL036057.1 | DL088461.1 | HI000173.1 | HH999942.1 | GN359769.1 | FZ432336.1 |
| DL026474.1 | GM656469.1 | GM984657.1 | DL036025.1 | DL088429.1 | HI000129.1 | HH979818.1 | GN013584.1 | FZ423317.1 |
| DL026378.1 | GM656437.1 | FB740444.1 | DL035993.1 | DL088397.1 | HI470722.1 | HH979712.1 | GN033534.1 | FZ419477.1 |
| DL022566.1 | GM649340.1 | GM596701.1 | DL035961.1 | DL117797.1 | HI470598.1 | HH979668.1 | GN033502.1 | FW378147.1 |
| DL022534.1 | GM649252.1 | GM009573.1 | DL035929.1 | DL117765.1 | HI544526.1 | HH967987.1 | GN033470.1 | FW377593.1 |
| DL022462.1 | GM649220.1 | DL477724.1 | DL035897.1 | DL117733.1 | HI560311.1 | HH999865.1 | GN033438.1 | FW377093.1 |
| DL022494.1 | GM642579.1 | DL464628.1 | DL031893.1 | DL117701.1 | HI564702.1 | HH999776.1 | GN033374.1 | FW376182.1 |
| DL022438.1 | GM642547.1 | DL464540.1 | DL031861.1 | DL117669.1 | HI568851.1 | HH999722.1 | GN033342.1 | FW381510.1 |
| DL022406.1 | GM642469.1 | DL463060.1 | CS603114.1 | DL117637.1 | HI564330.1 | HH998278.1 | GN033310.1 | FW381329.1 |
| DL019199.1 | GM635204.1 | DL462998.1 | CS603082.1 | DL122096.1 | HI564156.1 | HH998241.1 | GN033246.1 | FW381113.1 |
| DL014653.1 | GM635172.1 | DL462898.1 | CS603050.1 | DL125741.1 | HI563616.1 | HH998193.1 | GN033118.1 | FW381001.1 |
| DL010146.1 | GM635140.1 | DL461715.1 | CS603018.1 | DL125709.1 | HI563581.1 | HH998152.1 | GN033054.1 | FW375480.1 |
| DL010114.1 | GM635108.1 | DL475551.1 | CS602984.1 | DL125677.1 | HI558044.1 | HH993741.1 | GN032990.1 | FW369301.1 |
| DL010082.1 | GM635076.1 | DL481566.1 | CS602952.1 | DL125613.1 | HI575626.1 | HH979527.1 | GN032958.1 | FU262846.1 |
| DL010050.1 | GM630270.1 | FB504560.1 | CS602920.1 | DL125581.1 | HI551332.1 | HH979631.1 | GN032926.1 | FU259876.1 |
| DL010018.1 | GM630238.1 | FB509200.1 | CS602888.1 | DL125549.1 | HI544140.1 | HH979590.1 | GN032894.1 | FU265373.1 |
| DL014616.1 | GM630206.1 | GM630382.1 | CS602856.1 | DL121957.1 | HI508401.1 | HC007676.1 | GN032862.1 | FU250455.1 |

| | | | | | | | | |
|---|---|---|---|---|---|---|---|---|
| DL014584.1 | GM698596.1 | GM656523.1 | CS602824.1 | DL121925.1 | HI508235.1 | HC007584.1 | GN032830.1 | FU262458.1 |
| DL014552.1 | GM635041.1 | GM656491.1 | CS602792.1 | DL121893.1 | HI000040.1 | HC007552.1 | GN032798.1 | FU264999.1 |
| DL014520.1 | GM635009.1 | GM656459.1 | CS602760.1 | DL121861.1 | HI003677.1 | HC007520.1 | GN032766.1 | FU264507.1 |
| DL014456.1 | GM634977.1 | GM656427.1 | CS602728.1 | DL121829.1 | HI003641.1 | HC010685.1 | GN032735.1 | FU258490.1 |
| DL009998.1 | GM634945.1 | GM649274.1 | CS602696.1 | DL121797.1 | HI003590.1 | DM375845.1 | GN032703.1 | FU258424.1 |
| DL009966.1 | GM634913.1 | GM649242.1 | CS602664.1 | DL117534.1 | HI003555.1 | DM375473.1 | GN032671.1 | FU258392.1 |
| DL009934.1 | GM634881.1 | GM649210.1 | CS602600.1 | DL117502.1 | HI003503.1 | DM371130.1 | GN032638.1 | HC313122.1 |
| DL009902.1 | GM629947.1 | GM635130.1 | CS602472.1 | DL117470.1 | HI001833.1 | DM370660.1 | GN032606.1 | HC313054.1 |
| DL009870.1 | GM629915.1 | GM635098.1 | CS611212.1 | DL117438.1 | HC442324.1 | DM370628.1 | GN032574.1 | DM475778.1 |
| DL009838.1 | GM642265.1 | GM635066.1 | CS610145.1 | DL112759.1 | HC452153.1 | DM370596.1 | GN032543.1 | HC299782.1 |
| DL009806.1 | GM656332.1 | GM635063.1 | CS608201.1 | DL112727.1 | HC450061.1 | DM370564.1 | GN032511.1 | HC306246.1 |
| DL009774.1 | GM656300.1 | GM634967.1 | CS607929.1 | DL112695.1 | HC448956.1 | DM370532.1 | GN032479.1 | HC306089.1 |
| DL009742.1 | GM656268.1 | GM656322.1 | CS607764.1 | DL112663.1 | FU761012.1 | DM370500.1 | GN032447.1 | HC306049.1 |
| DL009710.1 | GM656204.1 | GM656290.1 | CS607540.1 | DL107762.1 | DL107962.1 | DM370468.1 | GN032415.1 | HC306009.1 |
| DL009678.1 | GM656172.1 | GM656258.1 | CS600681.1 | DL107730.1 | DL107930.1 | DM205459.1 | GN032383.1 | HC305969.1 |
| DL009646.1 | GM649179.1 | GM656226.1 | CS600280.1 | DL098321.1 | DL107898.1 | DM371730.1 | GN032350.1 | HC301630.1 |
| DL009614.1 | GM649147.1 | GM656194.1 | CS607102.1 | DL098289.1 | DL088601.1 | DM209377.1 | GN032318.1 | HC251731.1 |
| DL009582.1 | GM649115.1 | GM656162.1 | CS607020.1 | DL098257.1 | DL112915.1 | HC003095.1 | GN032254.1 | HC251389.1 |
| DL009550.1 | HW335139.1 | DL102486.1 | CS606806.1 | DL098225.1 | DL092326.1 | HB865050.1 | GN032222.1 | HC294074.1 |
| DL009518.1 | HW314197.1 | DL102454.1 | CS605120.1 | DL098193.1 | DL092294.1 | HB865018.1 | GN032190.1 | HC289413.1 |
| DL009486.1 | HW314029.1 | DL102422.1 | CS605088.1 | DL092151.1 | DL092262.1 | HB865010.1 | GN032158.1 | HC289347.1 |
| DL009454.1 | HW313763.1 | DL095398.1 | CS605056.1 | DL092119.1 | DL092230.1 | HB864954.1 | GN032126.1 | HC289315.1 |
| DL009422.1 | HW312172.1 | DL095366.1 | CS605029.1 | DL092087.1 | DL092198.1 | HB864922.1 | GN032094.1 | HC289084.1 |
| DL030147.1 | HW312122.1 | DL125344.1 | CS604997.1 | DL092023.1 | DL092166.1 | HB864890.1 | GN032062.1 | HC296935.1 |
| DL026338.1 | HW307961.1 | DL106954.1 | CS604933.1 | DL091991.1 | DL088487.1 | HB864858.1 | GN032030.1 | HC296011.1 |
| DL026274.1 | HW307866.1 | DL106922.1 | CS604901.1 | DL098733.1 | DL088455.1 | HB864826.1 | GN031998.1 | HC288906.1 |
| DL026242.1 | HW307834.1 | DL106886.1 | CS604869.1 | FB299252.1 | DL088423.1 | HB864794.1 | GN031966.1 | HC288869.1 |
| DL026210.1 | HW307802.1 | DL091227.1 | CS604837.1 | DD402028.1 | DL088391.1 | HB864762.1 | GN031934.1 | HC288832.1 |
| DL026178.1 | HW307770.1 | DL091195.1 | CS604805.1 | DD401996.1 | DL121887.1 | HB866944.1 | GN031902.1 | HC295438.1 |
| DL018988.1 | HW316582.1 | DL113538.1 | CS604773.1 | DD401964.1 | DL121855.1 | HB878168.1 | GN031870.1 | HC292362.1 |
| DL018956.1 | HW307732.1 | DL125919.1 | CS604709.1 | DD401903.1 | DL121823.1 | HB866502.1 | GN031838.1 | HC294924.1 |
| DL018924.1 | HW307700.1 | DL108373.1 | CS604677.1 | DD401871.1 | DL121791.1 | HC000355.1 | GN031805.1 | HC291798.1 |
| DL018892.1 | HW315945.1 | DL080575.1 | CS604645.1 | DD401839.1 | DL117528.1 | HB981253.1 | GN031741.1 | HC299189.1 |
| DL018828.1 | HW315560.1 | DL078920.1 | CS604611.1 | DD405393.1 | DL117496.1 | HB977427.1 | GN031708.1 | HC299131.1 |
| DJ417440.1 | HV743341.1 | DL074233.1 | CS604579.1 | DD401631.1 | DL117464.1 | HB976599.1 | GN031676.1 | HC294684.1 |
| DJ402730.1 | HV743301.1 | DL073044.1 | CS435262.1 | DD401599.1 | DL117432.1 | HB976138.1 | GN031644.1 | HC291162.1 |
| DJ381041.1 | HV743264.1 | DL022290.1 | CS435198.1 | DD401567.1 | DL112753.1 | DM193330.1 | GN031612.1 | HC290982.1 |
| DJ381009.1 | HV743225.1 | DL022258.1 | CS434835.1 | DD401535.1 | DL098315.1 | DM195296.1 | GN031548.1 | HC293565.1 |
| DJ380977.1 | HV704981.1 | DJ082663.1 | DD335664.1 | DD401503.1 | DL098283.1 | DM189840.1 | GN031516.1 | HC290605.1 |
| DJ380945.1 | HV744581.1 | DJ080725.1 | DD349507.1 | DD401471.1 | DL098251.1 | HB840233.1 | GN031484.1 | DM103435.1 |
| DJ380913.1 | HV702435.1 | DJ069480.1 | DD334592.1 | CS480606.1 | DL098219.1 | HB848136.1 | GN031452.1 | DM102930.1 |
| DJ380881.1 | HV701837.1 | DJ066370.1 | DD347836.1 | CS479228.1 | DL098187.1 | HB847876.1 | GN031419.1 | GN368734.1 |
| DJ380849.1 | HV701243.1 | DJ066338.1 | DD346851.1 | CS476464.1 | DL098155.1 | HB847640.1 | GN031387.1 | GN368414.1 |
| DJ380829.1 | HV701211.1 | DJ066271.1 | DD347170.1 | CS469177.1 | DL044746.1 | GN030855.1 | GN031355.1 | GN367618.1 |
| DJ389546.1 | HV701179.1 | DJ066237.1 | DD331529.1 | CS467570.1 | DL044714.1 | GN030823.1 | GN031323.1 | GN367582.1 |
| DJ389312.1 | HV701147.1 | DJ062857.1 | DD347829.1 | CS464625.1 | DL044682.1 | GN030791.1 | GN031290.1 | GN367541.1 |
| DJ388700.1 | HV701115.1 | DJ061675.1 | CS430799.1 | CS464829.1 | DL044530.1 | DM001676.1 | GN031258.1 | GN373315.1 |

| | | | | | | | | |
|---|---|---|---|---|---|---|---|---|
| DJ388659.1 | HV708181.1 | DJ061622.1 | CS423576.1 | CS464550.1 | DL044466.1 | DM001470.1 | GN031194.1 | GN370248.1 |
| CS724462.1 | HV701096.1 | DJ061526.1 | CS418537.1 | CS464497.1 | DL036570.1 | DM006946.1 | GN031161.1 | GN360157.1 |
| CS721833.1 | HV701064.1 | DJ061478.1 | CS418505.1 | DD392046.1 | DL036538.1 | DM006536.1 | GN031097.1 | GN360125.1 |
| DJ352105.1 | HV701032.1 | DJ061406.1 | CS418638.1 | DD391939.1 | DL032239.1 | DM004996.1 | GN031065.1 | GN360093.1 |
| DJ357785.1 | HV695526.1 | DJ055186.1 | CS419551.1 | DD382837.1 | DL032207.1 | DM003497.1 | GN031033.1 | GN356162.1 |
| DJ357753.1 | HV695430.1 | DJ056273.1 | CS419064.1 | HW069955.1 | DL032175.1 | GM996586.1 | GN031001.1 | GN359892.1 |
| DJ357721.1 | HV694842.1 | CS810624.1 | CS418774.1 | HW069791.1 | DL048445.1 | GM996490.1 | GN030969.1 | GN366318.1 |
| DJ357687.1 | HV694765.1 | CS812984.1 | CS418742.1 | HW069759.1 | DL048413.1 | GM996203.1 | GN030938.1 | GN366286.1 |
| DJ357655.1 | HV700728.1 | CS812758.1 | CS418710.1 | HW084947.1 | DL048381.1 | GN010003.1 | GN030906.1 | GN359879.1 |
| DJ357623.1 | HV700664.1 | DJ021063.1 | CS418678.1 | HW097741.1 | DL048349.1 | GM994683.1 | GN030874.1 | GN359847.1 |
| DJ357591.1 | HV694639.1 | CS803355.1 | CS418646.1 | HW067065.1 | DL044271.1 | GM994304.1 | GN030842.1 | GN359815.1 |
| DJ357284.1 | HV700246.1 | CS802330.1 | CS417161.1 | HW065056.1 | DL040095.1 | GM992940.1 | GN030778.1 | GN359751.1 |
| DJ350052.1 | HV693892.1 | DJ047081.1 | CS416831.1 | HW060210.1 | DL032038.1 | GM992462.1 | DL037896.1 | GN359667.1 |
| DJ355101.1 | HV699824.1 | DJ046497.1 | CS416266.1 | HW047622.1 | DL032006.1 | GM977074.1 | DL049696.1 | GN359635.1 |
| DJ354937.1 | HV699496.1 | DJ050550.1 | CS415802.1 | HW047247.1 | DL031974.1 | GM992099.1 | DL049664.1 | GN359571.1 |
| DJ361247.1 | HV699151.1 | DJ049436.1 | CS410960.1 | HV969579.1 | DL028133.1 | GM975872.1 | DL027961.1 | GN359507.1 |
| DJ361213.1 | HV698337.1 | DJ048825.1 | CS410714.1 | HV956153.1 | DL028101.1 | GM990853.1 | DL027868.1 | GN359343.1 |
| DJ354229.1 | HV698227.1 | CS793883.1 | CS414857.1 | HV951080.1 | DL028069.1 | GM989786.1 | DL027836.1 | GN359311.1 |
| DJ354097.1 | HV689999.1 | CS720809.1 | CS414785.1 | HV957525.1 | DL028037.1 | GN000589.1 | DL027804.1 | GN365272.1 |
| DJ339860.1 | HV688788.1 | CS799087.1 | CS410125.1 | HV951771.1 | DL028005.1 | GM969577.1 | DL027772.1 | GN359270.1 |
| DJ339828.1 | HI979288.1 | CS227316.1 | DD327516.1 | HV780579.1 | DL027973.1 | GM980877.1 | DJ357635.1 | GN359206.1 |
| DJ339796.1 | HI989138.1 | CS206051.1 | DD323500.1 | HV779884.1 | DL060743.1 | GN007491.1 | DJ340886.1 | GN359142.1 |
| DJ339732.1 | HI989090.1 | CS208081.1 | DD327000.1 | HV778422.1 | DL044238.1 | FB986343.1 | DJ341031.1 | GN364167.1 |
| DJ339700.1 | HI988918.1 | CS207907.1 | DD329925.1 | HV774724.1 | DL044206.1 | GM970136.1 | DJ354952.1 | GN359080.1 |
| DJ339668.1 | HI987428.1 | CS189614.1 | DD329748.1 | HV760357.1 | DL044174.1 | GM651682.1 | DJ354253.1 | GN359048.1 |
| DJ339603.1 | FW508668.1 | CS193201.1 | DD326847.1 | HV743675.1 | DL048247.1 | GM644786.1 | DJ361192.1 | GN363563.1 |
| DJ339571.1 | FW560574.1 | CS176653.1 | DD329571.1 | HV743383.1 | DL048215.1 | GM644754.1 | DJ339840.1 | GN363029.1 |
| DJ339539.1 | FW561971.1 | CS174643.1 | DD321811.1 | HV700897.1 | DL048183.1 | GM644722.1 | DJ339776.1 | GN346509.1 |
| DJ339297.1 | FW552166.1 | CS160724.1 | DD321768.1 | HW287331.1 | DL048151.1 | GM633060.1 | DJ339648.1 | GN346475.1 |
| DJ339083.1 | FW552129.1 | CS155779.1 | CS390501.1 | HW302699.1 | DL048119.1 | GM625225.1 | DJ339583.1 | GN348298.1 |
| DJ334259.1 | FW554622.1 | CS144285.1 | CS398359.1 | HW286476.1 | DL048087.1 | GM625193.1 | DJ326904.1 | GN346830.1 |
| DJ333882.1 | FW555595.1 | CS141655.1 | CS389322.1 | HW302541.1 | DL043882.1 | GM625161.1 | DJ122186.1 | GM656790.1 |
| DJ330234.1 | FW556533.1 | AY658556.1 | CS389289.1 | HW295582.1 | DL040065.1 | GM624039.1 | DJ090059.1 | GM656758.1 |
| DJ329290.1 | FW561697.1 | AY658524.1 | CS389247.1 | HW307461.1 | DL040033.1 | GM624007.1 | DJ082712.1 | GM649765.1 |
| DJ328781.1 | FW562762.1 | AY658492.1 | CS389200.1 | HW294146.1 | DL040001.1 | GM623975.1 | DJ082658.1 | GM642701.1 |
| DJ326989.1 | FW565215.1 | AY658460.1 | CS389162.1 | HW294081.1 | DJ047124.1 | GM623943.1 | DJ084902.1 | GM642669.1 |
| DJ326629.1 | FW562623.1 | AY658428.1 | CS388974.1 | HW294047.1 | DJ046986.1 | GM625117.1 | DJ071629.1 | GM642637.1 |
| DJ122507.1 | FW561201.1 | AY658396.1 | CS401628.1 | HW306882.1 | DJ051985.1 | GM625085.1 | DJ080416.1 | GM630489.1 |
| DJ122007.1 | FW508164.1 | AY658364.1 | CS401294.1 | HV802913.1 | DJ045452.1 | GM625053.1 | DJ069477.1 | GM630457.1 |
| CS603814.1 | FW508114.1 | AY658332.1 | CS406725.1 | HV802881.1 | DJ050211.1 | GM625021.1 | DJ066763.1 | GM630425.1 |
| CS603462.1 | FW562451.1 | AY658300.1 | CS406454.1 | HV818963.1 | DJ049445.1 | GM624989.1 | DJ066400.1 | GM630393.1 |
| CS603430.1 | FW555398.1 | AY658268.1 | CS406005.1 | HV818570.1 | DJ048834.1 | GM624957.1 | DJ066367.1 | GM630361.1 |
| CS603398.1 | FW553170.1 | FW381486.1 | CS405406.1 | HV812978.1 | CS793894.1 | GM623835.1 | DJ066335.1 | GM630329.1 |
| CS603366.1 | FW553092.1 | FW370106.1 | CS410008.1 | HV817913.1 | CS721583.1 | GM623803.1 | DJ066301.1 | GM630297.1 |
| CS603334.1 | FW556663.1 | HH735636.1 | DL118522.1 | HV822145.1 | CS800055.1 | GM623771.1 | DJ066267.1 | GM621522.1 |
| CS603302.1 | FW560830.1 | HH733724.1 | DL118490.1 | HV784496.1 | CS719262.1 | GM623739.1 | DJ066213.1 | GM621490.1 |
| CS603270.1 | FW559385.1 | FW361965.1 | DL118458.1 | HV780243.1 | CS798869.1 | GM827621.1 | DJ065249.1 | GM649463.1 |

| | | | | | | | | |
|---|---|---|---|---|---|---|---|---|
| CS603238.1 | FW506874.1 | FW361304.1 | DL118426.1 | HV778758.1 | CS716771.1 | GM658918.1 | DJ055700.1 | GM649431.1 |
| CS603206.1 | FW510485.1 | HC917635.1 | DL114030.1 | HV778624.1 | CS715275.1 | GM658886.1 | DJ061403.1 | GM649399.1 |
| CS603174.1 | HH733848.1 | FW337285.1 | DL113998.1 | HV778229.1 | CS805313.1 | GM658854.1 | CS161651.1 | GM670039.1 |
| CS603142.1 | HH733485.1 | FW336104.1 | DL113966.1 | HV775779.1 | CS680733.1 | GM658822.1 | CS159786.1 | GM656534.1 |
| CS603110.1 | FW368810.1 | HC873811.1 | DL113934.1 | HV775196.1 | CS793039.1 | GM644607.1 | CS159597.1 | GM656502.1 |
| CS603078.1 | FW367218.1 | FW310591.1 | DL113902.1 | HV764772.1 | CS792476.1 | GM637417.1 | CS159307.1 | GM656470.1 |
| CS603046.1 | FW366204.1 | HC679512.1 | DL113870.1 | HV764708.1 | CS791972.1 | GM637385.1 | CS148825.1 | GM656438.1 |
| CS603014.1 | FW351234.1 | HC679167.1 | DL109069.1 | HV774550.1 | DJ044928.1 | GM637353.1 | CS141614.1 | GM649341.1 |
| CS602980.1 | FW351174.1 | HC504252.1 | DL109037.1 | HV774484.1 | DJ044887.1 | GM637321.1 | CS134733.1 | GM649309.1 |
| CS602948.1 | FW351014.1 | HC502384.1 | DL109005.1 | HV764402.1 | DJ028188.1 | GM637289.1 | CS135964.1 | GM649253.1 |
| CS602916.1 | FW349920.1 | HC501840.1 | DL108973.1 | HV764260.1 | DJ030384.1 | GM637257.1 | CS133026.1 | GM649221.1 |
| CS602884.1 | HD122531.1 | HC499847.1 | DL108941.1 | HV767443.1 | DJ029986.1 | GM623656.1 | CS123409.1 | GM649189.1 |
| CS602852.1 | HD082684.1 | FW300642.1 | DL108909.1 | HV761168.1 | CS721634.1 | GM623624.1 | CS122923.1 | GM642580.1 |
| CS602788.1 | HD082424.1 | HC487996.1 | DL104303.1 | HV766939.1 | CS674191.1 | GM623592.1 | CS121751.1 | GM642548.1 |
| CS602756.1 | HD088245.1 | HC486541.1 | DL104271.1 | HV766778.1 | CS691858.1 | GM623560.1 | CS106117.1 | GM642468.1 |
| CS602724.1 | HD077955.1 | HC475398.1 | DL104239.1 | HV760615.1 | DJ012077.1 | GM658709.1 | CS106047.1 | GM642451.1 |
| CS602628.1 | HD070439.1 | HC491744.1 | DL104207.1 | HV766043.1 | DJ008400.1 | GM658677.1 | CS179680.1 | GM642419.1 |
| CS602500.1 | FW345119.1 | FV533459.1 | DL104175.1 | HV766011.1 | DJ008368.1 | GM658645.1 | CS327366.1 | GM635237.1 |
| CS602468.1 | FW345009.1 | FV531699.1 | DL104143.1 | HV502595.1 | DD321798.1 | GM658613.1 | CS326330.1 | GM635205.1 |
| CS611204.1 | FW344977.1 | HC305989.1 | DL104111.1 | HV502563.1 | HC289343.1 | GM658549.1 | CS330854.1 | GM635173.1 |
| CS610262.1 | FW344726.1 | HC302634.1 | DL099697.1 | HV502531.1 | HC289311.1 | GM651357.1 | CS329179.1 | GM635141.1 |
| CS602086.1 | HC471769.1 | HC305918.1 | DL099665.1 | HV502499.1 | HC296931.1 | GM628290.1 | CS318803.1 | GM635109.1 |
| CS601990.1 | HC471737.1 | HC305758.1 | DL099601.1 | HV502467.1 | HC295998.1 | GM046381.1 | CS298533.1 | GM635077.1 |
| CS607925.1 | HC471693.1 | HC291582.1 | DL099569.1 | HV492677.1 | HC295711.1 | GM627934.1 | CS297032.1 | GM630271.1 |
| CS607536.1 | GM637079.1 | HC289365.1 | DL099537.1 | HV505317.1 | HC288864.1 | GM661744.1 | DD253207.1 | GM630239.1 |
| CS607092.1 | GM624926.1 | HC289331.1 | DL093527.1 | HV505212.1 | HC295494.1 | GM654750.1 | DD251642.1 | GM630207.1 |
| CS607010.1 | GM624894.1 | HC292840.1 | DL093495.1 | HV494726.1 | HC292559.1 | GM654718.1 | DD248477.1 | GM737351.1 |
| CS606923.1 | GM624862.1 | HC296886.1 | DL093431.1 | HV507620.1 | HC292314.1 | GM654654.1 | CS288133.1 | GM635010.1 |
| CS606802.1 | GM632645.1 | HC292708.1 | DL093399.1 | HV453660.1 | HB485524.1 | GM647596.1 | CS287609.1 | GM634978.1 |
| CS605116.1 | GM658455.1 | HC295614.1 | DL093367.1 | HV451953.1 | HB475508.1 | GM647564.1 | DD178517.1 | GM634946.1 |
| CS605084.1 | GM651175.1 | HC292410.1 | DL089752.1 | HV453000.1 | HB469147.1 | GM627893.1 | DD172701.1 | GM634882.1 |
| CS605052.1 | GM651143.1 | HC295301.1 | DL033189.1 | HV445492.1 | HB468029.1 | GM627861.1 | DD165461.1 | GM629948.1 |
| CS605025.1 | GM651111.1 | HC292158.1 | DL033157.1 | HV448963.1 | DM152674.1 | GM627829.1 | DD182207.1 | GM629916.1 |
| DD401873.1 | GM651079.1 | HC291978.1 | DL033125.1 | HV450928.1 | DM156108.1 | GM627797.1 | DD176003.1 | GM642402.1 |
| DD401841.1 | GM651047.1 | HC291726.1 | DL029117.1 | HV444185.1 | HB461516.1 | GM661715.1 | CS157814.1 | FB709026.1 |
| DD401809.1 | GM651015.1 | HC294504.1 | DL029085.1 | HV443998.1 | HB455021.1 | GM661651.1 | CS157782.1 | GM887751.1 |
| DD401777.1 | GM650983.1 | HC293930.1 | DL029053.1 | HV437544.1 | HB444504.1 | GM661619.1 | CS157943.1 | FB983740.1 |
| DD401745.1 | GM644120.1 | HC293750.1 | DL029021.1 | HV349484.1 | DM069069.1 | GM661587.1 | CS157911.1 | FB753596.1 |
| DD401713.1 | GM644088.1 | HC290797.1 | DL028989.1 | HV351584.1 | GN090933.1 | GM661555.1 | CS157879.1 | GM879321.1 |
| DD401681.1 | DL233408.1 | HC293248.1 | DL028957.1 | HV341324.1 | GN087188.1 | GM661523.1 | CS157847.1 | GM680730.1 |
| DD405395.1 | DL232088.1 | HC289969.1 | DL025111.1 | HV344078.1 | GN089850.1 | GM654529.1 | CS124700.1 | GM618714.1 |
| DD405363.1 | DL220455.1 | HC293056.1 | DL024983.1 | HV343850.1 | GN093654.1 | GM654497.1 | HW115376.1 | GM060298.1 |
| DD405331.1 | DL206283.1 | HC289859.1 | DL021771.1 | HV343658.1 | GN089190.1 | GM654465.1 | HW069936.1 | GM841788.1 |
| DD401633.1 | FB714468.1 | HC208020.1 | DL021739.1 | HV343514.1 | GN082817.1 | GM654433.1 | HW069804.1 | GM841720.1 |
| DD401601.1 | FB713985.1 | HC207395.1 | DL021675.1 | HV347639.1 | GN088788.1 | GM654401.1 | HW084924.1 | GM604044.1 |
| DD401569.1 | FB713857.1 | HC201542.1 | DL021643.1 | HV344842.1 | GN088034.1 | GM654369.1 | HW065069.1 | GM706714.1 |
| DD401537.1 | FB713793.1 | HC199497.1 | DL021611.1 | HV347309.1 | M27056.1 | GM647280.1 | HW049576.1 | GM706682.1 |

| | | | | | | | | |
|---|---|---|---|---|---|---|---|---|
| DD401505.1 | FB713729.1 | DM462694.1 | DL017405.1 | HV036665.1 | L08941.1 | GM647248.1 | HV951093.1 | GM706650.1 |
| DD401473.1 | FB712759.1 | HC187673.1 | DL017373.1 | HV036225.1 | M94410.1 | GM647216.1 | HV957539.1 | GM706612.1 |
| CS480733.1 | FB708486.1 | HC089567.1 | DL017277.1 | HV031611.1 | DM059615.1 | GM870241.1 | HV957471.1 | GM706580.1 |
| CS479247.1 | FB707957.1 | HC089535.1 | DL017245.1 | FZ436001.1 | DM058833.1 | GM627550.1 | HV945091.1 | GM706385.1 |
| CS476470.1 | FB671097.1 | HC089503.1 | DL012952.1 | FZ435804.1 | DM045518.1 | GM627518.1 | HV777012.1 | GM603497.1 |
| CS467734.1 | FB667095.1 | HC089471.1 | DL012920.1 | FZ425930.1 | DM045380.1 | GM661491.1 | HV775596.1 | FB742333.1 |
| CS467513.1 | CS721885.1 | HC089439.1 | DL012888.1 | FZ437387.1 | DM045233.1 | GM661427.1 | HV743711.1 | FB721892.1 |
| CS464634.1 | FB676599.1 | HC089406.1 | DL012824.1 | FZ423868.1 | DM039603.1 | GM661395.1 | HW302497.1 | FB720252.1 |
| CS464702.1 | FB676535.1 | HC089374.1 | DL012792.1 | FZ423327.1 | DM056327.1 | GM661363.1 | HW295729.1 | FB717732.1 |
| CS464499.1 | FB675468.1 | HC089342.1 | DL045632.1 | FZ422764.1 | DM044825.1 | GM661331.1 | HW295687.1 | FB706259.1 |
| DD392050.1 | FB701478.1 | HC089309.1 | DL045600.1 | FZ422532.1 | DM060671.1 | GM654337.1 | HW295166.1 | FB677408.1 |
| DD391854.1 | CS809031.1 | HC089277.1 | DL045568.1 | FZ423866.1 | GN068784.1 | GM654305.1 | HW294992.1 | DL460019.1 |
| DD382839.1 | FB707082.1 | HC089093.1 | DL045536.1 | FZ432335.1 | GN067918.1 | GM654273.1 | HW089644.1 | DL262712.1 |
| DD400163.1 | FB660487.1 | GM644820.1 | DL045504.1 | FZ423315.1 | GN067886.1 | GM654241.1 | HW102820.1 | DL260583.1 |
| DD400131.1 | FB660046.1 | GM659294.1 | DL045472.1 | FZ431936.1 | GN067790.1 | FB667097.1 | HW084180.1 | DL259340.1 |
| CS459112.1 | FB660079.1 | GM741822.1 | DL041360.1 | FZ419476.1 | GN067762.1 | CS721887.1 | AB691538.1 | DL257043.1 |
| CS063842.1 | FB665217.1 | GM621673.1 | DL041328.1 | FZ415416.1 | GN067698.1 | FB669803.1 | HW061869.1 | DL241040.1 |
| CS061203.1 | FB654419.1 | GM621641.1 | DL037409.1 | FZ420875.1 | GN067634.1 | FB676600.1 | HW061836.1 | FB674306.1 |
| CS061032.1 | FB654873.1 | GM647012.1 | DL037377.1 | FZ417036.1 | GN065095.1 | FB676568.1 | HW065411.1 | DL236615.1 |
| CS059011.1 | FB583261.1 | GM640046.1 | DL037345.1 | FZ416969.1 | GN060118.1 | FB676536.1 | HW065316.1 | DL233410.1 |
| AY967388.1 | FB653433.1 | GM646870.1 | DL037313.1 | FZ419857.1 | GN059992.1 | FB675469.1 | HW065218.1 | DL086859.1 |
| AY967356.1 | DL124139.1 | GM646838.1 | DL033078.1 | FZ419822.1 | GN047430.1 | FB701479.1 | HW061318.1 | DL086827.1 |
| AY967324.1 | DL124107.1 | GM646806.1 | DL033046.1 | FZ419790.1 | GM625050.1 | CS809032.1 | HW064852.1 | DL086795.1 |
| AY967292.1 | DL119789.1 | GM646474.1 | DL033014.1 | FW582541.1 | GM625018.1 | FB707083.1 | HW055554.1 | DL086763.1 |
| AY967260.1 | DL119757.1 | GM646442.1 | DL032982.1 | FW582849.1 | GM623832.1 | FB660047.1 | HW064716.1 | DL086731.1 |
| AY967228.1 | DL119725.1 | GM646410.1 | DL032950.1 | FW582503.1 | GM658915.1 | FB660080.1 | HW060677.1 | DL086699.1 |
| AY967196.1 | DL135318.1 | GM626868.1 | DL028782.1 | HH980569.1 | GM658883.1 | FB654849.1 | HW062419.1 | DL086667.1 |
| AY967164.1 | DL119487.1 | GM626836.1 | DL024936.1 | HH980498.1 | GM658851.1 | FB654421.1 | HW062387.1 | DL105876.1 |
| AY967132.1 | DL119455.1 | GM626804.1 | DL024904.1 | HH980430.1 | GM658819.1 | FB654874.1 | HW056251.1 | DL105844.1 |
| AY967100.1 | DL119423.1 | DL095425.1 | DL024872.1 | HH980357.1 | GM644604.1 | FB583262.1 | HW064429.1 | DL105812.1 |
| AY967068.1 | DL123670.1 | DL095361.1 | DL024840.1 | HH980297.1 | GM637414.1 | FB653435.1 | HW062362.1 | DL096529.1 |
| AY967036.1 | DL123638.1 | DL128774.1 | DL024808.1 | HH980222.1 | GM637382.1 | FB580216.1 | HW062330.1 | DL096497.1 |
| AY967004.1 | DL123606.1 | DL111652.1 | DL024776.1 | HH980158.1 | GM637318.1 | FB580184.1 | HW055878.1 | DL096465.1 |
| AY966972.1 | DL123574.1 | DL111620.1 | DL021468.1 | HH980125.1 | GM637286.1 | CQ801675.1 | HW043766.1 | DL110742.1 |
| AY966940.1 | DL119384.1 | DL111588.1 | DL021436.1 | HH979880.1 | GM637254.1 | CQ800991.1 | HW042053.1 | DL110710.1 |
| CS057820.1 | DL119352.1 | DL094228.1 | DL021404.1 | HH996708.1 | GM623653.1 | CQ798794.1 | HW043551.1 | DL110678.1 |
| CS056288.1 | DL114828.1 | DL094196.1 | DL017198.1 | HH996643.1 | GM623621.1 | CQ795483.1 | HW042009.1 | DL090676.1 |
| CS055393.1 | DL114796.1 | DL094164.1 | DL017166.1 | HH996596.1 | GM623589.1 | CQ795451.1 | HW041977.1 | DL086575.1 |
| CS054772.1 | DL114764.1 | DL100497.1 | DL017134.1 | HH996547.1 | GM623557.1 | CQ788136.1 | HW041945.1 | DL086543.1 |
| CS053039.1 | DL114732.1 | DL100433.1 | DL017102.1 | HH998452.1 | GM658706.1 | CQ787514.1 | HW041913.1 | DL086511.1 |
| CS052459.1 | DL114700.1 | DL100401.1 | DL017070.1 | HH998404.1 | GM658674.1 | CQ787482.1 | HW049511.1 | DL086447.1 |
| CS052389.1 | DL114668.1 | DL100369.1 | DL017038.1 | HH998365.1 | GM658642.1 | CQ787450.1 | HW049399.1 | DL086415.1 |
| CS052355.1 | DL127012.1 | DL100337.1 | DL012777.1 | HH999997.1 | GM658610.1 | CQ787410.1 | HW049367.1 | DL115549.1 |
| CS052321.1 | DL141540.1 | DL124150.1 | DL012745.1 | HH999951.1 | GM658546.1 | CQ787370.1 | HW049336.1 | DL115517.1 |
| CS048828.1 | DL104904.1 | DL124118.1 | DL012713.1 | HH979819.1 | GM644235.1 | CQ787336.1 | HW049304.1 | DL115485.1 |
| AX799694.1 | DL123470.1 | DL124086.1 | DL012681.1 | HH979669.1 | GM637141.1 | CQ787270.1 | HW049272.1 | DL115453.1 |
| AX799204.1 | DL123438.1 | DL124054.1 | DL012649.1 | HH976343.1 | GM637109.1 | CQ787238.1 | HW049240.1 | DL105669.1 |

| | | | | | | | | |
|---|---|---|---|---|---|---|---|---|
| AX798912.1 | DL123406.1 | DL124022.1 | DL012617.1 | HH999866.1 | GM637077.1 | CQ786934.1 | HW041811.1 | DL105637.1 |
| AX796932.1 | DL123374.1 | DL123990.1 | DL049437.1 | HH999777.1 | GM624924.1 | CQ784719.1 | HW041779.1 | DL105605.1 |
| AX796752.1 | DL119120.1 | DL119800.1 | DL049405.1 | HH999723.1 | GM624892.1 | CQ784655.1 | HW047559.1 | DL100800.1 |
| AX795592.1 | DL119088.1 | DL119768.1 | HB865991.1 | HH998279.1 | GM624860.1 | CQ784468.1 | HI369091.1 | DL100768.1 |
| AX795560.1 | DL119056.1 | DL119736.1 | HB865373.1 | HH998242.1 | GM632643.1 | CQ772687.1 | HH954751.1 | DL100736.1 |
| AX795440.1 | DL119024.1 | DL119704.1 | HB865027.1 | HH998194.1 | GM658453.1 | CQ771654.1 | HI424334.1 | DL096322.1 |
| BD189469.1 | DL114628.1 | DL119672.1 | HB864977.1 | HH998153.1 | GM651173.1 | CQ771622.1 | HI424119.1 | DL096290.1 |
| AX794210.1 | DL114596.1 | DL119640.1 | HB864987.1 | HH993744.1 | GM651141.1 | CQ768808.1 | HI424087.1 | DL096258.1 |
| AX777469.1 | DL114532.1 | DL119608.1 | HB864931.1 | HH979528.1 | GM651109.1 | CQ759500.1 | HI424055.1 | CS606974.1 |
| AX774563.1 | DL114500.1 | DL115013.1 | HB864899.1 | HH979632.1 | GM651077.1 | CQ756620.1 | HI423797.1 | CS606912.1 |
| AX773252.1 | DL114468.1 | DL114981.1 | HB864867.1 | HH979593.1 | GM651045.1 | CQ754678.1 | HI413613.1 | CS606838.1 |
| AX768202.1 | DL104901.1 | DL114949.1 | HB864835.1 | HH932390.1 | GM634460.1 | CQ754036.1 | HI503524.1 | CS606796.1 |
| AX766882.1 | DL104869.1 | DL114917.1 | HB864803.1 | HH932008.1 | GM634428.1 | AX074121.1 | HI516097.1 | CS605995.1 |
| AX766732.1 | DL104837.1 | DL114885.1 | HB864771.1 | HH829711.1 | GM634396.1 | AX068195.1 | HI003304.1 | CS605051.1 |
| AX766390.1 | DL104805.1 | DL114853.1 | HB859427.1 | FW394269.1 | GM634364.1 | AX061297.1 | HI284336.1 | CS605019.1 |
| AX765960.1 | DL095914.1 | DL123933.1 | HB866518.1 | FW398172.1 | GM629473.1 | AX060542.1 | HI284284.1 | CS604987.1 |
| AX764645.1 | DL095882.1 | DL123901.1 | HB866108.1 | FW398135.1 | GM629431.1 | AX058854.1 | HI574505.1 | CS604923.1 |
| AX764613.1 | DL095850.1 | DL123869.1 | HC001060.1 | FW394250.1 | GM629399.1 | AX057968.1 | HI574473.1 | CS604891.1 |
| AX764581.1 | DL041081.1 | DL123837.1 | HC000277.1 | FW394079.1 | GM629367.1 | AX057294.1 | HI574441.1 | CS604859.1 |
| AX752153.1 | DL037130.1 | DL123805.1 | HB977451.1 | FW398057.1 | GM629335.1 | AX056090.1 | HI574409.1 | CS604827.1 |
| BD185677.1 | DL028663.1 | DL123773.1 | HB976763.1 | FW397866.1 | GM629303.1 | AX055011.1 | HI574377.1 | CS604795.1 |
| BD183118.1 | DL028631.1 | DL119594.1 | HB976616.1 | FW393462.1 | GM648566.1 | AX052953.1 | HI001463.1 | CS604763.1 |
| BD182772.1 | DL028599.1 | DL119562.1 | HB976184.1 | FW392977.1 | GM648534.1 | AX046146.1 | HI001367.1 | CS604731.1 |
| BD180899.1 | DL028567.1 | DL119530.1 | DM193650.1 | FW418243.1 | GM648502.1 | AX040777.1 | HI001333.1 | CS604699.1 |
| BD180806.1 | DL048686.1 | DL119498.1 | DM193401.1 | HH936626.1 | GM629194.1 | AX039524.1 | HI003227.1 | CS604667.1 |
| AX744003.1 | DL037077.1 | DL119466.1 | GN037325.1 | HH835190.1 | DL036567.1 | AX038889.1 | HI003170.1 | CS604633.1 |
| AX742851.1 | DL037045.1 | DL119434.1 | GN034793.1 | HH834326.1 | DL036535.1 | AX036756.1 | HI003104.1 | CS604601.1 |
| AX739969.1 | DL028354.1 | DL123745.1 | GN030752.1 | HH833918.1 | DL018706.1 | AX036024.1 | HI003058.1 | CS604569.1 |
| AX721698.1 | DL021168.1 | DL123713.1 | GN030656.1 | HH833672.1 | DL018674.1 | AX035971.1 | HI003022.1 | CS596756.1 |
| BD178183.1 | DL021136.1 | DL123681.1 | GN030624.1 | HH833640.1 | DL018642.1 | AX035428.1 | HI002921.1 | CS598815.1 |
| BD177361.1 | HV504701.1 | DL123649.1 | GN030592.1 | HH833608.1 | DL018610.1 | AX034327.1 | HI001285.1 | CS597729.1 |
| BD177069.1 | HV504669.1 | DL123617.1 | GN030560.1 | HH833576.1 | DL014182.1 | AX032989.1 | HI001235.1 | CS593363.1 |
| AX718181.1 | HV504605.1 | DL123585.1 | GN030528.1 | HH833544.1 | DL014150.1 | AX027717.1 | HI001186.1 | CS584934.1 |
| AX709549.1 | HV504988.1 | DL119395.1 | GN030496.1 | HH833512.1 | DL014118.1 | AX023758.1 | HI001124.1 | CS588483.1 |
| AX708513.1 | HV504198.1 | DL119363.1 | GN030464.1 | HH833480.1 | DL014086.1 | AX023616.1 | HC731037.1 | CS575312.1 |
| AX705184.1 | HV491265.1 | DL119331.1 | GN030425.1 | HH833448.1 | DL030035.1 | AX023582.1 | HC728197.1 | CS576677.1 |
| AX701592.1 | HV503770.1 | DL119267.1 | GN030441.1 | HH833416.1 | DL029971.1 | AX016744.1 | HC688467.1 | CS565545.1 |
| AX701060.1 | HV503738.1 | DL119235.1 | GN030368.1 | HH833384.1 | DL046628.1 | AX011474.1 | HC688385.1 | CS545385.1 |
| AX145349.1 | HV503706.1 | DL114839.1 | GN030336.1 | HB855100.1 | DL046596.1 | AX011442.1 | HC490786.1 | CS561005.1 |
| AX145317.1 | HV503674.1 | DL114807.1 | GN030272.1 | HB850753.1 | DL046564.1 | AX011410.1 | HC490754.1 | CS560458.1 |
| AX145285.1 | HV503642.1 | DL114775.1 | GN030240.1 | HB855988.1 | DL046532.1 | AX011377.1 | HC490722.1 | DD419839.1 |
| AX145253.1 | FW571814.1 | DL114743.1 | GN030208.1 | HB855900.1 | DL046500.1 | AX011345.1 | HC490690.1 | CS542029.1 |
| AX145221.1 | HI968056.1 | DL114711.1 | GN030144.1 | HB855424.1 | DL042646.1 | AX010939.1 | HC679166.1 | CS541289.1 |
| AX145189.1 | HI967880.1 | DL114679.1 | GN030112.1 | DM179539.1 | DL042614.1 | AX009285.1 | HC678805.1 | CS542552.1 |
| AX145157.1 | HI980537.1 | DL114647.1 | GN030080.1 | DM179149.1 | DL042582.1 | AX008218.1 | HC510445.1 | CS537965.1 |
| AX145125.1 | HI988899.1 | DL127156.1 | GN029952.1 | DM170821.1 | DL034129.1 | AX004602.1 | HC504669.1 | CS534836.1 |
| AX145093.1 | HI987465.1 | DL127028.1 | GN029920.1 | DM185749.1 | DL029836.1 | AX004057.1 | HC504251.1 | BD374806.1 |

| | | | | | | | | |
|---|---|---|---|---|---|---|---|---|
| AX145061.1 | HI987424.1 | DL141560.1 | GN029822.1 | DM188011.1 | DL029740.1 | AX003040.1 | DJ344585.1 | BD373910.1 |
| AX145029.1 | FW552366.1 | DL141437.1 | GN029790.1 | DM187973.1 | DL029737.1 | AF083271.1 | DJ354949.1 | BD356452.1 |
| AX144997.1 | FW552199.1 | DL123545.1 | GN014101.1 | DM187723.1 | DL018309.1 | A47593.1 | DJ361564.1 | BD353560.1 |
| AX144965.1 | FW552124.1 | DL123513.1 | GN013384.1 | DM169203.1 | DL018277.1 | A38675.1 | DJ361222.1 | BD342478.1 |
| AX144933.1 | FW561711.1 | DL123481.1 | GN034222.1 | DM187602.1 | DL018245.1 | A35739.1 | DJ361189.1 | BD341867.1 |
| AX144900.1 | FW555591.1 | DL123449.1 | GN010337.1 | HB491789.1 | DL018213.1 | A33039.1 | DJ339869.1 | BD349764.1 |
| AX144868.1 | FW563146.1 | DL123417.1 | GN010226.1 | HB489511.1 | DL007501.1 | A29518.1 | DJ339837.1 | BD320230.1 |
| AX144836.1 | FW561693.1 | DL123385.1 | GN033556.1 | HB488760.1 | DL005432.1 | A35198.1 | DJ339741.1 | BD325633.1 |
| AX144804.1 | FW561661.1 | DL119195.1 | GN033524.1 | HB488676.1 | DD401526.1 | A34038.1 | CS398382.1 | BD325540.1 |
| AX144772.1 | FW562822.1 | DL119163.1 | GN033492.1 | DM164196.1 | DD401494.1 | A30885.1 | CS389294.1 | BD319846.1 |
| AX144740.1 | FW562743.1 | DL119131.1 | GN033460.1 | DM159695.1 | DD401462.1 | A30327.1 | CS389205.1 | BD319470.1 |
| AX144708.1 | FW565211.1 | DL119099.1 | GN033396.1 | DM164008.1 | CS479868.1 | A27305.1 | CS389168.1 | BD318831.1 |
| AX144676.1 | FW562596.1 | DL119067.1 | GN033364.1 | DM163974.1 | CS478265.1 | HW267837.1 | CS406469.1 | BD307860.1 |
| AX144644.1 | FW566795.1 | DL119035.1 | GN033332.1 | DM163305.1 | CS467755.1 | HW267795.1 | CS405730.1 | BD307643.1 |
| AX144611.1 | FW508110.1 | DL114639.1 | GN033300.1 | DM163265.1 | CS466047.1 | HW267742.1 | CS402131.1 | BD292805.1 |
| AX144547.1 | FW562447.1 | DL114607.1 | GN033268.1 | HB486508.1 | CS464669.1 | HW267437.1 | CS383383.1 | BD292428.1 |
| AX144355.1 | FW379803.1 | DL114575.1 | GN033236.1 | HB485572.1 | DD382828.1 | HW265987.1 | CS362759.1 | BD291581.1 |
| AX144033.1 | HH736083.1 | DL114543.1 | GN033204.1 | GN089858.1 | DD400152.1 | HW263026.1 | CS361280.1 | BD299606.1 |
| AX143967.1 | HH735909.1 | DL114511.1 | GN033172.1 | GN089794.1 | CS460112.1 | HW262962.1 | CS359724.1 | BD298674.1 |
| AX143903.1 | HH733744.1 | DL114479.1 | GN033140.1 | GN082892.1 | CS458930.1 | HW262914.1 | CS362348.1 | BD298506.1 |
| AX143839.1 | FW368806.1 | DL114447.1 | GN033108.1 | GN089194.1 | DD361283.1 | HW262871.1 | BD269157.1 | BD296935.1 |
| AX143775.1 | FW368175.1 | DL109550.1 | GN033076.1 | GN082821.1 | DD367611.1 | HW262571.1 | BD269125.1 | BD296230.1 |
| HW043444.1 | HD122424.1 | DL109518.1 | GN033044.1 | GN088796.1 | DD362900.1 | HW261405.1 | BD268881.1 | BD284589.1 |
| HW041997.1 | HD114746.1 | DL109486.1 | GN033012.1 | GN077249.1 | DD368338.1 | HW261341.1 | BD265636.1 | BD280116.1 |
| HW041965.1 | HD082417.1 | DL104880.1 | GN032980.1 | M25496.1 | DD368147.1 | HW261309.1 | BD265595.1 | BD289749.1 |
| HW041933.1 | HD081918.1 | DL017800.1 | GN032916.1 | DM058843.1 | DD361317.1 | HW261277.1 | BD263738.1 | BD287711.1 |
| HW041835.1 | HD088211.1 | DL017768.1 | GN032884.1 | DM042153.1 | CS457833.1 | HW261245.1 | BD263467.1 | BD287339.1 |
| HW049387.1 | HC471039.1 | DL017736.1 | GN032852.1 | DM045523.1 | A10884.1 | HW261213.1 | BD263435.1 | BD277257.1 |
| HW049356.1 | HC500971.1 | DL017704.1 | GN032820.1 | DM044953.1 | CS457188.1 | HW261181.1 | BD263022.1 | BD276264.1 |
| HW049324.1 | HC500237.1 | DL017672.1 | GN032788.1 | DM039607.1 | CS456728.1 | HW261149.1 | BD251790.1 | BD273671.1 |
| HW049292.1 | HC499857.1 | DL017640.1 | GN032756.1 | DM044848.1 | CS456090.1 | HW261117.1 | BD251249.1 | BD273166.1 |
| HW049260.1 | FW300946.1 | DL022374.1 | GN032725.1 | DM044666.1 | CS450656.1 | HW261085.1 | BD250876.1 | BD272139.1 |
| HW041799.1 | HC488028.1 | DL022342.1 | GN032693.1 | DM060675.1 | DD360946.1 | HW261053.1 | BD247928.1 | BD271074.1 |
| HW041767.1 | HC486497.1 | DL022310.1 | GN032661.1 | DM059967.1 | DD357990.1 | HW261021.1 | BD247124.1 | BD270494.1 |
| HW043120.1 | HC484129.1 | DL022278.1 | GN032628.1 | GN067890.1 | DD357916.1 | HW260989.1 | BD247027.1 | DD231223.1 |
| HW041494.1 | HC475376.1 | DL022246.1 | GN032564.1 | GN067766.1 | DD357837.1 | HW260957.1 | BD246952.1 | CQ898615.1 |
| HW045684.1 | HC466631.1 | DL022214.1 | GN032533.1 | GN067702.1 | DD355904.1 | HW260925.1 | BD244502.1 | CQ898583.1 |
| HW048283.1 | HC466418.1 | DL022182.1 | GN032469.1 | GN067638.1 | A19560.1 | HW260893.1 | BD242440.1 | CQ898513.1 |
| HW053882.1 | HC482496.1 | DL025729.1 | GN032437.1 | GN067606.1 | A12250.1 | HW260861.1 | BD238071.1 | CQ897003.1 |
| HW044051.1 | HC491754.1 | DL025697.1 | GN032405.1 | GN065109.1 | A08618.1 | FW576750.1 | CS073254.1 | CQ895563.1 |
| HV985971.1 | HC491661.1 | DL025665.1 | GN032373.1 | GN061126.1 | CS444613.1 | FW576639.1 | CS070588.1 | CQ895457.1 |
| HV985939.1 | HC481581.1 | DL025633.1 | GN032340.1 | GN059997.1 | CS446906.1 | HI004535.1 | CS061700.1 | CQ893704.1 |
| HV985907.1 | FV531709.1 | DL025601.1 | GN032308.1 | GN051387.1 | CS441595.1 | HI002432.1 | CS059818.1 | CQ890051.1 |
| HV985862.1 | FV531677.1 | DL025569.1 | GN032276.1 | GN046271.1 | CS438958.1 | HI002374.1 | AY967398.1 | CQ877783.1 |
| HW029056.1 | FV523750.1 | GM601155.1 | GN032244.1 | DM019384.1 | CS437393.1 | HI002312.1 | AY967366.1 | CQ875536.1 |
| HV984395.1 | FV530939.1 | FB722258.1 | GN032212.1 | DM023389.1 | BD497473.1 | HI000515.1 | AY967334.1 | CQ874989.1 |
| HV984318.1 | FV522807.1 | FB720268.1 | GN032180.1 | GM623836.1 | BD451719.1 | HI006800.1 | AY967302.1 | CQ873289.1 |

| | | | | | | | | |
|---|---|---|---|---|---|---|---|---|
| HV984233.1 | FV522625.1 | FB719724.1 | GN032148.1 | GM623804.1 | BD449812.1 | HI180159.1 | AY967270.1 | CQ871416.1 |
| HV986634.1 | FV528445.1 | FB717779.1 | GN032116.1 | GM623772.1 | BD397353.1 | HI178189.1 | AY967238.1 | CQ871222.1 |
| HV961494.1 | FV534444.1 | FB677441.1 | GN032084.1 | GM623740.1 | BD429495.1 | HI214559.1 | AY967206.1 | CQ869294.1 |
| HV969564.1 | FV534412.1 | FB676998.1 | GN032052.1 | GM827623.1 | BD397072.1 | HI214527.1 | AY967174.1 | CQ868904.1 |
| HV960533.1 | FV534089.1 | GM061159.1 | GN032020.1 | GM658919.1 | BD438711.1 | HI214461.1 | AY967142.1 | CQ868789.1 |
| HV960207.1 | HC046783.1 | DL260642.1 | GN031988.1 | GM658887.1 | BD406399.1 | HI214429.1 | AY967110.1 | CQ866774.1 |
| HV960147.1 | HC046751.1 | DL260068.1 | GN031956.1 | GM658855.1 | BD428040.1 | HI212998.1 | AY967078.1 | CQ859612.1 |
| AY658031.1 | HC046719.1 | DL259354.1 | GN031924.1 | GM658823.1 | BD437151.1 | HI212929.1 | AY967046.1 | CQ858160.1 |
| AY657999.1 | HC046035.1 | DL241387.1 | GN031892.1 | GM651535.1 | BD426878.1 | HI546346.1 | AY967014.1 | CQ858065.1 |
| AY657967.1 | HC045997.1 | DL241155.1 | GN031860.1 | GM651503.1 | BD394508.1 | HI546314.1 | AY966982.1 | CQ857738.1 |
| AY657935.1 | HC045965.1 | DL241794.1 | GN031828.1 | GM651471.1 | BD394002.1 | HI546144.1 | AY966950.1 | CQ857493.1 |
| AY657903.1 | HC045933.1 | DL240643.1 | GN031795.1 | GM651439.1 | BD453343.1 | HI586966.1 | CS055061.1 | CQ855926.1 |
| AY657871.1 | HC045901.1 | FB748397.1 | GN031698.1 | GM651407.1 | BD453314.1 | HI210992.1 | CS054188.1 | CQ855149.1 |
| AY657839.1 | HC045869.1 | FB748891.1 | GN031666.1 | GM644608.1 | BD453282.1 | HI210960.1 | CS052520.1 | CQ854242.1 |
| AY657775.1 | HC045837.1 | FB748750.1 | GN031634.1 | GM637418.1 | BD450468.1 | HI573169.1 | CS052400.1 | CS502636.1 |
| AY657743.1 | HC045805.1 | FB748136.1 | GM630987.1 | GM637386.1 | BD391409.1 | HI473103.1 | CS052331.1 | CS499914.1 |
| AY657711.1 | HC045773.1 | DL233419.1 | GM630955.1 | GM637354.1 | BD388860.1 | HI472901.1 | CS052298.1 | CS498363.1 |
| AY657679.1 | HC045741.1 | DL232138.1 | GM630923.1 | GM637322.1 | BD378633.1 | HI577462.1 | CS051001.1 | DD412378.1 |
| AY657647.1 | HC045709.1 | DL232100.1 | GM630898.1 | GM637290.1 | BD375867.1 | HI577382.1 | CS048040.1 | DD412322.1 |
| AY657615.1 | HC045679.1 | DL207311.1 | GM622116.1 | GM637258.1 | BD374647.1 | HI072385.1 | CS029245.1 | DD411932.1 |
| AY657583.1 | DM378904.1 | DL213337.1 | GM622084.1 | GM623657.1 | BD350064.1 | HI004361.1 | CS025749.1 | DD410174.1 |
| AY657551.1 | HC035518.1 | DL206826.1 | GM622052.1 | GM623625.1 | BD356406.1 | HI004233.1 | CS024019.1 | CS492094.1 |
| AY657519.1 | HC037094.1 | DL219988.1 | GM622020.1 | GM623593.1 | BD353555.1 | HI002270.1 | AX458598.1 | CS493057.1 |
| AY657487.1 | HC022132.1 | DL219502.1 | GM621988.1 | GM623561.1 | BD359753.1 | HI002233.1 | AX454040.1 | CS490930.1 |
| AY657455.1 | HC025551.1 | DL226843.1 | GM621956.1 | GM658710.1 | BD342461.1 | HI002183.1 | A08531.1 | CS490615.1 |
| AY657423.1 | HC025497.1 | DL206309.1 | GM635855.1 | GM658678.1 | BD341816.1 | HI002136.1 | AX443471.1 | CS489140.1 |
| AY657391.1 | HC025465.1 | DL230372.1 | GM635823.1 | GM658646.1 | BD325618.1 | HI002101.1 | AX440951.1 | CS486443.1 |
| AY657359.1 | HC025401.1 | DL230340.1 | GM635791.1 | GM658614.1 | BD325522.1 | HI000447.1 | AX430082.1 | CS482998.1 |
| AY657327.1 | HC024827.1 | DL097341.1 | GM635759.1 | GM658550.1 | BD314364.1 | HI000399.1 | AX429281.1 | CS482962.1 |
| AY657295.1 | HC010419.1 | DL121296.1 | GM635727.1 | GM651358.1 | BD313203.1 | HI000344.1 | AX428407.1 | CS482926.1 |
| AY657263.1 | HC010324.1 | DL121264.1 | GM635695.1 | GM644399.1 | BD319284.1 | HI208703.1 | AX418262.1 | CS482882.1 |
| AY657231.1 | HC007671.1 | DL121232.1 | GM635663.1 | GM644367.1 | CS611825.1 | HI553285.1 | AX417862.1 | CS482745.1 |
| AY657199.1 | HC007547.1 | DL121200.1 | GM630859.1 | GM644335.1 | CS604537.1 | HI553245.1 | AX411712.1 | DD401432.1 |
| AY657167.1 | HC007515.1 | DL101894.1 | GM630827.1 | GM637209.1 | CS604505.1 | HI553181.1 | AX399389.1 | DD401400.1 |
| AY657135.1 | HC010680.1 | DL101832.1 | GM630795.1 | GM637177.1 | CS604473.1 | HI583965.1 | AX397851.1 | DD405215.1 |
| AY657103.1 | DM375840.1 | DL101798.1 | GM630731.1 | GM637145.1 | CS604409.1 | HI568958.1 | AX392309.1 | DD405183.1 |
| AY657071.1 | DL098215.1 | DL101766.1 | GM630699.1 | GM637113.1 | CS604377.1 | HI002060.1 | AX391539.1 | DD405151.1 |
| AY657039.1 | DL098183.1 | DL101734.1 | GM621924.1 | GM637081.1 | CS604345.1 | HI002017.1 | AX380784.1 | DD405119.1 |
| M19550.1 | FB316734.1 | DL097320.1 | GM621892.1 | GM624928.1 | CS604313.1 | HI001934.1 | AX364546.1 | DD406785.1 |
| J02533.1 | FB317766.1 | DL097288.1 | GM621860.1 | GM624896.1 | CS604185.1 | HC491662.1 | AX364514.1 | DD402430.1 |
| M64694.1 | CS368444.1 | DL097256.1 | GM621828.1 | GM624864.1 | CS604089.1 | HC481647.1 | AX364482.1 | DD402398.1 |
| M20258.1 | CS368188.1 | DL097224.1 | GM621796.1 | GM624800.1 | DD272526.1 | HC481582.1 | AX364450.1 | DD107339.1 |
| HV549727.1 | CS368060.1 | DL097192.1 | GM621764.1 | GM632647.1 | CS299493.1 | FV533503.1 | AX364418.1 | DD106655.1 |
| HV549412.1 | CS367676.1 | DL097160.1 | GM657325.1 | GM632583.1 | DD251649.1 | FV531710.1 | AX364386.1 | DD092949.1 |
| HV549282.1 | DL080669.1 | DL095332.1 | GM657293.1 | GM632607.1 | DD257762.1 | FV531678.1 | AX364354.1 | DD118616.1 |
| HV549084.1 | DL075891.1 | DL095300.1 | GM657229.1 | GM658457.1 | CS288160.1 | FV523751.1 | AX364259.1 | DD091017.1 |
| HV548995.1 | DL079073.1 | DL095236.1 | GM657197.1 | GM658369.1 | CS287616.1 | FV522808.1 | AX364194.1 | DD104231.1 |

| | | | | | | | | |
|---|---|---|---|---|---|---|---|---|
| HV544323.1 | DL079041.1 | DL095204.1 | GM657165.1 | GM651177.1 | CS287399.1 | FV522632.1 | AX364145.1 | DD103429.1 |
| HV543617.1 | DL078957.1 | DL095172.1 | GM649973.1 | GM651145.1 | DD240740.1 | FV530190.1 | BD007225.1 | DD057936.1 |
| HV547408.1 | DL078925.1 | DL125339.1 | GM649941.1 | GM651113.1 | DD240581.1 | FV528446.1 | BD004706.1 | DD057904.1 |
| HV543498.1 | DL074251.1 | DL125307.1 | GM649877.1 | GM651081.1 | DD238900.1 | FV534535.1 | BD002015.1 | DD057872.1 |
| HV539679.1 | DL073056.1 | DL125275.1 | GM649845.1 | GM651049.1 | DD247580.1 | FV534413.1 | AX354807.1 | DD053300.1 |
| HV538934.1 | DL081571.1 | DL125243.1 | GM649813.1 | GM651017.1 | E41546.1 | FV534091.1 | HW381362.1 | DD052284.1 |
| HV538610.1 | DL013503.1 | DL125211.1 | GM643005.1 | GM650985.1 | E43941.1 | HC462768.1 | HB845746.1 | DD052226.1 |
| HV538454.1 | DL013471.1 | DL125179.1 | GM642973.1 | GM644122.1 | E47142.1 | HC461349.1 | HB845498.1 | DD041905.1 |
| HV537606.1 | DL013439.1 | DL116599.1 | GM642940.1 | GM644090.1 | E37377.1 | AY312992.1 | HB837331.1 | DD042875.1 |
| HV543294.1 | DL042645.1 | DL116567.1 | GM642908.1 | GM644058.1 | E35592.1 | AF422697.1 | HB845208.1 | DD039027.1 |
| HV542750.1 | DL042613.1 | DL116535.1 | GM642876.1 | GM636932.1 | E33329.1 | HC456314.1 | HB845051.1 | DD032283.1 |
| HV542718.1 | DL042581.1 | DL116503.1 | GM642844.1 | GM653186.1 | E36134.1 | HC453707.1 | HB836734.1 | DD031197.1 |
| HV542580.1 | DL034128.1 | DL116471.1 | GM642812.1 | GM646091.1 | E06032.1 | HC453675.1 | HB843947.1 | DD027312.1 |
| HV542181.1 | DL029835.1 | DL116439.1 | GM635630.1 | GM646059.1 | E04629.1 | HC453643.1 | HB843523.1 | DD023885.1 |
| HV541493.1 | DL029739.1 | DL111846.1 | GM635598.1 | GM646027.1 | E04412.1 | AF053825.1 | HB843190.1 | DD029392.1 |
| HV541036.1 | DL018308.1 | DL111814.1 | GM635566.1 | GM639128.1 | E03145.1 | HC452098.1 | HB835681.1 | CS118623.1 |
| HV515057.1 | DL018276.1 | DL111750.1 | GM635534.1 | GM626517.1 | E02973.1 | HC450051.1 | HB835253.1 | CS118523.1 |
| HV533065.1 | DL018244.1 | DL111718.1 | GM635502.1 | GM626485.1 | E01876.1 | FU774063.1 | HB842142.1 | CS118490.1 |
| HV532755.1 | DL018212.1 | DL111686.1 | GM635470.1 | GM626453.1 | E01671.1 | FU759996.1 | HB841602.1 | CS118457.1 |
| HV536538.1 | DL007500.1 | DL128897.1 | GM630664.1 | GM626421.1 | E01209.1 | FU759939.1 | HB834768.1 | CS118425.1 |
| HV534657.1 | DL005431.1 | DL128864.1 | GM630632.1 | GM626389.1 | E00700.1 | FU757921.1 | HB841012.1 | CS118392.1 |
| HV532451.1 | DJ493071.1 | DL128798.1 | GM630600.1 | GM626357.1 | E00575.1 | FU757631.1 | HB840468.1 | CS118359.1 |
| HV535997.1 | DJ491551.1 | DL128765.1 | GM621729.1 | GM660382.1 | E00305.1 | FU757599.1 | GM741849.1 | CS118325.1 |
| HV532380.1 | DJ446850.1 | DL128733.1 | GM621697.1 | DL089270.1 | E00091.1 | DM152673.1 | GM741817.1 | CS118291.1 |
| HV537345.1 | DJ446817.1 | DL116397.1 | GM621665.1 | DL089238.1 | DD102368.1 | DM156107.1 | GM716205.1 | CS118259.1 |
| HV535647.1 | DJ446632.1 | DL116365.1 | GM621633.1 | DL125929.1 | DD115472.1 | HB461515.1 | GM036706.1 | CS118227.1 |
| HI375891.1 | DJ437034.1 | DL111644.1 | GM621601.1 | DL125897.1 | CS104443.1 | HB455484.1 | DL233422.1 | CS118194.1 |
| HI375014.1 | DJ436972.1 | DL111612.1 | GM621569.1 | DL122559.1 | CS104226.1 | HB455146.1 | DL233389.1 | CS118160.1 |
| HC045504.1 | DJ436812.1 | DL111580.1 | FB505800.1 | DL122527.1 | CS102906.1 | HB444503.1 | DL206314.1 | CS118128.1 |
| HC045472.1 | DJ436735.1 | DL111548.1 | GM657117.1 | DL118081.1 | CS102778.1 | DM143454.1 | FB713742.1 | CS118096.1 |
| HC045440.1 | DJ444663.1 | DL111516.1 | GM657053.1 | DL118049.1 | CS102714.1 | DM121664.1 | FB713710.1 | CS118063.1 |
| HC045376.1 | DJ444558.1 | DL101702.1 | GM657021.1 | DL118017.1 | CS102682.1 | HB441223.1 | FB708167.1 | CS118029.1 |
| HC045344.1 | DJ438347.1 | DL101670.1 | GM656989.1 | DL103633.1 | CS102618.1 | HB432481.1 | FB707395.1 | CS117993.1 |
| HC045312.1 | DD402428.1 | DL101638.1 | GM656957.1 | DL099027.1 | CS102586.1 | HB416492.1 | FB669118.1 | CS117961.1 |
| HC045280.1 | DD402396.1 | DL101606.1 | GM656926.1 | DL098995.1 | CS102554.1 | HB416447.1 | FB666099.1 | CS114799.1 |
| GM637180.1 | DD402364.1 | DL101574.1 | GM656862.1 | DL098963.1 | CS102522.1 | HB412595.1 | FB670941.1 | CS114653.1 |
| GM637148.1 | DD402332.1 | DL101542.1 | GM656830.1 | DL088986.1 | CS102490.1 | HB394270.1 | FB676548.1 | CS106397.1 |
| GM637084.1 | DD402300.1 | DL097128.1 | GM656798.1 | DL117867.1 | CS088913.1 | HB394238.1 | FB676516.1 | CS104695.1 |
| GM637052.1 | DD402268.1 | DL097096.1 | GM656766.1 | DL117835.1 | CS084258.1 | HB397718.1 | FB706176.1 | CS102973.1 |
| GM624899.1 | DD402236.1 | DL097064.1 | GM649773.1 | DL021458.1 | CS079799.1 | HB397652.1 | FB704848.1 | CS102877.1 |
| GM624867.1 | DD402204.1 | DL097032.1 | GM649741.1 | DL021426.1 | CS078819.1 | GN360120.1 | FB701531.1 | CS102781.1 |
| GM624833.1 | DD405616.1 | DL095140.1 | GM649709.1 | DL021394.1 | CS063823.1 | GN360088.1 | FB701789.1 | CS102717.1 |
| GM632650.1 | DD405584.1 | DL095108.1 | GM649645.1 | DL017188.1 | CS061889.1 | GN356157.1 | FB665383.1 | CS102685.1 |
| GM632580.1 | DD405552.1 | DL095076.1 | GM649613.1 | DL017156.1 | AY967376.1 | GN360047.1 | FB654381.1 | CS102653.1 |
| GM632610.1 | DD405520.1 | DL095044.1 | GM642805.1 | DL017124.1 | AY967344.1 | GN360015.1 | FB654894.1 | CS102621.1 |
| GM741940.1 | DD405488.1 | DL095012.1 | GM642773.1 | DL017092.1 | AY967312.1 | DL035095.1 | FB573213.1 | CS102589.1 |
| GM658430.1 | DD401986.1 | DL094980.1 | GM642741.1 | DL017060.1 | AY967280.1 | DL035063.1 | FB295421.1 | CS102557.1 |

| | | | | | | | | |
|---|---|---|---|---|---|---|---|---|
| GM658340.1 | DD401925.1 | DL094948.1 | GM642709.1 | DL017028.1 | AY967248.1 | DL035031.1 | DJ052037.1 | CS102525.1 |
| GM651148.1 | DD401893.1 | DL106634.1 | GM642677.1 | DL049427.1 | AY967216.1 | DL030716.1 | DJ048783.1 | CS102493.1 |
| GM651116.1 | DD401861.1 | DL106602.1 | GM642645.1 | DL049363.1 | AY967184.1 | DL030684.1 | CS800125.1 | CS088983.1 |
| GM651084.1 | DD401829.1 | FW592640.1 | GM642613.1 | DL049331.1 | AY967152.1 | DL026747.1 | CS799999.1 | CS085788.1 |
| GM651052.1 | DD401621.1 | FW577838.1 | GM635335.1 | DL041082.1 | AY967120.1 | DL026715.1 | CS715916.1 | E63234.1 |
| GM651020.1 | DD401589.1 | FW577598.1 | GM635303.1 | DL037131.1 | AY967088.1 | DL026683.1 | CS713108.1 | E64760.1 |
| GM650988.1 | DD401557.1 | FW577077.1 | GM635271.1 | DL028664.1 | AY967056.1 | DL019504.1 | DJ045300.1 | E54537.1 |
| GM644189.1 | DD401525.1 | FW576804.1 | GM630433.1 | DL028632.1 | AY967024.1 | DL019472.1 | DJ044963.1 | BD000710.1 |
| GM644157.1 | DD401493.1 | FW576763.1 | GM660454.1 | DL028600.1 | AY966992.1 | DL019440.1 | DJ044948.1 | E58873.1 |
| GM644125.1 | CS479867.1 | FW576729.1 | GM660422.1 | DL028568.1 | AY966960.1 | DL019408.1 | DJ044900.1 | AX356492.1 |
| GM644093.1 | CS478264.1 | FW576603.1 | GM660390.1 | DL035666.1 | AY966928.1 | DL014909.1 | DJ028149.1 | AX350741.1 |
| GM644061.1 | CS468386.1 | FW575890.1 | GM660358.1 | DL035634.1 | AX587728.1 | DL014877.1 | DJ027931.1 | AX349184.1 |
| GM644029.1 | CS463952.1 | FW575451.1 | GM653171.1 | DL035602.1 | BD166164.1 | DL014845.1 | CS721652.1 | AX348863.1 |
| GM636935.1 | CS466045.1 | FW571681.1 | GM653139.1 | DL035570.1 | AX592715.1 | DL030459.1 | DD327517.1 | AX348464.1 |
| GM636871.1 | CS464533.1 | FW572726.1 | GM653107.1 | DL035538.1 | BD143209.1 | DL026503.1 | CS398364.1 | AX347582.1 |
| GM632463.1 | DD384321.1 | FW573700.1 | DL012513.1 | DL035506.1 | AX587895.1 | CS675518.1 | CS389323.1 | AX347417.1 |
| GM632431.1 | DD382827.1 | FW574730.1 | DL012481.1 | DL031502.1 | AX587641.1 | CS792473.1 | CS389290.1 | AX347319.1 |
| GM632399.1 | DD400151.1 | FW572685.1 | DL012449.1 | DL031470.1 | AX576929.1 | CS791962.1 | CS389248.1 | AX347243.1 |
| GM623532.1 | CS459133.1 | FW572653.1 | DL012417.1 | DL031438.1 | AX556849.1 | DJ042843.1 | CS389201.1 | AX347211.1 |
| GM623500.1 | DD361282.1 | FW572602.1 | DL048845.1 | DL031406.1 | AX556815.1 | DJ044925.1 | CS401296.1 | AX347173.1 |
| GM623468.1 | DD367610.1 | FW574394.1 | DL045427.1 | DL031374.1 | AX554145.1 | DJ044884.1 | CS406457.1 | DL095878.1 |
| GM623436.1 | CQ944187.1 | FW568748.1 | DL045395.1 | DL023561.1 | AX548056.1 | DJ028179.1 | CS408111.1 | DL141428.1 |
| GM623404.1 | CQ944155.1 | HI564100.1 | DL045363.1 | DL023529.1 | AX540474.1 | DJ028131.1 | CS407641.1 | DL114429.1 |
| GM623372.1 | CQ944123.1 | HI563700.1 | DL045331.1 | DL023497.1 | AX528972.1 | DJ030375.1 | CS380612.1 | DL114397.1 |
| GM658338.1 | CQ944091.1 | HI563666.1 | DL045299.1 | DL023465.1 | AX528909.1 | DJ029981.1 | DD308860.1 | DL114365.1 |
| GM643891.1 | CQ944059.1 | HI563648.1 | DL041251.1 | DL023433.1 | AX528460.1 | DJ026316.1 | DD291553.1 | DL104574.1 |
| GM643859.1 | CQ944027.1 | HI563625.1 | DL041219.1 | DL023401.1 | AX527904.1 | CS721623.1 | DD291027.1 | DL104542.1 |
| GM643827.1 | CQ943995.1 | HI563592.1 | DL041187.1 | DL020386.1 | AX525468.1 | CS691342.1 | CS376013.1 | DL104510.1 |
| GM636830.1 | CQ943963.1 | HI558082.1 | DL041155.1 | DL020322.1 | AX523908.1 | CS682283.1 | CS362755.1 | DL093926.1 |
| GM636798.1 | CQ943931.1 | HI508560.1 | DL041123.1 | DL015791.1 | A30507.1 | CS680799.1 | CS359950.1 | DL093894.1 |
| GM636766.1 | CQ943899.1 | HI583083.1 | DL041091.1 | DL015759.1 | AX179472.1 | CS691850.1 | CS359648.1 | DL141055.1 |
| GM632262.1 | CQ943867.1 | HI575434.1 | DL037204.1 | DL015727.1 | AX164119.1 | DJ008397.1 | CS362338.1 | DL126661.1 |
| GM632230.1 | CQ903046.1 | HI551158.1 | DL037172.1 | DL047089.1 | AX028016.1 | DJ008365.1 | BD269153.1 | DL123342.1 |
| GM632198.1 | CQ898681.1 | HI544149.1 | DL037140.1 | DL043075.1 | AX521669.1 | DJ008030.1 | BD268980.1 | DL123310.1 |
| GM632166.1 | CQ898641.1 | HI508259.1 | DL037108.1 | DL039098.1 | AX521531.1 | DJ004228.1 | BD265631.1 | DL093750.1 |
| GM632134.1 | CQ898609.1 | HI000056.1 | DL028737.1 | DL035474.1 | AX521499.1 | CS671203.1 | BD265530.1 | DL109152.1 |
| GM632102.1 | CQ898486.1 | HI000012.1 | DL028705.1 | DL035442.1 | AX512851.1 | CS646223.1 | BD263733.1 | DL109120.1 |
| GM623331.1 | CQ897414.1 | HI003600.1 | DL028673.1 | DL035410.1 | HW338178.1 | CS646191.1 | BD251781.1 | DL029527.1 |
| GM623299.1 | CQ895551.1 | HI003516.1 | DL028609.1 | DL035378.1 | HW338050.1 | CS644239.1 | BD247120.1 | DL029495.1 |
| GM623267.1 | CQ894658.1 | HI001842.1 | DL028577.1 | DL035346.1 | HW337794.1 | DD462044.1 | BD247023.1 | DL029463.1 |
| GM623235.1 | CQ893651.1 | HI001782.1 | DL024763.1 | DL031182.1 | HW337282.1 | DD461893.1 | BD246915.1 | DL046129.1 |
| GM741809.1 | CQ891736.1 | HI575284.1 | DL047322.1 | DL031150.1 | HW155984.1 | CS189620.1 | CS236410.1 | DL049704.1 |
| GM658323.1 | DM179525.1 | HI001686.1 | DL047290.1 | DL027245.1 | HW144721.1 | CS193221.1 | DD173113.1 | DL049672.1 |
| GM658291.1 | DM178918.1 | HI001624.1 | DL047258.1 | DL027213.1 | HW126687.1 | CS183149.1 | DD166532.1 | DL045664.1 |
| GM658259.1 | DM170845.1 | HI001582.1 | DL043436.1 | DL027181.1 | HW126457.1 | CS177182.1 | DD165453.1 | DL041689.1 |
| GM658227.1 | DM170813.1 | HI001533.1 | DL043404.1 | DL023369.1 | HW125397.1 | CS174650.1 | DD165117.1 | DL049631.1 |
| GM658195.1 | DM185716.1 | HI003367.1 | DL043372.1 | DL023337.1 | HW144366.1 | CS172326.1 | CS118638.1 | DL049599.1 |

| | | | | | | | | |
|---|---|---|---|---|---|---|---|---|
| GM658163.1 | DM188065.1 | HI003329.1 | DL043340.1 | DL023305.1 | HW144334.1 | CS173518.1 | CS118606.1 | DL033302.1 |
| GM636652.1 | DM187862.1 | HI071600.1 | DL043308.1 | DL023273.1 | HW124786.1 | CS166435.1 | CS118573.1 | DL033270.1 |
| GM658113.1 | HB559457.1 | HI505599.1 | DL043276.1 | DL023241.1 | HW120950.1 | CS159795.1 | CS118505.1 | DL033238.1 |
| GM658081.1 | DM160583.1 | HI464735.1 | DL039459.1 | DL023209.1 | HW120155.1 | CS159168.1 | CS118472.1 | DL033206.1 |
| GM658049.1 | DM164060.1 | HI464703.1 | DL039427.1 | DL023177.1 | HW081485.1 | CS155785.1 | CS118440.1 | DL033142.1 |
| GM658017.1 | DM163999.1 | HI464671.1 | DL039395.1 | DL020098.1 | HW101681.1 | CS146540.1 | CS118408.1 | DL029134.1 |
| GM627970.1 | DM163966.1 | HI464639.1 | DL039363.1 | DL020066.1 | HW097000.1 | CS141711.1 | CS118375.1 | DL029102.1 |
| GM715466.1 | DM163588.1 | HI464438.1 | DL039331.1 | DL020034.1 | HW096744.1 | CS141565.1 | CS118341.1 | DL023661.1 |
| GM661748.1 | DM163297.1 | HI528755.1 | DL039299.1 | CS603263.1 | HW065292.1 | CS141521.1 | CS118307.1 | DL023629.1 |
| GM654754.1 | DM162299.1 | HI464017.1 | DL035675.1 | CS603231.1 | HW065259.1 | CS141489.1 | CS118274.1 | DL023597.1 |
| GM654722.1 | GN033124.1 | HI549581.1 | DL035643.1 | CS603199.1 | HW065226.1 | CS140820.1 | CS118242.1 | DL015923.1 |
| GM654690.1 | GN032996.1 | HI542574.1 | DL035611.1 | CS603167.1 | HW061326.1 | CS138830.1 | CS118209.1 | DL015891.1 |
| GM654626.1 | GN032964.1 | HI463292.1 | DL035579.1 | CS603135.1 | HW064900.1 | CS130420.1 | DL014069.1 | DL015859.1 |
| GM654594.1 | GN032932.1 | HI463010.1 | DL035547.1 | CS603103.1 | HW064828.1 | CS123422.1 | DL014037.1 | DL047633.1 |
| GM654561.1 | GN032900.1 | HI462574.1 | DL035515.1 | CS603071.1 | HW056520.1 | CS122937.1 | DL026127.1 | DL047601.1 |
| GM647568.1 | GN032868.1 | HH716026.1 | DL031511.1 | CS603039.1 | HW060693.1 | CS122406.1 | DL026095.1 | DL047569.1 |
| GM640444.1 | GN032804.1 | HH713821.1 | DL031479.1 | CS603007.1 | HW062430.1 | CS106163.1 | DL026063.1 | DL047537.1 |
| GM627897.1 | GN032741.1 | HH961358.1 | DL031447.1 | CS602973.1 | HW060504.1 | CS106072.1 | DL026031.1 | DL039674.1 |
| GM627865.1 | GN032709.1 | HH961326.1 | DL031415.1 | CS602941.1 | HW062370.1 | CS180384.1 | DL025999.1 | DL039642.1 |
| GM627833.1 | GN032677.1 | HH961294.1 | DL031383.1 | CS602909.1 | HW042061.1 | CS179716.1 | DL025967.1 | DL039610.1 |
| GM627801.1 | GN032612.1 | HH961262.1 | DL031351.1 | CS602877.1 | HW042029.1 | CS179474.1 | DL018576.1 | DL035730.1 |
| GM871577.1 | GN032580.1 | HH961230.1 | DL027542.1 | CS602845.1 | HW043559.1 | CS327419.1 | DL018544.1 | DL039317.1 |
| GM870417.1 | GN032549.1 | HH975395.1 | DL027510.1 | CS602781.1 | HW041985.1 | CS323670.1 | DL018512.1 | DL035693.1 |
| GM661687.1 | GN032485.1 | HH975093.1 | DL027478.1 | CS602557.1 | HW041953.1 | CS331002.1 | DL018480.1 | DL035661.1 |
| GM661655.1 | GN032453.1 | HH964372.1 | DL027446.1 | CS602333.1 | HW041921.1 | CS330139.1 | DL018448.1 | DL035629.1 |
| GM661623.1 | GN032421.1 | HH982380.1 | DL027414.1 | CS611185.1 | HW041823.1 | CS329656.1 | DL018416.1 | DL023556.1 |
| GM661591.1 | GN032389.1 | HH974666.1 | DL027382.1 | CS610254.1 | HW049407.1 | CS322789.1 | DL014014.1 | DL023524.1 |
| GM661559.1 | GN032324.1 | HH974553.1 | DL023570.1 | CS602047.1 | HW049375.1 | DD261112.1 | DL013982.1 | DL023492.1 |
| GM661527.1 | GN032292.1 | HH981898.1 | DL023538.1 | CS602015.1 | HW049344.1 | DD260997.1 | DL013950.1 | DL023460.1 |
| GM654533.1 | GN032260.1 | HH998090.1 | DL023506.1 | CS055305.1 | HW049312.1 | DD272635.1 | DL013918.1 | DL023428.1 |
| GM654501.1 | GN032228.1 | HH998027.1 | DL023474.1 | AX822278.1 | HW049280.1 | DD213758.1 | DL013886.1 | DL023396.1 |
| GM654469.1 | GN032196.1 | HH997983.1 | DL023442.1 | AX817938.1 | HW049248.1 | DD213726.1 | DL013850.1 | DL020381.1 |
| GM654405.1 | GN032164.1 | HH999665.1 | DL023410.1 | AX814467.1 | HW041787.1 | DD213694.1 | DL030114.1 | DL020349.1 |
| GM654373.1 | GN032132.1 | HH997931.1 | DL023378.1 | AX814404.1 | HW040712.1 | DD213662.1 | DL030082.1 | DL020317.1 |
| GM655010.1 | GN032100.1 | HH997845.1 | DL020363.1 | AX814199.1 | HW050560.1 | DD213630.1 | DL030050.1 | DL015818.1 |
| GM654978.1 | GN032068.1 | HH997786.1 | DL020331.1 | AX805930.1 | HW047224.1 | DD213580.1 | DL030018.1 | DL015786.1 |
| GM044464.1 | GN032004.1 | HH997747.1 | DL020299.1 | AX799999.1 | HW053870.1 | DD213548.1 | DL029986.1 | DL015754.1 |
| GM837740.1 | GN031972.1 | HH999467.1 | DL020267.1 | AX799570.1 | HW049580.1 | DD218250.1 | DL029954.1 | DD431798.1 |
| GM755066.1 | GN031940.1 | HH999424.1 | DL020235.1 | AX796890.1 | HW040845.1 | DD215918.1 | DL046643.1 | CS540137.1 |
| GM043746.1 | GN031908.1 | HH999374.1 | DL020203.1 | AX795552.1 | HV985991.1 | DD215886.1 | DL046611.1 | CS543233.1 |
| GM642966.1 | GN031876.1 | HH997663.1 | DL015800.1 | BD192900.1 | HV985959.1 | DD215822.1 | DL046579.1 | CS539252.1 |
| GM642933.1 | GN031844.1 | HH997626.1 | DL015768.1 | BD188835.1 | HV985927.1 | DD215790.1 | DL046547.1 | CS537861.1 |
| GM642901.1 | GN031779.1 | HH997569.1 | DL015736.1 | BD187688.1 | HV450399.1 | DD215758.1 | DL046515.1 | CS537394.1 |
| GM642869.1 | GN031747.1 | HH997494.1 | DL015704.1 | AX787366.1 | HV453585.1 | DD212267.1 | DL046483.1 | CS503968.1 |
| GM630657.1 | GN031714.1 | HH997399.1 | DL015672.1 | AX773475.1 | HV450062.1 | DD211840.1 | DL042661.1 | CS502666.1 |
| GM630625.1 | GN031682.1 | HH999271.1 | DL011182.1 | AX772739.1 | HV455244.1 | DD192534.1 | DL042629.1 | CS498465.1 |
| GM630593.1 | GN031650.1 | HH999212.1 | DL011150.1 | AX752635.1 | HV453157.1 | DD192492.1 | DL042597.1 | DD418606.1 |

| | | | | | | | | |
|---|---|---|---|---|---|---|---|---|
| GM621658.1 | GN031618.1 | HC678958.1 | DL011118.1 | AX751528.1 | HV452929.1 | DD211106.1 | DL042565.1 | CS490611.1 |
| GM621626.1 | GN031554.1 | HC678788.1 | DL011086.1 | BD185379.1 | HV449119.1 | DD191627.1 | DL042533.1 | CS489138.1 |
| GM621594.1 | GN031522.1 | HC678560.1 | DL011054.1 | BD182999.1 | HV448796.1 | DD200154.1 | DL042501.1 | CS486441.1 |
| GM621562.1 | GN031393.1 | HC668139.1 | DL011022.1 | BD180860.1 | HV450670.1 | DD187322.1 | DL042469.1 | CS482996.1 |
| GM657142.1 | GN031361.1 | HC510271.1 | DL015623.1 | AX743954.1 | HV444706.1 | BD349457.1 | DL038673.1 | CS482924.1 |
| GM657078.1 | GN031329.1 | HC504601.1 | DL047226.1 | BD177616.1 | HV444163.1 | BD325716.1 | DL038641.1 | CS482878.1 |
| GM657046.1 | GN031296.1 | HC493110.1 | DL047194.1 | AX167247.1 | HV444096.1 | BD325613.1 | DL038609.1 | DD401430.1 |
| GM657014.1 | GN031264.1 | HC504197.1 | DL047162.1 | AX167088.1 | HV437574.1 | BD325514.1 | DL034304.1 | DD401398.1 |
| GM656982.1 | GN031232.1 | HC502881.1 | DL047130.1 | AX166292.1 | HV349318.1 | BD314358.1 | DL034272.1 | DD405213.1 |
| GM656950.1 | GN031200.1 | DL121768.1 | DL047098.1 | AX155097.1 | HV348781.1 | BD313709.1 | DL034240.1 | DD405181.1 |
| GM656919.1 | GN031168.1 | DL121736.1 | DL047066.1 | AX151105.1 | HV341346.1 | CQ795478.1 | DL034208.1 | DD405149.1 |
| GM656887.1 | GN031135.1 | DL121704.1 | DL043244.1 | AX146313.1 | HV341228.1 | CQ795446.1 | DL034144.1 | DD405117.1 |
| GM656791.1 | GN031103.1 | DL121672.1 | DL043212.1 | AX145726.1 | HV345268.1 | CQ794295.1 | DL034112.1 | DD408547.1 |
| GM656759.1 | GN031071.1 | DL121640.1 | DL043180.1 | AX145694.1 | HV344944.1 | CQ792546.1 | DL029915.1 | DD406775.1 |
| GM649766.1 | GN031039.1 | DL121608.1 | DL043148.1 | AX145662.1 | HV347057.1 | CQ788637.1 | DL029883.1 | CS274786.1 |
| GM649638.1 | GN031007.1 | DL087749.1 | DL043116.1 | AX145630.1 | HV344668.1 | CQ787445.1 | DL029819.1 | CS263129.1 |
| GM642702.1 | GN030975.1 | DL087717.1 | DL043084.1 | AX145566.1 | HV339984.1 | CQ787405.1 | DL029787.1 | CS252534.1 |
| GM642670.1 | GN030944.1 | DL087685.1 | DL039267.1 | AX145534.1 | HV339920.1 | CQ787365.1 | DL029755.1 | CS249818.1 |
| GM642638.1 | GN030880.1 | DL087653.1 | DL039235.1 | AX145502.1 | HV339888.1 | CQ787297.1 | DL025945.1 | CS250605.1 |
| GM635328.1 | GN030848.1 | DL087621.1 | DL039203.1 | AX145470.1 | HV339660.1 | CQ787265.1 | DL025913.1 | CS248882.1 |
| GM635296.1 | GN030816.1 | DL087589.1 | DL039171.1 | AX145438.1 | HI642555.1 | CQ787233.1 | DL025881.1 | CS244157.1 |
| GM630490.1 | GN030784.1 | DL125520.1 | DM370766.1 | AX145406.1 | HI638969.1 | CQ786929.1 | DL025849.1 | CS244237.1 |
| GM630458.1 | DM008342.1 | DL125488.1 | DM370668.1 | AX145373.1 | HI637692.1 | CQ784709.1 | DL025817.1 | CS244205.1 |
| GM630426.1 | DM006937.1 | DL125456.1 | DM370636.1 | AX145341.1 | HI380640.1 | CQ784645.1 | DL025785.1 | CS243161.1 |
| GM630394.1 | DM000510.1 | DL125424.1 | DM370604.1 | AX145309.1 | HI380137.1 | CQ778527.1 | DL069497.1 | CS237719.1 |
| GM630362.1 | DM005043.1 | DL125392.1 | DM370572.1 | AX145277.1 | HI379167.1 | CQ778495.1 | DL018388.1 | CS203487.1 |
| GM630330.1 | DM003490.1 | DL125360.1 | DM370540.1 | AX145245.1 | HI378153.1 | CQ774400.1 | DL018356.1 | AX142855.1 |
| GM621523.1 | DL106883.1 | DL121571.1 | DM370508.1 | AX145213.1 | HI378121.1 | CQ772555.1 | DL018324.1 | AX142599.1 |
| GM739515.1 | DL106852.1 | DL121539.1 | DM370476.1 | AX145181.1 | HI376607.1 | CQ771249.1 | DL018292.1 | AX142469.1 |
| GM649464.1 | DL106820.1 | DL121507.1 | DM367600.1 | AX145149.1 | HI375879.1 | CQ759766.1 | DL018260.1 | AX142341.1 |
| GM649432.1 | DL106788.1 | DL121475.1 | DM371971.1 | AX145117.1 | HI369602.1 | CQ757695.1 | DL018228.1 | AX142277.1 |
| GM649400.1 | DL091230.1 | DL121443.1 | DM365912.1 | AX145085.1 | HI369099.1 | CQ756602.1 | DL013826.1 | AX142213.1 |
| GM670040.1 | DL091198.1 | DL121411.1 | DM204188.1 | AX145053.1 | HI424127.1 | CQ754498.1 | DL013794.1 | AX142019.1 |
| GM656535.1 | DL087230.1 | DL117199.1 | HC003045.1 | AX145021.1 | HI424095.1 | CQ754031.1 | DL013762.1 | HW066538.1 |
| GM656503.1 | DL087198.1 | DL117167.1 | HC001729.1 | AX144989.1 | HI424063.1 | AX962027.1 | DL013730.1 | HW083286.1 |
| GM656471.1 | DL102091.1 | DL117135.1 | HB865984.1 | AX144957.1 | HI423837.1 | AX960393.1 | DL013666.1 | HW083254.1 |
| GM656439.1 | DL102059.1 | DL117103.1 | HB865372.1 | AX144924.1 | HI423773.1 | AX960361.1 | DL005428.1 | HW083164.1 |
| FB717763.1 | DL102027.1 | DL117071.1 | HB865026.1 | AX144892.1 | HI422919.1 | AX958540.1 | DL000692.1 | HW082951.1 |
| FB715074.1 | DL101910.1 | DL117039.1 | HB864978.1 | AX144860.1 | HC920299.1 | AX958101.1 | DL005862.1 | HW099485.1 |
| FB677409.1 | DL101878.1 | DL112451.1 | HB864986.1 | AX144828.1 | HD057715.1 | AX958062.1 | DL000613.1 | HW103589.1 |
| GM061144.1 | DL101814.1 | DL112419.1 | HB864930.1 | AX144796.1 | HD057679.1 | AX937799.1 | DL000581.1 | HW103517.1 |
| DL262713.1 | DL101782.1 | DL112387.1 | HB864898.1 | AX144764.1 | U31739.1 | AX924177.1 | CS494657.1 | HW103130.1 |
| DL260061.1 | DL101750.1 | DL112355.1 | HB864866.1 | AX144732.1 | M22872.1 | AX923398.1 | DD418807.1 | HW102797.1 |
| DL258271.1 | DL095220.1 | DL112323.1 | HB864834.1 | AX144700.1 | GM637780.1 | AX923145.1 | DD418651.1 | HW102765.1 |
| DL242130.1 | DL121114.1 | DL112291.1 | HB864802.1 | AX144668.1 | GM633212.1 | AX141955.1 | DD418590.1 | HW102307.1 |
| DL240953.1 | DL111862.1 | DL143740.1 | HB864770.1 | AX144636.1 | GM633180.1 | AX141889.1 | DD412392.1 | HW065325.1 |
| DL240635.1 | DL111830.1 | DL107277.1 | HB859192.1 | AX144595.1 | GM677242.1 | AX141697.1 | DD411676.1 | HW065227.1 |

| | | | | | | | | |
|---|---|---|---|---|---|---|---|---|
| FB674307.1 | DL111798.1 | DL107245.1 | HB866106.1 | AX144339.1 | GM651769.1 | AX141633.1 | DD411480.1 | HW058484.1 |
| DL236684.1 | HW302529.1 | DL107213.1 | HC000879.1 | AX144275.1 | GM644809.1 | AX141441.1 | DD414342.1 | HW064829.1 |
| DL233412.1 | HW302344.1 | DL107181.1 | HB999511.1 | AX144209.1 | GM636535.1 | AX141377.1 | DD414271.1 | HW057847.1 |
| DL232125.1 | HW295269.1 | DL107149.1 | HB977450.1 | AX144145.1 | GM636503.1 | AX138283.1 | DD417773.1 | HW056521.1 |
| DL213255.1 | HW302059.1 | DL107117.1 | HB976614.1 | AX143951.1 | GM636471.1 | AX137843.1 | DD414037.1 | HW060694.1 |
| DL206286.1 | HW302015.1 | DL102504.1 | HB976183.1 | AX143823.1 | GM650582.1 | AX134427.1 | CS491929.1 | HW062396.1 |
| FB714903.1 | HW295200.1 | DL102472.1 | DM202498.1 | AX143631.1 | GM631836.1 | AF366904.1 | CS490475.1 | HW060506.1 |
| FB713796.1 | HW285861.1 | DL102440.1 | DM193400.1 | AX143567.1 | GM631804.1 | AX119323.1 | CS488379.1 | HW062371.1 |
| FB708009.1 | HW306954.1 | DL102408.1 | DM196032.1 | AX143503.1 | GM622841.1 | AX108460.1 | CS486792.1 | HW062307.1 |
| FB672286.1 | HW294134.1 | DL102376.1 | DM195095.1 | AX143439.1 | GM657798.1 | AX108243.1 | CS485298.1 | HW062210.1 |
| FB672139.1 | HW291256.1 | DL102344.1 | DM198846.1 | AX143375.1 | GM704070.1 | AX107119.1 | CS483998.1 | HW056158.1 |
| DL125204.1 | HW291159.1 | DL097731.1 | HB806119.1 | AX143311.1 | GM657616.1 | AX101164.1 | CS482943.1 | HW042062.1 |
| DL125172.1 | HW291112.1 | DL097699.1 | HB822113.1 | AX142927.1 | GM650360.1 | AX100343.1 | CS482906.1 | HW042030.1 |
| DL111839.1 | HW291062.1 | DL097667.1 | HB805793.1 | AX142863.1 | GM635067.1 | AX099344.1 | CS482861.1 | HW043560.1 |
| DL111807.1 | HW291030.1 | DL097635.1 | HB839910.1 | AX142671.1 | GM635064.1 | AX097510.1 | CS482818.1 | HH935024.1 |
| DL111775.1 | HW290966.1 | DL097603.1 | HB847930.1 | AX142349.1 | GM634968.1 | AX093090.1 | CS482781.1 | HH834131.1 |
| DL128890.1 | HW290934.1 | DL097571.1 | HB847701.1 | AX142285.1 | GM669844.1 | AX088783.1 | CS482304.1 | HH833867.1 |
| DL116422.1 | HW290903.1 | DL097539.1 | HB839418.1 | AX142221.1 | GM656323.1 | AX088739.1 | CS485115.1 | FW390824.1 |
| DL116358.1 | HW290871.1 | DL095512.1 | HB847416.1 | AX141899.1 | GM656291.1 | AX085346.1 | CS510544.1 | FW392867.1 |
| DL111605.1 | HW290614.1 | DL095480.1 | HB839180.1 | AX141769.1 | GM656259.1 | AX081487.1 | CS502925.1 | HH821447.1 |
| DL111573.1 | HV335462.1 | DL095448.1 | HB838776.1 | AX141641.1 | GM656227.1 | AX078901.1 | CS502783.1 | HH821124.1 |
| DL111541.1 | HV322200.1 | DL095416.1 | HB805285.1 | AX141513.1 | GM656195.1 | AX077854.1 | CS502717.1 | HH822008.1 |
| DL111509.1 | HV321820.1 | DL095384.1 | HB847001.1 | AX141449.1 | GM656163.1 | AX076579.1 | CS502681.1 | HH821966.1 |
| DL111477.1 | HV333657.1 | DL095352.1 | HB838589.1 | HW248919.1 | GM648958.1 | AX076207.1 | CS502649.1 | FW381494.1 |
| DL101695.1 | HV323568.1 | DL091538.1 | HB840360.1 | HW244250.1 | GM634551.1 | AX069271.1 | CS502617.1 | HD088209.1 |
| DL101663.1 | HV187412.1 | DL091506.1 | HB846169.1 | HW237890.1 | GM634519.1 | AX063642.1 | CS502411.1 | FW345500.1 |
| DL097121.1 | HV208648.1 | DL091474.1 | HB815013.1 | HW237849.1 | GM634487.1 | AX061430.1 | CS502423.1 | FW345036.1 |
| DL097089.1 | HV236071.1 | DL091442.1 | HB809087.1 | HW248685.1 | GM629682.1 | AX060455.1 | CS501258.1 | FW344680.1 |
| DL097057.1 | HV306172.1 | DL091378.1 | HB837770.1 | HW242469.1 | GM879657.1 | AX059769.1 | CS499058.1 | FW343726.1 |
| DL095133.1 | HV305909.1 | DL087485.1 | HB808933.1 | HW242313.1 | GM840855.1 | AX057958.1 | CS499025.1 | FW343594.1 |
| DL095101.1 | HV302966.1 | DL087453.1 | HB845691.1 | HW237056.1 | FB712024.1 | AX052944.1 | CS498888.1 | FW343212.1 |
| DL095037.1 | HV302934.1 | DL087421.1 | HB845415.1 | HW243426.1 | GM723126.1 | AX047079.1 | CS498435.1 | HC924160.1 |
| DL095005.1 | HV308613.1 | DL087389.1 | HB837211.1 | HW243089.1 | GM042091.1 | AX046137.1 | CS495627.1 | HC922998.1 |
| DL094973.1 | HV304161.1 | DL112231.1 | HB813989.1 | HW243016.1 | GM041708.1 | AX042390.1 | DD413372.1 | HC921523.1 |
| DL106691.1 | FW552196.1 | DL112199.1 | HB845200.1 | HW241451.1 | GM040839.1 | AX040767.1 | DD418806.1 | HC920681.1 |
| DL106659.1 | FW552158.1 | DL112167.1 | HB844958.1 | DL027844.1 | GM040260.1 | AX040181.1 | DD418650.1 | HD068875.1 |
| DL106563.1 | FW561811.1 | DL112135.1 | HB844281.1 | DL027812.1 | FB709046.1 | AX039519.1 | DD412337.1 | HD063943.1 |
| DL106531.1 | FW555588.1 | DL112103.1 | HB836577.1 | DL027780.1 | CS728605.1 | AX037319.1 | DD411675.1 | HC920302.1 |
| DL106499.1 | FW563143.1 | DL112071.1 | HB835902.1 | DL023872.1 | CS728573.1 | HW350671.1 | DD411479.1 | HD033229.1 |
| DL128729.1 | FW556523.1 | DL107068.1 | HB843171.1 | DL023840.1 | CS727350.1 | HW340207.1 | DD414340.1 | HD057680.1 |
| AX011346.1 | FW561690.1 | DL107036.1 | HB835673.1 | DL023808.1 | GM836380.1 | HW339897.1 | DD414270.1 | HD053533.1 |
| AX010940.1 | FW561658.1 | DL106972.1 | GN033299.1 | DL035894.1 | GM949535.1 | HW347915.1 | DD417772.1 | U31093.1 |
| AX010374.1 | FW562570.1 | DL106940.1 | GN033235.1 | DL031538.1 | GM006113.1 | HW363493.1 | DD414035.1 | M23079.1 |
| AX009286.1 | FW505272.1 | DL106908.1 | GN033203.1 | CS682280.1 | FB985404.1 | HW307860.1 | CS491927.1 | AY538748.1 |
| AX006384.1 | FW508150.1 | DL102295.1 | GN033139.1 | CS674187.1 | GM879372.1 | HW307796.1 | CS491742.1 | HC918496.1 |
| AX003041.1 | FW508093.1 | DL102263.1 | GN033107.1 | CS691849.1 | GM879311.1 | HW307758.1 | CS485296.1 | FW335020.1 |
| A12712.1 | FW553149.1 | DL106464.1 | GN033075.1 | DJ008396.1 | GM839627.1 | HW307726.1 | CS485176.1 | FW339656.1 |

| | | | | | | | | |
|---|---|---|---|---|---|---|---|---|
| A48483.1 | FW556656.1 | DL106432.1 | GN033043.1 | DJ008364.1 | GM841778.1 | HW307694.1 | CS483878.1 | FW337320.1 |
| A38676.1 | FW560800.1 | DL106356.1 | GN033011.1 | DJ008332.1 | FB721300.1 | HW315905.1 | CS482942.1 | FW337031.1 |
| A35740.1 | HI931502.1 | DL106392.1 | GN032979.1 | DJ004227.1 | FB718059.1 | HW315721.1 | CS482905.1 | FW340879.1 |
| A35704.1 | HI936407.1 | DL106336.1 | GN032947.1 | DJ004191.1 | FB702167.1 | HW315680.1 | CS482860.1 | HC887695.1 |
| A29519.1 | HI583961.1 | DL106304.1 | GN032915.1 | CS671085.1 | GM061223.1 | HW315589.1 | CS482817.1 | HC887338.1 |
| A29437.1 | HI577328.1 | DL136035.1 | GN032851.1 | CS669878.1 | DL128879.1 | HW315554.1 | CS482777.1 | HC046937.1 |
| A34039.1 | HI568949.1 | DL128512.1 | GN032819.1 | CS647415.1 | DL128812.1 | HW315446.1 | CS482303.1 | HC046905.1 |
| A31572.1 | HI003875.1 | DL128443.1 | GN032787.1 | CS646222.1 | DL128747.1 | HW315161.1 | CS485114.1 | HC046877.1 |
| A30328.1 | HI002055.1 | DL124749.1 | GN032755.1 | CS646190.1 | DL116283.1 | HW315097.1 | DD404996.1 | HC046845.1 |
| A32025.1 | HI001966.1 | DL124717.1 | GN032724.1 | DD460648.1 | DL111658.1 | HW315065.1 | DD404964.1 | HC046813.1 |
| A27720.1 | HI000298.1 | DL124685.1 | GN032692.1 | DD459406.1 | DL111626.1 | HW314823.1 | DD404932.1 | HC046781.1 |
| A27456.1 | HI000254.1 | DL124653.1 | GN032660.1 | DD458775.1 | DL111594.1 | HW314769.1 | DD404900.1 | HC046749.1 |
| A16277.1 | HI000212.1 | DL124621.1 | GN032627.1 | DD453848.1 | DL111562.1 | HW115420.1 | DD404868.1 | HC046717.1 |
| A20274.1 | HI000154.1 | DL124589.1 | GN032595.1 | CS632077.1 | DL101716.1 | HW099804.1 | DD401445.1 | HC039691.1 |
| A20746.1 | HI000122.1 | DL124557.1 | GN032532.1 | CS631279.1 | DL101684.1 | HW084937.1 | DD401413.1 | HC046065.1 |
| A14115.1 | HI137428.1 | DL118520.1 | GN032500.1 | CS631200.1 | DL101652.1 | HW097685.1 | DD401200.1 | HC046033.1 |
| A08536.1 | HI204488.1 | DL118488.1 | GN032468.1 | CS631136.1 | DL101620.1 | HW065510.1 | DD405196.1 | HC045963.1 |
| A03894.1 | HI203116.1 | DL118456.1 | GN032436.1 | CS627835.1 | DL106552.1 | HV956202.1 | DD405164.1 | HC045899.1 |
| A32069.1 | HI538841.1 | DL114028.1 | GN032404.1 | CS627182.1 | DL106520.1 | HV951102.1 | DD405100.1 | HC045867.1 |
| A32028.1 | HI552116.1 | DL113996.1 | GN032372.1 | CS632201.1 | DL128585.1 | HV953056.1 | DD405068.1 | HC045835.1 |
| A30530.1 | HI470708.1 | DL113964.1 | GN032339.1 | CS632149.1 | DL112681.1 | HV946231.1 | E49300.1 | HC045803.1 |
| A28877.1 | HI470591.1 | DL113932.1 | GN032307.1 | CS623696.1 | FB328919.1 | HV944606.1 | E40574.1 | HC045771.1 |
| A28014.1 | HI559252.1 | DL113900.1 | GN032243.1 | CS626354.1 | CS367332.1 | HV943091.1 | E33085.1 | HC045739.1 |
| A25933.1 | HI568903.1 | DL113868.1 | GN032179.1 | CS133151.1 | CS368244.1 | HV779874.1 | E32391.1 | HC045677.1 |
| A04447.1 | HI568844.1 | DL109067.1 | GN032147.1 | CS123420.1 | CS368116.1 | HV767842.1 | E06933.1 | DM378697.1 |
| A26070.1 | HI564245.1 | DL109035.1 | GN032115.1 | CS122936.1 | CS367988.1 | HW041986.1 | E05677.1 | HC037090.1 |
| A24756.1 | HI564117.1 | DL109003.1 | GN032051.1 | CS122405.1 | CS367860.1 | HW041954.1 | E04532.1 | HC036986.1 |
| A22540.1 | HI583136.1 | DL108971.1 | HV449914.1 | CS106162.1 | CS367668.1 | HW041922.1 | E04197.1 | HC026834.1 |
| A22084.1 | HI563641.1 | DL108939.1 | HV445633.1 | CS180382.1 | DL075247.1 | HW041824.1 | E04046.1 | HC025527.1 |
| A16251.1 | HI563609.1 | DL108907.1 | HV451088.1 | CS179712.1 | DL078921.1 | HW049408.1 | E03308.1 | HC025495.1 |
| A12821.1 | HI563574.1 | DL104301.1 | HV436860.1 | CS179215.1 | DL074234.1 | HW049376.1 | E02925.1 | HC025463.1 |
| A21629.1 | HI558014.1 | DL104269.1 | HV444630.1 | CS327374.1 | DL042641.1 | HW049345.1 | E02679.1 | HC025431.1 |
| A21069.1 | HI508543.1 | DL104237.1 | HV444004.1 | CS326341.1 | DL042577.1 | HW049313.1 | E02382.1 | DL016402.1 |
| A21127.1 | HI538012.1 | DL104205.1 | HV438480.1 | CS323669.1 | DL034124.1 | HW049281.1 | E02220.1 | DL016370.1 |
| A18331.1 | HI000033.1 | DL104173.1 | HV437638.1 | CS330975.1 | DL005589.1 | HW049249.1 | E02080.1 | DL016306.1 |
| A14605.1 | HI003670.1 | DL104141.1 | HV443089.1 | CS330138.1 | DJ491547.1 | HW046211.1 | E01803.1 | DL016242.1 |
| A16020.1 | HI003622.1 | DL104109.1 | HV349087.1 | CS329500.1 | DJ446846.1 | HW041788.1 | E01693.1 | DL014442.1 |
| A15705.1 | HI003583.1 | DL099663.1 | HV351587.1 | DD271403.1 | DJ432282.1 | HW040713.1 | E01607.1 | DL009341.1 |
| A14593.1 | HI003545.1 | DL099599.1 | HV334776.1 | DD261111.1 | DJ445559.1 | HW043070.1 | E01380.1 | DL009309.1 |
| A13637.1 | HI003495.1 | DL099567.1 | FZ418105.1 | DD259339.1 | DJ445127.1 | HW050565.1 | E00759.1 | DL009277.1 |
| HV600379.1 | HI001822.1 | DL099535.1 | FZ421004.1 | CS297354.1 | CS810625.1 | HW053732.1 | E00635.1 | DL009245.1 |
| HV585090.1 | HI001764.1 | DL092715.1 | FZ420791.1 | DD257781.1 | CS812986.1 | HV985992.1 | E00441.1 | DL009213.1 |
| HV584890.1 | HI544127.1 | DL092683.1 | FZ417475.1 | CS288162.1 | CS811260.1 | HV985960.1 | E00338.1 | DL018703.1 |
| HV584786.1 | HI465300.1 | DL092651.1 | FZ417041.1 | CS287617.1 | DJ021064.1 | HV985928.1 | E00221.1 | DL018639.1 |
| HV579124.1 | HI001607.1 | DL092587.1 | FZ416710.1 | CS287400.1 | CS803356.1 | HV985896.1 | DD146389.1 | DL018607.1 |
| HV579005.1 | HI001561.1 | DL088972.1 | FZ419864.1 | DD240741.1 | CS802331.1 | HW028871.1 | DD115368.1 | DL014211.1 |
| HV592488.1 | HI001508.1 | DL088940.1 | FZ419829.1 | DD235034.1 | DJ052621.1 | HV984258.1 | DD146290.1 | DL014179.1 |

| | | | | | | | | |
|---|---|---|---|---|---|---|---|---|
| HV592419.1 | HI003386.1 | DL088908.1 | FZ419795.1 | E41547.1 | DJ046969.1 | HV961483.1 | DD146258.1 | DL014147.1 |
| HV578518.1 | HI003348.1 | DL103283.1 | FZ416529.1 | E39989.1 | DJ046498.1 | HV959617.1 | DD146203.1 | CS693528.1 |
| HV577339.1 | HI003307.1 | DL103251.1 | FW591962.1 | E47180.1 | HW160398.1 | HV963586.1 | DD086343.1 | DJ008394.1 |
| HV598704.1 | HI550375.1 | DL103219.1 | FW591725.1 | CS118782.1 | HW155788.1 | HV969950.1 | DD069886.1 | DJ008362.1 |
| HV582973.1 | HI464687.1 | DL107971.1 | FW591104.1 | CS118749.1 | HW155701.1 | HV962319.1 | DD143282.1 | DJ004225.1 |
| HV601498.1 | HI464655.1 | DL107939.1 | FW590904.1 | CS118717.1 | HW155669.1 | HV503173.1 | DD112410.1 | DJ004189.1 |
| HV581980.1 | HC195216.1 | DL107907.1 | FW590735.1 | CS118621.1 | HW144933.1 | HV503141.1 | DD084342.1 | DJ003339.1 |
| HV601355.1 | HV515475.1 | DL092449.1 | FW590307.1 | CS118588.1 | HW155554.1 | HV502777.1 | DD098192.1 | CS671015.1 |
| HV601183.1 | HV515443.1 | DL092417.1 | FW590117.1 | CS118553.1 | HW155433.1 | HV502745.1 | DD141298.1 | CS669874.1 |
| HV601077.1 | HV515411.1 | DL092385.1 | FW593456.1 | CS118487.1 | HW155299.1 | HV502713.1 | DD153588.1 | CS673647.1 |
| HV574871.1 | HV515379.1 | DL088770.1 | FW592638.1 | CS118455.1 | HW158426.1 | HV502553.1 | DD158452.1 | CS647410.1 |
| HV574235.1 | HV515347.1 | DL088738.1 | FW577834.1 | CS118423.1 | HW154674.1 | HV505385.1 | DD158420.1 | CS646220.1 |
| HV585644.1 | HV515283.1 | DL088706.1 | FW577581.1 | CS118390.1 | HW158027.1 | HV505290.1 | DD157801.1 | CS646188.1 |
| HV571049.1 | HV515251.1 | DL088674.1 | FW576599.1 | CS118356.1 | HW154092.1 | HV492443.1 | DD155788.1 | DD460646.1 |
| HV570487.1 | HV511218.1 | DL088642.1 | FW575888.1 | CS118323.1 | HV752932.1 | HV450400.1 | DD152107.1 | DD460136.1 |
| HV560067.1 | HV507916.1 | DL088610.1 | FW575364.1 | CS118289.1 | HV759257.1 | HV453586.1 | DD082383.1 | DD459773.1 |
| HV555947.1 | HV509333.1 | DL088578.1 | FW571673.1 | CS118257.1 | HV758699.1 | HV450063.1 | DD096304.1 | DD458773.1 |
| HV302078.1 | HV508678.1 | DL112956.1 | FW572724.1 | CS118225.1 | HV755045.1 | HV455245.1 | DD081764.1 | DD453846.1 |
| HV030234.1 | HV508643.1 | DL098505.1 | FW573880.1 | CS118191.1 | HV745725.1 | HV451941.1 | DD123300.1 | DD456662.1 |
| HV037876.1 | HV508611.1 | DL098473.1 | FW574728.1 | CS118126.1 | HV745331.1 | HV453158.1 | DD148510.1 | CS632067.1 |
| HV036141.1 | HV508579.1 | DL098441.1 | FW572683.1 | CS118094.1 | HV749318.1 | HV452807.1 | DD147797.1 | CS631268.1 |
| FZ435997.1 | HV508547.1 | DL098409.1 | FW573478.1 | CS118027.1 | HV750935.1 | HV449121.1 | DD147765.1 | CS631196.1 |
| FZ430242.1 | HV508515.1 | DL098377.1 | HH759215.1 | CS117991.1 | HV747488.1 | HV448825.1 | DD147733.1 | CS642142.1 |
| FZ423910.1 | HV508487.1 | DL098345.1 | HH759183.1 | CS117959.1 | HV753739.1 | HV450737.1 | DD147701.1 | CS627831.1 |
| FZ436951.1 | HV512289.1 | DL092335.1 | HH759151.1 | CS114718.1 | HV753622.1 | HV444456.1 | HC292612.1 | CS625508.1 |
| FZ432330.1 | HV512257.1 | DL092271.1 | HH759119.1 | CS111460.1 | HV743328.1 | HV437575.1 | HC288877.1 | DD137431.1 |
| FZ416172.1 | HV512225.1 | DL092207.1 | HH759087.1 | CS106395.1 | HV742532.1 | HV437534.1 | HC288844.1 | DD132340.1 |
| FZ413163.1 | HV512193.1 | DL092175.1 | HH759055.1 | CS102971.1 | HV502610.1 | HV348782.1 | HC295596.1 | DD135015.1 |
| FZ418924.1 | HI583959.1 | DL088560.1 | HH759023.1 | CS102907.1 | HV502578.1 | HV341347.1 | HC295454.1 | DD106567.1 |
| FZ411766.1 | HI565600.1 | DL088528.1 | HH758991.1 | CS102811.1 | HV502546.1 | HV341229.1 | HC288710.1 | DD092945.1 |
| FZ411664.1 | HI003873.1 | DL088496.1 | HH758959.1 | CS102747.1 | HV502514.1 | FZ417529.1 | HC292062.1 | DD117038.1 |
| FZ416945.1 | HI002053.1 | DL088464.1 | HH758927.1 | CS102715.1 | HV502482.1 | FZ419847.1 | HC291894.1 | DD102595.1 |
| FZ419855.1 | HI001964.1 | DL088432.1 | HH758895.1 | CS102683.1 | HV502450.1 | FZ419814.1 | HC299197.1 | DD057932.1 |
| FZ419820.1 | HI000296.1 | DL088400.1 | HH758143.1 | CS102651.1 | HV450361.1 | FW582246.1 | HC299161.1 | DD057900.1 |
| FZ419788.1 | HI000252.1 | DL117800.1 | HH757439.1 | CS102619.1 | HV452052.1 | FW590756.1 | HC291630.1 | DD057868.1 |
| FW580720.1 | HI000209.1 | DL117768.1 | HH756367.1 | CS102587.1 | HV449937.1 | FW590722.1 | HC294398.1 | DD057840.1 |
| HH757464.1 | HI000152.1 | DL117736.1 | HH756192.1 | CS102555.1 | HV453149.1 | FW590294.1 | HC293834.1 | DD057056.1 |
| HH757432.1 | HI000120.1 | DL117704.1 | HI401094.1 | CS102523.1 | HV449385.1 | FW593388.1 | HC293654.1 | DD053909.1 |
| HI401444.1 | HI520487.1 | DL117672.1 | HI642929.1 | CS102491.1 | HV451237.1 | HQ161060.1 | HC290701.1 | DD053296.1 |
| HI401235.1 | HI204486.1 | DL117640.1 | HI641841.1 | BD014222.1 | HV445103.1 | FW577632.1 | HC208418.1 | DD041901.1 |
| HI401086.1 | HI180936.1 | DL122099.1 | HI641297.1 | BD014041.1 | HV344860.1 | FW577311.1 | HC289885.1 | DD038713.1 |
| HI644951.1 | HI204206.1 | DL122035.1 | HI640753.1 | BD263222.1 | HI001502.1 | FW575564.1 | HC289851.1 | DD037259.1 |
| HI643014.1 | HI470589.1 | DL122003.1 | HI637706.1 | BD251208.1 | HI003377.1 | FW573456.1 | HC207917.1 | DD032566.1 |
| HI642470.1 | HI564738.1 | DL125744.1 | HI380412.1 | BD249872.1 | HI003339.1 | FW574613.1 | HC207362.1 | DD031188.1 |
| HI637699.1 | HI564681.1 | DL125712.1 | HI380331.1 | BD249017.1 | HI003300.1 | FW570977.1 | HC199346.1 | DD030664.1 |
| HI380395.1 | HI564241.1 | DL125680.1 | HI379305.1 | BD248297.1 | HI550412.1 | FW420577.1 | HC197703.1 | DD027305.1 |
| DL102493.1 | HI564115.1 | DL125648.1 | HI568931.1 | BD247444.1 | HI464745.1 | FW420399.1 | HB394283.1 | CS085663.1 |

| | | | | | | | | |
|---|---|---|---|---|---|---|---|---|
| DL102461.1 | HI583102.1 | DL125616.1 | HI568858.1 | BD247043.1 | HI464713.1 | FW503500.1 | HB394251.1 | CS082068.1 |
| DL102397.1 | HI563639.1 | DL125584.1 | HI559129.1 | BD246967.1 | HI464681.1 | FW501715.1 | HB394173.1 | CS079798.1 |
| DL097624.1 | HI563607.1 | DL125552.1 | HI558651.1 | BD245165.1 | HI464649.1 | FW496907.1 | HB393656.1 | CS078853.1 |
| DL097592.1 | HI563572.1 | DL121960.1 | HI564209.1 | BD243817.1 | HI550135.1 | FW420374.1 | HB403046.1 | CS064595.1 |
| DL097560.1 | HI508525.1 | DL121928.1 | HI564098.1 | BD243432.1 | HI505024.1 | FW420342.1 | HB403013.1 | AY967375.1 |
| DL095533.1 | HI544257.1 | DL121896.1 | HI563623.1 | BD242508.1 | HI504980.1 | FW420176.1 | HB402981.1 | AY967343.1 |
| DL095501.1 | HI000029.1 | DL121864.1 | HI563590.1 | BD242450.1 | HI542516.1 | FW496647.1 | DM113493.1 | AY967311.1 |
| DL095437.1 | HI003668.1 | DL121832.1 | HI558078.1 | BD238555.1 | HH961368.1 | HI653922.1 | DM119389.1 | AY967279.1 |
| DL095405.1 | HI003619.1 | DL121800.1 | HI508558.1 | BD236972.1 | HH961336.1 | HI653561.1 | DM115855.1 | AY967247.1 |
| DL091495.1 | HI003581.1 | DL117537.1 | HI575656.1 | BD235954.1 | HH961304.1 | HI653512.1 | GM616297.1 | AY967215.1 |
| DL112156.1 | HI003543.1 | DL117505.1 | HI544147.1 | BD235559.1 | HH961272.1 | HI653400.1 | GM038816.1 | AY967183.1 |
| DL112092.1 | HI001762.1 | DL117473.1 | HI508415.1 | BD234408.1 | HH961240.1 | HI653342.1 | GM000649.1 | AY967151.1 |
| DL107089.1 | HI001726.1 | DL117441.1 | HI508249.1 | BD231158.1 | HH964263.1 | HI653197.1 | GM043429.1 | AY967119.1 |
| DL107057.1 | HI469538.1 | DL013200.1 | HI000053.1 | BD227193.1 | HH974973.1 | HI651894.1 | HB388047.1 | AY967087.1 |
| DL106993.1 | HI544125.1 | DL017989.1 | HI000010.1 | BD227122.1 | HH974588.1 | HI651031.1 | HB386663.1 | AY967055.1 |
| DL106961.1 | HI001645.1 | DL017957.1 | HI003598.1 | BD226842.1 | HH974531.1 | HI646808.1 | HB385987.1 | AY967023.1 |
| DL106929.1 | HI001602.1 | DL017925.1 | HI003563.1 | CQ981112.1 | HH998100.1 | FU757627.1 | HB385778.1 | AY966991.1 |
| DL106897.1 | HI001559.1 | DL017893.1 | HI003513.1 | CQ979566.1 | HH998047.1 | FU757303.1 | HB342012.1 | AY966959.1 |
| DL102188.1 | HI001506.1 | DL017861.1 | HI001840.1 | CQ976602.1 | HH997993.1 | HC441398.1 | HB341964.1 | AY966927.1 |
| DL102156.1 | HI003381.1 | DL017829.1 | HI001780.1 | CQ972389.1 | HH997934.1 | HC436123.1 | HB340459.1 | CS057758.1 |
| DL102124.1 | HI003345.1 | DL017798.1 | HI001742.1 | CQ972357.1 | HH999585.1 | HC358401.1 | HB339945.1 | CS052307.1 |
| DL118110.1 | HI003305.1 | DL037375.1 | HI465575.1 | CQ971114.1 | HH997894.1 | HC358213.1 | HA642297.1 | CS051893.1 |
| DL118078.1 | HI550416.1 | DL037343.1 | HI575282.1 | CQ970790.1 | HH997805.1 | HC358082.1 | HA641429.1 | CS048768.1 |
| DL118046.1 | HI550373.1 | DL024934.1 | HI001683.1 | CQ947280.1 | HH997757.1 | HC321438.1 | HA641103.1 | CS047279.1 |
| DL118014.1 | DM193207.1 | DL024902.1 | HI001622.1 | CQ947116.1 | HH999491.1 | HC324491.1 | HA639884.1 | CS039444.1 |
| DL103630.1 | DM199580.1 | DL021498.1 | HI001580.1 | CQ944186.1 | HH999438.1 | HC318697.1 | HA639231.1 | CS036968.1 |
| DL099024.1 | DM194717.1 | DL021466.1 | HI001530.1 | CQ944122.1 | HH999396.1 | HC316801.1 | HA638521.1 | AX359995.1 |
| DL098992.1 | DM189627.1 | DL021434.1 | HI003365.1 | CQ944090.1 | HH997636.1 | HC310054.1 | DM106883.1 | AX358357.1 |
| DL098960.1 | DM203071.1 | DL021402.1 | HI003327.1 | CQ944058.1 | HH997513.1 | HC310022.1 | DM102736.1 | AX357908.1 |
| DL089015.1 | HB840183.1 | DL017196.1 | HI071598.1 | CQ943994.1 | HH997418.1 | HC309990.1 | DM105590.1 | BD010285.1 |
| DL088983.1 | HB848643.1 | DL017164.1 | HI505597.1 | CQ943962.1 | HH997365.1 | HC309939.1 | DM100606.1 | BD006313.1 |
| DL117896.1 | HB848036.1 | DL017132.1 | HI464733.1 | CQ943930.1 | HH999313.1 | HC307901.1 | DM095104.1 | E50842.1 |
| DL117864.1 | HB839614.1 | DL017100.1 | HI464701.1 | CQ943898.1 | HH999230.1 | HC307834.1 | DM094909.1 | E52003.1 |
| DL117832.1 | HB847593.1 | DL017068.1 | HW260707.1 | CQ943866.1 | HH999187.1 | HC307500.1 | DM103655.1 | E63989.1 |
| DL108150.1 | HB847220.1 | DL017036.1 | HW260675.1 | CQ898672.1 | HH999141.1 | HC306927.1 | GN367553.1 | E54526.1 |
| DL098810.1 | HB838871.1 | DL012775.1 | HW260643.1 | CQ898640.1 | HH999110.1 | FU262987.1 | GM625555.1 | E59813.1 |
| DL098778.1 | HB847135.1 | DL012743.1 | HW260611.1 | CQ898608.1 | HH999061.1 | FU262776.1 | GM638434.1 | AX354616.1 |
| DL098746.1 | HB846708.1 | DL012711.1 | HW260579.1 | CQ898576.1 | HH999013.1 | FU259850.1 | GM638402.1 | AX353667.1 |
| DL092736.1 | HB838301.1 | DL012679.1 | HW260547.1 | CQ898274.1 | HH998953.1 | FU265396.1 | GM638370.1 | CS172459.1 |
| DL092704.1 | HB846305.1 | DL012647.1 | HW260483.1 | CQ897150.1 | HH997256.1 | FU250446.1 | GM638306.1 | CS172310.1 |
| DL088961.1 | HB845980.1 | DL012615.1 | HW260451.1 | CQ895550.1 | HH997162.1 | GN360084.1 | GM638274.1 | CS161652.1 |
| DL088929.1 | HB837562.1 | DL049435.1 | HW260419.1 | CQ893649.1 | HH998905.1 | GN360043.1 | GM625527.1 | CS160722.1 |
| DL088801.1 | HB845854.1 | DL049403.1 | HW260387.1 | CQ890957.1 | HH998847.1 | GN359979.1 | GM624304.1 | CS159787.1 |
| DL021758.1 | HB845518.1 | DL049371.1 | HW260355.1 | CQ878660.1 | HH998796.1 | GN359838.1 | GM624272.1 | CS159598.1 |
| DL021726.1 | HB825987.1 | DL049339.1 | HW260323.1 | CQ877114.1 | HH998737.1 | GN359774.1 | GM652547.1 | CS148830.1 |
| DL021694.1 | HB845340.1 | DL016257.1 | HW260291.1 | CQ875529.1 | HH997082.1 | GN359710.1 | GM652515.1 | CS144416.1 |
| DL021662.1 | HB844793.1 | DL016225.1 | HW260259.1 | AX427669.1 | HH996978.1 | GN359626.1 | GM652483.1 | CS141615.1 |

| | | | | | | | | |
|---|---|---|---|---|---|---|---|---|
| DL021630.1 | HB844358.1 | DL028222.1 | HW260227.1 | AX427209.1 | HH981104.1 | GN359594.1 | GM652451.1 | CS141549.1 |
| DL017328.1 | HB836754.1 | DL028158.1 | HW260195.1 | AX419967.1 | HH998692.1 | GN359498.1 | GM652419.1 | CS134734.1 |
| DL012939.1 | HB836210.1 | DL011773.1 | HW260163.1 | AX418277.1 | HH998651.1 | GN359462.1 | GM652387.1 | CS135966.1 |
| DL041347.1 | HB843283.1 | DL011741.1 | HW260131.1 | AX417914.1 | HH998523.1 | GN359430.1 | GM659907.1 | CS123410.1 |
| DL041283.1 | HB835422.1 | DL011709.1 | HW260099.1 | AX417127.1 | HH996909.1 | GN365393.1 | GM659875.1 | CS122924.1 |
| DL021487.1 | HB807548.1 | DL011677.1 | HW260067.1 | AX407339.1 | HH996788.1 | GN359071.1 | GM659843.1 | CS122346.1 |
| DL021455.1 | HB842894.1 | DL011645.1 | HW260035.1 | AX404728.1 | HH996753.1 | GN363008.1 | GM659811.1 | CS121756.1 |
| DL021423.1 | HB842335.1 | DL011613.1 | HW260003.1 | AX395266.1 | HH994528.1 | GN344841.1 | GM659779.1 | CS106118.1 |
| DL017185.1 | HB842311.1 | DL048596.1 | HW259939.1 | AX392379.1 | AX145458.1 | GN348289.1 | GM659747.1 | CS106048.1 |
| DL017153.1 | HB841893.1 | DL048564.1 | HW259028.1 | AX391608.1 | AX145426.1 | GN346573.1 | GM746246.1 | CS179684.1 |
| DL017121.1 | HB841703.1 | DL048532.1 | HW258907.1 | AX384572.1 | AX145393.1 | GN346541.1 | GM659711.1 | CS327367.1 |
| DL017089.1 | HB840630.1 | DL048500.1 | HW258842.1 | AX380944.1 | AX145361.1 | GN131000.1 | GM659679.1 | CS323579.1 |
| DL017057.1 | HB823081.1 | DL048468.1 | HW257294.1 | AX375576.1 | AX145329.1 | GN131880.1 | GM659647.1 | CS108624.1 |
| DL017025.1 | CS084385.1 | DL044851.1 | HW257198.1 | AX370399.1 | AX145297.1 | GN116455.1 | GM659615.1 | CS106693.1 |
| DL049424.1 | CS084250.1 | DL044819.1 | HW257134.1 | AX364556.1 | AX145265.1 | GN116423.1 | GM659583.1 | CS104312.1 |
| DL049392.1 | CS082046.1 | DL044787.1 | HW256974.1 | AX364524.1 | AX145233.1 | GN116391.1 | GM659550.1 | CS102996.1 |
| DL049360.1 | CS075287.1 | DL044755.1 | HW256910.1 | AX364492.1 | AX145201.1 | GM655032.1 | GM652358.1 | CS102964.1 |
| DL049328.1 | BD171819.1 | DL044723.1 | HW256750.1 | AX364460.1 | AX145169.1 | GM655000.1 | GM652326.1 | CS102932.1 |
| DL009540.1 | AX601604.1 | DL044691.1 | HW256718.1 | AX364428.1 | AX145137.1 | GM694895.1 | GM652294.1 | CS102804.1 |
| DL009508.1 | AX601468.1 | DL044603.1 | HW251216.1 | AX364396.1 | AX145105.1 | GM755057.1 | GM652262.1 | CS102772.1 |
| DL009476.1 | AX593502.1 | DL044571.1 | HW250977.1 | AX364364.1 | AX145073.1 | GM752697.1 | GM652230.1 | CS102644.1 |
| DL009444.1 | AX587894.1 | DL044539.1 | HV748887.1 | AX364269.1 | AX145041.1 | GM834967.1 | GM652198.1 | CS102612.1 |
| DL009412.1 | AX587798.1 | DL044507.1 | HV748482.1 | AX364205.1 | AX145009.1 | DL480713.1 | GM645399.1 | CS102580.1 |
| DL034325.1 | BD160936.1 | DL044475.1 | HV751134.1 | AX364172.1 | AX144977.1 | GM652743.1 | GM645335.1 | CS102548.1 |
| DL009409.1 | BD143738.1 | DL040459.1 | HV753566.1 | AX360309.1 | AX144945.1 | GM652711.1 | GM645303.1 | CS102516.1 |
| DL009377.1 | AX587729.1 | DL040427.1 | HV753484.1 | AX358358.1 | AX144912.1 | GM652679.1 | GM645271.1 | CS102484.1 |
| DL009345.1 | AX587470.1 | DL040395.1 | HV743433.1 | AX357917.1 | AX144880.1 | GM645489.1 | GM645239.1 | CS082048.1 |
| DL009313.1 | AX576406.1 | DL040363.1 | HV743307.1 | BD010479.1 | AX144848.1 | GM645457.1 | GM659513.1 | CS073260.1 |
| DL009281.1 | AX556841.1 | DL040331.1 | HV743270.1 | BD006316.1 | AX144816.1 | GM645425.1 | GM659481.1 | CS070727.1 |
| DL009249.1 | AX556797.1 | DL040299.1 | HV743232.1 | E50843.1 | AX144784.1 | GM638532.1 | GM659449.1 | CS064590.1 |
| DL009217.1 | AX555166.1 | DL036675.1 | HV704449.1 | E63990.1 | AX144752.1 | GM638500.1 | CS254289.1 | AX600188.1 |
| DL018675.1 | AX553945.1 | DL036611.1 | HV704259.1 | E59814.1 | AX144720.1 | GM638468.1 | CS253948.1 | AX597697.1 |
| DL018643.1 | AX552516.1 | DL036579.1 | HV744593.1 | AX354617.1 | AX144656.1 | GM633737.1 | CS254103.1 | AX594159.1 |
| DL018611.1 | AX543911.1 | DL036547.1 | HV702352.1 | AX353670.1 | AX144624.1 | GM633705.1 | CS254071.1 | AX593504.1 |
| DL014215.1 | AX537310.1 | DL036515.1 | HV702260.1 | AX352677.1 | AX144571.1 | GM633673.1 | CS253987.1 | BD166156.1 |
| DL014151.1 | AX525279.1 | DL032312.1 | HV701869.1 | AX351178.1 | AX144379.1 | GM625838.1 | CS249745.1 | BD143744.1 |
| DL014119.1 | AX521999.1 | DL032280.1 | HV701255.1 | AX348792.1 | AX144315.1 | GM625806.1 | CS250541.1 | AX587631.1 |
| DL014087.1 | HW390736.1 | DL032248.1 | HV701216.1 | AX348105.1 | AX144251.1 | GM633668.1 | CS250327.1 | AX576408.1 |
| DL014000.1 | HW399779.1 | DL032216.1 | HV701184.1 | AX347577.1 | AX144121.1 | FB506775.1 | CS247214.1 | AX573480.1 |
| DL013968.1 | HW399356.1 | DL032184.1 | HV701152.1 | AX347316.1 | AX144057.1 | GM621875.1 | CS244969.1 | AX556843.1 |
| DL013936.1 | HW399292.1 | DL032152.1 | HV701120.1 | AX347240.1 | AX143991.1 | GM657340.1 | CS244171.1 | AX556809.1 |
| DL030036.1 | HW408918.1 | DL040281.1 | HV701069.1 | AX347170.1 | AX143607.1 | GM642988.1 | CS244251.1 | AX555168.1 |
| DL029972.1 | HW390496.1 | DL048422.1 | HV701037.1 | AX346968.1 | AX143543.1 | GM642955.1 | CS244219.1 | AX537317.1 |
| DL046629.1 | HW390460.1 | DL048390.1 | HV695226.1 | AX344924.1 | HV763968.1 | GM630647.1 | CS244187.1 | AX528838.1 |
| DL046597.1 | HW390395.1 | DL048358.1 | HV695194.1 | AX344524.1 | HV767474.1 | GM630615.1 | CS243279.1 | AX528401.1 |
| DL046565.1 | HW389053.1 | DL048326.1 | HV695130.1 | AX342788.1 | HV767005.1 | GM630583.1 | CS243072.1 | AX525421.1 |
| DL046533.1 | HW388328.1 | DL044440.1 | HV695018.1 | HW261240.1 | HV766825.1 | GM621648.1 | CS239627.1 | A30448.1 |

| | | | | | | | | |
|---|---|---|---|---|---|---|---|---|
| DL042647.1 | HW387498.1 | DL044408.1 | HV694847.1 | DL106923.1 | HV766701.1 | GM621616.1 | CS238974.1 | AX179522.1 |
| DL042615.1 | HW380923.1 | DL044376.1 | HV694812.1 | DL109722.1 | HV766323.1 | GM621584.1 | CS236040.1 | AX179462.1 |
| DL042583.1 | HW363662.1 | DL044344.1 | HV694770.1 | DL109690.1 | HV753232.1 | GM621552.1 | CS228597.1 | A28856.1 |
| CS537864.1 | HW363631.1 | DL044312.1 | HV700733.1 | DL127616.1 | HV758698.1 | GM657132.1 | DD173110.1 | A25199.1 |
| CS537398.1 | HW355325.1 | DL044280.1 | HV694745.1 | DL124131.1 | HV745724.1 | GM656909.1 | DD166308.1 | A28261.1 |
| CS606967.1 | HW338790.1 | DL040104.1 | HV043012.1 | DL119479.1 | HV694793.1 | GM656877.1 | DD165528.1 | A26547.1 |
| CS606823.1 | HW338662.1 | DL032111.1 | HV111940.1 | DL119447.1 | HV694758.1 | GM656845.1 | DD165450.1 | A25581.1 |
| CS606246.1 | HW338406.1 | DL032079.1 | HV041726.1 | DL119415.1 | HV694082.1 | GM642628.1 | DD165154.1 | A25119.1 |
| CS299495.1 | HW337894.1 | DL032047.1 | HV098618.1 | DL114820.1 | HV693315.1 | GM044621.1 | DD159562.1 | A22468.1 |
| CS297357.1 | HW337638.1 | DL032015.1 | HV119941.1 | DL114788.1 | HV689455.1 | GM646481.1 | DD170877.1 | A14545.1 |
| DD251653.1 | HW337254.1 | DL031983.1 | HV189876.1 | DL114756.1 | HV688888.1 | GM646449.1 | DD182188.1 | A11920.1 |
| CS287618.1 | HW336506.1 | DL031951.1 | HV227864.1 | DL114724.1 | HV585549.1 | GM646417.1 | DD175898.1 | A21828.1 |
| CS287401.1 | HW329482.1 | DL028142.1 | HV218249.1 | DL114692.1 | HV585140.1 | GM626875.1 | CS103352.1 | A21490.1 |
| DD240744.1 | HW329401.1 | DL028110.1 | HV245428.1 | DL114660.1 | HV585108.1 | GM626843.1 | CS106377.1 | A19976.1 |
| DD234581.1 | HW329281.1 | DL028078.1 | HV214864.1 | DL141579.1 | HV592373.1 | GM626811.1 | CS105943.1 | A16540.1 |
| DD246632.1 | HW329273.1 | DL028046.1 | HV234158.1 | DL123462.1 | HV585554.1 | GM653508.1 | CS103436.1 | A18692.1 |
| DD241384.1 | HW335960.1 | DL028014.1 | HV236010.1 | DL123430.1 | HV570621.1 | GM653476.1 | CS103334.1 | A16289.1 |
| E41548.1 | HW328833.1 | DL027982.1 | HV301802.1 | DL123398.1 | FZ421507.1 | GM653444.1 | CS103082.1 | A05417.1 |
| E40094.1 | HW328634.1 | DL027950.1 | HV306394.1 | DL123366.1 | FZ413935.1 | GM653412.1 | CS102985.1 | A15954.1 |
| E37386.1 | HV964074.1 | DL036460.1 | HV302946.1 | DL119112.1 | FZ419840.1 | GM653380.1 | CS102953.1 | A15354.1 |
| E35594.1 | HV958811.1 | DL036428.1 | HV308812.1 | DL119080.1 | FZ419806.1 | GM646381.1 | CS102889.1 | A14321.1 |
| E33331.1 | HV963545.1 | DL036396.1 | HV304173.1 | DL119048.1 | FW591759.1 | GM646349.1 | CS102793.1 | A12547.1 |
| E36136.1 | HV963330.1 | DL036364.1 | HV311092.1 | DL119016.1 | FW590746.1 | HB820037.1 | CS102729.1 | A12192.1 |
| E06035.1 | HV965579.1 | DL036332.1 | HV214561.1 | DL114620.1 | FW589137.1 | HB845338.1 | CS102697.1 | A04967.1 |
| E05787.1 | HV965307.1 | DL044247.1 | HV304090.1 | DL114588.1 | FW577152.1 | HB844791.1 | CS102665.1 | A07647.1 |
| E04669.1 | HV956045.1 | DL044215.1 | HV226822.1 | DL114556.1 | GN046002.1 | HB844356.1 | CS102633.1 | A06759.1 |
| E04459.1 | HV951142.1 | DL044183.1 | HV040136.1 | DL114524.1 | HH779773.1 | GM652772.1 | CS102601.1 | A06145.1 |
| E03956.1 | HV950609.1 | HV220404.1 | HV191836.1 | DL114492.1 | HH777935.1 | GM652740.1 | CS102569.1 | A05253.1 |
| E03642.1 | HV947136.1 | HV226387.1 | HV188469.1 | DL114460.1 | HH820934.1 | GM652708.1 | CS102537.1 | A03834.1 |
| E03160.1 | HV952957.1 | HV039589.1 | HV194385.1 | DL014504.1 | HH819344.1 | GM652676.1 | CS102505.1 | A07199.1 |
| E02316.1 | HV951957.1 | HV117903.1 | HV191318.1 | DL014472.1 | HH759230.1 | GM645486.1 | CS101260.1 | A01449.1 |
| E01673.1 | HV939874.1 | HV117224.1 | HV182461.1 | DL009950.1 | HI401106.1 | GM645454.1 | CS091388.1 | A05102.1 |
| E01425.1 | HV945592.1 | HV116991.1 | HV182429.1 | DL009339.1 | HI637685.1 | GM645422.1 | CS086828.1 | A00704.1 |
| E01335.1 | HV323289.1 | HV038602.1 | HV182397.1 | DL009307.1 | HI178656.1 | GM638529.1 | CS083815.1 | M38306.1 |
| E01211.1 | HV187362.1 | HV038526.1 | HV182365.1 | DL009275.1 | HI202820.1 | GM638497.1 | CS082538.1 | M12615.1 |
| E00970.1 | HV195746.1 | HV182330.1 | HV117850.1 | DL009243.1 | HI177962.1 | GM638465.1 | CS082421.1 | M22593.1 |
| E00307.1 | HV208794.1 | HV182298.1 | HV117791.1 | DL018701.1 | HI214580.1 | GM633734.1 | CS082357.1 | M31471.1 |
| E00109.1 | HV245449.1 | HV202607.1 | HV117759.1 | DL018669.1 | HI214548.1 | GM633702.1 | CS082286.1 | M15316.1 |
| DD146635.1 | HV197647.1 | HV187856.1 | FW503807.1 | DL018637.1 | HI214514.1 | GM624715.1 | CS082109.1 | K01151.1 |
| DD115288.1 | HV188368.1 | HV222995.1 | HV331770.1 | DL014209.1 | HI214450.1 | GM624683.1 | CS080407.1 | M11247.1 |
| DD099323.1 | HV182442.1 | HV234299.1 | HV324760.1 | DL014177.1 | HI214418.1 | GM659919.1 | CS079303.1 | M19559.1 |
| CQ981119.1 | HV182410.1 | HV312984.1 | HV323866.1 | DL014145.1 | HI213811.1 | GM659887.1 | CS078835.1 | AF411596.1 |
| CQ975457.1 | HV182378.1 | HV038051.1 | HV043006.1 | DD431742.1 | HI185489.1 | GM659855.1 | CS075526.1 | LT897788.1 |
| CQ973476.1 | HV217083.1 | HV037760.1 | HV111937.1 | CS540040.1 | HI212983.1 | GM659823.1 | CS075360.1 | GN044364.1 |
| CQ976606.1 | HV235480.1 | HV037711.1 | HV111849.1 | CS539917.1 | HI546303.1 | GM659791.1 | CS074047.1 | HC039685.1 |
| CQ972359.1 | FW420813.1 | HV036669.1 | HV098614.1 | CS531813.1 | HI586944.1 | GM652338.1 | CS073114.1 | HC039628.1 |
| CQ971793.1 | FW495948.1 | HV036291.1 | HV119940.1 | CS534802.1 | HI210981.1 | GM652306.1 | CS072180.1 | HC046059.1 |

| | | | | | | | | |
|---|---|---|---|---|---|---|---|---|
| CQ947282.1 | FW420557.1 | HV031616.1 | HV189875.1 | CS501496.1 | HI210949.1 | GM652274.1 | CS070578.1 | HC035647.1 |
| CQ947163.1 | FW420455.1 | HV031518.1 | HV227859.1 | CS500217.1 | HI570425.1 | GM627987.1 | CS070128.1 | HC021036.1 |
| CQ947118.1 | FW420366.1 | FZ435885.1 | HV218248.1 | CS498449.1 | HI473084.1 | GM627955.1 | CS067279.1 | HC025489.1 |
| CQ944188.1 | FW420334.1 | FZ434956.1 | HV245427.1 | CS496118.1 | HI472922.1 | GM627923.1 | CS063853.1 | HC025425.1 |
| CQ944156.1 | FW420054.1 | FW512090.1 | HV214863.1 | DD419547.1 | HI210925.1 | GM715061.1 | CS062976.1 | HC010411.1 |
| CQ944124.1 | HI653912.1 | FW508357.1 | HV301801.1 | DD418602.1 | HI209399.1 | GM647269.1 | CS061690.1 | HC007830.1 |
| CQ944092.1 | HI651008.1 | FW508262.1 | HV306393.1 | DD411498.1 | HI072161.1 | GM647237.1 | CS061037.1 | HC007603.1 |
| CQ944060.1 | HI646733.1 | FW559263.1 | HV306183.1 | DD417803.1 | HI004339.1 | GM647205.1 | CS059015.1 | HC007571.1 |
| CQ944028.1 | AY145512.1 | FW552306.1 | HV302945.1 | DD410118.1 | HI004275.1 | GM647173.1 | AY967391.1 | HC010672.1 |
| CQ943964.1 | HH797409.1 | FW552272.1 | HV308811.1 | CS492961.1 | HI002259.1 | GM647141.1 | AY967359.1 | GN090811.1 |
| CQ943932.1 | HH794934.1 | FW552235.1 | HV305738.1 | CS486435.1 | HI002208.1 | GM647109.1 | AY967327.1 | GN089897.1 |
| CQ943900.1 | HH998046.1 | FW562057.1 | HI653599.1 | CS482991.1 | HI002165.1 | GM647045.1 | AY967295.1 | GN089830.1 |
| CQ943868.1 | HH997992.1 | FW552175.1 | HI653527.1 | CS482956.1 | HI002125.1 | GM647016.1 | AY967263.1 | GN082910.1 |
| CQ903047.1 | HH999686.1 | FW552139.1 | HI653476.1 | CS482918.1 | HI000497.1 | FB712022.1 | AY967231.1 | GN082837.1 |
| A01395.1 | HH999584.1 | FW555603.1 | HI653361.1 | CS482874.1 | AX037308.1 | GM041706.1 | AY967199.1 | GN089170.1 |
| CQ898683.1 | HH997893.1 | FW557015.1 | HI653280.1 | E41543.1 | AX035989.1 | GM040837.1 | AY967167.1 | GN082807.1 |
| CQ898642.1 | HH997801.1 | FW562770.1 | HI653222.1 | E43925.1 | AX030368.1 | CS729406.1 | AY967135.1 | GN082367.1 |
| CQ898578.1 | HH997756.1 | FW565448.1 | HI652908.1 | E06948.1 | AX026038.1 | CS728603.1 | AY967103.1 | GN088023.1 |
| CQ898503.1 | HH999490.1 | FW562668.1 | HI651046.1 | E06722.1 | AX023632.1 | CS728571.1 | AX328289.1 | GN075907.1 |
| CQ896782.1 | HH999437.1 | FW559900.1 | HI647556.1 | E06019.1 | AX023598.1 | CS727347.1 | AX326768.1 | L08864.1 |
| CQ895552.1 | HH997635.1 | FW505135.1 | HH807325.1 | E05658.1 | AX023565.1 | CS727120.1 | AX323773.1 | M88476.1 |
| CQ894667.1 | HH997578.1 | FW508122.1 | HH804171.1 | E05200.1 | HW315182.1 | GM720296.1 | AX322708.1 | DM059605.1 |
| CQ893661.1 | HH997364.1 | FW562467.1 | HH797228.1 | E04602.1 | HW315150.1 | GM869489.1 | AX319640.1 | DM058891.1 |
| CQ891737.1 | HH999302.1 | FW555416.1 | HH779678.1 | E04343.1 | HW315054.1 | FB985402.1 | AX306878.1 | DM058813.1 |
| CQ890961.1 | HH999229.1 | FW553187.1 | HH777921.1 | E04132.1 | HW160817.1 | GM879370.1 | AX306572.1 | DM045497.1 |
| CQ888118.1 | HH999185.1 | FW553126.1 | HH820957.1 | E03906.1 | HW069966.1 | GM839625.1 | AX268114.1 | DM045334.1 |
| CQ879725.1 | HH999140.1 | FW559397.1 | HH759323.1 | E03616.1 | HW069934.1 | GM060200.1 | AX260030.1 | DM045055.1 |
| CQ877778.1 | HH999109.1 | FW559349.1 | HH759212.1 | E02731.1 | HW069862.1 | GM603468.1 | AX304319.1 | DM044873.1 |
| CQ875531.1 | HH999060.1 | FW506884.1 | HH759180.1 | E02291.1 | HW069802.1 | GM838944.1 | AX303585.1 | DM039593.1 |
| CQ874984.1 | HH999002.1 | FW510231.1 | HH759148.1 | E01491.1 | HW084958.1 | GM601059.1 | AX302477.1 | DM044756.1 |
| CQ874249.1 | HH998952.1 | FW506305.1 | HH759084.1 | E01305.1 | HW084919.1 | FB717611.1 | AX299859.1 | GN071179.1 |
| AX418280.1 | HH997237.1 | FW509468.1 | HH759052.1 | E00965.1 | HW097649.1 | FB702165.1 | AX297683.1 | GN067943.1 |
| AX407349.1 | HH997161.1 | HI934011.1 | HH759020.1 | E00813.1 | HW065067.1 | FB677465.1 | AX287563.1 | GN067964.1 |
| AX404737.1 | HH978157.1 | HI933467.1 | HH758988.1 | E00694.1 | HW049574.1 | GM061221.1 | AX286558.1 | GM658306.1 |
| AX399386.1 | HH998904.1 | HI646714.1 | HH758956.1 | E00547.1 | HV778443.1 | DL110030.1 | AX283693.1 | GM658242.1 |
| AX395270.1 | HH998846.1 | HI646238.1 | HH758924.1 | DD144779.1 | HV757325.1 | DL109998.1 | AX283227.1 | GM658210.1 |
| AX394153.1 | HH998795.1 | HH807082.1 | HH758892.1 | DD069499.1 | HV702578.1 | DL109966.1 | CQ969082.1 | GM621675.1 |
| AX392383.1 | HH998736.1 | HH804185.1 | HH757468.1 | DD098105.1 | HW302495.1 | DL109720.1 | CQ967871.1 | GM621643.1 |
| AX391692.1 | HH997081.1 | HH796600.1 | HH757436.1 | DD141705.1 | HW295685.1 | DL109688.1 | CQ967768.1 | GM642719.1 |
| AX384791.1 | HH997027.1 | HH794724.1 | FW368716.1 | DD158392.1 | HV947302.1 | DL124129.1 | CQ963563.1 | GM739603.1 |
| AX384574.1 | HH996977.1 | HH793037.1 | FW368460.1 | DD157372.1 | HV947133.1 | DL124001.1 | CQ957853.1 | GM670012.1 |
| AX384026.1 | HH981102.1 | HH792732.1 | FW368346.1 | DD155698.1 | HV951954.1 | DL119509.1 | CQ955911.1 | GM656520.1 |
| AX377928.1 | HH998650.1 | HH779682.1 | FW368145.1 | DD152407.1 | HV951866.1 | DL119477.1 | CQ947128.1 | GM656488.1 |
| AX365600.1 | HH998522.1 | HH777925.1 | FW363399.1 | DD083027.1 | HV946835.1 | DL119445.1 | CQ945864.1 | GM649271.1 |
| AX364675.1 | HH996908.1 | HH774440.1 | FW363354.1 | DD139585.1 | HV939760.1 | DL119413.1 | CQ944198.1 | GM649239.1 |
| AX364558.1 | HH996787.1 | HH820964.1 | FW362940.1 | DD149496.1 | HV943228.1 | DL114818.1 | CQ944166.1 | GM618814.1 |
| AX364526.1 | HH996752.1 | HH820922.1 | FW367126.1 | DD147810.1 | HV856240.1 | DL114786.1 | CQ944134.1 | GM863542.1 |

| | | | | | | | | |
|---|---|---|---|---|---|---|---|---|
| AX364494.1 | HH994525.1 | HH759216.1 | FW362660.1 | DD147778.1 | HV931772.1 | DL114754.1 | CQ944102.1 | FB725707.1 |
| AX364462.1 | HH980711.1 | HH759184.1 | FW349829.1 | DD147746.1 | HV936730.1 | DL114722.1 | CQ944070.1 | GM881777.1 |
| AX364398.1 | HH969782.1 | HH759152.1 | FW366593.1 | DD147714.1 | HV925508.1 | DL114690.1 | CQ944038.1 | GM616057.1 |
| AX364366.1 | HH977296.1 | HH759120.1 | FW362070.1 | DD147682.1 | HV932704.1 | DL114658.1 | CQ943974.1 | GM731823.1 |
| AX364271.1 | HH980618.1 | HH759088.1 | FW366208.1 | DD146946.1 | HV509846.1 | DL127285.1 | CQ943942.1 | GM840518.1 |
| AX364239.1 | HH980514.1 | HH759056.1 | FW351242.1 | CQ840171.1 | HV508635.1 | DL123460.1 | CQ943910.1 | GM040835.1 |
| AX358365.1 | HH980446.1 | HH759024.1 | FW351184.1 | DD463389.1 | HV508571.1 | DL123428.1 | CQ943878.1 | GM839522.1 |
| AX357200.1 | HH987317.1 | HH758992.1 | FW366032.1 | CS647375.1 | HV508539.1 | DL123396.1 | CQ918565.1 | CS729403.1 |
| E63233.1 | HH980373.1 | HH758960.1 | FW360680.1 | CS646213.1 | HV508507.1 | DL123364.1 | A19499.1 | CS728633.1 |
| BD000709.1 | HH980341.1 | HH758928.1 | FW350065.1 | DD460127.1 | HV508405.1 | DL119110.1 | CQ898652.1 | CS728601.1 |
| E59816.1 | HH980260.1 | HH758896.1 | HD122549.1 | DD458764.1 | HV512281.1 | DL119078.1 | CQ898620.1 | CS727345.1 |
| HC025399.1 | HH980179.1 | HH757472.1 | HD114106.1 | DD453839.1 | HV512249.1 | DL119046.1 | CQ898588.1 | CS727262.1 |
| HC010666.1 | HH980141.1 | HH757440.1 | HD082718.1 | CS631742.1 | HV512217.1 | DL119014.1 | CQ898556.1 | FB715330.1 |
| HC010417.1 | HH996688.1 | HH756193.1 | HD082432.1 | CS631246.1 | HV512185.1 | DL014438.1 | CQ898254.1 | GM720294.1 |
| HC010313.1 | HH996625.1 | HI401628.1 | HD082300.1 | CS631182.1 | HV512153.1 | DL009660.1 | CQ893709.1 | GM949531.1 |
| HC007701.1 | HH996565.1 | HI401243.1 | HD088251.1 | CS627817.1 | HV512121.1 | DL009337.1 | CQ892576.1 | GM009456.1 |
| HC007669.1 | HH998513.1 | HI401095.1 | HC473822.1 | CS632131.1 | HV504784.1 | DL009305.1 | CQ890275.1 | GM887862.1 |
| HC007637.1 | HH998434.1 | HI422779.1 | HD070400.1 | CS626242.1 | HV504752.1 | DL009241.1 | CQ889054.1 | GM869487.1 |
| HC007577.1 | HH998382.1 | HI416364.1 | FW345123.1 | DD438420.1 | HV504720.1 | DL014175.1 | CQ888084.1 | GM869167.1 |
| HC007545.1 | HH998349.1 | HI416158.1 | FW343614.1 | DD438015.1 | HV504656.1 | DL014143.1 | CQ882006.1 | GM773641.1 |
| HC007513.1 | HH999973.1 | HI415919.1 | FW343424.1 | DD437047.1 | HV504624.1 | DJ354266.1 | CQ877818.1 | FB983048.1 |
| HC005710.1 | HH999920.1 | HI415822.1 | FW342970.1 | DD435329.1 | HV491629.1 | DJ361194.1 | CQ877236.1 | GM888080.1 |
| DM370685.1 | HH979845.1 | HI415790.1 | HC923146.1 | CS620785.1 | HV455601.1 | DJ340471.1 | CQ876143.1 | GM879307.1 |
| DM370653.1 | HH979770.1 | HI415758.1 | HC921626.1 | CS616572.1 | AB212907.1 | DJ339842.1 | CQ875541.1 | FB722588.1 |
| DM370621.1 | HH979688.1 | HI415182.1 | HB866508.1 | DD090736.1 | HI930655.1 | DJ339778.1 | CQ874994.1 | GM042051.1 |
| DM370589.1 | HH999887.1 | HI414045.1 | HB866096.1 | DD117021.1 | HI930271.1 | DJ339746.1 | CQ874722.1 | GM721244.1 |
| DM370557.1 | HH999843.1 | HI414013.1 | HB981731.1 | DD090328.1 | HI935077.1 | DJ339650.1 | CQ873294.1 | GM839364.1 |
| GM658449.1 | HH999793.1 | HI413757.1 | HB976755.1 | DD089287.1 | M23200.1 | DJ339585.1 | CQ869299.1 | GM863350.1 |
| GM651105.1 | HH998311.1 | HI413451.1 | HB976178.1 | DD102588.1 | HI918654.1 | HV542559.1 | CQ868816.1 | GM603465.1 |
| GM644210.1 | HH998262.1 | HI410950.1 | DM193383.1 | DD057925.1 | HI918278.1 | HV542020.1 | CQ868295.1 | FB721292.1 |
| GM636924.1 | HH998225.1 | HI516541.1 | DM193333.1 | DD057893.1 | HI661448.1 | HV541506.1 | CQ867001.1 | FB715268.1 |
| GM636860.1 | HH998178.1 | HI516088.1 | DM195299.1 | DD057861.1 | HI660611.1 | FW381560.1 | CQ861214.1 | FB702163.1 |
| GM623521.1 | HH996537.1 | HI541037.1 | DM195079.1 | DD057833.1 | FW497340.1 | FW369986.1 | CQ859617.1 | DL014436.1 |
| GM623489.1 | HH993651.1 | GM011205.1 | DM189853.1 | DD053289.1 | FW420420.1 | FW369338.1 | CQ858165.1 | DL014205.1 |
| GM623457.1 | HH986619.1 | GM888573.1 | HB811484.1 | DD051868.1 | FW420395.1 | FW369194.1 | CQ857840.1 | DL014173.1 |
| GM623425.1 | HH979573.1 | GM887775.1 | HB848142.1 | DD046353.1 | FW420363.1 | FW368167.1 | CQ857498.1 | DL014141.1 |
| GM623393.1 | HH931977.1 | FB662048.1 | HB847920.1 | DD033982.1 | FW420331.1 | FW351260.1 | CQ855753.1 | DJ044907.1 |
| GM623361.1 | GM652640.1 | GM005814.1 | HB839342.1 | DD032550.1 | HH996749.1 | S72080.1 | CQ853734.1 | DJ028156.1 |
| GM643816.1 | GM645482.1 | FB984702.1 | HB847372.1 | DD023869.1 | HH980615.1 | M21211.1 | CQ847384.1 | DJ027946.1 |
| GM741830.1 | GM645450.1 | GM888355.1 | HB832566.1 | DD023576.1 | HH980511.1 | J05015.1 | BD075622.1 | DJ040570.1 |
| GM658312.1 | GM645418.1 | GM879377.1 | HB846927.1 | DD020513.1 | HH980443.1 | AY346130.1 | BD074952.1 | DJ026297.1 |
| GM658280.1 | GM633762.1 | DL194197.1 | HB838579.1 | DD029205.1 | HH980370.1 | FW342543.1 | BD074803.1 | CS688537.1 |
| GM658248.1 | GM633730.1 | DL188827.1 | HB846470.1 | DD028804.1 | HH980338.1 | FW337281.1 | BD073587.1 | DJ008420.1 |
| GM658216.1 | GM633698.1 | DL196348.1 | HB846120.1 | DD010174.1 | HH980173.1 | FW341456.1 | BD070699.1 | DJ008388.1 |
| GM658102.1 | GM831928.1 | DL193878.1 | HB815312.1 | DD019753.1 | HH980138.1 | FW340706.1 | BD063675.1 | DJ004219.1 |
| GM658038.1 | GM634545.1 | DL196201.1 | DL027344.1 | DD017637.1 | HH979894.1 | HC874693.1 | BD062192.1 | DJ003329.1 |
| GM658006.1 | GM634513.1 | DL193801.1 | DL027312.1 | DD003323.1 | HH996737.1 | HC873725.1 | BD057159.1 | DD491146.1 |

| | | | | | | | | |
|---|---|---|---|---|---|---|---|---|
| GM657974.1 | GM634481.1 | DL193769.1 | DL027280.1 | BD412587.1 | HH996662.1 | HC889141.1 | BD017746.1 | CS671009.1 |
| GM657942.1 | GM884939.1 | DL193700.1 | DL027248.1 | BD434180.1 | HH996622.1 | FV533457.1 | BD016702.1 | DD463390.1 |
| GM650750.1 | GM664831.1 | DL183243.1 | DL027216.1 | BD453851.1 | HH996560.1 | FV532233.1 | BD014234.1 | CS647378.1 |
| GM650718.1 | GM648728.1 | FB513471.1 | DL027184.1 | BD453819.1 | HH998475.1 | FV531697.1 | BD014201.1 | HW259915.1 |
| GM650686.1 | GM648696.1 | FB513116.1 | DL023372.1 | CQ813885.1 | HH998431.1 | FV530904.1 | BD014161.1 | HW259512.1 |
| GM650590.1 | GM648664.1 | FB571334.1 | DL023340.1 | CQ813853.1 | HH998379.1 | FV522795.1 | AX481601.1 | HW259185.1 |
| GM643694.1 | GM648632.1 | FB570199.1 | DL023308.1 | CQ813821.1 | HH998346.1 | FV522612.1 | AX478053.1 | HW258917.1 |
| FB775373.1 | GM648600.1 | FB571386.1 | DL023276.1 | CQ813789.1 | HH999917.1 | FV534226.1 | AX473167.1 | HW258850.1 |
| FB766177.1 | GM634448.1 | CS696170.1 | DL023244.1 | CQ813708.1 | HH979835.1 | HC465518.1 | AX472627.1 | HW257398.1 |
| FB764689.1 | GM629461.1 | CS696106.1 | DL023212.1 | CQ812664.1 | HH979767.1 | HC461767.1 | E61328.1 | HW257334.1 |
| FB743921.1 | GM619894.1 | CS696074.1 | DL023180.1 | CQ802189.1 | HH979685.1 | HC453694.1 | E64480.1 | HW257302.1 |
| FB743857.1 | DL106949.1 | CS696042.1 | DL020165.1 | CQ801295.1 | HH999882.1 | HC453662.1 | AX469465.1 | HW257270.1 |
| FB743803.1 | DL106917.1 | CS695977.1 | DL020133.1 | CQ800901.1 | HH999790.1 | AF023672.1 | AX468887.1 | HW257238.1 |
| FB761825.1 | DL111943.1 | CS695913.1 | DL020101.1 | CQ800649.1 | HH998308.1 | HC449552.1 | AX466458.1 | HW257174.1 |
| GM036246.1 | DL095212.1 | CS695881.1 | DL020069.1 | CQ797812.1 | HH998259.1 | FU761098.1 | AX460295.1 | HW257110.1 |
| GM017427.1 | DL106188.1 | CS695849.1 | DL020037.1 | CQ795470.1 | HH998222.1 | FU759913.1 | AX458654.1 | HW256982.1 |
| GM889749.1 | DL119106.1 | CS695817.1 | DL015442.1 | CQ792258.1 | HH998171.1 | FU758384.1 | AX458540.1 | HW256950.1 |
| GM747637.1 | DL119074.1 | CS695785.1 | DL010920.1 | CQ788576.1 | HH996534.1 | FU767881.1 | AX454148.1 | HW256822.1 |
| GM746705.1 | DL119042.1 | CS695753.1 | DL010888.1 | CQ787684.1 | HH993639.1 | HC358392.1 | AX453997.1 | HW251030.1 |
| GM008847.1 | DL119010.1 | CS695721.1 | DL010856.1 | CQ786568.1 | HH986616.1 | HC358204.1 | AX453431.1 | HW240630.1 |
| FB660837.1 | DL104855.1 | CS695689.1 | DL010824.1 | CQ784693.1 | HI213017.1 | HC358073.1 | AX451634.1 | HW238939.1 |
| FB983182.1 | DL104564.1 | CS695561.1 | DL023174.1 | CQ784627.1 | HI212977.1 | HC046867.1 | AX443300.1 | HI967441.1 |
| GM866036.1 | DL104532.1 | CS695497.1 | DL023145.1 | CQ778519.1 | HI212638.1 | HC046835.1 | AX441324.1 | HI987445.1 |
| GM865630.1 | DL049284.1 | CS695465.1 | DL023113.1 | CQ778397.1 | HI547166.1 | HC046803.1 | AX429490.1 | HC194694.1 |
| FB726925.1 | DL028119.1 | CS695303.1 | DL023049.1 | CQ771673.1 | HI212484.1 | HC046771.1 | AX429236.1 | FW512095.1 |
| GM864062.1 | DD271429.1 | DL176559.1 | DL023017.1 | CQ759686.1 | HI588173.1 | HC046707.1 | AX428223.1 | FW511947.1 |
| GM968167.1 | DD259905.1 | DL176516.1 | DL022985.1 | AX458227.1 | HI546329.1 | DM380133.1 | AX427235.1 | FW554688.1 |
| GM731830.1 | DD272433.1 | DL176459.1 | DL019810.1 | AX457067.1 | HI546297.1 | HC025517.1 | AX427120.1 | FW555608.1 |
| GM879659.1 | CS305123.1 | DL176372.1 | DL010725.1 | AX454046.1 | HI587846.1 | HC025421.1 | AX419732.1 | FW555576.1 |
| GM615594.1 | CS297027.1 | DL176298.1 | DL010693.1 | A34180.1 | HI211038.1 | HC010656.1 | AX418388.1 | FW560489.1 |
| GM840857.1 | CS287898.1 | DL182007.1 | DL010661.1 | AX441498.1 | HI210975.1 | HC010407.1 | AX404875.1 | HI565798.1 |
| FB712038.1 | DD246856.1 | DL181466.1 | DL010629.1 | AX430183.1 | HI210943.1 | HC008576.1 | AX402392.1 | HI472917.1 |
| DL096314.1 | E41534.1 | DL181373.1 | DL047053.1 | AX428409.1 | HI570419.1 | HC007843.1 | AX398676.1 | HI580528.1 |
| DL096282.1 | E40769.1 | DL174590.1 | DL047021.1 | AX427132.1 | HI569859.1 | HC007819.1 | AX398330.1 | HI209394.1 |
| DL096250.1 | E46868.1 | DL176783.1 | DL046989.1 | AX427057.1 | HI569623.1 | HB386681.1 | AX394295.1 | HI072078.1 |
| DL094236.1 | E33631.1 | DL176705.1 | DL046957.1 | AX418265.1 | HI565950.1 | HB340002.1 | AX384693.1 | HI004265.1 |
| DL094204.1 | E05886.1 | FB357041.1 | DL046925.1 | AX411720.1 | HI472916.1 | HA641572.1 | AX376961.1 | HI002286.1 |
| DL094172.1 | E05011.1 | FB344634.1 | DL046893.1 | AX399392.1 | HI584054.1 | HA641171.1 | AX376771.1 | HI002253.1 |
| DL090489.1 | CQ816990.1 | FB359900.1 | DL046861.1 | AX393434.1 | HI583983.1 | HA635493.1 | AX374880.1 | HI002199.1 |
| DL086292.1 | CQ816948.1 | FB342773.1 | DL043039.1 | AX392311.1 | HI209393.1 | HA640064.1 | AX370678.1 | HI002156.1 |
| DL086260.1 | CQ815800.1 | DL088350.1 | DL043007.1 | AX384559.1 | HI072076.1 | HA644432.1 | HW350982.1 | HI002120.1 |
| DL086228.1 | CQ815505.1 | DL088318.1 | DL042975.1 | AX383801.1 | HI004327.1 | GN368267.1 | HW350936.1 | HI000467.1 |
| DL110162.1 | CQ814074.1 | DL088286.1 | DL042943.1 | AX380799.1 | HI002285.1 | GN360107.1 | GM632464.1 | HI000415.1 |
| DL110130.1 | CQ814041.1 | DL088254.1 | DL042911.1 | AX375479.1 | HI002251.1 | GN356188.1 | GM632432.1 | HI000373.1 |
| DL110098.1 | CQ814009.1 | DL088222.1 | DL042879.1 | AX364633.1 | HI002198.1 | GN360002.1 | GM623533.1 | HI000316.1 |
| DL105165.1 | CQ813977.1 | DL088190.1 | DL038966.1 | AX364548.1 | HI002155.1 | GN359906.1 | GM623501.1 | HI203328.1 |
| DL105133.1 | CQ813945.1 | DL088158.1 | DL038934.1 | AX364484.1 | HI002117.1 | GN359797.1 | GM623469.1 | HI553269.1 |

| | | | | | | | | |
|---|---|---|---|---|---|---|---|---|
| DL119949.1 | CQ813913.1 | DL088126.1 | DL035118.1 | AX364452.1 | HI000463.1 | GN359765.1 | GM623437.1 | HI207834.1 |
| DL119917.1 | CQ813881.1 | DL088094.1 | DL031082.1 | AX364420.1 | HI000414.1 | GN359733.1 | DL123137.1 | HI583850.1 |
| DL119885.1 | CQ813849.1 | DL088062.1 | DL031050.1 | AX364388.1 | HI000372.1 | GN359649.1 | DL123105.1 | HI577090.1 |
| DL119853.1 | CQ813817.1 | DL088030.1 | CS502404.1 | AX364356.1 | HI000315.1 | GN359585.1 | DL123073.1 | HI520875.1 |
| DL119821.1 | CQ813698.1 | DL087998.1 | CS501245.1 | AX364147.1 | HI553268.1 | GN359553.1 | DL123041.1 | HI520493.1 |
| DL115130.1 | CQ812171.1 | DL091939.1 | CS500061.1 | AX361212.1 | HI553196.1 | GN359421.1 | DL123009.1 | HI003851.1 |
| DL110066.1 | CQ802183.1 | DL091907.1 | CS499225.1 | AX357087.1 | HI545531.1 | GN359389.1 | DL122977.1 | HI002085.1 |
| DL110002.1 | CQ800805.1 | DL091875.1 | CS498527.1 | BD004710.1 | HI521281.1 | GN359325.1 | DL118691.1 | HI002033.1 |
| DL109970.1 | CQ800637.1 | DL091843.1 | CS498408.1 | E64036.1 | HI539190.1 | GN365383.1 | DL118659.1 | HI001950.1 |
| DL109724.1 | CQ800438.1 | DL091779.1 | DD418614.1 | BD002017.1 | HI207771.1 | GN359481.1 | DL118627.1 | HI000282.1 |
| DL109692.1 | CQ799270.1 | DL102900.1 | DD418579.1 | E54589.1 | HI583948.1 | GN359156.1 | DL114231.1 | HI000242.1 |
| DL012114.1 | CQ797806.1 | DL102868.1 | DD412384.1 | BD001724.1 | HI583849.1 | GN359092.1 | DL114199.1 | HI000198.1 |
| DL012082.1 | CQ795503.1 | DL102836.1 | DD412049.1 | AX451704.1 | HI577087.1 | GN359062.1 | DL114167.1 | HI000142.1 |
| DL012050.1 | CQ795466.1 | DL102804.1 | DD411472.1 | A34177.1 | HI520874.1 | GN343910.1 | DL114135.1 | HI000110.1 |
| DL012018.1 | CQ788536.1 | DL102772.1 | DD410769.1 | AX440953.1 | HI520492.1 | GN349499.1 | DL114103.1 | HI471223.1 |
| DL016482.1 | CQ787465.1 | DL102740.1 | DD417723.1 | AX430083.1 | HI207622.1 | GN340550.1 | DL114071.1 | HI204472.1 |
| DL020805.1 | CQ787429.1 | DL097531.1 | DD410182.1 | AX429284.1 | HI003850.1 | GN337631.1 | DL089770.1 | HI204250.1 |
| AX824417.1 | CQ786564.1 | DL097499.1 | CS491167.1 | AX428408.1 | HI003709.1 | GN336556.1 | DL126211.1 | HI470579.1 |
| AX824327.1 | CQ784685.1 | DL097467.1 | CS490627.1 | AX418264.1 | HD048980.1 | GN112639.1 | DL126179.1 | HI551382.1 |
| AX823793.1 | CQ778515.1 | DL121294.1 | CS486784.1 | AX411719.1 | HD052442.1 | GN113557.1 | DL122963.1 | HI568864.1 |
| AX817189.1 | CQ774658.1 | DL121262.1 | CS483124.1 | AX399391.1 | HD033679.1 | GM645480.1 | DL122931.1 | HI564221.1 |
| AX816155.1 | CQ759682.1 | DL121230.1 | CS482968.1 | AX393431.1 | HD051843.1 | GM645448.1 | DL122899.1 | HI564104.1 |
| AX813412.1 | CQ756686.1 | DL121198.1 | CS482893.1 | AX392310.1 | HD057698.1 | GM645416.1 | DL122867.1 | HI544153.1 |
| AX799984.1 | CQ754051.1 | DL101892.1 | CS484798.1 | HW350993.1 | HD057666.1 | GM624388.1 | DL122835.1 | HI585956.1 |
| AX799563.1 | HW366489.1 | DL101796.1 | DD401438.1 | HW350961.1 | HD057652.1 | GM638344.1 | DL122803.1 | HC499875.1 |
| AX798500.1 | HW381360.1 | DL101764.1 | DD401406.1 | HW350885.1 | HD062026.1 | GM643135.1 | DL122771.1 | FW304068.1 |
| AX255837.1 | HW353575.1 | DL101732.1 | DD405189.1 | HW340461.1 | HD061776.1 | FB506766.1 | DL118517.1 | FW302611.1 |
| AX253433.1 | HW249202.1 | DL097318.1 | DD405157.1 | HW350662.1 | HD061744.1 | GM046076.1 | DL114025.1 | FW300633.1 |
| AX242317.1 | HW267402.1 | DL097286.1 | DD405125.1 | HW350294.1 | M27786.1 | GM715042.1 | DL113961.1 | FW298802.1 |
| AX242285.1 | HW266132.1 | DL097254.1 | DD405061.1 | HW339864.1 | M60649.1 | GM045838.1 | DL113929.1 | HC469546.1 |
| AX242221.1 | HW266045.1 | DL097222.1 | DD405029.1 | HW339636.1 | M21408.1 | GM037896.1 | DL113897.1 | HC486529.1 |
| AX242189.1 | HW265868.1 | DL097190.1 | DD406924.1 | HW363630.1 | HC918608.1 | GM036714.1 | DL113865.1 | HC486468.1 |
| AX242157.1 | HW263041.1 | DL097158.1 | DD410096.1 | HW355324.1 | HC917616.1 | FB766248.1 | DL109064.1 | HC475393.1 |
| AX242125.1 | HW262977.1 | DL095330.1 | DD403442.1 | HW260463.1 | FW334452.1 | FB766148.1 | DL109032.1 | HC466660.1 |
| AX242093.1 | HW262834.1 | DL095234.1 | DD402404.1 | HW260431.1 | FW340295.1 | HV753616.1 | DL109000.1 | HC491771.1 |
| AX242029.1 | HW262740.1 | DL095202.1 | DD402372.1 | HW260367.1 | FW342477.1 | HV753492.1 | DL104298.1 | HC491739.1 |
| AX241965.1 | HW261420.1 | DL095170.1 | DD402340.1 | HW260303.1 | FW341726.1 | HV509844.1 | DL104266.1 | HC473849.1 |
| AX241901.1 | HW261388.1 | DL125337.1 | DD402308.1 | HW260271.1 | FW336783.1 | HV509295.1 | DL104202.1 | HC491066.1 |
| AX241869.1 | HW261356.1 | DL125305.1 | DD402276.1 | HW260207.1 | FW335899.1 | HV508633.1 | DL104170.1 | FV533445.1 |
| AX241805.1 | HW261324.1 | DL125273.1 | DD402244.1 | HW260175.1 | HC868229.1 | HV508569.1 | DL104138.1 | FV531694.1 |
| AX241773.1 | HW261292.1 | DL125241.1 | DD402212.1 | HW260143.1 | HC868090.1 | HV508537.1 | DL099596.1 | FV530989.1 |
| AX241741.1 | HW261260.1 | DL125209.1 | DD402180.1 | HW260079.1 | HC868038.1 | HV508505.1 | DL099564.1 | FV530891.1 |
| AX241709.1 | HW261228.1 | DL125177.1 | DD402148.1 | HW260047.1 | HC867837.1 | HV508403.1 | DL099532.1 | FV522832.1 |
| AX241677.1 | HW261164.1 | DL091362.1 | DD402084.1 | HW260015.1 | HC867754.1 | HV512247.1 | DL093522.1 | FV522792.1 |
| AX241645.1 | HW261132.1 | DL116597.1 | DD405772.1 | HW259983.1 | HC867696.1 | HV512215.1 | DL093490.1 | GN360136.1 |
| AX241517.1 | HW261100.1 | DL116565.1 | DD405740.1 | HW259951.1 | HC867658.1 | HV512183.1 | DL093458.1 | GN356185.1 |
| AX241485.1 | HW261068.1 | DL116533.1 | DD405708.1 | HW259919.1 | HC867570.1 | HV512151.1 | DL093426.1 | GN360031.1 |

| | | | | | | | | |
|---|---|---|---|---|---|---|---|---|
| AX241421.1 | HW261036.1 | DL116501.1 | DD405676.1 | HW258922.1 | HC867481.1 | HV512119.1 | DL093362.1 | GN359762.1 |
| AX205112.1 | HW261004.1 | DL116469.1 | DD405644.1 | HW258854.1 | HC867394.1 | HV491627.1 | DL089619.1 | GN359582.1 |
| AX203097.1 | HW260940.1 | DL116437.1 | CS330146.1 | HW257572.1 | HC882468.1 | HV455599.1 | DL126139.1 | GN359486.1 |
| AX180721.1 | HW260908.1 | DL111844.1 | CS329668.1 | HW257402.1 | HC856105.1 | HQ161057.1 | DL126107.1 | GN365497.1 |
| AX180078.1 | HW260876.1 | DL111780.1 | FB721899.1 | HW257338.1 | HC869765.1 | FW582918.1 | DL126075.1 | GN359450.1 |
| AX175076.1 | HW260844.1 | DL111748.1 | FB720254.1 | HW257306.1 | HC869710.1 | FW590741.1 | DL126043.1 | GN359354.1 |
| AX172510.1 | HW260812.1 | DL111716.1 | DL115518.1 | HW257146.1 | HC869614.1 | FW575894.1 | DL126011.1 | GN359322.1 |
| AX172452.1 | HW260780.1 | DL111684.1 | DL115486.1 | HW257050.1 | HC053837.1 | FW575461.1 | DL125979.1 | GN364828.1 |
| AY034054.1 | HW260748.1 | DL128895.1 | DL115454.1 | HW256986.1 | HC051953.1 | FW568778.1 | DL122755.1 | GN359249.1 |
| AX146306.1 | HW260716.1 | DL128861.1 | DL105670.1 | HW256858.1 | HC051661.1 | FW570986.1 | DL122699.1 | AX034875.1 |
| AX145719.1 | HW260684.1 | DL128828.1 | DL105638.1 | HW251172.1 | HC051245.1 | FW570778.1 | DL122667.1 | AX028776.1 |
| AX145687.1 | HW260652.1 | DL128796.1 | DL105606.1 | HW250837.1 | HC050173.1 | HI001329.1 | DL118381.1 | AX027730.1 |
| AX145655.1 | HW260620.1 | DL128763.1 | DL100865.1 | HW247868.1 | HC045512.1 | HI003212.1 | DL118349.1 | AX026029.1 |
| AX145623.1 | HW260588.1 | DL128731.1 | DL100833.1 | HW247775.1 | HC045479.1 | HI003096.1 | DL118317.1 | AX024954.1 |
| AX145591.1 | HW260556.1 | DL116395.1 | DL100801.1 | HW240978.1 | HC045447.1 | HI003054.1 | DL118285.1 | AX023595.1 |
| AX145559.1 | HW260524.1 | DL116363.1 | DL100769.1 | HW247719.1 | HC045415.1 | HI003014.1 | DL118253.1 | AX020994.1 |
| AX145527.1 | HW260492.1 | DL111642.1 | DL100737.1 | HW240634.1 | HC045351.1 | HI002951.1 | DL118221.1 | AX020361.1 |
| AX145495.1 | HW260460.1 | DL111610.1 | DL096323.1 | HW240602.1 | HC045319.1 | HI002914.1 | DL113825.1 | AX019212.1 |
| AX145463.1 | HW260428.1 | DL111578.1 | DL096291.1 | HW239420.1 | HC045287.1 | HI001280.1 | DL113793.1 | AX009733.1 |
| AX145431.1 | HW260396.1 | DL111546.1 | DL096259.1 | HW238947.1 | HC042799.1 | HI001231.1 | DL113731.1 | AX008545.1 |
| AX145399.1 | HW260364.1 | DL111514.1 | DL096227.1 | HW238384.1 | HC047467.1 | HI001180.1 | DL113753.1 | AX006474.1 |
| AX145366.1 | HW260332.1 | DL101700.1 | DL096195.1 | HW238244.1 | HC047435.1 | HI001109.1 | DL113697.1 | AX004804.1 |
| AX145334.1 | HW260300.1 | DL101668.1 | DL096163.1 | HW238021.1 | HC047403.1 | HI001087.1 | DL113665.1 | AX002977.1 |
| AX145302.1 | HW260268.1 | DL101636.1 | DL120183.1 | HV549605.1 | HC047371.1 | HI001031.1 | DL108864.1 | AF034783.1 |
| AX145270.1 | HW260204.1 | DL101604.1 | DL120151.1 | HV549100.1 | HC047339.1 | HI000997.1 | DL108832.1 | A41483.1 |
| AX145238.1 | HW260172.1 | DL097126.1 | DL120119.1 | HV544371.1 | HC047307.1 | HI000950.1 | DL108800.1 | A24434.1 |
| AX145206.1 | HW260140.1 | DL097094.1 | DL120087.1 | HV544215.1 | DL480008.1 | HI002853.1 | DL108768.1 | A35718.1 |
| HW259925.1 | HW260108.1 | DL097062.1 | DL120055.1 | HV547855.1 | DL479435.1 | HI002821.1 | DL011940.1 | A35502.1 |
| HW258932.1 | HW260076.1 | DL097030.1 | DL120023.1 | HV547823.1 | DL465764.1 | HI002776.1 | DL011908.1 | HW288905.1 |
| HW258872.1 | HW260044.1 | DL095138.1 | DL094341.1 | HV538622.1 | DL477849.1 | HI002721.1 | DL011876.1 | HW287774.1 |
| HW258823.1 | HW260012.1 | DL095106.1 | DL094309.1 | HV543194.1 | DL464762.1 | HI002676.1 | DL011844.1 | HW268995.1 |
| HW257408.1 | CS119323.1 | DL095074.1 | DL094277.1 | HV542022.1 | DL464576.1 | HI002638.1 | DL011812.1 | HW285160.1 |
| HW257376.1 | CS119289.1 | DL095042.1 | DL094213.1 | HV541507.1 | DL470954.1 | HI002603.1 | DL024351.1 | HW269954.1 |
| HW257312.1 | CS119225.1 | DL094978.1 | DL094181.1 | HV515082.1 | DL477020.1 | HI002553.1 | DL024319.1 | HW269778.1 |
| HW257216.1 | CS119193.1 | DL094946.1 | DL094149.1 | HV534817.1 | DL476639.1 | HI002491.1 | DL024287.1 | HW269297.1 |
| HW256864.1 | CS119161.1 | DL106664.1 | DL090466.1 | HV532703.1 | DL462986.1 | HI004503.1 | DL024255.1 | HW269265.1 |
| HW256704.1 | CS119129.1 | DL106568.1 | DL090434.1 | HV515806.1 | DL481987.1 | HI002408.1 | DL024191.1 | HW269110.1 |
| HW254334.1 | CS119096.1 | DL106536.1 | DL090402.1 | HV515597.1 | DL481721.1 | HI002331.1 | DL040875.1 | HW269078.1 |
| HW251048.1 | CS119062.1 | DL106504.1 | DL090370.1 | HV515501.1 | DL460988.1 | HI000539.1 | DL040843.1 | HW249165.1 |
| HW240856.1 | CS119029.1 | DL124444.1 | DL086301.1 | HV515469.1 | GM746257.1 | HI180581.1 | DL040811.1 | HW267638.1 |
| HW247746.1 | CS118996.1 | DL124412.1 | DL086269.1 | HV515437.1 | GM712143.1 | HI178233.1 | DL040779.1 | HW267395.1 |
| HW239295.1 | CS118964.1 | DL124380.1 | DL086205.1 | HV515405.1 | GM631445.1 | HI214575.1 | DL016414.1 | HW266288.1 |
| HW239145.1 | CS118931.1 | DL120291.1 | DL110320.1 | HV515373.1 | GM631413.1 | HI214543.1 | DL016350.1 | HW266191.1 |
| HW238551.1 | CS118898.1 | DL120259.1 | DL110288.1 | HV515341.1 | GM631381.1 | HI179728.1 | DL016318.1 | HW266162.1 |
| HW238256.1 | CS118865.1 | DL120227.1 | DL105451.1 | HV515309.1 | GM631349.1 | HI214445.1 | DL016286.1 | HW266130.1 |
| HW244243.1 | CS118832.1 | DL115824.1 | DL105419.1 | HV515277.1 | GM631317.1 | HI213795.1 | DL028155.1 | HW266000.1 |
| HW237927.1 | CS118799.1 | DL115792.1 | DL105387.1 | HV515245.1 | GM622345.1 | HI181604.1 | DL011738.1 | HW265866.1 |

| | | | | | | | | |
|---|---|---|---|---|---|---|---|---|
| HW237842.1 | CS118767.1 | DL115760.1 | DL105355.1 | HV511423.1 | GM657515.1 | HI213018.1 | DL011706.1 | HW264496.1 |
| HW248667.1 | CS118670.1 | DL115728.1 | DL105323.1 | HV507794.1 | GM657483.1 | HI212978.1 | DL116811.1 | HW263039.1 |
| HW242386.1 | AX144618.1 | DL115696.1 | DL124342.1 | HI401105.1 | GM657451.1 | HI546330.1 | DL116779.1 | HW262975.1 |
| HW237371.1 | AX144559.1 | DL115664.1 | DL124310.1 | HI637684.1 | GM657419.1 | HI546298.1 | DL116747.1 | HW262827.1 |
| HW242306.1 | AX144237.1 | DL115632.1 | DL110171.1 | HI378113.1 | GM657387.1 | HI210976.1 | DL111962.1 | HW262738.1 |
| HW237040.1 | AX144173.1 | DL111238.1 | DL110139.1 | HI375904.1 | GM657355.1 | HI210944.1 | DL111930.1 | HW262590.1 |
| HW243340.1 | AX144045.1 | DL111142.1 | DL110107.1 | HI373214.1 | GM650163.1 | HC733820.1 | DL111898.1 | HW261418.1 |
| HV512161.1 | AX143787.1 | DL111110.1 | DL105174.1 | HI371031.1 | GM650131.1 | HC688459.1 | DL106863.1 | HW261386.1 |
| HV512129.1 | AX143339.1 | DL111078.1 | DL105142.1 | HH977295.1 | GM650099.1 | HC687198.1 | DL106831.1 | HW261354.1 |
| HV504760.1 | AX143275.1 | DL106274.1 | DL105110.1 | HH980617.1 | GM650067.1 | HC490846.1 | DL106799.1 | HW261322.1 |
| HV504728.1 | AX143211.1 | DL106242.1 | DL100600.1 | HH980513.1 | GM650035.1 | HC490814.1 | DL106767.1 | HW261290.1 |
| HV504696.1 | AX143147.1 | DL106210.1 | DL100568.1 | HH980445.1 | GM650003.1 | HC490718.1 | DL106735.1 | HW261226.1 |
| HV504664.1 | AX143083.1 | DL106178.1 | DL100536.1 | HH980372.1 | GM643195.1 | HC490686.1 | DL106703.1 | HW261194.1 |
| HV504632.1 | AX143019.1 | DL101405.1 | DL119990.1 | HH980340.1 | GM643130.1 | HC678797.1 | DL091273.1 | HW261162.1 |
| HV504600.1 | AX142827.1 | DL101373.1 | DL119958.1 | HH980256.1 | GM643098.1 | HC510430.1 | DL091241.1 | HW261130.1 |
| HV504983.1 | AX142763.1 | DL101341.1 | DL049638.1 | HH980178.1 | GM643066.1 | HC508649.1 | DL091209.1 | HW261098.1 |
| HV503805.1 | AX142571.1 | DL106071.1 | DL049606.1 | HH980140.1 | GM643034.1 | HC504662.1 | DL087369.1 | HW261066.1 |
| HV500906.1 | AX142505.1 | DL106039.1 | DL041559.1 | HH996667.1 | GM636013.1 | HC502242.1 | DL087337.1 | HW261034.1 |
| HV502774.1 | AX142441.1 | DL106007.1 | DL041527.1 | HV950605.1 | GM635981.1 | DM209280.1 | DL087305.1 | HW261002.1 |
| HV502742.1 | AX142377.1 | DL105975.1 | DL037672.1 | HV947301.1 | GM635949.1 | HC003007.1 | DL087273.1 | HW260970.1 |
| HV502710.1 | AX142185.1 | DL105943.1 | DL037640.1 | HV947122.1 | GM635917.1 | HB865030.1 | DL087241.1 | HW260938.1 |
| HV502678.1 | AX142121.1 | DL105911.1 | DL037608.1 | HV951953.1 | GM635885.1 | HB864974.1 | DL087209.1 | HW260906.1 |
| HV502646.1 | AX141797.1 | DL101298.1 | DL037576.1 | HV939723.1 | GM631282.1 | HB864990.1 | DL102102.1 | HW260874.1 |
| GN067879.1 | AX141605.1 | DL101266.1 | DL037544.1 | HV943295.1 | GM631250.1 | HB864934.1 | DL102070.1 | HW260842.1 |
| GN067595.1 | AX141541.1 | DL101234.1 | DL037512.1 | HV943227.1 | GM631218.1 | HB864902.1 | DL102038.1 | HW260810.1 |
| GN052372.1 | AX141413.1 | DL101202.1 | DL033309.1 | FW561794.1 | GM631186.1 | HB864870.1 | DL102006.1 | HW260778.1 |
| GN059786.1 | AX127755.1 | DL101170.1 | DL033277.1 | FW555609.1 | GM631068.1 | HB864838.1 | DL101974.1 | HW260746.1 |
| GN051254.1 | AX113858.1 | DL096724.1 | DL033245.1 | FW555577.1 | GM631036.1 | HB864806.1 | DL101942.1 | HW260714.1 |
| GN047360.1 | AX113692.1 | DL096692.1 | DL033213.1 | FW560490.1 | GM631096.1 | HB864774.1 | DL097528.1 | HW260682.1 |
| DM010443.1 | AX112248.1 | DL096660.1 | DL033181.1 | FW507406.1 | GM622321.1 | DM192906.1 | DL097496.1 | HW260650.1 |
| DM023754.1 | AX111606.1 | DL096628.1 | DL033149.1 | HI930654.1 | GM622289.1 | DM195144.1 | DL097464.1 | HW260618.1 |
| DM016050.1 | AX107888.1 | DL096596.1 | DL033117.1 | HI930269.1 | GM622257.1 | DM189898.1 | DL121291.1 | HW260586.1 |
| DM026463.1 | AX088389.1 | DL096564.1 | DL029109.1 | HI935075.1 | GM622225.1 | HB828402.1 | DL121259.1 | HW260554.1 |
| DM026239.1 | AX083740.1 | DL094736.1 | DL029077.1 | HI918277.1 | GM622193.1 | HB847769.1 | DL121227.1 | HW260522.1 |
| GM717407.1 | HW340845.1 | DL094704.1 | DL029045.1 | FW310531.1 | GM622161.1 | HB839489.1 | DL101889.1 | HW260490.1 |
| GM656142.1 | HW351109.1 | DL094640.1 | DL029013.1 | FW308498.1 | GM631020.1 | HB838784.1 | DL101825.1 | HW260458.1 |
| GM648957.1 | HW351077.1 | DL094608.1 | DL028981.1 | FW333481.1 | GM630988.1 | HB847030.1 | DL101793.1 | HW260426.1 |
| GM634550.1 | HW351045.1 | DL094576.1 | DL028949.1 | FW307699.1 | GM630956.1 | HB838740.1 | DL101761.1 | HW260394.1 |
| GM634518.1 | HW350951.1 | DL090961.1 | DL024975.1 | FW332926.1 | GM630924.1 | HB846177.1 | DL101729.1 | HW260362.1 |
| GM634486.1 | HW340500.1 | DL090929.1 | DL021763.1 | FW332324.1 | GM622117.1 | HB837833.1 | DL097219.1 | HW260298.1 |
| GM629681.1 | HW350875.1 | DL090897.1 | DL021731.1 | FW305528.1 | GM622085.1 | GM639234.1 | DL097187.1 | HW260266.1 |
| GM664572.1 | HW350657.1 | DL086961.1 | DL021699.1 | HC757685.1 | GM622053.1 | GM639202.1 | DL097155.1 | HW260234.1 |
| GM648733.1 | HW350358.1 | DL086929.1 | DL021667.1 | HC687199.1 | GM622021.1 | GM639170.1 | DL095295.1 | HW260202.1 |
| GM648701.1 | HW339716.1 | DL086897.1 | DL021635.1 | HC490847.1 | GM621989.1 | GM639138.1 | DL095231.1 | HW260170.1 |
| GM648669.1 | HW339570.1 | DL086865.1 | DL017397.1 | HC490815.1 | GM621957.1 | GM639106.1 | DL095199.1 | HW260138.1 |
| GM648637.1 | HW349780.1 | DL121927.1 | DL017365.1 | HC490783.1 | GM635824.1 | GM639074.1 | DL125334.1 | HW260106.1 |
| GM648605.1 | HW349607.1 | DL121895.1 | DL017333.1 | HC490719.1 | GM635792.1 | GM626495.1 | DL125302.1 | HW260074.1 |

| | | | | | | | | |
|---|---|---|---|---|---|---|---|---|
| GM634453.1 | HW348566.1 | DL121863.1 | DL012976.1 | HC490687.1 | GM635760.1 | GM626463.1 | DL125270.1 | HW260042.1 |
| GM629466.1 | HW347804.1 | DL121831.1 | DL041352.1 | HC299875.1 | GM635728.1 | GM626431.1 | DL125238.1 | HW260010.1 |
| GM655744.1 | HW347708.1 | DL121799.1 | DL041288.1 | HC306144.1 | GM635664.1 | GM626399.1 | DL125206.1 | HW259978.1 |
| FB727185.1 | HW347676.1 | DL117536.1 | DL037401.1 | HC306104.1 | GM630860.1 | GM626367.1 | DL125174.1 | HW259946.1 |
| GM615671.1 | HW347644.1 | DL117504.1 | DL037369.1 | HC306064.1 | GM630828.1 | GM626335.1 | DL104265.1 | HW259914.1 |
| GM042862.1 | HW347611.1 | DL117472.1 | DL037337.1 | HC306024.1 | GM630796.1 | GM660520.1 | DL104233.1 | HW259791.1 |
| GM721254.1 | HW347509.1 | DL117440.1 | DL021492.1 | HC305984.1 | GM630732.1 | GM660488.1 | DL104169.1 | HW259509.1 |
| GM839626.1 | HW347539.1 | DL112761.1 | DL021460.1 | HC302630.1 | GM630700.1 | GM660456.1 | DL104137.1 | HW259415.1 |
| FB702166.1 | HW347483.1 | DL112729.1 | DL014648.1 | HC305633.1 | GM621925.1 | GM660392.1 | DL099595.1 | HW259353.1 |
| GM061222.1 | HW363656.1 | DL112697.1 | DL010173.1 | HC305373.1 | GM621893.1 | GM660360.1 | DL099563.1 | HW259180.1 |
| DL258518.1 | HW363625.1 | DL112665.1 | DD089935.1 | HC289359.1 | GM621861.1 | GM653173.1 | DL099531.1 | HW258916.1 |
| DL241168.1 | HW347330.1 | DL107732.1 | DD103258.1 | HC289327.1 | GM621829.1 | GM653141.1 | DL093521.1 | HW257429.1 |
| DL241023.1 | HW347370.1 | DL103096.1 | DD102532.1 | HC292660.1 | GM621797.1 | GM653109.1 | DL093489.1 | HW257397.1 |
| FB748855.1 | HW347058.1 | DL103064.1 | DD052068.1 | HC295462.1 | GM621765.1 | GM653077.1 | DL093457.1 | HW257365.1 |
| FB674295.1 | HW355241.1 | DL103032.1 | DD057941.1 | HC295253.1 | GM657326.1 | DL101830.1 | DL093425.1 | HW096237.1 |
| DL236214.1 | HW355203.1 | DL098323.1 | DD057909.1 | HC292110.1 | GM657294.1 | DL101839.1 | DL093393.1 | HW086520.1 |
| DL231398.1 | HW344756.1 | DL098291.1 | DD057877.1 | HC291930.1 | GM657230.1 | DL101800.1 | DL093361.1 | HW072169.1 |
| DL119478.1 | HW344708.1 | DL098259.1 | DD057845.1 | HC299201.1 | GM657198.1 | DL101768.1 | DL126138.1 | HW104656.1 |
| DL119446.1 | HW344520.1 | DL098227.1 | DD054767.1 | HC299165.1 | GM657166.1 | DL101736.1 | DL126106.1 | HW104622.1 |
| DL119414.1 | HW344469.1 | DL098195.1 | DD053305.1 | HC291678.1 | GM649974.1 | DL097322.1 | DL126074.1 | HW104550.1 |
| DL114819.1 | HW344601.1 | DL098163.1 | DD052231.1 | HC294446.1 | GM649942.1 | DL097290.1 | DL126042.1 | HW104518.1 |
| DL114787.1 | HW344674.1 | DL092153.1 | DD052176.1 | HC293882.1 | GM649878.1 | DL097258.1 | DL126010.1 | HW072000.1 |
| DL114755.1 | HW344554.1 | DL092121.1 | CQ784661.1 | HC293702.1 | GM649846.1 | DL097226.1 | DL125978.1 | HW104469.1 |
| DL114723.1 | HW344735.1 | DL092089.1 | CQ784471.1 | HC289965.1 | GM649814.1 | DL097194.1 | DL122754.1 | HW104349.1 |
| DL114691.1 | HW344503.1 | DL092025.1 | CQ772701.1 | HC293008.1 | GM643006.1 | DL097162.1 | DL122698.1 | HW104317.1 |
| DL114659.1 | HW344635.1 | DL091993.1 | CQ771593.1 | HC201527.1 | GM642974.1 | DL095334.1 | DL122666.1 | HW104283.1 |
| DL127286.1 | HW317165.1 | FB332229.1 | CQ768334.1 | HC199473.1 | GM642941.1 | DL095302.1 | DL122602.1 | HW071097.1 |
| DL141578.1 | HW326523.1 | FB299282.1 | CQ766921.1 | HC199111.1 | GM642909.1 | DL095270.1 | DL118380.1 | HW070699.1 |
| DL123493.1 | HW326367.1 | CS368084.1 | CQ759549.1 | HC194276.1 | GM642877.1 | DL095238.1 | DL118348.1 | HW104241.1 |
| DL123461.1 | HW326326.1 | CS367956.1 | CQ758819.1 | HC089563.1 | GM642845.1 | DL095206.1 | DL113824.1 | HW104201.1 |
| DL123429.1 | HW326285.1 | CS367828.1 | CQ756623.1 | HC089531.1 | GM642813.1 | DL095174.1 | DL113792.1 | HW104121.1 |
| DL123397.1 | HW338966.1 | DL029314.1 | CQ754692.1 | HC089499.1 | GM635631.1 | DL125341.1 | DL113732.1 | HW104048.1 |
| DL123365.1 | HW338894.1 | DL029282.1 | CQ754039.1 | DM065115.1 | HH996779.1 | DL125309.1 | DL113752.1 | HW103888.1 |
| DL119111.1 | HW338638.1 | DL046010.1 | AY220728.1 | DM064150.1 | HH994571.1 | DL125277.1 | DL113696.1 | HW099560.1 |
| DL119079.1 | HW338510.1 | DL045978.1 | AX963140.1 | GN091350.1 | HH987444.1 | DL125245.1 | DL113664.1 | HW099509.1 |
| DL119047.1 | HW338382.1 | DL069479.1 | AX962035.1 | GN090772.1 | HH954713.1 | DL125213.1 | DL108863.1 | HW067598.1 |
| DL119015.1 | HW338254.1 | DL049716.1 | AX960369.1 | GN089882.1 | HH962800.1 | DL125181.1 | DL108831.1 | HW067369.1 |
| DL114491.1 | DL102594.1 | DL049684.1 | AX959319.1 | GN089818.1 | HH977284.1 | DL106695.1 | DL108799.1 | HW088847.1 |
| DL114459.1 | DL102562.1 | DL049652.1 | BD006297.1 | GN082904.1 | HH980610.1 | DL121164.1 | DL108767.1 | HW083240.1 |
| DL095937.1 | DL102530.1 | DL041861.1 | AX952146.1 | GN080159.1 | HH980506.1 | DL121100.1 | DL108735.1 | HW083208.1 |
| DL095905.1 | DL098020.1 | DL041829.1 | AX935361.1 | GN082357.1 | HH980438.1 | DL121036.1 | DL104057.1 | HW088688.1 |
| DL095873.1 | DL097988.1 | DL041797.1 | AX923407.1 | GN075919.1 | HH957941.1 | DL121004.1 | DL099491.1 | HW088579.1 |
| DL114424.1 | DL097956.1 | DL041765.1 | AX923373.1 | L09139.1 | HH980365.1 | DL116601.1 | DL099459.1 | HW081883.1 |
| DL114392.1 | DL112646.1 | DL041733.1 | AX825035.1 | L08920.1 | HH980317.1 | DL116569.1 | DL099427.1 | HW081627.1 |
| DJ492755.1 | DL112614.1 | DL041701.1 | AX824448.1 | DM058801.1 | HH980168.1 | DL116537.1 | DL099395.1 | HW081591.1 |
| DJ491578.1 | DL112582.1 | DL037878.1 | AX824338.1 | DM048642.1 | HH980133.1 | DL116505.1 | DL099363.1 | HW085249.1 |
| DL007777.1 | DL112550.1 | DL037846.1 | AX823873.1 | GM648884.1 | HH980062.1 | DL116473.1 | DL099331.1 | HW099471.1 |

| | | | | | | | | |
|---|---|---|---|---|---|---|---|---|
| DJ446845.1 | DL112518.1 | DL037814.1 | AX822468.1 | GM664825.1 | HH979888.1 | DL116441.1 | DL089546.1 | HW103550.1 |
| DJ446141.1 | DL112486.1 | DL037750.1 | AX818076.1 | GM037885.1 | HH996729.1 | DL111848.1 | DL089514.1 | HW084532.1 |
| DJ445558.1 | DL097929.1 | DL037718.1 | AX816097.1 | FB766246.1 | HH996657.1 | DL111816.1 | DL089482.1 | HW103430.1 |
| DJ440544.1 | DL097897.1 | DL037686.1 | AX814471.1 | FB766139.1 | HH996617.1 | DL111752.1 | DL089450.1 | HW102879.1 |
| DJ444658.1 | DL097865.1 | DL049611.1 | AX814293.1 | FB765674.1 | HH996555.1 | DL111720.1 | DL089418.1 | HW102815.1 |
| DJ444591.1 | DL097833.1 | DL049483.1 | AX802921.1 | FB747854.1 | HH998468.1 | DL111688.1 | DL089386.1 | HW058666.1 |
| DJ439129.1 | DL097801.1 | DL041660.1 | AX384580.1 | FB763992.1 | HH998420.1 | DL128899.1 | DL113616.1 | HW062133.1 |
| DJ433809.1 | DL097769.1 | DL041628.1 | AX382506.1 | FB743942.1 | HH998374.1 | DL128866.1 | HV503270.1 | HW061942.1 |
| DJ438342.1 | DL097737.1 | DL041596.1 | AX376957.1 | FB743909.1 | HH998341.1 | DL128832.1 | HV503238.1 | HW061863.1 |
| DJ438307.1 | DL095678.1 | DL041564.1 | AX370659.1 | FB743877.1 | HH999959.1 | DL128800.1 | HV503206.1 | HW061831.1 |
| DJ438275.1 | DL095646.1 | DL041532.1 | AX364681.1 | FB743845.1 | FU759554.1 | DL128735.1 | HV503151.1 | HW059114.1 |
| DJ402666.1 | DL095614.1 | DL037677.1 | AX364564.1 | FB743791.1 | FU764139.1 | DL116399.1 | HV503087.1 | HW061751.1 |
| DJ416204.1 | DL095582.1 | DL037645.1 | AX364532.1 | FB708872.1 | FU757612.1 | DL116367.1 | HV503055.1 | HW061676.1 |
| DJ400823.1 | DL095550.1 | DL037613.1 | AX364500.1 | GM952839.1 | FU762944.1 | DL116335.1 | HV503023.1 | HW061644.1 |
| DJ400791.1 | DL117387.1 | DL037581.1 | AX364468.1 | GM890137.1 | FU762435.1 | DL116303.1 | HV502991.1 | HW061602.1 |
| DJ398705.1 | DL117355.1 | DL037549.1 | AX364436.1 | GM963589.1 | FU756334.1 | DL116271.1 | HV502959.1 | HW061560.1 |
| DJ391327.1 | DL117323.1 | DL037517.1 | AX364404.1 | GM889510.1 | FU762092.1 | DL116239.1 | HV502927.1 | HW058846.1 |
| DD261100.1 | DL117291.1 | DL033314.1 | AX364372.1 | GM008880.1 | HC438978.1 | DL111646.1 | HV502895.1 | HW065311.1 |
| DD260302.1 | DL117259.1 | DL033282.1 | AX364340.1 | DL038018.1 | HC438426.1 | DL111614.1 | HV502831.1 | HW065279.1 |
| DD259334.1 | DL117227.1 | DL033250.1 | AX364245.1 | DL012166.1 | HC440482.1 | DL111582.1 | HV492859.1 | HV582165.1 |
| CS297035.1 | DL091742.1 | DL033218.1 | AX364213.1 | DJ388665.1 | HC435962.1 | DL111550.1 | HV502787.1 | HV582013.1 |
| DD253214.1 | DL091710.1 | DL033186.1 | AX364180.1 | CS724468.1 | HC358230.1 | DL111518.1 | HV502755.1 | HV575489.1 |
| DD251645.1 | DL091678.1 | DL033154.1 | AX363234.1 | CS724428.1 | HC358198.1 | DL111486.1 | HV502723.1 | HV586596.1 |
| DD251507.1 | DL091646.1 | DL033122.1 | AX358641.1 | CS723755.1 | HC358067.1 | DL101704.1 | HV502691.1 | HV575295.1 |
| CS288140.1 | DL091614.1 | DL029114.1 | AX358421.1 | HH758962.1 | HC358087.1 | DL101672.1 | HV502659.1 | HV581336.1 |
| CS287612.1 | DL091582.1 | DL029082.1 | E50935.1 | HH758930.1 | HC357221.1 | DL101640.1 | HV502627.1 | HV601159.1 |
| DD240734.1 | DL087971.1 | DL029050.1 | BD001982.1 | HH758898.1 | HC320742.1 | DL101608.1 | HI424138.1 | HV585987.1 |
| DD238916.1 | DL087939.1 | DL029018.1 | E54554.1 | HH758866.1 | HC356200.1 | DL101576.1 | HI424106.1 | HV585703.1 |
| DD236735.1 | DL087907.1 | DL028986.1 | E55081.1 | HH757474.1 | HC325480.1 | DL101544.1 | HI424074.1 | HV580377.1 |
| BD291408.1 | DL087875.1 | DL028954.1 | E64645.1 | HH757442.1 | A07562.1 | DL097130.1 | HI423816.1 | HV574161.1 |
| BD291302.1 | DL087843.1 | DL024980.1 | E58945.1 | HH756255.1 | HC324979.1 | DL097098.1 | HI423784.1 | HV570609.1 |
| BD299629.1 | DL104848.1 | DL021768.1 | AX356505.1 | HI401634.1 | HC324508.1 | DL097066.1 | HI423546.1 | HV568121.1 |
| BD298370.1 | DL104816.1 | DL021736.1 | AX354676.1 | HI401245.1 | HC324086.1 | DL097034.1 | HI422771.1 | HV568057.1 |
| BD078113.1 | DL104784.1 | DL021704.1 | AX352736.1 | HI401098.1 | HC323145.1 | DL097002.1 | HI416360.1 | HV568025.1 |
| BD074969.1 | DL104752.1 | DL021672.1 | AX352339.1 | HD118202.1 | HC315546.1 | DL096970.1 | HI416150.1 | HV567993.1 |
| BD074934.1 | DL104720.1 | DL021640.1 | AX351207.1 | HI645148.1 | HC313932.1 | DL095142.1 | HI415883.1 | HV567961.1 |
| BD072840.1 | DL095925.1 | DL017402.1 | AX351097.1 | HI643050.1 | HC319652.1 | DL095110.1 | HI415818.1 | HV567905.1 |
| BD070051.1 | DL095893.1 | DL017338.1 | AX348918.1 | HI002669.1 | HC309748.1 | DL095078.1 | HI415786.1 | HV567002.1 |
| BD064987.1 | DL095861.1 | DL012981.1 | AX348469.1 | HI002635.1 | HC308857.1 | DL095046.1 | HI415754.1 | HV566117.1 |
| BD016717.1 | DL095829.1 | DL012949.1 | AX347324.1 | HI002600.1 | HC307869.1 | DL095014.1 | HH761267.1 | HV565826.1 |
| BD014471.1 | DL095797.1 | DL045629.1 | AX347286.1 | HI002550.1 | HC307804.1 | DL094982.1 | HI414041.1 | HV572130.1 |
| BD014216.1 | DL094142.1 | DL045597.1 | AX347248.1 | HI004561.1 | HC307381.1 | DL094950.1 | HI414009.1 | HV571499.1 |
| BD014176.1 | DL094110.1 | DL045565.1 | AX347216.1 | HI004433.1 | HC312265.1 | DL106636.1 | HI413446.1 | HV571427.1 |
| AX490795.1 | DL094078.1 | DL045533.1 | AX347178.1 | HI002465.1 | FU258276.1 | DL106604.1 | HI516601.1 | HV560169.1 |
| AX474384.1 | DL094046.1 | DL045469.1 | AX347038.1 | HI002405.1 | FU258244.1 | DL106572.1 | HI516527.1 | HV559558.1 |
| AX468155.1 | DL093982.1 | DL028703.1 | AX345072.1 | HI002327.1 | FU258212.1 | DL106540.1 | HI516126.1 | HV558758.1 |
| AX463608.1 | DL093950.1 | DL028671.1 | AX344534.1 | HI000535.1 | FU258180.1 | HW257140.1 | HI516084.1 | HV555482.1 |

| | | | | | | | | |
|---|---|---|---|---|---|---|---|---|
| AX458726.1 | DL090335.1 | DL028607.1 | AX343927.1 | HI180553.1 | FU258148.1 | HW257108.1 | HI547686.1 | HV562094.1 |
| AX458615.1 | DL090303.1 | DL028575.1 | AX338545.1 | HI201374.1 | FU260930.1 | HW257076.1 | HI547615.1 | HV562030.1 |
| AX454049.1 | DL090239.1 | DL024761.1 | AX328282.1 | HI180172.1 | FU260735.1 | HW257044.1 | HI284303.1 | HV561998.1 |
| AX451740.1 | DL090207.1 | DL024729.1 | AX327952.1 | HI202812.1 | FU257904.1 | HW257012.1 | HI284271.1 | HV561873.1 |
| AX451297.1 | DL090175.1 | DL024697.1 | AX306561.1 | HI177878.1 | FU257840.1 | HW256948.1 | HI571694.1 | HV561579.1 |
| A35245.1 | DL086170.1 | DL021357.1 | AX268506.1 | HI214572.1 | FU257776.1 | HW256916.1 | HI574524.1 | HV561547.1 |
| A34183.1 | DL086138.1 | DL021325.1 | AX258851.1 | HI214540.1 | FU257711.1 | HW256884.1 | HI574492.1 | HV550131.1 |
| AX441516.1 | DL086106.1 | DL016895.1 | AX301007.1 | HI177808.1 | FU260839.1 | HW256820.1 | HI574460.1 | HV554340.1 |
| A25435.1 | DL086074.1 | DL016831.1 | AX299849.1 | HI214442.1 | FU260951.1 | HW256756.1 | HI574428.1 | HV552883.1 |
| AX427156.1 | DL086042.1 | DL012373.1 | AX297550.1 | HI200554.1 | FU265381.1 | HW251349.1 | HI574396.1 | HV552734.1 |
| AX418270.1 | DL086010.1 | DL012341.1 | HW294144.1 | HI181600.1 | FU262704.1 | HW251083.1 | HI571446.1 | HV550975.1 |
| AX417873.1 | DL085997.1 | DL012309.1 | HW294111.1 | HI213015.1 | FU262666.1 | HW251027.1 | HI574373.1 | DQ377843.1 |
| AX411735.1 | DL114412.1 | DL012277.1 | HW294079.1 | HI212975.1 | FU250482.1 | HW250352.1 | HI573649.1 | AY659382.1 |
| AX406830.1 | DL114380.1 | DL012213.1 | HW294045.1 | HI547164.1 | GN373342.1 | HW247850.1 | HI553813.1 | AY659350.1 |
| AX399445.1 | DL114348.1 | DL060762.1 | HW293158.1 | HI212476.1 | GN370765.1 | HW240908.1 | HI637152.1 | AY659318.1 |
| AX397873.1 | DL109451.1 | DL048694.1 | HW291168.1 | HI203433.1 | GN360563.1 | HW240812.1 | HI636969.1 | AY659286.1 |
| AX393449.1 | DL109419.1 | DL048662.1 | CQ890967.1 | HI546327.1 | GN360133.1 | HW240780.1 | HI636931.1 | AY659254.1 |
| AX392369.1 | DL109387.1 | DL045244.1 | CQ877781.1 | HI546295.1 | GN356182.1 | HW240724.1 | HI001450.1 | AY659222.1 |
| AX384567.1 | DL109355.1 | DL045212.1 | CQ877142.1 | HI587349.1 | GN366512.1 | HW240692.1 | HI001391.1 | AY659190.1 |
| AX380803.1 | DL109323.1 | DL045180.1 | CQ875534.1 | HI210973.1 | GN366480.1 | HV951601.1 | HI001354.1 | AY659158.1 |
| AX376944.1 | DL109291.1 | DL045148.1 | CQ874706.1 | HI210941.1 | GN366448.1 | HV951569.1 | HI001320.1 | AY659126.1 |
| AX375507.1 | DL104685.1 | DL045116.1 | CQ873287.1 | HI569855.1 | GN366415.1 | HV951537.1 | HI003203.1 | AY659094.1 |
| AX367176.1 | DL104589.1 | DL045084.1 | CQ871414.1 | HI569621.1 | GN366383.1 | HV955812.1 | HI003085.1 | AY659062.1 |
| HW291155.1 | DL104557.1 | DL040972.1 | CQ871220.1 | HI566166.1 | GN366351.1 | HV950602.1 | HI003045.1 | AY659030.1 |
| HW291058.1 | DL104525.1 | DL040940.1 | CQ868902.1 | HI473070.1 | GN360028.1 | HV957876.1 | HI003004.1 | AY658998.1 |
| HW291026.1 | DL093941.1 | DL040908.1 | CQ867566.1 | HI472914.1 | GN359996.1 | HV949011.1 | HI002942.1 | AY658966.1 |
| HW290994.1 | DL093909.1 | DL037085.1 | CQ866705.1 | HI577557.1 | GN359964.1 | HV948918.1 | HI002905.1 | AY658934.1 |
| HW290962.1 | DL093877.1 | DL037053.1 | CQ860048.1 | HI209391.1 | GN366326.1 | HV947298.1 | HI001268.1 | FZ437069.1 |
| HW298837.1 | DL093845.1 | DL037021.1 | CQ859610.1 | HI072074.1 | GN366294.1 | HV947107.1 | HI001214.1 | FZ423464.1 |
| HW290899.1 | DL093813.1 | DL036989.1 | CQ858158.1 | HI004323.1 | GN366262.1 | HV952899.1 | HI001164.1 | FZ423247.1 |
| HW290867.1 | DL093781.1 | DL036957.1 | CQ858060.1 | HI002283.1 | GN359791.1 | HV952295.1 | HI001059.1 | FZ422880.1 |
| HW290545.1 | DL090070.1 | DL036925.1 | CQ855924.1 | HI002249.1 | GN359759.1 | HV952231.1 | HC471613.1 | FZ431987.1 |
| HW289985.1 | DL090038.1 | DL032690.1 | CQ855147.1 | HI002196.1 | GN359695.1 | HV951949.1 | HC728992.1 | FZ422785.1 |
| HW249435.1 | DL090006.1 | DL032658.1 | CQ846979.1 | HI002153.1 | GN359611.1 | HV946823.1 | HC728703.1 | FZ422710.1 |
| HW267764.1 | DL089974.1 | DL024548.1 | CQ840684.1 | HI002115.1 | GN359547.1 | HV946281.1 | HC727682.1 | FZ416467.1 |
| HW267679.1 | DL085905.1 | DL024516.1 | CQ828075.1 | HI000460.1 | GN359483.1 | HV940064.1 | HC688443.1 | FZ418962.1 |
| HW266056.1 | DL085873.1 | DL024484.1 | CQ821235.1 | HI000412.1 | GN365477.1 | HV945833.1 | HC686940.1 | FZ412366.1 |
| HW265960.1 | DL085841.1 | DL024452.1 | CQ818561.1 | HI000370.1 | GN359447.1 | HV945752.1 | HC490837.1 | FZ421473.1 |
| HW263050.1 | DL085809.1 | DL024388.1 | CQ817011.1 | HI000313.1 | GM645003.1 | HV945177.1 | HC490773.1 | FZ421221.1 |
| HW262984.1 | DL126708.1 | DL021176.1 | E64562.1 | HI553266.1 | GM644971.1 | HV944806.1 | HC490741.1 | FZ415248.1 |
| HW262949.1 | DL126676.1 | DL021144.1 | E58875.1 | HI553194.1 | GM644939.1 | HV943285.1 | HC490709.1 | FZ421009.1 |
| HW262841.1 | DL126644.1 | DL021112.1 | AX356496.1 | HI539188.1 | GM644907.1 | HV820271.1 | HC490677.1 | FZ414807.1 |
| HW261395.1 | DL126612.1 | DL021080.1 | AX354622.1 | HI207754.1 | GM644875.1 | HV815334.1 | HC490548.1 | FZ413929.1 |
| HW261363.1 | CS806059.1 | DL021048.1 | AX353935.1 | HC679618.1 | GM644843.1 | HV932152.1 | HC490513.1 | FZ416715.1 |
| HW261331.1 | CS675245.1 | DL021016.1 | AX352730.1 | HC490979.1 | GM638044.1 | HV931959.1 | HC686464.1 | FZ416683.1 |
| HW261299.1 | CS810213.1 | DL016810.1 | AX350850.1 | HC490947.1 | GM638012.1 | HV929276.1 | HC490410.1 | FZ419834.1 |
| HW261267.1 | CS809320.1 | DL016746.1 | AX349186.1 | HC490915.1 | GM637980.1 | HV931857.1 | HC490282.1 | FZ419800.1 |

| | | | | | | | | |
|---|---|---|---|---|---|---|---|---|
| HW261235.1 | CS791228.1 | DL016650.1 | AX349104.1 | HC490883.1 | GM637948.1 | HV925770.1 | HC490217.1 | FZ419768.1 |
| HW261203.1 | CS791004.1 | DL016618.1 | AX348905.1 | HC687195.1 | GM637916.1 | HV931463.1 | HC472303.1 | FZ419736.1 |
| HW261107.1 | CS790967.1 | DL012160.1 | AX348466.1 | HC490843.1 | GM637884.1 | HV936713.1 | HC472270.1 | FZ419701.1 |
| HW261075.1 | DJ042533.1 | DL012128.1 | AX347590.1 | HC490811.1 | GM637852.1 | HV931225.1 | HC472238.1 | FZ416536.1 |
| HW261043.1 | DJ045045.1 | DL012096.1 | AX347421.1 | HC490779.1 | GM633444.1 | HV925504.1 | HC472206.1 | HQ161056.1 |
| HW261011.1 | DJ044959.1 | DL012064.1 | AX347321.1 | HC490747.1 | GM633348.1 | HV939176.1 | HC472174.1 | FW582893.1 |
| HW260979.1 | DJ028603.1 | DL012032.1 | AX347281.1 | HC490715.1 | GM633316.1 | HV936404.1 | HC471942.1 | FW582266.1 |
| HW260947.1 | DJ044937.1 | DL016432.1 | AX347245.1 | HC490683.1 | GM633284.1 | HV936367.1 | HC471910.1 | FW591907.1 |
| HW260915.1 | DJ044896.1 | DL039773.1 | AX347213.1 | HC490651.1 | GM637829.1 | HV936317.1 | HC471878.1 | FW591753.1 |
| HW260883.1 | DJ044659.1 | DL039741.1 | AX347175.1 | HC490619.1 | GM637797.1 | HV930429.1 | HC678843.1 | FW591167.1 |
| HW260851.1 | DJ030179.1 | DL039709.1 | AX347005.1 | HC490587.1 | GM637765.1 | HV503437.1 | HC678778.1 | FW590934.1 |
| HW260819.1 | DJ030060.1 | DL036085.1 | AX345055.1 | HC490555.1 | GM637733.1 | HV503405.1 | HC678415.1 | FW590740.1 |
| HW260787.1 | DJ030012.1 | DL036053.1 | AX344529.1 | HC490519.1 | GM637701.1 | HV503373.1 | HC509930.1 | FW590672.1 |
| HW260755.1 | DJ029936.1 | DL035989.1 | AX343860.1 | HC490487.1 | GM637669.1 | HV503341.1 | CS244181.1 | FW589122.1 |
| HW260723.1 | DJ029304.1 | DL035957.1 | AX339641.1 | HC686475.1 | GM633261.1 | HV503309.1 | CS243273.1 | FW588149.1 |
| HW260691.1 | CS560465.1 | DL035925.1 | AX339351.1 | HC490448.1 | GM633229.1 | HV503277.1 | CS228106.1 | FW593176.1 |
| HW260659.1 | DD434605.1 | DL031921.1 | AX338528.1 | HC490416.1 | GM633197.1 | HV503245.1 | CS200908.1 | FW593046.1 |
| HW260627.1 | DD432682.1 | DL031889.1 | AX329385.1 | HC490384.1 | GM633165.1 | HV503213.1 | CS227254.1 | FW592985.1 |
| HW260595.1 | DD421976.1 | DL031857.1 | AX328279.1 | HC490352.1 | GM633133.1 | HV503190.1 | CS226727.1 | FW592675.1 |
| HW260563.1 | DD420777.1 | DL031825.1 | AX327949.1 | HC490320.1 | GM633101.1 | HV503158.1 | CS208776.1 | FW592643.1 |
| HW260531.1 | CS543088.1 | DL031793.1 | AX320697.1 | HC490288.1 | GM625522.1 | HV503126.1 | CS186193.1 | FW592611.1 |
| HW260499.1 | CS542564.1 | DL031761.1 | AX317529.1 | HC490223.1 | GM625490.1 | HV503094.1 | CS182203.1 | FW592579.1 |
| HW260467.1 | CS537449.1 | DL027856.1 | AX317309.1 | HC472309.1 | GM625458.1 | HV503062.1 | CS183869.1 | FW577908.1 |
| HW260435.1 | CS537184.1 | DL027824.1 | AX306688.1 | HC472276.1 | GM625426.1 | HV502966.1 | CS177450.1 | FW577601.1 |
| HW260403.1 | CS510541.1 | DL027792.1 | AX306480.1 | HC472244.1 | GM625394.1 | HV502934.1 | CS174657.1 | FW577120.1 |
| HW260371.1 | CS502715.1 | DL027760.1 | AX258845.1 | HC472212.1 | GM625362.1 | HV502870.1 | CS174645.1 | FW576962.1 |
| HW260307.1 | CS502647.1 | DL023948.1 | AX303573.1 | HC472180.1 | GM625330.1 | HV502838.1 | CS172266.1 | FW576733.1 |
| HW260275.1 | CS502583.1 | DL023916.1 | AX300960.1 | HC471948.1 | GM711158.1 | HV492867.1 | CS166442.1 | HI921902.1 |
| HW260211.1 | CS502337.1 | DL023884.1 | AX288238.1 | HC471916.1 | GM651754.1 | HV502794.1 | CS159802.1 | HI918275.1 |
| HW260179.1 | CS501254.1 | DL023852.1 | AX286673.1 | HC471884.1 | GM651722.1 | HV502762.1 | CS159576.1 | HI917099.1 |
| HW260147.1 | CS499317.1 | DL023820.1 | AX281532.1 | GM635916.1 | GM651690.1 | HV502730.1 | CS159199.1 | HI916963.1 |
| HV512287.1 | CS498532.1 | DL023788.1 | AX279943.1 | GM635884.1 | GM651658.1 | HV502698.1 | CS155786.1 | HI661445.1 |
| HV512255.1 | CS497209.1 | DL020773.1 | AX279610.1 | GM631281.1 | GM651626.1 | HV502666.1 | CS148767.1 | HI661207.1 |
| HV512223.1 | CS494774.1 | DL020741.1 | AX278272.1 | GM631095.1 | GM644794.1 | HV502634.1 | CS144351.1 | HI660601.1 |
| HV512191.1 | DD412335.1 | DL020709.1 | AX256101.1 | GM622320.1 | GM644762.1 | HV502602.1 | CS141528.1 | HI659013.1 |
| AH002304.2 | DD412071.1 | DL020677.1 | AX253572.1 | GM622288.1 | GM644730.1 | HV502570.1 | CS141496.1 | HI658950.1 |
| M20980.1 | DD411627.1 | DL020645.1 | AX252395.1 | GM622256.1 | GM637604.1 | HV502538.1 | CS141128.1 | HI658099.1 |
| M19975.1 | DD411477.1 | DL020613.1 | AX247555.1 | GM622224.1 | GM637572.1 | HV502506.1 | CS140535.1 | HI657322.1 |
| AY781341.1 | DD414324.1 | DL016210.1 | AX242325.1 | GM622192.1 | GM637540.1 | HV502474.1 | CS132040.1 | HI656353.1 |
| AF226875.1 | DD410774.1 | DL016146.1 | AX242293.1 | GM622160.1 | GM637508.1 | HV497336.1 | CS130950.1 | FW421373.1 |
| AF545505.1 | DD417770.1 | DL011592.1 | AX242261.1 | GM631019.1 | GM637476.1 | HV505353.1 | CS123560.1 | FW503027.1 |
| AH003171.2 | CS491856.1 | DL011560.1 | AX242229.1 | GM634782.1 | GM633068.1 | HV505232.1 | CS122497.1 | FW496166.1 |
| HV566133.1 | CS491738.1 | DL011528.1 | AX242197.1 | GM634750.1 | GM633036.1 | HV505010.1 | CS106082.1 | FW502863.1 |
| HV572324.1 | CS490472.1 | DL011496.1 | AX242101.1 | GM629880.1 | GM633004.1 | HV502156.1 | CS181483.1 | FW497330.1 |
| HV560183.1 | CS488273.1 | DL011464.1 | AX241941.1 | GM629848.1 | GM655231.1 | HV453668.1 | CS179747.1 | FW503894.1 |
| HV551454.1 | CS487194.1 | DL011432.1 | AX241909.1 | GM629816.1 | GM655199.1 | HV449756.1 | CS326350.1 | FW499111.1 |
| HV554543.1 | CS484383.1 | DL011400.1 | AX241877.1 | GM629752.1 | GM655167.1 | HV449722.1 | CS323677.1 | FW499038.1 |

| | | | | | | | | |
|---|---|---|---|---|---|---|---|---|
| HV551395.1 | CS483428.1 | DL031614.1 | AX241845.1 | GM629720.1 | GM648174.1 | HV453039.1 | CS323595.1 | FW504497.1 |
| HV550386.1 | CS482975.1 | DL031582.1 | AX241813.1 | GM629695.1 | GM648142.1 | HV445659.1 | DD224658.1 | FW504944.1 |
| AY659390.1 | CS482856.1 | DL031550.1 | AX241749.1 | GM697028.1 | GM648110.1 | HV451134.1 | DD231482.1 | FW504770.1 |
| AY659358.1 | CS482815.1 | DL027741.1 | HW340476.1 | GM669377.1 | GM648078.1 | HV448524.1 | DD231410.1 | FW504738.1 |
| AY659326.1 | CS482774.1 | DL027709.1 | HW350676.1 | GM656129.1 | GM648046.1 | HV448355.1 | CS276974.1 | FW500573.1 |
| AY659294.1 | DD401443.1 | DL027677.1 | HW350644.1 | GM656097.1 | GM648014.1 | DL113188.1 | A18436.1 | FW498837.1 |
| AY659262.1 | CS418675.1 | DL027645.1 | HW340169.1 | GM656065.1 | GM641211.1 | DL113156.1 | A00340.1 | FW420481.1 |
| AY659230.1 | CS414645.1 | DL027581.1 | HW339952.1 | GM656033.1 | GM641179.1 | DL113092.1 | DD212850.1 | FW420449.1 |
| AY659198.1 | CS417158.1 | DL023769.1 | HW350252.1 | GM656001.1 | GM641147.1 | DL113060.1 | DD214020.1 | FW420417.1 |
| AY659166.1 | CS416700.1 | DL023737.1 | HW349952.1 | GM655969.1 | GM641115.1 | DL108259.1 | DD216641.1 | FW501751.1 |
| AY659134.1 | CS416226.1 | DD240663.1 | HW348596.1 | GM648944.1 | GM641083.1 | DL108227.1 | DD213971.1 | FW498797.1 |
| AY659102.1 | CS415773.1 | DD240605.1 | HW348496.1 | GM648912.1 | GM641051.1 | DL108163.1 | DD213878.1 | FW497040.1 |
| AY659070.1 | CS416443.1 | DD240493.1 | HW347990.1 | GM648880.1 | GM628891.1 | DL108131.1 | DD213814.1 | FW420392.1 |
| AY659038.1 | CS410908.1 | DD240461.1 | HW363498.1 | GM648848.1 | GM628859.1 | DL108099.1 | DD216489.1 | FW420360.1 |
| AY659006.1 | CS410693.1 | DD240109.1 | HW317047.1 | GM648816.1 | GM628827.1 | DL103493.1 | DD216447.1 | FW420328.1 |
| AY658974.1 | CS410576.1 | DD238043.1 | HW326500.1 | GM642013.1 | GM628795.1 | DL103461.1 | DD213797.1 | GU271153.1 |
| AY658942.1 | CS414852.1 | DD236640.1 | HW339042.1 | GM641981.1 | GM628763.1 | DL103429.1 | DD213765.1 | FW496531.1 |
| AY658910.1 | DD326900.1 | DD235488.1 | HW338458.1 | GM641949.1 | GM628731.1 | DL103397.1 | DD213733.1 | FW501575.1 |
| AY658878.1 | DD329907.1 | DD234712.1 | HW338330.1 | GM641917.1 | GM618950.1 | DL098919.1 | DD213701.1 | HI653814.1 |
| AY658846.1 | DD326844.1 | DD248260.1 | HW338202.1 | GM641885.1 | GM628683.1 | DL098855.1 | DD213669.1 | HI653642.1 |
| AY658814.1 | DD321807.1 | DD246847.1 | HW338074.1 | GM634633.1 | GM628651.1 | DL098823.1 | DD213637.1 | HI653607.1 |
| AY658782.1 | DD321765.1 | E41527.1 | HW337818.1 | GM634601.1 | GM628619.1 | DL098791.1 | DD213605.1 | HI653537.1 |
| AY658750.1 | DD325237.1 | E46948.1 | GM658011.1 | GM634569.1 | GM628587.1 | DL098759.1 | DD213587.1 | HI653496.1 |
| AY658718.1 | CS390498.1 | E40700.1 | GM657979.1 | GM634537.1 | GM628555.1 | DL092717.1 | DD213555.1 | HI653428.1 |
| AY658686.1 | CS390084.1 | E37930.1 | GM657947.1 | GM634505.1 | GM628523.1 | DL092685.1 | DD213505.1 | HI653376.1 |
| AY658654.1 | CS389468.1 | E49567.1 | GM650755.1 | GM634473.1 | GM716108.1 | DL092653.1 | DD216035.1 | HI653235.1 |
| AY658622.1 | CS389286.1 | E06602.1 | GM650723.1 | GM629668.1 | GM654973.1 | DL092589.1 | DD216003.1 | HI651102.1 |
| AY658590.1 | CS389197.1 | E05819.1 | GM650691.1 | GM629604.1 | GM641016.1 | DL088974.1 | DD215971.1 | HI650596.1 |
| AY658558.1 | CS389159.1 | E03543.1 | GM643699.1 | GM629572.1 | GM640984.1 | DL088942.1 | DD218336.1 | HI647564.1 |
| AY658526.1 | CS400481.1 | E02922.1 | GM643667.1 | GM629540.1 | GM640920.1 | DL028525.1 | DD215893.1 | HI646318.1 |
| AY658494.1 | CS401290.1 | E02644.1 | GM636542.1 | GM629508.1 | GM640888.1 | DL028461.1 | DD215861.1 | AY145507.1 |
| AY658462.1 | CS406682.1 | E02369.1 | GM636510.1 | GM717285.1 | GM640856.1 | DL028429.1 | DD215829.1 | HH807100.1 |
| AY658430.1 | CS406441.1 | E02212.1 | GM636478.1 | GM664819.1 | GM628502.1 | DL028397.1 | DD215797.1 | HH806456.1 |
| AY658398.1 | CS406001.1 | E02061.1 | GM632070.1 | GM655937.1 | GM628470.1 | DL028365.1 | DD215765.1 | HH806390.1 |
| AY658366.1 | CS403587.1 | E01685.1 | GM643497.1 | GM655905.1 | GM628406.1 | DL024551.1 | DD217861.1 | HH806326.1 |
| AY658334.1 | CS408099.1 | E01251.1 | GM643465.1 | GM655873.1 | GM628374.1 | DL024519.1 | CS254885.1 | HH804198.1 |
| AY658302.1 | CS402203.1 | E00888.1 | GM643432.1 | GM655841.1 | GM628342.1 | DL024487.1 | DD212322.1 | HH827438.1 |
| AY658270.1 | CS402112.1 | E00618.1 | GM636443.1 | GM655809.1 | GM628310.1 | DL024455.1 | DD211113.1 | HH827406.1 |
| AY658238.1 | CS401798.1 | E00013.1 | GM631843.1 | GM655769.1 | GM662083.1 | DL024423.1 | DD191353.1 | HH827374.1 |
| AY658206.1 | CS380541.1 | DD088023.1 | GM631811.1 | GM648752.1 | GM662051.1 | DL024391.1 | DD208692.1 | HH832144.1 |
| AY658174.1 | CS382626.1 | DD146172.1 | GM631779.1 | GM648720.1 | GM662019.1 | DL021179.1 | DD187269.1 | HH827011.1 |
| AY658142.1 | CS376597.1 | DD086339.1 | GM622848.1 | GM648688.1 | GM661987.1 | DL021147.1 | DD206890.1 | HH826871.1 |
| AY658110.1 | CS376329.1 | DD069883.1 | GM657901.1 | GM648656.1 | GM661955.1 | DL021115.1 | DD206858.1 | HH826530.1 |
| AY658078.1 | DD309291.1 | DD112557.1 | GM657869.1 | GM648624.1 | GM661923.1 | DL021083.1 | DD206796.1 | HH822369.1 |
| FW381488.1 | DD309001.1 | DD143255.1 | GM657837.1 | GM648592.1 | DL120543.1 | DL021051.1 | CS249480.1 | HH822050.1 |
| FW375274.1 | DD308755.1 | DD084337.1 | GM657805.1 | GM641789.1 | DL116012.1 | DL021019.1 | DD163492.1 | HH821994.1 |
| FW369342.1 | DD291754.1 | DD098189.1 | GM657772.1 | GM641757.1 | DL115980.1 | DL016813.1 | DD163286.1 | FW379761.1 |

| | | | | | | | | |
|---|---|---|---|---|---|---|---|---|
| HH736075.1 | DD291492.1 | DD132357.1 | GM639006.1 | GM641725.1 | DL093115.1 | DL016781.1 | AX958641.1 | FW379718.1 |
| HH735638.1 | DD291023.1 | DD141441.1 | GM638974.1 | GM641693.1 | DL093083.1 | DL016749.1 | BD006298.1 | FW379686.1 |
| HH733728.1 | HV236019.1 | DD141290.1 | GM626167.1 | GM641660.1 | DL089276.1 | DL016717.1 | AX958015.1 | FW379653.1 |
| FW368416.1 | HV301809.1 | DD153585.1 | GM626135.1 | GM641628.1 | DL089244.1 | DL016685.1 | AX952259.1 | FW379621.1 |
| FW351374.1 | HV301750.1 | DD158437.1 | GM652968.1 | GM634440.1 | DL125935.1 | DL016653.1 | AX938891.1 | FW379589.1 |
| FW351212.1 | HV306187.1 | DD158416.1 | GM652936.1 | GM634408.1 | DL125903.1 | DL016621.1 | AX937039.1 | FW379579.1 |
| HD122357.1 | HV307805.1 | DD157781.1 | GM652904.1 | GM634376.1 | DL125871.1 | DL012163.1 | AX925692.1 | FW379547.1 |
| HD115788.1 | HV303112.1 | DD157356.1 | GM652872.1 | GM634344.1 | DL125839.1 | DL012131.1 | AX923408.1 | FW378304.1 |
| HC678811.1 | HV302949.1 | DD155785.1 | GM652840.1 | GM634312.1 | DL125807.1 | DL012099.1 | AX923374.1 | FW377975.1 |
| HC678744.1 | HV308866.1 | DD151839.1 | GM652808.1 | GM634280.1 | DL125775.1 | DL012067.1 | AX840316.1 | FW377826.1 |
| HC504672.1 | HV305754.1 | DD096750.1 | GM652775.1 | GM629444.1 | DL122565.1 | DL012035.1 | AX829101.1 | FW373084.1 |
| HC500963.1 | HV308545.1 | DD081761.1 | GM638786.1 | GM629379.1 | DL122533.1 | DL012003.1 | AX825040.1 | FW373052.1 |
| HC499849.1 | HV304143.1 | DD081688.1 | GM638754.1 | GM629347.1 | DL118119.1 | DL016595.1 | AX824449.1 | FW373020.1 |
| FW301869.1 | HV312313.1 | DD148364.1 | GM626111.1 | GM629315.1 | DL118087.1 | DL016563.1 | AX824339.1 | FW377115.1 |
| FW298750.1 | FZ425971.1 | DD147794.1 | GM038283.1 | GM655750.1 | DL118055.1 | DL016531.1 | AX823771.1 | FW372001.1 |
| FW298081.1 | FZ429321.1 | DD147762.1 | FB775378.1 | GM655603.1 | DL118023.1 | DL016499.1 | AX822293.1 | FW371969.1 |
| HC488002.1 | FZ437352.1 | DD147730.1 | FB774879.1 | GM655571.1 | DL113334.1 | DL016467.1 | AX818081.1 | FW371937.1 |
| HC486481.1 | FZ423962.1 | DD147698.1 | FB766223.1 | GM648578.1 | DL113302.1 | DL016435.1 | AX816098.1 | FW371905.1 |
| HC483597.1 | FZ436787.1 | DD147273.1 | FB765832.1 | GM648546.1 | DL113270.1 | DL011913.1 | AX814472.1 | FW376209.1 |
| HC474608.1 | FZ436701.1 | DD081442.1 | FB764697.1 | GM648514.1 | DL108469.1 | DL011881.1 | DL107239.1 | FW381547.1 |
| HC491778.1 | FZ423417.1 | DD080946.1 | FB764087.1 | GM648482.1 | DL108357.1 | DL011849.1 | DL107207.1 | FW376317.1 |
| HC491746.1 | FZ422773.1 | DD138764.1 | FB746526.1 | GM648450.1 | DL108341.1 | DL011817.1 | DL102466.1 | FW381131.1 |
| HC491638.1 | FZ422540.1 | DD122266.1 | FB744406.1 | GM648418.1 | DL108309.1 | DL024356.1 | DL102434.1 | FW381025.1 |
| HC488308.1 | FZ415923.1 | DD137243.1 | FB743974.1 | GM641615.1 | DL103671.1 | DL024324.1 | DL102402.1 | FW369982.1 |
| FV531701.1 | FZ421464.1 | DD136425.1 | FB743926.1 | GM641583.1 | DL103639.1 | DL024292.1 | DL102370.1 | FW374989.1 |
| FV530914.1 | FZ421397.1 | DD135765.1 | FB743894.1 | GM641551.1 | DL099065.1 | DL024228.1 | DL102338.1 | FW380000.1 |
| FV522839.1 | FZ418106.1 | DD135705.1 | FB743862.1 | GM641519.1 | DL099033.1 | DL024196.1 | DL097629.1 | FW374492.1 |
| FV522799.1 | FZ412040.1 | DD134021.1 | FB743820.1 | GM641487.1 | DL099001.1 | DL045056.1 | DL097597.1 | FW379852.1 |
| FV522616.1 | FZ421005.1 | DD132291.1 | FB761722.1 | GM641455.1 | DL098937.1 | DL045024.1 | DL097565.1 | FW369334.1 |
| HC047475.1 | FZ420876.1 | DD107473.1 | FB761224.1 | GM634267.1 | DL092831.1 | DL044992.1 | DL095538.1 | HH736053.1 |
| HC047027.1 | FZ420852.1 | DD107422.1 | FB760063.1 | DL107462.1 | DL092799.1 | DL044960.1 | DL095506.1 | HH735630.1 |
| HC046995.1 | FZ420792.1 | DD107357.1 | GM952610.1 | DL107418.1 | DL089152.1 | DL044896.1 | DL095474.1 | HH755065.1 |
| HC046963.1 | FZ417001.1 | DD093916.1 | GM890110.1 | DL107422.1 | DL089120.1 | DL044864.1 | DL095442.1 | HH734153.1 |
| HC046931.1 | FZ416711.1 | DD093884.1 | GM963555.1 | DL102593.1 | DL089088.1 | DL040848.1 | DL095410.1 | HH733712.1 |
| HC046899.1 | FZ419830.1 | DD093852.1 | GM889360.1 | DL102561.1 | DL089056.1 | DL040816.1 | DL095378.1 | HD119499.1 |
| HC046871.1 | FZ419796.1 | DD092959.1 | GM889316.1 | DL102529.1 | DL089024.1 | DL040784.1 | DL095346.1 | FW360288.1 |
| HC046839.1 | FZ419696.1 | DD092318.1 | GM009367.1 | AX418895.1 | DL088992.1 | DL036865.1 | DL091468.1 | FW365392.1 |
| HC046807.1 | FZ416530.1 | DD119263.1 | GM008853.1 | AX418393.1 | DL122351.1 | DL036833.1 | DL091436.1 | FW360183.1 |
| HC046775.1 | HQ233647.1 | DD118652.1 | GM887743.1 | AX418120.1 | DL122319.1 | DL036801.1 | DL091404.1 | FW359916.1 |
| HC046743.1 | FW582262.1 | DD091231.1 | GM007128.1 | AX404892.1 | DL117969.1 | DL036769.1 | DL091372.1 | FW359854.1 |
| HC046711.1 | FW591963.1 | BD320151.1 | DL124791.1 | AX398679.1 | DL117937.1 | DL036737.1 | DL087511.1 | FW359767.1 |
| HV504195.1 | FW591105.1 | BD325561.1 | DL120766.1 | AX392853.1 | DL117905.1 | DL036705.1 | DL108155.1 | FW359685.1 |
| HI930657.1 | FW590736.1 | BD314444.1 | DL120734.1 | AX391498.1 | DL117873.1 | DL032502.1 | DL108123.1 | FW359528.1 |
| HI930275.1 | FW588680.1 | BD313295.1 | DL116171.1 | AX382516.1 | DL117841.1 | DL032470.1 | DL108091.1 | FW368822.1 |
| HI935079.1 | FW587544.1 | BD312785.1 | DL116139.1 | AX379584.1 | DL113120.1 | DL032438.1 | DL103485.1 | FW368692.1 |
| HI918280.1 | HI642606.1 | BD319296.1 | DL116107.1 | AX376990.1 | DL113088.1 | DL032406.1 | DL103453.1 | FW368641.1 |
| HI657452.1 | HI642062.1 | BD318874.1 | DL116075.1 | AX375324.1 | DL113056.1 | DL032374.1 | DL103421.1 | FW368399.1 |

| | | | | | | | | |
|---|---|---|---|---|---|---|---|---|
| HI656474.1 | HI641518.1 | BD317368.1 | DL116043.1 | AX370728.1 | DL108255.1 | DL032342.1 | DL103389.1 | FW363799.1 |
| FW495947.1 | HI640974.1 | BD309110.1 | DL120567.1 | AX366989.1 | DL108223.1 | DL024157.1 | DL098815.1 | FW368154.1 |
| FW420422.1 | HI637815.1 | BD308153.1 | DL120535.1 | AX364696.1 | DL108191.1 | DL024125.1 | DL098783.1 | FW363540.1 |
| FW420398.1 | HI380413.1 | BD300453.1 | DL115908.1 | AX364539.1 | DL108159.1 | DL024093.1 | DL098751.1 | FW363471.1 |
| FW420365.1 | HI380332.1 | BD293454.1 | DL115876.1 | AX364507.1 | DL108127.1 | DL024061.1 | DL092709.1 | FW363211.1 |
| FW420333.1 | HI379465.1 | BD293414.1 | DL115844.1 | AX364475.1 | DL108095.1 | DL024029.1 | DL092677.1 | FW362973.1 |
| FW420048.1 | HI378744.1 | BD292830.1 | DL128466.1 | AX364443.1 | DL103489.1 | DL023997.1 | DL092645.1 | FW362882.1 |
| FW496543.1 | HI377630.1 | BD292447.1 | DL128433.1 | AX364411.1 | DL103457.1 | DL020790.1 | DL092613.1 | FW367138.1 |
| HH928931.1 | HI377251.1 | BD291385.1 | DL124675.1 | AX364379.1 | DL103425.1 | DL016387.1 | DL092581.1 | HC187664.1 |
| HH931929.1 | HI372274.1 | BD299784.1 | DL124643.1 | AX364347.1 | DL103393.1 | DL016355.1 | DL088966.1 | HC187078.1 |
| HH830441.1 | HI371012.1 | BD298688.1 | DL124611.1 | AX364220.1 | DL040939.1 | DL016323.1 | DL088934.1 | HC090622.1 |
| HH826941.1 | HI369903.1 | BD296985.1 | DL124579.1 | AX364187.1 | DL040907.1 | FW563125.1 | DL088902.1 | HC089561.1 |
| FW381485.1 | HI369125.1 | BD296887.1 | DL120396.1 | AX357305.1 | DL037084.1 | FW558553.1 | DL103277.1 | HC089529.1 |
| FW369195.1 | HI369082.1 | BD295242.1 | DL096914.1 | AX356675.1 | DL037052.1 | FW557315.1 | DL108029.1 | HC089497.1 |
| HH735635.1 | HI368439.1 | BD283717.1 | DL096882.1 | BD006815.1 | DL037020.1 | FW561675.1 | DL107965.1 | HC089465.1 |
| HC923272.1 | HI424142.1 | BD287345.1 | DL096850.1 | E54575.1 | DL028361.1 | FW557082.1 | DL107933.1 | HC089433.1 |
| HC922945.1 | HI424110.1 | GN088989.1 | DL128074.1 | E59174.1 | DL024547.1 | FW557050.1 | DL107901.1 | HC089400.1 |
| HC920504.1 | HI424078.1 | GN082352.1 | DL120282.1 | AX354704.1 | DL024515.1 | FW557017.1 | DL092443.1 | HC089368.1 |
| HD064837.1 | HI423852.1 | GN081995.1 | DL120250.1 | AX352801.1 | DL024483.1 | FW556983.1 | DL092411.1 | HC089336.1 |
| HD053215.1 | HI423820.1 | DL233063.1 | DL120218.1 | AX351104.1 | DL024451.1 | FW562772.1 | DL092379.1 | HC089303.1 |
| HD048996.1 | HI423564.1 | GN078434.1 | DL115815.1 | AX348527.1 | DL024419.1 | FW562715.1 | DL088604.1 | HC089271.1 |
| HD033940.1 | HI545497.1 | GN075950.1 | DL115783.1 | AX277251.1 | DL024387.1 | FW560092.1 | DL112950.1 | HC087854.1 |
| HD033690.1 | HI521560.1 | L08879.1 | DL115751.1 | AX347457.1 | DL021175.1 | FW560060.1 | DL098531.1 | HC086295.1 |
| HD057703.1 | HI521274.1 | DM059628.1 | DL115719.1 | AX347375.1 | DL021143.1 | FW562688.1 | DL092329.1 | HC085683.1 |
| HD057671.1 | HI539186.1 | DM058852.1 | DL115687.1 | AX347293.1 | DL021111.1 | FW562555.1 | DL092297.1 | HC085247.1 |
| HD062031.1 | HI583976.1 | DM058786.1 | DL115655.1 | AX347255.1 | DL021079.1 | FW559902.1 | DL092265.1 | AY774921.1 |
| U17001.1 | HI583943.1 | DM063072.1 | DL045667.1 | AX347223.1 | DL021047.1 | FW505141.1 | DL092201.1 | AY774871.1 |
| M60480.1 | HI573014.1 | DM057723.1 | DL041692.1 | AX347185.1 | DL021015.1 | FW508124.1 | DL088490.1 | AY774810.1 |
| M29019.1 | HI520630.1 | DM041706.1 | CS359598.1 | AX347127.1 | DL016809.1 | FW508066.1 | DL088458.1 | AY774753.1 |
| M54794.1 | HI520488.1 | DM056982.1 | BD269138.1 | AX345383.1 | DL016777.1 | FW553189.1 | DL088426.1 | AY774697.1 |
| HC917634.1 | HI207614.1 | DM045536.1 | BD268159.1 | AX344855.1 | DL016713.1 | FW553128.1 | DL117794.1 | AY774640.1 |
| FW337282.1 | HI003704.1 | DM045257.1 | BD268116.1 | AX344133.1 | DL016649.1 | FW563890.1 | DL117762.1 | AY774582.1 |
| FW340707.1 | HI002079.1 | DM044979.1 | BD266622.1 | AX342860.1 | DL016617.1 | FW559426.1 | DL122093.1 | AY774521.1 |
| HC874694.1 | HI002028.1 | DM039612.1 | BD265608.1 | AX342201.1 | DL012159.1 | FW559401.1 | DL125738.1 | AY774475.1 |
| HC873765.1 | HI001945.1 | DM039475.1 | BD263448.1 | AX339379.1 | DL012127.1 | FW559353.1 | DL125706.1 | AY774353.1 |
| HC881786.1 | HI000277.1 | DM044671.1 | BD261812.1 | AX339189.1 | DL012095.1 | FW507402.1 | DL048689.1 | AY774196.1 |
| HC449557.1 | HI000237.1 | DM060712.1 | BD251211.1 | AX039417.1 | DL012063.1 | FW506093.1 | DL048657.1 | AY774135.1 |
| FU759914.1 | HI000193.1 | DM060680.1 | BD249881.1 | AX039182.1 | DL012031.1 | HI933909.1 | DL045239.1 | AY774001.1 |
| FU758385.1 | HI000137.1 | GN067956.1 | BD249020.1 | AX037562.1 | DL011999.1 | HI936977.1 | DL045207.1 | HC084637.1 |
| FU757776.1 | HI205884.1 | GN067831.1 | BD247506.1 | AX036644.1 | DL016431.1 | HI936910.1 | DL045175.1 | HC070222.1 |
| HC358393.1 | HI471207.1 | GM712961.1 | BD246970.1 | AX035458.1 | DL011941.1 | HI932080.1 | DL045143.1 | HC070174.1 |
| HC358205.1 | HI180720.1 | GM652757.1 | BD245171.1 | AX034872.1 | DL011909.1 | HI930650.1 | DL045111.1 | HC070338.1 |
| HC358074.1 | HI538861.1 | GM652725.1 | BD244578.1 | AX033489.1 | DL011877.1 | HI936202.1 | DL045079.1 | HC083886.1 |
| HC325501.1 | HI470574.1 | GM652693.1 | DD231206.1 | AX030191.1 | DL011845.1 | FW362876.1 | DL037080.1 | HC083739.1 |
| HC325029.1 | HI544682.1 | GM652661.1 | DD229866.1 | AX028770.1 | DL011813.1 | FW362666.1 | DL037048.1 | HC083707.1 |
| HC324129.1 | HI538341.1 | GM652629.1 | DD227766.1 | AX027727.1 | DL024352.1 | FW366220.1 | DL037016.1 | HC083212.1 |
| HC314446.1 | HI559201.1 | GM652597.1 | DD227412.1 | AX027328.1 | DL024320.1 | FW351420.1 | DL028357.1 | HC081612.1 |

| | | | | | | | | |
|---|---|---|---|---|---|---|---|---|
| HC318831.1 | HI564713.1 | GM645503.1 | DD227377.1 | AX025617.1 | DL024288.1 | FW351308.1 | DL024543.1 | DM460862.1 |
| HC316589.1 | HI568933.1 | GM645471.1 | DD226583.1 | AX024949.1 | DL024256.1 | FW351251.1 | DL024511.1 | HC062179.1 |
| HC307886.1 | HI568859.1 | GM645439.1 | DD224652.1 | AX023658.1 | DL024192.1 | FW351190.1 | DL024479.1 | HC062009.1 |
| HC307811.1 | HI559130.1 | GM746437.1 | DD224163.1 | AX023626.1 | DL040876.1 | FW366105.1 | DL024447.1 | HC061930.1 |
| HC307390.1 | HI558652.1 | GM638610.1 | DD223777.1 | AX020978.1 | DL040844.1 | FW361514.1 | DL024415.1 | HC061851.1 |
| HB475479.1 | HI564211.1 | GM638578.1 | DD233561.1 | AX018594.1 | DL040812.1 | FW350348.1 | DL024383.1 | HC059851.1 |
| HB474894.1 | HI564099.1 | GM638546.1 | DD231560.1 | AX017671.1 | DL040780.1 | HD122555.1 | DL021171.1 | HC059241.1 |
| HB469627.1 | HI563699.1 | GM638514.1 | DD231404.1 | AX014755.1 | DL040748.1 | HD121707.1 | DL021139.1 | HC059102.1 |
| HB468756.1 | DL125776.1 | GM638450.1 | CS265966.1 | AX002761.1 | DL036861.1 | HD115945.1 | DL021107.1 | HC058659.1 |
| DM152590.1 | DL125748.1 | GM633751.1 | DD217643.1 | AX011484.1 | DL036829.1 | HD114848.1 | DL021075.1 | HC058558.1 |
| DM147633.1 | DL122534.1 | GM633719.1 | DD213997.1 | AX011452.1 | DL032370.1 | HD113250.1 | DL021043.1 | HC058385.1 |
| HB451956.1 | DL122502.1 | GM633687.1 | DD213840.1 | AX011420.1 | DL014615.1 | HD082446.1 | DL021011.1 | HC058109.1 |
| HB463483.1 | DL122438.1 | GM625820.1 | DD213808.1 | AX011387.1 | DL014583.1 | HD082377.1 | DL012187.1 | HC057819.1 |
| HB447895.1 | DL122406.1 | GM625788.1 | DD216483.1 | AX011355.1 | DL014551.1 | HD088557.1 | DL012155.1 | HC057202.1 |
| DM140521.1 | DL122374.1 | GM625756.1 | DD216441.1 | AX011275.1 | DL014519.1 | HD086808.1 | DL012123.1 | HC056008.1 |
| DM137797.1 | DL118120.1 | GM638441.1 | DD213759.1 | AX010959.1 | DL014487.1 | HD078945.1 | DL012091.1 | DM459939.1 |
| DM130825.1 | DL118088.1 | GM624700.1 | DD213695.1 | AX010830.1 | DL014455.1 | HD070773.1 | DL012059.1 | DM459757.1 |
| GN032380.1 | DL118056.1 | GM624668.1 | DD213663.1 | AX010629.1 | DL009997.1 | HD070417.1 | DL012027.1 | DM385968.1 |
| GN032347.1 | DL118024.1 | GM624636.1 | DD213631.1 | AX010383.1 | DL009965.1 | HD069969.1 | DL011995.1 | DM385934.1 |
| GN032315.1 | DL113335.1 | GM624604.1 | DD220789.1 | AX010114.1 | DL009933.1 | FW345019.1 | DL011873.1 | DM462137.1 |
| GN032283.1 | DL113303.1 | GM624572.1 | DD213581.1 | AX007117.1 | DL009901.1 | FW344004.1 | DL024284.1 | DM383026.1 |
| GN032251.1 | DL113271.1 | GM624540.1 | DD213549.1 | AX006471.1 | DL009869.1 | FW343692.1 | DL024252.1 | DM381869.1 |
| GN032219.1 | DL108470.1 | GM624507.1 | DD213494.1 | AX004775.1 | DL009837.1 | FW343626.1 | DL024220.1 | HC054888.1 |
| GN032187.1 | DL108438.1 | GM624475.1 | DD215919.1 | AX003191.1 | DL009805.1 | FW343562.1 | DL032462.1 | HC053902.1 |
| GN032155.1 | DL108356.1 | GM624443.1 | DD215887.1 | AX002974.1 | DL009773.1 | FW343498.1 | DL032366.1 | GN094586.1 |
| GN032123.1 | DL108342.1 | GM624411.1 | DD215855.1 | A18866.1 | DL009741.1 | FW343021.1 | DM475774.1 | GN094553.1 |
| GN032091.1 | DL108310.1 | GM624379.1 | DD215823.1 | A51657.1 | DL009709.1 | HC923236.1 | HC299781.1 | GN094521.1 |
| GN032059.1 | DL103672.1 | GM624347.1 | DD215759.1 | A35778.1 | DL009677.1 | HC921404.1 | HC306244.1 | GN094489.1 |
| GN032027.1 | DL103640.1 | GM633650.1 | DD217784.1 | A35715.1 | DL009645.1 | HC920489.1 | HB846885.1 | GN094457.1 |
| GN031995.1 | DL103608.1 | GM633618.1 | DD212268.1 | A29446.1 | DL009613.1 | HD067823.1 | HB838540.1 | GN113553.1 |
| GN031963.1 | DL099098.1 | GM633586.1 | DD212180.1 | A32837.1 | DL009581.1 | HD053203.1 | HB815201.1 | DM090558.1 |
| GN031931.1 | DL099066.1 | GM633554.1 | DD211844.1 | A32219.1 | DL009549.1 | HD052976.1 | HB826898.1 | DM065407.1 |
| GN031899.1 | DL099034.1 | GM633522.1 | DD211107.1 | A30343.1 | DL009517.1 | FU262466.1 | HB838049.1 | DM065003.1 |
| GN031867.1 | CS367700.1 | GM633490.1 | DD210757.1 | A23369.1 | DL009485.1 | FU253498.1 | HB827351.1 | DM064088.1 |
| GN031835.1 | DL080910.1 | GM625584.1 | DD200155.1 | A06238.1 | DL009453.1 | FU261572.1 | HB837096.1 | DM077660.1 |
| GN031802.1 | DL076099.1 | GM625552.1 | DD187511.1 | A28237.1 | DL030146.1 | FU269787.1 | HB845184.1 | DM070843.1 |
| GN031673.1 | DL080744.1 | GM638399.1 | DD206916.1 | A21003.1 | DL026273.1 | FU258432.1 | HB844884.1 | DM070639.1 |
| GN031641.1 | DL080448.1 | GM638367.1 | DD206884.1 | A21079.1 | DL026241.1 | FU258336.1 | HB844244.1 | DM070587.1 |
| GN031609.1 | DL075903.1 | GM624333.1 | DD206852.1 | A19555.1 | DL026209.1 | HC313144.1 | HB836525.1 | DM070479.1 |
| GN031577.1 | DL075871.1 | GM624301.1 | DD206790.1 | A14634.1 | DL026177.1 | HC311306.1 | HB843722.1 | DM069114.1 |
| GN031513.1 | DL075844.1 | GM624269.1 | AX657107.1 | A12290.1 | DL018987.1 | DM475257.1 | HB836259.1 | DM068822.1 |
| GN031481.1 | DL075812.1 | GM652544.1 | BD175963.1 | A10833.1 | DL018955.1 | DM466103.1 | HB843107.1 | GN087812.1 |
| GN031449.1 | DL075767.1 | GM652512.1 | AX643796.1 | DD213704.1 | DL018923.1 | DM465928.1 | HB835525.1 | GN091116.1 |
| GN031416.1 | DL074580.1 | GM652480.1 | AX642157.1 | DD213672.1 | DL018891.1 | HC299798.1 | HB834919.1 | GN087221.1 |
| GN031384.1 | DL074489.1 | GM652448.1 | BD171983.1 | DD213640.1 | DL018859.1 | HC306256.1 | HB842518.1 | GN089878.1 |
| GN031352.1 | DL079053.1 | GM652384.1 | AX601615.1 | DD213608.1 | DL018827.1 | HC306139.1 | HB850394.1 | GN089814.1 |
| GN031287.1 | DL078969.1 | GM659904.1 | AX601383.1 | DD213590.1 | DL034334.1 | HC306099.1 | HB841975.1 | GN082902.1 |

| | | | | | | | | |
|---|---|---|---|---|---|---|---|---|
| CS559292.1 | DL078937.1 | GM659872.1 | AX601351.1 | DD213558.1 | DL014238.1 | HC306059.1 | HB841723.1 | GN082831.1 |
| DD420108.1 | DL078906.1 | GM659840.1 | AX598969.1 | DD213526.1 | DL009386.1 | HC306019.1 | HB841381.1 | DL233320.1 |
| DD432989.1 | DL073472.1 | GM659808.1 | AX598865.1 | DD216038.1 | DL009290.1 | HC305979.1 | HB840822.1 | DL232695.1 |
| DD421578.1 | DL072286.1 | GM659776.1 | AX598813.1 | DD216006.1 | DL009258.1 | HC302626.1 | HB648684.1 | GN078629.1 |
| CS540029.1 | DL072229.1 | GM659744.1 | AX594174.1 | DD215974.1 | DL009226.1 | HC305369.1 | HB647052.1 | GN078809.1 |
| CS501979.1 | DL071902.1 | GM746209.1 | AX593516.1 | DD215928.1 | DL018684.1 | HC301464.1 | HB645743.1 | L09129.1 |
| CS498712.1 | DL013611.1 | GM659708.1 | AX587830.1 | DD215896.1 | DL018652.1 | HC301412.1 | HB645711.1 | L08782.1 |
| CS498441.1 | DL013579.1 | GM659676.1 | AX587737.1 | DD215864.1 | DL018620.1 | HC301317.1 | HB645677.1 | DM059408.1 |
| CS495833.1 | DL013547.1 | GM659644.1 | AX587638.1 | DD215832.1 | DL009072.1 | HC301028.1 | HB645530.1 | DM058731.1 |
| DD418594.1 | DL013515.1 | GM659612.1 | AX587534.1 | DD215800.1 | DL009040.1 | HC254832.1 | HB855099.1 | DM057731.1 |
| CS287605.1 | DL013483.1 | GM659580.1 | BD161916.1 | DD215768.1 | DL014224.1 | HC291486.1 | HB850752.1 | GM623731.1 |
| DD236706.1 | DL013451.1 | GM659547.1 | BD161068.1 | DD217989.1 | DL014160.1 | HC293978.1 | HB855987.1 | GM623699.1 |
| DD246857.1 | DL013419.1 | GM652355.1 | BD160735.1 | CS038866.1 | DL014128.1 | HC289355.1 | DM171091.1 | GM623667.1 |
| DD241173.1 | DL013387.1 | GM652323.1 | BD143213.1 | CS038825.1 | DL014096.1 | HC289323.1 | DM179148.1 | GM623635.1 |
| E41535.1 | DL018078.1 | GM652291.1 | AX587801.1 | CS029938.1 | DL014064.1 | HC289185.1 | DM170820.1 | GM623603.1 |
| E41694.1 | DL018046.1 | GM652259.1 | AX587696.1 | CS022577.1 | DL026154.1 | HC296856.1 | DM185748.1 | GM623571.1 |
| E41512.1 | DL018014.1 | GM645396.1 | AX587595.1 | CS021419.1 | DL026122.1 | HC295912.1 | DM188010.1 | GM658720.1 |
| E40770.1 | DL013263.1 | GM642970.1 | AX577532.1 | CS018282.1 | DL026090.1 | DL104024.1 | DM187721.1 | GM658688.1 |
| E46869.1 | DL013199.1 | GM642937.1 | AX556852.1 | CS017167.1 | DL026058.1 | DL104030.1 | HB489510.1 | GM658592.1 |
| E33637.1 | DL017988.1 | GM642905.1 | AX556818.1 | CS016586.1 | DL026026.1 | DL103974.1 | HB488758.1 | GM651368.1 |
| E05893.1 | DL017956.1 | GM642873.1 | AX555915.1 | CS016554.1 | DL025994.1 | DL103942.1 | DM165685.1 | GM651336.1 |
| E05531.1 | DL017924.1 | GM642841.1 | AX555187.1 | CS016474.1 | DL025962.1 | DL103910.1 | GM715851.1 | GM651304.1 |
| E05021.1 | DL017892.1 | DL012741.1 | AX554276.1 | CS016118.1 | DL014009.1 | DL099496.1 | DL479998.1 | GM651272.1 |
| E04540.1 | DL017828.1 | DL042674.1 | KF664574.1 | CQ990440.1 | CS602499.1 | DL099464.1 | DL465631.1 | GM651240.1 |
| E03830.1 | DL017797.1 | DL038793.1 | AH003158.2 | CQ986636.1 | CS602467.1 | DL099432.1 | DL464719.1 | GM651208.1 |
| E03567.1 | DL017765.1 | DL038761.1 | DQ250237.1 | CQ986602.1 | CS611202.1 | DL024731.1 | DL476966.1 | GM644409.1 |
| E02958.1 | DL017733.1 | DL038729.1 | DQ250205.1 | CQ986570.1 | CS611070.1 | DL024699.1 | DL463070.1 | GM644377.1 |
| E02691.1 | DL017701.1 | DL038697.1 | AF427148.1 | CQ986536.1 | CS607964.1 | DL021359.1 | DL476421.1 | GM644345.1 |
| E02539.1 | DL017669.1 | DL035073.1 | AF105011.1 | CQ986504.1 | CS607922.1 | DL021327.1 | DL462976.1 | GM644313.1 |
| E02265.1 | DL017637.1 | DL035041.1 | U01670.1 | CQ983156.1 | CS607535.1 | DL016993.1 | DL469767.1 | GM644281.1 |
| E01699.1 | DL022275.1 | DL034897.1 | AF202544.1 | CQ983110.1 | CS600260.1 | DL016961.1 | DL475568.1 | GM644249.1 |
| E01389.1 | DL022243.1 | DL034834.1 | KJ608143.1 | CQ983018.1 | CS607091.1 | DL016929.1 | DL481625.1 | GM644217.1 |
| E00911.1 | DL022211.1 | DL030726.1 | HW391106.1 | CQ982977.1 | CS607007.1 | DL016897.1 | DL460976.1 | GM637219.1 |
| E00257.1 | DL025726.1 | DL030694.1 | HW408412.1 | CQ982839.1 | CS606922.1 | DL016865.1 | GM712165.1 | GM637187.1 |
| DD146209.1 | DL025694.1 | DL030534.1 | HD062421.1 | CQ982771.1 | CS606801.1 | DL016833.1 | GM711981.1 | GM637155.1 |
| DD100895.1 | DL025566.1 | DL026725.1 | HD061090.1 | CQ980536.1 | CS605115.1 | DL012343.1 | GM827644.1 | GM637123.1 |
| DD112415.1 | DL029708.1 | DL026693.1 | HD055945.1 | CQ973140.1 | CS605083.1 | DL012311.1 | GM674988.1 | GM637091.1 |
| DD142883.1 | DL029676.1 | DL026661.1 | M23650.1 | CQ972557.1 | CS599812.1 | DL012279.1 | FB509210.1 | GM637059.1 |
| CQ972442.1 | DL029644.1 | DL026629.1 | M36450.1 | CQ977471.1 | CS605024.1 | DL012215.1 | FB509178.1 | GM624906.1 |
| CQ972378.1 | DL029612.1 | DL026597.1 | M81125.1 | CQ976904.1 | CS604992.1 | DL048696.1 | FB509144.1 | DL219992.1 |
| CQ972314.1 | DL029580.1 | DL026565.1 | HC917445.1 | CQ976040.1 | CS604960.1 | DL048664.1 | GM827444.1 | DL219911.1 |
| CQ970238.1 | DL025539.1 | DL022753.1 | FW334594.1 | CQ975975.1 | CS604928.1 | DL045246.1 | GM772970.1 | DL219682.1 |
| CQ947137.1 | DL025507.1 | DL022721.1 | FW341968.1 | CQ972337.1 | CS604864.1 | DL045214.1 | GM709011.1 | DL219596.1 |
| CQ944175.1 | DL025475.1 | DL022689.1 | FW337162.1 | CQ972305.1 | CS604832.1 | DL045182.1 | GM708979.1 | DL219510.1 |
| CQ944143.1 | DL025443.1 | DL022657.1 | FW341148.1 | CQ971138.1 | CS604800.1 | DL045150.1 | GM655008.1 | DL206311.1 |
| CQ944111.1 | DL025411.1 | DL022625.1 | HC868184.1 | CQ770992.1 | CS604768.1 | DL045118.1 | GM654976.1 | DL220826.1 |
| CQ944079.1 | DL025379.1 | DL022593.1 | HC887860.1 | CQ768383.1 | CS604736.1 | DL045086.1 | GM044433.1 | DL206149.1 |

| | | | | | | | | |
|---|---|---|---|---|---|---|---|---|
| CQ944047.1 | DL011942.1 | DL019578.1 | HC887341.1 | CQ767060.1 | CS604704.1 | DL040974.1 | GM642963.1 | FB715006.1 |
| CQ944015.1 | DL011910.1 | DL019546.1 | HC880422.1 | CQ766069.1 | CS604672.1 | DL040942.1 | GM642931.1 | FB714192.1 |
| CQ943983.1 | DL011878.1 | DL019514.1 | HC877781.1 | CQ765862.1 | CS604638.1 | DL040910.1 | GM630655.1 | FB713965.1 |
| CQ943951.1 | DL011846.1 | DL019482.1 | HC870102.1 | CQ759573.1 | CS604606.1 | DL037087.1 | GM630623.1 | DL120263.1 |
| CQ943919.1 | DL011814.1 | DL019450.1 | HC869695.1 | CQ758825.1 | CS604574.1 | DL037055.1 | GM630591.1 | DL120231.1 |
| CQ943887.1 | DL024353.1 | DL014983.1 | HC050265.1 | CQ756678.1 | CS597518.1 | DL037023.1 | GM621656.1 | DL120199.1 |
| CQ943855.1 | DL024321.1 | DL014951.1 | HC045496.1 | CQ755002.1 | CS593089.1 | DL036991.1 | GM621624.1 | DL115796.1 |
| AY822465.1 | DL024289.1 | DL014919.1 | HC045464.1 | CQ754043.1 | CS599604.1 | DL036959.1 | GM621592.1 | DL115764.1 |
| CQ898629.1 | DL024257.1 | DL014887.1 | HC045432.1 | CQ753253.1 | CS589275.1 | DL036927.1 | GM621560.1 | DL115700.1 |
| CQ898597.1 | DL024225.1 | DL010269.1 | HC045400.1 | CQ654944.1 | GM638449.1 | DL032692.1 | GM657140.1 | DL115668.1 |
| CQ898565.1 | DL024193.1 | DL010237.1 | HC045368.1 | CQ654814.1 | GM633750.1 | DL032660.1 | GM657076.1 | DL115636.1 |
| CQ891377.1 | DL045053.1 | DL010205.1 | HC045336.1 | CQ654380.1 | GM633718.1 | DL028524.1 | GM657044.1 | DL111242.1 |
| CQ877372.1 | DL045021.1 | DL034670.1 | HC045272.1 | CQ654198.1 | GM633686.1 | DL028492.1 | GM657012.1 | DL111210.1 |
| CQ875489.1 | DL044989.1 | DL034638.1 | HC044077.1 | CQ654013.1 | GM625819.1 | DL028460.1 | GM656980.1 | DL111178.1 |
| CQ873315.1 | HW311253.1 | DL030386.1 | HC047452.1 | AX963146.1 | GM625787.1 | DL028428.1 | GM656948.1 | DL111146.1 |
| CQ871398.1 | HW311156.1 | DL030346.1 | HC047420.1 | AX962039.1 | GM625755.1 | DL028396.1 | GM656917.1 | DL111114.1 |
| CQ869334.1 | HW307873.1 | DL026529.1 | HC047388.1 | AX960832.1 | GM633672.1 | DL028364.1 | GM656853.1 | DL111082.1 |
| CQ867313.1 | HW307841.1 | DL026473.1 | HC047356.1 | AX960373.1 | GM624699.1 | DL024550.1 | GM656821.1 | DL106278.1 |
| AX300165.1 | HW307809.1 | DL026441.1 | HC047324.1 | AX960341.1 | GM624667.1 | DL024518.1 | GM656789.1 | DL106246.1 |
| AX284146.1 | HW307777.1 | DL026409.1 | HC047292.1 | AX958649.1 | GM624635.1 | DL024486.1 | GM656757.1 | DL106214.1 |
| AF411475.1 | HW316589.1 | DL026377.1 | HC047260.1 | AX957773.1 | GM624603.1 | DL024454.1 | GM642700.1 | DL106182.1 |
| AX279806.1 | HW307739.1 | DL022565.1 | HC047036.1 | AX958018.1 | GM624571.1 | DL024390.1 | GM642668.1 | DL106150.1 |
| AX256440.1 | HW307707.1 | DL022533.1 | HC047004.1 | AX956812.1 | GM624539.1 | DL021178.1 | GM642636.1 | DL106118.1 |
| AX254831.1 | HW315785.1 | DL022493.1 | HC046972.1 | AX952270.1 | GM631818.1 | DL021146.1 | GM630488.1 | DL101505.1 |
| AX242245.1 | HW315627.1 | DL022437.1 | HC046940.1 | AX938903.1 | GM622855.1 | DL021114.1 | GM621521.1 | DL101473.1 |
| AX242181.1 | HW315567.1 | DL022405.1 | HC046908.1 | AX937256.1 | GM622823.1 | DL021082.1 | GM699200.1 | DL101441.1 |
| AX242149.1 | HW315533.1 | DL014652.1 | HC046880.1 | AX934479.1 | GM704287.1 | DL021050.1 | GM649462.1 | DL101409.1 |
| AX242117.1 | HW315142.1 | DL010177.1 | HC046848.1 | AX925783.1 | GM657908.1 | DL021018.1 | GM649430.1 | DL101377.1 |
| AX242021.1 | HW314956.1 | DL010145.1 | HC046816.1 | AX924036.1 | GM657876.1 | DL016812.1 | GM626819.1 | DL096739.1 |
| AX241893.1 | HW314908.1 | DL010113.1 | HC046784.1 | AX923411.1 | GM657844.1 | DL016780.1 | GM653634.1 | DL111039.1 |
| AX241861.1 | HW314846.1 | DL010081.1 | HC046752.1 | AX923378.1 | GM657779.1 | DL016748.1 | GM653602.1 | DL111007.1 |
| AX241829.1 | HW314629.1 | DL010049.1 | HC046720.1 | AX840796.1 | GM657747.1 | DL016716.1 | BD225781.1 | DL110975.1 |
| AX241701.1 | HW151145.1 | DJ357590.1 | HC039694.1 | AX838977.1 | GM643388.1 | DL016684.1 | BD225749.1 | DL110943.1 |
| AX241637.1 | HW112619.1 | DJ363056.1 | HC046639.1 | AX824452.1 | GM643356.1 | DL016652.1 | BD225717.1 | DL110911.1 |
| AX241605.1 | HW072744.1 | DJ350051.1 | HC046607.1 | AX824342.1 | GM643324.1 | DL016620.1 | BD225685.1 | DL110879.1 |
| AX241541.1 | HW069990.1 | DJ355100.1 | HC046575.1 | AX823776.1 | GM643260.1 | DL012162.1 | BD225653.1 | DL106075.1 |
| AX241413.1 | HW069958.1 | DJ354936.1 | HC046543.1 | AX818084.1 | GM643228.1 | DL012130.1 | BD225621.1 | DL106043.1 |
| AY044145.1 | HW069886.1 | DJ361246.1 | HC046511.1 | AX816388.1 | GM636239.1 | DL012098.1 | BD224827.1 | DL106011.1 |
| AX234655.1 | HW069794.1 | DJ361212.1 | HC046479.1 | AX816105.1 | GM636207.1 | DL012066.1 | BD221599.1 | DL105979.1 |
| AX225298.1 | HW084950.1 | DJ354228.1 | HC046415.1 | AX814475.1 | GM636175.1 | DL012034.1 | BD205386.1 | DL105947.1 |
| AX207309.1 | HW097744.1 | DJ339859.1 | HC046383.1 | AX809455.1 | GM636143.1 | DL016562.1 | DD282927.1 | DL105915.1 |
| AX207113.1 | HW067068.1 | DJ339827.1 | HC046351.1 | AX805942.1 | GM636111.1 | DL016530.1 | DD281069.1 | DL101302.1 |
| AX202581.1 | HW065059.1 | DJ339763.1 | HC046319.1 | AX800007.1 | GM636079.1 | DL016498.1 | DD279440.1 | DL101270.1 |
| AX202438.1 | HW057917.1 | DJ339731.1 | HC046068.1 | HW386275.1 | GM631671.1 | DL016466.1 | DD278997.1 | DL101238.1 |
| AX193974.1 | HW060593.1 | DJ339699.1 | HC046036.1 | HW386243.1 | GM631639.1 | DL016434.1 | CS355965.1 | DL101206.1 |
| AX180311.1 | HW047326.1 | DJ339667.1 | HC045998.1 | HW386115.1 | GM631607.1 | DL011912.1 | CS350541.1 | DL101174.1 |
| AX172952.1 | HV961627.1 | DJ339570.1 | HC045966.1 | HW385923.1 | GM631575.1 | DL011880.1 | CS353182.1 | DL101142.1 |

| | | | | | | | | |
|---|---|---|---|---|---|---|---|---|
| AX151023.1 | HV969753.1 | DJ339082.1 | HC045934.1 | HW380897.1 | GM631543.1 | DL011848.1 | CS352972.1 | DL096728.1 |
| HW350883.1 | HV956156.1 | DJ336589.1 | HC045870.1 | CS118760.1 | GM631511.1 | DL011816.1 | CS352411.1 | DL096696.1 |
| HW340459.1 | HV951083.1 | DJ333881.1 | HC045838.1 | CS118727.1 | GM622740.1 | DL024355.1 | CS283932.1 | DL096664.1 |
| HW350660.1 | HV957528.1 | DJ328774.1 | HC045806.1 | CS118695.1 | GM622708.1 | DL013728.1 | CS279309.1 | DL096632.1 |
| HW339862.1 | HV957459.1 | DJ327053.1 | HC045774.1 | CS118663.1 | GM622676.1 | DL013664.1 | CS277021.1 | DL096600.1 |
| HW339575.1 | HV944654.1 | DJ327021.1 | HC045742.1 | CS118631.1 | GM704176.1 | DJ490773.1 | CS273348.1 | DL096568.1 |
| HW348296.1 | FW562520.1 | DJ326604.1 | HC045710.1 | CS118565.1 | GM650374.1 | DL000685.1 | CS254954.1 | DL094740.1 |
| HW363659.1 | FW566807.1 | DJ129309.1 | HC045680.1 | CS118531.1 | GM622452.1 | DL000547.1 | CS021531.1 | DL094708.1 |
| HW363628.1 | FW505132.1 | DJ122502.1 | DM378905.1 | CS118465.1 | GM622420.1 | DL005236.1 | CS019412.1 | DL094676.1 |
| HW355322.1 | FW508121.1 | DJ122006.1 | HC037096.1 | CS118433.1 | GM622388.1 | DJ493831.1 | CS018332.1 | DL094644.1 |
| HW244293.1 | FW562402.1 | DJ082697.1 | HC022620.1 | CS118400.1 | GM622356.1 | DL008510.1 | CS016579.1 | DL094612.1 |
| HW248653.1 | FW555409.1 | DJ082576.1 | HC026844.1 | CS118333.1 | GM703822.1 | DL003040.1 | CS016541.1 | DL094580.1 |
| HW242298.1 | FW553186.1 | DJ081984.1 | HC025570.1 | CS118299.1 | GM657510.1 | DL002941.1 | CS014562.1 | DL094548.1 |
| HW241800.1 | FW553125.1 | DJ081577.1 | GM659126.1 | CS118267.1 | GM657478.1 | DJ491565.1 | CQ990432.1 | DL090933.1 |
| HW241756.1 | FW566101.1 | DJ078395.1 | GM658966.1 | CS118235.1 | GM657446.1 | DL002531.1 | CQ986595.1 | DL090901.1 |
| HW241559.1 | FW559431.1 | DJ080274.1 | GM651774.1 | CS118136.1 | GM657414.1 | DJ447627.1 | CQ986563.1 | DL086965.1 |
| HW241375.1 | FW559396.1 | DJ081431.1 | GM644782.1 | CS118071.1 | GM657382.1 | DJ446832.1 | CQ983184.1 | DL086933.1 |
| HW161272.1 | FW559348.1 | DJ071518.1 | GM644750.1 | CS118038.1 | GM650158.1 | DJ446690.1 | CQ983100.1 | DL086901.1 |
| HW155697.1 | FW506883.1 | DJ066782.1 | GM644718.1 | CS117969.1 | GM650126.1 | DJ474320.1 | CQ983067.1 | DL086869.1 |
| HW144844.1 | FW510492.1 | DJ066386.1 | GM633056.1 | CS115032.1 | GM650094.1 | DJ437360.1 | CQ982901.1 | DL086837.1 |
| HW155429.1 | FW510230.1 | DJ066354.1 | GM625189.1 | CS114661.1 | GM643125.1 | DJ437327.1 | CQ982823.1 | DL086773.1 |
| HW155295.1 | FW506246.1 | DJ066322.1 | GM625157.1 | CS114317.1 | GM643093.1 | DJ437246.1 | CQ982742.1 | DL086741.1 |
| HW155211.1 | FW509464.1 | DJ066288.1 | GM624035.1 | AX143069.1 | GM643061.1 | DJ437181.1 | CQ982677.1 | DL086709.1 |
| HW154656.1 | HI933790.1 | DJ066254.1 | GM624003.1 | AX143005.1 | GM643029.1 | DL015733.1 | CQ982592.1 | DL086677.1 |
| HV929586.1 | HI936430.1 | DJ068022.1 | GM623971.1 | AX142749.1 | GM636008.1 | DL011179.1 | CQ981122.1 | DL086645.1 |
| HV802956.1 | HI930647.1 | DJ065233.1 | GM623939.1 | AX142621.1 | GM635976.1 | DL047255.1 | CQ975460.1 | DL086613.1 |
| HV802924.1 | HI930267.1 | DJ067020.1 | GM625049.1 | AX142557.1 | GM635944.1 | DL047223.1 | CQ975362.1 | DL105886.1 |
| HV802892.1 | HI949805.1 | DJ061598.1 | GM625017.1 | AX142427.1 | GM635912.1 | DL047191.1 | CQ975330.1 | DL105854.1 |
| HV817777.1 | HI929253.1 | GN362481.1 | GM827614.1 | AX142363.1 | GM635880.1 | DL047159.1 | CQ973482.1 | DL105790.1 |
| HV778347.1 | HI918270.1 | GN346512.1 | GM658914.1 | AX142299.1 | GM648510.1 | DL047127.1 | CQ979899.1 | DL105758.1 |
| HV777436.1 | HI917018.1 | GN346478.1 | GM658882.1 | AX142171.1 | GM648478.1 | DL047095.1 | CQ972490.1 | DL105726.1 |
| HV763965.1 | HI916903.1 | GN344147.1 | GM658850.1 | AX141783.1 | GM648446.1 | DL047063.1 | CQ972394.1 | DL101017.1 |
| HV766821.1 | HI923664.1 | GN346898.1 | GM658818.1 | AX141719.1 | GM648414.1 | DL043241.1 | CQ972362.1 | DL100985.1 |
| K02756.1 | HI657792.1 | GN346585.1 | GM637413.1 | AX141463.1 | GM641611.1 | DL043209.1 | CQ972330.1 | DL096539.1 |
| M62934.1 | HI657212.1 | GN346553.1 | GM637381.1 | AX141399.1 | GM634263.1 | DL043177.1 | CQ971799.1 | DL096507.1 |
| M11828.1 | FW421367.1 | GN340207.1 | GM637349.1 | AX139918.1 | GM629266.1 | DL043145.1 | CQ971124.1 | DL096443.1 |
| M14401.1 | FW498194.1 | GN339922.1 | GM637317.1 | AX138857.1 | GM629234.1 | DL043113.1 | CQ971028.1 | DL096379.1 |
| HV549332.1 | FW503022.1 | GN339091.1 | GM637285.1 | AX134858.1 | GM629202.1 | DL043081.1 | CQ970042.1 | DL096347.1 |
| HV549098.1 | FW496126.1 | GN130956.1 | GM637253.1 | AX127585.1 | GM629138.1 | DL039264.1 | CQ967275.1 | DL094519.1 |
| HV544369.1 | FW497432.1 | GN334500.1 | GM623652.1 | AX119975.1 | GM629106.1 | DL039232.1 | CQ947121.1 | DL094487.1 |
| HV544173.1 | FW504223.1 | GN333926.1 | CS603258.1 | AX114305.1 | GM634068.1 | DL039200.1 | CQ945510.1 | DL094455.1 |
| HV543460.1 | FW503859.1 | GN116532.1 | CS603194.1 | AX113783.1 | GM634036.1 | DL039168.1 | CQ944191.1 | DL094423.1 |
| HV538620.1 | FW499105.1 | GN116500.1 | CS603162.1 | AX113553.1 | GM634004.1 | DL039104.1 | CQ944159.1 | DL093168.1 |
| HV538464.1 | FW504981.1 | GN116467.1 | CS603130.1 | AX111914.1 | GM633940.1 | DL035480.1 | CQ944127.1 | DL089553.1 |
| HV543192.1 | FW504939.1 | GN116435.1 | CS603098.1 | AX108617.1 | GM633908.1 | DL035448.1 | CQ944095.1 | DL089521.1 |
| HV542550.1 | HD082721.1 | GN116403.1 | CS603066.1 | AX108280.1 | GM633876.1 | DL035416.1 | CQ944063.1 | DL042807.1 |
| HV541505.1 | HD082439.1 | GN116339.1 | CS603034.1 | AX103645.1 | GM655517.1 | DL035384.1 | CQ944031.1 | DL042775.1 |

| | | | | | | | | |
|---|---|---|---|---|---|---|---|---|
| HV532985.1 | HD082303.1 | GN116307.1 | CS603002.1 | AX100368.1 | GM655485.1 | DL035352.1 | CQ943999.1 | DL042743.1 |
| HV534662.1 | HD086705.1 | GN095157.1 | CS602968.1 | AX097521.1 | GM655453.1 | DL035320.1 | CQ943967.1 | DL042711.1 |
| HV516608.1 | HD070446.1 | GN094514.1 | CS602936.1 | AX097487.1 | GM648364.1 | DL031316.1 | CQ943935.1 | DL038862.1 |
| HV515786.1 | FW345126.1 | GN094482.1 | CS602904.1 | AX093145.1 | GM648332.1 | DL031284.1 | CQ943903.1 | DL038830.1 |
| HV515595.1 | FW344984.1 | GN094450.1 | CS602808.1 | AX092882.1 | GM648300.1 | DL031252.1 | CQ943871.1 | DL038798.1 |
| HV515499.1 | FW344193.1 | DM074533.1 | CS611327.1 | AX088802.1 | GM648268.1 | DL031220.1 | AX241717.1 | DL038766.1 |
| HV515467.1 | FW344001.1 | DM078969.1 | BD453342.1 | AX088750.1 | GM648236.1 | DL031188.1 | AX241685.1 | DL038734.1 |
| HV515435.1 | FW343684.1 | DM063717.1 | BD453313.1 | AX088694.1 | GM648204.1 | DL027347.1 | AX241653.1 | DL038702.1 |
| HV515403.1 | FW343620.1 | GM712959.1 | BD453281.1 | AX082992.1 | GM641401.1 | DL027315.1 | AX241621.1 | DL035078.1 |
| HV515371.1 | FW343432.1 | GM652755.1 | BD450460.1 | AX080919.1 | GM641369.1 | DL027283.1 | AX241589.1 | DL035046.1 |
| HV515339.1 | FW342675.1 | GM652723.1 | BD413564.1 | AX080768.1 | GM641337.1 | DL027251.1 | AX241557.1 | DL035014.1 |
| HV515307.1 | FW342977.1 | GM652691.1 | BD388832.1 | AX080467.1 | GM641305.1 | DL027219.1 | AX241525.1 | DL034982.1 |
| HV515275.1 | HC923155.1 | GM652659.1 | BD350056.1 | AX079168.1 | GM641273.1 | DL027187.1 | AX241493.1 | DL034950.1 |
| HV515243.1 | HC921636.1 | GM652627.1 | BD356390.1 | AX077287.1 | GM641241.1 | DL027155.1 | AX241429.1 | DL034918.1 |
| HV511018.1 | HC920706.1 | GM652595.1 | BD353554.1 | AX073924.1 | GM629050.1 | DL023343.1 | AX241122.1 | DL010402.1 |
| HV513411.1 | HD068609.1 | GM645501.1 | BD359749.1 | AJ286126.1 | HI002148.1 | DL023311.1 | AX241090.1 | DL034863.1 |
| HH928907.1 | HD067820.1 | GM645469.1 | BD359027.1 | AX068360.1 | HI002110.1 | DL023279.1 | AX241058.1 | DL034839.1 |
| HH931925.1 | HD067642.1 | GM645437.1 | BD342958.1 | AX060077.1 | HI000455.1 | DL023247.1 | AX241026.1 | DL034799.1 |
| HH826939.1 | HD066661.1 | GM638608.1 | BD342456.1 | AX058551.1 | HI000407.1 | DL023215.1 | AX240994.1 | DL034767.1 |
| FW377983.1 | HD064617.1 | GM638576.1 | BD341815.1 | AX058561.1 | HI000365.1 | DL023183.1 | AX240962.1 | DL034735.1 |
| FW371940.1 | HD063565.1 | GM638544.1 | BD349691.1 | AX058362.1 | HI201813.1 | DL020168.1 | AX240930.1 | DL034703.1 |
| FW369985.1 | HD053123.1 | GM638512.1 | BD349458.1 | AX057300.1 | HI553293.1 | DL020136.1 | AX235766.1 | DL030699.1 |
| FW375269.1 | HD052885.1 | GM638448.1 | BD325721.1 | AX056666.1 | HI553189.1 | DL020008.1 | AX224367.1 | DL030539.1 |
| AF025672.2 | HD051872.1 | GM633749.1 | BD314359.1 | HV767878.1 | HI539619.1 | DL015445.1 | AX207892.1 | DL026730.1 |
| FW343636.1 | HD048865.1 | GM633717.1 | BD313677.1 | HV767456.1 | HI586920.1 | DL010923.1 | AX207285.1 | DL026698.1 |
| HC923253.1 | HC920325.1 | GM633685.1 | BD319283.1 | HV766940.1 | HI539183.1 | DL010891.1 | AX205121.1 | DL026666.1 |
| HC922899.1 | HD033852.1 | GM625818.1 | BD319403.1 | HV766805.1 | HI583973.1 | DL010827.1 | AX202415.1 | DL026634.1 |
| HC920499.1 | HD033446.1 | GM625786.1 | BD318905.1 | HV766725.1 | HI583877.1 | DL023116.1 | AX196247.1 | DL026602.1 |
| HD068797.1 | HD057693.1 | GM625754.1 | BD318126.1 | HV760616.1 | HI207672.1 | DL023084.1 | AF401224.1 | DL026570.1 |
| HD067662.1 | HD057661.1 | GM624666.1 | BD308663.1 | HV766523.1 | HI002075.1 | DL023052.1 | AX188576.1 | DL022758.1 |
| HD033688.1 | HD057647.1 | GM624634.1 | BD306492.1 | HV766092.1 | HI002025.1 | DL023020.1 | AX166846.1 | DL022726.1 |
| HD057701.1 | HD061467.1 | GM633648.1 | BD300352.1 | HV766044.1 | HI001942.1 | DL022988.1 | AX172936.1 | DL022694.1 |
| HD057669.1 | M20975.1 | GM633616.1 | BD291414.1 | HV766012.1 | HI000274.1 | DJ030008.1 | AX168184.1 | DL022662.1 |
| HD062029.1 | M84650.1 | GM633584.1 | BD291308.1 | HV752849.1 | HI000234.1 | DJ029933.1 | AX167250.1 | DL022630.1 |
| L36849.1 | HC918579.1 | GM633552.1 | BD299996.1 | HV757522.1 | HI000190.1 | DJ026767.1 | AX166295.1 | DL022598.1 |
| J03721.1 | FW334407.1 | GM633520.1 | BD299635.1 | HV750066.1 | HI000134.1 | CS691575.1 | AX155098.1 | DL019583.1 |
| M12913.1 | FW342474.1 | GM633488.1 | BD295082.1 | HV758717.1 | HI205050.1 | CS686854.1 | AX148817.1 | DL019551.1 |
| M83832.1 | FW341708.1 | GM625582.1 | BD279843.1 | HV694681.1 | HI538857.1 | CS694079.1 | BD349499.1 | DL019519.1 |
| AY326434.1 | FW341581.1 | GM638397.1 | BD276258.1 | HV694432.1 | FW337538.1 | CS693263.1 | BD325623.1 | DL019487.1 |
| FW342480.1 | AY774297.1 | GM638365.1 | BD274303.1 | HV700345.1 | FW341579.1 | CS674201.1 | BD325530.1 | DL019455.1 |
| FW337275.1 | AY774187.1 | GM624331.1 | BD273659.1 | HV700221.1 | FW341206.1 | CS680829.1 | BD324948.1 | DL019423.1 |
| FW340705.1 | AY774126.1 | GM624299.1 | BD273236.1 | HV693625.1 | HC887349.1 | DJ008407.1 | BD314370.1 | DL015020.1 |
| HC874692.1 | AY774055.1 | GM624267.1 | AX657137.1 | HV693205.1 | HC867552.1 | DJ008375.1 | BD313207.1 | DL014988.1 |
| HC873724.1 | AY773993.1 | GM652542.1 | AX657105.1 | HV699084.1 | HC873722.1 | DJ011714.1 | BD319289.1 | DL014956.1 |
| HC880626.1 | U26456.1 | GM652510.1 | AX657061.1 | HV698617.1 | HC872739.1 | DJ008089.1 | BD319463.1 | DL014924.1 |
| HB865032.1 | AF311743.1 | GM652478.1 | BD176072.1 | HV698533.1 | HC880549.1 | DD494965.1 | BD318828.1 | DL014892.1 |
| HB864972.1 | AF223929.1 | GM652446.1 | AX644850.1 | HV690126.1 | HC879857.1 | CS669142.1 | BD308700.1 | DL014860.1 |

| | | | | | | | | |
|---|---|---|---|---|---|---|---|---|
| HB864992.1 | HC084627.1 | GM652414.1 | AX643792.1 | HV688800.1 | HC889118.1 | CS672575.1 | CQ868901.1 | DL014828.1 |
| HB864936.1 | HC070333.1 | GM659902.1 | AX643062.1 | HV600534.1 | HC870913.1 | DD462669.1 | CQ868493.1 | DL010370.1 |
| HB864904.1 | HC083876.1 | GM659870.1 | AX642153.1 | HV579912.1 | HC869870.1 | CS646654.1 | CQ867550.1 | DL010338.1 |
| HB864872.1 | HC083734.1 | GM659838.1 | BD171981.1 | HV579751.1 | HC869575.1 | CS646201.1 | CQ866704.1 | DL010306.1 |
| HB864840.1 | HC083525.1 | GM659806.1 | BD171522.1 | HV585127.1 | HC869543.1 | DD460155.1 | CQ859609.1 | DL010274.1 |
| HB864808.1 | HC069947.1 | GM659774.1 | AX614793.1 | HV585095.1 | HC876930.1 | DD459942.1 | CQ855923.1 | DL010210.1 |
| HB864776.1 | HC081829.1 | GM659742.1 | AX601797.1 | HV584863.1 | HC876892.1 | DD455160.1 | CQ849476.1 | DL034675.1 |
| HB864744.1 | DM460344.1 | GM746201.1 | AX601613.1 | GN359817.1 | HC876942.1 | DD458987.1 | CQ840188.1 | DL034643.1 |
| HB866527.1 | HC061999.1 | GM712092.1 | AX601381.1 | GN359753.1 | HC868465.1 | DD458752.1 | CQ834127.1 | DL034611.1 |
| HB976624.1 | HC061920.1 | GM659706.1 | AX601349.1 | GN359721.1 | FW304551.1 | DD453827.1 | CQ828074.1 | DL034579.1 |
| DM193662.1 | HC061371.1 | GM659674.1 | AX598917.1 | GN359669.1 | FW309613.1 | DD457859.1 | CQ827547.1 | DL034547.1 |
| DM192908.1 | HC057889.1 | GM659642.1 | AX587923.1 | GN359605.1 | FW333963.1 | DD456894.1 | CQ824419.1 | DL034515.1 |
| CS119064.1 | DM460306.1 | GM659610.1 | AX587730.1 | GN359573.1 | DL120477.1 | DD456417.1 | CQ819077.1 | DL030487.1 |
| CS119031.1 | DM459746.1 | GM659578.1 | AX587633.1 | GN359509.1 | DL116010.1 | CS631730.1 | CQ818560.1 | DL030447.1 |
| CS118998.1 | DM385651.1 | GM659545.1 | AX587676.1 | GN359409.1 | DL115914.1 | CS642303.1 | CQ817010.1 | DL030415.1 |
| CS118966.1 | DM381855.1 | GM652353.1 | AX592716.1 | GN359377.1 | DL115882.1 | CS631222.1 | CQ816969.1 | CS798101.1 |
| CS118933.1 | HC054878.1 | GM634596.1 | BD144978.1 | GN359345.1 | DL115850.1 | CS627768.1 | CQ816931.1 | CS715780.1 |
| CS118901.1 | HC051942.1 | GM634564.1 | BD143210.1 | GN359281.1 | DL106492.1 | CS632847.1 | CQ815740.1 | CS694395.1 |
| CS118867.1 | HC051651.1 | GM634532.1 | AX587642.1 | GN365276.1 | DL106460.1 | CS419508.1 | CQ814523.1 | CS806125.1 |
| CS118834.1 | HC050009.1 | GM634500.1 | AX587592.1 | GN359272.1 | DL106428.1 | CS414535.1 | CQ814058.1 | CS713107.1 |
| CS118801.1 | HC045506.1 | GM634468.1 | AX577499.1 | GN359240.1 | DL128439.1 | CS417156.1 | CQ814025.1 | CS675412.1 |
| CS118769.1 | HC045474.1 | GM664727.1 | AX556850.1 | GN359176.1 | DL124681.1 | CS416593.1 | CQ813993.1 | CS810219.1 |
| CS118736.1 | HC045410.1 | GM655932.1 | AX556816.1 | GN359144.1 | DL124649.1 | CS416219.1 | CQ813961.1 | CS791266.1 |
| CS118672.1 | HC045378.1 | GM648771.1 | AX555181.1 | GN364169.1 | DL124617.1 | CS415771.1 | CQ813929.1 | CS791231.1 |
| CS118640.1 | HC045346.1 | GM648747.1 | AX554146.1 | GN359050.1 | DL124585.1 | CS410691.1 | CQ813897.1 | CS790640.1 |
| CS118608.1 | HC045314.1 | GM648715.1 | AX548057.1 | GN363567.1 | DL096920.1 | CS414846.1 | CQ813865.1 | CS791007.1 |
| CS118575.1 | HC045282.1 | GM648683.1 | L02352.1 | GN363031.1 | DL096888.1 | CS414780.1 | CQ813833.1 | CS790970.1 |
| CS118540.1 | HC042766.1 | GM648651.1 | M60033.1 | GN346511.1 | DL096856.1 | DD320074.1 | CQ813801.1 | CS792488.1 |
| AX241636.1 | HC047462.1 | GM648619.1 | M19567.1 | GN346477.1 | DL096792.1 | DD327033.1 | CQ813728.1 | DJ031802.1 |
| AX241572.1 | HC047398.1 | GM641816.1 | AB541025.1 | GN343939.1 | DL096760.1 | FU258176.1 | CQ812776.1 | DJ045299.1 |
| AX241508.1 | HC047366.1 | GM641784.1 | LT986711.1 | GN346584.1 | DL146283.1 | FU257900.1 | CQ802127.1 | DJ044962.1 |
| AX241476.1 | HC047334.1 | GM641752.1 | LT908473.1 | GN346552.1 | DL138936.1 | FU257836.1 | CQ801674.1 | DJ044940.1 |
| AX241412.1 | GN359476.1 | GM641720.1 | JQ309940.2 | GN340168.1 | DL124473.1 | FU257804.1 | CQ800789.1 | DJ044899.1 |
| AY044144.1 | GN359444.1 | GM641687.1 | AH003168.2 | GM623531.1 | DL124441.1 | FU257772.1 | CQ798793.1 | DJ028148.1 |
| AX234652.1 | GN359412.1 | AX241753.1 | M65078.1 | GM623499.1 | DL124409.1 | FU257739.1 | CQ795482.1 | DJ030203.1 |
| AX225296.1 | GN359380.1 | AX241721.1 | M31611.1 | GM623467.1 | DL124377.1 | FU257707.1 | CQ788865.1 | DJ030087.1 |
| AX207306.1 | GN359348.1 | AX241689.1 | DQ250203.1 | GM623435.1 | DL120288.1 | HB423120.1 | AX023755.1 | DJ030023.1 |
| AX207112.1 | GN359316.1 | AX241657.1 | AF416612.1 | GM623403.1 | DL120256.1 | HB423082.1 | AX023647.1 | DJ029941.1 |
| AX202437.1 | GN359284.1 | AX241625.1 | L78438.1 | GM623371.1 | DL120224.1 | HB416461.1 | AX023615.1 | DJ043652.1 |
| AX193972.1 | GN350105.1 | AX241561.1 | M24230.1 | GM650980.1 | DL115821.1 | HB412954.1 | AX023581.1 | DJ043620.1 |
| AX189768.1 | GN365283.1 | AX241529.1 | AY005806.1 | GM643890.1 | DL115789.1 | HB396997.1 | AX021725.1 | CS721650.1 |
| AX180310.1 | GN359275.1 | AX241497.1 | KJ608141.1 | GM643858.1 | DL115757.1 | HB396567.1 | AX019760.1 | CS691627.1 |
| AF394912.1 | GN359243.1 | AX241465.1 | HW390745.1 | GM643826.1 | DL115725.1 | HB394814.1 | AX018977.1 | CS686964.1 |
| AX172951.1 | GN359211.1 | AX241433.1 | HW405829.1 | GM636829.1 | DL115693.1 | HB393703.1 | AX016227.1 | CS674206.1 |
| AX151021.1 | GN359147.1 | AX241126.1 | HW399407.1 | GM636797.1 | DL115661.1 | HB397671.1 | AX011473.1 | CS677757.1 |
| AX151757.1 | GN359115.1 | AX241094.1 | HW399318.1 | GM636765.1 | DL115629.1 | DM112030.1 | AX011441.1 | DJ009231.1 |
| AX145742.1 | GN359083.1 | HW267610.1 | AX769730.1 | GM632261.1 | DL111235.1 | DM115850.1 | AX011409.1 | DJ008412.1 |

| | | | | | | | | |
|---|---|---|---|---|---|---|---|---|
| AX145710.1 | GN359053.1 | HW266209.1 | AX754994.1 | GM632229.1 | DL111171.1 | GM616289.1 | AX011376.1 | DJ008380.1 |
| AX145678.1 | GN358847.1 | HW263743.1 | AX752629.1 | GM623330.1 | DL111139.1 | GM604350.1 | AX011344.1 | DJ008348.1 |
| AX145646.1 | GN363573.1 | HW262998.1 | M16781.1 | GM623298.1 | DL111107.1 | GM038735.1 | AX010938.1 | DJ008316.1 |
| AX145614.1 | GN363038.1 | HW262918.1 | BD181610.1 | GM623266.1 | DL111075.1 | GM000684.1 | AX010515.1 | DJ011442.1 |
| AX145582.1 | GN362483.1 | HW262875.1 | AX319350.1 | GM623234.1 | DL106271.1 | HB388028.1 | AX009278.1 | DD140155.1 |
| AX145550.1 | GN346514.1 | HW262772.1 | AX275256.1 | GM741840.1 | DL106239.1 | HB387128.1 | AX006193.1 | DD081469.1 |
| AX145518.1 | GN346480.1 | HW262577.1 | AX258829.1 | GM741808.1 | DL106175.1 | HB340406.1 | AX003763.1 | DD081182.1 |
| AX145486.1 | GN346900.1 | HW261409.1 | AX300949.1 | GM658322.1 | DL101402.1 | HA642285.1 | AX002819.1 | DD080800.1 |
| AX145454.1 | GN346587.1 | HW261377.1 | AX286668.1 | GM658290.1 | DL101370.1 | HA641616.1 | A57353.1 | DD138771.1 |
| AX145422.1 | GN346555.1 | HW261345.1 | AX282312.1 | GM658258.1 | DL101338.1 | HA641488.1 | A46505.1 | DD122270.1 |
| AX145389.1 | GN339107.1 | HW261313.1 | AX278055.1 | GM658226.1 | DL103455.1 | HA635384.1 | A35738.1 | DD137696.1 |
| AX145357.1 | GN130791.1 | HW261281.1 | AX256072.1 | GM658194.1 | DL103423.1 | HA639794.1 | A35537.1 | DD137312.1 |
| AX145325.1 | GN334570.1 | HW261249.1 | AX253472.1 | GM658162.1 | DL103391.1 | DM102538.1 | A33038.1 | DD136474.1 |
| AX145293.1 | GN116534.1 | HW261217.1 | AX247548.1 | GM632078.1 | DL098785.1 | DM105624.1 | A34037.1 | DD135513.1 |
| AX145261.1 | GN116502.1 | HW261185.1 | AX242320.1 | GM658112.1 | DL098753.1 | DM105584.1 | A33979.1 | DD135336.1 |
| AX145229.1 | GN116469.1 | HW261153.1 | AX242288.1 | GM658080.1 | DL092743.1 | DM094905.1 | A23356.1 | DD134025.1 |
| AX145197.1 | GN116437.1 | HW261121.1 | AX242256.1 | GM658048.1 | DL092679.1 | GM651539.1 | A27452.1 | DD133030.1 |
| AX145165.1 | GN116405.1 | HW261089.1 | AX242224.1 | GM658016.1 | DL092647.1 | GM651507.1 | A20137.1 | DD132295.1 |
| AX145133.1 | GN116373.1 | HW261057.1 | AX242192.1 | GM657984.1 | DL092615.1 | GM651443.1 | A19605.1 | DD080702.1 |
| AX145101.1 | GN116309.1 | HW261025.1 | AX242160.1 | GM657952.1 | DL092583.1 | GM651411.1 | A15383.1 | DD107156.1 |
| AX145069.1 | GN112575.1 | HW260993.1 | AX242128.1 | GM650760.1 | DL088968.1 | GM651379.1 | A13851.1 | DD093920.1 |
| AX145037.1 | GN115512.1 | HW260929.1 | AX242032.1 | GM650728.1 | DL016142.1 | GM644580.1 | A10444.1 | DD093888.1 |
| AX145005.1 | GN115294.1 | HW260897.1 | AX241968.1 | GM650696.1 | DL070825.1 | GM644548.1 | A08530.1 | DD093856.1 |
| AX144973.1 | GN114937.1 | HW260865.1 | AX241904.1 | GM643704.1 | DL031642.1 | GM644516.1 | A05826.1 | DD093378.1 |
| AX144941.1 | GN114865.1 | HW260833.1 | AX241872.1 | GM643672.1 | DL031610.1 | GM644452.1 | A32066.1 | DD092969.1 |
| AX144908.1 | GN094580.1 | HW260801.1 | AX241840.1 | GM643632.1 | DL031578.1 | GM644420.1 | A32024.1 | DD092355.1 |
| AX144876.1 | DL122318.1 | HW260769.1 | AX241808.1 | GM636643.1 | DL031546.1 | GM637422.1 | A30412.1 | DD119267.1 |
| AX144844.1 | DL117968.1 | HW260737.1 | AX241776.1 | GM636611.1 | DL027737.1 | GM637390.1 | A29090.1 | DD061437.1 |
| AX144812.1 | DL117936.1 | HW260705.1 | AX241744.1 | GM636579.1 | DL027705.1 | GM637358.1 | A27941.1 | DD118657.1 |
| AX144780.1 | DL117904.1 | HW260673.1 | AX241680.1 | GM636547.1 | DL027673.1 | GM637326.1 | A25896.1 | DD118572.1 |
| AX144748.1 | DL117872.1 | HW260641.1 | AX241648.1 | GM636515.1 | DL027641.1 | GM637294.1 | A20235.1 | DD061345.1 |
| AX144716.1 | DL117840.1 | HW260577.1 | AX241584.1 | GM636483.1 | DL027609.1 | GM637262.1 | A04441.1 | DD061291.1 |
| AX144684.1 | DL113119.1 | HW260545.1 | AX241552.1 | GM632075.1 | DL027577.1 | GM623725.1 | A25701.1 | DD091167.1 |
| AX144652.1 | DL113087.1 | HW260513.1 | AX241520.1 | GM623112.1 | DL023669.1 | GM623693.1 | A24419.1 | DD118113.1 |
| AX144620.1 | DL113055.1 | HW260449.1 | AX241488.1 | GM623080.1 | DL023637.1 | GM623661.1 | A20745.1 | DD104531.1 |
| AX144563.1 | DL108254.1 | HW260417.1 | AX241456.1 | GM650470.1 | DL023605.1 | GM623629.1 | A23640.1 | DD090689.1 |
| AX144499.1 | DL108222.1 | HW260385.1 | AX241424.1 | GM650438.1 | DL023573.1 | GM623597.1 | A22076.1 | DD117215.1 |
| HV760394.1 | DL108190.1 | HW260353.1 | AX235761.1 | GM650406.1 | DL015995.1 | GM623565.1 | A16158.1 | DD116431.1 |
| HV752238.1 | DL108158.1 | HW260321.1 | AX235202.1 | GM643598.1 | DL015963.1 | GM658714.1 | A12741.1 | DD089600.1 |
| HV749444.1 | DL108126.1 | HW260289.1 | AX223892.1 | GM643566.1 | DL015931.1 | GM658682.1 | A21625.1 | CS103457.1 |
| HV750930.1 | DL108094.1 | HW260257.1 | AX212310.1 | GM643534.1 | DL015899.1 | GM658650.1 | A20893.1 | CS103394.1 |
| HV753493.1 | DL103488.1 | HW260225.1 | AX203100.1 | GM643502.1 | DL015867.1 | GM658554.1 | A20136.1 | CS103362.1 |
| HV704839.1 | DL103456.1 | HW260193.1 | HW355217.1 | GM643470.1 | DL011249.1 | GM651362.1 | A19403.1 | CS103304.1 |
| HV704290.1 | DL103424.1 | HW260161.1 | HW338950.1 | GM643437.1 | DL011217.1 | GM651330.1 | DL086951.1 | CS103086.1 |
| HV704136.1 | DL103392.1 | HW260129.1 | HW338822.1 | GM636448.1 | DL047641.1 | GM651298.1 | DL086919.1 | CS102987.1 |
| HV744632.1 | DL098786.1 | HW260097.1 | HW338566.1 | DL212465.1 | DL047609.1 | GM644403.1 | DL086887.1 | CS102955.1 |
| HV703149.1 | DL098754.1 | HW260065.1 | HW338438.1 | DL227048.1 | DJ326985.1 | GM644371.1 | DL086855.1 | CS102923.1 |

| | | | | | | | | |
|---|---|---|---|---|---|---|---|---|
| HV701287.1 | DL092744.1 | HW260033.1 | HW338310.1 | DL206287.1 | DJ128228.1 | GM644339.1 | DL086791.1 | CS102891.1 |
| HV701225.1 | DL092712.1 | HW260001.1 | HW338182.1 | DL213565.1 | DJ122488.1 | GM644307.1 | DL086759.1 | CS102859.1 |
| HV701193.1 | DL092680.1 | HW259969.1 | HW337926.1 | FB714906.1 | DJ090100.1 | GM644275.1 | DL086727.1 | CS102827.1 |
| HV701129.1 | DL092648.1 | HW259741.1 | HW337670.1 | FB714481.1 | DJ082571.1 | GM644243.1 | DL086695.1 | CS102763.1 |
| HV708197.1 | DL092616.1 | HW259333.1 | HW337286.1 | FB713797.1 | DJ081977.1 | GM637245.1 | DL086663.1 | CS102731.1 |
| HV708123.1 | DL092584.1 | HW258972.1 | HW321804.1 | FB713765.1 | DJ081426.1 | GM637213.1 | DL105872.1 | CS102667.1 |
| HV701078.1 | DL088969.1 | HW258905.1 | HW336902.1 | FB708015.1 | DJ066779.1 | GM637181.1 | DL105840.1 | CS102635.1 |
| HV701046.1 | DL088937.1 | HW257420.1 | HW336746.1 | FB668109.1 | DJ071357.1 | GM637149.1 | DL105808.1 | CS102603.1 |
| HV695540.1 | DL088905.1 | HW257388.1 | HW336618.1 | FB667103.1 | DJ066383.1 | GM637117.1 | DL096525.1 | CS102571.1 |
| HV444122.1 | DL103312.1 | HW257356.1 | HW336538.1 | FB666792.1 | DJ066318.1 | GM637085.1 | DL096493.1 | CS102539.1 |
| HV444018.1 | DL103280.1 | HW257292.1 | HW321044.1 | FB676571.1 | DJ066284.1 | GM637053.1 | DL096461.1 | CS102507.1 |
| HV437727.1 | DL103248.1 | HW257260.1 | HW329411.1 | FB676539.1 | DJ066250.1 | GM624900.1 | DL110738.1 | CS101262.1 |
| HV341334.1 | DL103216.1 | HW257228.1 | HW335969.1 | FB705904.1 | DJ068015.1 | GM624868.1 | DL110706.1 | CS091843.1 |
| HV322572.1 | DL107968.1 | HW257068.1 | HW328678.1 | GM655864.1 | DJ065228.1 | GM624832.1 | DL115513.1 | CS088898.1 |
| FZ412046.1 | DL107936.1 | HW256876.1 | HW328551.1 | GM648775.1 | DJ066989.1 | GM632651.1 | DL115481.1 | CS086830.1 |
| FW590742.1 | DL107904.1 | HW256748.1 | HW319587.1 | GM648743.1 | DJ061210.1 | GM632579.1 | DL115449.1 | CS086504.1 |
| FW575895.1 | DL092446.1 | HW256716.1 | HW328438.1 | GM648711.1 | DJ055286.1 | GM632611.1 | DL105697.1 | CS086472.1 |
| FW420451.1 | DL092414.1 | HW251064.1 | HW328335.1 | GM648679.1 | DJ057673.1 | GM741941.1 | DL105665.1 | CS084861.1 |
| GM630281.1 | DL092382.1 | HV504105.1 | HW328156.1 | GM648647.1 | DJ060408.1 | GM658429.1 | DL105633.1 | CS082425.1 |
| GM630249.1 | DL028733.1 | HV504073.1 | HW318468.1 | GM648615.1 | DJ056885.1 | GM658373.1 | DL105601.1 | CS082361.1 |
| GM630217.1 | DL028669.1 | HV504009.1 | HW335176.1 | GM634463.1 | DJ059898.1 | GM658341.1 | DL100796.1 | CS082290.1 |
| GM630185.1 | DL028637.1 | HV503977.1 | HW314388.1 | GM634431.1 | DJ053113.1 | GM651149.1 | DL100764.1 | CS082113.1 |
| GM630153.1 | DL028605.1 | HV503945.1 | HW314147.1 | GM634399.1 | DJ053081.1 | GM651117.1 | DL100732.1 | CS081643.1 |
| GM630121.1 | DL028573.1 | HV503913.1 | HW312100.1 | GM634367.1 | CS810637.1 | GM651085.1 | DL096318.1 | CS080409.1 |
| GM621178.1 | DL024759.1 | HV503881.1 | HW311895.1 | GM629476.1 | CS810529.1 | GM651053.1 | DL096286.1 | CS079568.1 |
| GM738523.1 | DL024727.1 | HV503849.1 | HW311240.1 | GM629434.1 | CS812641.1 | GM651021.1 | DL096254.1 | CS078837.1 |
| GM698667.1 | DL024695.1 | HV503817.1 | HW311026.1 | GM629338.1 | CS811054.1 | GM650989.1 | DL022233.1 | CS078230.1 |
| GM698613.1 | DL012371.1 | HV491220.1 | HV819460.1 | GM648569.1 | DJ016607.1 | GM644190.1 | DL025348.1 | CS078174.1 |
| GM697928.1 | DL048843.1 | HV491105.1 | HV932910.1 | GM648537.1 | DJ015677.1 | GM644158.1 | DL025316.1 | CS077203.1 |
| GM635052.1 | DL048692.1 | HV503777.1 | HV932683.1 | GM648505.1 | CS803368.1 | GM644126.1 | DL025284.1 | CS074561.1 |
| GM635020.1 | DL048660.1 | HV503745.1 | HV932651.1 | GM641606.1 | CS803335.1 | GM644094.1 | DL025220.1 | CS073118.1 |
| GM634988.1 | DL045242.1 | HV503713.1 | HV929719.1 | GM629165.1 | DJ047303.1 | GM644062.1 | DL021784.1 | CS070130.1 |
| GM634956.1 | DL045210.1 | HV503681.1 | HV802935.1 | GM629133.1 | DJ046994.1 | GM644030.1 | DL038454.1 | CS063856.1 |
| GM634924.1 | DL045178.1 | HV503525.1 | HV802903.1 | GM629101.1 | DJ052209.1 | GM636904.1 | DL038422.1 | CS062978.1 |
| GM634892.1 | DL045146.1 | HV503493.1 | HV818560.1 | GM634063.1 | DJ051993.1 | GM636872.1 | DL038390.1 | CS061692.1 |
| GM630086.1 | DL045114.1 | HV503461.1 | HV784273.1 | GM634031.1 | DD405592.1 | DM164205.1 | DL038358.1 | CS061046.1 |
| GM630054.1 | DL045082.1 | HV503397.1 | HV777560.1 | GM633999.1 | DD405528.1 | DM164026.1 | DL038326.1 | CS059017.1 |
| GM630022.1 | DL040970.1 | HV503365.1 | HV775700.1 | GM633967.1 | DD405496.1 | DM164011.1 | DL013163.1 | AY967393.1 |
| GM629990.1 | DL040938.1 | HV503333.1 | HV775022.1 | GM716516.1 | DD405464.1 | DM163977.1 | DL013131.1 | AY967361.1 |
| GM629958.1 | DL040906.1 | HV503301.1 | HV764762.1 | GM655512.1 | DD402026.1 | GN067829.1 | DL013099.1 | AY967329.1 |
| GM629926.1 | DL037083.1 | BD074948.1 | HV764698.1 | GM655480.1 | DD401994.1 | GN067769.1 | DL013067.1 | AY967297.1 |
| GM642412.1 | DL037051.1 | BD074784.1 | HV763995.1 | GM655448.1 | DD401962.1 | GN067737.1 | DL013035.1 | AY967265.1 |
| GM642380.1 | DL037019.1 | BD073413.1 | HV774117.1 | GM601146.1 | DD401901.1 | GN065232.1 | DL013003.1 | AY967233.1 |
| GM642348.1 | DL024546.1 | BD063671.1 | HV561911.1 | FB720135.1 | DD401869.1 | GN052461.1 | DL033738.1 | AY967201.1 |
| GM642316.1 | DL024514.1 | BD061588.1 | HV554549.1 | FB715072.1 | DD401837.1 | GN052429.1 | DL042140.1 | AY967169.1 |
| GM642230.1 | DL024482.1 | BD057141.1 | HV553154.1 | FB706020.1 | DD401597.1 | GN052386.1 | DL042108.1 | AY967137.1 |
| GM642276.1 | DL024450.1 | BD017723.1 | HV550394.1 | FB677407.1 | DD401565.1 | GN051434.1 | DL042937.1 | AY967105.1 |

| | | | | | | | | |
|---|---|---|---|---|---|---|---|---|
| GM642220.1 | DL024418.1 | BD016685.1 | HV550345.1 | GM597045.1 | DD401533.1 | GN045885.1 | DL042905.1 | AY967073.1 |
| GM656343.1 | DL024386.1 | BD014229.1 | AY659396.1 | GM061140.1 | DD401501.1 | DM024189.1 | DL042873.1 | AY967041.1 |
| GM656311.1 | DL021174.1 | BD014157.1 | AY659364.1 | DL260582.1 | DD401469.1 | DM011022.1 | DL038992.1 | AY967009.1 |
| GM656279.1 | DL021142.1 | E63779.1 | AY659332.1 | DL257042.1 | CS480598.1 | DM022272.1 | DL038960.1 | AY966977.1 |
| GM656247.1 | DL021110.1 | AX481253.1 | AY659300.1 | DL241178.1 | CS479226.1 | DM022200.1 | DL038928.1 | AY966945.1 |
| GM656215.1 | DL021078.1 | AX473138.1 | BD453794.1 | DL241039.1 | CS469000.1 | DM022152.1 | DL027139.1 | CS057842.1 |
| GM656183.1 | DL021014.1 | AX472281.1 | BD433658.1 | FB748878.1 | CS466065.1 | DM038365.1 | DL027107.1 | CS057466.1 |
| GM649158.1 | DL012190.1 | AX468449.1 | BD453770.1 | FB674305.1 | DD392038.1 | DM021889.1 | DL027075.1 | CS056433.1 |
| GM649126.1 | DL012158.1 | AX463659.1 | BD453738.1 | DL238828.1 | DD391922.1 | DM015979.1 | DL027043.1 | CQ873563.1 |
| GM649094.1 | DL012126.1 | A23989.1 | BD453706.1 | DL236612.1 | DD382835.1 | DM027039.1 | DL027011.1 | CQ873299.1 |
| GM649062.1 | DL012094.1 | A10356.1 | BD453674.1 | DL233409.1 | DD400159.1 | GN045763.1 | DL026979.1 | HV549303.1 |
| GM649030.1 | DL012062.1 | AX460076.1 | BD453642.1 | DL220458.1 | DD400097.1 | GN040063.1 | DL030906.1 | HV549224.1 |
| GM642195.1 | DL027051.1 | AX458514.1 | BD453610.1 | DL206284.1 | DD393673.1 | GN034788.1 | DL030867.1 | HV549192.1 |
| GM642163.1 | DL027019.1 | AX456489.1 | BD493414.1 | FB714897.1 | CS463730.1 | GN030715.1 | DL030835.1 | HV549160.1 |
| GM642131.1 | DL026987.1 | AX455876.1 | BD453560.1 | FB713954.1 | CS462997.1 | GN030683.1 | DL030803.1 | HV549090.1 |
| GM642099.1 | DL026955.1 | AX454140.1 | BD453528.1 | FB713794.1 | CS460159.1 | GN030651.1 | DL030771.1 | HV549009.1 |
| GM642067.1 | DL030914.1 | AX453993.1 | BD453496.1 | FB713762.1 | CS459104.1 | GN030619.1 | DL030739.1 | HV544360.1 |
| GM642035.1 | DL030875.1 | AX451627.1 | BD453464.1 | FB713730.1 | DD361290.1 | GN030587.1 | DL026930.1 | HV544167.1 |
| GM634848.1 | DL030843.1 | AX441385.1 | BD453432.1 | FB712762.1 | DD367618.1 | GN030555.1 | DL019751.1 | HV547849.1 |
| GM634816.1 | DL030811.1 | AX443296.1 | BD453400.1 | DL095732.1 | DD367331.1 | GN030523.1 | DL019719.1 | HV543738.1 |
| GM634784.1 | DL030779.1 | AX441273.1 | BD453255.1 | DL095700.1 | DD373566.1 | GN030459.1 | DL015156.1 | HV547273.1 |
| GM634752.1 | DL030747.1 | AX429439.1 | BD453191.1 | DL095668.1 | DD362917.1 | GN030331.1 | DL010538.1 | HV547414.1 |
| GM634720.1 | DL019779.1 | AX428536.1 | BD442385.1 | DL095636.1 | DD362224.1 | GN030299.1 | DL010506.1 | HV543505.1 |
| GM629882.1 | DL019783.1 | AX427114.1 | BD420380.1 | DL095604.1 | DD368157.1 | GN030203.1 | DL010474.1 | HV539534.1 |
| GM629850.1 | DL019727.1 | AX411450.1 | BD497461.1 | DL095572.1 | DD361324.1 | GN030075.1 | DL010442.1 | HV538616.1 |
| GM629818.1 | DL015164.1 | AX404813.1 | BD451716.1 | DL117409.1 | CS457840.1 | GN030011.1 | DL010410.1 | HV538460.1 |
| GM629786.1 | DL015132.1 | AX403246.1 | BD398290.1 | DL117377.1 | CS457144.1 | GN029915.1 | DL038848.1 | HV537717.1 |
| GM841798.1 | DL015100.1 | AX395280.1 | BD440511.1 | DL117345.1 | BD261823.1 | GN029849.1 | DL038784.1 | HV543186.1 |
| GM841766.1 | DL015068.1 | AX391840.1 | BD449766.1 | DL091636.1 | BD251448.1 | GN029817.1 | DL038752.1 | HV542724.1 |
| GM603723.1 | DL010546.1 | AX385071.1 | BD429591.1 | DL091604.1 | BD251232.1 | GM637600.1 | DL038720.1 | HV542692.1 |
| GM706724.1 | DL010514.1 | HW315169.1 | BD429481.1 | DL091572.1 | BD248421.1 | GM637568.1 | DL035096.1 | HV542187.1 |
| GM706692.1 | DL010482.1 | HW315105.1 | BD407492.1 | DL087961.1 | BD247526.1 | GM637504.1 | DL035064.1 | HV541868.1 |
| GM706660.1 | GM647348.1 | HW315073.1 | BD428775.1 | DL087929.1 | BD247051.1 | GM637472.1 | DL035032.1 | HV541049.1 |
| GM706396.1 | GM647316.1 | HW314782.1 | BD460608.1 | DL121760.1 | BD245181.1 | GM633064.1 | DL030717.1 | HV515076.1 |
| GM706362.1 | GM647284.1 | HW314727.1 | BD427995.1 | DL121728.1 | BD243953.1 | GM633032.1 | DL030685.1 | HV533133.1 |
| GM706329.1 | GM647252.1 | HW314617.1 | BD427310.1 | DL121696.1 | BD242457.1 | GM633000.1 | CS619004.1 | HV536544.1 |
| GM706322.1 | GM647220.1 | HW121468.1 | BD395074.1 | DL121664.1 | BD241792.1 | GM632968.1 | CS614441.1 | HV536319.1 |
| FB722374.1 | GM627678.1 | HW106132.1 | AX512766.1 | DL121632.1 | BD240781.1 | GM632936.1 | CS611830.1 | HV537435.1 |
| FB720272.1 | GM627646.1 | HW112779.1 | BD194482.1 | DL121600.1 | BD238477.1 | GM632904.1 | CS604540.1 | HV535875.1 |
| FB715310.1 | GM870255.1 | HW112675.1 | BD188800.1 | DL125448.1 | BD238035.1 | GM625325.1 | CS604508.1 | HV532394.1 |
| FB706330.1 | GM627586.1 | HW070113.1 | AX787358.1 | DL125416.1 | CS021533.1 | GM625293.1 | CS604476.1 | HV537250.1 |
| FB677445.1 | GM627554.1 | HW069953.1 | AX781523.1 | DL125384.1 | CS019534.1 | GM625261.1 | CS604412.1 | HV535450.1 |
| FB677000.1 | GM627522.1 | HW084945.1 | AX773271.1 | DL125352.1 | CS018543.1 | GM625229.1 | CS604380.1 | HV516274.1 |
| GM708624.1 | GM661495.1 | HW097739.1 | AX770676.1 | DL121467.1 | CS018479.1 | GM625197.1 | CS604348.1 | HV528234.1 |
| GM061163.1 | GM661463.1 | HW067063.1 | AX769725.1 | DL121435.1 | CS018247.1 | GM625165.1 | CS604316.1 | HV515591.1 |
| DL460034.1 | GM661431.1 | HW087988.1 | AX754990.1 | DL117191.1 | CS016581.1 | GM625133.1 | CS604188.1 | HV515559.1 |
| DL459989.1 | GM661399.1 | HW065051.1 | AX752767.1 | DL117159.1 | CQ990434.1 | GM624043.1 | CS603964.1 | HV515527.1 |

| | | | | | | | | |
|---|---|---|---|---|---|---|---|---|
| DL260644.1 | GM661367.1 | HW047620.1 | AX752628.1 | DL117127.1 | CQ986631.1 | GM624011.1 | CS603484.1 | HV515495.1 |
| DL260264.1 | GM661335.1 | HW047244.1 | BD182994.1 | DL102496.1 | CQ986597.1 | GM623979.1 | CS603452.1 | HV515463.1 |
| DL260230.1 | GM654341.1 | HV969577.1 | M19566.1 | DL102464.1 | CQ986565.1 | GM623947.1 | DJ438197.1 | HV515431.1 |
| DL260150.1 | GM654309.1 | HV956405.1 | BD181609.1 | DL102432.1 | CQ983186.1 | GM625121.1 | DJ400828.1 | HV515399.1 |
| DL259833.1 | GM654277.1 | HV956151.1 | BD181301.1 | DL102400.1 | CQ983104.1 | GM625089.1 | DJ400796.1 | HV515367.1 |
| DL258281.1 | GM654245.1 | HV957508.1 | BD180855.1 | DL102368.1 | CQ983070.1 | GM625057.1 | DJ398953.1 | HV515335.1 |
| DL257644.1 | GM647188.1 | HV948584.1 | AX743758.1 | DL102336.1 | CQ983004.1 | GM625025.1 | DJ391345.1 | HV515303.1 |
| DL241390.1 | GM647156.1 | HV944649.1 | AX741752.1 | DL097627.1 | CQ982972.1 | GM624993.1 | DJ418052.1 | HV515239.1 |
| DL241157.1 | GM647124.1 | HV780576.1 | BD177333.1 | DL097595.1 | CQ982935.1 | GM624961.1 | DJ417997.1 | HV511361.1 |
| DL241827.1 | GM647092.1 | HV779882.1 | AX719153.1 | DL097563.1 | CQ982903.1 | GM623935.1 | DJ402750.1 | HV511251.1 |
| FB748698.1 | GM647052.1 | HV778415.1 | AX708824.1 | DL095536.1 | CQ982825.1 | GM623903.1 | DJ402718.1 | FW562406.1 |
| FB742185.1 | FB676540.1 | HV774722.1 | AX703585.1 | DL095504.1 | CQ981124.1 | GM623871.1 | DJ381029.1 | FW556902.1 |
| FB742127.1 | FB676508.1 | HV773785.1 | AX699453.1 | DL095472.1 | CQ975462.1 | GM623839.1 | DJ380997.1 | FW553190.1 |
| FB748140.1 | FB704810.1 | HV692943.1 | AX699421.1 | DL095440.1 | CQ974095.1 | GM623807.1 | DJ380965.1 | FW563893.1 |
| DL234088.1 | FB701523.1 | HW302696.1 | AX685556.1 | DL095408.1 | CQ973486.1 | GM623775.1 | DJ380933.1 | FW566292.1 |
| DL233421.1 | CS809046.1 | HW302539.1 | AX683862.1 | DL095376.1 | CQ976622.1 | GM623743.1 | DJ380901.1 | FW559425.1 |
| DL233387.1 | FB707287.1 | HV755980.1 | AX676273.1 | DL095344.1 | CQ975970.1 | GM658922.1 | DJ380869.1 | FW559402.1 |
| DL232136.1 | FB701081.1 | HV753389.1 | A57343.1 | DL085799.1 | CQ972396.1 | GM658890.1 | DJ380817.1 | FW559354.1 |
| DL232102.1 | FB660512.1 | HV760023.1 | A29537.1 | DL123347.1 | CQ972364.1 | GM658858.1 | DJ389468.1 | FW510250.1 |
| DL220402.1 | FB660051.1 | HV748683.1 | A35731.1 | DL009380.1 | CQ972332.1 | GM658826.1 | DJ388681.1 | FW506094.1 |
| DL213345.1 | FB580557.1 | HV748523.1 | A35017.1 | DL009348.1 | CQ972119.1 | GM658794.1 | CS726865.1 | HI933586.1 |
| DL206829.1 | FB654429.1 | HV748386.1 | A34021.1 | DL009316.1 | CQ971128.1 | GM651538.1 | CS724927.1 | HI936978.1 |
| DL206665.1 | FB656252.1 | HV751287.1 | A33974.1 | DL009252.1 | CQ971059.1 | GM651506.1 | CS724538.1 | HI936911.1 |
| DL220157.1 | FB580284.1 | HV747583.1 | A32816.1 | DL009220.1 | CQ970875.1 | GM651474.1 | CS724405.1 | HI932081.1 |
| FB360084.1 | FB653523.1 | HV750900.1 | A30377.1 | DL018678.1 | CQ970044.1 | GM651442.1 | CS723811.1 | HI936469.1 |
| DL088352.1 | FB580188.1 | HV753781.1 | A29897.1 | DL018646.1 | CQ968020.1 | GM651410.1 | DJ353081.1 | HI930651.1 |
| DL088320.1 | FB580124.1 | HV743343.1 | A23341.1 | DL018614.1 | CQ963549.1 | GM644611.1 | DJ358575.1 | HI949809.1 |
| DL088288.1 | DL200054.1 | HV743303.1 | A31640.1 | DL014218.1 | CQ955896.1 | GM644579.1 | DJ359482.1 | HI934096.1 |
| DL088256.1 | DL195149.1 | HV743228.1 | A26300.1 | DL014186.1 | CQ947172.1 | GM644547.1 | DJ357709.1 | HI929260.1 |
| DL088224.1 | DL189491.1 | HV502785.1 | A23405.1 | CS792477.1 | CQ947123.1 | GM644515.1 | DJ357611.1 | HI921900.1 |
| DL088192.1 | DL199821.1 | HV502753.1 | A16252.1 | CS791973.1 | CQ944193.1 | GM644451.1 | DJ357489.1 | HI918274.1 |
| DL088160.1 | DL199789.1 | HV502721.1 | A19185.1 | DJ044929.1 | CQ944161.1 | GM644419.1 | DJ344660.1 | HI661442.1 |
| DL088128.1 | DL184471.1 | HV502689.1 | A13578.1 | DJ044888.1 | CQ944129.1 | GM637421.1 | DJ354279.1 | HI661269.1 |
| DL088096.1 | DL196198.1 | HV502657.1 | A08360.1 | DJ028135.1 | CQ944097.1 | GM637389.1 | DJ354188.1 | HI661028.1 |
| DL088064.1 | DL196085.1 | HV502625.1 | A01483.1 | DJ029987.1 | CQ944065.1 | GM637357.1 | DJ360413.1 | HI923668.1 |
| DL088032.1 | DL193798.1 | HV502593.1 | A31927.1 | DJ026278.1 | CQ944033.1 | GM637325.1 | DJ339848.1 | HI658097.1 |
| DL088000.1 | DL193766.1 | HV502561.1 | A31081.1 | CS682331.1 | CQ944001.1 | GM637293.1 | E38280.1 | HI657282.1 |
| DL091941.1 | DL184044.1 | HV502465.1 | A30288.1 | CS691863.1 | CQ943969.1 | GM637261.1 | E06809.1 | HI656350.1 |
| DL091909.1 | DL183348.1 | HV497257.1 | A25891.1 | DJ012309.1 | CQ943937.1 | GM623724.1 | E06033.1 | HI654468.1 |
| DL091877.1 | DL183216.1 | HV492713.1 | A20022.1 | DJ008401.1 | CQ943905.1 | GM623692.1 | E05775.1 | FW498199.1 |
| DL091845.1 | FB571379.1 | HV505313.1 | A28460.1 | DJ008369.1 | CQ943873.1 | GM623660.1 | E05662.1 | FW503026.1 |
| DL091813.1 | CS696199.1 | HV505209.1 | A27396.1 | DJ008049.1 | A12561.1 | GM625120.1 | E05205.1 | FW496153.1 |
| DL091781.1 | CS696071.1 | HV494723.1 | A26059.1 | DD495504.1 | CQ898647.1 | GM625088.1 | E04417.1 | FW502862.1 |
| DL102902.1 | CS696039.1 | HV507618.1 | A24403.1 | CS668191.1 | HW408448.1 | GM625056.1 | E04177.1 | FW497329.1 |
| DL102870.1 | CS695910.1 | HV452180.1 | A25919.1 | CS646229.1 | HW408416.1 | GM625024.1 | E03640.1 | FW503887.1 |
| DL102838.1 | CS695846.1 | HV453657.1 | A24543.1 | CS646195.1 | HW408384.1 | GM624992.1 | E03152.1 | FW499110.1 |
| DL102806.1 | CS695750.1 | AY657707.1 | A14553.1 | CS644243.1 | HW390751.1 | GM624960.1 | E02977.1 | FW499037.1 |

| | | | | | | | | |
|---|---|---|---|---|---|---|---|---|
| DL102774.1 | CS695654.1 | AY657675.1 | A12374.1 | DD462048.1 | HW399324.1 | GM623934.1 | E02570.1 | FW504943.1 |
| DL102742.1 | DL106068.1 | AY657643.1 | A21548.1 | DD460143.1 | HW409000.1 | GM623870.1 | E02017.1 | FW500474.1 |
| DL107682.1 | DL106036.1 | AY657611.1 | A21231.1 | DD455455.1 | HW390658.1 | GM623838.1 | E01877.1 | FW498997.1 |
| DL107650.1 | DL106004.1 | AY657579.1 | A20092.1 | DD458202.1 | HW390523.1 | GM623806.1 | E01422.1 | HI546296.1 |
| DL107618.1 | DL105972.1 | AY657547.1 | A18407.1 | DD453812.1 | HW390414.1 | GM623774.1 | E01210.1 | HI587844.1 |
| DL107586.1 | DL105940.1 | AY657515.1 | A18745.1 | CS376127.1 | HW389670.1 | GM623742.1 | E00969.1 | HI211036.1 |
| DL107522.1 | DL105908.1 | AY657483.1 | A18185.1 | CS375992.1 | HW389150.1 | GM654968.1 | E00822.1 | HI210974.1 |
| DL096441.1 | DL101295.1 | AY657451.1 | A17117.1 | CS375756.1 | HW389021.1 | GM640915.1 | E00590.1 | HI210942.1 |
| DL096377.1 | DL101263.1 | AY657419.1 | A05506.1 | CS359740.1 | HW388634.1 | GM640883.1 | E00306.1 | HI570418.1 |
| DL096345.1 | DL101231.1 | AY657387.1 | A14450.1 | CS359604.1 | HW386238.1 | GM640851.1 | DD084742.1 | HI472915.1 |
| DL094517.1 | DL101199.1 | AY657355.1 | A13604.1 | BD269141.1 | HW380847.1 | GM628497.1 | DD112540.1 | HI583982.1 |
| DL094485.1 | DL101167.1 | AY657323.1 | HC046935.1 | BD268162.1 | HW380815.1 | GM662110.1 | DD069503.1 | HI209392.1 |
| DL094453.1 | DL096721.1 | AY657291.1 | HC046903.1 | BD268119.1 | HW379428.1 | GM654828.1 | DD098147.1 | HI004389.1 |
| DL094389.1 | DL096689.1 | AY657259.1 | HC046875.1 | BD266724.1 | HW047619.1 | GM654796.1 | DD152073.1 | HI004325.1 |
| DL094357.1 | DL096657.1 | AY657227.1 | HC046843.1 | BD265612.1 | HW047243.1 | GM654764.1 | DD158399.1 | HI004261.1 |
| DL110846.1 | DL096625.1 | AY657195.1 | HC046811.1 | BD263451.1 | HV969576.1 | GM647771.1 | DD157376.1 | HI002284.1 |
| DL110814.1 | DL096593.1 | AY657163.1 | HC046747.1 | BD263419.1 | HV959658.1 | GM647739.1 | DD156987.1 | DL102373.1 |
| DL110782.1 | DL096561.1 | AY657131.1 | HC046715.1 | BD261822.1 | HV957506.1 | GM647707.1 | DD155702.1 | DL102341.1 |
| DL110750.1 | DL094733.1 | AY657099.1 | HC039689.1 | BD251231.1 | HV944648.1 | GM640808.1 | DD154055.1 | DL097632.1 |
| DL110686.1 | DL094701.1 | AY657067.1 | HC039632.1 | BD246976.1 | HV780472.1 | GM640775.1 | DD152992.1 | DL097568.1 |
| DL090748.1 | DL094669.1 | AY657035.1 | HC046063.1 | BD245179.1 | HV779881.1 | GM640743.1 | DD151861.1 | DL095541.1 |
| DL090716.1 | DL090958.1 | M20400.1 | HC045707.1 | BD244613.1 | HV774721.1 | GM640711.1 | DD097297.1 | DL095509.1 |
| DL090684.1 | DL090926.1 | M10536.1 | HC045675.1 | BD243952.1 | HV773784.1 | GM640679.1 | DD096863.1 | DL095477.1 |
| DL090652.1 | DL086958.1 | M13382.1 | HC021360.1 | BD243438.1 | HV743667.1 | GM640647.1 | DD082111.1 | DL095445.1 |
| DL090620.1 | DL086926.1 | M17341.1 | HC025525.1 | BD242456.1 | HV743380.1 | GM628293.1 | DD149107.1 | DL095413.1 |
| DL090588.1 | DL086894.1 | K00903.1 | HC025493.1 | BD241791.1 | HW287328.1 | GM627969.1 | DD147876.1 | DL095349.1 |
| DL086583.1 | DL086862.1 | HV549078.1 | HC025461.1 | BD238034.1 | HW302691.1 | GM627937.1 | DD147782.1 | DL091439.1 |
| DL086551.1 | DL086830.1 | HV544282.1 | HC025429.1 | BD235642.1 | HW295902.1 | GM661747.1 | DD147750.1 | DL091407.1 |
| DL086519.1 | DL086798.1 | HV543986.1 | HC010664.1 | BD234854.1 | HW302538.1 | GM654753.1 | DD147718.1 | DL091375.1 |
| DL086487.1 | DL086766.1 | HV547340.1 | HC010415.1 | BD234427.1 | HW295547.1 | GM654721.1 | DD147686.1 | DL087482.1 |
| DL086455.1 | DL086734.1 | HV543492.1 | HC010307.1 | BD234381.1 | HW307420.1 | GM654689.1 | DD147007.1 | DL087450.1 |
| DL086423.1 | DL086702.1 | HV546999.1 | HC009026.1 | BD232904.1 | HW294143.1 | GM654657.1 | DD138530.1 | DL087418.1 |
| DL115621.1 | DL086670.1 | HV539597.1 | HC007827.1 | BD232006.1 | HW293143.1 | GM654625.1 | DD138201.1 | DL112260.1 |
| DL115589.1 | DL086638.1 | HV539190.1 | HC007575.1 | BD231164.1 | DL023368.1 | GM654593.1 | DD132342.1 | DL112228.1 |
| DL115557.1 | DL086606.1 | HV538810.1 | HC010676.1 | BD231130.1 | DL023336.1 | GM654560.1 | CQ826740.1 | DL112196.1 |
| DL115525.1 | DL105879.1 | HV538576.1 | DM375814.1 | BD229096.1 | DL023304.1 | GM647567.1 | CQ821392.1 | DL112164.1 |
| DL115493.1 | DL105847.1 | HV538410.1 | HC005708.1 | BD227215.1 | DL023272.1 | GM640572.1 | CQ821222.1 | DL112132.1 |
| DL115461.1 | DL105815.1 | HV543341.1 | HC005031.1 | BD226908.1 | DL023240.1 | GM627896.1 | CQ819074.1 | DL112100.1 |
| DL115436.1 | CQ831871.1 | HV543216.1 | DM003488.1 | BD226753.1 | DL023208.1 | GM627864.1 | CQ817006.1 | DL112068.1 |
| DL115404.1 | CQ831497.1 | HV542746.1 | GM995275.1 | BD225782.1 | DL023176.1 | GM627832.1 | CQ816961.1 | DL107065.1 |
| DL115372.1 | CQ830723.1 | HV542714.1 | GN009990.1 | BD225750.1 | DL023173.1 | GM627800.1 | CQ816927.1 | DL107033.1 |
| DL115340.1 | CQ829830.1 | HV542177.1 | GN007454.1 | BD225718.1 | DL023141.1 | GM661654.1 | CQ815737.1 | DL107001.1 |
| DL110642.1 | CQ828079.1 | HV527880.1 | GM991943.1 | BD225686.1 | DL023109.1 | GM661622.1 | CQ814055.1 | DL106969.1 |
| DL110610.1 | CQ826848.1 | HV515150.1 | GM976375.1 | BD225654.1 | DL023077.1 | DL201954.1 | CQ814022.1 | DL106905.1 |
| DL110578.1 | CQ824423.1 | HV514901.1 | GM987029.1 | BD225622.1 | DL023045.1 | DL184470.1 | CQ813990.1 | DL102220.1 |
| DL105677.1 | CQ821289.1 | HV514659.1 | GN000755.1 | BD224830.1 | DL023013.1 | DL198988.1 | CQ813958.1 | DL102164.1 |
| DL105613.1 | CQ818941.1 | HH829710.1 | GN000378.1 | BD223248.1 | DL022981.1 | DL193915.1 | CQ813926.1 | DL102132.1 |

| | | | | | | | | |
|---|---|---|---|---|---|---|---|---|
| DL105581.1 | CQ818564.1 | FW394268.1 | GM981436.1 | BD222242.1 | DL010721.1 | DL196197.1 | CQ813894.1 | DL116904.1 |
| DL105517.1 | CQ817015.1 | FW394249.1 | GM974784.1 | BD221642.1 | DL010689.1 | DL193765.1 | CQ813862.1 | DL116872.1 |
| DL100904.1 | CQ816975.1 | FW394207.1 | GM984658.1 | BD194679.1 | DL010657.1 | DL183347.1 | CQ813830.1 | DL116840.1 |
| DL100872.1 | CQ816935.1 | FW398056.1 | FB985448.1 | DD216032.1 | DL010625.1 | DL183174.1 | CQ813798.1 | DL116812.1 |
| DL100840.1 | CQ815746.1 | FW397864.1 | FB740446.1 | DD216000.1 | DL042939.1 | FB571330.1 | CQ803123.1 | DL116780.1 |
| DL100808.1 | CQ814546.1 | FW393448.1 | GM596703.1 | DD215968.1 | DL042907.1 | CS803577.1 | CQ798788.1 | DL116748.1 |
| DL100776.1 | CQ814062.1 | FW392976.1 | GM970125.1 | DD215922.1 | DL042875.1 | CS696198.1 | CQ796831.1 | DL112059.1 |
| DL100744.1 | CQ814029.1 | FW396442.1 | FB987376.1 | DD215890.1 | DJ008048.1 | CS696166.1 | CQ795629.1 | DL112027.1 |
| DL096330.1 | CQ813997.1 | FW396313.1 | GM009575.1 | DD215826.1 | DJ004231.1 | CS696134.1 | DL106821.1 | DL111995.1 |
| DL096298.1 | CQ813965.1 | HH835189.1 | DL477929.1 | DD215794.1 | DD495502.1 | CS696102.1 | DL106789.1 | DL111963.1 |
| DL096266.1 | CQ813933.1 | HH833916.1 | DL477725.1 | DD215762.1 | DD465029.1 | CS696038.1 | DL091199.1 | DL111931.1 |
| DL096234.1 | CQ813901.1 | HH833671.1 | DL464629.1 | DD217814.1 | CS646228.1 | CS696005.1 | DL087231.1 | DL111899.1 |
| DL096202.1 | CQ813869.1 | HH833639.1 | DL476675.1 | CS254875.1 | CS646194.1 | CS695973.1 | DL087199.1 | DL106864.1 |
| DL096170.1 | CQ813837.1 | HH833607.1 | DL463061.1 | DD212303.1 | CS644242.1 | CS695941.1 | DL102092.1 | DL106832.1 |
| DL120190.1 | CQ813805.1 | HH833575.1 | DL462999.1 | DD209954.1 | DD460656.1 | CS695877.1 | DL102060.1 | DL106800.1 |
| DL120158.1 | CQ813732.1 | HH833543.1 | DL462899.1 | DD187261.1 | DD460508.1 | CS695845.1 | DL102028.1 | DL106768.1 |
| DL120126.1 | CQ812847.1 | HH833511.1 | DL461716.1 | DD206887.1 | DD455448.1 | CS695813.1 | DL114060.1 | DL106736.1 |
| DL120094.1 | CQ806528.1 | HH833479.1 | DL475552.1 | DD206855.1 | DD458983.1 | CS695781.1 | DL126232.1 | DL106704.1 |
| DL120062.1 | CQ802131.1 | HH833447.1 | GM664579.1 | DD206823.1 | DD453899.1 | CS695749.1 | DL126200.1 | DL091274.1 |
| DL120030.1 | CQ802027.1 | HH833415.1 | GM648734.1 | DD206793.1 | DD456724.1 | CS695717.1 | DL126168.1 | DL091242.1 |
| DL096142.1 | CQ800994.1 | HH833383.1 | GM648702.1 | CS249477.1 | DD456316.1 | CS695589.1 | DL122952.1 | DL091210.1 |
| DL094316.1 | CQ800793.1 | FW381690.1 | GM648670.1 | DD181022.1 | CS631719.1 | CS695557.1 | DL122920.1 | DL091178.1 |
| DL094284.1 | CQ798401.1 | HH928677.1 | GM648638.1 | CS102624.1 | CS631287.1 | CS695525.1 | DL113950.1 | DL087370.1 |
| DL094252.1 | CQ796852.1 | HH931886.1 | GM648606.1 | CS102592.1 | CS631208.1 | CS695429.1 | DL113918.1 | DL087338.1 |
| DL094220.1 | CQ796371.1 | HH821860.1 | GM634454.1 | CS102560.1 | CS642169.1 | CS695397.1 | DL113886.1 | DL087306.1 |
| DL094188.1 | CQ795454.1 | HH822332.1 | GM629467.1 | DJ380905.1 | CS640892.1 | DL176508.1 | DL113854.1 | DL087274.1 |
| DL094156.1 | CQ793259.1 | HH822015.1 | GM620107.1 | DJ380873.1 | CS627843.1 | DL176286.1 | DL109053.1 | DL087242.1 |
| DL090541.1 | CQ790260.1 | HC089260.1 | GM634054.1 | DJ380821.1 | CS627325.1 | DL164123.1 | DL109021.1 | DL087210.1 |
| DL090509.1 | CQ789540.1 | HC086184.1 | GM662967.1 | DJ379345.1 | CS632288.1 | DL176773.1 | DL108989.1 | CS368016.1 |
| DL090473.1 | CQ787485.1 | HC085660.1 | GM648350.1 | DJ379262.1 | CS632157.1 | FB355752.1 | DL104287.1 | DL076097.1 |
| HW154483.1 | CQ787453.1 | AY775005.1 | GM648318.1 | DJ389472.1 | CS623706.1 | FB355448.1 | DL104255.1 | DL075901.1 |
| HW154451.1 | CQ787413.1 | AY774948.1 | GM648286.1 | DJ388688.1 | CS623652.1 | FB344504.1 | DL104223.1 | DL075842.1 |
| HW147488.1 | CQ787373.1 | AY774908.1 | GM648254.1 | CS724935.1 | CS626368.1 | FB359888.1 | DL099585.1 | DL075810.1 |
| HW154352.1 | CQ787339.1 | AY774852.1 | GM648222.1 | CS724549.1 | CS625891.1 | DL088090.1 | DL122688.1 | DL075765.1 |
| HW147140.1 | CQ787305.1 | AY774736.1 | GM641387.1 | CS724410.1 | DD437996.1 | DL088058.1 | DL122656.1 | DL075577.1 |
| HW154022.1 | CQ787241.1 | AY774679.1 | GM641355.1 | CS723866.1 | DD451210.1 | DL088026.1 | DL122592.1 | DL075269.1 |
| HW153767.1 | CQ786937.1 | AY774622.1 | GM641323.1 | CS722278.1 | E04462.1 | DL087994.1 | DL099513.1 | DL079091.1 |
| HW146396.1 | CQ784725.1 | AY774554.1 | GM641291.1 | DJ353115.1 | E04181.1 | DL091935.1 | DL099481.1 | DL079051.1 |
| HW145785.1 | HW387369.1 | AY774507.1 | GM641259.1 | DJ357809.1 | E03188.1 | DL086669.1 | DL099449.1 | DL078967.1 |
| HW153477.1 | HW386239.1 | AY774451.1 | GM629068.1 | DJ357679.1 | E02775.1 | DL105878.1 | DL045008.1 | DL073102.1 |
| HW145520.1 | HW380893.1 | AY774337.1 | GM629036.1 | DJ357647.1 | E02329.1 | DL105846.1 | DL040768.1 | DL013609.1 |
| HW145141.1 | HW380816.1 | AY774287.1 | GM629004.1 | DJ357615.1 | E02191.1 | DL105814.1 | DL020806.1 | DL013577.1 |
| HW153267.1 | HW368274.1 | AF284212.1 | GM880089.1 | DJ357583.1 | E02042.1 | DL101009.1 | DL016403.1 | DL013545.1 |
| HW157438.1 | FW348835.1 | HC070442.1 | DL106885.1 | DJ363049.1 | E01550.1 | DL100977.1 | DL016371.1 | DL013513.1 |
| HW152011.1 | HW366468.1 | HC070102.1 | DL091228.1 | DJ344272.1 | E01348.1 | DL100945.1 | DL016339.1 | DL013481.1 |
| HW151767.1 | HW364613.1 | HC083728.1 | DL091196.1 | DJ361205.1 | E01214.1 | DL096531.1 | DL016307.1 | DL013449.1 |
| HW150674.1 | HW381043.1 | HC083551.1 | DL087228.1 | DJ354203.1 | E01023.1 | DL096499.1 | DL016275.1 | DL013417.1 |

| | | | | | | | | |
|---|---|---|---|---|---|---|---|---|
| HW150558.1 | HW353876.1 | HC069866.1 | DL087196.1 | DJ360419.1 | E00866.1 | GM642452.1 | DL016243.1 | DL013385.1 |
| HW150494.1 | HW352843.1 | HC060544.1 | DL101961.1 | DJ339852.1 | E00310.1 | GM642420.1 | DL011727.1 | DL018108.1 |
| HW159694.1 | HW352555.1 | AF301132.1 | DL101908.1 | DJ339820.1 | DD102377.1 | GM635206.1 | DL011695.1 | DL018076.1 |
| HW144669.1 | HW352427.1 | HC059758.1 | DL101876.1 | DJ339756.1 | DD088002.1 | GM635174.1 | DL011663.1 | DL018044.1 |
| HW144629.1 | HW341714.1 | HC059653.1 | DL101812.1 | DJ339724.1 | DD087789.1 | GM635142.1 | DL011631.1 | DL018012.1 |
| HW144521.1 | HW350681.1 | DJ045451.1 | DL101748.1 | DJ339660.1 | DD112895.1 | GM635110.1 | DL011599.1 | DL013261.1 |
| HW126642.1 | HW350647.1 | DJ050210.1 | DL095218.1 | CS457126.1 | DD099331.1 | GM635078.1 | DL048454.1 | DL013229.1 |
| HW126554.1 | HW349955.1 | DJ049444.1 | DL111860.1 | CS450596.1 | DD084814.1 | GM630272.1 | DL044741.1 | DL013197.1 |
| HW125529.1 | HW339481.1 | DJ048833.1 | DL111828.1 | DD359172.1 | DD070085.1 | GM630208.1 | DL044709.1 | DL017986.1 |
| HW144382.1 | HW348520.1 | CS793893.1 | DL111796.1 | DD357919.1 | DD084679.1 | GM630176.1 | DL005430.1 | DL017954.1 |
| HW144350.1 | HW347995.1 | CS800054.1 | DL122685.1 | DD357840.1 | DD152077.1 | GM737352.1 | DJ491582.1 | DL017922.1 |
| HW144286.1 | HW363646.1 | CS719261.1 | DL122653.1 | DD357618.1 | DD158407.1 | GM737103.1 | DJ446816.1 | DL017890.1 |
| HW124662.1 | HW363501.1 | CS798838.1 | DL122589.1 | DD357241.1 | DD158365.1 | GM635043.1 | DJ446424.1 | DL017858.1 |
| HW123667.1 | HW355441.1 | CS716770.1 | DL113811.1 | CS447936.1 | DD157348.1 | GM635011.1 | DJ436811.1 | DL017826.1 |
| HW123471.1 | HW317050.1 | CS706595.1 | DL113539.1 | A05964.1 | DD155706.1 | GM634979.1 | DJ444596.1 | DL017795.1 |
| HW122765.1 | HW326507.1 | CS716124.1 | DL092730.1 | A01613.1 | DD153641.1 | GM634947.1 | DJ434790.1 | DL017763.1 |
| HW122274.1 | HW338214.1 | CS805542.1 | DL092698.1 | CS443948.1 | DD152587.1 | GM634915.1 | DJ438346.1 | DL017731.1 |
| HV753826.1 | HW338086.1 | CS805312.1 | CQ878333.1 | CS443400.1 | DD151917.1 | GM634883.1 | DJ438279.1 | DL017699.1 |
| HV747477.1 | HW337830.1 | DD288251.1 | CQ874862.1 | CS441653.1 | DD157156.1 | GM629949.1 | DJ402672.1 | DL017667.1 |
| HV750785.1 | HW337190.1 | CS356777.1 | CQ868892.1 | CS438964.1 | DD096867.1 | GM629917.1 | DJ402602.1 | DL017635.1 |
| HV755987.1 | HW321898.1 | CS355963.1 | CQ867382.1 | DD346810.1 | DD082123.1 | GM642403.1 | DJ402285.1 | DL022273.1 |
| HV753488.1 | HW336806.1 | CS350540.1 | CQ859632.1 | DD349616.1 | DD081751.1 | GM642239.1 | DJ416208.1 | DL022241.1 |
| HV743901.1 | HW321643.1 | CS353181.1 | CQ857859.1 | CS433051.1 | DD149124.1 | GM642267.1 | DJ400827.1 | HC047364.1 |
| HV743869.1 | HW321119.1 | CS352409.1 | AX364668.1 | CS412187.1 | DD147999.1 | GM669913.1 | DJ400795.1 | HC047332.1 |
| HV743837.1 | HW329331.1 | CS283931.1 | AX364551.1 | CS416147.1 | DD147786.1 | GM656334.1 | DJ391344.1 | HC047300.1 |
| HV743805.1 | HW336165.1 | CS279308.1 | AX364519.1 | CS415723.1 | DD147754.1 | GM656302.1 | DJ417460.1 | HC047268.1 |
| HV743437.1 | HW335977.1 | CS273347.1 | AX364487.1 | CS415537.1 | DD147722.1 | GM656270.1 | DJ402749.1 | HC047044.1 |
| HV743313.1 | HW328745.1 | CS254953.1 | AX364455.1 | CS414837.1 | DD147690.1 | GM656206.1 | DJ381028.1 | HC047012.1 |
| HV743275.1 | HW328681.1 | CS252550.1 | AX364423.1 | DD320525.1 | DD147098.1 | GM656174.1 | DJ380996.1 | HC046980.1 |
| HV743236.1 | HW328490.1 | CS249833.1 | AX364359.1 | DD327195.1 | DD140006.1 | GM649181.1 | DD401620.1 | HC046948.1 |
| HV705233.1 | HW328451.1 | CS247229.1 | AX364264.1 | DD327024.1 | DD138754.1 | GM649149.1 | DD401588.1 | HC046916.1 |
| HV705066.1 | HW328353.1 | CS244160.1 | AX364232.1 | DD329897.1 | DD132350.1 | GM649117.1 | DD401556.1 | HC046888.1 |
| HV704453.1 | HW335656.1 | CS244208.1 | AX364200.1 | CS252551.1 | DD133851.1 | GM649085.1 | DD401524.1 | HC046856.1 |
| HV704421.1 | HW328208.1 | CS244636.1 | AX364167.1 | CS247244.1 | DD132329.1 | GM649053.1 | DD401492.1 | HC046824.1 |
| HV744597.1 | HW328089.1 | CS243167.1 | AX358338.1 | CS245380.1 | DD094900.1 | GM649021.1 | DD401460.1 | HC046792.1 |
| HV703532.1 | HW318493.1 | CS239694.1 | BD008860.1 | CS244161.1 | DD107447.1 | GM642154.1 | CS479866.1 | HC046760.1 |
| HV703090.1 | HW318259.1 | CS195877.1 | E59057.1 | CS244241.1 | DD107341.1 | GM642122.1 | CS468381.1 | HC046728.1 |
| HV702478.1 | HW335141.1 | CS227426.1 | BD002023.1 | CS244209.1 | AX528977.1 | GM642090.1 | CS466043.1 | HC046679.1 |
| HV702366.1 | HW314207.1 | CS227249.1 | E54594.1 | CS243269.1 | AX528945.1 | GM642058.1 | CS464532.1 | HC046647.1 |
| HV702264.1 | HW314032.1 | CS189622.1 | E59809.1 | CS243175.1 | HC731338.1 | GM642026.1 | DD400694.1 | HC046615.1 |
| HV702062.1 | HW312174.1 | CS193223.1 | AX354612.1 | CS195880.1 | HC471767.1 | GM629745.1 | DD400150.1 | HC046583.1 |
| HV701282.1 | HW312124.1 | DD213582.1 | AX351253.1 | CS227430.1 | HC471735.1 | GM629713.1 | DD361281.1 | HC046551.1 |
| HV701220.1 | HW311834.1 | DD213550.1 | AX347309.1 | CS227250.1 | HC471727.1 | GM736681.1 | DD367609.1 | HC046519.1 |
| HV701188.1 | HW311248.1 | DD213496.1 | AX347271.1 | CS206001.1 | HC471639.1 | GM886939.1 | DD363153.1 | HC046487.1 |
| HV701156.1 | AX346995.1 | DD220647.1 | AX347235.1 | CS189623.1 | HC471607.1 | GM669611.1 | DD362897.1 | HC046455.1 |
| HV701124.1 | AX345013.1 | DD220592.1 | AX347201.1 | CS188227.1 | HC729133.1 | GM669163.1 | DD368336.1 | HC046423.1 |
| HV708779.1 | AX343855.1 | DD218324.1 | AX347165.1 | CS177185.1 | HC729051.1 | GM656026.1 | DD368143.1 | HC046391.1 |

| | | | | | | | | |
|---|---|---|---|---|---|---|---|---|
| HV708528.1 | AX339639.1 | DD215920.1 | AX337947.1 | CS174653.1 | HC727622.1 | GM655994.1 | A10902.1 | HC046359.1 |
| HV708478.1 | AX339349.1 | DD215888.1 | AX328974.1 | CS172541.1 | HC689131.1 | GM655962.1 | CS457186.1 | HC046327.1 |
| HV701105.1 | AX338526.1 | DD215856.1 | AX328153.1 | CS172332.1 | HC688431.1 | GM648969.1 | CS456726.1 | HC046076.1 |
| HV701073.1 | AX329383.1 | DD215824.1 | AX306622.1 | CS166438.1 | HC490831.1 | GM648937.1 | CS456087.1 | HC046044.1 |
| HV701041.1 | AX328277.1 | DD215792.1 | AX306348.1 | CS159798.1 | HC490767.1 | GM648905.1 | CS453957.1 | HC046012.1 |
| HV695535.1 | AX319364.1 | DD215760.1 | AX268351.1 | CS159609.1 | HC490735.1 | GM648873.1 | DD360943.1 | HC046252.1 |
| HV695230.1 | AX317306.1 | DD211845.1 | AX258712.1 | CS159193.1 | HC490703.1 | GM648841.1 | DD360258.1 | HC046220.1 |
| HV695198.1 | AX306468.1 | DD192547.1 | AX286177.1 | CS158033.1 | HC490671.1 | GM648809.1 | DD357834.1 | HC046188.1 |
| HV694926.1 | AX266950.1 | DD192494.1 | AX283497.1 | CS150841.1 | HC490507.1 | DL095734.1 | DD357599.1 | HC046156.1 |
| HV694816.1 | AX258842.1 | DD211108.1 | AX280162.1 | CS150668.1 | HC490372.1 | DL095702.1 | DD355876.1 | HC046124.1 |
| HV700673.1 | AX300958.1 | DD210765.1 | HW241619.1 | CS144311.1 | HC490243.1 | DL095670.1 | CS447661.1 | HC046006.1 |
| HV694438.1 | AX286671.1 | DD208184.1 | HW243080.1 | CS142052.1 | HC472297.1 | DL095638.1 | A19554.1 | HC045974.1 |
| HV700634.1 | AX283149.1 | DD187255.1 | HW241414.1 | CS141764.1 | HC472264.1 | DL095606.1 | A12246.1 | HC045942.1 |
| HV700351.1 | AX282315.1 | DD206853.1 | HW161084.1 | CS141524.1 | HC472200.1 | DL095574.1 | A08468.1 | HC045878.1 |
| HV700227.1 | AX281530.1 | DD206821.1 | HW160402.1 | CS141492.1 | HC472168.1 | DL117411.1 | M29304.1 | HC045846.1 |
| HV693637.1 | AX279940.1 | DD206791.1 | HW155790.1 | CS140823.1 | HC471968.1 | DL117379.1 | K02913.1 | HC045814.1 |
| HV218607.1 | AX279608.1 | DD163415.1 | HW155769.1 | CS140531.1 | HC471936.1 | DL117347.1 | M67488.1 | HC045782.1 |
| HV227969.1 | AX278068.1 | CQ787513.1 | HW155703.1 | CS132436.1 | HC471904.1 | DL117315.1 | M60110.1 | HC045718.1 |
| HV218464.1 | AX256075.1 | CQ787481.1 | HW155671.1 | CS123428.1 | HC471872.1 | DL117283.1 | M19563.1 | HC045688.1 |
| HV214868.1 | AX253478.1 | CQ787449.1 | HW155501.1 | CS122955.1 | HC678831.1 | DL117251.1 | LT908470.1 | HC045656.1 |
| HV236020.1 | AX252300.1 | CQ787409.1 | HW155435.1 | CS122467.1 | HC678766.1 | DL117219.1 | AH002287.2 | HC045592.1 |
| HV301810.1 | AX247551.1 | CQ787369.1 | HW158439.1 | CS106169.1 | HC509354.1 | DL091638.1 | AH003155.2 | HC045560.1 |
| HV301751.1 | AX242323.1 | CQ787335.1 | HW154713.1 | CS106076.1 | HC508171.1 | DL091606.1 | M35072.1 | HC045528.1 |
| HV301617.1 | AX242291.1 | CQ787269.1 | HW154495.1 | CS179728.1 | HC504634.1 | DL091574.1 | K01301.1 | DM379016.1 |
| HV301585.1 | AX242195.1 | CQ787237.1 | HW158106.1 | CS179562.1 | HC494415.1 | DL087963.1 | DQ250232.1 | HC035673.1 |
| HV306188.1 | AX242163.1 | CQ786933.1 | HW101673.1 | DD163418.1 | HC504265.1 | DL087931.1 | DQ229852.1 | HC037225.1 |
| HV307806.1 | AX242131.1 | CQ784717.1 | HW096983.1 | DD181021.1 | GN363013.1 | DL121762.1 | AF191636.1 | HC037055.1 |
| HV302950.1 | AX242099.1 | CQ784653.1 | HW096675.1 | DD161458.1 | GN346505.1 | DL121730.1 | AY271256.1 | HC022644.1 |
| HV302731.1 | AX241971.1 | CQ784467.1 | HW072893.1 | DD167019.1 | GN346471.1 | DL121698.1 | AF283515.1 | HC025506.1 |
| HV312747.1 | AX241939.1 | CQ772686.1 | HW096202.1 | DD173103.1 | GN342144.1 | DL121666.1 | HW154838.1 | HC025474.1 |
| HV308870.1 | HW155716.1 | CQ771653.1 | HW104359.1 | DD172257.1 | GN346714.1 | DL121634.1 | HW158460.1 | HC025442.1 |
| HV305755.1 | HW155679.1 | CQ771621.1 | HW071113.1 | HI415026.1 | GN346578.1 | DL121602.1 | HW154717.1 | HC024897.1 |
| HV308547.1 | HW155605.1 | CQ771589.1 | HW070910.1 | HI414036.1 | GN346546.1 | DL087775.1 | HW154498.1 | HC010352.1 |
| HV311029.1 | HW144775.1 | CQ768276.1 | HW099520.1 | HI413999.1 | GN337984.1 | DL087743.1 | HW154466.1 | HC010182.1 |
| HV317242.1 | HW158474.1 | CQ756619.1 | HW068959.1 | HI413595.1 | GN131005.1 | DL087711.1 | HW154371.1 | HC009868.1 |
| HV226859.1 | HW154725.1 | CQ754502.1 | HW083249.1 | HI413440.1 | GN131885.1 | DL087647.1 | HW158129.1 | HC008392.1 |
| CQ868287.1 | HW154638.1 | CQ754035.1 | HW083217.1 | FW379640.1 | GN116525.1 | DL087615.1 | HW154015.1 | HC020914.1 |
| CQ866990.1 | HW154471.1 | AB105218.1 | HW088227.1 | FW379608.1 | GN116493.1 | DL128935.1 | HW153782.1 | HC007776.1 |
| CQ861213.1 | HW154378.1 | AX963133.1 | HW099480.1 | FW379262.1 | GN116460.1 | DL125450.1 | HW153584.1 | HC007680.1 |
| CQ859648.1 | HW158134.1 | AX962031.1 | HW084541.1 | FW376133.1 | GN116428.1 | DL125418.1 | HW157487.1 | HC007648.1 |
| CQ859616.1 | HW154104.1 | AX959227.1 | HW102824.1 | FW375463.1 | GN116396.1 | DL125386.1 | HW151797.1 | HC007588.1 |
| CQ858164.1 | HW153787.1 | AX958066.1 | HW102759.1 | FW375237.1 | GN116364.1 | DL125354.1 | HW151066.1 | HC007556.1 |
| CQ857838.1 | HW153706.1 | AX958010.1 | HW089278.1 | FW380417.1 | GN116332.1 | DL121533.1 | HW155983.1 | HC007524.1 |
| CQ855930.1 | HW157492.1 | AX952716.1 | HV549106.1 | FW380256.1 | GN116300.1 | DL121501.1 | HW144720.1 | HC010737.1 |
| CQ855752.1 | HW160336.1 | AX937803.1 | HV549062.1 | FW379806.1 | GN115064.1 | DL121469.1 | HW125396.1 | DM376696.1 |
| CQ854369.1 | HW151824.1 | AX931833.1 | HV548887.1 | HH736086.1 | GN094507.1 | DL121437.1 | HW144365.1 | DM375481.1 |
| CQ849509.1 | HW151510.1 | AX923403.1 | HV547730.1 | HH735912.1 | GN094475.1 | DL121405.1 | HW144333.1 | DM091322.1 |

| | | | | | | | | |
|---|---|---|---|---|---|---|---|---|
| CQ849413.1 | HW159796.1 | AX923369.1 | HV543476.1 | HH733847.1 | DM073828.1 | DL117193.1 | HW144301.1 | DM068815.1 |
| CQ848442.1 | HV944932.1 | AX826927.1 | HV538645.1 | HH733365.1 | DM078719.1 | DL117161.1 | HW124785.1 | GN087804.1 |
| CQ840577.1 | HV931349.1 | AX825029.1 | HV538538.1 | FW363725.1 | DM077579.1 | DL117129.1 | HW122423.1 | GN091068.1 |
| CQ832050.1 | HV931261.1 | AX824440.1 | HV543200.1 | FW363523.1 | GM638139.1 | DL112317.1 | HV767063.1 | GN087203.1 |
| CQ831678.1 | HV936385.1 | A15643.1 | HV541512.1 | FW362226.1 | GM638107.1 | DL112285.1 | HV766845.1 | GN089864.1 |
| CQ830726.1 | HV930750.1 | A11139.1 | HV541333.1 | HC490508.1 | GM638075.1 | DL107271.1 | HV760599.1 | GN089800.1 |
| CQ826860.1 | HV819644.1 | A09216.1 | HV533391.1 | HC490405.1 | GM633671.1 | BD271043.1 | HV766281.1 | GN089197.1 |
| CQ821630.1 | HV819463.1 | A08832.1 | HV535828.1 | HC490373.1 | GM644960.1 | DD231224.1 | HV766063.1 | GN082824.1 |
| CQ818946.1 | HV803280.1 | A07753.1 | HV537390.1 | HC490244.1 | GM633337.1 | DD230447.1 | HV766031.1 | GN088987.1 |
| CQ818567.1 | HV932933.1 | A06407.1 | HV515987.1 | HC472298.1 | GM633305.1 | DD228421.1 | HV750174.1 | GN081978.1 |
| CQ817735.1 | HV932686.1 | A05981.1 | HV515601.1 | HC472265.1 | GM633273.1 | DD227771.1 | HV757227.1 | DL232989.1 |
| CQ816979.1 | HV932654.1 | HC358217.1 | HV515569.1 | HC472233.1 | GM633250.1 | DD227385.1 | HV758672.1 | L08949.1 |
| CQ816938.1 | HV802938.1 | HC358086.1 | HV515441.1 | HC472201.1 | GM633218.1 | DD225221.1 | HV745891.1 | DM059625.1 |
| CQ815749.1 | HV802906.1 | HC358054.1 | HV515409.1 | HC472169.1 | GM633186.1 | DD224657.1 | HV745362.1 | DM058784.1 |
| CQ814959.1 | HV818563.1 | HC435692.1 | HV515377.1 | HC471969.1 | GM633154.1 | DD231577.1 | HV753627.1 | DM056944.1 |
| CQ814065.1 | HV817906.1 | A00972.1 | HV515345.1 | HC471937.1 | GM633122.1 | DD231481.1 | HV753522.1 | DM045534.1 |
| CQ814032.1 | HV542606.1 | HC324495.1 | HV515313.1 | HC471905.1 | GM659178.1 | DD231441.1 | HV753408.1 | DM045430.1 |
| CQ814000.1 | HV541775.1 | HC319209.1 | HV515281.1 | HC490533.1 | GM659127.1 | DD231409.1 | HV743217.1 | DM045251.1 |
| CQ813968.1 | HV536626.1 | HC318709.1 | HV515249.1 | HC678768.1 | GM651775.1 | CS276973.1 | HV704742.1 | DM044973.1 |
| CQ813936.1 | HV536504.1 | HC317599.1 | HV511459.1 | HC677956.1 | GM644783.1 | A00338.1 | HV704364.1 | DM039469.1 |
| CQ813904.1 | HV536209.1 | HC310058.1 | HV514263.1 | HC509356.1 | GM644751.1 | DD219619.1 | HV704164.1 | DM055894.1 |
| CQ813872.1 | HV530205.1 | HC310026.1 | HV507914.1 | HC504849.1 | GM644719.1 | DD214019.1 | HV703990.1 | DM044669.1 |
| CQ813840.1 | HV515731.1 | HC309994.1 | HV508676.1 | HC494416.1 | GM633057.1 | DD216634.1 | HV702427.1 | DM060742.1 |
| CQ813808.1 | HV515577.1 | HC309962.1 | BD130719.1 | HC504266.1 | GM625190.1 | DD213909.1 | HV701404.1 | DM060710.1 |
| CQ813735.1 | HV515545.1 | HC307842.1 | BD130687.1 | HC499861.1 | GM625158.1 | DD213877.1 | HV701235.1 | DM055602.1 |
| AX347289.1 | HV515513.1 | HC307791.1 | BD130585.1 | HC488031.1 | GM624036.1 | DD213813.1 | HV701203.1 | GN075487.1 |
| AX347251.1 | HV515481.1 | FU262991.1 | BD107025.1 | HC485947.1 | GM624004.1 | DD216446.1 | HV701171.1 | GN067861.1 |
| AX347219.1 | HV515417.1 | FU262781.1 | HW380922.1 | HC484163.1 | GM623972.1 | DD213796.1 | HV701139.1 | GM652321.1 |
| AX347181.1 | HV515385.1 | FU259869.1 | HW368183.1 | HC475379.1 | GM623940.1 | DD213764.1 | HV708136.1 | GM652289.1 |
| AX347093.1 | HV515353.1 | FU265400.1 | HW381363.1 | HC466602.1 | GM650118.1 | DD213732.1 | HV701088.1 | GM652257.1 |
| AX345345.1 | HV515321.1 | FU264496.1 | HW317126.1 | HC474194.1 | GM650086.1 | DD213700.1 | HV695518.1 | GM645394.1 |
| AX342197.1 | HV515289.1 | DM187707.1 | HW326331.1 | HC491801.1 | GM643085.1 | DD213668.1 | HV695422.1 | GM645362.1 |
| AX338572.1 | HV515257.1 | HB559477.1 | HW338914.1 | HC491789.1 | GM643053.1 | DD213636.1 | HV694799.1 | GM645330.1 |
| AX328285.1 | HV511155.1 | HB491703.1 | HW338658.1 | HC491757.1 | GM643021.1 | DD213604.1 | HV700807.1 | GM645266.1 |
| AX327960.1 | HV510348.1 | DM164070.1 | HW338274.1 | HC491725.1 | GM635872.1 | DD213586.1 | HV694667.1 | GM645234.1 |
| AX323385.1 | HV510283.1 | DM164002.1 | HW338146.1 | HC089452.1 | GM622212.1 | DD213554.1 | HV208644.1 | GM659380.1 |
| AX322067.1 | HV509349.1 | DM163969.1 | HW337506.1 | HC089420.1 | GM622180.1 | DD220596.1 | HV306171.1 | GM659348.1 |
| AX319614.1 | HV508684.1 | DM163300.1 | HW337122.1 | HC089387.1 | GM622148.1 | DD216034.1 | HV302965.1 | GM652156.1 |
| AX317543.1 | HV508585.1 | DM163255.1 | HW321829.1 | HC089355.1 | FB506782.1 | DD216002.1 | HV302933.1 | GM652124.1 |
| AX317444.1 | HV508553.1 | DM162312.1 | HW336866.1 | HC089322.1 | GM642993.1 | DD215970.1 | HV305822.1 | GM652060.1 |
| AX306762.1 | HV508521.1 | HB477358.1 | HW336502.1 | HC089290.1 | GM642960.1 | DD218335.1 | HV308612.1 | GM652028.1 |
| AX305186.1 | HV508493.1 | HB486503.1 | HW329272.1 | HC089258.1 | GM642928.1 | DD215924.1 | HV308519.1 | GM645201.1 |
| AX268679.1 | HV508461.1 | HB474907.1 | HW335994.1 | AY775003.1 | GM630652.1 | DD215892.1 | HV304160.1 | GM645169.1 |
| AX258897.1 | HV512568.1 | HB469145.1 | HW335959.1 | AY774945.1 | GM630620.1 | AX709452.1 | HV247049.1 | GM645137.1 |
| AX304311.1 | HV512295.1 | HB468028.1 | HW328832.1 | AY774905.1 | GM630588.1 | AX699462.1 | HV235772.1 | GM645105.1 |
| AX299853.1 | HV512263.1 | GN094474.1 | HW328728.1 | AY774846.1 | DL104323.1 | AX699430.1 | HV305482.1 | GM645073.1 |
| AX297655.1 | HV512199.1 | DM078718.1 | HW328633.1 | AY774783.1 | DL093611.1 | AX698626.1 | HV040105.1 | GM645041.1 |

| | | | | | | | | |
|---|---|---|---|---|---|---|---|---|
| AX287060.1 | HV512167.1 | GN090932.1 | HW328420.1 | AY774731.1 | DL123160.1 | AX675276.1 | HV188416.1 | GM638242.1 |
| AX283689.1 | HV512135.1 | GN087187.1 | HW319183.1 | AY774550.1 | DL123128.1 | AX670752.1 | HV200231.1 | GM638210.1 |
| AX283223.1 | HV511882.1 | GN089992.1 | HW335783.1 | AY774448.1 | DL118682.1 | AX670720.1 | HV194344.1 | GM638178.1 |
| AX282873.1 | GN010292.1 | GN089848.1 | HW328260.1 | AY774385.1 | DL118650.1 | AX665427.1 | HV182448.1 | GM638146.1 |
| AX282189.1 | GN033543.1 | GN082816.1 | HW328108.1 | AY774332.1 | DL118618.1 | AX664344.1 | HV182416.1 | GM638114.1 |
| AX279951.1 | GN033511.1 | GN088786.1 | HW318557.1 | AY774285.1 | DL114222.1 | AX662225.1 | HV182384.1 | GM638082.1 |
| AX279716.1 | GN033479.1 | GN081961.1 | HW335587.1 | AY774168.1 | DL114190.1 | AX662045.1 | HV117715.1 | GM651894.1 |
| AX278754.1 | GN033447.1 | GN088033.1 | HW335167.1 | AY774115.1 | DL114158.1 | AX657144.1 | FW505271.1 | GM651862.1 |
| AX278088.1 | GN033415.1 | DL233087.1 | HW314338.1 | AY774042.1 | DL114126.1 | AX657112.1 | FW508143.1 | GM651830.1 |
| AX254645.1 | GN033351.1 | L08940.1 | HW314119.1 | AY645669.1 | DL114094.1 | AX657068.1 | FW508091.1 | GM651798.1 |
| AX253008.1 | GN033319.1 | L08860.1 | HW311869.1 | K01699.1 | DL114062.1 | BD174854.1 | FW562443.1 | GM644999.1 |
| AX251507.1 | GN033287.1 | M94409.1 | HW311229.1 | AF298593.1 | DL137272.1 | BD174664.1 | FW553148.1 | GM638040.1 |
| AX242331.1 | GN033255.1 | DM059614.1 | HW308454.1 | HC070100.1 | DL126202.1 | AX648079.1 | FW556607.1 | GM637976.1 |
| AX242299.1 | GN033159.1 | DM063455.1 | HW307883.1 | HC083726.1 | DL126170.1 | AX643976.1 | FW560795.1 | GM637944.1 |
| AX242235.1 | GN033063.1 | DM058829.1 | HW307851.1 | HC083549.1 | DL122954.1 | AX642219.1 | FW506002.1 | GM637912.1 |
| AX242203.1 | GN033031.1 | GM659894.1 | HW307819.1 | HC083517.1 | DL122922.1 | AX622935.1 | FW505339.1 | GM637880.1 |
| AX242171.1 | GN032999.1 | GM659862.1 | HW307787.1 | HC069790.1 | DL039446.1 | AX616535.1 | HI931500.1 | GM637848.1 |
| AX242107.1 | GN032967.1 | GM659830.1 | HW307749.1 | DM381045.1 | DL039318.1 | BD172039.1 | HI935089.1 | GM633440.1 |
| AX242075.1 | GN032935.1 | GM659798.1 | HW307717.1 | AF080093.1 | DL035694.1 | AX601323.1 | HI918255.1 | GM633408.1 |
| AX242043.1 | GN032903.1 | GM659766.1 | HW307685.1 | HC058622.1 | DL035662.1 | AX599026.1 | HI660828.1 | GM633376.1 |
| AX241979.1 | GN032871.1 | GM659734.1 | HW315863.1 | HC057873.1 | DL035630.1 | AX598768.1 | CS583651.1 | GM633344.1 |
| AX241947.1 | GN032839.1 | GM652345.1 | HW315654.1 | HC057614.1 | DL023557.1 | AX598230.1 | CS574810.1 | GM633312.1 |
| AX241915.1 | GN032807.1 | GM652313.1 | HW315544.1 | DM459786.1 | DL023525.1 | AX594243.1 | CS573061.1 | GM633280.1 |
| AX241883.1 | GN032775.1 | GM652281.1 | HV961474.1 | DM381532.1 | DL023493.1 | AX593521.1 | CS544006.1 | GM637825.1 |
| AX241851.1 | GN032744.1 | GM645386.1 | HV964073.1 | HC051934.1 | DL023461.1 | AX587846.1 | CS570715.1 | GM633257.1 |
| AX241819.1 | GN032712.1 | GM645354.1 | HV958810.1 | HC050940.1 | DL023429.1 | AX589673.1 | CS546683.1 | GM633225.1 |
| AX241755.1 | GN032680.1 | GM645322.1 | HV963619.1 | HC049737.1 | DL023397.1 | BD161073.1 | DD147776.1 | GM633193.1 |
| AX241723.1 | GN032648.1 | GM645290.1 | HV963544.1 | HC045498.1 | DL020382.1 | BD160754.1 | DD147744.1 | GM633161.1 |
| AX241691.1 | GN032615.1 | GM645258.1 | HV963329.1 | HC045466.1 | DL020350.1 | AX145730.1 | DD147712.1 | GM633129.1 |
| AX241659.1 | GN032583.1 | GM645226.1 | HV969830.1 | GN360049.1 | DL020318.1 | AX145698.1 | DD147680.1 | GM633097.1 |
| AX241627.1 | GN032552.1 | GM678620.1 | HV956044.1 | GN360017.1 | DL015819.1 | AX145666.1 | DD138008.1 | GM625390.1 |
| AX241595.1 | GN032520.1 | GM652148.1 | HV947304.1 | GN359953.1 | DL015787.1 | AX145634.1 | DD135981.1 | GM625358.1 |
| AX241563.1 | GN032488.1 | GM645193.1 | HV947135.1 | GN359889.1 | DL015755.1 | AX145570.1 | DD132319.1 | GM644811.1 |
| AX241531.1 | GN032456.1 | GM645161.1 | HV502800.1 | GN366315.1 | DL043071.1 | AX145538.1 | DD093002.1 | GM711123.1 |
| AX241499.1 | GN032392.1 | GM645129.1 | HV502768.1 | GN366283.1 | DL035406.1 | AX145506.1 | DD092933.1 | GM644790.1 |
| AX241467.1 | GN032359.1 | GM645097.1 | HV502736.1 | GN359664.1 | DL035374.1 | AX145474.1 | DD091009.1 | GM644758.1 |
| AX241435.1 | GN032295.1 | GM645065.1 | HV502704.1 | GN359536.1 | DL035342.1 | AX145442.1 | DD117029.1 | GM644726.1 |
| AX241128.1 | GN032263.1 | GM645033.1 | HV502672.1 | GM646849.1 | DL031178.1 | AX145410.1 | DD089922.1 | GM649192.1 |
| AX241096.1 | GN032231.1 | GM638074.1 | HV502640.1 | GM646817.1 | DL023365.1 | AX145377.1 | DD089298.1 | GM642583.1 |
| AX241064.1 | GN032199.1 | GM633670.1 | HV502608.1 | GM640014.1 | DL023333.1 | AX145345.1 | DD102591.1 | GM642551.1 |
| AX241032.1 | GN032167.1 | GM633336.1 | HV502544.1 | GM639982.1 | DL023301.1 | AX145313.1 | DD057928.1 | GM642454.1 |
| AX241000.1 | GN032135.1 | GM633304.1 | HV502512.1 | GM639950.1 | DL023269.1 | AX145281.1 | DD057896.1 | GM642422.1 |
| CS174658.1 | GN032103.1 | GM633249.1 | HV502448.1 | GM639918.1 | DL023237.1 | AX145217.1 | DD057864.1 | GM635240.1 |
| CS174535.1 | GN032071.1 | GM633217.1 | HV492731.1 | GM627280.1 | DL023205.1 | AX145185.1 | DD057836.1 | GM635208.1 |
| CS174659.1 | GN032039.1 | GM633185.1 | HV505360.1 | GM627248.1 | DD460649.1 | AX145153.1 | DD057050.1 | GM635176.1 |
| CS166443.1 | GN032007.1 | GM633153.1 | HV451988.1 | GM627216.1 | DD460139.1 | AX145121.1 | DD053292.1 | GM635112.1 |
| CS095707.1 | GN031975.1 | GM633121.1 | HV449934.1 | GM627184.1 | DD458776.1 | AX145089.1 | DD051871.1 | GM635080.1 |

| | | | | | | | | |
|---|---|---|---|---|---|---|---|---|
| CS091383.1 | GN031943.1 | GM633089.1 | HV451140.1 | GM627152.1 | DD453894.1 | AX145057.1 | DD041897.1 | GM630274.1 |
| CS085919.1 | GN031911.1 | GM884067.1 | HV444026.1 | GM660976.1 | DD456716.1 | AX145025.1 | DD038505.1 | GM630210.1 |
| CS083134.1 | GN031879.1 | GM648355.1 | HV341433.1 | GM660944.1 | CS631281.1 | AX144993.1 | DD172707.1 | GM630178.1 |
| CS082411.1 | GN031847.1 | GM648323.1 | HV344896.1 | GM646749.1 | CS631202.1 | AX144961.1 | DD165471.1 | GM737354.1 |
| CS082347.1 | GN031815.1 | GM648291.1 | HI973432.1 | GM646717.1 | CS631138.1 | AX144929.1 | DD163902.1 | GM698655.1 |
| CS080378.1 | GN031782.1 | GM648259.1 | HI988452.1 | GM646685.1 | CS643943.1 | AX144896.1 | CS001103.1 | GM698601.1 |
| CS078830.1 | GN031750.1 | GM648227.1 | HI987449.1 | GM646653.1 | CS623769.1 | AX144832.1 | CS188235.1 | GM698559.1 |
| CS078159.1 | GN031717.1 | GM648195.1 | FW508930.1 | GM646621.1 | CS623697.1 | AX144800.1 | CS172889.1 | GM635045.1 |
| CS073679.1 | GN031685.1 | GM641360.1 | FW555580.1 | GM639818.1 | CS626355.1 | AX144768.1 | CS157816.1 | GM635013.1 |
| CS070533.1 | GN031653.1 | GM641328.1 | FW561682.1 | GM639786.1 | DD437993.1 | AX144736.1 | CS157945.1 | GM634981.1 |
| CS068667.1 | GN031621.1 | GM641296.1 | FW561650.1 | GM639754.1 | DD449083.1 | AX144704.1 | CS157913.1 | GM634949.1 |
| CS064606.1 | GN031589.1 | GM641264.1 | FW562436.1 | DL115781.1 | DD436071.1 | AX144672.1 | CS157881.1 | GM634917.1 |
| CS063840.1 | GN031557.1 | GM641232.1 | FW553141.1 | DL115749.1 | CS620304.1 | HV601499.1 | CS124702.1 | GM634885.1 |
| CS061196.1 | GN031525.1 | GM629073.1 | FW555214.1 | DL115717.1 | CS616492.1 | HV581981.1 | CS119858.1 | GM629951.1 |
| AY967386.1 | GN031461.1 | GM629041.1 | FW367491.1 | DL115685.1 | CS614440.1 | HV601356.1 | CS119530.1 | GM629919.1 |
| AY967354.1 | GN031428.1 | GM629009.1 | FW367282.1 | DL115653.1 | CS613382.1 | HV601078.1 | CS119497.1 | GM642405.1 |
| AY967322.1 | GN031396.1 | GM648162.1 | FW351371.1 | DL111195.1 | CS612781.1 | HV574872.1 | CS119430.1 | GM642237.1 |
| AY967290.1 | GN031364.1 | CS118944.1 | FW361289.1 | DL106092.1 | CS611829.1 | FW572718.1 | CQ758866.1 | GM642269.1 |
| AY967258.1 | GN031332.1 | CS118912.1 | FW345545.1 | DL106060.1 | CS604539.1 | FW573833.1 | CQ755437.1 | GM697213.1 |
| AY967226.1 | GN031300.1 | CS118878.1 | FW344922.1 | DL106028.1 | CS604507.1 | FW574620.1 | CQ754026.1 | GM656336.1 |
| AY967194.1 | GN031267.1 | CS118846.1 | FW343706.1 | DL105996.1 | CS604475.1 | FW574375.1 | AX962916.1 | GM656304.1 |
| AY967162.1 | FB713790.1 | CS118812.1 | FW343640.1 | DL105964.1 | CS604411.1 | FW571751.1 | AX958974.1 | GM656272.1 |
| AY967130.1 | FB708464.1 | CS118715.1 | FW304277.1 | DL105932.1 | CS604379.1 | HI968134.1 | AX937793.1 | GM656208.1 |
| AY967098.1 | FB667089.1 | CS118683.1 | HC487995.1 | DL105900.1 | CS604347.1 | HI967418.1 | AX923393.1 | GM656176.1 |
| AY967066.1 | FB666656.1 | CS118651.1 | HC474180.1 | DL101191.1 | CS604315.1 | HI989139.1 | AX840257.1 | GM649183.1 |
| AY967034.1 | CS721882.1 | CS118586.1 | HC491775.1 | DL101159.1 | CS604283.1 | HI988920.1 | AX838388.1 | GM649151.1 |
| AY967002.1 | FB676564.1 | CS118551.1 | HC491743.1 | DL101127.1 | CS604187.1 | HI987429.1 | AX824357.1 | GM649119.1 |
| AY966970.1 | FB676500.1 | CS118519.1 | HC491070.1 | DL096713.1 | CS604059.1 | FW552167.1 | AX823791.1 | GM649087.1 |
| AY966938.1 | FB705960.1 | CS118485.1 | HC488159.1 | DL096681.1 | DD084743.1 | FW552130.1 | AX823757.1 | GM649055.1 |
| CS057815.1 | FB701549.1 | CS118453.1 | HH999428.1 | DL096649.1 | DD112541.1 | FW554623.1 | AX814395.1 | GM649023.1 |
| CS055389.1 | FB701822.1 | CS118421.1 | HH999380.1 | DL096617.1 | DD084629.1 | FW555596.1 | AX801191.1 | GM648991.1 |
| CS052353.1 | FB660482.1 | CS118388.1 | HH997668.1 | DL096585.1 | DD069504.1 | HI284300.1 | AX573484.1 | GM642156.1 |
| CS050984.1 | FB665210.1 | CS118321.1 | HH997630.1 | DL096553.1 | DD098150.1 | HI571688.1 | AX556844.1 | GM642124.1 |
| CS048824.1 | FB654413.1 | CS118287.1 | HH997573.1 | DL094725.1 | DD157377.1 | HI574521.1 | AX556810.1 | GM642092.1 |
| CS047669.1 | FB583258.1 | CS118223.1 | HH997498.1 | DL094693.1 | DD155703.1 | HI574457.1 | AX537318.1 | AX364182.1 |
| CQ972367.1 | FB573884.1 | CS118156.1 | HH997359.1 | DL090950.1 | DD152993.1 | HI574425.1 | AX530413.1 | AX363236.1 |
| CQ972335.1 | FB573330.1 | CS118124.1 | HH999224.1 | DL090918.1 | DD152584.1 | HI574393.1 | AX528451.1 | AX359928.1 |
| CQ971805.1 | FB573974.1 | CS118092.1 | HH999180.1 | DL086982.1 | DD151914.1 | HI574370.1 | AX525429.1 | AX358643.1 |
| CQ971134.1 | DL199982.1 | CS118059.1 | HH999135.1 | DL086950.1 | DD097298.1 | HI581357.1 | A30450.1 | AX358424.1 |
| CQ971069.1 | DL199781.1 | CS117989.1 | HH999096.1 | DL086918.1 | DD096864.1 | HI553733.1 | A33911.1 | BD006785.1 |
| CQ970883.1 | DL199749.1 | CS117957.1 | HH999055.1 | DL086886.1 | DD082113.1 | HI593059.1 | AX179526.1 | E50937.1 |
| CQ969079.1 | DL191603.1 | CS106393.1 | HH998994.1 | DL086854.1 | DD081748.1 | HI637149.1 | AX179464.1 | E63241.1 |
| CQ963552.1 | DL196190.1 | CS103272.1 | HH998947.1 | DL086790.1 | DD149116.1 | HI636966.1 | AX164094.1 | BD001999.1 |
| CQ947126.1 | DL193822.1 | CS102937.1 | HH997220.1 | DL086758.1 | DD147877.1 | HI001447.1 | A20355.1 | E54558.1 |
| CQ944196.1 | DL193790.1 | CS102905.1 | HH970259.1 | DL086726.1 | DD147783.1 | HI001386.1 | AF430212.1 | E55380.1 |
| CQ944132.1 | DL193758.1 | CS102841.1 | HH998894.1 | DL086694.1 | DD147751.1 | HI001351.1 | AF430180.1 | BD000349.1 |
| CQ944100.1 | DL183799.1 | CS102745.1 | HH998833.1 | DL086662.1 | DD147719.1 | HI003200.1 | AX505185.1 | BD000188.1 |

| | | | | | | | | |
|---|---|---|---|---|---|---|---|---|
| CQ944068.1 | FB511515.1 | CS102713.1 | HH998790.1 | DL105871.1 | DD147095.1 | HI003138.1 | AX498155.1 | AX356507.1 |
| CQ944036.1 | DL110737.1 | CS102649.1 | HH998731.1 | DL105839.1 | DD138210.1 | HI003081.1 | AX496847.1 | AX354684.1 |
| CQ944004.1 | DL110705.1 | CS102585.1 | HH997076.1 | DL105807.1 | DD132343.1 | HI003042.1 | BD138729.1 | AX353940.1 |
| CQ943972.1 | DL110673.1 | CS102553.1 | HH997022.1 | DL096524.1 | DD135260.1 | HI003001.1 | BD137873.1 | AX352742.1 |
| CQ943940.1 | DL115512.1 | CS102521.1 | HH996968.1 | DL096492.1 | CQ787296.1 | HI002939.1 | BD136039.1 | AX352350.1 |
| CQ943908.1 | DL115480.1 | CS102489.1 | HH981092.1 | DL096460.1 | CQ787264.1 | HI002902.1 | BD135124.1 | AX351209.1 |
| CQ943876.1 | DL115448.1 | CS088912.1 | GM709023.1 | DL075252.1 | CQ787232.1 | HI001265.1 | BD134637.1 | AX351099.1 |
| CQ918563.1 | DL105696.1 | BD016721.1 | GM708971.1 | DL079074.1 | CQ784707.1 | HI001209.1 | BD133522.1 | AX349342.1 |
| A18005.1 | DL105664.1 | BD014475.1 | GM713275.1 | DL079042.1 | CQ784643.1 | HI001154.1 | BD131928.1 | AX349119.1 |
| CQ898782.1 | DM026245.1 | BD014220.1 | GM833979.1 | DL078958.1 | CQ778526.1 | HI001056.1 | BD130821.1 | AX348944.1 |
| CQ898650.1 | GN043980.1 | BD014185.1 | FB506763.1 | DL074254.1 | CQ778494.1 | HI001017.1 | BD130789.1 | AX348471.1 |
| CQ898618.1 | GN041714.1 | BD014038.1 | GM884934.1 | DL073057.1 | CQ774398.1 | HI000975.1 | BD130723.1 | AX347434.1 |
| CQ898586.1 | GM741829.1 | AX490811.1 | GM664822.1 | DL013504.1 | CQ758873.1 | HI002879.1 | BD130691.1 | AX347356.1 |
| CQ898554.1 | GM741787.1 | AX468926.1 | CS728562.1 | DL013472.1 | HW258935.1 | HI002841.1 | BD130593.1 | AX347288.1 |
| CQ897009.1 | GM658311.1 | AX468159.1 | CS727338.1 | DL013408.1 | HW258874.1 | HI002803.1 | BD129641.1 | AX347250.1 |
| CQ895577.1 | GM658279.1 | AX463619.1 | GM887795.1 | DL013284.1 | HW258825.1 | HI002744.1 | BD107265.1 | AX347218.1 |
| CQ895477.1 | GM658215.1 | AX458627.1 | GM869160.1 | DL017690.1 | HW257410.1 | HI002709.1 | BD094357.1 | AX347180.1 |
| CQ893707.1 | GM658101.1 | AX457970.1 | GM879382.1 | DL017658.1 | HW257314.1 | HI002660.1 | BD087155.1 | AX347092.1 |
| CQ890227.1 | GM658069.1 | AX456480.1 | FB728354.1 | DL017626.1 | HW257282.1 | HI002624.1 | BD080659.1 | AX345344.1 |
| CQ889052.1 | GM658037.1 | AX454123.1 | GM680707.1 | DL022296.1 | HW257218.1 | HI002587.1 | BD080160.1 | AX343935.1 |
| CQ879657.1 | GM658005.1 | AX451338.1 | GM842479.1 | DL022264.1 | HW257122.1 | HI002527.1 | BD080128.1 | AX342810.1 |
| CQ877815.1 | GM657973.1 | AX448935.1 | GM863435.1 | DL025347.1 | HW257090.1 | HI001479.1 | HW380835.1 | AX338568.1 |
| CQ877234.1 | GM657941.1 | A34189.1 | GM603724.1 | DL025315.1 | HW257058.1 | HI004543.1 | HW365681.1 | AX328284.1 |
| CQ876141.1 | GM650749.1 | AX443274.1 | FB722553.1 | DL025283.1 | HW256994.1 | HI004415.1 | HW381367.1 | AX327959.1 |
| CQ875539.1 | GM650717.1 | A25452.1 | FB715252.1 | DL025219.1 | HW256898.1 | HI002396.1 | HW381030.1 | AX323382.1 |
| CQ874992.1 | GM650685.1 | AX429315.1 | GM708730.1 | DL044268.1 | HW256738.1 | HI002316.1 | HW350965.1 | AX320980.1 |
| CQ874720.1 | GM636536.1 | AX427668.1 | GM061165.1 | DL040092.1 | HW256706.1 | HI000519.1 | GM631799.1 | AX319613.1 |
| CQ871419.1 | GM636504.1 | AX427206.1 | DL241391.1 | DL032035.1 | HW240890.1 | HD061134.1 | GM622868.1 | AX317542.1 |
| CQ871225.1 | GM636472.1 | AX419861.1 | DL240646.1 | DL032003.1 | HW240762.1 | M13171.1 | GM622836.1 | AX305052.1 |
| CQ869297.1 | GM657940.1 | AX418545.1 | FB749040.1 | DL031971.1 | HW240706.1 | M31335.1 | GM641432.1 | AX266990.1 |
| CQ866972.1 | GM643491.1 | AX418276.1 | DJ011751.1 | DL028130.1 | HW240642.1 | HC916594.1 | GM641382.1 | AX259987.1 |
| CQ861212.1 | GM643459.1 | AX417908.1 | DD491115.1 | DL028098.1 | HW239299.1 | HC918550.1 | GM641350.1 | AX258895.1 |
| CQ858163.1 | GM631837.1 | AX411813.1 | CS671002.1 | DL028066.1 | HW239150.1 | FW334598.1 | GM629063.1 | AX301150.1 |
| CQ857496.1 | GM631805.1 | AX407062.1 | DD463271.1 | DL028034.1 | HW238613.1 | FW337453.1 | GM629031.1 | AX299852.1 |
| CQ856126.1 | GM631773.1 | AX399464.1 | CS646207.1 | DL028002.1 | HW238553.1 | FW336065.1 | GM628999.1 | AX297652.1 |
| CQ855929.1 | GM622842.1 | AX392376.1 | DD453833.1 | DL027970.1 | HW238260.1 | FW335584.1 | GM715991.1 | AX287020.1 |
| AX376959.1 | GM889748.1 | AX391607.1 | DD456096.1 | DL044264.1 | HW248801.1 | HC868197.1 | GM638678.1 | AX283688.1 |
| AX370676.1 | GM746703.1 | AX384571.1 | CS631736.1 | DL044235.1 | HW244245.1 | HC887870.1 | GM037903.1 | HW341624.1 |
| AX364575.1 | GM009361.1 | AX376948.1 | CS631234.1 | DL044203.1 | HW237935.1 | HC887345.1 | FB774855.1 | HW350683.1 |
| AX364534.1 | GM008846.1 | AX375511.1 | CS631170.1 | DL044171.1 | HW237844.1 | HC883727.1 | FB747996.1 | HW350649.1 |
| AX364502.1 | GM888383.1 | AX370391.1 | CS627085.1 | DL048244.1 | HW248672.1 | HC880537.1 | FB764037.1 | HW350547.1 |
| AX364470.1 | FB660836.1 | AX364555.1 | DD438009.1 | DL048212.1 | HW242432.1 | HC889199.1 | FB746059.1 | HW340076.1 |
| AX364438.1 | GM687055.1 | AX364491.1 | DD449145.1 | DL048180.1 | HW242308.1 | HC877787.1 | FB743947.1 | HW349959.1 |
| AX364406.1 | FB985069.1 | AX364459.1 | DD449054.1 | DL048148.1 | HW243421.1 | HC869866.1 | FB743914.1 | HW339554.1 |
| AX364374.1 | FB983602.1 | AX364427.1 | DD441525.1 | DL047861.1 | HW243084.1 | HC869667.1 | FB743882.1 | HW364011.1 |
| AX364342.1 | FB983201.1 | AX364395.1 | CS255263.1 | DL040062.1 | HW241442.1 | HC869539.1 | FB743850.1 | HW347997.1 |
| AX364247.1 | FB983181.1 | AX364363.1 | CS253656.1 | DL040030.1 | HW163840.1 | HC357535.1 | FB743796.1 | HW347796.1 |

| | | | | | | | | |
|---|---|---|---|---|---|---|---|---|
| AX364215.1 | GM866018.1 | AX364204.1 | CS244174.1 | DL039998.1 | HV984257.1 | A07155.1 | FB761770.1 | HW347764.1 |
| DL123893.1 | GM865629.1 | HW380839.1 | CS244254.1 | DL047657.1 | HV961482.1 | HC324880.1 | DL128561.1 | HW347668.1 |
| DL123861.1 | GM864061.1 | HW368620.1 | CS244222.1 | DL043675.1 | HV959616.1 | HC324499.1 | DL128396.1 | HW347603.1 |
| DL119490.1 | FB725715.1 | HW375065.1 | CS244190.1 | DL036042.1 | HV964128.1 | HC314798.1 | DL131238.1 | HW363503.1 |
| DL119458.1 | GM731829.1 | HW373011.1 | CS243075.1 | DL031750.1 | HV959216.1 | HC318721.1 | DL114815.1 | HW355181.1 |
| DL119426.1 | GM879658.1 | HW381034.1 | CS203616.1 | DL008229.1 | HV963634.1 | HC310062.1 | DL114783.1 | HW317054.1 |
| DL123737.1 | GM615593.1 | HW070184.1 | CS193662.1 | DJ491584.1 | HV963341.1 | HC309998.1 | DL114751.1 | HW326509.1 |
| DL123705.1 | GM840856.1 | HW069780.1 | CS191097.1 | DL007797.1 | HV969949.1 | HC309966.1 | DL114687.1 | HW153760.1 |
| DL123673.1 | FB712025.1 | HW069547.1 | CS177787.1 | DJ446851.1 | HV962082.1 | HC308497.1 | DL127192.1 | HW145780.1 |
| DL123609.1 | GM723189.1 | HW084936.1 | CS174735.1 | DJ446633.1 | HV961706.1 | HC307850.1 | DL141447.1 | HW145131.1 |
| DL123577.1 | GM041709.1 | HW089594.1 | CS172444.1 | DJ437136.1 | HV951615.1 | HC307795.1 | DL119107.1 | HW152286.1 |
| DL119387.1 | GM040263.1 | HW089032.1 | CS159811.1 | DJ437036.1 | HV951583.1 | FU263039.1 | DL119075.1 | HW151999.1 |
| DL119355.1 | FB709054.1 | HW097683.1 | CS159779.1 | DJ436816.1 | HV951173.1 | FU259874.1 | DL119043.1 | HW151680.1 |
| DL119323.1 | CS728638.1 | HV959867.1 | CS141596.1 | DJ445567.1 | HV950631.1 | FU250454.1 | DL119011.1 | HW144747.1 |
| DL119291.1 | CS728574.1 | HV956201.1 | CS141537.1 | DJ444559.1 | HV701204.1 | FU264506.1 | DL031200.1 | HW126546.1 |
| DL114831.1 | CS727351.1 | HV951101.1 | CS141505.1 | DJ434794.1 | HV701172.1 | FU258458.1 | DL019494.1 | HW125523.1 |
| DL114799.1 | FB715340.1 | HV957481.1 | CS139109.1 | DJ433879.1 | HV701140.1 | HC313121.1 | DL019430.1 | HW144377.1 |
| DL114767.1 | GM836381.1 | HV953055.1 | CS134726.1 | DJ438348.1 | HV708209.1 | HC313053.1 | DL019398.1 | HW144313.1 |
| DL114735.1 | GM949536.1 | HV944605.1 | CS122777.1 | DJ438281.1 | HV701089.1 | DL099518.1 | DL014435.1 | HW124954.1 |
| DL114703.1 | GM006120.1 | HV943089.1 | BD270924.1 | DJ402287.1 | HV695214.1 | DL099486.1 | DD401916.1 | HW124523.1 |
| DL114671.1 | FB985405.1 | HV774713.1 | DD231071.1 | DJ400829.1 | HV695118.1 | DL099454.1 | DD401852.1 | HW123532.1 |
| DL130780.1 | FB983331.1 | HV767837.1 | DD227395.1 | DJ400797.1 | HV694800.1 | DL099422.1 | DD401820.1 | HW123464.1 |
| DL141543.1 | GM879373.1 | HV743735.1 | DD227153.1 | DJ419889.1 | HV700808.1 | DL089541.1 | DD401788.1 | HW122704.1 |
| DL123537.1 | GM879312.1 | HB647042.1 | DD224790.1 | DJ398954.1 | HV694733.1 | DL089509.1 | DD401548.1 | HW120098.1 |
| DL013574.1 | GM680720.1 | HB645447.1 | DD224361.1 | DJ391346.1 | HV694669.1 | DL089477.1 | DD401516.1 | HW118286.1 |
| DL013542.1 | DL092319.1 | HB855973.1 | DD223915.1 | DJ418053.1 | HV693340.1 | DL089445.1 | DD401484.1 | HW117539.1 |
| DL013510.1 | DL092287.1 | HB855778.1 | DD231451.1 | DJ390557.1 | HV699489.1 | DL113547.1 | DD401452.1 | HW115955.1 |
| DL013478.1 | DL092255.1 | GM633243.1 | DD213980.1 | DJ402719.1 | HV698220.1 | DL113515.1 | CS479844.1 | HW115619.1 |
| DL013446.1 | DL092223.1 | GM633211.1 | DD213104.1 | DJ381030.1 | HV698136.1 | DL099308.1 | CS476138.1 | HW115040.1 |
| DL013414.1 | DL092191.1 | GM633179.1 | CS254915.1 | DJ380998.1 | HV689462.1 | DL093140.1 | CS464652.1 | HV951894.1 |
| DL013382.1 | DL088416.1 | GM651768.1 | DD193422.1 | DJ380966.1 | HV600832.1 | DL093108.1 | DD400175.1 | HV945826.1 |
| DL013226.1 | DL088384.1 | GM644808.1 | DD206899.1 | DJ380934.1 | HV585148.1 | DL093076.1 | DD400142.1 | HV939954.1 |
| DL013194.1 | FB316731.1 | GM625183.1 | DD206867.1 | DJ380902.1 | HV585083.1 | DL089269.1 | A13001.1 | HV945745.1 |
| DL017983.1 | FB328920.1 | GM625151.1 | DD206805.1 | DJ380870.1 | HV592412.1 | DL089237.1 | CS459124.1 | HV943517.1 |
| DL017951.1 | FB317750.1 | GM623997.1 | DD206773.1 | DJ380818.1 | HV313062.1 | DL125928.1 | DD367719.1 | HV942759.1 |
| DL017919.1 | CS480862.1 | GM623965.1 | HH975095.1 | CS592270.1 | HV309529.1 | DL125896.1 | DD361273.1 | HV942056.1 |
| DL017887.1 | CS368310.1 | FB506773.1 | HH982453.1 | CS593400.1 | HV200232.1 | DL122558.1 | DD367533.1 | HV815894.1 |
| DL017855.1 | CS368246.1 | GM642986.1 | HH974672.1 | CS597721.1 | HV194345.1 | DL122526.1 | DD362647.1 | HV932245.1 |
| DL017823.1 | CS368054.1 | GM642953.1 | HH974555.1 | CS593356.1 | HV182449.1 | DL118112.1 | DD368135.1 | HV932145.1 |
| DL017792.1 | CS367990.1 | GM630645.1 | HH963836.1 | CS584893.1 | HV182417.1 | DL118080.1 | DD361307.1 | HV937044.1 |
| DL017760.1 | CS367734.1 | GM630613.1 | HH998092.1 | CS576152.1 | HV182385.1 | DL026622.1 | CS453770.1 | HV695222.1 |
| DL017696.1 | CS367670.1 | GM630581.1 | HH998029.1 | CS575305.1 | HV203127.1 | DL026590.1 | CS450639.1 | HV695190.1 |
| DL017664.1 | DJ084893.1 | GM621646.1 | HH997985.1 | CS574831.1 | HV235487.1 | DL026558.1 | BD453787.1 | HV182393.1 |
| DL017632.1 | DJ069482.1 | GM621614.1 | HH999667.1 | CS574128.1 | HV232478.1 | DL019571.1 | BD433651.1 | HV221024.1 |
| DL022270.1 | DJ066372.1 | GM621582.1 | HH997933.1 | CS573069.1 | HV117717.1 | DL019539.1 | BD453763.1 | HV117787.1 |
| DL022238.1 | DJ066340.1 | FB506036.1 | HH997851.1 | CS580614.1 | HV038647.1 | DL019475.1 | BD453731.1 | HV112790.1 |
| DL022206.1 | DJ066307.1 | GM657130.1 | HH997788.1 | CS544062.1 | HV038587.1 | DL019443.1 | BD453699.1 | HV112544.1 |

| | | | | | | | | |
|---|---|---|---|---|---|---|---|---|
| DL025561.1 | DJ066273.1 | GM642722.1 | HH997749.1 | CS565539.1 | HV182347.1 | DL019411.1 | BD453667.1 | HV038655.1 |
| DL025353.1 | DJ066239.1 | GM638810.1 | HH999471.1 | CS549430.1 | HV182315.1 | DL014912.1 | BD453635.1 | HV038595.1 |
| DL025321.1 | DJ061680.1 | GM638778.1 | HH999426.1 | CS561173.1 | HV216216.1 | DL014880.1 | BD453603.1 | HV182355.1 |
| DL025289.1 | DJ061625.1 | GM638746.1 | HH999376.1 | CS559088.1 | HV228427.1 | DL026498.1 | BD453553.1 | HV182323.1 |
| DL025225.1 | DJ061577.1 | GM638650.1 | HH997666.1 | DD420047.1 | HV306505.1 | DL026466.1 | BD453521.1 | HV182291.1 |
| DL025193.1 | DJ061481.1 | GM626103.1 | HH997628.1 | DD431818.1 | HV035635.1 | DL026434.1 | BD453489.1 | HV190851.1 |
| DL021885.1 | DJ061417.1 | GM037972.1 | HH997571.1 | CS539993.1 | FZ435990.1 | DL026402.1 | BD453457.1 | HV234586.1 |
| DL021853.1 | DJ060186.1 | FB774863.1 | HH997496.1 | CS543234.1 | FZ427587.1 | DL010188.1 | BD453425.1 | HV228160.1 |
| DL021821.1 | DJ053299.1 | FB766163.1 | HH997401.1 | CS537862.1 | FZ430353.1 | DL014512.1 | BD453393.1 | HV234190.1 |
| DL021789.1 | CS810626.1 | FB764685.1 | HH997357.1 | CS537395.1 | FZ430138.1 | DL014480.1 | BD453248.1 | HV312966.1 |
| DL017423.1 | CS812988.1 | FB746163.1 | HH999285.1 | CS499900.1 | HW260661.1 | DL014448.1 | BD453216.1 | HV302090.1 |
| DL038459.1 | CS811262.1 | FB743951.1 | HH999222.1 | CS498360.1 | HW260597.1 | DL009830.1 | BD496249.1 | HV030242.1 |
| DL038427.1 | DJ021065.1 | FB743918.1 | HH999174.1 | CS497134.1 | HW260565.1 | DL009798.1 | BD497445.1 | HV037877.1 |
| DL038395.1 | DJ019951.1 | FB743886.1 | HH999133.1 | DD418607.1 | HW260533.1 | DL009766.1 | BD398646.1 | FZ435966.1 |
| DL038363.1 | CS803357.1 | FB743854.1 | HH999092.1 | DD288448.1 | HW260501.1 | DL009542.1 | BD408020.1 | FZ435874.1 |
| DL038331.1 | HV502614.1 | FB743800.1 | HH999053.1 | DD287659.1 | HW260469.1 | DL009510.1 | BD407575.1 | FZ430246.1 |
| DL013168.1 | HV502582.1 | FB761789.1 | HH998992.1 | DD278892.1 | HW260437.1 | DL009478.1 | BD428455.1 | FZ424098.1 |
| DL013136.1 | HV502518.1 | FB742936.1 | HH998945.1 | CS355959.1 | HW260405.1 | DJ328047.1 | BD405241.1 | FZ423911.1 |
| DL013104.1 | HV502486.1 | FB708881.1 | HH997216.1 | CS355202.1 | HW260373.1 | DJ326981.1 | BD426668.1 | FZ423860.1 |
| DL013072.1 | HV505366.1 | DL098000.1 | HC757680.1 | CS350538.1 | HW260341.1 | DJ128223.1 | BD404617.1 | HH791300.1 |
| DL013040.1 | FW552194.1 | DL097968.1 | HC733785.1 | CS348909.1 | HW260309.1 | DJ122450.1 | BD445852.1 | HH779675.1 |
| DL013008.1 | FW552156.1 | DL097936.1 | HC733776.1 | CS352969.1 | HW260277.1 | DJ086521.1 | BD453368.1 | HH777918.1 |
| DL023144.1 | FW552119.1 | DL120274.1 | HC733699.1 | CS352598.1 | HW260245.1 | DJ082686.1 | BD453332.1 | HH774433.1 |
| DL023080.1 | FW561809.1 | DL120242.1 | HC732456.1 | CS352403.1 | HW260213.1 | DJ081969.1 | BD453304.1 | HH773135.1 |
| DL023048.1 | FW555625.1 | DL120210.1 | HC731351.1 | CS284252.1 | HW260181.1 | DJ086337.1 | BD453272.1 | HH820913.1 |
| DL023016.1 | FW563141.1 | DL111093.1 | HC731319.1 | CS283929.1 | HW260149.1 | DJ081533.1 | BD402325.1 | HH759319.1 |
| DL022984.1 | FW561688.1 | DL101420.1 | HC731287.1 | CS273202.1 | HW260117.1 | DJ080758.1 | BD388658.1 | HH759209.1 |
| DL019809.1 | FW565192.1 | DL101217.1 | HC731255.1 | CS252539.1 | HW260085.1 | DJ080178.1 | BD376116.1 | HH759177.1 |
| DL010724.1 | FW562568.1 | DL094687.1 | HC471780.1 | CS249831.1 | HW260053.1 | DJ069489.1 | BD375698.1 | HH759145.1 |
| DL010692.1 | FW562482.1 | DL090944.1 | HC471748.1 | CS250638.1 | HW260021.1 | DJ071399.1 | BD375402.1 | HH759081.1 |
| DL010660.1 | FW562442.1 | DL090912.1 | HC471682.1 | CS248883.1 | HW259989.1 | DJ066775.1 | AX958967.1 | HH759049.1 |
| DL010628.1 | FW555363.1 | DL086976.1 | HC471708.1 | CS245375.1 | HW259957.1 | DJ066415.1 | AX934536.1 | HH759017.1 |
| DL047052.1 | FW553147.1 | DL086944.1 | HC471652.1 | CS244158.1 | FW592654.1 | DJ066379.1 | AX954795.1 | HH758985.1 |
| DL047020.1 | FW556606.1 | DL117889.1 | HC471620.1 | CS244238.1 | HQ161055.1 | DJ066347.1 | AX938918.1 | HH758953.1 |
| DL046988.1 | FW509392.1 | DL117857.1 | HC729932.1 | CS244206.1 | FW576617.1 | DJ066314.1 | AX923420.1 | HH757465.1 |
| DL046956.1 | FW367667.1 | DL117825.1 | HC471508.1 | CS243162.1 | FW575546.1 | DJ066280.1 | AX923387.1 | HH757433.1 |
| DL046924.1 | FW367378.1 | DL088954.1 | HC471566.1 | CS237722.1 | FW572363.1 | DJ066246.1 | AX839012.1 | HH756174.1 |
| DL046892.1 | FW366498.1 | DL088922.1 | HC729370.1 | CS231154.1 | FW574610.1 | DJ061636.1 | AX832725.1 | HI401447.1 |
| DL046860.1 | FW351377.1 | DL088624.1 | HC729077.1 | CS229987.1 | FW571736.1 | DJ061444.1 | AX824686.1 | HI401087.1 |
| DL042942.1 | FW351219.1 | DL088592.1 | HC729006.1 | CS227328.1 | HI968051.1 | DJ055578.1 | AX824351.1 | HI642691.1 |
| DL042910.1 | HD122360.1 | DL024916.1 | HC728816.1 | CS227247.1 | HI967748.1 | DJ055490.1 | AX823785.1 | HI642147.1 |
| DL042878.1 | HD115793.1 | DL024852.1 | HC727696.1 | CS205998.1 | HI973485.1 | DJ061188.1 | AX823751.1 | HI641603.1 |
| DL038965.1 | HD084539.1 | DL027807.1 | HC726153.1 | DD161382.1 | HI969348.1 | DJ054979.1 | BD130577.1 | HI641059.1 |
| DL038933.1 | HD113815.1 | DL027775.1 | HC688455.1 | DD167016.1 | HI987419.1 | DJ060404.1 | BD106991.1 | HI637700.1 |
| DL031081.1 | HD082406.1 | DL023867.1 | HC679619.1 | DD166569.1 | HI422683.1 | DJ053433.1 | BD091314.1 | HH982234.1 |
| DL030956.1 | HD081719.1 | DL023835.1 | HC490980.1 | DD166217.1 | HI415741.1 | DJ056349.1 | BD083865.1 | HH974545.1 |
| DL027144.1 | HD087680.1 | DL023803.1 | HC490948.1 | DD159670.1 | HI414028.1 | DJ053077.1 | BD081962.1 | HH998071.1 |

| | | | | | | | | |
|---|---|---|---|---|---|---|---|---|
| DL027112.1 | HD079750.1 | DL031533.1 | HC490916.1 | DD165141.1 | HI413991.1 | CS810633.1 | BD081727.1 | HH998019.1 |
| DL027080.1 | HD071338.1 | DL023688.1 | HC490884.1 | DD159540.1 | HI413620.1 | CS810521.1 | BD081532.1 | HH997959.1 |
| DL027048.1 | FW345032.1 | DL023656.1 | HC687196.1 | CS716089.1 | HI413585.1 | CS813007.1 | BD080154.1 | HH999657.1 |
| DL027016.1 | FW345000.1 | DL023624.1 | HC490844.1 | CS796323.1 | HI503568.1 | FB292092.1 | BD079951.1 | HH997908.1 |
| DL042871.1 | FW344946.1 | DL023592.1 | HC490812.1 | CS675438.1 | HI516567.1 | FB292229.1 | BD074963.1 | HH997837.1 |
| DL030911.1 | FW343720.1 | DL015918.1 | HC490780.1 | CS680726.1 | HI516103.1 | DJ022624.1 | BD073868.1 | HH997773.1 |
| DL030872.1 | FW343652.1 | DL015854.1 | HC490716.1 | CS790989.1 | HI516071.1 | CS803364.1 | BD070726.1 | HH999511.1 |
| DD010449.1 | FW343524.1 | DL047628.1 | HC490684.1 | CS792472.1 | HI284348.1 | BD242455.1 | BD069513.1 | HH999456.1 |
| DD010131.1 | HC922991.1 | DL047596.1 | HC490652.1 | CS791961.1 | HI284290.1 | BD241789.1 | BD016711.1 | HH999416.1 |
| DD019563.1 | HC922473.1 | DL047532.1 | HC490620.1 | DJ045217.1 | HI574511.1 | BD240740.1 | E63270.1 | HH999363.1 |
| DD017355.1 | HC921520.1 | DL039669.1 | HC490588.1 | DJ044924.1 | HI574447.1 | BD237287.1 | E50487.1 | HH997652.1 |
| DD014180.1 | HC920514.1 | CS667625.1 | HC490556.1 | DJ044883.1 | HI283456.1 | BD235959.1 | BD014210.1 | HH997618.1 |
| BD495445.1 | HD068867.1 | CS647387.1 | HC490520.1 | DJ030371.1 | HI574360.1 | BD235640.1 | BD014170.1 | HH995713.1 |
| BD453899.1 | HD063940.1 | CS646217.1 | HC490488.1 | DJ029980.1 | HI581388.1 | BD234426.1 | BD013810.1 | HH999700.1 |
| BD453867.1 | DM462248.1 | CS646179.1 | HC490449.1 | DJ026273.1 | HI578401.1 | BD234362.1 | AX490769.1 | HH999249.1 |
| BD453835.1 | DM464641.1 | DD460418.1 | HC490417.1 | CS691341.1 | HI593982.1 | BD232308.1 | E64420.1 | HH999204.1 |
| BD453803.1 | DM462710.1 | DD460133.1 | HC490385.1 | CS410807.1 | HI588454.1 | BD231843.1 | E66924.1 | HH999155.1 |
| BD493553.1 | HC190456.1 | DD459655.1 | HC490353.1 | CS414830.1 | HI636997.1 | BD231163.1 | AX468902.1 | HH998122.1 |
| BD453747.1 | HC089572.1 | DD458770.1 | HC490321.1 | DD327648.1 | HI001473.1 | BD231129.1 | AX467167.1 | HH999079.1 |
| BD453715.1 | HC089540.1 | DD453843.1 | HC490289.1 | DD327019.1 | HI001373.1 | BD229095.1 | A18208.1 | HH999036.1 |
| BD453683.1 | HC089508.1 | DD453693.1 | HC490256.1 | DD329893.1 | HI001340.1 | BD227163.1 | AX458597.1 | HH998973.1 |
| BD453651.1 | HC089476.1 | DD456646.1 | HC490224.1 | CS389266.1 | HI003263.1 | BD226907.1 | AX458221.1 | HH998927.1 |
| BD453619.1 | HC089444.1 | CS631746.1 | HC472310.1 | CS389181.1 | HI003185.1 | BD226752.1 | AX456938.1 | HH997187.1 |
| BD453582.1 | HC089411.1 | CS631254.1 | HC472277.1 | CS406501.1 | HI003124.1 | BD226413.1 | AX454010.1 | HH997144.1 |
| BD453569.1 | HC089379.1 | CS631190.1 | HC472245.1 | CS402164.1 | HI003064.1 | HC069789.1 | AX443470.1 | HH998881.1 |
| BD453537.1 | HC089347.1 | CS642128.1 | HC472213.1 | CS382085.1 | HI003030.1 | DM461199.1 | AX440946.1 | HH998821.1 |
| BD453505.1 | HC089314.1 | CS627825.1 | HC472181.1 | CS382603.1 | HI002991.1 | U26412.1 | AX430071.1 | HH998759.1 |
| BD453473.1 | HC089282.1 | CS632196.1 | HC471949.1 | DD308885.1 | HI002927.1 | GM675316.1 | AX428406.1 | HH997047.1 |
| BD453441.1 | HC089250.1 | CS626247.1 | HC471917.1 | DD298333.1 | HI001292.1 | GM742775.1 | AX427050.1 | HH997005.1 |
| BD453375.1 | HC087517.1 | AX143613.1 | HC471885.1 | DD307932.1 | HI001246.1 | FB509175.1 | AX418261.1 | HH996951.1 |
| BD453232.1 | HC086495.1 | AX143229.1 | HC471789.1 | DD292507.1 | HI001195.1 | GM774139.1 | AX411711.1 | HH998706.1 |
| BD453200.1 | AY774936.1 | AX142973.1 | HC471830.1 | DD319811.1 | HI001144.1 | GM710192.1 | AX399388.1 | HH998668.1 |
| BD421159.1 | AY774896.1 | AX142909.1 | HC471798.1 | CS376416.1 | HI001097.1 | GM709008.1 | HW153773.1 | HH998599.1 |
| BD441299.1 | AY774834.1 | AX142845.1 | HC679502.1 | CS376122.1 | HI001041.1 | GM708976.1 | HW153283.1 | HH998544.1 |
| BD497558.1 | AY774660.1 | AX142717.1 | HC678793.1 | CS362661.1 | HI001007.1 | GM655005.1 | HW144706.1 | HH996923.1 |
| BD430775.1 | AY774606.1 | AX142653.1 | HC668141.1 | CS362621.1 | HI000961.1 | GM044931.1 | HW125555.1 | HH996767.1 |
| BD450797.1 | AY774537.1 | AX142589.1 | HC668108.1 | CS360560.1 | HI002863.1 | GM044223.1 | HW144356.1 | HH977638.1 |
| BD408107.1 | AY774436.1 | AX142525.1 | HC510419.1 | CS359735.1 | HI002831.1 | GM752800.1 | HW144324.1 | HH954140.1 |
| BD408025.1 | AY774373.1 | AX142459.1 | HC508645.1 | CS359596.1 | HI002793.1 | DL467562.1 | HW144292.1 | HH980638.1 |
| BD419033.1 | AY774319.1 | AX142395.1 | HB476486.1 | BD269136.1 | HI002733.1 | GM712946.1 | HW124771.1 | HH980497.1 |
| BD429572.1 | AY774272.1 | AX142203.1 | HB485600.1 | BD268157.1 | FU264481.1 | GM652748.1 | HW122861.1 | HH980429.1 |
| CS038684.1 | AY774216.1 | AX142075.1 | HB475770.1 | BD268109.1 | FU264305.1 | GM652716.1 | HW046687.1 | HH980356.1 |
| CS037087.1 | AY774154.1 | AX142009.1 | HB475525.1 | BD265606.1 | DM467411.1 | GM652652.1 | HV986648.1 | HH980296.1 |
| CS023839.1 | AY774022.1 | AX141945.1 | HB475467.1 | BD264365.1 | HC299769.1 | GM652620.1 | HV959128.1 | HH980221.1 |
| CS022573.1 | AF470454.1 | AX141687.1 | HB475228.1 | BD263547.1 | HC306192.1 | GM645494.1 | HV963624.1 | HH980157.1 |
| CS021534.1 | AF274974.1 | AX141495.1 | HB464823.1 | BD263446.1 | HC306076.1 | GM645462.1 | HV963327.1 | HH980124.1 |
| CS019536.1 | HC084828.1 | AX141431.1 | HB463820.1 | BD251209.1 | HC305996.1 | GM645430.1 | HV956092.1 | GN032042.1 |

| | | | | | | | | |
|---|---|---|---|---|---|---|---|---|
| CQ990435.1 | HC084181.1 | AX141367.1 | HB471285.1 | DD135258.1 | HC302639.1 | GM638537.1 | HV956028.1 | GN032010.1 |
| CQ986632.1 | HC083718.1 | AX141303.1 | HB469607.1 | DD133438.1 | HC302322.1 | GM638505.1 | HV950606.1 | GN031978.1 |
| CQ986598.1 | HC083541.1 | AX127334.1 | HB469110.1 | DD107443.1 | HC305924.1 | GM638473.1 | HV753221.1 | GN031946.1 |
| CQ986566.1 | HC081749.1 | AX113703.1 | HB468043.1 | DD107337.1 | HC305884.1 | GM650464.1 | HV758997.1 | GN031914.1 |
| CQ986532.1 | M35873.1 | HW152042.1 | DM148842.1 | DD091015.1 | HC305844.1 | GM643496.1 | HV760395.1 | GN031882.1 |
| CQ983152.1 | HC061876.1 | HW151063.1 | DM148794.1 | DD104228.1 | HC305804.1 | GM643464.1 | HV749446.1 | GN031850.1 |
| CQ983071.1 | HC056942.1 | HW159788.1 | DM152833.1 | DD089928.1 | HC305764.1 | GM643431.1 | HV753494.1 | GN031818.1 |
| CQ983008.1 | L09153.1 | HW144715.1 | DM148096.1 | DD102597.1 | HC305724.1 | GM636442.1 | HV600957.1 | GN031720.1 |
| CQ982973.1 | L08930.1 | HW126450.1 | DM152712.1 | DD057934.1 | HC305684.1 | GM631842.1 | HV570614.1 | GN031688.1 |
| CQ982680.1 | L08919.1 | HW125390.1 | DM152572.1 | DD057902.1 | HC305644.1 | GM631810.1 | HV569656.1 | GN031656.1 |
| CQ982602.1 | M62710.1 | HW144362.1 | DM147324.1 | DD057870.1 | HC305041.1 | GM631778.1 | HV567021.1 | GN031624.1 |
| CQ981125.1 | DM059608.1 | HW124864.1 | DM156261.1 | DD057842.1 | HC294218.1 | GM622847.1 | HV565841.1 | GN031560.1 |
| CQ975463.1 | DM056875.1 | HW124782.1 | DM155877.1 | DD053298.1 | HC251242.1 | GM741196.1 | HV572454.1 | GN031528.1 |
| CQ974380.1 | DM045352.1 | HW123009.1 | DM146480.1 | DD052282.1 | HC291366.1 | GM657900.1 | HV572314.1 | GN031496.1 |
| CQ973488.1 | DM045090.1 | HW122420.1 | DM146447.1 | DD052223.1 | HC289380.1 | GM657868.1 | HV558761.1 | GN031464.1 |
| CQ976623.1 | DM044882.1 | HW122124.1 | DM146351.1 | DD041903.1 | HC289336.1 | GM657836.1 | HV565505.1 | GN031431.1 |
| AX587905.1 | DM039596.1 | HW120874.1 | DM151017.1 | DD046655.1 | HV325779.1 | GM657804.1 | HV302956.1 | GN031399.1 |
| AX587703.1 | DM060862.1 | HW117992.1 | DM155730.1 | DD042873.1 | FW565191.1 | GM657771.1 | HV309040.1 | GN031367.1 |
| AX587603.1 | DM044782.1 | HW115144.1 | DM150257.1 | DD039012.1 | FW562481.1 | GM636199.1 | HV312358.1 | GN031335.1 |
| AX577656.1 | DM060664.1 | FW577214.1 | DM150116.1 | DD037264.1 | FW562441.1 | GM631631.1 | HV118201.1 | GN031303.1 |
| AX574412.1 | GN068385.1 | FW576615.1 | DM149950.1 | DD032570.1 | FW553146.1 | GM631599.1 | HV203382.1 | GN031238.1 |
| AX556823.1 | HW261012.1 | FW576105.1 | HB461819.1 | DD032256.1 | FW556605.1 | GM704218.1 | HV200222.1 | GN031206.1 |
| AX555192.1 | HW260980.1 | HM124448.1 | HB455314.1 | DD031195.1 | FW510507.1 | GM704164.1 | HV217031.1 | GN031174.1 |
| AX554281.1 | HW260948.1 | FW573712.1 | HB454802.1 | DD030666.1 | FW505328.1 | GM657654.1 | HV038771.1 | GN031141.1 |
| AX544463.1 | HW260916.1 | FW572357.1 | HB447880.1 | DD027308.1 | HI930285.1 | GM622444.1 | HV038701.1 | GN031109.1 |
| AX537564.1 | HW260884.1 | FW573340.1 | HB445472.1 | DD206882.1 | HI918285.1 | GM657406.1 | HV190734.1 | GN031077.1 |
| AX536407.1 | HW260852.1 | HI973445.1 | DM069113.1 | DD206850.1 | HI918253.1 | GM639983.1 | HV234454.1 | GN031045.1 |
| AX529072.1 | HW260820.1 | HI988968.1 | DM069050.1 | DD206818.1 | HI660689.1 | GM639919.1 | HV225424.1 | GN031013.1 |
| AX528109.1 | HW260788.1 | HI969344.1 | DM068821.1 | CS250104.1 | HI657457.1 | GM639834.1 | HI002954.1 | GN030981.1 |
| AX523916.1 | HW260756.1 | FW368417.1 | GN087811.1 | DD181015.1 | FW420817.1 | GM627281.1 | HI002916.1 | GN030950.1 |
| AX523634.1 | HW260724.1 | FW367665.1 | GN091113.1 | DD173404.1 | FW495957.1 | GM627249.1 | HI001282.1 | GN030918.1 |
| A33389.1 | HW260692.1 | FW351375.1 | GN089876.1 | DD167015.1 | FW418989.1 | GM627217.1 | HI001233.1 | GN030886.1 |
| AX179485.1 | HW260660.1 | FW361307.1 | GN089812.1 | DD178545.1 | FW418231.1 | GM627185.1 | HI001183.1 | GN030822.1 |
| AX179422.1 | HW260628.1 | HD115790.1 | GN082901.1 | DD166216.1 | FW396287.1 | GM627153.1 | HI001112.1 | GN030790.1 |
| A28422.1 | HW260596.1 | HD085074.1 | GN089218.1 | DD159649.1 | FW396662.1 | GM660977.1 | HI001089.1 | DM002004.1 |
| A09537.1 | HW260564.1 | HD113808.1 | GN082830.1 | DD159536.1 | HH834395.1 | GM660945.1 | HI001033.1 | DM001675.1 |
| AX521539.1 | HW260532.1 | HD087589.1 | GN079084.1 | CS203953.1 | HH833981.1 | GM646782.1 | HI000999.1 | DM006945.1 |
| AX521507.1 | HW260500.1 | HD079734.1 | DL233319.1 | AF397140.1 | HH924314.1 | GM646750.1 | HI000952.1 | DM006691.1 |
| AF430193.1 | HW260468.1 | FW345030.1 | DL232694.1 | CS157790.1 | HH925715.1 | GM646718.1 | HD070778.1 | DM004997.1 |
| AF430161.1 | HW260436.1 | FW344998.1 | GN078627.1 | CS157951.1 | HH827053.1 | GM646686.1 | HD070419.1 | DM003496.1 |
| AX511422.1 | HW260404.1 | FW344940.1 | GN075925.1 | CS157919.1 | HH826950.1 | GM646654.1 | HD069972.1 | DL488432.1 |
| AX505147.1 | HW260372.1 | FW343714.1 | GN078808.1 | CS141096.1 | HH822199.1 | GM646622.1 | FW345540.1 | DM009385.1 |
| AX498407.1 | HW260340.1 | FW343648.1 | L08913.1 | CS124708.1 | HH821962.1 | GM639819.1 | FW345141.1 | GM643087.1 |
| AX497095.1 | HW260308.1 | FW343584.1 | L08784.1 | CS124634.1 | FW332350.1 | GM639787.1 | FW345021.1 | GM643055.1 |
| BD138744.1 | HW260244.1 | HC922981.1 | DM058858.1 | CS119877.1 | FW306359.1 | GM639755.1 | FW344411.1 | GM643023.1 |
| BD138632.1 | HW260212.1 | HC920509.1 | DM058796.1 | CS119537.1 | FW305567.1 | GM044801.1 | FW344222.1 | GM635856.1 |
| BD135802.1 | HW260180.1 | HD068862.1 | DM058730.1 | CS119436.1 | FW310643.1 | GM646582.1 | FW344036.1 | GM622214.1 |

| | | | | | | | | |
|---|---|---|---|---|---|---|---|---|
| BD132941.1 | HW260148.1 | HC486483.1 | DM057730.1 | CS119403.1 | HC767601.1 | GM646518.1 | FW343696.1 | GM622182.1 |
| BD130802.1 | HW260116.1 | HC466624.1 | DM045540.1 | CS119370.1 | HC767368.1 | GM646486.1 | FW343630.1 | GM622150.1 |
| BD130736.1 | HW260084.1 | HC474624.1 | DM056744.1 | CS119338.1 | HC757169.1 | GM646454.1 | FW343566.1 | GM631009.1 |
| BD130704.1 | HW260052.1 | HC491747.1 | DM045279.1 | CS119304.1 | HC757137.1 | GM646422.1 | FW343502.1 | GM630977.1 |
| BD130615.1 | HW260020.1 | HC488312.1 | DM044996.1 | CS119272.1 | HC754644.1 | GM639555.1 | FW342700.1 | GM630945.1 |
| BD105786.1 | HW259988.1 | FV531702.1 | DM039616.1 | CS119240.1 | HC742026.1 | GM639523.1 | HC922747.1 | GM630913.1 |
| BD105665.1 | HW259956.1 | FV522840.1 | DM039583.1 | CS119208.1 | HC741957.1 | GM626880.1 | HC922150.1 | FB506784.1 |
| AX114301.1 | HW259924.1 | FV522800.1 | DM044700.1 | CS119176.1 | HC732132.1 | GM626816.1 | HC921417.1 | GM630881.1 |
| AX113759.1 | HW259667.1 | FV530128.1 | DM060748.1 | DL075889.1 | HC731665.1 | GM660764.1 | HC920494.1 | GM642962.1 |
| AX113549.1 | HW258930.1 | FV528428.1 | DM060716.1 | DL075249.1 | HC471759.1 | GM660732.1 | HD068788.1 | GM642930.1 |
| AX107875.1 | HW257407.1 | FV519160.1 | DM060684.1 | DL078923.1 | HC471663.1 | GM653513.1 | HD067657.1 | GM630654.1 |
| AX107006.1 | HW257375.1 | FV534403.1 | DM044395.1 | DL074245.1 | HC471631.1 | GM653481.1 | HD066666.1 | GM630622.1 |
| AX103642.1 | HW257279.1 | HC461787.1 | GN069430.1 | DL073052.1 | HC471599.1 | GM653449.1 | HD064774.1 | GM630590.1 |
| AX100351.1 | HW257023.1 | HC460860.1 | GN067931.1 | DL013501.1 | HC689106.1 | GM653417.1 | HD063927.1 | GM621655.1 |
| AX097518.1 | HW256991.1 | AY273891.1 | GN067867.1 | DL013469.1 | HC688488.1 | GM653385.1 | HD053206.1 | GM621591.1 |
| AX097482.1 | HW256927.1 | HC456305.1 | GN067835.1 | DL013437.1 | HC688415.1 | GM646386.1 | HD052978.1 | GM621559.1 |
| AX088747.1 | HW256895.1 | HC453699.1 | GN067775.1 | DL013405.1 | HC687682.1 | GM646354.1 | HD051891.1 | GM657139.1 |
| AX088691.1 | HW256799.1 | HC453667.1 | GN067743.1 | DL047954.1 | HC490855.1 | GM646322.1 | FU762946.1 | GM657107.1 |
| AX085490.1 | HW256735.1 | HC460362.1 | GN067647.1 | DL047858.1 | HC490823.1 | GM646290.1 | FU762509.1 | GM657075.1 |
| AX082945.1 | HW251047.1 | HC459800.1 | GN065525.1 | DL040059.1 | HC490791.1 | GM646258.1 | FU756369.1 | GM657043.1 |
| AX080757.1 | FZ421042.1 | AF073995.1 | GN062490.1 | DL040027.1 | HC490759.1 | GM646226.1 | FU756336.1 | GM640911.1 |
| AX079165.1 | FZ417512.1 | HC452130.1 | GN060838.1 | DL039995.1 | HC490727.1 | GM639391.1 | FU756302.1 | GM640879.1 |
| AX077281.1 | FZ417015.1 | HC451928.1 | GN060524.1 | DL043672.1 | HC490695.1 | GM639359.1 | FU762197.1 | GM640847.1 |
| AX076477.1 | FZ413941.1 | HB423194.1 | GM651997.1 | DL027842.1 | HC490531.1 | GM639327.1 | HC439126.1 | GM628493.1 |
| AX067253.1 | FZ419844.1 | HB403661.1 | GM651965.1 | DL027810.1 | HC490499.1 | GM639295.1 | HC438527.1 | GM662106.1 |
| AX062336.1 | FZ419810.1 | HB397069.1 | GM651933.1 | DL027778.1 | HC490267.1 | GM639263.1 | HC436484.1 | GM654824.1 |
| AX058994.1 | FW582230.1 | HB394262.1 | GM711646.1 | DL023870.1 | HC472288.1 | GM626716.1 | HC359029.1 | GM654792.1 |
| AX058556.1 | FW591769.1 | HB394230.1 | GM831282.1 | DL023838.1 | HC472256.1 | DL090951.1 | HC358388.1 | GM654760.1 |
| AX052917.1 | FW590752.1 | HB393673.1 | GM831180.1 | DL023806.1 | HC472224.1 | DL090919.1 | HC358200.1 | GM647767.1 |
| AX047139.1 | FW589142.1 | DM114852.1 | GM830868.1 | DL031536.1 | HC472192.1 | DL086983.1 | HC358069.1 | GM647735.1 |
| AX046150.1 | HQ161054.1 | GN368621.1 | GM711494.1 | DL042643.1 | GN067594.1 | AY659205.1 | HC357779.1 | GM647703.1 |
| AX040783.1 | FW577623.1 | HB340019.1 | GM830722.1 | DL042611.1 | GN053502.1 | AY659173.1 | HC357223.1 | GM628289.1 |
| AX039563.1 | FW577222.1 | HA641518.1 | FB509367.1 | DL042579.1 | GN052403.1 | AY659141.1 | HC321182.1 | GM627965.1 |
| AX039321.1 | FW576106.1 | HA639656.1 | FB509331.1 | DL034126.1 | GN052371.1 | AY659109.1 | HC356235.1 | GM627933.1 |
| AX036759.1 | FW575543.1 | HA643899.1 | FB509299.1 | DL029833.1 | GN059785.1 | AY659077.1 | HC325483.1 | GM661775.1 |
| AX036027.1 | HM124449.1 | HV245296.1 | FB509267.1 | DL018306.1 | GN051253.1 | AY659045.1 | HC325068.1 | GM661743.1 |
| AX035975.1 | FW572358.1 | HV309225.1 | FB509235.1 | DL018274.1 | GN059184.1 | AY659013.1 | HC324535.1 | GM654749.1 |
| AX035432.1 | HI564239.1 | HV302960.1 | GM675064.1 | DL018242.1 | DM023753.1 | FZ429162.1 | HC324122.1 | GM654717.1 |
| AX029039.1 | HI564114.1 | HV312652.1 | FB509220.1 | DL018210.1 | DM021911.1 | FZ437145.1 | HC323215.1 | GM654685.1 |
| AX028719.1 | HI583101.1 | HI001037.1 | FB509118.1 | DL005429.1 | DM016037.1 | FZ428186.1 | HC315622.1 | GM654653.1 |
| AX027720.1 | HI563638.1 | HI001003.1 | GM827570.1 | DJ491549.1 | DM026462.1 | FZ423024.1 | HC314438.1 | GM647595.1 |
| AX024594.1 | HI563606.1 | HI000956.1 | GM674197.1 | DJ446848.1 | DM026238.1 | FZ416167.1 | HC313943.1 | GM647563.1 |
| AX023766.1 | HI563571.1 | HI002859.1 | FB508991.1 | DJ446206.1 | GN045163.1 | FZ415478.1 | HC320351.1 | CS728549.1 |
| AX023651.1 | HI544256.1 | HI002827.1 | FB508959.1 | DJ432659.1 | GN041516.1 | FZ421416.1 | HC319663.1 | CS727221.1 |
| AX023619.1 | HI000028.1 | HI002789.1 | GM709021.1 | DJ444661.1 | GN039775.1 | FZ421068.1 | HC318794.1 | GM036651.1 |
| AX023585.1 | HI003667.1 | HI002729.1 | GM708969.1 | DJ444595.1 | GN034808.1 | FZ417551.1 | HC318090.1 | FB707952.1 |
| AX023234.1 | HI001802.1 | HI002644.1 | GM655018.1 | DJ444556.1 | GN030700.1 | FZ416908.1 | HC316429.1 | GM836390.1 |

| | | | | | | | | |
|---|---|---|---|---|---|---|---|---|
| AX019627.1 | HI001759.1 | HI002610.1 | DL121770.1 | DJ434788.1 | GN030636.1 | FZ419848.1 | HC310321.1 | GM888540.1 |
| HW260480.1 | HI469537.1 | HI002571.1 | DL121738.1 | BD251207.1 | GN030604.1 | FZ419815.1 | HC310106.1 | GM869013.1 |
| HW260448.1 | HI544124.1 | HI002506.1 | DL121706.1 | BD249870.1 | GN030540.1 | FW590723.1 | HC310041.1 | FB728427.1 |
| HW260416.1 | HI465296.1 | HI004451.1 | DL121674.1 | BD248282.1 | GN030508.1 | FW590629.1 | HC310009.1 | GM680777.1 |
| HW260384.1 | HI001643.1 | HI002414.1 | DL121642.1 | BD247393.1 | GN030396.1 | FW590295.1 | HC309977.1 | GM618702.1 |
| HW260352.1 | HI001601.1 | HI002346.1 | DL121610.1 | BD246966.1 | GN030453.1 | FW588456.1 | HC309750.1 | CS603102.1 |
| HW260320.1 | HI001505.1 | HI002301.1 | DL087783.1 | BD243814.1 | GN030380.1 | HQ161061.1 | HC308992.1 | CS603070.1 |
| HW260288.1 | HI003380.1 | HI000504.1 | DL087751.1 | BD243467.1 | GN030284.1 | HI918290.1 | HC307875.1 | CS603038.1 |
| HW260256.1 | HI003342.1 | HI202821.1 | DL087719.1 | BD243431.1 | GN030252.1 | HI918258.1 | HC307806.1 | CS603006.1 |
| HW260224.1 | HI003303.1 | HI177963.1 | DL087687.1 | BD242669.1 | GN030220.1 | HI916849.1 | HC307383.1 | CS602972.1 |
| HW260192.1 | HI550415.1 | HI214581.1 | DL087655.1 | BD242449.1 | GN029802.1 | HI657169.1 | HC312267.1 | CS602940.1 |
| HW260160.1 | HI464754.1 | HI214549.1 | DL087623.1 | BD238374.1 | GN013586.1 | HI655090.1 | FU258278.1 | CS602908.1 |
| HW260096.1 | HI464716.1 | HI179891.1 | DL087591.1 | BD236968.1 | GN012525.1 | HI660318.1 | FU258246.1 | CS602876.1 |
| HW260064.1 | HI464684.1 | HI214451.1 | DL125490.1 | BD235953.1 | GN033536.1 | FW503003.1 | FU258214.1 | CS602844.1 |
| HW260032.1 | HI464652.1 | HI214419.1 | DL125458.1 | BD235738.1 | GM648732.1 | FW498002.1 | FU258182.1 | CS602812.1 |
| HW260000.1 | HI464593.1 | HI213812.1 | DL125426.1 | BD233939.1 | GM648700.1 | FW504052.1 | FU258150.1 | CS602780.1 |
| HW259968.1 | HI505028.1 | HI185494.1 | DL125394.1 | BD232452.1 | GM648668.1 | FW504753.1 | FU255027.1 | CS602748.1 |
| HW259936.1 | HI504998.1 | HI213210.1 | DL125362.1 | BD231833.1 | GM648636.1 | FW503561.1 | FU258094.1 | CS602716.1 |
| HW259740.1 | HI463140.1 | HI212984.1 | DL121573.1 | BD231157.1 | GM648604.1 | FW500534.1 | FU258062.1 | CS602684.1 |
| HW258839.1 | HI462587.1 | HI546336.1 | DL121541.1 | BD227114.1 | GM634452.1 | FW420578.1 | FU258030.1 | CS602172.1 |
| HW257355.1 | HH725185.1 | HI546304.1 | DL121509.1 | BD226841.1 | GM634324.1 | FW420464.1 | FU257965.1 | DD106657.1 |
| HW257323.1 | HH714035.1 | HI586945.1 | DL121477.1 | BD224632.1 | GM629465.1 | FW420400.1 | FU260934.1 | DD091019.1 |
| HW257291.1 | HH961371.1 | HI210982.1 | DL121445.1 | BD223241.1 | AY658847.1 | FW503501.1 | FU260737.1 | DD118100.1 |
| HW257227.1 | HH961339.1 | HI210950.1 | DL121413.1 | BD222645.1 | AY658815.1 | FW501716.1 | FU257906.1 | DD090299.1 |
| HW257195.1 | HH961307.1 | HI473085.1 | DL117201.1 | BD222072.1 | AY658783.1 | FW496908.1 | FU257874.1 | DD089932.1 |
| HW257131.1 | HH961275.1 | HI472923.1 | DL117169.1 | BD218141.1 | AY658751.1 | FW420375.1 | FU257842.1 | DD057938.1 |
| HW257067.1 | HH961243.1 | HI210926.1 | DL117137.1 | BD204977.1 | AY658719.1 | FW420343.1 | FU257810.1 | DD057906.1 |
| HW256971.1 | HH964271.1 | HI580537.1 | DL117105.1 | DD283535.1 | AY658687.1 | HI654066.1 | FU257745.1 | DD057874.1 |
| HW256907.1 | HH981945.1 | HI209400.1 | DL117073.1 | DD282404.1 | AY658655.1 | HI653565.1 | FU260841.1 | DD054757.1 |
| HW256843.1 | HH998103.1 | HI004341.1 | DL117041.1 | DD288364.1 | AY658623.1 | HI653513.1 | FU260953.1 | DD052286.1 |
| HW251205.1 | HH997996.1 | HI004277.1 | DL112453.1 | CS356326.1 | AY658591.1 | HI653401.1 | FU262759.1 | DD052228.1 |
| HW251063.1 | HH997937.1 | HI002260.1 | DL112421.1 | CS355953.1 | AY658559.1 | HI653343.1 | FU265351.1 | DD059284.1 |
| HW250263.1 | HH999646.1 | HI002213.1 | DL112389.1 | CS353176.1 | AY658527.1 | HI653198.1 | FU262668.1 | DD058545.1 |
| HW249712.1 | HH997897.1 | HC035646.1 | DL112357.1 | CS352517.1 | AY658495.1 | HI651895.1 | FU250484.1 | DD041907.1 |
| HW249640.1 | HH997816.1 | HC021035.1 | DL112325.1 | CS352396.1 | AY658463.1 | HI651032.1 | FU262468.1 | DD048079.1 |
| HW247841.1 | HH997760.1 | HC025520.1 | DL112293.1 | CS283926.1 | AY658431.1 | HI650446.1 | FU265036.1 | DD042877.1 |
| HW240803.1 | HH999494.1 | HC025488.1 | DL107279.1 | DD206912.1 | AY658399.1 | AY710294.1 | FU262077.1 | DD030009.1 |
| HW240715.1 | HH999441.1 | HC025456.1 | DL107247.1 | DD206880.1 | AY658367.1 | HH804839.1 | FU264312.1 | DD037271.1 |
| HW240651.1 | HH999399.1 | HC025424.1 | DL107215.1 | DD206848.1 | AY658335.1 | HH802660.1 | FU269790.1 | DD027316.1 |
| HW241316.1 | HH997693.1 | HC007959.1 | DL107183.1 | DD206816.1 | AY658303.1 | HH797209.1 | GN360569.1 | DD026705.1 |
| HW239394.1 | HH997639.1 | HC007662.1 | DL107151.1 | CS250100.1 | AY658271.1 | FW369077.1 | GN366885.1 | DD023887.1 |
| HW239175.1 | HC047029.1 | HC010671.1 | DL107119.1 | DD181012.1 | AY658239.1 | FW379604.1 | GN360135.1 | DD023796.1 |
| HW238755.1 | HC046965.1 | DM375809.1 | DL102506.1 | DD173364.1 | AY658207.1 | FW377075.1 | GN360103.1 | DD028680.1 |
| HW238562.1 | HC046933.1 | DM375336.1 | DL102474.1 | CS023788.1 | AY658175.1 | FW381495.1 | GN356184.1 | DD229868.1 |
| HW238491.1 | HC046901.1 | DM371034.1 | DL102442.1 | CS018739.1 | AY658143.1 | FW375407.1 | CS623734.1 | DD227768.1 |
| HW238404.1 | HC046873.1 | DM370678.1 | DL102410.1 | CS018320.1 | AY658111.1 | FW375288.1 | CS623664.1 | DD224654.1 |
| HW242956.1 | HC046841.1 | DM370646.1 | DL102378.1 | CS017707.1 | AY658079.1 | FW375232.1 | CS623622.1 | DD224168.1 |

| | | | | | | | | |
|---|---|---|---|---|---|---|---|---|
| HW237949.1 | HC046809.1 | DM370614.1 | DL102346.1 | CS016573.1 | AY658047.1 | FW369627.1 | CS627116.1 | DD223871.1 |
| HV802943.1 | HC046777.1 | DM370582.1 | DL097733.1 | CS016271.1 | AY658015.1 | FW369459.1 | DD450370.1 | DD233570.1 |
| HV802911.1 | HC046745.1 | DM370550.1 | DL097701.1 | CQ990425.1 | AY657983.1 | HH736082.1 | DD450333.1 | DD231566.1 |
| HV802879.1 | HC046713.1 | DM370518.1 | DL097669.1 | CQ986621.1 | AY657951.1 | HH735908.1 | DD450301.1 | DD231406.1 |
| HV813715.1 | HC039630.1 | DM370486.1 | DL097637.1 | CQ986557.1 | AY657887.1 | HH733742.1 | DD450269.1 | CS273079.1 |
| HV818568.1 | HC046061.1 | DM371995.1 | DL097605.1 | CQ985778.1 | AY657855.1 | HD119649.1 | DD450237.1 | A01106.1 |
| HV817758.1 | HC046029.1 | DM371305.1 | DL097573.1 | CQ981111.1 | AY657823.1 | FW368174.1 | DD450130.1 | DD221291.1 |
| HV784493.1 | HC036978.1 | DM209286.1 | DL097541.1 | CQ979565.1 | AY657791.1 | FW363500.1 | DD438008.1 | DD214016.1 |
| HV778615.1 | HC025523.1 | HC003069.1 | DL095514.1 | CQ972388.1 | AY657759.1 | FW367202.1 | DD448963.1 | DD216622.1 |
| HV777258.1 | HC025459.1 | HB865036.1 | DL095482.1 | CQ972356.1 | AY657727.1 | FU264494.1 | DD441524.1 | DD213906.1 |
| HV775767.1 | HC025427.1 | HB864968.1 | DL095450.1 | CQ971787.1 | AY657695.1 | HC313239.1 | DD436985.1 | DD213874.1 |
| HV774926.1 | HC010662.1 | HB864996.1 | DL095418.1 | CQ971112.1 | AY657663.1 | HC299773.1 | DD435322.1 | DD213842.1 |
| HV764706.1 | HC010413.1 | HB864940.1 | DL095386.1 | CQ967268.1 | AY657631.1 | HC306237.1 | CS620855.1 | DD213810.1 |
| HV774548.1 | HC009024.1 | HB864908.1 | DL095354.1 | AX675242.1 | AY657599.1 | HC306197.1 | CS620449.1 | DD216485.1 |
| HV774477.1 | HC007665.1 | HB864876.1 | DL091540.1 | BD397242.1 | AY657567.1 | HC302643.1 | CS620258.1 | DD216443.1 |
| HV764247.1 | HC007541.1 | HB864844.1 | DL091508.1 | BD439112.1 | AY657535.1 | HC305929.1 | CS619979.1 | DD213793.1 |
| HV761141.1 | HC010674.1 | HB864812.1 | DL091476.1 | BD428749.1 | AY657503.1 | HC305889.1 | CS618045.1 | DD213761.1 |
| HV766934.1 | DM375812.1 | HB864780.1 | DL091444.1 | BD428464.1 | AY657471.1 | HC305849.1 | CS622390.1 | DD213729.1 |
| HV766775.1 | DM375339.1 | HB864748.1 | DL091412.1 | BD427307.1 | AY657439.1 | HC305809.1 | CS617095.1 | DD213697.1 |
| DL123220.1 | HC005706.1 | GM709029.1 | DL091380.1 | BD427092.1 | AY657407.1 | HC305769.1 | CS616561.1 | DD213665.1 |
| DL123188.1 | DM371082.1 | GM708997.1 | DL087583.1 | BD426722.1 | AY657375.1 | HC305729.1 | CS613952.1 | DD213633.1 |
| DL118902.1 | GN033504.1 | GM834843.1 | DL087551.1 | BD394365.1 | AY657343.1 | HC305689.1 | CS613802.1 | DD213601.1 |
| DL118870.1 | GN033440.1 | GM645483.1 | DL087519.1 | BD453372.1 | AY657311.1 | HC305649.1 | CS612713.1 | DD213583.1 |
| DL118838.1 | GN033344.1 | GM645451.1 | DL087487.1 | BD453336.1 | AY657279.1 | HC302008.1 | CS604522.1 | DD213551.1 |
| DL123163.1 | GN033312.1 | GM645419.1 | DL087455.1 | BD453308.1 | AY657247.1 | HC294266.1 | CS604490.1 | DD213498.1 |
| DL109254.1 | GN033280.1 | GM633763.1 | DL087423.1 | BD453276.1 | AY657215.1 | HC251280.1 | CS604458.1 | DD220648.1 |
| DL109222.1 | GN033248.1 | GM633731.1 | DL087391.1 | BD393235.1 | AY657183.1 | HC293990.1 | CS604426.1 | DD216031.1 |
| DL109190.1 | GN033184.1 | GM633699.1 | DL112233.1 | BD402360.1 | AY657151.1 | HC289384.1 | CS604362.1 | DD215999.1 |
| DL109158.1 | GN033088.1 | FB506769.1 | DL112201.1 | BD388697.1 | AY657119.1 | HC289340.1 | CS604330.1 | DD215967.1 |
| DL109126.1 | GN033024.1 | GM649950.1 | DL112169.1 | BD376272.1 | AY657087.1 | HC292948.1 | CS604266.1 | DD218331.1 |
| DL109094.1 | GN032960.1 | GM642982.1 | DL112137.1 | BD375423.1 | AY657055.1 | HC295995.1 | CS603786.1 | DD215921.1 |
| DL104360.1 | GN032928.1 | GM621674.1 | DL112105.1 | BD356279.1 | L23212.1 | HC295707.1 | CS208840.1 | DD215857.1 |
| DL104328.1 | GN032832.1 | GM621642.1 | DL112073.1 | BD353339.1 | M10653.1 | HC288898.1 | CS223194.1 | DD215825.1 |
| DL025352.1 | GN032800.1 | GM647266.1 | DL107070.1 | BD341810.1 | M30813.1 | HC292518.1 | CS203615.1 | DD215793.1 |
| DL025320.1 | GN032768.1 | GM647234.1 | DL107038.1 | CQ802826.1 | K00899.1 | HC292278.1 | CS207928.1 | DD212302.1 |
| DL025288.1 | GN032737.1 | GM647202.1 | DL107006.1 | CQ801299.1 | J02204.1 | HC295020.1 | CS207857.1 | DD192495.1 |
| DL025224.1 | GN032705.1 | GM714583.1 | DL106974.1 | CQ800905.1 | M16483.1 | HW155024.1 | CS193661.1 | DD211109.1 |
| DL021884.1 | GN032673.1 | GM647170.1 | DL106942.1 | CQ795474.1 | DM384087.1 | HW154499.1 | CS188664.1 | DD200178.1 |
| DL021820.1 | GN032640.1 | GM647013.1 | DL106910.1 | CQ795442.1 | DM381899.1 | HW154467.1 | CS188632.1 | DD208185.1 |
| DL021788.1 | GN032608.1 | GM646871.1 | DL102297.1 | CQ792511.1 | HC055547.1 | HW147457.1 | CS186216.1 | DD187260.1 |
| DL038458.1 | GN032576.1 | GM646839.1 | DL102265.1 | CQ788629.1 | HC053256.1 | HW158130.1 | CS182531.1 | DD206854.1 |
| DL038426.1 | GN032545.1 | DM016016.1 | DL102233.1 | CQ787473.1 | HC045302.1 | HW153783.1 | CS183906.1 | DD206822.1 |
| DL038394.1 | GN032449.1 | GN043710.1 | DL102225.1 | CQ787439.1 | HC047476.1 | HW153384.1 | CS183829.1 | CQ816970.1 |
| DL038362.1 | GN032385.1 | GN042981.1 | DL102169.1 | CQ787361.1 | HC047028.1 | HW157488.1 | CS177786.1 | CQ816932.1 |
| DL013167.1 | GN032352.1 | GN042230.1 | DL102137.1 | CQ784701.1 | HC046996.1 | HW151799.1 | CS174734.1 | CQ815741.1 |
| DL013103.1 | GN032320.1 | GN030758.1 | DL117005.1 | CQ784637.1 | HC046964.1 | HW151067.1 | CS174632.1 | CQ814091.1 |
| DL013039.1 | GN032288.1 | GN030662.1 | DL116973.1 | CQ778523.1 | HC046932.1 | HW072899.1 | CS176092.1 | CQ814059.1 |

| | | | | | | | | |
|---|---|---|---|---|---|---|---|---|
| DL013007.1 | GN032256.1 | GN030402.1 | DL116941.1 | CQ778491.1 | HC046900.1 | HW096369.1 | CS172443.1 | CQ814026.1 |
| DL033742.1 | GN032224.1 | GN030278.1 | DL105039.1 | CQ774812.1 | HC046872.1 | HW105066.1 | CS173283.1 | CQ813994.1 |
| DL029736.1 | GN032192.1 | GN030246.1 | DL105007.1 | CQ772352.1 | HC046840.1 | HW096208.1 | CS165436.1 | CQ813962.1 |
| DL042144.1 | GN032160.1 | GN030214.1 | DL104975.1 | CQ759690.1 | HC046808.1 | HW104296.1 | CS161523.1 | CQ813930.1 |
| DL042112.1 | GN032128.1 | GM634543.1 | DL104943.1 | CQ758867.1 | HC046776.1 | HW070921.1 | CS159778.1 | CQ813898.1 |
| DL042080.1 | GN032096.1 | GM634511.1 | DL104911.1 | CQ756598.1 | HC046744.1 | HW103984.1 | CS159589.1 | CQ813866.1 |
| DL029533.1 | GN032064.1 | GM634479.1 | DL029711.1 | CQ754027.1 | HC046712.1 | HW103746.1 | CS159215.1 | CQ813834.1 |
| DL029501.1 | GN032032.1 | GM648726.1 | DL029679.1 | AX958097.1 | HC039629.1 | HW099582.1 | CS148785.1 | CQ813802.1 |
| DL029469.1 | GN032000.1 | GM648694.1 | DL029647.1 | AX600857.1 | HC046092.1 | HW099524.1 | CS145755.1 | CQ813729.1 |
| DL044532.1 | GN031968.1 | GM648662.1 | DL029615.1 | AX598960.1 | HC046060.1 | HW067330.1 | CS144369.1 | CQ803127.1 |
| DL044500.1 | GN031936.1 | GM648630.1 | DL029583.1 | AX594164.1 | HC046028.1 | HW088927.1 | CS141939.1 | CQ802128.1 |
| DL044468.1 | GN031904.1 | GM648598.1 | DL025542.1 | AX593506.1 | HB856287.1 | HW083221.1 | CS141536.1 | DL025285.1 |
| DL036604.1 | GN031872.1 | GM619890.1 | DL025510.1 | BD166159.1 | HB855970.1 | HW088255.1 | CS141504.1 | DL025221.1 |
| DL036572.1 | GN031840.1 | DL106947.1 | DL025478.1 | BD161779.1 | HB855730.1 | HW103588.1 | CS141244.1 | DL017419.1 |
| DL036540.1 | GN031710.1 | DL106915.1 | DL025446.1 | AX576539.1 | DM170840.1 | HW103516.1 | CS140738.1 | DL038455.1 |
| DL032241.1 | GN031678.1 | DL111941.1 | DL025414.1 | AX573757.1 | DM170808.1 | HW103162.1 | CS139108.1 | DL039384.1 |
| DL032209.1 | GN031646.1 | DL101953.1 | DL025382.1 | AX573488.1 | DM188003.1 | HW103129.1 | CS134725.1 | DL039352.1 |
| DL032177.1 | GN031614.1 | DL128486.1 | DL033940.1 | AX565545.1 | DM187954.1 | HW102796.1 | CS131929.1 | DL039320.1 |
| DL048447.1 | GN031550.1 | DL101210.1 | DL025329.1 | AX556845.1 | DM177483.1 | HW102764.1 | CS123624.1 | DL035664.1 |
| DL048415.1 | GN031518.1 | DL086777.1 | DL025297.1 | AX556811.1 | HB559437.1 | HW102303.1 | CS123359.1 | DL035632.1 |
| DL048383.1 | GN031486.1 | DL089919.1 | DL025265.1 | AX555658.1 | HB491550.1 | HW061844.1 | CS122776.1 | DL035600.1 |
| DL048351.1 | GN031389.1 | DL092278.1 | DL025233.1 | AX544011.1 | HB489350.1 | HW065771.1 | CS122285.1 | DL035568.1 |
| DL048319.1 | GN031325.1 | DL092246.1 | DL025201.1 | AX537319.1 | HB488941.1 | HV575312.1 | CS106194.1 | DL035536.1 |
| DL044337.1 | GN031292.1 | DL092214.1 | DL025169.1 | AX528968.1 | HB488407.1 | HV586235.1 | CS106102.1 | DL035504.1 |
| DL044305.1 | GN031260.1 | DL092182.1 | DL021957.1 | AX528452.1 | DM163994.1 | HV570879.1 | AX958357.1 | DL031500.1 |
| DL044273.1 | GN031196.1 | DL000696.1 | DL021925.1 | A33928.1 | DM163961.1 | HV566139.1 | CS179804.1 | DL031468.1 |
| DL040097.1 | GN031131.1 | DJ493855.1 | DL021893.1 | AX179527.1 | DM163578.1 | HV573966.1 | CS179632.1 | DL031436.1 |
| DL032040.1 | GN031099.1 | CS602892.1 | DL021861.1 | AX179466.1 | GN031999.1 | HV558938.1 | CS179197.1 | DL031404.1 |
| DL032008.1 | GN031067.1 | CS602860.1 | DL021797.1 | AX164103.1 | HH977175.1 | HV555940.1 | CS328409.1 | DL031372.1 |
| DL031976.1 | GN031035.1 | CS602828.1 | DL017591.1 | AX028012.1 | HH980620.1 | HV554548.1 | CS326364.1 | DL023559.1 |
| DL028135.1 | GN030971.1 | CS608396.1 | DL017559.1 | AX027980.1 | HH980516.1 | HV550393.1 | CS332057.1 | DL023527.1 |
| DL028103.1 | GN030940.1 | CS607032.1 | DL017495.1 | AX003222.1 | HH980448.1 | HV550344.1 | CS323603.1 | DL023495.1 |
| DL028071.1 | GN030876.1 | CS606944.1 | DL017463.1 | A20357.1 | HH980375.1 | AY659395.1 | CS323371.1 | DL023463.1 |
| DL028007.1 | GN030844.1 | CS606855.1 | DL038467.1 | AX521665.1 | HH980343.1 | AY659363.1 | CS323310.1 | DL023431.1 |
| DL027975.1 | GN030812.1 | CS604615.1 | DL038435.1 | AX512658.1 | HH980283.1 | AY659331.1 | CS323258.1 | DL023399.1 |
| DL036389.1 | GN030780.1 | CS593001.1 | DL038403.1 | HW351430.1 | HH980181.1 | AY659299.1 | CS323168.1 | DL020384.1 |
| DL036357.1 | DL094232.1 | CS589288.1 | DL038371.1 | HW350966.1 | HH980143.1 | AY659267.1 | CS330846.1 | DL020320.1 |
| DL036325.1 | DL094200.1 | CS585202.1 | DL038339.1 | HW350890.1 | HH980073.1 | AY659235.1 | CS330384.1 | DL015821.1 |
| DL036293.1 | DL094168.1 | CS362758.1 | DL013176.1 | HW350667.1 | HH996690.1 | AY659203.1 | CS329311.1 | DL015789.1 |
| DL044240.1 | DL124297.1 | CS361279.1 | DL013144.1 | HW340092.1 | HH996627.1 | HQ161059.1 | CS320118.1 | DL015757.1 |
| DL044208.1 | DL110158.1 | CS359953.1 | DL013112.1 | HW350316.1 | HH996567.1 | FW577310.1 | CS319615.1 | DL015725.1 |
| DL044176.1 | DL110126.1 | CS359723.1 | DL013080.1 | HW350169.1 | HH998515.1 | FW576619.1 | CS322156.1 | DL043073.1 |
| DL048249.1 | DL110094.1 | BD269156.1 | DL013016.1 | HW349930.1 | HH998384.1 | FW575550.1 | DD271426.1 | DL039256.1 |
| DL048217.1 | DL105161.1 | BD269124.1 | DL033879.1 | HW348576.1 | HH998351.1 | FW573644.1 | DD261141.1 | DL035472.1 |
| DL048185.1 | DL115126.1 | BD268880.1 | DL033847.1 | HW347891.1 | HH999975.1 | FW573455.1 | DD259355.1 | DL035440.1 |
| DL048153.1 | DL110062.1 | BD265635.1 | DL033815.1 | HW363666.1 | HH999922.1 | FW574612.1 | DD259183.1 | DL035408.1 |
| DL048121.1 | DL029521.1 | BD265579.1 | DL033783.1 | HW339006.1 | HH979777.1 | HI968053.1 | DD272168.1 | DL035376.1 |

| | | | | | | | | |
|---|---|---|---|---|---|---|---|---|
| DL048089.1 | DL029489.1 | BD263434.1 | DL033751.1 | HW338550.1 | HH979690.1 | HI967872.1 | CS305324.1 | DL035344.1 |
| DL048057.1 | DL049698.1 | BD251789.1 | DL033719.1 | HW338422.1 | HH999889.1 | HI979339.1 | CS001494.1 | DL031180.1 |
| DL048026.1 | DL049666.1 | BD251248.1 | DL042340.1 | HW337910.1 | HH999796.1 | HI987458.1 | CS001430.1 | DL031148.1 |
| DL047994.1 | DL044260.1 | BD250868.1 | DL042249.1 | HW337526.1 | HH999743.1 | HI987421.1 | FW359999.1 | DL027243.1 |
| DL047930.1 | DL044196.1 | BD247927.1 | DL042217.1 | HW337398.1 | HH998315.1 | HI935273.1 | FW359911.1 | DL027211.1 |
| DL047898.1 | DL044164.1 | BD247123.1 | DL042185.1 | HW337270.1 | HH998264.1 | HI934942.1 | FW359851.1 | DL027179.1 |
| DL047866.1 | DL043668.1 | BD247026.1 | DL042153.1 | HW337142.1 | HH998227.1 | HI918288.1 | FW359679.1 | DL023367.1 |
| DL040067.1 | DL027870.1 | BD246951.1 | DL042089.1 | HW336886.1 | HH996539.1 | HI918256.1 | FW368690.1 | DL023335.1 |
| DL040035.1 | DL027838.1 | BD244501.1 | DL046436.1 | HW336602.1 | HH993660.1 | HI660829.1 | FW363797.1 | DL023303.1 |
| DL040003.1 | DL027806.1 | BD242439.1 | DL046404.1 | HW329405.1 | HH986621.1 | HI659097.1 | FW368152.1 | DL023271.1 |
| DL039971.1 | DL027774.1 | BD241009.1 | DL046372.1 | HW329285.1 | HH979508.1 | HI657154.1 | HC196132.1 | DL023239.1 |
| DL039939.1 | DL023866.1 | BD237993.1 | DL046340.1 | HW336152.1 | HH979617.1 | HI660316.1 | HC195239.1 | DL023207.1 |
| DL039907.1 | DL023802.1 | BD236923.1 | DL046308.1 | HW328840.1 | HH979575.1 | FW497999.1 | HC194250.1 | DL023175.1 |
| DJ328052.1 | DD450573.1 | BD235678.1 | DL046276.1 | HW328547.1 | HH931979.1 | FW503730.1 | HC193976.1 | CS597724.1 |
| DJ326983.1 | DD449165.1 | BD235394.1 | DL027584.1 | HW328425.1 | FW394287.1 | FW499019.1 | HC190396.1 | CS593358.1 |
| DJ326594.1 | CS618819.1 | BD233673.1 | DL027552.1 | HW319205.1 | FW394230.1 | FW498883.1 | HC187661.1 | CS582399.1 |
| DJ128226.1 | CS617770.1 | BD231748.1 | DL023740.1 | HW328265.1 | FW394194.1 | FW420576.1 | HC187076.1 | CS576391.1 |
| DJ122472.1 | CS616584.1 | BD231146.1 | DL023708.1 | HW328152.1 | DL464620.1 | FW420430.1 | HC090218.1 | CS576667.1 |
| DJ088764.1 | CS616229.1 | BD229997.1 | DL023676.1 | HW318568.1 | DL464537.1 | FW420373.1 | HC089559.1 | CS565541.1 |
| DJ082567.1 | CS614353.1 | BD227084.1 | DL023644.1 | HW318452.1 | DL463057.1 | FW420341.1 | HC089527.1 | CS569610.1 |
| DJ081973.1 | CS612723.1 | BD226831.1 | DL023580.1 | AX799561.1 | DL462995.1 | FW496646.1 | HC089495.1 | CS561177.1 |
| DJ086345.1 | CS611723.1 | BD224575.1 | DL020565.1 | AX798984.1 | DL462895.1 | HI653921.1 | HC089463.1 | CS559090.1 |
| DJ081540.1 | CS604500.1 | BD223705.1 | DL020533.1 | AX798498.1 | FB504557.1 | HI653399.1 | HC089431.1 | DD420049.1 |
| DJ080760.1 | CS603476.1 | BD223023.1 | DL020469.1 | AX798164.1 | GM831917.1 | HI653329.1 | HC089398.1 | DD421786.1 |
| DJ069491.1 | CS603444.1 | BD205925.1 | DL020437.1 | AX472470.1 | GM712409.1 | HI653196.1 | HC089366.1 | DD431823.1 |
| FB712557.1 | CS603412.1 | DD283686.1 | DL020405.1 | BD194478.1 | GM743319.1 | HI651891.1 | HC089333.1 | CS539995.1 |
| GM721286.1 | CS603348.1 | CS050960.1 | DL016002.1 | BD191696.1 | FB509197.1 | HI651017.1 | HC089301.1 | CS543247.1 |
| GM060291.1 | CS603156.1 | CS024018.1 | DL015970.1 | AX781452.1 | FB509165.1 | AY383629.1 | HC089269.1 | CS542546.1 |
| GM841718.1 | DD400178.1 | CS018507.1 | DL015874.1 | AX773267.1 | FB509129.1 | HH821123.1 | HC086574.1 | CS531842.1 |
| GM604028.1 | DD400145.1 | CS016563.1 | DL015842.1 | AX767208.1 | GM709129.1 | HH827355.1 | HC086287.1 | CS537865.1 |
| GM603693.1 | CS460034.1 | CS007961.1 | DL011384.1 | AX766561.1 | GM043991.1 | FW369073.1 | HC085674.1 | CS537399.1 |
| GM706383.1 | DD361276.1 | CQ986611.1 | DL011352.1 | AX754681.1 | GM650486.1 | FW378019.1 | AY687640.1 | CS534832.1 |
| GM603495.1 | DD367536.1 | CQ981101.1 | DL011320.1 | AX752752.1 | GM631832.1 | FW381493.1 | AY774965.1 | CS502399.1 |
| FB742331.1 | DD362654.1 | AX708719.1 | DL011288.1 | AX746470.1 | GM631800.1 | FW375402.1 | AY774919.1 | CS498364.1 |
| GM601145.1 | DD361623.1 | AX705406.1 | DL011256.1 | BD184757.1 | GM622837.1 | FW369388.1 | AY774869.1 | CS497136.1 |
| FB715286.1 | DD368138.1 | AX703497.1 | DL011224.1 | BD181491.1 | GM646872.1 | HH736080.1 | AY774751.1 | DD418609.1 |
| FB715071.1 | CS457175.1 | AX685813.1 | DL047648.1 | BD181203.1 | GM646840.1 | HH735906.1 | AY774635.1 | DD418574.1 |
| FB677406.1 | CS456721.1 | AX683733.1 | DL047616.1 | BD180851.1 | GM646808.1 | HH733738.1 | AY774578.1 | DD412324.1 |
| GM061240.1 | CS456075.1 | AX665922.1 | DL047584.1 | BD180763.1 | GM627175.1 | FW368172.1 | AY774409.1 | DD411933.1 |
| DL460016.1 | CS453773.1 | AX664357.1 | DL047552.1 | AX743754.1 | GM627143.1 | FW367386.1 | AY774350.1 | CS376125.1 |
| DL459977.1 | CS450572.1 | AX663010.1 | DL047520.1 | AY181091.1 | GM646476.1 | FW366500.1 | AY774302.1 | CS375988.1 |
| DL262705.1 | DD357908.1 | AX659121.1 | DL047488.1 | BD177540.1 | GM646444.1 | FW351379.1 | AY774248.1 | CS375792.1 |
| DL260581.1 | CS448870.1 | AX657125.1 | DL043538.1 | BD177300.1 | GM646412.1 | FW394389.1 | AY774063.1 | CS362713.1 |
| DL259821.1 | CS447268.1 | AX657090.1 | DL039657.1 | AX720727.1 | GM626870.1 | FW394233.1 | AY773996.1 | CS360564.1 |
| DL258265.1 | CS446158.1 | BD175041.1 | DL039625.1 | AX717719.1 | GM626838.1 | FW394197.1 | HC084633.1 | CS359738.1 |
| DL241177.1 | CS442967.1 | BD174691.1 | DL039593.1 | AX717356.1 | GM626806.1 | FW397473.1 | HC083882.1 | BD269139.1 |
| DL241037.1 | CS438914.1 | AX642061.1 | DL039561.1 | AX708731.1 | DL095427.1 | FW418232.1 | HC083737.1 | BD268909.1 |

| | | | | | | | | |
|---|---|---|---|---|---|---|---|---|
| FB674304.1 | CS436014.1 | GM628468.1 | DL039529.1 | AX705317.1 | DL095363.1 | FW396289.1 | HC083528.1 | BD268160.1 |
| DL125447.1 | CS434849.1 | GM628404.1 | DL039497.1 | AX703573.1 | DL128840.1 | FW396663.1 | DM460854.1 | BD268117.1 |
| DL125415.1 | DD347407.1 | GM628372.1 | DL047323.1 | AX006720.1 | DL128808.1 | HH936042.1 | HC061847.1 | BD267561.1 |
| DL125383.1 | DD353778.1 | GM628340.1 | DL047291.1 | AX003022.1 | DL111654.1 | FU756125.1 | HC061377.1 | BD266623.1 |
| DL125351.1 | DD353676.1 | GM628308.1 | DL047259.1 | AJ286130.1 | DL111622.1 | FU772126.1 | HC059931.1 | BD265610.1 |
| DL117126.1 | CS426812.1 | GM662113.1 | DL043405.1 | A57335.1 | DL111590.1 | FU773355.1 | HC059665.1 | BD264407.1 |
| DL102495.1 | CS425052.1 | GM662081.1 | DL043373.1 | A44216.1 | DL097042.1 | FU758410.1 | HC059236.1 | BD263449.1 |
| DL102431.1 | CS424095.1 | GM662049.1 | DL043341.1 | A25434.1 | DL128613.1 | FU757782.1 | HC058881.1 | BD262925.1 |
| DL102399.1 | CS423186.1 | GM662017.1 | DL043309.1 | A35727.1 | DL125147.1 | FU757625.1 | HC058381.1 | BD261814.1 |
| DL102367.1 | CS422812.1 | GM661985.1 | DL043277.1 | A35302.1 | DL123334.1 | FU757298.1 | HC058105.1 | BD251958.1 |
| DL102335.1 | BD395931.1 | GM661953.1 | DL039460.1 | A29460.1 | DL118705.1 | HC441394.1 | HC057895.1 | BD251212.1 |
| DL097626.1 | BD437683.1 | GM661921.1 | DL039428.1 | A29389.1 | DL114117.1 | HC358399.1 | HC057198.1 | BD249882.1 |
| DL097594.1 | BD426713.1 | GM654831.1 | A23028.1 | A34518.1 | DL114085.1 | HC358211.1 | HB464822.1 | BD247517.1 |
| DL097562.1 | BD394364.1 | GM654799.1 | A22291.1 | A33970.1 | DL114053.1 | HC358080.1 | HB463819.1 | BD247048.1 |
| DL095535.1 | BD453371.1 | GM654767.1 | A18452.1 | A32457.1 | DL122945.1 | HC325389.1 | HB463601.1 | BD246971.1 |
| DL095503.1 | BD453335.1 | GM647774.1 | A16440.1 | A30363.1 | DL122913.1 | HC319591.1 | HB471283.1 | BD245175.1 |
| DL095439.1 | BD453307.1 | GM647742.1 | A14255.1 | A26282.1 | DL025210.1 | HC318838.1 | HB469606.1 | BD244588.1 |
| DL095407.1 | BD453275.1 | GM647710.1 | A21780.1 | A13704.1 | DL011954.1 | HC318691.1 | HB469108.1 | GN359983.1 |
| DL095375.1 | BD393215.1 | GM647678.1 | A21211.1 | A31392.1 | DL011858.1 | HC316670.1 | HB468042.1 | GN359887.1 |
| DL112254.1 | BD402329.1 | GM647646.1 | A14680.1 | A25926.1 | DL023589.1 | HC307897.1 | DM148841.1 | GN359682.1 |
| DL112222.1 | BD413435.1 | GM640811.1 | A18862.1 | A12742.1 | DL015915.1 | HC307832.1 | DM148793.1 | GN359662.1 |
| DL112190.1 | BD388669.1 | GM640778.1 | A16030.1 | A17982.1 | DL015851.1 | FU262774.1 | DM152832.1 | GN359466.1 |
| DL112158.1 | BD378213.1 | GM640746.1 | A13387.1 | A11184.1 | DL047625.1 | FU265426.1 | DM148095.1 | GN359402.1 |
| DL112126.1 | BD376267.1 | GM640714.1 | A12754.1 | A06520.1 | DL047593.1 | HB475492.1 | DM152711.1 | GN359338.1 |
| DL112094.1 | BD375701.1 | GM640682.1 | A12436.1 | A04786.1 | DL047561.1 | HB475315.1 | DM152571.1 | GN365263.1 |
| DL107091.1 | BD375405.1 | GM640650.1 | A11244.1 | A31731.1 | DL047529.1 | HB474759.1 | DM147323.1 | GN359169.1 |
| DL107059.1 | BD356505.1 | GM628296.1 | A10849.1 | A30188.1 | DL039666.1 | HB464038.1 | DM146479.1 | GN364162.1 |
| DL107027.1 | BD359626.1 | FB701525.1 | A10371.1 | A29289.1 | DL035850.1 | HB470628.1 | DM146349.1 | GN359043.1 |
| DL106995.1 | BD341809.1 | CS809113.1 | A03656.1 | A29008.1 | DL043414.1 | HB469133.1 | DM155729.1 | GN363780.1 |
| DL106963.1 | BD349229.1 | FB701781.1 | A09796.1 | A28262.1 | CS608332.1 | HB461479.1 | DM150256.1 | GN363012.1 |
| DL106931.1 | BD325697.1 | FB701105.1 | A08052.1 | A26689.1 | CS600689.1 | HB455475.1 | DM150109.1 | GN342136.1 |
| DL106899.1 | BD325495.1 | FB660591.1 | A07373.1 | A26055.1 | CS607491.1 | HB451975.1 | DM149930.1 | GN348293.1 |
| DL102158.1 | BD314345.1 | FB660053.1 | A05149.1 | A25582.1 | CS607039.1 | DM141124.1 | HB461818.1 | GN346577.1 |
| DL102126.1 | BD319277.1 | FB580561.1 | A07988.1 | A24081.1 | CS606949.1 | DM121634.1 | HB461402.1 | GN346545.1 |
| DL111957.1 | BD317868.1 | FB580593.1 | A06077.1 | A25162.1 | A21466.1 | HB435683.1 | HB455313.1 | GN340292.1 |
| DL111925.1 | BD308641.1 | FB654928.1 | A01767.1 | A14546.1 | A19564.1 | HB441217.1 | HB455091.1 | GN335345.1 |
| DL114433.1 | BD307737.1 | FB665234.1 | A05339.1 | A11921.1 | A17119.1 | HB423261.1 | HA640319.1 | GN116524.1 |
| DL114401.1 | BD300345.1 | FB654433.1 | A04277.1 | A21829.1 | A12548.1 | GM659888.1 | HB444488.1 | GN116459.1 |
| DL114369.1 | BD293533.1 | FB580359.1 | A01387.1 | A21491.1 | A10858.1 | GM659856.1 | HB446932.1 | GN116427.1 |
| DL114337.1 | CS646214.1 | FB573198.1 | A00961.1 | A20582.1 | A10545.1 | GM659824.1 | HB444516.1 | GN116395.1 |
| DL104578.1 | CS646176.1 | FB580190.1 | A00283.1 | A20082.1 | A07613.1 | GM659792.1 | DM121078.1 | GN116363.1 |
| DL104546.1 | DD459652.1 | FB580126.1 | HW290135.1 | A16543.1 | A04746.1 | GM652339.1 | DM134514.1 | GN116331.1 |
| DL104514.1 | DD454895.1 | FB580078.1 | HW288962.1 | A18695.1 | A28291.1 | GM652307.1 | DM138467.1 | GN116299.1 |
| DL093930.1 | DD458765.1 | DL200024.1 | HW274688.1 | A17100.1 | A22629.1 | GM652275.1 | DM138435.1 | GN112542.1 |
| DL093898.1 | DD453506.1 | DL189523.1 | HW269948.1 | A15955.1 | A15402.1 | GM645284.1 | DM143378.1 | GN094819.1 |
| DL126665.1 | DD456126.1 | DL199727.1 | HW269254.1 | A15449.1 | A28185.1 | GM645252.1 | DM138345.1 | GM623620.1 |
| DL123346.1 | CS631743.1 | DL194196.1 | HW268788.1 | A12197.1 | A26526.1 | GM645220.1 | DM138313.1 | GM623588.1 |

| | | | | | | | | |
|---|---|---|---|---|---|---|---|---|
| DL123314.1 | CS631248.1 | DL188826.1 | HW267806.1 | A09992.1 | A26030.1 | GM645187.1 | DM121666.1 | GM623556.1 |
| DL123282.1 | DD247329.1 | DL193877.1 | HW267635.1 | A15469.1 | A25194.1 | GM638132.1 | DM137790.1 | GM644234.1 |
| DL123250.1 | DD246863.1 | DL193800.1 | HW267393.1 | A07700.1 | A23310.1 | GM638100.1 | DM137123.1 | GM637140.1 |
| DL123218.1 | E41539.1 | DL193768.1 | HW265864.1 | A06904.1 | A24710.1 | GM638068.1 | DM131197.1 | GM637108.1 |
| DL123186.1 | E41745.1 | DL183351.1 | HW263037.1 | A05261.1 | A23296.1 | GM741827.1 | DM130794.1 | GM637076.1 |
| DL109252.1 | E40781.1 | DL183235.1 | HW262588.1 | HV961478.1 | A18475.1 | GM658309.1 | HB436406.1 | GM624923.1 |
| DL109220.1 | E36673.1 | FB513413.1 | HW261416.1 | HV960819.1 | A21789.1 | GM658277.1 | HB435560.1 | GM624891.1 |
| DL109188.1 | E35566.1 | FB513115.1 | HW261384.1 | HV964299.1 | A19604.1 | GM658245.1 | HB441236.1 | GM624859.1 |
| DL109156.1 | E33242.1 | FB571333.1 | HW261352.1 | HV959606.1 | A18864.1 | GM658213.1 | HB440983.1 | GM632674.1 |
| DL109124.1 | E38121.1 | FB571277.1 | HW261320.1 | HV964077.1 | A18405.1 | GM658099.1 | HV767903.1 | GM632642.1 |
| DL109092.1 | E06683.1 | FB570198.1 | HW261288.1 | HV959180.1 | A17967.1 | GM658067.1 | HV766729.1 | GM658452.1 |
| DL104358.1 | E05544.1 | FB571385.1 | HW261256.1 | HV963765.1 | A15902.1 | GM658035.1 | HV760620.1 | GM651172.1 |
| DL104326.1 | E04096.1 | CS696201.1 | HW261192.1 | HV963333.1 | A13389.1 | GM658003.1 | HV766530.1 | GM651140.1 |
| DL029732.1 | E03839.1 | CS696137.1 | HW261160.1 | HV965658.1 | A12756.1 | GM657971.1 | HV766314.1 | GM651108.1 |
| DL025350.1 | E03108.1 | CS695976.1 | HW261128.1 | HV969941.1 | A10854.1 | GM631835.1 | HV766048.1 | GM651044.1 |
| DL025318.1 | E02966.1 | CS695944.1 | HW261096.1 | HV965327.1 | A10373.1 | GM631803.1 | HV766016.1 | GM651012.1 |
| DL025286.1 | E02434.1 | CS695912.1 | HW261064.1 | HV961909.1 | A10277.1 | GM646647.1 | HV750373.1 | GM644213.1 |
| DL025222.1 | E02272.1 | CS695880.1 | HW261032.1 | HV956409.1 | A08079.1 | GM646615.1 | HV750341.1 | GM644149.1 |
| DL021786.1 | E02143.1 | CS695752.1 | HW261000.1 | HV956056.1 | A05153.1 | GM627021.1 | HV752863.1 | GM644117.1 |
| DL038456.1 | E01487.1 | CS695688.1 | HW260968.1 | HV951611.1 | A08247.1 | GM044572.1 | HV758991.1 | GM636927.1 |
| DL038424.1 | E01400.1 | CS695656.1 | HW260936.1 | HV951579.1 | A07044.1 | GM646479.1 | HV752624.1 | GM636895.1 |
| DL038392.1 | E01287.1 | CS695528.1 | HW260904.1 | HV951547.1 | A05798.1 | GM646447.1 | HV758657.1 | GM636863.1 |
| DL038360.1 | E00955.1 | CS695496.1 | HW260872.1 | HV950615.1 | A00328.1 | GM646415.1 | HV758625.1 | GM623524.1 |
| DL013165.1 | E00788.1 | CS695464.1 | HW260840.1 | HV947308.1 | M20283.1 | GM626873.1 | HV755163.1 | GM623492.1 |
| DL013133.1 | E00266.1 | DL106070.1 | HW260808.1 | HV953007.1 | M22135.1 | GM626841.1 | HV748941.1 | GM623428.1 |
| DL013101.1 | DD099261.1 | DL106038.1 | HW260776.1 | HV704342.1 | J02464.1 | GM626809.1 | HV748488.1 | GM623396.1 |
| DL013069.1 | DD069492.1 | DL106006.1 | HW260712.1 | HC438655.1 | M60901.1 | GM653538.1 | HV748404.1 | GM623364.1 |
| DL013037.1 | DD152065.1 | DL105974.1 | HW260680.1 | HC441390.1 | M65236.1 | GM653506.1 | AY657651.1 | GM643883.1 |
| DL013005.1 | DD158387.1 | DL105942.1 | HW260648.1 | HC358397.1 | M34061.1 | GM653442.1 | AY657619.1 | GM643851.1 |
| DL033740.1 | DD154889.1 | DL105910.1 | HW260616.1 | HC358209.1 | HW069891.1 | GM653378.1 | AY657587.1 | GM643819.1 |
| DL029734.1 | DD082933.1 | DL101297.1 | HW260584.1 | HC358078.1 | HW069859.1 | GM646379.1 | AY657555.1 | GM636822.1 |
| DL233407.1 | AX701748.1 | DL101265.1 | HW260552.1 | HC325065.1 | HW069767.1 | GM646347.1 | AY657523.1 | GM636758.1 |
| DL232087.1 | AX699058.1 | DL101233.1 | HW260520.1 | HC319589.1 | HW084955.1 | GM646315.1 | AY657491.1 | GM632254.1 |
| DL231667.1 | AX662192.1 | DL101201.1 | HW260456.1 | HC318835.1 | HW084916.1 | GM639416.1 | AY657459.1 | GM632222.1 |
| DL206557.1 | AX657129.1 | DL101169.1 | HW260424.1 | HC316615.1 | HW084880.1 | GM639384.1 | AY657427.1 | GM654821.1 |
| DL206282.1 | AX657097.1 | DL096723.1 | HW260360.1 | HC310506.1 | HW065064.1 | GM639352.1 | AY657395.1 | GM654789.1 |
| FB713728.1 | AX642269.1 | DL096691.1 | HW260328.1 | HC309417.1 | HW057922.1 | GM626709.1 | AY657363.1 | GM654757.1 |
| DL097944.1 | BD171820.1 | DL096659.1 | HW260296.1 | HC307893.1 | HW043938.1 | GM646080.1 | AY657331.1 | GM647700.1 |
| DL112634.1 | AX608840.1 | DL096627.1 | HW260264.1 | HC307815.1 | HW050534.1 | GM646048.1 | AY657299.1 | GM628286.1 |
| DL112602.1 | AX601373.1 | DL096595.1 | HW260232.1 | HC306921.1 | HV959842.1 | DL096518.1 | AY657267.1 | GM046375.1 |
| DL112570.1 | AX601341.1 | DL096563.1 | HW260200.1 | HB976956.1 | HV969762.1 | DL096486.1 | AY657235.1 | GM627962.1 |
| DL095730.1 | AX600185.1 | DL094735.1 | HW260168.1 | DM193205.1 | HV956161.1 | DL096454.1 | AY657203.1 | GM627930.1 |
| DL095698.1 | AX594158.1 | DL094703.1 | HW260136.1 | DM194714.1 | HV951088.1 | DL115321.1 | AY657171.1 | GM715081.1 |
| DL095666.1 | AX593503.1 | DL094639.1 | HW260104.1 | DM203069.1 | HV957534.1 | DL110655.1 | AY657139.1 | GM654746.1 |
| DL095634.1 | AX587802.1 | DL094607.1 | HW260072.1 | HB840113.1 | HV948608.1 | DL094233.1 | AY657107.1 | GM647592.1 |
| DL095602.1 | AX587731.1 | DL094575.1 | HW260040.1 | HB848032.1 | HV953042.1 | DL094169.1 | AY657075.1 | GM647560.1 |
| DL095570.1 | AX108995.1 | DL090960.1 | HW260008.1 | HB847785.1 | HV945052.1 | DL090486.1 | AY657043.1 | GM661711.1 |

| | | | | | | | | |
|---|---|---|---|---|---|---|---|---|
| DL117407.1 | AX107931.1 | DL090928.1 | HW259976.1 | HB839610.1 | HV942306.1 | DL114120.1 | M17195.1 | GM647276.1 |
| DL117375.1 | AX107112.1 | DL090896.1 | HW259944.1 | HB847589.1 | HV780043.1 | AX814393.1 | K02754.1 | GM647244.1 |
| DL091958.1 | AX100871.1 | DL086960.1 | HW259912.1 | HB847216.1 | HV778440.1 | AX587630.1 | M22029.1 | GM647212.1 |
| DL091634.1 | AX097502.1 | DL086928.1 | HW105473.1 | HB838867.1 | HV773873.1 | AX587471.1 | M10978.1 | GM870229.1 |
| DL091602.1 | AX093082.1 | DL086896.1 | HW105282.1 | HB847131.1 | HV757473.1 | AX576407.1 | M34498.1 | GM627616.1 |
| DL091570.1 | AX085933.1 | DL086864.1 | HW105097.1 | HB846668.1 | HV757322.1 | AX573318.1 | HV549871.1 | GM627578.1 |
| DL087959.1 | AX081307.1 | HV702441.1 | HW072605.1 | HB838238.1 | HV702566.1 | AX556842.1 | HV549254.1 | GM627546.1 |
| DL087927.1 | AX077719.1 | HV702354.1 | HW104685.1 | HB846301.1 | HW287435.1 | AX556807.1 | HV549222.1 | GM645903.1 |
| DL121758.1 | AX074317.1 | HV702261.1 | HW072161.1 | HB827353.1 | HW287339.1 | AX555631.1 | HV549190.1 | GM639036.1 |
| DL121726.1 | AX068058.1 | HV702024.1 | HW086400.1 | HB837935.1 | HW303248.1 | AX555167.1 | HV549088.1 | GM639004.1 |
| DL121694.1 | AX057058.1 | HV701217.1 | HW104427.1 | HB837558.1 | HW302707.1 | AX553946.1 | HV549007.1 | GM638972.1 |
| DL121662.1 | AX052937.1 | HV701185.1 | HW104381.1 | HB845850.1 | HW286770.1 | AX552569.1 | HV544354.1 | GM652838.1 |
| DL121598.1 | AX052902.1 | HV701153.1 | HW104281.1 | HB845514.1 | HW286181.1 | AX537316.1 | HV548053.1 | GM652806.1 |
| DL125446.1 | AX045399.1 | HV701121.1 | HW104271.1 | HB836960.1 | HW302389.1 | AX528837.1 | HV547412.1 | GM638816.1 |
| DL125414.1 | AX037312.1 | HV708719.1 | HW104239.1 | HB844789.1 | HW295720.1 | AX528400.1 | HV543503.1 | GM638784.1 |
| DL125382.1 | AX036355.1 | HV708525.1 | HW104159.1 | HB844354.1 | HW295590.1 | AX522000.1 | HV538614.1 | GM638752.1 |
| DL125350.1 | AX036007.1 | HV708226.1 | HW104119.1 | HB836750.1 | HW302046.1 | AX179521.1 | HV538458.1 | GM626109.1 |
| DL117189.1 | AX033562.1 | HV701070.1 | HW103886.1 | HB844053.1 | HW302002.1 | AX179461.1 | HV537624.1 | GM038280.1 |
| DL117157.1 | AX028790.1 | HV701038.1 | FZ435887.1 | HB843539.1 | HW295161.1 | AX164079.1 | HV542722.1 | FB775376.1 |
| DL117125.1 | AX025093.1 | HV695532.1 | FZ422121.1 | HB836193.1 | HW294985.1 | AX028009.1 | HV542690.1 | FB764693.1 |
| DL102494.1 | AX023684.1 | HV695131.1 | FZ434958.1 | HB843279.1 | HW285787.1 | A33490.1 | HV542185.1 | FB764068.1 |
| DL102462.1 | AX023636.1 | HV695019.1 | FZ426101.1 | HB843029.1 | HW293214.1 | A20351.1 | HV541498.1 | FB743972.1 |
| DL102430.1 | AX023602.1 | HV694923.1 | FZ429777.1 | HB835385.1 | HW293039.1 | AF430210.1 | HV540036.1 | FB743924.1 |
| DL102398.1 | AX023570.1 | HV694848.1 | HI583946.1 | HB842307.1 | HW292977.1 | AF430178.1 | HV515171.1 | DD206915.1 |
| DL102366.1 | AX019312.1 | HV700734.1 | HI583847.1 | HB841199.1 | HW292762.1 | AX512475.1 | HV533090.1 | DD206883.1 |
| DL102334.1 | AX014767.1 | HV694433.1 | HI577006.1 | HB647015.1 | HW291243.1 | AX505245.1 | HV532759.1 | DD206851.1 |
| DL097625.1 | HW314593.1 | HV693206.1 | HI520872.1 | HB645731.1 | HW291178.1 | AX505181.1 | HV536542.1 | DD206819.1 |
| DL097561.1 | HW115413.1 | HV699085.1 | HI520490.1 | HB645699.1 | HW291145.1 | AX496845.1 | HV532384.1 | CS250436.1 |
| DL095534.1 | HW115378.1 | HV698535.1 | HI207618.1 | GM652494.1 | HW291081.1 | BD140257.1 | HV537237.1 | DD181018.1 |
| DL095502.1 | HW069906.1 | HV698175.1 | HI003848.1 | GM659918.1 | HW291049.1 | BD137866.1 | HV535448.1 | CQ794361.1 |
| DL095470.1 | HW069806.1 | HV698112.1 | HI003706.1 | GM659886.1 | HW291017.1 | BD135101.1 | HV531944.1 | CQ792307.1 |
| DL095438.1 | HW068046.1 | HV689919.1 | HI002082.1 | GM659854.1 | HW290985.1 | BD131926.1 | HV528101.1 | CQ788133.1 |
| DL123345.1 | HW049578.1 | HV689004.1 | HI002030.1 | GM659822.1 | HW290953.1 | BD130819.1 | HV515589.1 | CQ787511.1 |
| DL123313.1 | HV963292.1 | HV688821.1 | HI001947.1 | GM659790.1 | HW290890.1 | BD130787.1 | HV515557.1 | CQ787447.1 |
| DL123281.1 | HV951095.1 | HV600535.1 | HI000279.1 | GM659722.1 | HW290858.1 | BD130755.1 | HV515525.1 | CQ787407.1 |
| DL123249.1 | HV957542.1 | HV585533.1 | HI000239.1 | GM659690.1 | HW290476.1 | BD130689.1 | HV515493.1 | CQ787299.1 |
| DL123217.1 | HV957473.1 | HV579752.1 | HI000195.1 | GM645282.1 | HW298500.1 | BD130589.1 | HV515461.1 | CQ787267.1 |
| DL123185.1 | HV945094.1 | HV585128.1 | HI000139.1 | GM645218.1 | HW290137.1 | BD129639.1 | HV515429.1 | CQ787235.1 |
| DL109155.1 | HV780050.1 | HV585096.1 | HI205890.1 | GM651910.1 | HW297995.1 | BD094347.1 | HV515397.1 | CQ786931.1 |
| DL109123.1 | HV777024.1 | HV585058.1 | HI471213.1 | GM631801.1 | HW144755.1 | BD087153.1 | HV515365.1 | CQ784713.1 |
| DL109091.1 | HV764392.1 | HV584864.1 | HI201576.1 | GM622838.1 | HW126648.1 | BD084025.1 | HV515333.1 | CQ784649.1 |
| DL104357.1 | HV775602.1 | HV579011.1 | HI180968.1 | FB508280.1 | HW126566.1 | HW380924.1 | HI573662.1 | CQ778529.1 |
| DL104325.1 | HW302514.1 | FZ436003.1 | HI204244.1 | GM643044.1 | HW125552.1 | HW380833.1 | HI566226.1 | CQ772589.1 |
| DL123162.1 | HW295689.1 | FZ435883.1 | HI470576.1 | GM719324.1 | HW144392.1 | HW381365.1 | HI594013.1 | CQ771651.1 |
| DL123130.1 | HW302332.1 | FZ435809.1 | HI544686.1 | GM656140.1 | HW144353.1 | HW381028.1 | HI636976.1 | CQ771619.1 |
| DL118684.1 | DM381893.1 | FZ434954.1 | HI538343.1 | GM648987.1 | HW124693.1 | HW293046.1 | HI001455.1 | CQ771587.1 |
| DL118652.1 | HC055543.1 | FZ425941.1 | HI559207.1 | GM634548.1 | HW123867.1 | HW291153.1 | HI001401.1 | CQ758876.1 |

| | | | | | | | | |
|---|---|---|---|---|---|---|---|---|
| DL118620.1 | HC053254.1 | FZ430059.1 | HI564715.1 | GM634516.1 | HW123799.1 | HW291056.1 | HI001359.1 | CQ754500.1 |
| DL114224.1 | HC051854.1 | FZ429266.1 | HI568937.1 | GM634484.1 | HW123474.1 | HW291024.1 | HI001325.1 | CQ754033.1 |
| DL114192.1 | HC049844.1 | FZ423960.1 | HI568861.1 | GM629679.1 | HW121949.1 | HW290897.1 | HI003208.1 | D88395.1 |
| DL114160.1 | DM191212.1 | FZ423870.1 | HI572786.1 | GM664834.1 | HW121653.1 | HW290865.1 | HI003089.1 | AX962029.1 |
| DL114128.1 | HC045304.1 | FZ436699.1 | HI558655.1 | GM648731.1 | HW120384.1 | HW289976.1 | HI003050.1 | AX960395.1 |
| DL114096.1 | HC047474.1 | FZ432343.1 | HI564215.1 | GM648699.1 | HW120106.1 | HW267815.1 | HI003009.1 | AX959103.1 |
| DL114064.1 | HC046870.1 | FZ428242.1 | HI564101.1 | GM648635.1 | HW120072.1 | HW265953.1 | HI002910.1 | AX958103.1 |
| DL126204.1 | HC046838.1 | FZ423342.1 | HI563701.1 | GM648603.1 | HW119944.1 | HW263046.1 | HI001273.1 | AX952429.1 |
| DL126172.1 | HC046806.1 | FZ431981.1 | HI563667.1 | GM634451.1 | HW119912.1 | HW263014.1 | HI001227.1 | AX937801.1 |
| DL122956.1 | HC046774.1 | FZ422767.1 | HI558086.1 | GM629464.1 | HW119880.1 | HW262839.1 | HI001173.1 | AX923401.1 |
| DL122924.1 | HC046742.1 | FZ415921.1 | HI563626.1 | GM619909.1 | HW119848.1 | HW261393.1 | HI001101.1 | AX840302.1 |
| DL122892.1 | HC039627.1 | FZ415209.1 | HI563593.1 | DL227075.1 | HW119816.1 | HW261361.1 | HI001083.1 | BD135462.1 |
| DL122860.1 | HC046090.1 | FZ420998.1 | HI563557.1 | FB713750.1 | HW119776.1 | HW261329.1 | HI001026.1 | BD131936.1 |
| DL122828.1 | GN368275.1 | FZ420850.1 | HI508652.1 | FB713017.1 | HW119741.1 | HW261297.1 | HI000993.1 | BD131903.1 |
| DL079084.1 | GN367482.1 | FZ420743.1 | HI583084.1 | FB712616.1 | HW119646.1 | HW261265.1 | HI002849.1 | BD130828.1 |
| DL079044.1 | GN360110.1 | FZ417470.1 | HI575435.1 | FB707446.1 | HW118929.1 | HW261233.1 | DL126078.1 | BD130796.1 |
| DL078960.1 | GN356191.1 | FZ416709.1 | HI544150.1 | FB674471.1 | HW118897.1 | HW261201.1 | DL126046.1 | BD130764.1 |
| DL078928.1 | GN360079.1 | FZ419863.1 | HI585884.1 | FB667456.1 | HW118445.1 | HW261169.1 | DL126014.1 | BD130603.1 |
| DL074257.1 | GN360037.1 | FZ419826.1 | HI000013.1 | FB666439.1 | HW105502.1 | HW261137.1 | DL125982.1 | BD129648.1 |
| DL073095.1 | GN359909.1 | FZ419794.1 | HI003601.1 | FB671497.1 | HW115600.1 | HW261105.1 | DL125950.1 | BD107647.1 |
| DL013506.1 | GN359864.1 | FW591961.1 | HI003568.1 | FB676556.1 | HW115126.1 | HW261073.1 | DL122758.1 | BD103199.1 |
| DL013474.1 | GN359832.1 | FW591899.1 | HI003517.1 | FB676492.1 | HW114474.1 | HW261041.1 | DL122702.1 | BD095064.1 |
| DL013410.1 | GN359800.1 | FW591724.1 | HI001843.1 | FB705620.1 | HW114441.1 | HW261009.1 | DL122670.1 | BD090538.1 |
| DL013286.1 | GN359768.1 | FW590873.1 | HI001783.1 | FB701805.1 | HW114393.1 | HW260977.1 | DL122606.1 | BD087165.1 |
| DL013254.1 | GN359736.1 | FW590734.1 | HI001747.1 | FB700930.1 | HW113627.1 | HW260945.1 | DL118384.1 | BD082676.1 |
| DL013222.1 | GN359524.1 | FW590116.1 | HI469624.1 | FB654397.1 | HW087566.1 | HW260913.1 | DL118352.1 | BD081622.1 |
| DL013190.1 | GN359456.1 | FW588678.1 | HI465601.1 | FB657097.1 | HW101700.1 | HW260881.1 | DL118320.1 | BD081296.1 |
| DL017979.1 | GN359360.1 | HH980129.1 | HI575285.1 | FB654857.1 | HW101662.1 | HW260849.1 | DL118288.1 | BD080674.1 |
| DL017947.1 | GN359296.1 | HH980058.1 | HI001625.1 | DL113536.1 | HW087224.1 | HW260817.1 | DL118256.1 | BD080325.1 |
| DL017915.1 | GN365386.1 | HH979884.1 | HI001583.1 | DL088622.1 | HW087124.1 | HW260753.1 | DL118224.1 | BD080135.1 |
| DL017692.1 | GN359223.1 | HH996714.1 | HI003368.1 | DL088590.1 | HW087092.1 | HW260721.1 | DL113828.1 | BD074977.1 |
| DL017660.1 | GN359065.1 | HH996647.1 | HI003330.1 | DL092347.1 | HW096517.1 | HW260689.1 | DL113796.1 | BD074941.1 |
| DL022266.1 | GN363075.1 | HH996607.1 | HI505600.1 | FB571343.1 | HW087048.1 | HW260657.1 | DL113728.1 | BD057810.1 |
| DL022234.1 | GN362972.1 | HH996551.1 | HI464736.1 | FB571287.1 | HW087016.1 | HW260625.1 | DL113756.1 | BD023625.1 |
| DL025559.1 | GN346600.1 | HH998464.1 | HI464704.1 | FB570992.1 | HW086981.1 | HW260561.1 | DL113700.1 | BD016724.1 |
| DL025349.1 | GN346535.1 | HH998408.1 | HI464672.1 | CS696083.1 | HW086943.1 | HW260529.1 | DL113668.1 | BD015697.1 |
| DL025317.1 | GM637339.1 | HH998370.1 | HI464640.1 | CS695954.1 | HW086909.1 | HW260497.1 | DL108867.1 | BD014494.1 |
| HV508494.1 | GM637307.1 | HH999955.1 | HC869708.1 | CS695794.1 | HW105485.1 | HW260465.1 | DL108835.1 | BD014223.1 |
| HV512296.1 | GM637275.1 | HH999906.1 | HC869644.1 | CS695762.1 | HW105099.1 | HW260433.1 | DL108803.1 | BD014188.1 |
| HV512264.1 | GM649686.1 | HH979823.1 | HC876976.1 | CS695730.1 | HW072796.1 | HW260401.1 | DL108771.1 | BD014097.1 |
| HV512232.1 | GM739602.1 | HH979673.1 | HC869392.1 | CS695698.1 | HW072607.1 | HW260369.1 | DL108739.1 | AX490825.1 |
| HV512200.1 | GM656519.1 | HH976517.1 | HC869360.1 | DL176469.1 | AY658902.1 | HW260337.1 | DL108707.1 | AX474392.1 |
| HV512168.1 | GM656487.1 | HH999870.1 | HC876936.1 | DL176389.1 | AY658870.1 | HV549104.1 | DL104025.1 | E51066.1 |
| HV512136.1 | GM656455.1 | HH999781.1 | HC876897.1 | DL175300.1 | AY658838.1 | HV547857.1 | DL104029.1 | AX468384.1 |
| JA660265.1 | GM649270.1 | HH999727.1 | FW310255.1 | DL176736.1 | AY658806.1 | HV547443.1 | DL103973.1 | AX463651.1 |
| HV504944.1 | GM649238.1 | HH998288.1 | FW331856.1 | DL163178.1 | AY658774.1 | HV543474.1 | DL103941.1 | AX461601.1 |
| HV504912.1 | GM649206.1 | HH998246.1 | FW309713.1 | FB344350.1 | AY658742.1 | HV539052.1 | DL099495.1 | AX459917.1 |

| | | | | | | | | |
|---|---|---|---|---|---|---|---|---|
| HV504880.1 | GM634963.1 | HH998201.1 | FW308797.1 | FB360097.1 | AY658710.1 | HV538624.1 | DL099463.1 | AX458630.1 |
| HV504848.1 | GM656318.1 | HH998158.1 | FW307695.1 | FB343369.1 | AY658678.1 | HV538468.1 | DL099431.1 | AX458480.1 |
| HV504767.1 | GM656286.1 | HH979535.1 | FW332863.1 | DL088295.1 | AY658646.1 | HV543198.1 | DL099399.1 | AX456483.1 |
| HV504735.1 | FB742926.1 | HH979638.1 | FW332696.1 | DL047654.1 | AY658614.1 | HV542596.1 | DL099367.1 | AX455742.1 |
| HV504703.1 | FB730279.1 | HH979598.1 | FW332602.1 | DL047622.1 | AY658582.1 | HV541510.1 | DL099335.1 | AX454128.1 |
| HV504671.1 | FB708877.1 | HH932016.1 | FW332318.1 | DL047590.1 | AY658550.1 | HV541325.1 | DL093325.1 | AX451981.1 |
| HV504639.1 | GM894299.1 | FW394273.1 | FW306301.1 | DL047558.1 | AY658518.1 | HV514610.1 | DL093293.1 | AX451341.1 |
| HV504607.1 | GM890143.1 | FW398139.1 | HC855987.1 | DL047526.1 | AY658486.1 | FB707443.1 | DL093261.1 | A34196.1 |
| HV492395.1 | GM747067.1 | FW394254.1 | HC769808.1 | DJ049401.1 | AY658454.1 | FB671027.1 | DL089550.1 | AX443280.1 |
| HV504200.1 | GM869490.1 | FW394212.1 | HC768310.1 | DJ048819.1 | AY658422.1 | DL099608.1 | DL089518.1 | A29527.1 |
| HV455584.1 | FB983195.1 | FW394087.1 | HC767348.1 | CS793782.1 | AY658390.1 | DL099576.1 | DL089486.1 | AX428433.1 |
| HV504164.1 | FB754297.1 | FW397874.1 | HC757679.1 | CS720177.1 | AY658358.1 | DL099544.1 | DL089422.1 | AX427714.1 |
| HV504132.1 | FB753863.1 | HI372271.1 | HC757125.1 | CS716810.1 | AY658326.1 | DL122647.1 | DL089390.1 | AX427217.1 |
| HV504100.1 | GM752105.1 | HI369414.1 | HC733786.1 | CS707362.1 | AY658294.1 | DL113533.1 | DL108681.1 | HW339030.1 |
| HV504068.1 | GM865610.1 | HI369111.1 | HC733775.1 | CS716738.1 | AY658262.1 | DL038022.1 | DL113620.1 | HW338958.1 |
| HV504036.1 | GM618813.1 | HI369079.1 | HC732455.1 | CS716030.1 | AY658230.1 | DL049726.1 | DL113588.1 | HW338702.1 |
| HV504004.1 | GM863541.1 | HI369022.1 | HC731710.1 | CS675427.1 | AY658198.1 | DL049694.1 | DL113556.1 | HW338446.1 |
| HV503940.1 | GM732418.1 | HI424139.1 | HC731350.1 | CS790645.1 | AY658166.1 | DL049662.1 | DL113524.1 | HW338318.1 |
| HV503876.1 | GM616031.1 | HI424107.1 | HC731318.1 | DJ031164.1 | AY658134.1 | DL011538.1 | DL113460.1 | HW338062.1 |
| HV503844.1 | GM040834.1 | HI424075.1 | HC731286.1 | DJ044967.1 | AY658102.1 | DL023587.1 | DL108563.1 | HW337934.1 |
| HV491269.1 | CS728632.1 | HI423849.1 | HC731254.1 | DJ044904.1 | AY658070.1 | DL047623.1 | DL108531.1 | HW337806.1 |
| HV503772.1 | CS728568.1 | HI423547.1 | HC471779.1 | DJ028153.1 | AY658038.1 | DL047591.1 | DL103893.1 | HW337678.1 |
| HV503740.1 | CS728536.1 | HI416361.1 | HC471747.1 | DJ027939.1 | AY658006.1 | DL047559.1 | DL103861.1 | HW337550.1 |
| HV503708.1 | CS727344.1 | HI415916.1 | HC471683.1 | DJ040561.1 | AY657974.1 | DL047527.1 | DL103829.1 | HW337422.1 |
| HV503676.1 | CS727261.1 | HI415884.1 | HC471707.1 | CS721660.1 | AY657942.1 | CS631244.1 | DL103797.1 | HW337294.1 |
| HV503644.1 | FB709023.1 | HI415819.1 | HC471651.1 | DJ004216.1 | AY657910.1 | CS631180.1 | DL103765.1 | HW321850.1 |
| HV455326.1 | FB715328.1 | HI415787.1 | HC471619.1 | BD236955.1 | AY657878.1 | CS627815.1 | DL103733.1 | HW336910.1 |
| HV503177.1 | DL088950.1 | HI415755.1 | HC730065.1 | BD235723.1 | AY657846.1 | DD438346.1 | DL093149.1 | HW336626.1 |
| HI979285.1 | DL088918.1 | HI414010.1 | HC729848.1 | BD234397.1 | AY657814.1 | DD435328.1 | DL093117.1 | HW336546.1 |
| HI989001.1 | DL025209.1 | HI413447.1 | HC471507.1 | BD233674.1 | AY657782.1 | CS617422.1 | DL093085.1 | BD246968.1 |
| HI988908.1 | CS612720.1 | HI516606.1 | HC471565.1 | BD231147.1 | AY657750.1 | CS616571.1 | DL112730.1 | BD245166.1 |
| HI987469.1 | CS611718.1 | HI516530.1 | HC729338.1 | BD227085.1 | AY657718.1 | CS612719.1 | DL112698.1 | BD244575.1 |
| HI987426.1 | CS604337.1 | HI516085.1 | HC728812.1 | BD226832.1 | AY657686.1 | CS603216.1 | DL112666.1 | BD243819.1 |
| FW552164.1 | CS603441.1 | HI547687.1 | HC727694.1 | BD224576.1 | AY657654.1 | CS423007.1 | DL107861.1 | BD243469.1 |
| FW552126.1 | CS603409.1 | HI284304.1 | HC726072.1 | BD223028.1 | AY657622.1 | CS419070.1 | DL107797.1 | BD242671.1 |
| FW554618.1 | CS602703.1 | HI284272.1 | HC688627.1 | BD206022.1 | AY657590.1 | CS410927.1 | DL107765.1 | BD242509.1 |
| FW555593.1 | CS607548.1 | HI571696.1 | HC688454.1 | DD283687.1 | AY657558.1 | CS410878.1 | DL107733.1 | BD242451.1 |
| FW567403.1 | CS501442.1 | HI574525.1 | HC688338.1 | DD283519.1 | AY657526.1 | CS410757.1 | DL107701.1 | BD238382.1 |
| FW560121.1 | CS498444.1 | HI574493.1 | DM193346.1 | DD282348.1 | AY657494.1 | CS414817.1 | DL103097.1 | BD236974.1 |
| FW556531.1 | CS495889.1 | HI574461.1 | DM196033.1 | DD286554.1 | AY657462.1 | DD323519.1 | DL103065.1 | BD235955.1 |
| FW561695.1 | DD418597.1 | HI574429.1 | DM190448.1 | CS349897.1 | AY657430.1 | DD327009.1 | DL103033.1 | BD235771.1 |
| FW561663.1 | DD411491.1 | HI574397.1 | DM195104.1 | CS283949.1 | AY657398.1 | CS398385.1 | DL098324.1 | BD235576.1 |
| FW562826.1 | DD417790.1 | HI571449.1 | DM189862.1 | CS283068.1 | AY657366.1 | CS389295.1 | DL098292.1 | BD235362.1 |
| FW562758.1 | CS492167.1 | HI561760.1 | DM203058.1 | CS277043.1 | AY657334.1 | CS389254.1 | DL098260.1 | BD231159.1 |
| FW565213.1 | CS492950.1 | FW377358.1 | HB806184.1 | CS276846.1 | AY657302.1 | CS389206.1 | DL098228.1 | BD231047.1 |
| FW562601.1 | CS489594.1 | FW381512.1 | HB822115.1 | CS272577.1 | AY657270.1 | CS389169.1 | DL098196.1 | BD227123.1 |
| FW561188.1 | CS486428.1 | FW381117.1 | HB805795.1 | CS265240.1 | FB743875.1 | CS406472.1 | DL098164.1 | BD226843.1 |

| | | | | | | | | |
|---|---|---|---|---|---|---|---|---|
| FW508112.1 | DD401421.1 | FW375492.1 | HB839912.1 | CS254296.1 | FB743843.1 | CS402135.1 | DL092154.1 | BD226736.1 |
| FW562489.1 | DD401389.1 | FW375245.1 | HB848578.1 | BD357352.1 | FB743789.1 | CS401807.1 | DL092122.1 | BD225681.1 |
| FW560914.1 | DD405204.1 | FW369645.1 | HB847932.1 | BD350548.1 | FB761763.1 | CS383384.1 | DL092090.1 | BD225649.1 |
| FW555396.1 | DD405172.1 | FW380262.1 | HB847703.1 | BD341689.1 | FB761606.1 | DD292809.1 | DL092058.1 | BD225617.1 |
| FW553168.1 | AX528824.1 | HH736039.1 | HB839420.1 | BD349543.1 | FB742912.1 | CS362760.1 | DL092026.1 | BD224819.1 |
| FW553090.1 | AX528122.1 | HH735918.1 | HB847464.1 | BD325586.1 | FB761495.1 | BD439073.1 | DL091994.1 | BD222119.1 |
| FW556661.1 | AX527664.1 | HH735623.1 | HB839182.1 | BD312807.1 | FB735800.1 | BD438930.1 | DL091962.1 | BD218879.1 |
| HI003675.1 | AX524915.1 | HD119615.1 | HB838778.1 | BD319272.1 | FB735589.1 | BD394875.1 | FB332230.1 | BD204991.1 |
| HI003630.1 | AX523925.1 | FW359980.1 | HB805287.1 | BD300815.1 | FB760128.1 | BD426667.1 | FB299284.1 | DD288447.1 |
| HI003588.1 | AX521963.1 | FW359896.1 | HB847003.1 | BD300481.1 | FB708870.1 | BD404616.1 | CS368470.1 | DD286607.1 |
| HI001831.1 | A33808.1 | FW359838.1 | HB838707.1 | BD290036.1 | FB676820.1 | BD453367.1 | CS368086.1 | CS356335.1 |
| HI001770.1 | A33887.1 | FW359751.1 | HB846171.1 | BD299624.1 | GM035753.1 | BD453366.1 | CS368022.1 | CS355957.1 |
| HI465671.1 | AX179504.1 | FW359659.1 | HB815605.1 | BD298240.1 | GM017398.1 | BD453303.1 | CS367830.1 | CS350537.1 |
| HI544132.1 | AX076862.1 | FW368717.1 | HB820861.1 | BD295270.1 | GM952837.1 | BD453271.1 | CS367702.1 | CS348906.1 |
| HI465489.1 | AX027969.1 | FW368679.1 | HB815015.1 | BD289988.1 | GM894240.1 | BD445342.1 | CS367638.1 | CS352954.1 |
| HI001613.1 | A09562.1 | FW368147.1 | HB809089.1 | BD274173.1 | GM893725.1 | BD402311.1 | CS367574.1 | CS284250.1 |
| HI001566.1 | AX521548.1 | FW367643.1 | HB837816.1 | BD273549.1 | GM890135.1 | BD375697.1 | CS367338.1 | DD029390.1 |
| HI001513.1 | AX521516.1 | FW363356.1 | HB845693.1 | BD271935.1 | GM963586.1 | BD373861.1 | DL080911.1 | DD028673.1 |
| HI003396.1 | AX513526.1 | FW367563.1 | HB845417.1 | CQ898630.1 | GM889456.1 | BD350549.1 | DL080745.1 | DD017166.1 |
| HI003354.1 | AF430202.1 | FW362957.1 | HB837238.1 | CQ898598.1 | DL097702.1 | BD341798.1 | DL080449.1 | DD012910.1 |
| HI003314.1 | AF430170.1 | FW367128.1 | HB813991.1 | CQ898566.1 | DL097670.1 | BD339006.1 | DL075904.1 | BD412639.1 |
| HI550423.1 | AX511248.1 | FW362768.1 | HB845202.1 | CQ888099.1 | DL097638.1 | BD325686.1 | CQ654292.1 | BD443838.1 |
| HI550380.1 | AX505229.1 | FW362661.1 | HB844988.1 | CQ877373.1 | DL097606.1 | BD325591.1 | CQ654203.1 | BD453860.1 |
| HI505481.1 | AX505165.1 | FW349830.1 | HB844563.1 | CQ873319.1 | DL097574.1 | BD312812.1 | CQ654107.1 | BD453828.1 |
| HI464724.1 | AX497564.1 | FW362072.1 | HB844283.1 | CQ871399.1 | DL097542.1 | BD319273.1 | CQ654015.1 | BD453796.1 |
| HI464692.1 | AX496673.1 | FW366209.1 | HB836626.1 | CQ869335.1 | DL095515.1 | BD301576.1 | AX963148.1 | BD453773.1 |
| HI464660.1 | BD140203.1 | FW351391.1 | HB843850.1 | CQ868887.1 | DL095483.1 | BD300484.1 | AX962177.1 | BD453772.1 |
| HI505329.1 | BD139566.1 | FW366033.1 | HB836361.1 | CQ859627.1 | DL095451.1 | BD290037.1 | AX960375.1 | BD453740.1 |
| HI505039.1 | BD138719.1 | FW350066.1 | HB835904.1 | CQ859595.1 | DL095419.1 | CS102481.1 | AX960343.1 | BD453708.1 |
| HI542541.1 | BD138413.1 | HD123186.1 | HB843173.1 | CQ858104.1 | DL095387.1 | CS097957.1 | AX959607.1 | BD453676.1 |
| HI504576.1 | BD138379.1 | HD121597.1 | HB835675.1 | CQ857853.1 | DL095355.1 | CS084383.1 | AX958651.1 | BD453644.1 |
| HI462684.1 | AX035460.1 | AY774627.1 | HB819141.1 | CQ855811.1 | DL091541.1 | CS075284.1 | AX957832.1 | BD453612.1 |
| HI503810.1 | AX034874.1 | AY774511.1 | HB818775.1 | CQ854066.1 | DL091509.1 | CS073256.1 | AX957775.1 | BD453575.1 |
| HH716014.1 | AX033493.1 | AY774462.1 | HB842661.1 | CQ848453.1 | DL091477.1 | CS072229.1 | BD010842.1 | BD453562.1 |
| HH714043.1 | AX030195.1 | AY774400.1 | HB806855.1 | CQ841123.1 | DL091445.1 | CS070667.1 | AX958076.1 | BD453530.1 |
| HH961379.1 | AX028774.1 | AY774344.1 | HB842136.1 | CQ830287.1 | DL091381.1 | CS059819.1 | AX958020.1 | BD453498.1 |
| HH961347.1 | AX027729.1 | AY774292.1 | HB841543.1 | CQ824366.1 | DL087584.1 | AY967399.1 | AX957041.1 | BD453466.1 |
| HH961315.1 | AX027346.1 | AY774240.1 | HB840941.1 | CQ816992.1 | DL087552.1 | AY967367.1 | AX952752.1 | BD453434.1 |
| HH961283.1 | AX026024.1 | AY774183.1 | HB840442.1 | CS018361.1 | DL087520.1 | AY967335.1 | AX952272.1 | BD453402.1 |
| HH961251.1 | AX024952.1 | AY774052.1 | HB650233.1 | CS016561.1 | DL087488.1 | AY967303.1 | AX938905.1 | BD453257.1 |
| HH975178.1 | AX023967.1 | AY773989.1 | HB649987.1 | CS007955.1 | DL087456.1 | AY967271.1 | AX934511.1 | BD453225.1 |
| HH982231.1 | AX023662.1 | AF284216.1 | HB646995.1 | BD143612.1 | DL087424.1 | AY967239.1 | AX924040.1 | BD453193.1 |
| HH974644.1 | AX023628.1 | HC084358.1 | HB645720.1 | AX576371.1 | DL087392.1 | AY967207.1 | AX923413.1 | BD411072.1 |
| HH963798.1 | AX023594.1 | HC070331.1 | HB645688.1 | AX573310.1 | DL112234.1 | AY967175.1 | AX923380.1 | BD497471.1 |
| HH998068.1 | AX023555.1 | HC083873.1 | HB645615.1 | AX557105.1 | DL112202.1 | AY967143.1 | AX840535.1 | BD451760.1 |
| HH998007.1 | AX020990.1 | HC083732.1 | HB855024.1 | AX556836.1 | DL112170.1 | AY967111.1 | AX839988.1 | BD451718.1 |
| HH997956.1 | AX019717.1 | HC083523.1 | HB850813.1 | AX555840.1 | DL112138.1 | AY967079.1 | AX829280.1 | BD408100.1 |

| | | | | | | | | |
|---|---|---|---|---|---|---|---|---|
| HH999654.1 | AX019192.1 | HC069870.1 | HB858617.1 | AX555093.1 | DL112106.1 | AY967047.1 | AX825990.1 | BD449774.1 |
| HH997905.1 | AX018604.1 | HC081554.1 | HB856836.1 | AX539581.1 | DL112074.1 | AY967015.1 | AX825256.1 | BD418725.1 |
| HH997834.1 | AX017673.1 | DM460342.1 | HB856024.1 | AX537267.1 | DL107071.1 | AY966983.1 | AX824454.1 | BD397071.1 |
| HH997768.1 | AX014757.1 | HC061995.1 | HB855910.1 | AX529544.1 | DL107007.1 | AY966951.1 | AX824344.1 | BD438710.1 |
| HH999508.1 | AX002769.1 | HC061915.1 | HB855443.1 | AX528128.1 | DL106975.1 | AY966919.1 | AX823778.1 | BD406398.1 |
| HH999451.1 | AX011486.1 | HC061366.1 | DM179341.1 | AX525162.1 | DL106943.1 | CS054192.1 | AX822496.1 | BD395648.1 |
| HH999339.1 | AX011454.1 | HC057730.1 | DM179157.1 | AX523927.1 | DL106911.1 | CS052521.1 | AX816108.1 | BD427312.1 |
| HH997647.1 | AX011422.1 | HC057625.1 | DM179077.1 | A33812.1 | DL102298.1 | CS052367.1 | AX814414.1 | BD395076.1 |
| HH997615.1 | AX011389.1 | DM460282.1 | DM170829.1 | A33889.1 | DL102234.1 | CS051002.1 | AX814380.1 | BD394502.1 |
| HH995710.1 | AX011357.1 | DM459243.1 | GN031570.1 | AX179509.1 | DL102226.1 | CS050399.1 | AX811304.1 | BD445618.1 |
| HH999246.1 | AX011325.1 | DM385649.1 | GN031538.1 | AX179449.1 | DL102170.1 | CS048041.1 | AX802095.1 | CS065992.1 |
| HH999152.1 | AX010961.1 | DM383913.1 | GN031506.1 | AX164056.1 | DL102138.1 | AX380785.1 | AX806010.1 | CS079801.1 |
| HH998118.1 | AX010631.1 | DM383631.1 | GN031474.1 | A28440.1 | DL117006.1 | AX375478.1 | AX799699.1 | CS078869.1 |
| HH999076.1 | AX010385.1 | DM381845.1 | GN031441.1 | A09575.1 | DL116974.1 | GN031284.1 | AX798346.1 | CS078821.1 |
| HH999031.1 | AX010122.1 | HC051940.1 | GN031409.1 | AF430204.1 | DL116942.1 | GN031252.1 | AX555198.1 | AY967377.1 |
| HH998965.1 | AX009732.1 | HC051647.1 | GN031377.1 | AF430172.1 | DL116910.1 | GN031188.1 | AX553567.1 | AY967345.1 |
| HH998922.1 | AX009656.1 | HC051015.1 | GN031345.1 | AX511564.1 | DL116878.1 | GN031155.1 | AX551936.1 | AY967313.1 |
| HH997141.1 | AX008534.1 | HC050300.1 | GN031313.1 | AX497566.1 | DL116846.1 | GN031123.1 | AX551088.1 | AY967281.1 |
| HH960113.1 | AX007119.1 | FB986372.1 | GN031280.1 | BD137585.1 | DL116818.1 | GN031091.1 | AX543889.1 | AY967249.1 |
| HH998868.1 | AX006473.1 | GM968448.1 | GN031248.1 | BD137222.1 | DL116786.1 | CS191098.1 | AX539576.1 | AY967217.1 |
| HH998809.1 | AX004800.1 | GM970565.1 | GN031216.1 | BD131920.1 | DL116754.1 | CS177809.1 | AX538727.1 | AY967185.1 |
| HH998754.1 | AX004389.1 | GM043758.1 | GN031184.1 | BD131384.1 | DL116722.1 | CS174736.1 | AX538015.1 | AY967153.1 |
| HH997094.1 | AX003203.1 | DL465749.1 | GN031119.1 | A14935.1 | DL116690.1 | CS172296.1 | AX537260.1 | AY967121.1 |
| HH997040.1 | AX002976.1 | DL477842.1 | GN031087.1 | A13390.1 | DL116658.1 | CS165465.1 | AX528954.1 | AY967089.1 |
| HH997002.1 | A76777.1 | DL464553.1 | GN031055.1 | A12766.1 | DL116626.1 | CS161567.1 | AX528815.1 | AY967057.1 |
| HH996947.1 | A41482.1 | DL463073.1 | GN030991.1 | A10857.1 | DL112033.1 | CS159780.1 | AX528116.1 | AY967025.1 |
| HH998703.1 | A24426.1 | DL476426.1 | GN030960.1 | A03745.1 | DL112001.1 | CS159221.1 | AX526828.1 | AY966993.1 |
| HC058515.1 | A35717.1 | DL462979.1 | GN030928.1 | A08282.1 | DL111969.1 | CS141598.1 | AX524912.1 | AY966961.1 |
| DM383623.1 | A29448.1 | DL469780.1 | GN030896.1 | A07380.1 | DL111937.1 | CS141538.1 | AX523922.1 | AY966929.1 |
| DM381922.1 | A35501.1 | DL481630.1 | GN030864.1 | A06082.1 | DL111905.1 | CS141506.1 | AX523640.1 | CS057764.1 |
| DM381493.1 | A32256.1 | DL460979.1 | GM636876.1 | A02271.1 | DL111893.1 | CS139110.1 | AX521940.1 | CS055299.1 |
| HC051933.1 | A30345.1 | GM712168.1 | GM632468.1 | A05800.1 | DL106870.1 | CS134727.1 | A33416.1 | CS052811.1 |
| HC051596.1 | A23371.1 | GM651926.1 | GM632436.1 | K02164.1 | DL106838.1 | CS138044.1 | A33868.1 | CS052309.1 |
| HC049735.1 | A30921.1 | GM831166.1 | GM632404.1 | M12795.1 | DL106806.1 | CS122804.1 | AX179439.1 | CS051899.1 |
| HC045497.1 | A21234.1 | FB509260.1 | GM632372.1 | M15949.1 | DL106742.1 | CS122287.1 | AX076853.1 | CS048770.1 |
| HC045433.1 | A19561.1 | GM631848.1 | GM632308.1 | M11132.1 | DL106710.1 | CS106108.1 | AX027966.1 | HV035634.1 |
| HC045401.1 | A17103.1 | GM631816.1 | GM623537.1 | M12440.1 | DL091344.1 | CS106011.1 | A28433.1 | FZ435957.1 |
| HC045369.1 | A15275.1 | GM622853.1 | GM623505.1 | M35460.1 | DL091312.1 | CS326610.1 | AX521545.1 | FZ427585.1 |
| HC045337.1 | A10855.1 | GM622821.1 | GM623473.1 | MK685261.1 | DL091280.1 | CS323605.1 | AX521513.1 | FZ430352.1 |
| HC049362.1 | A10502.1 | GM657938.1 | GM623441.1 | GN051251.1 | DL091248.1 | CS330848.1 | AX513491.1 | FZ430137.1 |
| HC047421.1 | A07611.1 | GM657906.1 | GM623409.1 | AH002296.2 | DL091216.1 | CS330108.1 | AF430167.1 | FZ437140.1 |
| HC047389.1 | A00971.1 | GM657874.1 | GM623377.1 | AF509587.1 | DL087376.1 | DD265898.1 | AX511241.1 | FZ422978.1 |
| HC047357.1 | A32047.1 | GM657842.1 | GM623345.1 | M31341.1 | DL087344.1 | DD272324.1 | AX505223.1 | FZ415475.1 |
| HC047325.1 | A30166.1 | GM657777.1 | GM643896.1 | DQ250253.1 | DL087312.1 | CS305252.1 | AX505159.1 | FZ421518.1 |
| HC047293.1 | A28157.1 | GM657745.1 | GM643864.1 | AY589055.2 | DL087280.1 | CS297026.1 | AX498609.1 | FZ421413.1 |
| HC047261.1 | A26923.1 | GM643386.1 | GM636835.1 | HG528078.1 | DL087248.1 | CS157937.1 | AX497561.1 | FW420429.1 |
| HC047037.1 | A22628.1 | GM643354.1 | GM636803.1 | KM396322.1 | DL087216.1 | CS157905.1 | BD140198.1 | FW420340.1 |

| | | | | | | | | |
|---|---|---|---|---|---|---|---|---|
| HC047005.1 | A15401.1 | GM643322.1 | GM636771.1 | HW399714.1 | DL102109.1 | CS119808.1 | BD139562.1 | FW496639.1 |
| HC046973.1 | A28181.1 | GM643258.1 | GM629270.1 | HW399343.1 | DL102077.1 | CS119489.1 | BD138375.1 | HI653920.1 |
| HC046941.1 | A26525.1 | GM643226.1 | GM629206.1 | HW390489.1 | DL102045.1 | CS119456.1 | BD137465.1 | HI653556.1 |
| HC046909.1 | A26029.1 | GM636237.1 | GM629142.1 | HW238014.1 | DL102013.1 | CS119388.1 | BD134013.1 | HI653394.1 |
| HC046881.1 | A23309.1 | GM631669.1 | GM629110.1 | HW237916.1 | DL101981.1 | CS119356.1 | BD133201.1 | HI653195.1 |
| HC046849.1 | A24709.1 | GM631637.1 | GM634040.1 | HW237872.1 | AY659349.1 | CS119258.1 | BD130808.1 | HI651890.1 |
| HC046817.1 | A23183.1 | GM631605.1 | GM634008.1 | HW237435.1 | AY659317.1 | CS119162.1 | BD130743.1 | HI651016.1 |
| HC046753.1 | A23863.1 | GM631573.1 | GM633976.1 | HW242296.1 | AY659285.1 | CS119130.1 | BD130710.1 | HI646687.1 |
| HC046721.1 | A22298.1 | GM631541.1 | GM633944.1 | HW242264.1 | AY659253.1 | CS119097.1 | BD130497.1 | AY383628.1 |
| DM102628.1 | A18474.1 | GM631509.1 | GM633912.1 | HW237025.1 | AY659221.1 | CS119063.1 | BD124163.1 | HH794940.1 |
| DM109925.1 | HI002250.1 | GM704174.1 | GM633880.1 | HW241548.1 | AY659189.1 | CS119030.1 | BD105809.1 | HH793106.1 |
| DM095088.1 | HI002197.1 | GM636055.1 | GM883182.1 | HW241371.1 | AY659157.1 | CS118932.1 | BD090733.1 | HH792575.1 |
| DM094871.1 | HI002116.1 | GM622450.1 | GM662847.1 | HW144833.1 | AY659125.1 | CS118900.1 | BD087720.1 | HH779964.1 |
| GN368438.1 | HI000461.1 | GM622418.1 | GM655521.1 | HW155293.1 | AY659093.1 | CS118866.1 | BD085734.1 | HH779666.1 |
| GN373298.1 | HI000413.1 | GM622386.1 | GM655489.1 | HW099472.1 | AY659061.1 | CS118735.1 | BD081720.1 | HH774424.1 |
| GN360089.1 | HI000371.1 | GM622354.1 | GM655457.1 | HW102816.1 | AY659029.1 | CS118703.1 | BD081517.1 | HH820985.1 |
| GN356158.1 | HI000314.1 | GM657508.1 | GM655425.1 | HW061832.1 | AY658997.1 | CS118639.1 | BD080710.1 | HH759308.1 |
| GN359779.1 | HI553195.1 | GM657476.1 | GM655393.1 | HW061818.1 | AY658965.1 | CS118607.1 | BD080438.1 | FW396290.1 |
| GN359715.1 | HI545529.1 | GM657444.1 | GM655361.1 | HW065214.1 | AY658933.1 | CS118574.1 | BD080147.1 | FW396664.1 |
| GN359683.1 | HI521280.1 | GM657412.1 | GM648368.1 | HW065182.1 | AY658901.1 | CS118539.1 | BD079172.1 | HH833860.1 |
| GN359663.1 | HI539189.1 | GM657380.1 | GM648336.1 | HW058471.1 | AY658869.1 | CS118473.1 | BD077103.1 | FW390819.1 |
| GN359567.1 | HI583947.1 | GM650156.1 | GM648304.1 | HW064888.1 | AY658837.1 | CS118441.1 | BD075652.1 | HH925717.1 |
| GN359435.1 | HI583848.1 | GM650124.1 | GM648272.1 | HW064848.1 | AY658805.1 | CS118409.1 | BD074954.1 | HH821964.1 |
| GN359307.1 | HI520873.1 | GM650092.1 | GM648240.1 | HW055440.1 | AY658773.1 | CS118376.1 | AX253025.1 | FW378015.1 |
| GN365264.1 | HI520491.1 | GM643123.1 | GM648208.1 | HW064744.1 | AY658741.1 | CS118342.1 | AX251539.1 | HD082407.1 |
| GN359138.1 | HI207620.1 | GM643091.1 | GM641405.1 | HW064712.1 | AY658709.1 | CS118308.1 | AX242334.1 | HD081721.1 |
| GN359044.1 | HI003707.1 | GM643059.1 | GM641373.1 | HW064680.1 | AY658677.1 | CS118243.1 | AX242302.1 | HD077947.1 |
| DM023379.1 | HI002083.1 | GM643027.1 | GM641341.1 | HW060673.1 | AY658645.1 | CS118210.1 | HI177805.1 | FW345361.1 |
| DM010742.1 | HI002031.1 | GM636006.1 | GM641309.1 | HW062415.1 | AY658613.1 | CS118112.1 | HI214440.1 | FW345033.1 |
| DM022261.1 | HI001948.1 | GM635974.1 | GM641277.1 | HW056327.1 | AY658581.1 | CS118079.1 | HI213780.1 | FW345001.1 |
| DM015972.1 | HI000280.1 | GM635942.1 | GM641245.1 | HW056242.1 | AY658549.1 | CS118046.1 | HI213013.1 | FW344678.1 |
| DM037121.1 | HI000240.1 | GM646029.1 | GM629054.1 | HW062358.1 | AY658517.1 | CS118011.1 | HI212973.1 | FW343722.1 |
| GN045525.1 | HI000196.1 | GM639130.1 | GM629022.1 | HW062326.1 | AY658485.1 | CS115733.1 | HI212614.1 | FW343654.1 |
| GN043558.1 | HI000140.1 | GM626519.1 | GM628990.1 | HW056078.1 | AY658453.1 | CS114512.1 | HI547162.1 | FW343590.1 |
| GN042579.1 | HI471214.1 | GM626487.1 | GM628958.1 | HW055756.1 | AY658421.1 | CS110175.1 | HI212468.1 | FW343210.1 |
| GN041738.1 | HI201577.1 | GM626455.1 | GM628926.1 | HW042049.1 | AY658389.1 | CS106381.1 | HI002290.1 | HC922994.1 |
| GN041619.1 | HI538865.1 | GM626391.1 | GM881911.1 | HW042017.1 | AY658357.1 | CS102989.1 | HI546325.1 | HC921521.1 |
| GN034819.1 | HI470744.1 | GM626359.1 | GM655232.1 | HW043501.1 | AY658325.1 | CS102925.1 | HI546293.1 | HC920515.1 |
| GN030740.1 | HI470577.1 | GM626327.1 | GM655200.1 | HW042005.1 | AY658293.1 | CS102893.1 | HI586989.1 | HD068868.1 |
| GN030708.1 | HI551359.1 | GM660416.1 | GM648175.1 | HW041973.1 | AY658261.1 | HW390302.1 | HI211005.1 | HD065024.1 |
| GN030644.1 | HI559210.1 | GM660384.1 | GM648143.1 | HW041909.1 | AY658229.1 | HW389601.1 | HI210971.1 | HD063941.1 |
| GN030612.1 | HI564717.1 | GM660352.1 | GM648111.1 | HW049363.1 | AY658197.1 | HW389044.1 | HI210939.1 | HC920297.1 |
| GN030580.1 | HI568946.1 | GM653165.1 | GM648079.1 | HW049332.1 | AY658165.1 | HW388311.1 | HI569851.1 | HD033765.1 |
| GN030548.1 | HI568862.1 | GM653133.1 | GM648047.1 | HW049268.1 | AY658133.1 | HW387488.1 | HI566162.1 | HD057710.1 |
| GN030516.1 | HI572812.1 | GM653101.1 | GM648015.1 | DL101838.1 | AY658101.1 | HW366488.1 | HI472912.1 | HD057678.1 |
| GN030356.1 | HI559133.1 | GM653069.1 | GM641212.1 | DL101799.1 | AY658069.1 | HW381359.1 | HI071985.1 | U20187.1 |
| GN030324.1 | HI564217.1 | GM653037.1 | GM641180.1 | DL101767.1 | AY658037.1 | HW338898.1 | HI004319.1 | L19522.1 |

| | | | | | | | | |
|---|---|---|---|---|---|---|---|---|
| GN030228.1 | HI564102.1 | GM653005.1 | GM641148.1 | DL101735.1 | AY658005.1 | HW338642.1 | HI002281.1 | M69037.1 |
| GN030132.1 | HI563702.1 | GM639011.1 | GM641116.1 | DL097321.1 | AY657973.1 | HW338130.1 | HI002244.1 | AY496269.1 |
| FB743892.1 | HI563668.1 | GM638979.1 | GM641084.1 | DL097289.1 | AY657941.1 | HW338002.1 | HI002194.1 | HC917433.1 |
| FB743860.1 | HI568109.1 | GM638947.1 | GM641052.1 | DL097257.1 | AY657909.1 | HW337746.1 | HI002151.1 | FW341963.1 |
| FB743806.1 | HI563627.1 | GM638915.1 | GM628892.1 | DL097225.1 | AY657877.1 | HW337618.1 | HI002113.1 | FW337028.1 |
| FB735872.1 | HI563594.1 | GM638883.1 | GM628860.1 | DL097193.1 | AY657845.1 | HW337106.1 | HI000458.1 | FW336142.1 |
| FB730017.1 | HB841801.1 | GM638851.1 | GM628828.1 | DL097161.1 | AY657813.1 | HW336486.1 | HI000410.1 | HC318839.1 |
| GM036249.1 | HB841598.1 | GM626172.1 | GM628796.1 | DL095333.1 | AY657781.1 | HW320840.1 | HI000368.1 | HC318694.1 |
| GM952593.1 | HB817831.1 | GM626140.1 | GM628764.1 | DL095269.1 | AY657749.1 | HW329378.1 | HI000311.1 | HC316708.1 |
| GM952002.1 | HB840943.1 | GM652973.1 | GM628732.1 | DL095237.1 | AY657717.1 | HW336131.1 | HI553264.1 | HC307898.1 |
| DL097559.1 | HB840444.1 | GM652941.1 | GM618951.1 | DL095205.1 | AY657685.1 | HW328828.1 | HI553192.1 | HC307833.1 |
| DL095532.1 | HB650236.1 | GM652909.1 | GM628684.1 | DL095173.1 | AY657653.1 | HW328721.1 | HH996553.1 | HC307499.1 |
| DL095500.1 | HB649412.1 | GM652877.1 | GM628652.1 | DL125340.1 | AY657621.1 | HW328610.1 | HH998466.1 | HC306926.1 |
| DL095468.1 | HB648470.1 | GM652845.1 | GM628588.1 | DL125308.1 | AY657589.1 | HW328462.1 | HH998413.1 | FU262775.1 |
| DL095436.1 | HB645721.1 | GM652813.1 | GM628556.1 | DL125276.1 | AY657557.1 | HW328303.1 | HH998372.1 | FU265395.1 |
| DL095404.1 | HB645617.1 | GM652780.1 | GM662187.1 | DL125244.1 | AY657525.1 | HW335668.1 | HH998339.1 | FU264491.1 |
| DL095372.1 | HB855025.1 | GM638823.1 | GM662155.1 | DL125212.1 | AY657493.1 | HW328244.1 | HH999957.1 | HC299770.1 |
| DL112155.1 | HB858831.1 | GM638791.1 | GM662123.1 | DL125180.1 | AY657461.1 | HW318377.1 | HH999908.1 | HC306117.1 |
| DL112123.1 | HB856837.1 | GM638759.1 | GM654974.1 | DL101717.1 | FZ417004.1 | HW314333.1 | HH979825.1 | HC306077.1 |
| DL112091.1 | HB856025.1 | GM626116.1 | GM641017.1 | DL121163.1 | FZ413928.1 | GN366664.1 | HH979675.1 | HC306037.1 |
| DL107088.1 | HB855911.1 | GM626084.1 | GM640985.1 | DL116600.1 | FZ416714.1 | GN366482.1 | HH976524.1 | HC305997.1 |
| DL107056.1 | HB855445.1 | GM626052.1 | GM640921.1 | DL116568.1 | FZ416682.1 | GN366450.1 | HH999872.1 | HC302640.1 |
| DL106992.1 | DM179549.1 | GM626020.1 | GM640889.1 | DL116536.1 | FZ419833.1 | GN366417.1 | HH999729.1 | HC302323.1 |
| DL106960.1 | DM179079.1 | GM625988.1 | GM640857.1 | DL116504.1 | FZ419799.1 | GN366385.1 | HH998297.1 | HC305042.1 |
| DL106928.1 | DM178843.1 | GM625956.1 | GM615428.1 | DL116472.1 | FZ419767.1 | GN366353.1 | HH998252.1 | HC305962.1 |
| DL106896.1 | DM170830.1 | GM645610.1 | FB712134.1 | DL116440.1 | FZ419735.1 | GN360062.1 | HH998215.1 | HB475021.1 |
| DL102189.1 | DM185644.1 | FB776237.1 | FB709051.1 | DL111847.1 | FZ419700.1 | GN360030.1 | HH998162.1 | HB469135.1 |
| DL102155.1 | DM170652.1 | FB775383.1 | GM841728.1 | DL111815.1 | HQ233650.1 | GN359998.1 | HH979537.1 | HB468763.1 |
| DL102123.1 | DM170586.1 | FB766229.1 | GM603721.1 | DL111751.1 | FW582891.1 | GN359966.1 | HH924301.1 | DM148862.1 |
| DL111922.1 | DM188255.1 | FB765900.1 | GM706722.1 | DL111719.1 | FW582642.1 | GN359934.1 | HH932609.1 | DM152634.1 |
| DL106855.1 | DM176993.1 | FB764705.1 | GM706690.1 | DL111687.1 | FW582335.1 | GN359902.1 | HH932019.1 | HB455477.1 |
| DL106823.1 | HB492062.1 | FB764668.1 | GM706658.1 | DL128898.1 | FW582265.1 | GN366328.1 | FW417370.1 | HB455134.1 |
| DL106791.1 | HB491540.1 | FB747038.1 | GM706394.1 | DL128865.1 | FW591906.1 | GN366296.1 | FW416859.1 | GN359333.1 |
| DL091201.1 | HB489276.1 | FB762773.1 | GM706359.1 | DL128831.1 | FW591166.1 | GN366264.1 | FW394275.1 | GN365392.1 |
| DL087233.1 | HB488783.1 | FB743931.1 | FB742341.1 | DL128799.1 | FW590933.1 | GN359857.1 | FW416387.1 | GN359260.1 |
| DL087201.1 | HB488703.1 | FB743899.1 | GM706320.1 | DL109167.1 | FW590739.1 | GN359825.1 | FW394256.1 | GN359228.1 |
| DL102094.1 | DM161014.1 | FB743867.1 | DL015905.1 | DL109135.1 | FW588901.1 | GN359793.1 | FW394182.1 | GN359196.1 |
| DL102062.1 | DM164032.1 | FB743835.1 | DL015873.1 | DL109103.1 | FW588146.1 | GN359761.1 | FW397877.1 | GN359164.1 |
| DL102030.1 | DM164017.1 | DL117194.1 | DL015841.1 | DL104497.1 | FW593460.1 | GN359729.1 | FW397577.1 | GN359132.1 |
| DL101913.1 | DM163984.1 | DL117162.1 | DL011383.1 | DL104465.1 | FW593045.1 | GN359697.1 | FW418670.1 | GN359100.1 |
| DL101881.1 | DM163950.1 | DL117130.1 | DL011351.1 | DL104433.1 | FW592984.1 | GN359677.1 | FW418601.1 | GN359070.1 |
| DL101817.1 | DM163544.1 | DL117098.1 | DL011319.1 | DL104401.1 | FW592807.1 | GN359645.1 | FW418037.1 | HV701234.1 |
| DL101785.1 | DM163279.1 | DL117066.1 | DL011287.1 | DL104369.1 | FW592642.1 | GN359613.1 | FW396754.1 | HV701170.1 |
| DL113952.1 | DM162568.1 | DL117034.1 | DL011255.1 | DL104337.1 | FW577841.1 | GN359581.1 | FW396371.1 | HV708542.1 |
| DL113920.1 | DM161495.1 | DL112446.1 | DL011223.1 | DL099923.1 | FW577600.1 | GN359549.1 | FW395236.1 | HV708206.1 |
| DL113888.1 | HB483689.1 | DL112414.1 | DL047647.1 | DL099891.1 | FW577119.1 | GN359517.1 | FW395610.1 | HV708135.1 |
| DL113856.1 | HB477331.1 | DL112382.1 | DL047615.1 | DL099859.1 | FW576810.1 | GN359485.1 | HH935705.1 | HV701055.1 |

| | | | | | | | | |
|---|---|---|---|---|---|---|---|---|
| DL109055.1 | GM636739.1 | DL112350.1 | DL047583.1 | DL099827.1 | FW576765.1 | GN365496.1 | HH934829.1 | HV700922.1 |
| DL109023.1 | GM636707.1 | DL112318.1 | DL047551.1 | DL099795.1 | FW576731.1 | GN359449.1 | HH834348.1 | HV694830.1 |
| DL108991.1 | GM636675.1 | DL112286.1 | DL047487.1 | DL099763.1 | FW576658.1 | GN359417.1 | HH833949.1 | HV694666.1 |
| DL104289.1 | GM632267.1 | DL143697.1 | DL039656.1 | DL099731.1 | FW575892.1 | GN359385.1 | HH833646.1 | HV700009.1 |
| DL104257.1 | GM632235.1 | DL107272.1 | DL039624.1 | DL093721.1 | FW575453.1 | GN359353.1 | HH833614.1 | HV693338.1 |
| DL104225.1 | GM632203.1 | DL107240.1 | DL039592.1 | DL093689.1 | FW572728.1 | GN359321.1 | HH833582.1 | HV699523.1 |
| DL099587.1 | GM632171.1 | DL107208.1 | DL039560.1 | DL093657.1 | FW568976.1 | GN359289.1 | HH833550.1 | HV689460.1 |
| DL099555.1 | GM632139.1 | DL102499.1 | DL039496.1 | DL093593.1 | FW574732.1 | GN365340.1 | HH833518.1 | HV695545.1 |
| DL126130.1 | GM632107.1 | DL102467.1 | DL014305.1 | DL093561.1 | FW572687.1 | GN364827.1 | HH833486.1 | HV585145.1 |
| DL126098.1 | GM623336.1 | DL102435.1 | DL014273.1 | DL089946.1 | FW572655.1 | GN359248.1 | HH833454.1 | HV585113.1 |
| DL122690.1 | GM623304.1 | DL102403.1 | DL014241.1 | DL089914.1 | FW573488.1 | GN359216.1 | HH833422.1 | HV579307.1 |
| DL122658.1 | GM623272.1 | DL102371.1 | DL009389.1 | DL089882.1 | DL010273.1 | GN359184.1 | HH833390.1 | HV592410.1 |
| DL118404.1 | GM623240.1 | DL102339.1 | DL009357.1 | DL089850.1 | DL010241.1 | GN359152.1 | HH833365.1 | HV226937.1 |
| DL108855.1 | GM623208.1 | DL097630.1 | DL009325.1 | DL089818.1 | DL010209.1 | GN359120.1 | HH833333.1 | HV235771.1 |
| DL108823.1 | GM623176.1 | DL097598.1 | DL009293.1 | DL089786.1 | DL034674.1 | GN359088.1 | HH832549.1 | HV312205.1 |
| DL108791.1 | GM623144.1 | DL097566.1 | DL009261.1 | DL085781.1 | DL034642.1 | GN359058.1 | HH836835.1 | HV305480.1 |
| DL108759.1 | AX240968.1 | DL095539.1 | DL009229.1 | DL085749.1 | DL034578.1 | GN363043.1 | FW392849.1 | HV040104.1 |
| DL108727.1 | AX240936.1 | DL095507.1 | DL018687.1 | DL085717.1 | DL034546.1 | GN362732.1 | FW392715.1 | HV188415.1 |
| DL108695.1 | HW249674.1 | DL095475.1 | DL009075.1 | DL085685.1 | DL034514.1 | GN362488.1 | HC726069.1 | HV200486.1 |
| DL099515.1 | HW247844.1 | DL095443.1 | DL009043.1 | DL085653.1 | DL030486.1 | GN346519.1 | HC688451.1 | HV200230.1 |
| DL099483.1 | HW240966.1 | DL095411.1 | DL014227.1 | DL137528.1 | DL030390.1 | GN346485.1 | HC491009.1 | HV182447.1 |
| DL099451.1 | HW240870.1 | DL095347.1 | DL014195.1 | DL123142.1 | DL026521.1 | GN344774.1 | HC490977.1 | HV182415.1 |
| DL099419.1 | HW240806.1 | DL091469.1 | DL014163.1 | DL123110.1 | DL026533.1 | GN343722.1 | HC490913.1 | HV182383.1 |
| DL089570.1 | HW240774.1 | DL091437.1 | DL014131.1 | DL123078.1 | DL026477.1 | GN349483.1 | HC490881.1 | HV220759.1 |
| DL089538.1 | HW240622.1 | DL091405.1 | DL014099.1 | DL123046.1 | DL026445.1 | GN346592.1 | HC490841.1 | HV203125.1 |
| DL089506.1 | HW247936.1 | DL091373.1 | DL014067.1 | DL123014.1 | DL026413.1 | GN346560.1 | HC490809.1 | HV117711.1 |
| DL012191.1 | HW239401.1 | DL087480.1 | DL014035.1 | DL122982.1 | DL026381.1 | GN340529.1 | HC490777.1 | HV038673.1 |
| DL048683.1 | HW239181.1 | DL087416.1 | DL026125.1 | DL118792.1 | DL022569.1 | GN340249.1 | HC490745.1 | HV038644.1 |
| DL037074.1 | HW238888.1 | DL112258.1 | DL026093.1 | DL118760.1 | DL022537.1 | GN340215.1 | HC490681.1 | HV038585.1 |
| DL037042.1 | HW238593.1 | DL112226.1 | DL026061.1 | DL118728.1 | DL022497.1 | GN339146.1 | HC490649.1 | HV182345.1 |
| DL021069.1 | HW238494.1 | DL112194.1 | DL026029.1 | DL118696.1 | DL022441.1 | GN337427.1 | HC490617.1 | HV182313.1 |
| DL021037.1 | HW238407.1 | DL112162.1 | DL025997.1 | DL118664.1 | DL022409.1 | GN130970.1 | HC490585.1 | HV182281.1 |
| DL012181.1 | HW243002.1 | DL112130.1 | DL025965.1 | DL118632.1 | DL019394.1 | GN335374.1 | HC490553.1 | HV182045.1 |
| DL012149.1 | HW237952.1 | DL112098.1 | DL018574.1 | DL114236.1 | DL019362.1 | GN334580.1 | DL097538.1 | HV202376.1 |
| DL012117.1 | HW237897.1 | DL112066.1 | DL018542.1 | DL114204.1 | DL019330.1 | GN333940.1 | DL095511.1 | HV029705.1 |
| DL012085.1 | HW237863.1 | DL107063.1 | DL018510.1 | DL114172.1 | DL019298.1 | GN116539.1 | DL095447.1 | HV035623.1 |
| DL012053.1 | HW248699.1 | DL107031.1 | DL018478.1 | DL114140.1 | DL019266.1 | GN116507.1 | DL095415.1 | FZ435988.1 |
| DL012021.1 | HW242509.1 | DL106999.1 | DL018446.1 | DL114108.1 | DL019234.1 | GN116474.1 | DL095351.1 | FZ427584.1 |
| DL011931.1 | HW242320.1 | DL106967.1 | DL018414.1 | DL114076.1 | DL019202.1 | GN116442.1 | DL091505.1 | HH822201.1 |
| DL011899.1 | HW242288.1 | DL106903.1 | DL014012.1 | DL114046.1 | DL014656.1 | GN116410.1 | DL091441.1 | FW378011.1 |
| DL024278.1 | HW242256.1 | DL102218.1 | DL013980.1 | DL126216.1 | DL010181.1 | GN116378.1 | DL091409.1 | FW381567.1 |
| DL024246.1 | HW241475.1 | DL102162.1 | DL013948.1 | DL126184.1 | DL010149.1 | GN116346.1 | DL091377.1 | FW381491.1 |
| DL024214.1 | HW163791.1 | DL102130.1 | DL013916.1 | DL126152.1 | DL010117.1 | GN116314.1 | DL087484.1 | FW375397.1 |
| DL019718.1 | HW163488.1 | DL116810.1 | DL013884.1 | DL122936.1 | DL010085.1 | GN112580.1 | DL087452.1 | FW375278.1 |
| DL015155.1 | HW161418.1 | DL116778.1 | DL013848.1 | DL122904.1 | DL010053.1 | GN115698.1 | DL087420.1 | HH736078.1 |
| DL010537.1 | HW160677.1 | DL116746.1 | DL030112.1 | DL122872.1 | DL010021.1 | GN112658.1 | DL087388.1 | HD119645.1 |
| DL010505.1 | HW160629.1 | DL111961.1 | DL030080.1 | DL122840.1 | DL019182.1 | GN115078.1 | DL112230.1 | HD119570.1 |

| | | | | | | | | |
|---|---|---|---|---|---|---|---|---|
| DL010473.1 | HW160597.1 | DL111929.1 | DL030048.1 | DL122776.1 | DL019150.1 | GN114942.1 | DL112198.1 | FW368170.1 |
| DL010441.1 | HW155907.1 | DL111897.1 | DL030016.1 | DL118586.1 | DL019118.1 | GM694786.1 | DL112134.1 | HD034002.1 |
| DL010409.1 | HW155753.1 | DL106862.1 | DL029984.1 | DL118554.1 | DL019086.1 | GM044491.1 | DL112102.1 | HD033078.1 |
| DL042728.1 | HW155687.1 | DL106830.1 | DL029952.1 | HH833367.1 | DL019054.1 | GM755087.1 | DL112070.1 | HD065411.1 |
| DL042696.1 | HW150104.1 | DL106798.1 | DL046641.1 | HH833335.1 | DL014619.1 | GM837006.1 | DL107067.1 | HD057709.1 |
| DL038783.1 | HW150072.1 | DL106766.1 | DL046609.1 | HH832551.1 | DL014587.1 | GM836940.1 | DL107003.1 | HD057677.1 |
| DL038751.1 | HW150040.1 | DL106734.1 | DL046577.1 | HH836846.1 | DL014555.1 | GM754533.1 | DL106971.1 | HD062213.1 |
| DL038719.1 | HW150008.1 | DL106702.1 | DL046545.1 | FW390805.1 | DL014523.1 | GM754395.1 | DL106939.1 | M34378.1 |
| HW321089.1 | HW149976.1 | DL091272.1 | DL046513.1 | FW392717.1 | DL014491.1 | GM694182.1 | DL106907.1 | M64922.1 |
| HW329413.1 | HW144813.1 | DL091240.1 | DL046481.1 | HH931895.1 | DL014459.1 | GM752863.1 | DL102294.1 | HC918446.1 |
| HW335971.1 | HV531940.1 | DL091208.1 | DL042659.1 | HH925817.1 | DL010001.1 | GM713273.1 | DL102262.1 | HC917432.1 |
| HW328739.1 | HV516232.1 | DL092380.1 | DL042627.1 | HC492344.1 | FB292739.1 | GM833977.1 | DL102222.1 | FW341962.1 |
| HW328558.1 | HV528049.1 | DL088605.1 | DL042595.1 | HC474338.1 | FB292246.1 | DL480644.1 | DL102166.1 | FW337027.1 |
| HW328440.1 | HV515585.1 | DL098532.1 | DL042563.1 | HC491961.1 | DD468096.1 | DL467543.1 | DL102134.1 | HC873816.1 |
| HW328337.1 | HV515553.1 | DL098468.1 | DL042531.1 | HC491768.1 | DJ019826.1 | DL467489.1 | DL117002.1 | HC873721.1 |
| HW328158.1 | HV515521.1 | DL092330.1 | DL042499.1 | HC491736.1 | DJ015932.1 | GM660117.1 | DL116970.1 | HC880380.1 |
| HW318649.1 | HV515457.1 | DL092298.1 | DL038671.1 | HC481662.1 | DJ015685.1 | GM660085.1 | DL116938.1 | HC689119.1 |
| HW318472.1 | HV515425.1 | DL092266.1 | DL038639.1 | HC481563.1 | CS803381.1 | GM660053.1 | DL116906.1 | HC688489.1 |
| HW335178.1 | HV515361.1 | DL092234.1 | DL038607.1 | HC491461.1 | CS802107.1 | GM660021.1 | DL116874.1 | HC688416.1 |
| HW314149.1 | HV515329.1 | DL092202.1 | DL034302.1 | HC491061.1 | FB295404.1 | GM659989.1 | DL116842.1 | HC687683.1 |
| HW313723.1 | HV515297.1 | DL092170.1 | DL034270.1 | HC488130.1 | FB295030.1 | GM659957.1 | DL116814.1 | HC490856.1 |
| HW312167.1 | HV515265.1 | DL088491.1 | DL034238.1 | HC470680.1 | DJ047314.1 | GM712982.1 | DL116782.1 | HC490792.1 |
| HW311946.1 | HV515233.1 | DL088459.1 | DL034206.1 | FV532169.1 | DJ047021.1 | GM652762.1 | DL116750.1 | HC490760.1 |
| HW311031.1 | HV510356.1 | DL088427.1 | DL034142.1 | FV531691.1 | DJ052350.1 | GM652730.1 | DL112061.1 | HC490696.1 |
| HW307862.1 | HV510323.1 | DL088395.1 | DL029913.1 | FV531409.1 | DJ052217.1 | GM652698.1 | DL112029.1 | HC490532.1 |
| HW307798.1 | HV510031.1 | DL117795.1 | DL029881.1 | FV523610.1 | DJ052034.1 | GM661819.1 | DL111997.1 | HC490500.1 |
| HW049378.1 | HV513362.1 | DL117763.1 | DL029849.1 | FV530968.1 | DJ045764.1 | GM661787.1 | DL111965.1 | HC472289.1 |
| HW049347.1 | HV513330.1 | DL117731.1 | DL029817.1 | FV530838.1 | DJ050490.1 | GM661755.1 | DL111933.1 | HC472257.1 |
| HW049315.1 | HV513078.1 | DL117699.1 | DL029753.1 | FV522829.1 | DJ049705.1 | GM661723.1 | DL111901.1 | HC472225.1 |
| HW049283.1 | HV508625.1 | DL117667.1 | DL025943.1 | FV522789.1 | DJ048846.1 | GM654729.1 | DL106866.1 | HC472193.1 |
| HW049251.1 | HV508593.1 | HW311126.1 | DL025911.1 | FV530351.1 | DJ048813.1 | GM654697.1 | DL106834.1 | HC471961.1 |
| HW046213.1 | HV508529.1 | HW308079.1 | DL025879.1 | FV530204.1 | DJ048781.1 | GM654665.1 | DL106802.1 | HC471929.1 |
| HW043253.1 | HV508503.1 | HW307868.1 | DL025847.1 | FV528649.1 | CS800123.1 | GM654633.1 | DL106770.1 | HC471897.1 |
| HW041790.1 | HV512393.1 | HW307804.1 | DL025815.1 | FV534653.1 | CS799997.1 | GM654601.1 | DL106738.1 | HC471865.1 |
| HW043073.1 | HV512271.1 | HW316584.1 | DL025783.1 | FV534478.1 | CS716864.1 | GM647575.1 | DL106706.1 | HC678817.1 |
| HW050570.1 | HV512239.1 | HW307734.1 | DL069494.1 | FV534426.1 | CS716799.1 | GM647543.1 | DL091340.1 | HC678750.1 |
| HW053734.1 | HV512175.1 | HW307702.1 | DL018354.1 | FV534188.1 | CS707283.1 | GM647511.1 | DL091308.1 | HC504296.1 |
| HV985994.1 | HV492270.1 | HW315957.1 | DL018322.1 | FV533995.1 | CS806653.1 | GM647479.1 | DL091276.1 | HC471031.1 |
| HV985962.1 | HV504951.1 | HW315731.1 | DL018290.1 | HC460533.1 | CS796407.1 | GM647447.1 | DL091244.1 | HC500966.1 |
| HV985930.1 | HV504919.1 | HW315691.1 | DL018258.1 | HC465768.1 | CS715321.1 | GM647415.1 | DL091212.1 | HC499852.1 |
| HV985898.1 | HV504887.1 | HW315601.1 | DL018226.1 | AF519544.1 | CS805422.1 | GM640612.1 | DL091180.1 | FW301300.1 |
| HW028873.1 | HV504855.1 | HW315562.1 | DL013824.1 | HC456294.1 | CS809481.1 | GM640580.1 | DL087372.1 | FW300769.1 |
| HV984260.1 | HV504823.1 | HV549079.1 | HW102609.1 | HC453872.1 | CS791230.1 | GM640548.1 | DL087340.1 | FW300529.1 |
| HV961485.1 | HV507453.1 | HV548992.1 | HW084157.1 | HC453720.1 | CS790639.1 | GM640483.1 | DL087308.1 | FW298757.1 |
| HV960462.1 | HV504774.1 | HV544285.1 | HW061939.1 | HC453688.1 | CS791006.1 | GM640451.1 | DL087276.1 | HC488008.1 |
| HV959626.1 | HV504742.1 | HV543987.1 | HW061860.1 | HC460198.1 | CS790969.1 | GM627904.1 | DL087244.1 | HC025524.1 |
| HV964131.1 | HV504710.1 | HV547840.1 | HW061828.1 | HC458396.1 | CS792487.1 | GM627840.1 | DL087212.1 | HC025492.1 |

| | | | | | | | | |
|---|---|---|---|---|---|---|---|---|
| HV966033.1 | HV504678.1 | HV547803.1 | HW062175.1 | AY382173.1 | DJ031847.1 | GM627808.1 | DL102105.1 | HC025428.1 |
| HV963590.1 | HV504646.1 | HV543494.1 | HW061814.1 | HC442350.1 | DJ042535.1 | GM627776.1 | DL102073.1 | HC010663.1 |
| HV965935.1 | HV504614.1 | HV539602.1 | HW061715.1 | HC452251.1 | DJ045298.1 | GM627744.1 | DL102041.1 | HC010414.1 |
| HV969952.1 | HV491619.1 | HV538577.1 | HW065795.1 | HC452169.1 | DJ044961.1 | GM647399.1 | DL102009.1 | HC009025.1 |
| HV962321.1 | HV504431.1 | HV538451.1 | HW065243.1 | HC452061.1 | DJ028605.1 | GM870600.1 | DL101977.1 | HC007666.1 |
| HV962085.1 | HV504207.1 | HV543365.1 | HW065210.1 | HC451977.1 | DJ044939.1 | GM661694.1 | DL101945.1 | HC007606.1 |
| HV961775.1 | HV455591.1 | HV543217.1 | HW065100.1 | HC450600.1 | DJ044898.1 | GM661662.1 | DL101013.1 | HC007574.1 |
| HV697974.1 | HV504171.1 | HV542747.1 | HW058436.1 | HC449026.1 | DJ028147.1 | GM661630.1 | DL100981.1 | HC007542.1 |
| HV689465.1 | HV504139.1 | HV542715.1 | HW061081.1 | HC045533.1 | DJ027928.1 | GM661598.1 | DL100949.1 | HC010675.1 |
| HV695887.1 | HV504107.1 | HV542611.1 | HW055403.1 | DM380117.1 | DJ030200.1 | GM661566.1 | DL096535.1 | DM375813.1 |
| HV695646.1 | HV504075.1 | HV542178.1 | HW060944.1 | HC035739.1 | DJ030083.1 | GM661534.1 | DL096503.1 | DM375340.1 |
| HV695582.1 | HV504043.1 | HV541033.1 | HW056586.1 | AB526355.1 | DJ030016.1 | GM654540.1 | DL096471.1 | HC005707.1 |
| HV600336.1 | HV504011.1 | HV515161.1 | HW056554.1 | HC025511.1 | DJ029939.1 | GM654508.1 | DL094515.1 | HC004952.1 |
| HV584757.1 | HV503979.1 | HV514903.1 | HW056504.1 | HC025479.1 | DJ043619.1 | GM654476.1 | DL094483.1 | DM370682.1 |
| HV579120.1 | HV503947.1 | HV532752.1 | HW064740.1 | HC025447.1 | DJ026288.1 | GM654412.1 | DL110844.1 | DM370650.1 |
| HV579001.1 | FW572211.1 | HV536509.1 | HW064667.1 | HC025415.1 | CS691626.1 | GM654380.1 | DL110780.1 | DM370618.1 |
| HV592482.1 | FW574381.1 | HV534611.1 | HW064608.1 | HC024945.1 | CS686962.1 | GM647387.1 | DL110748.1 | DM370586.1 |
| HV592447.1 | FW568744.1 | HV532108.1 | HW060545.1 | HC010650.1 | DD104528.1 | GM647355.1 | DL110716.1 | DM370554.1 |
| HV578514.1 | FW572009.1 | HV535642.1 | HW064453.1 | HC010190.1 | DD090688.1 | GM647323.1 | DL110684.1 | DM370522.1 |
| HV592283.1 | FW571774.1 | HV515582.1 | HW062354.1 | HC010110.1 | DD117214.1 | GM647291.1 | DL090746.1 | DM370490.1 |
| HV578482.1 | HI968141.1 | HV515550.1 | HW062258.1 | HC008502.1 | DD116399.1 | GM647259.1 | DL090714.1 | DM204605.1 |
| HV577247.1 | HI967433.1 | HV515518.1 | HV515301.1 | HC007837.1 | DD089599.1 | GM647227.1 | DL090682.1 | HC003075.1 |
| HV582880.1 | DL031314.1 | HV515486.1 | HV515269.1 | HC007813.1 | DD103278.1 | GM640424.1 | DL090650.1 | HB865040.1 |
| HV601494.1 | DL031282.1 | HV515454.1 | HV515237.1 | HC007781.1 | DD103203.1 | GM640392.1 | DL090618.1 | HB864964.1 |
| HV601350.1 | DL031250.1 | HV515422.1 | HV511236.1 | HC007749.1 | DD102583.1 | GM640360.1 | DL090586.1 | HB865000.1 |
| HV586238.1 | DL031218.1 | HV515358.1 | HV511010.1 | HC007717.1 | DD102450.1 | GM640328.1 | DL086581.1 | HB864944.1 |
| HV601071.1 | DL031186.1 | HV515294.1 | HV510392.1 | HC007685.1 | DD057917.1 | GM640296.1 | DL086549.1 | HB864912.1 |
| HV574860.1 | DL031154.1 | HV515262.1 | HV510360.1 | HC007653.1 | DD057885.1 | GM640264.1 | DL086517.1 | HB864880.1 |
| HV585640.1 | DL027345.1 | HV515230.1 | HV513270.1 | HC007625.1 | DD057853.1 | GM640232.1 | DL086485.1 | HB864848.1 |
| HV571039.1 | DL027313.1 | HV510434.1 | HV509995.1 | HC007593.1 | DD056822.1 | GM627685.1 | DL086453.1 | HB864816.1 |
| HV573207.1 | DL027281.1 | HV513327.1 | HV508597.1 | HC007529.1 | DD054306.1 | GM627653.1 | DL086421.1 | HB864784.1 |
| HV560298.1 | DL027249.1 | HI988922.1 | HV508565.1 | HC010768.1 | DD054008.1 | GM870297.1 | DL115523.1 | HB864752.1 |
| HV559933.1 | DL027217.1 | HI987474.1 | HV508533.1 | DM374722.1 | DD053313.1 | GM627633.1 | DL115491.1 | HB859733.1 |
| HV559386.1 | DL027185.1 | HI987430.1 | HV512895.1 | HC005491.1 | DD052473.1 | FB713805.1 | DL115459.1 | HB866541.1 |
| HV555943.1 | DL027153.1 | FW508670.1 | HV508473.1 | HC003693.1 | DD052239.1 | FB713709.1 | DL115434.1 | HB999692.1 |
| HV561915.1 | DL023341.1 | FW559252.1 | HV512416.1 | DM370971.1 | DD052206.1 | FB713642.1 | DL115402.1 | HB976960.1 |
| FZ419849.1 | DL023309.1 | FW560577.1 | HV512243.1 | DM370939.1 | DD051842.1 | FB713000.1 | DL115370.1 | GN031809.1 |
| FZ419816.1 | DL023277.1 | FW552300.1 | HV512147.1 | DM370907.1 | DD042736.1 | FB708166.1 | DL115338.1 | GN031711.1 |
| FW591775.1 | DL023245.1 | FW552266.1 | JA660230.1 | DM370836.1 | DD047270.1 | FB707312.1 | DL105675.1 | GN031679.1 |
| FW590839.1 | DL023213.1 | FW552229.1 | HV504955.1 | DM370802.1 | DD044314.1 | FB670985.1 | DL013790.1 | GN031647.1 |
| FW590724.1 | DL023181.1 | FW562018.1 | HV504923.1 | DM370767.1 | DD030105.1 | FB667742.1 | DL013758.1 | GN031615.1 |
| FW590296.1 | DL020166.1 | FW552168.1 | HV504891.1 | DM370669.1 | DD039056.1 | FB667430.1 | DL013726.1 | GN031551.1 |
| FW589153.1 | DL020102.1 | FW552133.1 | HV504827.1 | DM370637.1 | DD038330.1 | FB667354.1 | DL013694.1 | GN031519.1 |
| FW588457.1 | DL020070.1 | FW554626.1 | HV504778.1 | DM370605.1 | DD037404.1 | FB667119.1 | DL013662.1 | GN031390.1 |
| FW593438.1 | DL015443.1 | FW555597.1 | HV504714.1 | DM370573.1 | DD037084.1 | FB666808.1 | DL000817.1 | GN031358.1 |
| HQ161062.1 | DL010921.1 | FW560340.1 | HV504682.1 | DM370541.1 | DD033909.1 | FB666181.1 | DJ493975.1 | GN031326.1 |
| FW576912.1 | DL010889.1 | FW561667.1 | HV504650.1 | DM370509.1 | DD032781.1 | FB670940.1 | DJ493823.1 | GN031293.1 |

| | | | | | | | | |
|---|---|---|---|---|---|---|---|---|
| FW576716.1 | DL010857.1 | FW557009.1 | HV504618.1 | DM370477.1 | DD031278.1 | FB704342.1 | DL008723.1 | GN031261.1 |
| FW576677.1 | DL010825.1 | FW562764.1 | HV491623.1 | DM367603.1 | DD030696.1 | FB676547.1 | DL003038.1 | GN031229.1 |
| FW576638.1 | DL027152.1 | FW562631.1 | HV504969.1 | DM205509.1 | DD026922.1 | FB676515.1 | DJ491890.1 | GN031197.1 |
| FW576331.1 | DL023146.1 | FW561203.1 | HV504563.1 | DM205173.1 | CS086829.1 | FB704847.1 | DJ491563.1 | GN031165.1 |
| FW575877.1 | DL023082.1 | FW566633.1 | HV504531.1 | DM371248.1 | CS084181.1 | FB701530.1 | DL002528.1 | GN031132.1 |
| FW575336.1 | DL023050.1 | FW505281.1 | HV504499.1 | DM365913.1 | CS082540.1 | CS810729.1 | DJ491067.1 | GN031100.1 |
| FW571911.1 | DL023018.1 | FW508116.1 | HV504467.1 | DM204195.1 | CS082423.1 | FB701787.1 | DJ446829.1 | GN031036.1 |
| HI375916.1 | CS630760.1 | FW560950.1 | HV504435.1 | HC003049.1 | CS082111.1 | FB702560.1 | DJ446688.1 | GN030972.1 |
| HI372209.1 | CS627764.1 | FW555400.1 | HV504211.1 | HC001732.1 | CS080408.1 | FB660760.1 | DJ442346.1 | GN030941.1 |
| HI369620.1 | CS632413.1 | FW553181.1 | HV455595.1 | DL039139.1 | CS079516.1 | FB581895.1 | DJ437277.1 | GN030909.1 |
| HI369157.1 | CS626952.1 | FW556665.1 | HV504175.1 | DL039107.1 | CS077807.1 | FB654443.1 | DJ437244.1 | GN030877.1 |
| HI369102.1 | DD438039.1 | FW559389.1 | HV504143.1 | DL035483.1 | CS075582.1 | FB654379.1 | DJ437179.1 | GN030845.1 |
| HI368608.1 | DD438001.1 | FW506876.1 | HV504111.1 | DL035451.1 | CS074048.1 | FB657029.1 | DJ437149.1 | DM006933.1 |
| HI424975.1 | DD449092.1 | FW510487.1 | HV504079.1 | DL035419.1 | CS073117.1 | FB654885.1 | DJ437112.1 | DM000227.1 |
| GM648865.1 | DD435294.1 | FW381691.1 | HV504047.1 | DL035387.1 | CS072181.1 | FB573605.1 | DJ437079.1 | DM005068.1 |
| GM648833.1 | CS617850.1 | FW381947.1 | HV504015.1 | DL035355.1 | CS070129.1 | FB582153.1 | DJ436994.1 | DM004986.1 |
| GM648801.1 | CS616545.1 | HH931887.1 | HV503983.1 | DL035323.1 | CS067346.1 | FB580239.1 | DJ436918.1 | DM003487.1 |
| GM634554.1 | CS614913.1 | HH931481.1 | HV503951.1 | DL031319.1 | CS063854.1 | FB580195.1 | DJ436850.1 | GM984191.1 |
| GM634522.1 | CS613795.1 | HH821471.1 | HV503919.1 | DL031287.1 | CS062977.1 | FB580163.1 | DJ436763.1 | GM983960.1 |
| GM634490.1 | CS613756.1 | HH821162.1 | HV503887.1 | DL031255.1 | CS061691.1 | FB580131.1 | DJ436669.1 | GM983063.1 |
| GM629685.1 | CS612789.1 | HH822342.1 | HV503855.1 | DL031223.1 | CS061038.1 | DL200078.1 | DJ436510.1 | GM996512.1 |
| GM664844.1 | CS611851.1 | HH821974.1 | HV503823.1 | DL031191.1 | CS059016.1 | DL200029.1 | DJ445695.1 | GM995274.1 |
| GM664583.1 | CS604547.1 | HH821874.1 | HV491137.1 | DL031159.1 | AY967392.1 | DL189561.1 | DJ444826.1 | GM979527.1 |
| GM648737.1 | CS604515.1 | FW379676.1 | HV503783.1 | DL027350.1 | AY967360.1 | DL189467.1 | DJ444572.1 | GN007453.1 |
| GM648705.1 | CS604483.1 | FW379643.1 | HV503751.1 | DL027318.1 | AY967328.1 | DL199828.1 | DJ439618.1 | GM991941.1 |
| GM648673.1 | CS604387.1 | FW379611.1 | HV503719.1 | CS647190.1 | AY967296.1 | DL199732.1 | DJ434388.1 | GM647270.1 |
| GM648641.1 | CS604355.1 | FW376136.1 | HV503687.1 | CS646204.1 | AY967264.1 | DL199700.1 | DJ438365.1 | GM647206.1 |
| GM648609.1 | CS604259.1 | FW381112.1 | FZ420746.1 | DD460518.1 | AY967232.1 | DL199668.1 | DJ438359.1 | GM661481.1 |
| GM634457.1 | CS604163.1 | FW375479.1 | FZ417002.1 | DD459945.1 | AY967200.1 | DL194675.1 | DJ438292.1 | GM661449.1 |
| GM634393.1 | CS604067.1 | FW369636.1 | FZ416712.1 | DD455868.1 | AY967168.1 | DL194639.1 | DJ438211.1 | GM661417.1 |
| GM629470.1 | CS603843.1 | FW380259.1 | FZ416680.1 | DD455163.1 | AY967136.1 | DL194551.1 | DJ427988.1 | GM661385.1 |
| GM629428.1 | CS603459.1 | HH736033.1 | FZ419831.1 | DD458755.1 | AY967104.1 | GM644973.1 | DJ402583.1 | GM661353.1 |
| GM629396.1 | CS603427.1 | HH733944.1 | FZ419797.1 | DD453830.1 | AY967072.1 | GM644941.1 | DJ402549.1 | GM661321.1 |
| GM629364.1 | CS603395.1 | HH733910.1 | FZ419765.1 | DD457866.1 | AY967040.1 | GM644909.1 | DJ402295.1 | GM647174.1 |
| GM629332.1 | CS603363.1 | HH733592.1 | FZ419732.1 | DD452622.1 | AY967008.1 | GM644877.1 | DJ401521.1 | GM647142.1 |
| GM629300.1 | CS603331.1 | FW365382.1 | FZ419697.1 | DD456491.1 | AY966976.1 | GM644845.1 | DJ400840.1 | GM647110.1 |
| GM648531.1 | CS603299.1 | CS486622.1 | FZ416531.1 | DD456326.1 | AY966944.1 | GM644813.1 | DJ400808.1 | GM647046.1 |
| GM648499.1 | CS603267.1 | CS483001.1 | HQ233648.1 | DD451434.1 | CS056313.1 | GM638014.1 | DJ400488.1 | GM647017.1 |
| FB713980.1 | CS603235.1 | CS482929.1 | FW582887.1 | CS631733.1 | CS055986.1 | GM637982.1 | DJ399342.1 | GM646875.1 |
| FB713820.1 | CS603203.1 | CS482886.1 | FW582640.1 | CS642211.1 | CS053085.1 | GM637950.1 | DJ415870.1 | GM646843.1 |
| FB713756.1 | CS603171.1 | CS482749.1 | FW582263.1 | CS631228.1 | CS052514.1 | GM637918.1 | CS798097.1 | GM646811.1 |
| FB707511.1 | CS603139.1 | DD401434.1 | FW581847.1 | CS627774.1 | CS052292.1 | GM637886.1 | CS806565.1 | GM627178.1 |
| FB674505.1 | CS603107.1 | DD401402.1 | FW591106.1 | CS632818.1 | CS050990.1 | GM637854.1 | CS715303.1 | GM660970.1 |
| FB671081.1 | CS603075.1 | DD405217.1 | FW590737.1 | CS632689.1 | CS050955.1 | GM633446.1 | CS790966.1 | GM660938.1 |
| FB670795.1 | CS603043.1 | DD405185.1 | FW588681.1 | CS632617.1 | CS048863.1 | GM633350.1 | CS792484.1 | GM646743.1 |
| FB667085.1 | CS603011.1 | DD405153.1 | FW588144.1 | CS632183.1 | CS047264.1 | GM633318.1 | DJ045039.1 | GM646711.1 |
| FB704849.1 | CS602977.1 | DD405121.1 | FW592804.1 | CS626892.1 | CS039316.1 | GM633286.1 | DJ044958.1 | GM646679.1 |

| | | | | | | | | |
|---|---|---|---|---|---|---|---|---|
| FB676562.1 | CS602945.1 | DD408559.1 | FW592672.1 | CS623614.1 | CS039284.1 | GM637831.1 | DJ028601.1 | DL193899.1 |
| FB676498.1 | CS602913.1 | DD410054.1 | DL044151.1 | DD450363.1 | CS039252.1 | GM637799.1 | DJ044936.1 | DL193816.1 |
| FB705654.1 | CS349696.1 | DD405959.1 | DL044119.1 | DD450299.1 | CS039220.1 | GM637767.1 | DJ044895.1 | DL193784.1 |
| FB705953.1 | CS353187.1 | DD402400.1 | DL023409.1 | DD450267.1 | CS039116.1 | GM637735.1 | DJ044658.1 | DL193743.1 |
| FB701819.1 | CS353005.1 | DD402336.1 | DL023377.1 | DD450235.1 | CS038882.1 | GM637703.1 | DJ028144.1 | FB571349.1 |
| FB580683.1 | CS284491.1 | DD402304.1 | DL020362.1 | DD450162.1 | CS038833.1 | GM637671.1 | DJ030175.1 | FB571293.1 |
| FB654865.1 | CS275990.1 | DD402272.1 | DL020330.1 | DD450128.1 | CS037903.1 | GM633263.1 | DJ030059.1 | CS249790.1 |
| FB573879.1 | CS273587.1 | DD402240.1 | DL020298.1 | DD441741.1 | CS037244.1 | GM633231.1 | DJ030011.1 | CS244152.1 |
| FB573839.1 | CS253982.1 | DD402208.1 | DL020266.1 | DD436741.1 | AX664352.1 | GM633199.1 | DJ029935.1 | CS244200.1 |
| DL191597.1 | CS249881.1 | CS326345.1 | DL020234.1 | DD435319.1 | AX662242.1 | GM633167.1 | DJ026768.1 | CS243762.1 |
| DL196188.1 | CS250216.1 | CS323673.1 | DL020202.1 | CS620447.1 | AX662175.1 | GM633135.1 | CS721646.1 | CS243153.1 |
| DL193820.1 | CS244964.1 | CS323591.1 | DL015799.1 | CS619952.1 | AX659116.1 | GM633103.1 | CS685278.1 | CS239580.1 |
| DL120278.1 | CS244166.1 | CS322842.1 | DL015767.1 | CS617091.1 | AX657681.1 | GM625524.1 | CS678763.1 | DD117031.1 |
| DL120246.1 | CS244246.1 | CS330142.1 | DL015735.1 | CS616551.1 | AX657120.1 | GM625492.1 | CS693265.1 | DD090290.1 |
| DL120214.1 | CS244214.1 | CS329664.1 | DL011181.1 | CS614190.1 | AX657085.1 | GM625460.1 | DJ008408.1 | DD089923.1 |
| DL115811.1 | CS244182.1 | DD271410.1 | DL010999.1 | CS613800.1 | BD176042.1 | GM625396.1 | DJ008376.1 | DD102592.1 |
| DL115779.1 | CS243274.1 | DD261126.1 | DL047225.1 | CS613768.1 | BD175781.1 | GM625364.1 | DD495645.1 | DD057929.1 |
| DL101221.1 | CS239594.1 | DD259343.1 | DL047193.1 | CS612974.1 | BD175615.1 | GM625332.1 | CS670997.1 | DD057897.1 |
| DL101189.1 | CS239706.1 | DD265333.1 | DL047161.1 | CS604520.1 | BD174937.1 | GM659319.1 | CS669143.1 | DD057865.1 |
| DL101157.1 | CS237760.1 | CS302572.1 | DL047129.1 | CS604488.1 | BD174402.1 | GM659287.1 | CS604993.1 | DD057837.1 |
| DL101125.1 | CS231718.1 | DD251666.1 | DL047097.1 | CS604456.1 | AJ005292.1 | GM659255.1 | CS604961.1 | DD057053.1 |
| DL096615.1 | CS227256.1 | DD251591.1 | DL047065.1 | CS604424.1 | AX642231.1 | GM659223.1 | CS604929.1 | DD053293.1 |
| DL096583.1 | CS226728.1 | DD253798.1 | DL043243.1 | CS604392.1 | AX641922.1 | GM659191.1 | CS604865.1 | DD051872.1 |
| DL096551.1 | CS223275.1 | DD253695.1 | DL043211.1 | CS604360.1 | AX616605.1 | GM659159.1 | CS604833.1 | DD058901.1 |
| DL094723.1 | CS208777.1 | CS287621.1 | DL043179.1 | CS604328.1 | BD172295.1 | GM659108.1 | CS604801.1 | DD041898.1 |
| DL094691.1 | CS207921.1 | CS287423.1 | DL043147.1 | CS604264.1 | AX614975.1 | GM659076.1 | CS604769.1 | DD046368.1 |
| DL090948.1 | CS207841.1 | DD240747.1 | DL043115.1 | CS604232.1 | AX601761.1 | GM659044.1 | CS604737.1 | DD037223.1 |
| DL090916.1 | CS188748.1 | DD237943.1 | DL043083.1 | CS604200.1 | AX600116.1 | GM659012.1 | CS604705.1 | DD034197.1 |
| DL086980.1 | HH998454.1 | DD235095.1 | DL039266.1 | CS604104.1 | AX599040.1 | GM658980.1 | CS604673.1 | DD027245.1 |
| DL086948.1 | HH998405.1 | DD247145.1 | DL039234.1 | CS200920.1 | AX598988.1 | GM651756.1 | CS604639.1 | DD023875.1 |
| DL086916.1 | HH998366.1 | E43120.1 | DL039202.1 | CS226733.1 | AX598880.1 | GM651724.1 | CS604607.1 | DD028813.1 |
| DL105869.1 | HH999998.1 | E43957.1 | DL039170.1 | CS208838.1 | AX597793.1 | GM651692.1 | CS604575.1 | DD010178.1 |
| DL105837.1 | HH999952.1 | E49204.1 | DL039106.1 | CS223188.1 | AX597481.1 | GM651660.1 | CS593090.1 | DD017645.1 |
| DL105805.1 | HH979820.1 | E33337.1 | DL035482.1 | CS203550.1 | AX593486.1 | GM651628.1 | CS589276.1 | BD412625.1 |
| DL096522.1 | HH979670.1 | E36139.1 | DL035450.1 | CS207926.1 | AX593187.1 | GM651595.1 | CS597738.1 | BD434072.1 |
| DL096490.1 | HH976344.1 | E35142.1 | DL035418.1 | CS193246.1 | AX587874.1 | GM644796.1 | CS585654.1 | BD453887.1 |
| DL096458.1 | HH999867.1 | E06038.1 | DL035386.1 | CS188630.1 | AX587779.1 | GM644732.1 | CS585070.1 | BD453855.1 |
| DL089440.1 | HH999778.1 | CS203957.1 | DL035354.1 | CS186208.1 | AX587685.1 | GM644700.1 | CS584687.1 | BD453823.1 |
| DL113542.1 | HH999724.1 | CS001386.1 | DL035322.1 | CS182522.1 | AX587580.1 | GM644636.1 | CS588492.1 | BD453791.1 |
| DL136976.1 | HH998280.1 | AF405702.1 | DL031318.1 | CS183886.1 | BD161162.1 | GM637606.1 | CS574920.1 | BD433655.1 |
| DL118107.1 | HH998243.1 | CS157794.1 | DL031286.1 | CS177740.1 | AX587996.1 | GM637574.1 | CS573046.1 | BD453767.1 |
| DL118075.1 | HH998195.1 | CS157955.1 | DL031254.1 | CS174036.1 | AX587918.1 | GM637542.1 | CS545502.1 | BD453735.1 |
| DL118043.1 | HH998154.1 | CS157923.1 | DL031222.1 | CS176256.1 | AX587869.1 | GM637510.1 | CS544974.1 | BD453703.1 |
| DL118011.1 | HH996437.1 | CS157891.1 | DL031190.1 | CS172440.1 | AX587769.1 | GM637478.1 | CS561192.1 | BD453671.1 |
| DL108369.1 | HH991325.1 | CS157859.1 | DL027349.1 | CS172276.1 | AX587716.1 | GM633070.1 | CS560464.1 | BD453639.1 |
| DL103563.1 | HH979529.1 | CS157827.1 | DL027317.1 | CS106096.1 | AX587616.1 | GM633038.1 | HW293034.1 | BD453607.1 |
| DL099021.1 | HH979634.1 | CS143640.1 | DL027285.1 | CS165420.1 | AX587565.1 | GM633006.1 | HW292757.1 | BD493411.1 |

| | | | | | | | | |
|---|---|---|---|---|---|---|---|---|
| DL098989.1 | HH979594.1 | CS124716.1 | DL027253.1 | CS159808.1 | AX584315.1 | GM632974.1 | HW291173.1 | BD453557.1 |
| DL098957.1 | HH932011.1 | CS124594.1 | DL027221.1 | CS159619.1 | AX575320.1 | GM632942.1 | HW291131.1 | BD453525.1 |
| DL038451.1 | HH829712.1 | CS124197.1 | DL027189.1 | CS156109.1 | AX556961.1 | GM632910.1 | HW291076.1 | CQ888106.1 |
| DL038419.1 | FW394411.1 | CS120367.1 | DL027157.1 | CS144365.1 | AX556832.1 | GM632878.1 | HW291044.1 | CQ887981.1 |
| DL038387.1 | HC047458.1 | CS119541.1 | DL023345.1 | CS142500.1 | AX555822.1 | GM625267.1 | HW291012.1 | CQ877627.1 |
| DL038355.1 | HC047426.1 | CS119507.1 | DL023313.1 | CS141534.1 | AX555203.1 | GM625235.1 | HW290948.1 | CQ875507.1 |
| DL038323.1 | HC047362.1 | CS119475.1 | DL023281.1 | CS141502.1 | AX555090.1 | GM697925.1 | HW290916.1 | CQ874863.1 |
| DL013160.1 | HC047330.1 | CS119440.1 | DL023249.1 | CS139085.1 | AX553569.1 | GM635051.1 | HW290885.1 | CQ869283.1 |
| DL013128.1 | HC047298.1 | CS119374.1 | DL023217.1 | CS134658.1 | AX551113.1 | GM635019.1 | HW290853.1 | CQ868893.1 |
| DL013096.1 | HC047266.1 | CS119342.1 | DL023185.1 | CS132066.1 | AX544476.1 | GM634987.1 | HW290132.1 | CQ861196.1 |
| DL013064.1 | HC047010.1 | CS119276.1 | DL020170.1 | CS131489.1 | AX242240.1 | GM634955.1 | HW298237.1 | CQ859633.1 |
| DL013032.1 | HC046978.1 | CS119244.1 | DL020138.1 | CS123605.1 | AX242208.1 | GM634923.1 | HW298082.1 | CQ859601.1 |
| DL013000.1 | HC046946.1 | CS119180.1 | DL020106.1 | CS122745.1 | AX242176.1 | GM634891.1 | HW288959.1 | CQ855860.1 |
| DL033735.1 | HC046914.1 | CS119148.1 | DL020010.1 | CS122261.1 | AX242112.1 | GM630085.1 | HW287769.1 | CQ854738.1 |
| DL042105.1 | HC046854.1 | CS119081.1 | DL015447.1 | CS106189.1 | AX242080.1 | GM630053.1 | HW285135.1 | CQ846363.1 |
| DL029526.1 | HC046822.1 | CS119016.1 | DL010925.1 | HW151686.1 | AX242048.1 | GM630021.1 | HW269697.1 | CQ829255.1 |
| DL029494.1 | HC046790.1 | CS118983.1 | DL010893.1 | HW150492.1 | AX241952.1 | GM629989.1 | HW269240.1 | CQ829217.1 |
| DL041886.1 | HC046758.1 | CS118951.1 | DL010829.1 | HW144519.1 | AX241888.1 | GM629957.1 | HW269105.1 | CQ828028.1 |
| DL019716.1 | HC046726.1 | CS118918.1 | DL023118.1 | HW126551.1 | AX241856.1 | GM629925.1 | HW268482.1 | CQ821313.1 |
| DL010535.1 | HC046694.1 | CS118885.1 | DL023086.1 | HW125527.1 | AX241824.1 | GM642411.1 | HW267853.1 | CQ821036.1 |
| DD402393.1 | HC046677.1 | CS118819.1 | DL023054.1 | HW144380.1 | AX241760.1 | GM642231.1 | HW267803.1 | CQ816999.1 |
| DD402361.1 | HC046581.1 | CS118786.1 | DL023022.1 | HW144348.1 | AX241728.1 | GM656342.1 | HW267621.1 | CQ816956.1 |
| DD402329.1 | HC046549.1 | CS118754.1 | DL022990.1 | HW144316.1 | AX241696.1 | GM656310.1 | HW266157.1 | CQ816922.1 |
| DD402297.1 | HC046517.1 | CS118721.1 | DL019975.1 | HW124822.1 | AX241632.1 | GM656278.1 | HW265995.1 | CQ814049.1 |
| DD402265.1 | HC046485.1 | CS118689.1 | DL019943.1 | HW123665.1 | AX241600.1 | GM656246.1 | HW265861.1 | CQ814017.1 |
| DD402233.1 | HC046453.1 | CS118657.1 | DL019911.1 | HW104005.1 | AX241568.1 | GM656214.1 | HW263034.1 | CQ813985.1 |
| DD402201.1 | HC046389.1 | CS118625.1 | DL019879.1 | HW070680.1 | AX241504.1 | GM656182.1 | HV774487.1 | CQ813953.1 |
| DD405581.1 | HC046357.1 | CS118592.1 | DL019847.1 | HW099618.1 | AX241472.1 | GM649157.1 | HV767895.1 | CQ813921.1 |
| DD405517.1 | HC046325.1 | CS118557.1 | DL019815.1 | HW099550.1 | AX241440.1 | GM649125.1 | HV766946.1 | CQ813889.1 |
| DD405485.1 | HC046074.1 | CS118525.1 | DL015412.1 | HW069678.1 | AX241408.1 | GM649093.1 | HV766811.1 | CQ813857.1 |
| DD401983.1 | HC046010.1 | CS118492.1 | DL015380.1 | HW089223.1 | AX241101.1 | GM649061.1 | HV766727.1 | CQ813825.1 |
| DD401922.1 | HC046250.1 | BD174772.1 | DL015316.1 | HW089009.1 | AX241069.1 | GM649029.1 | HV760618.1 | CQ813793.1 |
| DD401858.1 | HC046218.1 | AX648072.1 | DL015284.1 | HW083503.1 | AX241037.1 | GM642194.1 | HV766526.1 | CQ812764.1 |
| DD401826.1 | HC046186.1 | AX643800.1 | DL014617.1 | HW088826.1 | AX241005.1 | GM642162.1 | HV766094.1 | AX344910.1 |
| DD117037.1 | HC046154.1 | AX642214.1 | DL014585.1 | HW083235.1 | AX240973.1 | GM642130.1 | HV766046.1 | AX344504.1 |
| DD090336.1 | HC046122.1 | BD171599.1 | DL014553.1 | HW082964.1 | AX240941.1 | GM642098.1 | HV766014.1 | AX342743.1 |
| DD089925.1 | HC046004.1 | AX601585.1 | DL014521.1 | HW082732.1 | AX240909.1 | GM642066.1 | HV752853.1 | AX337953.1 |
| DD102594.1 | HC045972.1 | AX601385.1 | DL014457.1 | HW097467.1 | AX239711.1 | CQ986574.1 | HV757534.1 | AX328155.1 |
| DD057931.1 | HC045940.1 | AX601353.1 | DL009999.1 | HW088192.1 | AX235250.1 | CQ986508.1 | HV750068.1 | AX303213.1 |
| DD057899.1 | HC045908.1 | AX599023.1 | DL009967.1 | HW081784.1 | AX225273.1 | CQ983373.1 | HV758623.1 | AX283498.1 |
| DD057867.1 | HC045876.1 | AX598817.1 | DL009935.1 | HW081586.1 | AX208073.1 | CQ983160.1 | HV755065.1 | AX280169.1 |
| DD057839.1 | HC045812.1 | AX598105.1 | DL009903.1 | HW099466.1 | AX207300.1 | CQ983114.1 | HV760046.1 | AX253432.1 |
| DD053295.1 | HC045780.1 | BD096863.1 | DL009871.1 | HW103537.1 | AX205195.1 | CQ983079.1 | HV749597.1 | AX242316.1 |
| DD051874.1 | HC045748.1 | BD090896.1 | DL009839.1 | HW102874.1 | AX202560.1 | CQ983030.1 | HV748401.1 | AX242284.1 |
| DD058903.1 | HC045716.1 | BD087730.1 | DL009807.1 | HW102605.1 | AX202433.1 | CQ982981.1 | AY657841.1 | AX242156.1 |
| DD041900.1 | HC045686.1 | BD085735.1 | DL009775.1 | HW064636.1 | AX195201.1 | CQ982944.1 | AY657809.1 | AX241932.1 |
| DD046590.1 | HC045622.1 | BD081952.1 | DL009743.1 | HW061891.1 | AX193734.1 | CQ982912.1 | AY657777.1 | AX241900.1 |

| | | | | | | | | |
|---|---|---|---|---|---|---|---|---|
| DD038704.1 | HC045590.1 | BD081519.1 | DL009711.1 | HW061858.1 | AX180287.1 | CQ982846.1 | AY657745.1 | AX241836.1 |
| DD034264.1 | HC045526.1 | BD080711.1 | DL009679.1 | HW061826.1 | AX175367.1 | CQ982784.1 | AY657713.1 | AX241804.1 |
| DD032565.1 | DM378911.1 | BD080443.1 | DL009647.1 | HW061812.1 | AX173379.1 | CQ982619.1 | AY657681.1 | AX241772.1 |
| DD032250.1 | HC035671.1 | BD079296.1 | DL009615.1 | HW058824.1 | AX172947.1 | CQ982543.1 | AY657649.1 | AX241740.1 |
| DD031187.1 | HC037108.1 | BD077104.1 | DL009583.1 | HW065208.1 | AX172497.1 | CQ980556.1 | AY657617.1 | AX241708.1 |
| DD023879.1 | HC022642.1 | BD075750.1 | DL009551.1 | HW065098.1 | AX172308.1 | CQ975341.1 | AY657585.1 | AX241676.1 |
| DD017650.1 | HC025472.1 | BD074955.1 | DL009519.1 | HW058537.1 | AX167418.1 | CQ974771.1 | AY657553.1 | AX241612.1 |
| DD009911.1 | HC025440.1 | BD073862.1 | HW320830.1 | HW058432.1 | AX167201.1 | CQ973144.1 | AY657521.1 | AX241580.1 |
| BD412631.1 | HC025408.1 | BD070711.1 | HW329373.1 | HW064842.1 | AX155550.1 | CQ972929.1 | AY657489.1 | AX241548.1 |
| BD434077.1 | HC024864.1 | BD063999.1 | HW336350.1 | HW061078.1 | AX151195.1 | CQ979050.1 | AY657457.1 | AX241516.1 |
| BD453889.1 | HC010426.1 | BD062552.1 | HW336126.1 | HW056584.1 | AX147183.1 | CQ977477.1 | AY657425.1 | AX241484.1 |
| BD453857.1 | HC008378.1 | BD057280.1 | HW328697.1 | HW056552.1 | AX145738.1 | CQ977195.1 | AY657393.1 | AX241452.1 |
| BD453825.1 | HC020912.1 | BD017756.1 | HW328605.1 | HW056402.1 | AX145706.1 | CQ976304.1 | AY657361.1 | AX241420.1 |
| BD453793.1 | HC007806.1 | BD016705.1 | HW319231.1 | HW060578.1 | AX145674.1 | CQ975979.1 | AY657329.1 | AF411479.1 |
| BD433657.1 | HC007742.1 | BD014164.1 | HW328457.1 | HW060527.1 | AX145642.1 | CQ975804.1 | AY657297.1 | HW390740.1 |
| BD453769.1 | HC007678.1 | BD013387.1 | HW328359.1 | HW056320.1 | AX145610.1 | CQ972469.1 | AY657265.1 | HW399803.1 |
| BD453737.1 | HC007618.1 | AX482627.1 | HW328287.1 | HW056221.1 | AX145578.1 | CQ972437.1 | AY657233.1 | HW399360.1 |
| BD453705.1 | DM091319.1 | AX481678.1 | HW319062.1 | HW062384.1 | AX145546.1 | CQ972373.1 | AY657201.1 | HW399312.1 |
| BD453673.1 | GN087801.1 | E61341.1 | HW328095.1 | HW062352.1 | AX145514.1 | CQ972309.1 | AY657169.1 | HW399200.1 |
| BD453641.1 | GN089860.1 | E64483.1 | HW318505.1 | HW062320.1 | AX145482.1 | CQ971604.1 | AY657137.1 | HW408960.1 |
| BD453609.1 | GN089796.1 | AX469471.1 | HW335149.1 | HV491135.1 | AX145450.1 | CQ969210.1 | AY657105.1 | HW382551.1 |
| BD493413.1 | GN082893.1 | AX468890.1 | HW314069.1 | HV503781.1 | AX145418.1 | CQ967876.1 | AY657073.1 | HW390549.1 |
| BD453559.1 | GN088985.1 | AX468465.1 | HW311845.1 | HV503749.1 | AX145385.1 | CQ967776.1 | AY657041.1 | HW390502.1 |
| BD453527.1 | GN088521.1 | AX466977.1 | HW311290.1 | HV503717.1 | AX145353.1 | CQ964448.1 | M25899.1 | HW390464.1 |
| BD453495.1 | GN092005.1 | AX464583.1 | HW307842.1 | HV503685.1 | AX145321.1 | CQ957871.1 | M20738.1 | HW390355.1 |
| BD453463.1 | GN077221.1 | AX458583.1 | HW307810.1 | HV503653.1 | AX145289.1 | CQ955602.1 | M14876.1 | HW389555.1 |
| BD453431.1 | U49851.1 | AX458203.1 | HW307778.1 | HV503593.1 | AX145257.1 | AX357312.1 | M22553.1 | HW387807.1 |
| BD453399.1 | L08752.1 | AX456528.1 | HW316590.1 | HV503561.1 | AX145225.1 | AX356676.1 | M12505.1 | HW380836.1 |
| BD453254.1 | DM059623.1 | AX454155.1 | HW307740.1 | HV503529.1 | AX145193.1 | BD011664.1 | M64610.1 | FW347347.1 |
| BD453222.1 | DM058844.1 | AX453541.1 | HW307708.1 | HV503497.1 | HV542755.1 | BD006817.1 | M59842.1 | HW374972.1 |
| CQ801441.1 | DM042451.1 | AX452039.1 | HW315698.1 | HV503465.1 | HV542723.1 | BD002009.1 | HV549731.1 | HW312150.1 |
| CQ800907.1 | DM040882.1 | AX451640.1 | HW315645.1 | HV503433.1 | HV542186.1 | E54576.1 | HV549298.1 | HW312096.1 |
| CQ795476.1 | DM056942.1 | AX443303.1 | HW315568.1 | HV503401.1 | HV541830.1 | E55389.1 | HV549124.1 | HW311891.1 |
| CQ794279.1 | DM045424.1 | A23861.1 | HW315535.1 | HV503369.1 | HV541499.1 | BD000201.1 | HV549086.1 | HW311768.1 |
| CQ792513.1 | GM639759.1 | A22916.1 | HW315143.1 | HV503337.1 | HV541048.1 | E59175.1 | HV544348.1 | HW311236.1 |
| CQ787403.1 | GM639727.1 | A22289.1 | HW315079.1 | HV503305.1 | HV540037.1 | AX354708.1 | HV544117.1 | HW309212.1 |
| CQ787363.1 | GM639695.1 | A16409.1 | HW315023.1 | HV503273.1 | HV533132.1 | AX352803.1 | HV547845.1 | HW307888.1 |
| CQ787295.1 | GM639663.1 | A22128.1 | HW314957.1 | HV503241.1 | HV532782.1 | AX352516.1 | HV543720.1 | HW307856.1 |
| CQ784705.1 | GM646586.1 | A21757.1 | HW314909.1 | HV503209.1 | HV536543.1 | AX351105.1 | HV543500.1 | HW307792.1 |
| CQ784641.1 | GM646522.1 | A20291.1 | HW314848.1 | HV503186.1 | HV536317.1 | AX349046.1 | HV539691.1 | HW307754.1 |
| CQ778525.1 | GM646458.1 | A13672.1 | HW314746.1 | HV503154.1 | HV532488.1 | AX348528.1 | HV538612.1 | HW307722.1 |
| CQ778493.1 | GM646426.1 | A19546.1 | HW314639.1 | HV503122.1 | HV532385.1 | AX277252.1 | HV538456.1 | HW315871.1 |
| CQ771205.1 | GM639527.1 | A17931.1 | HW151171.1 | HV503090.1 | HV537249.1 | AX347458.1 | HV537608.1 | HW315550.1 |
| CQ759702.1 | GM626852.1 | A17040.1 | HW113243.1 | HV503058.1 | HV535449.1 | AX347376.1 | HV543144.1 | HW315398.1 |
| CQ756600.1 | GM626820.1 | A15382.1 | HW259772.1 | HV503026.1 | HV531958.1 | AX347294.1 | HV542720.1 | HW315157.1 |
| CQ754455.1 | GM653635.1 | A16029.1 | HW258910.1 | HV502994.1 | HV528106.1 | AX347256.1 | HV542688.1 | HW315093.1 |
| CQ754029.1 | GM653603.1 | A15847.1 | HW258845.1 | HV502962.1 | HV515590.1 | AX347224.1 | HV542183.1 | HW314813.1 |

| | | | | | | | | |
|---|---|---|---|---|---|---|---|---|
| AX962025.1 | GM660768.1 | A13975.1 | HW257425.1 | HV502930.1 | HV515558.1 | AX347186.1 | HV541819.1 | HW314763.1 |
| AX960391.1 | GM653517.1 | A13382.1 | HW257361.1 | HV502898.1 | HV515526.1 | AX345616.1 | HV541495.1 | HW070167.1 |
| AX960359.1 | GM653485.1 | A12753.1 | HW257329.1 | HV502866.1 | HV515494.1 | AX344134.1 | DQ156557.1 | HW069941.1 |
| AX937797.1 | GM653453.1 | A10837.1 | HW257297.1 | HV502834.1 | HV515462.1 | AJ414578.1 | HV515061.1 | HW069809.1 |
| AX142337.1 | GM653421.1 | A10370.1 | HW257265.1 | HV492862.1 | HV515430.1 | AX339190.1 | DJ028599.1 | HW068049.1 |
| AX142081.1 | GM653389.1 | A09229.1 | HW257041.1 | HV502790.1 | HV515398.1 | AX329438.1 | DJ044935.1 | HW089024.1 |
| AX141951.1 | GM646390.1 | A08277.1 | HW256881.1 | HV502758.1 | HV515366.1 | AX328290.1 | DJ044894.1 | HV956198.1 |
| AX141885.1 | GM646358.1 | A08047.1 | HW256849.1 | HV502726.1 | HV515334.1 | AX326769.1 | DJ044657.1 | HV951098.1 |
| AX141565.1 | GM646326.1 | A06450.1 | HW256785.1 | HV502694.1 | HV515238.1 | AX323774.1 | DJ028142.1 | HV949969.1 |
| AX141501.1 | GM646294.1 | A06036.1 | HW256721.1 | HV502662.1 | HV510393.1 | AX319641.1 | DJ033850.1 | HV942842.1 |
| AX141437.1 | GM646262.1 | A04929.1 | HW254445.1 | HV502630.1 | HV510361.1 | AX306880.1 | DJ030173.1 | HW099579.1 |
| AX141373.1 | GM646230.1 | A01184.1 | HW251080.1 | HV502598.1 | HV510064.1 | AX306590.1 | BD266838.1 | HW099521.1 |
| AX141309.1 | GM639427.1 | A07987.1 | HW251024.1 | HV502566.1 | HV513367.1 | AX306323.1 | BD265619.1 | HW102177.1 |
| AX138281.1 | GM639395.1 | A06076.1 | HW250349.1 | HV502534.1 | HV513271.1 | AX268765.1 | BD263425.1 | HW088903.1 |
| AX137834.1 | GM639363.1 | A01766.1 | HW249718.1 | HV502502.1 | HV508665.1 | AX260031.1 | BD251971.1 | HW088813.1 |
| AX136894.1 | GM639331.1 | A05312.1 | HW249677.1 | HV502470.1 | HV508630.1 | AX259234.1 | BD251467.1 | HW097517.1 |
| AX134956.1 | GM639299.1 | A04275.1 | HW247847.1 | HV497322.1 | HV508566.1 | AX304320.1 | BD251237.1 | HW088232.1 |
| AX134224.1 | GM639267.1 | A01385.1 | HW247807.1 | HV492719.1 | HV508534.1 | AX300061.1 | BD250306.1 | HW102886.1 |
| AX127337.1 | GM660565.1 | A00282.1 | HW240873.1 | HV505349.1 | HV508499.1 | AX297723.1 | BD249062.1 | HW102825.1 |
| AX118836.1 | GM660533.1 | K02389.1 | HW240777.1 | HV507703.1 | HV508474.1 | AX287803.1 | BD247114.1 | HW102793.1 |
| AX107117.1 | GM653346.1 | M12841.1 | HW240721.1 | HV450044.1 | HV512417.1 | AX286560.1 | BD247017.1 | HW061874.1 |
| AX101038.1 | GM653314.1 | K02753.1 | HW240657.1 | HW260452.1 | HV512276.1 | AX283694.1 | BD246845.1 | HW061841.1 |
| AX097930.1 | GM653282.1 | M10179.1 | HW247940.1 | HW260388.1 | HV512244.1 | AX283228.1 | BD244791.1 | HW065435.1 |
| AX097508.1 | GM653250.1 | M33416.1 | HW239406.1 | HW260356.1 | HV512212.1 | AF368502.1 | BD243447.1 | HW065289.1 |
| AX093087.1 | GM653218.1 | M55324.1 | HW239186.1 | HW260324.1 | HV512148.1 | AX282199.1 | BD242717.1 | HW065256.1 |
| AX088681.1 | DL091270.1 | OM981240.1 | HW238894.1 | HW260292.1 | JA660232.1 | AX281226.1 | BD242462.1 | HW065223.1 |
| CS119532.1 | DL091238.1 | MK327979.1 | HW238531.1 | HW260260.1 | HV492184.1 | AX279965.1 | BD242382.1 | HW058480.1 |
| CS119466.1 | DL091206.1 | U21234.1 | HW238412.1 | HW260228.1 | FZ429270.1 | AX279776.1 | BD240793.1 | HW064825.1 |
| CS119432.1 | DL087206.1 | AH002292.2 | HW238373.1 | HW260196.1 | FZ437402.1 | AX278765.1 | BD238040.1 | HW055563.1 |
| CS119300.1 | DL102099.1 | M35112.1 | HW243005.1 | HW260164.1 | FZ437355.1 | AX278107.1 | BD237489.1 | HW064784.1 |
| CS119268.1 | DL102067.1 | DQ250185.1 | HW242967.1 | HW260132.1 | FZ436855.1 | AX256282.1 | BD236847.1 | HW064689.1 |
| CS119236.1 | DL102035.1 | AY521454.1 | HW238008.1 | HV822843.1 | FZ436789.1 | AX254826.1 | BD235798.1 | HW064645.1 |
| AX666542.1 | DL097525.1 | AY383481.1 | HW244270.1 | HV812996.1 | FZ436703.1 | AX253168.1 | BD235650.1 | HW062424.1 |
| AX662196.1 | DL097493.1 | AF003707.1 | HW237902.1 | HV817915.1 | FZ423103.1 | AX251576.1 | BD234877.1 | HW062392.1 |
| AX657133.1 | DL097461.1 | AF543345.1 | HW237867.1 | HV822255.1 | FZ431985.1 | AX249913.1 | BD233454.1 | HW056343.1 |
| AX657101.1 | DL101916.1 | AF527046.1 | HW237430.1 | HV816785.1 | FZ422779.1 | AX242336.1 | BD232351.1 | HW064466.1 |
| AX615145.1 | DL101886.1 | S71730.1 | HW242323.1 | HV784498.1 | FZ413375.1 | AX242304.1 | BD231170.1 | HW062367.1 |
| BD171488.1 | DL101822.1 | KJ668650.1 | HW242291.1 | HV780571.1 | FZ418823.1 | AX242272.1 | BD231137.1 | HW062335.1 |
| AX614779.1 | DL101790.1 | HW408834.1 | HW242259.1 | HV780155.1 | FZ421399.1 | HW351287.1 | BD227423.1 | HV208590.1 |
| AX601793.1 | DL101758.1 | HW408770.1 | HW237018.1 | HV779583.1 | FZ421219.1 | HW351159.1 | BD226446.1 | HV218083.1 |
| AX601609.1 | DL101726.1 | HW408738.1 | HW243436.1 | HV778824.1 | FZ421007.1 | HW350700.1 | BD225788.1 | HV307961.1 |
| AX601513.1 | DL097216.1 | HW408647.1 | HW248347.1 | HV778231.1 | FZ420878.1 | HW350655.1 | BD225756.1 | HV306169.1 |
| AX601377.1 | DL097184.1 | HW408615.1 | HW241739.1 | HV775825.1 | FZ420747.1 | HW350586.1 | BD225724.1 | HV302963.1 |
| AX601345.1 | DL097152.1 | HW408487.1 | HW241543.1 | HV764710.1 | FZ414799.1 | GM642459.1 | BD225692.1 | HV226931.1 |
| AX601312.1 | DL095228.1 | HW408360.1 | HW151765.1 | HV769878.1 | FZ417003.1 | GM642427.1 | BD225660.1 | HV247040.1 |
| AX593507.1 | DL095196.1 | HW158818.1 | HW159693.1 | HV763595.1 | FZ413927.1 | GM635245.1 | BD225628.1 | HV305476.1 |
| BD166160.1 | DL095164.1 | HW158386.1 | HW159658.1 | HV767881.1 | FZ416713.1 | GM635213.1 | BD224842.1 | HV188411.1 |

| | | | | | | | | |
|---|---|---|---|---|---|---|---|---|
| BD161050.1 | DL125267.1 | HW154650.1 | HW144668.1 | HV766942.1 | FZ416681.1 | GM635181.1 | BD224181.1 | HV200229.1 |
| BD144624.1 | DL125235.1 | GN081999.1 | HW144628.1 | HV766806.1 | FZ419832.1 | GM635149.1 | BD223261.1 | HV182446.1 |
| BD143206.1 | DL125203.1 | DL233272.1 | HW144520.1 | HV766726.1 | FZ419798.1 | GM635117.1 | BD222981.1 | HV182382.1 |
| AX587891.1 | DL125171.1 | DL232594.1 | HW126553.1 | HV760617.1 | FZ419766.1 | GM635085.1 | BD222516.1 | HV220758.1 |
| AX576545.1 | DL111838.1 | GN078436.1 | HW125528.1 | HV766525.1 | FZ419734.1 | GM630279.1 | BD222001.1 | HV203124.1 |
| AX573492.1 | DL111806.1 | GN078784.1 | HW144381.1 | HV766311.1 | FZ419698.1 | GM630215.1 | BD217964.1 | HV235484.1 |
| AX565546.1 | DL111774.1 | L08911.1 | HW144349.1 | HV766093.1 | FZ416534.1 | GM630183.1 | BD209846.1 | HV112897.1 |
| AX556846.1 | DL128889.1 | M20261.1 | HW144317.1 | HV766045.1 | HQ233649.1 | GM630151.1 | BD205393.1 | FW498880.1 |
| AX556812.1 | DL128855.1 | DM059630.1 | HW144285.1 | HV766013.1 | FW582641.1 | GM630119.1 | BD195022.1 | FW420459.1 |
| AX098931.1 | DL128822.1 | DM058854.1 | HW124825.1 | HV755840.1 | FW582264.1 | GM635050.1 | DD283501.1 | FW496895.1 |
| AX097507.1 | DL116421.1 | DM058793.1 | HW123470.1 | HV592294.1 | FW591965.1 | GM635018.1 | CS353537.1 | FW420370.1 |
| AX097386.1 | DL116389.1 | DM063076.1 | HW122738.1 | HV582154.1 | FW590932.1 | GM634986.1 | CS353031.1 | FW420338.1 |
| AX093086.1 | DL116357.1 | DM045538.1 | HW121649.1 | HV581986.1 | FW590738.1 | GM634954.1 | DD236638.1 | FW496635.1 |
| AX088780.1 | DL111636.1 | DM056696.1 | HW120380.1 | HV575482.1 | FW590122.1 | GM634890.1 | DD235245.1 | HI653830.1 |
| AX088722.1 | DL111604.1 | DM045268.1 | HW120102.1 | HV586268.1 | FW588900.1 | GM630084.1 | DD234710.1 | HI653554.1 |
| AX085335.1 | DL111572.1 | DM044990.1 | HW115075.1 | HV601086.1 | FW588145.1 | GM630052.1 | DD248259.1 | HI653392.1 |
| AX080901.1 | DL111540.1 | DM044673.1 | HW114437.1 | HV574483.1 | FW592673.1 | GM630020.1 | DD247169.1 | HI651888.1 |
| AX078779.1 | DL111508.1 | DM060746.1 | HW113662.1 | HV580576.1 | FW592641.1 | GM629988.1 | DD246846.1 | HI651014.1 |
| AX077105.1 | DL111476.1 | DM060682.1 | HW117662.1 | HV585653.1 | HH796570.1 | GM629956.1 | E46947.1 | AX242315.1 |
| AX076575.1 | DL101694.1 | DM044368.1 | HW081525.1 | HV570659.1 | HH795980.1 | GM629924.1 | E37929.1 | AX242283.1 |
| AX061424.1 | DL101662.1 | GN075512.1 | HW087217.1 | HV569313.1 | HH794726.1 | GM642410.1 | E44036.1 | AX242219.1 |
| AX068067.1 | DL101630.1 | GN067897.1 | HW028621.1 | HV568107.1 | HH791753.1 | GM642232.1 | E37592.1 | AX242187.1 |
| AX052941.1 | DL097120.1 | GN067865.1 | HV986640.1 | HV568051.1 | HH779706.1 | GM642274.1 | E39200.1 | AX242155.1 |
| AX049323.1 | DL097088.1 | GN067801.1 | HV969567.1 | HV567899.1 | HH777927.1 | GM656341.1 | E05673.1 | AX242123.1 |
| AX040761.1 | DL114435.1 | GN067677.1 | HV960891.1 | HV566760.1 | HH774195.1 | GM656309.1 | E05425.1 | AX242027.1 |
| AX038740.1 | DL114403.1 | DM003505.1 | HV960570.1 | HV566062.1 | HH820966.1 | GM656277.1 | E03692.1 | AX241995.1 |
| AX036012.1 | DL114371.1 | DL489802.1 | HV960150.1 | HV573537.1 | HH820924.1 | GM656245.1 | E03542.1 | AX241931.1 |
| AX028798.1 | DL114339.1 | DM003142.1 | HV959932.1 | HV573503.1 | HH759577.1 | GM656213.1 | E02917.1 | AX241899.1 |
| AX023690.1 | DL104580.1 | DM003106.1 | HV959695.1 | HV572424.1 | HH759186.1 | GM656181.1 | E01775.1 | AX241835.1 |
| AX023640.1 | DL104548.1 | DM008738.1 | HV959481.1 | HV572124.1 | HH759154.1 | GM656149.1 | E01684.1 | AX241803.1 |
| AX023607.1 | DL104516.1 | GM998433.1 | HV970073.1 | HV571421.1 | HH759122.1 | GM649156.1 | E01581.1 | AX241771.1 |
| AX023574.1 | DL093932.1 | GM997936.1 | HV963868.1 | HV558752.1 | HH759090.1 | GM649124.1 | E01250.1 | AX241739.1 |
| AX020374.1 | DL093900.1 | GM997547.1 | HV963661.1 | HV561852.1 | HH759058.1 | GM649092.1 | E00322.1 | AX241707.1 |
| AX019926.1 | DL093868.1 | GM996594.1 | HV966129.1 | HV554504.1 | HH759026.1 | GM649060.1 | DD143661.1 | AX241675.1 |
| AX019435.1 | DL090093.1 | GM996498.1 | HV963262.1 | HV554019.1 | HH758994.1 | GM649028.1 | DD069973.1 | AX241643.1 |
| AX018739.1 | DL090029.1 | GM996248.1 | HV965987.1 | HV339965.1 | BD398163.1 | GM642193.1 | DD069882.1 | AX241611.1 |
| AX014771.1 | DL123348.1 | GM980117.1 | HV962412.1 | HV342424.1 | BD408121.1 | GM642161.1 | DD112555.1 | AX241579.1 |
| AX010929.1 | DL123316.1 | GM994844.1 | HV962272.1 | HV335556.1 | BD408082.1 | GM642129.1 | DD143254.1 | AX241547.1 |
| AX008753.1 | DL123284.1 | GM994465.1 | HV962178.1 | HV342011.1 | BD450225.1 | GM642097.1 | DD084336.1 | AX241515.1 |
| HW350967.1 | DL123252.1 | GM994338.1 | HV950503.1 | HV341805.1 | BD439818.1 | GM642065.1 | DD098185.1 | AX241483.1 |
| HW350892.1 | DL042142.1 | GM992948.1 | HV957871.1 | HV323135.1 | BD449661.1 | GM642033.1 | DD132356.1 | AF411478.1 |
| HW350668.1 | DL042110.1 | GM992644.1 | HV949006.1 | HV333135.1 | BD449380.1 | GM634846.1 | DD141440.1 | AX212287.1 |
| HW340093.1 | DL042078.1 | GM992270.1 | HV947294.1 | HV332143.1 | BD418446.1 | GM634814.1 | DD153623.1 | AX207675.1 |
| HW339889.1 | DL029531.1 | GM992045.1 | HV953250.1 | HV332101.1 | BD429044.1 | GM952835.1 | DD158436.1 | AX207119.1 |
| HW349931.1 | DL029499.1 | GM991913.1 | HV951946.1 | HV325397.1 | BD396636.1 | GM894238.1 | DD158413.1 | AX205063.1 |
| HW347910.1 | DL029467.1 | GM976285.1 | HV951809.1 | HV331790.1 | BD428591.1 | GM893719.1 | DD157724.1 | AX203095.1 |
| HW363667.1 | DL021104.1 | GM990788.1 | HV946792.1 | HV324877.1 | BD437757.1 | GM890253.1 | DD157355.1 | AX195447.1 |

| | | | | | | | | |
|---|---|---|---|---|---|---|---|---|
| HW326534.1 | DL021072.1 | GM989734.1 | HV946278.1 | HV324780.1 | BD437671.1 | GM890125.1 | DD153650.1 | AX193986.1 |
| HW338938.1 | DL021040.1 | GM989053.1 | HV940061.1 | HV324043.1 | BD437230.1 | GM963584.1 | DD152596.1 | AX191709.1 |
| HW338682.1 | DL021008.1 | GM987268.1 | HV945937.1 | HV043013.1 | BD437120.1 | GM889328.1 | DD096745.1 | AX189782.1 |
| HW338554.1 | DL016738.1 | GM999531.1 | HV944544.1 | HV111852.1 | BD394867.1 | GM828396.1 | DD081760.1 | AX179708.1 |
| HW338298.1 | DL012184.1 | GM969672.1 | HV943281.1 | HV098621.1 | BD446176.1 | GM888716.1 | DD081683.1 | AX174861.1 |
| HW338170.1 | DL012152.1 | GM969400.1 | HV942955.1 | HV187255.1 | BD393742.1 | GM008872.1 | DD147793.1 | AX172508.1 |
| HW337914.1 | DL012120.1 | GM968443.1 | HV942062.1 | HV189877.1 | BD453357.1 | GM888519.1 | DD161082.1 | AX172442.1 |
| HW096699.1 | DL012088.1 | GN007499.1 | HV932149.1 | HV218553.1 | BD453327.1 | GM008187.1 | DD178466.1 | AX167810.1 |
| HW072896.1 | DL012056.1 | FB986397.1 | HV929263.1 | HV227947.1 | BD453295.1 | GM872374.1 | DD166304.1 | AX153821.1 |
| HW105299.1 | DL012024.1 | FB986057.1 | HV925762.1 | HV234139.1 | BD452695.1 | GM006308.1 | DD165974.1 | AY034052.1 |
| HW096206.1 | DL011934.1 | FB741745.1 | HV931457.1 | HV214865.1 | BD444975.1 | GM005939.1 | HC042760.1 | AX146304.1 |
| HW104294.1 | DL011902.1 | GM969015.1 | HV815187.1 | HV234162.1 | BD413601.1 | FB984708.1 | HC047461.1 | AX145717.1 |
| HW071227.1 | DL024281.1 | GM970612.1 | HV925501.1 | HV236011.1 | BD444961.1 | GM003582.1 | HC047397.1 | AX145685.1 |
| HW071117.1 | DL024249.1 | GM970174.1 | HV936397.1 | HV301807.1 | BD389051.1 | FB983575.1 | HC047365.1 | AX145653.1 |
| HW070912.1 | DL024217.1 | FB736413.1 | HV936314.1 | HV306185.1 | BD376365.1 | FB983218.1 | HC047333.1 | AX145621.1 |
| HW103731.1 | DL016408.1 | GM969859.1 | HV925244.1 | HV303092.1 | BD376048.1 | FB983166.1 | HC047269.1 | AX145589.1 |
| HW099580.1 | DL016376.1 | GM969740.1 | HV819478.1 | HV301533.1 | BD374050.1 | FB753634.1 | HC047045.1 | AX145557.1 |
| HW099522.1 | DL016344.1 | FB777981.1 | HV819368.1 | HV308817.1 | BD360143.1 | GM865886.1 | HC047013.1 | AX145525.1 |
| HW068961.1 | DL016248.1 | GM009519.1 | HV803291.1 | HV302649.1 | BD356473.1 | GM618792.1 | HC046981.1 | AX145493.1 |
| HW083251.1 | DL011732.1 | DL480006.1 | HV930257.1 | HV311093.1 | BD350312.1 | GM865367.1 | HC046949.1 | AX145461.1 |
| HW083219.1 | DL011700.1 | DL465762.1 | HV932730.1 | HI424108.1 | BD359223.1 | FB725963.1 | HC046917.1 | AX145429.1 |
| HW088816.1 | DL011668.1 | DL477847.1 | HV932697.1 | HI424076.1 | BD345165.1 | FB724507.1 | HC046857.1 | AX145397.1 |
| HW088251.1 | DL011636.1 | DL464574.1 | HV932665.1 | HI423786.1 | BD342519.1 | GM618428.1 | HC046825.1 | HW260115.1 |
| HW081602.1 | DL011604.1 | DL470950.1 | HV932633.1 | HI423562.1 | BD341877.1 | GM731774.1 | HC046793.1 | HW260083.1 |
| HW084509.1 | DL048459.1 | DL477013.1 | HV929579.1 | HH762726.1 | BD338947.1 | FB711608.1 | HC046761.1 | HW260051.1 |
| HW103127.1 | DL029839.1 | DL476632.1 | HV803151.1 | HI416362.1 | BD325661.1 | GM967132.1 | HC046729.1 | HW260019.1 |
| HW102887.1 | DL029743.1 | DL462984.1 | HV803119.1 | HI416154.1 | CQ986573.1 | GM042808.1 | HC046697.1 | HW259987.1 |
| HW102794.1 | DL018280.1 | DL476032.1 | HV803087.1 | GN367548.1 | CQ986539.1 | GM041235.1 | HC046680.1 | HW259955.1 |
| HW102761.1 | DL018248.1 | DL481962.1 | HV802949.1 | GN373337.1 | CQ983372.1 | FB709620.1 | HC046648.1 | HW259923.1 |
| HW061875.1 | DL018216.1 | GM643677.1 | HV802917.1 | GN370749.1 | CQ982980.1 | GM839361.1 | HC046616.1 | HW258818.1 |
| HW061842.1 | DL013814.1 | GM643637.1 | HV802885.1 | GN360128.1 | CQ982911.1 | CS728655.1 | HC046584.1 | HW257310.1 |
| HV702748.1 | DL013782.1 | GM636648.1 | HV802844.1 | GN356165.1 | CQ982844.1 | CS728591.1 | HC046552.1 | HW257246.1 |
| FB570823.1 | DL013750.1 | GM636616.1 | HV822845.1 | GN360055.1 | CQ982782.1 | CS728559.1 | HC046520.1 | HW257214.1 |
| CS696185.1 | DL013718.1 | GM636584.1 | HV813017.1 | GN359991.1 | CQ982617.1 | CS727589.1 | HC046488.1 | HW257182.1 |
| CS696153.1 | DL013686.1 | GM636520.1 | HV702477.1 | GN359895.1 | CQ982542.1 | CS727335.1 | HC046456.1 | HW257086.1 |
| CS696089.1 | DL000623.1 | GM636488.1 | HV702263.1 | GN366321.1 | CQ975372.1 | FB708984.1 | HC046424.1 | HW256958.1 |
| CS696057.1 | DL005726.1 | GM636456.1 | HV701281.1 | GN366289.1 | CQ974770.1 | GM719139.1 | HC046392.1 | HW256926.1 |
| CS696025.1 | DL005435.1 | GM632048.1 | HV701219.1 | GN359818.1 | CQ973143.1 | GM011207.1 | HC046360.1 | HW256798.1 |
| CS695960.1 | DJ493965.1 | GM632016.1 | HV701187.1 | GN359754.1 | CQ972803.1 | GM888671.1 | HC046328.1 | HW256702.1 |
| CS695896.1 | DL008500.1 | GM631984.1 | HV701155.1 | GN013378.1 | CQ977475.1 | GM887783.1 | HC046077.1 | HW251046.1 |
| CS695832.1 | DJ491555.1 | GM623117.1 | HV701123.1 | GN010221.1 | CQ977102.1 | GM685843.1 | HC046045.1 | HW240854.1 |
| CS695800.1 | DJ446854.1 | GM623085.1 | HV708777.1 | GN033551.1 | CQ976303.1 | GM003681.1 | HC046013.1 | HW247744.1 |
| CS695768.1 | DJ446821.1 | GM623053.1 | HV708527.1 | GN033519.1 | CQ975978.1 | FB754124.1 | HC046253.1 | HW240606.1 |
| CS695736.1 | DJ437350.1 | GM623021.1 | HV708477.1 | GN033487.1 | CQ975798.1 | GM879379.1 | HC046221.1 | HW239142.1 |
| CS695704.1 | DJ437223.1 | GM622989.1 | HV701104.1 | GN033423.1 | CQ972372.1 | GM680787.1 | HC046189.1 | HW238389.1 |
| CS695672.1 | DJ437171.1 | GM622957.1 | HV701072.1 | GN033391.1 | CQ972340.1 | FB728338.1 | HC046007.1 | HW238252.1 |
| CS695512.1 | DJ437141.1 | GM650443.1 | HV695534.1 | GN033359.1 | CQ971570.1 | GM842467.1 | HC045975.1 | HW244236.1 |

| | | | | | | | | |
|---|---|---|---|---|---|---|---|---|
| CS695480.1 | DJ437040.1 | GM650411.1 | HV695502.1 | GN033199.1 | CQ971079.1 | FB722372.1 | HC045943.1 | HW237925.1 |
| CS695448.1 | DJ436823.1 | GM643603.1 | HV695470.1 | GN033135.1 | CQ970075.1 | DL105362.1 | GN367746.1 | HW237880.1 |
| CS695416.1 | DJ445570.1 | GM643571.1 | HV695438.1 | GN033103.1 | CQ969106.1 | DL030388.1 | GN367587.1 | HW248663.1 |
| CS695384.1 | DJ430807.1 | GM643539.1 | HV695165.1 | GN033039.1 | CQ967774.1 | DL026531.1 | GN367549.1 | HW237524.1 |
| DL176422.1 | DJ444562.1 | GM643507.1 | HV694957.1 | GN033007.1 | CQ964447.1 | DL026475.1 | GN373338.1 | HW061873.1 |
| DL174611.1 | DJ434798.1 | GM643475.1 | HV694925.1 | GN032975.1 | CQ957870.1 | DL026443.1 | GN370761.1 | HW061840.1 |
| DL174455.1 | DJ438351.1 | GM643442.1 | HV694850.1 | GN032943.1 | CQ956000.1 | DL026411.1 | GN367866.1 | HW065222.1 |
| DL176755.1 | DJ438317.1 | HV932148.1 | HV694783.1 | GN032911.1 | CQ947455.1 | DL026379.1 | GN360161.1 | HW061322.1 |
| DL102883.1 | DJ438284.1 | HV929262.1 | HV700736.1 | GN032879.1 | CQ947131.1 | DL022567.1 | GN360097.1 | HW058479.1 |
| DL102851.1 | DJ438202.1 | HV925759.1 | HV700704.1 | GN032847.1 | CQ944201.1 | DL022535.1 | GN356166.1 | HW064824.1 |
| DL102819.1 | DJ427848.1 | HV931456.1 | HV700672.1 | GN032815.1 | CQ944169.1 | DL022461.1 | GN366657.1 | HW055560.1 |
| DL016399.1 | DJ402639.1 | HV931391.1 | HV694748.1 | GN032783.1 | CQ944137.1 | DL022495.1 | GN366476.1 | HW064720.1 |
| DL016367.1 | DJ402607.1 | HV820134.1 | HV694684.1 | GN032752.1 | CQ944105.1 | DL022439.1 | GN366443.1 | HW064688.1 |
| DL016303.1 | DJ402541.1 | HV815185.1 | HV694647.1 | GN032720.1 | CQ944073.1 | DL022407.1 | GN366411.1 | HW064644.1 |
| DL016271.1 | CS617803.1 | HV925498.1 | HV700226.1 | GN032688.1 | CQ944041.1 | DL019200.1 | GN366347.1 | HW062423.1 |
| DL016239.1 | CS616853.1 | HV694434.1 | HV693208.1 | GN032656.1 | CQ944009.1 | DL014654.1 | GN359960.1 | HW062391.1 |
| DL011723.1 | CS614453.1 | HV700225.1 | HV699507.1 | GN032623.1 | CQ943977.1 | DL010179.1 | GN359928.1 | HW062334.1 |
| DL011659.1 | CS613790.1 | HV693207.1 | HV699087.1 | GN032591.1 | CQ943945.1 | DL010147.1 | GN366290.1 | HW056101.1 |
| DL011627.1 | CS613386.1 | HV699506.1 | HV698177.1 | GN032559.1 | CQ943913.1 | DL010115.1 | GN366258.1 | HW042089.1 |
| DL044737.1 | CS612784.1 | HV699086.1 | HC729000.1 | GN032528.1 | CQ943881.1 | DL010083.1 | GN359755.1 | HW042057.1 |
| DL044705.1 | CS611836.1 | HV698537.1 | HC728801.1 | GN032496.1 | CQ943849.1 | DL010051.1 | GN359691.1 | HW042025.1 |
| DL027171.1 | CS604542.1 | HV689943.1 | HC727690.1 | GN032464.1 | CQ918573.1 | DL010019.1 | GN359639.1 | HW043555.1 |
| DL022975.1 | CS604478.1 | HV695681.1 | FU772391.1 | GN032432.1 | CQ903786.1 | DL030079.1 | GN359575.1 | HW042013.1 |
| DL014503.1 | CS604446.1 | HV688822.1 | FU772342.1 | GN032368.1 | CQ898655.1 | DL030047.1 | GN359543.1 | HW041981.1 |
| DL014471.1 | CS604414.1 | HV600536.1 | FU760027.1 | GN032335.1 | CQ898623.1 | DL030015.1 | GN359511.1 | DL176629.1 |
| DL014439.1 | CS604382.1 | HV579916.1 | FU764387.1 | GN032303.1 | CQ898591.1 | DL029983.1 | GN359411.1 | DL176473.1 |
| DL030258.1 | CS604350.1 | HV579754.1 | FU757698.1 | GN032271.1 | CQ898559.1 | DL029951.1 | GN365280.1 | DL176401.1 |
| DL009306.1 | CS604318.1 | HV585129.1 | FU756913.1 | GN032207.1 | CQ898257.1 | DL046640.1 | GN359242.1 | DL174609.1 |
| DL009274.1 | CS604222.1 | HV584959.1 | HC438975.1 | GN032175.1 | CQ897931.1 | DL046576.1 | GN359210.1 | DL174447.1 |
| DL009242.1 | CS604190.1 | HV584865.1 | HC438424.1 | GN032143.1 | CQ893712.1 | DL046544.1 | GN359178.1 | DL176744.1 |
| DL018700.1 | CS603998.1 | HV579189.1 | HC436476.1 | GN032111.1 | CQ892579.1 | DL046512.1 | GN359114.1 | DL163231.1 |
| DL018668.1 | CS603806.1 | HV592878.1 | HC358228.1 | GN032079.1 | CQ891354.1 | DL046480.1 | GN363571.1 | CS368046.1 |
| DL018636.1 | CS603774.1 | HV579012.1 | HC358196.1 | GN032047.1 | CQ890281.1 | DL042658.1 | GN363033.1 | CS367918.1 |
| DL014208.1 | CS603678.1 | HV578974.1 | HC358065.1 | GN032015.1 | CQ888087.1 | DL042626.1 | GN346513.1 | DL080573.1 |
| DL014176.1 | CS603486.1 | HV592426.1 | HC357632.1 | GN031983.1 | CQ879662.1 | DL042594.1 | GN346479.1 | DL075916.1 |
| DL014144.1 | CS603454.1 | HV592295.1 | HC434754.1 | GN031951.1 | CQ877351.1 | DL042562.1 | GN346899.1 | DL078918.1 |
| DL042640.1 | CS603422.1 | HV591561.1 | HC357075.1 | GN031919.1 | AX429494.1 | DL042530.1 | GN346586.1 | DL074231.1 |
| DL042576.1 | CS603390.1 | HV582260.1 | HC320722.1 | GN031855.1 | AX429242.1 | DL042498.1 | GN346554.1 | DL077374.1 |
| DL038556.1 | CS603358.1 | HV582155.1 | HC356184.1 | GN031725.1 | AX428231.1 | DL038670.1 | GN339096.1 | DL022224.1 |
| CS604752.1 | CS603326.1 | HV581987.1 | HC325470.1 | GN031661.1 | AX427123.1 | DL038638.1 | GN130958.1 | DL011721.1 |
| CS604622.1 | CS603294.1 | HV601363.1 | HC324506.1 | GN031629.1 | AX419093.1 | DL038606.1 | GM633441.1 | DL011689.1 |
| CS604590.1 | CS603262.1 | HV586269.1 | HC320343.1 | GN031565.1 | HV503124.1 | DL034301.1 | GM633409.1 | DL011657.1 |
| CS592362.1 | CS603230.1 | HV601194.1 | HC316425.1 | GN031533.1 | HV503092.1 | DL034269.1 | GM633377.1 | DL011625.1 |
| CS592090.1 | CS603198.1 | HV601087.1 | HC309746.1 | GN031501.1 | HV503060.1 | DL034237.1 | GM633345.1 | DL031202.1 |
| CS598043.1 | CS603166.1 | HV574484.1 | HC308772.1 | GN031469.1 | HV503028.1 | DL034205.1 | GM633313.1 | DL042928.1 |
| CS593392.1 | CS603134.1 | HV580577.1 | HC307867.1 | GN031436.1 | HV502996.1 | DL034173.1 | GM633281.1 | DL009304.1 |
| CS597712.1 | HI003198.1 | HV585698.1 | HC307373.1 | GN031404.1 | HV502964.1 | DL034109.1 | GM637826.1 | DL009272.1 |

| | | | | | | | | |
|---|---|---|---|---|---|---|---|---|
| CS585251.1 | HI003136.1 | HV585655.1 | HC312263.1 | GN031372.1 | HV502932.1 | DL029912.1 | GM633258.1 | DL009240.1 |
| CS583653.1 | HI003076.1 | HV585551.1 | FU258178.1 | GN031340.1 | HV502900.1 | DL029848.1 | GM633226.1 | DL009086.1 |
| CS575803.1 | HI003040.1 | HV574080.1 | FU257870.1 | GN031275.1 | HV502868.1 | DL029816.1 | GM633194.1 | DL014206.1 |
| CS573062.1 | HI002999.1 | HV570599.1 | FU257838.1 | GN031211.1 | HV502836.1 | DL029784.1 | GM633162.1 | DL014174.1 |
| CS570716.1 | HI002937.1 | HV569345.1 | FU257806.1 | GN031179.1 | HV502792.1 | DL029752.1 | GM633130.1 | DL014142.1 |
| CS546685.1 | HI002900.1 | HV568052.1 | FU257741.1 | GN031146.1 | HV502760.1 | DL025942.1 | GM625519.1 | DL042638.1 |
| CS560402.1 | HI001263.1 | HV567900.1 | FU271506.1 | GN031114.1 | HV502728.1 | DL025910.1 | GM625487.1 | DL042574.1 |
| DD431740.1 | HI001203.1 | HV566063.1 | FU263077.1 | GN031082.1 | HV502696.1 | DL025878.1 | GM625455.1 | DL029764.1 |
| CS540038.1 | HI001152.1 | HV573538.1 | DM382858.1 | GN031050.1 | HV502664.1 | DL025846.1 | GM625423.1 | CS604403.1 |
| CS539888.1 | HI001051.1 | GM992194.1 | DM381857.1 | GN030986.1 | HV502632.1 | DL025814.1 | GM625391.1 | CS603475.1 |
| CS541693.1 | HI001015.1 | GM992109.1 | HC054880.1 | GN030955.1 | HV502600.1 | DL025782.1 | GM625359.1 | CS603443.1 |
| CS531812.1 | HI000973.1 | GM992042.1 | HC053727.1 | GN030923.1 | HV502568.1 | DL069492.1 | GM651776.1 | CS603411.1 |
| CS502509.1 | HI002874.1 | GM976278.1 | HC051943.1 | GN030891.1 | HV502536.1 | DL018385.1 | GM744675.1 | CS603187.1 |
| BD226838.1 | HI002839.1 | GM989728.1 | HC051653.1 | GN030859.1 | HV502504.1 | DL018353.1 | GM651751.1 | CS603027.1 |
| BD226718.1 | HI002801.1 | GM969588.1 | HC051201.1 | GN030827.1 | HV502472.1 | DL018321.1 | GM651719.1 | CS608333.1 |
| BD225483.1 | HI002742.1 | GM969397.1 | HC050010.1 | GN030795.1 | HV497324.1 | DL018289.1 | GM651687.1 | CS607040.1 |
| BD224582.1 | HI002697.1 | GM969933.1 | HC045507.1 | GN031162.1 | HV492683.1 | DL018257.1 | DL041658.1 | CS606950.1 |
| BD223802.1 | HI002657.1 | GN007496.1 | HC045475.1 | GM657985.1 | HV505351.1 | DL018225.1 | DL041626.1 | CS606868.1 |
| BD222642.1 | HI002622.1 | GM984679.1 | HC045443.1 | GM657953.1 | HV505226.1 | DL013823.1 | DL041594.1 | CS606815.1 |
| BD211176.1 | HI002585.1 | FB986394.1 | HC045411.1 | GM650761.1 | HV494740.1 | DL013791.1 | DL041562.1 | DD357907.1 |
| BD206113.1 | HI002522.1 | FB985969.1 | HC045379.1 | GM650729.1 | HV505008.1 | DL013759.1 | DL041530.1 | DD357798.1 |
| DD283529.1 | HI001477.1 | GM968450.1 | HC045347.1 | GM650697.1 | HV502193.1 | DL013727.1 | DL041498.1 | DD357589.1 |
| DD283241.1 | HI004475.1 | GM970571.1 | HC045315.1 | GM643705.1 | HV453666.1 | DL013695.1 | DL037675.1 | DD357226.1 |
| DD282375.1 | HI004411.1 | FB736148.1 | HC045283.1 | GM643673.1 | HV450087.1 | DL013663.1 | DL037643.1 | DD355836.1 |
| DD288474.1 | FW304474.1 | DL465753.1 | HC049375.1 | GM643633.1 | HV450046.1 | DL000546.1 | DL037611.1 | A18666.1 |
| DD281290.1 | FW310244.1 | DL477844.1 | HC047463.1 | GM636644.1 | HV449920.1 | DJ493828.1 | DL037579.1 | A09634.1 |
| DD286566.1 | FW331771.1 | DL464555.1 | HC047399.1 | GM636612.1 | HV453037.1 | DJ492415.1 | DL037547.1 | CS444540.1 |
| CS353465.1 | FW309552.1 | DL470946.1 | HC047367.1 | GM636580.1 | HV451132.1 | DL008509.1 | DL037515.1 | CS446153.1 |
| CS353173.1 | FW308786.1 | DL483302.1 | HC047335.1 | GM636548.1 | HV448204.1 | DL003039.1 | DL033312.1 | CS442966.1 |
| CS352514.1 | FW332167.1 | DL463075.1 | HC047303.1 | GM636516.1 | HV436965.1 | DL002940.1 | DL033280.1 | CS438913.1 |
| CS352393.1 | HC769895.1 | DL462981.1 | HC047271.1 | GM636484.1 | HV436930.1 | DJ491596.1 | DL033248.1 | CS436013.1 |
| CS283955.1 | HC767409.1 | DL469821.1 | HC047047.1 | GM632076.1 | HV436862.1 | DL002529.1 | DL033216.1 | CS434848.1 |
| CS283850.1 | HC757396.1 | DL481892.1 | HC047015.1 | GM623113.1 | HV444632.1 | DJ491068.1 | DL033184.1 | DD353772.1 |
| CS276869.1 | HC757146.1 | DL481633.1 | HC046983.1 | HB837700.1 | HV444348.1 | DJ446830.1 | DL033152.1 | DD347206.1 |
| CS272541.1 | HC742876.1 | DL460981.1 | HC046951.1 | HB845638.1 | HV444113.1 | DJ446831.1 | DL033120.1 | CS426810.1 |
| CS274692.1 | HC755774.1 | GM832175.1 | HC046919.1 | HB845405.1 | HV438123.1 | DJ474319.1 | DL029112.1 | CS425051.1 |
| CS265608.1 | HC754692.1 | GM712170.1 | HC040758.1 | HB837121.1 | HV443179.1 | DJ442347.1 | DL029080.1 | CS424094.1 |
| CS254302.1 | HC732141.1 | GM831170.1 | HC046859.1 | HB813979.1 | HV443091.1 | DJ437359.1 | DL029048.1 | CS423172.1 |
| FU761101.1 | HC471768.1 | GM830958.1 | HC046827.1 | HB845190.1 | HV437453.1 | DJ437326.1 | DL029016.1 | CS422585.1 |
| FU758387.1 | HC471694.1 | FB509326.1 | HC046795.1 | HB844932.1 | HV437311.1 | DJ437278.1 | DL028984.1 | CS417738.1 |
| FU757778.1 | HC471696.1 | FB509294.1 | HC046763.1 | HB844479.1 | HV349089.1 | DJ437245.1 | DL028952.1 | CS411019.1 |
| FU757621.1 | HC471640.1 | FB509230.1 | HC046731.1 | HB843791.1 | HV339052.1 | DJ436920.1 | DL024978.1 | CS410932.1 |
| FU757236.1 | HC471608.1 | FB509215.1 | HC046699.1 | HB836302.1 | HV344084.1 | DJ436670.1 | DL021766.1 | CS410881.1 |
| FU756565.1 | HC729053.1 | FB509183.1 | HC046682.1 | HB843113.1 | FW505138.1 | DJ436511.1 | DL021734.1 | CS414821.1 |
| HC358395.1 | HC728982.1 | FB509151.1 | HC046650.1 | HB835663.1 | FW508123.1 | DJ431640.1 | DL021702.1 | DD323522.1 |
| HC358207.1 | HC689132.1 | FB509113.1 | HC046618.1 | HB819129.1 | FW508065.1 | CS721647.1 | DL021670.1 | DD327012.1 |
| HC358076.1 | HC688432.1 | DL112416.1 | HC046586.1 | HB835174.1 | FW562404.1 | CS686958.1 | DL021638.1 | DD328868.1 |

| | | | | | | | | |
|---|---|---|---|---|---|---|---|---|
| HC325099.1 | HC490832.1 | DL112384.1 | HC046522.1 | HB842572.1 | FW556831.1 | CS686862.1 | DL021606.1 | CS398396.1 |
| HC319587.1 | HC490800.1 | DL112352.1 | HC046490.1 | HB806723.1 | FW553188.1 | CS680831.1 | DL017400.1 | CS389299.1 |
| HC318833.1 | HC490768.1 | DL112320.1 | HC046458.1 | HB842030.1 | FW553127.1 | CS677747.1 | DL017368.1 | CS389257.1 |
| HC316593.1 | HC490736.1 | DL112288.1 | HC046426.1 | HB841729.1 | FW563879.1 | DJ008409.1 | DL017336.1 | CS389211.1 |
| HC307888.1 | HC490704.1 | DL143737.1 | HC046394.1 | HB841482.1 | FW566114.1 | DJ008313.1 | DL012979.1 | CS389172.1 |
| HC307813.1 | HC490672.1 | DL107274.1 | HC046362.1 | HB806585.1 | FW559427.1 | DJ011747.1 | DL012947.1 | CS399165.1 |
| HC307392.1 | HC490543.1 | DL107242.1 | HC046330.1 | HB840883.1 | FW506317.1 | DJ004240.1 | DL014918.1 | BD453637.1 |
| HC306806.1 | HC045434.1 | DL107210.1 | HC046047.1 | HB833628.1 | HI934024.1 | DJ010517.1 | DL014886.1 | BD453605.1 |
| HB866535.1 | HC045402.1 | DL107114.1 | HC046015.1 | HB648714.1 | HI933688.1 | HW155756.1 | DL010268.1 | BD493382.1 |
| HB866207.1 | HC045370.1 | DL102501.1 | HC046287.1 | HB645682.1 | HI930649.1 | HW150107.1 | DL010236.1 | BD453555.1 |
| HB999686.1 | HC045338.1 | DL102469.1 | HC046255.1 | HB850786.1 | HI949807.1 | HW150043.1 | DL010204.1 | BD453523.1 |
| HB976836.1 | HC045274.1 | DL102437.1 | HC046159.1 | DM038363.1 | HI929257.1 | HW144826.1 | DL034669.1 | BD453491.1 |
| HB976630.1 | HC049366.1 | DL102405.1 | HC046127.1 | DM015978.1 | HI919634.1 | HW155204.1 | DL034637.1 | BD453459.1 |
| DM189907.1 | HC047454.1 | DL022209.1 | HC046095.1 | DM027074.1 | HI918272.1 | HW159073.1 | DL030385.1 | BD453427.1 |
| DM194705.1 | HC047422.1 | DL025756.1 | HC045881.1 | GN045760.1 | HI657230.1 | HW158785.1 | DL030345.1 | BD453395.1 |
| DM203067.1 | HC047390.1 | DL025724.1 | HC045849.1 | GN044281.1 | HI656341.1 | HW154649.1 | DL026526.1 | BD453250.1 |
| HB840059.1 | HC047358.1 | DL025692.1 | HC045817.1 | GN041781.1 | HI654400.1 | HW154482.1 | DL026528.1 | BD453218.1 |
| HB847585.1 | HC047326.1 | DL025596.1 | HC045785.1 | GN034787.1 | FW421370.1 | HW154450.1 | DL026472.1 | BD453147.1 |
| HB847212.1 | HC047294.1 | DL025564.1 | HC045753.1 | GN030746.1 | FW498196.1 | HW147484.1 | DL026376.1 | BD493661.1 |
| HB838863.1 | HC047262.1 | DL029706.1 | DM202497.1 | GN030714.1 | FW496132.1 | HW154211.1 | DL022564.1 | BD450242.1 |
| HB805441.1 | HC047038.1 | DL029674.1 | DM193638.1 | GN030682.1 | FW497435.1 | HW146919.1 | DL022532.1 | BD408022.1 |
| HB847065.1 | HC047006.1 | DL025537.1 | DM193399.1 | GN030650.1 | FW503885.1 | HW153766.1 | DL022436.1 | BD429476.1 |
| HB846664.1 | HC046974.1 | DL025505.1 | DM191866.1 | GN030586.1 | FW499107.1 | HW145784.1 | DL022404.1 | BD407579.1 |
| HB838166.1 | HC046942.1 | DL040392.1 | DM190446.1 | GN030554.1 | FW504941.1 | HW145519.1 | DL014651.1 | BD428457.1 |
| HB846232.1 | HC046910.1 | DL040360.1 | DM189860.1 | GN030522.1 | FW504735.1 | HW145139.1 | DL010144.1 | BD460858.1 |
| HB809980.1 | HC046882.1 | DL040328.1 | GM654408.1 | GN030490.1 | FW503678.1 | HW153364.1 | DL010112.1 | BD406390.1 |
| HB837908.1 | HC046850.1 | DL036576.1 | GM654376.1 | GN030458.1 | FW500470.1 | HW153266.1 | DL010080.1 | BD395068.1 |
| HB837554.1 | HC046818.1 | DL036544.1 | GM647383.1 | GN030435.1 | HI213014.1 | HW157346.1 | DL010048.1 | BD404623.1 |
| HB845510.1 | HC046754.1 | DL032309.1 | GM647351.1 | GN030330.1 | HI212974.1 | HW152009.1 | DL010016.1 | BD453370.1 |
| HB845283.1 | HC046722.1 | DL032277.1 | GM647319.1 | GN030266.1 | HI547163.1 | HW061714.1 | DL019017.1 | BD453334.1 |
| HB844709.1 | HC046690.1 | DL032245.1 | GM626363.1 | GN030234.1 | HI212469.1 | HW058829.1 | DL014614.1 | GN359703.1 |
| HB844350.1 | HC046641.1 | DL032213.1 | GM626331.1 | GN030202.1 | HI546326.1 | HW065307.1 | DL014582.1 | GN359651.1 |
| HB836746.1 | HC046609.1 | DL032181.1 | GM752754.1 | GN030074.1 | HI546294.1 | HW065274.1 | DL014518.1 | GN359587.1 |
| HB843999.1 | HC046577.1 | DL032149.1 | GM660356.1 | GN029946.1 | HI587348.1 | HW065209.1 | DL014486.1 | GN359555.1 |
| HB836118.1 | HC046545.1 | DL048419.1 | GM653169.1 | GN029914.1 | HI210972.1 | HW065099.1 | DL014454.1 | GN359391.1 |
| HB843255.1 | HC046513.1 | DL048387.1 | GM653137.1 | GN029848.1 | HI210940.1 | HW058433.1 | DL009996.1 | GN359295.1 |
| HB835318.1 | HC046481.1 | DL048355.1 | GM653105.1 | GN013377.1 | HI570416.1 | HW064883.1 | DL009964.1 | GN350116.1 |
| HB842845.1 | HC046449.1 | DL048323.1 | GM653073.1 | GN010220.1 | HI569853.1 | HW064843.1 | DL009932.1 | GN365385.1 |
| HB842247.1 | HC046385.1 | DL044437.1 | GM653041.1 | GN033550.1 | HI565934.1 | HW064811.1 | DL009900.1 | GN359222.1 |
| HB841885.1 | HC046353.1 | DL044405.1 | GM653009.1 | GN033518.1 | HI565787.1 | HW061079.1 | DL009868.1 | GN359158.1 |
| HB841664.1 | HC046070.1 | DL044373.1 | GM652977.1 | GN033486.1 | HI473065.1 | HW064806.1 | DL009836.1 | GN362970.1 |
| HB841195.1 | HC046038.1 | DL044341.1 | GM645946.1 | GN033454.1 | HI472913.1 | HW056503.1 | DL009804.1 | GN346504.1 |
| HB841095.1 | HC046000.1 | DL044309.1 | GM645914.1 | GN033422.1 | HI209390.1 | HW064739.1 | DL009772.1 | GN337635.1 |
| HB840561.1 | HC045968.1 | DL044277.1 | GM645882.1 | GN033390.1 | HI072073.1 | HW064707.1 | DL009740.1 | GN336558.1 |
| HB647013.1 | HC045936.1 | DL040101.1 | GM645850.1 | GN033358.1 | HI004257.1 | HW064666.1 | DL009708.1 | GN334004.1 |
| HB645729.1 | HC045904.1 | DL032108.1 | GM645818.1 | GN033326.1 | HI002282.1 | HW060528.1 | DL009676.1 | GN112641.1 |
| HB645697.1 | HC045872.1 | DL032076.1 | GM639015.1 | GN033294.1 | HI002246.1 | HW056222.1 | DL009644.1 | DM065119.1 |

| | | | | | | | | |
|---|---|---|---|---|---|---|---|---|
| GM741823.1 | HC045840.1 | DL032044.1 | GM638983.1 | GN033262.1 | HV514051.1 | HW062353.1 | DL009612.1 | DM064154.1 |
| GM658305.1 | HC045808.1 | DL032012.1 | GM638951.1 | GN033230.1 | HV510390.1 | HW062321.1 | DL009580.1 | DM077412.1 |
| GM658273.1 | HC045776.1 | DL031948.1 | GM638887.1 | GN033166.1 | HV510358.1 | HW062224.1 | DL009548.1 | FB507303.1 |
| GM658241.1 | HC045744.1 | DL028139.1 | GM638855.1 | GN033134.1 | HV510056.1 | HW042076.1 | DL009516.1 | GM622124.1 |
| GM658209.1 | HC045712.1 | DL028107.1 | GM626176.1 | GN033102.1 | HV513364.1 | HW042044.1 | DL009484.1 | GM621836.1 |
| GM047084.1 | CS047281.1 | DL028075.1 | GM626144.1 | GN033070.1 | HV513332.1 | HW043596.1 | DL009452.1 | GM641381.1 |
| GM654811.1 | CS038929.1 | DL028043.1 | GM660279.1 | GN033038.1 | HV509289.1 | HW042000.1 | DL009420.1 | GM641349.1 |
| GM654779.1 | CS036970.1 | DL028011.1 | GM660247.1 | GN033006.1 | HV508627.1 | HW041968.1 | DL030145.1 | GM629062.1 |
| GM646475.1 | AX241969.1 | DL027979.1 | GM660215.1 | GN032910.1 | HV508595.1 | HW041936.1 | DL026272.1 | GM629030.1 |
| GM646443.1 | AX241937.1 | DL036393.1 | GM660183.1 | GN032878.1 | HV508563.1 | HW041896.1 | DL026240.1 | GM628998.1 |
| GM646411.1 | AX241905.1 | DL036361.1 | GM660151.1 | GN032846.1 | HV508531.1 | HW041838.1 | DL026208.1 | GM047083.1 |
| GM626869.1 | AX241873.1 | DL036329.1 | GM660328.1 | GN032814.1 | HV508471.1 | HW049390.1 | DL026176.1 | GM654810.1 |
| GM626837.1 | AX241841.1 | DL036297.1 | GM652945.1 | GN032782.1 | HV512414.1 | HW049359.1 | DL018986.1 | GM639411.1 |
| GM626805.1 | AX241809.1 | DL044244.1 | GM652913.1 | GN032751.1 | HV512273.1 | HW049327.1 | DL018954.1 | GM639379.1 |
| GM639412.1 | AX241777.1 | DL044212.1 | GM652881.1 | GN032719.1 | HV512241.1 | HW049295.1 | DL018922.1 | GM639347.1 |
| GM639380.1 | AX241745.1 | DL044180.1 | GM652849.1 | GN032687.1 | HV512177.1 | HW049263.1 | DL018890.1 | GM639080.1 |
| GM639348.1 | AX241681.1 | DL044116.1 | GM652817.1 | GN032655.1 | HV492313.1 | HW043297.1 | CS670995.1 | CS695795.1 |
| GM949530.1 | AX241649.1 | DL044084.1 | GM652784.1 | GN032622.1 | HV492148.1 | HW041802.1 | CS672570.1 | CS695699.1 |
| GM887858.1 | AX241585.1 | DL048221.1 | GM645786.1 | GN032590.1 | HV504953.1 | HW041770.1 | DD462502.1 | CS695667.1 |
| GM869486.1 | AX241553.1 | DL048189.1 | GM645754.1 | GN032558.1 | HV504921.1 | HW043132.1 | CS647652.1 | DL176619.1 |
| GM869166.1 | AX241521.1 | DL048157.1 | GM645722.1 | GN032527.1 | HV504889.1 | HW041547.1 | CS646653.1 | DL176470.1 |
| GM773640.1 | AX241489.1 | DL048125.1 | GM645690.1 | GN032495.1 | HV504857.1 | HW048301.1 | CS646200.1 | DL176396.1 |
| GM879367.1 | AX235762.1 | DL048093.1 | GM638827.1 | GN032463.1 | HV504825.1 | HW053846.1 | HW061809.1 | DL175301.1 |
| GM879306.1 | AX223894.1 | DL048061.1 | GM638763.1 | GN032431.1 | HV507455.1 | HV985974.1 | HW065238.1 | DL181415.1 |
| FB722587.1 | AX212311.1 | DL048030.1 | GM626120.1 | GN032399.1 | HV504776.1 | HV985942.1 | HW058495.1 | DL176737.1 |
| GM721242.1 | AX207224.1 | DL047966.1 | GM626056.1 | GN032367.1 | HV504712.1 | HV985910.1 | HW064839.1 | DL163179.1 |
| FB742764.1 | AX205117.1 | DL047934.1 | GM626024.1 | GN032334.1 | HV504680.1 | HV985865.1 | HW057646.1 | FB344351.1 |
| GM600752.1 | AX203101.1 | DL047902.1 | GM625992.1 | GN032302.1 | HV504648.1 | HV974797.1 | HW056499.1 | FB343370.1 |
| FB721286.1 | AX194354.1 | DL047870.1 | GM625960.1 | GN032270.1 | HV504616.1 | HV752620.1 | HW064661.1 | DL101212.1 |
| FB717569.1 | AX175107.1 | DL040071.1 | FB776241.1 | GN032238.1 | HV504593.1 | HV755130.1 | HW062550.1 | DL115122.1 |
| FB706360.1 | AX172868.1 | DL040039.1 | FB775656.1 | GN032206.1 | HV504561.1 | HV749719.1 | HW062406.1 | DL110058.1 |
| GM868902.1 | AX172463.1 | DL040007.1 | FB774896.1 | GM649671.1 | HV504529.1 | HV760141.1 | HW060575.1 | DL110026.1 |
| GM061218.1 | AX167031.1 | DL039975.1 | FB766237.1 | GM649639.1 | HV504497.1 | HV752232.1 | HW060524.1 | DL109994.1 |
| DL258288.1 | AX166278.1 | DL039943.1 | FB764711.1 | GM649607.1 | HV504433.1 | HV748899.1 | HW060475.1 | DL109962.1 |
| DL256295.1 | AY035210.1 | DL039911.1 | FB764673.1 | GM642703.1 | HV504209.1 | HV748441.1 | HW056317.1 | DL131406.1 |
| DL241164.1 | AX148744.1 | DL027948.1 | FB744716.1 | GM642671.1 | HV455593.1 | HV747614.1 | HW056218.1 | DL109748.1 |
| DL240249.1 | AX146310.1 | DL047826.1 | FB743935.1 | GM642639.1 | HV504173.1 | HV747476.1 | HW062381.1 | DL109716.1 |
| FB748851.1 | AX145723.1 | DL047794.1 | FB743903.1 | GM635329.1 | HV504141.1 | HV753569.1 | HW062317.1 | DL109684.1 |
| DL236210.1 | AX145659.1 | DL047762.1 | FB743871.1 | GM635297.1 | HV504109.1 | HV753487.1 | HW042072.1 | DL127366.1 |
| DL233428.1 | AX145595.1 | DL047730.1 | FB743839.1 | DL115756.1 | HV504077.1 | HV743436.1 | HW042040.1 | DL114814.1 |
| DL233395.1 | AX145563.1 | DL047698.1 | FB743785.1 | DL115724.1 | HV504045.1 | HV743312.1 | HW043570.1 | DL114782.1 |
| DL231810.1 | AX145531.1 | DL047666.1 | FB761598.1 | DL115692.1 | HV504013.1 | HV743274.1 | HW041996.1 | DL114750.1 |
| DL020830.1 | AX145499.1 | DL039652.1 | FB761487.1 | DL115660.1 | HV503981.1 | HV743235.1 | HW041964.1 | DL114718.1 |
| DL047592.1 | AX145435.1 | DL039620.1 | GM877305.1 | DL111234.1 | HV503917.1 | HV704949.1 | HW041932.1 | DL114686.1 |
| HV502638.1 | AX145403.1 | DL039588.1 | GM036118.1 | DL111202.1 | HV503853.1 | HV704420.1 | HW041834.1 | DL114654.1 |
| HV502542.1 | AX145370.1 | DL039524.1 | GM035745.1 | DL111170.1 | HV503821.1 | HV704265.1 | HW049422.1 | DL127185.1 |
| HV502510.1 | AX145338.1 | DL047318.1 | GM035293.1 | DL111138.1 | HV501014.1 | HV704200.1 | HW049386.1 | DL141446.1 |

| | | | | | | | | |
|---|---|---|---|---|---|---|---|---|
| HV502446.1 | AX145306.1 | DL047286.1 | GM952833.1 | DL111106.1 | HV116990.1 | HV744596.1 | HW049355.1 | DJ436702.1 |
| HV452373.1 | AX145274.1 | DL043464.1 | GM951786.1 | DL111074.1 | HV112419.1 | HV703530.1 | HW049291.1 | DJ445550.1 |
| HV453672.1 | AX145242.1 | DL043400.1 | GM890251.1 | DL106174.1 | HV038601.1 | AY659346.1 | HW049259.1 | DJ444636.1 |
| HV449930.1 | AX145210.1 | DL043368.1 | GM889325.1 | DL101401.1 | HV038525.1 | AY659314.1 | HW041798.1 | DJ433802.1 |
| HV453101.1 | AX145178.1 | DL043336.1 | GM828394.1 | DL101337.1 | HV182329.1 | AY659282.1 | HW041766.1 | DJ438337.1 |
| HV449381.1 | AX145146.1 | DL043272.1 | GM888714.1 | DL106067.1 | HV182297.1 | AY659250.1 | HW054004.1 | DJ438302.1 |
| HV451138.1 | AX145114.1 | DL039455.1 | GM008870.1 | DL106035.1 | HV202605.1 | AY659218.1 | HW048282.1 | DJ438250.1 |
| HV444126.1 | AX145082.1 | DL039423.1 | GM888516.1 | DL106003.1 | HV187837.1 | AY659186.1 | HW053881.1 | DJ401282.1 |
| HV437728.1 | AX145050.1 | DL039391.1 | GM006122.1 | DL105971.1 | HV187551.1 | AY659154.1 | HV553661.1 | DJ400818.1 |
| HV437506.1 | AX145018.1 | DL039359.1 | GM005892.1 | DL105907.1 | HV222978.1 | AY659122.1 | HV551420.1 | DJ400786.1 |
| HV341335.1 | AX144986.1 | DL039327.1 | FB984706.1 | DL101198.1 | HV234446.1 | AY659090.1 | HV555259.1 | DJ417451.1 |
| FW496534.1 | AX144954.1 | DL039295.1 | GM003580.1 | DL101166.1 | HV234233.1 | AY659058.1 | HV550370.1 | DJ387343.1 |
| HI654008.1 | AX144921.1 | DL035671.1 | FB983216.1 | DL101134.1 | HV302186.1 | AY659026.1 | AY659406.1 | DJ380839.1 |
| HI653909.1 | AX144889.1 | DL035639.1 | FB983164.1 | DL096720.1 | HV312981.1 | AY658994.1 | AY659374.1 | DJ389577.1 |
| HI646937.1 | AX144857.1 | DL035607.1 | GM681699.1 | DL096688.1 | HV302126.1 | DD420078.1 | AY659342.1 | CS464516.1 |
| AY145509.1 | AX144825.1 | DL035575.1 | GM865385.1 | DL096656.1 | HV037887.1 | DD432676.1 | AY659310.1 | DD400174.1 |
| HH779728.1 | HW069874.1 | DL035543.1 | GM881842.1 | DL096624.1 | HV037755.1 | DD433781.1 | AY659278.1 | DD400141.1 |
| HH777932.1 | HW084938.1 | DL035511.1 | GM618788.1 | DL096592.1 | HV037710.1 | DD420776.1 | AY659246.1 | DD381261.1 |
| HH819339.1 | HW089034.1 | DL031507.1 | FB725961.1 | DL096560.1 | HV037678.1 | CS541726.1 | AY659214.1 | CS459123.1 |
| HI580531.1 | HW097687.1 | DL091872.1 | GM840795.1 | DL094732.1 | HV031615.1 | CS543085.1 | AY659182.1 | DD367718.1 |
| HI209396.1 | HW065511.1 | DL091840.1 | FB711263.1 | DL094700.1 | FZ435972.1 | CS542563.1 | AY659150.1 | DD361272.1 |
| HI006456.1 | HV956203.1 | DL091808.1 | GM967130.1 | DL094668.1 | FZ435884.1 | CS538098.1 | AY659118.1 | DD367532.1 |
| HI004333.1 | HV951103.1 | DL091776.1 | GM840468.1 | DL090957.1 | FZ425947.1 | CS537448.1 | AY659086.1 | DD368134.1 |
| HI002288.1 | HV957483.1 | DL102897.1 | GM041233.1 | DL090829.1 | FZ429429.1 | CS537183.1 | AY659054.1 | DD361306.1 |
| HI002255.1 | HV953057.1 | DL102865.1 | FB709615.1 | DL086989.1 | FZ429320.1 | CS502916.1 | AY659022.1 | CS456717.1 |
| HI002202.1 | HV946241.1 | DL102801.1 | CS728589.1 | DL086957.1 | FZ429179.1 | CS502714.1 | AY658990.1 | CS453769.1 |
| HI002158.1 | HV943092.1 | DL102769.1 | CS727587.1 | DL086925.1 | FZ423871.1 | CS502646.1 | AY658958.1 | CS450638.1 |
| HI002122.1 | HV779875.1 | DL102737.1 | CS727433.1 | DL086893.1 | FZ432344.1 | CS502336.1 | AY658926.1 | DD359998.1 |
| HI000471.1 | HV774715.1 | DL107517.1 | FB708978.1 | DL086861.1 | FZ423343.1 | DD355755.1 | AY658894.1 | DD357937.1 |
| HI000417.1 | HV773778.1 | DL107489.1 | GM719137.1 | DL086829.1 | FZ422772.1 | CS449843.1 | AY658862.1 | DD357901.1 |
| HI000375.1 | HW302532.1 | DL107457.1 | GM014331.1 | DL086765.1 | FZ422538.1 | A06663.1 | AY658830.1 | A05961.1 |
| HI203334.1 | HW302349.1 | DL107393.1 | FW563121.1 | DL086733.1 | FZ419481.1 | A14567.1 | AY658798.1 | A04087.1 |
| HI553271.1 | HW295573.1 | DL102588.1 | FW557304.1 | DL086701.1 | FZ415922.1 | A02587.1 | AY658766.1 | CS446150.1 |
| HI521585.1 | HW302090.1 | DL102556.1 | FW557014.1 | CS368396.1 | FZ421458.1 | A06033.1 | AY658702.1 | CS438910.1 |
| HI583852.1 | HW295046.1 | DL102524.1 | FW562769.1 | CS368332.1 | FZ421396.1 | CS438998.1 | AY658670.1 | DD038501.1 |
| HI577093.1 | HW285863.1 | DL098014.1 | FW562696.1 | CS368140.1 | HI581366.1 | CS435226.1 | AY658638.1 | DD033981.1 |
| HI520495.1 | HW306959.1 | DL097982.1 | FW565447.1 | CS367948.1 | HI573658.1 | CS435194.1 | AY658606.1 | GN032506.1 |
| HI002087.1 | HW294136.1 | DL097950.1 | HI378135.1 | DL075840.1 | HI573565.1 | DD333027.1 | AY658574.1 | GN032474.1 |
| HI002035.1 | HW293097.1 | DL112640.1 | HI378103.1 | DL075808.1 | HI566222.1 | DD332704.1 | AY658542.1 | GN032442.1 |
| HI001952.1 | HW292745.1 | DL112608.1 | HI377137.1 | DL075763.1 | HI553830.1 | DD331845.1 | AY658510.1 | GN032410.1 |
| HI000284.1 | HW291258.1 | DL112576.1 | HI376679.1 | DL079748.1 | HI594009.1 | CS426727.1 | AY658478.1 | GN032378.1 |
| HI000244.1 | HW291161.1 | DL112544.1 | HI375894.1 | DL079089.1 | HI636973.1 | CS433065.1 | AY658446.1 | GN032313.1 |
| HI000200.1 | HW291119.1 | DL112512.1 | HI375020.1 | DL079049.1 | HI636936.1 | CS418629.1 | AY658414.1 | GN032281.1 |
| HI000144.1 | HW291064.1 | DL112480.1 | HI372273.1 | DL078965.1 | HI001453.1 | CS419546.1 | AY658382.1 | GN032249.1 |
| HI000112.1 | HW291032.1 | DL097827.1 | HI369901.1 | DL078933.1 | HI001398.1 | CS418738.1 | AY658350.1 | GN032217.1 |
| HI471354.1 | HW291000.1 | DL097763.1 | HI369081.1 | DL078902.1 | HI001357.1 | CS414598.1 | AY658318.1 | GN032185.1 |
| HI551865.1 | HW290936.1 | DL095736.1 | HI369024.1 | DL073100.1 | HI001323.1 | CS417157.1 | AY658286.1 | GN032153.1 |

| | | | | | | | | |
|---|---|---|---|---|---|---|---|---|
| HI559222.1 | HW298843.1 | DL095704.1 | HI368438.1 | DL013607.1 | HI003206.1 | CS416225.1 | AY658254.1 | GN032121.1 |
| HI568866.1 | HW290905.1 | DL095672.1 | HI424141.1 | DL013575.1 | HI003087.1 | CS415772.1 | AY658222.1 | GN032089.1 |
| HI564225.1 | HW290873.1 | DL095640.1 | HI424109.1 | DL013543.1 | HI003048.1 | CS410907.1 | AY658190.1 | GN032057.1 |
| HI564106.1 | HW290105.1 | DL095608.1 | HI424077.1 | DL013511.1 | HI003007.1 | CS410692.1 | AY658158.1 | GN032025.1 |
| HI583089.1 | HW289994.1 | DL105672.1 | HI423851.1 | DL013479.1 | HI002908.1 | CS414850.1 | AY658126.1 | GN031993.1 |
| HI544155.1 | HV750990.1 | DL105640.1 | HI423819.1 | DL013447.1 | HI001271.1 | CS414781.1 | AY658094.1 | GN031961.1 |
| HI586460.1 | HV747496.1 | DL105608.1 | HI423787.1 | DL013415.1 | DD370032.1 | DD329744.1 | AY658062.1 | GN031929.1 |
| HI000019.1 | HV753749.1 | DL100899.1 | HI423563.1 | DL013383.1 | DD361327.1 | DD326843.1 | AY658030.1 | GN031897.1 |
| FW300636.1 | HV753630.1 | DL100867.1 | HI416363.1 | DL018010.1 | CS457479.1 | DD321762.1 | AY657998.1 | GN031865.1 |
| FW298892.1 | HV753424.1 | DL100835.1 | HI416156.1 | DL013259.1 | CS457149.1 | DD325196.1 | AY657966.1 | GN031833.1 |
| FW304268.1 | HV743256.1 | DL100803.1 | HI415918.1 | DL013227.1 | CS456706.1 | CS390497.1 | AY657934.1 | GN031800.1 |
| HC491773.1 | AX085336.1 | DL100771.1 | HI415886.1 | DL013195.1 | CS454150.1 | CS398095.1 | AY657902.1 | GN031671.1 |
| HC491741.1 | AX081483.1 | DL100739.1 | HI415821.1 | DL017984.1 | CS452901.1 | CS389285.1 | AY657870.1 | GN031639.1 |
| HC473884.1 | AX078802.1 | DL096325.1 | HI415789.1 | DL017952.1 | CS451669.1 | CS389196.1 | AY657838.1 | GN031607.1 |
| FV533454.1 | AX077107.1 | DL096293.1 | HI415757.1 | DL017920.1 | CS451039.1 | CS389158.1 | AY657806.1 | GN031575.1 |
| FV531696.1 | AX076576.1 | DL096261.1 | HI415151.1 | DL017888.1 | CS450604.1 | CS401289.1 | AY657774.1 | GN031543.1 |
| FV530903.1 | AX076202.1 | DL096229.1 | HI414012.1 | DL017856.1 | CS450538.1 | CS406680.1 | AY657742.1 | GN031511.1 |
| FV522834.1 | AX068975.1 | DL096197.1 | HI413450.1 | DL017824.1 | DD353827.1 | CS406435.1 | AY657710.1 | GN031479.1 |
| FV522794.1 | AX063574.1 | DL096165.1 | HI410944.1 | DL017793.1 | DD359087.1 | CS404662.1 | AY657678.1 | GN031446.1 |
| FV522611.1 | AX061426.1 | DL120185.1 | HI516614.1 | DL017761.1 | DD357926.1 | CS196178.1 | AY657646.1 | GN031382.1 |
| AY138586.1 | AX059667.1 | DL120153.1 | HI516133.1 | DL017729.1 | DD357889.1 | CS200916.1 | AY657614.1 | GN031350.1 |
| HC453693.1 | AX052942.1 | DL120121.1 | HI516087.1 | DL017697.1 | DD357630.1 | CS226731.1 | AY657582.1 | GN031285.1 |
| FU264219.1 | AX045744.1 | DL120089.1 | HI547692.1 | DL017665.1 | DD357467.1 | CS208793.1 | AY657550.1 | GN031189.1 |
| HC306222.1 | HW311024.1 | DL120057.1 | HI284306.1 | DL017633.1 | DD355754.1 | CS207924.1 | AY657518.1 | GN031156.1 |
| HC306066.1 | HW308555.1 | DL120025.1 | HI284274.1 | DL022271.1 | CS449841.1 | CS186202.1 | AY657486.1 | GN031124.1 |
| HC302631.1 | HW307890.1 | DL094343.1 | HI571708.1 | DL022207.1 | A29484.1 | CS183877.1 | AY657454.1 | GN031092.1 |
| HC305954.1 | HW307858.1 | DL094311.1 | HI574527.1 | DL025754.1 | A14309.1 | CS183824.1 | AY657422.1 | GN031060.1 |
| HC305914.1 | HW307826.1 | DL094247.1 | HI574495.1 | DL025722.1 | A02585.1 | CS174712.1 | AY657390.1 | GN031028.1 |
| HC305834.1 | HW307794.1 | DL094215.1 | HI574463.1 | DL025690.1 | CS446135.1 | CS174033.1 | CS389191.1 | GN030996.1 |
| HC305794.1 | HW307756.1 | DL094183.1 | HI574431.1 | DL025594.1 | CS443889.1 | CS172437.1 | CS389030.1 | GN030964.1 |
| HC305754.1 | HW307724.1 | DL094151.1 | HI574399.1 | DL025562.1 | CS438993.1 | CS166518.1 | CS398652.1 | GN030933.1 |
| HC305674.1 | HW307692.1 | DL090468.1 | HI571455.1 | DL029544.1 | CS435257.1 | CS161438.1 | CS401606.1 | GN030901.1 |
| HC305634.1 | HW315875.1 | DL090436.1 | HI561762.1 | DL039133.1 | CS435225.1 | CS159617.1 | CS406672.1 | GN030869.1 |
| HC305374.1 | HW315670.1 | DL090404.1 | FW376186.1 | DL039101.1 | CS435193.1 | CS159207.1 | CS409985.1 | GN030837.1 |
| HC291546.1 | HW315585.1 | DL090372.1 | FW376281.1 | DL035477.1 | CS434494.1 | CS156087.1 | CS403497.1 | GN030805.1 |
| HC291270.1 | HW315552.1 | DL086367.1 | FW381345.1 | DL035445.1 | DD334496.1 | CS144361.1 | CS402982.1 | GN030773.1 |
| HC289328.1 | HW315400.1 | DL086335.1 | FW375494.1 | DL035413.1 | DD347355.1 | CS141532.1 | CS402176.1 | DM005048.1 |
| HC292804.1 | HW315095.1 | DL086303.1 | FW375247.1 | DL035381.1 | DD331842.1 | CS141500.1 | CS402097.1 | DM004978.1 |
| HC296880.1 | HW315063.1 | DL086271.1 | FW374984.1 | DL035349.1 | CS426724.1 | CS141155.1 | CS382134.1 | DM003479.1 |
| HC292672.1 | HW314821.1 | DL086239.1 | FW380264.1 | DL035317.1 | CS433064.1 | CS140734.1 | CS380440.1 | DJ052230.1 |
| HC295606.1 | HW069911.1 | DL086207.1 | FW379839.1 | DL031313.1 | CS423569.1 | CS136259.1 | CS382618.1 | DJ052039.1 |
| HC295464.1 | HW069871.1 | DL110322.1 | FW369306.1 | DL031249.1 | CS284621.1 | CS132062.1 | CS376592.1 | CS716028.1 |
| HC288715.1 | HW069815.1 | DL110290.1 | K02996.1 | DL031217.1 | CS283939.1 | CS123350.1 | DD308740.1 | CS713110.1 |
| HC295265.1 | HW069546.1 | DL105453.1 | HH736043.1 | DL031185.1 | CS267213.1 | CS122634.1 | DD292515.1 | CS675425.1 |
| HC292122.1 | HW084935.1 | DL105421.1 | HH735920.1 | DL031153.1 | CS252559.1 | CS122259.1 | DD291839.1 | CS810225.1 |
| HC291942.1 | HW068057.1 | DL105389.1 | HH735625.1 | FW392978.1 | CS253984.1 | CS106185.1 | DD291704.1 | CS809557.1 |
| HC299202.1 | HV959865.1 | DL105357.1 | HH733635.1 | HH936643.1 | CS250648.1 | CS106091.1 | DD291018.1 | CS790643.1 |

| | | | | | | | | |
|---|---|---|---|---|---|---|---|---|
| HC299166.1 | HV956200.1 | DL105325.1 | HD119618.1 | HH833641.1 | CS250616.1 | CS179763.1 | DD319827.1 | DJ031807.1 |
| GN032569.1 | HV951100.1 | DL124344.1 | FW359900.1 | HH833609.1 | CS250286.1 | CS179624.1 | CS376162.1 | DJ045305.1 |
| GN032538.1 | HV949973.1 | DL124312.1 | FW359755.1 | HH833577.1 | CS244966.1 | CS179193.1 | CS376001.1 | DJ031162.1 |
| HW103112.1 | HV953054.1 | DL110173.1 | FW359670.1 | HH833545.1 | CS244168.1 | CS326354.1 | CS375759.1 | DJ044965.1 |
| HW102881.1 | HV945137.1 | DL110141.1 | FW368817.1 | HH833513.1 | CS244248.1 | CS323706.1 | CS362718.1 | DJ044950.1 |
| HW102817.1 | HV943006.1 | DL110109.1 | FW368752.1 | HH833449.1 | CS244216.1 | CS323599.1 | CS378183.1 | DJ044902.1 |
| HW102752.1 | HV779763.1 | DL105176.1 | FW368391.1 | HH833417.1 | CS244184.1 | CS322991.1 | CS359743.1 | DJ027935.1 |
| HW084177.1 | HV777040.1 | DL105144.1 | FW368149.1 | HH833385.1 | CS239710.1 | DD118403.1 | CS359607.1 | CS721656.1 |
| HW062135.1 | HV819630.1 | DL105112.1 | FW363199.1 | FW381692.1 | CS237811.1 | DD091162.1 | BD269144.1 | CQ945787.1 |
| HW061865.1 | HV932714.1 | DL100698.1 | FW362968.1 | FW392842.1 | CS235774.1 | DD118109.1 | BD268122.1 | CS688518.1 |
| HW061833.1 | HV932681.1 | DL100666.1 | FW362815.1 | HH931888.1 | CS228476.1 | DD116395.1 | BD266835.1 | DJ011753.1 |
| HW062180.1 | HV802933.1 | DL100634.1 | FW362770.1 | HH822343.1 | CS235446.1 | DD089596.1 | BD264580.1 | CS244256.1 |
| HW065248.1 | HV802901.1 | DL100602.1 | FW362663.1 | HH822030.1 | CS200914.1 | DD102580.1 | BD263454.1 | CS244224.1 |
| HW065183.1 | HV818558.1 | DL100570.1 | FW349834.1 | FW379644.1 | CS226730.1 | DD102447.1 | BD262935.1 | CS244192.1 |
| HW058472.1 | HV786599.1 | DL100538.1 | FW366740.1 | FW379612.1 | CS203534.1 | DD102415.1 | BD261825.1 | CS243077.1 |
| HW064849.1 | HV784203.1 | DL119992.1 | FW361987.1 | FW379269.1 | CS186198.1 | DD052077.1 | BD251459.1 | CS237083.1 |
| HV515079.1 | HV780442.1 | DL119960.1 | FW351395.1 | HC305939.1 | CS182277.1 | DD052022.1 | BD251234.1 | CS227298.1 |
| HV535594.1 | HV780124.1 | DL119928.1 | FW351246.1 | HC305899.1 | CS183876.1 | DD057914.1 | BD250249.1 | CS203618.1 |
| HV515778.1 | HV778012.1 | DL119896.1 | FW351187.1 | HC305859.1 | CS179000.1 | DD057882.1 | BD249059.1 | CS207860.1 |
| HV515594.1 | HV777558.1 | DL119864.1 | FW350345.1 | HC305819.1 | CS174581.1 | DD057850.1 | BD247532.1 | CS189605.1 |
| HV515562.1 | HV777185.1 | DL119832.1 | FW360409.1 | HC305779.1 | CS174032.1 | DL099533.1 | BD247111.1 | CS191514.1 |
| HV515498.1 | HV775695.1 | DL115237.1 | HD122552.1 | HC305739.1 | CS203503.1 | DL093523.1 | BD245264.1 | CS177810.1 |
| HV515466.1 | HV775020.1 | DL115205.1 | HD121601.1 | HC305699.1 | CS172391.1 | DL093491.1 | BD242710.1 | CS174737.1 |
| HV515434.1 | HV764760.1 | DL013384.1 | HD084086.1 | HC305659.1 | CS166455.1 | DL093427.1 | BD242556.1 | CS176142.1 |
| HV515402.1 | HV764696.1 | DL018107.1 | FV534650.1 | HC301631.1 | CS159616.1 | DL093363.1 | BD242459.1 | CS172446.1 |
| HV515370.1 | HV764474.1 | DL144484.1 | FV534475.1 | HC301350.1 | CS159202.1 | DL126140.1 | BD241794.1 | CS172298.1 |
| HV515338.1 | HV763989.1 | DL126209.1 | FV534423.1 | HC301308.1 | BD283716.1 | DL126108.1 | BD240785.1 | CS165810.1 |
| HV515306.1 | HV515284.1 | DL126177.1 | FV534185.1 | HC294086.1 | BD289755.1 | DL126076.1 | BD238479.1 | CS159813.1 |
| HV515274.1 | HV515252.1 | DL122961.1 | FV534056.1 | HC289414.1 | BD287767.1 | DL126044.1 | BD238037.1 | CS159781.1 |
| HV515242.1 | HV511482.1 | DL122929.1 | HC465488.1 | HC289348.1 | BD287344.1 | DL126012.1 | BD237861.1 | CS159592.1 |
| HV509845.1 | HV507917.1 | DL122897.1 | HC460437.1 | HC289316.1 | BD278974.1 | DL125980.1 | BD235647.1 | CS159223.1 |
| HV509296.1 | HV510237.1 | DL122865.1 | AY192358.1 | HC289085.1 | BD274656.1 | DL122708.1 | BD235370.1 | CS148788.1 |
| HV508634.1 | HV508679.1 | DL122833.1 | AF506026.1 | HC296936.1 | BD273679.1 | DL122756.1 | CS018547.1 | CS141600.1 |
| HV508570.1 | HV508644.1 | DL122801.1 | AF312392.1 | HC296019.1 | BD273176.1 | DL122700.1 | CS018264.1 | CS141539.1 |
| HV508538.1 | HV508612.1 | DL122769.1 | HC456291.1 | HC288907.1 | BD271852.1 | DL122668.1 | CS016831.1 | CS141507.1 |
| HV508506.1 | HV508580.1 | DL118515.1 | HC453685.1 | HC288870.1 | BD271047.1 | DL118382.1 | CQ990436.1 | CS141287.1 |
| HV508478.1 | HV508548.1 | DL114023.1 | HC452242.1 | HC295568.1 | BD270853.1 | DL118350.1 | CQ986633.1 | CS134728.1 |
| HV508404.1 | HV508516.1 | DL113991.1 | HC727400.1 | HC295440.1 | BD270120.1 | DL118318.1 | CQ986599.1 | CS138046.1 |
| HV512280.1 | HV508488.1 | DL113959.1 | HC688447.1 | HC292374.1 | DD230059.1 | DL118286.1 | CQ986567.1 | CS133016.1 |
| HV512248.1 | HV512290.1 | DL113927.1 | HC490975.1 | HC295121.1 | DD228597.1 | DL118254.1 | CQ986533.1 | CS123402.1 |
| HV512216.1 | HV512258.1 | DL113895.1 | HC490943.1 | HC294936.1 | DD227389.1 | DL118222.1 | CQ983188.1 | CS122816.1 |
| HV512184.1 | HV512226.1 | DL113863.1 | HC490911.1 | HC291810.1 | DD226697.1 | DL113826.1 | CQ983072.1 | CS122288.1 |
