# Supplementary material for: A Sensitivity Study for Interpreting Nucleic Acid Sequence Screening Regulatory and Guidance Documentation: Toward a Foundational Synthetic Nucleic Acid Sequence Screening Framework
Source: Appl Biosaf. 2024 Sep 18;29(3):150–8. doi: 10.1089/apb.2023.0026 (PMC11447129; doi:10.1089/apb.2023.0026)
Supplement: Supplementary Data S2 [file apb.2023.0026_suppl_datas2.pdf]

Supplementary Material: Additional UltraSEQ Sequence of Concern (SoC) Metadata corresponding to Figure 2 are contained below.

Region: 1 to 170 Accession P01213:

- **Definition**: Proenkephalin-B
- **SoC name**: Dynorphin A(1-17)
- **Agent**: Homo sapiens
- **Function**: Mimics the effects of opioid drugs. The effects include decreased pain perception and response to stress. Dynorphin is noted as being 700 times more potent than Leu-enkephalins.
    - **Citation**: IUPHAR/BPS Guide to PHARMACOLOGY. International Union of Basic and Clinical Pharmacology. Retrieved 20 October 2017. Principal endogenous agonists at κ receptor
    - **Flag**: Protein Bioregulator
- **Rationale for inclusion**: Leu-enkephalins is a peptide that mimics the effects of the scheduled 1 drug (opioids).
- **Legitimate use case**: Dynorphin could be used as an opioid supplement to reduce pain perception.
    - **Citation**: IUPHAR/BPS Guide to PHARMACOLOGY. International Union of Basic and Clinical Pharmacology. Retrieved 20 October 2017. Principal endogenous agonists at κ receptor
- **Bioregulator**:
    - **System**: Central Nervous System
    - **Effect**: Opiate like activity
    - **Citations**: IUPHAR/BPS Guide to PHARMACOLOGY. International Union of Basic and Clinical Pharmacology. Retrieved 20 October 2017. Principal endogenous agonists at κ receptor

Region: 170 to 314 Accession P0DPI0

- **Definition:** Botulinum neurotoxin type A (BoNT/A)
- **SoC name:** Botulinum neurotoxin A heavy chain from Clostridium botulinum
- **Agent:** Clostridium botulinum
- **Controlled toxin:** AGL, CCL, EU, FSAP
- **Toxin:** Toxin Chain 2 Subunit A
- **Function:** Botulinum neurotoxin A heavy chain is responsible for host epithelial cell transcytosis, host nerve cell targeting and translocation of botulinum neurotoxin A light chain (LC) into host cytosol. Composed of 3 subdomains; the translocation domain (TD), and N-terminus and C-terminus of the receptor-binding domain (RBD) (PubMed:19096517). The RBD is responsible for binding to host epithelial cells and transcytosis across them; this uses different receptors than those on nerve cells (PubMed:21106906). RBD is also responsible for adherence of toxin to host nerve cell surface; HC alone prevents uptake of whole toxin by neural cells, and delays paralysis

onset by 75% (PubMed:6694738, PubMed:10413679). Isolated RBD also delays paralysis onset (PubMed:21106906). The N-terminus of the RBD binds to phosphatidylinositol, which might play a role in membrane-binding (PubMed:19161982). Binds to host protein receptor synaptic vesicle glycoproteins SV2A, SV2B and SV2C via lumenal loop 4 (PubMed:16545378, PubMed:6370252, PubMed:27294781, PubMed:24240280, PubMed:19650874, PubMed:27313224). Binding can be inhibited by protein fragments from either the HC or SV2C (PubMed:24240280). Isolated HC significantly decreases uptake and toxicity of whole BoNT/A, but also interferes with uptake of BoNT/E and to a lesser extent BoNT/F (PubMed:19650874). The RBD recognizes the N-linked glycan on 'Asn-559' of SV2A, SV2B and SV2C; hydrogen-bonding occurs via 10 well-defined water molecules and stacking of hydrophobic residues (PubMed:27294781). Binds one host GT1b ganglioside, which serves as a coreceptor (PubMed:14731268, PubMed:18704164, PubMed:27958736). Modeling shows the HC can bind both coreceptors (a ganglioside and SV2 protein) simultaneously at different sites (PubMed:24240280). Crystals of the RBD with a GT1b analog can be grown at pH 5.5, indicating the toxin-ganglioside complex could be stable within the endosome (PubMed:18704164). Isolated RBD binds NTNHA (a bacterial protein that protects toxin) with high affinity at pH 6.0 but not at pH 7.5 (PubMed:22363010). The N-terminal belt (residues 449-545) wraps around the perimeter of the LC, probably protecting Zn2+ in the active site; it is not required for channel formation by the TD domain but may serve to prevent premature LC dissociation from the translocation channel and to protect toxin prior to translocation (PubMed:22158863, PubMed:17907800, PubMed:19351593). The isolated TD forms transmembrane channels of about 15 Angstroms in the absence of a pH gradient; LC translocation requires a pH and redox gradient (pH 5.0/oxidizing in the cis compartment, pH 7.0/reducing in the trans compartment), LC does not unfold unless the cis pH is 6.0 or less (PubMed:2446925, PubMed:17666397, PubMed:19096517). Pores are presumably made by 1-2 toxin molecules (PubMed:23471747). While interaction with the RBD modulates the pH threshold for membrane insertion, the RBD is not essential for toxin degradation of SNAP25 in neural cells (PubMed:19096517).

    - **Flags**: Adherence, Invasion

- **Rationale for inclusion**: The clostridial neurotoxins are the most lethal known to man.
- **Legitimate use case**: Available under the name Botox (onabotulinumtoxinA, Allergan), Dysport (abobotulinumtoxinA, Ipsen Biopharmaceuticals) and Xeomin (incobotulinumtoxinA, Merz Pharmaceuticals) and used for the treatment of blepharospasm, strabismus, facial muscle tics, torticollis, migraines, hyperhydrosis. It is also used cosmetically to smooth wrinkles.
    - **Citation**: PMID10883646, PMID12195265, PMID1416815, PMID15222577, PMID28356439, PMID3773633, PMID9950563
